# Mapping geographical inequalities in access to drinking water and sanitation facilities in low-income and middle-income countries, 2000–17

**DOI:** 10.1016/S2214-109X(20)30278-3

**Published:** 2020-08-19

**Authors:** Local Burden of Disease WaSH Collaborators, Aniruddha Deshpande, Molly K Miller-Petrie, Paulina A Lindstedt, Mathew M Baumann, Kimberly B Johnson, Brigette F Blacker, Hedayat Abbastabar, Foad Abd-Allah, Ahmed Abdelalim, Ibrahim Abdollahpour, Kedir Hussein Abegaz, Ayenew Negesse Abejie, Lucas Guimarães Abreu, Michael R.M. Abrigo, Ahmed Abualhasan, Manfred Mario Kokou Accrombessi, Abdu A Adamu, Oladimeji M Adebayo, Isaac Akinkunmi Adedeji, Rufus Adesoji Adedoyin, Victor Adekanmbi, Olatunji O Adetokunboh, Tara Ballav Adhikari, Mohsen Afarideh, Marcela Agudelo-Botero, Mehdi Ahmadi, Keivan Ahmadi, Muktar Beshir Ahmed, Anwar E Ahmed, Temesgen Yihunie Akalu, Ali S Akanda, Fares Alahdab, Ziyad Al-Aly, Samiah Alam, Noore Alam, Genet Melak Alamene, Turki M Alanzi, James Albright, Ammar Albujeer, Jacqueline Elizabeth Alcalde-Rabanal, Animut Alebel, Zewdie Aderaw Alemu, Muhammad Ali, Mehran Alijanzadeh, Vahid Alipour, Syed Mohamed Aljunid, Ali Almasi, Amir Almasi-Hashiani, Hesham M Al-Mekhlafi, Khalid A Altirkawi, Nelson Alvis-Guzman, Nelson J. Alvis-Zakzuk, Saeed Amini, Arianna Maever L. Amit, Gianna Gayle Herrera Amul, Catalina Liliana Andrei, Mina Anjomshoa, Ansariadi Ansariadi, Carl Abelardo T. Antonio, Benny Antony, Ernoiz Antriyandarti, Jalal Arabloo, Hany Mohamed Amin Aref, Olatunde Aremu, Bahram Armoon, Amit Arora, Krishna K Aryal, Afsaneh Arzani, Mehran Asadi-Aliabadi, Daniel Asmelash, Hagos Tasew Atalay, Seyyede Masoume Athari, Seyyed Shamsadin Athari, Sachin R Atre, Marcel Ausloos, Shally Awasthi, Nefsu Awoke, Beatriz Paulina Ayala Quintanilla, Getinet Ayano, Martin Amogre Ayanore, Yared Asmare Aynalem, Samad Azari, Andrew S Azman, Ebrahim Babaee, Alaa Badawi, Mojtaba Bagherzadeh, Shankar M Bakkannavar, Senthilkumar Balakrishnan, Maciej Banach, Joseph Adel Mattar Banoub, Aleksandra Barac, Miguel A Barboza, Till Winfried Bärnighausen, Sanjay Basu, Vo Dinh Bay, Mohsen Bayati, Neeraj Bedi, Mahya Beheshti, Meysam Behzadifar, Masoud Behzadifar, Diana Fernanda Bejarano Ramirez, Michelle L Bell, Derrick A. Bennett, Habib Benzian, Dessalegn Ajema Berbada, Robert S Bernstein, Anusha Ganapati Bhat, Krittika Bhattacharyya, Soumyadeep Bhaumik, Zulfiqar A Bhutta, Ali Bijani, Boris Bikbov, Muhammad Shahdaat Bin Sayeed, Raaj Kishore Biswas, Somayeh Bohlouli, Soufiane Boufous, Oliver J Brady, Andrey Nikolaevich Briko, Nikolay Ivanovich Briko, Gabrielle B Britton, Alexandria Brown, Sharath Burugina Nagaraja, Zahid A Butt, Luis Alberto Cámera, Ismael R Campos-Nonato, Julio Cesar Campuzano Rincon, Jorge Cano, Josip Car, Rosario Cárdenas, Felix Carvalho, Carlos A Castañeda-Orjuela, Franz Castro, Ester Cerin, Binaya Chalise, Vijay Kumar Chattu, Ken Lee Chin, Devasahayam J Christopher, Dinh-Toi Chu, Natalie Maria Cormier, Vera Marisa Costa, Elizabeth A Cromwell, Abel Fekadu Fekadu Dadi, Tukur Dahiru, Saad M A Dahlawi, Rakhi Dandona, Lalit Dandona, Anh Kim Dang, Farah Daoud, Aso Mohammad Darwesh, Amira Hamed Darwish, Ahmad Daryani, Jai K Das, Rajat Das Gupta, Aditya Prasad Dash, Claudio Alberto Dávila-Cervantes, Nicole Davis Weaver, Fernando Pio De la Hoz, Jan-Walter De Neve, Dereje Bayissa Demissie, Gebre Teklemariam Demoz, Edgar Denova-Gutiérrez, Kebede Deribe, Assefa Desalew, Samath Dhamminda Dharmaratne, Preeti Dhillon, Meghnath Dhimal, Govinda Prasad Dhungana, Daniel Diaz, Isaac Oluwafemi Dipeolu, Hoa Thi Do, Christiane Dolecek, Kerrie E Doyle, Eleonora Dubljanin, Andre Rodrigues Duraes, Hisham Atan Edinur, Andem Effiong, Aziz Eftekhari, Nevine El Nahas, Maysaa El Sayed Zaki, Maha El Tantawi, Hala Rashad Elhabashy, Shaimaa I. El-Jaafary, Ziad El-Khatib, Hajer Elkout, Aisha Elsharkawy, Shymaa Enany, Daniel Adane Endalew, Babak Eshrati, Sharareh Eskandarieh, Arash Etemadi, Oluchi Ezekannagha, Emerito Jose A. Faraon, Mohammad Fareed, Andre Faro, Farshad Farzadfar, Alebachew Fasil Fasil, Mehdi Fazlzadeh, Valery L. Feigin, Wubalem Fekadu, Netsanet Fentahun, Seyed-Mohammad Fereshtehnejad, Eduarda Fernandes, Irina Filip, Florian Fischer, Carsten Flohr, Nataliya A. Foigt, Morenike Oluwatoyin Folayan, Masoud Foroutan, Richard Charles Franklin, Joseph Jon Frostad, Takeshi Fukumoto, Mohamed M Gad, Gregory M Garcia, Augustine Mwangi Gatotoh, Reta Tsegaye Gayesa, Ketema Bizuwork Gebremedhin, Yilma Chisha Dea Geramo, Hailay Abrha Gesesew, Kebede Embaye Gezae, Ahmad Ghashghaee, Farzaneh Ghazi Sherbaf, Tiffany K Gill, Paramjit Singh Gill, Themba G Ginindza, Alem Girmay, Zemichael Gizaw, Amador Goodridge, Sameer Vali Gopalani, Bárbara Niegia Garcia Goulart, Alessandra C Goulart, Ayman Grada, Manfred S Green, Mohammed Ibrahim Mohialdeen Gubari, Harish Chander Gugnani, Davide Guido, Rafael Alves Guimarães, Yuming Guo, Rajeev Gupta, Rahul Gupta, Giang Hai Ha, Juanita A. Haagsma, Nima Hafezi-Nejad, Dessalegn H Haile, Michael Tamene Haile, Brian J. Hall, Samer Hamidi, Demelash Woldeyohannes Handiso, Hamidreza Haririan, Ninuk Hariyani, Ahmed I. Hasaballah, Md. Mehedi Hasan, Amir Hasanzadeh, Hamid Yimam Hassen, Desta Haftu Hayelom, Mohamed I Hegazy, Behzad Heibati, Behnam Heidari, Delia Hendrie, Andualem Henok, Claudiu Herteliu, Fatemeh Heydarpour, Hagos Degefa de Hidru, Thomas R Hird, Chi Linh Hoang, Gillian I Hollerich, Praveen Hoogar, Naznin Hossain, Mehdi Hosseinzadeh, Mowafa Househ, Guoqing Hu, Ayesha Humayun, Syed Ather Hussain, Mamusha Aman A Hussen, Segun Emmanuel Ibitoye, Olayinka Stephen Ilesanmi, Milena D. Ilic, Mohammad Hasan Imani-Nasab, Usman Iqbal, Seyed Sina Naghibi Irvani, Sheikh Mohammed Shariful Islam, Rebecca Q Ivers, Chinwe Juliana Iwu, Nader Jahanmehr, Mihajlo Jakovljevic, Amir Jalali, Achala Upendra Jayatilleke, Ensiyeh Jenabi, Ravi Prakash Jha, Vivekanand Jha, John S Ji, Jost B. Jonas, Jacek Jerzy Jozwiak, Ali Kabir, Zubair Kabir, Tanuj Kanchan, André Karch, Surendra Karki, Amir Kasaeian, Gebremicheal Gebreslassie Kasahun, Habtamu Kebebe Kasaye, Gebrehiwot G Kassa, Getachew Mullu Kassa, Gbenga A. Kayode, Mihiretu M Kebede, Peter Njenga Keiyoro, Daniel Bekele Ketema, Yousef Saleh Khader, Morteza Abdullatif Khafaie, Nauman Khalid, Rovshan Khalilov, Ejaz Ahmad Khan, Junaid Khan, Md Nuruzzaman Khan, Khaled Khatab, Mona M Khater, Amir M Khater, Maryam Khayamzadeh, Mohammad Khazaei, Mohammad Hossein Khosravi, Jagdish Khubchandani, Ali Kiadaliri, Yun Jin Kim, Ruth W Kimokoti, Sezer Kisa, Adnan Kisa, Sonali Kochhar, Tufa Kolola, Hamidreza Komaki, Soewarta Kosen, Parvaiz A Koul, Ai Koyanagi, Kewal Krishan, Barthelemy Kuate Defo, Nuworza Kugbey, Pushpendra Kumar, G Anil Kumar, Manasi Kumar, Dian Kusuma, Carlo La Vecchia, Ben Lacey, Aparna Lal, Dharmesh Kumar Lal, Hilton Lam, Faris Hasan Lami, Van Charles Lansingh, Savita Lasrado, Georgy Lebedev, Paul H Lee, Kate E LeGrand, Mostafa Leili, Tsegaye Lolaso Lenjebo, Cheru Tesema Leshargie, Aubrey J Levine, Sonia Lewycka, Shanshan Li, Shai Linn, Shiwei Liu, Jaifred Christian F Lopez, Platon D Lopukhov, Muhammed Magdy Abd El Razek, D.R. Mahadeshwara Prasad, Phetole Walter Mahasha, Narayan B. Mahotra, Azeem Majeed, Reza Malekzadeh, Deborah Carvalho Malta, Abdullah A Mamun, Navid Manafi, Mohammad Ali Mansournia, Chabila Christopher Mapoma, Gabriel Martinez, Santi Martini, Francisco Rogerlândio Martins-Melo, Manu Raj Mathur, Benjamin K Mayala, Mohsen Mazidi, Colm McAlinden, Birhanu Geta Meharie, Man Mohan Mehndiratta, Entezar Mehrabi Nasab, Kala M Mehta, Teferi Mekonnen, Tefera Chane Mekonnen, Gebrekiros Gebremichael Meles, Hagazi Gebre Meles, Peter T N Memiah, Ziad A Memish, Walter Mendoza, Ritesh G Menezes, Seid Tiku Mereta, Tuomo J Meretoja, Tomislav Mestrovic, Workua Mekonnen Metekiya, Workua Mekonnen Metekiya, Bartosz Miazgowski, Ted R Miller, GK Mini, Erkin M Mirrakhimov, Babak Moazen, Bahram Mohajer, Yousef Mohammad, Dara K. Mohammad, Naser Mohammad Gholi Mezerji, Roghayeh Mohammadibakhsh, Shafiu Mohammed, Jemal Abdu Mohammed, Hassen Mohammed, Farnam Mohebi, Ali H Mokdad, Yoshan Moodley, Masoud Moradi, Ghobad Moradi, Mohammad Moradi-Joo, Paula Moraga, Linda Morales, Abbas Mosapour, Jonathan F. Mosser, Simin Mouodi, Seyyed Meysam Mousavi, Miliva Mozaffor, Sandra B Munro, Moses K. Muriithi, Christopher J L Murray, Kamarul Imran Musa, Ghulam Mustafa, Saravanan Muthupandian, Mehdi Naderi, Ahamarshan Jayaraman Nagarajan, Mohsen Naghavi, Gurudatta Naik, Vinay Nangia, Bruno Ramos Nascimento, Javad Nazari, Duduzile Edith Ndwandwe, Ionut Negoi, Henok Biresaw Netsere, Josephine W. Ngunjiri, Cuong Tat Nguyen, Huong Lan Thi Nguyen, QuynhAnh P Nguyen, Solomon Gedlu Nigatu, Dina Nur Anggraini Ningrum, Chukwudi A Nnaji, Marzieh Nojomi, Ole F Norheim, Jean Jacques Noubiap, Bogdan Oancea, Felix Akpojene Ogbo, In-Hwan Oh, Andrew T Olagunju, Jacob Olusegun Olusanya, Bolajoko Olubukunola Olusanya, Obinna E Onwujekwe, Doris V. Ortega-Altamirano, Osayomwanbo Osarenotor, Frank B Osei, Mayowa O Owolabi, Mahesh P A, Jagadish Rao. Padubidri, Smita Pakhale, Adrian Pana, Eun-Kee Park, Sangram Kishor Patel, Ashish Pathak, Ajay Patle, Kebreab Paulos, Veincent Christian Filipino Pepito, Norberto Perico, Aslam Pervaiz, Julia Moreira Pescarini, Konrad Pesudovs, Hai Quang Pham, David M Pigott, Thomas Pilgrim, Meghdad Pirsaheb, Mario Poljak, Ian Pollock, Maarten J Postma, Farshad Pourmalek, Akram Pourshams, Sergio I Prada, Liliana Preotescu, Hedley Quintana, Navid Rabiee, Mohammad Rabiee, Amir Radfar, Alireza Rafiei, Fakher Rahim, Siavash Rahimi, Vafa Rahimi-Movaghar, Muhammad Aziz Rahman, Mohammad Hifz Ur Rahman, Fatemeh Rajati, Chhabi Lal Ranabhat, Puja C Rao, Davide Rasella, Goura Kishor Rath, Salman Rawaf, Lal Rawal, Wasiq Faraz Rawasia, Giuseppe Remuzzi, Vishnu Renjith, Andre M.N. Renzaho, Serge Resnikoff, Seyed Mohammad Riahi, Ana Isabel Ribeiro, Jennifer Rickard, Leonardo Roever, Luca Ronfani, Enrico Rubagotti, Salvatore Rubino, Anas M Saad, Siamak Sabour, Ehsan Sadeghi, Sahar Saeedi Moghaddam, Yahya Safari, Rajesh Sagar, Mohammad Ali Sahraian, S. Mohammad Sajadi, Mohammad Reza Salahshoor, Nasir Salam, Ahsan Saleem, Hosni Salem, Marwa Rashad Salem, Yahya Salimi, Hamideh Salimzadeh, Abdallah M Samy, Juan Sanabria, Itamar S Santos, Milena M. Santric-Milicevic, Bruno Piassi Sao Jose, Sivan Yegnanarayana Iyer Saraswathy, Nizal Sarrafzadegan, Benn Sartorius, Brijesh Sathian, Thirunavukkarasu Sathish, Maheswar Satpathy, Monika Sawhney, Mehdi Sayyah, Alyssa N Sbarra, Lauren E Schaeffer, David C Schwebel, Anbissa Muleta Senbeta, Subramanian Senthilkumaran, Sadaf G Sepanlou, Edson Serván-Mori, Azadeh Shafieesabet, Amira A Shaheen, Izza Shahid, Masood Ali Shaikh, Ali S Shalash, Mehran Shams-Beyranvand, MohammadBagher Shamsi, Morteza Shamsizadeh, Mohammed Shannawaz, Kiomars Sharafi, Rajesh Sharma, Aziz Sheikh, B Suresh Kumar Shetty, Wondimeneh Shibabaw Shiferaw, Mika Shigematsu, Jae Il Shin, Rahman Shiri, Reza Shirkoohi, K M Shivakumar, Si Si, Soraya Siabani, Tariq Jamal Siddiqi, Diego Augusto Santos Silva, Virendra Singh, Narinder Pal Singh, Balbir Bagicha Singh Singh, Jasvinder A. Singh, Ambrish Singh, Dhirendra Narain Sinha, Malede Mequanent Sisay, Eirini Skiadaresi, David L Smith, Adauto Martins Soares Filho, Mohammad Reza Sobhiyeh, Anton Sokhan, Joan B Soriano, Muluken Bekele Sorrie, Ireneous N Soyiri, Emma Elizabeth Spurlock, Chandrashekhar T Sreeramareddy, Agus Sudaryanto, Mu'awiyyah Babale Sufiyan, Hafiz Ansar Rasul Suleria, Bryan L. Sykes, Rafael Tabarés-Seisdedos, Takahiro Tabuchi, Degena Bahrey Tadesse, Ingan Ukur Tarigan, Bineyam Taye, Yonatal Mesfin Tefera, Arash Tehrani-Banihashemi, Shishay Wahdey Tekelemedhin, Merhawi Gebremedhin Tekle, Mohamad-Hani Temsah, Berhe Etsay Tesfay, Fisaha Haile Tesfay, Zemenu Tadesse Tessema, Kavumpurathu Raman Thankappan, Akhil Soman ThekkePurakkal, Nihal Thomas, Robert L Thompson, Alan J Thomson, Roman Topor-Madry, Marcos Roberto Tovani-Palone, Eugenio Traini, Bach Xuan Tran, Khanh Bao Tran, Irfan Ullah, Bhaskaran Unnikrishnan, Muhammad Shariq Usman, Olalekan A Uthman, Benjamin S. Chudi Uzochukwu, Pascual R Valdez, Santosh Varughese, Yousef Veisani, Francesco S Violante, Sebastian Vollmer, Feleke Gebremeskel W/hawariat, Yasir Waheed, Mitchell Taylor Wallin, Yuan-Pang Wang, Yafeng Wang, Kinley Wangdi, Daniel J Weiss, Girmay Teklay Weldesamuel, Adhena Ayaliew Werkneh, Ronny Westerman, Taweewat Wiangkham, Kirsten E Wiens, Tissa Wijeratne, Charles Shey Wiysonge, Haileab Fekadu Wolde, Dawit Zewdu Wondafrash, Tewodros Eshete Wonde, Getasew Taddesse Worku, Ali Yadollahpour, Seyed Hossein Yahyazadeh Jabbari, Tomohide Yamada, Mehdi Yaseri, Hiroshi Yatsuya, Alex Yeshaneh, Mekdes Tigistu Yilma, Paul Yip, Engida Yisma, Naohiro Yonemoto, Mustafa Z Younis, Hebat-Allah Salah A Yousof, Chuanhua Yu, Hasan Yusefzadeh, Siddhesh Zadey, Telma Zahirian Moghadam, Zoubida Zaidi, Sojib Bin Zaman, Mohammad Zamani, Hamed Zandian, Heather J Zar, Taddese Alemu Zerfu, Yunquan Zhang, Arash Ziapour, Sanjay Zodpey, Yves Miel H Zuniga, Simon I Hay, Robert C Reiner

## Abstract

**Background:**

Universal access to safe drinking water and sanitation facilities is an essential human right, recognised in the Sustainable Development Goals as crucial for preventing disease and improving human wellbeing. Comprehensive, high-resolution estimates are important to inform progress towards achieving this goal. We aimed to produce high-resolution geospatial estimates of access to drinking water and sanitation facilities.

**Methods:**

We used a Bayesian geostatistical model and data from 600 sources across more than 88 low-income and middle-income countries (LMICs) to estimate access to drinking water and sanitation facilities on continuous continent-wide surfaces from 2000 to 2017, and aggregated results to policy-relevant administrative units. We estimated mutually exclusive and collectively exhaustive subcategories of facilities for drinking water (piped water on or off premises, other improved facilities, unimproved, and surface water) and sanitation facilities (septic or sewer sanitation, other improved, unimproved, and open defecation) with use of ordinal regression. We also estimated the number of diarrhoeal deaths in children younger than 5 years attributed to unsafe facilities and estimated deaths that were averted by increased access to safe facilities in 2017, and analysed geographical inequality in access within LMICs.

**Findings:**

Across LMICs, access to both piped water and improved water overall increased between 2000 and 2017, with progress varying spatially. For piped water, the safest water facility type, access increased from 40·0% (95% uncertainty interval [UI] 39·4–40·7) to 50·3% (50·0–50·5), but was lowest in sub-Saharan Africa, where access to piped water was mostly concentrated in urban centres. Access to both sewer or septic sanitation and improved sanitation overall also increased across all LMICs during the study period. For sewer or septic sanitation, access was 46·3% (95% UI 46·1–46·5) in 2017, compared with 28·7% (28·5–29·0) in 2000. Although some units improved access to the safest drinking water or sanitation facilities since 2000, a large absolute number of people continued to not have access in several units with high access to such facilities (>80%) in 2017. More than 253 000 people did not have access to sewer or septic sanitation facilities in the city of Harare, Zimbabwe, despite 88·6% (95% UI 87·2–89·7) access overall. Many units were able to transition from the least safe facilities in 2000 to safe facilities by 2017; for units in which populations primarily practised open defecation in 2000, 686 (95% UI 664–711) of the 1830 (1797–1863) units transitioned to the use of improved sanitation. Geographical disparities in access to improved water across units decreased in 76·1% (95% UI 71·6–80·7) of countries from 2000 to 2017, and in 53·9% (50·6–59·6) of countries for access to improved sanitation, but remained evident subnationally in most countries in 2017.

**Interpretation:**

Our estimates, combined with geospatial trends in diarrhoeal burden, identify where efforts to increase access to safe drinking water and sanitation facilities are most needed. By highlighting areas with successful approaches or in need of targeted interventions, our estimates can enable precision public health to effectively progress towards universal access to safe water and sanitation.

**Funding:**

Bill & Melinda Gates Foundation.

## Introduction

WHO's Integrated Global Action Plan for the Prevention and Control of Pneumonia and Diarrhoea emphasises the need for preventive measures.^1^ Unsafe water and unsafe sanitation were the first and second leading risk factors for under-5 mortality from diarrhoeal diseases globally in 2017.^2^ These risks increase susceptibility to the spread of infectious agents that cause diarrhoea, including rotavirus and *Vibrio cholerae*.^3^, ^4^, ^5^, ^6^ They are also linked to the spread of neglected tropical diseases (NTDs),^7^, ^8^, ^9^, ^10^ and adverse outcomes such as stunting, wasting, and underweight.^11^, ^12^, ^13^ Low access to safe water and sanitation has also been linked to broader social outcomes such as reductions in school attendance (particularly for girls who are menstruating), losses to economic productivity, and undue burden on women of time spent collecting water.^14^, ^15^

Access to safe drinking water and sanitation are human rights,^16^ conferring benefits to human wellbeing beyond their impact on health. The global health and development community has prioritised access by including safe water and sanitation targets in both the Millennium Development Goals and more recently in the Sustainable Development Goals (SDGs), in which the UN called for access to be universal (ie, 100% access) and equitable. Despite substantial expansion of access during the Millennium Development Goals era, it has been previously estimated that less than 75% of the population in many countries in sub-Saharan Africa and south and southeast Asia had access to improved facilities in 2017.^17^

**Research in context**

**Evidence before this study**

In light of the health risks associated with unsafe drinking water and sanitation, as well as broader considerations of human development, the Sustainable Development Goals (SDGs) included the target of universal access to safe facilities by 2030. The WHO and United Nations Children's Fund (WHO–UNICEF) Joint Monitoring Programme estimates access to water and sanitation nationally and by urban and rural areas, while the Global Burden of Diseases, Injuries, and Risk Factors (GBD) study quantifies the health risks posed by unsafe water and sanitation nationally and for subnational regions in select countries. Although these and other efforts provide valuable insights, they mask local and cross-boundary variation and ultimately result in an incomplete picture of areas in greatest need of intervention. The limited availability of accurate and wide-ranging estimates monitoring local geographical inequalities presents a barrier to achieving universal access to safe water and sanitation facilities. Studies have used model-based geostatistics to map various health and sociodemographic factors, highlighting the potential to apply these methods for mapping access to water and sanitation across low-income and middle-income countries (LMICs).

**Added value of this study**

To our knowledge, this study presents the first high-resolution subnational estimates of access to safe drinking water and sanitation facilities across more than 88 LMICs and across all indicators of access from 2000 to 2017. Our Bayesian geostatistical models and extensive geolocated dataset account for spatial and temporal trends, and our suite of highly resolved spatial covariates leverage the relationships between access to water and sanitation and other variables for improved estimation. Additionally, this is the first application of ordinal regression methods on water and sanitation data at large spatial scales, allowing us to appropriately account for the mutually exclusive and collectively exhaustive (ie, accounting for 100% of the population) relationships between the relevant indicators of access. We report increasing access to safe facilities over time, but trends varied regionally, and many people continued to have no access to safe drinking water and sanitation facilities in 2017 across the LMICs studied. We estimate that 143 300 (95% uncertainty interval 126 100–163 000) deaths of children younger than 5 years were attributable to unsafe water in sub-Saharan Africa in 2017, yet increases in access to safe water averted more than 18 100 (15 700–21 200) child deaths in the region in that year. Averted child deaths were concentrated in select units that had great progress in access, while child diarrhoeal mortality increased in other units, probably due in part to decreases in access to piped water. Although geographical inequality decreased in most LMICs from 2000, in some cases the lowest level of access remained unchanged, effectively leaving behind some units, even as progress was made nationally.

**Implications of all the available evidence**

Despite considerable progress since 2000, geographical inequalities remain an obstacle to reaching the SDG target of universal access to safely managed facilities. Our estimates highlight where the most substantial improvements were achieved over time, identifying areas with successful strategies for adaptation elsewhere. Our identification of areas in which access to safe facilities is low, combined with high-resolution estimates of high diarrhoeal burden and child malnutrition prevalence, calls attention to communities with high susceptibility to the spread of infectious diseases. Although access to safe facilities is increasing in many countries, subnational inequalities point to the need for targeted interventions, particularly to reach communities with the lowest access and to increase access to the safest facility types. Our findings can inform local level monitoring of progress towards the SDG target, and provide a resource for decision makers to target areas most in need of additional resources.

Previous estimates of access have been reported primarily at the national level, as well as at the subnational level across Africa and for a subset of other countries.^2^, ^17^, ^18^, ^19^, ^20^, ^21^, ^22^, ^23^ The WHO and United Nations Children's Fund (WHO–UNICEF) Joint Monitoring Programme (JMP) has analysed inequality of access by wealth quintile and urban-rural status, as well as within subnational regions for select locations.^24^, ^25^ These analyses, however, do not provide comprehensive estimates over space and time across low-income and middle-income countries (LMICs) at fine spatial scales. Understanding variation in water and sanitation access in second administrative-level units (eg, districts, counties; henceforth termed units) is imperative to identifying low-access areas at heightened risk of disease transmission,^26^, ^27^ and areas that have successfully achieved high levels of access. Previous studies have used model-based geostatistics to map health indicators such as under-5 mortality,^27^ diarrhoea incidence and prevalence,^28^ and child growth failure,^29^ along with sociodemographic factors such as educational attainment,^30^ and interventions such as insecticide-treated bednet coverage^31^ and childhood vaccines.^32^ We aimed to extend these methods to estimate access to safe drinking water and sanitation facilities at fine spatial resolutions.

## Methods

### Overview

To produce a comprehensive baseline of comparable estimates, we leveraged the indicators used by the JMP^24^ and the Global Burden of Disease (GBD) study^2^ with a Bayesian geostatistical model. With use of data from 600 unique datasets, we produced estimates of the relative (proportion) and absolute (number of people) access to water and sanitation facilities across continuous geographical surfaces and aggregated resulting predictions to national and subnational levels (reported as second administrative-level units) in more than 88 LMICs from 2000 to 2017. We modelled access by facility-type indicators and for specific facilities with an ordinal modelling framework. We report regional results according to the GBD 2017 geographical hierarchy.^2^ We highlight specific types of transitions made in access to facility types and report the number of child diarrhoeal deaths attributed to unsafe facilities and averted by increased access to safe facilities. Finally, we present an analysis of variation in subnational geographical inequality in access.

### Study design

We generated estimates for two sets of four mutually exclusive and collectively exhaustive indicators of access—one set for drinking water and one for sanitation—with uncertainty intervals (UIs), from 2000 to 2017. Each set of four indicators collectively accounts for 100% of the population in the respective geographical area. We used an ordinal regression framework in which each indicator was modelled using an ensemble modelling framework for each country individually. We produced subnational-level estimates for 88 LMICs for water and 89 LMICs for sanitation, first estimating continent-wide surfaces at a resolution of approximately 5 × 5 km, and then aggregating to second and first administrative and national boundaries. We included low, low-middle, and middle development countries, as classified by their Socio-demographic Index quintile,^33^ an indicator based on education, fertility, and income (appendix p 94). Despite their relatively high Socio-demographic Index, China and Libya were included to maintain geographical continuity. Countries were excluded if sufficient data were not available for reliable estimation with use of our modelling paradigm. A complete list of the countries included is available in the appendix (p 94). This study complied with the Guidelines for Accurate and Transparent Health Estimates Reporting (appendix p 93).^34^ Further details on methods and software used are available in the appendix (pp 9–11).

### Data

**Figure 1:**
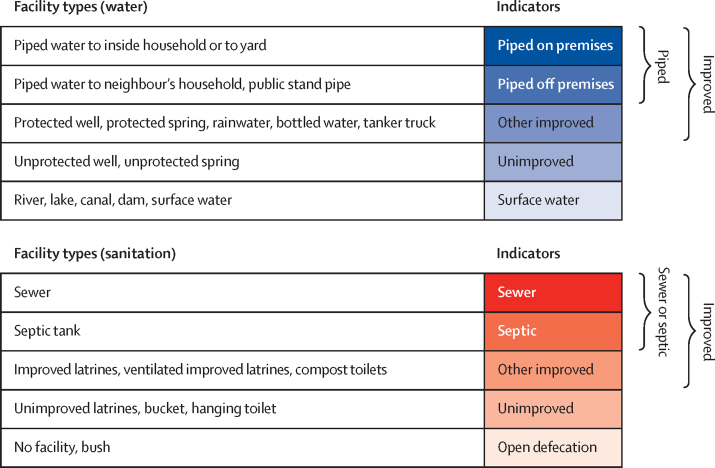
Access to drinking water and sanitation indicators The mutually exclusive and collectively exhaustive indicators modelled for water and sanitation. Each set of indicators collectively account for 100% of the population in the respective geographical area. The water indicators (piped on premises or piped off premises [piped], other improved, unimproved, and surface water) and the sanitation indicators (sewer or septic, other improved, unimproved, and open defecation) are outlined along with each indicator's corresponding facility types. Facility types are categorised into the standardised indicators as defined by the WHO–UNICEF Joint Monitoring Programme to ensure concordance with global monitoring targets and comparability across locations.

Data were collated from Demographic and Health Surveys, Multiple Indicator Cluster Surveys, and other household surveys and censuses across 88 countries for water and 89 for sanitation from 2000 to 2017 (inclusion criteria details in appendix p 6). We used data from 600 unique sources—501 for water and 457 for sanitation (some sources contained both water and sanitation data). For water, 60·2% of our data by weighted sample size comprised geopositioned points, and 69·3% of weighted sanitation data were points. All other data were areal data. Drinking water facilities were categorised as piped (piped on or off premises), other improved (protected wells and springs, bottled water, rainwater collection, bought water), unimproved (unprotected wells and springs), or surface water (figure 1). Sanitation facilities were categorised as sewer or septic (sewer or septic tanks), other improved (improved latrines, ventilated improved latrines, composting toilets), unimproved (flush toilets to open channels, unimproved latrines), or open defecation (figure 1), using standardised definitions from the JMP.^17^, ^24^ The resulting schema yielded mutually exclusive and collectively exhaustive indicators for water and sanitation access.^17^

### Statistical analysis

To account for the ordinal data structure of the indicators of access for water (piped, other improved, unimproved, and surface water) and sanitation (sewer or septic, other improved, unimproved, and open defecation), this analysis used an ordinal continuation-ratio modelling strategy.^35^ This approach allows for the simultaneous modelling of mutually exclusive categorical responses, as shown in similar geospatial analyses.^30^, ^32^ We first modelled the proportion of the population with access to sewer or septic sanitation using a binomial model. We then modelled the proportion of the population with access to other improved sanitation conditioned on not having access to sewer or septic sanitation. Subsequently, we modelled the proportion with access to unimproved sanitation conditioned on not having access to sewer or septic sanitation or other improved sanitation. The estimates from the second and third conditional models were then combined with the estimates from the first to generate a full set of estimates of access for all four indicators. In this manner, the estimates and their associated uncertainty incorporate the mutually exclusive and collectively exhaustive data structure. The same approach was used for the set of four indicators of access to drinking water facilities.

For each model used in this strategy, we used an ensemble modelling approach. Applying a stacking ensemble modelling approach, the data were initially fit with seven gridded-raster covariates (appendix p 28) to three independent models using a generalised additive model, boosted regression trees, and lasso regression. This generalised stacking approach has been successfully used in similar geospatial analyses of health and social data to optimise predictive performance.^30^, ^32^ Predictions were generated from each of these child models to create raster covariates to be used in the parent model. Subsequently, the data were fit with the raster covariates from the child models, with a binomial model using a spatially explicit mixed-effects generalised linear model via integrated nested Laplace approximation. Predictions were generated from this model by drawing 250 samples from the posterior distribution and taking the mean of these draws. The 95% UIs were generated by calculating the 2·5th and 97·5th percentiles of the drawn samples. Uncertainty for estimates are presented for piped and improved water and sanitation indicators in the appendix (pp 39–42). To ensure our predictions were aligned with national-level temporal trends, we fitted a generalised additive linear model to nationally representative data for each country. We calibrated the geospatial model to estimates from the national model by ensuring that, when aggregated to the national level, the geospatial estimates matched the corresponding estimates from the national model. Model fits were assessed via five-fold cross-validation and calculating out-of-sample root-mean squared errors, bias, and 95% coverage for each model (appendix pp 10–11). Analyses were done with R, version 3.5.0. Maps were produced with ArcGIS Desktop 10.6. Further details for model specification and methods can be found in the appendix (pp 9–11).

We measured access as the proportion of the population accessing the corresponding water and sanitation facility type in a given geographical area at a specific time. To assess progress over time, we calculated the mean annual change from 2000 to 2017.

To assess the effect of changes in water and sanitation access on diarrhoeal disease mortality in children younger than 5 years, we used a comparative risk assessment framework to construct counterfactuals and assess child deaths averted due to increased access.^2^ We used estimates of diarrhoeal mortality in children younger than 5 years available at the same spatial scales from the geospatial analysis described by Reiner and colleagues^36^ for this counterfactual analysis; as such, only countries with data available from Reiner and colleagues were included. We combined these mortality estimates with risk ratios estimated in GBD 2017,^2^ which associated different types of water and sanitation facilities with varied risks of diarrhoeal disease. To use the risk ratios for different categories of water access from GBD, we combined our estimates of water access with household water treatment prevalence data from GBD to create concordant categories of water facility exposures. With these data, we calculated population attributable fractions of child diarrhoeal deaths to unsafe water and sanitation.^2^, ^26^ We then used access estimates in 2000 to calculate a counterfactual population attributable fraction. Using these population attributable fractions in conjunction with the Reiner and colleagues estimates of child diarrhoeal disease mortality across units,^36^ we calculated attributable under-5 deaths for water and sanitation in 2017, as well as the number of averted child deaths in 2017 due to changes in water and sanitation access since 2000. We propagated uncertainty by repeating the calculation for values from each of the 250 draws of the posterior from our model. This methodology is further outlined in the appendix (p 11).

Additionally, we estimated inequality using subnational variation across units and the Gini coefficient,^37^ which summarises the distribution of each indicator across the population, with a value of zero representing perfect equality and a value of one representing maximum inequality (appendix pp 11–12).

### Role of the funding source

The funder had no role in study design, data collection, data analysis, data interpretation, or writing of the report. The corresponding author had full access to all the data in the study and had final responsibility for the decision to submit for publication.

## Results

**Figure 2:**
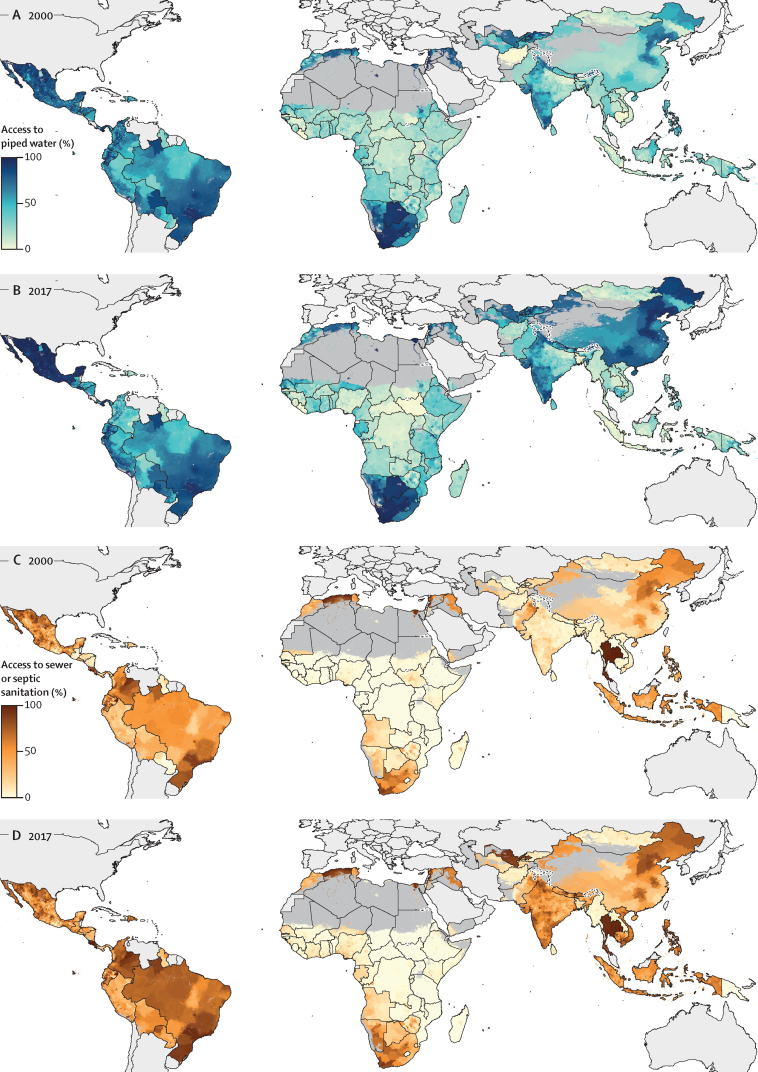
Access to piped water and sewer or septic sanitation at the second-administrative-unit level, 2000 and 2017 Access was modelled with use of model-based geostatistics for continuous continent-wide surfaces and aggregated to the second administrative level. The results for piped water are shown for years 2000 (A) and 2017 (B). The results for sewer or septic sanitation are also shown for 2000 (C) and 2017 (D). Maps reflect administrative boundaries, land cover, lakes, and population; dark grey-coloured grid cells were classified as barren or sparsely vegetated and had fewer than ten people per 1 × 1-km grid cell, or were not included in these analyses.^38^, ^39^, ^40^, ^41^, ^42^, ^43^Interactive visualisation tools are available online.

Access to all facility-type indicators for drinking water varied spatially and temporally. Across all LMICs assessed, access to piped water increased between 2000 and 2017 (from 40·0% [95% UI 39·4–40·7] to 50·3% [50·0–50·5] of the population), but this trend differed across regions (figure 2). Access to improved water overall increased from 82·6% (95% UI 82·3–82·8) in 2000 to 87·0% (86·8–87·1) in 2017 (figure 2; appendix p 32). Access levels for each unit are presented in the appendix and online through our data visualisation tool.

Although access to piped water was lowest in sub-Saharan Africa compared with other regions in 2017, notable areas of high access are apparent within sub-Saharan Africa (figure 2). Across sub-Saharan Africa, at least 80% of the population had access (henceforth referred to as high access) to piped water in 2017 in 5·9% (95% UI 5·7–6·2) of units, but 54·0% (53·5–54·8) of units had decreases in piped water access from 2000 to 2017, such as the Western Urban district of Sierra Leone (52·9% decrease [50·2–55·1]; figure 2). Units with high access to piped water facilities in sub-Saharan Africa mostly corresponded to large urban centres, such as Addis Ababa, Ethiopia (97·7% [95% UI 97·0–98·1]), and the Department of Guédiaway in Dakar, Senegal (92·9% [91·0–94·2]). However, in urban centres and other densely populated areas, a high absolute number of people continued to have no access even where unit-level access was high. For example, unit-level access to piped water was 90·5% (95% UI 85·2–95·3) in Casablanca, Morocco, yet 398 300 (198 500–618 500) people had no access. Large increases in piped water access also occurred from 2000 to 2017 for several African countries. In Niger, national-level piped water access increased from 23·5% (95% UI 22·7–24·4) to 47·6% (46·6–48·5), with a 1·3% mean annual percentage-point increase. In sub-Saharan Africa, 19·3% (95% UI 16·2–19·1) of units increased piped water access by more than 10 percentage points (figure 2). Access to improved water facilities overall was widespread in Africa, despite the relatively lower levels of piped water access: 56·8% (95% UI 56·3–57·3) of units had high access (>80%) to improved water in 2017 (appendix p 32).

In south Asia, piped water access was relatively low, with just 8·5% (95% UI 7·8–9·2) of units with high access to piped water in 2017 (figure 2). However, improved water access overall was relatively high in the region, increasing from 83·1% (95% UI 82·9–83·3) in 2000 to 92·5% (92·3–92·6) in 2017. Access to piped water was much higher in southeast Asia, east Asia, and Oceania in 2017, where 58·1% (95% UI 57·5–58·5) of units had high access. However, most of this piped water access was concentrated in China; when excluding China, access to piped water was just 3·2% (95% UI 2·8–3·7) in the region. Access to improved water remained relatively stable in southeast Asia, east Asia, and Oceania over the study period. Access was 91·2% (95% UI 90·6–91·7) in 2000 and 88·7% (88·3–89·1) in 2017. In southeast Asia, east Asia, and Oceania overall, access to improved water was high (>80%) in 77·0% (95% UI 76·1–77·8) of units in 2017, and the mean annual change in access was more than 2 percentage points in 7·1% (6·3–7·8) of units since 2000.

Access to piped water was relatively high in much of Latin America: 51·0% (95% UI 49·0–53·1) of units had high access to piped water in 2017 (figure 2). Improved water access increased in Latin America since 2000, from 89·6% (89·3–89·7) to 93·2% (93·1–93·3). Individual units also had large increases in access to improved water—eg, 96·4% (94·4–97·9) of units in Peru increased improved water access by more than 10 percentage points.

**Figure 3:**
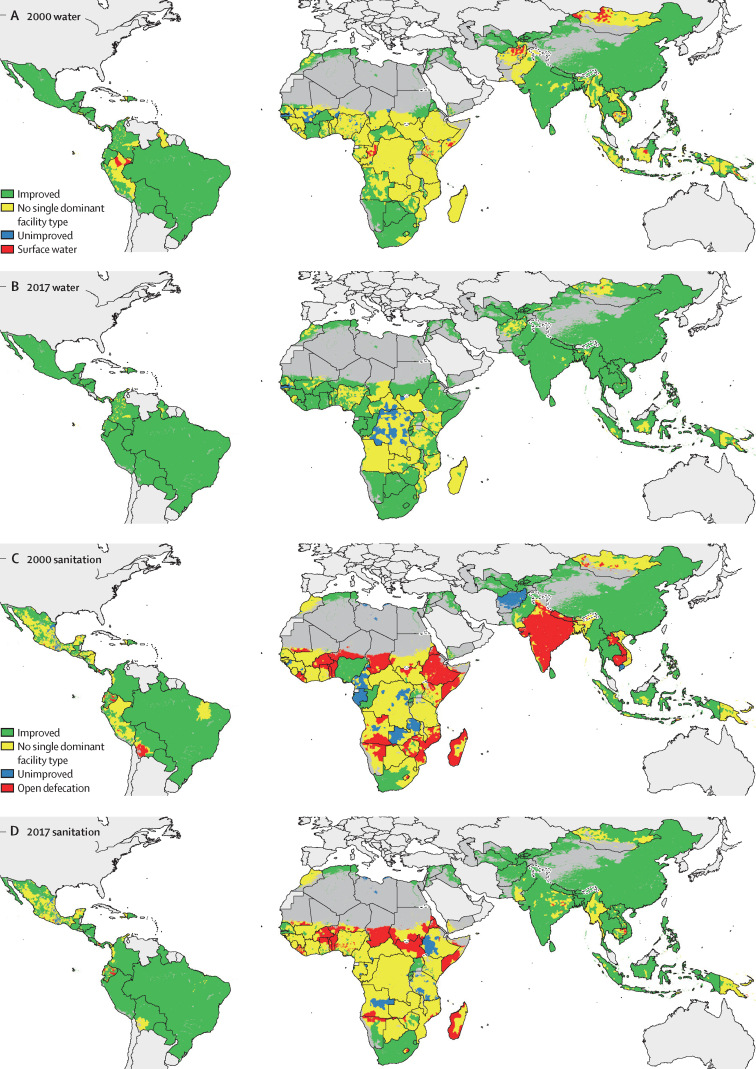
Water and sanitation facility types used at the second-administrative-unit level, 2000 and 2017 The co-distribution of improved, unimproved, and no facility access is shown for water for 2000 (A) and 2017 (B) and sanitation for 2000 (C), and 2017 (D). Green denotes second administrative-level units where most of the population (>60%) had access to improved facilities, blue denotes a more than 60% reliance on unimproved facilities, and red denotes more than 60% relying or surface water in A and B or practicing open defecation in C and D. Yellow indicates that there was no single dominant facility type used by more than 60% of the unit's population. Maps reflect administrative boundaries, land cover, lakes, and population; dark grey-coloured grid cells were classified as barren or sparsely vegetated and had fewer than ten people per 1 × 1-km grid cell, or were not included in these analyses.^38^, ^39^, ^40^, ^41^, ^42^, ^43^

We sought to identify which units made transitions from no facility (ie, surface water or open defecation) in 2000 to improved facilities in 2017, compared with more gradual transitions (from no facility to unimproved facilities, or unimproved to improved facilities) over the study period. To do so, we compared the most common type of water and sanitation access in each unit (access level of more than 60% of the population; figure 3). Across all LMICs, 397 (95% UI 371–428) units in 2000 had 60% or more of their populations that relied on surface water. Of these, 176 (156–197) units had substantial upgrades—transitioning to 60% or more using improved facilities by 2017. In comparison, 780 (95% UI 741–825) units had 60% or more of people relying on surface water or 60% or more of people using unimproved facilities in 2000; of these, relatively incremental upgrades (either from surface water to unimproved facilities, or from unimproved to improved water facilities) occurred in 182 (95% UI 160–205) units. The full array of model outputs can be accessed online via our customised online visualisation tools.

**Figure 4:**
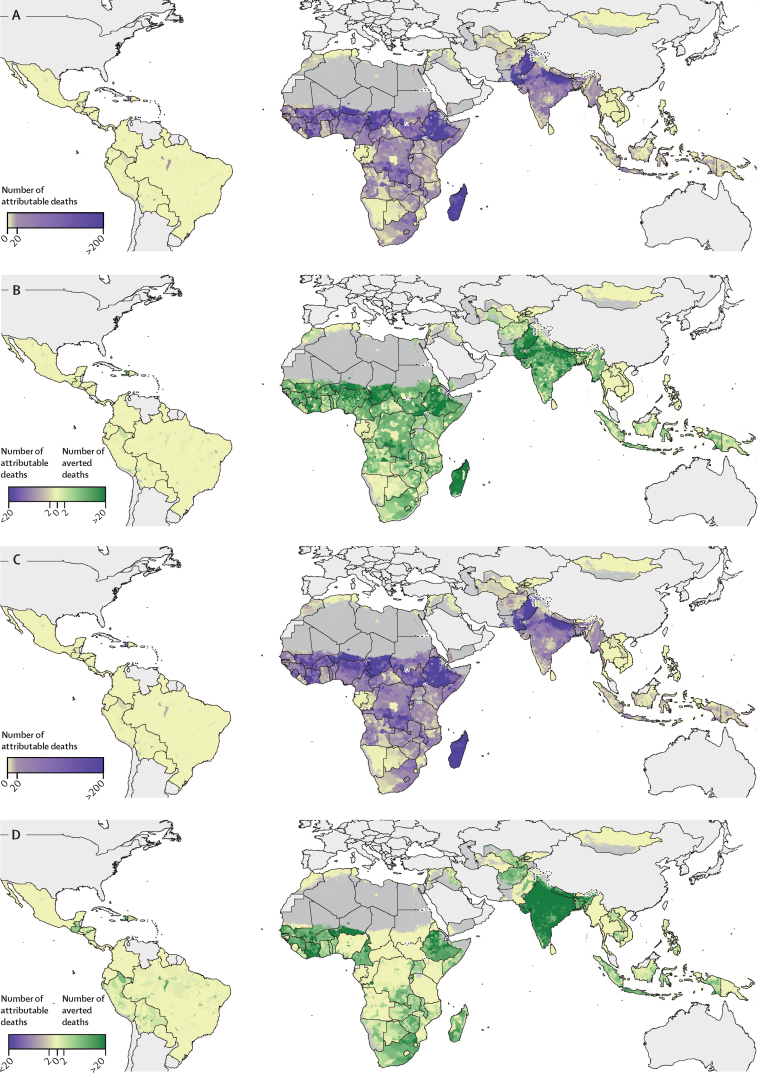
Effect of changes in access to water and sanitation in 2017 on child diarrhoeal deaths at the second-administrative-unit level Deaths are calculated under the counterfactual scenario in which access to safe water and sanitation remained at the values observed in the year 2000. The number of deaths attributed given access levels observed in 2017 is shown for water (A) and sanitation (C). The number of deaths averted (shown in green) or caused (shown in purple) in 2017 due to changes in access levels compared with 2000 is shown for water (B) and sanitation (D). Maps reflect administrative boundaries, land cover, lakes, and population; dark grey-coloured grid cells were classified as barren or sparsely vegetated and had fewer than ten people per 1 × 1-km grid cell, or were not included in these analyses.^38^, ^39^, ^40^, ^41^, ^42^, ^43^

We used a comparative risk assessment framework to estimate the number of deaths in children younger than 5 years attributed to unsafe water and sanitation in 2017 and the number of child deaths averted due to changes in access. In 2017, 143 300 (95% UI 126 100–163 000) deaths in children younger than 5 years in sub-Saharan Africa were attributable to unsafe water, and 18 100 (15 700–21 200) child deaths were averted by increases in safe water access (figure 4). In southeast Asia, east Asia, and Oceania, 9470 (95% UI 8650–10 300) child deaths were attributable to unsafe water in 2017, whereas increases in safe water averted at least 1310 (1200–1440) child deaths in 2017.

**Figure 5:**
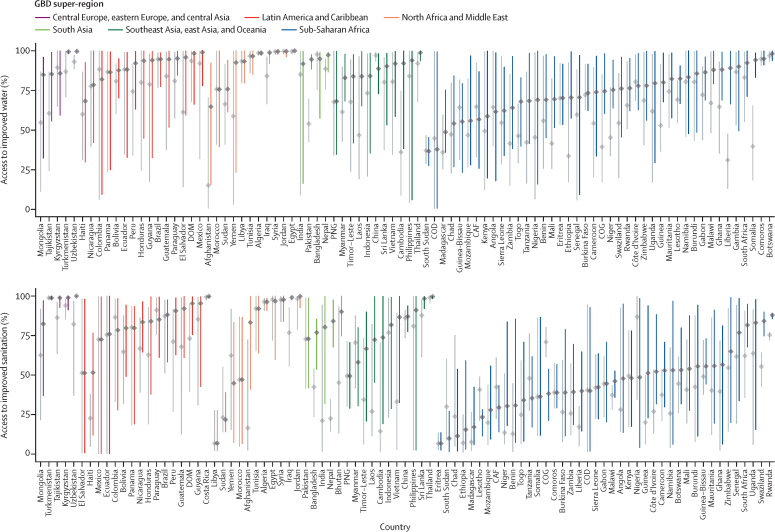
Geographical inequality in access to improved water and sanitation Persistence of geographical inequality in access to improved facility types for water and sanitation and changes since 2000 are shown. Each bar's height plots the level of access to improved water and sanitation, from the lowest to the highest access second administrative-level unit in 2000 (grey) and 2017 (coloured by region). Mean access at the national level is shown as grey dots. Colours correspond to Global Burden of Disease regions. Countries not shown were excluded from the study due to limited data availability. CAF=Central African Republic. COD=Democratic Republic of Congo. COG=Republic of Congo. DOM=Dominican Republic. GNQ=Equatorial Guinea. PNG=Papua New Guinea.

Subnational disparities in access to improved drinking water and sanitation facilities, defined as the range of values from the unit with the highest level of access to the unit with the lowest level of access, are evident across LMICs (figure 5). For improved water, disparities decreased in 76·1% (95% UI 71·6–80·7) of LMICs from 2000 to 2017. El Salvador and Mexico had among the greatest reductions in disparity for improved water, although absolute and relative inequalities still persisted in 2017; the lowest access in Mexico was 56·7% (95% UI 29·8–77·1) less than the national mean in 2000 (3·0 times lower), and 20·8% (5·7–36·8) less than the mean (1·3 times lower) in 2017. Disparities in access to improved water changed in different ways across countries. In Ethiopia, the gap between the units with the lowest and highest access closed largely because the lowest level of access in 2000 drew closer to the highest level of access by 2017, increasing mean access to improved water nationally in 2017. By comparison, mean access to improved water was increased nationally in Mozambique by 2017, but the lowest access level was similar in 2000 and 2017, while the highest level of access increased, widening the total range. Inequalities as determined with use of the Gini coefficient revealed additional trends. In 2000, 25 LMICs had Gini coefficients that exceeded 0·15 for improved water access, whereas in 2017, only nine remained higher than that level.

Access to sewer or septic sanitation across all LMICs increased from 28·7% (95% UI 28·5–29·0) in 2000 to 46·3% (46·1–46·5) in 2017, whereas access to improved sanitation overall increased from 60·0% (59·8–60·1) to 75·8% (75·7–75·9; figure 2, appendix p 36). Some regions saw large increases in access to sewer or septic sanitation since 2000, whereas access remained low across the study period in others.

Access to sewer or septic sanitation in sub-Saharan Africa was concentrated in similar areas to piped water, but just 1·0% (95% UI 1·0–1·0) of units had high access in the region, such as Bulawayo, Zimbabwe (96·5% access [95·8–97·1]; figure 2). In places with high unit-level access, many individuals can remain without access to sewer or septic sanitation. For instance, despite 88·6% (87·2–89·7) of the population of Harare, Zimbabwe having access, more than 253 000 people did not have access in 2017.

In south Asia, there was high access to sewer or septic sanitation in 11·3% (95% UI 9·4–12·9) of units in 2017 (figure 2). Improved sanitation overall had high unit-level access in 69·0% (95% UI 67·6–70·4) of units in south Asia, increasing from a regional access level of 29·4% (29·1–29·6) in 2000 to 79·4% (79·2–79·7) in 2017. Access to sewer or septic sanitation was higher in southeast Asia, east Asia, and Oceania compared with south Asia in 2017; 42·0% (95% UI 40·8–43·1) of units in the region had high access. Substantial increases in sewer or septic sanitation access occurred over the study period, such as in Rapti District, Mid-Western Region, Nepal, (49·3% [95% UI 43·5–54·9] increase; mean annual change of 2·7 percentage points). Unit-level access to improved sanitation was high in 67·8% (95% UI 66·8–69·0) of units in southeast Asia, east Asia, and Oceania, increasing from 81·6% (81·2–82·0) to 85·9% (85·7–86·2) over the study period.

In Latin America, there was high access to sewer or septic sanitation in 32·8% (95% UI 31·7–33·8) of units in 2017 (figure 2). Latin America notably had an 11·8% increase in access to sewer or septic sanitation over the period (from 59·5% [59·3–59·8] to 71·3% [70·9–71·6]). Large increases occurred in sewer or septic sanitation from 2000, including in San Pedro Sula, Cortés, Honduras (increase of 35·9% [32·4–39·5], mean annual change of 2·0 percentage points). Access to improved sanitation overall was also high across the region, with more than half (54·5% [53·6–55·6]) of units in Latin America with high (>80%) access.

Of the 1830 (95% UI 1797–1863) units in which 60% or more of people practised open defecation in 2000, 686 (664–711) transitioned substantially to having access to improved facilities in 2017 (figure 3). By contrast, 580 (550–610) of the 5630 (5560–5690) units in which 60% or more of people practised open defecation or in which 60% or more of people used unimproved facilities in 2000 transitioned incrementally by 2017. Subnationally, many units in Ethiopia, such as Afder Zone, Somali, increased access to improved sanitation (11·6% [12·4–16·8] in 2000; 26·0% [22·8–29·0] in 2017) while substantially reducing open defecation (79·7% [77·0–82·1] in 2000; 43·3% [39·1–47·2] in 2017) over the period. Populations with high reliance on open defecation in 2017, were mostly concentrated in trans-border regions in southern Angola–northern Namibia and in west and central Africa.

According to our comparative risk assessment framework, in 2017, 182 300 (95% UI 159 900–208 200) under-5 child deaths in sub-Saharan Africa were attributed to unsafe sanitation, whereas increases in safe sanitation access averted at least 10 100 (8970–11 400) child deaths in the region (figure 4). In southeast Asia, east Asia, and Oceania, increases in safe sanitation averted at least 2750 (95% UI 2530–3040) child deaths, with 7810 (7050–8700) child deaths attributable to unsafe sanitation in 2017, compared with 1840 (1660–2070) averted child deaths and 4400 (4080–4690) child deaths attributable to unsafe sanitation in Latin America in 2017.

Subnational disparities in improved sanitation decreased in 53·9% (95% UI 50·6–59·6) of countries from 2000, and large decreases occurred in Vietnam and Cambodia, among other locations, although disparities were evident across LMICs in 2017 (figure 5). The lowest-access unit in Cambodia was 4·9 times less than the national mean in 2000, and just 1·4 times less than in 2017. Temporal trends in disparities varied in LMICs. In Namibia, mean access to improved sanitation increased overall from 2000 to 2017, but the lowest level of access remained relatively unchanged since 2000. Conversely, the highest level of access and the lowest level of access increased substantially in Cambodia over the study period. With use of the Gini coefficient, we found that many countries had consistent subnational inequality in improved sanitation, with Gini coefficients of more than 0·15 in 37 LMICS in 2000 and 30 LMICs in 2017. Notably, Chad, Libya, and Togo had Gini coefficients of more than 0·35 in 2017.

## Discussion

Access to safe drinking water and sanitation facilities has improved globally between 2000, and 2017, but disparities in access varied across LMICs, presenting a barrier to achieving the SDG goal of universal access (100% access). Many units, such as in Cambodia for drinking water, had sizeable transitions, with the great majority of the population relying on the lowest quality of facility types in 2000 but accessing improved facilities by 2017. These units are exemplars that merit further study to identify drivers of success for replication elsewhere. In many countries, however, such progress was concurrent with increasing geographical inequality, as some units were effectively left behind. Our local-level estimates provide information to better target interventions to ensure progress towards greater access without increasing geographical inequality.

Estimates of access at the unit level support local monitoring of progress towards the SDG targets. Although our estimates of access to safe drinking water and sanitation facilities represent a best-case scenario of SDG attainment, in that they do not capture all the elements of safe management as defined by the JMP, even in the best-case scenario it is evident that many locations will need to scale up access to attain the goal of universal coverage. These results also show that estimates of access stratified by urban or rural status only or at the first administrative level are likely to mask further localised heterogeneity. By providing estimates at the second administrative level, in which programmatic decisions are often made, our results enable local decision makers to target resources and programmes with greater precision. Given that household-level water, sanitation, and hygiene interventions have had mixed results,^44^, ^45^, ^46^ these estimates can support targeting interventions at the community level to maximise efficiency and serve areas most in need of access.

Although increases in access to improved water facilities overall were observed in sub-Saharan Africa, access to piped water remained low in 2017. Decreases in piped water access were particularly apparent in some regions of sub-Saharan Africa, where demographic changes might be outpacing infrastructure development. The bulk of interventions in sub-Saharan Africa have focused on increasing access to improved wells or springs, and the relatively high rates of access to other improved water facilities in LMICs in 2017 indicates that these interventions have largely been successful. Further investment in piped drinking water is needed to scale up and maintain access and to ensure consistent and quality access. Although initially costly, these efforts will ultimately improve economic productivity, supporting national development and stability.^47^, ^48^

Our estimates revealed several units in which access to the safest facility types improved substantially. Exemplars in increasing piped water access include Svay Pao District, Battambang, Cambodia; Jantetelco Municipality, Morelos, Mexico; and Harari People's National Regional State, Ethiopia. Alongside economic growth nationally, the autonomous Phnom Penh Water Supply Authority has been credited with substantial expansion of Cambodia's urban piped water supply, whereas national and regional policy and government investment are likely to have played a role in Mexico and Ethiopia.^49^, ^50^, ^51^ Further research on successful interventions in these locations could help other units to adapt similar strategies. The same is true for exemplars of increased sewer or septic sanitation, including Kampong Chhnang District, Cambodia, and Bagmati Zone, Central Development Region, Nepal, where potential drivers include governance guided by national and regional policies, infrastructure construction and management, and (in the case of Nepal) social movements.^52^, ^53^, ^54^, ^55^ Urbanisation generally leads to increased water and sanitation infrastructure, although informal settlements in urban areas are often excluded, presenting a challenge to achieving equity in access.^56^ Although access to the safest facility types is lowest across sub-Saharan Africa, several units have high access in the region. These exemplars indicate that increasing access to piped systems in sub-Saharan Africa is entirely possible, despite demands on infrastructure and long-term maintenance. Here, we consider the safest facilities to be those with the lowest health risk, defined as the lowest associated risk-ratio for diarrhoeal disease. Considerations of cost-effectiveness and logistical needs will probably influence what technology is the most appropriate for any single community, and these decisions can be more effectively made at the local level.

In countries where access to safer facilities increased only in units with existing access to improved facilities—eg, transitioning from improved wells to piped—the population relying on the worst facility types was effectively left behind. Transitions to the safest facility type have the greatest potential to protect health compared with more moderate transitions.^11^, ^57^ Improved facilities might not prevent environmental contamination and disease transmission after long-term use or in poor environmental conditions.^58^ Although piped water and sewer or septic sanitation have the greatest potential to prevent deaths from enteric diseases,^2^ piped systems are also susceptible to contamination,^59^ and water quality is not currently captured in our estimates. Despite having a smaller effect on health outcomes, transitions from surface water or open defecation to unimproved facilities, nonetheless represent important progress and potentially improved quality of life, including time saved for education and economic productivity.^14^, ^15^ Decision makers would benefit from detailed local information on the differences in increases by type of access to better target future interventions.

Achieving universal access in line with the SDG target is likely to require tailored interventions framed within a broader focus on reducing disparities. Countries such as Mozambique were able to increase mean access to improved water facilities, but although the highest level of access increased, the lowest level was relatively stable from 2000 to 2017, potentially reflecting improvements concentrated in urban centres with large populations where access was already higher. Targeted rural interventions might be needed to serve those with the lowest levels of access, for whom increases in access have not been as substantial. Ethiopia, for example, was able to increase the lowest levels of access to improved water facilities by 2017. In remote communities where a small number of people continue to have no access despite a high national access level, local-level investments in infrastructure are probably most suitable to address this disparity. Conversely, in Nigeria, where large numbers of people concentrated in single units do not have access to safe sanitation, more centralised solutions might best serve these dense urban populations. Examples such as improved water access in Cambodia, which saw pronounced increases in both the lowest access and highest access relative to 2000 levels, potentially present models for adoption elsewhere. Previous studies have identified poor, indigenous, and rural communities as the least likely to have access.^51^, ^60^ Although the disparity in access between urban and rural areas has long been recognised, our estimates underscore the ongoing need for investment in rural water and sanitation.

Our results identified several units in which changes in access to safe water averted relatively few child deaths, or decreased access to safe water led to increased child diarrhoeal mortality. This finding largely reflects decreases in piped water access in some units—potentially driven in part by conflict and instability,^61^ particularly in areas such as in northeastern Nigeria—and suggests that what improvements in access did occur were not for the safest forms of facilities or that the improvements were not of a sufficient magnitude to have a major effect. In addition, our estimates do not capture other elements of safety, such as safe water storage and safe disposal of child faeces, which could drive reductions in diarrhoeal deaths.^4^ Although the absolute number of deaths averted in a unit might not be large, the number of deaths attributable to unsafe water and sanitation in 2017 remains high, indicating that efforts to expand access would also reduce child mortality. Investments in increasing access to improved water and sanitation facilities are likely to have the greatest effect on reducing child deaths due to diarrhoea when coupled with other measures that protect children.^1^, ^26^ Other factors, such as treatment with oral rehydration solution, could have a combined effect with water and sanitation access on preventing child diarrhoeal deaths. Coverage of oral rehydration therapy over the study period has been mapped in a companion article by Wiens and colleagues.^62^ More broadly, strengthening primary health-care systems and addressing social and economic determinants of health will also be imperative to successfully reduce disease and prevent child deaths. Our estimates can help by identifying areas susceptible to disease spread, aiding vaccine-targeting efforts, and assisting disease elimination campaigns in focusing on where they will be most successful. The relationship between access to water and sanitation and the spread of NTDs provides a particular opportunity to coordinate and improve disease prevention efforts across sectors.^10^, ^63^, ^64^ Water, sanitation, and hygiene are also integral to the treatment of some NTDs, such as lymphatic filariasis and podoconiosis.^65^, ^66^ Our estimates facilitate targeting infrastructure investments toward communities with a high burden of NTDs, such as those identified through the Global Trachoma Mapping Project,^67^ and low levels of access to drinking water and sanitation facilities.

The geostatistical nature of this analysis allows for explicit incorporation of geopositioned data, more effectively capturing local variation in access over space and time, compared with studies that use exclusively areal data.^13^ Additionally, the use of a continuation-ratio modelling framework appropriately accounts for the ordinal relationships between indicators of water or sanitation facility type. This study also uses an extensive suite of covariates to leverage the complex relationships between water and sanitation access and environmental, social, and public health correlates at the local level. Our findings highlight the need to increase investment, assess existing interventions, scale up and expand successes, and improve monitoring of access to these facilities. Ultimately, these estimates, in combination with parallel work on oral rehydration therapy,^62^ provide an actionable atlas to progress toward universal access, reduce child diarrhoeal mortality and the spread of disease, and improve wellbeing worldwide.

The data and methodology underlying these results have several limitations. First, SDG 1.4.1 aims to achieve universal access to basic services, and SDG 6 aims to achieve universal access to safely managed services; however, current data are insufficient to produce reliable estimates of these dimensions of access at the spatial and temporal scale presented here. Consequently, this analysis focused on access by facility type classification (figure 1), and our estimates provide a best-case scenario for the SDGs (all improved facilities are safely managed and provide basic services). Second, despite the fine spatial resolution of this study, these results might not fully represent intra-urban disparities in water and sanitation. Third, to incorporate the vast quantity of areal data in a geostatistical framework, areal data were transformed into geopositioned point data over the corresponding geographical area. This method could result in smoothed estimates in areas with predominantly areal data. Fourth, our data do not capture the impacts of conflicts or climate change-related weather events and disasters, and data for locations affected by these factors might not reflect current conditions. Fifth, survey data are subject to known biases and inaccuracies in reporting, and these issues coupled with data scarcity in some locations could affect the accuracy of our estimates. Sixth, our analysis of inequality is limited to variation in access and does not encompass social and economic factors affecting inequality in access. Seventh, uncertainty in existing population estimates affects the precision of our count estimates of access. Finally, although our model generates estimates of uncertainty considering the covariates as well as spatiotemporal trends, uncertainty is not explicitly incorporated from the survey design or the intermediate covariates generated from our stacking procedure (appendix p 9) due to computational limitations.

We plan to adopt the newly updated global indicators of water and sanitation access, including categories of basic and safely managed, by the WHO–UNICEF JMP. Although our study identified the best performing units and diverse modes of improvement across facilities, it was beyond our scope to identify the specific factors and interventions that contributed to these successes. Further research on potential shared characteristics across countries and units achieving high and equitable access could inform potential avenues for policy makers to adopt, particularly in light of the shifting focus from improved facility access to safely managed services. This analysis provides a comprehensive set of estimates across all facility types and locations; additional research with these methods to explore aspects presented here in greater detail would further enable prioritisation and targeting of water and sanitation interventions at the local level.

Despite substantial gains in some regions, accelerated progress will be necessary to achieve universal and equitable access to the safest forms of drinking water and sanitation facilities in line with SDG targets. Sub-Saharan Africa, in particular, would probably benefit from a precision public health approach to increasing access. This analysis improves on traditional national and subnational estimates, providing an analysis of both absolute and relative progress and identifies communities with low access as well as exemplars of improved access at the second administrative level. Our results indicate that vast geographical inequalities persist in both the proportion and number of people with access within countries, as well as in improvements of the quality of facilities over time. Local estimates can guide targeting of disease prevention efforts, particularly vaccines and interventions for nutrition and NTDs, to the communities with the lowest access. Ultimately, our estimates provide a resource for researchers, policy makers, and implementers to improve drinking water and sanitation access at local to national geographical scales, ensuring that all have access to this basic human right.

Correspondence to: Dr Robert C Reiner Jr, Institute for Health Metrics and Evaluation, Department of Health Metrics Science, School of Medicine, University of Washington, Seattle, WA 98121, USA bcreiner@uw.edu

For the **source code** see https://github.com/ihmeuw/lbd/tree/wash-lmic-2020

For the **study data** see http://ghdx.healthdata.org/record/ihme-data/lmic-wash-access-geospatial-estimates-2000-2017

## Data sharing

The source code used to generate estimates is available online. The study data, including full sets of estimates at the first and second administrative levels, are also available online.
