# Supplementary material for: Mapping geographical inequalities in access to drinking water and sanitation facilities in low-income and middle-income countries, 2000–17
Source: Lancet Glob Health. 2020 Aug 19;8(9):e1162–85. doi: 10.1016/S2214-109X(20)30278-3 (PMC7443708; doi:10.1016/S2214-109X(20)30278-3)
Supplement: Supplementary appendix [file mmc1.pdf]

THE LANCET
Global Health

Supplementary appendix

This appendix formed part of the original submission and has been peer reviewed. We post it as supplied by the authors.

Supplement to: Local Burden of Disease WaSH Collaborators. Mapping geographical inequalities in access to drinking water and sanitation facilities in low-income and middle-income countries, 2000–17. *Lancet Glob Health* 2020; **8:** e1162–85.

Mapping geographical inequalities in access to drinking water and sanitation facilities in low-income and middle-income countries, 2000–17

Appendix

Contents

0.0 Gather compliance **6**

1.0 Data **6**

1.1 Model geographies 6

1.2 Data inclusion criteria 6

1.3 Summary of included data sources 6

1.4 Facility type classification 6

1.5 Processing data for modelling 7

1.6 Processing areal data for geostatistical modelling 7

1.7 Creating ordinal indicators 7

1.8 Data vetting 8

1.8.1 Facility type classification *8*

1.8.2 Data validity *8*

2.0 Covariates **8**

2.1 Covariate selection 8

2.2 Modelling covariates via stacked generalisation 9

3.0 Modelling **9**

3.1 Ensemble modelling 9

3.2 Priors 9

3.4 Estimate generation 10

3.5 Modelling national time trends and harmonization 10

3.6 Model validation 10

3.6.1 In-sample validation *10*

3.6.2 Out-of-sample validation *11*

4.0 Supplemental analyses **11**

4.1 Counterfactual analysis of diarrhoeal mortality 11

4.2 Gini coefficient 11

5.0 Appendix Figures **13**

Appendix Figure 1.Geospatial modelling flowchart for water and sanitation 16
Appendix Figure 2. Data processing flow chart 17
Appendix Figure 3. Drinking water facility data availability by type and country, 2000–2017 18
Appendix Figure 4. Sanitation facility data availability by type and country, 2000–2017 22
Appendix Figure 5. Geospatial covariates for water and sanitation 28
Appendix Figure 6. Stacker plots 29
Appendix Figure 7. Finite elements mesh 30
Appendix Figure 8. Mean access to piped water, at 5 × 5-km resolution and second administrative level, 2000 and 2017 31
Appendix Figure 9. Mean access to improved water, at 5 × 5-km resolution and second administrative level, 2000 and 2017 32
Appendix Figure 10. Mean access to unimproved water, at 5 × 5-km resolution and second administrative level, 2000 and 2017 33
Appendix Figure 11. Mean access to surface water, at 5 × 5-km resolution and second administrative level, 2000 and 2017 34
Appendix Figure 12. Mean access to piped sanitation, at 5 × 5-km resolution and second administrative level, 2000 and 2017 35
Appendix Figure 13. Mean access to improved sanitation, at 5 × 5-km resolution and second administrative level, 2000 and 2017 36
Appendix Figure 14. Mean access to unimproved sanitation, at 5 × 5-km resolution and second administrative level, 2000 and 2017 37
Appendix Figure 15. Mean open defecation, at 5 × 5-km resolution and second administrative level, 2000 and 2017 38
Appendix Figure 16. 95% uncertainty intervals for access to piped water, at 5 × 5-km resolution and second administrative level, 2017 39
Appendix Figure 17. 95% uncertainty intervals for access to improved water, at 5 × 5-km resolution and second administrative level, 2017 40
Appendix Figure 18. 95% uncertainty intervals for access to piped sanitation, at 5 × 5-km resolution and second administrative level, 2017 41
Appendix Figure 19. 95% uncertainty intervals for access to improved sanitation, at 5 × 5-km resolution and second administrative level, 2017 42

Appendix Figure 20. Ternary water, at the first administrative and national level, 2000 and 2017......43

Appendix Figure 21. Ternary sanitation, at the first administrative and national level, 2000 and 2017......44

Appendix Figure 22. In sample statistics for piped water model by country and year......46

Appendix Figure 23. In sample statistics for piped water model by first administrative level and year......47

Appendix Figure 24. In sample statistics for piped water model by second administrative level and year......48

Appendix Figure 25. In sample statistics for conditional improved water model by country and year......49

Appendix Figure 26. In sample statistics for conditional improved water model by first administrative level and year......50

Appendix Figure 27. In sample statistics for conditional improved water model by second administrative level and year......51

Appendix Figure 28. In sample statistics for conditional unimproved water model by country and year......52

Appendix Figure 29. In sample statistics for conditional unimproved water model by first administrative level and year......53

Appendix Figure 30. In sample statistics for conditional unimproved water model by second administrative level and year......54

Appendix Figure 31. In sample statistics for piped sanitation model by country and year......55

Appendix Figure 32. In sample statistics for piped sanitation model by first administrative level and year......56

Appendix Figure 33. In sample statistics for piped sanitation model by second administrative level and year......57

Appendix Figure 34. In sample statistics for conditional improved sanitation model by country and year......58

Appendix Figure 35. In sample statistics for conditional improved sanitation model by first administrative level and year......59

Appendix Figure 36. In sample statistics for conditional improved sanitation model by second administrative level and year......60

Appendix Figure 37. In sample statistics for conditional unimproved sanitation model by country and year......61

Appendix Figure 38. In sample statistics for conditional unimproved sanitation model by first administrative level and year 62

Appendix Figure 39. In sample statistics for conditional unimproved sanitation model by second administrative level and year 63

Appendix Figure 40. Out-of-sample statistics for piped water model by country and year 65

Appendix Figure 41. Out-of-sample statistics for piped water model by first administrative level and year 66

Appendix Figure 42. Out-of-sample statistics for piped water model by second administrative level and year 67

Appendix Figure 43. Out-of-sample statistics for conditional improved water model by country and year 68

Appendix Figure 44. Out-of-sample statistics for conditional improved water model by first administrative level and year 69

Appendix Figure 45. Out-of-sample statistics for conditional improved water model by second administrative level and year 70

Appendix Figure 46. Out-of-sample for conditional unimproved water model by country and year 71

Appendix Figure 47. Out-of-sample for conditional unimproved water model by first administrative level and year 72

Appendix Figure 48. Out-of-sample for conditional unimproved water model by second administrative level and year 73

Appendix Figure 49. Out-of-sample for piped sanitation model country and year 74

Appendix Figure 50. Out-of-sample for piped sanitation model first administrative level and year 75

Appendix Figure 51. Out-of-sample for piped sanitation model second administrative level and year 76

Appendix Figure 52. Out-of-sample statistics for conditional improved sanitation model by country and year 77

Appendix Figure 53. Out-of-sample statistics for conditional improved sanitation model by first administrative level and year 78

Appendix Figure 54. Out-of-sample statistics for conditional improved sanitation model by second administrative level and year 79

Appendix Figure 55. Out-of-sample statistics for conditional unimproved sanitation model by country and year 80

Appendix Figure 56. Out-of-sample statistics for conditional unimproved sanitation model by first administrative level and year......81

Appendix Figure 57. Out-of-sample statistics for conditional unimproved sanitation model by second administrative level and year......82

Appendix Figure 58. Correlation between improved water and World Health Organization Joint Monitoring Programme improved water estimates......83

Appendix Figure 59. Correlation between improved sanitation and World Health Organization Joint Monitoring Programme improved sanitation estimates......84

Appendix Figure 60. Correlation between open defecation and World Health Organization Joint Monitoring Programme open defecation estimates......85

Appendix Figure 61. All indicator data validation plot......86

Appendix Figure 62. Indicator-specific data validation plot......87

Appendix Figure 63. Rate, ratio, and percentage of deaths averted by access to improved drinking water facility at the second-administrative-unit level......88

Appendix Figure 64. Rate, ratio, and percentage of deaths averted by access to improved sanitation facility at the second-administrative-unit level......89

6.0 Appendix Tables......91

7.0 Appendix References......149

8.0 Author contributions......150

9.0 Piped water proportion by country......157

10.0 Improved water proportion by country......2191

11.0 Sewer or septic sanitation proportion by country......3208

12.0 Improved sanitation proportion by country......4416

13.0 Open defecation proportion by country......5624

0.0 Gather compliance

Please see Appendix Table 1 for discussion on how this study meets the conditions for GATHER guidelines.

1.0 Data

1.1 Model geographies

This analysis selected 88 countries for water and 89 countries for sanitation based on their Socio-demographic Index (SDI) (see Appendix Table 2).[1] The SDI is a measure of education, fertility, and income. Countries included were in the middle, lower-middle, or low SDI quintiles with certain exceptions. China and Libya were included despite higher-middle SDI status to create better geographic continuity. Countries were excluded due to lack of available data in the Global Health Data Exchange (GHDx)[2] or to support the modeling paradigm from either the water or sanitation analysis or both. We also excluded island nations with fewer than 1 million inhabitants, which were the nations of Fiji, São Tomé and Príncipe, Solomon Islands, Maldives, Vanuatu, Samoa, Saint Lucia, Kiribati, St. Vincent and the Grenadines, Grenada, Micronesia, Tonga, Seychelles, Dominica, Marshall Islands, and American Samoa. These countries were typically data-scarce and did not have sufficient geographic continuity for a geospatial analytic approach to be advantageous over a national one.

We subdivided the spatial modelling domain by country (see Appendix Figure 3). By modelling each country individually, we were able to account for potential non-stationarity in the relationships between covariates and the outcome across the modelling domain as each country would be modelled independently. Furthermore, subdividing the modeling domain allowed for computational feasibility.

1.2 Data inclusion criteria

For a survey or census to be considered for this analysis, we required that it fit our country inclusion criteria outlined above, included geography information more granular than the national level, had data collected during the time frame of 2000 to 2017, had survey weights if the survey was not self-weighted, and had data on household drinking water source or toilet type. Furthermore, sources were only included for modelling if they were representative of the entire population during the time period and across the geographic area of measurement. Additionally, certain sources were excluded if the associated estimates seemed implausible based on expert review of estimates and comparison with other sources (including WHO-UNICEF Joint Monitoring Programme [JMP] estimates) in the same country and time period (see Appendix Section 1.8.2). After screening 2,175 household survey and census sources that were tagged for water, sanitation, and hygiene relevance in the GHDx,[3] 833 sources met all of the inclusion criteria and were extracted and collated for analysis (see Appendix Figure 2). They were then further vetted and outliers were removed from modelling.

1.3 Summary of included data sources

The household and census surveys used in modelling access to water and sanitation can be found at http://ghdx.healthdata.org/record/ihme-data/lmic-drinking-water-and-sanitation-facilities-access-geospatial-estimates-2000-2017 and are visualised in Appendix Figures 4a–e and 5a–e respectively. 501 data sources from 88 countries were included in the water model, and 457 data sources from 89 countries were included in the sanitation model.

1.4 Facility type classification

The types of water and sanitation facilities reported were categorised into the standard indicators developed by the WHO-UNICEF JMP.[4] Each type of drinking water facility was classified as piped, improved well, unimproved well, improved spring, unimproved spring, other improved, other unimproved, or surface water.

Similarly, each sanitation facility was classified as sewer or septic, other improved flush toilet, unimproved flush toilet, improved latrine, unimproved latrine, other improved, other unimproved, or open defecation.

Wells, springs, flush toilets, and latrines can be categorised as improved or unimproved depending on their respective construction. When responses were ambiguous regarding the details of their construction, summary mean access was calculated at each space-time location for ambiguous versions of the corresponding facility (ambiguous wells, ambiguous latrines, etc.). Then these estimates were divided into improved and unimproved based on the ratio of improved to unimproved of the corresponding facility prevalent in the same country. If a country had less than five unique data sources, the ratio was derived from across the modelling region the data source was in. For example, the mean prevalence of ambiguous latrines in Nigeria was divided into prevalence of improved and unimproved latrines based on the ratio of improved to unimproved latrines in Nigeria from other data sources. These estimates were then combined with the overall access levels to improved and unimproved facilities correspondingly.

After classification, the facility type data were appropriately combined in accordance with JMP definitions to yield the respective water and sanitation indicators. This resulted in piped, other improved, unimproved, and surface water for water indicators, and sewer or septic, other improved, unimproved, and open defecation for sanitation indicators.

1.5 Processing data for modelling

Data corresponding to each georeferenced point were summarised as survey weighted means. For data without corresponding GPS coordinates, data were summarised across the smallest spatial area the data were representative over to produce areal estimates. Household sizes and survey weights were used to produce weighted means of access representative at the individual level. The household sizes and survey weights were further used in a Kish approximation[5] of an effective sample size for each mean in order to account for the complex survey design of most of the data used.

When household size information was missing, the corresponding cluster of data was adjusted as follows. Overall, data were tagged as urban, rural, or unspecified. For each category, logistic regressions were fit on means calculated with and without household sizes (assuming household size equals 1). A single indicator variable was used as a covariate. The ratios obtained as the fixed effect corresponding to the indicator variable acted as an adjustment scalar for adjusting urban, rural, or unspecified data with missing household size information. Additionally, if more than 20% of a cluster was missing information on water source or sanitation facility, we dropped the cluster from the analysis. Furthermore, if a cluster or a data source was missing weights and the source used a weighted sampling strategy, we excluded the source

1.6 Processing areal data for geostatistical modelling

For areal data, 10,000 locations were randomly sampled from the area using population values from WorldPop[6] raster to weight the sampled points. K-means clustering was performed over these points to generate integration points (1 point per 1,000 WorldPop grid cells encompassing the area) to be used in the modelling. Weights were assigned to integration points as the proportion of the original 10,000 points that entered the k-means cluster. Each of these integration points was assigned the areal mean. The integration points were included in the input dataset for further modelling. In this manner, the spatial variation of covariates could be leveraged and areal data could be incorporated into the geostatistical model. The above resampling method used is consistent with the resampling conducted in the previously published geospatial modelling of diphtheria-pertussis-tetanus vaccine coverage.[7]

1.7 Creating ordinal indicators

In order to appropriately account for the mutually exclusive and collectively exhaustive nature of the indicators, we used a continuation ratio ordinal regression framework. Data were processed to generate a set

of independent and conditional variables that were subsequently modelled. The following outlines how this framework was implemented for the water indicators:

$$Water\ facilities\colon \{Piped, Other\ Improved, Unimproved, Surface\}$$
$$Modeled\ Variables\colon \{Piped, Other\ Improved_{conditional}, Unimproved_{conditional}\}$$
$$1 = Piped + \ Other\ Improved + Unimproved + Surface$$
$$Other\ Improved_{conditional} = \frac{Other\ Improved}{1 - Piped}$$
$$Unimproved_{conditional} = \frac{Unimproved}{1 - Piped - Other\ Improved}$$

Sanitation indicators were also modelled via variables generated in an ordinal continuation ratio framework as above. In the case of sanitation, sewer or septic sanitation was the independent category just as piped water was in the case of water.

1.8 Data vetting

1.8.1 Facility type classification

Prior to modelling, all categorisations of types of water and sanitation facilities by the JMP standard indicators were thoroughly reviewed. Using the JMP classifications as a guide, every unique type of water and sanitation facility was independently scrutinised to ensure proper categorisation. In the case of facility types in a foreign language, translations were checked and verified through survey documentation or Google Translate. These final vetted lists of categorised drinking water and sanitation facilities were used in subsequent data vetting and model runs.

1.8.2 Data validity

Following the vetting of indicator string matching, all surveys were systematically reviewed for data quality. Country-specific diagnostic plots were produced for water and sanitation plotting each data source's national estimate of each water and sanitation indicator (see Appendix Figure 62). The estimate for each indicator from each data source was compared to the other available data sources for that country for similar years, as well as estimates for the corresponding indicator and country from the JMP (see Appendix Figure 63). If the data source's estimate was considered implausibly different from other sources as well as the JMP estimate, then the source was excluded. Each country-specific diagnostic plot was independently reviewed, considering country and time trends, as well as whether a survey was nationally or subnationally representative. After data source exclusions, of the 826 data sources extracted and collated for inclusion in the water analysis, 501 sources were included in the final model dataset. Similarly, of the 747 data sources extracted and collated for inclusion in the sanitation analysis, 457 sources were included in the final model dataset (see Appendix Figure 2).

2.0 Covariates

2.1 Covariate selection

The first stage of modelling used 7 covariates, composed of various environmental and social indicators (see Appendix Tables 3–5). Each covariate was formatted at a 5 × 5-km resolution from 2000 to 2017 (see Appendix Figure 6). They included travel time to nearest settlement, aridity, elevation, urbanicity, length of growing season, irrigation, and time.

In the first stage, for each country, three types of models were fit to the data: generalised additive models (GAM), boosted regression trees (BRT), and lasso regression. In doing so, we attempted to account for the varying and potentially non-linear relationship between each of the covariates and the outcome indicator. Each of the seven covariates was used in the BRT and lasso regression model.

2.2 Modelling covariates via stacked generalisation

As noted above, for each country, three models were fit to the data: GAM, BRT, and lasso regression. Each model was fit using five-fold cross-validation (out-of-sample) as well as on the entire dataset at once (in-sample). Out-of-sample predictions were generated from each model and logit transformed for subsequent use as covariates in the geostatistical model. The logit transformation was conducted to ensure the covariate and the outcome water and sanitation indicator data would be in the same functional space. The in-sample predictions from these "sub-models" were used as covariates when generating predictions using the fitted relationships from the geostatistical model.

3.0 Modelling
3.1 Ensemble modelling

A Bayesian spatiotemporal generalised linear regression model was used to fit the binomial data of access to water and sanitation using a logit link function in each country. The model was defined as follows:

$$C_i|p_i,\, N_i \sim Binomial(p_i,\, N_i)$$
$$logit(p_i) = \beta_0 + \boldsymbol{\beta X_i} + \epsilon_{GP_i}$$
$$\epsilon_i \sim N(0, \sigma^2_{nug})$$
$$\epsilon_i \sim N(0, \sigma^2_{ctry})$$
$$\epsilon_{GP}|\boldsymbol{\Sigma}_{\textbf{space}}, \sim GP(0,\, \boldsymbol{\Sigma}_{\textbf{space}})$$
$$\boldsymbol{\Sigma}_{\textbf{space}} = \frac{2^{1-\nu}}{\tau \times \Gamma(\nu)} \times (\kappa \boldsymbol{D})^{\nu} \times \mathrm{K}_{\nu}(\kappa \boldsymbol{D})$$

.

Within each country, the number of individuals at cluster, C_i, from a sample of individuals, N_i, were modelled as binomial counts. The counts, C_i, probabilities, p_i, predictions, and residuals, ϵ, were all indexed in space and time. The logit access, $logit(p_i)$, was modelled as a linear combination of the out-of-sample predictions from three sub-models, $\boldsymbol{X}$ and a spatial Gaussian process, ϵ_{GP_i}. The spatial Gaussian process had a Matérn spatial process. This allowed for the correlation structure in the residuals to be leveraged for improved predictions. The posterior distributions were fit using R-INLA[8,9] with the stochastic partial differential equation (SPDE) approximation to Gaussian process regression. Data points from sources that were not nationally representative were not included in estimation of the linear fixed effects, $\boldsymbol{X}$, but only in the spatial Gaussian process, ϵ_{GP_i}.

3.2 Priors

The following priors were used for all models:

- $\beta_0 \sim N(\mu = 0, \sigma^2 = 1000)$,
- $\boldsymbol{\beta} \sim^{iid} N\left(\mu = \frac{1}{\#\text{ ensemble models}}, \sigma^2 = 1000\right)$
- $\theta_1 = \log(\tau) \sim N(\mu_{\theta_1}, \sigma^2_{\theta_1})$
- $\theta_2 = \log(\kappa) \sim N(\mu_{\theta 2}, \sigma^2_{\theta_2})$

Uncorrelated multivariate normal priors were used as determined by INLA based on the finite elements mesh construction for the log-transformed spatial hyperparameters κ and τ. The mean (μ) and variance (σ^2) parameters for the hyperpriors selected by INLA for each country can be found in Appendix Tables 7–12. α and γ in the *loggamma* distribution represent scale and shape, respectively.

3.4 Estimate generation

Models were fit in INLA consistent with methods used in the previously published geospatial modelling of diphtheria-pertussis-tetanus vaccine coverage.[10]

Georeferenced data points were modelled with weights set to 1. Integration points as a result of k-means clustering were modelled with weights corresponding to the weight generated from the k-means algorithm as described above. The weights for each data point represented the relative contribution to the log-likelihood. In this manner, points as a result of resampling contributed less than true georeferenced points to the model fit. Additionally, the contribution of points from the resampling process to the log-likelihood estimation was diffused across the polygon.

The covariates for the model were the out-of-sample predictions from each sub-model as described above. After the fitting process, estimates were generated by taking 250 draws from the posterior distribution and using the in-sample predictions from the sub-models as covariates to generate prediction surfaces across the modelled country. The 2.5th and 97.5th percentiles were calculated across draws for each grid cell to generate 95% uncertainty intervals in addition to means. Grid-cell-level estimates were aggregated to the second administrative level (eg, districts, counties) and first administrative level (eg, states, provinces) by calculating population-weighted averages across each respective administrative boundary.

3.5 Modelling national time trends and harmonization

To capture national time trends in access to water and sanitation, separate generalized additive models were fitted to national aggregates of data sources that were nationally representative for each country. The relationship with covariates were modelled as thin-plate splines in countries where there was more than three years of data. In countries where they had three or fewer years of data available in the modeling period, the relationships were estimated as linear fixed effects.

3.6 Model validation

For model validation, the INLA model was tested in and out of sample as described below before the estimates were harmonized to national time trends. This was done to test the performance of the geostatistical model itself. Certain countries were excluded by indicator as their data could not support five-fold cross validation and the exclusions represent a small minority of the full sample of the data. The exclusions are as follows: 1) Bolivia, Brazil, Gabon, Indonesia, Cambodia, Madagascar, Peru, Papua New Guinea, Tanzania, South Africa, and Vietnam for piped water, 2) Bolivia, Brazil, Botswana, Central African Republic, Gambia, Indonesia, Kyrgyzstan, Morocco, and Mongolia for conditional improved water, 3) Gambia, Jordan, and Libya for conditional unimproved water, 3) Mexico and Swaziland for sewer or septic sanitation, 4) Mexico for conditional improved sanitation, and 5) Libya and Mozambique for conditional unimproved sanitation.

3.6.1 In-sample validation

To assess the in-sample fit of our model, we calculated mean error (ME, or bias), root-mean-squared-error (RMSE), and 95% coverage of our predictive intervals (the proportion of observed data that fall within our predicted 95% uncertainty intervals). We present these calculations at the first and second administrative-level units for 2000, 2005, 2010, and 2017 for each indicator and country in Appendix Tables 13–18. Appendix Figures 23–40 show in sample prediction versus observed data.

3.6.2 Out-of-sample validation

We examined the predictive validity of our modelling strategy using five-fold out-of-sample cross-validation. Folds were created by randomly assigning entire first administrative-level units, stratified by country, to one of five folds. For each modelling country, we ran the entire modelling process once per fold, in addition to the full in-sample runs described above, generating a complete set of out-of-sample predictions. Using these out-of-sample predictions, we then calculated mean error (ME, or bias), root-mean-squared-error (RMSE, which summarises total variance), and 95% coverage of our predictive intervals (the proportion of observed out-of-sample data that fall within our predicted 95% credible intervals) aggregated to the spatial holdout level. Appendix Figures 41–58 show out-of-sample prediction versus observed data. Similarly, Appendix Tables 19–24 summarise out-of-sample statistics.

4.0 Supplemental analyses

4.1 Counterfactual analysis of diarrhoeal mortality

In order to assess the impacts of changes in access to water and sanitation, we conducted counterfactual analyses in a comparative risk assessment framework of the impact of water and sanitation access on diarrhoeal mortality. First, we used risk ratios from the Global Burden of Diseases, Injuries, and Risk Factors Study 2017[9] associated with unsafe water and sanitation to calculate the attributable fraction of diarrhoeal deaths to unsafe water and sanitation for each second administrative-level unit in 2017 as follows.

Furthermore, we constructed a counterfactual attributable fraction by conducting the above calculation with levels of water and sanitation access observed in 2000. We calculated the number of deaths averted in 2017 due to changes since 2000 as the difference in the attributable deaths between the observed and the counterfactual (see Appendix Figures 64–65).

$$PAF_{counterfactual} = \frac{\sum(access_{2000} * risk\ ratios) - 1}{\sum(access_{2000} * risk\ ratios)}$$

$$PAF_{observed} = \frac{\sum(access_{2017} * risk\ ratios) - 1}{\sum(access_{2017} * risk\ ratios)}$$

$$Number\ averted = deaths_{2017} * \frac{(1 - PAF_{observed})}{(1 - PAF_{counterfactual})(PAF_{counterfactual} - PAF_{observed})}$$

$$Rate\ averted = \frac{Number\ averted}{Total\ Population} * 1000$$

$$Percent\ averted = \frac{Number\ averted}{Numer\ averted + Observed\ Deaths}$$

$$Ratio\ averted = \frac{Number\ averted}{Observed\ Deaths - Number\ Averted}$$

4.2 Gini coefficient

The Gini coefficient[11] is a popular measure of inequality, originally applied to economics. For income inequality, the Gini coefficient assesses the magnitude of disparity between the richest and poorest individuals. In this context, equality corresponds to wealth uniformly distributed across the population, and inequality corresponds to a small number of individuals possessing the majority of the wealth. The Gini coefficient for wealth can be calculated directly from the Lorenz curve, which sorts individuals by their income and plots cumulative percentages of individuals against their corresponding fraction of wealth. For the purposes of this study, "wealth" is defined as improved water or improved sanitation for each second administrative-level unit. The Gini coefficient is then calculated as one minus twice the area under the Lorenz curve. An alternative formulation of the Gini coefficient calculates the relative mean absolute

difference in wealth, and then observes that the Gini coefficient is half the resulting quantity. If x_i is the wealth of the ith individual (out of n individuals), the Gini coefficient, G, is given as:

$$G = \frac{\sum_{i=1}^{n}\sum_{j=1}^{n}|x_i - x_j|}{2n\sum_{i=1}^{n}x_i}$$

5.0 Appendix Figures

Appendix Figure 1.Geospatial modelling flowchart for water and sanitation 16
Appendix Figure 2. Data processing flow chart 17
Appendix Figure 3. Drinking water facility data availability by type and country, 2000–2017 18
Appendix Figure 4. Sanitation facility data availability by type and country, 2000–2017 ..22
Appendix Figure 5. Geospatial covariates for water and sanitation 28
Appendix Figure 6. Stacker plots 29
Appendix Figure 7. Finite elements mesh...30
Appendix Figure 8. Mean access to piped water, at 5 × 5-km resolution and second administrative level, 2000 and 2017 31
Appendix Figure 9. Mean access to improved water, at 5 × 5-km resolution and second administrative level, 2000 and 2017 32
Appendix Figure 10. Mean access to unimproved water, at 5 × 5-km resolution and second administrative level, 2000 and 2017 33
Appendix Figure 11. Mean access to surface water, at 5 × 5-km resolution and second administrative level, 2000 and 2017 34
Appendix Figure 12. Mean access to piped sanitation, at 5 × 5-km resolution and second administrative level, 2000 and 2017 35
Appendix Figure 13. Mean access to improved sanitation, at 5 × 5-km resolution and second administrative level, 2000 and 2017 36
Appendix Figure 14. Mean access to unimproved sanitation, at 5 × 5-km resolution and second administrative level, 2000 and 2017 37
Appendix Figure 15. Mean open defecation, at 5 × 5-km resolution and second administrative level, 2000 and 2017 38
Appendix Figure 16. 95% uncertainty intervals for access to piped water, at 5 × 5-km resolution and second administrative level, 2017...39
Appendix Figure 17. 95% uncertainty intervals for access to improved water, at 5 × 5-km resolution and second administrative level, 2017...40
Appendix Figure 18. 95% uncertainty intervals for access to piped sanitation, at 5 × 5-km resolution and second administrative level, 2017...41
Appendix Figure 19. 95% uncertainty intervals for access to improved sanitation, at 5 × 5-km resolution and second administrative level, 2017 42
Appendix Figure 20. Ternary water, at the first administrative and national level, 2000 and 2017 43
Appendix Figure 21. Ternary sanitation, at the first administrative and national level, 2000 and 2017..44
Appendix Figure 22. In sample statistics for piped water model by country and year.......46

Appendix Figure 23. In sample statistics for piped water model by first administrative level and year 47
Appendix Figure 24. In sample statistics for piped water model by second administrative level and year 48
Appendix Figure 25. In sample statistics for conditional improved water model by country and year 49
Appendix Figure 26. In sample statistics for conditional improved water model by first administrative level and year 50
Appendix Figure 27. In sample statistics for conditional improved water model by second administrative level and year 51
Appendix Figure 28. In sample statistics for conditional unimproved water model by country and year 52
29Appendix Figure 29. In sample statistics for conditional unimproved water model by first administrative level and year 53
Appendix Figure 30. In sample statistics for conditional unimproved water model by second administrative level and year....54
Appendix Figure 31. In sample statistics for piped sanitation model by country and year 55
Appendix Figure 32. In sample statistics for piped sanitation model by first administrative level and year 56
Appendix Figure 33. In sample statistics for piped sanitation model by second administrative level and year 57
Appendix Figure 34. In sample statistics for conditional improved sanitation model by country and year 58
Appendix Figure 35. In sample statistics for conditional improved sanitation model by first administrative level and year 59
Appendix Figure 36. In sample statistics for conditional improved sanitation model by second administrative level and year....60
Appendix Figure 37. In sample statistics for conditional unimproved sanitation model by country and year 61
Appendix Figure 38. In sample statistics for conditional unimproved sanitation model by first administrative level and year 62
Appendix Figure 39. In sample statistics for conditional unimproved sanitation model by second administrative level and year....63
Appendix Figure 40. Out-of-sample statistics for piped water model by country and year 65
Appendix Figure 41. Out-of-sample statistics for piped water model by first administrative level and year....66
Appendix Figure 42. Out-of-sample statistics for piped water model by second administrative level and year....67
Appendix Figure 43. Out-of-sample statistics for conditional improved water model by country and year 68

Appendix Figure 44. Out-of-sample statistics for conditional improved water model by first administrative level and year 69
Appendix Figure 45. Out-of-sample statistics for conditional improved water model by second administrative level and year 70
Appendix Figure 46. Out-of-sample for conditional unimproved water model by country and year 71
Appendix Figure 47. Out-of-sample for conditional unimproved water model by first administrative level and year 72
Appendix Figure 48. Out-of-sample for conditional unimproved water model by second administrative level and year 73
Appendix Figure 49. Out-of-sample for piped sanitation model country and year 74
Appendix Figure 50. Out-of-sample for piped sanitation model first administrative level and year75
Appendix Figure 51. Out-of-sample for piped sanitation model second administrative level and year 76
Appendix Figure 52. Out-of-sample statistics for conditional improved sanitation model by country and year 77
Appendix Figure 53. Out-of-sample statistics for conditional improved sanitation model by first administrative level and year 78
Appendix Figure 54. Out-of-sample statistics for conditional improved sanitation model by second administrative level and year 79
Appendix Figure 55. Out-of-sample statistics for conditional unimproved sanitation model by country and year 80
Appendix Figure 56. Out-of-sample statistics for conditional unimproved sanitation model by first administrative level and year 81
Appendix Figure 57. Out-of-sample statistics for conditional unimproved sanitation model by second administrative level and year 82
Appendix Figure 58. Correlation between improved water and World Health Organization Joint Monitoring Programme improved water estimates 83
Appendix Figure 59. Correlation between improved sanitation and World Health Organization Joint Monitoring Programme improved sanitation estimates 84
Appendix Figure 60. Correlation between open defecation and World Health Organization Joint Monitoring Programme open defecation estimates 85
Appendix Figure 61. All indicator data validation plot 86
Appendix Figure 62. Indicator-specific data validation plot 87
Appendix Figure 63. Rate, ratio, and percentage of deaths averted by access to improved drinking water facility at the second-administrative-unit level 88
Appendix Figure 64. Rate, ratio, and percentage of deaths averted by access to improved sanitation facility at the second-administrative-unit level 89

Appendix Figure 1.Geospatial modelling flowchart for water and sanitation

The geospatial modelling process consists of four sections. First (in blue), we compile all available survey data that can be referenced to a coordinate/point (eg, survey cluster) or small polygon unit and calculate access to drinking water sources and sanitation facilities at the respective level (Section 1.0). Data matched to polygons are resampled into pseudo points using a k-means clustering algorithm (Section 1.6). Covariates are subsequently merged to the points and pseudo points via a spatial join. Second (green), we use the point data and their associated covariates and a stacked generalisation ensemble model (Section 2.2). The children models, boosted regression trees, generalised additive models, and lasso regression are fit using a 5-fold cross-validation process (Section 3.1). The cross-validated predictions from each model then serve as the covariate values for the main/parent model (spatiotemporal GPR model) (Section 3.1). The predictions from when the child models are fit on all the data (rather than 4/5[ths] implied by the cross-validation) are then used to create posterior predictions of access to water and sanitation facilities prevalence in a continuous grid for the years 2000–2017 (Section 3.4). Finally (orange), we aggregate our estimates to first administrative-level units (Section 3.4).

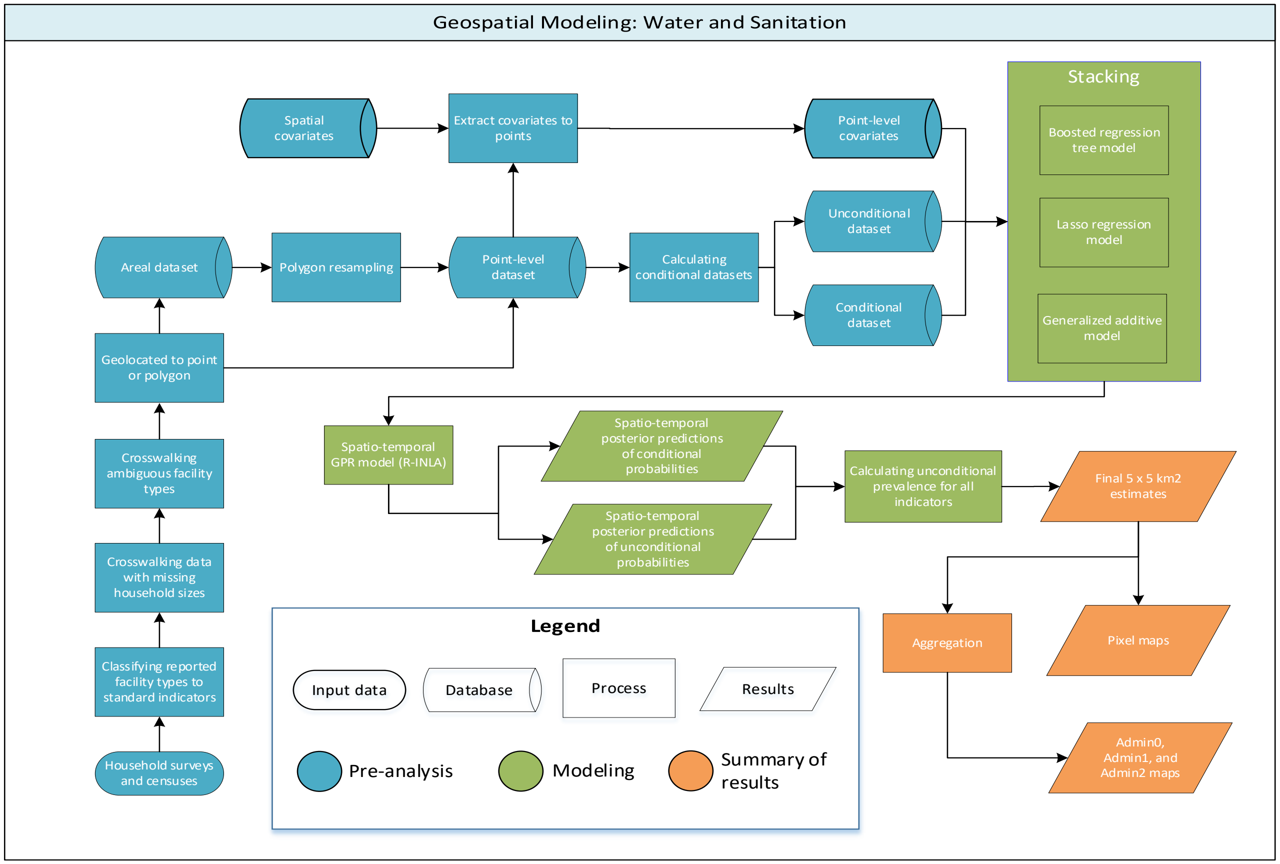

Appendix Figure 2. Data processing flow chart

Flow chart of source identification, inclusion, and exclusion criteria. This chart illustrates the database searched for this analysis, the GHDx,[12] the resulting number of potential sources identified from the search, and the number and reasons for excluding sources based on the inclusion criteria for this analysis (Section 1.2). We excluded data after reviewing its data collection methodology and based on evaluating against other available data for the country and known plausibility of change in water and sanitation access.

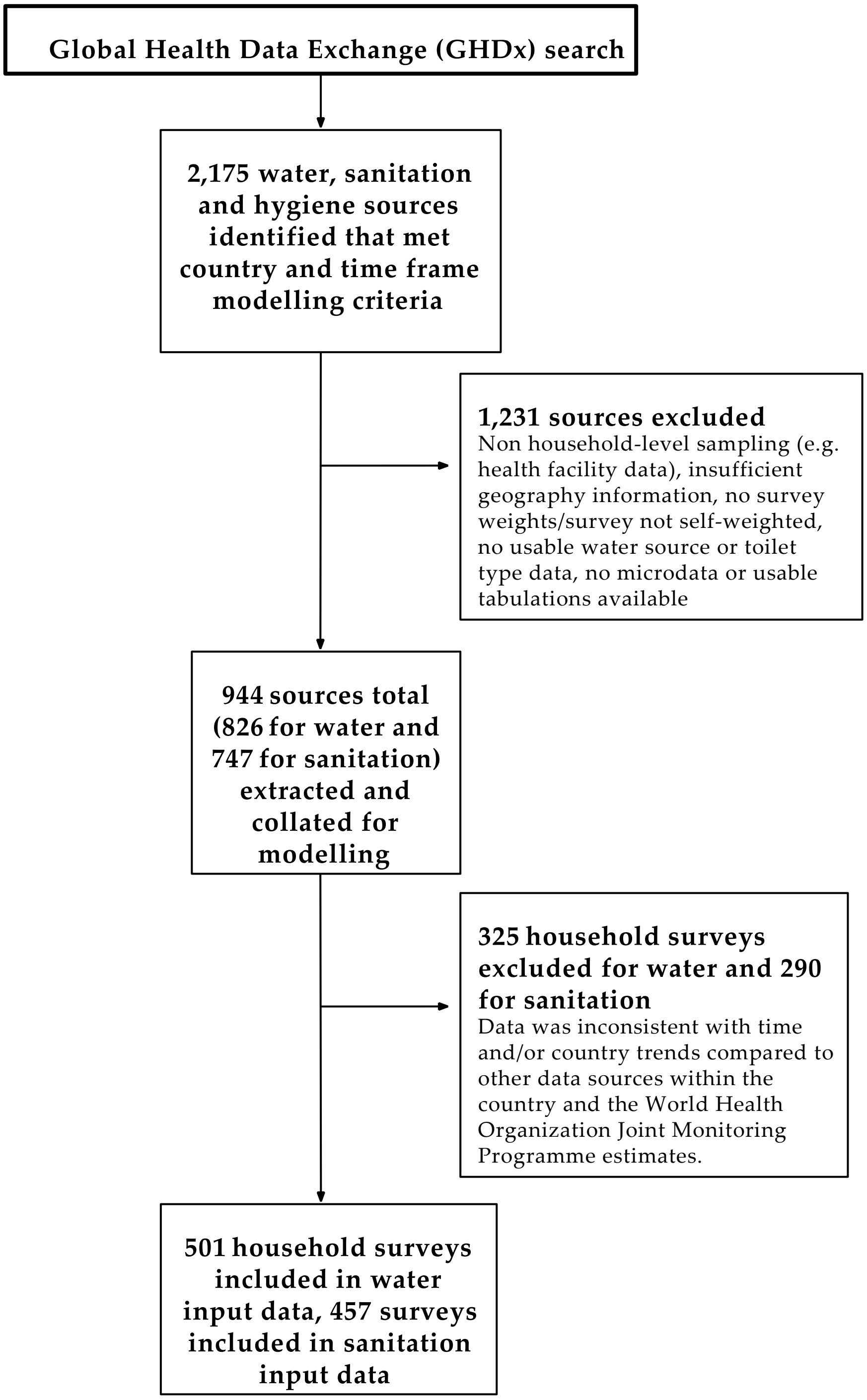

Appendix Figure 3. Drinking water facility data availability by type and country, 2000–2017

All data are shown by country and year of survey and mapped at their corresponding geopositioned coordinate or area. In the left panel, the total number of points and polygons (areal) for each country are plotted by data source, type, and sample size. Sample size represents the number of individual microdata records for each survey. In the right panel, mean access to drinking water source for the input coordinate or area is mapped. Figure **a)** shows drinking water facility data availability in Africa by type and country from 2000 to 2017. Figure **b)** shows drinking water facility data availability in Latin America and the Caribbean by type and country from 2000 to 2017. Figure **c)** shows drinking water facility data availability in southeast Asia by type and country from 2000 to 2017. Figure **d)** shows drinking water facility data availability in south Asia by type and country from 2000 to 2017. Figure **e)** shows drinking water facility data availability in the Middle East and central Asia by type and country from 2000 to 2017.

a)

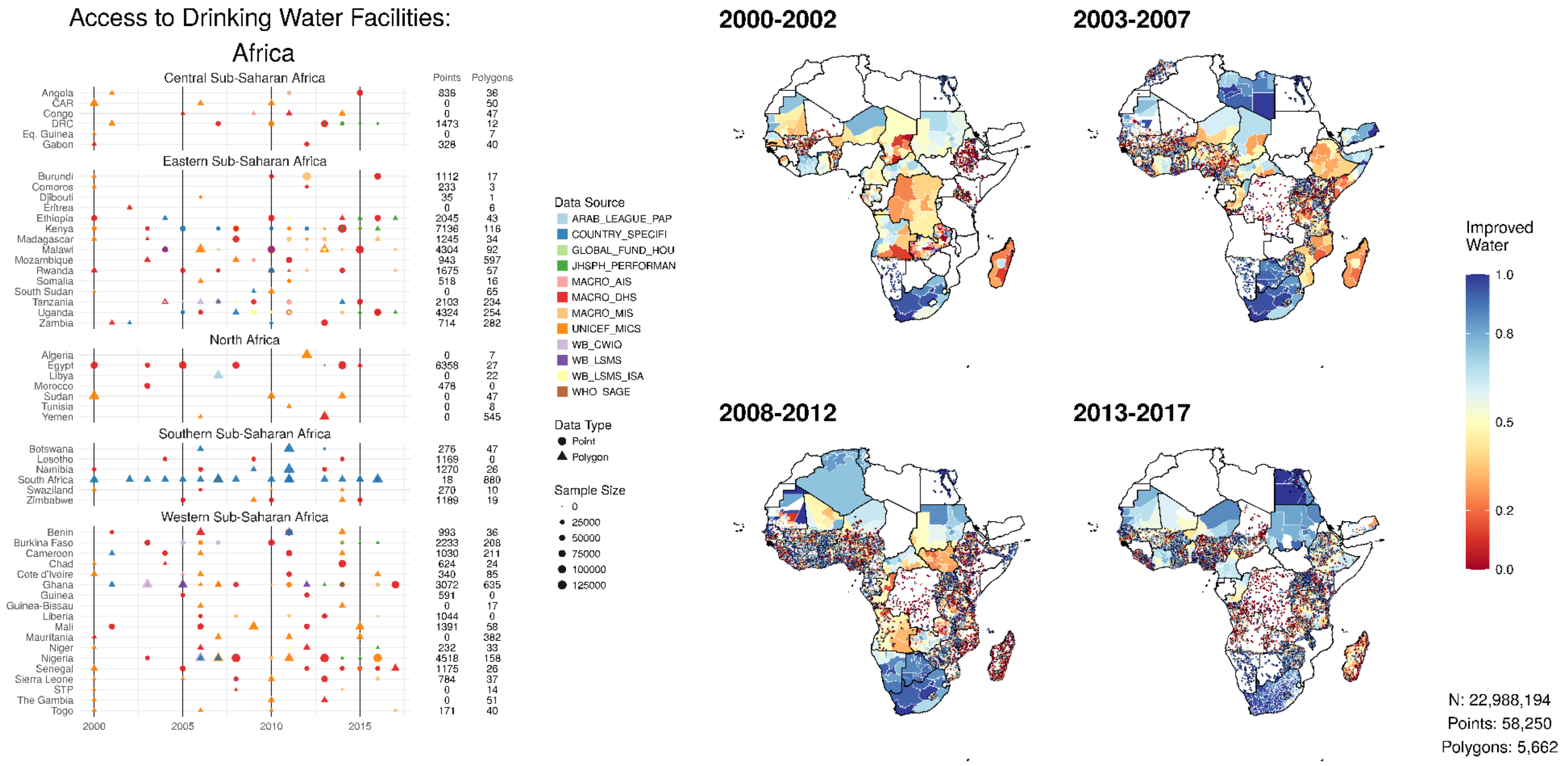

b)

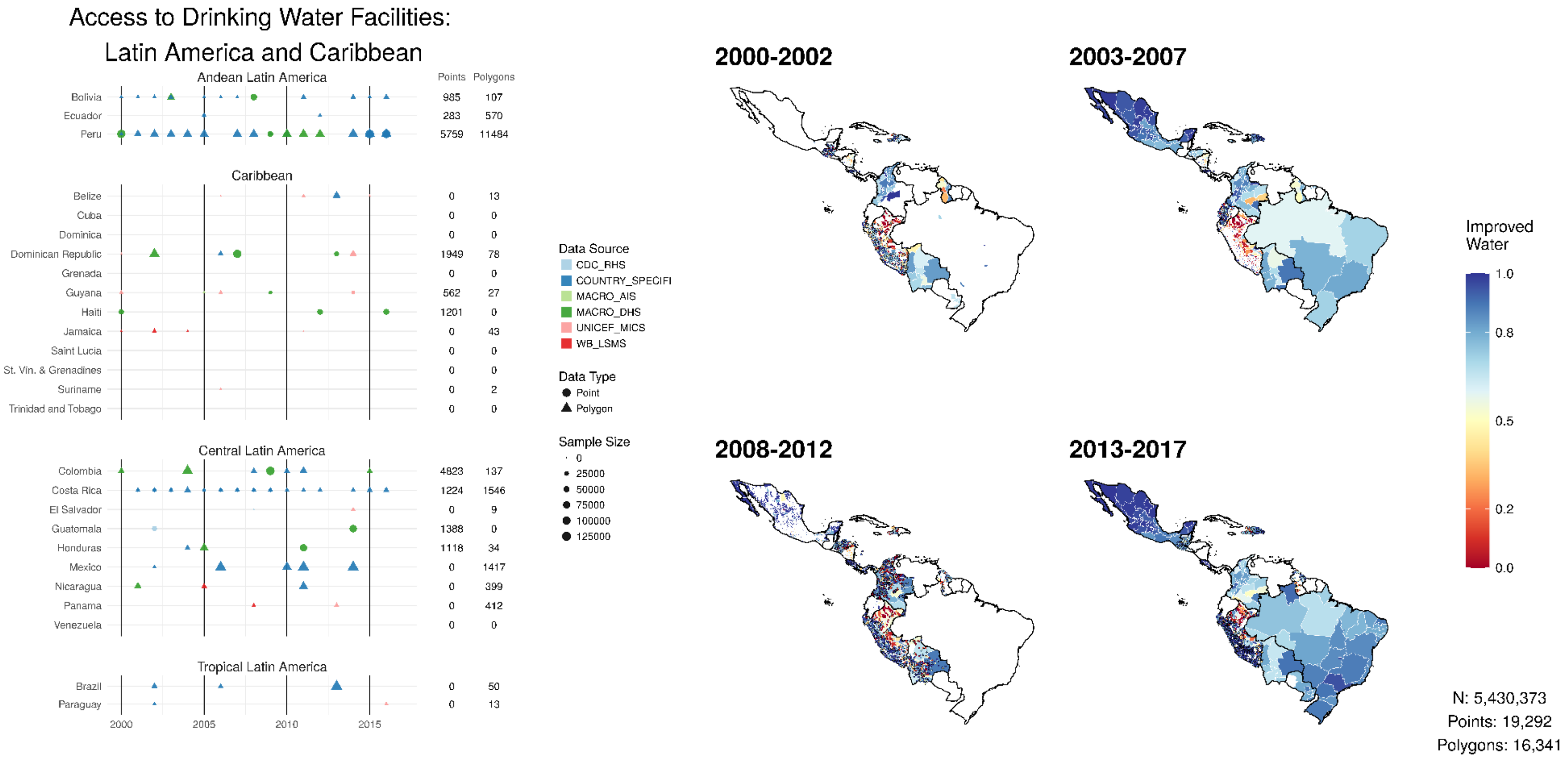
Access to Drinking Water Facilities:
Latin America and Caribbean
Andean Latin America
Points Polygons
Bolivia 985 107
Ecuador 283 570
Peru 5759 11484
Caribbean
Belize 0 13
Cuba 0 0
Dominica 0 0
Dominican Republic 1949 78
Grenada 0 0
Guyana 562 27
Haiti 1201 0
Jamaica 0 43
Saint Lucia 0 0
St. Vin. & Grenadines 0 0
Suriname 0 2
Trinidad and Tobago 0 0
Central Latin America
Colombia 4823 137
Costa Rica 1224 1546
El Salvador 0 9
Guatemala 1388 0
Honduras 1118 34
Mexico 0 1417
Nicaragua 0 399
Panama 0 412
Venezuela 0 0
Tropical Latin America
Brazil 0 50
Paraguay 0 13
2000
2005
2010
2015
Data Source
CDC_RHS
COUNTRY_SPECIFI
MACRO_AIS
MACRO_DHS
UNICEF_MICS
WB_LSMS
Data Type
Point
Polygon
Sample Size
0
25000
50000
75000
100000
125000
2000-2002
2003-2007
2008-2012
2013-2017
Improved Water
1.0
0.8
0.5
0.2
0.0
N: 5,430,373
Points: 19,292
Polygons: 16,341

c)

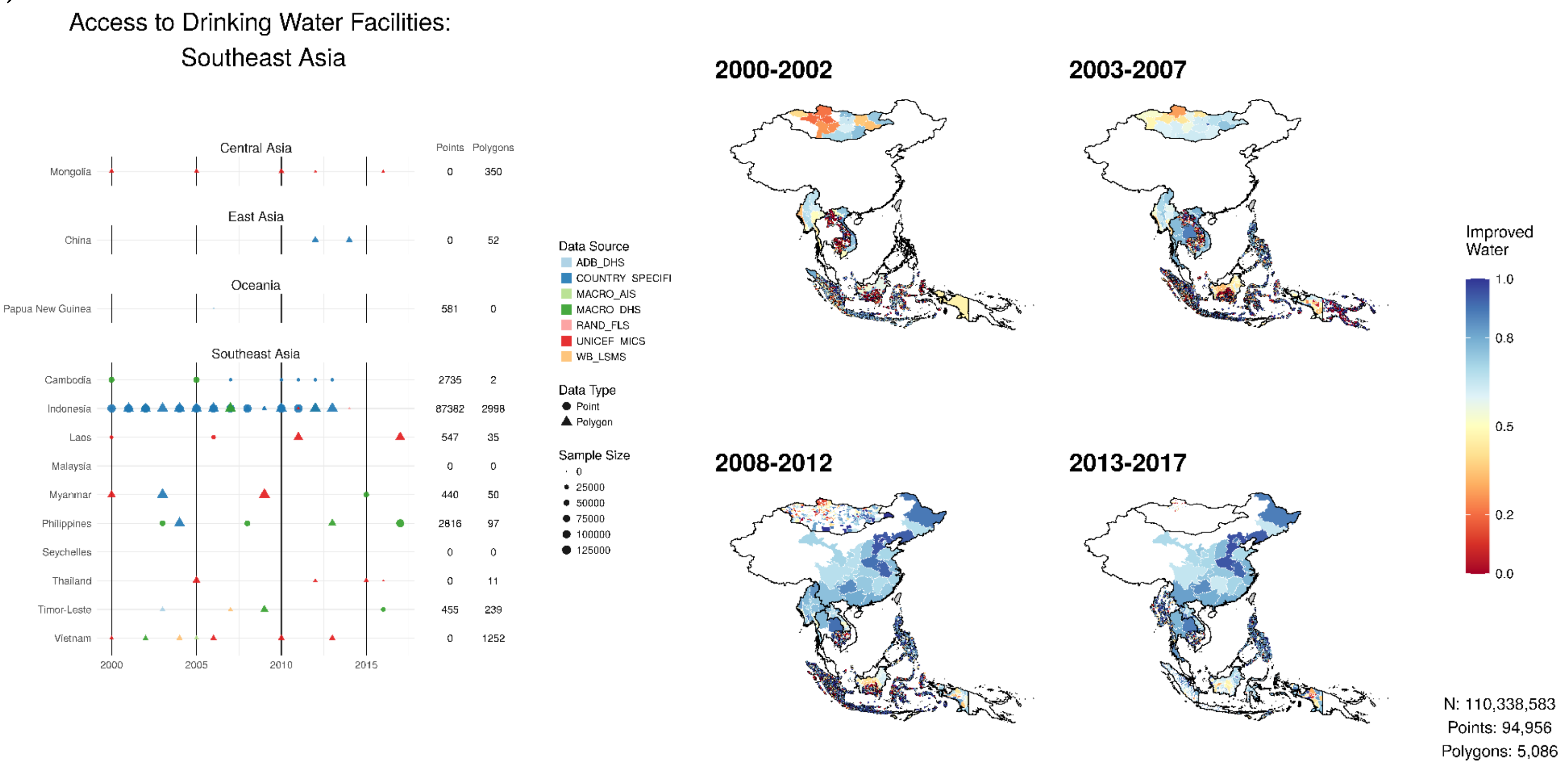

Access to Drinking Water Facilities:
Southeast Asia
Central Asia
Points
Polygons
Mongolia
0
350
East Asia
China
0
52
Oceania
Papua New Guinea
581
0
Southeast Asia
Cambodia
2735
2
Indonesia
87382
2998
Laos
547
35
Malaysia
0
0
Myanmar
440
50
Philippines
2816
97
Seychelles
0
0
Thailand
0
11
Timor-Leste
455
239
Vietnam
0
1252
2000
2005
2010
2015
Data Source
ADB_DHS
COUNTRY_SPECIFI
MACRO_AIS
MACRO_DHS
RAND_FLS
UNICEF_MICS
WB_LSMS
Data Type
Point
Polygon
Sample Size
0
25000
50000
75000
100000
125000
2000-2002
2003-2007
2008-2012
2013-2017
Improved Water
1.0
0.8
0.5
0.2
0.0
N: 110,338,583
Points: 94,956
Polygons: 5,086

d)

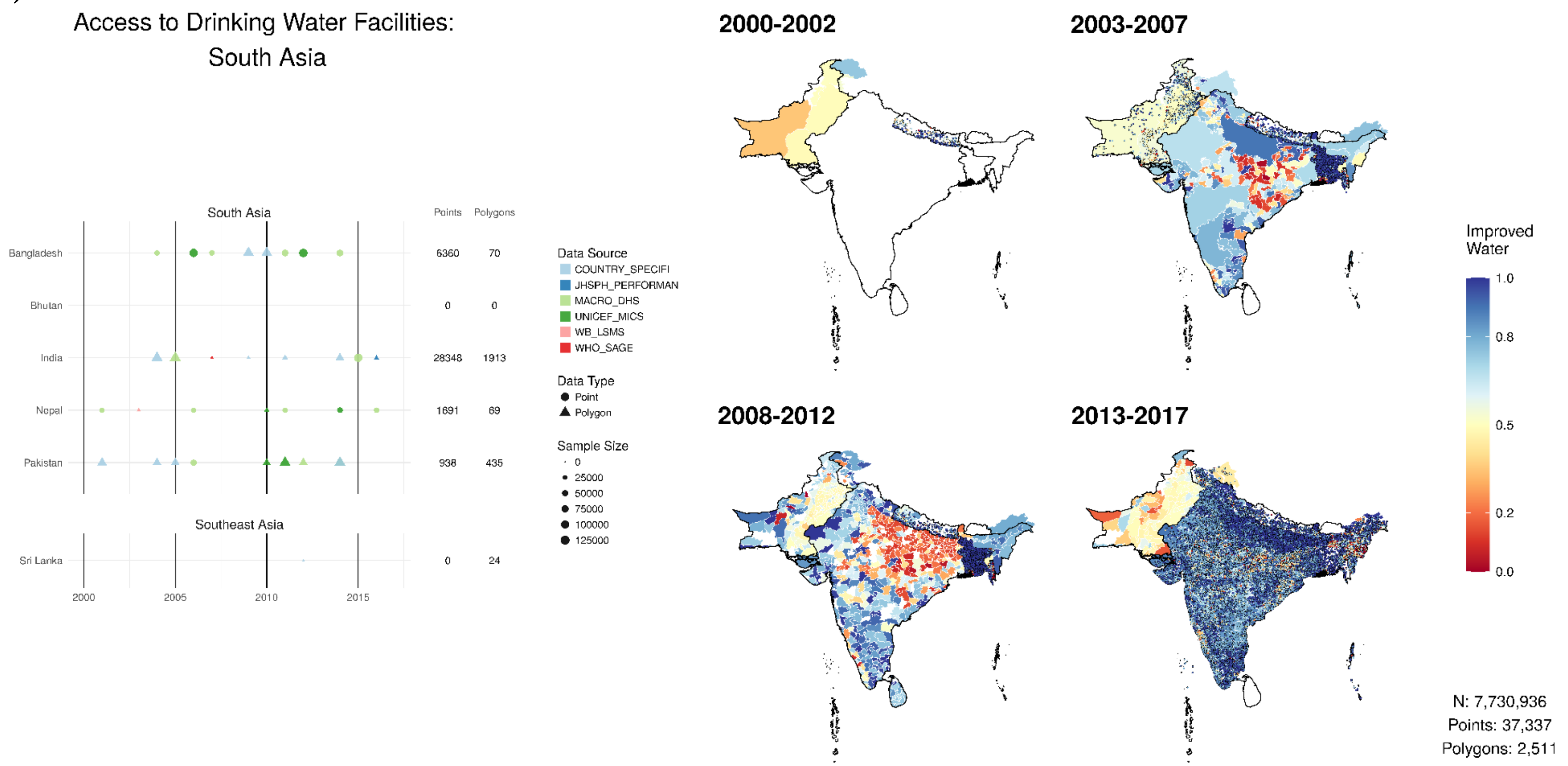

Access to Drinking Water Facilities:
South Asia
South Asia
Points
Polygons
Bangladesh
Bhutan
India
Nepal
Pakistan
6360
70
0
0
28348
1913
1691
69
938
435
Southeast Asia
Sri Lanka
0
24
2000
2005
2010
2015
Data Source
COUNTRY_SPECIFI
JHSPH_PERFORMAN
MACRO_DHS
UNICEF_MICS
WB_LSMS
WHO_SAGE
Data Type
Point
Polygon
Sample Size
0
25000
50000
75000
100000
125000
2000-2002
2003-2007
2008-2012
2013-2017
Improved Water
1.0
0.8
0.5
0.2
0.0
N: 7,730,936
Points: 37,337
Polygons: 2,511

e)

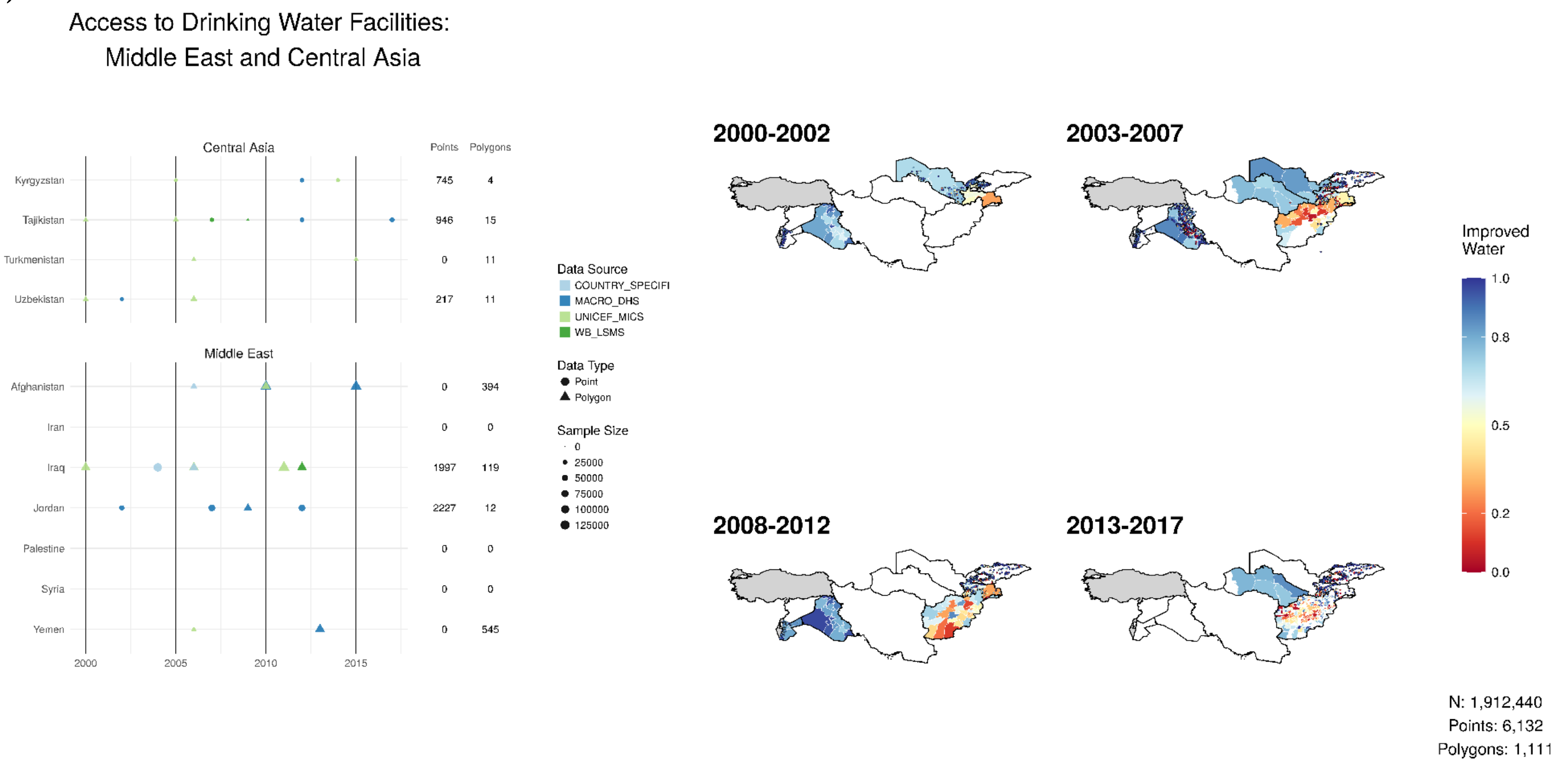

Appendix Figure 4. Sanitation facility data availability by type and country, 2000–2017

All data are shown by country and year of survey and mapped at their corresponding geopositioned coordinate or area. In the left panel, the total number of points and polygons (areal) for each country are plotted by data source, type, and sample size. Sample size represents the number of individual microdata records for each survey. In the right panel, mean access to sanitation facilities for the input coordinate or area is mapped. Figure **a)** shows sanitation facility data availability in Africa by type and country from 2000 to 2017. Figure **b)** shows sanitation facility data availability in Latin America and the Caribbean by type and country from 2000 to 2017. Figure **c)** shows sanitation facility data availability in southeast Asia by type and country from 2000 to 2017. Figure **d)** shows sanitation facility data availability in south Asia by type and country from 2000 to 2017. Figure **e)** shows sanitation facility data availability in the Middle East and central Asia by type and country from 2000 to 2017.

a)

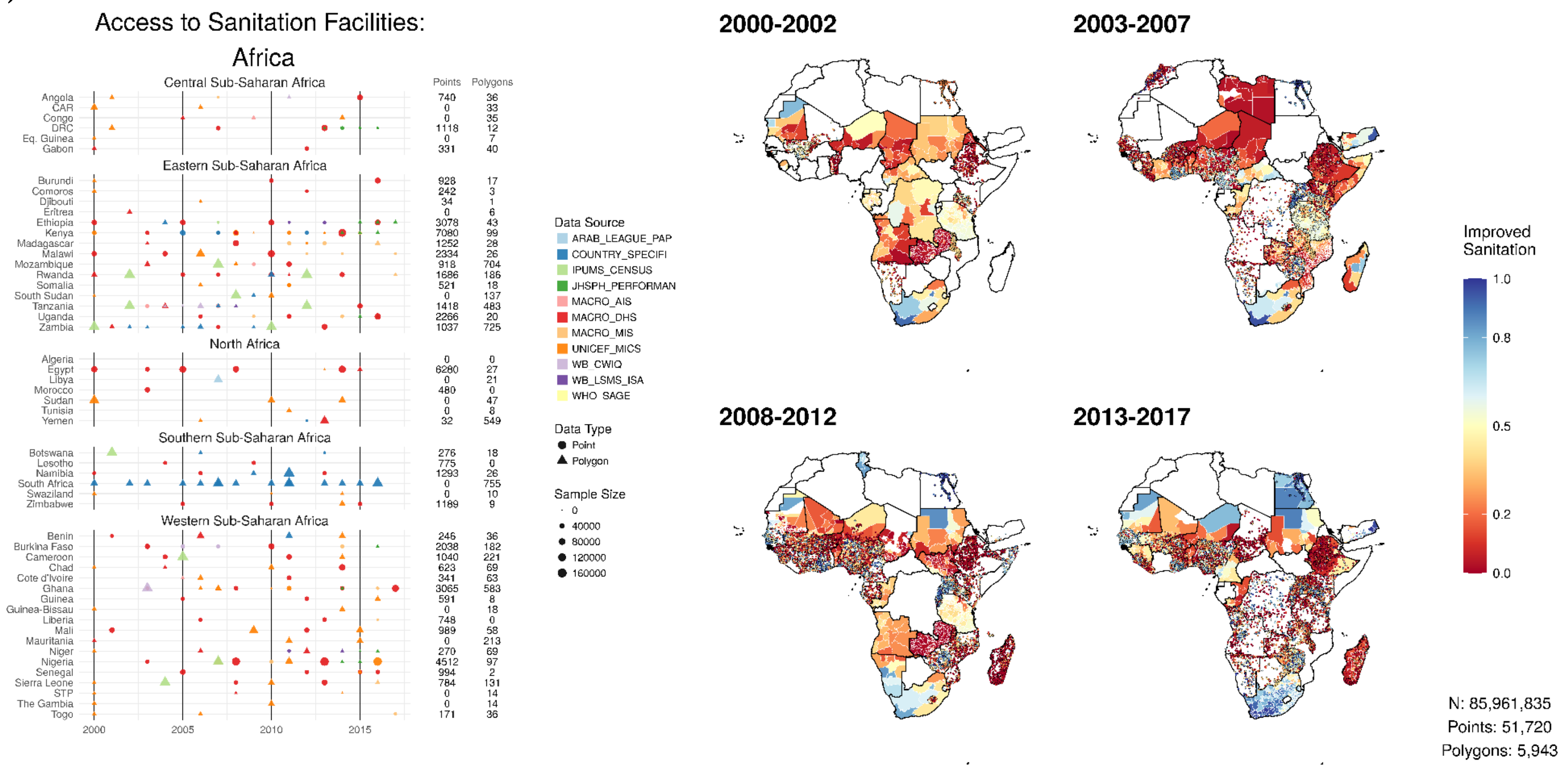

Access to Sanitation Facilities: Africa
Central Sub-Saharan Africa
Points
Polygons
Angola 740 36
CAR 0 33
Congo 0 35
DRC 1118 12
Eq. Guinea 0 7
Gabon 331 40
Eastern Sub-Saharan Africa
Burundi 928 17
Comoros 242 3
Djibouti 34 1
Eritrea 0 6
Ethiopia 3078 43
Kenya 7080 99
Madagascar 1252 28
Malawi 2334 26
Mozambique 918 704
Rwanda 1686 186
Somalia 521 18
South Sudan 0 137
Tanzania 1418 483
Uganda 2266 20
Zambia 1037 725
North Africa
Algeria 0 0
Egypt 6280 27
Libya 0 21
Morocco 480 0
Sudan 0 47
Tunisia 0 8
Yemen 32 549
Southern Sub-Saharan Africa
Botswana 276 18
Lesotho 775 0
Namibia 1293 26
South Africa 0 755
Swaziland 0 10
Zimbabwe 1189 9
Western Sub-Saharan Africa
Benin 246 36
Burkina Faso 2038 182
Cameroon 1040 221
Chad 623 69
Cote d'Ivoire 341 63
Ghana 3065 583
Guinea 591 8
Guinea-Bissau 0 18
Liberia 748 0
Mali 989 58
Mauritania 0 213
Niger 270 69
Nigeria 4512 97
Senegal 994 2
Sierra Leone 784 131
STP 0 14
The Gambia 0 14
Togo 171 36
2000
2005
2010
2015
Data Source
ARAB_LEAGUE_PAP
COUNTRY_SPECIFI
IPUMS_CENSUS
JHSPH_PERFORMAN
MACRO_AIS
MACRO_DHS
MACRO_MIS
UNICEF_MICS
WB_CWIQ
WB_LSMS_ISA
WHO_SAGE
Data Type
Point
Polygon
Sample Size
0
40000
80000
120000
160000
2000-2002
2003-2007
2008-2012
2013-2017
Improved Sanitation
1.0
0.8
0.5
0.2
0.0
N: 85,961,835
Points: 51,720
Polygons: 5,943

b)

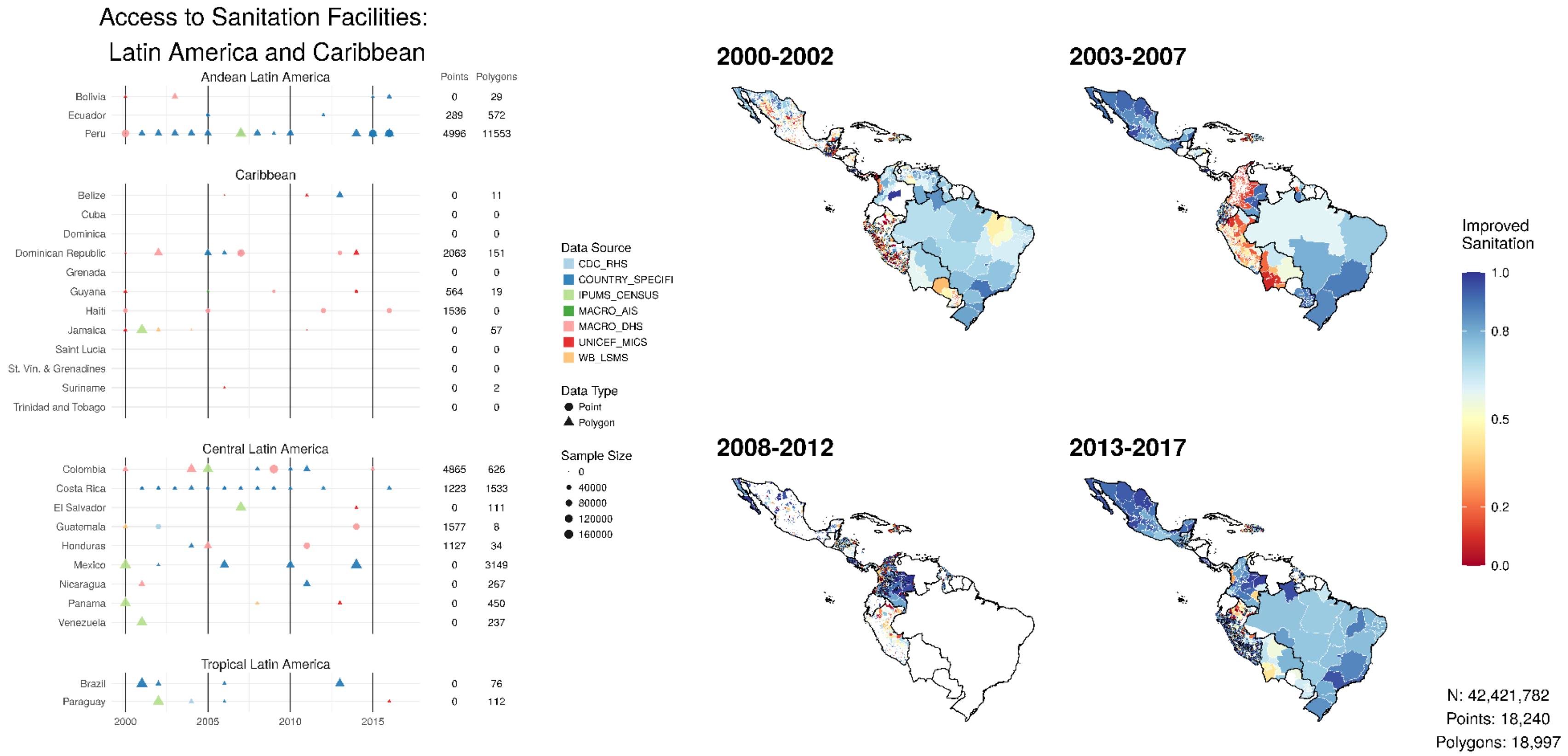

Access to Sanitation Facilities:
Latin America and Caribbean
Andean Latin America
Points
Polygons
Bolivia 0 29
Ecuador 289 572
Peru 4996 11553
Caribbean
Belize 0 11
Cuba 0 0
Dominica 0 0
Dominican Republic 2063 151
Grenada 0 0
Guyana 564 19
Haiti 1536 0
Jamaica 0 57
Saint Lucia 0 0
St. Vin. & Grenadines 0 0
Suriname 0 2
Trinidad and Tobago 0 0
Central Latin America
Colombia 4865 626
Costa Rica 1223 1533
El Salvador 0 111
Guatemala 1577 8
Honduras 1127 34
Mexico 0 3149
Nicaragua 0 267
Panama 0 450
Venezuela 0 237
Tropical Latin America
Brazil 0 76
Paraguay 0 112
2000
2005
2010
2015
Data Source
CDC_RHS
COUNTRY_SPECIFI
IPUMS_CENSUS
MACRO_AIS
MACRO_DHS
UNICEF_MICS
WB_LSMS
Data Type
Point
Polygon
Sample Size
0
40000
80000
120000
160000
2000-2002
2003-2007
2008-2012
2013-2017
Improved
Sanitation
1.0
0.8
0.5
0.2
0.0
N: 42,421,782
Points: 18,240
Polygons: 18,997

c)

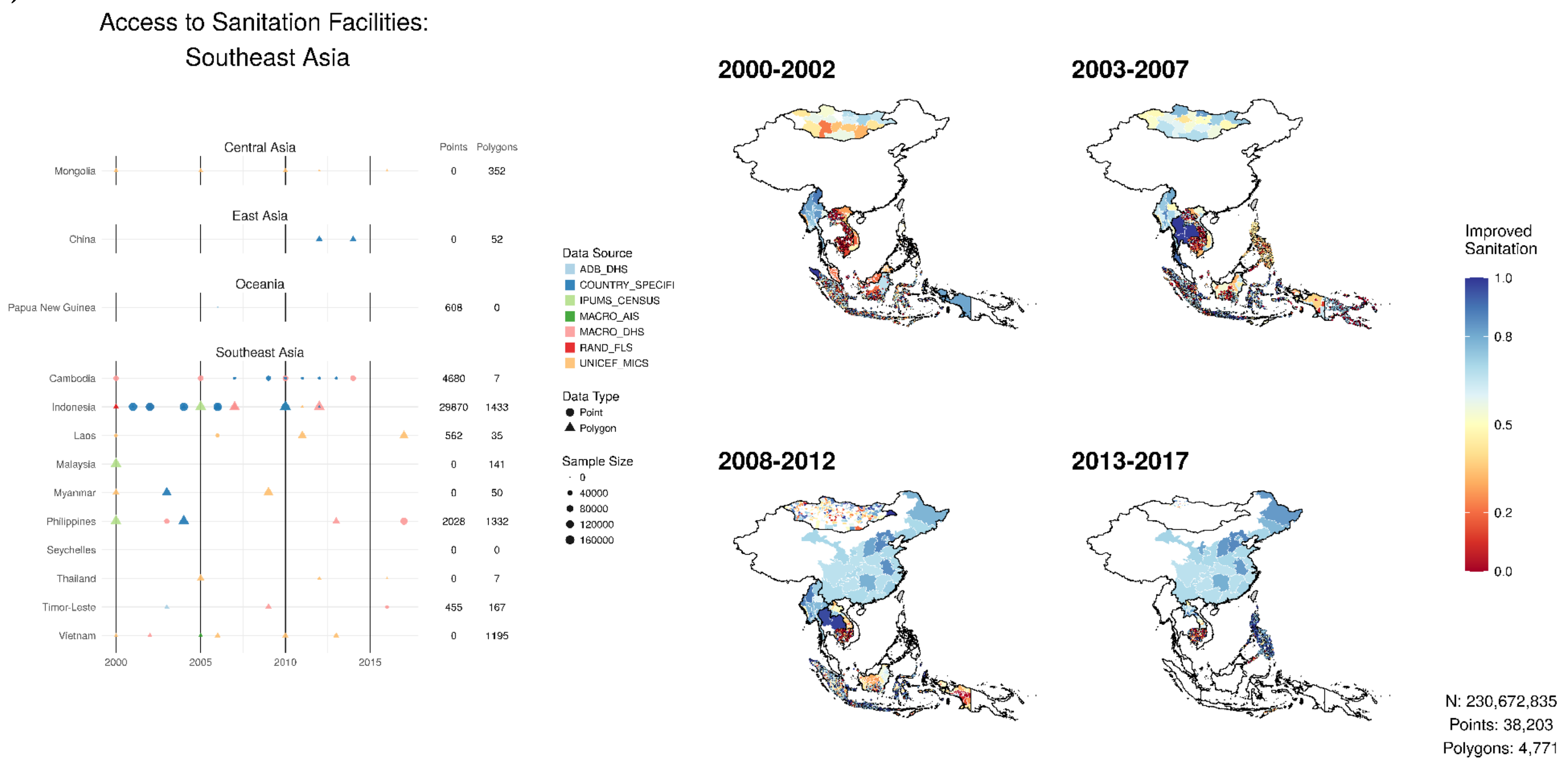

Access to Sanitation Facilities:
Southeast Asia
Central Asia
Mongolia
East Asia
China
Oceania
Papua New Guinea
Southeast Asia
Cambodia
Indonesia
Laos
Malaysia
Myanmar
Philippines
Seychelles
Thailand
Timor-Leste
Vietnam
2000
2005
2010
2015
Points
Polygons
0 352
0 52
608 0
4680 7
29870 1433
562 35
0 141
0 50
2028 1332
0 0
0 7
455 167
0 1195
Data Source
ADB_DHS
COUNTRY_SPECIFI
IPUMS_CENSUS
MACRO_AIS
MACRO_DHS
RAND_FLS
UNICEF_MICS
Data Type
Point
Polygon
Sample Size
0
40000
80000
120000
160000
2000-2002
2003-2007
2008-2012
2013-2017
Improved Sanitation
1.0
0.8
0.5
0.2
0.0
N: 230,672,835
Points: 38,203
Polygons: 4,771

d)

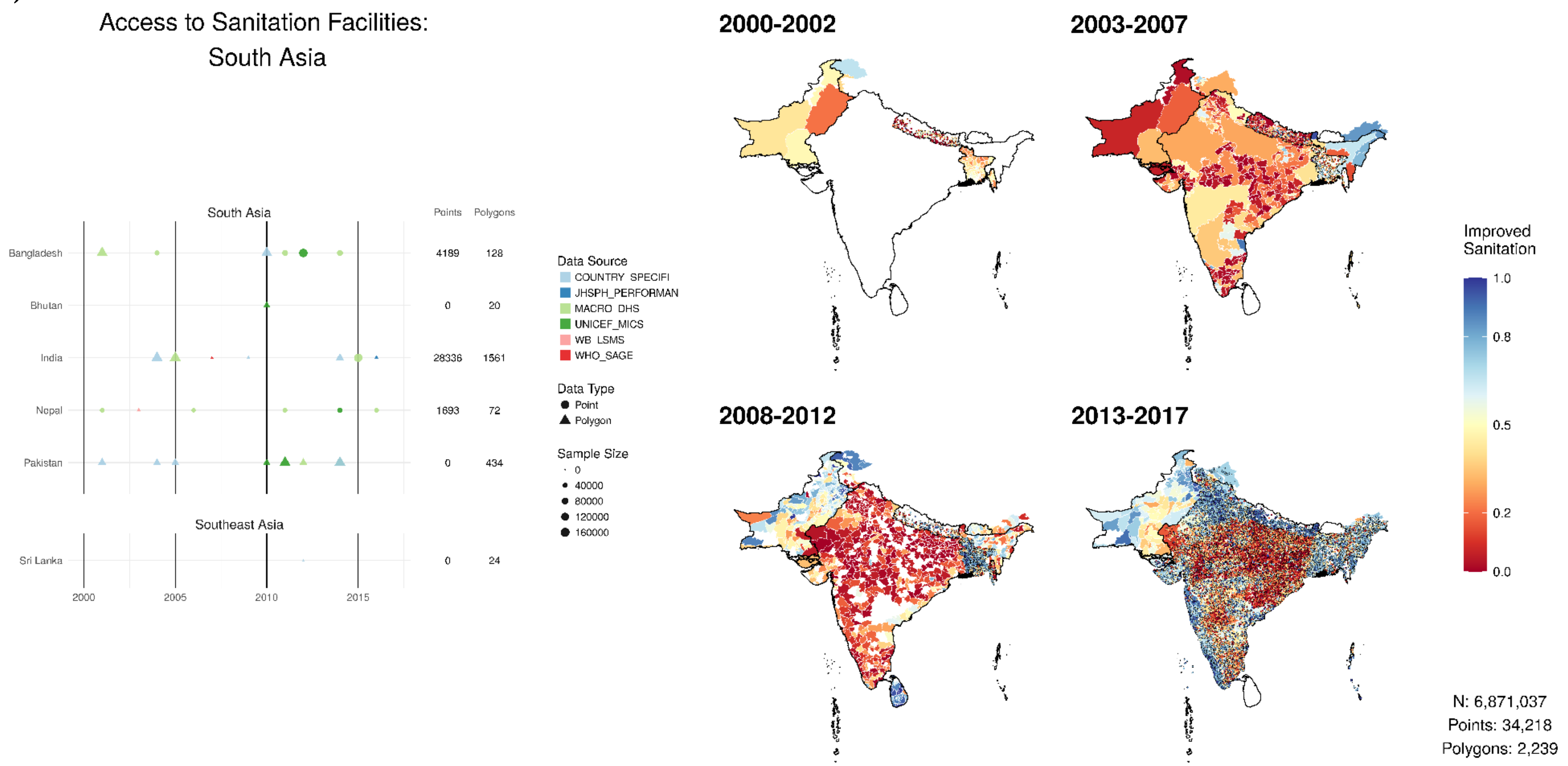

Access to Sanitation Facilities:
South Asia
South Asia
Points
Polygons
Bangladesh
4189
128
Bhutan
0
20
India
28336
1561
Nepal
1693
72
Pakistan
0
434
Southeast Asia
Sri Lanka
0
24
2000
2005
2010
2015
Data Source
COUNTRY_SPECIFI
JHSPH_PERFORMAN
MACRO_DHS
UNICEF_MICS
WB_LSMS
WHO_SAGE
Data Type
Point
Polygon
Sample Size
0
40000
80000
120000
160000
2000-2002
2003-2007
2008-2012
2013-2017
Improved Sanitation
1.0
0.8
0.5
0.2
0.0
N: 6,871,037
Points: 34,218
Polygons: 2,239

e)

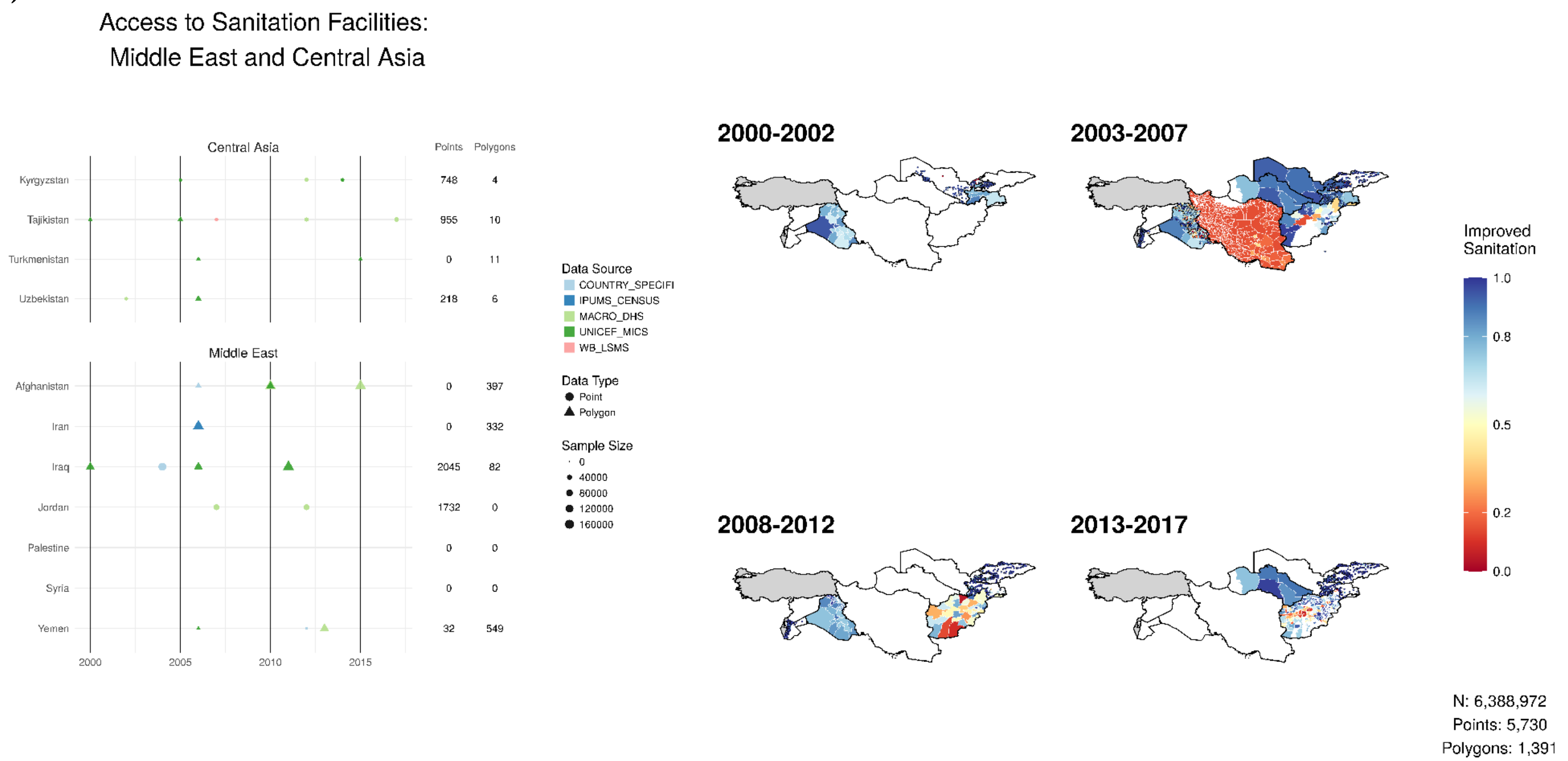

Access to Sanitation Facilities:
Middle East and Central Asia
Central Asia
Points
Polygons
Kyrgyzstan
748
4
Tajikistan
955
10
Turkmenistan
0
11
Uzbekistan
218
6
Middle East
Afghanistan
0
397
Iran
0
332
Iraq
2045
82
Jordan
1732
0
Palestine
0
0
Syria
0
0
Yemen
32
549
2000
2005
2010
2015
Data Source
COUNTRY_SPECIFI
IPUMS_CENSUS
MACRO_DHS
UNICEF_MICS
WB_LSMS
Data Type
Point
Polygon
Sample Size
0
40000
80000
120000
160000
2000-2002
2003-2007
2008-2012
2013-2017
Improved Sanitation
1.0
0.8
0.5
0.2
0.0
N: 6,388,972
Points: 5,730
Polygons: 1,391

Appendix Figure 5. Geospatial covariates for water and sanitation

Twenty-three covariate raster layers of possible socioeconomic and environmental correlates of access to improved water and sanitation prevalence were used as inputs for the stacking modelling process. Time-varying (^{TV}) covariates are presented for the year 2017. For the year of production of non-time-varying covariates, please refer to the individual covariate citation in Table 3 for additional detail. Covariates are labelled as follows: access to roads [*access2*], aridity^{TV} [*aridity*], elevation [*elevation*], urban or rural^{TV} [*ghlsurbanicity*], growing season length [*growingseason*], irrigation [*irrigation*], time [*year_cov*]. Maps reflect administrative boundaries, land cover, lakes, and population; grey-coloured grid cells were classified as "barren or sparsely vegetated" and had fewer than ten people per 1 × 1-km grid cell, or were not included in these analyses.[13]

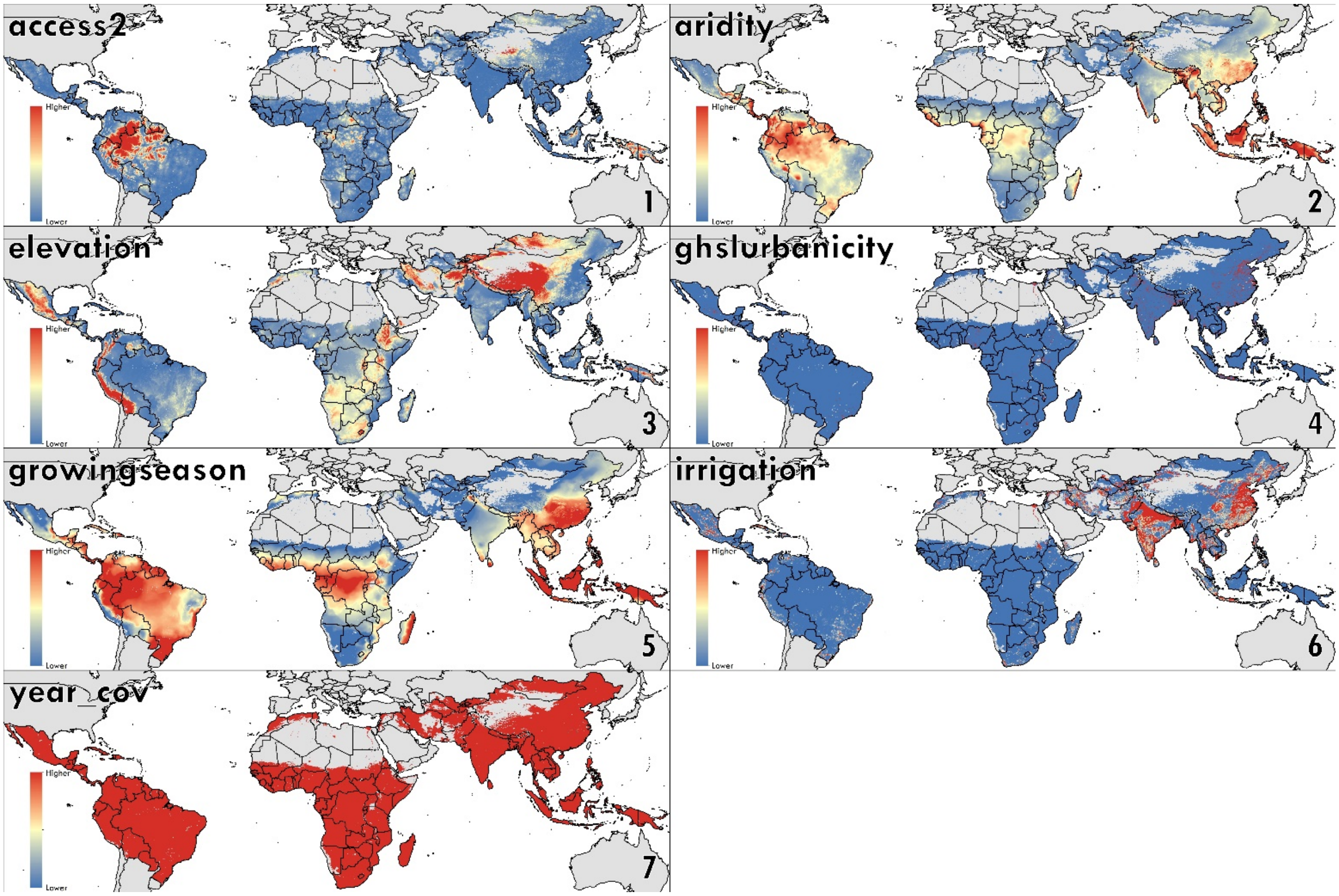

Appendix Figure 6. Stacker plots

Below the ensemble modelling process is illustrated. As an example, we have plotted predictions of access to piped water in 2000. Panels **A-C** depict predictions from generalised additive model (**A)**, boosted regression trees (**B)**, and penalised lasso regression child models (**C)**. Panel (**D)** illustrates predictions from our ensemble Bayesian geostatistical model that uses the child model predictions as covariates for prediction.

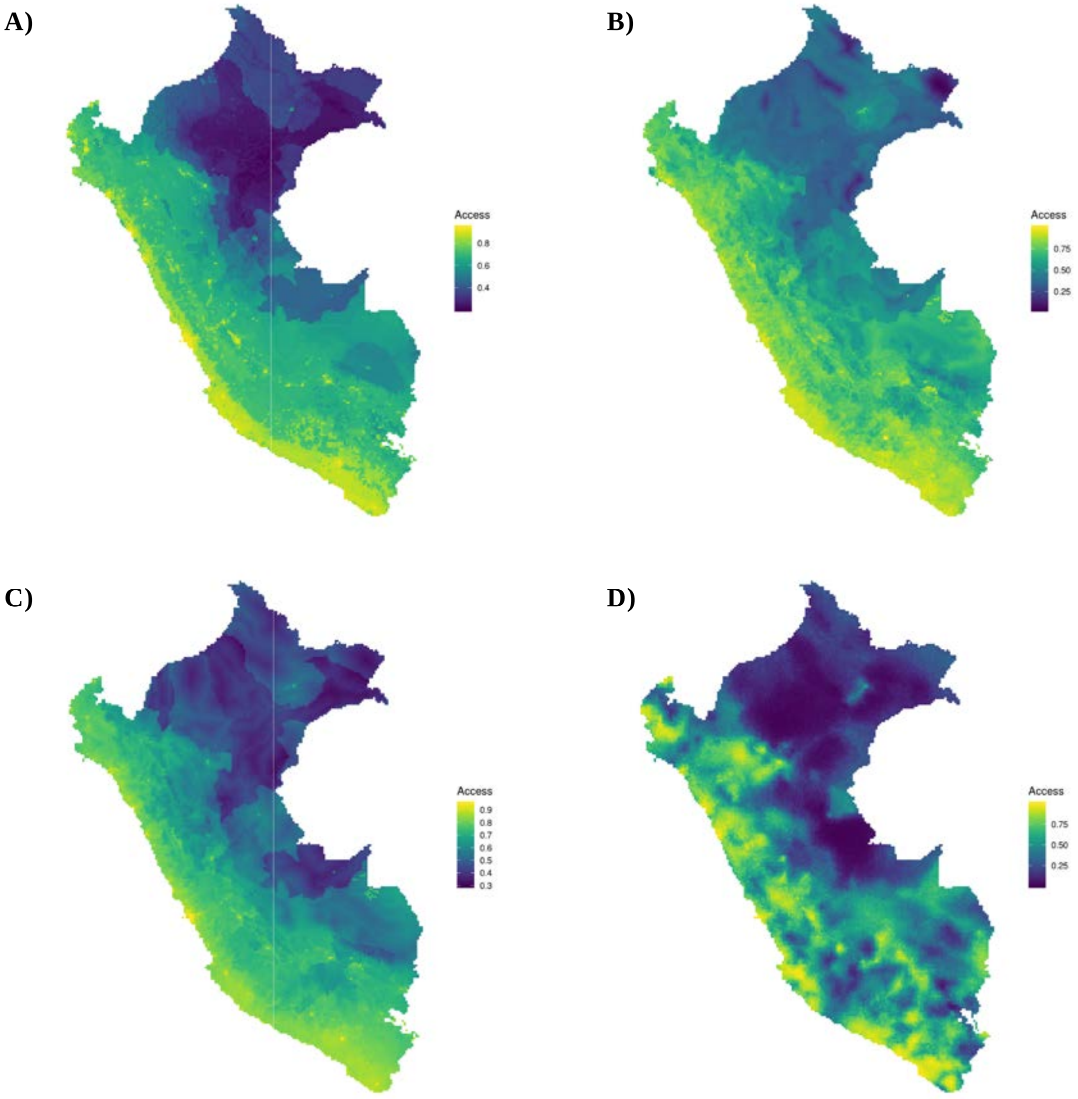

Appendix Figure 7. Finite elements mesh

The finite elements mesh used to fit the space-time correlated error for Lesotho is shown as an example. The land area to be modelled over is divided into discrete areas via Delaunay triangulation.

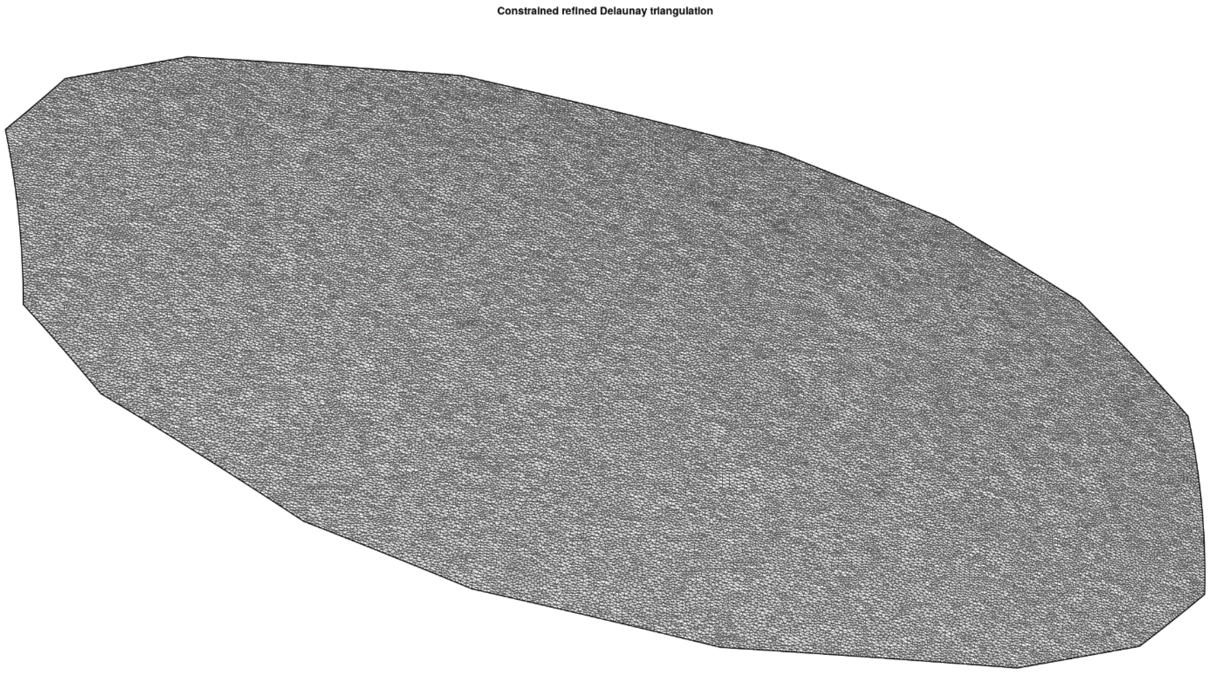

Appendix Figure 8. Mean access to piped water, at 5 × 5-km resolution and second administrative level, 2000 and 2017

Panels **a-b** present mean access to piped water at the 5 × 5-km resolution in 2000 and 2017. Panels **c-d** present mean access to piped water at the second administrative level in 2000 and 2017. Maps reflect administrative boundaries, land cover, lakes, and population; grey-coloured grid cells were classified as "barren or sparsely vegetated" and had fewer than ten people per 1 × 1-km grid cell, or were not included in these analyses.[5,11–15]

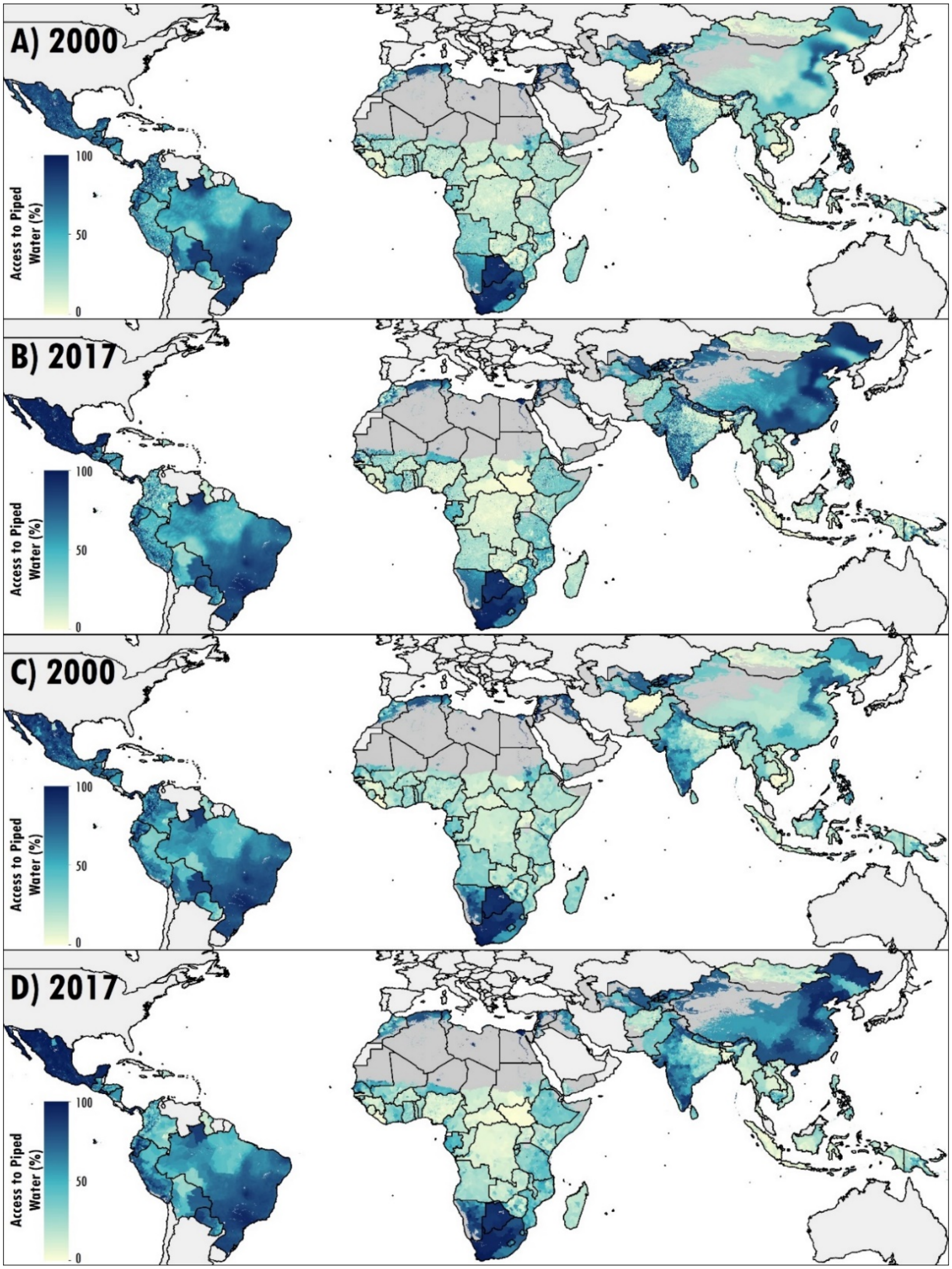

Appendix Figure 9. Mean access to improved water, at 5 × 5-km resolution and second administrative level, 2000 and 2017

Panels **a-b** present mean access to improved water at the 5 × 5-km resolution in 2000 and 2017. Panels **c-d** present mean access to improved water at the second administrative level in 2000 and 2017. Maps reflect administrative boundaries, land cover, lakes, and population; grey-coloured grid cells were classified as "barren or sparsely vegetated" and had fewer than ten people per 1 × 1-km grid cell, or were not included in these analyses.[5,11–15]

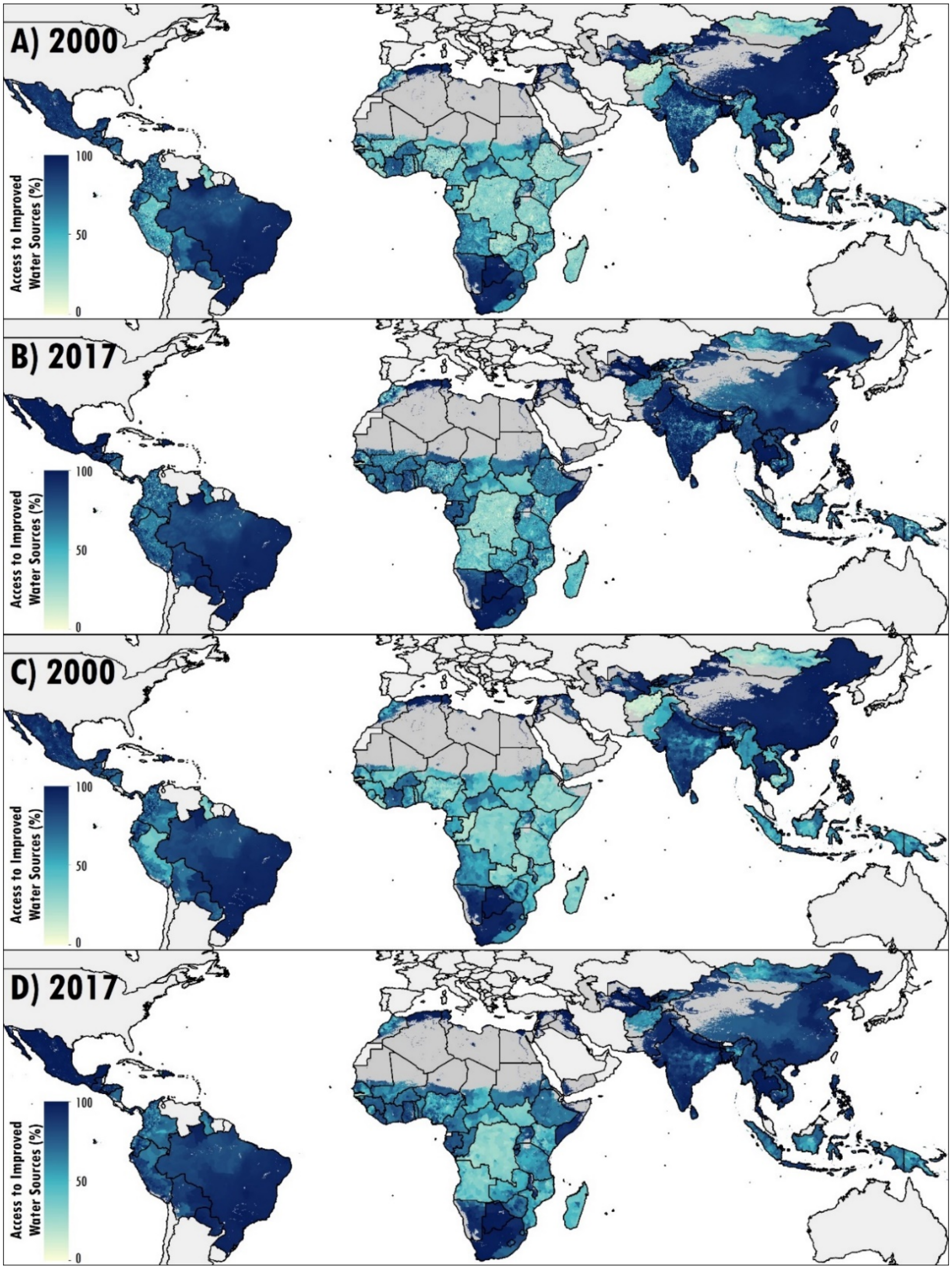

Appendix Figure 10. Mean access to unimproved water, at 5 × 5-km resolution and second administrative level, 2000 and 2017

Panels **a-b** present mean access to unimproved water at the 5 × 5-km resolution in 2000 and 2017. Panels **c-d** present mean access to unimproved water at the second administrative level in 2000 and 2017. Maps reflect administrative boundaries, land cover, lakes, and population; grey-coloured grid cells were classified as "barren or sparsely vegetated" and had fewer than ten people per 1 × 1-km grid cell, or were not included in these analyses.[5,11–15]

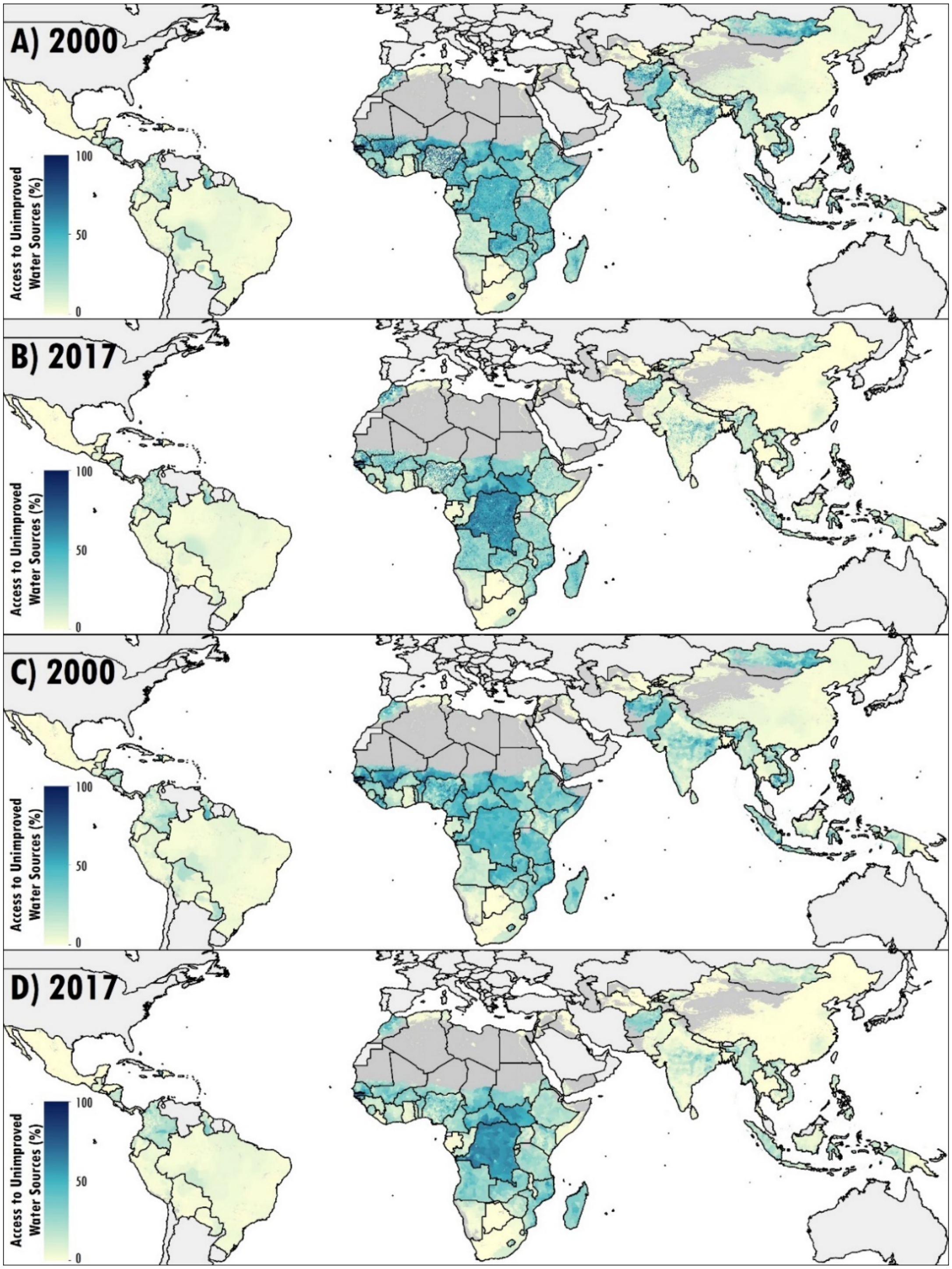

Appendix Figure 11. Mean access to surface water, at 5 × 5-km resolution and second administrative level, 2000 and 2017

Panels **a-b** present mean access to surface water at the 5 × 5-km resolution in 2000 and 2017. Panels **c-d** present mean access to surface water at the second administrative level in 2000 and 2017. Maps reflect administrative boundaries, land cover, lakes, and population; grey-coloured grid cells were classified as "barren or sparsely vegetated" and had fewer than ten people per 1 × 1-km grid cell, or were not included in these analyses.[5,11–15]

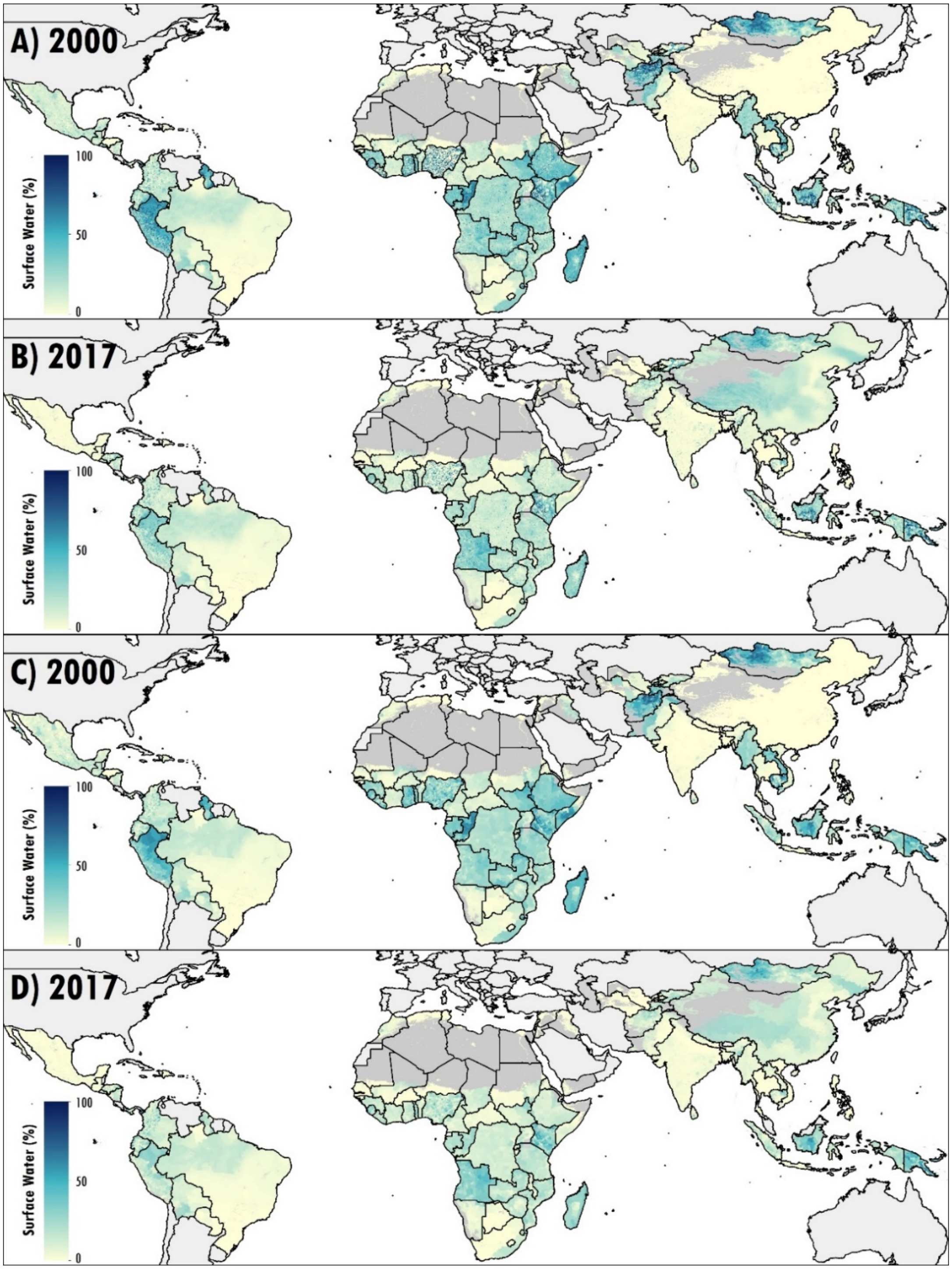

Appendix Figure 12. Mean access to piped sanitation, at 5 × 5-km resolution and second administrative level, 2000 and 2017

Panels **a-b** present mean access to piped sanitation at the 5 × 5-km resolution in 2000 and 2017. Panels **c-d** present mean access to piped sanitation at the second administrative level in 2000 and 2017. Maps reflect administrative boundaries, land cover, lakes, and population; grey-coloured grid cells were classified as "barren or sparsely vegetated" and had fewer than ten people per 1 × 1-km grid cell, or were not included in these analyses.[5,11–15]

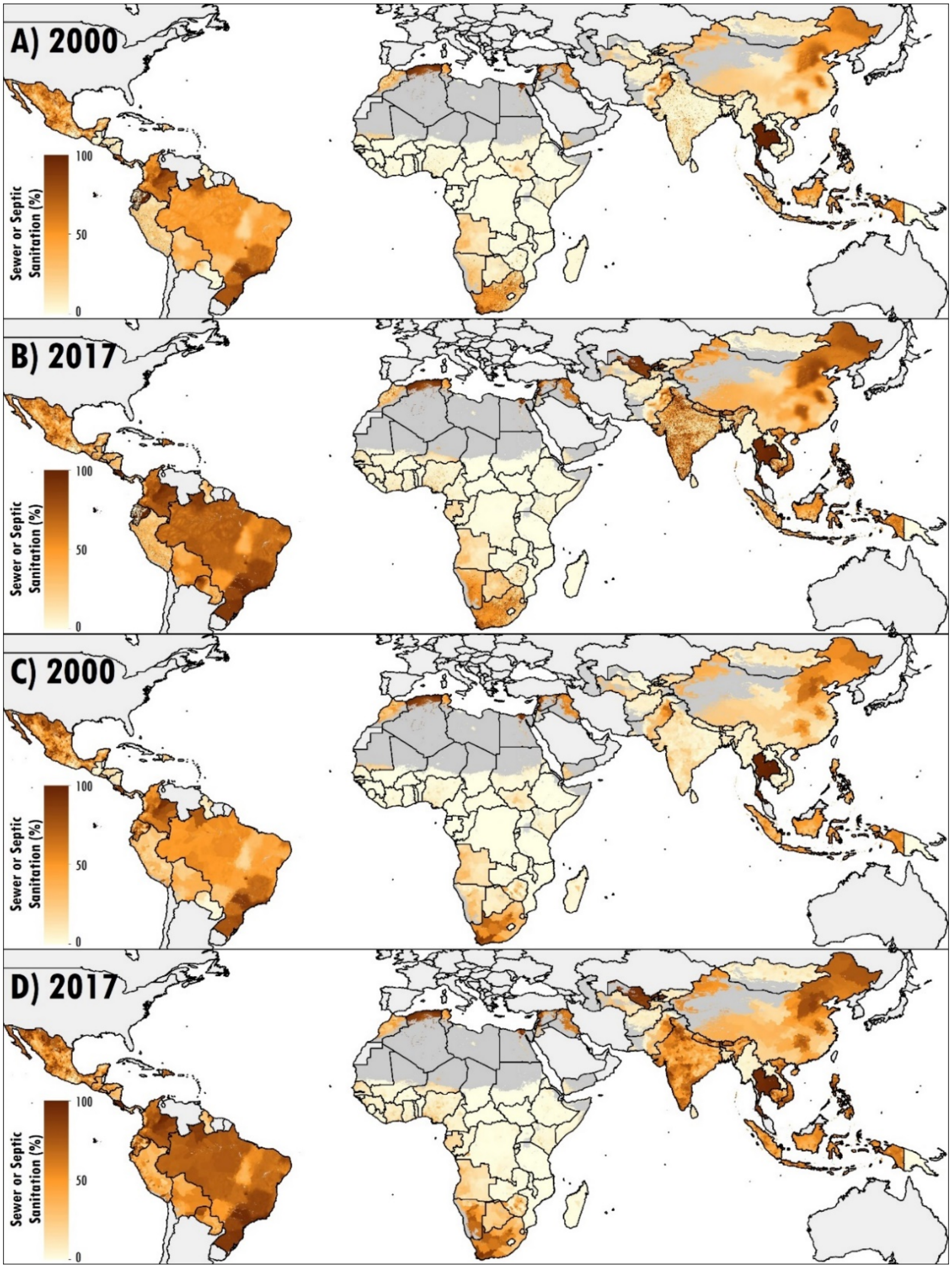

Appendix Figure 13. Mean access to improved sanitation, at 5 × 5-km resolution and second administrative level, 2000 and 2017

Panels **a-b** present mean access to improved sanitation at the 5 × 5-km resolution in 2000 and 2017. Panels **c-d** present mean access to improved sanitation at the second administrative level in 2000 and 2017. Maps reflect administrative boundaries, land cover, lakes, and population; grey-coloured grid cells were classified as "barren or sparsely vegetated" and had fewer than ten people per 1 × 1-km grid cell, or were not included in these analyses[5,11–15]

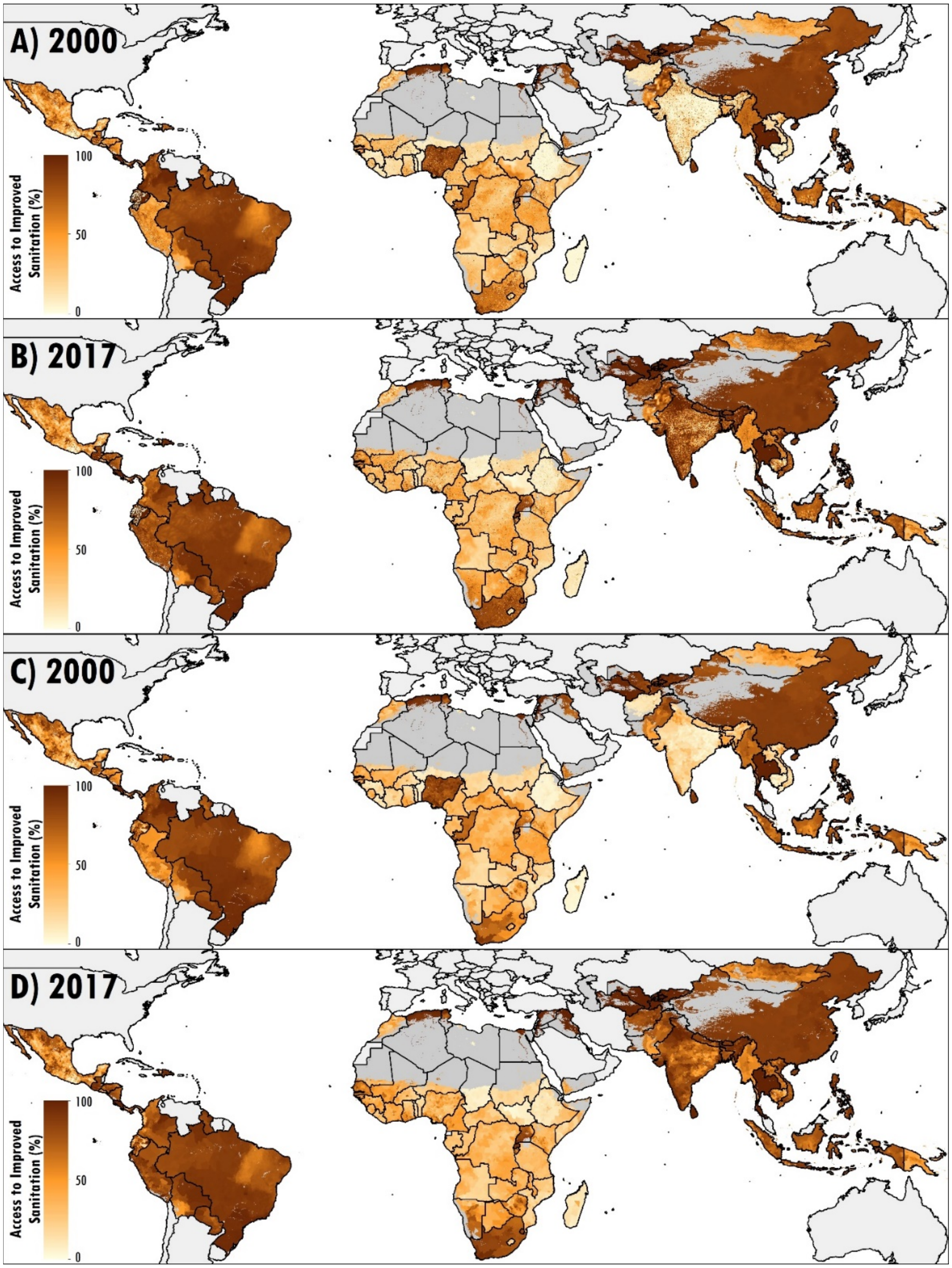

Appendix Figure 14. Mean access to unimproved sanitation, at 5 × 5-km resolution and second administrative level, 2000 and 2017

Panels **a-b** present mean access to unimproved sanitation at the 5 × 5-km resolution in 2000 and 2017. Panels **c-d** present mean access to unimproved sanitation at the second administrative level in 2000 and 2017. Maps reflect administrative boundaries, land cover, lakes, and population; grey-coloured grid cells were classified as “barren or sparsely vegetated” and had fewer than ten people per 1 × 1-km grid cell, or were not included in these analyses.[5,11–15]

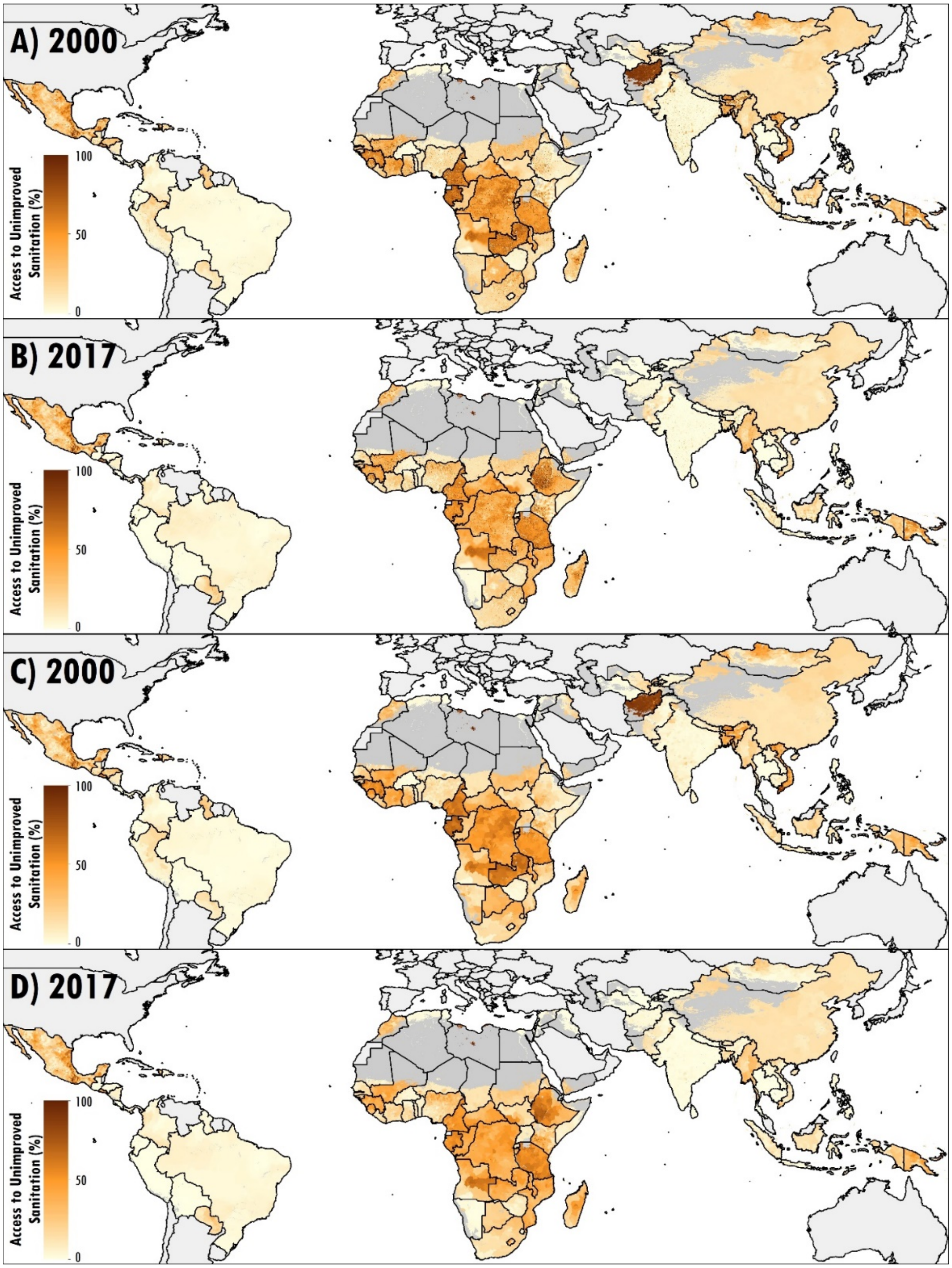

Appendix Figure 15. Mean open defecation, at 5 × 5-km resolution and second administrative level, 2000 and 2017

Panels **a-b** present mean open defecation at the 5 × 5-km resolution in 2000 and 2017. Panels **c-d** present mean open defecation at the second administrative level in 2000 and 2017. Maps reflect administrative boundaries, land cover, lakes, and population; grey-coloured grid cells were classified as “barren or sparsely vegetated” and had fewer than ten people per 1 × 1-km grid cell, or were not included in these analyses.[5,11–15]

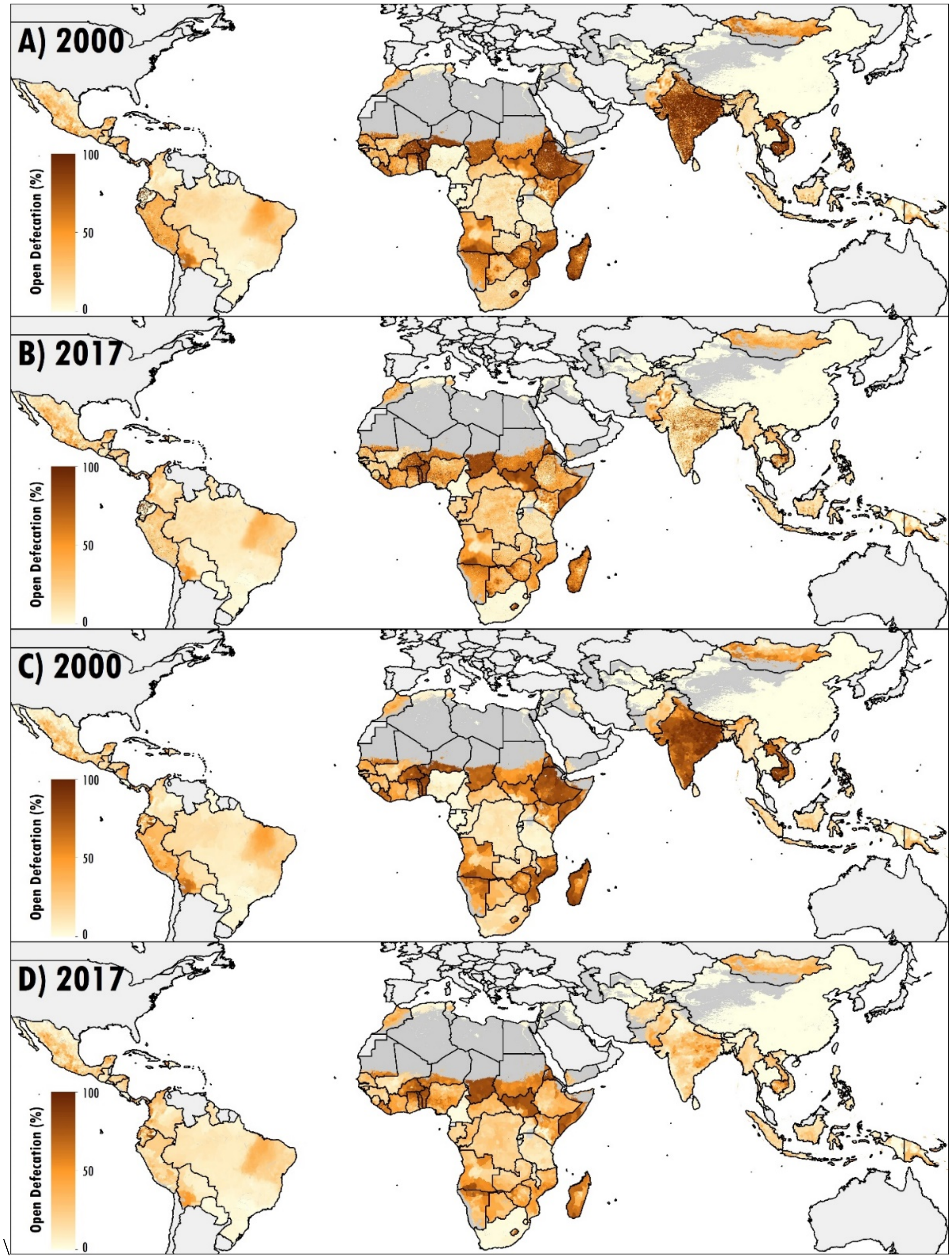

\

Appendix Figure 16. 95% uncertainty intervals for access to piped water, at 5 × 5-km resolution and second administrative level, 2017

95% uncertainty intervals were calculated as the difference between the 97.5th percentile and the 2.5th percentile of the posterior distribution. Panel **A)** presents uncertainty at the 5 × 5-km gird cell and panel **B)** presents uncertainty at the second administrative level. Maps reflect administrative boundaries, land cover, lakes, and population; grey-coloured grid cells were classified as "barren or sparsely vegetated" and had fewer than ten people per 1 × 1-km grid cell, or were not included in these analyses.[5,11–15]

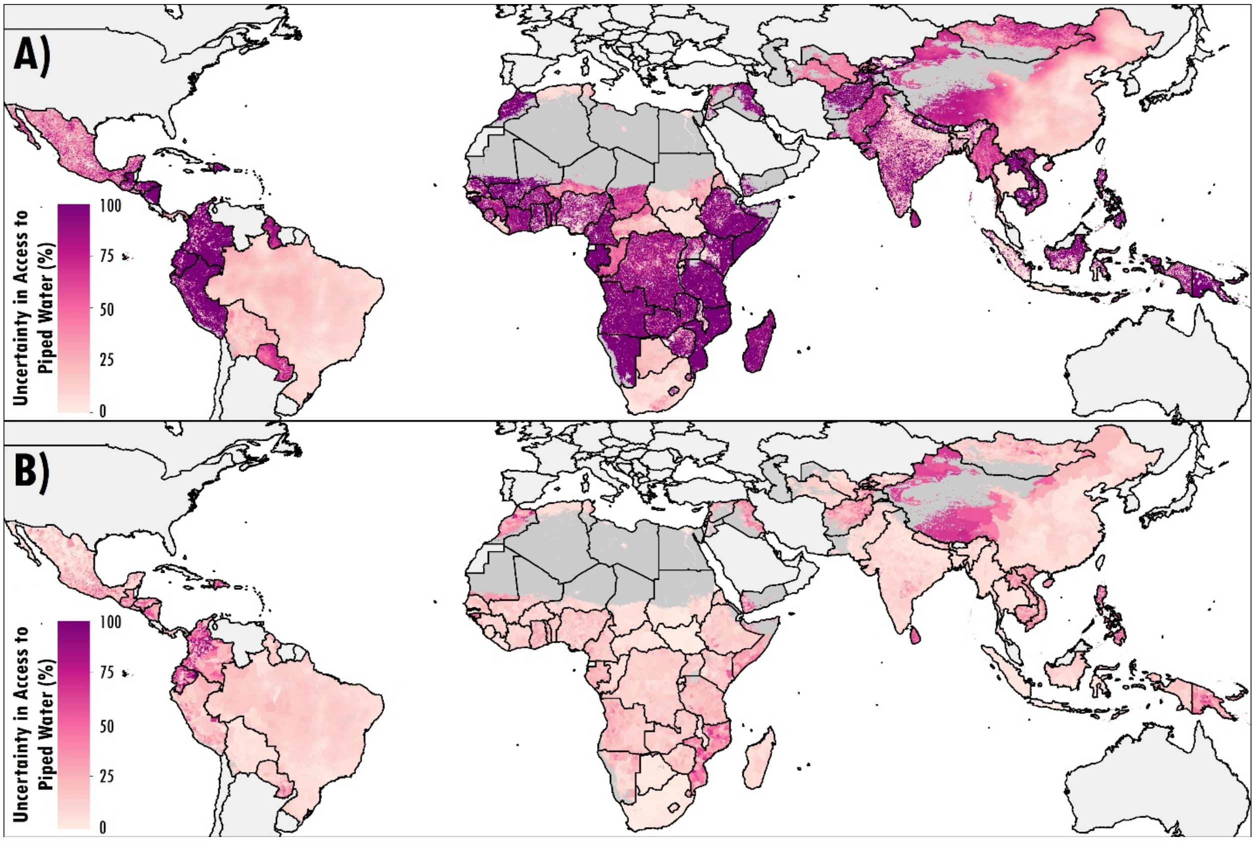

b

Appendix Figure 17. 95% uncertainty intervals for access to improved water, at 5 × 5-km resolution and second administrative level, 2017

95% uncertainty intervals were calculated as the difference between the 97.5th percentile and the 2.5th percentile of the posterior distribution. Panel **A)** presents uncertainty at the 5 × 5-km gird cell and panel **B)** presents uncertainty at the second administrative level. Maps reflect administrative boundaries, land cover, lakes, and population; grey-coloured grid cells were classified as "barren or sparsely vegetated" and had fewer than ten people per 1 × 1-km grid cell, or were not included in these analyses.[5,11–15]

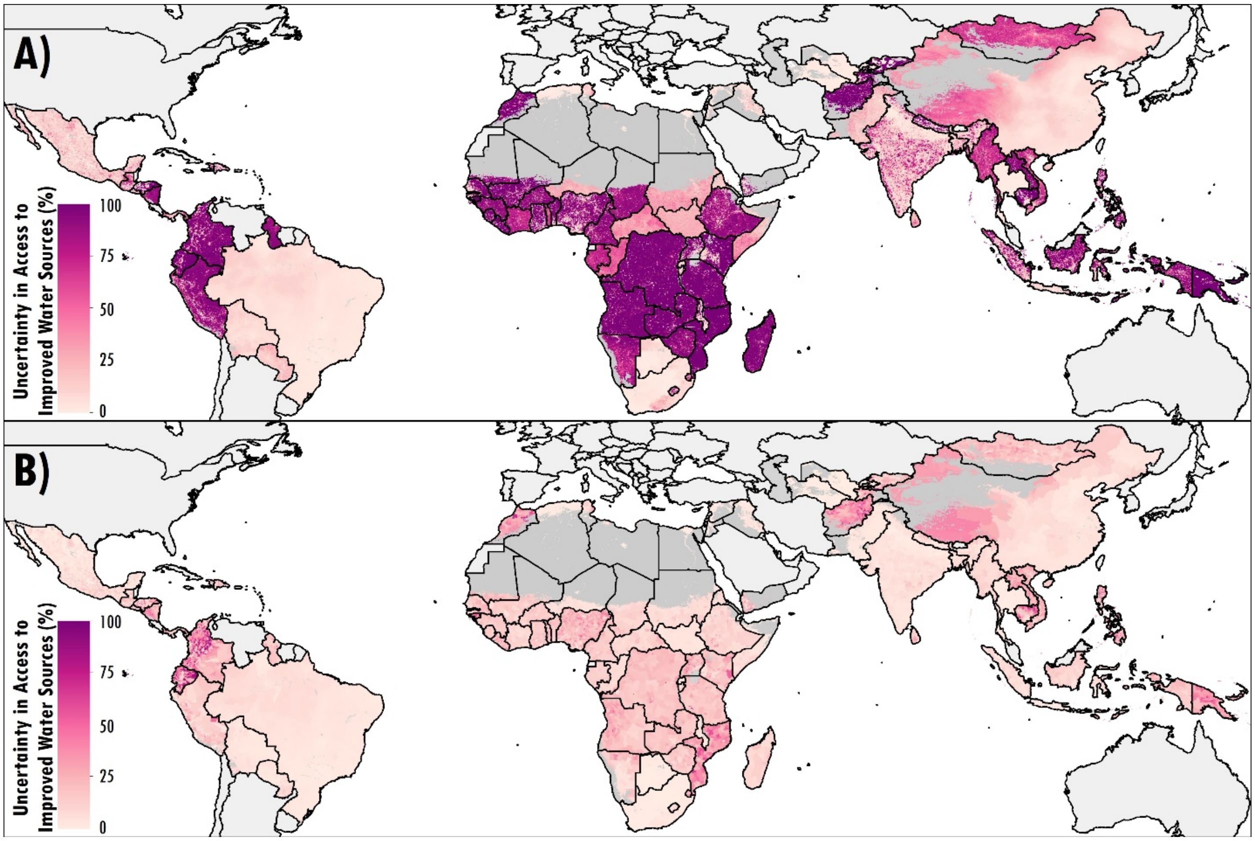

Appendix Figure 18. 95% uncertainty intervals for access to piped sanitation, at 5 × 5-km resolution and second administrative level, 2017

95% uncertainty intervals were calculated as the difference between the 97.5th percentile and the 2.5th percentile of the posterior distribution. Panel **A)** presents uncertainty at the 5 × 5-km gird cell and panel **B)** presents uncertainty at the second administrative level. Maps reflect administrative boundaries, land cover, lakes, and population; grey-coloured grid cells were classified as “barren or sparsely vegetated” and had fewer than ten people per 1 × 1-km grid cell, or were not included in these analyses.[5,11–15]

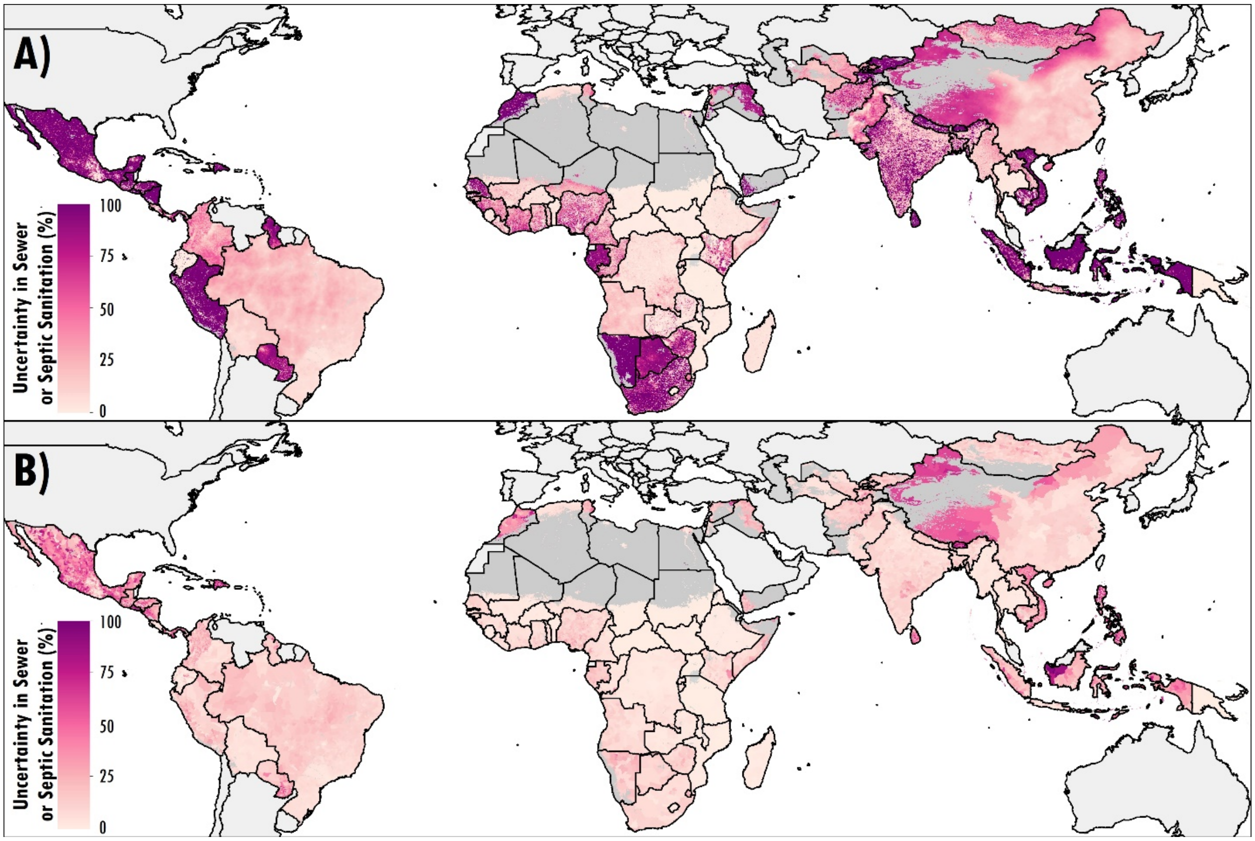

Appendix Figure 19. 95% uncertainty intervals for access to improved sanitation, at 5 × 5-km resolution and second administrative level, 2017

95% uncertainty intervals were calculated as the difference between the 97.5th percentile and the 2.5th percentile of the posterior distribution. Panel **A)** presents uncertainty at the 5 × 5-km gird cell and panel **B)** presents uncertainty at the second administrative level. Maps reflect administrative boundaries, land cover, lakes, and population; grey-coloured grid cells were classified as "barren or sparsely vegetated" and had fewer than ten people per 1 × 1-km grid cell, or were not included in these analyses. [5,11–15]

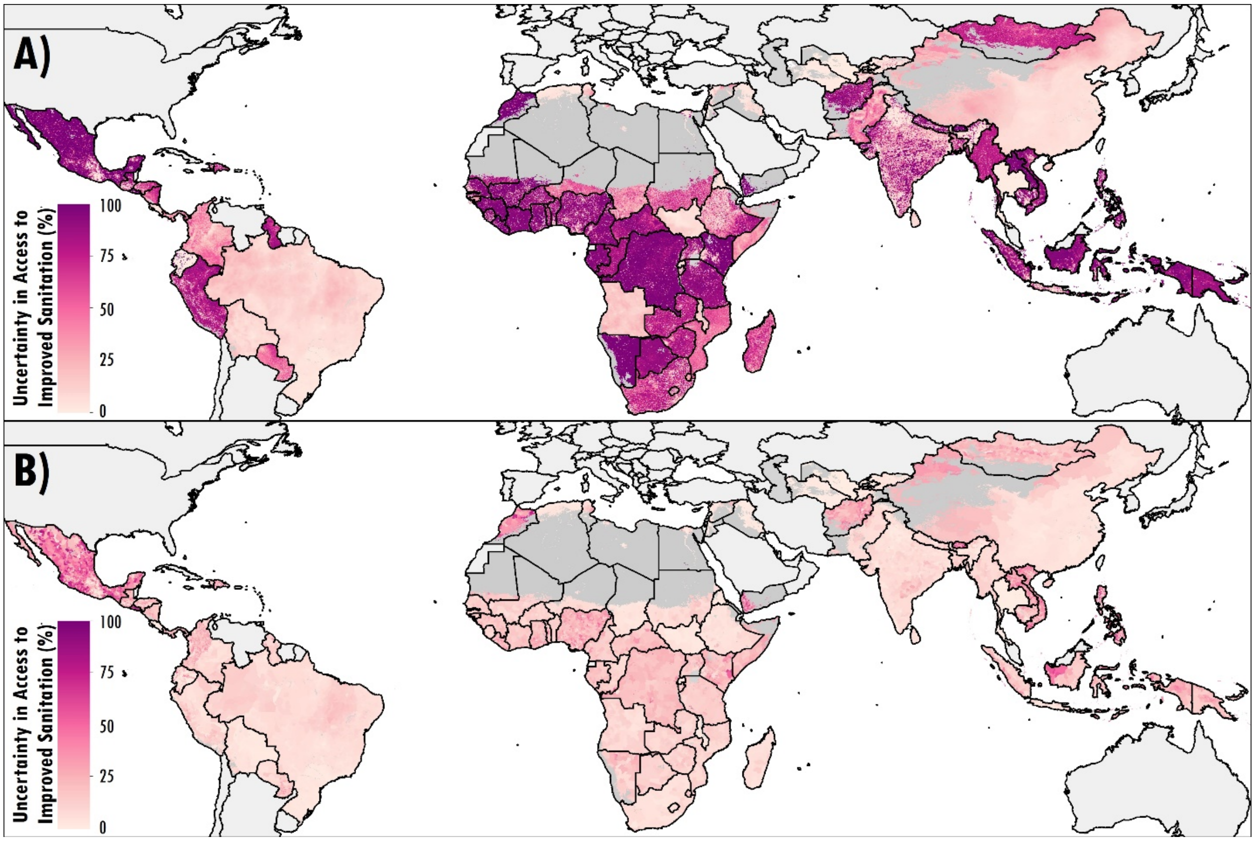

Appendix Figure 20. Ternary water, at the first administrative and national level, 2000 and 2017

The co-distribution of improved, unimproved, and no facility access is shown for water at the first administrative-level (**A**: 2000, **B**: 2017) and national level (**C**: 2000, **D**: 2017). Green denotes first administrative-level units where the majority of the population (>60%) had access to improved facilities, blue denotes >60% reliance on unimproved, and red denotes >60% having no facility access. Yellow indicates that there was no single dominant facility type used by >60% of the unit's population. Maps reflect administrative boundaries, land cover, lakes, and population; grey-coloured grid cells were classified as "barren or sparsely vegetated" and had fewer than ten people per 1 × 1-km grid cell, or were not included in these analyses.[5,11–15]

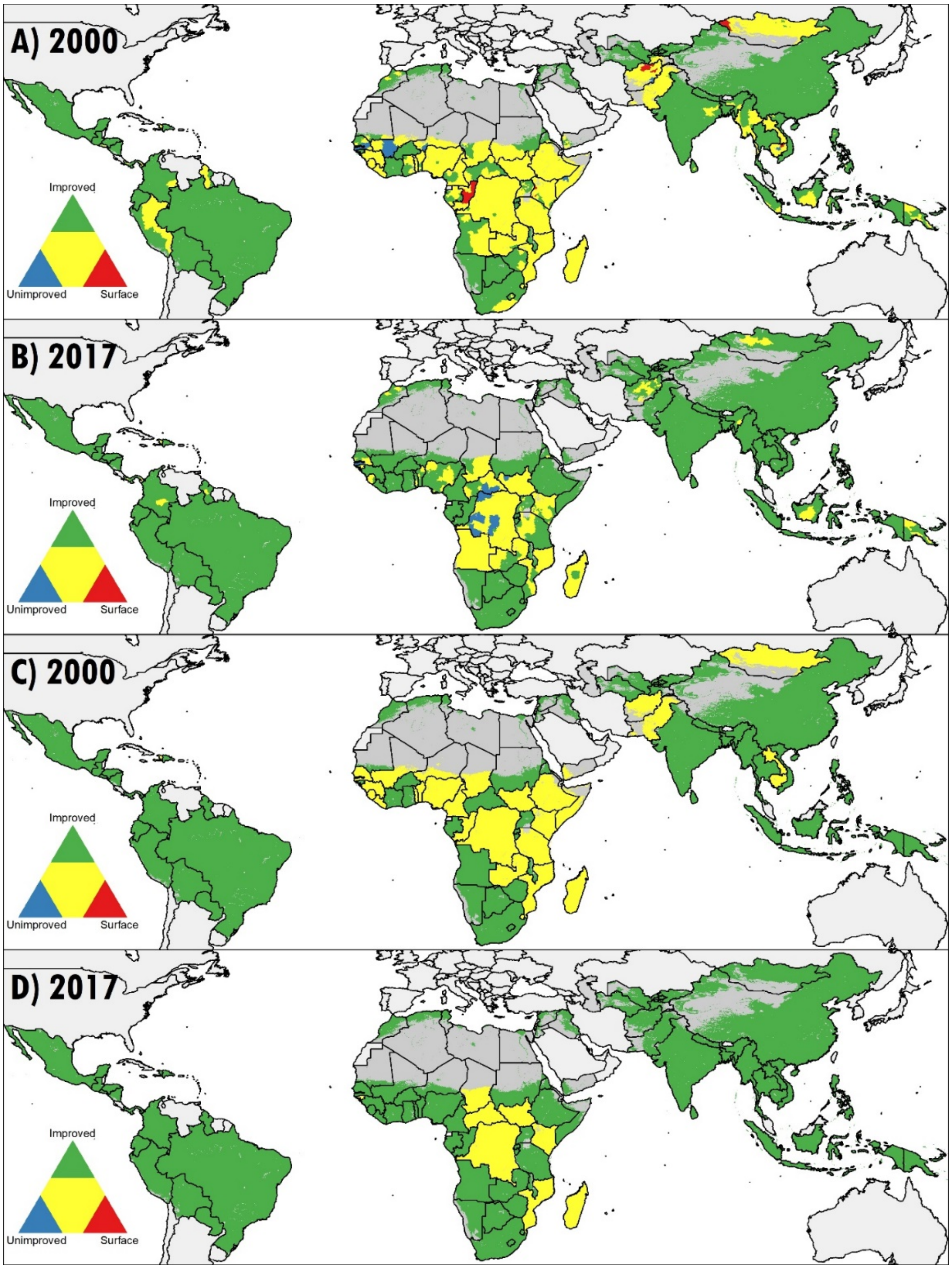

Appendix Figure 21. Ternary sanitation, at the first administrative and national level, 2000 and 2017

The co-distribution of improved, unimproved, and no facility access is shown for sanitation at the first administrative-level (**A**: 2000, **B**: 2017) and national level (**C**: 2000, **D**: 2017). Green denotes first administrative-level units where the majority of the population (>60%) had access to improved facilities, blue denotes >60% reliance on unimproved, and red denotes >60% having no facility access. Yellow indicates that there was no single dominant facility type used by >60% of the unit's population. Maps reflect administrative boundaries, land cover, lakes, and population; grey-coloured grid cells were classified as "barren or sparsely vegetated" and had fewer than ten people per 1 × 1-km grid cell, or were not included in these analyses.[5,11–15]

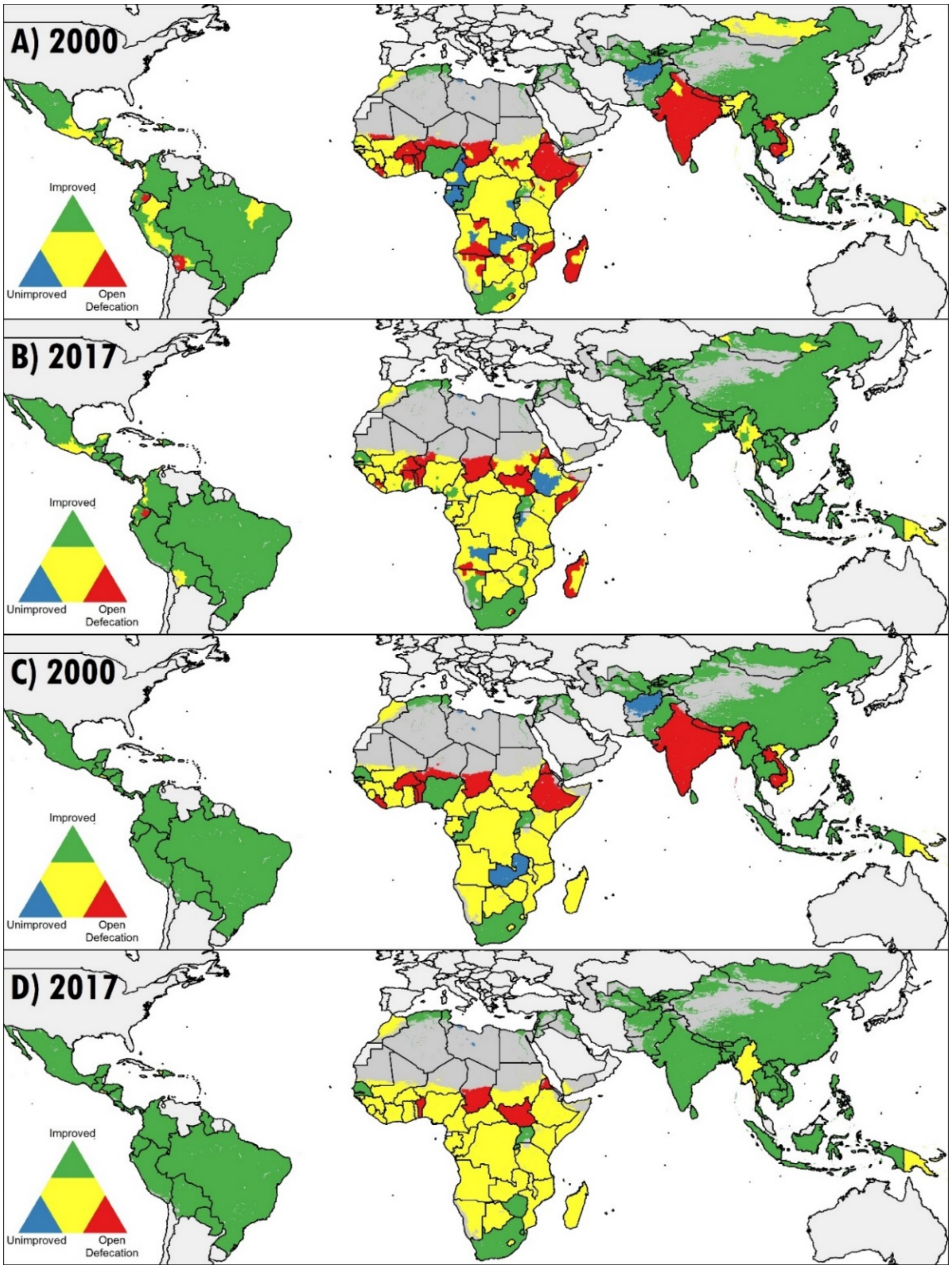

Appendix Figures 22–39. In sample prediction comparisons

For each indicator modelled, predictions were generated for each of the INLA model as described above before being harmonized to national trends (Section 3.6.1). These predictions were compared against the data to generate scatter plots for comparison. This analysis was conducted at country, first administrative, and second administrative geographical scales where the predictions and the dataset were aggregated to the corresponding scale prior to plotting for comparison. In the following analyses, certain countries were excluded due to data sparsity for the particular analytical framework as follows: 1) Bolivia, Brazil, Gabon, Indonesia, Cambodia, Madagascar, Peru, Papua New Guinea, Tanzania, South Afirca, and Vietnam for piped water, 2) Bolivia, Brazil, Botswana, Central African Republic, Gambia, Indonesia, Kyrgyzstan, Morocco, and Mongolia for conditional improved water, 3) Gambia, Jordan, and Libya for conditional unimproved water, 3) Mexico and Swaziland for sewer or septic sanitation, 4) Mexico for conditional improved sanitation, and 5) Libya and Mozambique for conditional unimproved sanitation.

Appendix Figure 22. In sample statistics for piped water model by country and year

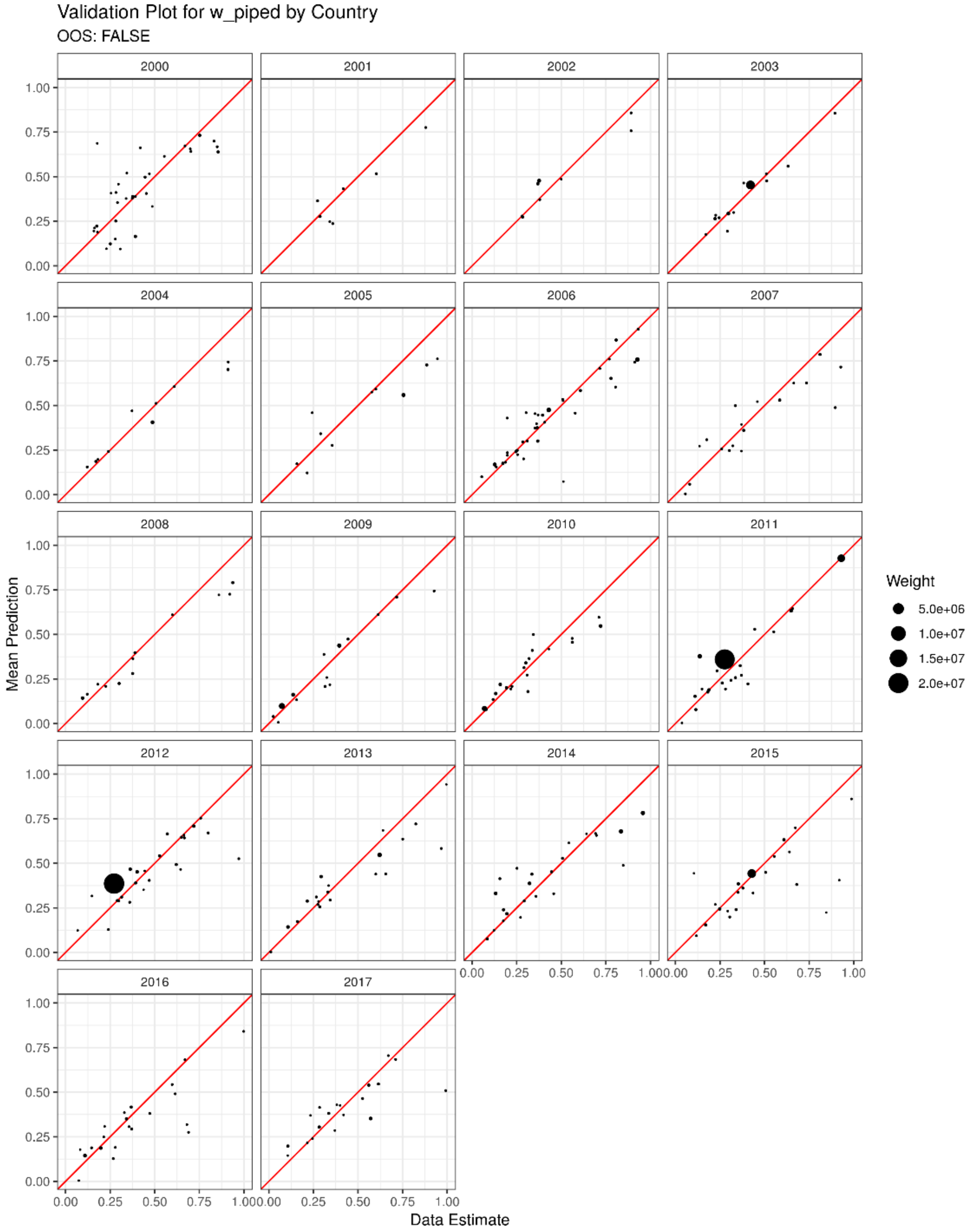

Appendix Figure 23. In sample statistics for piped water model by first administrative level and year

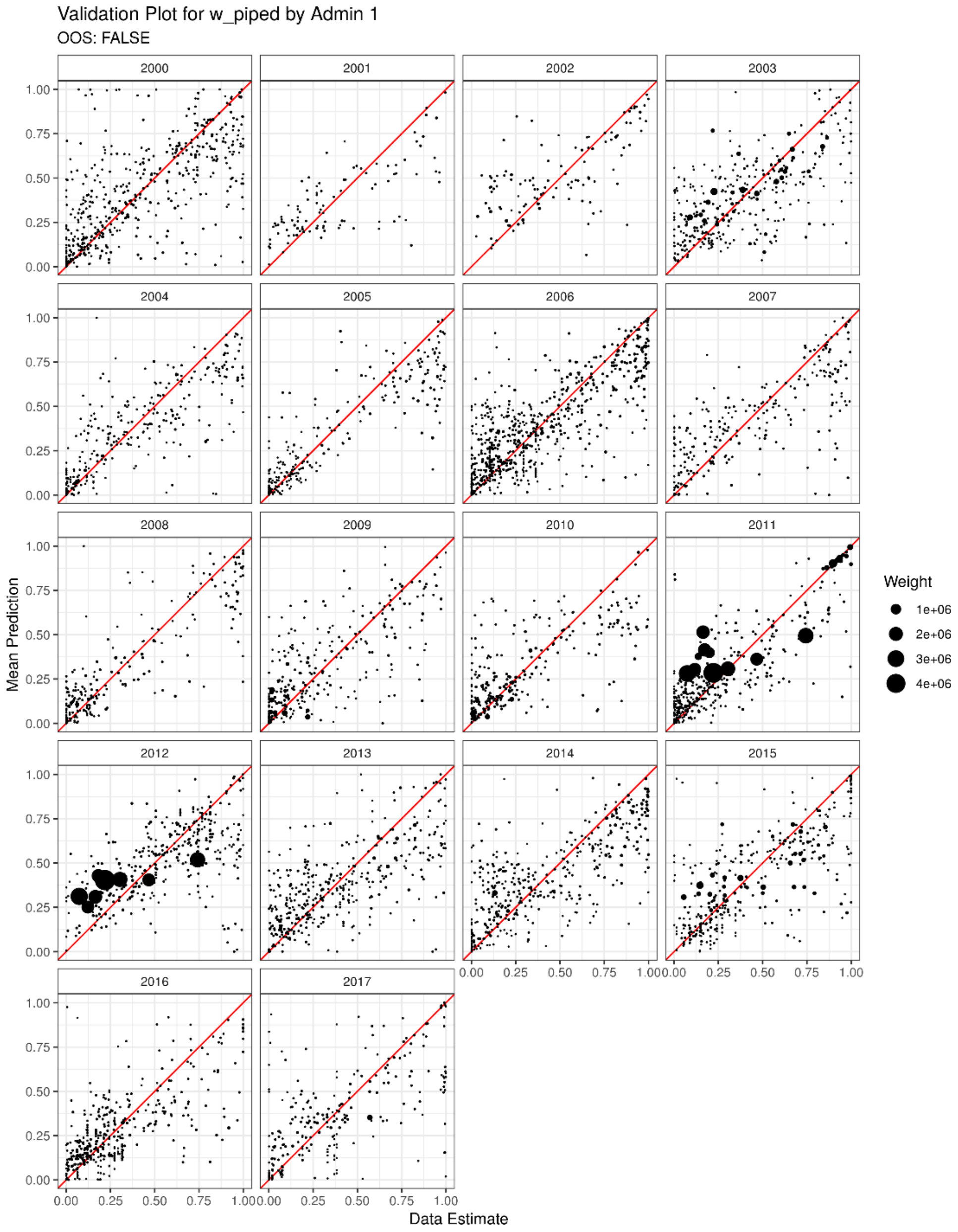

Appendix Figure 24. In sample statistics for piped water model by second administrative level and year

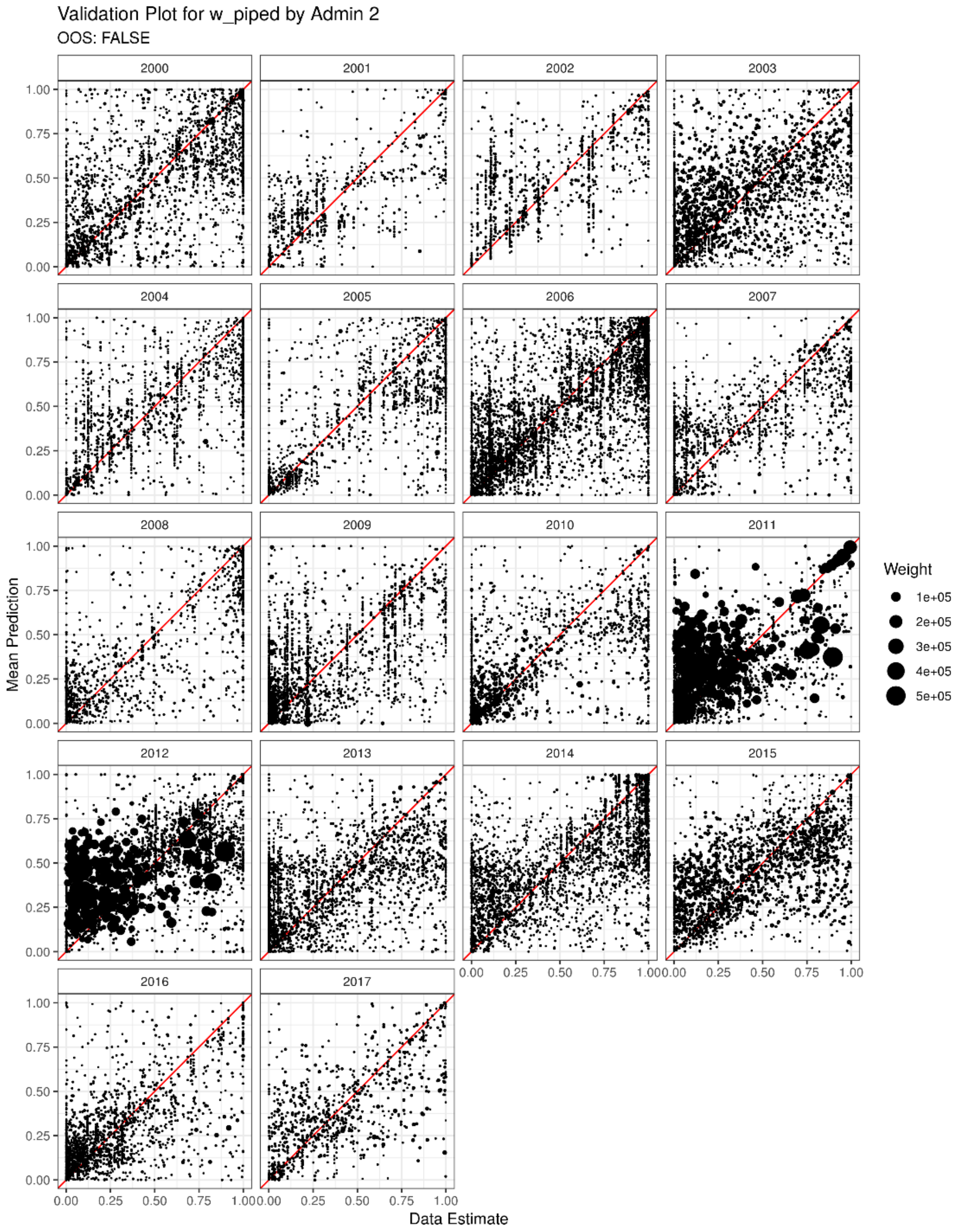

Appendix Figure 25. In sample statistics for conditional improved water model by country and year

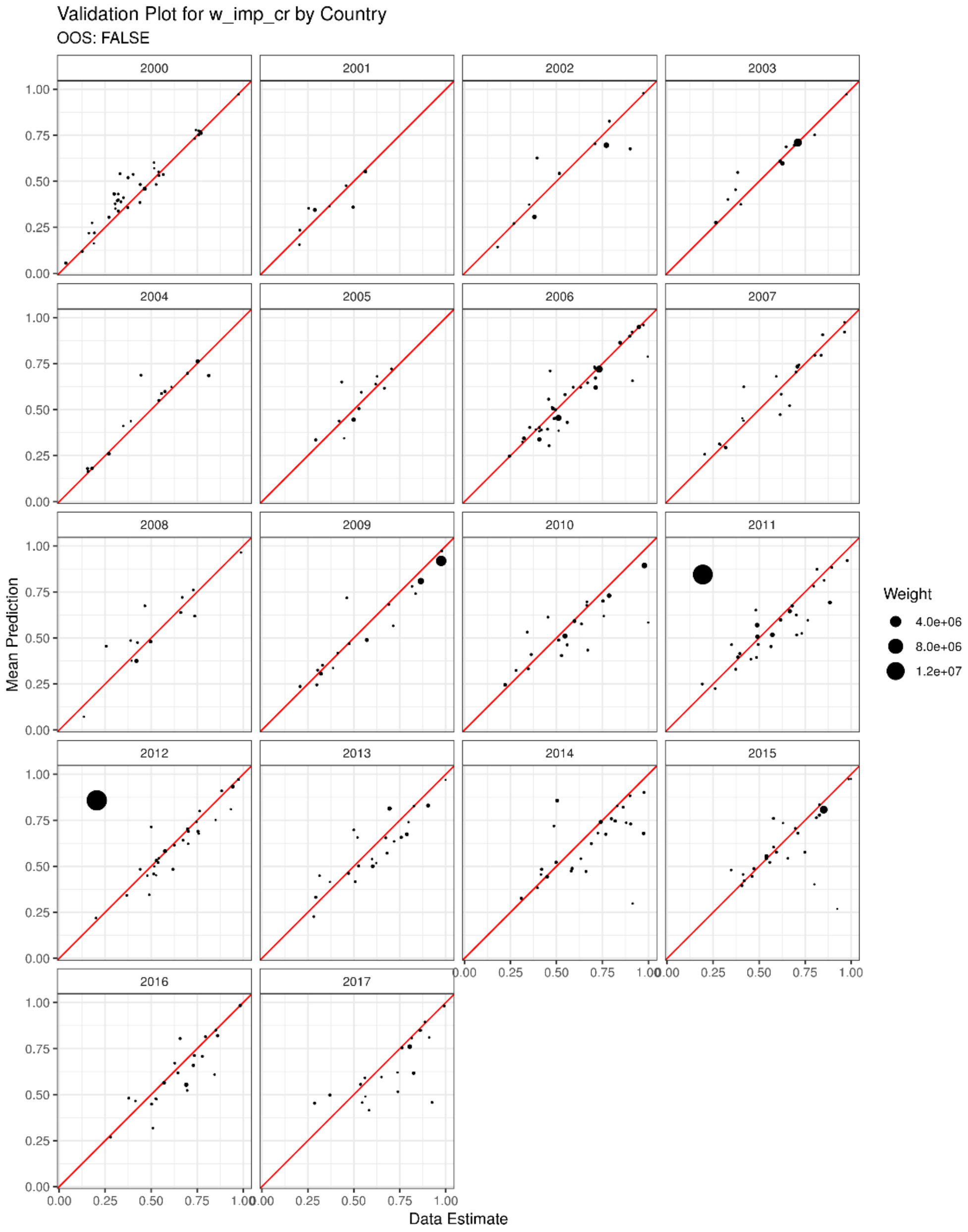

Appendix Figure 26. In sample statistics for conditional improved water model by first administrative level and year

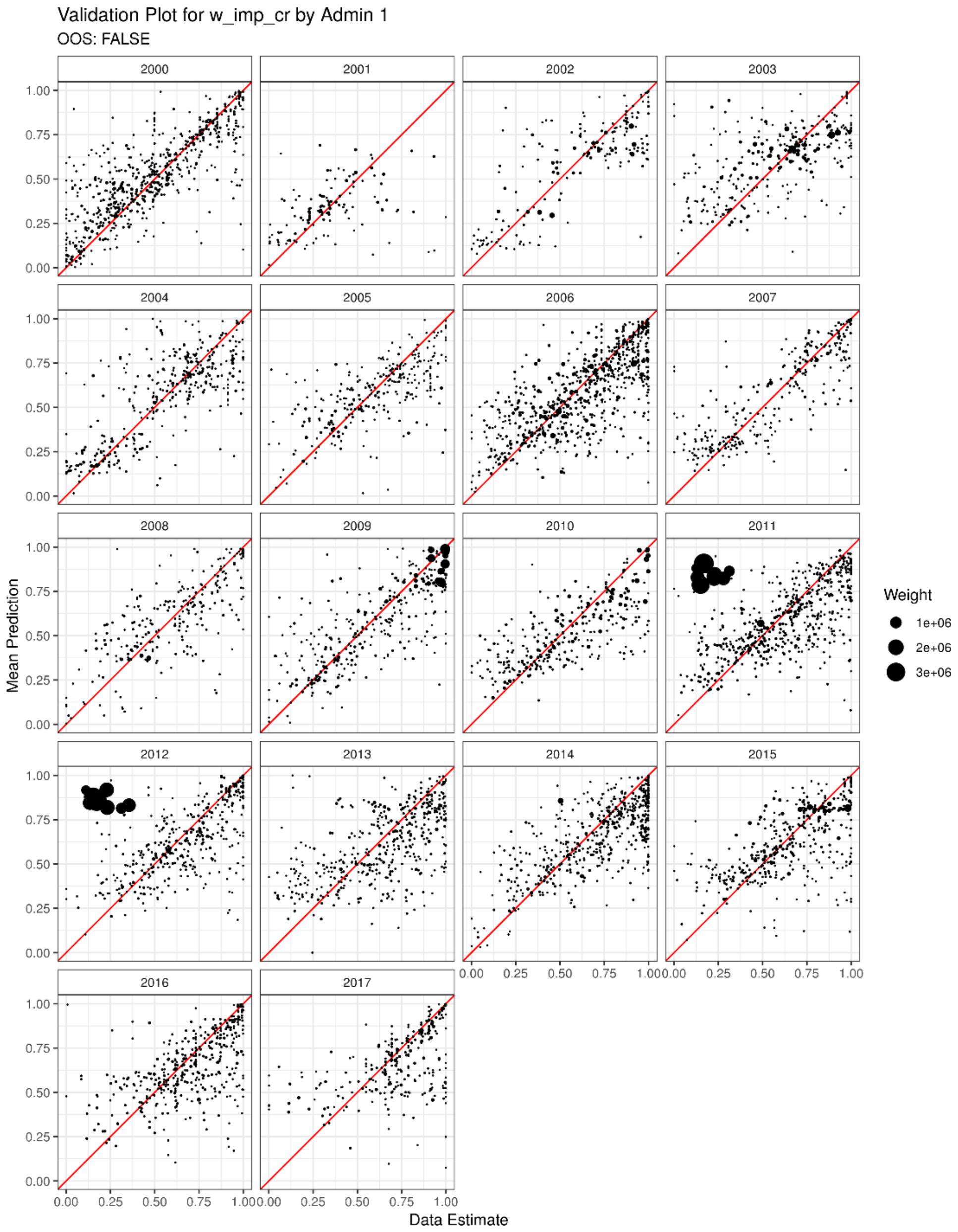

Appendix Figure 27. In sample statistics for conditional improved water model by second administrative level and year

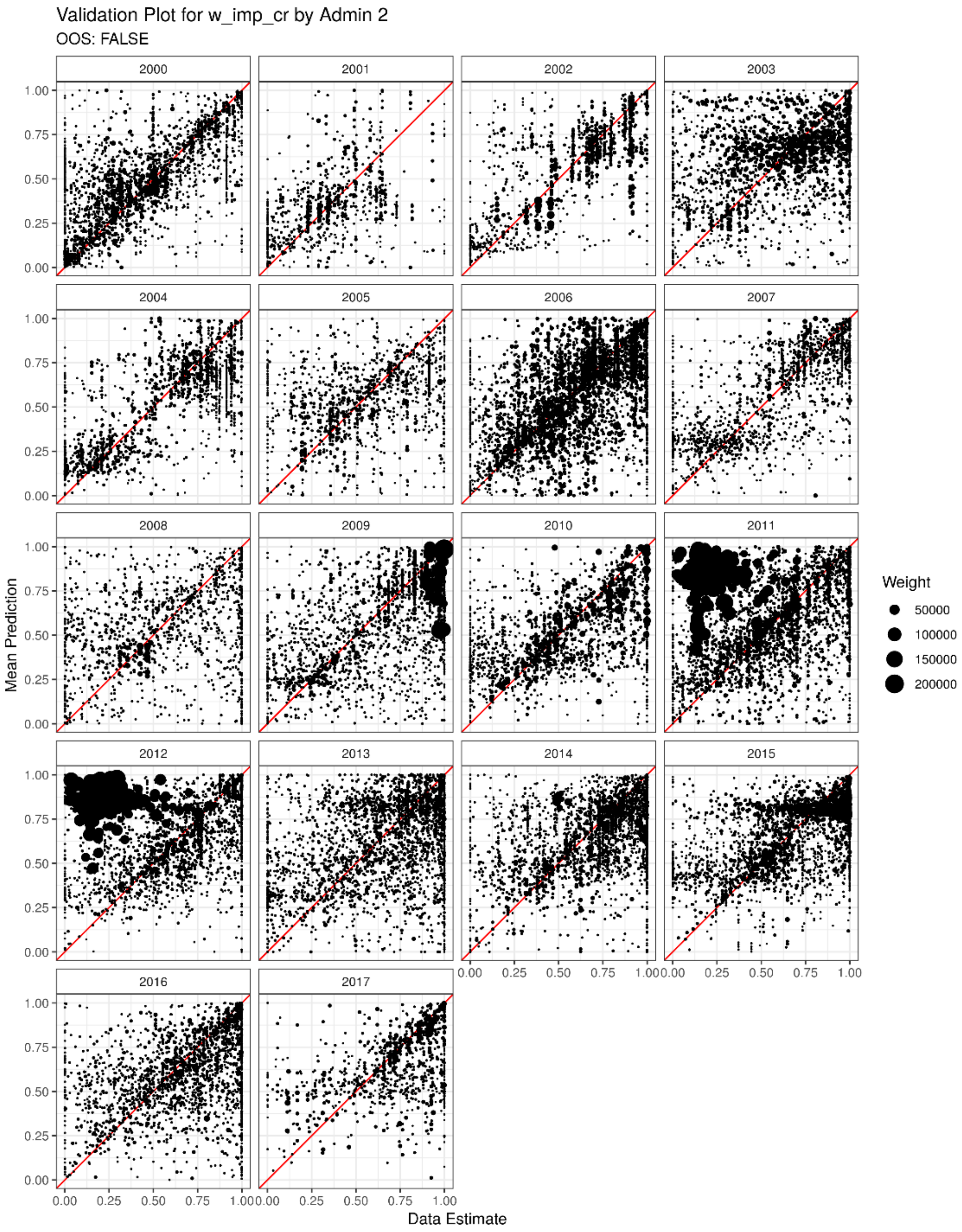

Appendix Figure 28. In sample statistics for conditional unimproved water model by country and year

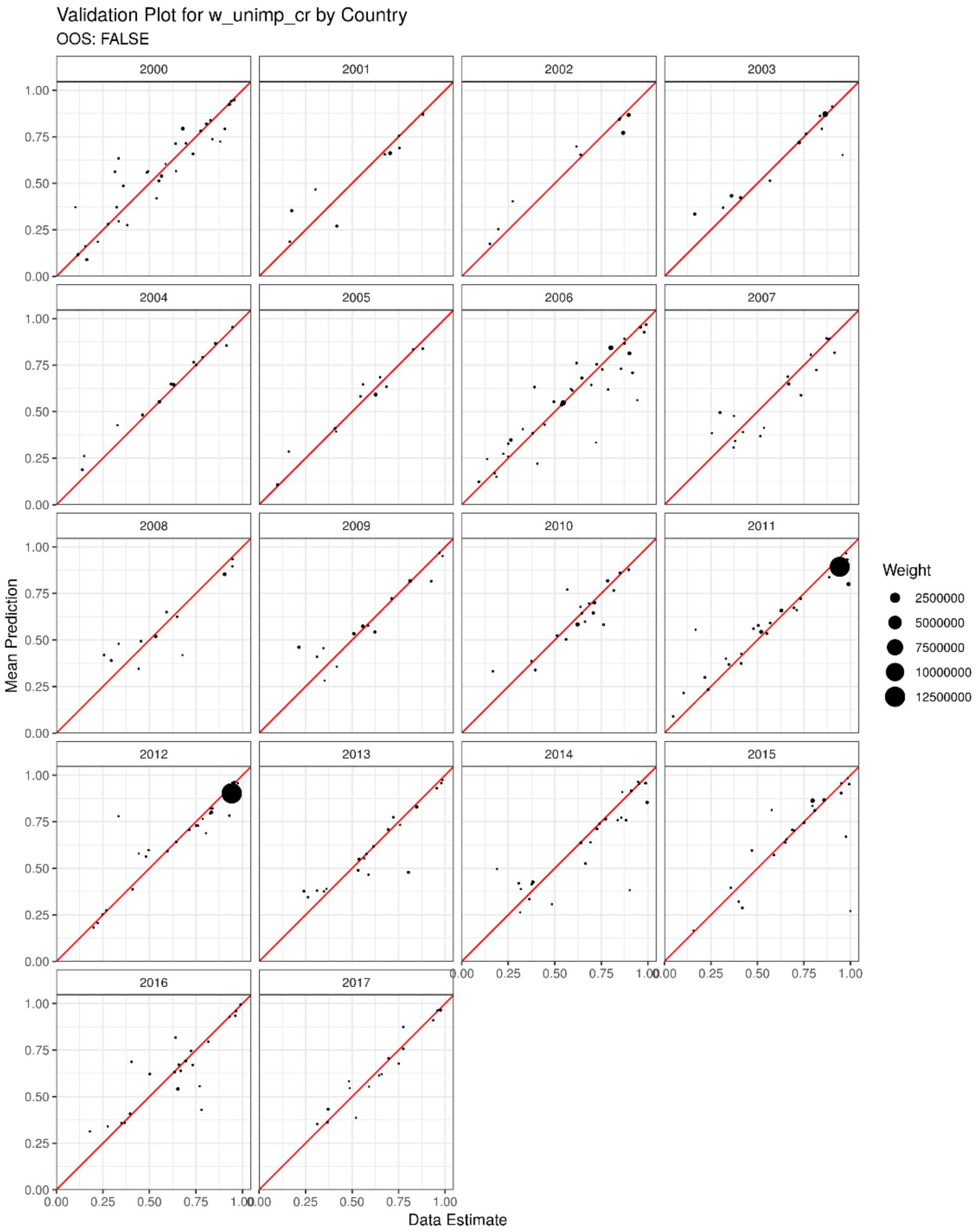

Appendix Figure 29. In sample statistics for conditional unimproved water model by first administrative level and year

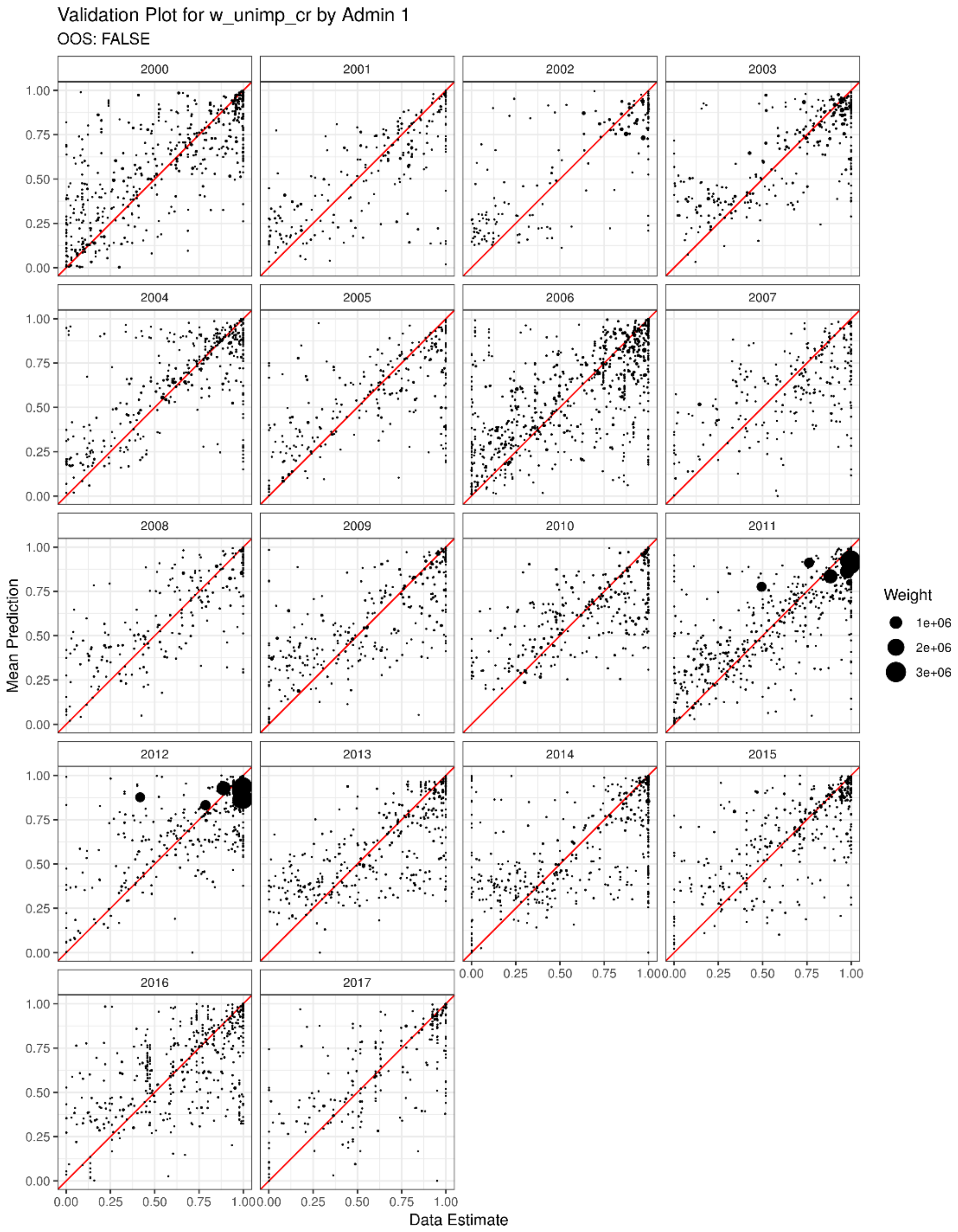

Appendix Figure 29. In sample statistics for conditional unimproved water model by second administrative level and year

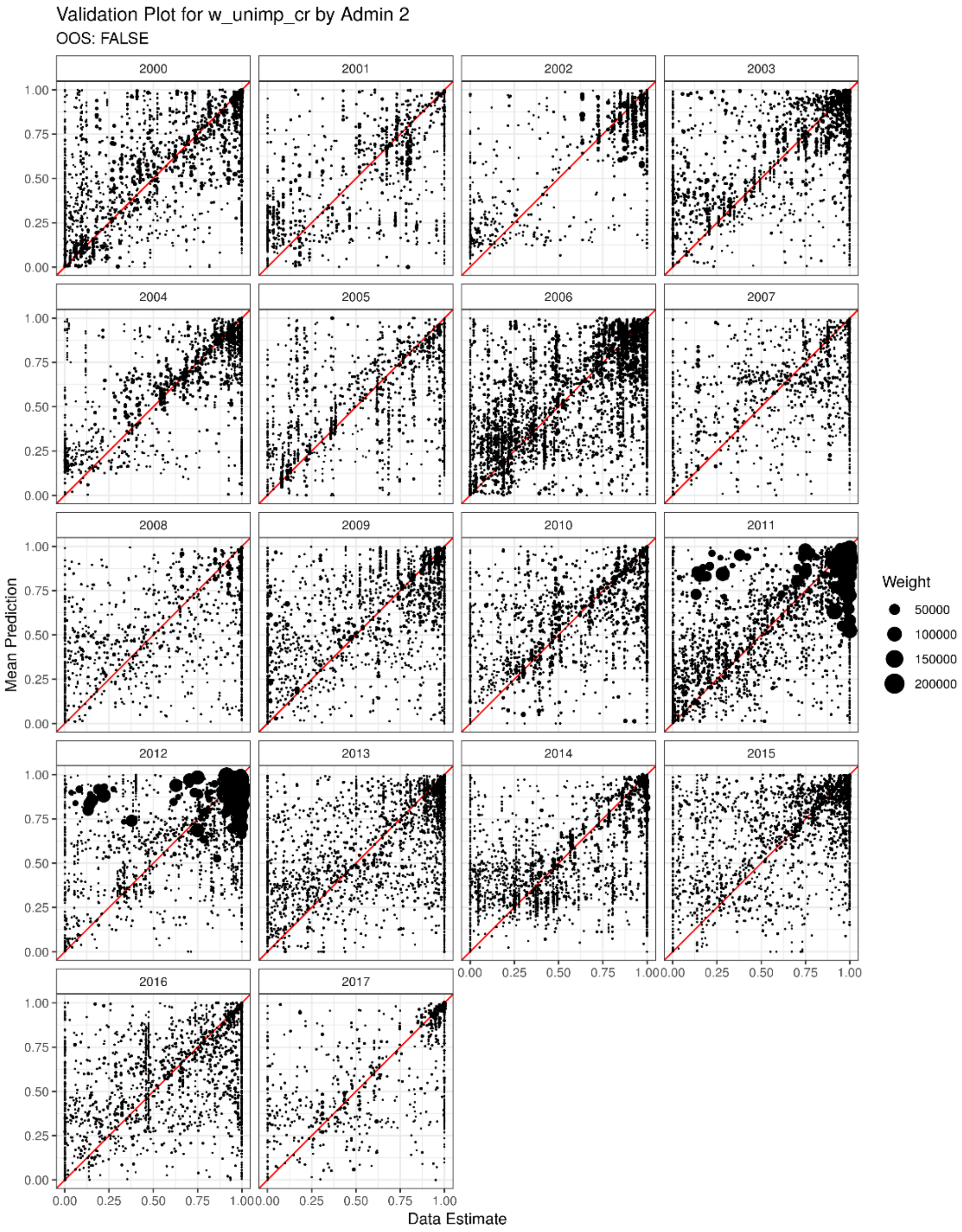

Appendix Figure 30. In sample statistics for piped sanitation model by country and year

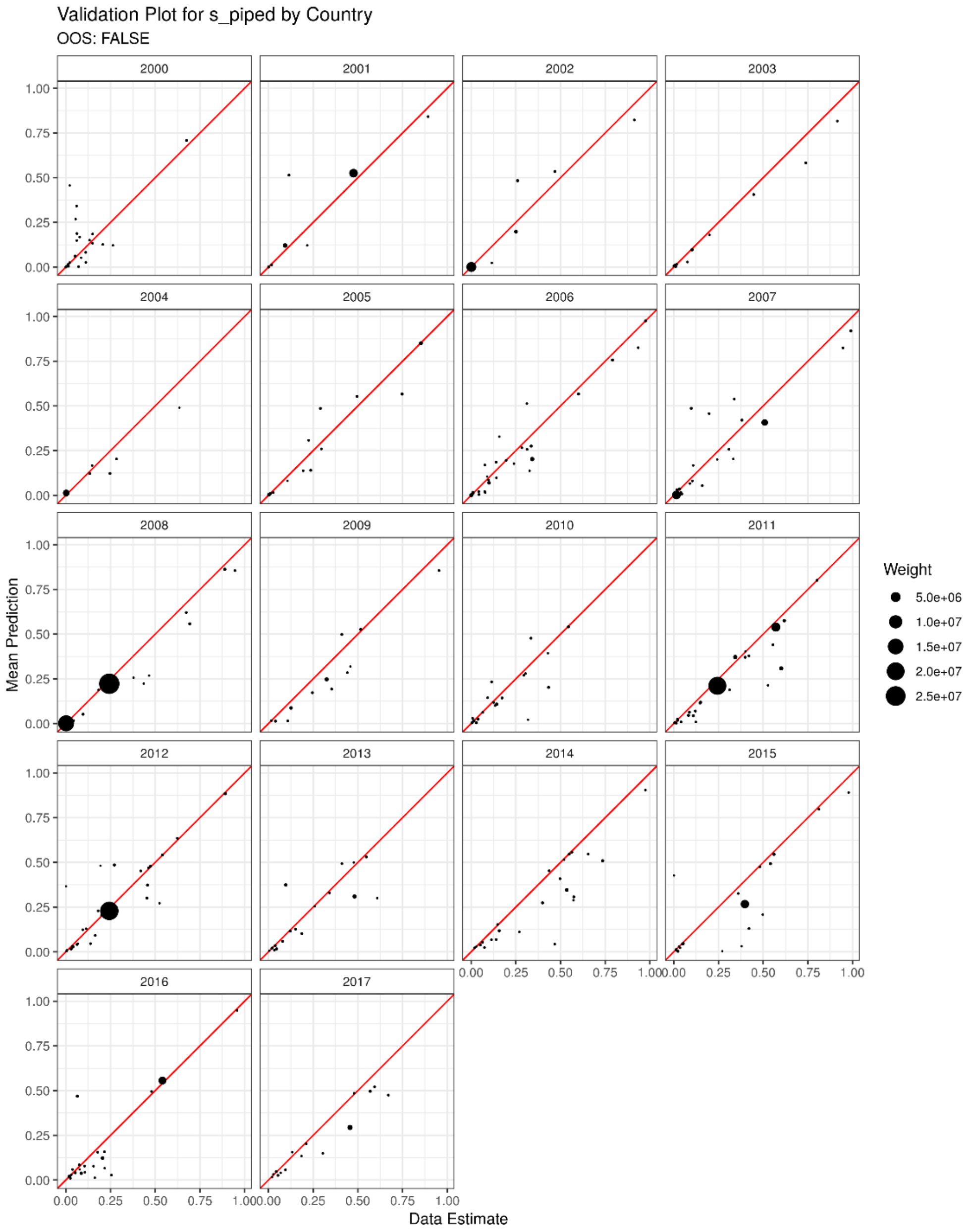

Appendix Figure 31. In sample statistics for piped sanitation model by first administrative level and year

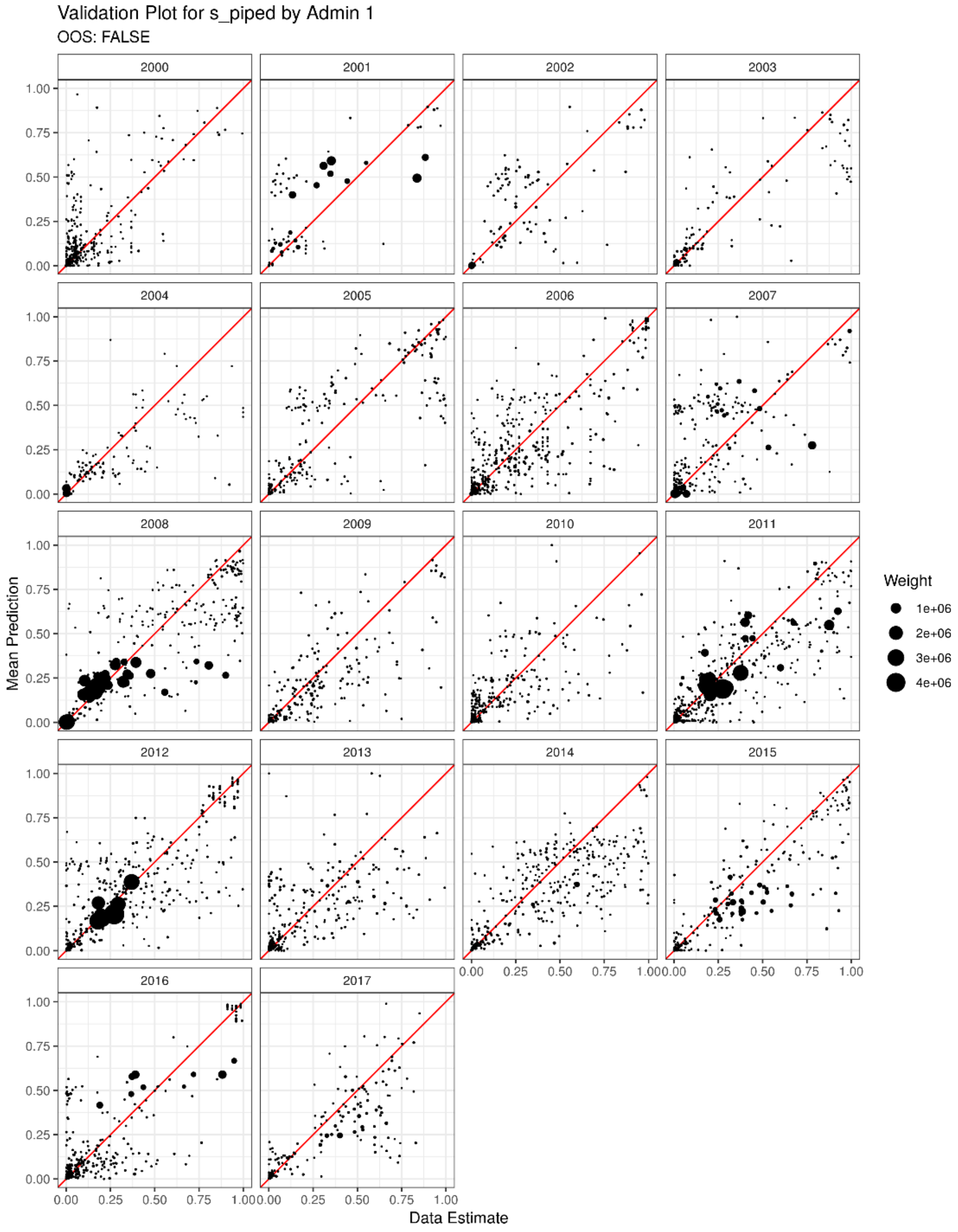

Appendix Figure 32. In sample statistics for piped sanitation model by second administrative level and year

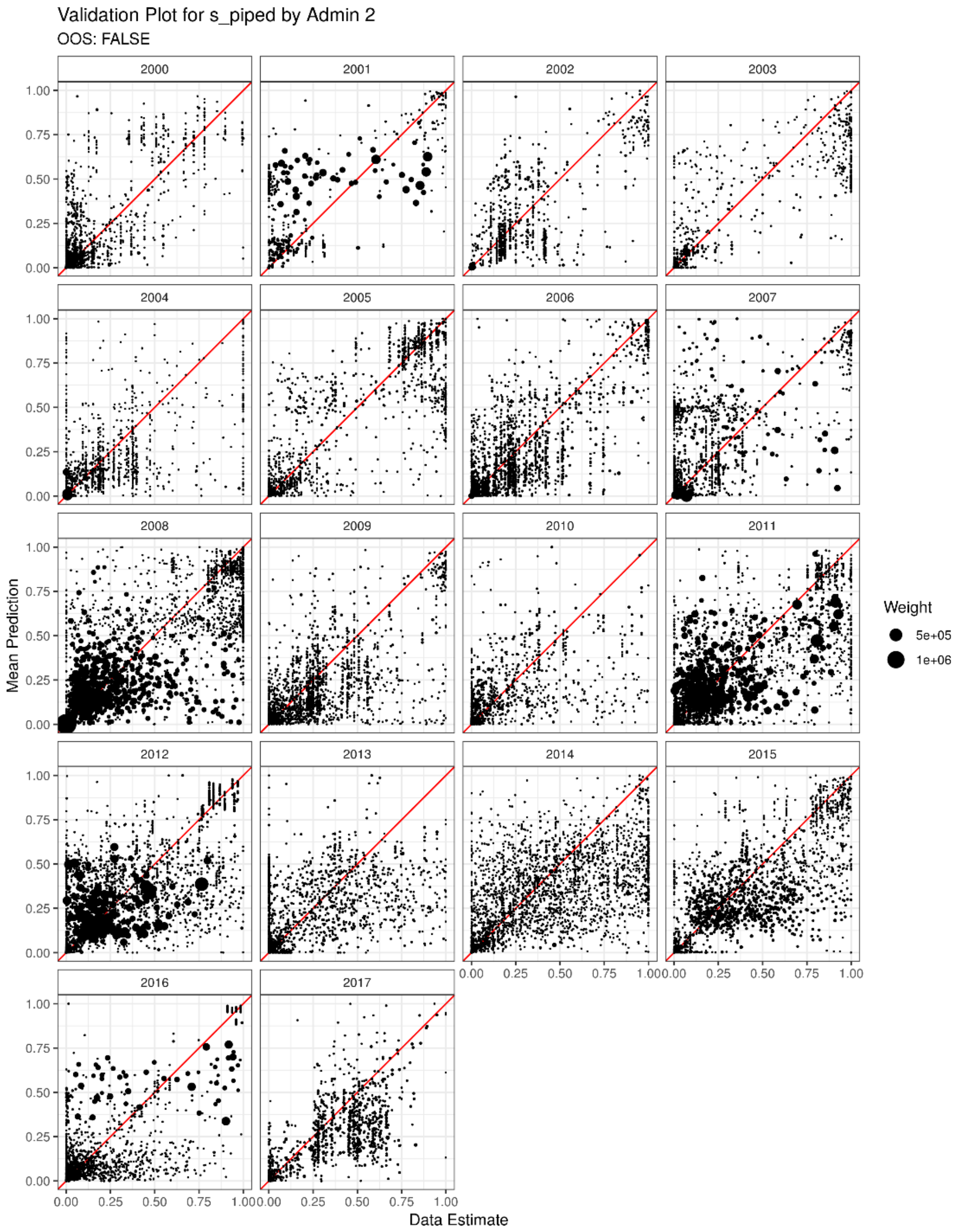

Appendix Figure 33. In sample statistics for conditional improved sanitation model by country and year

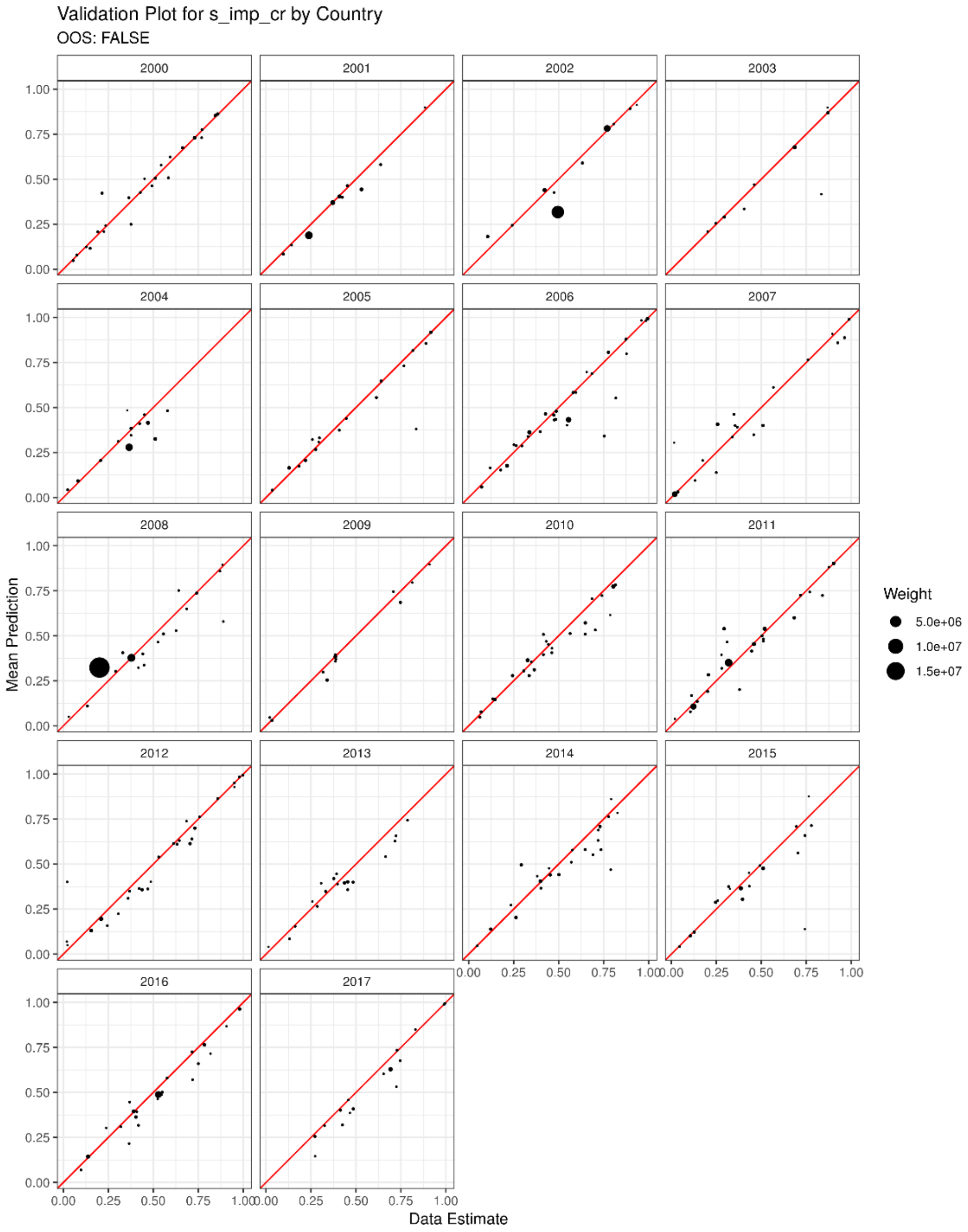

Appendix Figure 34. In sample statistics for conditional improved sanitation model by first administrative level and year

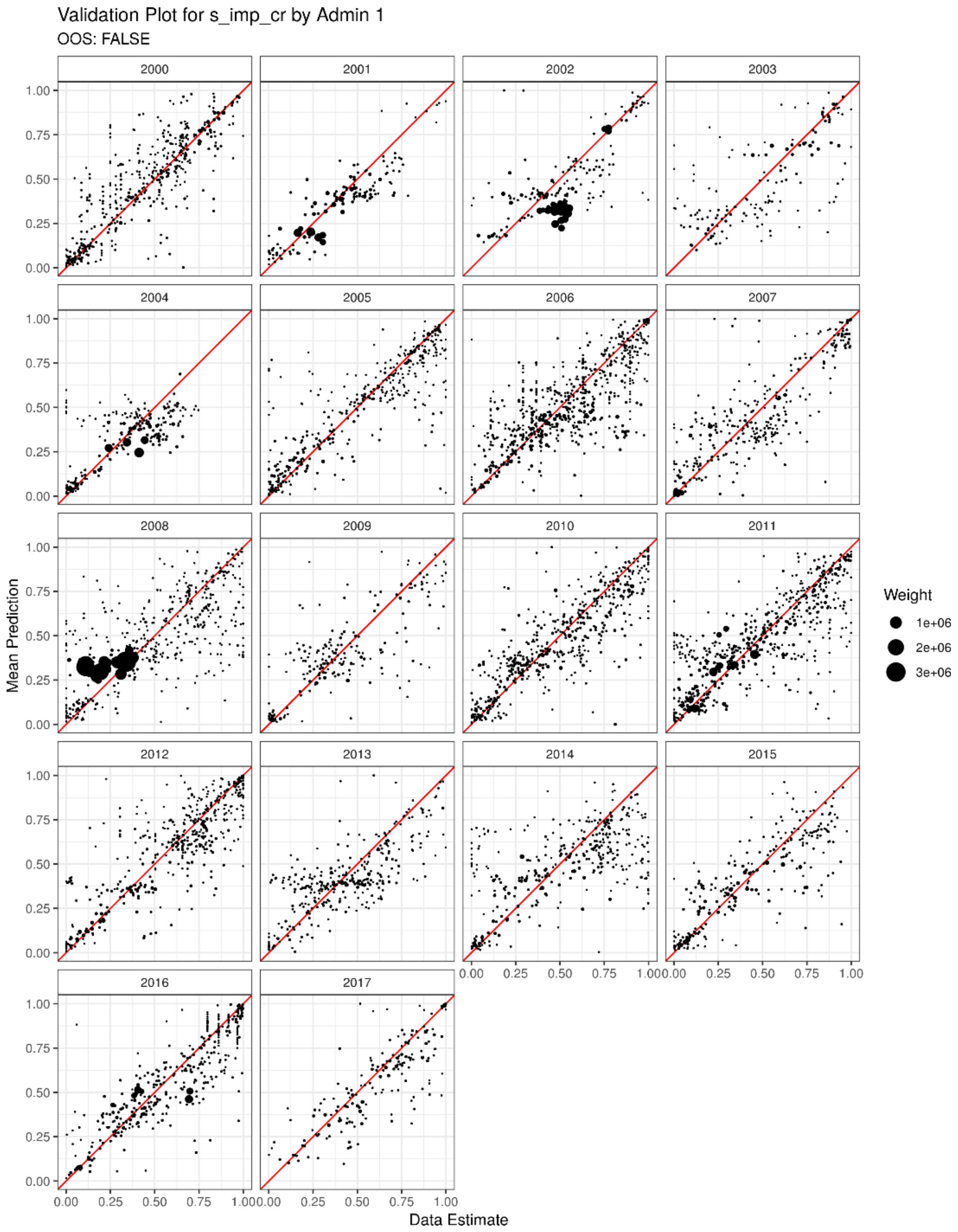

Appendix Figure 35. In sample statistics for conditional improved sanitation model by second administrative level and year

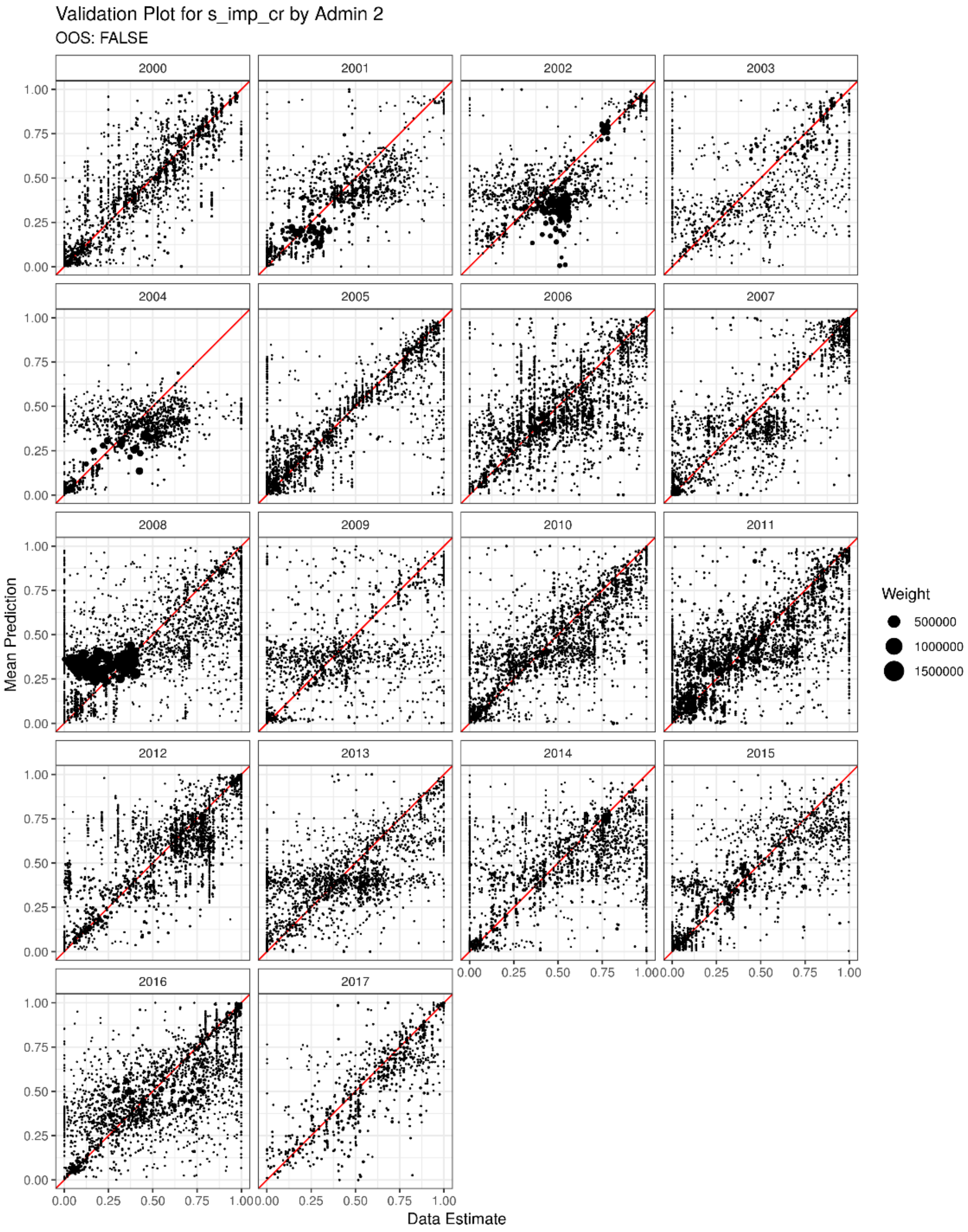

Appendix Figure 36. In sample statistics for conditional unimproved sanitation model by country and year

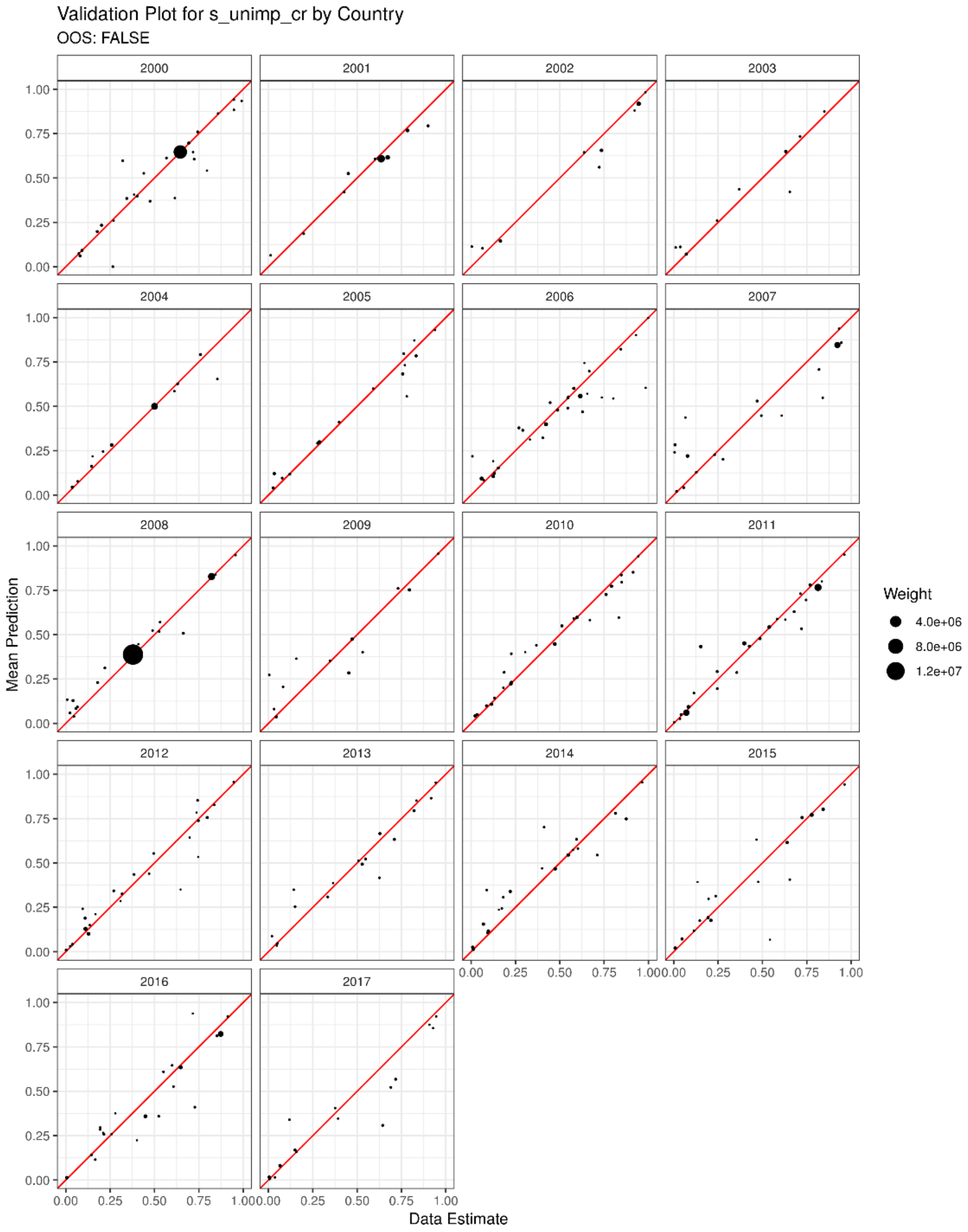

Appendix Figure 37. In sample statistics for conditional unimproved sanitation model by first administrative level and year

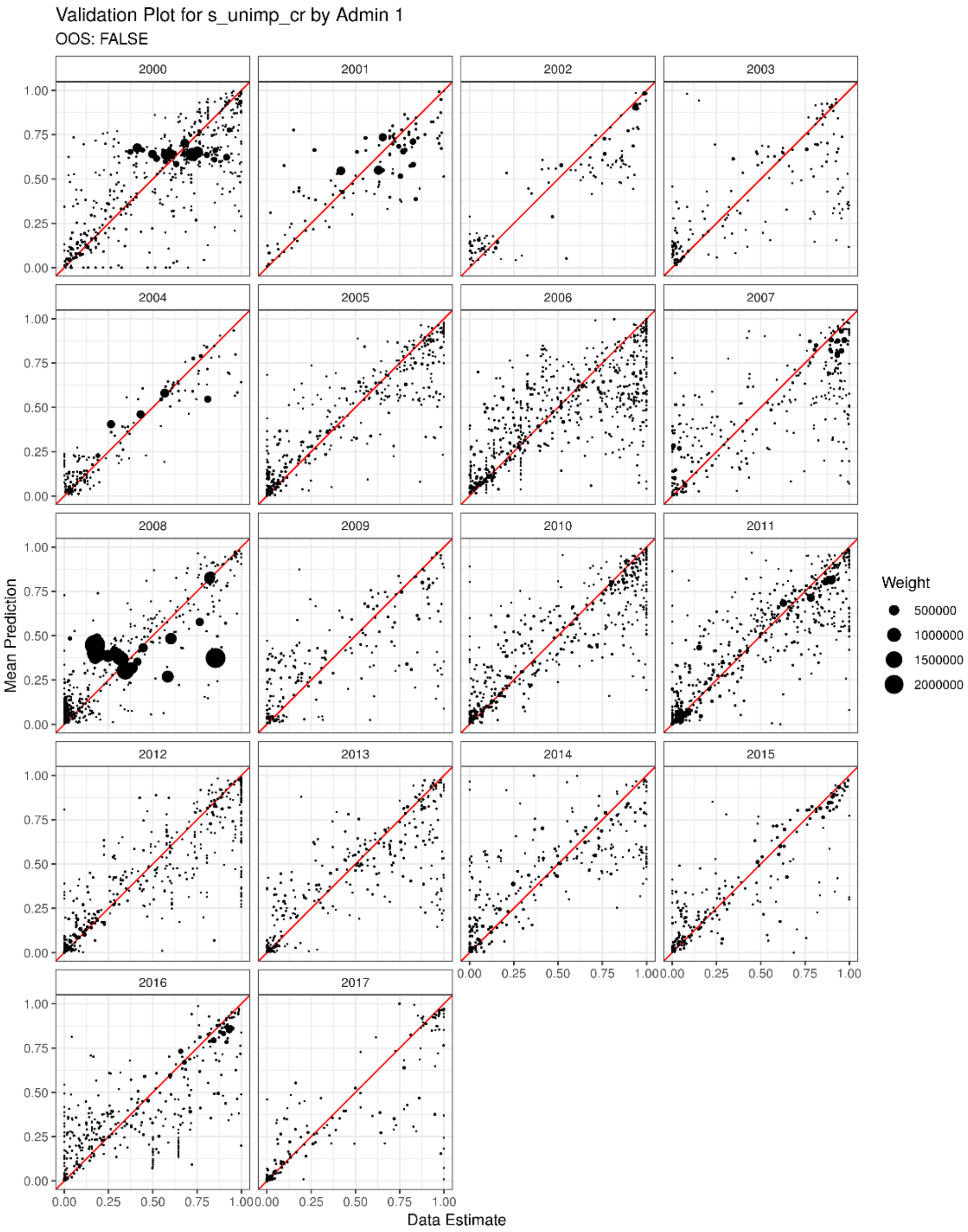

Appendix Figure 38. In sample statistics for conditional unimproved sanitation model by second administrative level and year

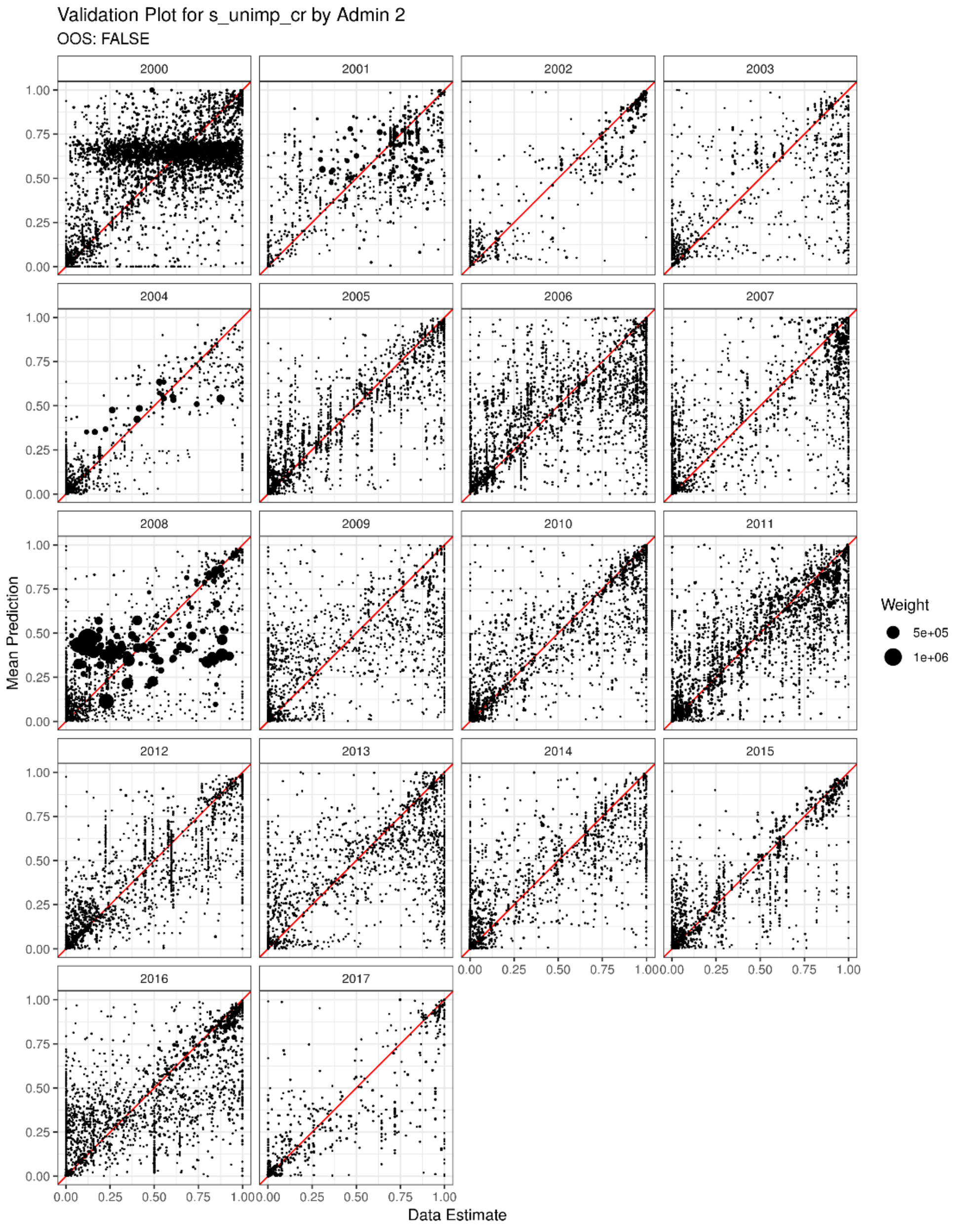

Appendix Figures 40–57. Out-of-sample prediction comparisons

For each indicator modelled, predictions were generated for each of the five holdout datasets in the fivefold cross validation framework described above (Section 3.6.2). These predictions were compared against the data to generate scatter plots for comparison. This analysis was conducted at country, first administrative, and second administrative geographical scales where the predictions and the dataset were aggregated to the corresponding scale prior to plotting for comparison. In the following analyses, certain countries were excluded due to data sparsity for the particular analytical framework as follows: 1) Bolivia, Brazil, Gabon, Indonesia, Cambodia, Madagascar, Peru, Papua New Guinea, Tanzania, South Afirca, and Vietnam for piped water, 2) Bolivia, Brazil, Botswana, Central African Republic, Gambia, Indonesia, Kyrgyzstan, Morocco, and Mongolia for conditional improved water, 3) Gambia, Jordan, and Libya for conditional unimproved water, 3) Mexico and Swaziland for sewer or septic sanitation, 4) Mexico for conditional improved sanitation, and 5) Libya and Mozambique for conditional unimproved sanitation.

Appendix Figure 39. Out-of-sample statistics for piped water model by country and year

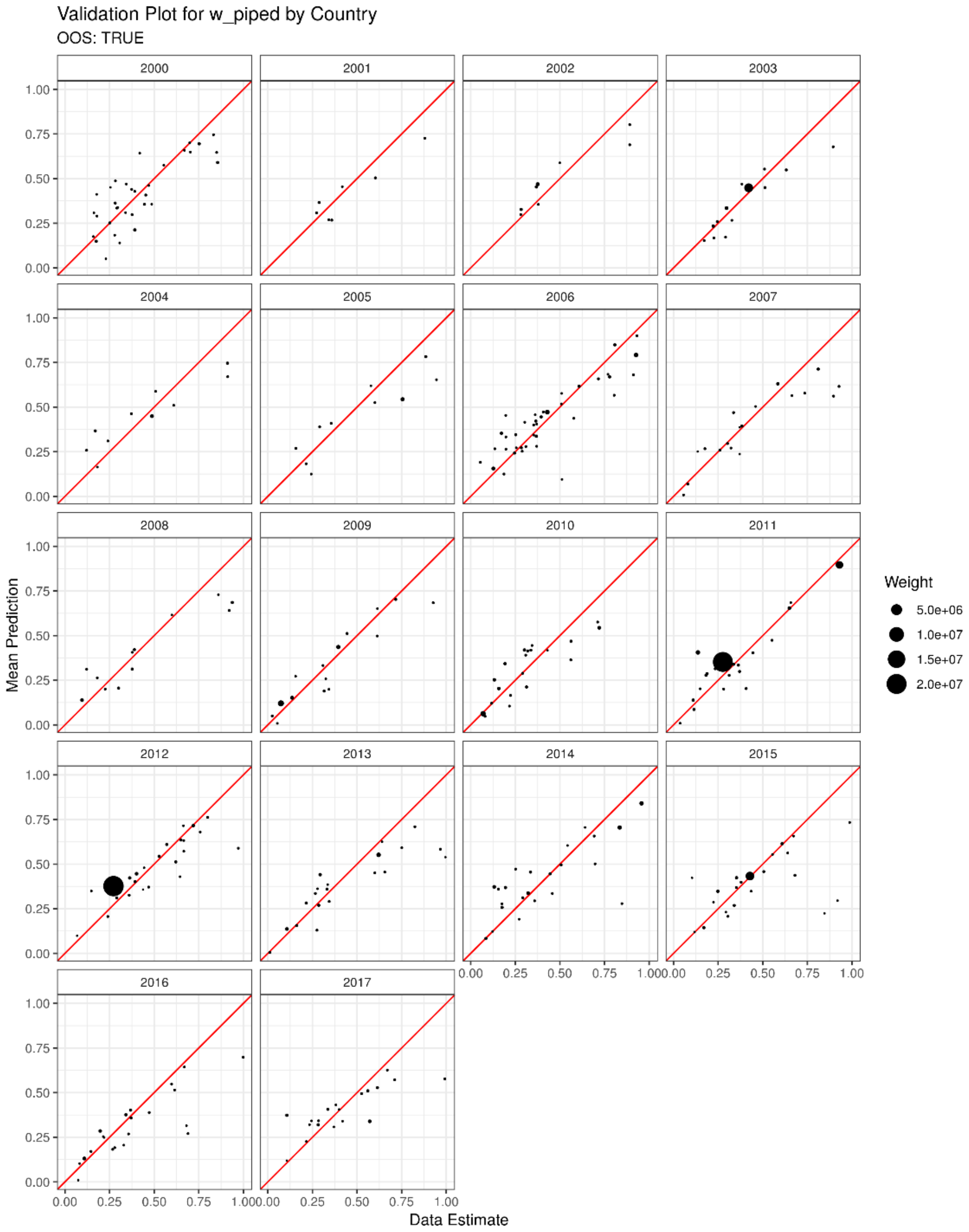

Appendix Figure 40. Out-of-sample statistics for piped water model by first administrative level and year

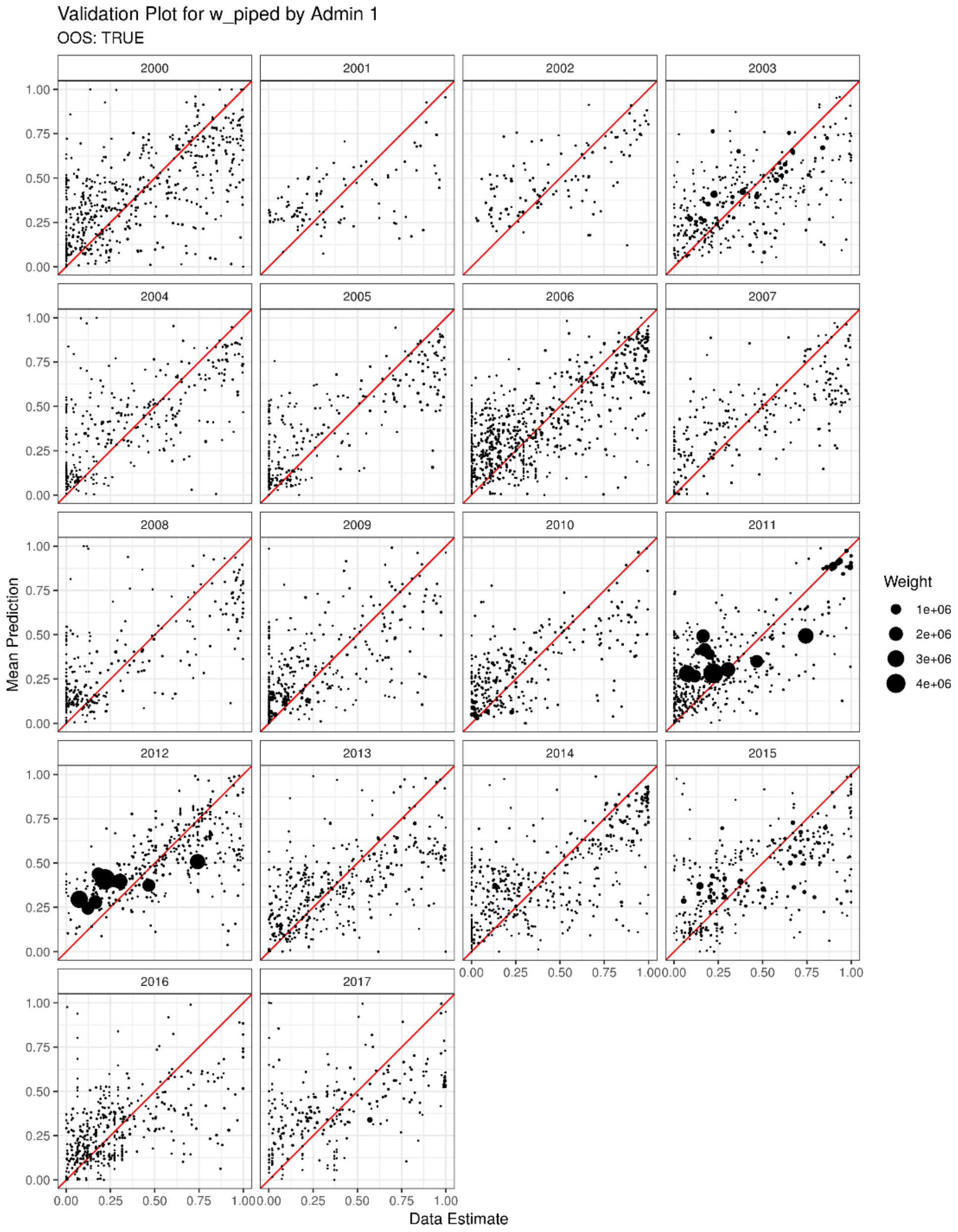

Appendix Figure 41. Out-of-sample statistics for piped water model by second administrative level and year

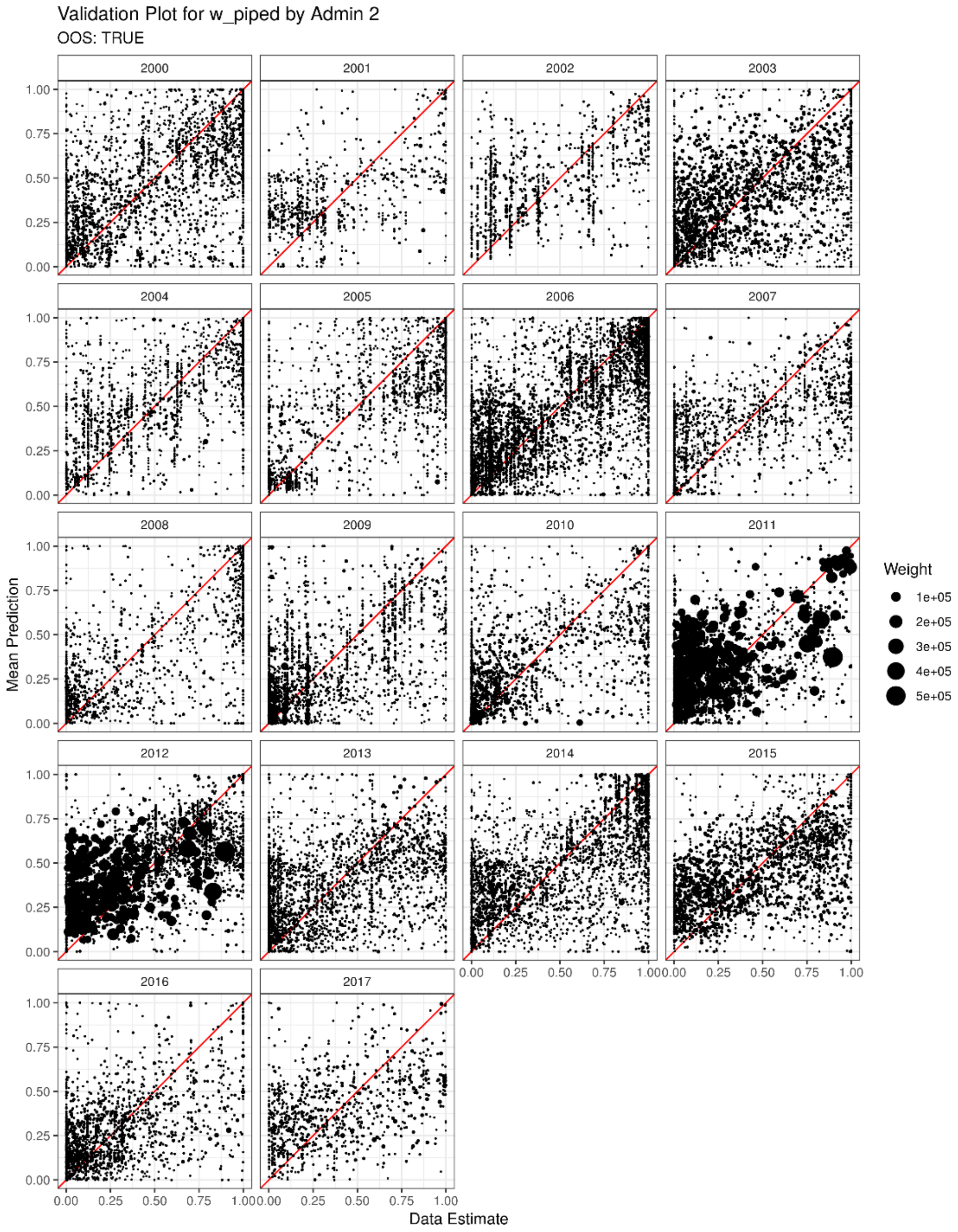

Appendix Figure 42. Out-of-sample statistics for conditional improved water model by country and year

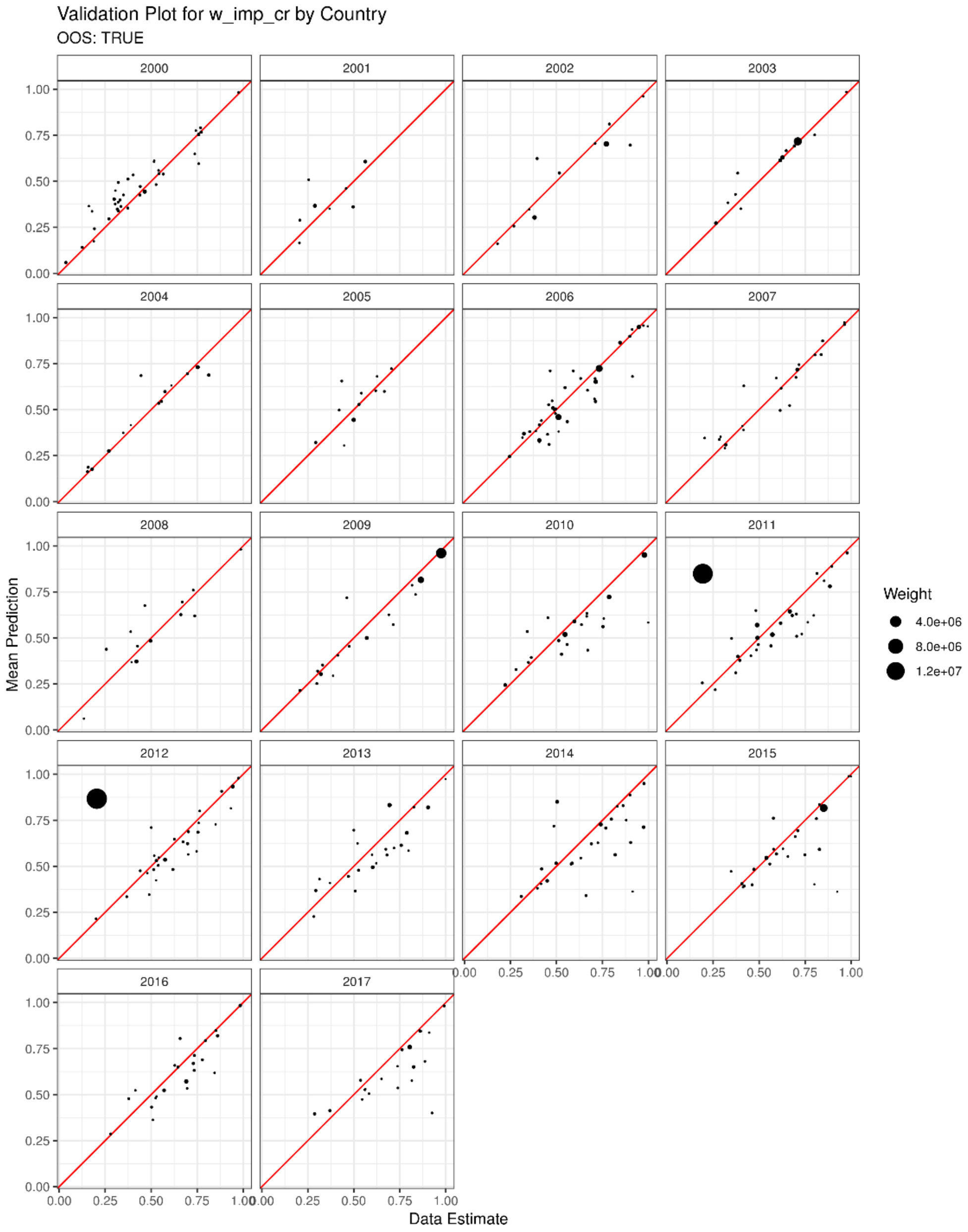

Appendix Figure 43. Out-of-sample statistics for conditional improved water model by first administrative level and year

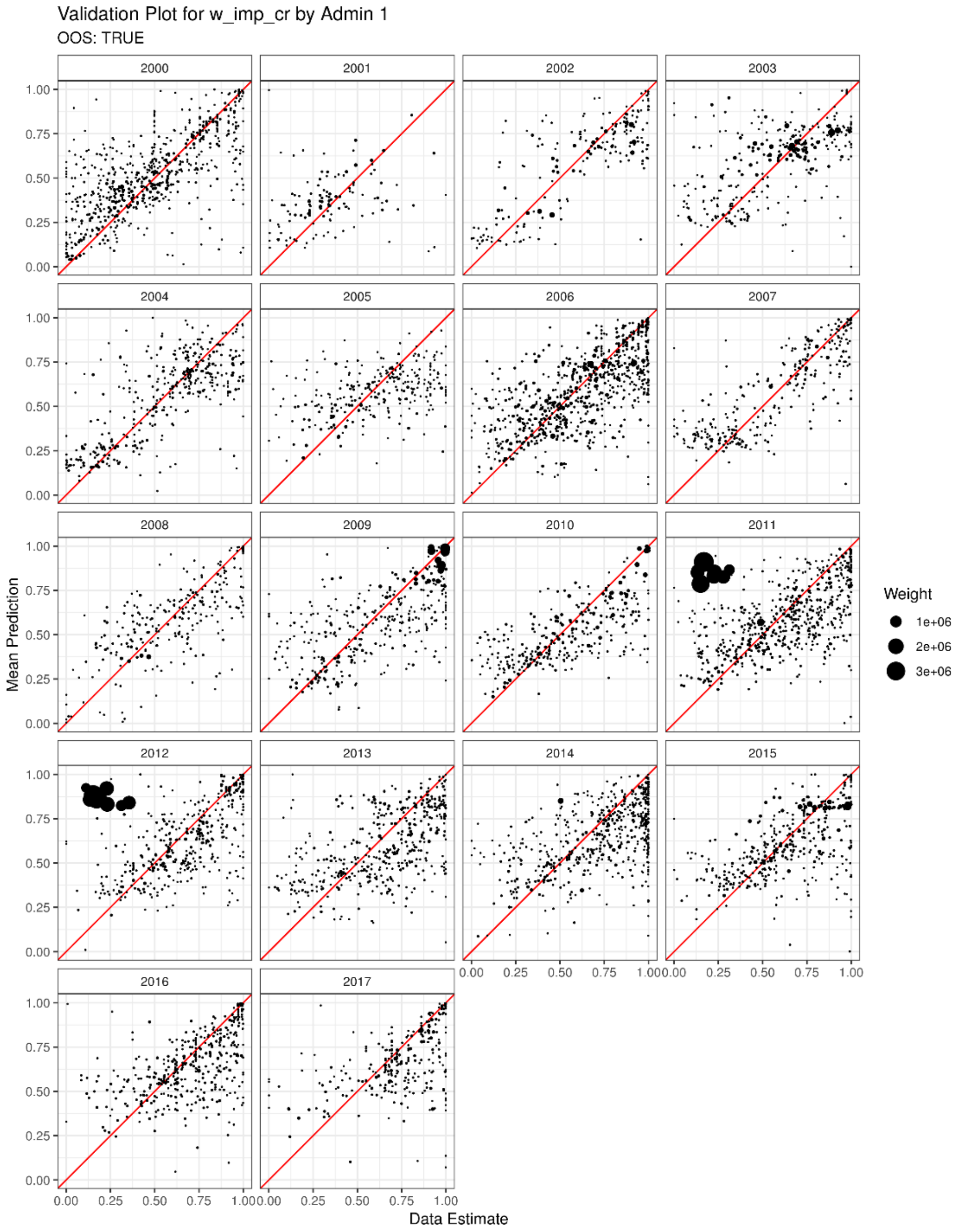

Appendix Figure 44. Out-of-sample statistics for conditional improved water model by second administrative level and year

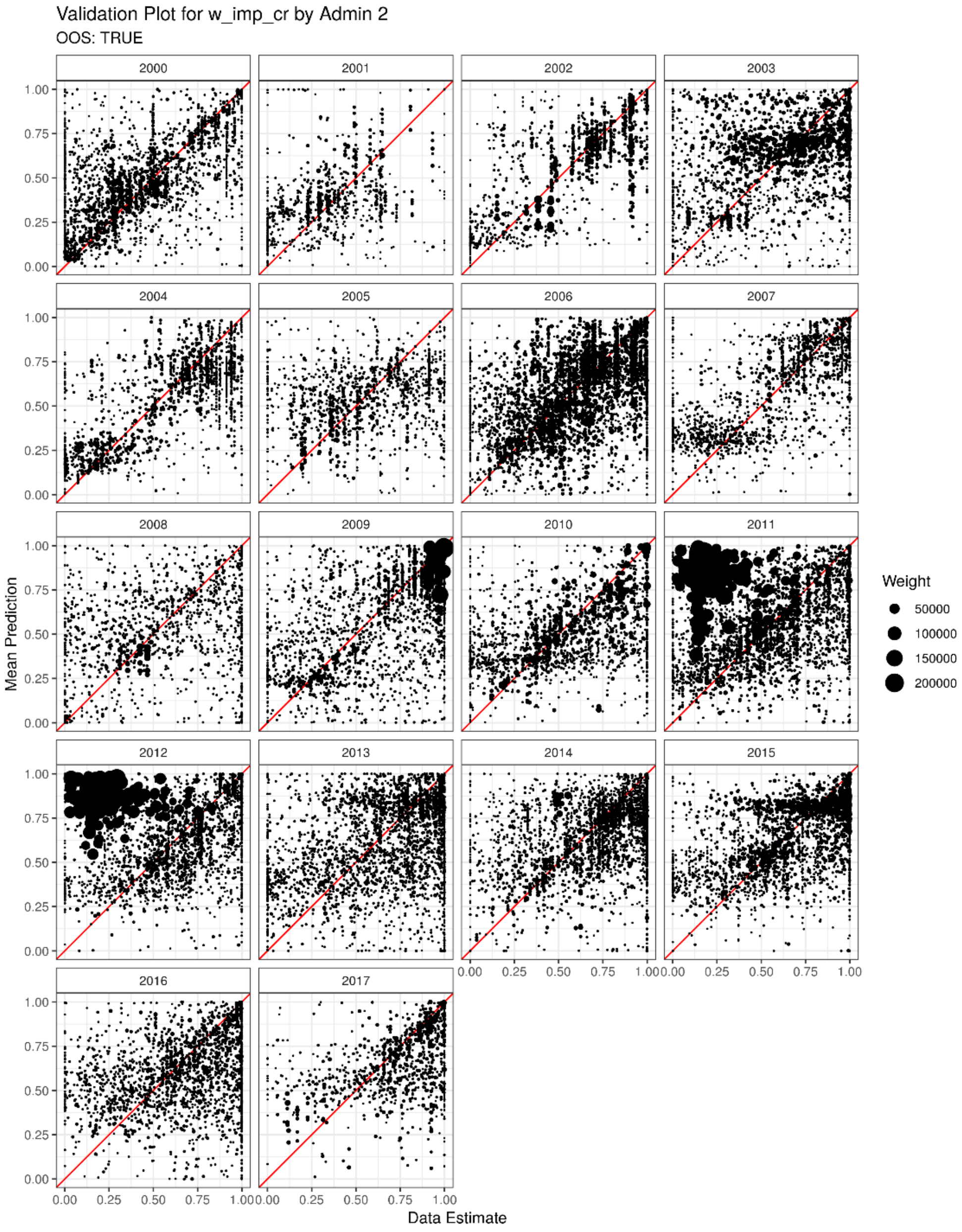

Appendix Figure 45. Out-of-sample for conditional unimproved water model by country and year

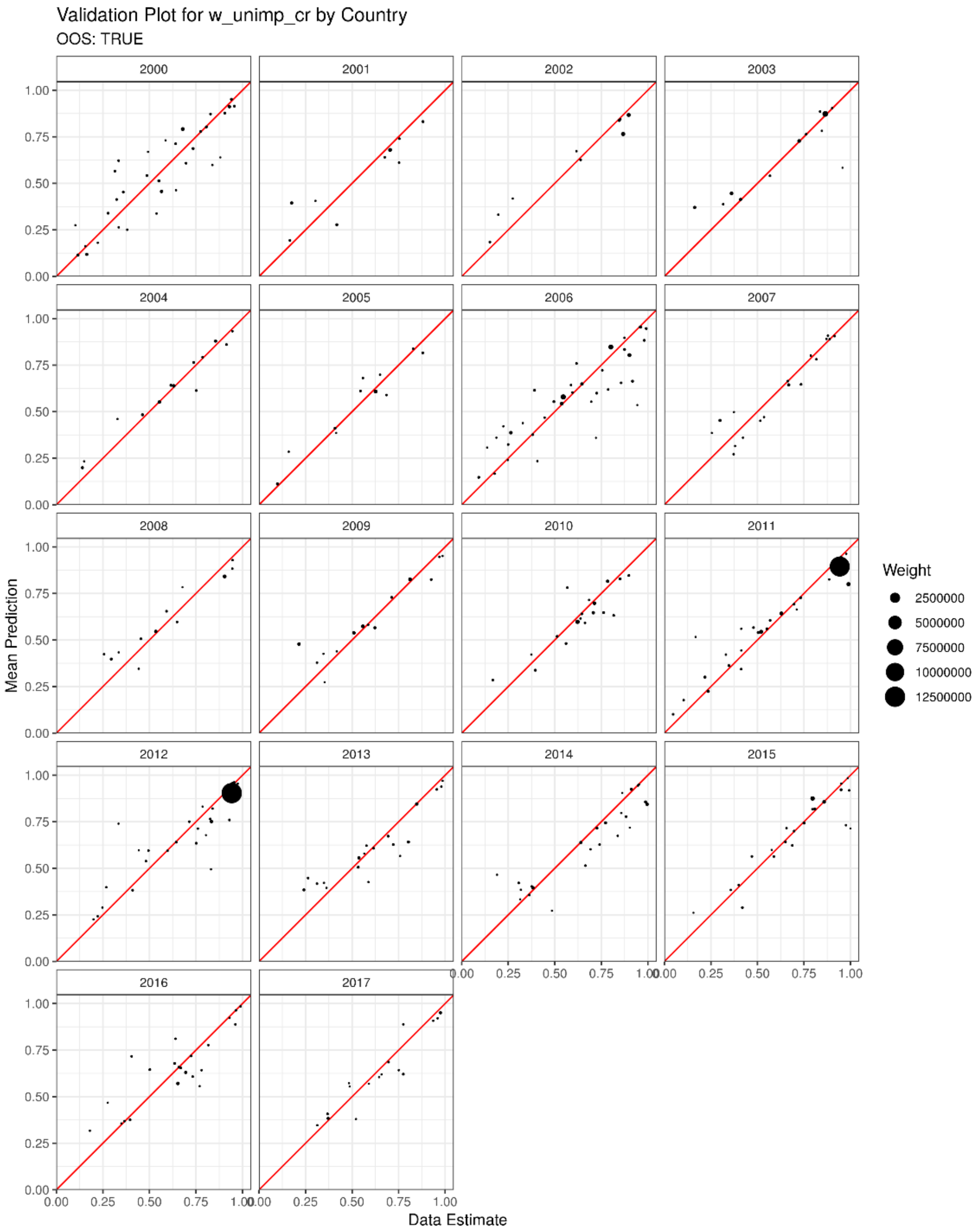

Appendix Figure 46. Out-of-sample for conditional unimproved water model by first administrative level and year

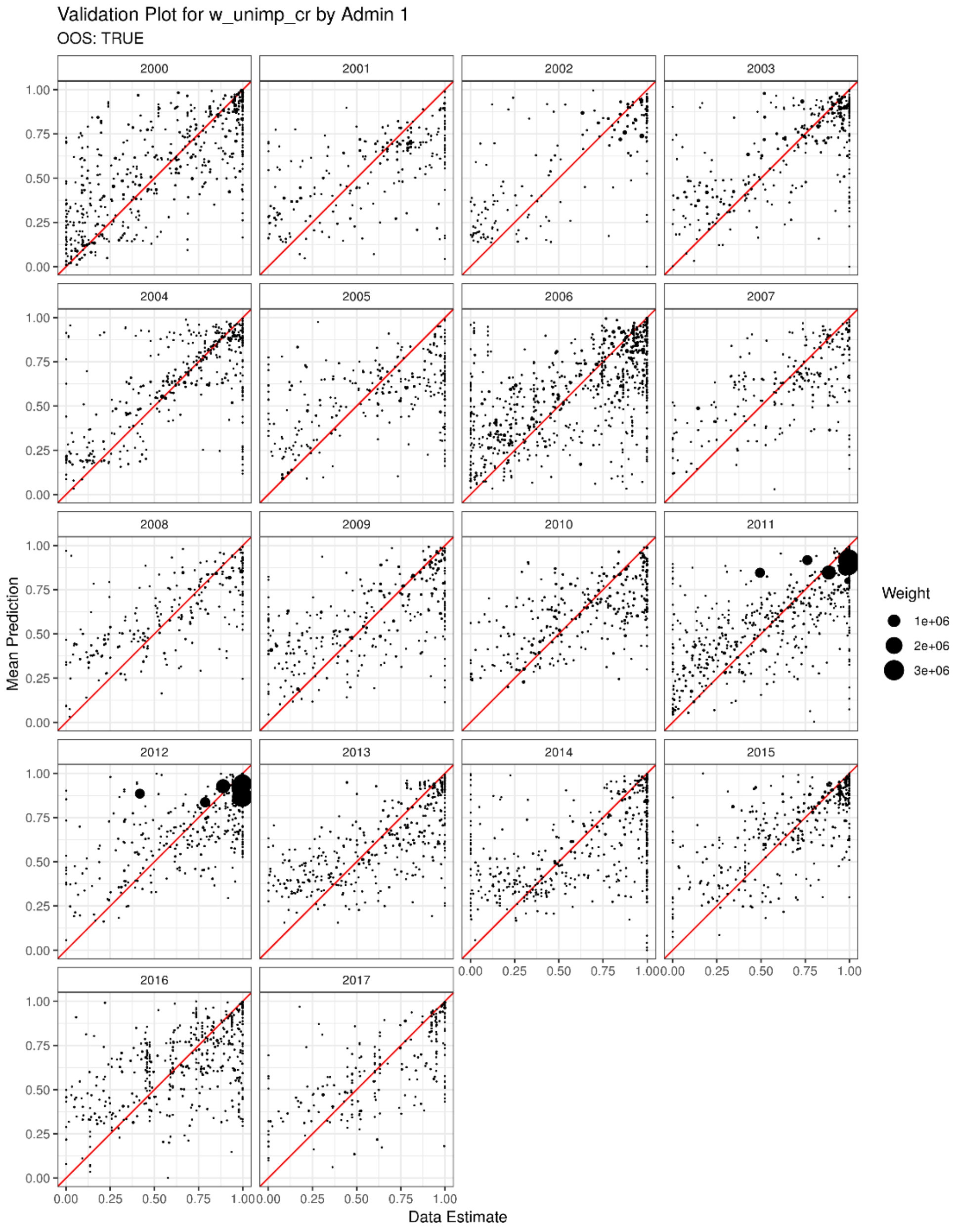

Appendix Figure 47. Out-of-sample for conditional unimproved water model by second administrative level and year

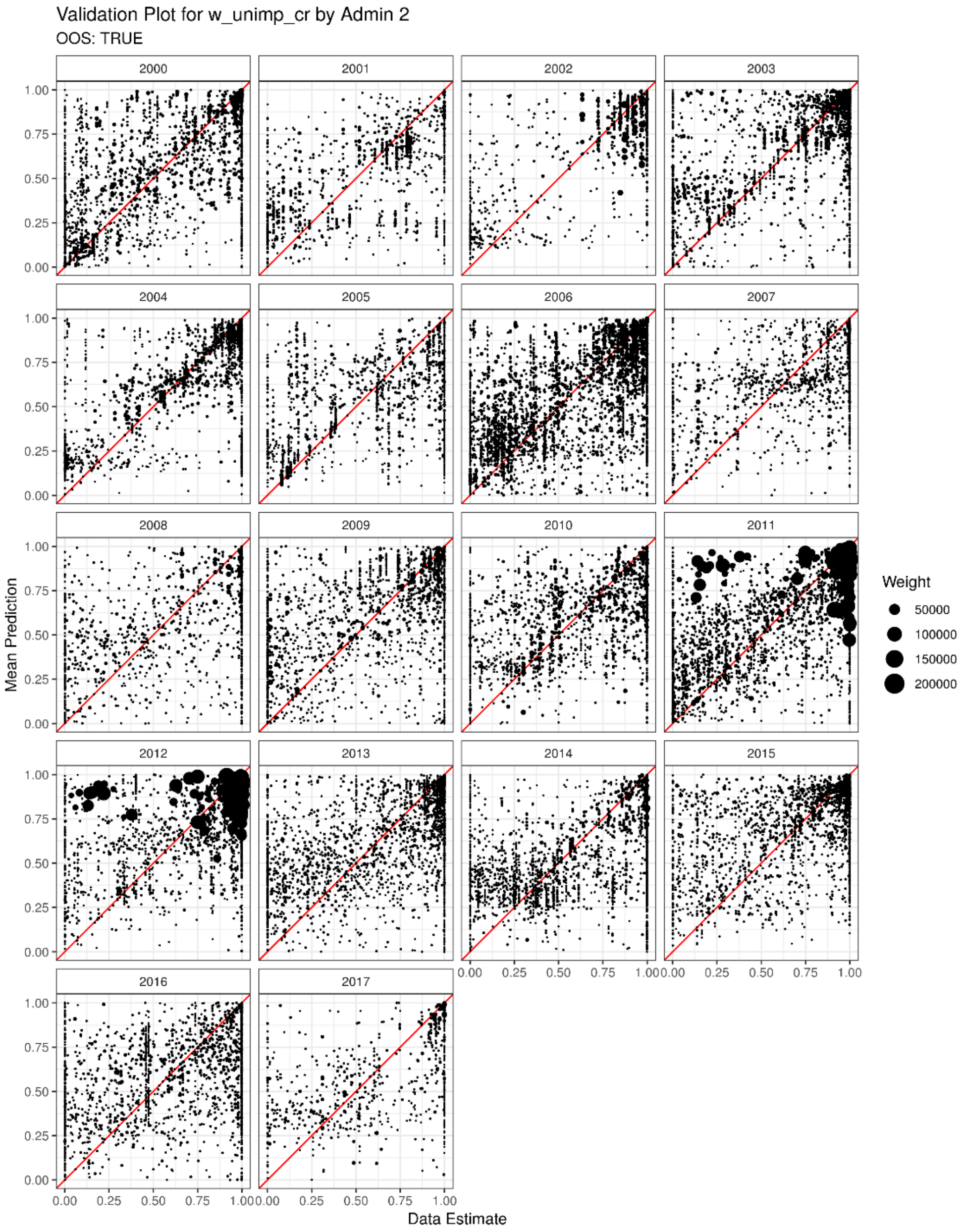

Appendix Figure 48. Out-of-sample for piped sanitation model country and year

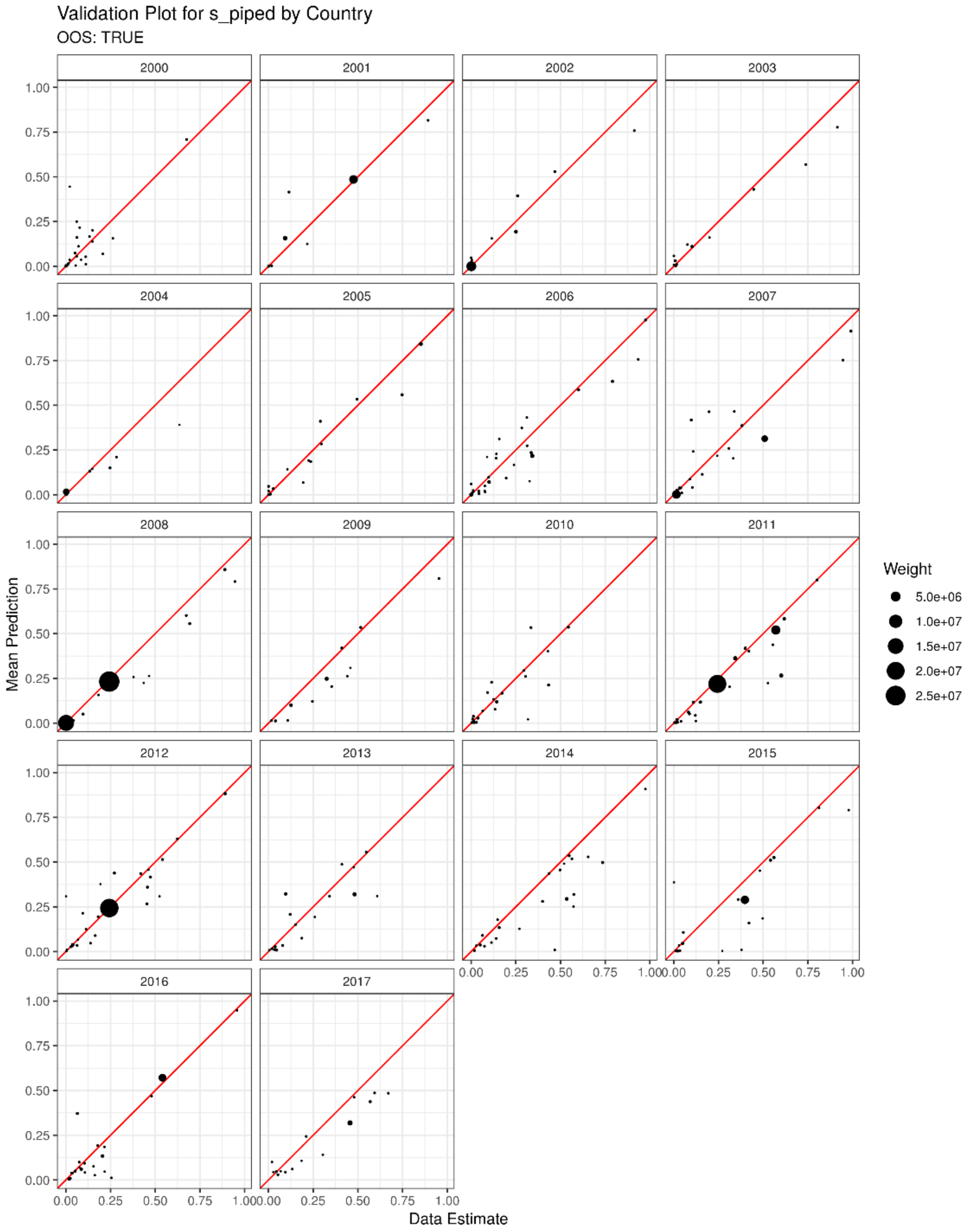

Appendix Figure 49. Out-of-sample for piped sanitation model first administrative level and year

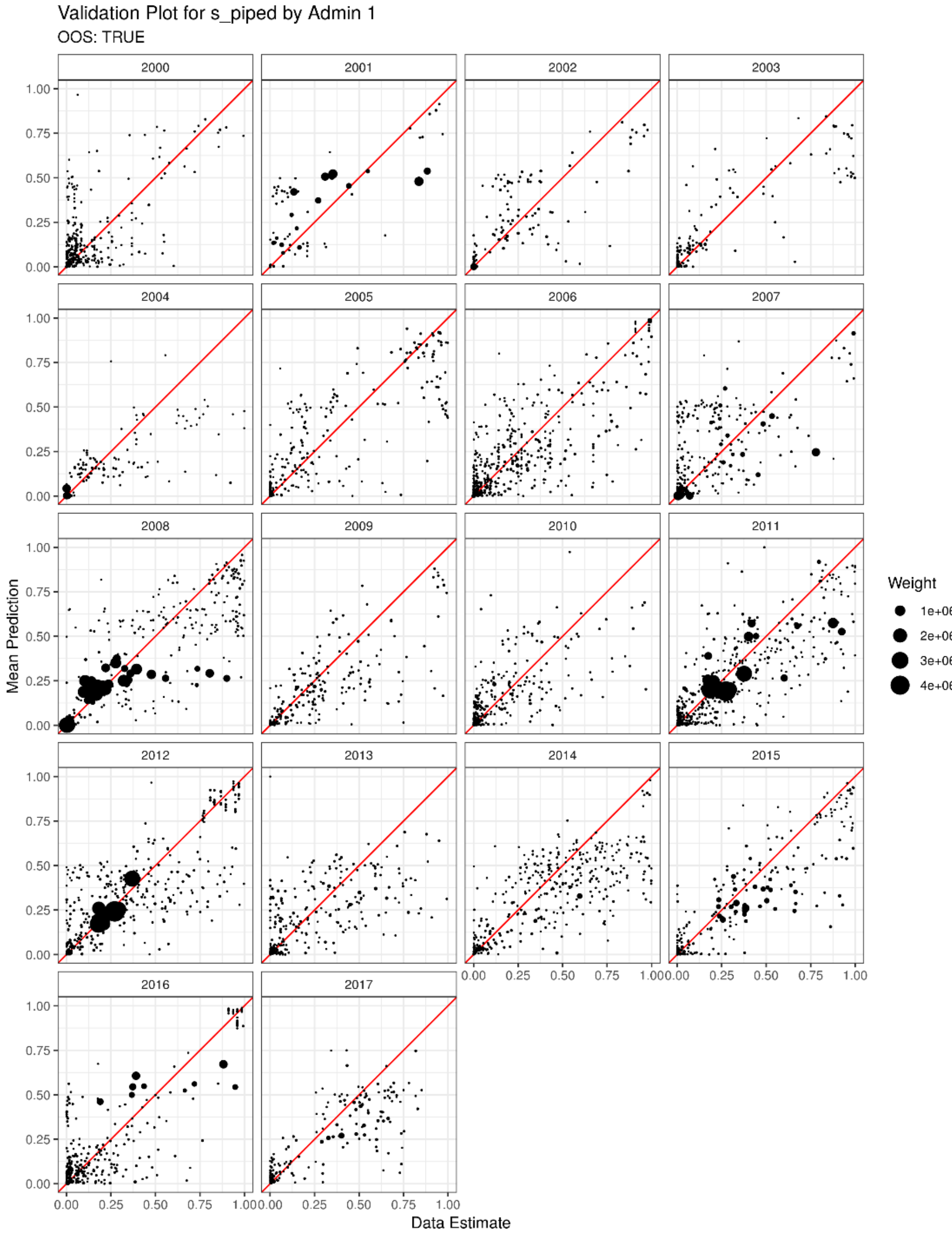

Appendix Figure 50. Out-of-sample for piped sanitation model second administrative level and year

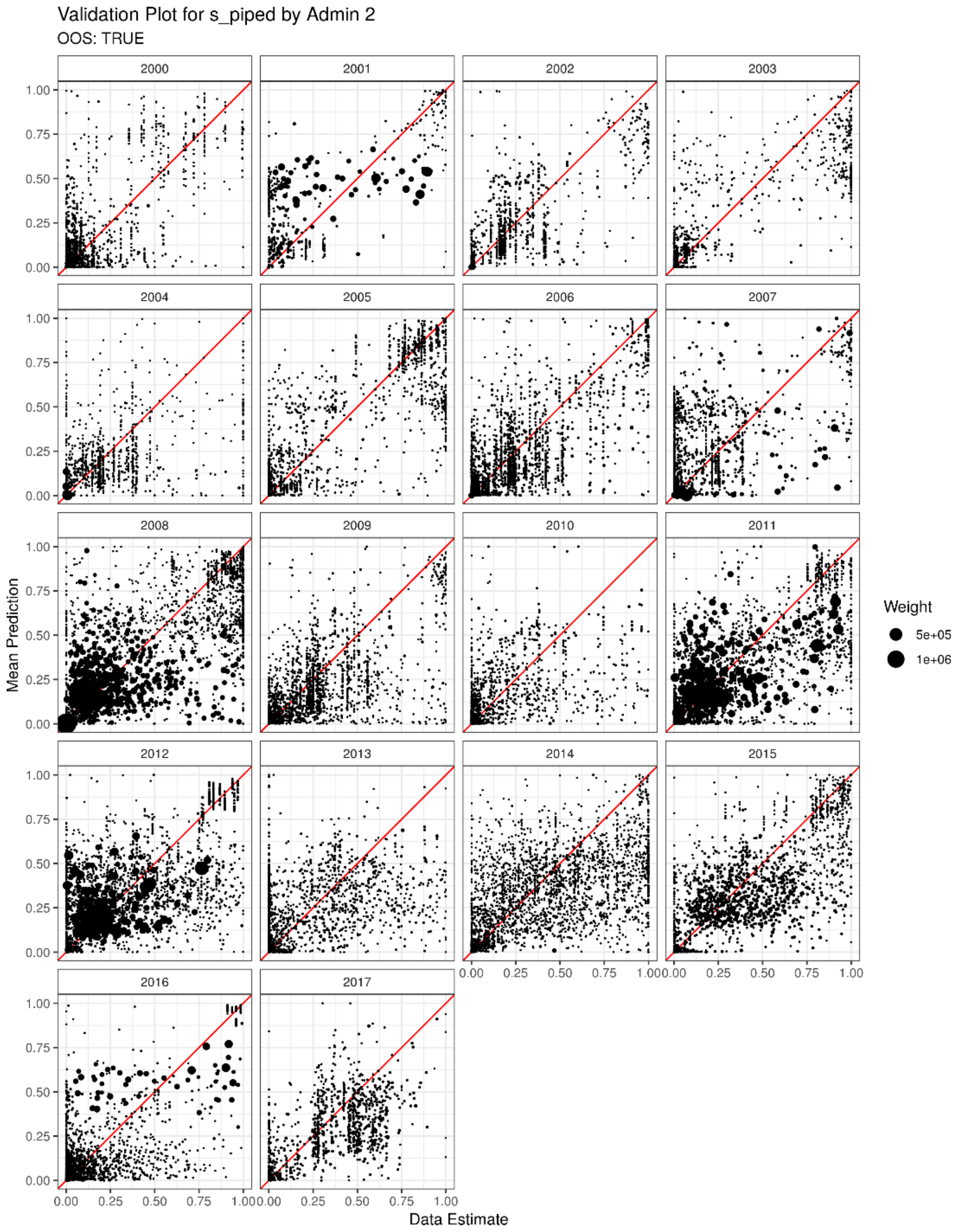

Appendix Figure 51. Out-of-sample statistics for conditional improved sanitation model by country and year

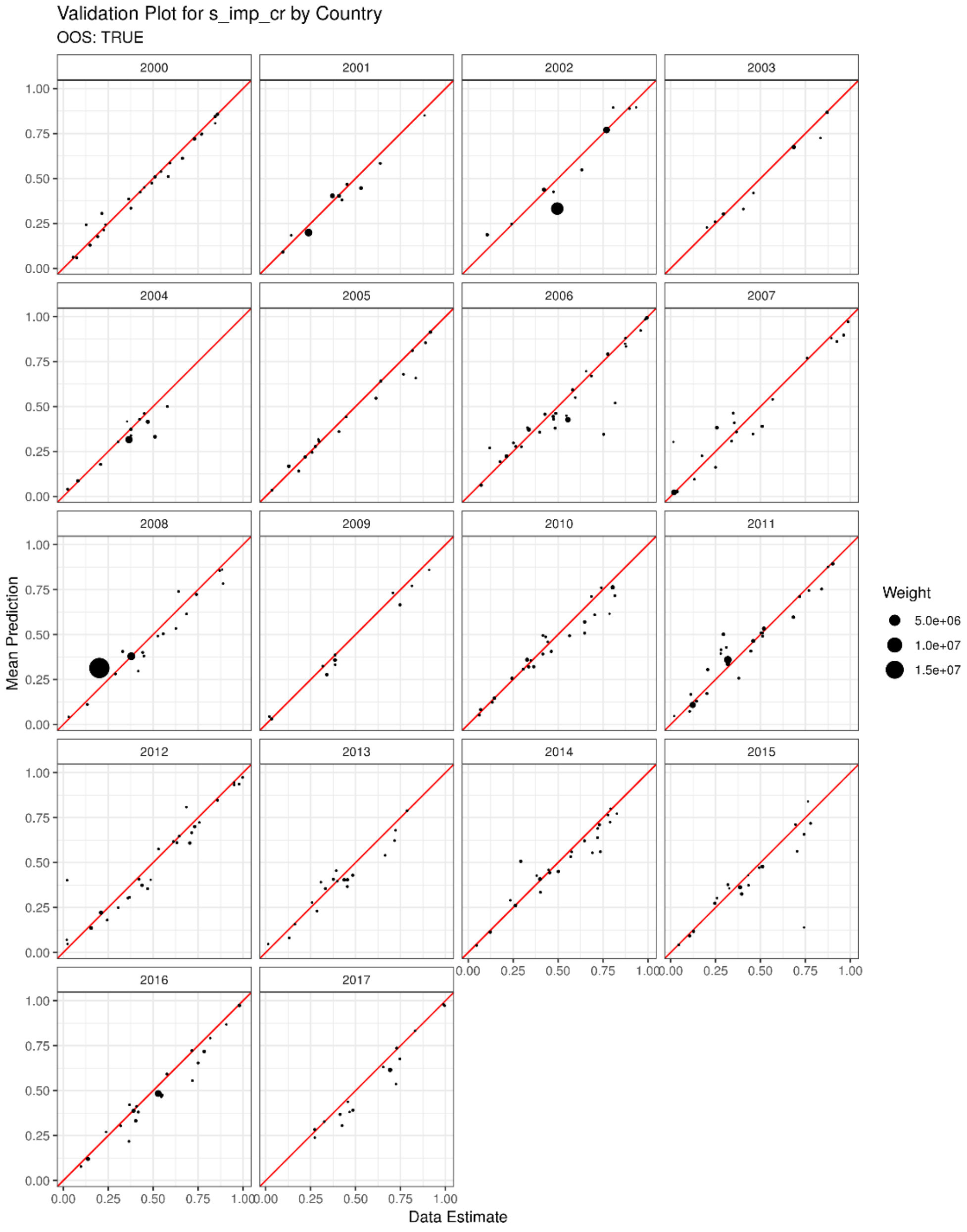

Appendix Figure 52. Out-of-sample statistics for conditional improved sanitation model by first administrative level and year

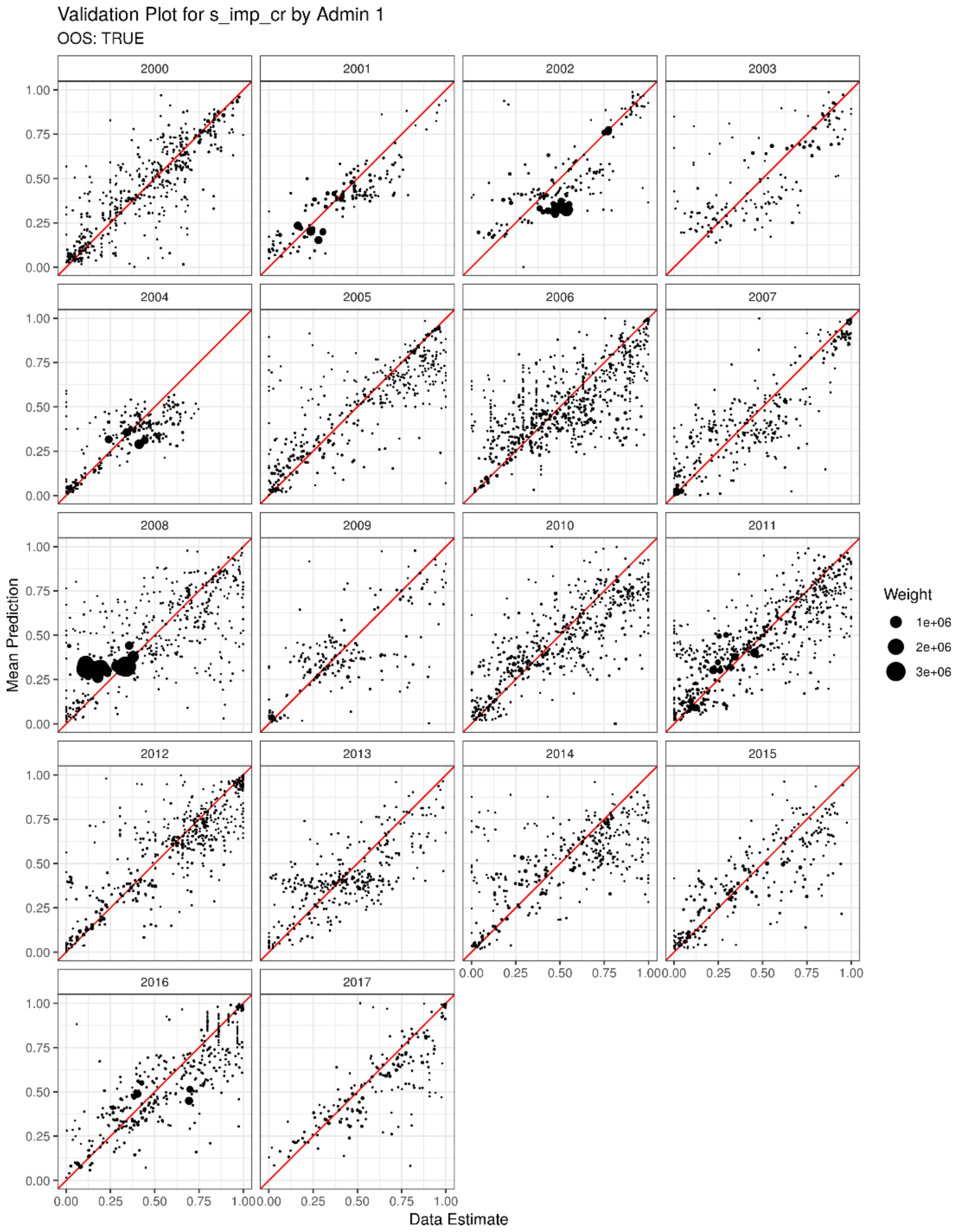

Appendix Figure 53. Out-of-sample statistics for conditional improved sanitation model by second administrative level and year

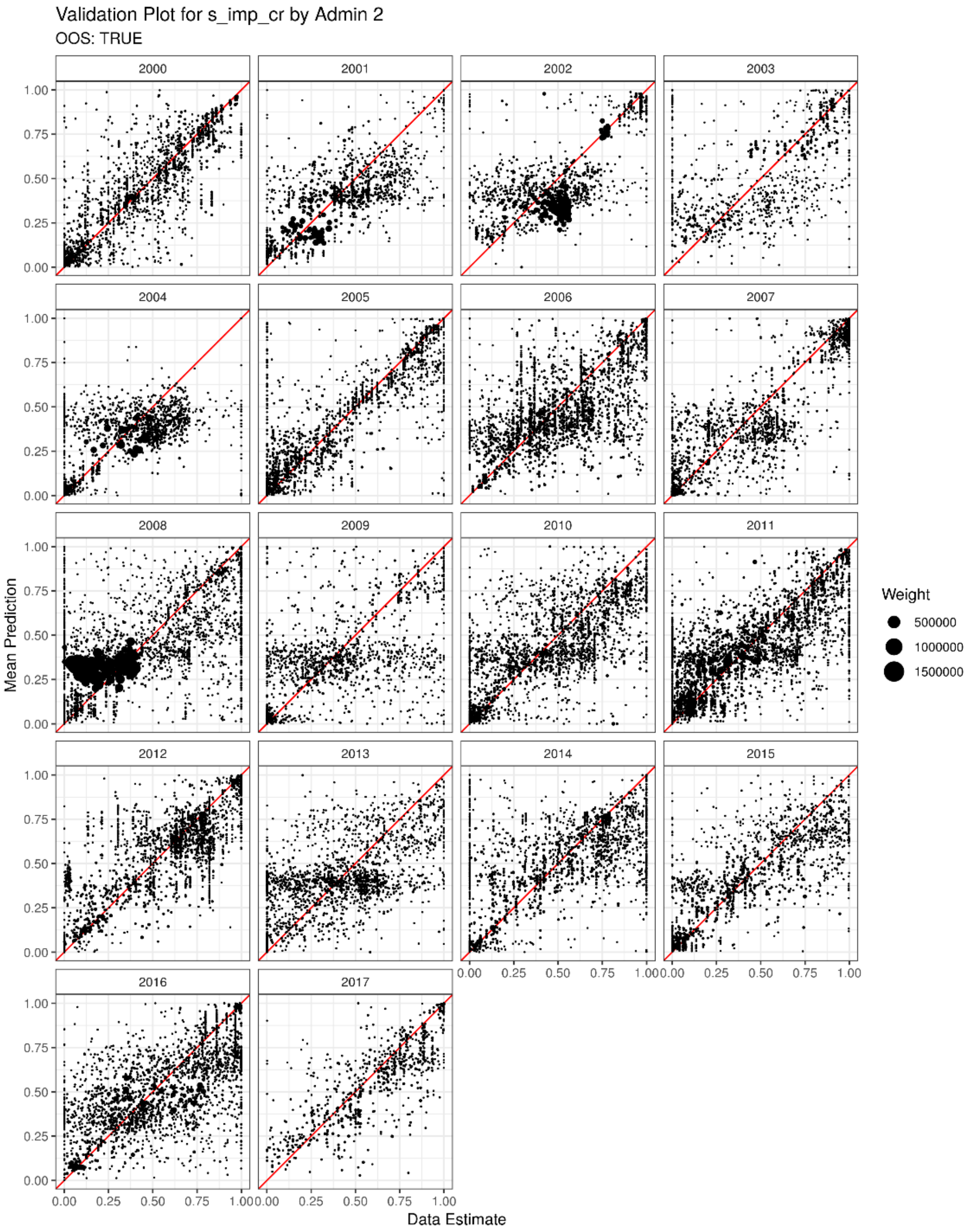

Appendix Figure 54. Out-of-sample statistics for conditional unimproved sanitation model by country and year

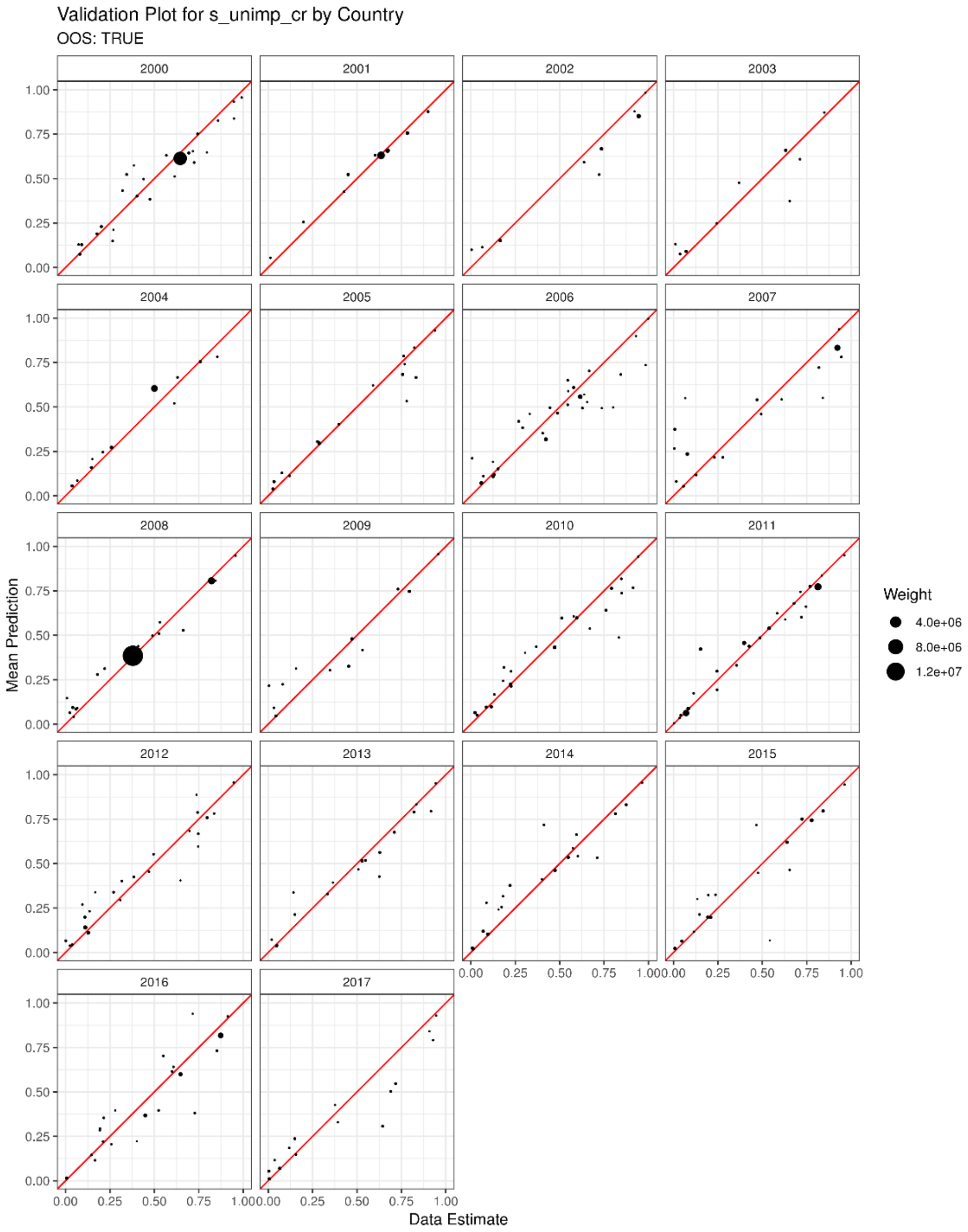

Appendix Figure 55. Out-of-sample statistics for conditional unimproved sanitation model by first administrative level and year

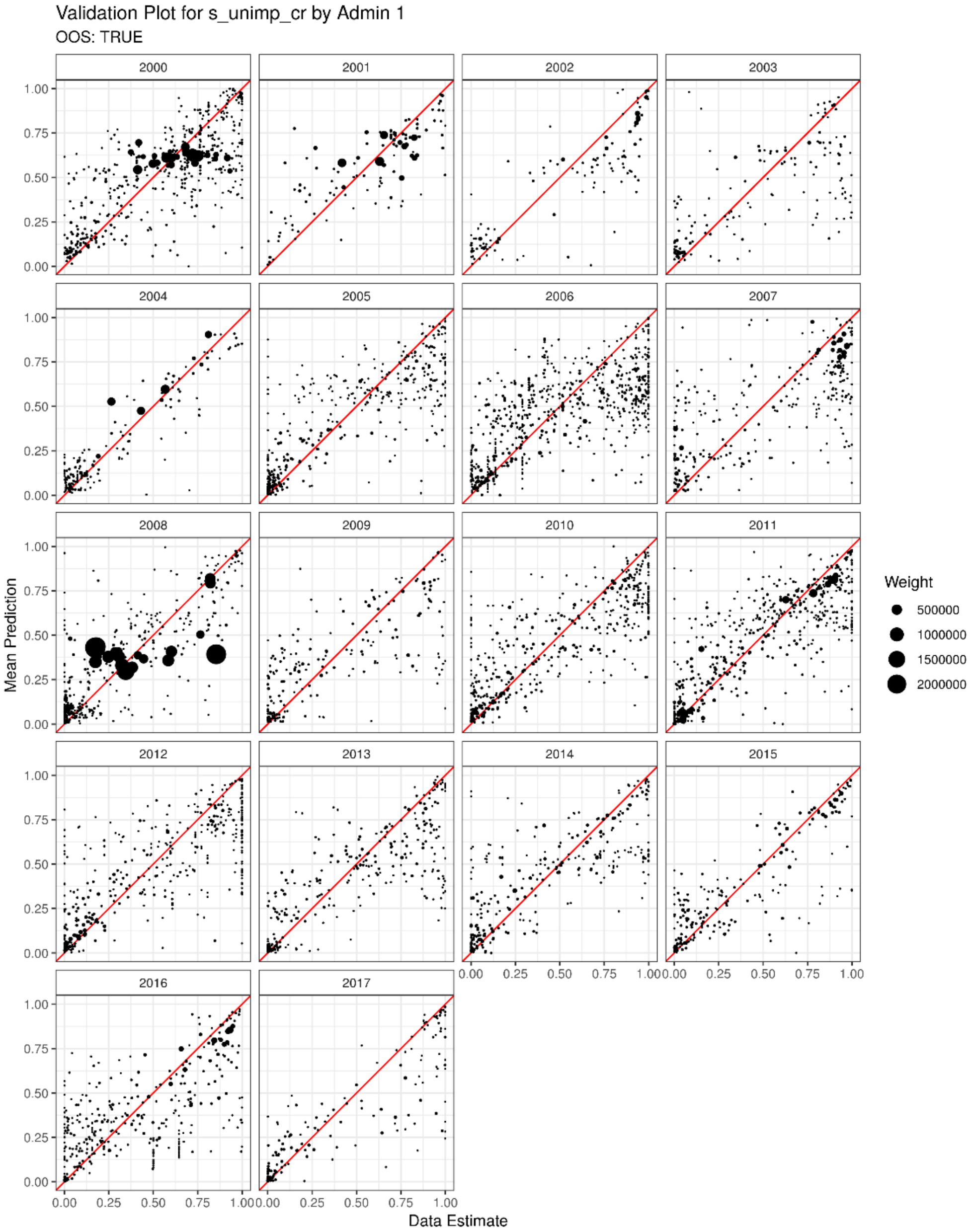

Appendix Figure 56. Out-of-sample statistics for conditional unimproved sanitation model by second administrative level and year

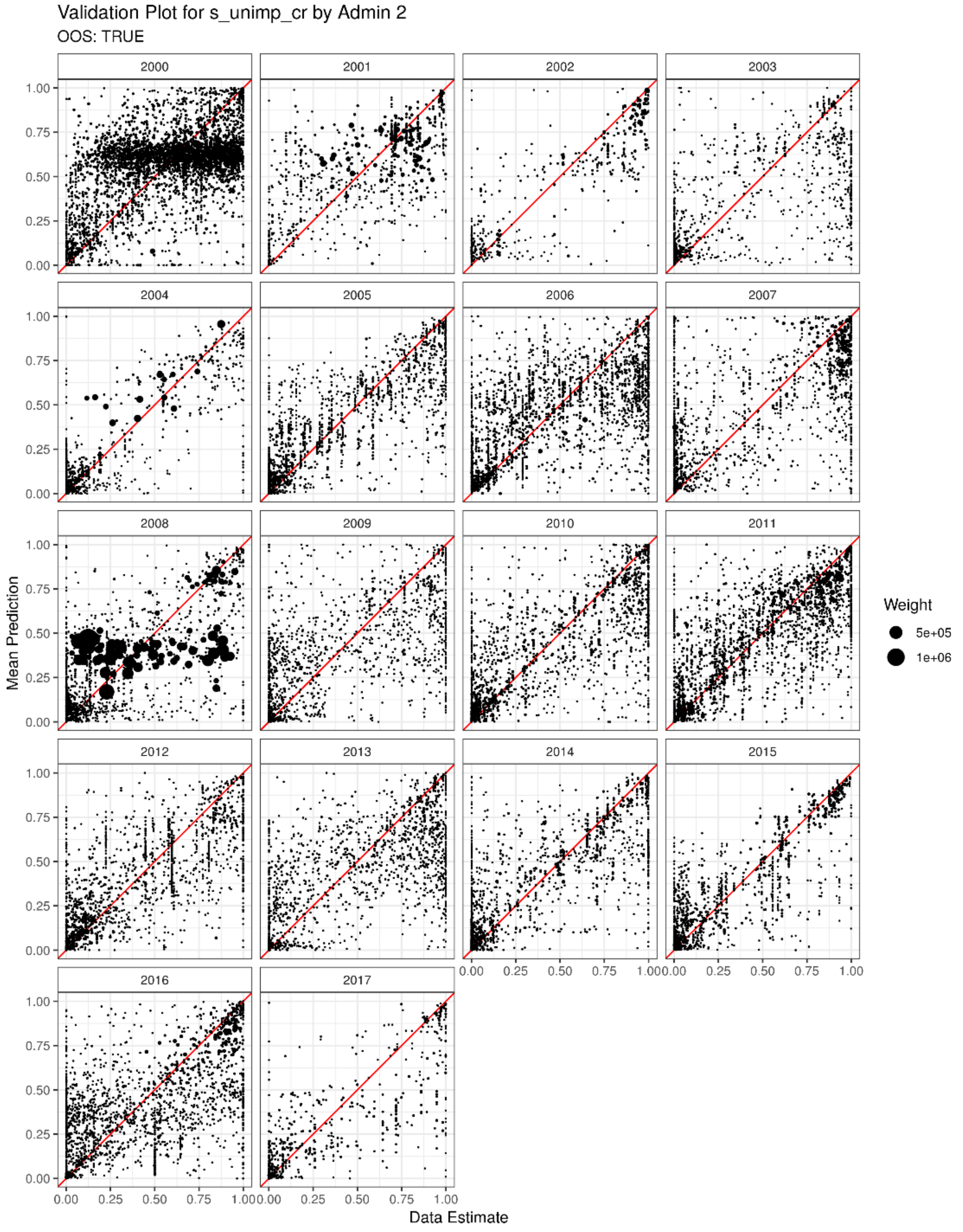

Appendix Figure 57. Correlation between improved water and World Health Organization Joint Monitoring Programme improved water estimates

We plotted the correlation between our country level estimates of access to improved water to the Joint Monitoring Programme's estimates of country level access to improved water. Country level estimates are represented as points and coloured by the GBD region the country is in.

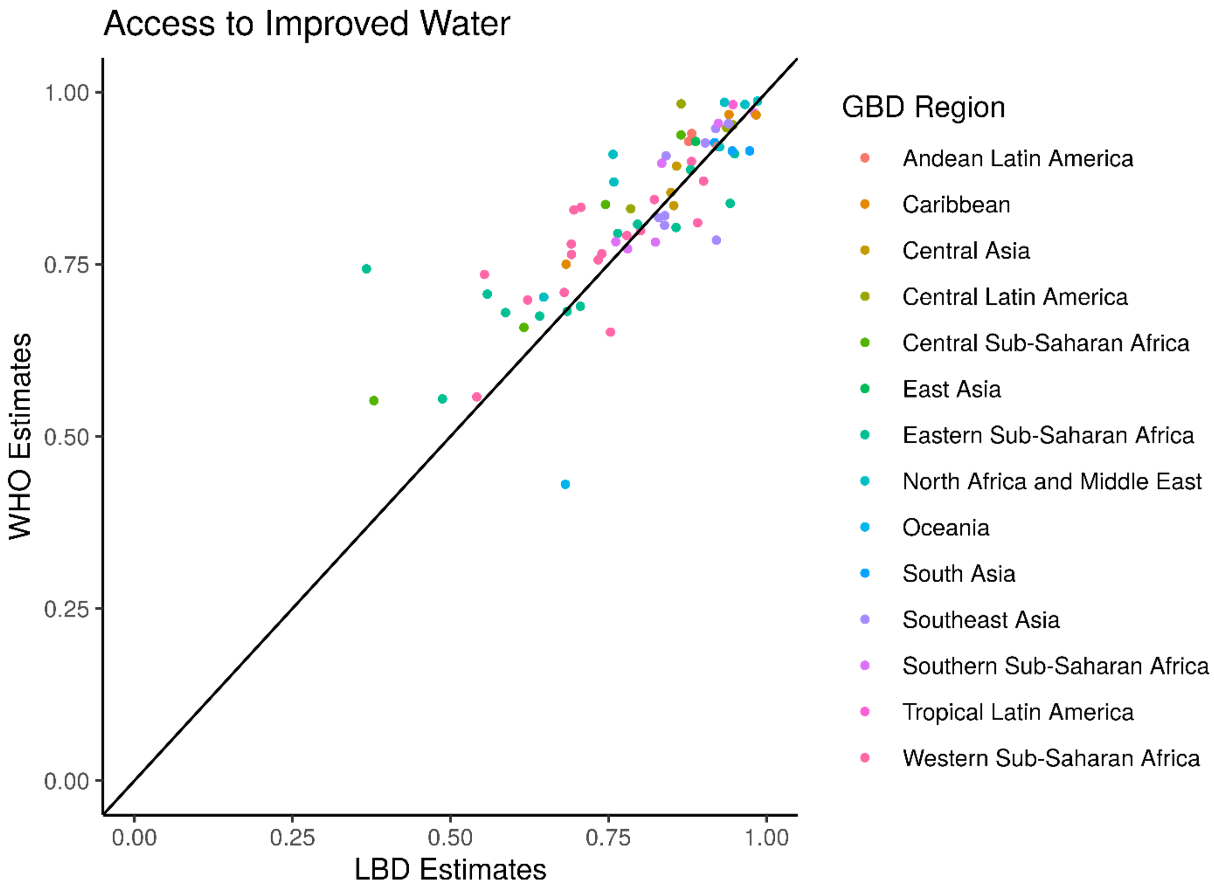

Appendix Figure 58. Correlation between improved sanitation and World Health Organization Joint Monitoring Programme improved sanitation estimates

We plotted the correlation between our country level estimates of access to improved sanitation to the Joint Monitoring Programme's estimates of country level access to improved sanitation. Country level estimates are represented as points and coloured by the GBD region the country is in.

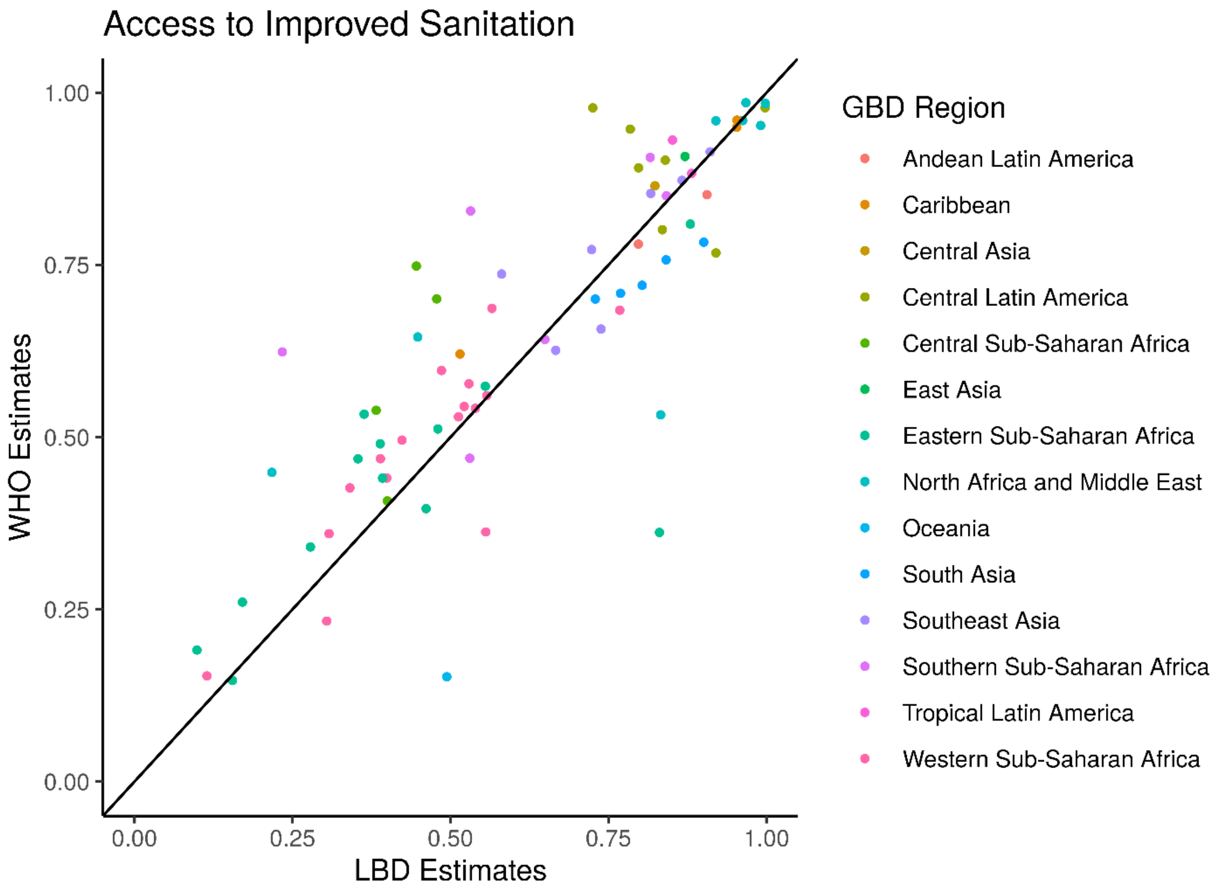

Appendix Figure 59. Correlation between open defecation and World Health Organization Joint Monitoring Programme open defecation estimates

We plotted the correlation between our country level estimates of open defecation to the Joint Monitoring Programme's estimates of country level open defecation. Country level estimates are represented as points and coloured by the GBD region the country is in.

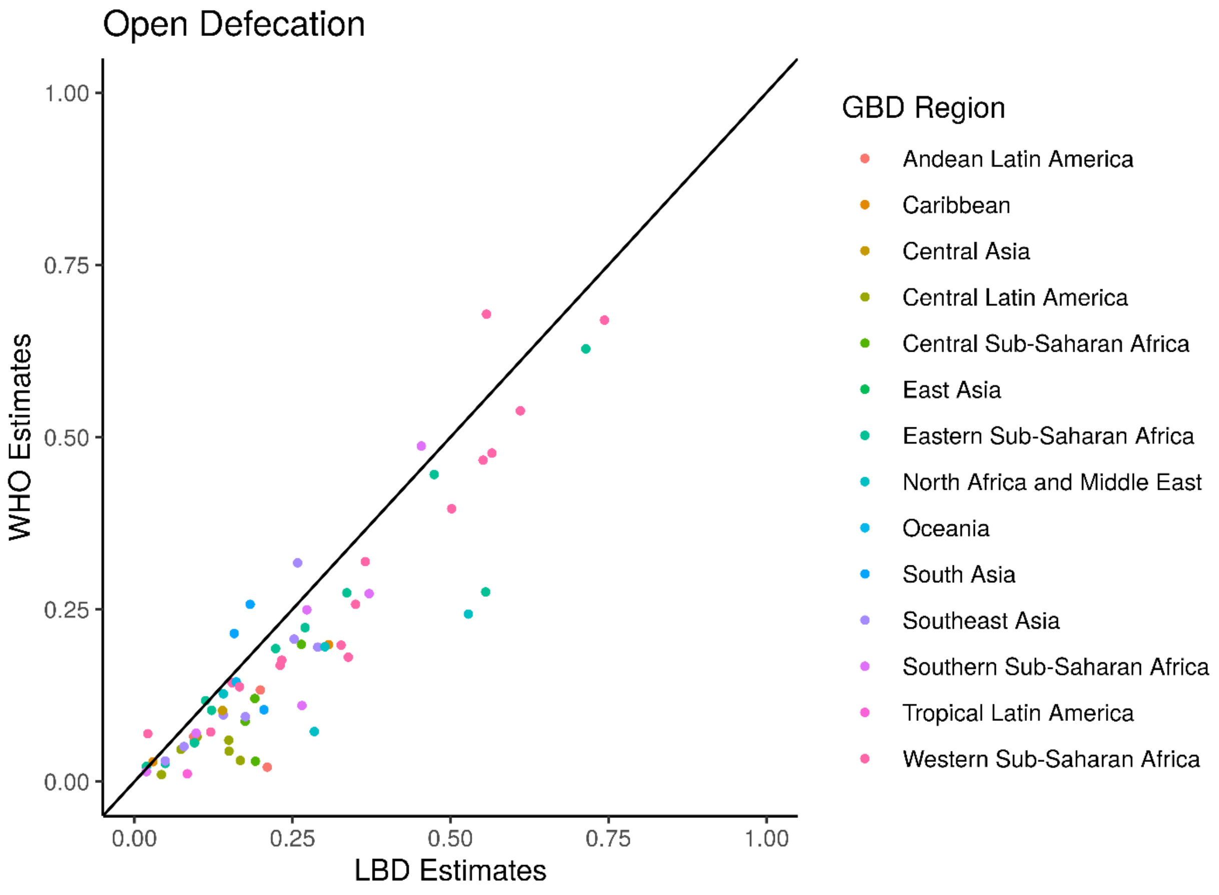

Appendix Figure 60. All indicator data validation plot

This is an example of a country-specific, indicator-specific data quality validation plot for South Africa. Data vetting procedures are fully explained in Section 1.8.2, outlining how surveys were either included or excluded for the final dataset based on these validation plots. Each survey is shown aggregated to the country level by survey year. Each individual survey is represented by a circle, and each level of the indicator is represented by a different colour.

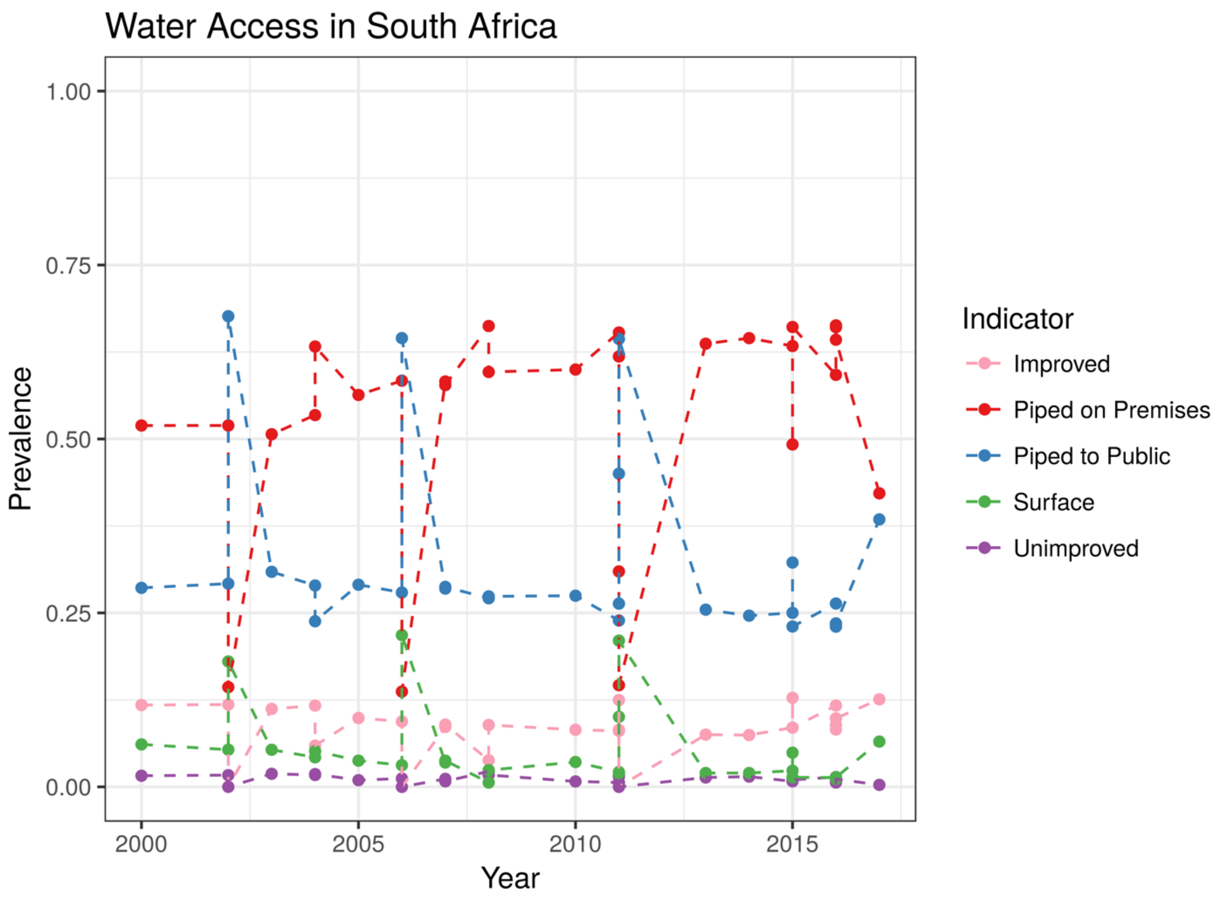

Appendix Figure 61. Indicator-specific data validation plot

This is an example of a country-specific data-quality validation plot for South Africa for access to improved water sources. Each individual survey is represented by a circle or triangle respective of its spatial representativeness. The black line represents the WHO Joint Monitoring Programme trend line. Surveys in blue represent included surveys, while surveys in pink are excluded due to being off trend.

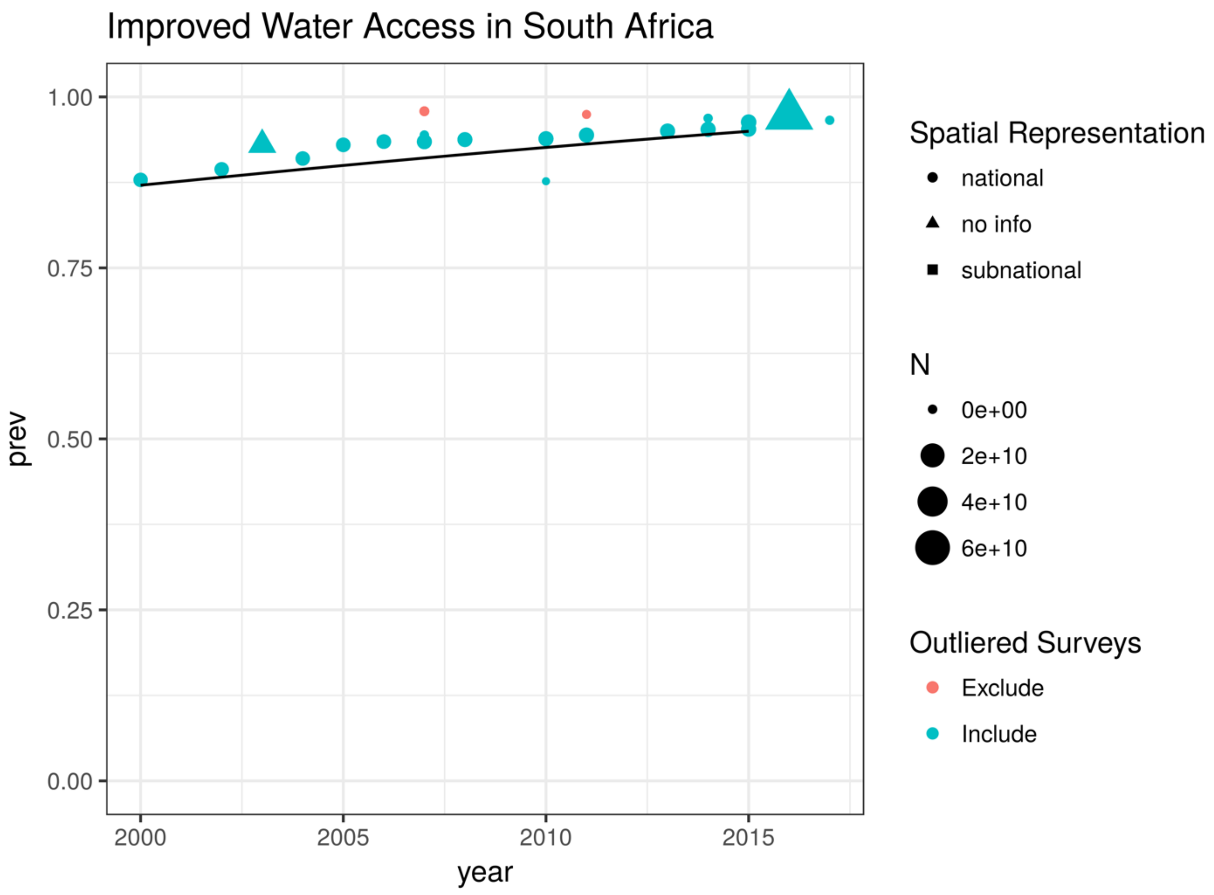

Appendix Figure 62. Rate, ratio, and percentage of deaths averted by access to improved drinking water facility at the second-administrative-unit level

Deaths are calculated under the counterfactual scenario where access to safe water and sanitation remained at the values observed in the year 2000. The deaths caused (purple) and averted (green) per 1,000 population given access levels observed in 2017 is shown for water **(A)**. The ratio of deaths caused (purple) and averted (green) due to changes in access levels compared to 2000 to the change in diarrheal disease deaths overall compared to 2000 is shown for water **(B)**. Lastly, the percent of deaths caused (purple) or averted (green) out of total diarrheal disease deaths in 2017 is mapped for water **(C)**. Maps reflect administrative boundaries, land cover, lakes, and population; grey-coloured grid cells were classified as "barren or sparsely vegetated" and had fewer than ten people per 1 × 1-km grid cell, or were not included in these analyses.[5,11–15]

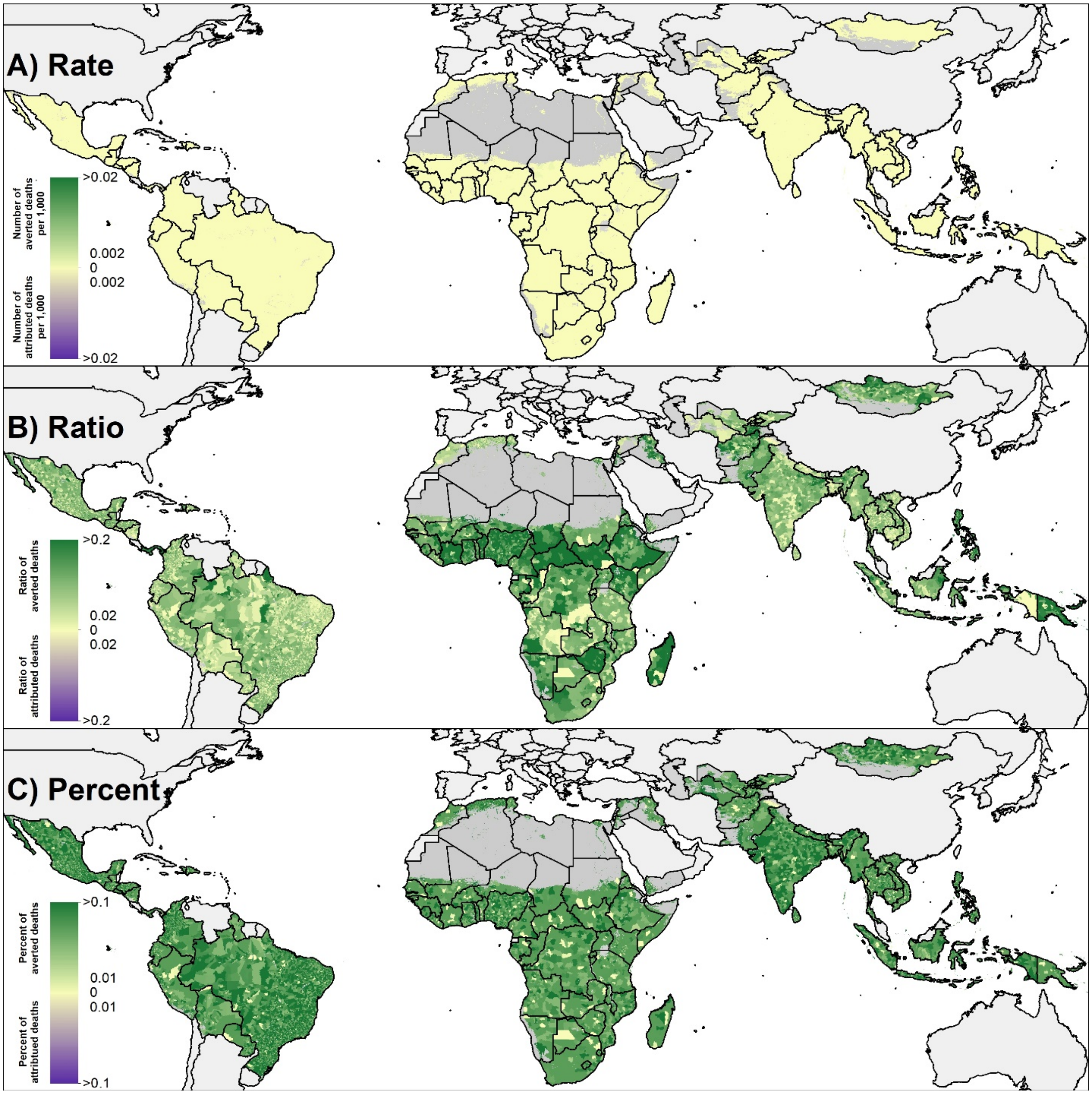

Appendix Figure 63. Rate, ratio, and percentage of deaths averted by access to improved sanitation facility at the second-administrative-unit level

Deaths are calculated under the counterfactual scenario where access to safe water and sanitation remained at the values observed in the year 2000. The deaths caused (purple) and averted (green) per 1,000 population given access levels observed in 2017 is shown for sanitation **(A)**. The ratio of deaths caused (purple) and averted (green) due to changes in access levels compared to 2000 to the change in diarrheal disease deaths overall compared to 2000 is shown for sanitation **(B)**. Lastly, the percent of deaths caused (purple) or averted (green) out of total diarrheal disease deaths in 2017 is mapped for sanitation **(C)**. Maps reflect administrative boundaries, land cover, lakes, and population; grey-coloured grid cells were classified as "barren or sparsely vegetated" and had fewer than ten people per 1 × 1-km grid cell, or were not included in these analyses.[5,11–15]

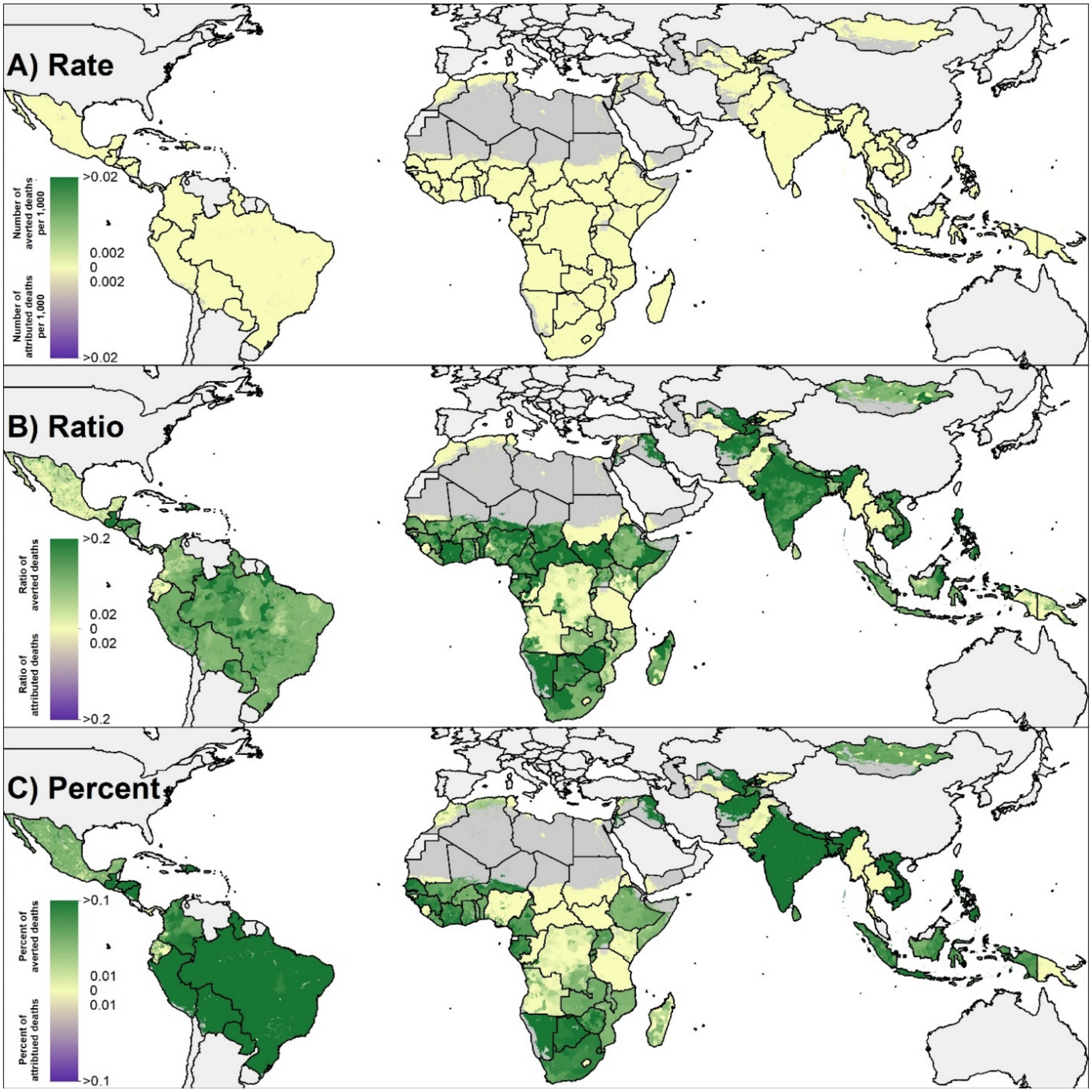
A) Rate
Number of averted deaths per 1,000
Number of attributed deaths per 1,000
>0.02
0.002
0
0.002
>0.02
B) Ratio
Ratio of averted deaths
Ratio of attributed deaths
>0.2
0.02
0
0.02
>0.2
C) Percent
Percent of averted deaths
Percent of attribued deaths
>0.1
0.01
0
0.01
>0.1

6.0 Appendix Tables

Appendix Table 1. Compliance with the Guidelines for Accurate and Transparent Health Estimates Reporting 93
Appendix Table 2. List of countries included in analysis, stratified by Socio-demographic Index .93
Appendix Table 3. Covariates used in modelling framework 95
Appendix Table 4. Covariates used in the water and sanitation generalised additive model (GAM) 96
Appendix Table 5. Covariates used in the boosted regression trees (BRT) model 97
Appendix Table 6. Fixed parameters used for boosted regression trees 98
Appendix Table 7. Fitted parameters by region for model of access to piped water 99
Appendix Table 8. Fitted parameters by region for model of access to improved water 105
Appendix Table 9. Fitted parameters by region for model of access to unimproved water..........112
Appendix Table 10. Fitted parameters by region for model of access to piped sanitation 120
Appendix Table 11. Fitted parameters by region for model of access to improved sanitation 127
Appendix Table 12. Fitted parameters by region for model of access to unimproved sanitation ..134
Appendix Table 13. Predictive in sample metrics aggregated to the country level for water indicators..142
Appendix Table 14. Predictive in sample metrics aggregated to the first administrative level for water indicators 142
Appendix Table 15. Predictive in sample metrics aggregated to the second administrative level for water indicators 143
Appendix Table 16. Predictive in sample metrics aggregated to the country level for sanitation indicators..143
Appendix Table 17. Predictive in sample metrics aggregated to the first administrative level for sanitation indicators 144
Appendix Table 18. Predictive in sample metrics aggregated to the second administrative level for sanitation indicators 144
Appendix Table 19. Predictive out-of-sample metrics aggregated to the country level for water indicators..145
Appendix Table 20. Predictive out-of-sample metrics aggregated to the first administrative level for water indicators..145
Appendix Table 21. Predictive out-of-sample metrics aggregated to the second administrative level for water indicators..146
Appendix Table 22. Predictive out-of-sample metrics aggregated to the country level for sanitation indicators..146
Appendix Table 23. Predictive out-of-sample metrics aggregated to the first administrative level for sanitation indicators..147
Appendix Table 24. Predictive out-of-sample metrics aggregated to the second administrative level for sanitation indicators..147
Appendix Table 25a-b. Risk ratios used in counterfactual analyses..148
Appendix Table 25a. Risk ratios associated with access to water facilities. 148

Appendix Table 25b. Risk ratios associated with access to water facilities................................148

Appendix Table 1. Compliance with the Guidelines for Accurate and Transparent Health Estimates Reporting

Item #	Checklist item	Reported on page #
**Objectives and funding**		
**1**	Define the indicator(s), populations (including age, sex, and geographic entities), and time period(s) for which estimates were made.	Manuscript: Methods section. Appendix: Sections 1.0, 2.1.
**2**	List the funding sources for the work.	Manuscript: Methods section.
**Data Inputs**		
For all data inputs from multiple sources that are synthesised as part of the study:		
**3**	Describe how the data were identified and how the data were accessed.	Manuscript: Methods section. Appendix: Sections 1.0, 2.1.
**4**	Specify the inclusion and exclusion criteria. Identify all ad-hoc exclusions.	Manuscript: Methods section. Appendix: Section 1.0.
**5**	Provide information on all included data sources and their main characteristics. For each data source used, report reference information or contact name/institution, population represented, data collection method, year(s) of data collection, sex and age range, diagnostic criteria or measurement method, and sample size, as relevant.	Manuscript: Methods section. Appendix: Sections 1.0, 2.1; Appendix Figures 4, 5; http://ghdx.healthdata.org/record/ihme-data/lmic-wash-access-geospatial-estimates-2000-2017
**6**	Identify and describe any categories of input data that have potentially important biases (e.g., based on characteristics listed in item 5).	Manuscript: Methods section. Appendix: Section 1.8.
For data inputs that contribute to the analysis but were not synthesised as part of the study:		
**7**	Describe and give sources for any other data inputs.	Appendix: Sections: 1.0, 2.1
For all data inputs:		
**8**	Provide all data inputs in a file format from which data can be efficiently extracted (e.g., a spreadsheet rather than a PDF), including all relevant meta-data listed in item 5. For any data inputs that cannot be shared because of ethical or legal reasons, such as third-party ownership, provide a contact name or the name of the institution that retains the right to the data.	Available at http://ghdx.healthdata.org/record/ihme-data/lmic-wash-access-geospatial-estimates-2000-2017
**Data analysis**		
**9**	Provide a conceptual overview of the data analysis method. A diagram may be helpful.	Manuscript: Methods section. Appendix Figure 1.
**10**	Provide a detailed description of all steps of the analysis, including mathematical formulae. This description should cover, as relevant, data cleaning, data pre-processing, data adjustments and weighting of data sources, and mathematical or statistical model(s).	Manuscript: Methods section. Appendix: Sections 1.4-3.6.
**11**	Describe how candidate models were evaluated and how the final model(s) were selected.	Appendix: Section 3.5.
**12**	Provide the results of an evaluation of model performance, if done, as well as the results of any relevant sensitivity analysis.	Appendix: Section 3.0.
**13**	Describe methods for calculating uncertainty of the estimates. State which sources of uncertainty were, and were not, accounted for in the uncertainty analysis.	Appendix: Section 3.0.
**14**	State how analytic or statistical source code used to generate estimates can be accessed.	Available at https://github.com/ihmeuw/lbd/tree/wash-lmic-2020
**Results and Discussion**		
**15**	Provide published estimates in a file format from which data can be efficiently extracted.	Raster files for spatial data and CSVs of first and second administrative-level estimates to be made available at http://ghdx.healthdata.org/record/ihme-data/lmic-wash-access-geospatial-estimates-2000-2017
**16**	Report a quantitative measure of the uncertainty of the estimates (e.g., uncertainty intervals).	Manuscript: Methods section. Appendix: Section 3.4
**17**	Interpret results in light of existing evidence. If updating a previous set of estimates, describe the reasons for changes in estimates.	Manuscript: Discussion section.
**18**	Discuss limitations of the estimates. Include a discussion of any modelling assumptions or data limitations that affect interpretation of the estimates.	Manuscript: Discussion section.

Appendix Table 2. List of countries included in analysis, stratified by Socio-demographic Index

Low SDI	Low-middle SDI	Middle SDI	High-middle SDI
Afghanistan	Angola	Algeria	China

Bangladesh	Bhutan	Botswana	Libya
Benin	Bolivia	Brazil	
Burkina Faso	Cambodia	Colombia	
Burundi	Cameroon	Costa Rica	
Central African Republic	Dominican Republic	Ecuador	
Chad	Egypt	Gabon	
Comoros	El Salvador	Indonesia	
Côte d’Ivoire	Ghana	Jordan	
Democratic Republic of the Congo	Guatemala	Mexico	
Eritrea	Guyana	Mongolia	
Ethiopia	Honduras	Namibia	
Guinea	India	Panama	
Guinea-Bissau	Iraq	Paraguay	
Haiti	Kenya	Peru	
Liberia	Kyrgyzstan	Philippines	
Madagascar	Laos	South Africa	
Malawi	Lesotho	Sri Lanka	
Mali	Mauritania	Syria	
Mozambique	Morocco	Thailand	
Nepal	Myanmar	Tunisia	
Niger	Nicaragua	Turkmenistan	
Papua New Guinea	Nigeria	Uzbekistan	
Rwanda	Pakistan	Vietnam	
Senegal	Republic of the Congo		
Sierra Leone	Sudan		
Somalia	Swaziland (eSwatini)		
South Sudan	Tajikistan		
Tanzania	Timor-Leste		
The Gambia	Zambia		
Togo	Zimbabwe		
Uganda			
Yemen			

Appendix Table 3. Covariates used in modelling framework

A variety of socioeconomic and environmental variables were used to predict access to water and sanitation facilities. Where available, the finest spatiotemporal resolution of gridded datasets was used.

Covariate	Temporal resolution	Source	Reference
**Travel time to nearest settlement**	Static	Oxford	Weiss, D. J. *et al.* A global map of travel time to cities to assess inequalities in accessibility in 2015. *Nature* **533**, 333-336 (2018).<br><br>Available at: https://map.ox.ac.uk/research-project/accessibility_to_cities/
**Aridity**	Annual	WorldClim (derived)	Zomer, R.J., Trabucco, A., Bossio, D.A. & Verchot, L.V. Climate change mitigation: A spatial analysis of global land suitability for clean development mechanism afforestation and reforestation. *Agriculture Ecosystems & Environment* **126**, 67-80 (2008).<br><br>Global Aridity Index (Global-Aridity) and Global Potential Evapo-Transpiration (Global-PET) Methodology and Geospatial Dataset Description (2009). Available at: http://www.cgiar-csi.org/data/global-aridity-and-pet-database
**Elevation**		NOAA GLOBE	Available at: https://www.ngdc.noaa.gov/mgg/topo/gltiles.html
**Urbanicity**	Annual	European Commission/ GHS	Pesaresi, M. et al. Operating procedure for the production of the Global Human Settlement Layer from Landsat data of the epochs 1975, 1990, 2000, and 2014. (Publications Office of the European Union, 2016).<br><br>Available at: http://ghsl.jrc.ec.europa.eu/data.php
**Growing season Length**	Annual	FAO Global Agro-Ecological Zone project	FAO. GAEZ - Global Agro-Ecological Zones data portal. Available at: http://www.fao.org/nr/gaez/about-data-portal/en/. (Accessed: 25th July 2017) FAO. GAEZ - Global Agro-Ecological Zones users guide. (2012).
**Irrigation**	Static	University of Frankfurt and FAO	Siebert, S., Doll, P., Hoogeveen, J., Faures, J.-M., Frenken, K., & Feick, S. Development and validation of the global map of irrigation areas. *Hydrology and Earth System Sciences* **9**, 535-547 (2005).<br><br>Goethe-Universität. Generation of a digital global map of irrigation areas. Available at: https://www.unifrankfurt.de/45218039/Global_Irrigation_Map. (Accessed: 25th July 2017) Also from: http://www.fao.org/nr/water/aquastat/irrigationmap/index10.stm
**Time**	Annual		

Appendix Table 4. Covariates used in the water and sanitation generalised additive model (GAM)

Covariate	Temporal resolution	Source	Reference
**Travel time to nearest settlement**	Static	Oxford	Weiss, D. J. *et al.* A global map of travel time to cities to assess inequalities in accessibility in 2015. *Nature* **533**, 333-336 (2018).<br><br>Available at: https://map.ox.ac.uk/research-project/accessibility_to_cities/
**Aridity**	Annual	WorldClim (derived)	Zomer, R.J., Trabucco, A., Bossio, D.A. & Verchot, L.V. Climate change mitigation: A spatial analysis of global land suitability for clean development mechanism afforestation and reforestation. *Agriculture Ecosystems & Environment* **126**, 67-80 (2008).<br><br>Global Aridity Index (Global-Aridity) and Global Potential Evapo-Transpiration (Global-PET) Methodology and Geospatial Dataset Description (2009). Available at: http://www.cgiar-csi.org/data/global-aridity-and-pet-database
**Elevation**		NOAA GLOBE	Available at: https://www.ngdc.noaa.gov/mgg/topo/gltiles.html
**Urbanicity**	Annual	European Commission/ GHS	Pesaresi, M. et al. Operating procedure for the production of the Global Human Settlement Layer from Landsat data of the epochs 1975, 1990, 2000, and 2014. (Publications Office of the European Union, 2016).<br><br>Available at: http://ghsl.jrc.ec.europa.eu/data.php
**Growing season Length**	Annual	FAO Global Agro-Ecological Zone project	FAO. GAEZ - Global Agro-Ecological Zones data portal. Available at: http://www.fao.org/nr/gaez/about-data-portal/en/. (Accessed: 25th July 2017) FAO. GAEZ - Global Agro-Ecological Zones users guide. (2012).
**Irrigation**	Static	University of Frankfurt and FAO	Siebert, S., Doll, P., Hoogeveen, J., Faures, J.-M., Frenken, K., & Feick, S. Development and validation of the global map of irrigation areas. *Hydrology and Earth System Sciences* **9**, 535-547 (2005).<br><br>Goethe-Universität. Generation of a digital global map of irrigation areas. Available at: https://www.unifrankfurt.de/45218039/Global_Irrigation_Map. (Accessed: 25th July 2017) Also from: http://www.fao.org/nr/water/aquastat/irrigationmap/index10.stm
**Time**	Annual		

Appendix Table 5. Covariates used in the boosted regression trees (BRT) model

Covariate	Temporal resolution	Source	Reference
**Travel time to nearest settlement**	Static	Oxford	Weiss, D. J. *et al.* A global map of travel time to cities to assess inequalities in accessibility in 2015. *Nature* **533**, 333-336 (2018).<br><br>Available at: https://map.ox.ac.uk/research-project/accessibility_to_cities/
**Aridity**	Annual	WorldClim (derived)	Zomer, R.J., Trabucco, A., Bossio, D.A. & Verchot, L.V. Climate change mitigation: A spatial analysis of global land suitability for clean development mechanism afforestation and reforestation. *Agriculture Ecosystems & Environment* **126**, 67-80 (2008).<br><br>Global Aridity Index (Global-Aridity) and Global Potential Evapo-Transpiration (Global-PET) Methodology and Geospatial Dataset Description (2009). Available at: http://www.cgiar-csi.org/data/global-aridity-and-pet-database
**Elevation**		NOAA GLOBE	Available at: https://www.ngdc.noaa.gov/mgg/topo/gltiles.html
**Urbanicity**	Annual	European Commission/ GHS	Pesaresi, M. et al. Operating procedure for the production of the Global Human Settlement Layer from Landsat data of the epochs 1975, 1990, 2000, and 2014. (Publications Office of the European Union, 2016).<br><br>Available at: http://ghsl.jrc.ec.europa.eu/data.php
**Growing season Length**	Annual	FAO Global Agro-Ecological Zone project	FAO. GAEZ - Global Agro-Ecological Zones data portal. Available at: http://www.fao.org/nr/gaez/about-data-portal/en/. (Accessed: 25th July 2017) FAO. GAEZ - Global Agro-Ecological Zones users guide. (2012).
**Irrigation**	Static	University of Frankfurt and FAO	Siebert, S., Doll, P., Hoogeveen, J., Faures, J.-M., Frenken, K., & Feick, S. Development and validation of the global map of irrigation areas. *Hydrology and Earth System Sciences* **9**, 535-547 (2005).<br><br>Goethe-Universität. Generation of a digital global map of irrigation areas. Available at: https://www.unifrankfurt.de/45218039/Global_Irrigation_Map. (Accessed: 25th July 2017) Also from: http://www.fao.org/nr/water/aquastat/irrigationmap/index10.stm

Appendix Table 6. Fixed parameters used for boosted regression trees

Parameter	Value
**Tree Complexity**	5
**Learning Rate**	0.0025
**Bag Fraction**	0.8
**Number of Trees**	5000

Appendix Table 7. Fitted parameters by region for model of access to piped water

Lower, median, and upper quantiles (0.025%, 0.50%, 0.975%) are displayed for the main parameters by region. The first four columns provide information on the fixed effects: the intercept (int) and the covariates (gam, gbm, and enet) corresponding to the predicted ensemble rasters. Fitted values for the spatiotemporal field are shown in the next two columns. Countries are abbreviated by their ISO3 codes.

	Quantiles	int	gam	gbm	lasso	Nominal range	Nominal variance
AFG	0.025	-2.00920	0.38700	-1.30335	1.52104	-11.19270	7.41130
	0.500	-1.65201	0.46260	-1.16625	1.70369	-10.80060	7.55460
	0.975	-1.29525	0.53847	-1.03064	1.88694	-10.58820	7.81380
AGO	0.025	-2.31970	-1.04784	-0.61196	2.38202	-10.98590	7.56710
	0.500	-2.05344	-0.93997	-0.56169	2.50170	-10.78280	7.64040
	0.975	-1.78752	-0.83240	-0.51166	2.62164	-10.66420	7.76700
BDI	0.025	-0.22378	0.28083	-0.08645	0.71639	-10.12640	4.10550
	0.500	-0.14788	0.31545	-0.06609	0.75062	-10.12380	4.10760
	0.975	-0.07204	0.35010	-0.04574	0.78478	-10.12200	4.11140
BEN	0.025	-1.34013	-0.12225	-0.54270	1.47474	-11.63220	8.29360
	0.500	-1.00351	-0.04671	-0.50717	1.55385	-11.59410	8.35770
	0.975	-0.66724	0.02880	-0.47174	1.63291	-11.55270	8.39530
BFA	0.025	-2.64400	-0.06282	-0.29545	1.15885	-11.45620	8.18160
	0.500	-2.31133	0.01597	-0.26029	1.24433	-11.42840	8.21020
	0.975	-1.97908	0.09477	-0.22539	1.32976	-11.40390	8.22890
BGD	0.025	-5.11749	-0.62437	-1.05224	2.47560	-10.96020	7.87810
	0.500	-4.77035	-0.55464	-1.00265	2.55728	-10.93270	7.90020
	0.975	-4.42394	-0.48491	-0.95327	2.63895	-10.90330	7.93190
BOL	0.025	-0.08403	0.61544	-0.56809	0.75717	-5.59350	4.19830
	0.500	0.16976	0.66723	-0.50937	0.84218	-5.44210	4.43570
	0.975	0.42341	0.71900	-0.45073	0.92710	-5.28760	4.66640
BRA	0.025	-0.01632	-0.26359	0.00188	1.02069	-3.75920	2.16430
	0.500	0.21135	-0.17965	0.07357	1.10584	-3.63840	2.61490
	0.975	0.43896	-0.09569	0.14528	1.19099	-3.50870	2.97090
BWA	0.025	-0.50114	0.29119	-0.77821	1.12857	-9.11340	7.59200
	0.500	-0.41651	0.38356	-0.65352	1.26973	-8.76240	7.77790
	0.975	-0.33176	0.47587	-0.52739	1.40967	-8.51000	8.03350
CAF	0.025	-0.79035	0.00726	0.54709	0.09275	-9.44780	7.28190
	0.500	-0.61096	0.07609	0.68290	0.24076	-9.14300	7.45600
	0.975	-0.43213	0.14502	0.81860	0.38873	-8.92810	7.70320
CHN	0.025	-0.28118	0.43336	-0.00762	0.20236	-4.37730	2.47520
	0.500	0.14325	0.57077	0.07372	0.35503	-4.28540	2.93900
	0.975	0.56787	0.70818	0.15631	0.50712	-4.17030	3.28040
CIV	0.025	-0.60695	1.40541	-0.10850	- 0.63655	-10.73690	8.07060
	0.500	-0.39220	1.54262	-0.05444	- 0.48794	-10.70200	8.10670

	Quantiles	int	gam	gbm	lasso	Nominal range	Nominal variance
	0.975	-0.17764	1.68030	-0.00093	-0.33997	-10.66820	8.13240
CMR	0.025	-2.03186	0.54373	-0.61186	0.83086	-11.23260	7.59520
	0.500	-1.68969	0.63395	-0.55829	0.92436	-10.90830	7.71500
	0.975	-1.34801	0.72427	-0.50490	1.01773	-10.71520	7.91690
COD	0.025	-3.22516	-0.20822	-0.23982	1.13440	-10.81050	7.48080
	0.500	-2.89694	-0.06384	-0.21159	1.27549	-10.78350	7.50300
	0.975	-2.56918	0.08083	-0.18360	1.41616	-10.74670	7.53010
COG	0.025	-1.20555	0.18471	-0.48942	0.91682	-9.52570	7.53980
	0.500	-1.02559	0.33140	-0.42493	1.09367	-9.46530	7.68750
	0.975	-0.84583	0.47800	-0.36067	1.27034	-9.40630	7.76280
COL	0.025	-1.69581	-0.44685	-0.60497	1.71751	-9.41160	3.22970
	0.500	0.10214	-0.31678	-0.55118	1.86798	-9.41080	3.23020
	0.975	1.89864	-0.18702	-0.49747	2.01853	-9.41000	3.23080
COM	0.025	-2.03221	-0.56699	0.05349	1.24608	-9.80500	3.97480
	0.500	-0.07492	-0.45770	0.11620	1.34139	-9.80110	3.99740
	0.975	1.88078	-0.34921	0.17914	1.43720	-9.79940	4.01960
DOM	0.025	-1.07963	0.28036	-0.61081	0.56686	-12.11440	7.34480
	0.500	0.24010	0.46733	-0.34919	0.88223	-11.29180	7.81240
	0.975	1.55878	0.65430	-0.08823	1.19722	-10.66580	8.42590
DZA	0.025	-0.21373	-0.02815	0.19319	0.32973	-4.70300	4.19010
	0.500	-0.10650	0.16362	0.31671	0.51960	-4.52650	4.47430
	0.975	0.00067	0.35529	0.44021	0.70921	-4.34780	4.75300
ECU	0.025	-0.02468	0.21648	-0.27792	0.86881	-9.06210	4.32620
	0.500	0.10066	0.28695	-0.22750	0.94056	-9.05530	4.33060
	0.975	0.22591	0.35738	-0.17713	1.01224	-9.04900	4.33510
EGY	0.025	4.28784	1.10476	-0.44248	0.12339	-9.81920	6.81720
	0.500	4.95806	1.17833	-0.39274	0.21439	-9.79820	6.82120
	0.975	5.64450	1.25227	-0.34325	0.30519	-9.75260	6.82290
ERI	0.025	-0.16275	0.16836	-0.17618	-0.18379	-7.19330	5.53350
	0.500	0.00884	0.49230	0.23120	0.27657	-6.55560	6.02930
	0.975	0.18029	0.81597	0.63825	0.73655	-6.11030	6.74020
ETH	0.025	-0.96320	0.33450	0.16830	0.27093	-11.37480	8.04430
	0.500	-0.79421	0.42978	0.19639	0.37380	-11.35560	8.06360
	0.975	-0.62538	0.52519	0.22453	0.47633	-11.33760	8.07700
GAB	0.025	-2.20547	-0.19036	-1.01517	1.64471	-11.42210	7.96670
	0.500	-1.51240	0.03180	-0.89918	1.86775	-11.35950	8.02360
	0.975	-0.82016	0.25513	-0.78686	2.09143	-11.26820	8.08070
GHA	0.025	-12.84028	0.52023	-0.54230	0.85849	-9.83530	2.81520
	0.500	-10.95745	0.58077	-0.50382	0.92304	-9.83480	2.81530

	Quantiles	int	gam	gbm	lasso	Nominal range	Nominal variance
	0.975	-9.07661	0.64142	-0.46541	0.98747	-9.83350	2.82350
GIN	0.025	-4.02633	-1.92822	-0.40837	2.86940	-10.92370	7.78420
	0.500	-3.56715	-1.73970	-0.32538	3.06518	-10.84390	7.84200
	0.975	-3.10898	-1.55590	-0.24109	3.26356	-10.77810	7.88970
GMB	0.025	-1.92727	-0.41921	-1.58907	2.42476	-15.48670	-2.42890
	0.500	0.03493	-0.24395	-1.45192	2.69533	-14.61770	3.08460
	0.975	1.99555	-0.07325	-1.32346	2.98069	-14.02530	6.91290
GNB	0.025	-2.23422	-0.25348	-1.44962	1.23168	-12.67160	6.98170
	0.500	-0.32982	-0.02436	-0.92131	1.94558	-11.93170	7.51190
	0.975	1.57304	0.20457	-0.39346	2.65894	-11.31020	8.14650
GTM	0.025	0.03974	0.69502	-0.97023	0.88715	-10.96850	7.59080
	0.500	0.39288	0.84583	-0.89553	1.04974	-10.47900	7.81450
	0.975	0.74575	0.99669	-0.82140	1.21224	-10.17340	8.17410
GUY	0.025	-1.97200	0.03826	-0.57913	1.22864	-10.53910	7.74520
	0.500	-1.51406	0.14717	-0.51611	1.36887	-10.45760	7.81210
	0.975	-1.05693	0.25608	-0.45336	1.50909	-10.32350	7.89160
HND	0.025	0.03696	1.34433	-2.06660	1.18054	-11.22140	7.77070
	0.500	0.41971	1.54318	-1.92063	1.37731	-10.88830	7.92720
	0.975	0.80218	1.74298	-1.77544	1.57382	-10.66690	8.15910
HTI	0.025	-2.01209	0.97197	-1.35684	1.20848	-10.31220	-5.96520
	0.500	-0.06501	1.03247	-1.31785	1.28542	-10.30960	-5.96400
	0.975	1.88050	1.09304	-1.27902	1.36226	-10.30770	-5.96280
IDN	0.025	-3.38273	1.03672	-0.53203	0.46755	-9.19180	-71.68110
	0.500	-2.06808	1.04487	-0.52393	0.47906	-9.18920	-71.66570
	0.975	-0.75454	1.05301	-0.51584	0.49057	-9.18780	-71.65800
IND	0.025	-0.07140	0.22209	0.18782	0.54568	-8.96190	4.16090
	0.500	-0.05856	0.23737	0.19939	0.56322	-8.96140	4.16150
	0.975	-0.04574	0.25269	0.21095	0.58074	-8.96080	4.16200
IRQ	0.025	-0.30503	0.54912	-2.27170	2.30813	-9.48200	6.76900
	0.500	-0.00671	0.64609	-2.13882	2.49259	-9.29110	6.96840
	0.975	0.29139	0.74317	-2.00668	2.67743	-9.09580	7.16210
JOR	0.025	0.04973	0.96933	-0.11982	0.05611	-10.21970	7.74480
	0.500	0.38940	0.99780	-0.09719	0.09939	-10.14890	7.80420
	0.975	0.72885	1.02628	-0.07460	0.14263	-10.08450	7.88330
KEN	0.025	-3.15953	0.39673	-0.13993	0.59341	-11.60630	7.89680
	0.500	-2.74396	0.45480	-0.10780	0.65298	-11.60230	7.90080
	0.975	-2.32896	0.51288	-0.07570	0.71250	-11.59810	7.90490
KGZ	0.025	-3.53761	2.10676	-7.92214	6.30669	-8.87870	3.66260
	0.500	-1.64123	2.35470	-7.83627	6.54226	-8.87610	3.66850
	0.975	0.25363	2.60361	-7.78867	6.78014	-8.87170	3.67390
KHM	0.025	-4.18439	1.70215	-0.25728	-0.65873	-11.59170	8.25400

	Quantiles	int	gam	gbm	lasso	Nominal range	Nominal variance
	0.500	-3.76440	1.78701	-0.22503	-0.56193	-11.58560	8.26050
	0.975	-3.34627	1.87217	-0.19296	-0.46549	-11.57750	8.26980
LAO	0.025	-2.66645	0.47019	-1.70594	1.12638	-10.80310	7.59780
	0.500	-2.08110	0.92091	-1.55575	1.63493	-10.75240	7.64570
	0.975	-1.49638	1.37236	-1.40239	2.13842	-10.68110	7.69540
LBR	0.025	-4.45997	-0.03716	-0.09862	1.02598	-10.76200	7.76480
	0.500	-4.08224	-0.01177	-0.05986	1.07151	-10.47170	7.92660
	0.975	-3.70868	0.01383	-0.02124	1.11710	-10.23350	8.12450
LBY	0.025	0.43370	-0.09535	-0.02947	0.84204	-6.10690	4.09880
	0.500	0.96638	0.00697	0.04061	0.95216	-5.99030	4.35470
	0.975	1.49874	0.10911	0.11117	1.06222	-5.87300	4.60740
LKA	0.025	-1.18992	-0.00034	-0.82722	1.48418	-9.87130	6.89960
	0.500	-0.78477	0.03993	-0.67528	1.63574	-9.16510	7.38960
	0.975	-0.37995	0.07947	-0.52439	1.78801	-8.58180	7.97550
LSO	0.025	-0.02069	0.26621	-0.12051	0.68441	-9.58470	5.17390
	0.500	0.05548	0.33141	-0.08401	0.75251	-9.58400	5.17410
	0.975	0.13160	0.39660	-0.04741	0.82052	-9.58230	5.18120
MAR	0.025	-1.83590	-0.41465	-0.44281	0.69713	-9.49530	0.63270
	0.500	0.03327	0.08896	-0.30392	1.21649	-9.37760	1.82110
	0.975	1.90094	0.59504	-0.16901	1.73321	-9.27100	2.41120
MDG	0.025	-2.64673	-2.49075	-0.45561	3.47144	-11.21500	8.05430
	0.500	-2.38387	-2.28221	-0.39671	3.67890	-11.19920	8.06700
	0.975	-2.12132	-2.07399	-0.33787	3.88641	-11.18670	8.08020
MEX	0.025	1.03162	-0.27209	-0.62297	1.66575	-10.07250	7.40350
	0.500	1.17366	-0.19188	-0.57606	1.76798	-10.04700	7.42800
	0.975	1.31578	-0.11188	-0.52943	1.87045	-10.01570	7.45680
MLI	0.025	-2.54980	-1.11344	-0.33093	2.21053	-11.13650	7.38620
	0.500	-2.08447	-1.01468	-0.29984	2.31459	-11.00430	7.43970
	0.975	-1.61957	-0.91627	-0.26899	2.41891	-10.92630	7.53000
MMR	0.025	-0.98437	0.31240	-0.88266	1.21613	-10.86250	7.58670
	0.500	-0.85173	0.40773	-0.77008	1.36231	-10.03100	7.87570
	0.975	-0.71923	0.50316	-0.65784	1.50843	-9.63620	8.48930
MNG	0.025	-2.34887	0.18718	-0.90673	1.47501	-9.62690	7.40920
	0.500	-2.21991	0.28791	-0.86388	1.57588	-9.47610	7.49750
	0.975	-2.09138	0.38868	-0.82133	1.67682	-9.36220	7.61130
MOZ	0.025	-2.29426	3.13687	-0.63343	-2.10486	-9.67350	3.12010
	0.500	-0.55789	3.41630	-0.59574	-1.82073	-9.61280	3.33260
	0.975	1.17708	3.69786	-0.55793	-1.53907	-9.53020	3.45890

	Quantiles	int	gam	gbm	lasso	Nominal range	Nominal variance
MRT	0.025	-0.89031	-0.21342	0.24556	0.77777	-11.52500	3.89470
	0.500	1.01342	-0.15388	0.29511	0.86322	-11.52010	3.92070
	0.975	2.91565	-0.10772	0.35074	0.94877	-11.51520	3.94310
MWI	0.025	-2.10564	0.43801	-0.39226	0.81659	-10.24900	3.25200
	0.500	-0.18504	0.49256	-0.36684	0.87432	-10.19070	3.65740
	0.975	1.73400	0.54709	-0.34160	0.93201	-10.14110	3.89620
NAM	0.025	0.08378	-0.49628	-0.27502	1.39127	-11.19650	8.11470
	0.500	0.38802	-0.33566	-0.22124	1.55693	-11.17650	8.13110
	0.975	0.69208	-0.17472	-0.16744	1.72182	-11.16060	8.14760
NER	0.025	-0.02301	0.56862	-0.59850	0.91662	-7.17550	5.83860
	0.500	0.08670	0.60425	-0.55888	0.95454	-7.06800	5.96560
	0.975	0.19630	0.63995	-0.51944	0.99261	-6.94620	6.08250
NGA	0.025	-2.66069	0.72674	-0.96688	1.01027	-11.23060	7.75270
	0.500	-2.46570	0.82091	-0.92537	1.10450	-11.08590	7.79970
	0.975	-2.27095	0.91503	-0.88397	1.19865	-11.00770	7.88300
NIC	0.025	-2.13598	-1.02801	-1.24334	1.66017	-16.33790	8.39200
	0.500	-0.18794	-0.34679	-1.04635	2.39428	-15.62080	8.66760
	0.975	1.75852	0.33508	-0.85265	3.12733	-15.13240	9.06740
NPL	0.025	-2.09820	1.67068	-1.40708	0.33078	-9.85020	4.85040
	0.500	-0.18537	1.79596	-1.32775	0.51095	-9.84800	4.85200
	0.975	1.72592	1.97540	-1.26286	0.67174	-9.84570	4.85370
PAK	0.025	-0.29350	0.75143	-1.57009	1.52973	-9.43160	7.30630
	0.500	-0.20900	0.85316	-1.50663	1.65351	-9.21770	7.41440
	0.975	-0.12458	0.95487	-1.44404	1.77767	-9.06450	7.56380
PAN	0.025	-0.79496	-0.31667	-0.16598	0.65863	-6.94090	3.90040
	0.500	0.31082	-0.10630	0.14351	0.96284	-6.59080	4.69730
	0.975	1.41589	0.10392	0.45310	1.26621	-6.20450	5.38150
PER	0.025	-0.00203	0.41520	0.16809	0.36852	-9.81300	3.41210
	0.500	0.02036	0.43058	0.18336	0.38607	-9.81210	3.41480
	0.975	0.04272	0.44595	0.19861	0.40360	-9.81130	3.41690
PHL	0.025	-1.32899	1.63066	-0.57281	- 0.28558	-10.62340	7.42810
	0.500	-0.97209	1.69741	-0.51624	- 0.18108	-10.60860	7.43780
	0.975	-0.61553	1.76423	-0.45977	- 0.07683	-10.59820	7.44810
PNG	0.025	-6.91688	- 10.39059	0.51146	9.08625	-9.95390	2.59160
	0.500	-5.06992	-9.38299	0.66593	9.78345	-9.77090	2.75660
	0.975	-3.22567	-8.71305	0.83591	10.7689 5	-9.57800	2.92190
PRY	0.025	-0.35561	-0.04949	-0.47797	1.31512	-9.01740	6.89410
	0.500	-0.19793	0.01771	-0.43063	1.41296	-8.72860	7.07850
	0.975	-0.04036	0.08487	-0.38336	1.51070	-8.49800	7.30470
	0.025	-1.24436	-0.22846	-0.43226	1.53667	-11.29620	8.05390

	Quantiles	int	gam	gbm	lasso	Nominal range	Nominal variance
RWA	0.500	-0.70912	-0.18730	-0.39818	1.58545	-11.24820	8.10760
	0.975	-0.17435	-0.14614	-0.36421	1.63424	-11.19480	8.15180
SDN	0.025	-0.43656	-0.12680	-0.76642	1.53897	-6.53100	4.90890
	0.500	-0.26342	0.00015	-0.70166	1.70154	-6.39720	5.04730
	0.975	-0.09044	0.12690	-0.63717	1.86423	-6.26000	5.17800
SEN	0.025	-1.94482	1.05087	-0.44945	0.25483	-8.83640	3.85400
	0.500	-0.02716	1.10464	-0.42403	0.31940	-8.83540	3.85490
	0.975	1.88895	1.15879	-0.39876	0.38364	-8.83410	3.85580
SLE	0.025	-4.08085	-0.06371	-0.36666	1.23967	-11.35220	7.89810
	0.500	-3.42975	0.01448	-0.33633	1.32188	-11.26170	7.95060
	0.975	-2.77981	0.09227	-0.30616	1.40446	-11.07870	8.02670
SLV	0.025	0.55803	0.49186	-0.90291	0.98606	-10.36690	7.80510
	0.500	1.03967	0.62940	-0.78031	1.15117	-10.31870	7.85570
	0.975	1.52110	0.76726	-0.65856	1.31604	-10.26370	7.89810
SOM	0.025	-2.94100	1.22481	-0.85689	0.29641	-10.97650	6.83960
	0.500	-2.03463	1.32415	-0.74779	0.42396	-10.68860	7.06870
	0.975	-1.12991	1.42405	-0.63929	0.55077	-10.42690	7.32150
SSD	0.025	-0.85146	0.12448	-0.13039	0.55042	-9.41430	7.20210
	0.500	-0.72228	0.29504	0.00127	0.70380	-9.12960	7.36580
	0.975	-0.59346	0.46542	0.13272	0.85709	-8.90370	7.56680
SWZ	0.025	0.01205	-0.22059	-0.44720	1.38989	-8.92810	3.20750
	0.500	0.15462	-0.10702	-0.39946	1.50659	-8.92500	3.21570
	0.975	0.29708	0.00653	-0.35207	1.62319	-8.91920	3.22380
SYR	0.025	0.08152	0.05171	0.06965	0.33220	-8.28130	6.40410
	0.500	0.41133	0.11690	0.31332	0.56771	-8.18720	6.58840
	0.975	0.74128	0.18670	0.56154	0.79822	-8.08880	6.74930
TCD	0.025	-1.69004	0.38954	-0.93283	1.29083	-10.68990	7.84390
	0.500	-1.53979	0.44956	-0.85333	1.40372	-10.39910	7.96370
	0.975	-1.39001	0.50974	-0.77421	1.51663	-10.22370	8.16210
TGO	0.025	-2.18618	-0.10937	0.62819	-0.08023	-11.88190	7.57890
	0.500	-1.52566	0.14535	0.72250	0.13247	-10.87000	7.97460
	0.975	-0.86649	0.39608	0.81835	0.34634	-10.28110	8.61970
THA	0.025	-0.23684	-0.02052	0.05904	0.58579	-4.30870	2.85840
	0.500	0.06166	0.11200	0.17525	0.71267	-4.10360	3.47150
	0.975	0.35987	0.24449	0.29130	0.83949	-3.87270	3.94740
TJK	0.025	-1.03067	0.66997	-0.19032	0.31167	-10.05490	6.58810
	0.500	0.20142	0.76237	-0.17204	0.40967	-10.05110	6.59110
	0.975	1.43253	0.85478	-0.15381	0.50754	-10.04810	6.59490
TKM	0.025	-0.01941	0.01325	0.37898	0.19853	-9.13470	7.04930
	0.500	0.04471	0.13665	0.51779	0.34546	-8.36880	7.38210
	0.975	0.10877	0.26006	0.65652	0.49221	-7.92610	7.94690
	0.025	-0.71540	0.37083	-0.95931	1.39856	-11.86170	8.37750

	Quantiles	int	gam	gbm	lasso	Nominal range	Nominal variance
TLS	0.500	-0.15094	0.41838	-0.89613	1.47774	-11.79770	8.46510
	0.975	0.41303	0.46597	-0.83328	1.55700	-11.72110	8.52290
TUN	0.025	-0.31225	0.05144	-0.04310	0.28175	-5.76500	4.47470
	0.500	-0.10273	0.28502	0.18317	0.53171	-5.37310	5.10830
	0.975	0.10674	0.51838	0.40918	0.78179	-4.98710	5.74980
TZA	0.025	-1.94390	1.11807	-1.06440	0.60088	-10.87980	7.74570
	0.500	-1.68530	1.25399	-0.99973	0.74592	-10.86920	7.75590
	0.975	-1.42699	1.39031	-0.93566	0.89057	-10.86200	7.76290
UGA	0.025	-3.53892	0.60360	-0.25932	0.47584	-11.01890	7.88820
	0.500	-3.21866	0.67678	-0.23150	0.55486	-11.01330	7.89560
	0.975	-2.89901	0.75011	-0.20410	0.63371	-11.00610	7.90870
UZB	0.025	-0.38492	-0.24401	-0.99910	1.54779	-9.81960	7.92600
	0.500	-0.30116	0.01206	-0.78640	1.77425	-9.43660	8.09750
	0.975	-0.21747	0.26789	-0.57405	2.00099	-9.16590	8.34090
VNM	0.025	-2.25932	1.30191	-0.32914	-0.31288	-10.55000	7.74330
	0.500	-1.97762	1.44065	-0.27975	-0.16072	-10.52330	7.77090
	0.975	-1.69645	1.57986	-0.23170	-0.00864	-10.50280	7.78840
YEM	0.025	-3.90885	-1.01890	-2.15281	3.79163	-16.39770	8.25010
	0.500	-2.59114	-0.87195	-2.07234	3.94428	-14.66670	8.95970
	0.975	-1.27462	-0.72551	-1.99256	4.09791	-13.19540	9.77670
ZAF	0.025	0.44037	0.44928	-0.19139	0.64471	-6.02710	4.59660
	0.500	0.65749	0.48223	-0.16751	0.68526	-5.97030	4.76620
	0.975	0.87449	0.51516	-0.14363	0.72577	-5.89900	4.88480
ZMB	0.025	-5.31448	-1.26428	-0.35503	2.13812	-11.61390	7.43070
	0.500	-4.75796	-1.03699	-0.32096	2.35796	-11.19960	7.59130
	0.975	-4.20290	-0.80953	-0.28702	2.57734	-10.95350	7.86700
ZWE	0.025	-2.34640	3.90118	-0.63489	-2.75871	-10.58350	7.23810
	0.500	-1.83489	4.11736	-0.57266	-2.54454	-10.39360	7.35370
	0.975	-1.32458	4.33468	-0.51101	-2.33162	-10.25930	7.51580

Appendix Table 8. Fitted parameters by region for model of access to improved water

Lower, median, and upper quantiles (0.025%, 0.50%, 0.975%) are displayed for the main parameters by region. The first four columns provide information on the fixed effects: the intercept (int) and the covariates (gam, gbm, and enet) corresponding to the predicted ensemble rasters. Fitted values for the spatiotemporal field are shown in the next two columns. Countries are abbreviated by their ISO3 codes.

	Quantiles	int	gam	gbm	lasso	Nominal range	Nominal variance
AFG	0.025	-0.29518	0.64106	-1.78422	1.87630	-12.06830	8.15890
	0.500	0.07539	0.71091	-1.69982	1.98885	-12.01540	8.18920
	0.975	0.44566	0.78078	-1.61565	2.10141	-11.96220	8.21890
AGO	0.025	-1.13163	-2.39897	-0.59642	3.52596	-10.13550	7.45770
	0.500	-0.95820	-2.20050	-0.52674	3.72823	-10.09220	7.48380
	0.975	-0.78496	-2.00278	-0.45959	3.93128	-10.06420	7.50430
BDI	0.025	-1.89806	0.71311	-0.82880	0.89905	-10.07280	5.46130
	0.500	0.04162	0.79761	-0.78210	0.98477	-10.07080	5.54710
	0.975	1.97972	0.88229	-0.73612	1.07029	-10.06870	5.63300
BEN	0.025	-0.03422	0.89068	-0.84210	0.74117	-11.40840	7.99780
	0.500	0.30488	0.94518	-0.77993	0.83475	-11.12650	8.11700
	0.975	0.64374	0.99986	-0.71792	0.92821	-10.94270	8.29980
BFA	0.025	0.21318	0.56945	-1.61739	1.68907	-10.86860	7.85080
	0.500	0.41551	0.68182	-1.50525	1.82340	-10.55100	7.99400
	0.975	0.61770	0.79404	-1.39351	1.95809	-10.33500	8.20150
BGD	0.025	2.45620	0.18169	-0.14623	0.55306	-9.68620	7.00690
	0.500	2.78891	0.33713	-0.04287	0.70601	-9.50810	7.14970
	0.975	3.12194	0.49336	0.05983	0.85804	-9.37420	7.34200
BOL	0.025	-0.12885	0.66642	-0.06896	0.20559	-5.01420	4.24220
	0.500	-0.01022	0.74242	-0.04051	0.29806	-4.78330	4.57220
	0.975	0.10827	0.81833	-0.01217	0.39059	-4.54660	4.89090
BRA	0.025	-0.02654	-0.38897	0.14574	0.41168	-2.72970	1.77830
	0.500	0.07497	-0.09375	0.36496	0.72845	-2.39130	2.47150
	0.975	0.17653	0.20282	0.58368	1.04456	-2.02210	3.07890
BWA	0.025	0.31628	-0.71514	-0.14093	1.35225	-6.26350	4.10990
	0.500	0.95117	-0.51639	-0.04957	1.56572	-6.14210	4.43800
	0.975	1.58568	-0.31822	0.04228	1.77924	-6.00330	4.70540
CAF	0.025	-0.18202	0.38072	-0.66916	0.91163	-6.59400	5.47790
	0.500	-0.07052	0.50370	-0.56611	1.06249	-6.44040	5.65420
	0.975	0.04089	0.62657	-0.46328	1.21326	-6.29550	5.83700
CHN	0.025	-0.24043	0.31943	-0.21814	0.31326	-3.72460	2.60640
	0.500	-0.04796	0.51485	-0.08208	0.56734	-3.49180	3.11130
	0.975	0.14452	0.71050	0.05376	0.82087	-3.24130	3.54900
CIV	0.025	0.21971	0.62938	-1.03957	0.91508	-10.01790	7.55330
	0.500	0.36078	0.77906	-0.90129	1.12215	-9.65660	7.75040
	0.975	0.50177	0.92876	-0.76333	1.32913	-9.39380	8.01730
CMR	0.025	-0.38006	0.58817	-0.84838	0.99787	-9.68110	7.32210
	0.500	-0.20231	0.66824	-0.78175	1.11366	-9.64360	7.36270
	0.975	-0.02472	0.74881	-0.71767	1.23069	-9.60310	7.39780
COD	0.025	-1.01804	1.29132	-1.45444	0.58860	-11.10340	7.70870
	0.500	-0.81097	1.51650	-1.34423	0.82590	-10.83270	7.80250
	0.975	-0.60359	1.76008	-1.23509	1.05027	-10.67980	7.96880

	Quantiles	int	gam	gbm	lasso	Nominal range	Nominal variance
COG	0.025	-0.20227	0.38638	-1.10397	1.34604	-9.71980	8.04530
	0.500	-0.09656	0.47536	-0.99307	1.51785	-9.64920	8.11170
	0.975	0.00905	0.56436	-0.88306	1.68978	-9.55520	8.17780
COL	0.025	-0.65269	0.57844	-0.77484	0.81178	-10.08610	7.27530
	0.500	-0.47473	0.70892	-0.68083	0.97196	-9.95320	7.33390
	0.975	-0.29692	0.83961	-0.58716	1.13193	-9.86730	7.42180
COM	0.025	-0.85820	-1.24781	-0.08443	1.81531	-10.91360	7.32550
	0.500	0.54436	-1.00513	-0.00833	2.01318	-10.68980	7.63060
	0.975	1.94642	-0.77639	0.06664	2.22710	-10.44000	7.84010
DOM	0.025	-1.38203	0.25883	-0.34764	0.29440	-12.84780	7.25480
	0.500	0.23557	0.42648	-0.05355	0.62837	-11.74930	7.81540
	0.975	1.85191	0.59600	0.23947	0.95873	-10.94230	8.56970
DZA	0.025	-0.12304	0.00884	0.30717	-0.01865	-4.62110	2.35760
	0.500	0.30681	0.29239	0.48337	0.22072	-4.19710	3.45490
	0.975	0.74229	0.57606	0.66818	0.45863	-3.69850	4.30940
ECU	0.025	-1.91816	0.26207	-1.06558	0.40829	-11.48380	7.19370
	0.500	-1.24142	0.78813	-0.69400	0.90957	-10.57010	7.65280
	0.975	-0.56607	1.31759	-0.33219	1.40841	-9.88850	8.26430
EGY	0.025	0.15063	0.40257	-0.30671	0.21644	-8.34940	5.96730
	0.500	0.39068	0.68925	-0.20267	0.51413	-7.66990	6.60530
	0.975	0.63283	0.98434	-0.09942	0.80240	-7.09530	7.37190
ERI	0.025	-0.18060	0.27057	-0.05481	0.43431	-4.22380	2.73290
	0.500	0.05360	0.30080	0.10502	0.59405	-3.72370	3.77760
	0.975	0.28764	0.33123	0.26474	0.75363	-3.13590	4.60910
ETH	0.025	-0.60687	-0.19251	-0.89983	1.70352	-10.23600	7.54340
	0.500	-0.50172	-0.05546	-0.83603	1.89150	-10.01570	7.62140
	0.975	-0.39668	0.08168	-0.77240	2.07927	-9.89640	7.76200
GAB	0.025	-0.10752	0.76481	-0.37326	-0.19305	-11.15970	7.71320
	0.500	0.07402	1.00024	-0.16209	0.16194	-10.02090	8.12220
	0.975	0.25545	1.23627	0.04868	0.51617	-9.41710	8.88000
GHA	0.025	0.56945	0.42751	-0.88176	1.21741	-11.87410	8.18540
	0.500	0.83797	0.51023	-0.83555	1.32516	-11.67930	8.30380
	0.975	1.10635	0.59296	-0.78954	1.43307	-11.48430	8.42240
GIN	0.025	-0.01746	0.20862	-0.87699	1.09905	-10.26830	7.44000
	0.500	0.25369	0.41850	-0.75322	1.33550	-9.96590	7.61350
	0.975	0.52464	0.62774	-0.63240	1.57343	-9.74790	7.85110
GMB	0.025	-1.98386	-0.65060	-0.72802	1.25929	-13.43220	6.69270
	0.500	-0.02252	-0.15943	-0.51499	1.67558	-13.42080	6.71010
	0.975	1.93725	0.33082	-0.30424	2.09202	-13.41260	6.71910
GNB	0.025	-1.32687	1.46333	-1.65956	0.75404	-13.34290	7.44640
	0.500	0.40112	1.63218	-1.49229	0.85947	-12.11270	8.03810
	0.975	2.12773	1.80034	-1.32580	0.96779	-11.20970	8.85370

	Quantiles	int	gam	gbm	lasso	Nominal range	Nominal variance
GTM	0.025	0.28808	1.39333	-0.17790	-0.79612	-10.43350	7.77910
	0.500	0.59507	1.62397	-0.09574	-0.52823	-10.37600	7.85180
	0.975	0.90204	1.85762	-0.01378	-0.26349	-10.31320	7.90470
GUY	0.025	0.27730	0.58741	-0.16747	0.09356	-10.60150	7.97330
	0.500	0.59821	0.77610	-0.07417	0.29771	-10.52910	8.05900
	0.975	0.91921	0.96726	0.01920	0.49960	-10.46440	8.12350
HND	0.025	-1.90804	-3.31200	-0.18145	3.68271	-9.63280	3.78550
	0.500	0.00990	-2.92360	-0.08535	4.00920	-9.63080	3.78640
	0.975	1.92630	-2.56693	0.01249	4.36549	-9.62930	3.78740
HTI	0.025	-0.87651	0.02683	-0.28892	1.05129	-12.41470	8.56770
	0.500	-0.28242	0.10949	-0.25397	1.14451	-12.39030	8.58760
	0.975	0.31117	0.19224	-0.21914	1.23756	-12.37070	8.60820
IDN	0.025	0.02485	0.68417	-0.02459	0.31802	-8.80450	-131.99890
	0.500	0.03446	0.69169	-0.01811	0.32642	-8.80430	-131.99800
	0.975	0.04408	0.69920	-0.01164	0.33482	-8.80400	-131.99720
IND	0.025	0.63001	0.20133	-0.72654	1.39875	-8.40330	5.94180
	0.500	0.80628	0.24673	-0.70559	1.45884	-8.40130	5.96340
	0.975	0.98240	0.29209	-0.68469	1.51889	-8.40020	5.99040
IRQ	0.025	0.24429	0.48075	-0.54103	0.55696	-10.05930	7.48280
	0.500	0.46266	0.66828	-0.46994	0.80176	-9.81610	7.61190
	0.975	0.68099	0.85606	-0.39922	1.04615	-9.64420	7.79220
JOR	0.025	1.21012	0.54144	-0.12867	0.34132	-10.45210	7.55670
	0.500	1.61507	0.63278	-0.07774	0.44468	-10.05620	7.76960
	0.975	2.02347	0.72491	-0.02648	0.54725	-9.76920	8.05620
KEN	0.025	-1.13557	0.99471	-0.71211	0.54615	-11.25850	7.82130
	0.500	-0.81219	1.05034	-0.66378	0.61344	-11.23470	7.84580
	0.975	-0.48915	1.10601	-0.61550	0.68063	-11.21430	7.86150
KGZ	0.025	-1.83683	-0.19022	0.29475	0.26931	-10.15580	6.64930
	0.500	-0.68212	0.02205	0.56195	0.72201	-10.12020	6.87680
	0.975	0.49468	0.41011	0.89579	0.95233	-10.07680	7.04250
KHM	0.025	0.07147	0.35500	-0.17803	0.67801	-9.34780	3.85810
	0.500	0.13242	0.40833	-0.14556	0.73726	-9.34530	4.06340
	0.975	0.19332	0.46163	-0.11314	0.79644	-9.34210	4.21410
LAO	0.025	-0.70642	-1.61585	-0.79440	2.49665	-11.75180	7.58590
	0.500	-0.34533	-1.29156	-0.65389	2.94615	-10.71270	7.95180
	0.975	0.01543	-0.95592	-0.51119	3.38007	-10.18560	8.65930
LBR	0.025	-0.76663	-0.00718	-1.12603	1.82312	-11.57990	8.00100
	0.500	-0.11349	0.10131	-1.05530	1.95406	-11.49960	8.00950
	0.975	0.53922	0.21013	-0.98567	2.08512	-11.44090	8.02230

	Quantiles	int	gam	gbm	lasso	Nominal range	Nominal variance
LBY	0.025	0.04113	-0.11474	-0.09870	0.65411	-5.72670	3.88000
	0.500	0.46755	0.03619	0.08477	0.87672	-5.52560	4.25720
	0.975	0.89523	0.18749	0.27917	1.09306	-5.32360	4.61100
LKA	0.025	-0.01008	0.03274	-0.54772	0.85852	-10.05860	7.08350
	0.500	0.24603	0.19684	-0.33230	1.13500	-9.11100	7.73550
	0.975	0.50200	0.36054	-0.11440	1.41011	-8.22740	8.42480
LSO	0.025	-0.71335	-0.29951	-1.19793	1.92336	-11.41150	7.83100
	0.500	-0.23961	-0.06877	-1.09841	2.16733	-10.95860	8.04840
	0.975	0.23372	0.16167	-0.99982	2.41174	-10.63310	8.34950
MAR	0.025	-1.72043	1.08864	-1.41111	-0.52439	-8.41460	0.40790
	0.500	0.12946	1.49039	-0.72071	0.22728	-8.26590	1.83520
	0.975	1.97791	1.89716	0.00008	0.94923	-8.10400	2.57250
MDG	0.025	-1.11968	0.50790	-0.63921	0.82658	-10.08590	7.74870
	0.500	-0.97781	0.59609	-0.55964	0.96354	-10.05930	7.77750
	0.975	-0.83611	0.68495	-0.48045	1.10012	-10.03560	7.79690
MEX	0.025	0.25542	0.47449	-0.64645	0.85095	-9.37290	7.23550
	0.500	0.33686	0.57551	-0.56785	0.99244	-9.11130	7.37870
	0.975	0.41833	0.67654	-0.48944	1.13373	-8.90720	7.55940
MLI	0.025	-0.33492	0.66754	-0.75026	0.76252	-11.86400	7.81480
	0.500	-0.13726	0.75841	-0.66944	0.91090	-11.14040	8.03060
	0.975	0.06024	0.84935	-0.58903	1.05944	-10.79410	8.46040
MMR	0.025	0.15011	-0.54743	-0.76278	1.96436	-9.68100	7.33220
	0.500	0.27943	-0.43189	-0.69434	2.12626	-9.44370	7.46570
	0.975	0.40866	-0.31685	-0.62633	2.28875	-9.28630	7.66400
MNG	0.025	0.09800	0.99659	-0.49125	0.23423	-9.21690	7.58200
	0.500	0.15625	1.09379	-0.44134	0.34760	-9.10180	7.64140
	0.975	0.21448	1.19105	-0.39193	0.46094	-9.01920	7.71870
MOZ	0.025	-1.36349	0.92961	-2.23916	1.37300	-10.01880	7.29710
	0.500	-1.14636	1.29758	-2.03306	1.73566	-9.82830	7.39930
	0.975	-0.92947	1.66606	-1.82814	2.09791	-9.70650	7.55840
MRT	0.025	-0.34822	0.46197	-1.54926	1.84826	-9.39100	6.69800
	0.500	-0.15392	0.52257	-1.47126	1.94866	-9.01500	6.91830
	0.975	0.04024	0.58314	-1.39436	2.04994	-8.74650	7.22660
MWI	0.025	0.00913	0.52923	-0.31505	0.69882	-10.60810	7.67720
	0.500	0.05235	0.55592	-0.29198	0.73607	-10.60770	7.67740
	0.975	0.09552	0.58259	-0.26895	0.77330	-10.60740	7.70330
NAM	0.025	0.78033	1.48134	-0.75047	-0.26683	-10.30480	7.60150
	0.500	1.10572	1.65174	-0.60102	-0.04919	-10.25820	7.64900
	0.975	1.43130	1.82364	-0.45562	0.16748	-10.20260	7.71500
NER	0.025	-0.05139	0.85204	0.19876	-0.25276	-9.51610	7.63110

	Quantiles	int	gam	gbm	lasso	Nominal range	Nominal variance
	0.500	-0.01312	0.89300	0.26907	- 0.16200	-8.76200	7.90910
	0.975	0.02511	0.93399	0.33923	- 0.07133	-8.27150	8.36680
NGA	0.025	-0.65723	1.15020	-1.27379	0.90369	-9.75540	7.22760
	0.500	-0.51913	1.22731	-1.23101	1.00364	-9.75360	7.22960
	0.975	-0.38122	1.30440	-1.18846	1.10365	-9.75150	7.23130
NIC	0.025	-0.03765	0.18480	-1.52837	1.74067	-8.17300	6.15730
	0.500	0.19429	0.41707	-1.35554	1.93834	-7.77250	6.48260
	0.975	0.42606	0.64913	-1.18302	2.13634	-7.45540	6.88660
NPL	0.025	-0.79199	0.61165	-0.60806	0.49241	-10.25250	6.89570
	0.500	-0.22901	0.79598	-0.48313	0.68699	-10.03630	7.17500
	0.975	0.33320	0.98236	-0.35921	0.88064	-9.74610	7.38770
PAK	0.025	-0.09905	1.06129	-0.58107	0.30431	-8.92110	7.26240
	0.500	-0.04354	1.12627	-0.52898	0.40270	-8.68080	7.38560
	0.975	0.01193	1.19127	-0.47718	0.50110	-8.50790	7.55670
PAN	0.025	-1.68122	0.00348	-0.02650	0.69795	-7.68870	3.85410
	0.500	-0.45309	0.10266	0.06002	0.83554	-6.91810	4.97710
	0.975	0.76764	0.20852	0.15152	0.96561	-6.21210	6.20230
PER	0.025	-0.23305	0.78836	-0.17017	0.22487	-10.33350	7.87820
	0.500	-0.16456	0.84366	-0.13877	0.29512	-10.07500	7.97430
	0.975	-0.09613	0.89899	-0.10745	0.36530	-9.92550	8.14280
PHL	0.025	1.44765	0.37849	-0.35356	0.55950	-10.71420	7.67380
	0.500	1.71258	0.53395	-0.28739	0.75354	-10.70020	7.69900
	0.975	1.97757	0.69018	-0.22154	0.94670	-10.67480	7.71310
PNG	0.025	-2.06334	1.05793	-0.17172	- 1.86938	-10.40290	6.45890
	0.500	-1.17138	1.94352	-0.05639	- 0.88996	-10.02530	6.87210
	0.975	-0.28251	2.94061	0.06140	- 0.01836	-9.65710	7.28840
PRY	0.025	-0.16851	-0.54469	-0.56164	1.66283	-9.20290	7.05100
	0.500	-0.08455	-0.37423	-0.50174	1.87597	-8.46520	7.41930
	0.975	-0.00059	-0.20401	-0.44219	2.08929	-7.95460	7.93980
RWA	0.025	-0.42216	1.02653	-0.67756	0.48994	-11.48100	8.20860
	0.500	-0.15454	1.06626	-0.62279	0.55658	-11.06120	8.39640
	0.975	0.11287	1.10602	-0.56813	0.62313	-10.74250	8.64360
SDN	0.025	-0.18260	-0.47426	-1.17289	2.24030	-6.80340	5.72530
	0.500	-0.12345	-0.33590	-1.09743	2.43336	-6.66920	5.86240
	0.975	-0.06436	-0.19779	-1.02259	2.62687	-6.55130	6.01390
SEN	0.025	-0.06933	0.46681	-0.43455	0.79634	-9.12280	3.71470
	0.500	0.00825	0.52590	-0.39050	0.86457	-9.12270	3.71480
	0.975	0.08577	0.58495	-0.34652	0.93277	-9.10950	3.72420

	Quantiles	int	gam	gbm	lasso	Nominal range	Nominal variance
SLE	0.025	-1.23868	0.53520	-0.65638	0.82979	-11.77710	8.15880
	0.500	-0.67507	0.65925	-0.61473	0.95590	-11.72750	8.23120
	0.975	-0.11194	0.78306	-0.57458	1.08256	-11.67100	8.27760
SLV	0.025	-1.94948	0.92753	-1.72718	0.70353	-9.72570	-16.07890
	0.500	0.00324	1.30011	-1.31958	1.02033	-9.72010	-16.07660
	0.975	1.95440	1.67340	-0.91476	1.33678	-9.71680	-16.07430
SOM	0.025	-0.13404	0.70009	-0.41694	0.42685	-9.68070	7.68040
	0.500	0.00910	0.77938	-0.33958	0.56051	-9.61870	7.74720
	0.975	0.15228	0.85947	-0.26293	0.69336	-9.54100	7.80070
SSD	0.025	0.02884	0.60653	-0.65138	0.43370	-9.02720	7.60590
	0.500	0.06373	0.84636	-0.47442	0.62790	-8.57780	7.86820
	0.975	0.09858	1.08614	-0.29755	0.82186	-8.14900	8.14190
SWZ	0.025	-0.69757	0.72168	-0.12950	-0.14068	-10.57370	7.90690
	0.500	-0.23756	0.83553	0.04629	0.11811	-10.46710	8.02550
	0.975	0.22184	0.94933	0.22179	0.37680	-10.33490	8.12070
SYR	0.025	0.08012	-0.11413	-0.07108	0.25846	-8.96390	5.82480
	0.500	0.60447	0.17938	0.21580	0.59343	-8.11630	6.49140
	0.975	1.13527	0.50851	0.50259	0.91683	-7.57240	7.60810
TCD	0.025	-0.00034	1.02907	-0.24192	-0.04023	-10.01440	7.62880
	0.500	0.11905	1.10907	-0.16484	0.05569	-9.85130	7.70440
	0.975	0.23829	1.18934	-0.08780	0.15128	-9.74540	7.81890
TGO	0.025	-0.29008	0.61656	-0.03114	-0.04750	-9.18020	7.65670
	0.500	-0.08523	0.78301	0.06442	0.15355	-9.05050	7.83410
	0.975	0.11945	0.95085	0.16830	0.34249	-8.91130	7.95420
THA	0.025	-0.17988	0.52821	-0.08827	0.17889	-4.15960	2.63050
	0.500	0.08778	0.67317	-0.02451	0.35124	-3.77630	3.38500
	0.975	0.35549	0.81908	0.03987	0.52183	-3.36040	4.03700
TJK	0.025	-0.63908	0.79767	-0.20974	0.03871	-11.33140	7.79520
	0.500	0.06372	0.94881	-0.14859	0.19992	-11.31570	7.80990
	0.975	0.76567	1.10074	-0.08746	0.35970	-11.29430	7.82920
TKM	0.025	-0.25470	0.08756	-0.26672	0.60087	-5.11110	2.33720
	0.500	0.56449	0.30506	-0.18250	0.87709	-4.84440	3.21230
	0.975	1.38567	0.53546	-0.09773	1.14076	-4.55240	3.86240
TLS	0.025	-0.01593	0.70399	-0.82785	0.82274	-11.69260	8.32560
	0.500	0.26657	0.79291	-0.74548	0.95258	-11.14860	8.54270
	0.975	0.54884	0.88193	-0.66374	1.08251	-10.76720	8.85280
TUN	0.025	-0.36194	-0.10946	-0.21265	0.48076	-7.10600	3.71680
	0.500	0.98888	0.08332	0.10468	0.78798	-6.75350	4.57450
	0.975	2.34575	0.32759	0.42906	1.08011	-6.35640	5.28820
TZA	0.025	-0.52272	1.52537	-0.38530	-0.60645	-11.42200	8.05520

	Quantiles	int	gam	gbm	lasso	Nominal range	Nominal variance
	0.500	-0.36718	1.70947	-0.30411	- 0.40563	-11.02440	8.18560
	0.975	-0.21179	1.89428	-0.22263	- 0.20574	-10.81280	8.43590
UGA	0.025	-1.87088	-0.99025	-1.16301	2.77526	-9.66960	2.40590
	0.500	0.01559	-0.84661	-1.09781	2.94442	-9.62020	2.73600
	0.975	1.90052	-0.70339	-1.03284	3.11396	-9.56190	2.93140
UZB	0.025	-0.20203	0.45492	-0.30331	0.38449	-3.74890	1.93420
	0.500	-0.04765	0.60953	-0.20871	0.59895	-3.06690	3.34860
	0.975	0.10684	0.76387	-0.11435	0.81400	-2.22020	4.49440
VNM	0.025	0.93331	1.43819	-0.70648	- 0.29722	-9.93070	7.40720
	0.500	1.09773	1.63974	-0.58846	- 0.05118	-9.64480	7.57200
	0.975	1.26221	1.84237	-0.47120	0.19404	-9.43120	7.79540
YEM	0.025	1.39442	0.07197	-1.17053	1.82081	-10.84800	7.50510
	0.500	2.02117	0.16426	-1.10435	1.93919	-10.84020	7.51290
	0.975	2.64785	0.25969	-1.03910	2.05731	-10.83180	7.52130
ZAF	0.025	0.37415	0.65731	-0.07278	0.22366	-5.72570	4.17610
	0.500	0.68979	0.72962	-0.03757	0.30792	-5.62950	4.38130
	0.975	1.00545	0.80199	-0.00236	0.39199	-5.53830	4.57470
ZMB	0.025	-0.78334	0.19825	-1.62003	1.79883	-10.40560	7.33430
	0.500	-0.54669	0.40550	-1.49488	2.08930	-10.13030	7.46560
	0.975	-0.31026	0.61316	-1.37079	2.37969	-9.93870	7.65430
ZWE	0.025	0.32048	0.92418	-1.14073	0.24681	-10.09440	7.26760
	0.500	0.54350	1.31458	-0.94510	0.63056	-9.75580	7.49460
	0.975	0.76644	1.70556	-0.75008	1.01359	-9.48280	7.77440

Appendix Table 9. Fitted parameters by region for model of access to unimproved water

Lower, median, and upper quantiles (0.025%, 0.50%, 0.975%) are displayed for the main parameters by region. The first four columns provide information on the fixed effects: the intercept (int) and the covariates (gam, gbm, and enet) corresponding to the predicted ensemble rasters. Fitted values for the spatiotemporal field are shown in the next two columns. Countries are abbreviated by their ISO3 codes.

	Quantiles	int	gam	gbm	lasso	Nominal range	Nominal variance
AFG	0.025	0.21293	0.61936	-0.76660	0.97073	-8.49900	3.53180
	0.500	0.27739	0.68308	-0.72352	1.04047	-8.49900	3.53190
	0.975	0.34181	0.74678	-0.68054	1.11014	-8.49000	3.53200
AGO	0.025	-0.98184	0.33204	-0.40924	0.47148	-10.21350	7.13450
	0.500	-0.78726	0.58714	-0.31820	0.73090	-9.85440	7.32070
	0.975	-0.59292	0.84383	-0.22775	0.98924	-9.59810	7.58030
BDI	0.025	-1.35715	1.23245	-0.84050	0.16414	-9.82140	6.35500
	0.500	0.34324	1.41052	-0.76070	0.34978	-9.81850	6.35610
	0.975	2.04223	1.59223	-0.68184	0.53338	-9.81660	6.35720

	Quantiles	int	gam	gbm	lasso	Nominal range	Nominal variance
BEN	0.025	-1.88702	1.28709	-0.38789	-0.08810	-9.26080	3.73230
	0.500	0.02732	1.35318	-0.34779	-0.00735	-9.25930	3.86020
	0.975	1.94010	1.42656	-0.30894	0.07167	-9.25740	3.95240
BFA	0.025	2.11935	0.52693	-0.72358	0.85122	-10.48100	7.75260
	0.500	2.39957	0.63957	-0.63254	0.99303	-10.42470	7.79600
	0.975	2.68058	0.75265	-0.54301	1.13523	-10.38220	7.82680
BGD	0.025	0.06828	-0.28275	-0.17524	0.58391	-9.19320	6.03730
	0.500	1.00057	0.01033	0.09586	0.89370	-8.96530	6.23670
	0.975	1.93198	0.30395	0.36693	1.20222	-8.79140	6.49110
BOL	0.025	-0.33730	0.25877	-0.12627	0.55624	-5.15120	3.55980
	0.500	0.01486	0.35699	-0.04358	0.68655	-4.97120	3.94400
	0.975	0.36665	0.45525	0.03885	0.81680	-4.78650	4.28630
BRA	0.025	0.50170	-0.31404	0.03695	0.71734	-3.87100	-3.20010
	0.500	0.95275	-0.11890	0.19633	0.92003	-3.65350	0.02180
	0.975	1.40698	0.07664	0.36187	1.12126	-3.42850	1.59480
BWA	0.025	-0.21058	-0.53278	0.43513	0.47925	-6.72570	5.11100
	0.500	-0.02930	-0.34483	0.63805	0.70749	-6.39630	5.45650
	0.975	0.15160	-0.15846	0.84079	0.93564	-6.10560	5.85750
CAF	0.025	-0.10948	0.92089	-0.17485	-0.09305	-8.76010	6.99290
	0.500	-0.02112	1.02865	-0.07610	0.04731	-8.45170	7.27110
	0.975	0.06727	1.13680	0.02292	0.18692	-8.11870	7.52680
CHN	0.025	-0.55476	0.16401	-0.40393	0.66141	-4.97810	1.85410
	0.500	0.26763	0.38062	-0.31848	0.93791	-4.83020	2.64170
	0.975	1.08893	0.60023	-0.23296	1.21072	-4.66410	3.14990
CIV	0.025	0.44831	0.71919	-0.42895	0.11102	-10.26790	7.80440
	0.500	0.66714	0.93165	-0.26855	0.33672	-10.21460	7.86080
	0.975	0.88599	1.14538	-0.10819	0.56092	-10.15490	7.90580
CMR	0.025	1.39364	-1.20437	-0.61307	2.36008	-9.92260	6.96790
	0.500	1.71262	-1.02852	-0.50104	2.53129	-9.72350	7.07460
	0.975	2.03170	-0.85810	-0.39268	2.70771	-9.58590	7.22540
COD	0.025	0.83990	0.33583	-0.95031	1.17526	-10.22080	7.48940
	0.500	1.01192	0.45046	-0.80249	1.35195	-10.19670	7.51410
	0.975	1.18388	0.56648	-0.65549	1.52815	-10.17610	7.53110
COG	0.025	-0.31773	-0.39934	-1.27527	2.13325	-7.77620	5.63400
	0.500	0.01746	-0.17155	-1.18047	2.35245	-7.58420	5.81480
	0.975	0.35241	0.05602	-1.08701	2.57183	-7.40590	6.00520
COL	0.025	0.25152	0.03011	-0.56893	1.11911	-10.03910	7.16190
	0.500	0.50837	0.17440	-0.47338	1.29905	-10.00510	7.20280
	0.975	0.76513	0.31879	-0.37802	1.47868	-9.98250	7.26720
	0.025	-1.42694	0.04706	-0.43330	1.01385	-10.55860	5.56000

	Quantiles	int	gam	gbm	lasso	Nominal range	Nominal variance
COM	0.500	0.39453	0.10410	-0.25982	1.15738	-9.64510	6.47240
	0.975	2.21557	0.16068	-0.09706	1.30870	-8.71730	7.33780
DOM	0.025	-0.71235	-0.32524	-1.35833	1.67643	-11.81010	7.21140
	0.500	-0.14800	-0.19322	-0.91084	2.10402	-10.20520	7.94440
	0.975	0.41563	-0.06151	-0.46422	2.53211	-9.18160	9.06770
DZA	0.025	-0.39958	0.04086	-0.22093	0.39595	-5.89790	3.16030
	0.500	0.55440	0.38543	-0.06876	0.68326	-5.56190	3.83010
	0.975	1.54006	0.71308	0.09131	0.97945	-5.20070	4.42320
ECU	0.025	-2.13569	-0.28329	-0.51565	0.93539	-9.22450	5.81980
	0.500	-1.17650	0.00186	-0.26291	1.27346	-8.87880	6.20780
	0.975	-0.21966	0.28555	-0.04730	1.62474	-8.54850	6.63900
EGY	0.025	-0.58980	-0.01182	0.03124	0.93543	-7.17300	2.45450
	0.500	-0.41686	-0.00256	0.04772	0.95484	-7.16160	2.45990
	0.975	-0.24403	0.00668	0.06423	0.97421	-7.15530	2.46500
ERI	0.025	-0.46151	-0.72486	0.09622	0.70809	-6.23240	4.47000
	0.500	-0.03207	-0.42452	0.39280	1.03150	-5.90110	4.95120
	0.975	0.39722	-0.12509	0.68949	1.35506	-5.57390	5.42860
ETH	0.025	0.08413	0.96048	-0.93560	0.58276	-9.87900	7.47420
	0.500	0.17801	1.09002	-0.83993	0.75003	-9.73330	7.53390
	0.975	0.27183	1.21968	-0.74463	0.91707	-9.64440	7.62880
GAB	0.025	-1.28890	-1.21964	-0.61130	1.78736	-10.15760	6.88730
	0.500	-0.91967	-0.80004	-0.31193	2.11103	-9.59140	7.34850
	0.975	-0.55121	-0.38535	-0.01489	2.44399	-9.05630	7.83300
GHA	0.025	-0.04148	0.40706	-0.17742	0.66005	-9.27720	4.22580
	0.500	0.01177	0.44398	-0.14530	0.70129	-9.27550	4.23410
	0.975	0.06498	0.48100	-0.11321	0.74245	-9.27390	4.23880
GIN	0.025	0.97367	0.50399	-1.23328	0.77166	-10.54520	7.68340
	0.500	1.35971	0.91735	-1.04020	1.12370	-10.46030	7.74780
	0.975	1.74579	1.34597	-0.85501	1.46641	-10.39000	7.82970
GMB	0.025	1.17887	-0.43978	-1.02899	1.53270	-9.24740	5.96330
	0.500	2.06798	-0.23078	-0.59663	1.82315	-8.41710	6.75790
	0.975	3.00088	-0.03821	-0.23294	2.19674	-7.79180	7.81220
GNB	0.025	-0.67875	-0.17639	-1.10424	1.78530	-11.75230	7.28860
	0.500	0.23206	-0.02210	-0.97875	1.99984	-10.48380	7.91760
	0.975	1.14271	0.12575	-0.86249	2.23261	-9.58200	8.79950
GTM	0.025	0.96537	0.94868	-0.40406	-0.61252	-11.55560	7.48460
	0.500	1.39629	1.35965	-0.21516	-0.14455	-10.48540	7.89120
	0.975	1.82830	1.77706	-0.01968	0.30952	-9.92320	8.64290
GUY	0.025	-1.41242	0.08267	0.00558	-0.17679	-10.49010	7.40220
	0.500	-0.93791	0.40479	0.39904	0.19642	-10.09390	7.61280

	Quantiles	int	gam	gbm	lasso	Nominal range	Nominal variance
	0.975	-0.46498	0.73566	0.78743	0.56541	-9.57570	7.99740
HND	0.025	-3.19079	-0.16888	-0.57510	1.41358	-9.83060	6.58480
	0.500	-2.42293	-0.07178	-0.47983	1.55369	-9.59970	6.78810
	0.975	-1.65726	0.00966	-0.39120	1.71230	-9.40520	7.01420
HTI	0.025	2.25235	0.37516	-0.42245	0.66508	-11.95360	7.88980
	0.500	2.77187	0.52434	-0.34585	0.82201	-11.22620	8.15470
	0.975	3.29269	0.67610	-0.27062	0.97645	-10.80200	8.60110
IDN	0.025	1.07645	0.39094	-0.32509	0.85460	-8.29580	2.80190
	0.500	2.28350	0.42037	-0.30746	0.88709	-8.29580	2.80200
	0.975	3.48979	0.44981	-0.28985	0.91952	-8.29460	2.80220
IND	0.025	-0.92570	0.21772	-1.20855	1.74386	-8.66620	4.06010
	0.500	0.57651	0.31925	-1.16553	1.84624	-8.66590	4.06050
	0.975	2.07759	0.42086	-1.12266	1.94854	-8.66560	4.06080
IRQ	0.025	-3.63147	-0.32171	-1.26146	1.98233	-8.90600	5.61730
	0.500	-2.74746	-0.13438	-1.11197	2.24682	-8.60180	5.82190
	0.975	-1.87011	0.05252	-0.96639	2.51410	-8.41340	6.11350
JOR	0.025	-0.94413	-37.60393	-1.60096	-33.94522	-6.88200	0.88340
	0.500	0.09017	0.97981	0.03688	0.28015	-3.94850	5.20240
	0.975	1.40021	35.09130	1.21162	38.97701	0.16260	10.31760
KEN	0.025	-2.06886	0.21905	-1.19563	1.64656	-9.49470	4.14990
	0.500	-0.19312	0.34478	-1.11956	1.77472	-9.49150	4.16250
	0.975	1.68110	0.47053	-1.04371	1.90281	-9.48700	4.16990
KGZ	0.025	-0.13164	0.07834	0.11815	0.54715	-11.79370	-4.06780
	0.500	0.04004	0.14285	0.21175	0.64530	-11.79340	-4.06760
	0.975	0.21156	0.20746	0.30533	0.74330	-11.79290	-4.04130
KHM	0.025	-1.87640	0.17661	-0.71337	1.11173	-9.65860	4.77170
	0.500	0.02179	0.31781	-0.61635	1.29842	-9.65730	4.77780
	0.975	1.91843	0.45980	-0.51949	1.48450	-9.65600	4.78430
LAO	0.025	0.17036	0.96587	-0.89010	0.18769	-11.62030	7.44130
	0.500	0.78653	1.19572	-0.72496	0.52941	-11.05520	7.73880
	0.975	1.40239	1.42700	-0.55991	0.86885	-10.63930	8.10780
LBR	0.025	-1.22973	-28.62190	-28.24177	53.45973	-9.92280	2.89070
	0.500	0.69811	-28.32935	-28.01437	53.64120	-9.91850	2.89260
	0.975	2.62440	-28.20011	-27.91753	54.07133	-9.91520	2.89560
LBY	0.025	-0.55575	-22.02953	-0.63413	-19.48553	-6.27850	1.64550

	Quantiles	int	gam	gbm	lasso	Nominal range	Nominal variance
	0.500	0.09243	0.45575	-0.06200	0.48384	-2.88070	5.03370
	0.975	0.87014	20.44072	0.62031	23.09218	1.73420	10.07660
LKA	0.025	1.53472	-0.29533	-1.11773	1.55349	-10.45440	6.23710
	0.500	2.37419	0.03298	-0.93471	1.90368	-9.49860	7.13960
	0.975	3.21490	0.36321	-0.75710	2.25261	-8.51520	8.00380
LSO	0.025	2.56068	-0.74483	-0.62230	1.67267	-11.24310	7.18750
	0.500	3.24235	-0.45274	-0.49913	1.95143	-10.27990	7.57410
	0.975	3.92817	-0.15929	-0.38029	2.23269	-9.78910	8.28800
MAR	0.025	2.94732	-0.03414	-0.71533	0.63040	-9.90540	6.00060
	0.500	3.84576	0.35130	-0.38576	1.03786	-9.15650	6.55720
	0.975	4.75334	0.73411	-0.05468	1.43886	-8.72880	7.57390
MDG	0.025	0.15575	1.27702	-0.93797	0.11299	-10.26050	7.61580
	0.500	0.30119	1.47370	-0.81560	0.34196	-10.02070	7.71590
	0.975	0.44653	1.67061	-0.69369	0.57066	-9.88310	7.89160
MEX	0.025	0.12847	0.47538	-0.08363	0.19204	-9.18050	6.73620
	0.500	0.22975	0.63435	-0.00489	0.37053	-8.73970	7.13610
	0.975	0.33070	0.79431	0.07353	0.54813	-8.21680	7.47150
MLI	0.025	1.50506	0.98792	-0.33087	-0.21019	-9.88830	7.17830
	0.500	1.75976	1.21586	-0.22437	0.00810	-9.87560	7.19230
	0.975	2.01461	1.44691	-0.11876	0.22473	-9.85850	7.20520
MMR	0.025	0.07658	0.71753	-0.82980	0.76642	-10.06030	7.55270
	0.500	0.27716	0.81931	-0.74403	0.92465	-10.01210	7.58710
	0.975	0.47768	0.92149	-0.65885	1.08279	-9.97770	7.61700
MNG	0.025	0.18466	0.31393	-0.74957	1.03637	-7.55870	5.63670
	0.500	0.34637	0.46617	-0.64881	1.18286	-7.46100	5.78070
	0.975	0.50800	0.61847	-0.54831	1.32888	-7.34020	5.89260
MOZ	0.025	1.03221	2.41542	-1.27567	-1.06917	-10.34330	7.56700
	0.500	1.26229	2.81248	-1.10389	-0.70800	-10.26310	7.64230
	0.975	1.49238	3.21213	-0.93469	-0.34875	-10.20910	7.68750
MRT	0.025	1.32716	-0.06005	-0.72721	1.56319	-9.23280	6.42300
	0.500	1.60041	-0.00494	-0.64966	1.65446	-8.88430	6.71640
	0.975	1.87497	0.05092	-0.57297	1.74585	-8.55190	7.02730
MWI	0.025	-1.86305	0.43259	-1.23717	1.44020	-9.68680	3.79070
	0.500	0.05246	0.56344	-1.14378	1.57890	-9.68560	3.79190
	0.975	1.96643	0.69376	-1.06157	1.73270	-9.68400	3.79350
NAM	0.025	-0.97838	0.20214	0.24415	-0.03914	-11.05410	8.07900
	0.500	-0.60000	0.36128	0.43457	0.20134	-11.02140	8.10640
	0.975	-0.22256	0.52829	0.62782	0.43696	-10.99380	8.13740

	Quantiles	int	gam	gbm	lasso	Nominal range	Nominal variance
NER	0.025	0.34213	-0.09645	-0.18686	0.83788	-6.28570	3.90000
	0.500	1.00507	0.07471	-0.09398	1.01815	-6.00990	4.35030
	0.975	1.67798	0.24904	-0.00008	1.19614	-5.72490	4.77800
NGA	0.025	-46.09499	-72.50372	33.56365	39.33331	-9.40650	3.50370
	0.500	-44.27163	-72.23624	33.65912	39.58928	-9.40570	3.56560
	0.975	-42.44998	-72.02651	33.78428	39.84985	-9.40490	3.62770
NIC	0.025	0.52985	-0.53068	-1.66239	2.53407	-8.36920	5.53540
	0.500	1.39043	-0.26794	-1.50326	2.77100	-8.14650	5.82120
	0.975	2.25102	-0.00518	-1.34601	3.00923	-7.91370	6.08570
NPL	0.025	-0.46198	0.34327	-2.03632	1.49473	-11.64610	7.83620
	0.500	0.57659	0.70737	-1.67147	1.97526	-11.60740	7.90300
	0.975	1.61483	1.07136	-1.32764	2.45253	-11.57630	7.98640
PAK	0.025	0.22583	0.31644	0.01263	0.35148	-7.82780	5.80390
	0.500	0.34656	0.43140	0.10050	0.46801	-7.36820	6.13160
	0.975	0.46724	0.54621	0.18838	0.58450	-7.08180	6.65670
PAN	0.025	-1.29904	-0.07697	-0.15497	0.72133	-7.58640	4.28010
	0.500	-0.16876	0.05601	0.04191	0.90186	-7.01940	5.05300
	0.975	0.95996	0.18969	0.23981	1.08045	-6.47470	5.85290
PER	0.025	-0.46097	1.00046	-0.45845	0.19109	-10.26840	7.56050
	0.500	-0.35269	1.09847	-0.40199	0.30377	-10.01790	7.68490
	0.975	-0.24454	1.19660	-0.34671	0.41654	-9.84930	7.86530
PHL	0.025	2.23547	-0.08147	-0.42447	0.94559	-10.27940	7.47550
	0.500	2.54472	0.05921	-0.23839	1.17623	-10.21810	7.56240
	0.975	2.85535	0.21389	-0.05130	1.39793	-10.14780	7.61950
PNG	0.025	-3.29524	-1.90167	-0.00866	0.59160	-9.18460	5.29200
	0.500	-2.09300	-0.73089	0.06965	1.66035	-8.68890	5.67300
	0.975	-0.90568	0.33375	0.15435	2.83299	-8.25370	6.06860
PRY	0.025	-0.42181	0.64839	-0.20035	0.23176	-7.43440	5.42370
	0.500	-0.11230	0.74034	-0.11501	0.37446	-7.17310	5.76710
	0.975	0.19676	0.83398	-0.02983	0.51600	-6.90310	6.09200
RWA	0.025	-0.40214	-0.03269	-0.60127	1.38660	-10.69070	7.44230
	0.500	0.16327	0.05485	-0.54065	1.48587	-10.37280	7.64450
	0.975	0.72824	0.14221	-0.48029	1.58528	-10.12200	7.89190
SDN	0.025	0.48275	-0.37235	0.03538	1.05401	-6.20500	4.14550
	0.500	0.90196	-0.25626	0.07983	1.17624	-6.08670	4.36680
	0.975	1.32116	-0.13982	0.12465	1.29785	-5.95430	4.57320
SEN	0.025	1.11997	0.43035	-0.20164	0.29164	-10.60630	7.40230
	0.500	1.44258	0.57817	-0.07587	0.49837	-10.10700	7.64290
	0.975	1.76726	0.72830	0.04715	0.70394	-9.70700	7.96320

	Quantiles	int	gam	gbm	lasso	Nominal range	Nominal variance
SLE	0.025	-0.60260	-0.22947	-1.55410	2.10103	-11.77860	7.85490
	0.500	-0.04423	0.01376	-1.37652	2.36221	-11.31000	8.05130
	0.975	0.51375	0.25685	-1.20440	2.62927	-11.00870	8.36040
SLV	0.025	-1.44807	-0.70661	-0.88406	1.44151	-12.44650	7.65500
	0.500	-0.28624	-0.38668	-0.57490	1.96118	-11.57750	8.05140
	0.975	0.87492	-0.06851	-0.26854	2.48541	-10.97060	8.62190
SOM	0.025	0.10953	0.21463	-0.58218	0.90446	-6.46440	3.71120
	0.500	1.07020	0.36686	-0.46475	1.09794	-6.24560	4.11080
	0.975	2.03103	0.52100	-0.34810	1.29005	-6.02340	4.47790
SSD	0.025	-0.20699	0.29608	0.12804	0.27344	-8.61350	6.49690
	0.500	-0.12602	0.40233	0.23131	0.36624	-8.12920	6.94490
	0.975	-0.04511	0.50859	0.33457	0.45891	-7.63190	7.38310
SWZ	0.025	-0.24430	-0.53592	-0.52368	1.46224	-12.05790	7.29500
	0.500	0.55467	-0.38496	-0.33001	1.71509	-10.62860	7.77280
	0.975	1.35315	-0.23441	-0.13772	1.96905	-9.91830	8.66400
SYR	0.025	-1.20464	0.00661	-0.03223	- 0.15488	-7.75730	1.74900
	0.500	0.45210	0.47072	0.04128	0.48721	-6.30980	4.11860
	0.975	2.12022	1.10806	0.12630	0.95067	-4.65830	6.41490
TCD	0.025	0.88238	0.68340	-0.40444	0.42741	-10.38660	7.55060
	0.500	1.03957	0.79303	-0.32330	0.53004	-10.03940	7.73310
	0.975	1.19704	0.90402	-0.24220	0.63151	-9.78120	7.99020
TGO	0.025	-0.43006	0.23582	-0.17551	0.12460	-9.75080	7.76020
	0.500	-0.10370	0.52938	0.06385	0.40652	-9.62990	7.90370
	0.975	0.22244	0.82283	0.30408	0.68730	-9.48400	8.01250
THA	0.025	-0.42182	-0.12166	0.08440	0.02154	-4.44900	2.29790
	0.500	-0.06264	0.26629	0.31178	0.41900	-3.88230	3.26330
	0.975	0.29696	0.65509	0.54049	0.81814	-3.23470	4.05900
TJK	0.025	-1.17405	-0.03494	-1.17885	1.17221	-10.88880	7.31940
	0.500	-0.64829	0.34628	-0.91280	1.56895	-10.40290	7.57990
	0.975	-0.12413	0.72626	-0.65360	1.96788	-10.00760	7.89580
TKM	0.025	0.14107	-0.39722	-0.12105	0.23291	-5.00540	-2.29280
	0.500	1.33166	0.10193	0.15141	0.74107	-4.56370	0.93630
	0.975	2.53441	0.61005	0.43455	1.24067	-4.08970	2.71700
TLS	0.025	-0.06972	0.36392	0.05118	0.43590	-9.66330	4.43660
	0.500	0.03960	0.41568	0.09470	0.48962	-9.65960	4.43670
	0.975	0.14885	0.46736	0.13826	0.54328	-9.65060	4.45600
TUN	0.025	-1.58838	-0.30407	-0.56754	0.48190	-6.22990	0.84770
	0.500	-0.03720	0.13091	-0.14905	0.99596	-5.54150	3.22500
	0.975	1.51427	0.68141	0.31626	1.39845	-4.63110	4.70740
TZA	0.025	0.41472	1.07974	-0.60062	- 0.19297	-10.20100	7.49720
	0.500	0.56206	1.37641	-0.46306	0.08706	-9.98940	7.59740
	0.975	0.70933	1.67352	-0.32654	0.36637	-9.86160	7.76070

	Quantiles	int	gam	gbm	lasso	Nominal range	Nominal variance
UGA	0.025	0.34116	0.31996	-0.91319	1.21707	-10.62110	7.68530
	0.500	0.65131	0.42971	-0.79423	1.36523	-10.61020	7.69320
	0.975	0.96129	0.53931	-0.67705	1.51357	-10.60210	7.70290
UZB	0.025	-0.65389	-0.36750	-0.78792	1.12853	-4.66330	1.62180
	0.500	0.01113	0.00746	-0.54059	1.53338	-4.28740	2.75550
	0.975	0.67440	0.38566	-0.29556	1.93626	-3.85660	3.51940
VNM	0.025	0.82392	0.83917	-0.00016	-0.31071	-9.99360	7.00560
	0.500	1.10897	1.02194	0.09865	-0.12110	-9.51270	7.29430
	0.975	1.39436	1.20683	0.19823	0.06616	-9.18760	7.71130
YEM	0.025	-1.18580	0.01567	-0.49972	1.02076	-8.83850	5.04520
	0.500	0.49859	0.22135	-0.43760	1.21582	-8.82590	5.30750
	0.975	2.18176	0.43250	-0.37696	1.40761	-8.81660	5.60130
ZAF	0.025	-0.50941	0.55739	-0.41750	0.39776	-4.48380	3.00520
	0.500	-0.18945	0.66325	-0.24890	0.58584	-4.29450	3.51640
	0.975	0.12914	0.76886	-0.08071	0.77391	-4.10530	3.95620
ZMB	0.025	0.57505	1.12349	-0.45161	-0.23834	-10.36080	7.57000
	0.500	0.80686	1.36086	-0.33194	-0.02772	-10.32840	7.60420
	0.975	1.03861	1.60026	-0.21623	0.18181	-10.29910	7.62720
ZWE	0.025	0.79038	0.92417	-0.14536	-0.92293	-10.59410	7.24800
	0.500	1.08491	1.37066	0.13247	-0.50837	-9.99840	7.57400
	0.975	1.37963	1.83356	0.41523	-0.10465	-9.60240	8.05830

Appendix Table 10. Fitted parameters by region for model of access to piped sanitation

Lower, median, and upper quantiles (0.025%, 0.50%, 0.975%) are displayed for the main parameters by region. The first four columns provide information on the fixed effects: the intercept (int) and the covariates (gam, gbm, and enet) corresponding to the predicted ensemble rasters. Fitted values for the spatiotemporal field are shown in the next two columns. Countries are abbreviated by their ISO3 codes.

	Quantiles	int	gam	gbm	lasso	Nominal range	Nominal variance
AFG	0.025	-3.91776	-0.31048	-1.11333	1.94395	-9.95070	7.31230
	0.500	-3.63295	-0.10920	-0.95082	2.05885	-9.91290	7.34490
	0.975	-3.34927	0.09433	-0.79135	2.17639	-9.86130	7.38200
AGO	0.025	-0.54990	0.29289	-0.71410	1.11215	-5.94660	4.76140
	0.500	-0.41889	0.39537	-0.63916	1.24377	-5.72510	5.00590
	0.975	-0.28811	0.49783	-0.56448	1.37533	-5.51810	5.26400
BDI	0.025	-4.23310	-0.32750	-0.15806	1.21764	-10.89640	7.55820
	0.500	-3.73730	-0.21677	-0.11623	1.33288	-10.45530	7.80300
	0.975	-3.24346	-0.10593	-0.07438	1.44794	-10.12660	8.12110
BEN	0.025	-1.39369	0.99198	-0.15587	0.02176	-10.34250	7.79110
	0.500	-1.18293	1.04164	-0.12508	0.08320	-10.03050	7.93390
	0.975	-0.97365	1.09228	-0.09450	0.14433	-9.81650	8.13810
BFA	0.025	-3.02818	0.34429	-0.85649	1.32027	-10.38410	7.52140
	0.500	-2.84261	0.39790	-0.79707	1.39914	-9.84900	7.78350
	0.975	-2.65826	0.45169	-0.73826	1.47823	-9.49590	8.18030
BGD	0.025	-2.56016	0.79753	-0.53495	0.51746	-10.00270	7.23030
	0.500	-2.16550	0.87322	-0.48243	0.60915	-9.97810	7.25440
	0.975	-1.77132	0.94896	-0.43008	0.70080	-9.95120	7.28380
BOL	0.025	-0.06232	0.69394	-0.02747	0.12386	-4.33720	3.71310
	0.500	0.06584	0.76877	0.01093	0.22031	-4.16100	4.09000
	0.975	0.19388	0.84357	0.04927	0.31667	-3.97370	4.42450
BRA	0.025	-0.18581	0.09186	-0.17136	0.85979	-4.59600	3.72440
	0.500	-0.07823	0.17547	-0.12773	0.95214	-4.51780	3.87090
	0.975	0.02927	0.25914	-0.08394	1.04428	-4.43930	4.01500
BTN	0.025	-1.85473	-1.33325	-1.27227	2.66832	-13.29000	7.05480
	0.500	0.08377	-1.03390	-1.09126	3.12523	-12.67840	7.50840
	0.975	2.02071	-0.73470	-0.91050	3.58175	-12.16390	8.04190
BWA	0.025	-2.20477	-0.04169	-0.82676	1.52420	-10.13280	7.18350
	0.500	-1.85028	0.06333	-0.72755	1.66420	-9.72790	7.43110
	0.975	-1.49659	0.16835	-0.62892	1.80439	-9.42880	7.75400
CAF	0.025	-0.90929	0.02297	-0.29879	0.74968	-9.96640	7.29360
	0.500	-0.72934	0.20672	-0.13083	0.92366	-8.99930	7.77160
	0.975	-0.55366	0.39116	0.03583	1.09847	-8.36520	8.46030
CHN	0.025	-0.30340	-0.03471	0.05055	0.72474	-4.59080	3.07270
	0.500	0.01872	0.06059	0.11527	0.82385	-4.49740	3.34700
	0.975	0.34066	0.15581	0.18025	0.92294	-4.40960	3.60070
	0.025	-2.40879	0.28927	-0.87395	1.15080	-10.61400	7.64390

	Quantiles	int	gam	gbm	lasso	Nominal range	Nominal variance
CIV	0.500	-2.12579	0.44740	-0.77811	1.33059	-10.22340	7.81790
	0.975	-1.84356	0.60532	-0.68272	1.51094	-9.98380	8.09620
CMR	0.025	-1.52655	0.89441	-0.16685	0.10204	-10.88610	7.63580
	0.500	-1.25728	0.95681	-0.13045	0.17250	-10.59460	7.80760
	0.975	-0.98920	1.02413	-0.09436	0.24097	-10.32080	7.98710
COD	0.025	-1.90516	0.40368	-0.32886	0.74427	-8.60860	6.17200
	0.500	-1.68013	0.45983	-0.28465	0.82476	-8.31390	6.44630
	0.975	-1.45650	0.51617	-0.24044	0.90501	-8.04560	6.74990
COG	0.025	-2.16339	0.78004	-0.50036	0.37278	-12.49280	8.52810
	0.500	-1.71132	0.84690	-0.38621	0.53990	-12.01880	8.68510
	0.975	-1.26233	0.91505	-0.27304	0.70550	-11.73700	8.95280
COL	0.025	0.28832	0.49626	-0.07570	0.38300	-7.06560	5.67780
	0.500	0.39570	0.55193	-0.01587	0.46396	-6.94750	5.79930
	0.975	0.50303	0.60763	0.04387	0.54482	-6.83250	5.92550
COM	0.025	-2.38113	-0.74516	-0.07089	1.50334	-10.70480	6.69130
	0.500	-0.96558	-0.59578	-0.05428	1.65027	-10.11670	7.29150
	0.975	0.44809	-0.44661	-0.03834	1.79732	-9.56190	7.92630
CRI	0.025	0.07996	-0.01039	-0.70473	1.37388	-10.80040	8.06480
	0.500	0.44878	0.10259	-0.63994	1.53728	-10.71810	8.15710
	0.975	0.81734	0.21549	-0.57532	1.70064	-10.61700	8.23170
DOM	0.025	-0.72168	0.23946	-0.11005	0.54545	-10.56920	6.92160
	0.500	0.03995	0.35971	-0.04269	0.68297	-9.90680	7.37680
	0.975	0.80097	0.47991	0.02464	0.82028	-9.37010	7.93280
DZA	0.025	-0.02899	-0.01204	-0.01662	0.46450	-4.97970	4.51290
	0.500	0.05886	0.18657	0.15015	0.66358	-4.74670	4.80440
	0.975	0.14693	0.38419	0.31675	0.86287	-4.51260	5.09420
ECU	0.025	2.87938	311.53982	-14.36052	-298.75158	-9.62180	2.42050
	0.500	4.81278	311.88389	-14.20995	-297.96607	-9.61870	3.01500
	0.975	6.74467	312.67075	-14.09975	-297.61960	-9.61290	3.31070
EGY	0.025	-1.89207	-1.35669	-0.01322	2.09990	-9.47110	5.03360
	0.500	-0.02741	-1.24388	0.01805	2.22575	-9.46870	5.03550
	0.975	1.83575	-1.13077	0.04930	2.35123	-9.46400	5.03720
ERI	0.025	-0.33921	-0.93460	0.68034	-0.49336	-6.85500	4.24040
	0.500	0.20531	-0.18112	1.04595	0.13448	-6.22190	5.16510
	0.975	0.74414	0.58517	1.41663	0.74343	-5.59620	6.12020
	0.025	-2.35200	0.08724	-0.14254	0.86173	-9.13180	7.08460

	Quantiles	int	gam	gbm	lasso	Nominal range	Nominal variance
ETH	0.500	-2.19980	0.15630	-0.09916	0.94278	-8.96520	7.18500
	0.975	-2.04871	0.22557	-0.05579	1.02361	-8.83400	7.30970
GAB	0.025	-2.69070	0.32644	-0.47571	0.87621	-10.46530	7.31580
	0.500	-2.21272	0.41997	-0.42410	1.00393	-10.12830	7.50130
	0.975	-1.73664	0.51357	-0.37272	1.13180	-9.89180	7.76650
GHA	0.025	-2.86691	0.83035	-0.73637	0.73758	-9.78520	7.34050
	0.500	-2.57785	0.88057	-0.68942	0.80857	-9.77920	7.34670
	0.975	-2.28993	0.93208	-0.64272	0.87911	-9.77170	7.35480
GIN	0.025	-2.95079	1.06246	-1.62255	1.21827	-10.63680	7.95180
	0.500	-2.63120	1.15942	-1.52289	1.36335	-10.55730	8.00640
	0.975	-2.31372	1.25729	-1.42490	1.50893	-10.47030	8.06390
GNB	0.025	-3.10454	-3.18377	-0.82954	3.90801	-10.67370	7.17050
	0.500	-2.27183	-2.74940	-0.66992	4.41850	-9.88320	7.65870
	0.975	-1.44445	-2.36777	-0.53344	5.00582	-9.24360	8.29320
GTM	0.025	-0.83565	1.00306	-0.21952	0.05536	-11.50300	8.02680
	0.500	-0.46377	1.05769	-0.18739	0.12962	-11.20630	8.15510
	0.975	-0.09220	1.11250	-0.15587	0.20419	-11.00970	8.34540
GUY	0.025	-1.45281	-0.02797	-0.18100	0.91096	-11.10740	7.43120
	0.500	-1.05600	0.08889	-0.13475	1.04587	-10.29810	7.80460
	0.975	-0.65989	0.20580	-0.08861	1.18061	-9.79930	8.40710
HND	0.025	-1.09441	-0.05733	-0.08450	0.90325	-10.66350	7.72820
	0.500	-0.71587	0.03001	-0.03162	1.00139	-10.62580	7.76680
	0.975	-0.33771	0.11603	0.02204	1.10022	-10.59240	7.79490
HTI	0.025	-2.36213	0.45284	-0.04693	0.36347	-12.95830	8.06130
	0.500	-2.00394	0.53761	-0.00060	0.46306	-11.47280	8.51100
	0.975	-1.64703	0.62278	0.04562	0.56209	-10.70960	9.37880
IDN	0.025	-0.05098	0.46257	0.00643	0.48006	-9.03380	1.85420
	0.500	-0.03322	0.48009	0.01933	0.50058	-9.03300	1.85480
	0.975	-0.01548	0.49760	0.03222	0.52109	-9.03240	1.85530
IND	0.025	NaN	NaN	NaN	NaN	-8.24950	-147.54600
	0.500	NaN	NaN	NaN	NaN	-8.24950	-147.54600
	0.975	NaN	NaN	NaN	NaN	-8.24930	-147.54370
IRQ	0.025	-0.19860	0.08996	-0.64376	0.83395	-10.13130	7.96640
	0.500	-0.02606	0.36060	-0.44584	1.08521	-10.08710	8.00930
	0.975	0.14636	0.63099	-0.24804	1.33627	-10.02900	8.05070
JOR	0.025	-3.04818	0.73182	-0.26588	0.42613	-11.81580	7.91140
	0.500	-2.38726	0.77301	-0.24494	0.47197	-11.35390	8.11310
	0.975	-1.72736	0.81436	-0.22407	0.51758	-11.05890	8.41430
KEN	0.025	-2.78688	0.77858	0.27271	-0.15075	-9.11980	6.47390
	0.500	-2.30834	0.80706	0.30497	-0.11203	-9.10470	6.48880

	Quantiles	int	gam	gbm	lasso	Nominal range	Nominal variance
	0.975	-1.83257	0.83561	0.33723	-0.07349	-9.09190	6.50170
KGZ	0.025	-7.32624	-0.13020	-0.63767	1.44822	-11.57120	7.82080
	0.500	-6.28048	0.00090	-0.58042	1.57955	-11.50190	7.87740
	0.975	-5.23736	0.13245	-0.52407	1.71079	-11.40450	7.94380
KHM	0.025	-1.32421	1.50694	-0.03303	-0.59454	-10.13790	7.66430
	0.500	-1.11262	1.55131	-0.01381	-0.53750	-10.11700	7.68550
	0.975	-0.90127	1.59576	0.00537	-0.48058	-10.10250	7.69860
LAO	0.025	-1.97133	1.38321	-1.26919	0.32172	-10.80270	7.69550
	0.500	-1.67683	1.54752	-1.12355	0.57538	-10.40150	7.88720
	0.975	-1.38372	1.71315	-0.98079	0.83104	11.72330	8.16610
LBR	0.025	-5.27614	0.16808	-1.00209	1.54777	-11.09090	7.65700
	0.500	-4.82559	0.26876	-0.93346	1.66464	-10.60760	7.85470
	0.975	-4.37777	0.36952	-0.86547	1.78176	-10.31500	8.17600
LBY	0.025	-0.11131	-0.17949	-0.07711	0.54951	-5.86660	4.82840
	0.500	0.01654	-0.04918	0.21526	0.83399	-5.55420	5.20780
	0.975	0.14365	0.08088	0.50762	1.11810	-5.26160	5.61260
LKA	0.025	-2.95910	-0.39956	-1.41855	2.39988	-11.71600	6.99300
	0.500	-1.24519	-0.26991	-1.27074	2.54063	-11.29760	7.37040
	0.975	0.46733	-0.13892	-1.12350	2.68051	-10.85730	7.76600
LSO	0.025	-3.12696	0.26108	-0.44627	0.95291	-10.06710	7.45250
	0.500	-2.68159	0.34544	-0.39999	1.05412	-9.77140	7.62670
	0.975	-2.24181	0.42989	-0.35429	1.15634	-9.54770	7.85310
MAR	0.025	-0.21263	0.24637	0.03113	0.55436	-9.40040	4.72360
	0.500	-0.10207	0.31271	0.06429	0.62295	-9.39550	4.73830
	0.975	0.00839	0.37901	0.09750	0.69146	-9.38630	4.74600
MDG	0.025	-2.79919	-0.34486	-0.16918	1.15399	-10.38970	7.53380
	0.500	-2.60193	-0.20791	-0.08094	1.28891	-9.90390	7.74740
	0.975	-2.40568	-0.07083	0.00703	1.42362	-9.60740	8.08700
MEX	0.025	-1.49042	-1.75443	0.56770	2.06699	-8.50160	-146.14600
	0.500	0.15625	-1.70130	0.58105	2.12024	-8.46350	-146.01150
	0.975	1.80159	-1.64824	0.59440	2.17347	-8.43100	-145.87690
MLI	0.025	-0.80660	0.97879	0.30799	-0.44349	-9.72390	7.42870
	0.500	-0.68048	1.02921	0.34792	-0.37755	-9.51940	7.52130
	0.975	-0.55487	1.08127	0.38795	-0.31255	-9.38400	7.65830
MMR	0.025	-0.88522	0.78944	-0.37620	0.31043	-9.75330	7.57150
	0.500	-0.76368	0.85853	-0.28582	0.42744	-9.56700	7.65170
	0.975	-0.64228	0.92798	-0.19572	0.54400	-9.45590	7.78400
MNG	0.025	-3.53850	-0.73865	-0.52237	1.97298	-10.20980	7.56140
	0.500	-3.36252	-0.60959	-0.48413	2.09376	-10.03000	7.64640

	Quantiles	int	gam	gbm	lasso	Nominal range	Nominal variance
	0.975	-3.18735	-0.48055	-0.44637	2.21452	-9.91950	7.78210
MOZ	0.025	-2.02829	-0.47452	0.30883	0.86492	-7.03480	-2.05420
	0.500	-0.45671	-0.38574	0.39356	0.99238	-6.95350	-1.05360
	0.975	1.11349	-0.29673	0.47705	1.11999	-6.85320	-0.43610
MRT	0.025	-2.23630	0.11838	-0.92272	1.59834	-9.38810	7.09070
	0.500	-2.01172	0.19663	-0.88421	1.68752	-9.32970	7.13660
	0.975	-1.78807	0.27484	-0.84594	1.77683	-9.28030	7.19040
MWI	0.025	-2.03048	-0.23209	0.32104	0.73415	-8.53280	-8.85560
	0.500	-0.13532	-0.15943	0.35322	0.80647	-8.45440	-8.20580
	0.975	1.75827	-0.08958	0.38533	0.88102	-8.37980	-7.80900
NAM	0.025	-1.48158	0.83781	-0.26332	0.25728	-11.52210	7.93080
	0.500	-1.16876	0.89261	-0.22095	0.32827	-11.18580	8.05510
	0.975	-0.85633	0.94747	-0.17885	0.39937	-10.99660	8.27630
NER	0.025	-0.56774	1.03435	-0.23303	0.05796	-10.25890	7.86910
	0.500	-0.46811	1.06954	-0.18613	0.11672	-9.89780	8.04070
	0.975	-0.36939	1.10485	-0.13928	0.17507	-9.59660	8.24380
NGA	0.025	-1.62950	1.06051	-0.47666	0.19839	-10.54470	7.55030
	0.500	-1.49027	1.14381	-0.43923	0.29545	-10.31200	7.63340
	0.975	-1.35124	1.22708	-0.40186	0.39239	-10.18560	7.78220
NIC	0.025	-3.05139	0.69090	-0.81640	0.75924	-13.75180	8.13120
	0.500	-1.79325	0.78869	-0.72440	0.93594	-12.88060	8.55040
	0.975	-0.53670	0.88754	-0.63300	1.11163	-12.14330	9.03620
NPL	0.025	-1.94803	1.06006	-0.38513	0.09795	-10.09340	-5.92220
	0.500	-0.03178	1.14116	-0.34755	0.20640	-10.02970	-5.21070
	0.975	1.88293	1.22261	-0.31007	0.31445	-9.95920	-4.81500
PAK	0.025	-1.07693	-0.11436	0.52114	0.03730	-7.04710	5.02240
	0.500	-0.70914	-0.01791	0.73076	0.28643	-6.89150	5.24280
	0.975	-0.34236	0.07996	0.93928	0.53611	-6.76630	5.57710
PAN	0.025	-2.19139	-0.07953	-0.38759	0.94850	-8.12990	4.65340
	0.500	-0.95475	0.08772	-0.21144	1.12435	-7.61810	5.37400
	0.975	0.28049	0.25506	-0.03760	1.30045	-7.14090	6.15150
PER	0.025	-1.53198	-1.06802	-0.43136	2.29475	-12.05940	8.50760
	0.500	-1.37272	-0.98594	-0.39991	2.38586	-12.05500	8.51150
	0.975	-1.21362	-0.90394	-0.36853	2.47691	-12.04900	8.51530
PHL	0.025	0.15298	-0.46486	-0.48143	1.53415	-10.44560	7.52090
	0.500	0.41070	-0.29807	-0.41827	1.71626	-10.41540	7.55260
	0.975	0.66826	-0.13129	-0.35555	1.89837	-10.38670	7.57490
PNG	0.025	-1.81707	-6.46462	-14.27261	-17.19958	-7.18060	1.91570
	0.500	0.06711	0.34131	-2.01233	-1.36300	-3.25850	5.02630

	Quantiles	int	gam	gbm	lasso	Nominal range	Nominal variance
	0.975	1.94240	11.48041	9.26654	14.37403	1.60490	9.67210
PRY	0.025	-0.38361	0.76520	-0.27922	0.41273	-10.70150	7.63580
	0.500	-0.18485	0.79728	-0.25748	0.46018	-10.25940	7.83500
	0.975	0.01361	0.82938	-0.23580	0.50759	-9.93160	8.10100
RWA	0.025	-1.90439	0.63560	-0.40582	0.27897	-10.72460	7.74410
	0.500	-1.55327	0.83490	-0.34666	0.51269	-10.20390	8.02620
	0.975	-1.20578	1.03650	-0.29058	0.74485	-9.78010	8.37210
SDN	0.025	-0.10489	0.93540	0.00391	0.02359	-9.44930	7.66770
	0.500	-0.03575	0.94709	0.01650	0.03641	-9.32040	7.72590
	0.975	0.03329	0.95875	0.02906	0.04926	-9.22680	7.80300
SEN	0.025	-2.75879	0.47165	-0.18054	0.59382	-11.55850	7.75240
	0.500	-2.25625	0.50731	-0.15331	0.64599	-11.32110	7.84710
	0.975	-1.75447	0.54306	-0.12614	0.69808	-11.16840	7.99650
SLE	0.025	-1.33779	0.61168	0.61054	-0.31327	-11.29170	8.11870
	0.500	-1.07764	0.63339	0.64627	-0.27987	-10.86590	8.29600
	0.975	-0.81912	0.65215	0.68581	-0.24679	-10.57530	8.55260
SLV	0.500	-0.74849	19.36934	5.55466	-25.22193	-15.26850	9.15660
	0.975	1.12971	19.83668	5.74034	-24.57693	-15.14500	9.22540
	0.975	3.00640	20.30848	5.92853	-23.94025	-15.06220	9.29610
SOM	0.025	-1.67596	-0.19152	0.54224	0.08277	-7.46880	5.22510
	0.500	-1.33726	-0.04753	0.75781	0.28901	-7.08440	5.72940
	0.975	-1.00295	0.09663	0.97376	0.49512	-6.77070	6.35930
SSD	0.025	-1.35991	0.37854	-0.74597	0.93040	-6.34170	4.63620
	0.500	-1.04383	0.49686	-0.58018	1.08141	-6.09210	4.99120
	0.975	-0.72934	0.62216	-0.41620	1.23177	-5.82290	5.31580
SWZ	0.025	-0.34106	-0.41735	-0.39891	1.31422	-12.54870	7.52080
	0.500	0.18087	-0.30121	-0.22486	1.52621	-10.37960	8.48540
	0.975	0.70236	-0.18516	-0.05169	1.73837	-8.78780	9.79190
SYR	0.025	-0.59989	-0.49881	0.18422	0.53159	-7.45610	5.02050
	0.500	0.09756	-0.28046	0.45249	0.82652	-7.23220	5.39040
	0.975	0.79638	-0.06588	0.73633	1.11343	-7.01320	5.74250
TCD	0.025	-1.79917	0.72935	-0.20838	0.25959	-10.28210	7.52130
	0.500	-1.58077	0.79874	-0.14629	0.34608	-9.68960	7.84810
	0.975	-1.36493	0.87282	-0.08357	0.43043	-9.22180	8.25810

	Quantiles	int	gam	gbm	lasso	Nominal range	Nominal variance
TGO	0.025	-2.91840	-0.35173	-0.07560	0.91773	-10.50270	7.24220
	0.500	-2.35461	-0.16767	0.03064	1.13711	-9.98300	7.54790
	0.975	-1.79310	0.01545	0.13679	1.35693	-9.60210	7.93310
THA	0.025	-0.02422	-0.55920	-0.50064	1.42883	-4.10950	1.35890
	0.500	0.38992	-0.31497	-0.40106	1.71560	-3.69820	2.50790
	0.975	0.80436	-0.06924	-0.30014	1.99981	-3.23370	3.33300
TJK	0.025	-4.27293	0.40137	0.27343	0.17740	-12.88410	7.78650
	0.500	-3.32638	0.45500	0.30451	0.24052	-12.11780	8.04440
	0.975	-2.38134	0.50858	0.33561	0.30354	-11.70030	8.50700
TKM	0.025	-0.18366	0.01388	0.56198	-0.06420	-8.86280	7.39480
	0.500	-0.06645	0.19208	0.73069	0.07708	-8.56850	7.55250
	0.975	0.05056	0.37089	0.89902	0.21812	-8.34460	7.75730
TLS	0.025	-1.01148	-0.50773	-0.05209	1.15183	-12.37550	7.64500
	0.500	-0.71003	-0.35080	0.04987	1.30069	-10.46400	8.07000
	0.975	-0.40892	-0.19398	0.15185	1.44947	-9.60110	8.96350
TUN	0.025	-0.14715	-0.22890	-0.02710	0.47981	-6.63710	5.03050
	0.500	0.12372	-0.01071	0.26656	0.74442	-6.27200	5.50990
	0.975	0.39455	0.20719	0.55972	1.00889	-5.94070	6.03940
TZA	0.025	-2.01869	1.01390	-0.03534	-0.05731	-10.27090	7.73240
	0.500	-1.88152	1.04010	-0.01735	-0.02272	-9.95190	7.86450
	0.975	-1.74518	1.06631	0.00070	0.01166	-9.76210	8.09110
UGA	0.025	-0.50118	0.08533	0.06193	0.42375	-10.32990	8.04150
	0.500	-0.34452	0.26716	0.14489	0.58787	-9.69650	8.36400
	0.975	-0.18928	0.44854	0.22932	0.75113	-9.14790	8.73280
UZB	0.025	-0.65908	-0.17577	0.75129	0.21028	-10.64690	8.22920
	0.500	-0.53598	-0.09986	0.82172	0.27789	-10.27260	8.37400
	0.975	-0.41310	-0.02383	0.89286	0.34491	-10.02800	8.59530
VNM	0.025	-0.25743	0.46249	-0.48857	0.48961	-10.60890	7.95570
	0.500	-0.04834	0.68138	-0.42091	0.73988	-10.57240	7.99550
	0.975	0.16058	0.90067	-0.35457	0.98985	-10.52710	8.02800
YEM	0.025	-0.02265	-6.74926	-2.17438	9.33588	-13.16810	6.38070
	0.500	1.93001	-6.50329	-2.05850	9.56111	-13.15990	6.38910
	0.975	3.88118	-6.26750	-1.95497	9.81062	-13.15120	6.39880
ZAF	0.025	0.79359	0.48256	-0.06256	0.52634	-10.06930	7.29950
	0.500	0.91645	0.50433	-0.05246	0.54813	-9.98850	7.33220
	0.975	1.03920	0.52609	-0.04237	0.56990	-9.94490	7.39130
ZMB	0.025	-3.68159	0.99889	-0.58206	0.41696	-11.57580	8.10030
	0.500	-3.24240	1.05377	-0.54269	0.48880	-11.57050	8.10600
	0.975	-2.80690	1.11051	-0.50409	0.55984	-11.56290	8.11480
ZWE	0.025	-4.64457	-0.08018	-0.26152	0.97078	-9.97320	7.24090
	0.500	-4.21724	0.07736	-0.20793	1.13017	-9.96270	7.25230
	0.975	-3.79122	0.23835	-0.15431	1.28657	-9.95180	7.26670

Appendix Table 11. Fitted parameters by region for model of access to improved sanitation

Lower, median, and upper quantiles (0.025%, 0.50%, 0.975%) are displayed for the main parameters by region. The first four columns provide information on the fixed effects: the intercept (int) and the covariates (gam, gbm, and enet) corresponding to the predicted ensemble rasters. Fitted values for the spatiotemporal field are shown in the next two columns. Countries are abbreviated by their ISO3 codes.

	Quantiles	int	gam	gbm	lasso	Nominal range	Nominal variance
AFG	0.025	-0.09061	0.52591	-0.84981	0.35874	-9.99940	7.30730
	0.500	0.14463	0.88941	-0.71047	0.82116	-9.95750	7.38190
	0.975	0.37970	1.25452	-0.57275	1.28271	-9.91800	7.42490
AGO	0.025	-0.78308	0.02142	-0.49803	1.00233	-4.86420	2.79240
	0.500	-0.31919	0.16445	-0.37577	1.21127	-4.63060	3.38820
	0.975	0.14360	0.30718	-0.25355	1.42014	-4.37520	3.89110
BDI	0.025	-0.81357	1.25885	-0.93480	0.31596	-11.20430	7.75780
	0.500	-0.41070	1.40534	-0.87453	0.46924	-10.71960	7.99660
	0.975	-0.00818	1.55199	-0.81449	0.62225	-10.37250	8.33080
BEN	0.025	-1.85084	0.53774	-0.41663	0.72203	-10.86660	7.97460
	0.500	-1.54415	0.58697	-0.37692	0.78994	-10.82990	8.01300
	0.975	-1.23787	0.63627	-0.33731	0.85779	-10.79080	8.04280
BFA	0.025	-1.17790	0.81353	-0.60917	0.56340	-11.15520	8.28320
	0.500	-0.99281	0.90280	-0.55670	0.65386	-11.13180	8.30730
	0.975	-0.80791	0.99207	-0.50437	0.74426	-11.11230	8.32240
BGD	0.025	-0.05666	0.90945	-0.32258	0.06549	-10.54660	8.20590
	0.500	0.06372	1.01289	-0.24148	0.22870	-10.51850	8.23410
	0.975	0.18401	1.11646	-0.16070	0.39169	-10.49020	8.25630
BOL	0.025	0.00255	0.46559	0.03878	0.15744	-5.63970	4.50380
	0.500	0.17870	0.59345	0.09640	0.31018	-5.50820	4.72020
	0.975	0.35478	0.72152	0.15393	0.46253	-5.37680	4.93850
BRA	0.025	-0.05939	0.34169	-0.16982	0.48495	-3.60530	2.01400
	0.500	0.16159	0.46533	-0.09499	0.62966	-3.46770	2.50690
	0.975	0.38253	0.58891	-0.02018	0.77415	-3.32000	2.88770
BTN	0.025	-2.08135	0.47364	-1.04128	1.26050	-13.57740	7.26940
	0.500	-0.14965	0.53624	-0.92356	1.38763	-12.91280	7.70490
	0.975	1.78051	0.59915	-0.80693	1.51467	-12.37230	8.23720
BWA	0.025	-1.13503	-0.15287	-0.82821	1.29124	-9.13570	6.59800
	0.500	-0.89336	0.12982	-0.67057	1.54087	-8.70150	6.98860
	0.975	-0.65203	0.41171	-0.51341	1.79115	-8.34740	7.47640
CAF	0.025	-0.90613	-2.28702	-1.59677	4.37726	-10.13680	7.51240
	0.500	-0.72450	-2.13174	-1.45577	4.58760	-9.86410	7.67240
	0.975	-0.54304	-1.97778	-1.31494	4.79883	-9.65090	7.87210
CHN	0.025	-0.26971	-0.25343	0.51892	0.24122	-3.70000	2.69370
	0.500	-0.09509	-0.07235	0.64159	0.43073	-3.47700	3.15680
	0.975	0.07948	0.10842	0.76464	0.61999	-3.23260	3.56220
	0.025	-0.74688	0.39569	-0.82857	0.74907	-11.52720	7.49210

	Quantiles	int	gam	gbm	lasso	Nominal range	Nominal variance
CIV	0.500	-0.42851	0.65191	-0.65936	1.00788	-10.41540	7.83320
	0.975	-0.11046	0.90754	-0.49132	1.26715	-9.87280	8.47760
CMR	0.025	-0.57884	0.53591	-1.47116	1.74275	-10.27060	7.47010
	0.500	-0.39628	0.60232	-1.41529	1.81293	-10.02240	7.58180
	0.975	-0.21387	0.66876	-1.35968	1.88317	-9.85540	7.74390
COD	0.025	-0.73211	-0.94412	-1.62814	3.07235	-10.28910	7.33080
	0.500	-0.56306	-0.74128	-1.50651	3.24783	-10.05340	7.44360
	0.975	-0.39416	-0.53880	-1.38550	3.42391	-9.89390	7.60890
COG	0.025	-0.69072	0.06731	-0.80562	1.44288	-12.47450	8.03950
	0.500	-0.50561	0.11673	-0.69739	1.58065	-11.10640	8.47800
	0.975	-0.32068	0.16615	-0.58922	1.71824	-10.31770	9.16970
COL	0.025	-0.45154	0.89185	-0.14399	0.12825	-5.88560	4.32680
	0.500	-0.18864	0.93146	-0.11542	0.18393	-5.71160	4.59120
	0.975	0.07378	0.97112	-0.08706	0.23965	-5.52380	4.83360
COM	0.025	-2.14979	-0.39982	0.01298	1.07789	-11.36110	7.00240
	0.500	-0.64884	-0.27104	0.08153	1.18951	-10.68820	7.55350
	0.975	0.85075	-0.14301	0.15019	1.30145	-10.10810	8.17990
CRI	0.025	0.73004	-0.21333	-1.42698	2.11327	-11.84230	7.81220
	0.500	1.26775	-0.02229	-1.31772	2.33673	-11.03260	8.16760
	0.975	1.80617	0.19188	-1.21075	2.54635	-10.48650	8.68900
DOM	0.025	-0.97061	0.33637	-0.49337	0.67662	-14.90980	8.10200
	0.500	0.42148	0.44252	-0.30970	0.86730	-13.19630	8.77790
	0.975	1.81243	0.54840	-0.12630	1.05797	-12.00110	9.73820
DZA	0.025	0.06568	-0.13880	-0.11133	0.61616	-4.66520	3.18750
	0.500	0.37540	0.10040	0.04172	0.85746	-4.41690	3.67350
	0.975	0.68725	0.34102	0.19463	1.09785	-4.15960	4.10700
ECU	0.025	-1.83969	38.52120	34.07391	-76.54137	-9.78400	2.65330
	0.500	0.10047	39.83641	34.41889	-74.37365	-9.66270	2.99880
	0.975	2.03903	41.93973	35.18941	-73.33673	-9.37920	3.15430
EGY	0.025	-0.13128	0.18264	0.24993	0.50038	-9.05400	3.52280
	0.500	-0.07510	0.20513	0.26884	0.52601	-9.05220	3.60820
	0.975	-0.01897	0.22762	0.28774	0.55161	-9.04890	3.69380
ERI	0.025	-0.25292	-0.12259	-0.07273	0.51154	-5.44960	3.96000
	0.500	-0.00797	-0.01232	0.20992	0.80549	-4.98520	4.73040
	0.975	0.23662	0.09101	0.49274	1.09899	-4.50110	5.45260
ETH	0.025	-0.55014	1.11406	0.26573	-0.48089	-10.33120	7.76140

	Quantiles	int	gam	gbm	lasso	Nominal range	Nominal variance
	0.500	-0.46648	1.14577	0.29511	-0.44089	-10.21070	7.80460
	0.975	-0.38292	1.17753	0.32447	-0.40097	-10.13790	7.87270
GAB	0.025	-1.77898	-1.21944	-0.93699	2.74295	-10.24540	7.18320
	0.500	-1.42171	-1.13222	-0.80105	2.93333	-9.80720	7.44820
	0.975	-1.06488	-1.04532	-0.66659	3.12505	-9.48860	7.80060
GHA	0.025	-0.59655	1.20441	-1.35629	0.78792	-10.42100	7.75580
	0.500	-0.35142	1.33034	-1.27116	0.94142	-10.40680	7.76510
	0.975	-0.10652	1.45501	-1.18639	1.09506	-10.39710	7.77440
GIN	0.025	-1.58588	-0.47430	-1.09307	2.14104	-11.24260	7.71870
	0.500	-1.25381	-0.31694	-1.00928	2.32623	-10.68030	7.95360
	0.975	-0.92213	-0.16011	-0.92620	2.51231	-10.34460	8.34400
GNB	0.025	-0.19787	-0.13957	-0.43524	0.09503	-8.89340	6.36580
	0.500	0.00942	0.30382	0.09231	0.60389	-7.73200	7.40770
	0.975	0.21655	0.74684	0.61942	1.11235	-6.55310	8.42430
GTM	0.025	-1.88459	0.52186	-1.07811	1.23771	-9.05250	1.45540
	0.500	0.03195	0.61546	-0.98775	1.37219	-8.96500	2.36460
	0.975	1.94695	0.70931	-0.89783	1.50667	-8.86380	2.88060
GUY	0.025	0.42489	-0.00859	-0.25763	0.52006	-11.31270	7.48890
	0.500	0.84029	0.30594	-0.13062	0.82343	-10.56530	7.96850
	0.975	1.25574	0.62162	-0.00213	1.12592	-9.86200	8.47270
HND	0.025	0.06446	0.82745	-0.66540	0.20991	-9.26820	7.57950
	0.500	0.16386	1.05557	-0.49216	0.43663	-9.04600	7.69200
	0.975	0.26318	1.28407	-0.31920	0.66277	-8.89900	7.85840
HTI	0.025	-0.94293	-0.08007	-1.00914	1.80058	-10.65820	7.89620
	0.500	-0.57877	0.02464	-0.96076	1.93605	-10.61320	7.94350
	0.975	-0.21492	0.12933	-0.91259	2.07148	-10.56770	7.98010
IDN	0.025	-0.42053	-0.23508	-0.29964	1.23254	-9.39470	7.13990
	0.500	-0.34572	-0.12467	-0.23511	1.35974	-9.28950	7.30160
	0.975	-0.27099	-0.01433	-0.17067	1.48687	-9.10650	7.39700
IND	0.025	-1.88424	0.26306	-0.68322	1.35179	-9.06280	2.97500
	0.500	-0.34138	0.28411	-0.66610	1.38200	-9.04830	3.05740
	0.975	1.20028	0.30516	-0.64901	1.41217	-9.03700	3.11870
IRQ	0.025	0.22872	0.57141	-0.00856	0.06302	-9.42510	7.31090
	0.500	0.37831	0.70799	0.06453	0.22800	-9.03560	7.54930
	0.975	0.52796	0.84688	0.13874	0.38798	-8.73880	7.86130
JOR	0.025	1.29247	0.09264	-0.70926	1.15031	-10.84030	7.41380
	0.500	1.79881	0.26624	-0.59904	1.33218	-10.32470	7.76790
	0.975	2.30716	0.44170	-0.48993	1.51397	-9.86140	8.15680
KEN	0.025	-1.47038	0.08132	-1.03501	1.60954	-10.52420	7.54310
	0.500	-1.18652	0.21142	-0.96140	1.74993	-10.47900	7.58230
	0.975	-0.90296	0.34143	-0.88793	1.89025	-10.41040	7.62790

	Quantiles	int	gam	gbm	lasso	Nominal range	Nominal variance
KGZ	0.025	3.70194	-0.80268	-0.84524	1.95517	-11.03490	6.93150
	0.500	4.31813	-0.55023	-0.68700	2.23697	-10.40520	7.64340
	0.975	4.93971	-0.29839	-0.53002	2.52071	-9.37690	8.07400
KHM	0.025	-2.49979	0.59104	-1.03536	1.13218	-10.46430	7.69380
	0.500	-2.27087	0.69291	-0.95061	1.25767	-10.29140	7.78170
	0.975	-2.04248	0.79510	-0.86647	1.38312	-10.16650	7.89760
LAO	0.025	-1.87099	0.68656	-1.46545	1.13425	-11.62330	8.01370
	0.500	-1.49276	0.89445	-1.29072	1.39703	-11.56480	8.29320
	0.975	-1.11506	1.10635	-1.12025	1.65821	-11.45650	8.42330
LBR	0.025	-2.28242	0.68666	-1.13111	1.07382	-11.29220	8.03120
	0.500	-1.82898	0.80064	-1.03317	1.23264	-11.23930	8.11370
	0.975	-1.37626	0.91479	-0.93603	1.39140	-11.17960	8.16470
LBY	0.025	-1.81762	0.00281	-0.06277	0.62005	-6.51610	4.15200
	0.500	-1.19530	0.09009	0.10874	0.80051	-6.32930	4.49120
	0.975	-0.57588	0.17869	0.28079	0.98003	-6.12720	4.79390
LKA	0.025	-0.45357	0.06195	-0.78327	1.22881	-9.60110	6.47390
	0.500	-0.11593	0.21547	-0.66893	1.45327	-8.70920	7.21540
	0.975	0.22151	0.36408	-0.55438	1.68211	-7.93600	8.06950
LSO	0.025	-1.01050	0.01421	-0.82179	1.27902	-10.34460	7.86810
	0.500	-0.72640	0.21503	-0.71073	1.49563	-10.24390	7.94110
	0.975	-0.44259	0.41620	-0.59984	1.71184	-10.16440	7.99660
MAR	0.025	-3.20643	0.03657	-0.05828	0.74342	-8.42360	5.74570
	0.500	-2.60036	0.12989	0.00659	0.85948	-8.16400	6.07280
	0.975	-2.00021	0.24836	0.07021	0.95746	-7.87930	6.35730
MDG	0.025	-2.40975	0.47499	-1.22384	1.47488	-9.92330	7.43790
	0.500	-2.22615	0.53998	-1.13874	1.59867	-9.73970	7.54110
	0.975	-2.04300	0.60532	-1.05450	1.72277	-9.60260	7.67380
MEX	0.025	-1.11949	0.65585	-0.64139	0.67795	-8.91020	6.99230
	0.500	-1.03008	0.77914	-0.57565	0.79653	-8.73090	7.07080
	0.975	-0.94080	0.90249	-0.51013	0.91500	-8.63200	7.20990
MLI	0.025	-0.85019	-0.46870	-0.53680	1.83479	-11.35360	7.92880
	0.500	-0.67818	-0.40535	-0.50213	1.90750	-11.03960	8.03990
	0.975	-0.50634	-0.34207	-0.46765	1.98029	-10.84880	8.21910
MMR	0.025	0.04377	0.62509	-0.33765	0.44317	-9.38240	7.61550
	0.500	0.13377	0.68369	-0.24166	0.55796	-9.35100	7.64990
	0.975	0.22370	0.74216	-0.14575	0.67270	-9.31820	7.67660
MNG	0.025	-0.02422	1.03108	-0.58774	0.24960	-9.59780	7.59960
	0.500	0.04387	1.15839	-0.55157	0.39326	-9.36890	7.72500
	0.975	0.11192	1.28576	-0.51577	0.53678	-9.19420	7.89050
MOZ	0.025	-0.36639	0.00039	0.31589	0.29490	-9.15630	7.42140
	0.500	-0.28760	0.15067	0.42085	0.42842	-8.95600	7.51340
	0.975	-0.20888	0.30084	0.52587	0.56170	-8.83830	7.67300
	0.025	-0.94123	0.23581	-0.68105	1.22689	-9.90080	6.97940

	Quantiles	int	gam	gbm	lasso	Nominal range	Nominal variance
MRT	0.500	-0.78330	0.31126	-0.63552	1.32418	-9.31410	7.30020
	0.975	-0.62564	0.38670	-0.59026	1.42161	-8.87430	7.72370
MWI	0.025	-0.01614	0.49824	0.17629	0.09104	-13.11610	8.20980
	0.500	0.00122	0.59511	0.21644	0.18841	-9.89500	9.08080
	0.975	0.01857	0.69191	0.25662	0.28566	-8.31060	10.91700
NAM	0.025	-1.24989	0.44585	-1.40520	1.61537	-10.37860	7.67240
	0.500	-1.10039	0.53697	-1.29107	1.75430	-10.07200	7.87320
	0.975	-0.95115	0.62755	-1.17714	1.89333	-9.77810	8.08120
NER	0.025	-0.05204	1.24879	0.36960	-0.75358	-9.43020	7.39120
	0.500	0.04234	1.29462	0.40642	-0.70107	-9.22600	7.50140
	0.975	0.13653	1.34061	0.44337	-0.64894	-9.06600	7.64230
NGA	0.025	-0.50438	0.69854	0.19436	-0.06924	-10.49440	7.74600
	0.500	-0.38026	0.74888	0.24400	0.00709	-10.48560	7.75600
	0.975	-0.25625	0.79922	0.29366	0.08330	-10.47430	7.76490
NIC	0.025	-0.16087	-0.99638	-0.90285	2.39440	-8.65470	6.55270
	0.500	-0.00446	-0.81886	-0.81358	2.63244	-8.09230	6.99950
	0.975	0.15182	-0.64204	-0.72452	2.87098	-7.61570	7.53250
NPL	0.025	-0.36757	0.74088	-0.71173	0.57258	-11.10780	8.02960
	0.500	-0.04822	0.87643	-0.63972	0.76344	-11.06870	8.06660
	0.975	0.27090	1.01272	-0.56827	0.95361	-11.01820	8.10340
PAK	0.025	-0.29224	-0.43767	-0.15837	0.71823	-6.81520	4.92440
	0.500	0.02042	-0.12249	0.06213	1.06024	-6.69400	5.13040
	0.975	0.33285	0.19221	0.28264	1.40206	-6.56180	5.32610
PAN	0.025	-0.25020	-0.15965	-0.40475	0.52201	-6.89210	4.64980
	0.500	0.23430	0.04663	0.02948	0.92391	-6.38150	5.29520
	0.975	0.71847	0.25271	0.46309	1.32583	-5.91400	5.99220
PER	0.025	-0.13081	0.14394	-0.60187	1.17929	-9.97910	7.70650
	0.500	-0.05921	0.24251	-0.55302	1.31046	-9.87020	7.75450
	0.975	0.01234	0.34104	-0.50425	1.44153	-9.80260	7.82990
PHL	0.025	-0.24004	0.24798	-0.70598	0.56244	-10.35790	7.27410
	0.500	0.13918	0.62798	-0.55506	0.92885	-10.30000	7.31970
	0.975	0.51806	1.00988	-0.40890	1.29364	-10.25040	7.38090
PNG	0.025	-0.37426	0.77730	-0.26202	-0.47117	-9.87150	6.71670
	0.500	-0.14702	1.21605	-0.16929	-0.04617	-9.20010	7.35510
	0.975	0.07972	1.65582	-0.07809	0.37734	-8.45200	7.93750
PRY	0.025	0.05070	0.51334	-0.32003	0.63065	-9.56290	7.76820
	0.500	0.15047	0.57086	-0.28234	0.71150	-9.50240	7.83960

	Quantiles	int	gam	gbm	lasso	Nominal range	Nominal variance
	0.975	0.25025	0.62833	-0.24471	0.79230	-9.42800	7.89230
RWA	0.025	-0.00266	0.73695	-0.32199	0.17413	-12.51100	8.13820
	0.500	0.03285	0.83869	-0.20852	0.36990	-9.68530	8.89440
	0.975	0.06833	0.94076	-0.09561	0.56550	-8.21540	10.27940
SDN	0.025	0.09871	0.85377	-0.53940	0.49170	-9.36850	7.43570
	0.500	0.16103	0.88920	-0.46215	0.57293	-9.35040	7.45500
	0.975	0.22331	0.92458	-0.38474	0.65395	-9.33260	7.46900
SEN	0.025	-0.31305	0.04626	-0.61997	1.31708	-10.20520	7.62800
	0.500	-0.09826	0.15065	-0.57843	1.42781	-10.16370	7.67520
	0.975	0.11635	0.25515	-0.53737	1.53847	-10.11820	7.71310
SLE	0.025	-0.88065	0.73781	-0.57788	0.59938	-11.05550	7.88370
	0.500	-0.60674	0.80825	-0.51730	0.70915	-10.67330	8.04690
	0.975	-0.33309	0.87882	-0.45699	0.81875	-10.43050	8.30550
SLV	0.500	-0.38607	0.08475	1.38582	-1.86015	-10.51290	7.43940
	0.975	-0.09671	0.26214	1.99563	-1.25725	-9.57610	7.93870
	0.975	0.19246	0.43986	2.60510	-0.65650	-8.87120	8.58940
SOM	0.025	-0.50288	-0.25527	0.30615	0.47941	-6.72060	5.22840
	0.500	-0.36002	-0.08186	0.43135	0.65050	-6.44520	5.56230
	0.975	-0.21735	0.09143	0.55650	0.82135	-6.17380	5.89960
SSD	0.025	-0.33724	0.05158	-0.32309	0.72659	-5.32160	3.75470
	0.500	0.02217	0.24986	-0.18150	0.93156	-5.13440	4.16220
	0.975	0.38119	0.44869	-0.04006	1.13594	-4.93110	4.51310
SWZ	0.025	-0.36000	0.20314	-0.33950	0.53283	-9.71810	7.70260
	0.500	0.01070	0.39350	-0.22433	0.83091	-9.28860	7.98080
	0.975	0.38112	0.58389	-0.10946	1.12870	-8.91920	8.22240
SYR	0.025	-0.18291	-0.14692	-0.20393	0.48283	-7.80450	5.24050
	0.500	0.32337	0.14653	0.01999	0.83283	-7.34800	5.77460
	0.975	0.82974	0.44276	0.24420	1.18018	-6.93430	6.35100
TCD	0.025	-1.48669	0.44919	-0.83951	1.06057	-10.71520	7.77930
	0.500	-1.34702	0.55851	-0.74755	1.18907	-10.22400	7.97130
	0.975	-1.20788	0.66762	-0.65594	1.31785	-9.93380	8.30690
TGO	0.025	-1.06293	0.69882	-0.75506	0.17278	-10.40410	7.70840
	0.500	-0.55442	1.06312	-0.59473	0.53222	-10.27830	7.79600
	0.975	-0.04649	1.42633	-0.43546	0.89201	-10.04360	7.91250
THA	0.025	-0.39972	0.26992	-0.29386	0.18965	-4.04600	0.71930
	0.500	-0.02086	0.57625	-0.15102	0.57463	-3.36770	2.44010
	0.975	0.35889	0.88682	-0.01031	0.95741	-2.54940	3.75880
TJK	0.025	0.71187	1.19743	-0.18276	-0.35543	-10.75700	7.29570

	Quantiles	int	gam	gbm	lasso	Nominal range	Nominal variance
	0.500	1.08235	1.32463	-0.12622	-0.19833	-10.18960	7.58410
	0.975	1.45321	1.45393	-0.07103	-0.04231	-9.77680	7.97720
TKM	0.025	-0.52764	-0.11645	-0.25747	-0.09775	-4.42550	1.42920
	0.500	-0.00416	0.31477	0.19377	0.45139	-3.79780	2.78690
	0.975	0.52398	0.82625	0.66580	0.98094	-3.05260	3.82770
TLS	0.025	-0.52316	0.48966	-0.40152	0.60257	-10.43850	8.10260
	0.500	-0.26290	0.58581	-0.33471	0.74900	-10.37000	8.17770
	0.975	-0.00287	0.68234	-0.26811	0.89492	-10.28310	8.23790
TUN	0.025	-0.02099	0.04212	-0.24334	0.62586	-5.98180	3.36440
	0.500	0.92052	0.21714	-0.06947	0.84983	-5.66140	4.16870
	0.975	1.86717	0.39224	0.11809	1.06541	-5.30880	4.81340
TZA	0.025	-1.26100	-0.71024	-1.20303	2.64446	-10.64370	7.91960
	0.500	-1.15410	-0.66615	-1.09081	2.75695	-10.42600	8.01060
	0.975	-1.04733	-0.62212	-0.97874	2.86947	-10.28560	8.15340
UGA	0.025	0.12029	0.99243	-0.44392	0.18298	-10.84580	8.17970
	0.500	0.27506	1.07987	-0.38252	0.30258	-10.81550	8.20890
	0.975	0.42973	1.16730	-0.32126	0.42214	-10.77680	8.23790
UZB	0.025	0.18993	1.19785	-0.10308	-0.39293	-9.72780	7.89260
	0.500	0.34007	1.30077	-0.04456	-0.25595	-9.68830	7.93390
	0.975	0.49041	1.40579	0.01278	-0.12056	-9.64770	7.96560
VNM	0.025	-0.28588	1.48430	-0.67860	-0.81169	-9.55360	6.68780
	0.500	0.06385	1.87076	-0.46457	-0.40644	-9.32950	6.89380
	0.975	0.41330	2.25970	-0.25149	-0.00278	-9.16000	7.18150
YEM	0.025	-1.01845	0.47352	-1.29004	1.28084	-12.56310	8.60310
	0.500	-0.58469	0.69199	-1.18900	1.49684	-12.50090	8.66070
	0.975	-0.15133	0.91164	-1.08844	1.71214	-12.41170	8.71090
ZAF	0.025	-0.30715	0.62261	-0.59211	0.91920	-13.38140	8.75830
	0.500	-0.24745	0.63754	-0.58002	0.94248	-12.38310	9.03100
	0.975	-0.18781	0.65246	-0.56795	0.96574	-11.85020	9.54910
ZMB	0.025	-0.19950	1.54561	-0.17898	-0.61335	-9.78350	7.48960
	0.500	-0.11064	1.63916	-0.12834	-0.51076	-9.56310	7.58210
	0.975	-0.02186	1.73276	-0.07782	-0.40837	-9.42290	7.72860

	Quantiles	int	gam	gbm	lasso	Nominal range	Nominal variance
ZWE	0.025	-0.17090	0.85726	-0.25345	-0.30875	-9.51120	7.71660
	0.500	-0.05942	1.12800	-0.07089	-0.05698	-9.47520	7.75500
	0.975	0.05196	1.39875	0.11129	0.19444	-9.44140	7.78280

Appendix Table 12. Fitted parameters by region for model of access to unimproved sanitation

Lower, median, and upper quantiles (0.025%, 0.50%, 0.975%) are displayed for the main parameters by region. The first four columns provide information on the fixed effects: the intercept (int) and the covariates (gam, gbm, and enet) corresponding to the predicted ensemble rasters. Fitted values for the spatiotemporal field are shown in the next two columns. Countries are abbreviated by their ISO3 codes.

	Quantiles	int	gam	gbm	lasso	Nominal range	Nominal variance
AFG	0.025	-0.86855	-0.02795	-1.27232	1.42544	-10.65190	7.21790
	0.500	-0.54961	0.22370	-1.04572	1.82200	-10.18920	7.40640
	0.975	-0.23109	0.47630	-0.82145	2.21873	-9.92460	7.73900
AGO	0.025	-0.47620	0.03455	-0.44104	1.07617	-5.80120	3.86020
	0.500	-0.03528	0.14556	-0.35236	1.20682	-5.62300	4.15150
	0.975	0.40526	0.25682	-0.26381	1.33712	-5.44100	4.43340
BDI	0.025	1.27697	0.37064	-0.42069	0.67669	-10.96670	8.08120
	0.500	1.70863	0.53363	-0.36547	0.83178	-10.94810	8.09810
	0.975	2.14038	0.69789	-0.31066	0.98587	-10.92300	8.11680
BEN	0.025	-2.53964	-0.10535	-0.88955	1.83659	-11.54300	7.58070
	0.500	-2.14972	-0.05021	-0.84395	1.89416	-10.73250	7.81440
	0.975	-1.76033	0.00486	-0.79852	1.95187	-10.35700	8.28960
BFA	0.025	-1.28117	0.65640	-0.34126	0.42017	-10.19270	7.59720
	0.500	-1.09790	0.74922	-0.27550	0.52629	-9.98480	7.69090
	0.975	-0.91490	0.84228	-0.20990	0.63215	-9.85400	7.83700
BGD	0.025	1.13390	0.22970	-1.27826	1.67555	-11.06880	8.11600
	0.500	1.37012	0.36388	-1.18952	1.82578	-11.03720	8.14800
	0.975	1.60620	0.49775	-1.10121	1.97622	-11.00190	8.17360
BOL	0.025	-0.08026	0.55052	0.11935	-0.27345	-4.22810	2.43610
	0.500	0.00506	0.77132	0.29342	-0.06486	-2.11920	4.14550
	0.975	0.08966	0.99454	0.46833	0.13987	1.46910	5.84800
BRA	0.025	-0.18516	-0.20973	-0.02799	0.92630	-4.36980	2.42190
	0.500	0.12153	-0.07491	0.02601	1.04901	-4.24680	2.89480
	0.975	0.42789	0.05936	0.07984	1.17203	-4.09920	3.23300
BTN	0.025	-0.20881	-0.42262	-1.05880	1.95687	-12.30800	8.52420
	0.500	0.65517	-0.25680	-0.91849	2.17512	-12.06140	8.74060

	Quantiles	int	gam	gbm	lasso	Nominal range	Nominal variance
	0.975	1.51864	-0.09318	-0.77880	2.39623	-11.70040	8.95740
BWA	0.025	-0.55607	0.59201	-0.99403	0.91362	-11.45720	8.13710
	0.500	-0.17120	0.76519	-0.86113	1.09593	-11.39200	8.31050
	0.975	0.21327	0.93850	-0.72891	1.27820	-11.32200	8.39700
CAF	0.025	-0.07097	0.39931	-0.20936	0.35816	-8.75510	7.12040
	0.500	-0.00048	0.56788	-0.09556	0.52759	-8.38570	7.34010
	0.975	0.06997	0.73634	0.01825	0.69682	-8.12890	7.64730
CHN	0.025	-0.90492	0.33179	0.31599	0.33189	-3.92700	2.75000
	0.500	-0.86647	0.33859	0.32272	0.33869	-3.79020	2.97090
	0.975	-0.82805	0.34539	0.32943	0.34549	-3.65040	3.17410
CIV	0.025	-1.63869	-1.40473	-1.47603	3.23802	-10.10160	7.30990
	0.500	-1.22793	-1.15344	-1.33145	3.48560	-10.03290	7.36730
	0.975	-0.81757	-0.90374	-1.18877	3.73480	-9.97210	7.43860
CMR	0.025	0.13841	2.53331	-0.21697	-1.52458	-10.80910	7.60540
	0.500	0.38420	2.60024	-0.16117	-1.43892	-10.55110	7.71590
	0.975	0.63012	2.66770	-0.10560	-1.35394	-10.37410	7.87420
COD	0.025	-0.31516	1.36041	-1.43284	0.69951	-9.58900	7.40870
	0.500	-0.20302	1.50728	-1.36677	0.85944	-9.55620	7.43780
	0.975	-0.09095	1.65425	-1.30135	1.01943	-9.50520	7.47940
COG	0.025	0.01733	0.15982	-0.53145	1.01267	-8.53390	6.57840
	0.500	0.11742	0.25356	-0.41206	1.15840	-8.05010	6.98700
	0.975	0.21747	0.34665	-0.29237	1.30419	-7.68540	7.53720
COL	0.025	-0.05710	0.11925	-0.18398	0.61901	-7.45810	5.68930
	0.500	0.06647	0.29155	-0.09982	0.80815	-7.20250	5.91780
	0.975	0.18982	0.46368	-0.01568	0.99722	-6.95850	6.15900
COM	0.025	-0.79508	-0.27087	-0.24076	1.17153	-9.86390	6.03200
	0.500	0.76946	-0.12640	-0.17907	1.30513	-9.41460	6.69800
	0.975	2.33320	0.01795	-0.11760	1.43944	-8.98500	7.39300
CRI	0.025	-1.37181	-0.13968	-0.22896	0.88376	-10.58270	7.59410
	0.500	-0.57173	0.07203	-0.16627	1.09402	-10.39490	7.73830
	0.975	0.21863	0.28216	-0.10346	1.30570	-10.12410	7.94720
DOM	0.025	-1.66379	0.57747	-0.68505	0.69264	-12.95930	7.27750
	0.500	-0.00118	0.70429	-0.54323	0.83890	-11.97960	7.85720
	0.975	1.66010	0.83171	-0.40177	0.98481	-11.22940	8.60980
DZA	0.025	-0.03976	0.03567	-0.09480	0.39094	-4.93610	3.39960
	0.500	0.28095	0.27637	0.07457	0.64877	-4.56360	3.91880
	0.975	0.60248	0.51739	0.24415	0.90591	-4.17790	4.40480
ECU	0.025	-1.46772	5.35357	-1.02373	-5.53429	-7.31300	3.04330
	0.500	0.42056	5.48469	-0.95276	-5.22526	-7.30790	3.16300

	Quantiles	int	gam	gbm	lasso	Nominal range	Nominal variance
	0.975	2.30692	5.79833	-0.91056	-5.09421	-7.29780	3.28290
EGY	0.025	-2.21873	0.36173	-0.11248	0.53738	-11.06850	7.72520
	0.500	-1.77108	0.44573	-0.07759	0.63196	-11.01910	7.81050
	0.975	-1.32485	0.52973	-0.04300	0.72646	-10.96240	7.86060
ERI	0.025	-0.63881	-0.69004	0.45349	0.46940	-6.52420	4.53440
	0.500	-0.07974	-0.50016	0.70845	0.79182	-6.23350	4.98220
	0.975	0.47884	-0.31048	0.96323	1.11394	-5.94490	5.42260
ETH	0.025	-0.36188	0.80145	0.19226	-0.18473	-10.96270	8.04590
	0.500	-0.26258	0.87445	0.21786	-0.09229	-10.94600	8.06270
	0.975	-0.16338	0.94747	0.24342	0.00000	-10.92960	8.07450
GAB	0.025	1.50038	0.40625	-0.35750	0.59460	-11.17120	7.46210
	0.500	2.06135	0.50023	-0.26457	0.76412	-10.62870	7.71950
	0.975	2.62338	0.59436	-0.17218	0.93397	-10.25520	8.12250
GHA	0.025	-1.12260	2.90377	-1.45947	-0.88771	-11.62550	7.62490
	0.500	-0.76859	3.05872	-1.36703	-0.69163	-10.88980	7.84920
	0.975	-0.41499	3.21524	-1.27530	-0.49672	-10.53570	8.29730
GIN	0.025	0.42024	0.11679	-1.05305	1.55188	-10.74390	7.77770
	0.500	0.80878	0.21445	-0.92479	1.71065	-10.69250	7.83260
	0.975	1.19708	0.31189	-0.79774	1.86994	-10.63820	7.87320
GNB	0.025	-0.05945	-0.22665	-0.95076	0.31032	-8.42310	6.55780
	0.500	0.35825	0.32449	-0.30986	0.98528	-8.06520	6.90630
	0.975	0.77562	0.87519	0.33052	1.65971	-7.75360	7.21180
GTM	0.025	-1.77743	1.35782	-0.87957	0.12367	-10.69250	7.99100
	0.500	-1.48336	1.48517	-0.78681	0.30213	-10.64250	8.04480
	0.975	-1.19026	1.61681	-0.69625	0.47747	-10.57900	8.08960
GUY	0.025	-1.07106	1.11063	-0.42617	-0.07421	-11.30000	7.66030
	0.500	-0.53028	1.25200	-0.35122	0.09946	-10.77150	7.89310
	0.975	0.00975	1.39501	-0.27816	0.27264	-10.44510	8.26570
HND	0.025	-0.11500	0.78876	-1.00449	0.69767	-9.93050	7.61770
	0.500	0.05680	0.99126	-0.90394	0.91292	-9.69790	7.72910
	0.975	0.22843	1.19569	-0.80527	1.12728	-9.54540	7.89680
HTI	0.025	-0.45238	0.98302	-1.46199	1.19800	-11.65030	8.09410
	0.500	-0.12413	1.08254	-1.39260	1.31013	-11.13570	8.29290
	0.975	0.20385	1.18221	-1.32355	1.42212	-10.81110	8.60880
IDN	0.025	0.96050	0.46782	-0.82453	1.17077	-9.44300	0.57360
	0.500	2.39982	0.52428	-0.78149	1.25716	-9.41970	0.75450
	0.975	3.83864	0.58072	-0.73853	1.34352	-9.38880	0.92830

	Quantiles	int	gam	gbm	lasso	Nominal range	Nominal variance
IND	0.025	-2.55494	0.82293	-0.65101	0.74269	-8.89850	2.92080
	0.500	-1.02151	0.84902	-0.62931	0.78031	-8.86280	3.03040
	0.975	0.51066	0.87521	-0.60764	0.81778	-8.79000	3.09910
IRQ	0.025	0.03296	-0.67279	-0.09666	1.17304	-7.59950	5.34360
	0.500	0.42689	-0.44251	0.03039	1.41644	-7.31180	5.65670
	0.975	0.82158	-0.21244	0.14704	1.66051	-7.05260	5.99810
JOR	0.025	-2.93284	0.29083	-0.04472	0.46600	-12.53130	8.04750
	0.500	-2.09698	0.42629	-0.03016	0.60355	-11.96830	8.39030
	0.975	-1.26831	0.56302	-0.01502	0.73969	-11.39940	8.72580
KEN	0.025	-1.95209	1.14409	-0.42491	0.06095	-8.99940	3.70170
	0.500	-0.08517	1.23209	-0.38541	0.15344	-8.99840	3.70270
	0.975	1.78024	1.31986	-0.34601	0.24589	-8.99740	3.70370
KGZ	0.025	-1.00043	-0.67837	-0.21372	0.84381	-10.98950	6.67030
	0.500	0.40685	-0.42221	0.20310	1.22185	-10.42190	7.10390
	0.975	1.81254	-0.16496	0.59866	1.61315	-9.97590	7.63930
KHM	0.025	-2.98227	1.21422	-1.43527	1.03114	-11.35860	7.87140
	0.500	-2.67535	1.26311	-1.37658	1.11347	-11.04200	8.02210
	0.975	-2.36912	1.31214	-1.31835	1.19586	-10.81630	8.23410
LAO	0.025	-2.57479	0.06749	-1.00869	0.97757	-10.39720	7.09810
	0.500	-2.16276	0.48096	-0.79875	1.31863	-9.91970	7.37860
	0.975	-1.75205	0.89605	-0.59190	1.65864	-9.59980	7.79370
LBR	0.025	-2.49700	0.13533	-0.87534	1.26602	-10.89730	7.45650
	0.500	-2.01634	0.33066	-0.76972	1.43937	-10.49480	7.66610
	0.975	-1.53652	0.52648	-0.66489	1.61207	-10.21400	7.96250
LBY	0.025	-0.18913	-17.53204	-5.54839	-11.68122	-5.68960	1.29860
	0.500	0.01072	-1.31047	0.31362	2.05214	-2.50270	5.49570
	0.975	0.22489	13.90581	6.12403	16.75251	2.08100	10.29320
LKA	0.025	-1.16121	-0.84894	-0.34177	1.33445	-9.10080	5.69350
	0.500	-0.50437	-0.51176	-0.17188	1.68117	-8.23940	6.41660
	0.975	0.15271	-0.17017	0.00489	2.02124	-7.63330	7.46640
LSO	0.025	-1.64089	-0.36162	-0.55984	1.46690	-10.35660	7.59970
	0.500	-1.30913	-0.20875	-0.45334	1.66198	-10.02190	7.77220
	0.975	-0.97777	-0.05586	-0.34746	1.85725	-9.78330	8.01090
MAR	0.025	-0.28490	0.18369	0.06671	0.52210	-9.16330	3.95520
	0.500	-0.17481	0.26906	0.12050	0.61045	-9.15400	3.97100
	0.975	-0.06481	0.35436	0.17426	0.69869	-9.13810	3.98100
MDG	0.025	-1.65851	0.47156	-1.14418	1.33162	-10.11950	6.99070
	0.500	-1.36011	0.58490	-1.04454	1.45964	-9.78700	7.14710
	0.975	-1.06203	0.69822	-0.94540	1.58776	-9.60440	7.41870
	0.025	0.03672	0.38567	-0.17308	0.59416	-7.85510	5.52170

	Quantiles	int	gam	gbm	lasso	Nominal range	Nominal variance
MEX	0.500	0.33293	0.46223	-0.13627	0.67402	-7.84120	5.53350
	0.975	0.62890	0.53874	-0.09952	0.75382	-7.82840	5.55150
MLI	0.025	0.55267	-2.89728	-0.07179	3.57273	-10.69770	7.32190
	0.500	0.86140	-2.75332	0.00198	3.75128	-10.50660	7.40410
	0.975	1.16992	-2.60989	0.07586	3.92992	-10.38440	7.53180
MMR	0.025	0.20935	1.16030	-0.35361	-0.10033	-9.51210	7.30020
	0.500	0.33458	1.23247	-0.25339	0.02080	-9.29140	7.41640
	0.975	0.45973	1.30482	-0.15308	0.14160	-9.15000	7.59820
MNG	0.025	-0.21291	0.72981	-0.45153	0.38308	-7.44190	5.70930
	0.500	-0.07798	0.82505	-0.35106	0.52588	-7.29470	5.82320
	0.975	0.05679	0.92081	-0.25065	0.66820	-7.17460	5.95770
MOZ	0.025	-0.09831	0.44502	-0.11343	0.44856	-9.17010	7.07620
	0.500	0.02470	0.53105	-0.07231	0.54081	-9.01820	7.16960
	0.975	0.14761	0.61708	-0.03012	0.63277	-8.86760	7.33300
MRT	0.025	-0.50800	0.25423	-0.71547	1.16533	-9.23650	6.57250
	0.500	-0.26783	0.35513	-0.63285	1.27741	-8.93810	6.76210
	0.975	-0.02804	0.45708	-0.55056	1.38928	-8.72330	7.02050
MWI	0.025	0.18447	0.68786	-0.45012	0.49075	-9.84960	7.73940
	0.500	0.30691	0.79219	-0.40113	0.60894	-9.67630	7.81270
	0.975	0.42929	0.89639	-0.35219	0.72708	-9.57250	7.93180
NAM	0.025	-1.15943	1.36991	-0.44984	-0.16322	-10.73910	7.88070
	0.500	-0.98380	1.42499	-0.37403	-0.05094	-10.44810	8.00700
	0.975	-0.80856	1.48036	-0.29822	0.06080	-10.25750	8.20170
NER	0.025	-0.29466	0.02197	0.15939	0.60284	-10.31860	7.76140
	0.500	-0.17844	0.11262	0.19650	0.69063	-10.12090	7.84140
	0.975	-0.06255	0.20357	0.23392	0.77809	-9.98710	7.95560
NGA	0.025	-0.32980	0.41400	-0.71326	1.03300	-10.75690	7.73180
	0.500	-0.16542	0.50402	-0.66022	1.15615	-10.74400	7.74230
	0.975	-0.00119	0.59406	-0.60736	1.27922	-10.73360	7.75340
NIC	0.025	-0.36729	-0.44538	-1.01800	1.95287	-8.70460	6.54880
	0.500	-0.11003	-0.26970	-0.89680	2.16652	-8.36320	6.81910
	0.975	0.14700	-0.09438	-0.77610	2.38061	-8.09570	7.15420
NPL	0.025	-2.20761	0.54878	-1.28629	1.45931	-10.89010	7.92070
	0.500	-1.90666	0.61598	-1.19977	1.58355	-10.82860	7.96210
	0.975	-1.60635	0.68416	-1.11438	1.70807	-10.77380	8.00080
PAK	0.025	-0.85461	-0.44365	-0.14661	0.63877	-7.04170	4.30550
	0.500	0.01728	-0.11316	0.11240	0.99885	-6.91140	4.59580
	0.975	0.88820	0.22213	0.37243	1.35606	-6.78870	4.89300
PAN	0.025	-0.74828	-0.21079	0.12366	0.22456	-7.02990	4.19510
	0.500	0.18495	0.01081	0.44938	0.53935	-6.59570	4.91570

	Quantiles	int	gam	gbm	lasso	Nominal range	Nominal variance
	0.975	1.11749	0.23255	0.77538	0.85362	-6.15950	5.61380
PER	0.025	-1.26223	0.15941	-0.72812	1.32151	-9.57940	7.42260
	0.500	-1.16686	0.24049	-0.67103	1.43062	-9.45200	7.49380
	0.975	-1.07189	0.32174	-0.61423	1.53955	-9.36320	7.59510
PHL	0.025	-3.22494	-0.02815	-0.63527	1.11952	-10.43250	7.23420
	0.500	-2.92056	0.17639	-0.51313	1.33607	-10.15000	7.37090
	0.975	-2.61712	0.38682	-0.39239	1.54950	-9.95590	7.56650
PNG	0.025	-0.35574	-3.83719	-0.89308	3.58768	-9.33970	2.77440
	0.500	1.44605	-2.85968	-0.63301	4.50055	-9.02570	3.33780
	0.975	3.24730	-1.98128	-0.41525	5.53938	-8.71810	4.08620
PRY	0.025	-0.29870	0.46390	-0.11419	0.26133	-6.34400	4.59500
	0.500	-0.10348	0.58977	-0.00670	0.41688	-5.70860	5.24450
	0.975	0.09193	0.71525	0.10162	0.57198	-5.11060	5.93530
RWA	0.025	-0.52080	0.49687	-0.97002	1.14034	-10.31770	7.77490
	0.500	-0.25679	0.57836	-0.85230	1.27358	-10.00270	7.92460
	0.975	0.00715	0.65893	-0.73269	1.40646	-9.78320	8.13590
SDN	0.025	0.33871	-0.14987	-0.24040	1.25562	-9.46430	7.29480
	0.500	0.42347	-0.10137	-0.20979	1.31122	-9.44430	7.31460
	0.975	0.50815	-0.05292	-0.17934	1.36681	-9.41830	7.34060
SEN	0.025	-0.33976	-0.91693	-0.79785	2.26668	-9.89520	7.00990
	0.500	0.00439	-0.75028	-0.70189	2.45222	-9.67000	7.14070
	0.975	0.34823	-0.58374	-0.60669	2.63797	-9.50620	7.31500
SLE	0.025	-1.92112	-0.72431	1.05385	0.45878	-9.81430	-25.61080
	0.500	0.01377	-0.65278	1.09602	0.55681	-9.74860	-25.54510
	0.975	1.94710	-0.58111	1.13810	0.65457	-9.68280	-25.47930
SLV	0.500	-0.07871	0.58757	-0.95343	0.24641	-9.13950	7.14090
	0.975	0.24510	1.04247	-0.70331	0.66062	-8.99380	7.38920
	0.975	0.56866	1.49750	-0.45357	1.07448	-8.84870	7.53710
SOM	0.025	-0.37449	-0.03985	0.02089	0.59806	-5.29100	3.65910
	0.500	-0.02160	0.11194	0.13098	0.75698	-5.05460	4.10550
	0.975	0.33073	0.26402	0.24115	0.91526	-4.80870	4.52170
SSD	0.025	-0.51869	-0.01871	-0.35255	0.88391	-5.38650	3.67340
	0.500	-0.09181	0.12778	-0.21486	1.08697	-5.23120	4.09730
	0.975	0.33458	0.27483	-0.07753	1.28978	-5.05040	4.44480
SWZ	0.025	-1.76612	0.30511	-1.88974	1.64652	-10.87710	6.52490
	0.500	-0.83896	0.47716	-1.48281	2.00533	-9.53860	7.48740
	0.975	0.08741	0.64969	-1.07731	2.36477	-8.41620	8.60790
SYR	0.025	-1.24778	-0.28932	-0.04487	0.42363	-8.15850	4.89050
	0.500	-0.15642	0.03582	0.19083	0.76645	-7.71690	5.44160
	0.975	0.93287	0.36486	0.44599	1.10055	-7.29310	6.00520
TCD	0.025	-1.47262	-0.35645	-0.19737	1.29796	-10.77370	7.91240
	0.500	-1.32743	-0.27013	-0.13294	1.40321	-10.47620	8.02290

	Quantiles	int	gam	gbm	lasso	Nominal range	Nominal variance
	0.975	-1.18265	-0.18361	-0.06853	1.50774	-10.30060	8.21470
TGO	0.025	-2.03196	-3.38970	-0.27237	3.09061	-9.12110	0.71830
	0.500	-0.10003	-2.68669	-0.13770	3.82255	-8.92290	1.97170
	0.975	1.83034	-1.99662	-0.00230	4.56907	-8.72420	2.67340
THA	0.025	-0.21910	0.27377	0.16416	-0.56214	-6.61110	0.15260
	0.500	0.00493	0.69063	0.44840	-0.14244	-2.83660	4.97210
	0.975	0.21691	1.12078	0.74998	0.25392	1.47150	10.04620
TJK	0.025	-0.76502	1.47938	-0.57684	-0.46279	-10.51560	7.15150
	0.500	0.21654	1.64143	-0.44511	-0.19586	-10.49780	7.17170
	0.975	1.19765	1.80933	-0.31533	0.06632	-10.47650	7.18860
TKM	0.025	-1.43439	-0.10713	-0.15952	-0.24838	-5.27470	-1.07440
	0.500	-0.14510	0.46750	0.12325	0.40300	-4.80150	1.79760
	0.975	1.14211	1.09067	0.41873	1.00762	-4.17200	3.35870
TLS	0.025	-0.22510	-0.00534	-0.83904	1.46194	-10.85060	7.81740
	0.500	0.08762	0.13454	-0.73702	1.60255	-10.34670	8.08150
	0.975	0.39999	0.27469	-0.63517	1.74267	-9.95250	8.41690
TUN	0.025	-0.37145	-0.14257	0.00332	0.37117	-6.89800	3.88540
	0.500	0.79787	0.02139	0.30976	0.66896	-6.54130	4.60500
	0.975	1.96938	0.18587	0.61790	0.96336	-6.14930	5.20310
TZA	0.025	0.67393	0.70713	-0.85393	0.99097	-10.05340	7.46080
	0.500	0.81456	0.75570	-0.80841	1.05267	-9.90240	7.52800
	0.975	0.95514	0.80433	-0.76291	1.11424	-9.81460	7.64520
UGA	0.025	0.38629	1.08880	-1.44419	0.85778	-10.82520	7.82650
	0.500	0.67952	1.24561	-1.30642	1.06071	-10.80230	7.87040
	0.975	0.97265	1.40332	-1.16931	1.26334	-10.77210	7.89670
UZB	0.025	-0.46957	-0.71557	-0.17912	-0.53138	-5.63510	0.59490
	0.500	0.00349	0.38793	0.04926	0.55858	-3.79270	3.36860
	0.975	0.48728	1.48116	0.29573	1.64890	-1.14440	5.55230
VNM	0.025	0.24231	1.15346	0.24109	-1.09182	-10.12530	7.76430
	0.500	0.43844	1.41335	0.39076	-0.80428	-10.07830	7.80210
	0.975	0.63451	1.67348	0.54088	-0.51782	-10.03190	7.85190
YEM	0.025	0.32598	-0.46576	-0.65609	1.87743	-10.28190	4.14200
	0.500	0.42887	-0.36582	-0.60864	1.97450	-10.28000	4.14660
	0.975	0.53169	-0.26606	-0.56129	2.07161	-10.27530	4.15700
ZAF	0.025	-0.03431	-0.12939	-0.13417	1.14827	-9.08090	7.25440
	0.500	0.02221	-0.08705	-0.11548	1.20252	-8.98090	7.29570

	Quantiles	int	gam	gbm	lasso	Nominal range	Nominal variance
	0.975	0.07870	-0.04470	-0.09680	1.25670	-8.92220	7.36640
ZMB	0.025	0.20030	1.13666	-0.84214	0.54989	-12.40190	7.90500
	0.500	0.47657	1.17705	-0.79387	0.61682	-11.68650	8.11210
	0.975	0.75272	1.21753	-0.74584	0.68372	-11.33550	8.52280
ZWE	0.025	-1.42807	0.67156	-0.82980	0.41318	-10.38780	7.73720
	0.500	-1.15974	0.93747	-0.65908	0.72177	-10.34450	7.77010
	0.975	-0.89197	1.20506	-0.49032	1.02983	-10.28680	7.81620

Appendix Tables 13-18. In sample validation metrics

Appendix Table 13. Predictive in sample metrics aggregated to the country level for water indicators

Indicator	Year	Mean err.	RMSE	Median SS	Corr.	95% Cov.
Piped	2000	0.032	0.139	29736.07	0.802	0.62
Piped	2005	0.111	0.157	38635.04	0.924	0.721
Piped	2010	0.009	0.074	55688.02	0.955	0.793
Piped	2017	0.051	0.15	39369.18	0.763	0.548
Conditional improved	2000	-0.036	0.07	37446.92	0.951	0.778
Conditional improved	2005	0.007	0.067	31968.37	0.789	0.735
Conditional improved	2010	0.044	0.077	42434.14	0.968	0.751
Conditional improved	2017	0.041	0.132	26357.32	0.762	0.739
Conditional unimproved	2000	-0.02	0.089	19358.4	0.946	0.727
Conditional unimproved	2005	0.009	0.037	13114.09	0.987	0.787
Conditional unimproved	2010	0.022	0.062	24987.68	0.903	0.702
Conditional unimproved	2017	-0.012	0.044	4431.835	0.988	0.781

Appendix Table 14. Predictive in sample metrics aggregated to the first administrative level for water indicators

Indicator	Year	Mean err.	RMSE	Median SS	Corr.	95% Cov.
Piped	2000	0.032	0.232	1392.716	0.68	0.624
Piped	2005	0.111	0.254	701	0.708	0.724
Piped	2010	0.009	0.169	3841.032	0.793	0.791
Piped	2017	0.051	0.208	1698.948	0.667	0.546
Conditional improved	2000	-0.036	0.135	919.507	0.846	0.78
Conditional improved	2005	0.007	0.181	363.5	0.582	0.735
Conditional improved	2010	0.044	0.141	2006.483	0.868	0.755
Conditional improved	2017	0.041	0.182	974.56	0.667	0.738
Conditional unimproved	2000	-0.02	0.2	486.48	0.805	0.73
Conditional unimproved	2005	0.009	0.222	149	0.742	0.788
Conditional unimproved	2010	0.022	0.184	937.735	0.727	0.703
Conditional unimproved	2017	-0.012	0.18	177.962	0.809	0.781

Appendix Table 15. Predictive in sample metrics aggregated to the second administrative level for water indicators

Indicator	Year	Mean err.	RMSE	Median SS	Corr.	95% Cov.
Piped	2000	0.032	0.268	228	0.624	0.625
Piped	2005	0.111	0.307	126	0.594	0.723
Piped	2010	0.009	0.216	407.049	0.697	0.797
Piped	2017	0.051	0.267	585.134	0.568	0.546
Conditional improved	2000	-0.036	0.167	231.823	0.774	0.778
Conditional improved	2005	0.007	0.232	187.718	0.438	0.735
Conditional improved	2010	0.044	0.186	287.5	0.771	0.755
Conditional improved	2017	0.041	0.222	407.308	0.567	0.735
Conditional unimproved	2000	-0.02	0.222	97.813	0.771	0.727
Conditional unimproved	2005	0.009	0.274	66.462	0.633	0.786
Conditional unimproved	2010	0.022	0.244	128.404	0.599	0.703
Conditional unimproved	2017	-0.012	0.234	77.721	0.726	0.783

Appendix Table 16. Predictive in sample metrics aggregated to the country level for sanitation indicators

Indicator	Year	Mean err.	RMSE	Median SS	Corr.	95% Cov.
Piped	2000	-0.024	0.093	29335.27	0.848	0.686
Piped	2005	0.009	0.085	48796.79	0.97	0.798
Piped	2010	0.014	0.079	53771.14	0.88	0.774
Piped	2017	0.118	0.135	39161.19	0.935	0.304
Conditional improved	2000	-0.005	0.058	54084.89	0.976	0.771
Conditional improved	2005	0.011	0.076	51230.94	0.969	0.76
Conditional improved	2010	0.019	0.054	55560.49	0.98	0.828
Conditional improved	2017	0.05	0.064	36724.17	0.98	0.795
Conditional unimproved	2000	0	0.03	26817.86	0.964	0.789
Conditional unimproved	2005	0.002	0.06	28697.44	0.986	0.794
Conditional unimproved	2010	0.001	0.047	33706.83	0.988	0.853
Conditional unimproved	2017	0.042	0.127	22303.74	0.935	0.814

Appendix Table 17. Predictive in sample metrics aggregated to the first administrative level for sanitation indicators

Indicator	Year	Mean err.	RMSE	Median SS	Corr.	95% Cov.
Piped	2000	-0.024	0.138	885.326	0.76	0.69
Piped	2005	0.009	0.171	1402	0.897	0.803
Piped	2010	0.014	0.157	1378.238	0.703	0.776
Piped	2017	0.118	0.178	3654.843	0.778	0.304
Conditional improved	2000	-0.005	0.128	1302.438	0.902	0.772
Conditional improved	2005	0.011	0.13	1147.759	0.918	0.759
Conditional improved	2010	0.019	0.153	1441.333	0.845	0.827
Conditional improved	2017	0.05	0.117	2668	0.901	0.795
Conditional unimproved	2000	0	0.14	833.061	0.619	0.789
Conditional unimproved	2005	0.002	0.138	587.67	0.924	0.794
Conditional unimproved	2010	0.001	0.14	1006.244	0.904	0.851
Conditional unimproved	2017	0.042	0.179	2144.865	0.862	0.816

Appendix Table 18. Predictive in sample metrics aggregated to the second administrative level for sanitation indicators

Indicator	Year	Mean err.	RMSE	Median SS	Corr.	95% Cov.
Piped	2000	-0.024	0.153	182.942	0.726	0.689
Piped	2005	0.009	0.19	277.712	0.875	0.801
Piped	2010	0.014	0.182	310	0.644	0.776
Piped	2017	0.118	0.217	644.322	0.665	0.306
Conditional improved	2000	-0.005	0.141	295	0.886	0.769
Conditional improved	2005	0.011	0.143	255.766	0.903	0.76
Conditional improved	2010	0.019	0.174	252	0.809	0.828
Conditional improved	2017	0.05	0.16	621.479	0.819	0.795
Conditional unimproved	2000	0	0.251	885.605	0.363	0.788
Conditional unimproved	2005	0.002	0.16	175.79	0.893	0.794
Conditional unimproved	2010	0.001	0.163	210.696	0.875	0.851
Conditional unimproved	2017	0.042	0.201	351	0.823	0.816

Appendix Tables 19-24. Out-of-sample validation metrics

Appendix Table 19. Predictive out-of-sample metrics aggregated to the country level for water indicators

Indicator	Year	Mean err.	RMSE	Median SS	Corr.	95% Cov.
Piped	2000	0.036	0.126	29736.07	0.839	0.741
Piped	2005	0.11	0.165	38635.04	0.902	0.792
Piped	2010	0.003	0.095	55688.02	0.897	0.824
Piped	2017	0.04	0.168	39369.18	0.677	0.66
Conditional improved	2000	-0.029	0.069	37446.92	0.949	0.793
Conditional improved	2005	0.006	0.07	31968.37	0.768	0.747
Conditional improved	2010	0.035	0.074	42434.14	0.96	0.79
Conditional improved	2017	0.057	0.128	26357.32	0.802	0.739
Conditional unimproved	2000	-0.013	0.096	19358.4	0.935	0.726
Conditional unimproved	2005	0	0.044	13114.09	0.98	0.749
Conditional unimproved	2010	0.025	0.063	24987.68	0.901	0.708
Conditional unimproved	2017	0.017	0.068	4431.835	0.967	0.797

Appendix Table 20. Predictive out-of-sample metrics aggregated to the first administrative level for water indicators

Indicator	Year	Mean err.	RMSE	Median SS	Corr.	95% Cov.
Piped	2000	0.036	0.257	1392.716	0.565	0.74
Piped	2005	0.11	0.268	701	0.656	0.785
Piped	2010	0.003	0.19	3841.032	0.727	0.824
Piped	2017	0.04	0.231	1698.948	0.538	0.66
Conditional improved	2000	-0.029	0.138	919.507	0.831	0.791
Conditional improved	2005	0.006	0.187	363.5	0.544	0.75
Conditional improved	2010	0.035	0.135	2006.483	0.873	0.789
Conditional improved	2017	0.057	0.179	974.56	0.701	0.74
Conditional unimproved	2000	-0.013	0.207	486.48	0.787	0.725
Conditional unimproved	2005	0	0.245	149	0.675	0.75
Conditional unimproved	2010	0.025	0.186	937.735	0.721	0.706
Conditional unimproved	2017	0.017	0.184	177.962	0.797	0.795

Appendix Table 21. Predictive out-of-sample metrics aggregated to the second administrative level for water indicators

Indicator	Year	Mean err.	RMSE	Median SS	Corr.	95% Cov.
Piped	2000	0.036	0.302	228	0.477	0.742
Piped	2005	0.11	0.324	126	0.515	0.79
Piped	2010	0.003	0.245	407.049	0.597	0.824
Piped	2017	0.04	0.3	585.134	0.382	0.66
Conditional improved	2000	-0.029	0.173	231.823	0.746	0.792
Conditional improved	2005	0.006	0.232	187.718	0.407	0.748
Conditional improved	2010	0.035	0.181	287.5	0.781	0.79
Conditional improved	2017	0.057	0.217	407.308	0.596	0.742
Conditional unimproved	2000	-0.013	0.231	97.813	0.746	0.727
Conditional unimproved	2005	0	0.281	66.462	0.594	0.749
Conditional unimproved	2010	0.025	0.237	128.404	0.598	0.706
Conditional unimproved	2017	0.017	0.246	77.721	0.687	0.795

Appendix Table 22. Predictive out-of-sample metrics aggregated to the country level for sanitation indicators

Indicator	Year	Mean err.	RMSE	Median SS	Corr.	95% Cov.
Piped	2000	-0.014	0.079	29335.27	0.882	0.769
Piped	2005	0.015	0.075	48796.79	0.978	0.815
Piped	2010	0.006	0.084	53771.14	0.867	0.806
Piped	2017	0.108	0.123	39161.19	0.965	0.354
Conditional improved	2000	0.007	0.036	54084.89	0.992	0.806
Conditional improved	2005	0.011	0.043	51230.94	0.992	0.808
Conditional improved	2010	0.021	0.051	55560.49	0.984	0.858
Conditional improved	2017	0.057	0.071	36724.17	0.976	0.831
Conditional unimproved	2000	0.028	0.039	26817.86	0.975	0.834
Conditional unimproved	2005	0.018	0.074	28697.44	0.981	0.851
Conditional unimproved	2010	0.012	0.07	33706.83	0.974	0.876
Conditional unimproved	2017	0.041	0.13	22303.74	0.936	0.86

Appendix Table 23. Predictive out-of-sample metrics aggregated to the first administrative level for sanitation indicators

Indicator	Year	Mean err.	RMSE	Median SS	Corr.	95% Cov.
Piped	2000	-0.014	0.131	885.326	0.749	0.776
Piped	2005	0.015	0.178	1402	0.887	0.816
Piped	2010	0.006	0.16	1378.238	0.689	0.806
Piped	2017	0.108	0.171	3654.843	0.782	0.353
Conditional improved	2000	0.007	0.119	1302.438	0.913	0.806
Conditional improved	2005	0.011	0.129	1147.759	0.92	0.806
Conditional improved	2010	0.021	0.163	1441.333	0.822	0.857
Conditional improved	2017	0.057	0.121	2668	0.895	0.83
Conditional unimproved	2000	0.028	0.137	833.061	0.649	0.833
Conditional unimproved	2005	0.018	0.156	587.67	0.904	0.85
Conditional unimproved	2010	0.012	0.154	1006.244	0.885	0.874
Conditional unimproved	2017	0.041	0.173	2144.865	0.883	0.859

Appendix Table 24. Predictive out-of-sample metrics aggregated to the second administrative level for sanitation indicators

Indicator	Year	Mean err.	RMSE	Median SS	Corr.	95% Cov.
Piped	2000	-0.014	0.156	182.942	0.681	0.778
Piped	2005	0.015	0.202	277.712	0.856	0.816
Piped	2010	0.006	0.191	310	0.603	0.807
Piped	2017	0.108	0.223	644.322	0.603	0.351
Conditional improved	2000	0.007	0.138	295	0.888	0.808
Conditional improved	2005	0.011	0.15	255.766	0.892	0.805
Conditional improved	2010	0.021	0.188	252	0.772	0.858
Conditional improved	2017	0.057	0.154	621.479	0.823	0.833
Conditional unimproved	2000	0.028	0.253	885.605	0.354	0.835
Conditional unimproved	2005	0.018	0.177	175.79	0.87	0.85
Conditional unimproved	2010	0.012	0.18	210.696	0.846	0.874
Conditional unimproved	2017	0.041	0.193	351	0.843	0.859

Appendix Table 25a-b. Risk ratios used in counterfactual analyses

We present the risk ratios used in our counterfactual analyses as adapted from the Global Burden of Diseases, Risk Factors, and Injuries 2017[9].

Appendix Table 26. Risk ratios associated with access to water facilities.

Water Facility Category	Risk Ratio
**Piped, boiled/filtered**	1
**Piped, solar/chlorinated**	1.65
**Piped, untreated**	2.4
**Other Improved, boiled/filtered**	1.12
**Other Improved, solar/chlorinated**	1.85
**Other Improved, untreated**	2.69
**Unimproved or Surface, boiled/filtered**	1.36
**Unimproved or Surface, solar/chlorinated**	2.25
**Unimproved or Surface, untreated**	3.28

Appendix Table 27. Risk ratios associated with access to water facilities.

Sanitation Facilities	Risk Ratio
**Sewer or Septic**	1
**Other Improved**	2.595
**Unimproved or Open Defecation**	3.242

7.0 Appendix References

1 Dicker D, Nguyen G, Abate D, *et al.* Global, regional, and national age-sex-specific mortality and life expectancy, 1950–2017: a systematic analysis for the Global Burden of Disease Study 2017. *Lancet* 2018; **392**: 1684–735.

2 Institute for Health Metrics and Evaluation. Global Health Data Exchange. 2018; published online June 20. http://ghdx.healthdata.org/ (accessed March 10, 2020).

3 WHO UNICEF Joint Monitoring Programme. Progress on drinking water, sanitation and hygiene: 2017 update and SDG baselines. Geneva: WHO & UNICEF, 2017.

4 Kish L. Survey sampling. New York, London: John Wiley & Sons, Inc., 1965.

5 WorldPop. WorldPop dataset. 2017; published online July 25. http://www.worldpop.org.uk/data/get_data/ (accessed July 24, 2017).

6 Mosser JF, Gagne-Maynard W, Rao PC, *et al.* Mapping diphtheria-pertussis-tetanus vaccine coverage in Africa, 2000–2016: a spatial and temporal modelling study. *Lancet* 2019; **393**: 1843–55.

7 Sk-Opt Module. 2018; published online June 20. https://scikit-optimize.github.io/ (accessed March 10, 2020).

8 Lidngren F, Rue H, Lindström J. An explicit link between Gaussian fields and Gaussian Markov random fields: the stochastic partial differential equation approach. *Stat Methodol Ser B* 2011; **73**: 423–98.

9 Stanaway JD, Afshin A, Gakidou E, *et al.* Global, regional, and national comparative risk assessment of 84 behavioural, environmental and occupational, and metabolic risks or clusters of risks for 195 countries and territories, 1990–2017: a systematic analysis for the Global Burden of Disease Study 2017. *Lancet* 2018; **392**: 1923–94.

10 De Maio FG. Income inequality measures. *J Epidemiol Community Health* 2007; **61**: 849–52.

11 Land Processes Distributed Active Archive Center. Combined MODIS 5.1 dataset. MCD12Q1 LP DAAC NASA Land Data Prod. Serv. 2017; published online June 1.

12 World Wildlife Fund & Center for Environmental Systems Research, University of Kassel G. Global lakes and wetlands database, level 3. 2004 https://www.worldwildlife.org/pages/global-lakes-and-wetlands-database (accessed June 1, 2017).

13 Lehner B, Döll P. Development and validation of a global database of lakes, reservoirs and wetlands. *J Hydrol* 2004; **296**: 1–22.

14 GeoNetwork. The Global Administrative Unit Layers (GAUL). 2015. http://www.fao.org/geonetwork/srv/en/main.home (accessed June 1, 2017).

15 Tatem AJ. WorldPop, open data for spatial demography. *Sci Data* 2017; **4**: 170004.

Managing the estimation or publications process

Simon I. Hay, Robert C. Reiner Jr., Brigette F. Blacker, Molly K. Miller-Petrie, Paulina A. Lindstedt, Natalie Cormier, Puja C. Rao

Writing the first draft of the manuscript

Aniruddha Deshpande, Molly K. Miller-Petrie

Primary responsibility for this manuscript focused on: applying analytical methods to produce estimates

Aniruddha Deshpande

Primary responsibility for this manuscript focused on: seeking, cataloguing, extracting, or cleaning data; designing or coding figures and tables

Aniruddha Deshpande, Paulina A. Lindstedt, Mathew M. Baumann, Alexandria Brown, Gregory M. Garcia, James R. Albright, Kimberly B. Johnson, Robert C. Reiner Jr.

Providing data or critical feedback on data sources

Aniruddha Deshpande, Paulina A. Lindstedt, Mathew M. Baumann, Vafa Rahimi-Movaghar, Fernando de la Hoz, Aslam Pervaiz, QuynhAnh Nguyen, Kimberly Johnson, Emma Spurlock, Shiwei Liu, Paulina Lindstedt, Hedayat Abbastabar, Ahmed Abdelalim, Michael Abrigo, Oladimeji Adebayo, Isaac Adedeji, Rufus Adedoyin, Victor Adekanmbi, Olatunji Adetokunboh, Tara Ballav Adhikari, Mohsen Afarideh, Marcela Agudelo-Botero, Muktar Ahmed, Ali Akanda, Fares Alahdab, Jacqueline Alcalde-Rabanal, Muhammad Ali, Mehran Alijanzadeh, Amir Almasi-Hashiani, Nelson Alvis-Guzman, Nelson Alvis-Zakzuk, Saeed Amini, Catalina Liliana Andrei, Jalal Arabloo, Olatunde Aremu, Bahram Armoon, Amit Arora, Krishna Aryal, Hagos Atalay, Shally Awasthi, Nefsu Awoke, Beatriz Paulina Ayala Quintanilla, Yared Aynalem, Samad Azari, Senthilkumar Balakrishnan, Maciej Banach, Joseph Banoub, Miguel Barboza, Sanjay Basu, Mohsen Bayati, Mahya Beheshti, Meysam Behzadifar, Diana Bejarano Ramirez, Michellr

Bell, Muhammad Shahdaat Bin Sayeed, Somayeh Bohlouli, Till Bärnighausen, Sharath Burugina Nagaraja, Julio Cesar Campuzano Rincon, Jorge Cano, Carlos Castañeda-Orjuela, Franz Castro, Binaya Chalise, Vijay Kumar Chattu, Ken Chin, Dinh-Toi Chu, Vera Costa, Abel Dadi, Saad Dahlawi, Lalit Dandona, Rakhi Dandona, Anh Dang, Amira Darwish, Ahmad Daryani, Rajat Das Gupta, Dereje Demissie, Samath Dharmaratne, Preeti Dhillon, Meghnath Dhimal, Govinda Dhungana, Christiane Dolecek, Hisham Edinur, Andem Effiong, Aziz Eftekhari, Maysaa El Sayed Zaki, Ziad El-Khatib, Hajer Elkout, Shymaa Enany, Daniel Endalew, Babak Eshrati, Oluchi Ezekannagha, Emerito Jose Faraon, Mohammad Fareed, Farshad Farzadfar, Valery Feigin, Netsanet Fentahun, Seyed-Mohammad Fereshtehnejad, Morenike Folayan, Takeshi Fukumoto, Ahmad Ghashghaee, Themba Ginindza, Alem Girmay, Bárbara Goulart, Ayman Grada, Harish Gugnani, Rahul Gupta, Nima Hafezi-Nejad, Michael Haile, Brian Hall, Hamidreza Haririan, Ninuk Hariyani, Amir Hasanzadeh, Hamid Hassen, Simon Hay, Desta Hayelom, Mohamed Hegazy, Delia Hendrie, Andualem Henok, Claudiu Herteliu, Thomas Hird, Chi Hoang, Praveen Hoogar, Mehdi Hosseinzadeh, Mowafa Househ, Guoqing Hu, Syed Hussain, Mamusha Hussen, Segun Ibitoye, Usman Iqbal, Seyed Sina Irvani, Sheikh Mohammed Shariful Islam, Nader Jahanmehr, Mihajlo Jakovljevic, Jost Jonas, Jacek Jozwiak, Zubair Kabir, André Karch, Hamidreza Karimi-Sari, Surendra Karki, Amir Kasaeian, Habtamu Kasaye, Gbenga Kayode, Mihiretu Kebede, Yousef Khader, Morteza Khafaie, Nauman Khalid, Rovshan Khalilov, Khaled Khatab, Amir Khater, Jagdish Khubchandani, Yun Jin Kim, Adnan Kisa, Sezer Kisa, Soewarta Kosen, Kewal Krishan, Barthelemy Kuate Defo, Pushpendra Kumar, Dian Kusuma, Dharmesh Lal, Hilton Lam, Van Lansingh, Savita Lasrado, Georgy Lebedev, Tsegaye Lenjebo, Shanshan Li, Jaifred Christian Lopez, Muhammed Magdy Abd El Razek, D. R. Mahadeshwara Prasad, Narayan Mahotra, Navid Manafi, Mohammad Ali Mansournia, Francisco Martins-Melo, Benjamin Mayala, Mohsen Mazidi, Colm McAlinden, Tefera Mekonnen, Gebrekiros Meles, Ziad Memish, Walter Mendoza, Ritesh Menezes, Bartosz Miazgowski, Erkin Mirrakhimov, Babak Moazen, Dara Mohammad, Naser Mohammad Gholi Mezerji, Shafiu Mohammed, Jemal Mohammed, Farnam Mohebi, Ali Mokdad, Masoud Moradi, Mohammad Moradi-Joo, Seyyed Meysam Mousavi, Miliva Mozaffor, Kamarul Imran Musa, Ghulam Mustafa, Saravanan Muthupandian, Ahamarshan Nagarajan, Mohsen Naghavi, Bruno Nascimento, Javad Nazari, Ionut Negoi, Josephine Ngunjiri, Chukwudi Nnaji, Marzieh Nojomi, Jean Jacques Noubiap, Andrew Olagunju, Bolajoko Olusanya, Obinna Onwujekwe, Mayowa Owolabi, Mahesh P A, Jagadish Rao. Padubidri, Sangram Patel, Ashish Pathak, Kebreab Paulos, Veincent Christian Pepito, Julia Pescarini, Maarten Postma, Sergio Prada, Amir Radfar, Alireza Rafiei, Siavash Rahimi, Mohammad Hifz Ur Rahman, Chhabi Ranabhat, Lal Rawal, Vishnu Renjith, Andre Renzaho, Ana Isabel Ribeiro, Jennifer Rickard, Leonardo Roever, Luca Ronfani, Enrico Rubagotti, Siamak Sabour, Ehsan Sadeghi, Sahar Saeedi Moghaddam, Yahya Safari, Rajesh Sagar, S. Mohammad Sajadi, Mohammad Salahshoor, Nasir Salam, Hosni Salem, Marwa Salem, Abdallah Samy, Milena Santric-Milicevic, Bruno Sao Jose, Sivan Saraswathy, Benn Sartorius, Brijesh Sathian, Maheswar Satpathy, Monika Sawhney, David Schwebel, Subramanian Senthilkumaran, Edson Serván-Mori, Amira Shaheen, Masood Shaikh, Ali Shalash, Mehran Shams-Beyranvand, Morteza Shamsizadeh, Mohammed Shannawaz, B Suresh Shetty, Wondimeneh Shiferaw, Jae Il Shin, Soraya Siabani, Diego Silva, Jasvinder Singh, Virendra Singh, Dhirendra Sinha, Malede Sisay, Eirini Skiadaresi, Adauto Soares Filho, Mohammad Reza Sobhiyeh, Muluken Sorrie, Chandrashekhar Sreeramareddy, Agus Sudaryanto, Hafiz Ansar Rasul Suleria, Bryan Sykes, Rafael Tabarés-Seisdedos, Degena Tadesse, Bineyam Taye, Nihal Thomas, Marcos Roberto Tovani-Palone, Irfan Ullah, Bhaskaran Unnikrishnan, Pascual Valdez, Feleke W/hawariat, Yasir Waheed, Yuan-Pang Wang, Yafeng Wang, Ronny Westerman, Taweewat Wiangkham, Tissa Wijeratne, Charles Wiysonge, Haileab

Wolde, Dawit Wondafrash, Tewodros Wonde, Ali Yadollahpour, Alex Yeshaneh, Naohiro Yonemoto, Mustafa Younis, Chuanhua Yu, Siddhesh Zadey, Zoubida Zaidi, Arash Ziapour, Yves Miel Zuniga

Development of methods or computational machinery

Aniruddha Deshpande, Alyssa Sbarra, Farah Daoud, QuynhAnh Nguyen, Joseph Frostad, Kirsten Wiens,Olatunji Adetokunboh, Amir Almasi-Hashiani, Samad Azari, Meysam Behzadifar, Muhammad Shahdaat Bin Sayeed, Sharath Burugina Nagaraja, Devasahayam Christopher, Ahmad Daryani, Aziz Eftekhari, Ahmad Ghashghaee, Ayman Grada, Michael Haile, Mowafa Househ, Rovshan Khalilov, Pushpendra Kumar, Cheru Leshargie, Mohsen Mazidi, Ali Mokdad, Mohsen Naghavi, Fakher Rahim, Chhabi Ranabhat, Lal Rawal, Mohammad Salahshoor, Abdallah Samy, Maheswar Satpathy, Degena Tadesse, Ronny Westerman, Tissa Wijeratne, Dawit Wondafrash, Arash Ziapour

Providing critical feedback on methods or results

Kirsten Wiens, Aniruddha Deshpande, Alyssa Sbarra, Molly Miller-Petrie, Jonathan Mosser, Kate LeGrand, Vafa Rahimi-Movaghar, Ali Kiadaliri, Lauren Schaeffer, Mohamed Gad, Fernando de la Hoz, Lauren Schaeffer, Aslam Pervaiz, David Smith, Roghayeh Mohammadibakhsh, Elizabeth Cromwell, Taddese Zerfu, Manasi Kumar, Hedayat Abbastabar, Foad Abd-Allah, Ahmed Abdelalim, Ibrahim Abdollahpour, Kedir Abegaz, Ayenew Abejie, Michael Abrigo, Manfred Accrombessi, Abdu Adamu, Oladimeji Adebayo, Isaac Adedeji, Rufus Adedoyin, Victor Adekanmbi, Olatunji Adetokunboh, Mohsen Afarideh, Marcela Agudelo-Botero, Keivan Ahmadi, Mehdi Ahmadi, Muktar Ahmed, Anwar Ahmed, Temesgen Akalu, Ali Akanda, Fares Alahdab, Ziyad Al-Aly, Noore Alam, Genet Melak Alamene, Turki Alanzi, Jacqueline Alcalde-Rabanal, Animut Alebel, Zewdie Alemu, Muhammad Ali, Mehran Alijanzadeh, Vahid Alipour, Syed Aljunid, Amir Almasi-Hashiani, Hesham Al-Mekhlafi, Khalid Altirkawi, Nelson Alvis-Guzman, Nelson Alvis-Zakzuk, Saeed Amini, Arianna Maever Amit, Gianna Gayle Amul, Catalina Liliana Andrei, Ansariadi Ansariadi, Carl Abelardo Antonio, Ernoiz Antriyandarti, Jalal Arabloo, Hany Aref, Olatunde Aremu, Amit Arora, Krishna Aryal, Hagos Atalay, Seyyed Shamsadin Athari, Sachin Atre, Shally Awasthi, Nefsu Awoke, Beatriz Paulina Ayala Quintanilla, Getinet Ayano, Martin Ayanore, Yared Aynalem, Samad Azari, Andrew Azman, Ebrahim Babaee, Shankar Bakkannavar, Senthilkumar Balakrishnan, Maciej Banach, Joseph Banoub, Aleksandra Barac, Miguel Barboza, Sanjay Basu, Mohsen Bayati, Neeraj Bedi, Mahya Beheshti, Meysam Behzadifar, Masoud Behzadifar, Diana Bejarano Ramirez, Michellr Bell, Derrick Bennett, Dessalegn Berbada, Robert Bernstein, Anusha Bhat, Soumyadeep Bhaumik, Ali Bijani, Boris Bikbov, Raaj Kishore Biswas, Somayeh Bohlouli, Soufiane Boufous, Oliver Brady, Gabrielle Britton, Till Bärnighausen, Sharath Burugina Nagaraja, Zahid Butt, Ismael Campos-Nonato, Jorge Cano, Carlos Castañeda-Orjuela, Franz Castro, Ester Cerin, Vijay Kumar Chattu, Ken Chin, Devasahayam Christopher, Dinh-Toi Chu, Luis Cámera, Vera Costa, Rosario Cárdenas, Tukur Dahiru, Saad

Dahlawi, Lalit Dandona, Rakhi Dandona, Anh Dang, Amira Darwish, Ahmad Daryani, Jai Das, Rajat Das Gupta, Aditya Dash, Jan-Walter De Neve, Dereje Demissie, Gebre Demoz, Edgar Denova- Gutiérrez, Kebede Deribe, Assefa Desalew, Samath Dharmaratne, Preeti Dhillon, Meghnath Dhimal, Daniel Diaz, Isaac Dipeolu, Kerrie Doyle, Andem Effiong, Aziz Eftekhari, Nevine El Nahas, Maysaa El Sayed Zaki, Maha El Tantawi, Hala Elhabashy, Ziad El-Khatib, Aisha Elsharkawy, Daniel Endalew, Sharareh Eskandarieh, Arash Etemadi, Oluchi Ezekannagha, Emerito Jose Faraon, Mohammad Fareed, Andre Faro, Farshad Farzadfar, Alebachew Fasil, Mehdi Fazlzadeh, Valery Feigin, Wubalem Fekadu, Netsanet Fentahun, Florian Fischer, Nataliya Foigt, Morenike Folayan, Masoud Foroutan, Richard Franklin, Takeshi Fukumoto, Augustine Gatotoh, Reta Gayesa, Ketema Gebremedhin, Yilma Geramo, Hailay Gesesew, Kebede Embaye Gezae, Ahmad Ghashghaee, Farzaneh Ghazi Sherbaf, Paramjit Gill, Themba Ginindza, Alem Girmay, Zemichael Gizaw, Amador Goodridge, Sameer Gopalani, Alessandra Goulart, Bárbara Goulart, Ayman Grada, Mohammed Gubari, Harish Gugnani, Davide Guido, Rajeev Gupta, Juanita Haagsma, Nima Hafezi-Nejad, Michael Haile, Dessalegn Haile, Brian Hall, Samer Hamidi, Demelash Woldeyohannes Handiso, Ninuk Hariyani, Md. Mehedi Hasan, Amir Hasanzadeh, Hamid Hassen, Simon Hay, Mohamed Hegazy, Behzad Heibati, Behnam Heidari, Delia Hendrie, Claudiu Herteliu, Fatemeh Heydarpour, Hagos Hidru, Thomas Hird, Chi Hoang, Praveen Hoogar, Naznin Hossain, Mehdi Hosseinzadeh, Mowafa Househ, Guoqing Hu, Ayesha Humayun, Syed Hussain, Mamusha Hussen, Olayinka Ilesanmi, Milena Ilic, Mohammad Hasan Imani-Nasab, Usman Iqbal, Seyed Sina Irvani, Sheikh Mohammed Shariful Islam, Rebecca Ivers, Chinwe Iwu, Mihajlo Jakovljevic, Amir Jalali, Achala Jayatilleke, Ensiyeh Jenabi, Ravi Jha, Vivekanand Jha, John Ji, Jost Jonas, Jacek Jozwiak, Ali Kabir, Zubair Kabir, Tanuj Kanchan, André Karch, Hamidreza Karimi-Sari, Surendra Karki, Amir Kasaeian, Gebremicheal Kasahun, Habtamu Kasaye, Gebrehiwot Kassa, Getachew Kassa, Gbenga Kayode, Mihiretu Kebede, Peter Keiyoro, Daniel Ketema, Yousef Khader, Morteza Khafaie, Rovshan Khalilov, Ejaz Khan, Junaid Khan, Md Khan, Khaled Khatab, Mona Khater, Maryam Khayamzadeh, Mohammad Khazaei, Mohammad Hossein Khosravi, Jagdish Khubchandani, Yun Jin Kim, Ruth Kimokoti, Adnan Kisa, Sezer Kisa, Shivakumar KM KMShivakumar, Tufa Kolola, Parvaiz Koul, Ai Koyanagi, Kewal Krishan, Barthelemy Kuate Defo, Pushpendra Kumar, Dian Kusuma, Carlo La Vecchia, Aparna Lal, Dharmesh Lal, Hilton Lam, Savita Lasrado, Mostafa Leili, Tsegaye Lenjebo, Sonia Lewycka, Shanshan Li, Shai Linn, Jaifred Christian Lopez, Muhammed Magdy Abd El Razek, D. R. Mahadeshwara Prasad, Phetole Mahasha, Azeem Majeed, Abdullah Mamun, Navid Manafi, Mohammad Ali Mansournia, Chabila Christopher Mapoma, Gabriel Martinez, Santi Martini, Francisco Martins-Melo, Manu Mathur, Benjamin Mayala, Mohsen Mazidi, Colm McAlinden, Kala Mehta, Teferi Mekonnen, Gebrekiros Meles, Hagazi Meles, Peter Memiah, Ziad Memish, Walter Mendoza, Ritesh Menezes, Seid Tiku Mereta, Tuomo Meretoja, Ted Miller, GK Mini, Erkin Mirrakhimov, Babak Moazen, Dara Mohammad, Yousef Mohammad, Naser Mohammad Gholi Mezerji, Hassen Mohammed, Jemal Mohammed, Shafiu Mohammed, Farnam Mohebi, Ali Mokdad, Yoshan Moodley, Ghobad Moradi, Mohammad Moradi-Joo, Linda Morales, Abbas Mosapour, Simin Mouodi, Miliva Mozaffor, Moses Muriithi, Kamarul Imran Musa, Ghulam Mustafa, Saravanan Muthupandian, Mehdi Naderi, Ahamarshan Nagarajan, Mohsen Naghavi, Gurudatta Naik, Vinay Nangia, Bruno Nascimento, Javad Nazari, Duduzile Ndwandwe, Ionut Negoi, Henok Netsere, Josephine Ngunjiri, Solomon Nigatu, Dina Ningrum, Chukwudi Nnaji, Ole Norheim, Jean Jacques Noubiap, Bogdan Oancea, Felix Ogbo, In-Hwan Oh, Andrew Olagunju, Bolajoko Olusanya, Obinna Onwujekwe, Doris Ortega-Altamirano, Frank Osei, Mayowa Owolabi, Mahesh P A, Jagadish Rao. Padubidri, Smita Pakhale, Eun-Kee Park, Sangram Patel, Ajay Patle, Kebreab Paulos, Veincent Christian Pepito, Julia Pescarini, Meghdad Pirsaheb, Maarten Postma, Farshad Pourmalek, Akram Pourshams, Sergio Prada, Hedley Quintana,

Navid Rabiee, Amir Radfar, Alireza Rafiei, Fakher Rahim, Mohammad Hifz Ur Rahman, Muhammad Aziz Rahman, Chhabi Ranabhat, Goura Rath, Salman Rawaf, Lal Rawal, Wasiq Rawasia, Vishnu Renjith, Andre Renzaho, Serge Resnikoff, Seyed Mohammad Riahi, Jennifer Rickard, Leonardo Roever, Luca Ronfani, Siamak Sabour, Ehsan Sadeghi, Yahya Safari, Rajesh Sagar, S. Mohammad Sajadi, Ahsan Saleem, Hosni Salem, Marwa Salem, Yahya Salimi, Hamideh Salimzadeh, Abdallah Samy, Juan Sanabria, Itamar Santos, Milena Santric-Milicevic, Sivan Saraswathy, Nizal Sarrafzadegan, Benn Sartorius, Brijesh Sathian, Thirunavukkarasu Sathish, Maheswar Satpathy, Monika Sawhney, Mehdi Sayyah, David Schwebel, Anbissa Senbeta, Sadaf Sepanlou, Edson Serván -Mori, Amira Shaheen, Masood Shaikh, Ali Shalash, Mehran Shams-Beyranvand, Morteza Shamsizadeh, Mohammed Shannawaz, Kiomars Sharafi, Aziz Sheikh, Wondimeneh Shiferaw, Mika Shigematsu, Jae Il Shin, Rahman Shiri, Reza Shirkoohi, Si Si, Soraya Siabani, Tariq Siddiqi, Diego Silva, Ambrish Singh, Jasvinder Singh, Narinder Singh, Virendra Singh, Malede Sisay, Eirini Skiadaresi, Joan Soriano, Muluken Sorrie, Ireneous Soyiri, Chandrashekhar Sreeramareddy, Agus Sudaryanto, Mu'awiyyah Sufiyan, Hafiz Ansar Rasul Suleria, Bryan Sykes, Rafael Tabarés -Seisdedos, Degena Tadesse, Ingan Tarigan, Yonatal Tefera, Arash Tehrani-Banihashemi, Shishay Tekelemedhin, Merhawi Tekle, Mohamad-Hani Temsah, Berhe Tesfay, Fisaha Tesfay, Kavumpurathu Thankappan, Nihal Thomas, Alan Thomson, Marcos Roberto Tovani-Palone, Eugenio Traini, Khanh Tran, Irfan Ullah, Bhaskaran Unnikrishnan, Olalekan Uthman, Pascual Valdez, Santosh Varughese, Yousef Veisani, Sebastian Vollmer, Yasir Waheed, Mitchell Wallin, Yafeng Wang, Yuan-Pang Wang, Kinley Wangdi, Girmay Weldesamuel, Adhena Werkneh, Ronny Westerman, Taweewat Wiangkham, Tissa Wijeratne, Charles Wiysonge, Haileab Wolde, Dawit Wondafrash, Tewodros Wonde, Getasew Worku, Ali Yadollahpour, Tomohide Yamada, Mehdi Yaseri, Alex Yeshaneh, Paul Yip, Engida Yisma, Naohiro Yonemoto, Mustafa Younis, Chuanhua Yu, Hasan Yusefzadeh, Siddhesh Zadey, Zoubida Zaidi, Sojib Bin Zaman, Mohammad Zamani, Hamed Zandian, Heather Zar, Yunquan Zhang, Sanjay Zodpey

Drafting the manuscript or revising it critically for important intellectual content

Aniruddha Deshpande, Molly Miller-Petrie, Jonathan Mosser, Kate LeGrand, Vafa Rahimi-Movaghar, Ali Kiadaliri, Lauren Schaeffer, Mohamed Gad, Gillian Hollerich, Nicole Davis Weaver, Sandra Munro, Kimberly Johnson, Robert Thompson, Kirsten Wiens, Robert C. Reiner Jr, Simon I. Hay, Foad Abd-Allah, Ahmed Abdelalim, Ahmed Abualhasan, Abdu Adamu, Oladimeji Adebayo, Isaac Adedeji, Rufus Adedoyin, Victor Adekanmbi, Olatunji Adetokunboh, Mohsen Afarideh, Muktar Ahmed, Temesgen Akalu, Ali Akanda, Fares Alahdab, Samiah Alam, Genet Melak Alamene, Jacqueline Alcalde-Rabanal, Animut Alebel, Muhammad Ali, Ali Almasi, Amir Almasi-Hashiani, Hesham Al-Mekhlafi, Nelson Alvis-Guzman, Nelson Alvis-Zakzuk, Saeed Amini, Catalina Liliana Andrei, Carl Abelardo Antonio, Ernoiz Antriyandarti, Jalal Arabloo, Olatunde Aremu, Amit Arora, Afsaneh Arzani, Mehran Asadi-Aliabadi, Hagos Atalay, Shally Awasthi, Nefsu Awoke, Beatriz Paulina Ayala Quintanilla, Martin Ayanore, Andrew Azman, Alaa Badawi, Shankar Bakkannavar, Senthilkumar Balakrishnan, Maciej Banach, Aleksandra Barac, Sanjay Basu, Neeraj Bedi, Meysam Behzadifar, Masoud Behzadifar, Michellr Bell, Derrick Bennett, Habib Benzian, Anusha Bhat, Boris Bikbov, Soufiane Boufous, Oliver Brady, Gabrielle Britton, Till Bärnighausen, Sharath

Burugina Nagaraja, Ismael Campos-Nonato, Jorge Cano, Josip Car, Felix Carvalho, Carlos Castañeda-Orjuela, Franz Castro, Ester Cerin, Vijay Kumar Chattu, Devasahayam Christopher, Luis Cámera, Abel Dadi, Anh Dang, Ahmad Daryani, Rajat Das Gupta, Jan-Walter De Neve, Dereje Demissie, Edgar Denova-Gutiérrez, Kebede Deribe, Samath Dharmaratne, Meghnath Dhimal, Govinda Dhungana, Daniel Diaz, Isaac Dipeolu, Kerrie Doyle, Eleonora Dubljanin, Andre Duraes, Claudio Dávila-Cervantes, Andem Effiong, Aziz Eftekhari, Nevine El Nahas, Maysaa El Sayed Zaki, Maha El Tantawi, Shaimaa El-Jaafary, Ziad El-Khatib, Aisha Elsharkawy, Shymaa Enany, Arash Etemadi, Wubalem Fekadu, Seyed-Mohammad Fereshtehnejad, Florian Fischer, Carsten Flohr, Nataliya Foigt, Morenike Folayan, Masoud Foroutan, Takeshi Fukumoto, Reta Gayesa, Hailay Gesesew, Paramjit Gill, Tiffany Gill, Themba Ginindza, Amador Goodridge, Sameer Gopalani, Alessandra Goulart, Bárbara Goulart, Ayman Grada, Manfred Green, Harish Gugnani, Rafael Guimarães, Rajeev Gupta, Rahul Gupta, Michael Haile, Brian Hall, Demelash Woldeyohannes Handiso, Ahmed Hasaballah, Amir Hasanzadeh, Hamid Hassen, Simon Hay, Claudiu Herteliu, Hagos Hidru, Thomas Hird, Chi Hoang, Praveen Hoogar, Naznin Hossain, Mowafa Househ, Syed Hussain, Mamusha Hussen, Segun Ibitoye, Olayinka Ilesanmi, Milena Ilic, Mohammad Hasan Imani-Nasab, Usman Iqbal, Seyed Sina Irvani, Sheikh Mohammed Shariful Islam, Rebecca Ivers, Chinwe Iwu, Mihajlo Jakovljevic, Ensiyeh Jenabi, Ravi Jha, Vivekanand Jha, Jost Jonas, Jacek Jozwiak, Ali Kabir, André Karch, Hamidreza Karimi-Sari, Habtamu Kasaye, Gbenga Kayode, Mihiretu Kebede, Yousef Khader, Nauman Khalid, Rovshan Khalilov, Ejaz Khan, Junaid Khan, Md Khan, Khaled Khatab, Mona Khater, Mohammad Khazaei, Mohammad Hossein Khosravi, Jagdish Khubchandani, Adnan Kisa, Shivakumar KM KMShivakumar, Sonali Kochhar, Tufa Kolola, Parvaiz Koul, Ai Koyanagi, Kewal Krishan, Barthelemy Kuate Defo, Nuworza Kugbey, Pushpendra Kumar, Dian Kusuma, Carlo La Vecchia, Ben Lacey, Faris Lami, Savita Lasrado, Georgy Lebedev, Paul Lee, Tsegaye Lenjebo, Sonia Lewycka, Shanshan Li, Jaifred Christian Lopez, Muhammed Magdy Abd El Razek, D. R. Mahadeshwara Prasad, Azeem Majeed, Abdullah Mamun, Navid Manafi, Mohammad Ali Mansournia, Francisco Martins-Melo, Manu Mathur, Mohsen Mazidi, Colm McAlinden, Birhanu Geta Meharie, Kala Mehta, Walter Mendoza, Ritesh Menezes, Tuomo Meretoja, Tomislav Mestrovic, Ted Miller, GK Mini, Babak Moazen, Bahram Mohajer, Dara Mohammad, Yousef Mohammad, Shafiu Mohammed, Farnam Mohebi, Ali Mokdad, Paula Moraga, Abbas Mosapour, Seyyed Meysam Mousavi, Miliva Mozaffor, Kamarul Imran Musa, Ghulam Mustafa, Saravanan Muthupandian, Ahamarshan Nagarajan, Vinay Nangia, Bruno Nascimento, Javad Nazari, Ionut Negoi, Henok Netsere, Josephine Ngunjiri, Ole Norheim, Jean Jacques Noubiap, Felix Ogbo, In-Hwan Oh, Andrew Olagunju, Bolajoko Olusanya, Obinna Onwujekwe, Doris Ortega-Altamirano, Osayomwanbo Osarenotor, Mayowa Owolabi, Mahesh P A, Jagadish Rao. Padubidri, Smita Pakhale, Veincent Christian Pepito, Norberto Perico, Julia Pescarini, Konrad Pesudovs, Thomas Pilgrim, Maarten Postma, Farshad Pourmalek, Navid Rabiee, Amir Radfar, Fakher Rahim, Mohammad Hifz Ur Rahman, Fatemeh Rajati, Chhabi Ranabhat, Davide Rasella, Goura Rath, Salman Rawaf, Lal Rawal, Wasiq Rawasia, Giuseppe Remuzzi, Vishnu Renjith, Andre Renzaho, Seyed Mohammad Riahi, Ana Isabel Ribeiro, Jennifer Rickard, Leonardo Roever, Luca Ronfani, Anas Saad, Rajesh Sagar, Nasir Salam, Hosni Salem, Marwa Salem, Yahya Salimi, Hamideh Salimzadeh, Abdallah Samy, Juan Sanabria, Itamar Santos, Milena Santric-Milicevic, Maheswar Satpathy, David Schwebel, Sadaf Sepanlou, Edson Serván -Mori, Azadeh Shafieesabet, Mehran Shams-Beyranvand, MohammadBagher Shamsi, Rajesh Sharma, Aziz Sheikh, Mika Shigematsu, Reza Shirkoohi, Tariq Siddiqi, Diego Silva, Jasvinder Singh, Balbir Singh, Eirini Skiadaresi, Anton Sokhan, Joan Soriano, Ireneous Soyiri, Chandrashekhar Sreeramareddy, Mu'awiyyah Sufiyan, Bryan Sykes, Rafael Tabarés -Seisdedos, Takahiro Tabuchi, Degena Tadesse, Bineyam Taye, Yonatal Tefera, Mohamad-Hani Temsah, Zemenu Tessema, Roman Topor-Madry, Marcos Roberto Tovani-Palone, Irfan Ullah, Bhaskaran

Unnikrishnan, Olalekan Uthman, Benjamin Uzochukwu, Santosh Varughese, Francesco Violante, Yuan-Pang Wang, Ronny Westerman, Tissa Wijeratne, Charles Wiysonge, Haileab Wolde, Dawit Wondafrash, Tewodros Wonde, Ali Yadollahpour, Seyed Hossein Yahyazadeh Jabbari, Hiroshi Yatsuya, Naohiro Yonemoto, Hebat-Allah Yousof, Zoubida Zaidi, Sojib Bin Zaman, Heather Zar

Management of the overall research enterprise (for example, through membership in the Scientific Council)

Robert C. Reiner Jr, Simon I. Hay, Aubrey J Levine, Muktar Ahmed, Saeed Amini, Meysam Behzadifar, Lalit Dandona, Aziz Eftekhari, Maysaa El Sayed Zaki, Simon Hay, Hamidreza Karimi-Sari, Amir Kasaeian, Rovshan Khalilov, Pushpendra Kumar, Muhammed Magdy Abd El Razek, Ali Mokdad, Mohsen Naghavi, Javad Nazari, Mahesh P A, Vishnu Renjith, Rajesh Sagar, Benn Sartorius, Morteza Shamsizadeh, Degena Tadesse, Roman Topor-Madry, Dawit Wondafrash

Piped Water by Country

Country	Year	Mean	Upper	Lower
**Central Europe, Eastern Europe, and Central Asia**				
Kyrgyzstan	2000	78.0%	79.1%	77.1%
Kyrgyzstan	2017	74.8%	75.8%	73.7%
Mongolia	2000	26.0%	26.8%	25.2%
Mongolia	2017	34.3%	35.7%	32.8%
Tajikistan	2000	47.8%	49.4%	46.5%
Tajikistan	2017	70.6%	72.0%	69.0%
Turkmenistan	2000	47.6%	50.2%	45.1%
Turkmenistan	2017	56.5%	58.6%	54.0%
Uzbekistan	2000	70.2%	72.0%	68.8%
Uzbekistan	2017	73.9%	75.6%	72.6%
**Latin America and Caribbean**				
Bolivia	2000	71.4%	71.7%	71.1%
Bolivia	2017	56.7%	57.4%	56.0%
Brazil	2000	77.9%	78.3%	77.6%
Brazil	2017	78.1%	78.4%	77.8%
Colombia	2000	85.0%	85.7%	84.3%
Colombia	2017	59.7%	60.6%	58.7%
Dominican Republic	2000	41.7%	51.6%	33.3%
Dominican Republic	2017	12.5%	18.3%	8.2%
Ecuador	2000	80.4%	84.4%	75.9%
Ecuador	2017	80.4%	84.1%	75.9%
El Salvador	2000	51.1%	53.8%	48.3%
El Salvador	2017	87.7%	89.2%	86.1%
Guatemala	2000	69.9%	71.6%	68.3%
Guatemala	2017	59.5%	61.5%	57.8%
Guyana	2000	48.6%	50.3%	46.9%
Guyana	2017	23.7%	24.8%	22.6%
Haiti	2000	45.2%	46.1%	44.3%
Haiti	2017	26.4%	27.3%	25.4%
Honduras	2000	68.2%	69.8%	66.4%
Honduras	2017	46.0%	47.5%	44.4%
Mexico	2000	83.5%	84.2%	82.8%
Mexico	2017	96.2%	96.5%	95.9%
Nicaragua	2000	60.2%	68.2%	51.7%
Nicaragua	2017	62.9%	70.0%	55.0%
Panama	2000	86.0%	87.7%	84.1%
Panama	2017	85.9%	87.6%	84.0%
Paraguay	2000	41.2%	43.2%	39.0%
Paraguay	2017	67.3%	68.8%	65.7%
Peru	2000	69.9%	70.5%	69.2%
Peru	2017	81.6%	82.1%	81.0%
**North Africa and Middle East**				
Afghanistan	2000	0.6%	0.9%	0.4%
Afghanistan	2017	19.7%	21.5%	17.7%
Algeria	2000	71.9%	72.2%	71.6%
Algeria	2017	71.9%	72.2%	71.5%
Egypt	2000	85.9%	86.5%	85.2%
Egypt	2017	99.2%	99.3%	99.1%
Iraq	2000	77.8%	80.4%	74.8%
Iraq	2017	59.6%	63.3%	55.6%
Jordan	2000	91.0%	91.3%	90.8%
Jordan	2017	56.1%	56.6%	55.6%
Libya	2000	58.6%	60.1%	57.2%
Libya	2017	58.5%	59.8%	57.3%
Morocco	2000	63.1%	65.2%	60.6%
Morocco	2017	63.2%	65.3%	60.8%

Country	Year	Mean	Upper	Lower
Sudan	2000	39.3%	40.0%	38.4%
Sudan	2017	26.4%	27.2%	25.6%
Syria	2000	80.9%	83.3%	77.7%
Syria	2017	80.8%	83.1%	77.4%
Tunisia	2000	66.6%	68.7%	64.2%
Tunisia	2017	66.6%	68.9%	64.1%
Yemen	2000	39.6%	41.7%	37.6%
Yemen	2017	27.7%	29.6%	25.7%
**South Asia**				
Bangladesh	2000	24.1%	25.2%	23.0%
Bangladesh	2017	7.8%	8.2%	7.4%
India	2000	38.9%	39.1%	38.7%
India	2017	45.6%	45.8%	45.4%
Nepal	2000	36.0%	38.2%	34.1%
Nepal	2017	53.8%	56.2%	51.8%
Pakistan	2000	24.2%	25.6%	22.9%
Pakistan	2017	38.3%	39.8%	36.9%
**Southeast Asia, East Asia, and Oceania**				
Cambodia	2000	4.4%	4.7%	4.1%
Cambodia	2017	53.5%	55.5%	51.7%
China	2000	37.3%	39.6%	35.1%
China	2017	74.9%	75.6%	74.0%
Indonesia	2000	20.0%	20.2%	19.8%
Indonesia	2017	9.9%	10.0%	9.7%
Laos	2000	13.3%	15.6%	11.2%
Laos	2017	11.2%	13.5%	9.5%
Myanmar	2000	18.2%	19.4%	17.1%
Myanmar	2017	12.3%	13.2%	11.5%
Papua New Guinea	2000	36.2%	38.9%	33.9%
Papua New Guinea	2017	36.2%	39.3%	33.3%
Philippines	2000	58.2%	59.4%	57.0%
Philippines	2017	30.7%	31.7%	29.6%
Sri Lanka	2000	27.4%	32.8%	22.6%
Sri Lanka	2017	35.0%	41.1%	29.9%
Thailand	2000	27.3%	27.6%	26.9%
Thailand	2017	20.1%	20.4%	19.8%
Timor-Leste	2000	36.7%	37.7%	35.5%
Timor-Leste	2017	63.8%	64.7%	62.9%
Vietnam	2000	17.3%	18.2%	16.3%
Vietnam	2017	28.5%	29.8%	27.4%
**Sub-Saharan Africa**				
Angola	2000	34.8%	37.1%	32.6%
Angola	2017	35.7%	36.9%	34.5%
Benin	2000	44.0%	45.0%	43.0%
Benin	2017	35.9%	37.0%	34.9%
Botswana	2000	93.4%	93.8%	93.0%
Botswana	2017	92.8%	93.3%	92.3%
Burkina Faso	2000	26.0%	27.9%	24.3%
Burkina Faso	2017	22.5%	24.0%	21.2%
Burundi	2000	17.7%	18.4%	17.1%
Burundi	2017	36.9%	37.7%	36.1%
Cameroon	2000	34.1%	35.6%	32.8%
Cameroon	2017	34.6%	36.2%	33.3%
Central African Republic	2000	18.2%	21.0%	15.1%

Country	Year	Mean	Upper	Lower
Central African Republic	2017	10.7%	13.4%	8.2%
Chad	2000	15.1%	16.1%	14.1%
Chad	2017	15.1%	16.2%	14.2%
Comoros	2000	64.5%	66.1%	63.1%
Comoros	2017	64.6%	66.2%	63.0%
Côte d'Ivoire	2000	34.6%	36.0%	33.3%
Côte d'Ivoire	2017	46.6%	48.4%	45.0%
Democratic Republic of the Congo	2000	25.9%	27.0%	24.8%
Democratic Republic of the Congo	2017	14.9%	15.6%	14.0%
Eritrea	2000	38.0%	39.8%	36.2%
Eritrea	2017	38.0%	39.8%	36.3%
Ethiopia	2000	25.3%	26.1%	24.3%
Ethiopia	2017	41.0%	42.1%	39.8%
Gabon	2000	60.4%	63.9%	56.4%
Gabon	2017	64.1%	67.7%	59.8%
Gambia	2000	56.1%	64.1%	48.2%
Gambia	2017	70.2%	77.8%	61.3%
Ghana	2000	27.3%	28.1%	26.5%
Ghana	2017	30.4%	31.2%	29.7%
Guinea	2000	19.2%	20.5%	17.7%
Guinea	2017	26.9%	28.4%	25.1%
Guinea-Bissau	2000	25.4%	38.2%	17.1%
Guinea-Bissau	2017	23.1%	37.4%	14.7%
Kenya	2000	30.2%	30.7%	29.7%
Kenya	2017	30.2%	30.9%	29.6%
Lesotho	2000	55.8%	57.1%	54.5%
Lesotho	2017	70.7%	72.0%	69.2%
Liberia	2000	1.6%	2.1%	1.3%
Liberia	2017	15.7%	16.6%	14.5%
Madagascar	2000	31.2%	33.1%	29.3%
Madagascar	2017	22.2%	23.5%	20.7%
Malawi	2000	27.3%	27.9%	26.7%
Malawi	2017	30.4%	31.0%	29.8%
Mali	2000	29.8%	31.0%	28.4%
Mali	2017	36.9%	37.9%	35.7%
Mauritania	2000	33.6%	37.7%	30.3%
Mauritania	2017	56.7%	61.1%	52.6%
Mozambique	2000	24.6%	25.9%	23.4%
Mozambique	2017	46.1%	47.8%	44.4%
Namibia	2000	67.7%	69.3%	66.3%
Namibia	2017	76.7%	78.0%	75.5%
Niger	2000	23.5%	24.4%	22.7%
Niger	2017	47.6%	48.5%	46.6%
Nigeria	2000	21.1%	21.8%	20.5%
Nigeria	2017	10.2%	10.5%	9.8%
Republic of Congo	2000	33.4%	40.1%	28.7%
Republic of Congo	2017	29.2%	35.2%	24.4%
Rwanda	2000	34.3%	34.9%	33.8%

Country	Year	Mean	Upper	Lower
Rwanda	2017	34.6%	35.3%	34.0%
Senegal	2000	44.2%	45.3%	43.2%
Senegal	2017	58.0%	59.1%	57.0%
Sierra Leone	2000	31.0%	32.6%	29.6%
Sierra Leone	2017	13.1%	14.0%	12.3%
Somalia	2000	20.3%	24.1%	16.8%
Somalia	2017	37.0%	42.3%	32.2%
South Africa	2000	79.2%	79.5%	79.0%
South Africa	2017	86.2%	86.4%	86.0%
South Sudan	2000	20.9%	23.1%	19.0%
South Sudan	2017	1.5%	1.7%	1.3%
Swaziland	2000	43.3%	45.2%	41.6%
Swaziland	2017	58.1%	60.2%	55.9%
Tanzania	2000	28.8%	29.8%	27.6%
Tanzania	2017	45.6%	46.8%	44.4%
Togo	2000	33.1%	37.2%	29.2%
Togo	2017	26.5%	30.1%	23.5%
Uganda	2000	12.8%	13.4%	12.3%
Uganda	2017	20.5%	21.2%	19.9%
Zambia	2000	29.5%	30.9%	28.3%
Zambia	2017	28.9%	30.2%	27.8%
Zimbabwe	2000	34.2%	35.6%	32.8%
Zimbabwe	2017	33.8%	35.1%	32.6%

Improved Water Proportion by Country

Country	Year	Mean	Upper	Lower
**Central Europe, Eastern Europe, and Central Asia**				
Kyrgyzstan	2000	89.4%	90.7%	88.0%
Kyrgyzstan	2017	85.8%	87.1%	84.4%
Mongolia	2000	54.7%	55.8%	53.7%
Mongolia	2017	84.9%	85.7%	84.0%
Tajikistan	2000	60.6%	62.2%	59.1%
Tajikistan	2017	85.3%	86.6%	84.2%
Turkmenistan	2000	86.9%	88.4%	84.9%
Turkmenistan	2017	99.4%	99.5%	99.3%
Uzbekistan	2000	93.1%	93.5%	92.5%
Uzbekistan	2017	99.6%	99.7%	99.6%
**Latin America and Caribbean**				
Bolivia	2000	80.8%	81.2%	80.5%
Bolivia	2017	87.7%	88.1%	87.4%
Brazil	2000	94.7%	94.9%	94.6%
Brazil	2017	94.7%	94.9%	94.6%
Colombia	2000	88.4%	88.9%	87.8%
Colombia	2017	82.0%	82.6%	81.4%
Dominican Republic	2000	93.6%	97.0%	87.1%
Dominican Republic	2017	98.4%	99.4%	95.9%
Ecuador	2000	88.3%	90.9%	85.4%
Ecuador	2017	88.2%	90.8%	85.4%
El Salvador	2000	61.4%	64.0%	58.4%
El Salvador	2017	95.9%	96.7%	94.9%
Guatemala	2000	84.0%	85.3%	82.6%
Guatemala	2017	94.7%	95.4%	93.9%
Guyana	2000	78.9%	80.9%	76.5%
Guyana	2017	94.1%	94.9%	93.1%
Haiti	2000	60.0%	61.1%	59.0%
Haiti	2017	68.3%	69.4%	67.2%
Honduras	2000	80.1%	81.4%	78.6%
Honduras	2017	93.7%	94.4%	93.0%
Mexico	2000	92.0%	92.5%	91.5%
Mexico	2017	99.0%	99.1%	98.9%
Nicaragua	2000	77.7%	81.5%	73.4%
Nicaragua	2017	78.5%	81.8%	74.7%
Panama	2000	86.7%	88.3%	84.8%
Panama	2017	86.5%	88.2%	84.6%
Paraguay	2000	81.0%	81.8%	79.9%
Paraguay	2017	95.2%	95.5%	94.8%
Peru	2000	74.4%	75.0%	73.7%
Peru	2017	92.2%	92.5%	91.8%
**North Africa and Middle East**				
Afghanistan	2000	15.2%	16.4%	13.9%
Afghanistan	2017	64.8%	66.6%	62.8%
Algeria	2000	98.5%	98.6%	98.5%
Algeria	2017	98.6%	98.6%	98.5%
Egypt	2000	99.7%	99.7%	99.6%
Egypt	2017	100.0%	100.0%	100.0%
Iraq	2000	84.1%	86.3%	81.6%
Iraq	2017	98.8%	99.1%	98.6%
Jordan	2000	99.7%	99.8%	99.7%
Jordan	2017	99.7%	99.7%	99.6%
Libya	2000	93.4%	93.9%	92.9%
Libya	2017	93.3%	93.9%	92.8%
Morocco	2000	75.8%	78.1%	73.7%
Morocco	2017	75.7%	77.8%	73.5%

Country	Year	Mean	Upper	Lower
Sudan	2000	66.4%	67.1%	65.8%
Sudan	2017	75.8%	76.6%	75.0%
Syria	2000	99.6%	99.7%	99.3%
Syria	2017	99.6%	99.7%	99.3%
Tunisia	2000	96.7%	98.0%	94.1%
Tunisia	2017	96.6%	98.0%	93.6%
Yemen	2000	58.8%	60.9%	56.8%
Yemen	2017	92.6%	93.4%	91.6%
**South Asia**				
Bangladesh	2000	97.7%	98.0%	97.4%
Bangladesh	2017	94.9%	95.5%	94.1%
India	2000	85.1%	85.3%	85.0%
India	2017	91.8%	91.9%	91.6%
Nepal	2000	88.6%	90.1%	87.3%
Nepal	2017	97.3%	97.8%	96.8%
Pakistan	2000	54.0%	55.1%	52.8%
Pakistan	2017	94.6%	95.2%	93.9%
**Southeast Asia, East Asia, and Oceania**				
Cambodia	2000	36.1%	37.6%	34.6%
Cambodia	2017	92.1%	93.2%	90.9%
China	2000	97.1%	97.8%	96.3%
China	2017	88.8%	89.4%	88.2%
Indonesia	2000	73.3%	73.5%	73.2%
Indonesia	2017	84.1%	84.3%	84.0%
Laos	2000	46.8%	49.7%	43.7%
Laos	2017	83.9%	85.9%	81.7%
Myanmar	2000	61.4%	62.8%	59.8%
Myanmar	2017	82.9%	83.9%	81.9%
Papua New Guinea	2000	67.7%	70.5%	64.9%
Papua New Guinea	2017	68.2%	71.2%	65.2%
Philippines	2000	84.0%	85.0%	83.0%
Philippines	2017	94.0%	94.4%	93.5%
Sri Lanka	2000	80.3%	83.7%	77.0%
Sri Lanka	2017	90.3%	92.3%	88.4%
Thailand	2000	92.0%	92.5%	91.5%
Thailand	2017	98.8%	98.9%	98.7%
Timor-Leste	2000	67.9%	69.0%	66.7%
Timor-Leste	2017	83.9%	84.5%	83.3%
Vietnam	2000	80.5%	81.7%	79.3%
Vietnam	2017	91.9%	92.6%	91.4%
**Sub-Saharan Africa**				
Angola	2000	64.4%	66.4%	62.4%
Angola	2017	61.6%	62.7%	60.4%
Benin	2000	55.9%	57.1%	54.8%
Benin	2017	69.1%	70.2%	68.0%
Botswana	2000	97.0%	97.2%	96.7%
Botswana	2017	98.1%	98.3%	98.0%
Burkina Faso	2000	72.2%	73.9%	70.3%
Burkina Faso	2017	73.4%	74.9%	71.7%
Burundi	2000	80.4%	81.3%	79.6%
Burundi	2017	85.7%	86.5%	84.9%
Cameroon	2000	54.3%	55.9%	52.9%
Cameroon	2017	74.0%	75.3%	72.5%
Central African Republic	2000	64.7%	65.9%	63.4%

Country	Year	Mean	Upper	Lower
Central African Republic	2017	56.8%	58.2%	55.4%
Chad	2000	47.4%	48.9%	45.8%
Chad	2017	54.2%	55.7%	52.7%
Comoros	2000	94.8%	97.0%	91.2%
Comoros	2017	94.9%	97.1%	91.7%
Côte d'Ivoire	2000	80.5%	81.8%	79.1%
Côte d'Ivoire	2017	77.9%	79.2%	76.6%
Democratic Republic of the Congo	2000	44.8%	46.0%	43.6%
Democratic Republic of the Congo	2017	37.9%	39.3%	36.6%
Eritrea	2000	70.1%	71.1%	69.3%
Eritrea	2017	70.2%	71.3%	69.3%
Ethiopia	2000	33.6%	34.5%	32.7%
Ethiopia	2017	70.5%	71.4%	69.6%
Gabon	2000	72.2%	74.9%	69.1%
Gabon	2017	86.5%	88.8%	84.4%
Gambia	2000	86.7%	91.6%	81.2%
Gambia	2017	90.0%	94.9%	83.2%
Ghana	2000	64.7%	65.5%	63.7%
Ghana	2017	88.1%	88.7%	87.6%
Guinea	2000	52.9%	55.5%	50.1%
Guinea	2017	80.1%	81.8%	78.1%
Guinea-Bissau	2000	64.3%	74.8%	54.3%
Guinea-Bissau	2017	55.4%	69.1%	44.1%
Kenya	2000	49.4%	49.9%	48.9%
Kenya	2017	58.7%	59.3%	58.1%
Lesotho	2000	69.2%	70.3%	68.0%
Lesotho	2017	82.4%	83.4%	81.3%
Liberia	2000	31.1%	34.0%	28.0%
Liberia	2017	89.1%	90.6%	87.3%
Madagascar	2000	36.1%	37.8%	34.1%
Madagascar	2017	48.7%	50.1%	47.2%
Malawi	2000	67.0%	67.6%	66.5%
Malawi	2017	88.0%	88.4%	87.6%
Mali	2000	41.6%	42.8%	40.2%
Mali	2017	69.5%	70.6%	68.3%
Mauritania	2000	74.4%	76.0%	72.7%
Mauritania	2017	82.3%	83.8%	80.7%
Mozambique	2000	46.8%	48.5%	45.0%
Mozambique	2017	55.8%	57.4%	54.2%
Namibia	2000	80.5%	81.8%	79.4%
Namibia	2017	83.4%	84.5%	82.3%
Niger	2000	45.3%	46.1%	44.6%
Niger	2017	75.3%	76.1%	74.7%
Nigeria	2000	45.4%	46.1%	44.6%
Nigeria	2017	69.1%	69.6%	68.6%
Republic of Congo	2000	39.4%	45.7%	34.9%
Republic of Congo	2017	74.5%	78.2%	70.5%
Rwanda	2000	65.6%	66.0%	65.1%

Improved Water Proportion by Country *(continued)*

Country	Year	Mean	Upper	Lower
Rwanda	2017	76.5%	76.9%	76.0%
Senegal	2000	59.7%	60.9%	58.8%
Senegal	2017	70.6%	71.7%	69.7%
Sierra Leone	2000	54.7%	56.7%	52.7%
Sierra Leone	2017	62.2%	64.3%	60.1%
Somalia	2000	39.7%	42.9%	36.5%
Somalia	2017	94.3%	95.1%	93.4%
South Africa	2000	83.2%	83.4%	83.0%
South Africa	2017	92.4%	92.5%	92.2%
South Sudan	2000	37.1%	38.9%	35.4%
South Sudan	2017	36.7%	37.5%	35.9%
Swaziland	2000	54.4%	56.5%	52.2%
Swaziland	2017	76.2%	78.3%	73.7%
Tanzania	2000	42.3%	43.4%	41.0%
Tanzania	2017	68.5%	69.7%	67.3%
Togo	2000	46.3%	50.4%	42.2%
Togo	2017	68.0%	73.7%	61.6%
Uganda	2000	61.8%	62.9%	61.0%
Uganda	2017	79.6%	80.6%	78.6%
Zambia	2000	41.6%	43.2%	40.2%
Zambia	2017	64.1%	65.5%	62.6%
Zimbabwe	2000	68.7%	70.3%	66.9%
Zimbabwe	2017	78.0%	79.3%	76.5%

Piped Water Proportion by Second Administrative Unit

Country	Second Administrative Unit	Year	Mean	Upper	Lower
**Central Europe, Eastern Europe, and Central Asia**					
Kyrgyzstan	Ak-Suu	2000	78.9%	89.0%	57.8%
Kyrgyzstan	Ak-Suu	2017	74.9%	86.1%	55.6%
Kyrgyzstan	Ak-Talaa	2000	55.2%	59.3%	51.3%
Kyrgyzstan	Ak-Talaa	2017	50.0%	55.7%	45.8%
Kyrgyzstan	Aksyi	2000	97.4%	98.5%	95.3%
Kyrgyzstan	Aksyi	2017	96.0%	97.8%	92.9%
Kyrgyzstan	Ala-Buka	2000	77.3%	85.4%	69.7%
Kyrgyzstan	Ala-Buka	2017	72.0%	81.2%	64.2%
Kyrgyzstan	Alai	2000	63.7%	82.1%	46.5%
Kyrgyzstan	Alai	2017	63.2%	78.3%	49.2%
Kyrgyzstan	Alamüdün	2000	81.7%	81.9%	81.6%
Kyrgyzstan	Alamüdün	2017	82.1%	82.5%	81.2%
Kyrgyzstan	Aravan	2000	84.0%	86.9%	80.4%
Kyrgyzstan	Aravan	2017	83.9%	87.9%	75.5%
Kyrgyzstan	At-Bashi	2000	49.6%	52.3%	46.3%
Kyrgyzstan	At-Bashi	2017	48.8%	51.3%	46.0%
Kyrgyzstan	Bakai-Ata	2000	74.9%	81.7%	67.3%
Kyrgyzstan	Bakai-Ata	2017	63.0%	70.4%	54.9%
Kyrgyzstan	Batken	2000	59.8%	73.5%	43.5%
Kyrgyzstan	Batken	2017	49.5%	65.3%	32.7%
Kyrgyzstan	Bazar-Korgon	2000	91.1%	94.1%	86.9%
Kyrgyzstan	Bazar-Korgon	2017	87.7%	91.5%	82.4%
Kyrgyzstan	Biškek	2000	70.5%	70.5%	70.4%
Kyrgyzstan	Biškek	2017	68.9%	69.1%	68.5%
Kyrgyzstan	Chatkal	2000	66.6%	85.7%	47.3%
Kyrgyzstan	Chatkal	2017	59.8%	79.9%	39.3%
Kyrgyzstan	Chong-Alay	2000	52.4%	99.4%	2.1%
Kyrgyzstan	Chong-Alay	2017	48.8%	98.9%	1.1%
Kyrgyzstan	Chui	2000	63.1%	64.4%	62.7%
Kyrgyzstan	Chui	2017	62.5%	63.0%	61.9%
Kyrgyzstan	Djety-Oguz	2000	69.7%	81.8%	60.8%
Kyrgyzstan	Djety-Oguz	2017	68.1%	79.1%	60.3%
Kyrgyzstan	Jaiyl	2000	79.2%	87.8%	71.7%
Kyrgyzstan	Jaiyl	2017	79.4%	86.0%	71.8%
Kyrgyzstan	Jumgal	2000	50.4%	50.5%	50.4%
Kyrgyzstan	Jumgal	2017	53.1%	53.1%	53.0%
Kyrgyzstan	Kadamjai	2000	71.3%	78.9%	64.7%
Kyrgyzstan	Kadamjai	2017	64.2%	71.3%	57.3%
Kyrgyzstan	Kara-Buura	2000	87.9%	92.2%	82.8%
Kyrgyzstan	Kara-Buura	2017	82.8%	88.0%	77.6%
Kyrgyzstan	Kara-Kuldja	2000	73.4%	90.3%	59.0%
Kyrgyzstan	Kara-Kuldja	2017	68.5%	86.7%	53.8%
Kyrgyzstan	Kara-Suu	2000	88.5%	89.5%	87.5%
Kyrgyzstan	Kara-Suu	2017	84.2%	85.6%	82.8%
Kyrgyzstan	Kemin	2000	85.1%	90.1%	79.4%
Kyrgyzstan	Kemin	2017	82.2%	88.2%	76.2%
Kyrgyzstan	Kochkor	2000	75.7%	78.3%	73.0%
Kyrgyzstan	Kochkor	2017	74.8%	77.8%	71.9%
Kyrgyzstan	Lailak	2000	79.5%	84.3%	73.3%
Kyrgyzstan	Lailak	2017	73.5%	79.2%	67.0%
Kyrgyzstan	Manas	2000	55.7%	63.8%	47.5%
Kyrgyzstan	Manas	2017	43.7%	54.6%	34.2%
Kyrgyzstan	Moskovsky	2000	80.4%	84.7%	75.6%
Kyrgyzstan	Moskovsky	2017	76.7%	81.8%	71.7%
Kyrgyzstan	Naryn	2000	70.8%	76.3%	66.8%
Kyrgyzstan	Naryn	2017	68.3%	71.7%	62.6%
Kyrgyzstan	Nookat	2000	75.8%	79.3%	72.7%
Kyrgyzstan	Nookat	2017	71.8%	75.7%	68.5%
Kyrgyzstan	Nooken	2000	87.7%	90.5%	83.2%
Kyrgyzstan	Nooken	2017	84.3%	88.5%	78.4%
Kyrgyzstan	Osh	2000	99.7%	99.9%	99.5%

Country	Second Administrative Unit	Year	Mean	Upper	Lower
Kyrgyzstan	Osh	2017	98.6%	99.4%	97.5%
Kyrgyzstan	Panfilov	2000	94.3%	95.4%	93.7%
Kyrgyzstan	Panfilov	2017	93.6%	94.8%	93.0%
Kyrgyzstan	Sokuluk	2000	92.0%	93.6%	89.8%
Kyrgyzstan	Sokuluk	2017	90.8%	92.8%	88.7%
Kyrgyzstan	Song-Kol	2000	100.0%	100.0%	100.0%
Kyrgyzstan	Song-Kol	2017	100.0%	100.0%	100.0%
Kyrgyzstan	Suzak	2000	86.5%	89.1%	83.1%
Kyrgyzstan	Suzak	2017	81.0%	84.5%	76.9%
Kyrgyzstan	Talas	2000	65.9%	71.5%	58.0%
Kyrgyzstan	Talas	2017	53.3%	59.8%	45.8%
Kyrgyzstan	Togus-Toro	2000	67.0%	76.1%	52.8%
Kyrgyzstan	Togus-Toro	2017	65.6%	76.5%	50.0%
Kyrgyzstan	Toktogul	2000	89.9%	94.8%	84.3%
Kyrgyzstan	Toktogul	2017	86.5%	92.0%	79.9%
Kyrgyzstan	Ton	2000	85.5%	89.0%	81.7%
Kyrgyzstan	Ton	2017	85.0%	88.2%	81.6%
Kyrgyzstan	Tüp	2000	92.2%	96.7%	82.6%
Kyrgyzstan	Tüp	2017	90.2%	95.8%	80.7%
Kyrgyzstan	Uzgen	2000	64.0%	69.0%	58.8%
Kyrgyzstan	Uzgen	2017	58.0%	63.4%	53.2%
Kyrgyzstan	Ysyk-Ata	2000	69.8%	71.3%	67.7%
Kyrgyzstan	Ysyk-Ata	2017	70.3%	73.0%	67.5%
Kyrgyzstan	Ysyk-Köl	2000	89.4%	93.4%	80.6%
Kyrgyzstan	Ysyk-Köl	2017	85.4%	90.5%	77.0%
Kyrgyzstan	Ysyk-Köl (lake)	2000	79.8%	82.1%	77.2%
Kyrgyzstan	Ysyk-Köl (lake)	2017	78.0%	81.5%	74.4%
Mongolia	Adaatsag	2000	6.7%	11.2%	3.2%
Mongolia	Adaatsag	2017	7.6%	14.5%	3.4%
Mongolia	Airag	2000	11.3%	15.9%	8.1%
Mongolia	Airag	2000	11.6%	17.7%	7.6%
Mongolia	Airag	2017	13.7%	21.0%	8.8%
Mongolia	Airag	2017	13.4%	20.0%	8.2%
Mongolia	Alag-Erdene	2000	14.0%	22.5%	8.3%
Mongolia	Alag-Erdene	2017	16.8%	28.9%	8.8%
Mongolia	Aldarkhaan	2000	10.9%	14.5%	7.9%
Mongolia	Aldarkhaan	2017	12.5%	16.1%	9.3%
Mongolia	Altai	2000	6.0%	8.2%	4.1%
Mongolia	Altai	2000	3.3%	5.8%	1.3%
Mongolia	Altai	2000	7.2%	11.0%	4.5%
Mongolia	Altai	2017	7.5%	10.9%	4.8%
Mongolia	Altai	2017	3.9%	8.7%	1.2%
Mongolia	Altai	2017	10.2%	18.9%	4.9%
Mongolia	Altanbulag	2000	28.4%	35.7%	23.9%
Mongolia	Altanbulag	2000	20.2%	39.4%	8.4%
Mongolia	Altanbulag	2017	30.1%	63.5%	8.4%
Mongolia	Altanbulag	2017	32.8%	41.7%	27.3%
Mongolia	Altanshiree	2000	30.4%	35.4%	25.5%
Mongolia	Altanshiree	2017	19.4%	27.9%	13.9%
Mongolia	Altantsögts	2000	6.2%	13.9%	2.2%
Mongolia	Altantsögts	2017	7.7%	16.7%	2.7%
Mongolia	Arbulag	2000	12.5%	19.0%	7.5%
Mongolia	Arbulag	2017	15.3%	24.5%	8.3%
Mongolia	Argalant	2000	20.3%	41.2%	6.3%
Mongolia	Argalant	2017	20.4%	41.7%	7.3%
Mongolia	Arkhust	2000	18.7%	39.8%	6.3%
Mongolia	Arkhust	2017	21.7%	43.2%	7.3%
Mongolia	Arvaikheer	2000	5.4%	7.5%	3.7%
Mongolia	Arvaikheer	2017	5.0%	6.8%	3.5%
Mongolia	Asgat	2000	9.4%	21.1%	2.8%

Country	Second Administrative Unit	Year	Mean	Upper	Lower
Mongolia	Asgat	2000	7.3%	11.9%	4.0%
Mongolia	Asgat	2017	10.9%	26.1%	3.4%
Mongolia	Asgat	2017	8.4%	15.5%	4.4%
Mongolia	Baatsagaan	2000	7.2%	11.6%	3.8%
Mongolia	Baatsagaan	2017	8.7%	15.0%	4.1%
Mongolia	Baruun Bayan-Ulaan	2000	6.4%	11.5%	2.9%
Mongolia	Baruun Bayan-Ulaan	2017	7.8%	16.7%	3.2%
Mongolia	Baruunbüren	2000	17.0%	28.0%	8.4%
Mongolia	Baruunbüren	2017	20.3%	33.5%	10.5%
Mongolia	Baruuntutuun	2000	13.1%	27.8%	5.9%
Mongolia	Baruuntutuun	2017	16.0%	35.8%	6.0%
Mongolia	Bat-Ölzii	2000	8.7%	16.1%	4.2%
Mongolia	Bat-Ölzii	2017	9.9%	20.4%	4.3%
Mongolia	Batnorov	2000	19.8%	26.7%	13.9%
Mongolia	Batnorov	2017	24.3%	32.8%	18.1%
Mongolia	Batshireet	2000	13.2%	19.6%	8.0%
Mongolia	Batshireet	2017	15.1%	23.6%	8.5%
Mongolia	Batsümber	2000	21.8%	41.0%	9.1%
Mongolia	Batsümber	2017	22.2%	36.3%	11.1%
Mongolia	Battsengel	2000	10.2%	20.8%	4.7%
Mongolia	Battsengel	2017	11.9%	26.4%	5.1%
Mongolia	Bayan	2000	16.2%	41.5%	4.1%
Mongolia	Bayan	2017	18.4%	53.8%	3.1%
Mongolia	Bayan-Adarga	2000	12.6%	21.6%	6.5%
Mongolia	Bayan-Adarga	2017	13.5%	23.7%	6.6%
Mongolia	Bayan-Agt	2000	15.5%	24.7%	9.6%
Mongolia	Bayan-Agt	2017	16.8%	26.1%	10.3%
Mongolia	Bayan-Öndör	2000	7.2%	12.4%	3.8%
Mongolia	Bayan-Öndör	2000	2.3%	3.8%	1.2%
Mongolia	Bayan-Öndör	2000	29.0%	32.1%	26.0%
Mongolia	Bayan-Öndör	2017	33.3%	37.2%	29.8%
Mongolia	Bayan-Öndör	2017	2.9%	5.3%	1.4%
Mongolia	Bayan-Öndör	2017	7.8%	17.0%	2.9%
Mongolia	Bayan-Önjüül	2000	7.5%	12.0%	4.1%
Mongolia	Bayan-Önjüül	2017	8.8%	16.2%	4.4%
Mongolia	Bayan-Ovoo	2000	17.0%	26.8%	9.8%
Mongolia	Bayan-Ovoo	2000	8.5%	14.8%	4.3%
Mongolia	Bayan-Ovoo	2000	9.3%	13.6%	6.1%
Mongolia	Bayan-Ovoo	2017	19.9%	35.8%	9.4%
Mongolia	Bayan-Ovoo	2017	10.1%	18.6%	4.9%
Mongolia	Bayan-Ovoo	2017	10.8%	16.6%	6.4%
Mongolia	Bayan-Uul	2000	6.2%	12.1%	3.0%
Mongolia	Bayan-Uul	2000	14.9%	21.1%	9.8%
Mongolia	Bayan-Uul	2017	7.4%	17.1%	3.2%
Mongolia	Bayan-Uul	2017	17.1%	27.1%	9.9%
Mongolia	Bayanbulag	2000	5.0%	9.8%	1.9%
Mongolia	Bayanbulag	2017	5.8%	15.0%	2.0%
Mongolia	Bayanchandmani	2000	11.1%	20.5%	5.7%
Mongolia	Bayanchandmani	2017	14.2%	23.4%	8.3%
Mongolia	Bayandalai	2000	7.0%	10.4%	4.0%
Mongolia	Bayandalai	2017	8.2%	15.4%	4.2%
Mongolia	Bayandelger	2000	8.9%	13.6%	5.5%
Mongolia	Bayandelger	2000	17.6%	32.1%	8.4%
Mongolia	Bayandelger	2017	11.3%	18.2%	6.9%
Mongolia	Bayandelger	2017	24.0%	41.7%	11.4%
Mongolia	Bayandun	2000	11.2%	16.3%	7.3%
Mongolia	Bayandun	2017	13.4%	21.3%	8.1%
Mongolia	Bayangol	2000	7.1%	12.3%	3.7%

Country	Second Administrative Unit	Year	Mean	Upper	Lower
Mongolia	Bayangol	2000	17.2%	35.4%	6.6%
Mongolia	Bayangol	2017	7.9%	17.4%	3.3%
Mongolia	Bayangol	2017	20.1%	37.8%	8.6%
Mongolia	Bayangovi	2000	4.6%	9.6%	2.1%
Mongolia	Bayangovi	2017	6.0%	15.8%	1.9%
Mongolia	Bayanjargalan	2000	9.4%	16.1%	4.9%
Mongolia	Bayanjargalan	2000	9.2%	16.8%	4.6%
Mongolia	Bayanjargalan	2017	10.7%	20.4%	5.2%
Mongolia	Bayanjargalan	2017	11.2%	21.8%	5.3%
Mongolia	Bayankhairkhan	2000	12.2%	19.9%	6.1%
Mongolia	Bayankhairkhan	2017	14.1%	22.9%	6.7%
Mongolia	Bayankhangai	2000	19.4%	45.1%	4.6%
Mongolia	Bayankhangai	2017	21.9%	47.0%	6.0%
Mongolia	Bayankhongor	2000	17.0%	18.7%	15.2%
Mongolia	Bayankhongor	2017	20.6%	22.5%	18.5%
Mongolia	Bayankhutag	2000	12.6%	17.7%	9.1%
Mongolia	Bayankhutag	2017	14.3%	18.8%	10.6%
Mongolia	Bayanlig	2000	5.4%	9.2%	3.1%
Mongolia	Bayanlig	2017	6.4%	14.3%	2.7%
Mongolia	Bayanmönkh	2000	7.7%	14.1%	3.3%
Mongolia	Bayanmönkh	2017	9.2%	17.3%	4.2%
Mongolia	Bayannuur	2000	10.5%	22.5%	3.4%
Mongolia	Bayannuur	2000	5.2%	9.0%	2.3%
Mongolia	Bayannuur	2017	10.7%	31.5%	2.3%
Mongolia	Bayannuur	2017	7.3%	14.2%	2.6%
Mongolia	Bayantal	2000	16.9%	40.0%	5.8%
Mongolia	Bayantal	2017	22.8%	52.4%	7.7%
Mongolia	Bayantes	2000	13.6%	19.3%	8.5%
Mongolia	Bayantes	2017	17.5%	30.2%	8.9%
Mongolia	Bayantsagaan	2000	5.7%	9.1%	3.1%
Mongolia	Bayantsagaan	2000	8.3%	14.6%	4.4%
Mongolia	Bayantsagaan	2017	9.2%	15.8%	4.8%
Mongolia	Bayantsagaan	2017	6.7%	11.8%	3.2%
Mongolia	Bayantsogt	2000	16.8%	30.9%	7.1%
Mongolia	Bayantsogt	2017	19.8%	35.9%	8.2%
Mongolia	Bayantümen	2000	38.2%	43.6%	33.9%
Mongolia	Bayantümen	2017	43.0%	53.2%	34.4%
Mongolia	Bayanzürkh	2000	7.4%	12.3%	3.8%
Mongolia	Bayanzürkh	2017	8.7%	18.2%	4.0%
Mongolia	Biger	2000	7.0%	11.5%	3.3%
Mongolia	Biger	2017	9.8%	17.5%	4.9%
Mongolia	Binder	2000	13.9%	19.3%	9.3%
Mongolia	Binder	2017	15.6%	21.3%	10.3%
Mongolia	Bogd	2000	5.6%	9.0%	3.4%
Mongolia	Bogd	2000	6.4%	10.8%	3.2%
Mongolia	Bogd	2017	6.5%	10.9%	3.7%
Mongolia	Bogd	2017	7.9%	13.8%	3.6%
Mongolia	Bökhmörön	2000	6.1%	11.8%	2.9%
Mongolia	Bökhmörön	2017	7.3%	13.4%	3.4%
Mongolia	Bömbögör	2000	6.4%	11.2%	2.9%
Mongolia	Bömbögör	2017	7.8%	14.2%	3.3%
Mongolia	Bornuur	2000	10.6%	29.1%	2.8%
Mongolia	Bornuur	2017	13.4%	29.8%	4.6%
Mongolia	Bugat	2000	18.5%	34.5%	9.5%
Mongolia	Bugat	2000	8.0%	11.8%	4.8%
Mongolia	Bugat	2000	7.8%	11.0%	5.8%
Mongolia	Bugat	2017	9.7%	14.6%	5.4%
Mongolia	Bugat	2017	9.4%	13.5%	6.8%
Mongolia	Bugat	2017	22.6%	35.1%	12.8%
Mongolia	Bulgan	2000	11.1%	21.7%	4.4%
Mongolia	Bulgan	2000	4.8%	9.1%	2.1%
Mongolia	Bulgan	2000	8.3%	15.4%	4.3%

Country	Second Administrative Unit	Year	Mean	Upper	Lower
Mongolia	Bulgan	2000	8.7%	14.0%	5.2%
Mongolia	Bulgan	2000	10.3%	15.8%	6.5%
Mongolia	Bulgan	2017	10.4%	23.8%	4.2%
Mongolia	Bulgan	2017	13.1%	21.6%	7.3%
Mongolia	Bulgan	2017	5.3%	10.0%	2.5%
Mongolia	Bulgan	2017	12.8%	27.1%	4.7%
Mongolia	Bulgan	2017	9.8%	16.8%	5.4%
Mongolia	Bürd	2000	13.1%	22.9%	6.7%
Mongolia	Bürd	2017	14.8%	30.5%	5.0%
Mongolia	Büregkhangai	2000	17.5%	31.4%	8.5%
Mongolia	Büregkhangai	2017	20.0%	34.5%	9.0%
Mongolia	Büren	2000	7.4%	13.7%	3.5%
Mongolia	Büren	2017	9.1%	19.7%	3.8%
Mongolia	Bürentogtokh	2000	15.9%	25.0%	8.9%
Mongolia	Bürentogtokh	2017	18.2%	31.0%	9.4%
Mongolia	Buutsagaan	2000	6.4%	11.4%	3.0%
Mongolia	Buutsagaan	2017	7.0%	13.9%	2.8%
Mongolia	Buyant	2000	21.8%	23.7%	19.5%
Mongolia	Buyant	2000	4.7%	12.2%	1.4%
Mongolia	Buyant	2017	5.9%	15.0%	1.7%
Mongolia	Buyant	2017	22.3%	25.0%	19.8%
Mongolia	Chandmani	2000	5.3%	8.7%	3.1%
Mongolia	Chandmani	2000	6.7%	10.8%	3.3%
Mongolia	Chandmani	2017	6.0%	10.7%	3.3%
Mongolia	Chandmani	2017	8.0%	14.1%	3.9%
Mongolia	Chandmani-Öndör	2000	12.8%	20.5%	7.2%
Mongolia	Chandmani-Öndör	2017	14.3%	21.8%	8.5%
Mongolia	Choibalsan	2000	16.4%	24.7%	7.9%
Mongolia	Choibalsan	2017	19.1%	29.9%	9.3%
Mongolia	Chuluunkhoroot	2000	18.3%	29.3%	11.3%
Mongolia	Chuluunkhoroot	2017	20.3%	35.9%	11.2%
Mongolia	Chuluut	2000	5.8%	10.7%	2.7%
Mongolia	Chuluut	2017	7.5%	15.8%	3.2%
Mongolia	Dadal	2000	14.0%	22.4%	8.0%
Mongolia	Dadal	2017	15.6%	23.0%	9.1%
Mongolia	Dalanjargalan	2000	11.2%	17.1%	6.7%
Mongolia	Dalanjargalan	2017	13.2%	21.9%	7.4%
Mongolia	Dalanzadgad	2000	56.6%	63.9%	49.5%
Mongolia	Dalanzadgad	2017	65.9%	74.2%	55.8%
Mongolia	Dariganga	2000	8.5%	13.5%	4.8%
Mongolia	Dariganga	2017	10.1%	20.4%	5.3%
Mongolia	Darkhan	2000	60.7%	62.5%	58.6%
Mongolia	Darkhan	2000	16.5%	23.8%	12.0%
Mongolia	Darkhan	2017	65.1%	67.1%	62.8%
Mongolia	Darkhan	2017	18.6%	29.8%	12.8%
Mongolia	Darvi	2000	6.7%	12.1%	3.0%
Mongolia	Darvi	2000	6.3%	10.3%	3.1%
Mongolia	Darvi	2017	9.7%	26.1%	3.0%
Mongolia	Darvi	2017	7.3%	13.1%	3.0%
Mongolia	Dashbalbar	2000	13.7%	19.5%	9.6%
Mongolia	Dashbalbar	2017	17.0%	26.4%	10.6%
Mongolia	Dashinchilen	2000	15.4%	29.5%	6.4%
Mongolia	Dashinchilen	2017	16.7%	38.1%	5.3%
Mongolia	Davst	2000	14.4%	22.8%	7.5%
Mongolia	Davst	2017	17.8%	28.4%	9.5%
Mongolia	Delger	2000	9.3%	16.0%	4.8%
Mongolia	Delger	2017	10.9%	18.3%	5.7%
Mongolia	Delgerekh	2000	9.8%	16.0%	5.3%
Mongolia	Delgerekh	2017	12.6%	22.2%	6.0%
Mongolia	Delgerkhaan	2000	6.6%	12.6%	2.6%

Country	Second Administrative Unit	Year	Mean	Upper	Lower
Mongolia	Delgerkhaan	2000	11.1%	26.7%	4.3%
Mongolia	Delgerkhaan	2017	7.9%	17.6%	2.5%
Mongolia	Delgerkhaan	2017	13.1%	36.1%	4.2%
Mongolia	Delgerkhangai	2000	9.5%	15.0%	5.8%
Mongolia	Delgerkhangai	2017	11.2%	19.7%	6.3%
Mongolia	Delgertsogt	2000	8.4%	16.0%	3.7%
Mongolia	Delgertsogt	2017	10.4%	22.0%	3.8%
Mongolia	Delüün	2000	4.1%	6.8%	2.2%
Mongolia	Delüün	2017	4.2%	6.9%	2.0%
Mongolia	Deren	2000	7.5%	13.5%	3.6%
Mongolia	Deren	2017	8.8%	18.0%	3.8%
Mongolia	Dörgön	2000	5.9%	10.7%	3.0%
Mongolia	Dörgön	2017	7.3%	15.2%	2.9%
Mongolia	Dörvöljin	2000	3.4%	6.0%	1.8%
Mongolia	Dörvöljin	2017	4.6%	12.3%	1.9%
Mongolia	Duut	2000	5.4%	13.0%	2.2%
Mongolia	Duut	2017	7.1%	19.6%	1.9%
Mongolia	Erdene	2000	12.6%	19.9%	8.0%
Mongolia	Erdene	2000	2.2%	3.6%	1.5%
Mongolia	Erdene	2000	11.2%	22.2%	5.9%
Mongolia	Erdene	2017	14.2%	22.0%	8.9%
Mongolia	Erdene	2017	13.7%	22.9%	7.3%
Mongolia	Erdene	2017	2.4%	3.8%	1.5%
Mongolia	Erdenebulgan	2000	13.9%	19.7%	8.9%
Mongolia	Erdenebulgan	2000	11.6%	15.6%	8.4%
Mongolia	Erdenebulgan	2017	11.2%	16.8%	7.4%
Mongolia	Erdenebulgan	2017	15.5%	24.2%	9.0%
Mongolia	Erdenebüren	2000	6.9%	12.3%	3.4%
Mongolia	Erdenebüren	2017	9.3%	17.8%	3.5%
Mongolia	Erdenedalai	2000	12.1%	16.8%	8.4%
Mongolia	Erdenedalai	2017	15.5%	21.8%	10.7%
Mongolia	Erdenekhairkhan	2000	4.6%	8.0%	2.2%
Mongolia	Erdenekhairkhan	2017	5.6%	9.6%	2.7%
Mongolia	Erdenemandal	2000	9.8%	16.4%	4.9%
Mongolia	Erdenemandal	2017	11.3%	21.4%	5.1%
Mongolia	Erdenesant	2000	15.4%	25.4%	9.1%
Mongolia	Erdenesant	2017	21.0%	38.7%	11.3%
Mongolia	Erdenetsagaan	2000	9.6%	13.7%	6.8%
Mongolia	Erdenetsagaan	2017	11.6%	17.4%	7.3%
Mongolia	Erdenetsogt	2000	7.3%	13.3%	3.7%
Mongolia	Erdenetsogt	2017	8.4%	15.2%	4.5%
Mongolia	Galshar	2000	7.6%	11.8%	4.3%
Mongolia	Galshar	2017	8.9%	13.9%	4.8%
Mongolia	Galt	2000	10.9%	22.7%	4.3%
Mongolia	Galt	2017	12.9%	29.4%	4.8%
Mongolia	Galuut	2000	6.7%	11.7%	3.5%
Mongolia	Galuut	2017	8.5%	15.1%	4.1%
Mongolia	Govi-Ugtaal	2000	7.0%	13.1%	3.4%
Mongolia	Govi-Ugtaal	2017	8.7%	16.7%	3.9%
Mongolia	Guchin-Us	2000	5.9%	11.7%	2.9%
Mongolia	Guchin-Us	2017	7.4%	17.0%	2.9%
Mongolia	Gurvan tes	2000	7.7%	11.3%	5.3%
Mongolia	Gurvan tes	2017	9.2%	15.7%	5.2%
Mongolia	Gurvanbulag	2000	14.3%	24.8%	7.2%
Mongolia	Gurvanbulag	2000	3.3%	6.5%	1.6%
Mongolia	Gurvanbulag	2017	14.6%	25.3%	7.4%
Mongolia	Gurvanbulag	2017	4.1%	10.7%	1.7%
Mongolia	Gurvansaikhan	2000	9.0%	14.2%	5.2%
Mongolia	Gurvansaikhan	2017	10.5%	16.4%	6.3%
Mongolia	Gurvanzagal	2000	13.0%	18.9%	7.4%
Mongolia	Gurvanzagal	2017	15.0%	22.1%	9.0%
Mongolia	Ider	2000	5.7%	10.0%	2.9%

Country	Second Administrative Unit	Year	Mean	Upper	Lower
Mongolia	Ider	2017	6.6%	12.1%	3.4%
Mongolia	Ikh-Tamir	2000	13.5%	19.4%	8.9%
Mongolia	Ikh-Tamir	2017	16.9%	24.8%	10.7%
Mongolia	Ikh-Uul	2000	8.4%	15.4%	3.6%
Mongolia	Ikh-Uul	2000	11.6%	27.6%	3.5%
Mongolia	Ikh-Uul	2017	7.2%	14.8%	2.8%
Mongolia	Ikh-Uul	2017	14.3%	39.8%	3.6%
Mongolia	Ikhkhet	2000	9.2%	13.9%	5.1%
Mongolia	Ikhkhet	2017	10.8%	18.1%	5.8%
Mongolia	Jargalan	2000	5.9%	11.1%	2.9%
Mongolia	Jargalan	2017	7.3%	14.7%	3.2%
Mongolia	Jargalant	2000	14.5%	16.4%	13.0%
Mongolia	Jargalant	2000	27.0%	46.3%	13.7%
Mongolia	Jargalant	2000	15.1%	27.6%	6.9%
Mongolia	Jargalant	2000	8.3%	15.5%	3.6%
Mongolia	Jargalant	2000	8.5%	21.7%	2.7%
Mongolia	Jargalant	2000	3.9%	6.6%	2.0%
Mongolia	Jargalant	2017	17.6%	31.3%	7.9%
Mongolia	Jargalant	2017	9.5%	19.6%	3.4%
Mongolia	Jargalant	2017	17.5%	19.5%	15.6%
Mongolia	Jargalant	2017	4.4%	9.0%	2.0%
Mongolia	Jargalant	2017	33.0%	51.1%	16.7%
Mongolia	Jargalant	2017	10.3%	25.3%	2.9%
Mongolia	Jargaltkhaan	2000	12.1%	24.0%	5.7%
Mongolia	Jargaltkhaan	2017	14.6%	35.4%	5.7%
Mongolia	Javkhlant	2000	20.3%	34.8%	10.4%
Mongolia	Javkhlant	2017	21.1%	39.2%	9.8%
Mongolia	Jinst	2000	6.8%	11.7%	3.5%
Mongolia	Jinst	2017	8.1%	16.3%	3.2%
Mongolia	Khairkhan	2000	10.6%	21.4%	4.6%
Mongolia	Khairkhan	2017	12.5%	28.9%	5.0%
Mongolia	Khairkhandulaan	2000	10.6%	17.9%	5.3%
Mongolia	Khairkhandulaan	2017	13.9%	28.8%	5.9%
Mongolia	Khaliun	2000	7.1%	11.4%	4.2%
Mongolia	Khaliun	2017	8.7%	14.5%	4.8%
Mongolia	Khalkhgol	2000	17.0%	38.8%	8.1%
Mongolia	Khalkhgol	2017	20.3%	46.6%	8.2%
Mongolia	Khalzan	2000	8.0%	13.8%	4.2%
Mongolia	Khalzan	2017	9.2%	18.2%	4.9%
Mongolia	Khan khongor	2000	20.1%	26.0%	15.1%
Mongolia	Khan khongor	2017	24.4%	32.5%	18.0%
Mongolia	Khanbogd	2000	11.6%	16.4%	8.1%
Mongolia	Khanbogd	2017	14.5%	23.9%	8.5%
Mongolia	Khangai	2000	5.4%	12.0%	2.1%
Mongolia	Khangai	2017	6.6%	14.2%	2.4%
Mongolia	Khangal	2000	10.9%	20.3%	4.3%
Mongolia	Khangal	2017	14.3%	26.4%	6.1%
Mongolia	Khankh	2000	14.7%	25.0%	7.2%
Mongolia	Khankh	2017	18.3%	36.3%	7.5%
Mongolia	Kharkhorin	2000	16.3%	23.4%	10.6%
Mongolia	Kharkhorin	2017	20.6%	32.5%	11.8%
Mongolia	Khashaat	2000	11.9%	20.8%	6.1%
Mongolia	Khashaat	2017	12.7%	22.1%	6.4%
Mongolia	Khatanbulag	2000	6.5%	8.7%	4.5%
Mongolia	Khatanbulag	2017	7.8%	11.4%	5.1%
Mongolia	Khatgal	2000	13.9%	31.6%	3.5%
Mongolia	Khatgal	2017	17.2%	40.0%	4.1%
Mongolia	Kherlen	2000	54.9%	58.0%	51.9%
Mongolia	Kherlen	2000	19.5%	24.9%	14.8%
Mongolia	Kherlen	2017	23.7%	31.1%	17.3%
Mongolia	Kherlen	2017	59.8%	64.4%	55.5%
Mongolia	Khishig-Öndör	2000	15.2%	28.3%	7.0%

Country	Second Administrative Unit	Year	Mean	Upper	Lower
Mongolia	Khishig-Öndör	2017	17.2%	34.7%	6.4%
Mongolia	Khökh morit	2000	4.2%	7.9%	2.0%
Mongolia	Khökh morit	2017	5.8%	12.1%	2.6%
Mongolia	Khölönbuir	2000	9.6%	15.3%	5.2%
Mongolia	Khölönbuir	2017	9.6%	16.6%	5.0%
Mongolia	Khongor	2000	31.5%	38.0%	27.0%
Mongolia	Khongor	2017	40.4%	46.6%	34.9%
Mongolia	Khotont	2000	12.4%	21.1%	6.3%
Mongolia	Khotont	2017	14.5%	28.4%	6.2%
Mongolia	Khovd	2000	62.8%	70.4%	55.1%
Mongolia	Khovd	2000	6.4%	13.6%	2.7%
Mongolia	Khovd	2017	8.1%	16.4%	3.4%
Mongolia	Khovd	2017	48.0%	55.4%	40.9%
Mongolia	Khövsgöl	2000	8.1%	13.3%	4.7%
Mongolia	Khövsgöl	2017	9.5%	18.0%	5.2%
Mongolia	Khüder	2000	14.8%	25.9%	7.6%
Mongolia	Khüder	2017	18.3%	32.2%	9.3%
Mongolia	Khujirt	2000	16.0%	40.2%	4.1%
Mongolia	Khujirt	2017	16.1%	34.4%	5.5%
Mongolia	Khuld	2000	9.7%	14.8%	5.7%
Mongolia	Khuld	2017	11.4%	19.0%	6.6%
Mongolia	Khüreemaral	2000	5.5%	10.2%	2.9%
Mongolia	Khüreemaral	2017	6.2%	14.2%	2.6%
Mongolia	Khürmen	2000	7.1%	10.5%	4.3%
Mongolia	Khürmen	2017	8.7%	13.8%	5.0%
Mongolia	Khushaat	2000	17.4%	29.1%	8.1%
Mongolia	Khushaat	2017	18.0%	32.3%	8.4%
Mongolia	Khutag-Öndör	2000	17.1%	27.9%	9.3%
Mongolia	Khutag-Öndör	2017	19.7%	35.5%	10.1%
Mongolia	Khyargas	2000	7.4%	13.3%	3.7%
Mongolia	Khyargas	2017	8.8%	15.7%	4.3%
Mongolia	Lün	2000	21.1%	46.7%	6.5%
Mongolia	Lün	2017	24.3%	55.8%	6.4%
Mongolia	Luus	2000	9.8%	16.7%	4.5%
Mongolia	Luus	2017	11.3%	21.7%	4.8%
Mongolia	Malchin	2000	7.0%	12.2%	3.6%
Mongolia	Malchin	2017	8.1%	14.5%	4.1%
Mongolia	Mandakh	2000	5.1%	8.2%	2.9%
Mongolia	Mandakh	2017	6.8%	13.2%	3.3%
Mongolia	Mandal	2000	15.5%	23.9%	9.1%
Mongolia	Mandal	2017	18.9%	27.8%	11.3%
Mongolia	Mandal-Ovoo	2000	9.0%	16.6%	5.0%
Mongolia	Mandal-Ovoo	2017	10.9%	29.1%	4.2%
Mongolia	Mankhan	2000	8.8%	15.5%	4.3%
Mongolia	Mankhan	2017	10.6%	18.7%	5.5%
Mongolia	Manlai	2000	6.9%	10.2%	4.4%
Mongolia	Manlai	2017	6.9%	13.2%	3.8%
Mongolia	Matad	2000	7.5%	10.1%	5.3%
Mongolia	Matad	2017	9.0%	15.6%	4.9%
Mongolia	Mogod	2000	15.6%	33.7%	6.3%
Mongolia	Mogod	2017	18.9%	51.9%	4.8%
Mongolia	Möngönmorit	2000	11.4%	17.5%	7.2%
Mongolia	Möngönmorit	2017	15.0%	25.4%	8.2%
Mongolia	Mönkhkhairkhan	2000	4.8%	11.0%	1.8%
Mongolia	Mönkhkhairkhan	2017	7.4%	25.1%	1.5%
Mongolia	Mörön	2000	14.3%	21.9%	8.2%
Mongolia	Mörön	2000	26.9%	33.3%	21.1%
Mongolia	Mörön	2017	29.6%	40.7%	22.1%
Mongolia	Mörön	2017	18.2%	29.1%	9.6%

Country	Second Administrative Unit	Year	Mean	Upper	Lower
Mongolia	Möst	2000	6.3%	15.3%	2.7%
Mongolia	Möst	2017	7.9%	19.4%	3.1%
Mongolia	Myangad	2000	7.4%	14.4%	3.3%
Mongolia	Myangad	2017	9.9%	19.5%	4.3%
Mongolia	Naran	2000	8.2%	13.3%	4.1%
Mongolia	Naran	2017	9.6%	16.3%	4.5%
Mongolia	Naranbulag	2000	6.5%	11.9%	3.1%
Mongolia	Naranbulag	2017	7.9%	17.9%	3.0%
Mongolia	Nariinteel	2000	10.0%	18.5%	4.0%
Mongolia	Nariinteel	2017	11.6%	24.2%	4.1%
Mongolia	Nogoonnuur	2000	6.9%	13.9%	2.9%
Mongolia	Nogoonnuur	2017	7.6%	14.7%	3.1%
Mongolia	Nomgon	2000	6.8%	9.8%	4.9%
Mongolia	Nomgon	2017	7.8%	11.4%	5.2%
Mongolia	Nömrög	2000	9.9%	19.2%	4.6%
Mongolia	Nömrög	2017	12.8%	29.0%	5.3%
Mongolia	Norovlin	2000	13.2%	26.9%	6.6%
Mongolia	Norovlin	2017	15.3%	36.5%	6.2%
Mongolia	Noyon	2000	8.4%	12.1%	5.1%
Mongolia	Noyon	2017	9.9%	15.5%	5.2%
Mongolia	Ögii nuur	2000	10.5%	23.1%	4.2%
Mongolia	Ögii nuur	2017	12.4%	33.6%	3.8%
Mongolia	Ölgii	2000	5.7%	12.9%	1.9%
Mongolia	Ölgii	2017	6.9%	16.2%	2.1%
Mongolia	Ölgii (city)	2000	4.4%	5.5%	3.5%
Mongolia	Ölgii (city)	2017	5.7%	7.0%	4.5%
Mongolia	Ölziit	2000	12.1%	22.0%	5.4%
Mongolia	Ölziit	2000	18.9%	26.8%	13.5%
Mongolia	Ölziit	2000	10.7%	20.0%	4.5%
Mongolia	Ölziit	2000	8.9%	12.4%	6.2%
Mongolia	Ölziit	2017	11.0%	19.1%	4.5%
Mongolia	Ölziit	2017	21.4%	30.4%	15.5%
Mongolia	Ölziit	2017	10.7%	15.6%	6.9%
Mongolia	Ölziit	2017	14.2%	25.9%	5.9%
Mongolia	Ömnödelger	2000	13.5%	19.0%	9.1%
Mongolia	Ömnödelger	2017	14.5%	24.6%	8.3%
Mongolia	Ömnögovi	2000	5.3%	9.6%	2.4%
Mongolia	Ömnögovi	2017	6.6%	14.6%	2.3%
Mongolia	Öndör-Ulaan	2000	8.6%	14.2%	4.2%
Mongolia	Öndör-Ulaan	2017	10.6%	18.4%	5.1%
Mongolia	Öndörkhangai	2000	8.6%	18.6%	4.0%
Mongolia	Öndörkhangai	2017	10.4%	22.3%	4.1%
Mongolia	Öndörshil	2000	8.6%	13.9%	4.5%
Mongolia	Öndörshil	2017	10.1%	19.8%	4.5%
Mongolia	Öndörshireet	2000	11.8%	21.1%	5.8%
Mongolia	Öndörshireet	2017	13.5%	27.0%	6.1%
Mongolia	Ongon	2000	8.0%	12.4%	5.1%
Mongolia	Ongon	2017	9.6%	16.5%	5.7%
Mongolia	Örgön	2000	41.1%	49.2%	32.4%
Mongolia	Örgön	2017	39.8%	46.5%	31.5%
Mongolia	Orkhon	2000	15.1%	20.1%	11.3%
Mongolia	Orkhon	2000	14.8%	33.1%	5.4%
Mongolia	Orkhon	2000	19.0%	35.1%	6.8%
Mongolia	Orkhon	2017	15.1%	28.5%	6.2%
Mongolia	Orkhon	2017	19.4%	25.0%	15.1%
Mongolia	Orkhon	2017	21.9%	41.8%	7.8%
Mongolia	Orkhontuul	2000	18.8%	34.3%	9.8%
Mongolia	Orkhontuul	2017	22.3%	37.2%	11.9%
Mongolia	Otgon	2000	4.2%	7.8%	2.2%
Mongolia	Otgon	2017	5.1%	12.1%	1.8%
Mongolia	Rashaant	2000	6.6%	18.8%	1.7%
Mongolia	Rashaant	2000	12.1%	20.6%	5.9%

Country	Second Administrative Unit	Year	Mean	Upper	Lower
Mongolia	Rashaant	2017	13.1%	23.6%	6.3%
Mongolia	Rashaant	2017	7.9%	22.4%	1.7%
Mongolia	Renchinlkhümbe	2000	11.1%	17.8%	6.2%
Mongolia	Renchinlkhümbe	2017	14.1%	24.4%	7.3%
Mongolia	Sagil	2000	8.5%	13.0%	4.8%
Mongolia	Sagil	2017	11.0%	21.2%	5.6%
Mongolia	Sagsai	2000	5.7%	11.2%	2.8%
Mongolia	Sagsai	2017	7.1%	14.7%	3.4%
Mongolia	Saikhan	2000	18.0%	32.8%	7.8%
Mongolia	Saikhan	2017	19.9%	37.3%	9.3%
Mongolia	Saikhan-Ovoo	2000	8.8%	15.2%	4.6%
Mongolia	Saikhan-Ovoo	2017	10.5%	18.4%	4.9%
Mongolia	Saikhandulaan	2000	8.8%	12.5%	5.9%
Mongolia	Saikhandulaan	2017	10.1%	14.4%	7.0%
Mongolia	Sainshand	2000	34.0%	43.4%	26.2%
Mongolia	Sainshand	2017	31.3%	40.1%	23.9%
Mongolia	Saintsagaan	2000	22.6%	30.7%	16.5%
Mongolia	Saintsagaan	2017	26.0%	37.5%	17.9%
Mongolia	Sant	2000	22.7%	40.8%	8.7%
Mongolia	Sant	2000	6.9%	12.9%	3.4%
Mongolia	Sant	2017	29.1%	57.4%	8.5%
Mongolia	Sant	2017	8.0%	15.6%	3.9%
Mongolia	Santmargats	2000	6.6%	14.3%	2.7%
Mongolia	Santmargats	2017	8.0%	18.9%	2.6%
Mongolia	Saykhan	2000	14.8%	19.5%	10.6%
Mongolia	Saykhan	2017	16.9%	21.9%	12.5%
Mongolia	Selenge	2000	33.6%	42.9%	26.1%
Mongolia	Selenge	2017	36.7%	47.7%	28.0%
Mongolia	Sergelen	2000	28.8%	46.1%	16.8%
Mongolia	Sergelen	2000	12.9%	19.2%	7.4%
Mongolia	Sergelen	2017	35.4%	53.1%	23.2%
Mongolia	Sergelen	2017	15.4%	25.5%	8.9%
Mongolia	Sevrei	2000	7.2%	11.0%	4.6%
Mongolia	Sevrei	2017	8.6%	13.1%	5.4%
Mongolia	Shaamar	2000	14.2%	24.0%	5.9%
Mongolia	Shaamar	2017	17.2%	28.8%	8.4%
Mongolia	Sharga	2000	8.6%	16.8%	3.8%
Mongolia	Sharga	2017	11.5%	24.2%	4.2%
Mongolia	Sharyngol	2000	14.0%	24.3%	7.6%
Mongolia	Sharyngol	2017	17.3%	30.0%	8.8%
Mongolia	Shilüüstei	2000	6.1%	13.1%	2.4%
Mongolia	Shilüüstei	2017	7.3%	14.8%	2.7%
Mongolia	Shine-Ider	2000	10.4%	29.8%	2.8%
Mongolia	Shine-Ider	2017	12.8%	35.7%	2.8%
Mongolia	Shinejinst	2000	4.0%	6.2%	2.4%
Mongolia	Shinejinst	2017	4.9%	7.8%	2.9%
Mongolia	Shiveegovi	2000	16.9%	31.6%	9.1%
Mongolia	Shiveegovi	2017	19.6%	41.4%	9.5%
Mongolia	Songino	2000	9.0%	16.0%	4.1%
Mongolia	Songino	2017	10.0%	19.9%	3.9%
Mongolia	Sükhbaatar	2000	25.4%	29.7%	21.7%
Mongolia	Sükhbaatar	2017	25.2%	30.9%	21.1%
Mongolia	Sümber	2000	16.2%	34.0%	5.0%
Mongolia	Sümber	2000	18.3%	23.3%	13.9%
Mongolia	Sümber	2017	18.2%	36.9%	6.1%
Mongolia	Sümber	2017	21.6%	29.1%	16.1%
Mongolia	Taishir	2000	8.1%	14.5%	3.9%
Mongolia	Taishir	2017	9.5%	17.2%	4.9%
Mongolia	Taragt	2000	11.5%	18.6%	6.0%
Mongolia	Taragt	2017	12.4%	20.0%	6.1%
Mongolia	Tarialan	2000	6.7%	11.9%	3.6%
Mongolia	Tarialan	2000	9.6%	17.9%	3.6%

Country	Second Administrative Unit	Year	Mean	Upper	Lower
Mongolia	Tarialan	2017	12.5%	24.2%	4.9%
Mongolia	Tarialan	2017	10.5%	17.2%	5.9%
Mongolia	Tariat	2000	6.4%	13.0%	2.8%
Mongolia	Tariat	2017	7.6%	14.3%	2.9%
Mongolia	Telmen	2000	8.9%	15.6%	4.4%
Mongolia	Telmen	2017	11.6%	23.6%	4.4%
Mongolia	Tes	2000	12.8%	27.4%	4.2%
Mongolia	Tes	2000	11.0%	17.3%	6.1%
Mongolia	Tes	2017	12.9%	20.7%	7.5%
Mongolia	Tes	2017	14.1%	43.2%	3.0%
Mongolia	Teshig	2000	18.7%	24.8%	12.4%
Mongolia	Teshig	2017	22.1%	31.6%	14.2%
Mongolia	Tögrög	2000	7.5%	15.5%	3.2%
Mongolia	Tögrög	2000	7.4%	14.5%	3.5%
Mongolia	Tögrög	2017	9.3%	18.8%	4.0%
Mongolia	Tögrög	2017	9.4%	20.7%	3.4%
Mongolia	Tolbo	2000	3.9%	8.1%	1.4%
Mongolia	Tolbo	2017	4.6%	9.8%	1.5%
Mongolia	Tömörbulag	2000	8.3%	16.1%	3.9%
Mongolia	Tömörbulag	2017	8.8%	15.5%	4.3%
Mongolia	Tonkhil	2000	5.4%	8.3%	3.4%
Mongolia	Tonkhil	2017	6.5%	12.1%	3.6%
Mongolia	Tosontsengel	2000	13.0%	22.0%	5.1%
Mongolia	Tosontsengel	2000	6.5%	17.3%	2.6%
Mongolia	Tosontsengel	2017	7.5%	19.9%	3.1%
Mongolia	Tosontsengel	2017	16.7%	33.9%	4.7%
Mongolia	Tsagaan-Ovoo	2000	11.3%	16.0%	7.4%
Mongolia	Tsagaan-Ovoo	2017	12.0%	17.2%	7.9%
Mongolia	Tsagaan-Uul	2000	11.6%	17.9%	6.4%
Mongolia	Tsagaan-Uul	2017	14.5%	24.7%	8.3%
Mongolia	Tsagaan-Üür	2000	13.1%	19.4%	8.7%
Mongolia	Tsagaan-Üür	2017	16.6%	31.5%	8.4%
Mongolia	Tsagaanchuluut	2000	6.8%	14.4%	2.6%
Mongolia	Tsagaanchuluut	2017	8.4%	18.5%	3.2%
Mongolia	Tsagaandelger	2000	7.7%	13.2%	3.9%
Mongolia	Tsagaandelger	2017	9.6%	16.9%	4.7%
Mongolia	Tsagaanhairhan	2000	8.1%	15.1%	3.8%
Mongolia	Tsagaanhairhan	2017	10.0%	19.7%	4.2%
Mongolia	Tsagaankhairkhan	2000	7.4%	19.9%	1.9%
Mongolia	Tsagaankhairkhan	2017	8.8%	25.4%	2.0%
Mongolia	Tsagaannuur	2000	18.9%	48.3%	5.9%
Mongolia	Tsagaannuur	2000	5.7%	16.6%	1.5%
Mongolia	Tsagaannuur	2000	5.5%	8.7%	3.2%
Mongolia	Tsagaannuur	2017	6.5%	10.4%	3.9%
Mongolia	Tsagaannuur	2017	6.9%	20.7%	1.4%
Mongolia	Tsagaannuur	2017	21.4%	48.6%	7.9%
Mongolia	Tsakhir	2000	4.2%	7.7%	2.0%
Mongolia	Tsakhir	2017	4.8%	8.5%	2.3%
Mongolia	Tseel	2000	13.2%	25.3%	5.7%
Mongolia	Tseel	2000	4.5%	7.7%	2.5%
Mongolia	Tseel	2017	5.5%	10.2%	2.8%
Mongolia	Tseel	2017	15.6%	31.9%	5.5%
Mongolia	Tsengel	2000	6.1%	13.4%	2.3%
Mongolia	Tsengel	2017	7.2%	15.0%	3.0%
Mongolia	Tsenkher	2000	11.4%	21.4%	4.4%
Mongolia	Tsenkher	2017	15.2%	29.8%	5.6%
Mongolia	Tsenkhermandal	2000	15.6%	27.7%	8.8%
Mongolia	Tsenkhermandal	2017	14.9%	29.5%	7.3%
Mongolia	Tsetseg	2000	5.9%	11.1%	2.7%
Mongolia	Tsetseg	2017	8.6%	22.9%	2.3%

Piped Water Proportion by Second Administrative Unit *(continued)*

Country	Second Administrative Unit	Year	Mean	Upper	Lower
Mongolia	Tsetsen-Uul	2000	6.8%	13.5%	2.7%
Mongolia	Tsetsen-Uul	2017	7.5%	14.5%	3.0%
Mongolia	Tsetserleg	2000	8.7%	14.5%	4.9%
Mongolia	Tsetserleg	2000	12.4%	21.2%	6.2%
Mongolia	Tsetserleg	2017	9.8%	16.8%	5.3%
Mongolia	Tsetserleg	2017	11.1%	17.9%	6.1%
Mongolia	Tsogt	2000	3.5%	5.9%	2.1%
Mongolia	Tsogt	2017	5.0%	10.2%	2.4%
Mongolia	Tsogt-Ovoo	2000	8.7%	13.1%	5.0%
Mongolia	Tsogt-Ovoo	2017	11.7%	21.8%	5.1%
Mongolia	Tsogttsetsii	2000	8.8%	12.7%	5.5%
Mongolia	Tsogttsetsii	2017	10.8%	18.2%	6.1%
Mongolia	Tüdevtei	2000	13.6%	27.6%	6.0%
Mongolia	Tüdevtei	2017	16.4%	29.6%	6.9%
Mongolia	Tümentsogt	2000	20.2%	31.0%	12.3%
Mongolia	Tümentsogt	2017	25.9%	40.5%	14.2%
Mongolia	Tünel	2000	22.9%	32.3%	15.7%
Mongolia	Tünel	2017	22.8%	33.6%	14.7%
Mongolia	Türgen	2000	5.8%	11.8%	2.3%
Mongolia	Türgen	2017	6.0%	12.9%	2.5%
Mongolia	Tüshig	2000	20.1%	58.5%	6.1%
Mongolia	Tüshig	2017	22.6%	59.9%	7.1%
Mongolia	Tüvshinshiree	2000	8.5%	14.2%	4.6%
Mongolia	Tüvshinshiree	2017	9.8%	17.3%	4.7%
Mongolia	Tüvshrüülekh	2000	10.2%	20.3%	3.9%
Mongolia	Tüvshrüülekh	2017	10.6%	22.4%	3.9%
Mongolia	Ugtaal	2000	13.6%	25.7%	5.6%
Mongolia	Ugtaal	2017	15.0%	28.5%	6.4%
Mongolia	Ulaan-Uul	2000	7.7%	13.2%	4.1%
Mongolia	Ulaan-Uul	2017	9.3%	17.0%	4.7%
Mongolia	Ulaanbadrakh	2000	11.4%	27.0%	5.0%
Mongolia	Ulaanbadrakh	2017	13.5%	29.5%	5.9%
Mongolia	Ulaangom	2000	10.3%	14.3%	7.7%
Mongolia	Ulaangom	2017	12.0%	20.1%	7.7%
Mongolia	Ulaankhus	2000	6.8%	14.5%	3.0%
Mongolia	Ulaankhus	2017	8.2%	15.7%	3.6%
Mongolia	Ulan Bator	2000	43.7%	45.8%	41.9%
Mongolia	Ulan Bator	2017	46.9%	49.3%	44.4%
Mongolia	Urgamal	2000	3.7%	7.6%	1.5%
Mongolia	Urgamal	2017	4.5%	9.7%	1.5%
Mongolia	Uulbayan	2000	7.9%	12.0%	4.9%
Mongolia	Uulbayan	2017	8.7%	12.4%	5.6%
Mongolia	Uyanga	2000	7.6%	14.7%	3.4%
Mongolia	Uyanga	2017	8.1%	15.0%	3.7%
Mongolia	Üyench	2000	7.6%	11.2%	4.7%
Mongolia	Üyench	2017	10.1%	15.7%	5.3%
Mongolia	Yaruu	2000	6.5%	11.4%	3.2%
Mongolia	Yaruu	2017	8.3%	18.9%	3.2%
Mongolia	Yeröö	2000	14.1%	21.1%	8.0%
Mongolia	Yeröö	2017	19.7%	33.0%	9.5%
Mongolia	Yesönbulag	2000	22.7%	26.9%	19.3%
Mongolia	Yesönbulag	2017	25.1%	29.5%	21.0%
Mongolia	Yesönzüil	2000	10.0%	18.3%	4.3%
Mongolia	Yesönzüil	2017	10.6%	21.5%	4.2%
Mongolia	Zaamar	2000	16.4%	28.5%	7.4%
Mongolia	Zaamar	2017	17.0%	30.8%	7.8%
Mongolia	Zag	2000	4.1%	8.1%	1.7%
Mongolia	Zag	2017	5.3%	13.3%	1.6%
Mongolia	Zamyn-Üüd	2000	15.7%	25.8%	8.2%
Mongolia	Zamyn-Üüd	2017	20.0%	34.0%	9.9%
Mongolia	Zavkhan	2000	3.6%	5.9%	2.0%
Mongolia	Zavkhan	2017	4.3%	7.3%	2.2%

Country	Second Administrative Unit	Year	Mean	Upper	Lower
Mongolia	Zavkhanmandal	2000	3.7%	7.9%	1.4%
Mongolia	Zavkhanmandal	2017	4.4%	10.8%	1.2%
Mongolia	Zereg	2000	7.4%	18.0%	2.0%
Mongolia	Zereg	2017	9.0%	21.0%	2.5%
Mongolia	Züünbayan-Ulaan	2000	10.9%	18.9%	5.4%
Mongolia	Züünbayan-Ulaan	2017	12.3%	23.0%	5.9%
Mongolia	Züünbüren	2000	23.1%	41.0%	11.5%
Mongolia	Züünbüren	2017	24.8%	39.7%	13.9%
Mongolia	Züüngovi	2000	10.3%	17.2%	5.2%
Mongolia	Züüngovi	2017	10.8%	22.4%	4.9%
Mongolia	Züünkhangai	2000	9.8%	18.6%	4.7%
Mongolia	Züünkhangai	2017	9.9%	26.0%	3.7%
Tajikistan	Asht	2000	58.6%	69.9%	41.0%
Tajikistan	Asht	2017	71.7%	83.0%	57.1%
Tajikistan	Ayni	2000	40.7%	60.9%	20.7%
Tajikistan	Ayni	2017	56.9%	73.5%	38.5%
Tajikistan	Baljuvon	2000	37.9%	82.0%	5.1%
Tajikistan	Baljuvon	2017	50.1%	89.6%	11.0%
Tajikistan	Bokhtar	2000	35.5%	39.5%	31.4%
Tajikistan	Bokhtar	2017	75.1%	77.5%	71.6%
Tajikistan	Danghara	2000	60.6%	78.7%	36.6%
Tajikistan	Danghara	2017	72.6%	87.7%	51.0%
Tajikistan	Darvoz	2000	50.6%	67.7%	30.6%
Tajikistan	Darvoz	2017	64.1%	79.9%	44.4%
Tajikistan	Farkhor	2000	35.7%	44.1%	27.2%
Tajikistan	Farkhor	2017	64.7%	73.2%	53.9%
Tajikistan	Fayzobod	2000	37.8%	47.8%	29.5%
Tajikistan	Fayzobod	2017	61.7%	72.2%	51.6%
Tajikistan	Ghafurov	2000	60.5%	62.8%	57.2%
Tajikistan	Ghafurov	2017	80.7%	82.5%	77.8%
Tajikistan	Ghonchi	2000	54.7%	71.2%	40.7%
Tajikistan	Ghonchi	2017	75.7%	89.4%	61.5%
Tajikistan	Hissor	2000	25.6%	27.6%	24.0%
Tajikistan	Hissor	2017	62.1%	64.3%	60.0%
Tajikistan	Isfara	2000	27.4%	34.3%	23.3%
Tajikistan	Isfara	2017	52.8%	61.7%	46.0%
Tajikistan	Ishkoshim	2000	31.8%	47.7%	16.1%
Tajikistan	Ishkoshim	2017	46.6%	65.8%	28.5%
Tajikistan	Istaravshan	2000	33.3%	43.2%	24.4%
Tajikistan	Istaravshan	2017	60.9%	71.5%	46.4%
Tajikistan	Jabor Rasulov	2000	49.0%	55.1%	42.0%
Tajikistan	Jabor Rasulov	2017	78.8%	83.3%	71.9%
Tajikistan	Jilikul	2000	17.9%	34.2%	10.9%
Tajikistan	Jilikul	2017	42.9%	61.2%	33.5%
Tajikistan	Jirgatol	2000	42.6%	59.0%	27.2%
Tajikistan	Jirgatol	2017	60.9%	76.3%	41.8%
Tajikistan	Jomi	2000	20.3%	23.9%	17.3%
Tajikistan	Jomi	2017	47.0%	51.3%	41.4%
Tajikistan	Khovaling	2000	54.6%	80.5%	24.4%
Tajikistan	Khovaling	2017	70.2%	89.2%	44.1%
Tajikistan	Khuroson	2000	22.2%	33.8%	11.8%
Tajikistan	Khuroson	2017	36.4%	48.8%	21.1%
Tajikistan	Kolkhozobod	2000	30.3%	44.8%	15.9%
Tajikistan	Kolkhozobod	2017	60.6%	71.1%	44.9%
Tajikistan	Konibodom	2000	28.2%	32.0%	25.3%
Tajikistan	Konibodom	2017	45.8%	53.3%	40.9%
Tajikistan	Kuhistoni Mastchoh	2000	49.8%	77.4%	22.3%
Tajikistan	Kuhistoni Mastchoh	2017	64.1%	89.2%	34.3%

Country	Second Administrative Unit	Year	Mean	Upper	Lower
Tajikistan	Kulob	2000	75.6%	77.7%	72.9%
Tajikistan	Kulob	2017	92.2%	93.0%	91.3%
Tajikistan	Matchin	2000	68.0%	84.2%	49.0%
Tajikistan	Matchin	2017	83.3%	94.8%	65.9%
Tajikistan	Moskva	2000	41.0%	50.5%	34.8%
Tajikistan	Moskva	2017	68.4%	78.9%	60.5%
Tajikistan	Muminobod	2000	61.9%	69.3%	49.4%
Tajikistan	Muminobod	2017	74.9%	79.8%	65.8%
Tajikistan	Murghob	2000	33.8%	46.3%	22.5%
Tajikistan	Murghob	2017	45.1%	58.1%	32.5%
Tajikistan	Norak	2000	59.6%	70.9%	45.6%
Tajikistan	Norak	2017	67.5%	74.5%	56.9%
Tajikistan	Nosir Khusrav	2000	35.8%	74.8%	3.0%
Tajikistan	Nosir Khusrav	2017	51.6%	82.2%	12.3%
Tajikistan	Nurobod	2000	37.4%	45.5%	24.7%
Tajikistan	Nurobod	2017	54.6%	65.5%	43.0%
Tajikistan	Pandjakent	2000	66.0%	74.2%	56.9%
Tajikistan	Pandjakent	2017	77.1%	83.0%	69.9%
Tajikistan	Panj	2000	53.5%	63.8%	44.4%
Tajikistan	Panj	2017	71.8%	80.9%	62.4%
Tajikistan	Qabodiyon	2000	40.7%	52.7%	29.9%
Tajikistan	Qabodiyon	2017	59.4%	71.0%	46.1%
Tajikistan	Qumsangir	2000	40.8%	53.4%	28.3%
Tajikistan	Qumsangir	2017	69.3%	80.5%	52.8%
Tajikistan	Rasht	2000	53.6%	68.0%	42.3%
Tajikistan	Rasht	2017	64.1%	79.5%	51.7%
Tajikistan	Roghun	2000	54.8%	77.4%	36.0%
Tajikistan	Roghun	2017	65.5%	81.6%	51.6%
Tajikistan	Roshtqala	2000	38.1%	47.8%	28.8%
Tajikistan	Roshtqala	2017	57.1%	66.6%	47.3%
Tajikistan	Rudaki	2000	82.6%	83.3%	81.9%
Tajikistan	Rudaki	2000	36.4%	38.2%	34.7%
Tajikistan	Rudaki	2017	96.6%	96.7%	96.4%
Tajikistan	Rudaki	2017	68.6%	71.0%	66.0%
Tajikistan	Rushon	2000	42.7%	57.6%	30.4%
Tajikistan	Rushon	2017	63.7%	76.1%	50.1%
Tajikistan	Sarband	2000	40.2%	58.6%	16.6%
Tajikistan	Sarband	2017	76.4%	90.2%	49.1%
Tajikistan	Shahrinav	2000	37.6%	40.8%	33.6%
Tajikistan	Shahrinav	2017	56.1%	59.6%	50.4%
Tajikistan	Shahriston	2000	58.1%	80.7%	27.3%
Tajikistan	Shahriston	2017	72.5%	89.1%	48.1%
Tajikistan	Shahrituz	2000	25.7%	35.1%	16.9%
Tajikistan	Shahrituz	2017	50.9%	60.8%	39.1%
Tajikistan	Shughnon	2000	62.2%	66.4%	58.5%
Tajikistan	Shughnon	2017	84.9%	87.7%	81.9%
Tajikistan	Shurobod	2000	47.4%	64.9%	28.1%
Tajikistan	Shurobod	2017	65.3%	77.5%	50.0%
Tajikistan	Sovet	2000	60.5%	69.1%	42.8%
Tajikistan	Sovet	2017	65.0%	72.6%	53.0%
Tajikistan	Spitamen	2000	30.7%	44.0%	17.2%
Tajikistan	Spitamen	2017	64.9%	76.8%	45.4%
Tajikistan	Tavildara	2000	38.7%	59.6%	18.2%
Tajikistan	Tavildara	2017	48.7%	72.3%	25.7%
Tajikistan	Tojikobod	2000	45.5%	72.0%	18.7%
Tajikistan	Tojikobod	2017	68.7%	89.8%	35.4%
Tajikistan	Tursunzoda	2000	57.6%	61.3%	52.6%
Tajikistan	Tursunzoda	2017	79.1%	81.6%	73.6%
Tajikistan	Vahdat	2000	34.2%	42.6%	25.4%
Tajikistan	Vahdat	2017	69.2%	76.9%	59.2%
Tajikistan	Vakhsh	2000	26.7%	34.2%	19.3%
Tajikistan	Vakhsh	2017	62.3%	69.4%	51.8%

Country	Second Administrative Unit	Year	Mean	Upper	Lower
Tajikistan	Vanj	2000	34.9%	51.3%	22.2%
Tajikistan	Vanj	2017	61.5%	75.6%	47.6%
Tajikistan	Varzob	2000	49.2%	55.4%	43.4%
Tajikistan	Varzob	2017	77.3%	83.5%	71.7%
Tajikistan	Vose	2000	66.7%	72.8%	60.4%
Tajikistan	Vose	2017	88.5%	92.6%	83.3%
Tajikistan	Yovon	2000	55.9%	60.1%	51.8%
Tajikistan	Yovon	2017	73.0%	76.3%	68.6%
Tajikistan	Zafarobod	2000	37.4%	62.5%	19.0%
Tajikistan	Zafarobod	2017	64.4%	85.3%	43.4%
Turkmenistan	Ahal	2000	56.2%	60.0%	52.6%
Turkmenistan	Ahal	2017	65.2%	68.8%	61.4%
Turkmenistan	Aşgabat	2000	73.1%	76.7%	68.8%
Turkmenistan	Aşgabat	2017	79.8%	83.1%	75.8%
Turkmenistan	Balkan	2000	53.1%	57.4%	48.5%
Turkmenistan	Balkan	2017	61.6%	65.5%	57.6%
Turkmenistan	Chardzhou	2000	52.6%	57.6%	47.2%
Turkmenistan	Chardzhou	2017	60.7%	64.9%	56.0%
Turkmenistan	Mary	2000	39.3%	44.3%	34.6%
Turkmenistan	Mary	2017	48.3%	53.3%	43.7%
Turkmenistan	Tashauz	2000	31.5%	37.9%	25.9%
Turkmenistan	Tashauz	2017	39.8%	46.1%	33.4%
Uzbekistan	Amudaryo	2000	69.0%	78.0%	59.5%
Uzbekistan	Amudaryo	2017	72.8%	79.8%	65.3%
Uzbekistan	Andijon	2000	69.4%	79.6%	58.5%
Uzbekistan	Andijon	2017	73.2%	82.9%	62.6%
Uzbekistan	Angor	2000	75.8%	85.2%	64.9%
Uzbekistan	Angor	2017	79.1%	87.6%	69.1%
Uzbekistan	Aral Sea	2000	70.3%	74.1%	66.6%
Uzbekistan	Aral Sea	2017	74.2%	77.5%	70.6%
Uzbekistan	Arnasoy	2000	68.6%	78.6%	56.8%
Uzbekistan	Arnasoy	2017	72.7%	81.6%	62.9%
Uzbekistan	Asaka	2000	66.0%	78.9%	50.7%
Uzbekistan	Asaka	2017	69.9%	81.6%	55.3%
Uzbekistan	Baliqchi	2000	69.5%	79.1%	58.9%
Uzbekistan	Baliqchi	2017	73.7%	82.3%	63.7%
Uzbekistan	Bandixon	2000	72.3%	80.5%	62.5%
Uzbekistan	Bandixon	2017	76.5%	83.9%	67.0%
Uzbekistan	Baxmal	2000	70.8%	78.0%	63.2%
Uzbekistan	Baxmal	2017	74.8%	81.1%	68.4%
Uzbekistan	Bekobod	2000	67.9%	77.8%	56.0%
Uzbekistan	Bekobod	2017	71.9%	80.9%	60.5%
Uzbekistan	Beruniy	2000	67.5%	75.4%	59.6%
Uzbekistan	Beruniy	2017	72.0%	77.3%	66.0%
Uzbekistan	Beshariq	2000	71.6%	79.5%	62.5%
Uzbekistan	Beshariq	2017	75.7%	83.0%	67.5%
Uzbekistan	Bo'ka	2000	71.5%	79.4%	62.4%
Uzbekistan	Bo'ka	2017	75.1%	82.3%	66.6%
Uzbekistan	Bo'stonliq	2000	72.5%	80.5%	63.7%
Uzbekistan	Bo'stonliq	2017	76.2%	82.3%	69.5%

Country	Second Administrative Unit	Year	Mean	Upper	Lower
Uzbekistan	Bo'zsuv	2000	67.8%	81.6%	52.0%
Uzbekistan	Bo'zsuv	2017	71.8%	84.3%	56.9%
Uzbekistan	Bog'dod	2000	66.5%	78.3%	55.6%
Uzbekistan	Bog'dod	2017	70.1%	80.9%	59.6%
Uzbekistan	Bog'ot	2000	71.3%	81.1%	59.0%
Uzbekistan	Bog'ot	2017	74.0%	82.6%	63.1%
Uzbekistan	Boyovut	2000	70.0%	77.1%	60.7%
Uzbekistan	Boyovut	2017	74.3%	80.5%	66.5%
Uzbekistan	Boysun	2000	71.4%	76.2%	66.0%
Uzbekistan	Boysun	2017	75.7%	80.0%	71.2%
Uzbekistan	Buloqboshi	2000	69.3%	81.6%	55.6%
Uzbekistan	Buloqboshi	2017	73.3%	84.3%	60.9%
Uzbekistan	Bulung'ur	2000	71.9%	80.0%	63.4%
Uzbekistan	Bulung'ur	2017	75.8%	82.9%	68.1%
Uzbekistan	Buvayda	2000	66.8%	81.0%	52.1%
Uzbekistan	Buvayda	2017	70.8%	84.0%	57.3%
Uzbekistan	Buxoro	2000	68.8%	78.1%	58.6%
Uzbekistan	Buxoro	2017	72.1%	80.5%	62.0%
Uzbekistan	Chimboy	2000	69.3%	82.3%	52.6%
Uzbekistan	Chimboy	2017	73.4%	81.7%	62.8%
Uzbekistan	Chinoz	2000	65.9%	75.8%	54.2%
Uzbekistan	Chinoz	2017	70.0%	79.3%	59.0%
Uzbekistan	Chiroqchi	2000	73.0%	79.2%	66.3%
Uzbekistan	Chiroqchi	2017	76.3%	82.3%	69.7%
Uzbekistan	Chortoq	2000	75.1%	83.7%	64.3%
Uzbekistan	Chortoq	2017	78.1%	85.9%	69.1%
Uzbekistan	Chust	2000	70.9%	78.9%	61.0%
Uzbekistan	Chust	2017	74.4%	81.6%	65.5%
Uzbekistan	Dang'ara	2000	66.8%	78.4%	54.5%
Uzbekistan	Dang'ara	2017	70.9%	81.6%	58.9%
Uzbekistan	Dehqonobod	2000	73.6%	80.6%	66.2%
Uzbekistan	Dehqonobod	2017	76.9%	83.6%	69.5%
Uzbekistan	Denov	2000	67.3%	79.6%	53.8%
Uzbekistan	Denov	2017	71.4%	82.2%	58.8%
Uzbekistan	Do'stlik	2000	70.3%	81.0%	56.4%
Uzbekistan	Do'stlik	2017	74.0%	84.4%	59.4%
Uzbekistan	Ellikqala	2000	68.5%	77.8%	58.3%
Uzbekistan	Ellikqala	2017	71.6%	78.0%	64.9%
Uzbekistan	Farg'ona	2000	73.3%	80.1%	65.3%
Uzbekistan	Farg'ona	2017	76.8%	83.1%	69.0%
Uzbekistan	Forish	2000	71.1%	74.5%	67.0%
Uzbekistan	Forish	2017	75.0%	78.2%	71.2%
Uzbekistan	Furqat	2000	65.1%	76.3%	50.7%
Uzbekistan	Furqat	2017	69.2%	79.4%	55.9%
Uzbekistan	G'allaorol	2000	70.1%	77.5%	61.4%
Uzbekistan	G'allaorol	2017	73.5%	80.4%	66.6%
Uzbekistan	G'ijduvon	2000	73.6%	78.9%	66.1%
Uzbekistan	G'ijduvon	2017	77.2%	81.2%	70.2%
Uzbekistan	G'uzor	2000	73.7%	80.8%	65.2%
Uzbekistan	G'uzor	2017	77.4%	83.8%	70.3%
Uzbekistan	Guliston	2000	68.7%	78.7%	54.5%
Uzbekistan	Guliston	2017	72.6%	81.6%	59.2%
Uzbekistan	Gurlan	2000	67.4%	76.3%	57.2%
Uzbekistan	Gurlan	2017	71.4%	79.2%	61.7%
Uzbekistan	Hazorasp	2000	72.5%	79.4%	63.4%
Uzbekistan	Hazorasp	2017	76.3%	81.6%	69.0%
Uzbekistan	Ishtixon	2000	68.9%	76.7%	61.4%
Uzbekistan	Ishtixon	2017	73.2%	80.0%	66.3%
Uzbekistan	Izboskan	2000	70.2%	80.2%	57.6%
Uzbekistan	Izboskan	2017	74.0%	82.9%	62.7%
Uzbekistan	Jalolquduq	2000	74.0%	83.0%	63.6%
Uzbekistan	Jalolquduq	2017	77.5%	85.7%	67.3%

Country	Second Administrative Unit	Year	Mean	Upper	Lower
Uzbekistan	Jarqo'rg'on	2000	77.2%	85.4%	68.7%
Uzbekistan	Jarqo'rg'on	2017	80.3%	87.2%	72.8%
Uzbekistan	Jizzax	2000	62.3%	72.9%	50.2%
Uzbekistan	Jizzax	2017	66.5%	76.4%	54.1%
Uzbekistan	Jomboy	2000	68.6%	78.4%	59.3%
Uzbekistan	Jomboy	2017	71.6%	80.0%	62.8%
Uzbekistan	Jondor	2000	72.6%	80.4%	64.2%
Uzbekistan	Jondor	2017	75.7%	81.3%	68.8%
Uzbekistan	Karmana	2000	70.2%	78.3%	60.3%
Uzbekistan	Karmana	2017	73.5%	81.7%	63.8%
Uzbekistan	Kasbi	2000	71.7%	79.5%	61.6%
Uzbekistan	Kasbi	2017	75.1%	82.5%	65.6%
Uzbekistan	Kattaqo'rg'on	2000	70.0%	78.1%	61.5%
Uzbekistan	Kattaqo'rg'on	2017	74.1%	81.1%	65.8%
Uzbekistan	Kegeyli	2000	70.7%	78.2%	60.2%
Uzbekistan	Kegeyli	2017	74.3%	79.1%	67.9%
Uzbekistan	Kitob	2000	73.3%	81.5%	62.5%
Uzbekistan	Kitob	2017	76.8%	84.6%	67.4%
Uzbekistan	Kogon	2000	71.5%	82.3%	57.5%
Uzbekistan	Kogon	2017	75.0%	85.3%	60.7%
Uzbekistan	Konimex	2000	74.7%	80.4%	67.7%
Uzbekistan	Konimex	2017	77.8%	82.0%	73.1%
Uzbekistan	Koson	2000	71.3%	80.7%	59.0%
Uzbekistan	Koson	2017	75.1%	83.0%	65.0%
Uzbekistan	Kosonsoy	2000	66.8%	77.3%	53.4%
Uzbekistan	Kosonsoy	2017	70.8%	80.6%	57.6%
Uzbekistan	Marhamat	2000	72.8%	83.6%	60.4%
Uzbekistan	Marhamat	2017	76.4%	86.2%	64.6%
Uzbekistan	Mingbuloq	2000	64.1%	73.0%	53.6%
Uzbekistan	Mingbuloq	2017	68.6%	76.6%	58.6%
Uzbekistan	Mirzacho'l	2000	69.6%	80.9%	55.9%
Uzbekistan	Mirzacho'l	2017	73.5%	82.8%	62.2%
Uzbekistan	Mirzaobod	2000	69.9%	79.7%	57.5%
Uzbekistan	Mirzaobod	2017	73.5%	82.6%	60.6%
Uzbekistan	Mo'ynoq	2000	70.4%	73.1%	67.9%
Uzbekistan	Mo'ynoq	2017	74.2%	76.6%	71.8%
Uzbekistan	Muborak	2000	75.5%	81.3%	69.1%
Uzbekistan	Muborak	2017	78.7%	84.2%	72.0%
Uzbekistan	Muzrabot	2000	76.7%	85.9%	64.8%
Uzbekistan	Muzrabot	2017	79.9%	87.7%	69.8%
Uzbekistan	Namangan	2000	62.5%	76.1%	44.1%
Uzbekistan	Namangan	2017	66.5%	80.1%	48.1%
Uzbekistan	Narpay	2000	74.4%	82.4%	64.0%
Uzbekistan	Narpay	2017	78.1%	85.1%	69.1%
Uzbekistan	Navbahor	2000	69.8%	80.4%	57.1%
Uzbekistan	Navbahor	2017	73.3%	83.6%	61.5%
Uzbekistan	Nishon	2000	72.8%	78.5%	66.7%
Uzbekistan	Nishon	2017	76.1%	81.4%	70.0%
Uzbekistan	Norin	2000	68.3%	80.6%	54.6%
Uzbekistan	Norin	2017	72.6%	83.6%	59.3%
Uzbekistan	Nukus	2000	66.4%	78.9%	51.7%
Uzbekistan	Nukus	2017	70.6%	81.4%	57.5%
Uzbekistan	Nurobod	2000	74.0%	79.8%	67.0%
Uzbekistan	Nurobod	2017	77.3%	82.2%	70.9%
Uzbekistan	Nurota	2000	71.9%	78.1%	64.6%
Uzbekistan	Nurota	2017	75.3%	79.4%	70.3%
Uzbekistan	O'rtachirchiq	2000	76.1%	83.3%	67.4%
Uzbekistan	O'rtachirchiq	2017	79.3%	86.0%	70.9%
Uzbekistan	O'zbekiston	2000	68.2%	76.0%	56.7%
Uzbekistan	O'zbekiston	2017	72.5%	79.3%	63.8%
Uzbekistan	Ohangaron	2000	72.4%	77.6%	65.9%
Uzbekistan	Ohangaron	2017	75.7%	80.1%	70.1%

Country	Second Administrative Unit	Year	Mean	Upper	Lower
Uzbekistan	Olot	2000	70.4%	80.0%	58.6%
Uzbekistan	Olot	2017	74.4%	82.3%	65.1%
Uzbekistan	Oltiariq	2000	66.4%	79.0%	53.4%
Uzbekistan	Oltiariq	2017	70.6%	81.6%	59.0%
Uzbekistan	Oltinko'l	2000	68.6%	78.3%	58.1%
Uzbekistan	Oltinko'l	2017	72.6%	81.4%	62.6%
Uzbekistan	Oltinsoy	2000	70.9%	80.4%	60.8%
Uzbekistan	Oltinsoy	2017	74.8%	83.9%	63.2%
Uzbekistan	Oqdaryo	2000	71.9%	79.4%	63.2%
Uzbekistan	Oqdaryo	2017	75.9%	83.8%	67.1%
Uzbekistan	Oqoltin	2000	70.4%	79.3%	61.3%
Uzbekistan	Oqoltin	2017	74.3%	82.4%	66.0%
Uzbekistan	Oqqo'rg'on	2000	66.7%	76.9%	57.0%
Uzbekistan	Oqqo'rg'on	2017	70.7%	79.6%	61.9%
Uzbekistan	Oxunboboev	2000	66.7%	77.5%	54.4%
Uzbekistan	Oxunboboev	2017	70.9%	80.8%	58.9%
Uzbekistan	Parkent	2000	72.6%	81.5%	62.0%
Uzbekistan	Parkent	2017	76.2%	84.0%	66.7%
Uzbekistan	Pastdarg'om	2000	73.1%	79.9%	65.1%
Uzbekistan	Pastdarg'om	2017	76.8%	83.0%	69.9%
Uzbekistan	Paxtachi	2000	71.4%	80.3%	61.6%
Uzbekistan	Paxtachi	2017	75.4%	82.1%	67.6%
Uzbekistan	Paxtakor	2000	65.2%	79.1%	49.4%
Uzbekistan	Paxtakor	2017	69.5%	82.5%	53.7%
Uzbekistan	Paxtaobod	2000	68.3%	80.6%	51.3%
Uzbekistan	Paxtaobod	2017	72.1%	83.1%	55.8%
Uzbekistan	Payariq	2000	71.9%	80.5%	62.4%
Uzbekistan	Payariq	2017	75.5%	83.4%	66.3%
Uzbekistan	Peshku	2000	72.4%	80.5%	62.8%
Uzbekistan	Peshku	2017	76.2%	81.7%	69.7%
Uzbekistan	Piskent	2000	66.9%	76.2%	57.4%
Uzbekistan	Piskent	2017	70.9%	79.1%	61.0%
Uzbekistan	Pop	2000	67.5%	75.2%	56.9%
Uzbekistan	Pop	2017	72.0%	78.5%	63.8%
Uzbekistan	Qamashi	2000	75.5%	83.0%	66.8%
Uzbekistan	Qamashi	2017	78.8%	85.6%	70.8%
Uzbekistan	Qanliko'l	2000	70.7%	80.2%	59.7%
Uzbekistan	Qanliko'l	2017	73.5%	81.3%	64.5%
Uzbekistan	Qarshi	2000	70.1%	79.5%	58.7%
Uzbekistan	Qarshi	2017	74.4%	82.5%	63.6%
Uzbekistan	Qibray	2000	54.9%	67.5%	43.2%
Uzbekistan	Qibray	2017	58.4%	70.9%	47.1%
Uzbekistan	Qiziltepa	2000	74.0%	82.0%	64.2%
Uzbekistan	Qiziltepa	2017	77.4%	84.3%	69.2%
Uzbekistan	Qiziriq	2000	74.7%	83.0%	66.4%
Uzbekistan	Qiziriq	2017	78.2%	86.7%	69.5%
Uzbekistan	Qo'ng'irot	2000	70.8%	74.6%	65.9%
Uzbekistan	Qo'ng'irot	2017	74.0%	76.4%	71.6%
Uzbekistan	Qo'rg'ontepa	2000	68.9%	77.2%	58.9%
Uzbekistan	Qo'rg'ontepa	2017	72.9%	80.4%	62.7%
Uzbekistan	Qo'shko'pir	2000	70.7%	78.9%	60.9%
Uzbekistan	Qo'shko'pir	2017	74.0%	81.9%	65.2%
Uzbekistan	Qo'shrabot	2000	71.0%	78.1%	61.9%
Uzbekistan	Qo'shrabot	2017	75.1%	80.9%	66.9%
Uzbekistan	Qorako'l	2000	71.7%	79.7%	62.0%
Uzbekistan	Qorako'l	2017	75.5%	81.6%	68.0%
Uzbekistan	Qorao'zak	2000	70.7%	75.7%	64.6%
Uzbekistan	Qorao'zak	2017	74.2%	78.8%	69.7%
Uzbekistan	Qorovulbozor	2000	72.4%	77.9%	64.3%
Uzbekistan	Qorovulbozor	2017	76.3%	82.3%	68.0%
Uzbekistan	Qumqo'rg'on	2000	71.6%	80.0%	61.9%
Uzbekistan	Qumqo'rg'on	2017	75.6%	83.0%	66.8%

Country	Second Administrative Unit	Year	Mean	Upper	Lower
Uzbekistan	Quva	2000	67.8%	77.9%	57.7%
Uzbekistan	Quva	2017	71.7%	80.8%	61.9%
Uzbekistan	Quyichirchiq	2000	68.1%	77.5%	56.2%
Uzbekistan	Quyichirchiq	2017	72.1%	80.4%	62.1%
Uzbekistan	Rishton	2000	60.4%	76.4%	45.6%
Uzbekistan	Rishton	2017	64.7%	79.5%	50.3%
Uzbekistan	Romitan	2000	73.9%	82.3%	65.3%
Uzbekistan	Romitan	2017	77.1%	83.2%	70.2%
Uzbekistan	Samarqand	2000	68.1%	78.1%	55.8%
Uzbekistan	Samarqand	2017	72.1%	81.7%	60.1%
Uzbekistan	Sariosiyo	2000	67.0%	75.0%	55.4%
Uzbekistan	Sariosiyo	2017	71.7%	78.4%	62.1%
Uzbekistan	Sayxunobod	2000	69.6%	77.3%	59.3%
Uzbekistan	Sayxunobod	2017	74.4%	80.7%	64.7%
Uzbekistan	Shahrisabz	2000	70.1%	81.2%	53.9%
Uzbekistan	Shahrisabz	2017	74.2%	83.4%	60.6%
Uzbekistan	Shahrixon	2000	65.5%	77.8%	51.1%
Uzbekistan	Shahrixon	2017	69.6%	80.8%	56.4%
Uzbekistan	Sharof Rashidov	2000	70.0%	79.8%	57.7%
Uzbekistan	Sharof Rashidov	2017	74.1%	82.8%	63.0%
Uzbekistan	Sherobod	2000	74.6%	81.2%	64.4%
Uzbekistan	Sherobod	2017	77.8%	84.2%	67.5%
Uzbekistan	Sho'rchi	2000	69.0%	81.3%	55.3%
Uzbekistan	Sho'rchi	2017	73.9%	82.3%	63.3%
Uzbekistan	Shofirkon	2000	71.7%	80.4%	60.4%
Uzbekistan	Shofirkon	2017	75.6%	82.9%	65.9%
Uzbekistan	Shovot	2000	67.9%	78.5%	55.2%
Uzbekistan	Shovot	2017	71.4%	81.7%	59.3%
Uzbekistan	Shumanay	2000	70.8%	83.3%	54.8%
Uzbekistan	Shumanay	2017	75.0%	83.3%	65.2%
Uzbekistan	Sirdaryo	2000	69.8%	78.2%	60.2%
Uzbekistan	Sirdaryo	2017	73.5%	81.2%	64.6%
Uzbekistan	So'x	2000	71.4%	83.2%	56.6%
Uzbekistan	So'x	2017	75.1%	85.6%	61.5%
Uzbekistan	Tashkent City	2000	83.5%	88.5%	77.5%
Uzbekistan	Tashkent City	2017	83.6%	88.7%	77.4%
Uzbekistan	Taxtako'pir	2000	68.5%	72.0%	64.2%
Uzbekistan	Taxtako'pir	2017	72.4%	75.5%	69.0%
Uzbekistan	Termiz	2000	74.5%	85.7%	61.3%
Uzbekistan	Termiz	2017	78.0%	88.1%	65.4%
Uzbekistan	To'raqo'rg'on	2000	65.8%	77.0%	52.9%
Uzbekistan	To'raqo'rg'on	2017	69.7%	80.0%	56.6%
Uzbekistan	To'rtko'l	2000	70.9%	77.5%	63.9%
Uzbekistan	To'rtko'l	2017	74.5%	79.2%	69.4%
Uzbekistan	Tomdi	2000	73.8%	78.4%	68.6%
Uzbekistan	Tomdi	2017	77.1%	80.2%	73.2%
Uzbekistan	Toshkent	2000	45.3%	60.2%	30.8%
Uzbekistan	Toshkent	2017	49.3%	63.6%	35.3%
Uzbekistan	Toshloq	2000	66.1%	81.3%	51.0%
Uzbekistan	Toshloq	2017	70.3%	83.9%	56.3%
Uzbekistan	Toyloq	2000	70.9%	82.1%	55.4%
Uzbekistan	Toyloq	2017	74.4%	84.3%	59.4%
Uzbekistan	Uchko'prik	2000	64.6%	76.3%	52.3%
Uzbekistan	Uchko'prik	2017	68.8%	79.8%	56.6%
Uzbekistan	Uchqo'rg'on	2000	68.1%	80.1%	54.2%
Uzbekistan	Uchqo'rg'on	2017	71.6%	82.9%	58.6%
Uzbekistan	Uchquduq	2000	70.9%	72.9%	68.7%
Uzbekistan	Uchquduq	2017	74.7%	76.5%	72.8%
Uzbekistan	Ulug'nor	2000	66.9%	77.5%	55.5%
Uzbekistan	Ulug'nor	2017	71.0%	80.4%	60.2%

Country	Second Administrative Unit	Year	Mean	Upper	Lower
Uzbekistan	Urganch	2000	68.6%	78.7%	58.2%
Uzbekistan	Urganch	2017	72.1%	83.5%	59.1%
Uzbekistan	Urgut	2000	69.4%	78.4%	59.0%
Uzbekistan	Urgut	2017	73.8%	81.6%	64.4%
Uzbekistan	Usmon Yusupov	2000	75.2%	79.8%	69.7%
Uzbekistan	Usmon Yusupov	2017	78.4%	82.6%	73.0%
Uzbekistan	Uychi	2000	66.8%	76.8%	54.1%
Uzbekistan	Uychi	2017	70.6%	80.2%	58.1%
Uzbekistan	Uzun	2000	70.8%	80.2%	58.9%
Uzbekistan	Uzun	2017	75.0%	81.9%	66.9%
Uzbekistan	Vobkent	2000	70.3%	78.3%	60.3%
Uzbekistan	Vobkent	2017	74.4%	81.5%	64.4%
Uzbekistan	Xatirchi	2000	70.6%	77.8%	62.9%
Uzbekistan	Xatirchi	2017	74.1%	81.0%	66.4%
Uzbekistan	Xiva	2000	70.8%	81.7%	59.4%
Uzbekistan	Xiva	2017	73.8%	84.1%	62.0%
Uzbekistan	Xo'jaobod	2000	69.4%	81.1%	55.8%
Uzbekistan	Xo'jaobod	2017	73.4%	83.6%	60.8%
Uzbekistan	Xo'jayli	2000	67.4%	76.3%	55.2%
Uzbekistan	Xo'jayli	2017	71.4%	79.5%	60.7%
Uzbekistan	Xonqa	2000	69.3%	79.1%	57.6%
Uzbekistan	Xonqa	2017	72.9%	82.5%	60.1%
Uzbekistan	Xovos	2000	65.8%	74.6%	57.5%
Uzbekistan	Xovos	2017	70.1%	77.9%	62.5%
Uzbekistan	Yagiqo'rg'on	2000	71.2%	78.1%	63.1%
Uzbekistan	Yagiqo'rg'on	2017	74.8%	81.6%	67.2%
Uzbekistan	Yakkabog'	2000	74.9%	82.8%	64.0%
Uzbekistan	Yakkabog'	2017	78.3%	85.7%	67.4%
Uzbekistan	Yangiariq	2000	66.7%	77.3%	54.3%
Uzbekistan	Yangiariq	2017	71.1%	80.9%	59.9%
Uzbekistan	Yangibozor	2000	68.1%	79.4%	53.6%
Uzbekistan	Yangibozor	2017	72.0%	82.2%	58.2%
Uzbekistan	Yangiobod	2000	69.5%	78.2%	59.8%
Uzbekistan	Yangiobod	2017	73.4%	80.7%	64.8%
Uzbekistan	Yangiyo'l	2000	63.3%	72.6%	50.9%
Uzbekistan	Yangiyo'l	2017	67.7%	77.3%	55.1%
Uzbekistan	Yozyovon	2000	67.1%	80.7%	52.1%
Uzbekistan	Yozyovon	2017	71.6%	82.3%	59.7%
Uzbekistan	Yuqorichirchiq	2000	58.1%	70.6%	45.6%
Uzbekistan	Yuqorichirchiq	2017	62.5%	74.2%	50.0%
Uzbekistan	Zafarobod	2000	68.0%	77.5%	58.3%
Uzbekistan	Zafarobod	2017	72.5%	81.3%	62.9%
Uzbekistan	Zangiota	2000	71.6%	76.9%	64.5%
Uzbekistan	Zangiota	2017	75.6%	80.6%	69.0%
Uzbekistan	Zarbdor	2000	66.7%	75.8%	55.8%
Uzbekistan	Zarbdor	2017	70.9%	79.3%	59.8%
Uzbekistan	Zomin	2000	70.5%	77.3%	62.1%
Uzbekistan	Zomin	2017	74.3%	80.0%	67.2%
**Latin America and Caribbean**					
Bolivia	Abel Iturralde	2000	60.7%	66.8%	55.1%
Bolivia	Abel Iturralde	2017	44.3%	50.8%	38.2%
Bolivia	Alonso de Ibáñez	2000	63.1%	65.6%	60.5%
Bolivia	Alonso de Ibáñez	2017	45.6%	48.3%	43.0%
Bolivia	Andrés Ibáñez	2000	86.0%	86.7%	85.2%
Bolivia	Andrés Ibáñez	2017	74.9%	76.1%	73.8%
Bolivia	Aniceto Arce	2000	78.2%	81.7%	73.9%
Bolivia	Aniceto Arce	2017	63.8%	68.9%	57.9%

Piped Water Proportion by Second Administrative Unit *(continued)*

Country	Second Administrative Unit	Year	Mean	Upper	Lower
Bolivia	Antonio Quijarro	2000	64.0%	66.2%	61.8%
Bolivia	Antonio Quijarro	2017	46.6%	49.4%	44.0%
Bolivia	Arani	2000	58.5%	60.4%	56.5%
Bolivia	Arani	2017	41.1%	43.0%	38.9%
Bolivia	Aroma	2000	76.2%	78.4%	74.0%
Bolivia	Aroma	2017	61.0%	64.1%	58.2%
Bolivia	Arque	2000	61.3%	64.5%	58.1%
Bolivia	Arque	2017	43.8%	46.9%	40.4%
Bolivia	Atahuallpa	2000	71.5%	74.9%	67.8%
Bolivia	Atahuallpa	2017	55.2%	59.5%	50.5%
Bolivia	Ayopaya	2000	61.7%	65.4%	58.0%
Bolivia	Ayopaya	2017	44.3%	48.6%	40.9%
Bolivia	Bautista Saavedra	2000	77.4%	81.3%	72.7%
Bolivia	Bautista Saavedra	2017	62.7%	68.1%	56.8%
Bolivia	Belisario Boeto	2000	68.0%	72.2%	63.5%
Bolivia	Belisario Boeto	2017	51.1%	56.7%	45.9%
Bolivia	Bernardino Bilbao	2000	62.4%	66.1%	58.1%
Bolivia	Bernardino Bilbao	2017	45.0%	49.1%	40.0%
Bolivia	Burnet O'Connor	2000	77.4%	80.1%	74.4%
Bolivia	Burnet O'Connor	2017	63.1%	66.7%	59.7%
Bolivia	Capinota	2000	61.7%	64.4%	58.7%
Bolivia	Capinota	2017	44.0%	46.8%	41.1%
Bolivia	Carangas	2000	72.0%	75.5%	68.8%
Bolivia	Carangas	2017	55.7%	60.4%	51.8%
Bolivia	Carrasco	2000	59.9%	62.0%	57.9%
Bolivia	Carrasco	2017	43.3%	45.4%	41.3%
Bolivia	Cercado	2000	78.8%	79.7%	77.8%
Bolivia	Cercado	2000	58.1%	59.0%	57.0%
Bolivia	Cercado	2000	70.2%	71.9%	68.8%
Bolivia	Cercado	2000	42.8%	45.5%	40.0%
Bolivia	Cercado	2017	64.2%	65.6%	62.5%
Bolivia	Cercado	2017	26.9%	29.1%	24.7%
Bolivia	Cercado	2017	53.5%	55.6%	51.5%
Bolivia	Cercado	2017	40.2%	41.3%	38.9%
Bolivia	Chapare	2000	57.0%	58.6%	55.4%
Bolivia	Chapare	2017	39.5%	41.1%	37.8%
Bolivia	Charcas	2000	62.6%	65.4%	59.8%
Bolivia	Charcas	2017	45.2%	48.4%	42.3%
Bolivia	Chayanta	2000	62.8%	64.7%	60.9%
Bolivia	Chayanta	2017	45.3%	47.6%	43.2%
Bolivia	Chiquitos	2000	86.2%	88.1%	84.6%
Bolivia	Chiquitos	2017	75.5%	78.1%	72.9%
Bolivia	Cordillera	2000	84.3%	86.2%	82.1%
Bolivia	Cordillera	2017	73.0%	75.6%	69.9%
Bolivia	Cornelio Saavedra	2000	62.8%	64.7%	60.8%
Bolivia	Cornelio Saavedra	2017	45.3%	47.4%	43.2%
Bolivia	Daniel Campos	2000	68.0%	74.3%	61.9%
Bolivia	Daniel Campos	2017	52.2%	60.4%	44.3%

Country	Second Administrative Unit	Year	Mean	Upper	Lower
Bolivia	Eduardo Avaroa	2000	69.9%	72.3%	67.3%
Bolivia	Eduardo Avaroa	2017	53.3%	56.1%	50.3%
Bolivia	Eliodoro Camacho	2000	75.7%	79.1%	72.7%
Bolivia	Eliodoro Camacho	2017	60.6%	64.7%	56.6%
Bolivia	Esteban Arce	2000	59.7%	62.7%	56.7%
Bolivia	Esteban Arce	2017	41.2%	44.9%	37.8%
Bolivia	Eustaquio Méndez	2000	79.0%	80.8%	77.1%
Bolivia	Eustaquio Méndez	2017	65.1%	67.8%	62.3%
Bolivia	Federico Román	2000	36.0%	38.4%	33.4%
Bolivia	Federico Román	2017	21.7%	23.4%	20.1%
Bolivia	Florida	2000	87.4%	89.8%	84.9%
Bolivia	Florida	2017	77.3%	81.1%	73.5%
Bolivia	Franz Tamayo	2000	75.4%	79.7%	70.8%
Bolivia	Franz Tamayo	2017	60.1%	66.0%	54.2%
Bolivia	Germán Jordán	2000	58.9%	61.5%	56.0%
Bolivia	Germán Jordán	2017	41.3%	44.0%	38.3%
Bolivia	Gran Chaco	2000	79.1%	81.1%	77.0%
Bolivia	Gran Chaco	2017	64.9%	67.3%	62.3%
Bolivia	Gualberto Villarroel	2000	74.2%	78.2%	69.2%
Bolivia	Gualberto Villarroel	2017	58.5%	63.8%	52.6%
Bolivia	Hernando Siles	2000	62.0%	64.7%	59.2%
Bolivia	Hernando Siles	2017	44.7%	48.4%	41.2%
Bolivia	Ichilo	2000	83.8%	85.4%	81.9%
Bolivia	Ichilo	2017	71.5%	74.1%	68.7%
Bolivia	Ignacio Warnes	2000	86.3%	87.6%	84.8%
Bolivia	Ignacio Warnes	2017	75.3%	77.3%	72.6%
Bolivia	Ingavi	2000	76.2%	78.0%	74.7%
Bolivia	Ingavi	2017	60.9%	63.3%	58.9%
Bolivia	Inquisivi	2000	72.7%	75.3%	70.3%
Bolivia	Inquisivi	2017	57.5%	60.7%	54.2%
Bolivia	Jaime Zudáñez	2000	61.1%	63.7%	58.7%
Bolivia	Jaime Zudáñez	2017	43.5%	46.2%	40.6%
Bolivia	José Ballivián	2000	43.4%	45.5%	41.7%
Bolivia	José Ballivián	2017	27.3%	29.3%	25.8%
Bolivia	José María Avilés	2000	79.0%	81.0%	76.6%
Bolivia	José María Avilés	2017	65.3%	68.0%	61.8%
Bolivia	José María Linares	2000	63.5%	65.7%	61.3%
Bolivia	José María Linares	2017	46.1%	48.8%	43.8%
Bolivia	José Miguel de Velasco	2000	85.5%	87.3%	83.4%

Country	Second Administrative Unit	Year	Mean	Upper	Lower
Bolivia	José Miguel de Velasco	2017	74.3%	77.2%	71.4%
Bolivia	Juana Azurduay de Padilla	2000	60.8%	63.5%	57.8%
Bolivia	Juana Azurduay de Padilla	2017	43.3%	46.2%	40.4%
Bolivia	Ladislao Cabrera	2000	70.2%	73.7%	66.4%
Bolivia	Ladislao Cabrera	2017	53.6%	57.9%	49.4%
Bolivia	Larecaja	2000	76.7%	79.5%	73.7%
Bolivia	Larecaja	2017	62.0%	65.7%	58.2%
Bolivia	Litoral	2000	71.1%	75.7%	65.4%
Bolivia	Litoral	2017	53.2%	58.5%	48.2%
Bolivia	Loayza	2000	76.0%	78.8%	73.6%
Bolivia	Loayza	2017	61.0%	64.8%	57.8%
Bolivia	Los Andes	2000	76.7%	79.0%	74.6%
Bolivia	Los Andes	2017	61.6%	64.7%	58.5%
Bolivia	Luis Calvo	2000	70.9%	73.6%	68.0%
Bolivia	Luis Calvo	2017	54.6%	58.1%	51.4%
Bolivia	Madre de Dios	2000	37.4%	39.6%	35.3%
Bolivia	Madre de Dios	2017	22.5%	24.1%	21.1%
Bolivia	Mamoré	2000	43.9%	49.5%	38.7%
Bolivia	Mamoré	2017	27.9%	32.2%	23.6%
Bolivia	Manco Kapac	2000	75.4%	79.4%	71.3%
Bolivia	Manco Kapac	2017	59.4%	64.8%	54.1%
Bolivia	Manuel María Caballero	2000	81.5%	84.4%	78.7%
Bolivia	Manuel María Caballero	2017	68.4%	72.7%	64.2%
Bolivia	Manuripi	2000	36.3%	39.0%	33.4%
Bolivia	Manuripi	2017	21.4%	23.7%	19.4%
Bolivia	Marbán	2000	59.0%	61.5%	56.5%
Bolivia	Marbán	2017	46.7%	49.7%	43.7%
Bolivia	Mizque	2000	58.1%	61.5%	54.5%
Bolivia	Mizque	2017	40.8%	44.0%	37.5%
Bolivia	Modesto Omiste	2000	64.9%	68.5%	60.5%
Bolivia	Modesto Omiste	2017	47.4%	51.4%	42.6%
Bolivia	Moxos	2000	42.7%	45.3%	40.2%
Bolivia	Moxos	2017	26.9%	29.1%	25.0%
Bolivia	Muñecas	2000	76.4%	80.5%	72.5%
Bolivia	Muñecas	2017	61.6%	66.9%	56.2%
Bolivia	Narciso Campero	2000	61.4%	64.0%	58.0%
Bolivia	Narciso Campero	2017	45.5%	48.4%	42.5%
Bolivia	Nor Chichas	2000	63.1%	66.0%	60.2%
Bolivia	Nor Chichas	2017	45.8%	48.8%	42.6%
Bolivia	Nor Cinti	2000	61.7%	63.7%	59.6%
Bolivia	Nor Cinti	2017	44.0%	46.6%	41.7%
Bolivia	Nor Lípez	2000	62.7%	67.5%	57.6%
Bolivia	Nor Lípez	2017	45.4%	50.8%	40.3%
Bolivia	Nor Yungas	2000	76.1%	78.1%	73.8%
Bolivia	Nor Yungas	2017	60.8%	63.7%	57.4%
Bolivia	Ñuflo de Chávez	2000	85.9%	87.5%	84.4%
Bolivia	Ñuflo de Chávez	2017	75.0%	77.5%	72.5%
Bolivia	Obispo Santistevan	2000	86.1%	87.6%	84.7%

Country	Second Administrative Unit	Year	Mean	Upper	Lower
Bolivia	Obispo Santistevan	2017	75.3%	77.8%	72.8%
Bolivia	Omasuyos	2000	76.5%	78.9%	74.4%
Bolivia	Omasuyos	2017	61.5%	64.6%	58.7%
Bolivia	Oropeza	2000	60.8%	61.8%	59.7%
Bolivia	Oropeza	2017	43.1%	44.3%	41.8%
Bolivia	Pacajes	2000	75.8%	78.6%	72.5%
Bolivia	Pacajes	2017	60.8%	64.4%	56.5%
Bolivia	Pantaleón Dalence	2000	70.9%	73.3%	68.8%
Bolivia	Pantaleón Dalence	2017	54.6%	57.6%	51.8%
Bolivia	Pedro Domingo Murillo	2000	75.9%	76.8%	74.8%
Bolivia	Pedro Domingo Murillo	2017	60.6%	61.9%	59.1%
Bolivia	Poopó	2000	70.9%	73.8%	68.2%
Bolivia	Poopó	2017	54.7%	58.6%	51.2%
Bolivia	Punata	2000	58.5%	60.6%	56.3%
Bolivia	Punata	2017	40.9%	43.1%	38.3%
Bolivia	Quillacollo	2000	61.1%	62.6%	59.9%
Bolivia	Quillacollo	2017	43.4%	45.0%	42.0%
Bolivia	Rafael Bustillo	2000	63.6%	65.6%	61.7%
Bolivia	Rafael Bustillo	2017	46.0%	48.2%	43.9%
Bolivia	Sajama	2000	72.4%	76.1%	68.6%
Bolivia	Sajama	2017	56.3%	60.8%	51.8%
Bolivia	Sara	2000	86.3%	87.9%	84.7%
Bolivia	Sara	2017	75.6%	78.2%	73.1%
Bolivia	Saucarí	2000	70.8%	74.6%	66.5%
Bolivia	Saucarí	2017	54.1%	58.8%	48.3%
Bolivia	Sud Chichas	2000	64.1%	66.7%	61.4%
Bolivia	Sud Chichas	2017	46.8%	49.4%	43.9%
Bolivia	Sud Cinti	2000	64.9%	67.7%	61.7%
Bolivia	Sud Cinti	2017	47.8%	51.1%	44.1%
Bolivia	Sud Lípez	2000	61.8%	70.4%	53.2%
Bolivia	Sud Lípez	2017	44.7%	54.6%	35.3%
Bolivia	Sud Yungas	2000	72.4%	74.6%	69.9%
Bolivia	Sud Yungas	2017	57.2%	60.3%	53.5%
Bolivia	Tapacarí	2000	62.1%	65.5%	58.4%
Bolivia	Tapacarí	2017	44.5%	48.6%	40.8%
Bolivia	Tomás Frías	2000	62.2%	64.2%	60.4%
Bolivia	Tomás Frías	2017	44.4%	46.7%	42.6%
Bolivia	Tomina	2000	60.8%	64.0%	58.0%
Bolivia	Tomina	2017	43.1%	46.9%	40.0%
Bolivia	Vaca Díez	2000	42.2%	43.6%	40.8%
Bolivia	Vaca Díez	2017	26.6%	27.7%	25.5%
Bolivia	Vallegrande	2000	83.2%	85.9%	80.3%
Bolivia	Vallegrande	2017	71.2%	75.3%	67.0%
Bolivia	Yacuma	2000	43.2%	47.0%	39.8%
Bolivia	Yacuma	2017	27.2%	30.0%	24.5%
Bolivia	Yamparáez	2000	61.2%	64.1%	58.5%
Bolivia	Yamparáez	2017	43.7%	46.6%	41.0%
Brazil	Abadia de Goiás	2000	76.8%	78.9%	74.5%
Brazil	Abadia de Goiás	2017	78.8%	80.8%	76.7%
Brazil	Abadia dos Dourados	2000	79.3%	84.2%	73.7%

Country	Second Administrative Unit	Year	Mean	Upper	Lower
Brazil	Abadia dos Dourados	2017	79.9%	84.7%	74.5%
Brazil	Abadiânia	2000	81.5%	83.8%	78.7%
Brazil	Abadiânia	2017	82.1%	84.3%	79.4%
Brazil	Abaeté	2000	80.2%	85.6%	73.2%
Brazil	Abaeté	2017	80.8%	86.1%	73.9%
Brazil	Abaetetuba	2000	37.1%	42.1%	33.1%
Brazil	Abaetetuba	2017	38.9%	44.0%	34.8%
Brazil	Abaiara	2000	67.1%	70.9%	63.5%
Brazil	Abaiara	2017	68.0%	71.7%	64.4%
Brazil	Abaíra	2000	77.0%	82.1%	71.6%
Brazil	Abaíra	2017	77.6%	82.7%	72.4%
Brazil	Abaré	2000	65.5%	71.4%	59.6%
Brazil	Abaré	2017	66.3%	72.1%	60.4%
Brazil	Abatiá	2000	89.0%	91.3%	86.4%
Brazil	Abatiá	2017	89.3%	91.6%	86.8%
Brazil	Abdon Batista	2000	75.5%	79.9%	70.6%
Brazil	Abdon Batista	2017	76.3%	80.5%	71.4%
Brazil	Abel Figueiredo	2000	51.1%	56.9%	44.8%
Brazil	Abel Figueiredo	2017	52.1%	57.9%	45.8%
Brazil	Abelardo Luz	2000	77.8%	82.0%	73.0%
Brazil	Abelardo Luz	2017	78.4%	82.5%	73.8%
Brazil	Abre Campo	2000	82.7%	86.3%	78.7%
Brazil	Abre Campo	2017	83.3%	86.8%	79.3%
Brazil	Abreu e Lima	2000	64.7%	66.8%	62.6%
Brazil	Abreu e Lima	2017	67.1%	69.2%	65.1%
Brazil	Abreulândia	2000	69.1%	74.5%	63.9%
Brazil	Abreulândia	2017	70.0%	75.2%	64.9%
Brazil	Acaiaca	2000	80.4%	83.9%	76.3%
Brazil	Acaiaca	2017	81.0%	84.5%	77.0%
Brazil	Açailândia	2000	62.8%	68.0%	57.4%
Brazil	Açailândia	2017	63.7%	68.8%	58.4%
Brazil	Acajutiba	2000	78.2%	82.1%	74.6%
Brazil	Acajutiba	2017	78.9%	82.7%	75.3%
Brazil	Acará	2000	37.3%	40.8%	33.6%
Brazil	Acará	2017	38.2%	41.7%	34.5%
Brazil	Acarapé	2000	69.5%	72.6%	65.9%
Brazil	Acarapé	2017	70.5%	73.6%	67.1%
Brazil	Acaraú	2000	67.7%	73.1%	61.8%
Brazil	Acaraú	2017	68.5%	73.9%	62.7%
Brazil	Acari	2000	79.3%	82.6%	75.8%
Brazil	Acari	2017	80.0%	83.2%	76.5%
Brazil	Acauã	2000	64.0%	69.5%	58.1%
Brazil	Acauã	2017	64.9%	70.3%	59.0%
Brazil	Acopiara	2000	70.4%	74.8%	66.4%
Brazil	Acopiara	2017	71.2%	75.5%	67.3%
Brazil	Acorizal	2000	66.6%	71.2%	61.5%
Brazil	Acorizal	2017	67.5%	72.0%	62.5%
Brazil	Acrelândia	2000	40.6%	45.9%	35.8%
Brazil	Acrelândia	2017	41.6%	46.9%	36.7%
Brazil	Acreúna	2000	76.5%	80.8%	71.1%
Brazil	Acreúna	2017	77.2%	81.4%	71.9%
Brazil	Açu	2000	81.9%	84.8%	79.0%
Brazil	Açu	2017	82.5%	85.3%	79.6%
Brazil	Açucena	2000	80.9%	85.0%	76.3%
Brazil	Açucena	2017	81.5%	85.5%	77.0%
Brazil	Adamantina	2000	91.2%	93.3%	88.3%
Brazil	Adamantina	2017	91.5%	93.6%	88.7%

Country	Second Administrative Unit	Year	Mean	Upper	Lower
Brazil	Adelândia	2000	76.1%	79.7%	71.6%
Brazil	Adelândia	2017	76.8%	80.3%	72.4%
Brazil	Adolfo	2000	92.9%	94.8%	90.8%
Brazil	Adolfo	2017	93.1%	95.0%	91.1%
Brazil	Adrianópolis	2000	86.4%	89.7%	82.7%
Brazil	Adrianópolis	2017	86.8%	90.0%	83.3%
Brazil	Adustina	2000	78.2%	81.6%	74.6%
Brazil	Adustina	2017	78.9%	82.2%	75.3%
Brazil	Afogados da Ingazeira	2000	68.0%	72.2%	63.4%
Brazil	Afogados da Ingazeira	2017	68.8%	73.0%	64.3%
Brazil	Afonso Bezerra	2000	83.2%	86.1%	80.2%
Brazil	Afonso Bezerra	2017	83.7%	86.5%	80.8%
Brazil	Afonso Cláudio	2000	79.8%	83.2%	76.8%
Brazil	Afonso Cláudio	2017	80.5%	83.7%	77.5%
Brazil	Afonso Cunha	2000	61.0%	65.3%	56.0%
Brazil	Afonso Cunha	2017	62.0%	66.2%	56.9%
Brazil	Afrânio	2000	65.4%	70.7%	60.0%
Brazil	Afrânio	2017	66.2%	71.5%	60.9%
Brazil	Afuá	2000	34.2%	38.4%	30.3%
Brazil	Afuá	2017	35.1%	39.3%	31.2%
Brazil	Agrestina	2000	65.5%	69.2%	62.5%
Brazil	Agrestina	2017	66.4%	70.0%	63.4%
Brazil	Agricolândia	2000	60.6%	65.2%	55.4%
Brazil	Agricolândia	2017	61.5%	66.1%	56.4%
Brazil	Agrolândia	2000	73.6%	77.9%	68.8%
Brazil	Agrolândia	2017	74.3%	78.5%	69.7%
Brazil	Agronômica	2000	74.3%	78.3%	70.1%
Brazil	Agronômica	2017	75.1%	79.0%	71.0%
Brazil	Água Azul do Norte	2000	38.5%	44.7%	32.2%
Brazil	Água Azul do Norte	2017	39.3%	45.5%	33.1%
Brazil	Água Boa	2000	71.6%	79.2%	64.3%
Brazil	Água Boa	2000	82.2%	86.9%	76.3%
Brazil	Água Boa	2017	72.4%	79.8%	65.2%
Brazil	Água Boa	2017	82.8%	87.4%	77.1%
Brazil	Água Branca	2000	64.4%	68.3%	60.4%
Brazil	Água Branca	2000	59.8%	64.4%	54.4%
Brazil	Água Branca	2000	67.5%	71.9%	63.5%
Brazil	Água Branca	2017	66.2%	69.9%	62.2%
Brazil	Água Branca	2017	69.7%	73.9%	65.9%
Brazil	Água Branca	2017	60.7%	65.3%	55.4%
Brazil	Água Clara	2000	79.4%	83.9%	74.3%
Brazil	Água Clara	2017	80.0%	84.4%	75.0%
Brazil	Água Comprida	2000	87.8%	90.9%	84.1%
Brazil	Água Comprida	2017	88.2%	91.2%	84.6%
Brazil	Água Doce	2000	78.0%	81.9%	73.8%
Brazil	Água Doce	2017	78.7%	82.5%	74.6%
Brazil	Água Doce do Maranhão	2000	60.3%	65.2%	55.9%
Brazil	Água Doce do Maranhão	2017	61.2%	66.1%	56.8%
Brazil	Água Doce do Norte	2000	80.2%	83.8%	76.4%

Country	Second Administrative Unit	Year	Mean	Upper	Lower
Brazil	Água Doce do Norte	2017	80.8%	84.3%	77.1%
Brazil	Água Fria	2000	78.8%	82.2%	74.6%
Brazil	Água Fria	2017	79.4%	82.7%	75.3%
Brazil	Água Fria de Goiás	2000	88.8%	91.5%	85.5%
Brazil	Água Fria de Goiás	2017	89.3%	91.8%	86.1%
Brazil	Água Limpa	2000	77.5%	81.6%	73.3%
Brazil	Água Limpa	2017	78.2%	82.2%	74.0%
Brazil	Água Nova	2000	75.3%	78.8%	71.7%
Brazil	Água Nova	2017	76.0%	79.4%	72.5%
Brazil	Água Preta	2000	62.4%	65.9%	58.9%
Brazil	Água Preta	2017	63.3%	66.8%	59.9%
Brazil	Água Santa	2000	80.3%	84.0%	75.4%
Brazil	Água Santa	2017	80.9%	84.5%	76.1%
Brazil	Aguai	2000	91.6%	93.4%	89.2%
Brazil	Aguai	2017	91.9%	93.6%	89.6%
Brazil	Aguanil	2000	81.8%	85.6%	77.2%
Brazil	Aguanil	2017	82.4%	86.1%	77.9%
Brazil	Águas Belas	2000	62.7%	66.7%	58.9%
Brazil	Águas Belas	2017	63.6%	67.6%	59.9%
Brazil	Águas da Prata	2000	90.7%	92.8%	88.1%
Brazil	Águas da Prata	2017	91.0%	93.1%	88.5%
Brazil	Águas de Chapecó	2000	75.7%	79.5%	71.4%
Brazil	Águas de Chapecó	2017	76.4%	80.2%	72.2%
Brazil	Águas de Lindóia	2000	91.5%	93.3%	89.6%
Brazil	Águas de Lindóia	2017	91.8%	93.5%	89.9%
Brazil	Águas de Santa Bárbara	2000	92.6%	94.6%	90.1%
Brazil	Águas de Santa Bárbara	2017	92.8%	94.8%	90.5%
Brazil	Águas de São Pedro	2000	92.8%	94.3%	91.0%
Brazil	Águas de São Pedro	2017	93.0%	94.5%	91.3%
Brazil	Águas Formosas	2000	81.7%	85.6%	76.3%
Brazil	Águas Formosas	2017	82.2%	86.1%	77.0%
Brazil	Águas Frias	2000	76.3%	80.8%	71.5%
Brazil	Águas Frias	2017	77.0%	81.4%	72.3%
Brazil	Águas Lindas de Goiás	2000	90.3%	91.8%	89.0%
Brazil	Águas Lindas de Goiás	2017	91.1%	92.4%	89.8%
Brazil	Águas Mornas	2000	74.3%	78.7%	69.1%
Brazil	Águas Mornas	2017	74.9%	79.2%	69.7%
Brazil	Águas Vermelhas	2000	79.9%	84.3%	74.6%
Brazil	Águas Vermelhas	2017	80.6%	84.8%	75.4%
Brazil	Agudo	2000	81.7%	85.1%	77.1%

Country	Second Administrative Unit	Year	Mean	Upper	Lower
Brazil	Agudo	2017	82.3%	85.6%	77.8%
Brazil	Agudos	2000	93.8%	95.6%	91.9%
Brazil	Agudos	2017	94.0%	95.7%	92.2%
Brazil	Agudos do Sul	2000	81.8%	84.5%	78.7%
Brazil	Agudos do Sul	2017	82.4%	85.0%	79.3%
Brazil	Aguiar	2000	69.1%	72.8%	65.1%
Brazil	Aguiar	2017	70.0%	73.6%	66.0%
Brazil	Aguiarnópolis	2000	70.5%	75.3%	65.9%
Brazil	Aguiarnópolis	2017	71.2%	76.0%	66.5%
Brazil	Aimorés	2000	75.9%	79.9%	72.0%
Brazil	Aimorés	2017	76.7%	80.5%	72.8%
Brazil	Aiquara	2000	76.1%	81.0%	71.3%
Brazil	Aiquara	2017	76.8%	81.6%	72.1%
Brazil	Aiuaba	2000	69.9%	73.9%	65.3%
Brazil	Aiuaba	2017	70.7%	74.7%	66.1%
Brazil	Aiuruoca	2000	85.2%	88.2%	81.6%
Brazil	Aiuruoca	2017	85.7%	88.6%	82.2%
Brazil	Ajuricaba	2000	82.2%	86.2%	77.8%
Brazil	Ajuricaba	2017	82.7%	86.7%	78.4%
Brazil	Alagoa	2000	86.8%	89.2%	84.0%
Brazil	Alagoa	2017	87.2%	89.6%	84.5%
Brazil	Alagoa Grande	2000	66.6%	69.7%	63.0%
Brazil	Alagoa Grande	2017	67.5%	70.6%	64.0%
Brazil	Alagoa Nova	2000	66.2%	69.3%	62.8%
Brazil	Alagoa Nova	2017	67.1%	70.1%	63.8%
Brazil	Alagoinha	2000	68.0%	71.6%	63.8%
Brazil	Alagoinha	2000	68.0%	71.0%	64.6%
Brazil	Alagoinha	2017	68.8%	71.8%	65.5%
Brazil	Alagoinha	2017	68.8%	72.4%	64.7%
Brazil	Alagoinha do Piauí	2000	63.9%	68.2%	59.8%
Brazil	Alagoinha do Piauí	2017	64.7%	69.1%	60.6%
Brazil	Alagoinhas	2000	77.4%	80.5%	73.5%
Brazil	Alagoinhas	2017	78.0%	81.1%	74.2%
Brazil	Alambari	2000	93.5%	95.1%	91.6%
Brazil	Alambari	2017	93.8%	95.3%	91.9%
Brazil	Albertina	2000	90.2%	92.3%	87.5%
Brazil	Albertina	2017	90.5%	92.6%	87.9%
Brazil	Alcântara	2000	58.2%	62.3%	53.3%
Brazil	Alcântara	2017	59.2%	63.3%	54.3%
Brazil	Alcântaras	2000	71.3%	74.7%	67.6%
Brazil	Alcântaras	2017	72.1%	75.4%	68.5%
Brazil	Alcantil	2000	68.0%	71.4%	64.3%
Brazil	Alcantil	2017	68.9%	72.2%	65.2%
Brazil	Alcinópolis	2000	77.2%	82.0%	71.2%
Brazil	Alcinópolis	2017	77.8%	82.6%	72.0%
Brazil	Alcobaca	2000	76.4%	82.5%	69.2%
Brazil	Alcobaca	2017	77.1%	83.1%	70.0%
Brazil	Aldeias Altas	2000	60.3%	64.7%	55.8%
Brazil	Aldeias Altas	2017	61.3%	65.6%	56.7%
Brazil	Alecrim	2000	80.8%	85.8%	74.3%
Brazil	Alecrim	2017	81.3%	86.2%	74.9%
Brazil	Alegre	2000	77.8%	81.4%	74.5%
Brazil	Alegre	2017	78.4%	82.0%	75.2%
Brazil	Alegrete	2000	82.7%	87.5%	76.3%
Brazil	Alegrete	2017	83.3%	87.9%	77.0%
Brazil	Alegrete do Piauí	2000	64.3%	69.2%	59.8%
Brazil	Alegrete do Piauí	2017	65.3%	70.0%	60.8%

Country	Second Administrative Unit	Year	Mean	Upper	Lower
Brazil	Alegria	2000	81.0%	85.2%	76.0%
Brazil	Alegria	2017	81.6%	85.7%	76.6%
Brazil	Além Paraíba	2000	77.6%	81.1%	73.5%
Brazil	Além Paraíba	2017	78.3%	81.8%	74.3%
Brazil	Alenquer	2000	40.0%	46.9%	33.5%
Brazil	Alenquer	2017	41.1%	48.0%	34.5%
Brazil	Alexandria	2000	76.3%	79.8%	72.7%
Brazil	Alexandria	2017	77.0%	80.4%	73.5%
Brazil	Alexania	2000	85.0%	87.1%	82.9%
Brazil	Alexania	2017	85.5%	87.6%	83.5%
Brazil	Alexânia	2000	84.3%	86.6%	82.0%
Brazil	Alexânia	2017	84.7%	87.0%	82.6%
Brazil	Alfenas	2000	85.4%	88.9%	81.2%
Brazil	Alfenas	2017	85.9%	89.2%	81.8%
Brazil	Alfredo Chaves	2000	79.5%	82.1%	77.0%
Brazil	Alfredo Chaves	2017	80.3%	82.8%	77.9%
Brazil	Alfredo Marconde	2000	89.1%	91.8%	86.4%
Brazil	Alfredo Marconde	2017	89.5%	92.1%	86.8%
Brazil	Alfredo Vasconcelos	2000	84.4%	87.4%	80.8%
Brazil	Alfredo Vasconcelos	2017	84.9%	87.8%	81.4%
Brazil	Alfredo Wagner	2000	76.1%	79.8%	70.6%
Brazil	Alfredo Wagner	2017	76.9%	80.4%	71.5%
Brazil	Algodão de Jandaíra	2000	73.7%	77.0%	70.4%
Brazil	Algodão de Jandaíra	2017	74.5%	77.7%	71.2%
Brazil	Alhandra	2000	64.7%	67.9%	61.7%
Brazil	Alhandra	2017	65.6%	68.8%	62.6%
Brazil	Aliança	2000	67.5%	70.9%	64.5%
Brazil	Aliança	2017	68.3%	71.7%	65.4%
Brazil	Aliança do Tocantins	2000	76.1%	80.1%	72.4%
Brazil	Aliança do Tocantins	2017	76.8%	80.7%	73.2%
Brazil	Almadina	2000	78.2%	82.1%	73.8%
Brazil	Almadina	2017	78.9%	82.7%	74.5%
Brazil	Almas	2000	76.9%	82.1%	71.4%
Brazil	Almas	2017	77.6%	82.7%	72.2%
Brazil	Almenara	2000	80.6%	85.2%	74.1%
Brazil	Almenara	2017	81.2%	85.7%	74.9%
Brazil	Almerim	2000	44.5%	50.3%	38.8%
Brazil	Almerim	2017	46.1%	51.8%	40.2%
Brazil	Almino Afonso	2000	78.3%	81.3%	74.8%
Brazil	Almino Afonso	2017	78.9%	81.8%	75.5%
Brazil	Almirante Tamandaré	2000	85.7%	87.8%	83.7%
Brazil	Almirante Tamandaré	2017	86.2%	88.2%	84.2%
Brazil	Aloândia	2000	76.7%	80.8%	72.5%
Brazil	Aloândia	2017	77.4%	81.4%	73.3%
Brazil	Alpercata	2000	79.4%	83.8%	75.2%
Brazil	Alpercata	2017	80.1%	84.4%	76.0%

Country	Second Administrative Unit	Year	Mean	Upper	Lower
Brazil	Alpestre	2000	76.5%	80.1%	72.3%
Brazil	Alpestre	2017	77.1%	80.6%	73.0%
Brazil	Alpinópolis	2000	84.9%	88.8%	80.1%
Brazil	Alpinópolis	2017	85.4%	89.2%	80.7%
Brazil	Alta Floresta	2000	65.6%	71.7%	56.8%
Brazil	Alta Floresta	2017	66.5%	72.5%	57.8%
Brazil	Alta Floresta d'Oeste	2000	30.8%	34.9%	26.3%
Brazil	Alta Floresta d'Oeste	2017	31.6%	35.8%	27.1%
Brazil	Altair	2000	91.3%	93.6%	88.8%
Brazil	Altair	2017	91.6%	93.8%	89.2%
Brazil	Altamira	2000	42.7%	50.5%	35.0%
Brazil	Altamira	2017	43.6%	51.4%	35.9%
Brazil	Altamira do Maranhão	2000	61.2%	66.2%	55.8%
Brazil	Altamira do Maranhão	2017	62.1%	67.0%	56.7%
Brazil	Altamira do Paran	2000	83.0%	87.0%	79.3%
Brazil	Altamira do Paran	2017	83.6%	87.4%	80.0%
Brazil	Altaneira	2000	69.3%	73.2%	65.0%
Brazil	Altaneira	2017	70.2%	73.9%	65.9%
Brazil	Alterosa	2000	85.5%	88.8%	81.8%
Brazil	Alterosa	2017	86.0%	89.2%	82.4%
Brazil	Altinho	2000	66.3%	69.8%	63.0%
Brazil	Altinho	2017	67.2%	70.6%	63.9%
Brazil	Altinópolis	2000	90.5%	92.7%	87.5%
Brazil	Altinópolis	2017	90.9%	93.0%	88.1%
Brazil	Alto Alegre	2000	85.4%	87.9%	82.4%
Brazil	Alto Alegre	2000	92.3%	94.4%	90.0%
Brazil	Alto Alegre	2000	82.1%	86.0%	77.5%
Brazil	Alto Alegre	2017	92.6%	94.6%	90.4%
Brazil	Alto Alegre	2017	85.4%	87.9%	82.4%
Brazil	Alto Alegre	2017	82.7%	86.5%	78.1%
Brazil	Alto Alegre do Maranho	2000	62.2%	66.4%	57.1%
Brazil	Alto Alegre do Maranho	2017	63.1%	67.2%	58.1%
Brazil	Alto Alegre do Pindaré	2000	58.5%	63.5%	53.0%
Brazil	Alto Alegre do Pindaré	2017	59.5%	64.3%	54.0%
Brazil	Alto Alegre dos Parecis	2000	30.8%	35.6%	26.1%
Brazil	Alto Alegre dos Parecis	2017	32.7%	37.7%	27.9%
Brazil	Alto Araguaia	2000	74.8%	80.2%	68.3%
Brazil	Alto Araguaia	2017	75.6%	80.9%	69.2%
Brazil	Alto Bela Vista	2000	75.7%	79.6%	70.8%
Brazil	Alto Bela Vista	2017	76.5%	80.2%	71.7%
Brazil	Alto Boa Vista	2000	70.9%	78.7%	61.8%
Brazil	Alto Boa Vista	2017	71.7%	79.3%	62.7%
Brazil	Alto Caparaó	2000	84.4%	87.3%	81.5%
Brazil	Alto Caparaó	2017	85.0%	87.8%	82.2%
Brazil	Alto do Rodrigues	2000	83.2%	86.5%	80.2%

Country	Second Administrative Unit	Year	Mean	Upper	Lower
Brazil	Alto do Rodrigues	2017	83.8%	87.0%	80.9%
Brazil	Alto Feliz	2000	82.5%	85.2%	79.9%
Brazil	Alto Feliz	2017	83.1%	85.7%	80.6%
Brazil	Alto Garças	2000	73.9%	79.3%	67.7%
Brazil	Alto Garças	2017	74.6%	79.9%	68.5%
Brazil	Alto Horizonte	2000	76.9%	81.9%	71.7%
Brazil	Alto Horizonte	2017	77.6%	82.5%	72.5%
Brazil	Alto Jequitibá	2000	80.6%	83.9%	76.7%
Brazil	Alto Jequitibá	2017	81.2%	84.4%	77.4%
Brazil	Alto Longá	2000	59.1%	63.7%	54.7%
Brazil	Alto Longá	2017	60.1%	64.6%	55.6%
Brazil	Alto Paraguai	2000	67.8%	74.1%	61.8%
Brazil	Alto Paraguai	2017	68.7%	74.8%	62.8%
Brazil	Alto Paraíso	2000	27.2%	31.6%	23.5%
Brazil	Alto Paraíso	2017	28.1%	32.5%	24.3%
Brazil	Alto Paraíso de Goiás	2000	84.0%	87.9%	79.1%
Brazil	Alto Paraíso de Goiás	2017	84.5%	88.3%	79.7%
Brazil	Alto Paraná	2000	85.9%	88.6%	82.8%
Brazil	Alto Paraná	2017	86.4%	89.0%	83.3%
Brazil	Alto Parnaiba	2000	65.6%	73.1%	58.5%
Brazil	Alto Parnaiba	2017	66.5%	73.9%	59.4%
Brazil	Alto Piquiri	2000	83.8%	87.6%	80.1%
Brazil	Alto Piquiri	2017	84.3%	88.1%	80.7%
Brazil	Alto Rio doce	2000	80.6%	84.0%	76.5%
Brazil	Alto Rio doce	2017	81.2%	84.6%	77.2%
Brazil	Alto Rio Novo	2000	80.1%	83.9%	76.1%
Brazil	Alto Rio Novo	2017	80.7%	84.4%	76.8%
Brazil	Alto Santo	2000	73.7%	77.5%	69.1%
Brazil	Alto Santo	2017	74.4%	78.1%	70.0%
Brazil	Alto Taquari	2000	77.9%	82.9%	72.3%
Brazil	Alto Taquari	2017	78.5%	83.4%	73.1%
Brazil	Altônia	2000	83.5%	87.0%	79.5%
Brazil	Altônia	2017	84.1%	87.4%	80.2%
Brazil	Altos	2000	60.7%	64.2%	57.0%
Brazil	Altos	2017	61.6%	65.0%	57.9%
Brazil	Alumínio	2000	93.8%	95.0%	92.6%
Brazil	Alumínio	2017	94.1%	95.2%	92.8%
Brazil	Alvarães	2000	63.3%	69.0%	57.1%
Brazil	Alvarães	2017	64.1%	69.8%	58.0%
Brazil	Alvarenga	2000	80.6%	84.2%	76.7%
Brazil	Alvarenga	2017	81.2%	84.7%	77.4%
Brazil	Álvares Florence	2000	90.4%	93.2%	87.3%
Brazil	Álvares Florence	2017	90.7%	93.5%	87.8%
Brazil	Álvares Machado	2000	89.0%	91.7%	86.4%
Brazil	Álvares Machado	2017	89.4%	92.0%	86.8%
Brazil	Álvaro de Carvalho	2000	93.1%	95.0%	90.6%
Brazil	Álvaro de Carvalho	2017	93.3%	95.2%	90.9%
Brazil	Alvinlândia	2000	92.4%	94.3%	90.1%
Brazil	Alvinlândia	2017	92.7%	94.5%	90.4%
Brazil	Alvinópolis	2000	81.2%	85.2%	77.6%
Brazil	Alvinópolis	2017	81.8%	85.7%	78.3%

Country	Second Administrative Unit	Year	Mean	Upper	Lower
Brazil	Alvorada	2000	75.9%	81.4%	70.4%
Brazil	Alvorada	2000	82.5%	84.4%	80.6%
Brazil	Alvorada	2017	76.6%	82.0%	71.2%
Brazil	Alvorada	2017	83.0%	84.9%	81.2%
Brazil	Alvorada d'Oeste	2000	28.2%	31.8%	24.8%
Brazil	Alvorada d'Oeste	2017	29.0%	32.6%	25.5%
Brazil	Alvorada de Minas	2000	80.9%	85.2%	74.9%
Brazil	Alvorada de Minas	2017	81.5%	85.7%	75.6%
Brazil	Alvorada do Gurguéia	2000	59.8%	66.4%	53.3%
Brazil	Alvorada do Gurguéia	2017	60.7%	67.3%	54.3%
Brazil	Alvorada do Norte	2000	83.3%	88.1%	78.4%
Brazil	Alvorada do Norte	2017	83.8%	88.5%	79.1%
Brazil	Alvorada do Sul	2000	86.2%	88.9%	83.4%
Brazil	Alvorada do Sul	2017	86.7%	89.3%	83.9%
Brazil	Amajari	2000	84.1%	87.9%	79.1%
Brazil	Amajari	2017	84.6%	88.3%	79.6%
Brazil	Amambai	2000	82.6%	86.3%	78.3%
Brazil	Amambai	2017	83.2%	86.8%	78.9%
Brazil	Amapá	2000	43.2%	49.5%	37.5%
Brazil	Amapá	2017	44.2%	50.5%	38.4%
Brazil	Amapá do Maranho	2000	47.0%	53.6%	40.8%
Brazil	Amapá do Maranho	2017	48.0%	54.6%	41.7%
Brazil	Amapora	2000	83.7%	87.2%	80.0%
Brazil	Amapora	2017	84.3%	87.6%	80.6%
Brazil	Amaraji	2000	66.1%	69.2%	62.6%
Brazil	Amaraji	2017	66.9%	70.0%	63.5%
Brazil	Amaral Ferrador	2000	81.3%	85.6%	76.8%
Brazil	Amaral Ferrador	2017	82.0%	86.1%	77.5%
Brazil	Amaralina	2000	76.2%	81.7%	70.1%
Brazil	Amaralina	2017	76.8%	82.3%	70.9%
Brazil	Amarante	2000	58.9%	63.4%	54.1%
Brazil	Amarante	2017	59.8%	64.4%	55.1%
Brazil	Amarante do Maranhão	2000	65.0%	70.4%	59.5%
Brazil	Amarante do Maranhão	2017	66.1%	71.4%	60.7%
Brazil	Amargosa	2000	79.8%	83.5%	74.6%
Brazil	Amargosa	2017	80.4%	84.0%	75.3%
Brazil	Amaturá	2000	58.0%	65.2%	50.7%
Brazil	Amaturá	2017	59.0%	66.1%	51.7%
Brazil	Amélia Rodrigues	2000	76.9%	80.1%	73.2%
Brazil	Amélia Rodrigues	2017	77.6%	80.7%	74.0%
Brazil	América dourada	2000	77.3%	83.0%	71.9%
Brazil	América dourada	2017	78.0%	83.6%	72.7%

Country	Second Administrative Unit	Year	Mean	Upper	Lower
Brazil	Americana	2000	93.4%	94.6%	91.9%
Brazil	Americana	2017	93.6%	94.8%	92.2%
Brazil	Americano do Brazil	2000	77.6%	81.0%	73.6%
Brazil	Americano do Brazil	2017	80.1%	83.2%	76.3%
Brazil	Américo Brasiliense	2000	93.8%	95.2%	91.7%
Brazil	Américo Brasiliense	2017	94.0%	95.4%	92.0%
Brazil	Américo de Campos	2000	92.4%	93.8%	90.7%
Brazil	Américo de Campos	2017	92.7%	94.0%	91.1%
Brazil	Ametista do Sul	2000	78.1%	81.7%	73.6%
Brazil	Ametista do Sul	2017	78.8%	82.3%	74.3%
Brazil	Amontada	2000	66.9%	71.4%	62.1%
Brazil	Amontada	2017	67.8%	72.2%	63.0%
Brazil	Amorinópolis	2000	76.4%	81.6%	70.9%
Brazil	Amorinópolis	2017	77.1%	82.2%	71.7%
Brazil	Amparo	2000	67.2%	71.4%	62.4%
Brazil	Amparo	2000	92.2%	93.4%	90.8%
Brazil	Amparo	2017	68.0%	72.2%	63.3%
Brazil	Amparo	2017	92.5%	93.7%	91.2%
Brazil	Amparo de São Francisco	2000	67.9%	70.8%	64.8%
Brazil	Amparo de São Francisco	2017	68.7%	71.6%	65.6%
Brazil	Amparo do Serra	2000	81.0%	84.7%	76.7%
Brazil	Amparo do Serra	2017	81.6%	85.2%	77.4%
Brazil	Ampére	2000	83.2%	86.8%	79.2%
Brazil	Ampére	2017	83.8%	87.2%	79.9%
Brazil	Anadia	2000	61.9%	65.2%	59.1%
Brazil	Anadia	2017	62.9%	66.1%	60.1%
Brazil	Anagé	2000	78.3%	83.1%	72.6%
Brazil	Anagé	2017	79.0%	83.7%	73.5%
Brazil	Anahy	2000	83.3%	87.1%	78.9%
Brazil	Anahy	2017	83.9%	87.6%	79.6%
Brazil	Anajas	2000	35.4%	42.0%	29.9%
Brazil	Anajas	2017	36.3%	42.9%	30.7%
Brazil	Anajatuba	2000	61.5%	65.9%	56.8%
Brazil	Anajatuba	2017	63.3%	67.5%	58.6%
Brazil	Analandia	2000	92.7%	94.2%	90.7%
Brazil	Analandia	2017	92.9%	94.4%	91.1%
Brazil	Anamã	2000	60.1%	65.6%	54.7%
Brazil	Anamã	2017	61.0%	66.5%	55.7%
Brazil	Ananás	2000	71.4%	75.5%	66.4%
Brazil	Ananás	2017	72.2%	76.2%	67.3%
Brazil	Ananindeua	2000	37.8%	40.1%	35.6%
Brazil	Ananindeua	2017	38.8%	41.1%	36.5%
Brazil	Anápolis	2000	80.1%	82.3%	77.5%
Brazil	Anápolis	2017	80.8%	82.9%	78.2%
Brazil	Anapu	2000	39.1%	48.0%	30.3%
Brazil	Anapu	2017	40.1%	49.0%	31.1%
Brazil	Anapuros	2000	62.9%	67.6%	57.8%
Brazil	Anapuros	2017	63.7%	68.5%	58.7%
Brazil	Anastácio	2000	78.5%	82.5%	74.2%
Brazil	Anastácio	2017	79.2%	83.0%	74.9%

Country	Second Administrative Unit	Year	Mean	Upper	Lower
Brazil	Anaurilândia	2000	83.2%	86.9%	79.4%
Brazil	Anaurilândia	2017	83.7%	87.3%	80.0%
Brazil	Anchieta	2000	76.2%	79.2%	72.4%
Brazil	Anchieta	2000	80.1%	84.5%	75.7%
Brazil	Anchieta	2017	80.7%	85.0%	76.4%
Brazil	Anchieta	2017	76.9%	79.8%	73.1%
Brazil	Andarai	2000	76.1%	82.1%	69.9%
Brazil	Andarai	2017	76.8%	82.6%	70.7%
Brazil	Andira	2000	90.2%	92.5%	87.7%
Brazil	Andira	2017	90.5%	92.7%	88.1%
Brazil	Andirá	2000	88.7%	91.2%	85.8%
Brazil	Andirá	2017	89.1%	91.5%	86.3%
Brazil	Andorinha	2000	74.5%	79.8%	68.2%
Brazil	Andorinha	2017	75.2%	80.4%	69.0%
Brazil	Andradas	2000	90.0%	92.2%	87.2%
Brazil	Andradas	2017	90.3%	92.5%	87.6%
Brazil	Andradina	2000	87.5%	90.1%	84.3%
Brazil	Andradina	2017	87.9%	90.5%	84.8%
Brazil	André da Rocha	2000	80.3%	83.9%	76.5%
Brazil	André da Rocha	2017	81.0%	84.4%	77.2%
Brazil	Andrelândia	2000	83.5%	86.4%	79.8%
Brazil	Andrelândia	2017	84.0%	86.8%	80.4%
Brazil	Angatuba	2000	93.1%	95.0%	90.8%
Brazil	Angatuba	2017	93.4%	95.1%	91.1%
Brazil	Angelândia	2000	83.7%	88.1%	77.5%
Brazil	Angelândia	2017	84.2%	88.5%	78.2%
Brazil	Angélica	2000	80.2%	84.1%	75.5%
Brazil	Angélica	2017	80.8%	84.6%	76.2%
Brazil	Angelim	2000	66.4%	69.4%	62.9%
Brazil	Angelim	2017	67.3%	70.2%	63.8%
Brazil	Angelina	2000	75.8%	79.8%	70.8%
Brazil	Angelina	2017	76.5%	80.4%	71.6%
Brazil	Angical	2000	74.7%	81.6%	67.2%
Brazil	Angical	2017	75.4%	82.2%	68.1%
Brazil	Angical do Piauí	2000	60.4%	65.2%	55.3%
Brazil	Angical do Piauí	2017	61.3%	66.0%	56.2%
Brazil	Angico	2000	73.8%	77.9%	69.6%
Brazil	Angico	2017	74.5%	78.6%	70.4%
Brazil	Angicos	2000	83.9%	86.6%	80.6%
Brazil	Angicos	2017	84.4%	87.1%	81.2%
Brazil	Angra dos Reis	2000	81.7%	84.7%	78.1%
Brazil	Angra dos Reis	2017	82.2%	85.2%	78.8%
Brazil	Anguera	2000	76.9%	80.8%	72.7%
Brazil	Anguera	2017	77.6%	81.4%	73.4%
Brazil	Ângulo	2000	84.8%	87.5%	81.6%
Brazil	Ângulo	2017	85.3%	87.9%	82.1%
Brazil	Anhanguera	2000	77.9%	82.6%	72.7%
Brazil	Anhanguera	2017	78.6%	83.2%	73.5%
Brazil	Anhembi	2000	92.9%	94.4%	90.8%
Brazil	Anhembi	2017	93.1%	94.6%	91.1%
Brazil	Anhumas	2000	88.7%	91.5%	85.9%
Brazil	Anhumas	2017	89.1%	91.8%	86.3%
Brazil	Anicuns	2000	77.2%	80.8%	73.3%
Brazil	Anicuns	2017	78.0%	81.5%	74.1%
Brazil	Anísio de Abreu	2000	64.4%	70.2%	57.0%

Country	Second Administrative Unit	Year	Mean	Upper	Lower
Brazil	Anísio de Abreu	2017	65.3%	71.0%	58.0%
Brazil	Anita Garibaldi	2000	76.7%	80.9%	72.2%
Brazil	Anita Garibaldi	2017	77.3%	81.5%	72.9%
Brazil	Anitápolis	2000	76.9%	80.7%	71.7%
Brazil	Anitápolis	2017	77.7%	81.4%	72.6%
Brazil	Anori	2000	59.2%	65.4%	53.3%
Brazil	Anori	2017	60.2%	66.2%	54.2%
Brazil	Anta Gorda	2000	82.0%	85.3%	78.2%
Brazil	Anta Gorda	2017	82.6%	85.8%	78.9%
Brazil	Antas	2000	75.3%	79.7%	70.3%
Brazil	Antas	2017	76.0%	80.4%	71.1%
Brazil	Antonina	2000	78.7%	82.2%	74.4%
Brazil	Antonina	2017	79.3%	82.8%	75.2%
Brazil	Antonina do Norte	2000	69.8%	74.0%	65.5%
Brazil	Antonina do Norte	2017	70.6%	74.7%	66.4%
Brazil	Antônio Almeida	2000	61.7%	68.3%	53.9%
Brazil	Antônio Almeida	2017	62.5%	69.1%	54.9%
Brazil	Antônio Cardoso	2000	76.9%	80.5%	73.4%
Brazil	Antônio Cardoso	2017	77.6%	81.1%	74.1%
Brazil	Antônio Carlos	2000	71.0%	75.8%	65.1%
Brazil	Antônio Carlos	2000	83.2%	86.4%	79.5%
Brazil	Antônio Carlos	2017	71.9%	76.6%	66.0%
Brazil	Antônio Carlos	2017	83.8%	86.9%	80.1%
Brazil	Antônio Dias	2000	80.9%	84.6%	76.9%
Brazil	Antônio Dias	2017	81.5%	85.2%	77.6%
Brazil	Antônio Gonçalves	2000	76.2%	81.5%	69.8%
Brazil	Antônio Gonçalves	2017	76.9%	82.0%	70.6%
Brazil	Antônio João	2000	82.4%	86.5%	77.9%
Brazil	Antônio João	2017	83.0%	87.0%	78.6%
Brazil	Antônio Martins	2000	78.8%	81.9%	75.7%
Brazil	Antônio Martins	2017	78.4%	81.5%	75.2%
Brazil	Antonio Olinto	2000	79.4%	82.9%	75.1%
Brazil	Antonio Olinto	2017	80.0%	83.4%	75.9%
Brazil	Antônio Prado	2000	80.8%	84.1%	77.2%
Brazil	Antônio Prado	2017	81.4%	84.7%	77.8%
Brazil	Antonio Prado de Minas	2000	80.3%	83.8%	76.4%
Brazil	Antonio Prado de Minas	2017	80.9%	84.3%	77.1%

Country	Second Administrative Unit	Year	Mean	Upper	Lower
Brazil	Aparecida	2000	90.6%	92.8%	88.3%
Brazil	Aparecida	2000	70.5%	74.1%	67.0%
Brazil	Aparecida	2017	90.9%	93.0%	88.7%
Brazil	Aparecida	2017	71.3%	74.8%	67.8%
Brazil	Aparecida de Goiânia	2000	79.0%	80.9%	76.9%
Brazil	Aparecida de Goiânia	2017	79.7%	81.6%	77.6%
Brazil	Aparecida do Rio doce	2000	78.3%	83.2%	72.8%
Brazil	Aparecida do Rio doce	2017	78.9%	83.8%	73.5%
Brazil	Aparecida do Rio Negro	2000	76.4%	80.1%	72.4%
Brazil	Aparecida do Rio Negro	2017	77.1%	80.7%	73.2%
Brazil	Aparecida do Taboado	2000	85.8%	89.7%	81.7%
Brazil	Aparecida do Taboado	2017	86.3%	90.1%	82.2%
Brazil	Aparecida doeste	2000	87.8%	91.2%	84.1%
Brazil	Aparecida doeste	2017	88.2%	91.5%	84.6%
Brazil	Aperibé	2000	77.8%	81.6%	73.8%
Brazil	Aperibé	2017	78.6%	82.2%	74.7%
Brazil	Apiacá	2000	76.5%	80.4%	72.0%
Brazil	Apiacá	2017	77.1%	81.0%	72.8%
Brazil	Apiacás	2000	66.5%	74.9%	57.4%
Brazil	Apiacás	2017	67.5%	75.8%	58.4%
Brazil	Apiaí	2000	90.7%	93.0%	88.0%
Brazil	Apiaí	2017	91.0%	93.3%	88.3%
Brazil	Apicum-Açu	2000	57.9%	66.7%	48.7%
Brazil	Apicum-Açu	2017	58.9%	67.5%	49.8%
Brazil	Apiúna	2000	72.7%	76.3%	69.6%
Brazil	Apiúna	2017	73.5%	77.0%	70.4%
Brazil	Apodi	2000	78.1%	81.1%	74.3%
Brazil	Apodi	2017	78.7%	81.7%	75.1%
Brazil	Aporá	2000	78.4%	82.6%	74.6%
Brazil	Aporá	2017	79.1%	83.2%	75.3%
Brazil	Aporé	2000	79.5%	84.5%	73.9%
Brazil	Aporé	2017	80.1%	85.0%	74.6%
Brazil	Apuarema	2000	77.8%	82.2%	72.9%
Brazil	Apuarema	2017	78.4%	82.8%	73.6%
Brazil	Apucarana	2000	86.0%	88.1%	83.2%
Brazil	Apucarana	2017	86.4%	88.5%	83.7%
Brazil	Apuí	2000	63.4%	73.6%	52.2%
Brazil	Apuí	2017	64.5%	74.5%	53.4%
Brazil	Apuiarés	2000	70.3%	73.8%	66.4%
Brazil	Apuiarés	2017	71.2%	74.6%	67.3%
Brazil	Aquidabã	2000	72.6%	75.3%	69.7%
Brazil	Aquidabã	2017	73.4%	76.0%	70.5%
Brazil	Aquidauana	2000	78.9%	82.6%	74.4%
Brazil	Aquidauana	2017	79.5%	83.2%	75.1%
Brazil	Aquiraz	2000	68.0%	70.4%	65.4%
Brazil	Aquiraz	2017	69.1%	71.4%	66.6%
Brazil	Arabutã	2000	76.3%	79.8%	71.4%
Brazil	Arabutã	2017	77.1%	80.5%	72.2%
Brazil	Aracagi	2000	69.5%	72.2%	66.3%
Brazil	Aracagi	2017	70.4%	73.1%	67.2%
Brazil	Aracai	2000	81.2%	84.9%	76.0%
Brazil	Aracai	2017	81.8%	85.4%	76.7%

Country	Second Administrative Unit	Year	Mean	Upper	Lower
Brazil	Aracaju	2000	76.4%	78.0%	74.4%
Brazil	Aracaju	2017	77.1%	78.7%	75.2%
Brazil	Araçariguama	2000	94.1%	95.1%	93.1%
Brazil	Araçariguama	2017	94.4%	95.3%	93.4%
Brazil	Araças	2000	75.4%	79.5%	70.9%
Brazil	Araças	2017	76.1%	80.1%	71.7%
Brazil	Aracati	2000	73.9%	77.8%	69.8%
Brazil	Aracati	2017	74.6%	78.4%	70.6%
Brazil	Aracatu	2000	77.7%	82.4%	72.4%
Brazil	Aracatu	2017	78.3%	82.9%	73.1%
Brazil	Araçatuba	2000	92.2%	94.2%	89.3%
Brazil	Araçatuba	2017	92.4%	94.4%	89.7%
Brazil	Araci	2000	77.7%	81.6%	73.2%
Brazil	Araci	2017	78.4%	82.2%	73.9%
Brazil	Aracitaba	2000	79.7%	83.1%	75.4%
Brazil	Aracitaba	2017	80.3%	83.7%	76.1%
Brazil	Araçoiaba	2000	69.8%	73.4%	66.0%
Brazil	Araçoiaba	2000	66.9%	69.9%	64.5%
Brazil	Araçoiaba	2017	70.6%	74.2%	66.9%
Brazil	Araçoiaba	2017	67.7%	70.7%	65.4%
Brazil	Araçoiaba da Serra	2000	94.0%	95.4%	92.6%
Brazil	Araçoiaba da Serra	2017	94.2%	95.5%	92.8%
Brazil	Aracruz	2000	76.0%	78.8%	73.0%
Brazil	Aracruz	2017	76.8%	79.6%	73.9%
Brazil	Araçu	2000	77.0%	80.4%	73.8%
Brazil	Araçu	2017	77.6%	81.0%	74.5%
Brazil	Araçuaí	2000	82.2%	87.1%	75.5%
Brazil	Araçuaí	2017	82.8%	87.5%	76.3%
Brazil	Aragarças	2000	72.7%	78.7%	66.5%
Brazil	Aragarças	2017	73.4%	79.3%	67.3%
Brazil	Aragoiânia	2000	76.7%	79.5%	74.0%
Brazil	Aragoiânia	2017	77.6%	80.3%	74.9%
Brazil	Aragominas	2000	68.7%	73.3%	64.3%
Brazil	Aragominas	2017	69.5%	74.0%	65.1%
Brazil	Araguacema	2000	60.0%	66.3%	53.8%
Brazil	Araguacema	2017	60.8%	67.1%	54.7%
Brazil	Araguaçu	2000	74.6%	80.0%	68.0%
Brazil	Araguaçu	2017	75.3%	80.6%	68.8%
Brazil	Araguaiana	2000	71.3%	76.8%	64.5%
Brazil	Araguaiana	2017	72.1%	77.5%	65.4%
Brazil	Araguaína	2000	74.5%	77.8%	70.9%
Brazil	Araguaína	2017	76.2%	79.3%	72.8%
Brazil	Araguainha	2000	72.8%	79.5%	65.6%
Brazil	Araguainha	2017	73.5%	80.1%	66.5%
Brazil	Araguanã	2000	66.0%	70.7%	61.3%
Brazil	Araguanã	2000	58.4%	64.6%	52.3%
Brazil	Araguanã	2017	66.9%	71.5%	62.2%
Brazil	Araguanã	2017	59.4%	65.5%	53.2%
Brazil	Araguapaz	2000	74.2%	79.6%	67.2%
Brazil	Araguapaz	2017	74.9%	80.3%	68.0%
Brazil	Araguari	2000	81.8%	85.8%	77.4%
Brazil	Araguari	2017	82.4%	86.2%	78.1%
Brazil	Araguatins	2000	63.8%	68.1%	59.6%
Brazil	Araguatins	2017	64.7%	68.9%	60.4%
Brazil	Araioses	2000	61.2%	65.8%	56.7%
Brazil	Araioses	2017	62.1%	66.7%	57.6%
Brazil	Aral Moreira	2000	82.0%	86.0%	77.6%
Brazil	Aral Moreira	2017	82.6%	86.5%	78.3%
Brazil	Aramari	2000	77.0%	80.6%	73.6%
Brazil	Aramari	2017	77.7%	81.2%	74.4%

Country	Second Administrative Unit	Year	Mean	Upper	Lower
Brazil	Arambaré	2000	82.6%	86.7%	78.6%
Brazil	Arambaré	2017	83.2%	87.2%	79.2%
Brazil	Arame	2000	62.4%	68.0%	56.6%
Brazil	Arame	2017	63.4%	68.9%	57.6%
Brazil	Aramina	2000	87.2%	90.4%	83.0%
Brazil	Aramina	2017	87.7%	90.8%	83.5%
Brazil	Arandu	2000	92.7%	94.7%	90.0%
Brazil	Arandu	2017	92.9%	94.9%	90.3%
Brazil	Arantina	2000	84.1%	87.3%	80.8%
Brazil	Arantina	2017	84.6%	87.7%	81.4%
Brazil	Arapeí	2000	84.7%	87.1%	81.9%
Brazil	Arapeí	2017	85.2%	87.5%	82.5%
Brazil	Arapiraca	2000	63.9%	66.7%	61.5%
Brazil	Arapiraca	2017	65.1%	67.8%	62.7%
Brazil	Arapoema	2000	63.7%	68.2%	57.4%
Brazil	Arapoema	2017	64.6%	69.0%	58.2%
Brazil	Araponga	2000	83.7%	86.8%	80.1%
Brazil	Araponga	2017	84.4%	87.3%	80.9%
Brazil	Arapongas	2000	86.1%	88.1%	83.2%
Brazil	Arapongas	2017	86.5%	88.6%	83.7%
Brazil	Araporã	2000	83.7%	87.3%	79.4%
Brazil	Araporã	2017	84.3%	87.7%	80.1%
Brazil	Arapoti	2000	87.5%	90.0%	84.4%
Brazil	Arapoti	2017	88.8%	91.2%	86.0%
Brazil	Arapu	2000	83.6%	87.1%	79.9%
Brazil	Arapu	2017	84.2%	87.6%	80.6%
Brazil	Arapuá	2000	81.3%	86.8%	74.0%
Brazil	Arapuá	2017	81.8%	87.2%	74.8%
Brazil	Araputanga	2000	67.6%	73.2%	60.6%
Brazil	Araputanga	2017	70.3%	75.8%	63.7%
Brazil	Araquari	2000	74.5%	78.0%	70.3%
Brazil	Araquari	2017	75.3%	78.7%	71.2%
Brazil	Arara	2000	71.2%	74.0%	68.0%
Brazil	Arara	2017	72.1%	74.9%	69.0%
Brazil	Araranguá	2000	76.5%	80.9%	72.0%
Brazil	Araranguá	2017	77.1%	81.4%	72.8%
Brazil	Araraquara	2000	93.8%	95.2%	91.9%
Brazil	Araraquara	2017	94.0%	95.4%	92.2%
Brazil	Araras	2000	92.6%	94.1%	91.0%
Brazil	Araras	2017	92.9%	94.4%	91.3%
Brazil	Ararendá	2000	68.1%	73.4%	63.3%
Brazil	Ararendá	2017	68.9%	74.1%	64.1%
Brazil	Arari	2000	61.6%	65.9%	56.7%
Brazil	Arari	2017	62.5%	66.8%	57.6%
Brazil	Araricá	2000	80.0%	82.6%	77.6%
Brazil	Araricá	2017	80.6%	83.2%	78.3%
Brazil	Araripe	2000	68.1%	72.0%	63.9%
Brazil	Araripe	2017	68.9%	72.8%	64.8%
Brazil	Araripina	2000	65.3%	69.7%	60.8%
Brazil	Araripina	2017	66.2%	70.5%	61.8%
Brazil	Araruama	2000	77.7%	80.7%	74.2%
Brazil	Araruama	2017	78.4%	81.3%	74.9%
Brazil	Araruna	2000	84.4%	87.3%	81.1%
Brazil	Araruna	2000	78.8%	81.4%	75.9%
Brazil	Araruna	2017	84.9%	87.8%	81.7%
Brazil	Araruna	2017	79.5%	82.0%	76.6%
Brazil	Arataca	2000	77.6%	82.0%	72.2%
Brazil	Arataca	2017	78.3%	82.5%	73.0%
Brazil	Aratiba	2000	77.3%	80.6%	73.7%
Brazil	Aratiba	2017	77.9%	81.2%	74.4%
Brazil	Aratuba	2000	71.7%	75.8%	67.8%
Brazil	Aratuba	2017	72.4%	76.4%	68.6%

Country	Second Administrative Unit	Year	Mean	Upper	Lower
Brazil	Aratuipe	2000	75.8%	79.6%	71.5%
Brazil	Aratuipe	2017	76.5%	80.2%	72.3%
Brazil	Aratuípe	2000	75.0%	78.8%	71.1%
Brazil	Aratuípe	2017	75.7%	79.5%	71.9%
Brazil	Arauá	2000	77.5%	80.3%	74.3%
Brazil	Arauá	2017	78.1%	80.9%	75.1%
Brazil	Araucária	2000	84.7%	86.6%	82.5%
Brazil	Araucária	2017	85.4%	87.2%	83.2%
Brazil	Araujos	2000	80.9%	84.9%	76.3%
Brazil	Araujos	2017	81.5%	85.4%	77.0%
Brazil	Araújos	2000	82.8%	85.2%	80.4%
Brazil	Araújos	2017	83.4%	85.7%	81.0%
Brazil	Araxá	2000	85.2%	89.1%	80.1%
Brazil	Araxá	2017	85.7%	89.5%	80.7%
Brazil	Arceburgo	2000	88.3%	91.0%	84.8%
Brazil	Arceburgo	2017	88.7%	91.3%	85.3%
Brazil	Arco-íris	2000	91.7%	94.1%	89.0%
Brazil	Arco-íris	2017	92.0%	94.3%	89.4%
Brazil	Arcos	2000	82.0%	86.1%	77.0%
Brazil	Arcos	2017	82.5%	86.6%	77.7%
Brazil	Arcoverde	2000	66.4%	70.2%	62.2%
Brazil	Arcoverde	2017	67.3%	71.0%	63.1%
Brazil	Areado	2000	85.1%	88.7%	81.5%
Brazil	Areado	2017	85.6%	89.1%	82.0%
Brazil	Areal	2000	78.6%	81.4%	75.0%
Brazil	Areal	2017	79.0%	81.8%	75.5%
Brazil	Arealva	2000	93.3%	95.0%	91.0%
Brazil	Arealva	2017	93.5%	95.2%	91.3%
Brazil	Areia	2000	66.4%	69.6%	63.3%
Brazil	Areia	2017	67.4%	70.5%	64.3%
Brazil	Areia Branca	2000	80.5%	83.8%	77.3%
Brazil	Areia Branca	2000	78.0%	79.9%	75.8%
Brazil	Areia Branca	2017	78.7%	80.6%	76.5%
Brazil	Areia Branca	2017	81.1%	84.3%	78.0%
Brazil	Areia de Baraúnas	2000	71.3%	75.9%	66.8%
Brazil	Areia de Baraúnas	2017	72.2%	76.6%	67.6%
Brazil	Areial	2000	70.3%	73.7%	67.5%
Brazil	Areial	2017	71.1%	74.4%	68.4%
Brazil	Areias	2000	85.1%	87.7%	82.0%
Brazil	Areias	2017	85.5%	88.1%	82.5%
Brazil	Areiópolis	2000	94.2%	95.8%	92.4%
Brazil	Areiópolis	2017	94.4%	95.9%	92.6%
Brazil	Arenápolis	2000	67.1%	72.8%	59.9%
Brazil	Arenápolis	2017	68.0%	73.6%	60.8%
Brazil	Arenópolis	2000	75.0%	80.8%	68.5%
Brazil	Arenópolis	2017	75.6%	81.3%	69.3%
Brazil	Arês	2000	82.0%	84.0%	79.7%
Brazil	Arês	2017	82.6%	84.6%	80.4%
Brazil	Argirita	2000	80.3%	83.6%	76.5%
Brazil	Argirita	2017	80.9%	84.1%	77.2%
Brazil	Aricanduva	2000	83.5%	87.7%	76.9%
Brazil	Aricanduva	2017	84.1%	88.1%	77.6%
Brazil	Arinos	2000	84.5%	89.7%	78.4%
Brazil	Arinos	2017	85.0%	90.1%	79.0%
Brazil	Aripuanã	2000	59.4%	67.0%	52.4%
Brazil	Aripuanã	2017	60.6%	68.1%	53.6%
Brazil	Ariquemes	2000	27.8%	31.7%	24.3%
Brazil	Ariquemes	2017	28.8%	32.8%	25.2%
Brazil	Ariranha	2000	93.2%	94.8%	91.3%
Brazil	Ariranha	2017	93.5%	95.0%	91.6%

Country	Second Administrative Unit	Year	Mean	Upper	Lower
Brazil	Ariranha do Ivaí	2000	83.3%	86.9%	79.2%
Brazil	Ariranha do Ivaí	2017	83.8%	87.3%	79.9%
Brazil	Armação dos Búzios	2000	75.8%	79.5%	70.5%
Brazil	Armação dos Búzios	2017	77.7%	81.3%	72.8%
Brazil	Armazém	2000	71.8%	76.2%	66.2%
Brazil	Armazém	2017	73.8%	78.0%	68.5%
Brazil	Arneiroz	2000	70.3%	75.3%	65.6%
Brazil	Arneiroz	2017	71.1%	76.0%	66.5%
Brazil	Aroazes	2000	61.2%	66.3%	55.5%
Brazil	Aroazes	2017	62.1%	67.2%	56.4%
Brazil	Aroeiras	2000	63.9%	67.5%	60.7%
Brazil	Aroeiras	2017	64.8%	68.3%	61.6%
Brazil	Arraial	2000	59.2%	64.2%	54.0%
Brazil	Arraial	2017	60.1%	65.0%	55.0%
Brazil	Arraial do Cabo	2000	77.9%	81.8%	73.0%
Brazil	Arraial do Cabo	2017	78.5%	82.3%	73.8%
Brazil	Arraias	2000	76.9%	81.7%	71.0%
Brazil	Arraias	2017	77.5%	82.2%	71.8%
Brazil	Arroio do Meio	2000	79.6%	83.5%	75.8%
Brazil	Arroio do Meio	2017	80.3%	84.0%	76.6%
Brazil	Arroio do Sal	2000	75.3%	80.2%	69.3%
Brazil	Arroio do Sal	2017	77.1%	81.8%	71.4%
Brazil	Arroio do Tigre	2000	83.2%	86.5%	79.3%
Brazil	Arroio do Tigre	2017	83.8%	87.0%	80.0%
Brazil	Arroio dos Ratos	2000	81.9%	84.8%	78.3%
Brazil	Arroio dos Ratos	2017	82.5%	85.3%	79.0%
Brazil	Arroio Grande	2000	79.8%	85.5%	73.6%
Brazil	Arroio Grande	2017	80.4%	86.0%	74.3%
Brazil	Arroio Trinta	2000	78.1%	82.0%	74.4%
Brazil	Arroio Trinta	2017	78.7%	82.5%	75.1%
Brazil	Artur Nogueira	2000	92.4%	93.8%	90.7%
Brazil	Artur Nogueira	2017	92.6%	94.1%	91.0%
Brazil	Aruanã	2000	72.2%	78.1%	65.8%
Brazil	Aruanã	2017	72.9%	78.7%	66.4%
Brazil	Aruja	2000	94.3%	95.1%	93.4%
Brazil	Aruja	2017	94.6%	95.3%	93.7%
Brazil	Arvoredo	2000	76.6%	81.0%	72.1%
Brazil	Arvoredo	2017	77.3%	81.6%	72.9%
Brazil	Arvorezinha	2000	83.3%	86.8%	79.8%
Brazil	Arvorezinha	2017	83.8%	87.2%	80.5%
Brazil	Ascurra	2000	72.1%	75.8%	68.7%
Brazil	Ascurra	2017	73.0%	76.6%	69.6%
Brazil	Aspásia	2000	87.5%	91.4%	83.9%
Brazil	Aspásia	2017	87.9%	91.7%	84.4%
Brazil	Assaí	2000	86.3%	88.7%	83.5%
Brazil	Assaí	2017	86.7%	89.0%	84.0%
Brazil	Assaré	2000	69.6%	73.3%	65.1%
Brazil	Assaré	2017	70.4%	74.0%	66.0%

Country	Second Administrative Unit	Year	Mean	Upper	Lower
Brazil	Assis	2000	91.1%	93.2%	88.8%
Brazil	Assis	2017	91.4%	93.5%	89.2%
Brazil	Assis Brazil	2000	45.5%	52.4%	38.9%
Brazil	Assis Brazil	2017	46.5%	53.5%	39.8%
Brazil	Assis Chateaubri	2000	84.4%	87.8%	80.5%
Brazil	Assis Chateaubri	2017	84.9%	88.2%	81.1%
Brazil	Assunção	2000	72.2%	76.2%	68.0%
Brazil	Assunção	2017	73.0%	76.9%	68.8%
Brazil	Assunção do Piauí	2000	67.2%	72.6%	61.7%
Brazil	Assunção do Piauí	2017	68.0%	73.4%	62.6%
Brazil	Astolfo Dutra	2000	78.8%	82.4%	74.7%
Brazil	Astolfo Dutra	2017	79.5%	83.0%	75.5%
Brazil	Astorga	2000	85.9%	88.5%	83.0%
Brazil	Astorga	2017	86.4%	88.9%	83.6%
Brazil	Atalaia do Norte	2000	57.6%	63.5%	50.1%
Brazil	Atalaia do Norte	2017	58.6%	64.4%	51.1%
Brazil	Atalanta	2000	74.6%	78.8%	69.9%
Brazil	Atalanta	2017	75.3%	79.4%	70.7%
Brazil	Ataleia	2000	80.8%	85.1%	75.6%
Brazil	Ataleia	2017	81.4%	85.6%	76.3%
Brazil	Ataléia	2000	61.8%	64.5%	59.1%
Brazil	Ataléia	2000	84.3%	87.0%	81.2%
Brazil	Ataléia	2017	84.8%	87.5%	81.8%
Brazil	Ataléia	2017	62.8%	65.4%	60.0%
Brazil	Atibaia	2000	93.3%	94.5%	92.1%
Brazil	Atibaia	2017	93.6%	94.7%	92.4%
Brazil	Atilio Vivacqua	2000	77.1%	80.3%	73.5%
Brazil	Atilio Vivacqua	2017	77.9%	81.0%	74.3%
Brazil	Augustinópolis	2000	68.3%	72.3%	64.3%
Brazil	Augustinópolis	2017	69.2%	73.1%	65.2%
Brazil	Augusto Corrêa	2000	41.8%	47.4%	36.7%
Brazil	Augusto Corrêa	2017	42.7%	48.4%	37.6%
Brazil	Augusto de Lima	2000	81.5%	86.5%	75.0%
Brazil	Augusto de Lima	2017	82.1%	87.0%	75.8%
Brazil	Augusto Pestana	2000	82.9%	86.6%	78.6%
Brazil	Augusto Pestana	2017	83.5%	87.0%	79.2%
Brazil	Augusto Severo	2000	79.9%	83.0%	76.4%
Brazil	Augusto Severo	2017	80.5%	83.5%	77.1%
Brazil	Aurelino Leal	2000	75.0%	80.0%	70.3%
Brazil	Aurelino Leal	2017	75.7%	80.6%	71.1%
Brazil	Auriflama	2000	89.9%	92.8%	86.9%
Brazil	Auriflama	2017	90.3%	93.0%	87.3%
Brazil	Aurilândia	2000	76.0%	80.3%	70.8%
Brazil	Aurilândia	2017	76.8%	80.9%	71.6%
Brazil	Aurora	2000	67.4%	71.0%	63.7%
Brazil	Aurora	2000	74.8%	78.7%	70.6%

Country	Second Administrative Unit	Year	Mean	Upper	Lower
Brazil	Aurora	2017	68.3%	71.9%	64.6%
Brazil	Aurora	2017	75.5%	79.3%	71.3%
Brazil	Aurora do Pará	2000	38.9%	43.7%	34.3%
Brazil	Aurora do Pará	2017	39.8%	44.7%	35.2%
Brazil	Aurora do Tocantins	2000	76.2%	81.4%	70.1%
Brazil	Aurora do Tocantins	2017	76.9%	82.0%	70.9%
Brazil	Autazes	2000	57.0%	61.6%	51.9%
Brazil	Autazes	2017	58.0%	62.6%	52.9%
Brazil	Avaí	2000	93.1%	94.9%	90.7%
Brazil	Avaí	2017	93.3%	95.1%	91.0%
Brazil	Avanhandava	2000	93.2%	95.1%	91.0%
Brazil	Avanhandava	2017	93.5%	95.3%	91.3%
Brazil	Avare	2000	93.6%	95.3%	91.3%
Brazil	Avare	2017	93.8%	95.5%	91.6%
Brazil	Aveiro	2000	43.1%	49.5%	36.5%
Brazil	Aveiro	2017	44.0%	50.5%	37.4%
Brazil	Avelino Lopes	2000	68.1%	75.2%	60.5%
Brazil	Avelino Lopes	2017	69.0%	75.9%	61.5%
Brazil	Avelinópolis	2000	76.4%	80.0%	73.0%
Brazil	Avelinópolis	2017	77.1%	80.6%	73.8%
Brazil	Axixá	2000	60.7%	64.8%	56.1%
Brazil	Axixá	2017	61.6%	65.7%	57.0%
Brazil	Axixá do Tocantins	2000	69.5%	73.3%	65.5%
Brazil	Axixá do Tocantins	2017	70.3%	74.0%	66.4%
Brazil	Babaçulândia	2000	73.2%	77.1%	68.9%
Brazil	Babaçulândia	2017	73.9%	77.7%	69.8%
Brazil	Bacabal	2000	59.8%	64.6%	55.1%
Brazil	Bacabal	2017	60.7%	65.5%	56.1%
Brazil	Bacabeira	2000	59.8%	64.4%	55.6%
Brazil	Bacabeira	2017	60.7%	65.2%	56.4%
Brazil	Bacuri	2000	59.4%	66.8%	51.6%
Brazil	Bacuri	2017	60.3%	67.6%	52.5%
Brazil	Bacurituba	2000	59.8%	64.7%	54.9%
Brazil	Bacurituba	2017	60.7%	65.6%	55.8%
Brazil	Bady Bassitt	2000	92.5%	94.2%	90.2%
Brazil	Bady Bassitt	2017	92.8%	94.4%	90.5%
Brazil	Baependi	2000	84.8%	87.9%	81.2%
Brazil	Baependi	2017	85.3%	88.3%	81.8%
Brazil	Bagé	2000	38.8%	44.7%	32.2%
Brazil	Bagé	2017	39.7%	45.7%	33.0%
Brazil	Baía da Traição	2000	69.5%	73.3%	65.9%
Brazil	Baía da Traição	2017	70.3%	74.0%	66.8%
Brazil	Baía Formosa	2000	75.1%	78.3%	71.9%
Brazil	Baía Formosa	2017	75.8%	79.0%	72.6%
Brazil	Baianópolis	2000	76.2%	82.7%	68.3%
Brazil	Baianópolis	2017	76.9%	83.2%	69.1%
Brazil	Baião	2000	37.9%	43.5%	32.7%
Brazil	Baião	2017	38.8%	44.5%	33.5%
Brazil	Baixa Grande	2000	78.9%	83.3%	74.2%
Brazil	Baixa Grande	2017	79.3%	83.7%	74.7%
Brazil	Baixa Grande do Ribeiro	2000	63.1%	70.4%	55.5%
Brazil	Baixa Grande do Ribeiro	2017	64.1%	71.3%	56.6%

Country	Second Administrative Unit	Year	Mean	Upper	Lower
Brazil	Baixio	2000	70.7%	74.5%	67.1%
Brazil	Baixio	2017	71.5%	75.2%	68.0%
Brazil	Baixo Guandu	2000	76.2%	79.8%	72.3%
Brazil	Baixo Guandu	2017	77.0%	80.4%	73.1%
Brazil	Baje	2000	83.5%	88.7%	78.3%
Brazil	Baje	2017	84.0%	89.0%	78.9%
Brazil	Balbinos	2000	93.1%	95.1%	90.6%
Brazil	Balbinos	2017	93.3%	95.3%	90.9%
Brazil	Baldim	2000	80.9%	84.5%	76.4%
Brazil	Baldim	2017	81.5%	85.1%	77.1%
Brazil	Baliza	2000	72.2%	78.6%	65.6%
Brazil	Baliza	2017	73.2%	79.4%	66.8%
Brazil	Balneário Arroio do Silva	2000	74.1%	77.9%	69.8%
Brazil	Balneário Arroio do Silva	2017	74.8%	78.6%	70.6%
Brazil	Balneário Barra do Sul	2000	73.4%	78.5%	67.9%
Brazil	Balneário Barra do Sul	2017	75.4%	80.2%	70.1%
Brazil	Balneário Camboriú	2000	72.6%	76.9%	67.7%
Brazil	Balneário Camboriú	2017	75.0%	79.1%	70.3%
Brazil	Balneário Gaivota	2000	74.1%	79.3%	68.6%
Brazil	Balneário Gaivota	2017	75.0%	80.0%	69.5%
Brazil	Balneário Pinhal	2000	80.9%	85.5%	76.0%
Brazil	Balneário Pinhal	2017	81.5%	86.0%	76.7%
Brazil	Balsa Nova	2000	82.1%	84.7%	79.2%
Brazil	Balsa Nova	2017	82.7%	85.2%	79.9%
Brazil	Bálsamo	2000	91.8%	93.7%	89.3%
Brazil	Bálsamo	2017	92.1%	94.0%	89.6%
Brazil	Balsas	2000	65.8%	73.1%	58.2%
Brazil	Balsas	2017	67.7%	74.7%	60.3%
Brazil	Bambuí	2000	83.9%	88.1%	78.9%
Brazil	Bambuí	2017	84.5%	88.5%	79.5%
Brazil	Banabuiú	2000	69.9%	74.6%	65.4%
Brazil	Banabuiú	2017	70.7%	75.3%	66.2%
Brazil	Bananal	2000	84.9%	87.2%	82.3%
Brazil	Bananal	2017	85.4%	87.6%	82.9%
Brazil	Bananeiras	2000	71.0%	73.8%	67.9%
Brazil	Bananeiras	2017	71.9%	74.6%	68.9%
Brazil	Bandeira	2000	82.7%	87.0%	77.3%
Brazil	Bandeira	2017	83.3%	87.4%	78.0%
Brazil	Bandeirante	2000	79.8%	84.2%	75.2%
Brazil	Bandeirante	2017	80.4%	84.7%	75.9%
Brazil	Bandeirantes	2000	79.3%	83.3%	74.1%
Brazil	Bandeirantes	2000	88.6%	90.9%	85.8%
Brazil	Bandeirantes	2017	79.9%	83.8%	74.9%
Brazil	Bandeirantes	2017	89.0%	91.3%	86.3%
Brazil	Bandeirantes do Tocantins	2000	72.7%	76.5%	68.3%
Brazil	Bandeirantes do Tocantins	2017	73.5%	77.2%	69.2%
Brazil	Bandiera do Sul	2000	88.5%	91.4%	85.0%
Brazil	Bandiera do Sul	2017	88.9%	91.7%	85.5%

Country	Second Administrative Unit	Year	Mean	Upper	Lower
Brazil	Bannach	2000	41.7%	49.4%	33.9%
Brazil	Bannach	2017	42.6%	50.3%	34.6%
Brazil	Banzaê	2000	76.5%	81.2%	71.2%
Brazil	Banzaê	2017	77.2%	81.8%	72.0%
Brazil	Barão de Antonina	2000	89.8%	92.4%	86.6%
Brazil	Barão de Antonina	2017	90.2%	92.6%	87.0%
Brazil	Barão de Cocais	2000	82.5%	86.0%	79.6%
Brazil	Barão de Cocais	2017	83.1%	86.5%	80.2%
Brazil	Barao de Cotegipe	2000	79.4%	82.5%	76.0%
Brazil	Barao de Cotegipe	2017	80.0%	83.1%	76.7%
Brazil	Barão de Grajaú	2000	61.7%	66.4%	57.0%
Brazil	Barão de Grajaú	2017	62.6%	67.3%	58.0%
Brazil	Barão de Melgaço	2000	69.3%	74.6%	63.2%
Brazil	Barão de Melgaço	2017	70.2%	75.3%	64.2%
Brazil	Barão de Monte Alto	2000	78.8%	82.3%	74.8%
Brazil	Barão de Monte Alto	2017	79.4%	82.8%	75.5%
Brazil	Barão do Triunfo	2000	83.0%	85.8%	80.3%
Brazil	Barão do Triunfo	2017	83.6%	86.3%	80.9%
Brazil	Baraúna	2000	79.0%	81.8%	75.8%
Brazil	Baraúna	2000	76.6%	80.0%	72.5%
Brazil	Baraúna	2017	80.1%	82.9%	77.1%
Brazil	Baraúna	2017	77.3%	80.6%	73.3%
Brazil	Barbacena	2000	84.8%	87.7%	81.3%
Brazil	Barbacena	2017	85.3%	88.2%	81.9%
Brazil	Barbalha	2000	69.3%	72.7%	65.6%
Brazil	Barbalha	2017	70.1%	73.4%	66.4%
Brazil	Barbosa	2000	92.4%	94.4%	90.1%
Brazil	Barbosa	2017	92.7%	94.6%	90.4%
Brazil	Barbosa Ferraz	2000	82.5%	85.7%	78.8%
Brazil	Barbosa Ferraz	2017	83.1%	86.2%	79.4%
Brazil	Barcarena	2000	36.2%	39.8%	33.2%
Brazil	Barcarena	2017	37.1%	40.8%	34.1%
Brazil	Barcelona	2000	83.1%	85.8%	80.5%
Brazil	Barcelona	2017	83.6%	86.3%	81.1%
Brazil	Barcelos	2000	71.4%	77.4%	62.9%
Brazil	Barcelos	2017	73.0%	78.8%	64.9%
Brazil	Bariri	2000	93.4%	95.0%	91.2%
Brazil	Bariri	2017	93.6%	95.2%	91.5%
Brazil	Baro	2000	83.8%	86.2%	81.3%
Brazil	Baro	2017	84.2%	86.6%	81.9%
Brazil	Barra	2000	72.3%	78.8%	63.3%
Brazil	Barra	2017	73.1%	79.4%	64.2%
Brazil	Barra Bonita	2000	94.0%	95.6%	92.2%
Brazil	Barra Bonita	2000	78.5%	82.6%	74.1%
Brazil	Barra Bonita	2017	94.2%	95.7%	92.5%
Brazil	Barra Bonita	2017	79.2%	83.2%	74.8%

Country	Second Administrative Unit	Year	Mean	Upper	Lower
Brazil	Barra d'Alcântara	2000	61.1%	66.0%	56.1%
Brazil	Barra d'Alcântara	2017	62.0%	66.8%	57.0%
Brazil	Barra da Choça	2000	80.8%	85.1%	76.1%
Brazil	Barra da Choça	2017	81.4%	85.6%	76.8%
Brazil	Barra da Estiva	2000	78.5%	83.6%	72.6%
Brazil	Barra da Estiva	2017	79.1%	84.0%	73.3%
Brazil	Barra de Guabira	2000	68.5%	72.1%	65.0%
Brazil	Barra de Guabira	2017	69.3%	72.9%	65.9%
Brazil	Barra de Santa Rosa	2000	77.2%	80.4%	73.7%
Brazil	Barra de Santa Rosa	2017	77.9%	81.0%	74.5%
Brazil	Barra de Santana	2000	65.9%	69.2%	62.7%
Brazil	Barra de Santana	2017	66.8%	70.1%	63.6%
Brazil	Barra de Santo Antônio	2000	59.6%	63.1%	56.0%
Brazil	Barra de Santo Antônio	2017	60.6%	64.1%	57.1%
Brazil	Barra de São Francisco	2000	80.6%	83.6%	76.9%
Brazil	Barra de São Francisco	2017	81.2%	84.1%	77.6%
Brazil	Barra de São Miguel	2000	57.4%	60.8%	53.7%
Brazil	Barra de São Miguel	2000	67.5%	71.6%	63.0%
Brazil	Barra de São Miguel	2017	68.3%	72.4%	63.9%
Brazil	Barra de São Miguel	2017	60.3%	63.6%	56.7%
Brazil	Barra do Chapéu	2000	89.4%	92.0%	86.2%
Brazil	Barra do Chapéu	2017	89.7%	92.3%	86.6%
Brazil	Barra do Corda	2000	61.7%	67.7%	56.1%
Brazil	Barra do Corda	2017	62.6%	68.6%	57.1%
Brazil	Barra do Garças	2000	72.3%	78.0%	66.4%
Brazil	Barra do Garças	2017	73.1%	78.7%	67.3%
Brazil	Barra do Guarita	2000	78.3%	82.7%	73.1%
Brazil	Barra do Guarita	2017	78.9%	83.2%	73.9%
Brazil	Barra do Jacaré	2000	89.9%	92.2%	87.1%
Brazil	Barra do Jacaré	2017	90.2%	92.5%	87.5%
Brazil	Barra do Mendes	2000	77.5%	83.0%	71.6%

Country	Second Administrative Unit	Year	Mean	Upper	Lower
Brazil	Barra do Mendes	2017	78.3%	83.6%	72.4%
Brazil	Barra do Ouro	2000	73.1%	78.1%	68.1%
Brazil	Barra do Ouro	2017	73.8%	78.8%	68.9%
Brazil	Barra do Piraí	2000	78.4%	80.9%	75.4%
Brazil	Barra do Piraí	2017	79.0%	81.5%	76.1%
Brazil	Barra do Quaraí	2000	80.3%	89.2%	68.9%
Brazil	Barra do Quaraí	2017	80.9%	89.6%	69.8%
Brazil	Barra do Ribeiro	2000	82.3%	85.3%	79.4%
Brazil	Barra do Ribeiro	2017	82.8%	85.8%	80.1%
Brazil	Barra do Rio Azul	2000	77.4%	80.8%	73.9%
Brazil	Barra do Rio Azul	2017	78.1%	81.4%	74.7%
Brazil	Barra do Rocha	2000	76.6%	81.1%	72.0%
Brazil	Barra do Rocha	2017	77.0%	81.4%	72.4%
Brazil	Barra do Turvo	2000	88.4%	91.3%	85.2%
Brazil	Barra do Turvo	2017	88.9%	91.6%	85.7%
Brazil	Barra dos Bugre	2000	66.3%	71.6%	60.2%
Brazil	Barra dos Bugre	2017	67.1%	72.4%	61.1%
Brazil	Barra dos Coqueiros	2000	74.3%	76.3%	72.2%
Brazil	Barra dos Coqueiros	2017	75.0%	76.9%	72.9%
Brazil	Barra Funda	2000	79.9%	83.7%	76.2%
Brazil	Barra Funda	2017	80.5%	84.2%	76.9%
Brazil	Barra Longa	2000	80.4%	84.1%	76.5%
Brazil	Barra Longa	2017	81.0%	84.6%	77.2%
Brazil	Barra Mansa	2000	82.3%	84.7%	79.7%
Brazil	Barra Mansa	2017	82.9%	85.2%	80.4%
Brazil	Barra Velha	2000	74.4%	77.7%	69.6%
Brazil	Barra Velha	2017	75.3%	78.6%	70.6%
Brazil	Barracão	2000	77.0%	81.0%	72.3%
Brazil	Barracão	2000	82.3%	86.4%	77.8%
Brazil	Barracão	2017	77.6%	81.6%	73.0%
Brazil	Barracão	2017	82.8%	86.8%	78.3%
Brazil	Barras	2000	59.6%	64.4%	55.2%
Brazil	Barras	2017	60.5%	65.2%	56.2%
Brazil	Barreira	2000	69.5%	73.0%	65.6%
Brazil	Barreira	2017	70.3%	73.8%	66.4%
Brazil	Barreiras	2000	77.7%	83.6%	71.9%
Brazil	Barreiras	2017	78.4%	84.1%	72.7%
Brazil	Barreiras do Piauí	2000	65.9%	73.4%	58.9%
Brazil	Barreiras do Piauí	2017	66.8%	74.2%	59.9%
Brazil	Barreirinha	2000	57.0%	61.3%	51.9%
Brazil	Barreirinha	2017	57.9%	62.2%	52.9%
Brazil	Barreirinhas	2000	61.4%	67.4%	54.3%
Brazil	Barreirinhas	2017	62.5%	68.4%	55.4%
Brazil	Barreiros	2000	64.0%	68.1%	59.6%
Brazil	Barreiros	2017	64.9%	68.9%	60.5%

Country	Second Administrative Unit	Year	Mean	Upper	Lower
Brazil	Barretos	2000	92.3%	94.4%	89.6%
Brazil	Barretos	2017	92.5%	94.6%	89.9%
Brazil	Barrinha	2000	92.5%	94.2%	89.9%
Brazil	Barrinha	2017	92.7%	94.4%	90.2%
Brazil	Barro	2000	71.1%	75.0%	67.7%
Brazil	Barro	2017	71.9%	75.7%	68.5%
Brazil	Barro Alto	2000	82.3%	86.1%	77.4%
Brazil	Barro Alto	2000	77.4%	82.7%	71.5%
Brazil	Barro Alto	2017	82.8%	86.6%	78.0%
Brazil	Barro Alto	2017	78.0%	83.3%	72.3%
Brazil	Barro Duro	2000	59.2%	63.6%	53.8%
Brazil	Barro Duro	2017	60.1%	64.5%	54.8%
Brazil	Barro Preto	2000	76.2%	80.2%	71.2%
Brazil	Barro Preto	2017	76.8%	80.7%	71.9%
Brazil	Barrolândia	2000	74.2%	78.3%	70.3%
Brazil	Barrolândia	2017	75.0%	78.9%	71.2%
Brazil	Barroquinha	2000	65.5%	70.7%	60.1%
Brazil	Barroquinha	2017	66.3%	71.5%	61.0%
Brazil	Barros Cassal	2000	83.4%	86.6%	79.6%
Brazil	Barros Cassal	2017	83.9%	87.1%	80.2%
Brazil	Barroso	2000	82.7%	86.3%	78.6%
Brazil	Barroso	2017	83.3%	86.7%	79.3%
Brazil	Barueri	2000	94.1%	95.0%	93.3%
Brazil	Barueri	2017	94.3%	95.1%	93.5%
Brazil	Bastos	2000	91.8%	93.8%	89.2%
Brazil	Bastos	2017	92.1%	94.1%	89.6%
Brazil	Bataguassu	2000	83.9%	87.4%	79.5%
Brazil	Bataguassu	2017	84.4%	87.8%	80.1%
Brazil	Bataiporã	2000	83.3%	86.6%	79.1%
Brazil	Bataiporã	2017	83.8%	87.1%	79.8%
Brazil	Batalha	2000	59.4%	63.3%	55.3%
Brazil	Batalha	2000	63.8%	66.9%	60.8%
Brazil	Batalha	2017	60.2%	64.2%	56.2%
Brazil	Batalha	2017	64.7%	67.8%	61.7%
Brazil	Batatais	2000	89.9%	92.2%	86.8%
Brazil	Batatais	2017	90.3%	92.5%	87.2%
Brazil	Baturité	2000	70.4%	74.1%	66.8%
Brazil	Baturité	2017	71.2%	74.8%	67.6%
Brazil	Bauru	2000	93.8%	95.6%	91.8%
Brazil	Bauru	2017	94.0%	95.7%	92.1%
Brazil	Bayeux	2000	67.6%	70.2%	65.0%
Brazil	Bayeux	2017	68.4%	71.0%	65.9%
Brazil	Bebedouro	2000	92.8%	94.5%	90.8%
Brazil	Bebedouro	2017	93.0%	94.7%	91.1%
Brazil	Beberibe	2000	69.7%	73.4%	65.5%
Brazil	Beberibe	2017	70.5%	74.1%	66.4%
Brazil	Bela Cruz	2000	68.0%	72.8%	62.4%
Brazil	Bela Cruz	2017	68.9%	73.5%	63.3%
Brazil	Bela Vista	2000	79.5%	84.6%	73.1%
Brazil	Bela Vista	2017	80.2%	85.2%	73.9%
Brazil	Bela Vista da Caroba	2000	82.3%	86.2%	78.5%
Brazil	Bela Vista da Caroba	2017	82.9%	86.6%	79.1%
Brazil	Bela Vista de Goiás	2000	78.9%	81.5%	75.9%
Brazil	Bela Vista de Goiás	2017	79.5%	82.0%	76.6%
Brazil	Bela Vista de Minas	2000	80.7%	84.6%	77.1%
Brazil	Bela Vista de Minas	2017	81.3%	85.1%	77.7%

Country	Second Administrative Unit	Year	Mean	Upper	Lower
Brazil	Bela Vista do Maranhão	2000	60.6%	65.3%	55.5%
Brazil	Bela Vista do Maranhão	2017	61.6%	66.2%	56.5%
Brazil	Bela Vista do Paraíso	2000	87.4%	89.4%	84.9%
Brazil	Bela Vista do Paraíso	2017	87.9%	89.7%	85.4%
Brazil	Bela Vista do Piauí	2000	61.4%	66.6%	55.4%
Brazil	Bela Vista do Piauí	2017	62.3%	67.5%	56.4%
Brazil	Bela Vista do Toldo	2000	77.9%	81.8%	73.8%
Brazil	Bela Vista do Toldo	2017	78.6%	82.4%	74.5%
Brazil	Belágua	2000	61.3%	66.9%	55.1%
Brazil	Belágua	2017	62.2%	67.7%	56.0%
Brazil	Belém	2000	72.0%	74.6%	68.9%
Brazil	Belém	2000	37.1%	39.5%	35.1%
Brazil	Belém	2000	60.0%	62.7%	57.5%
Brazil	Belém	2017	60.9%	63.7%	58.5%
Brazil	Belém	2017	38.0%	40.5%	36.0%
Brazil	Belém	2017	74.1%	76.6%	71.1%
Brazil	Belém de Maria	2000	63.6%	67.7%	60.1%
Brazil	Belém de Maria	2017	64.4%	68.5%	60.9%
Brazil	Belém de São Francisco	2000	63.8%	69.6%	57.8%
Brazil	Belém de São Francisco	2017	64.6%	70.3%	58.7%
Brazil	Belém do Brejo do Cruz	2000	77.6%	81.0%	74.0%
Brazil	Belém do Brejo do Cruz	2017	78.3%	81.6%	74.8%
Brazil	Belém do Piauí	2000	62.5%	67.4%	58.0%
Brazil	Belém do Piauí	2017	63.4%	68.2%	58.8%
Brazil	Belford Roxo	2000	77.8%	79.2%	76.2%
Brazil	Belford Roxo	2017	78.5%	79.9%	76.9%
Brazil	Belmiro Braga	2000	80.0%	83.2%	76.8%
Brazil	Belmiro Braga	2017	80.6%	83.8%	77.4%
Brazil	Belmonte	2000	79.7%	83.9%	75.5%
Brazil	Belmonte	2000	75.7%	81.2%	69.3%
Brazil	Belmonte	2017	80.3%	84.4%	76.2%
Brazil	Belmonte	2017	76.4%	81.8%	70.1%
Brazil	Belo Campo	2000	78.4%	83.3%	72.3%
Brazil	Belo Campo	2017	79.1%	83.9%	73.1%
Brazil	Belo Horizonte	2000	82.7%	85.0%	80.2%
Brazil	Belo Horizonte	2017	83.3%	85.5%	80.9%
Brazil	Belo Jardim	2000	67.3%	70.7%	64.0%
Brazil	Belo Jardim	2017	68.1%	71.5%	64.9%
Brazil	Belo Monte	2000	65.7%	69.0%	61.7%
Brazil	Belo Monte	2017	66.6%	69.8%	62.6%
Brazil	Belo Oriente	2000	79.8%	83.9%	75.1%
Brazil	Belo Oriente	2017	80.5%	84.5%	75.9%
Brazil	Belo Vale	2000	80.8%	84.2%	77.6%
Brazil	Belo Vale	2017	81.4%	84.7%	78.2%

Country	Second Administrative Unit	Year	Mean	Upper	Lower
Brazil	Belterra	2000	41.0%	47.3%	35.9%
Brazil	Belterra	2017	41.8%	48.1%	36.6%
Brazil	Beneditinos	2000	58.0%	62.4%	53.3%
Brazil	Beneditinos	2017	60.0%	64.3%	55.3%
Brazil	Benedito Leite	2000	61.5%	68.8%	53.0%
Brazil	Benedito Leite	2017	62.4%	69.7%	54.0%
Brazil	Benedito Novo	2000	73.9%	77.3%	70.3%
Brazil	Benedito Novo	2017	74.7%	78.0%	71.1%
Brazil	Benevides	2000	35.7%	38.1%	33.2%
Brazil	Benevides	2017	36.6%	39.1%	34.1%
Brazil	Benjamin Constant	2000	61.5%	67.8%	55.2%
Brazil	Benjamin Constant	2017	62.4%	68.6%	56.1%
Brazil	Benjamin Constant do Sul	2000	78.5%	81.6%	74.9%
Brazil	Benjamin Constant do Sul	2017	79.1%	82.1%	75.6%
Brazil	Bento de Abreu	2000	90.7%	93.0%	87.8%
Brazil	Bento de Abreu	2017	91.0%	93.3%	88.2%
Brazil	Bento Fernandes	2000	84.3%	87.0%	81.6%
Brazil	Bento Fernandes	2017	84.8%	87.4%	82.1%
Brazil	Bento Gonçalves	2000	83.4%	86.2%	80.5%
Brazil	Bento Gonçalves	2017	84.0%	86.7%	81.2%
Brazil	Bequimão	2000	60.0%	64.7%	54.5%
Brazil	Bequimão	2017	61.0%	65.6%	55.5%
Brazil	Berilo	2000	81.9%	86.6%	75.7%
Brazil	Berilo	2017	82.4%	87.0%	76.5%
Brazil	Berizal	2000	79.7%	84.8%	74.0%
Brazil	Berizal	2017	80.3%	85.3%	74.7%
Brazil	Bernardino Batista	2000	73.7%	77.6%	69.7%
Brazil	Bernardino Batista	2017	74.4%	78.2%	70.5%
Brazil	Bernardino de Campos	2000	92.6%	94.5%	90.2%
Brazil	Bernardino de Campos	2017	92.9%	94.7%	90.5%
Brazil	Bernardo do Mearim	2000	60.0%	65.2%	54.7%
Brazil	Bernardo do Mearim	2017	60.9%	66.1%	55.7%
Brazil	Bernardo Sayão	2000	65.8%	70.5%	60.8%
Brazil	Bernardo Sayão	2017	66.6%	71.3%	61.7%
Brazil	Bertioga	2000	87.4%	89.4%	85.0%
Brazil	Bertioga	2017	88.1%	90.0%	85.8%
Brazil	Bertolínia	2000	61.1%	67.4%	52.8%
Brazil	Bertolínia	2017	62.1%	68.3%	53.9%

Country	Second Administrative Unit	Year	Mean	Upper	Lower
Brazil	Bertópolis	2000	80.2%	84.9%	74.6%
Brazil	Bertópolis	2017	80.8%	85.3%	75.3%
Brazil	Beruri	2000	59.0%	64.4%	53.5%
Brazil	Beruri	2017	59.9%	65.3%	54.4%
Brazil	Betânia	2000	65.9%	71.3%	60.5%
Brazil	Betânia	2017	66.8%	72.1%	61.4%
Brazil	Betânia do Piauí	2000	64.2%	69.0%	59.1%
Brazil	Betânia do Piauí	2017	65.0%	69.7%	59.9%
Brazil	Betim	2000	81.7%	84.5%	78.8%
Brazil	Betim	2017	82.3%	85.0%	79.4%
Brazil	Bezerros	2000	68.7%	71.6%	65.8%
Brazil	Bezerros	2017	69.6%	72.4%	66.6%
Brazil	Bias Fortes	2000	81.0%	84.6%	76.8%
Brazil	Bias Fortes	2017	81.6%	85.1%	77.5%
Brazil	Bicas	2000	81.3%	84.3%	78.0%
Brazil	Bicas	2017	81.9%	84.8%	78.7%
Brazil	Biguaçu	2000	72.6%	77.6%	66.8%
Brazil	Biguaçu	2017	73.4%	78.3%	67.7%
Brazil	Bilac	2000	91.9%	93.9%	89.2%
Brazil	Bilac	2017	92.2%	94.1%	89.6%
Brazil	Biquinhas	2000	80.0%	85.7%	72.5%
Brazil	Biquinhas	2017	80.6%	86.1%	73.3%
Brazil	Birigui	2000	92.3%	94.3%	89.5%
Brazil	Birigui	2017	92.5%	94.6%	89.9%
Brazil	Biritiba Mirim	2000	93.6%	94.6%	92.2%
Brazil	Biritiba Mirim	2017	93.8%	94.8%	92.5%
Brazil	Biritinga	2000	79.0%	82.3%	74.4%
Brazil	Biritinga	2017	79.7%	82.9%	75.1%
Brazil	Bituruna	2000	80.4%	84.5%	75.5%
Brazil	Bituruna	2017	81.0%	85.0%	76.3%
Brazil	Blumenau	2000	72.9%	75.9%	69.8%
Brazil	Blumenau	2017	73.7%	76.6%	70.6%
Brazil	Boa Esperança	2000	76.6%	81.4%	72.9%
Brazil	Boa Esperança	2000	84.3%	87.6%	80.2%
Brazil	Boa Esperança	2000	83.7%	86.9%	79.3%
Brazil	Boa Esperança	2017	77.3%	82.0%	73.7%
Brazil	Boa Esperança	2017	84.2%	87.4%	79.9%
Brazil	Boa Esperança	2017	84.8%	88.0%	80.8%
Brazil	Boa Esperança do Iguaçu	2000	82.3%	86.3%	78.7%
Brazil	Boa Esperança do Iguaçu	2017	82.8%	86.7%	79.3%
Brazil	Boa Esperanca do Sul	2000	93.7%	95.2%	91.9%
Brazil	Boa Esperanca do Sul	2017	94.0%	95.4%	92.2%
Brazil	Boa Hora	2000	59.1%	63.9%	54.4%
Brazil	Boa Hora	2017	60.0%	64.8%	55.4%
Brazil	Boa Nova	2000	81.0%	85.0%	77.1%
Brazil	Boa Nova	2017	81.6%	85.5%	77.8%

Country	Second Administrative Unit	Year	Mean	Upper	Lower
Brazil	Boa Ventura	2000	67.2%	71.2%	63.0%
Brazil	Boa Ventura	2017	68.1%	71.9%	63.9%
Brazil	Boa Ventura de São Roque	2000	83.4%	87.8%	79.1%
Brazil	Boa Ventura de São Roque	2017	84.0%	88.3%	79.8%
Brazil	Boa Viagem	2000	70.8%	75.0%	66.2%
Brazil	Boa Viagem	2017	71.6%	75.7%	67.1%
Brazil	Boa Vista	2000	69.5%	73.5%	65.9%
Brazil	Boa Vista	2000	86.3%	87.5%	84.9%
Brazil	Boa Vista	2017	86.7%	87.9%	85.4%
Brazil	Boa Vista	2017	70.3%	74.3%	66.8%
Brazil	Boa Vista da Aparecida	2000	82.9%	86.0%	79.5%
Brazil	Boa Vista da Aparecida	2017	83.5%	86.5%	80.1%
Brazil	Boa Vista das Misses	2000	80.5%	84.5%	76.7%
Brazil	Boa Vista das Misses	2017	81.1%	85.0%	77.4%
Brazil	Boa Vista das Missões	2000	80.8%	84.6%	76.9%
Brazil	Boa Vista das Missões	2017	81.4%	85.1%	77.6%
Brazil	Boa Vista do Buricá	2000	79.8%	84.2%	74.7%
Brazil	Boa Vista do Buricá	2017	80.4%	84.7%	75.5%
Brazil	Boa Vista do Gurupi	2000	46.6%	52.5%	40.8%
Brazil	Boa Vista do Gurupi	2017	48.3%	54.1%	42.4%
Brazil	Boa Vista do Ramos	2000	57.8%	62.7%	52.2%
Brazil	Boa Vista do Ramos	2017	58.8%	63.7%	53.1%
Brazil	Boa Vista do Sul	2000	83.1%	85.8%	80.2%
Brazil	Boa Vista do Sul	2017	83.6%	86.3%	80.8%
Brazil	Boa Vista do Tupim	2000	75.7%	80.8%	69.8%
Brazil	Boa Vista do Tupim	2017	76.3%	81.4%	70.6%
Brazil	Boca da Mata	2000	60.1%	63.2%	57.1%
Brazil	Boca da Mata	2017	61.0%	64.0%	58.0%
Brazil	Boca do Acre	2000	50.5%	57.0%	44.3%
Brazil	Boca do Acre	2017	51.4%	57.9%	45.3%
Brazil	Bocaina	2000	94.4%	95.9%	92.6%
Brazil	Bocaina	2000	61.8%	67.2%	57.4%
Brazil	Bocaina	2017	94.6%	96.1%	92.9%
Brazil	Bocaina	2017	62.8%	68.0%	58.4%
Brazil	Bocaina de Minas	2000	86.1%	88.6%	83.4%
Brazil	Bocaina de Minas	2017	86.6%	89.0%	84.0%
Brazil	Bocaina do Sul	2000	76.8%	81.1%	71.7%
Brazil	Bocaina do Sul	2017	77.4%	81.6%	72.5%
Brazil	Bocaiúva	2000	82.4%	86.9%	77.7%
Brazil	Bocaiúva	2017	83.0%	87.4%	78.4%

Country	Second Administrative Unit	Year	Mean	Upper	Lower
Brazil	Bocaiúva do Sul	2000	86.3%	88.9%	83.5%
Brazil	Bocaiúva do Sul	2017	86.8%	89.3%	84.0%
Brazil	Bodó	2000	84.9%	87.5%	82.1%
Brazil	Bodó	2017	85.3%	87.9%	82.6%
Brazil	Bodocó	2000	67.1%	71.7%	62.2%
Brazil	Bodocó	2017	67.9%	72.5%	63.1%
Brazil	Bodoquena	2000	82.7%	87.1%	76.5%
Brazil	Bodoquena	2017	82.0%	86.5%	75.6%
Brazil	Bofete	2000	93.2%	94.8%	91.2%
Brazil	Bofete	2017	93.5%	95.0%	91.5%
Brazil	Boituva	2000	93.4%	94.8%	91.8%
Brazil	Boituva	2017	93.7%	95.0%	92.1%
Brazil	Bom Conselho	2000	64.1%	67.7%	60.7%
Brazil	Bom Conselho	2017	65.0%	68.5%	61.6%
Brazil	Bom despacho	2000	80.4%	84.7%	75.8%
Brazil	Bom despacho	2017	81.0%	85.2%	76.5%
Brazil	Bom Jardim	2000	63.1%	66.6%	60.0%
Brazil	Bom Jardim	2000	82.2%	85.0%	79.4%
Brazil	Bom Jardim	2017	64.0%	67.5%	60.9%
Brazil	Bom Jardim	2017	82.8%	85.5%	80.0%
Brazil	Bom Jardim da Serra	2000	81.4%	85.0%	77.5%
Brazil	Bom Jardim da Serra	2017	82.0%	85.5%	78.2%
Brazil	Bom Jardim de Goiás	2000	72.5%	78.1%	66.8%
Brazil	Bom Jardim de Goiás	2017	73.3%	78.8%	67.7%
Brazil	Bom Jardim de Minas	2000	83.8%	87.1%	80.7%
Brazil	Bom Jardim de Minas	2017	84.4%	87.5%	81.3%
Brazil	Bom Jardin	2000	59.7%	64.4%	55.6%
Brazil	Bom Jardin	2017	60.7%	65.4%	56.7%
Brazil	Bom Jesus	2000	82.4%	84.4%	80.3%
Brazil	Bom Jesus	2000	60.3%	67.4%	52.8%
Brazil	Bom Jesus	2000	71.1%	75.2%	67.7%
Brazil	Bom Jesus	2000	76.8%	81.1%	72.0%
Brazil	Bom Jesus	2000	80.0%	84.3%	75.3%
Brazil	Bom Jesus	2017	71.9%	75.9%	68.5%
Brazil	Bom Jesus	2017	80.6%	84.8%	76.0%
Brazil	Bom Jesus	2017	77.5%	81.7%	72.8%
Brazil	Bom Jesus	2017	61.2%	68.3%	53.7%
Brazil	Bom Jesus	2017	83.0%	84.9%	80.9%
Brazil	Bom Jesus da Lapa	2000	76.2%	82.0%	68.9%
Brazil	Bom Jesus da Lapa	2017	77.8%	83.3%	70.8%
Brazil	Bom Jesus da Penha	2000	86.4%	89.8%	82.1%
Brazil	Bom Jesus da Penha	2017	86.8%	90.1%	82.6%
Brazil	Bom Jesus da Serra	2000	79.3%	84.3%	73.6%
Brazil	Bom Jesus da Serra	2017	79.9%	84.8%	74.4%
Brazil	Bom Jesus das Selvas	2000	58.7%	64.1%	53.2%
Brazil	Bom Jesus das Selvas	2017	59.7%	65.2%	54.2%

Country	Second Administrative Unit	Year	Mean	Upper	Lower
Brazil	Bom Jesus de Goiás	2000	79.8%	83.5%	75.6%
Brazil	Bom Jesus de Goiás	2017	80.4%	84.1%	76.3%
Brazil	Bom Jesus do Amparo	2000	82.2%	85.5%	78.7%
Brazil	Bom Jesus do Amparo	2017	82.8%	86.0%	79.3%
Brazil	Bom Jesus do Galho	2000	81.2%	84.6%	77.4%
Brazil	Bom Jesus do Galho	2017	81.7%	85.1%	78.1%
Brazil	Bom Jesus do Itabapoana	2000	77.3%	81.3%	73.3%
Brazil	Bom Jesus do Itabapoana	2017	78.0%	81.9%	74.0%
Brazil	Bom Jesus do Norte	2000	76.8%	80.8%	72.7%
Brazil	Bom Jesus do Norte	2017	77.5%	81.4%	73.4%
Brazil	Bom Jesus do Oeste	2000	79.1%	83.1%	75.1%
Brazil	Bom Jesus do Oeste	2017	79.8%	83.6%	75.8%
Brazil	Bom Jesus do Sul	2000	82.0%	86.3%	77.2%
Brazil	Bom Jesus do Sul	2017	82.6%	86.8%	77.9%
Brazil	Bom Jesus do Tocantins	2000	48.9%	54.7%	43.6%
Brazil	Bom Jesus do Tocantins	2000	73.9%	78.5%	68.9%
Brazil	Bom Jesus do Tocantins	2017	49.8%	55.6%	44.5%
Brazil	Bom Jesus do Tocantins	2017	74.7%	79.3%	69.8%
Brazil	Bom Lugar	2000	61.0%	66.2%	56.2%
Brazil	Bom Lugar	2017	61.9%	67.1%	57.1%
Brazil	Bom Princípio	2000	79.3%	82.1%	76.5%
Brazil	Bom Princípio	2017	80.0%	82.7%	77.2%
Brazil	Bom Princípio do Piauí	2000	64.7%	68.8%	60.6%
Brazil	Bom Princípio do Piauí	2017	65.6%	69.6%	61.6%
Brazil	Bom Progresso	2000	81.0%	85.2%	76.3%
Brazil	Bom Progresso	2017	81.6%	85.7%	77.0%
Brazil	Bom Repouso	2000	92.7%	94.2%	90.9%
Brazil	Bom Repouso	2017	92.9%	94.4%	91.2%
Brazil	Bom Retiro	2000	77.0%	81.1%	71.7%
Brazil	Bom Retiro	2017	77.7%	81.7%	72.6%
Brazil	Bom Retiro do Sul	2000	80.2%	83.6%	76.7%
Brazil	Bom Retiro do Sul	2017	82.0%	85.2%	78.8%
Brazil	Bom Sucesso	2000	80.7%	84.6%	76.1%
Brazil	Bom Sucesso	2000	76.0%	79.6%	72.5%
Brazil	Bom Sucesso	2000	85.0%	87.6%	81.8%
Brazil	Bom Sucesso	2017	81.3%	85.1%	76.8%
Brazil	Bom Sucesso	2017	76.7%	80.2%	73.3%
Brazil	Bom Sucesso	2017	85.5%	88.1%	82.4%

Country	Second Administrative Unit	Year	Mean	Upper	Lower
Brazil	Bom Sucesso de Itararé	2000	89.7%	92.2%	86.4%
Brazil	Bom Sucesso de Itararé	2017	90.0%	92.5%	86.8%
Brazil	Bom Sucesso do Sul	2000	81.4%	85.4%	76.8%
Brazil	Bom Sucesso do Sul	2017	81.9%	85.9%	77.5%
Brazil	Bombinhas	2000	72.2%	77.2%	66.2%
Brazil	Bombinhas	2017	72.9%	77.8%	67.0%
Brazil	Bon Jesus dos Perdoes	2000	93.0%	94.2%	91.5%
Brazil	Bon Jesus dos Perdoes	2017	93.2%	94.4%	91.8%
Brazil	Bonfim	2000	80.8%	84.3%	77.1%
Brazil	Bonfim	2000	84.5%	87.2%	81.5%
Brazil	Bonfim	2017	81.4%	84.8%	77.8%
Brazil	Bonfim	2017	85.0%	87.7%	82.1%
Brazil	Bonfim do Piauí	2000	64.2%	69.6%	57.0%
Brazil	Bonfim do Piauí	2017	65.1%	70.4%	57.9%
Brazil	Bonfinópolis	2000	74.8%	80.5%	67.9%
Brazil	Bonfinópolis	2017	75.5%	81.1%	68.8%
Brazil	Bonfinópolis de Minas	2000	83.7%	88.9%	78.1%
Brazil	Bonfinópolis de Minas	2017	84.2%	89.3%	78.8%
Brazil	Boninal	2000	81.3%	85.8%	76.1%
Brazil	Boninal	2017	81.9%	86.3%	76.8%
Brazil	Bonito	2000	80.7%	85.4%	75.0%
Brazil	Bonito	2000	68.4%	71.8%	65.2%
Brazil	Bonito	2000	81.8%	86.1%	76.4%
Brazil	Bonito	2000	37.9%	42.5%	34.0%
Brazil	Bonito	2017	38.8%	43.5%	34.8%
Brazil	Bonito	2017	69.1%	72.5%	66.0%
Brazil	Bonito	2017	81.3%	85.9%	75.7%
Brazil	Bonito	2017	82.4%	86.6%	77.1%
Brazil	Bonito de Minas	2000	80.9%	86.9%	74.4%
Brazil	Bonito de Minas	2017	81.5%	87.3%	75.1%
Brazil	Bonito de Santa Fé	2000	69.4%	73.3%	65.9%
Brazil	Bonito de Santa Fé	2017	70.3%	74.1%	66.8%
Brazil	Bonópolis	2000	79.1%	81.2%	76.6%
Brazil	Bonópolis	2017	79.6%	81.5%	77.0%
Brazil	Boqueirão	2000	66.8%	70.5%	63.2%
Brazil	Boqueirão	2017	67.7%	71.3%	64.1%
Brazil	Boqueirão do Leão	2000	84.0%	87.5%	80.6%
Brazil	Boqueirão do Leão	2017	84.5%	87.9%	81.3%
Brazil	Boqueirão do Piauí	2000	59.2%	63.6%	55.1%
Brazil	Boqueirão do Piauí	2017	60.1%	64.5%	56.1%
Brazil	Boqueirao dos Cochos	2000	68.2%	72.0%	63.7%
Brazil	Boqueirao dos Cochos	2017	69.1%	72.7%	64.6%

Country	Second Administrative Unit	Year	Mean	Upper	Lower
Brazil	Boquira	2000	77.1%	82.9%	70.7%
Brazil	Boquira	2017	77.8%	83.4%	71.6%
Brazil	Borá	2000	91.4%	93.8%	89.0%
Brazil	Borá	2017	91.7%	94.0%	89.3%
Brazil	Boracéia	2000	93.2%	95.0%	91.0%
Brazil	Boracéia	2017	93.4%	95.2%	91.3%
Brazil	Borba	2000	58.5%	64.7%	51.6%
Brazil	Borba	2017	59.4%	65.5%	52.6%
Brazil	Borborema	2000	93.3%	95.1%	91.0%
Brazil	Borborema	2000	69.9%	72.8%	66.7%
Brazil	Borborema	2017	93.5%	95.3%	91.3%
Brazil	Borborema	2017	70.6%	73.4%	67.4%
Brazil	Borda da Mata	2000	89.4%	91.6%	86.6%
Brazil	Borda da Mata	2017	89.8%	91.9%	87.1%
Brazil	Borebi	2000	93.2%	95.0%	91.0%
Brazil	Borebi	2017	93.4%	95.2%	91.3%
Brazil	Borrazópolis	2000	83.4%	86.5%	79.8%
Brazil	Borrazópolis	2017	84.0%	86.9%	80.4%
Brazil	Bossoroca	2000	81.9%	86.4%	76.3%
Brazil	Bossoroca	2017	82.5%	86.8%	77.1%
Brazil	Botelhos	2000	88.1%	91.0%	84.5%
Brazil	Botelhos	2017	88.5%	91.3%	85.0%
Brazil	Botucatu	2000	93.9%	95.4%	92.2%
Brazil	Botucatu	2017	94.2%	95.6%	92.5%
Brazil	Botumirim	2000	82.8%	87.4%	76.9%
Brazil	Botumirim	2017	83.3%	87.8%	77.6%
Brazil	Botuporã	2000	77.6%	83.2%	71.2%
Brazil	Botuporã	2017	78.3%	83.7%	72.0%
Brazil	Botuverá	2000	71.6%	75.3%	67.5%
Brazil	Botuverá	2017	72.3%	76.0%	68.3%
Brazil	Braço do Norte	2000	72.7%	77.0%	68.2%
Brazil	Braço do Norte	2017	73.4%	77.7%	69.0%
Brazil	Braço do Trombudo	2000	73.7%	77.9%	68.8%
Brazil	Braço do Trombudo	2017	74.4%	78.6%	69.6%
Brazil	Braga	2000	80.8%	85.0%	76.5%
Brazil	Braga	2017	81.4%	85.5%	77.2%
Brazil	Bragança Paulista	2000	92.7%	94.1%	91.1%
Brazil	Bragança Paulista	2017	92.9%	94.3%	91.5%
Brazil	Braganey	2000	84.0%	87.5%	80.1%
Brazil	Braganey	2017	84.5%	87.9%	80.7%
Brazil	Braganga	2000	41.5%	46.7%	36.3%
Brazil	Braganga	2017	42.6%	47.8%	37.4%
Brazil	Branquinha	2000	60.7%	63.7%	57.4%
Brazil	Branquinha	2017	61.6%	64.5%	58.4%
Brazil	Bras Pires	2000	80.4%	83.8%	76.5%
Brazil	Bras Pires	2017	81.0%	84.3%	77.2%
Brazil	Brasabrantes	2000	77.3%	79.9%	74.9%
Brazil	Brasabrantes	2017	78.0%	80.5%	75.6%
Brazil	Brasilândia	2000	84.6%	87.7%	80.9%
Brazil	Brasilândia	2017	85.1%	88.0%	81.5%
Brazil	Brasilândia de Minas	2000	81.0%	85.3%	76.0%
Brazil	Brasilândia de Minas	2017	81.6%	85.8%	76.7%

Country	Second Administrative Unit	Year	Mean	Upper	Lower
Brazil	Brasilândia do Sul	2000	83.4%	86.9%	79.6%
Brazil	Brasilândia do Sul	2017	83.9%	87.3%	80.3%
Brazil	Brasilândia do Tocantins	2000	74.4%	78.9%	69.6%
Brazil	Brasilândia do Tocantins	2017	75.1%	79.5%	70.4%
Brazil	Brasiléia	2000	43.6%	48.1%	39.5%
Brazil	Brasiléia	2017	44.5%	49.1%	40.3%
Brazil	Brasília	2000	92.4%	93.0%	91.6%
Brazil	Brasília	2017	92.7%	93.3%	91.9%
Brazil	Brasília de Minas	2000	82.5%	87.2%	76.8%
Brazil	Brasília de Minas	2017	83.0%	87.7%	77.5%
Brazil	Brasnorte	2000	64.7%	73.2%	57.2%
Brazil	Brasnorte	2017	65.6%	73.9%	58.1%
Brazil	Brasópolis	2000	85.8%	88.9%	82.6%
Brazil	Brasópolis	2017	85.6%	89.1%	81.9%
Brazil	Brauna	2000	92.3%	94.3%	90.1%
Brazil	Brauna	2017	92.6%	94.5%	90.4%
Brazil	Braúnas	2000	90.8%	92.8%	88.5%
Brazil	Braúnas	2017	91.2%	93.1%	88.9%
Brazil	Brazil Novo	2000	41.5%	50.4%	32.1%
Brazil	Brazil Novo	2017	42.4%	51.3%	33.0%
Brazil	Brazileira	2000	60.7%	65.5%	55.9%
Brazil	Brazileira	2017	61.6%	66.4%	56.9%
Brazil	Brejão	2000	62.8%	66.3%	59.3%
Brazil	Brejão	2017	63.8%	67.1%	60.3%
Brazil	Brejetuba	2000	80.4%	83.9%	77.3%
Brazil	Brejetuba	2017	81.1%	84.5%	78.0%
Brazil	Brejinho	2000	80.1%	82.4%	77.8%
Brazil	Brejinho	2000	72.2%	76.5%	68.0%
Brazil	Brejinho	2017	80.7%	83.0%	78.5%
Brazil	Brejinho	2017	73.0%	77.2%	68.8%
Brazil	Brejinho de Nazaré	2000	74.7%	79.3%	70.4%
Brazil	Brejinho de Nazaré	2017	75.4%	79.9%	71.2%
Brazil	Brejo	2000	59.8%	64.7%	54.9%
Brazil	Brejo	2017	60.7%	65.5%	55.8%
Brazil	Brejo Alegre	2000	92.0%	94.2%	89.2%
Brazil	Brejo Alegre	2017	92.3%	94.4%	89.6%
Brazil	Brejo da Madre de deus	2000	66.4%	69.7%	62.9%
Brazil	Brejo da Madre de deus	2017	67.3%	70.6%	63.7%
Brazil	Brejo de Areia	2000	62.1%	67.3%	56.8%
Brazil	Brejo de Areia	2017	63.0%	68.1%	57.7%
Brazil	Brejo do Cruz	2000	75.5%	78.9%	71.6%
Brazil	Brejo do Cruz	2017	76.2%	79.6%	72.4%
Brazil	Brejo do Piauí	2000	61.5%	67.9%	54.6%
Brazil	Brejo do Piauí	2017	62.4%	68.8%	55.6%
Brazil	Brejo dos Santos	2000	76.1%	79.8%	72.7%
Brazil	Brejo dos Santos	2017	76.8%	80.4%	73.5%
Brazil	Brejo Grande	2000	66.0%	69.8%	62.4%
Brazil	Brejo Grande	2017	66.9%	70.6%	63.3%

Country	Second Administrative Unit	Year	Mean	Upper	Lower
Brazil	Brejo Grande do Araguaia	2000	60.5%	65.7%	55.4%
Brazil	Brejo Grande do Araguaia	2017	61.4%	66.6%	56.4%
Brazil	Brejo Santo	2000	67.5%	71.4%	63.8%
Brazil	Brejo Santo	2017	68.5%	72.4%	64.9%
Brazil	Brejões	2000	81.0%	84.8%	75.6%
Brazil	Brejões	2017	81.6%	85.3%	76.3%
Brazil	Brejolândia	2000	76.1%	82.8%	66.7%
Brazil	Brejolândia	2017	76.8%	83.4%	67.6%
Brazil	Breu Branco	2000	38.3%	43.2%	32.3%
Brazil	Breu Branco	2017	39.2%	44.3%	33.2%
Brazil	Breves	2000	42.6%	47.8%	36.9%
Brazil	Breves	2017	43.5%	48.8%	37.8%
Brazil	Brochier	2000	80.7%	83.8%	77.7%
Brazil	Brochier	2017	81.3%	84.3%	78.4%
Brazil	Brodosqui	2000	90.3%	92.6%	87.4%
Brazil	Brodosqui	2017	90.7%	92.8%	87.8%
Brazil	Brotas	2000	93.3%	94.9%	91.3%
Brazil	Brotas	2017	93.5%	95.0%	91.6%
Brazil	Brotas de Macaúbas	2000	77.6%	83.6%	70.9%
Brazil	Brotas de Macaúbas	2017	78.3%	84.1%	71.7%
Brazil	Brumadinho	2000	81.3%	84.2%	78.3%
Brazil	Brumadinho	2017	81.9%	84.7%	79.0%
Brazil	Brumado	2000	77.3%	82.3%	71.4%
Brazil	Brumado	2017	78.0%	82.8%	72.2%
Brazil	Brunópolis	2000	75.2%	79.8%	69.6%
Brazil	Brunópolis	2017	75.9%	80.5%	70.4%
Brazil	Brusque	2000	71.4%	74.9%	67.2%
Brazil	Brusque	2017	72.9%	76.3%	68.8%
Brazil	Bueno Brandão	2000	91.8%	93.3%	89.9%
Brazil	Bueno Brandão	2017	92.1%	93.5%	90.2%
Brazil	Buenópolis	2000	81.3%	86.0%	75.1%
Brazil	Buenópolis	2017	81.9%	86.5%	75.9%
Brazil	Buenos Aires	2000	66.3%	69.7%	63.4%
Brazil	Buenos Aires	2017	67.1%	70.5%	64.3%
Brazil	Buerarema	2000	76.9%	81.1%	71.8%
Brazil	Buerarema	2017	77.6%	81.7%	72.6%
Brazil	Bugre	2000	80.2%	84.2%	75.8%
Brazil	Bugre	2017	80.8%	84.7%	76.4%
Brazil	Buíque	2000	63.7%	67.7%	59.4%
Brazil	Buíque	2017	64.6%	68.5%	60.3%
Brazil	Bujari	2000	41.2%	44.3%	38.4%
Brazil	Bujari	2017	42.1%	45.2%	39.3%
Brazil	Bujaru	2000	35.2%	38.5%	31.6%
Brazil	Bujaru	2017	36.1%	39.4%	32.5%
Brazil	Buquim	2000	77.4%	80.0%	75.2%
Brazil	Buquim	2017	78.1%	80.6%	75.9%
Brazil	Buri	2000	93.0%	95.0%	90.5%
Brazil	Buri	2017	93.2%	95.2%	90.9%
Brazil	Buritama	2000	91.8%	94.1%	88.9%
Brazil	Buritama	2017	92.1%	94.3%	89.3%
Brazil	Buriti	2000	60.9%	65.6%	56.2%
Brazil	Buriti	2017	61.9%	66.5%	57.2%
Brazil	Buriti Alegre	2000	78.1%	82.6%	73.4%
Brazil	Buriti Alegre	2017	78.8%	83.1%	74.2%
Brazil	Buriti Bravo	2000	61.4%	67.1%	55.0%
Brazil	Buriti Bravo	2017	62.3%	68.0%	56.0%

Country	Second Administrative Unit	Year	Mean	Upper	Lower
Brazil	Buriti de Goiás	2000	75.4%	80.1%	70.0%
Brazil	Buriti de Goiás	2017	76.1%	80.7%	70.8%
Brazil	Buriti do Tocantins	2000	60.3%	65.3%	55.1%
Brazil	Buriti do Tocantins	2017	61.3%	66.2%	56.1%
Brazil	Buriti dos Lopes	2000	62.4%	67.1%	58.1%
Brazil	Buriti dos Lopes	2017	63.2%	67.9%	59.1%
Brazil	Buriti dos Montes	2000	66.2%	71.2%	61.4%
Brazil	Buriti dos Montes	2017	67.2%	72.1%	62.4%
Brazil	Buriticupu	2000	59.6%	65.7%	53.9%
Brazil	Buriticupu	2017	60.5%	66.5%	54.9%
Brazil	Buritinópolis	2000	81.5%	86.5%	75.9%
Brazil	Buritinópolis	2017	82.1%	86.9%	76.7%
Brazil	Buritirama	2000	69.5%	76.5%	61.4%
Brazil	Buritirama	2017	70.3%	77.2%	62.4%
Brazil	Buritirana	2000	67.5%	73.0%	61.6%
Brazil	Buritirana	2017	68.3%	73.7%	62.5%
Brazil	Buritis	2000	86.2%	90.4%	81.0%
Brazil	Buritis	2000	27.9%	31.9%	23.9%
Brazil	Buritis	2017	86.6%	90.7%	81.6%
Brazil	Buritis	2017	28.7%	32.7%	24.7%
Brazil	Buritizal	2000	87.2%	90.7%	82.6%
Brazil	Buritizal	2017	87.6%	91.0%	83.1%
Brazil	Buritizeiro	2000	81.5%	86.6%	75.3%
Brazil	Buritizeiro	2017	82.1%	87.1%	76.0%
Brazil	Butiá	2000	81.6%	84.8%	77.7%
Brazil	Butiá	2017	82.2%	85.3%	78.4%
Brazil	Caapiranga	2000	60.2%	66.1%	55.3%
Brazil	Caapiranga	2017	61.1%	66.9%	56.3%
Brazil	Caaporã	2000	64.7%	67.9%	61.3%
Brazil	Caaporã	2017	65.6%	68.8%	62.2%
Brazil	Caarapó	2000	81.9%	85.0%	78.2%
Brazil	Caarapó	2017	82.5%	85.5%	78.8%
Brazil	Caatiba	2000	78.2%	83.0%	72.7%
Brazil	Caatiba	2017	79.0%	83.6%	73.6%
Brazil	Cabaceiras	2000	67.5%	72.1%	63.2%
Brazil	Cabaceiras	2017	68.4%	72.9%	64.1%
Brazil	Cabaceiras do Paraguaçu	2000	76.5%	80.1%	72.3%
Brazil	Cabaceiras do Paraguaçu	2017	77.2%	80.8%	73.0%
Brazil	Cabeceira Grande	2000	88.4%	91.2%	84.9%
Brazil	Cabeceira Grande	2017	88.8%	91.5%	85.4%
Brazil	Cabeceiras	2000	88.1%	91.5%	84.1%
Brazil	Cabeceiras	2017	88.5%	91.8%	84.7%
Brazil	Cabeceiras do Piauí	2000	59.5%	64.6%	55.6%
Brazil	Cabeceiras do Piauí	2017	60.5%	65.5%	56.5%
Brazil	Cabedelo	2000	66.2%	69.1%	62.9%
Brazil	Cabedelo	2017	67.1%	70.0%	63.8%
Brazil	Cabixi	2000	33.1%	39.1%	28.0%
Brazil	Cabixi	2017	33.9%	40.0%	28.8%

Country	Second Administrative Unit	Year	Mean	Upper	Lower
Brazil	Cabo	2000	63.9%	66.4%	61.0%
Brazil	Cabo	2017	64.7%	67.3%	61.9%
Brazil	Cabo Frio	2000	77.6%	81.1%	73.1%
Brazil	Cabo Frio	2017	78.3%	81.7%	73.8%
Brazil	Cabo Verde	2000	87.0%	89.9%	83.2%
Brazil	Cabo Verde	2017	87.5%	90.3%	83.8%
Brazil	Cabrália Paulista	2000	93.1%	95.0%	91.1%
Brazil	Cabrália Paulista	2017	93.4%	95.2%	91.4%
Brazil	Cabreúva	2000	93.9%	94.8%	92.8%
Brazil	Cabreúva	2017	94.2%	95.0%	93.1%
Brazil	Cabrobó	2000	66.1%	71.7%	60.7%
Brazil	Cabrobó	2017	67.0%	72.4%	61.6%
Brazil	Caçador	2000	77.7%	82.5%	73.5%
Brazil	Caçador	2017	78.9%	83.5%	74.9%
Brazil	Caçapava	2000	92.5%	94.1%	91.0%
Brazil	Caçapava	2017	92.8%	94.3%	91.3%
Brazil	Caçapava do Sul	2000	84.5%	88.3%	80.1%
Brazil	Caçapava do Sul	2017	85.0%	88.7%	80.7%
Brazil	Cacaulândia	2000	27.7%	31.8%	24.0%
Brazil	Cacaulândia	2017	28.4%	32.7%	24.7%
Brazil	Cacequi	2000	81.5%	86.1%	75.7%
Brazil	Cacequi	2017	82.1%	86.6%	76.4%
Brazil	Cáceres	2000	69.4%	75.1%	62.2%
Brazil	Cáceres	2017	70.2%	75.8%	63.2%
Brazil	Cachoeira	2000	76.6%	79.8%	73.2%
Brazil	Cachoeira	2017	77.5%	80.6%	74.3%
Brazil	Cachoeira Alta	2000	80.3%	84.6%	75.7%
Brazil	Cachoeira Alta	2017	81.0%	85.2%	76.4%
Brazil	Cachoeira da Prata	2000	79.9%	83.7%	75.2%
Brazil	Cachoeira da Prata	2017	80.5%	84.3%	75.9%
Brazil	Cachoeira de Goias	2000	77.2%	81.5%	72.5%
Brazil	Cachoeira de Goias	2017	77.9%	82.1%	73.3%
Brazil	Cachoeira de Minas	2000	88.5%	90.9%	85.8%
Brazil	Cachoeira de Minas	2017	88.9%	91.2%	86.3%
Brazil	Cachoeira de Pajes	2000	80.9%	85.3%	74.5%
Brazil	Cachoeira de Pajes	2017	81.5%	85.8%	75.2%
Brazil	Cachoeira do Arari	2000	37.0%	42.1%	32.5%
Brazil	Cachoeira do Arari	2017	37.9%	43.2%	33.3%
Brazil	Cachoeira do Piriá	2000	45.3%	51.0%	39.6%
Brazil	Cachoeira do Piriá	2017	46.8%	52.5%	41.0%
Brazil	Cachoeira do Sul	2000	85.6%	88.9%	82.0%
Brazil	Cachoeira do Sul	2017	85.6%	88.9%	82.0%

Country	Second Administrative Unit	Year	Mean	Upper	Lower
Brazil	Cachoeira dos índios	2000	71.1%	74.9%	67.8%
Brazil	Cachoeira dos índios	2017	71.9%	75.7%	68.7%
Brazil	Cachoeira dourada	2000	79.8%	84.4%	75.4%
Brazil	Cachoeira dourada	2000	79.9%	84.4%	75.5%
Brazil	Cachoeira dourada	2017	80.5%	84.9%	76.2%
Brazil	Cachoeira dourada	2017	80.6%	84.9%	76.2%
Brazil	Cachoeira Grande	2000	61.0%	65.2%	56.1%
Brazil	Cachoeira Grande	2017	61.9%	66.1%	57.0%
Brazil	Cachoeira Paulista	2000	87.5%	89.8%	84.8%
Brazil	Cachoeira Paulista	2017	87.9%	90.1%	85.3%
Brazil	Cachoeiras de Macacu	2000	77.9%	80.2%	75.1%
Brazil	Cachoeiras de Macacu	2017	78.5%	80.8%	75.8%
Brazil	Cachoeirinha	2000	81.5%	83.3%	79.6%
Brazil	Cachoeirinha	2000	72.2%	76.2%	67.8%
Brazil	Cachoeirinha	2017	82.6%	84.4%	80.8%
Brazil	Cachoeirinha	2017	73.0%	76.9%	68.6%
Brazil	Cachoeiro de Itapemirim	2000	76.7%	79.5%	72.8%
Brazil	Cachoeiro de Itapemirim	2017	77.4%	80.2%	73.7%
Brazil	Cachoerinha	2000	66.3%	69.5%	62.9%
Brazil	Cachoerinha	2017	67.2%	70.3%	63.8%
Brazil	Cacimba de Areia	2000	69.1%	73.2%	64.5%
Brazil	Cacimba de Areia	2017	70.0%	74.0%	65.5%
Brazil	Cacimba de dentro	2000	76.3%	79.2%	73.1%
Brazil	Cacimba de dentro	2017	77.0%	79.8%	73.9%
Brazil	Cacimbas	2000	71.4%	75.6%	67.2%
Brazil	Cacimbas	2017	72.2%	76.3%	68.1%
Brazil	Cacimbinhas	2000	62.6%	65.6%	59.0%
Brazil	Cacimbinhas	2017	63.5%	66.5%	60.0%
Brazil	Cacique doble	2000	77.6%	81.4%	73.1%
Brazil	Cacique doble	2017	78.2%	82.0%	73.8%
Brazil	Cacoal	2000	30.0%	34.0%	26.7%
Brazil	Cacoal	2017	30.9%	34.9%	27.5%
Brazil	Caconde	2000	87.6%	90.6%	84.2%
Brazil	Caconde	2017	88.0%	90.9%	84.7%
Brazil	Caçu	2000	79.2%	83.6%	73.7%
Brazil	Caçu	2017	79.8%	84.1%	74.4%
Brazil	Caculé	2000	78.6%	83.7%	73.2%
Brazil	Caculé	2017	79.3%	84.3%	74.0%
Brazil	Caém	2000	76.8%	81.3%	71.9%
Brazil	Caém	2017	77.5%	81.9%	72.7%
Brazil	Caetanópolis	2000	81.3%	85.3%	76.6%
Brazil	Caetanópolis	2017	81.8%	85.8%	77.3%
Brazil	Caetanos	2000	77.2%	82.7%	71.1%
Brazil	Caetanos	2017	77.8%	83.3%	72.0%

Country	Second Administrative Unit	Year	Mean	Upper	Lower
Brazil	Caeté	2000	81.2%	84.3%	77.8%
Brazil	Caeté	2017	81.8%	84.8%	78.5%
Brazil	Caetés	2000	65.7%	69.2%	62.3%
Brazil	Caetés	2017	66.6%	70.0%	63.2%
Brazil	Caetité	2000	79.3%	84.1%	74.5%
Brazil	Caetité	2017	79.9%	84.6%	75.2%
Brazil	Cafarnaum	2000	78.4%	84.1%	73.1%
Brazil	Cafarnaum	2017	79.1%	84.7%	73.8%
Brazil	Cafeara	2000	85.5%	88.3%	82.1%
Brazil	Cafeara	2017	86.0%	88.7%	82.7%
Brazil	Cafelândia	2000	84.8%	88.6%	81.0%
Brazil	Cafelândia	2000	93.6%	95.6%	91.2%
Brazil	Cafelândia	2017	85.3%	89.0%	81.6%
Brazil	Cafelândia	2017	93.8%	95.8%	91.5%
Brazil	Cafezal do Sul	2000	83.3%	86.8%	79.9%
Brazil	Cafezal do Sul	2017	83.9%	87.3%	80.5%
Brazil	Caiabu	2000	89.6%	92.3%	86.7%
Brazil	Caiabu	2017	89.9%	92.6%	87.2%
Brazil	Caiana	2000	80.6%	83.7%	77.5%
Brazil	Caiana	2017	81.2%	84.2%	78.1%
Brazil	Caiapônia	2000	76.9%	82.1%	71.3%
Brazil	Caiapônia	2017	77.6%	82.6%	72.1%
Brazil	Caibaté	2000	81.6%	86.2%	76.4%
Brazil	Caibaté	2017	82.1%	86.7%	77.1%
Brazil	Caibi	2000	77.0%	81.3%	72.5%
Brazil	Caibi	2017	77.6%	81.8%	73.2%
Brazil	Caiçara	2000	78.8%	82.6%	73.9%
Brazil	Caiçara	2000	75.0%	77.6%	72.5%
Brazil	Caiçara	2017	79.4%	83.1%	74.6%
Brazil	Caiçara	2017	75.7%	78.2%	73.3%
Brazil	Caiçara do Norte	2000	84.4%	87.0%	81.5%
Brazil	Caiçara do Norte	2017	84.9%	87.4%	82.1%
Brazil	Caiçara do Rio do Vento	2000	77.0%	80.2%	73.4%
Brazil	Caiçara do Rio do Vento	2017	77.7%	80.8%	74.1%
Brazil	Caicó	2000	83.3%	86.9%	78.6%
Brazil	Caicó	2017	83.9%	87.4%	79.3%
Brazil	Caieiras	2000	94.0%	94.9%	93.1%
Brazil	Caieiras	2017	94.2%	95.1%	93.3%
Brazil	Cairu	2000	69.8%	75.1%	65.1%
Brazil	Cairu	2017	70.7%	75.9%	66.1%
Brazil	Caiuá	2000	86.1%	88.8%	82.5%
Brazil	Caiuá	2017	86.5%	89.2%	83.0%
Brazil	Cajamar	2000	93.7%	94.5%	92.6%
Brazil	Cajamar	2017	93.9%	94.7%	92.9%
Brazil	Cajapió	2000	60.3%	65.3%	55.4%
Brazil	Cajapió	2017	61.2%	66.2%	56.3%
Brazil	Cajari	2000	60.0%	64.4%	54.9%
Brazil	Cajari	2017	60.9%	65.3%	55.9%
Brazil	Cajati	2000	87.9%	91.2%	84.0%
Brazil	Cajati	2017	89.2%	92.1%	85.6%
Brazil	Cajazeiras	2000	72.5%	76.2%	69.2%
Brazil	Cajazeiras	2017	73.3%	76.9%	70.0%
Brazil	Cajazeiras do Piauí	2000	59.0%	63.9%	53.7%
Brazil	Cajazeiras do Piauí	2017	60.0%	64.9%	54.7%
Brazil	Cajazeirinhas	2000	68.2%	71.9%	64.6%
Brazil	Cajazeirinhas	2017	69.1%	72.7%	65.5%

Country	Second Administrative Unit	Year	Mean	Upper	Lower
Brazil	Cajobi	2000	92.9%	94.6%	91.0%
Brazil	Cajobi	2017	93.2%	94.8%	91.3%
Brazil	Cajueiro	2000	58.2%	61.1%	55.0%
Brazil	Cajueiro	2017	61.7%	64.6%	58.6%
Brazil	Cajueiro da Praia	2000	64.7%	70.3%	59.6%
Brazil	Cajueiro da Praia	2017	65.6%	71.1%	60.5%
Brazil	Cajuri	2000	81.6%	84.8%	77.7%
Brazil	Cajuri	2017	82.1%	85.3%	78.4%
Brazil	Cajuru	2000	90.1%	92.4%	87.1%
Brazil	Cajuru	2017	90.5%	92.7%	87.5%
Brazil	Calçado	2000	66.6%	69.6%	63.5%
Brazil	Calçado	2017	67.5%	70.5%	64.5%
Brazil	Calçoene	2000	44.5%	50.7%	38.3%
Brazil	Calçoene	2017	45.5%	51.7%	39.2%
Brazil	Caldas	2000	89.7%	91.9%	86.8%
Brazil	Caldas	2017	90.1%	92.2%	87.2%
Brazil	Caldas Brandão	2000	67.3%	70.9%	64.1%
Brazil	Caldas Brandão	2017	68.1%	71.7%	65.0%
Brazil	Caldas Novas	2000	78.2%	82.4%	73.7%
Brazil	Caldas Novas	2017	78.9%	82.9%	74.4%
Brazil	Caldazinha	2000	77.7%	80.0%	75.2%
Brazil	Caldazinha	2017	78.3%	80.6%	75.9%
Brazil	Caldeirão Grande	2000	76.6%	81.4%	71.0%
Brazil	Caldeirão Grande	2017	77.3%	82.0%	71.8%
Brazil	Caldeirão Grande do Piauí	2000	65.2%	69.3%	60.6%
Brazil	Caldeirão Grande do Piauí	2017	66.0%	70.1%	61.5%
Brazil	Califórnia	2000	85.5%	87.9%	82.6%
Brazil	Califórnia	2017	85.9%	88.3%	83.2%
Brazil	Calmon	2000	80.4%	84.4%	76.2%
Brazil	Calmon	2017	81.0%	84.8%	76.8%
Brazil	Calumbi	2000	67.0%	71.6%	61.9%
Brazil	Calumbi	2017	67.9%	72.4%	62.8%
Brazil	Camacan	2000	77.5%	82.2%	72.1%
Brazil	Camacan	2017	78.1%	82.8%	72.9%
Brazil	Camaçari	2000	76.5%	79.8%	72.9%
Brazil	Camaçari	2017	77.3%	80.5%	73.8%
Brazil	Camacho	2000	82.4%	86.3%	77.4%
Brazil	Camacho	2017	83.0%	86.7%	78.1%
Brazil	Camagua	2000	83.3%	86.9%	78.9%
Brazil	Camagua	2017	83.8%	87.3%	79.5%
Brazil	Camalaú	2000	65.9%	70.0%	61.3%
Brazil	Camalaú	2017	66.8%	70.8%	62.2%
Brazil	Camamu	2000	70.2%	76.4%	64.8%
Brazil	Camamu	2017	71.0%	77.1%	65.7%
Brazil	Camanducaia	2000	93.4%	94.6%	92.0%
Brazil	Camanducaia	2017	93.6%	94.8%	92.2%
Brazil	Camapuã	2000	78.5%	83.0%	72.4%
Brazil	Camapuã	2017	79.2%	83.5%	73.2%
Brazil	Camaragibe	2000	66.4%	68.4%	64.4%
Brazil	Camaragibe	2017	67.5%	69.4%	65.5%
Brazil	Camargo	2000	81.5%	84.7%	77.5%
Brazil	Camargo	2017	82.0%	85.2%	78.1%

Country	Second Administrative Unit	Year	Mean	Upper	Lower
Brazil	Cambará	2000	90.3%	92.5%	87.2%
Brazil	Cambará	2017	90.6%	92.7%	87.6%
Brazil	Cambará do Sul	2000	81.3%	85.1%	77.0%
Brazil	Cambará do Sul	2017	81.9%	85.5%	77.7%
Brazil	Cambé	2000	86.4%	88.4%	83.8%
Brazil	Cambé	2017	86.9%	88.8%	84.3%
Brazil	Cambira	2000	84.6%	87.1%	81.5%
Brazil	Cambira	2017	85.1%	87.5%	82.1%
Brazil	Camboriú	2000	74.0%	77.9%	69.3%
Brazil	Camboriú	2017	74.9%	78.6%	70.3%
Brazil	Cambuci	2000	77.1%	80.6%	73.2%
Brazil	Cambuci	2017	77.8%	81.2%	73.9%
Brazil	Cambuí	2000	90.6%	92.5%	88.6%
Brazil	Cambuí	2017	90.9%	92.7%	88.9%
Brazil	Cambuquira	2000	84.1%	87.3%	80.5%
Brazil	Cambuquira	2017	84.6%	87.7%	81.1%
Brazil	Cametá	2000	37.6%	43.4%	33.1%
Brazil	Cametá	2017	38.5%	44.4%	34.0%
Brazil	Camocim	2000	66.2%	71.9%	60.7%
Brazil	Camocim	2017	68.6%	74.0%	63.3%
Brazil	Camocim de São Félix	2000	69.2%	72.6%	66.1%
Brazil	Camocim de São Félix	2017	70.0%	73.4%	66.9%
Brazil	Campanário	2000	80.8%	85.7%	76.0%
Brazil	Campanário	2017	81.3%	86.2%	76.7%
Brazil	Campanha	2000	85.7%	88.5%	82.2%
Brazil	Campanha	2017	86.2%	88.9%	82.8%
Brazil	Campestre	2000	88.4%	90.9%	84.8%
Brazil	Campestre	2000	61.9%	65.4%	58.6%
Brazil	Campestre	2017	62.8%	66.3%	59.6%
Brazil	Campestre	2017	88.2%	90.8%	84.6%
Brazil	Campestre da Serra	2000	79.9%	83.3%	75.6%
Brazil	Campestre da Serra	2017	80.5%	83.8%	76.3%
Brazil	Campestre de Goiás	2000	75.9%	78.9%	72.8%
Brazil	Campestre de Goiás	2017	76.6%	79.5%	73.6%
Brazil	Campestre do Maranhão	2000	72.4%	77.2%	67.9%
Brazil	Campestre do Maranhão	2017	73.2%	77.8%	68.8%
Brazil	Campina da Lagoa	2000	84.2%	87.7%	79.9%
Brazil	Campina da Lagoa	2017	84.7%	88.1%	80.5%
Brazil	Campina das Missões	2000	81.0%	86.1%	75.1%
Brazil	Campina das Missões	2017	81.6%	86.5%	75.9%
Brazil	Campina do Monte Alegre	2000	92.9%	94.8%	90.2%
Brazil	Campina do Monte Alegre	2017	93.1%	95.0%	90.5%
Brazil	Campina do Simão	2000	83.3%	87.2%	79.0%
Brazil	Campina do Simão	2017	83.8%	87.7%	79.6%

Country	Second Administrative Unit	Year	Mean	Upper	Lower
Brazil	Campina Grande	2000	70.7%	73.8%	68.2%
Brazil	Campina Grande	2017	71.6%	74.6%	69.1%
Brazil	Campina Grande do Sul	2000	85.8%	88.1%	83.2%
Brazil	Campina Grande do Sul	2017	86.3%	88.5%	83.7%
Brazil	Campina Verde	2000	85.8%	89.5%	81.1%
Brazil	Campina Verde	2017	86.2%	89.9%	81.7%
Brazil	Campinaçu	2000	78.1%	83.5%	72.9%
Brazil	Campinaçu	2017	78.7%	84.0%	73.6%
Brazil	Campinápolis	2000	70.5%	78.1%	62.9%
Brazil	Campinápolis	2017	71.3%	78.8%	63.8%
Brazil	Campinas	2000	93.3%	94.4%	92.1%
Brazil	Campinas	2017	93.5%	94.6%	92.4%
Brazil	Campinas do Piauí	2000	60.5%	66.0%	55.0%
Brazil	Campinas do Piauí	2017	61.4%	66.9%	55.9%
Brazil	Campinas do Sul	2000	79.3%	82.9%	75.8%
Brazil	Campinas do Sul	2017	79.9%	83.4%	76.5%
Brazil	Campinorte	2000	78.3%	83.1%	73.5%
Brazil	Campinorte	2017	79.1%	83.6%	74.3%
Brazil	Campo	2000	84.0%	86.2%	81.5%
Brazil	Campo	2017	84.5%	86.8%	82.1%
Brazil	Campo Alegre	2000	64.1%	67.5%	61.2%
Brazil	Campo Alegre	2000	81.0%	84.0%	77.7%
Brazil	Campo Alegre	2017	81.5%	84.6%	78.4%
Brazil	Campo Alegre	2017	65.0%	68.3%	62.1%
Brazil	Campo Alegre de Goiás	2000	79.8%	84.6%	74.8%
Brazil	Campo Alegre de Goiás	2017	80.4%	85.1%	75.6%
Brazil	Campo Alegre de Lourdes	2000	66.5%	72.9%	58.4%
Brazil	Campo Alegre de Lourdes	2017	67.4%	73.7%	59.3%
Brazil	Campo Alegre do Fidalgo	2000	62.8%	68.8%	56.1%
Brazil	Campo Alegre do Fidalgo	2017	63.7%	69.5%	57.1%
Brazil	Campo Azul	2000	81.4%	86.5%	74.9%
Brazil	Campo Azul	2017	81.9%	87.0%	75.6%
Brazil	Campo Belo	2000	81.6%	85.2%	77.1%
Brazil	Campo Belo	2017	82.2%	85.7%	77.7%
Brazil	Campo Belo do Sul	2000	76.9%	81.2%	71.9%
Brazil	Campo Belo do Sul	2017	77.6%	81.8%	72.7%
Brazil	Campo Bom	2000	81.4%	83.6%	79.0%
Brazil	Campo Bom	2017	82.0%	84.1%	79.7%
Brazil	Campo Bonito	2000	84.2%	87.2%	80.4%
Brazil	Campo Bonito	2017	84.7%	87.6%	81.0%
Brazil	Campo do Brito	2000	76.5%	78.7%	74.4%

Country	Second Administrative Unit	Year	Mean	Upper	Lower
Brazil	Campo do Brito	2017	77.2%	79.3%	75.1%
Brazil	Campo do Meio	2000	82.7%	86.3%	78.1%
Brazil	Campo do Meio	2017	83.3%	86.8%	78.7%
Brazil	Campo do Tenente	2000	80.8%	84.1%	77.3%
Brazil	Campo do Tenente	2017	81.4%	84.6%	77.9%
Brazil	Campo Erê	2000	81.0%	85.2%	76.4%
Brazil	Campo Erê	2017	81.6%	85.7%	77.1%
Brazil	Campo Florido	2000	86.8%	90.1%	82.7%
Brazil	Campo Florido	2017	87.3%	90.4%	83.3%
Brazil	Campo Formoso	2000	76.0%	80.7%	70.3%
Brazil	Campo Formoso	2017	76.6%	81.3%	71.1%
Brazil	Campo Grande	2000	66.1%	69.0%	62.9%
Brazil	Campo Grande	2000	81.1%	82.9%	78.9%
Brazil	Campo Grande	2017	81.7%	83.5%	79.6%
Brazil	Campo Grande	2017	66.9%	69.8%	63.8%
Brazil	Campo Grande do Piauí	2000	63.5%	67.7%	59.3%
Brazil	Campo Grande do Piauí	2017	64.4%	68.6%	60.2%
Brazil	Campo Largo do Piauí	2000	59.9%	64.7%	55.4%
Brazil	Campo Largo do Piauí	2017	60.8%	65.6%	56.4%
Brazil	Campo Limpo Paulista	2000	93.8%	94.8%	92.6%
Brazil	Campo Limpo Paulista	2017	94.2%	95.1%	93.1%
Brazil	Campo Magro	2000	84.1%	86.1%	81.8%
Brazil	Campo Magro	2017	84.6%	86.6%	82.3%
Brazil	Campo Maior	2000	60.2%	64.4%	55.9%
Brazil	Campo Maior	2017	61.2%	65.3%	56.9%
Brazil	Campo Mourão	2000	85.3%	88.1%	81.8%
Brazil	Campo Mourão	2017	85.8%	88.5%	82.3%
Brazil	Campo Novo	2000	82.7%	86.4%	78.5%
Brazil	Campo Novo	2017	82.1%	85.9%	77.5%
Brazil	Campo Novo de Rondônia	2000	28.8%	33.2%	23.8%
Brazil	Campo Novo de Rondônia	2017	29.6%	34.1%	24.6%
Brazil	Campo Novo do Parecis	2000	68.2%	75.1%	60.7%
Brazil	Campo Novo do Parecis	2017	69.0%	75.8%	61.6%
Brazil	Campo Real	2000	82.7%	86.3%	78.5%
Brazil	Campo Real	2017	83.3%	86.8%	79.2%

Country	Second Administrative Unit	Year	Mean	Upper	Lower
Brazil	Campo Redondo	2000	80.6%	83.6%	77.7%
Brazil	Campo Redondo	2017	81.2%	84.1%	78.4%
Brazil	Campo Verde	2000	69.5%	74.8%	63.6%
Brazil	Campo Verde	2017	70.3%	75.6%	64.5%
Brazil	Campos	2000	76.3%	79.6%	72.2%
Brazil	Campos	2017	77.0%	80.2%	73.0%
Brazil	Campos Altos	2000	82.0%	87.2%	76.1%
Brazil	Campos Altos	2017	82.6%	87.6%	76.8%
Brazil	Campos Belos	2000	77.7%	82.6%	71.6%
Brazil	Campos Belos	2017	78.3%	83.2%	72.3%
Brazil	Campos Borges	2000	82.8%	86.5%	78.2%
Brazil	Campos Borges	2017	83.3%	86.9%	78.9%
Brazil	Campos de Júlio	2000	57.4%	66.3%	49.9%
Brazil	Campos de Júlio	2017	58.3%	67.2%	50.8%
Brazil	Campos do Jordão	2000	94.2%	95.1%	93.1%
Brazil	Campos do Jordão	2017	94.4%	95.3%	93.4%
Brazil	Campos Gerais	2000	83.4%	86.9%	79.1%
Brazil	Campos Gerais	2017	83.9%	87.3%	79.7%
Brazil	Campos Lindos	2000	72.8%	78.5%	66.2%
Brazil	Campos Lindos	2017	73.6%	79.2%	67.0%
Brazil	Campos Novos	2000	76.2%	80.2%	71.9%
Brazil	Campos Novos	2017	76.9%	80.8%	72.7%
Brazil	Campos Novos Paulista	2000	91.6%	93.8%	88.9%
Brazil	Campos Novos Paulista	2017	91.9%	94.1%	89.3%
Brazil	Campos Sales	2000	68.3%	72.7%	63.9%
Brazil	Campos Sales	2017	69.1%	73.4%	64.8%
Brazil	Campos Verdes	2000	76.7%	82.2%	70.5%
Brazil	Campos Verdes	2017	77.3%	82.7%	71.3%
Brazil	Campos Verdes de Goiás	2000	81.3%	84.7%	76.5%
Brazil	Campos Verdes de Goiás	2017	81.9%	85.2%	77.2%
Brazil	Camutanga	2000	70.4%	73.3%	67.7%
Brazil	Camutanga	2017	71.2%	74.0%	68.6%
Brazil	Canaã	2000	82.1%	85.4%	78.0%
Brazil	Canaã	2017	82.6%	85.9%	78.7%
Brazil	Canaã dos Carajás	2000	46.2%	52.3%	40.1%
Brazil	Canaã dos Carajás	2017	47.3%	53.4%	41.1%

Country	Second Administrative Unit	Year	Mean	Upper	Lower
Brazil	CanaBrava do Norte	2000	67.8%	76.7%	56.9%
Brazil	CanaBrava do Norte	2017	68.6%	77.4%	57.8%
Brazil	Cananéia	2000	86.4%	90.2%	81.9%
Brazil	Cananéia	2017	87.2%	90.9%	82.9%
Brazil	Canapi	2000	63.3%	67.0%	58.9%
Brazil	Canapi	2017	64.2%	67.8%	59.9%
Brazil	Canápolis	2000	83.2%	87.2%	79.3%
Brazil	Canápolis	2000	77.2%	84.6%	69.5%
Brazil	Canápolis	2017	83.7%	87.6%	79.9%
Brazil	Canápolis	2017	77.8%	85.1%	70.3%
Brazil	Canarana	2000	77.5%	83.0%	71.6%
Brazil	Canarana	2000	72.2%	79.9%	64.4%
Brazil	Canarana	2017	73.0%	80.5%	65.3%
Brazil	Canarana	2017	78.1%	83.5%	72.3%
Brazil	Canas	2000	88.0%	90.3%	85.4%
Brazil	Canas	2017	88.3%	90.6%	85.8%
Brazil	Canavieira	2000	59.6%	65.8%	52.0%
Brazil	Canavieira	2017	60.6%	66.7%	53.1%
Brazil	Canavieiras	2000	75.5%	81.3%	68.6%
Brazil	Canavieiras	2017	74.7%	80.7%	67.6%
Brazil	Candeal	2000	79.0%	82.6%	75.1%
Brazil	Candeal	2017	79.6%	83.1%	75.8%
Brazil	Candeias	2000	73.8%	76.8%	70.9%
Brazil	Candeias	2000	81.5%	85.3%	76.9%
Brazil	Candeias	2017	74.5%	77.5%	71.7%
Brazil	Candeias	2017	82.1%	85.7%	77.6%
Brazil	Candeias do Jamari	2000	27.6%	30.3%	24.7%
Brazil	Candeias do Jamari	2017	28.4%	31.2%	25.5%
Brazil	Candelária	2000	80.5%	84.1%	76.1%
Brazil	Candelária	2017	81.2%	84.6%	76.8%
Brazil	Candiba	2000	78.0%	83.1%	72.0%
Brazil	Candiba	2017	78.6%	83.7%	72.8%
Brazil	Cândido de Abreu	2000	82.8%	86.6%	78.3%
Brazil	Cândido de Abreu	2017	83.3%	87.1%	79.0%
Brazil	Cândido Godói	2000	82.0%	86.5%	76.5%
Brazil	Cândido Godói	2017	82.6%	87.0%	77.2%
Brazil	Cândido Mendes	2000	45.6%	53.7%	38.7%
Brazil	Cândido Mendes	2017	46.6%	54.6%	39.6%
Brazil	Cândido Mota	2000	90.3%	92.6%	87.9%
Brazil	Cândido Mota	2017	90.6%	92.9%	88.3%
Brazil	Cândido Rodrigues	2000	93.2%	94.7%	91.1%
Brazil	Cândido Rodrigues	2017	93.5%	94.9%	91.4%
Brazil	Cândido Sales	2000	78.7%	83.4%	73.1%
Brazil	Cândido Sales	2017	79.4%	84.0%	73.8%
Brazil	Candiota	2000	81.9%	86.9%	76.1%
Brazil	Candiota	2017	82.5%	87.4%	76.8%
Brazil	Candói	2000	82.0%	85.9%	78.2%
Brazil	Candói	2017	82.6%	86.4%	78.9%
Brazil	Canela	2000	82.0%	84.8%	78.8%
Brazil	Canela	2017	82.6%	85.3%	79.5%

Country	Second Administrative Unit	Year	Mean	Upper	Lower
Brazil	Canelinha	2000	72.3%	76.4%	67.6%
Brazil	Canelinha	2017	72.9%	76.9%	68.3%
Brazil	Canguaretama	2000	75.7%	78.6%	72.9%
Brazil	Canguaretama	2017	76.4%	79.3%	73.7%
Brazil	Canguçu	2000	84.2%	87.5%	80.1%
Brazil	Canguçu	2017	84.7%	88.0%	80.7%
Brazil	Canhoba	2000	68.9%	71.8%	65.6%
Brazil	Canhoba	2017	69.7%	72.6%	66.5%
Brazil	Canhotinho	2000	65.7%	68.8%	62.5%
Brazil	Canhotinho	2017	66.6%	69.7%	63.4%
Brazil	Canindé	2000	70.9%	74.7%	66.5%
Brazil	Canindé	2017	73.0%	76.6%	68.7%
Brazil	Canindé de São Francisco	2000	66.4%	70.4%	62.0%
Brazil	Canindé de São Francisco	2017	67.2%	71.1%	62.9%
Brazil	Canitar	2000	91.4%	93.5%	88.2%
Brazil	Canitar	2017	91.7%	93.8%	88.6%
Brazil	Canoas	2000	82.2%	84.1%	80.4%
Brazil	Canoas	2017	82.7%	84.6%	81.0%
Brazil	Canoinhas	2000	79.3%	82.9%	75.5%
Brazil	Canoinhas	2017	79.9%	83.4%	76.2%
Brazil	Cansanção	2000	76.7%	81.6%	70.4%
Brazil	Cansanção	2017	77.4%	82.2%	71.2%
Brazil	Cantá	2000	85.7%	87.2%	83.9%
Brazil	Cantá	2017	86.2%	87.6%	84.4%
Brazil	Cantagalo	2000	81.6%	86.1%	76.1%
Brazil	Cantagalo	2000	82.9%	86.7%	78.9%
Brazil	Cantagalo	2000	80.0%	83.3%	76.4%
Brazil	Cantagalo	2017	82.2%	86.6%	76.8%
Brazil	Cantagalo	2017	80.6%	83.9%	77.1%
Brazil	Cantagalo	2017	83.4%	87.1%	79.6%
Brazil	Cantanhede	2000	60.0%	64.7%	55.2%
Brazil	Cantanhede	2017	61.0%	65.6%	56.2%
Brazil	Canto do Buriti	2000	61.0%	67.3%	55.3%
Brazil	Canto do Buriti	2017	62.0%	68.2%	56.2%
Brazil	Canudos	2000	73.5%	79.0%	66.7%
Brazil	Canudos	2017	74.3%	79.7%	67.5%
Brazil	Canutama	2000	51.7%	59.2%	43.3%
Brazil	Canutama	2017	52.5%	59.8%	44.2%
Brazil	Capanema	2000	37.8%	42.6%	33.8%
Brazil	Capanema	2000	82.8%	86.7%	79.0%
Brazil	Capanema	2017	38.7%	43.5%	34.7%
Brazil	Capanema	2017	83.3%	87.1%	79.6%
Brazil	Capão Alto	2000	77.9%	82.3%	72.5%
Brazil	Capão Alto	2017	78.5%	82.9%	73.3%
Brazil	Capão Bonito	2000	92.9%	94.8%	90.5%
Brazil	Capão Bonito	2017	93.2%	95.0%	90.8%
Brazil	Capão da Canoa	2000	80.7%	82.6%	78.5%
Brazil	Capão da Canoa	2017	81.3%	83.2%	79.3%
Brazil	Capão do Leão	2000	78.1%	82.5%	73.1%
Brazil	Capão do Leão	2017	78.8%	83.1%	73.9%
Brazil	Caparaó	2000	80.9%	84.3%	77.5%
Brazil	Caparaó	2017	81.5%	84.8%	78.3%
Brazil	Capela	2000	58.6%	61.5%	55.7%
Brazil	Capela	2000	74.0%	76.1%	70.9%

Country	Second Administrative Unit	Year	Mean	Upper	Lower
Brazil	Capela	2017	59.7%	62.6%	56.8%
Brazil	Capela	2017	74.7%	76.8%	71.7%
Brazil	Capela de Santana	2000	75.0%	80.2%	68.9%
Brazil	Capela de Santana	2017	75.7%	80.8%	69.8%
Brazil	Capela do Alto	2000	94.0%	95.3%	92.4%
Brazil	Capela do Alto	2017	94.2%	95.5%	92.6%
Brazil	Capela do Alto Alegre	2000	77.9%	81.8%	72.9%
Brazil	Capela do Alto Alegre	2017	78.6%	82.4%	73.7%
Brazil	Capela Nova	2000	80.9%	84.3%	76.7%
Brazil	Capela Nova	2017	81.5%	84.8%	77.4%
Brazil	Capelinha	2000	83.5%	87.8%	77.5%
Brazil	Capelinha	2017	84.0%	88.3%	78.2%
Brazil	Capetinga	2000	87.7%	91.1%	83.4%
Brazil	Capetinga	2017	87.9%	91.3%	83.7%
Brazil	Capim	2000	69.2%	72.3%	66.2%
Brazil	Capim	2017	69.8%	72.9%	66.8%
Brazil	Capim Branco	2000	81.7%	84.7%	78.4%
Brazil	Capim Branco	2017	82.3%	85.2%	79.1%
Brazil	Capim Grosso	2000	78.6%	82.8%	73.9%
Brazil	Capim Grosso	2017	79.2%	83.4%	74.7%
Brazil	Capinópolis	2000	82.7%	86.6%	78.5%
Brazil	Capinópolis	2017	83.2%	87.0%	79.1%
Brazil	Capinzal	2000	77.0%	80.8%	72.7%
Brazil	Capinzal	2017	77.7%	81.4%	73.4%
Brazil	Capinzal do Norte	2000	60.6%	65.9%	55.0%
Brazil	Capinzal do Norte	2017	61.6%	66.7%	56.0%
Brazil	Capistrano	2000	71.5%	75.4%	67.9%
Brazil	Capistrano	2017	72.2%	76.1%	68.7%
Brazil	Capitão	2000	82.8%	86.4%	78.3%
Brazil	Capitão	2017	83.4%	86.8%	79.0%
Brazil	Capitão Andrade	2000	81.2%	85.7%	75.7%
Brazil	Capitão Andrade	2017	81.8%	86.2%	76.4%
Brazil	Capitão de Campos	2000	61.1%	65.6%	55.9%
Brazil	Capitão de Campos	2017	62.1%	66.6%	56.9%
Brazil	Capitão Enéas	2000	81.1%	84.6%	77.4%
Brazil	Capitão Enéas	2017	81.7%	85.0%	78.1%
Brazil	Capitão Gervásio Oliveira	2000	63.1%	69.3%	56.1%
Brazil	Capitão Gervásio Oliveira	2017	64.1%	70.1%	57.1%
Brazil	Capitão Leônidas Marques	2000	82.3%	85.7%	78.7%
Brazil	Capitão Leônidas Marques	2017	82.8%	86.2%	79.3%
Brazil	Capitão Poço	2000	40.7%	45.6%	36.3%
Brazil	Capitão Poço	2017	41.6%	46.6%	37.3%

Country	Second Administrative Unit	Year	Mean	Upper	Lower
Brazil	Capitólio	2000	82.4%	86.7%	76.9%
Brazil	Capitólio	2017	83.0%	87.1%	77.6%
Brazil	Capivari	2000	93.3%	94.4%	91.7%
Brazil	Capivari	2017	93.5%	94.6%	92.0%
Brazil	Capivari de Baixo	2000	73.5%	78.2%	68.0%
Brazil	Capivari de Baixo	2017	74.8%	79.3%	69.4%
Brazil	Capivari do Sul	2000	81.9%	85.6%	76.8%
Brazil	Capivari do Sul	2017	82.5%	86.1%	77.5%
Brazil	Capixaba	2000	39.1%	43.3%	34.8%
Brazil	Capixaba	2017	40.0%	44.2%	35.7%
Brazil	Capoeiras	2000	66.5%	69.9%	63.2%
Brazil	Capoeiras	2017	67.3%	70.7%	64.1%
Brazil	Caputira	2000	83.0%	86.2%	79.2%
Brazil	Caputira	2017	83.5%	86.6%	79.8%
Brazil	Caraá	2000	81.4%	84.7%	77.1%
Brazil	Caraá	2017	82.0%	85.2%	77.8%
Brazil	Caracaraí	2000	85.2%	87.9%	82.2%
Brazil	Caracaraí	2017	85.7%	88.3%	82.8%
Brazil	Caracol	2000	80.0%	85.9%	72.5%
Brazil	Caracol	2000	65.5%	71.8%	57.8%
Brazil	Caracol	2017	80.6%	86.4%	73.2%
Brazil	Caracol	2017	66.3%	72.6%	58.8%
Brazil	Caraguatatuba	2000	89.7%	92.4%	86.8%
Brazil	Caraguatatuba	2017	90.1%	92.7%	87.3%
Brazil	Caraí	2000	83.4%	87.8%	77.7%
Brazil	Caraí	2017	83.9%	88.2%	78.4%
Brazil	Caraíbas	2000	77.5%	82.7%	71.8%
Brazil	Caraíbas	2017	78.1%	83.1%	72.5%
Brazil	Carambeí	2000	85.1%	88.1%	81.9%
Brazil	Carambeí	2017	85.6%	88.5%	82.5%
Brazil	Caranaíba	2000	81.5%	85.1%	77.4%
Brazil	Caranaíba	2017	82.2%	85.6%	78.2%
Brazil	Carandaí	2000	82.7%	86.2%	78.8%
Brazil	Carandaí	2017	83.3%	86.6%	79.4%
Brazil	Carangola	2000	80.5%	83.8%	76.9%
Brazil	Carangola	2017	81.1%	84.3%	77.6%
Brazil	Carapicuíba	2000	94.3%	95.2%	93.5%
Brazil	Carapicuíba	2017	94.5%	95.3%	93.7%
Brazil	Caratinga	2000	82.7%	85.8%	78.9%
Brazil	Caratinga	2017	83.2%	86.3%	79.5%
Brazil	Carauari	2000	61.9%	70.4%	52.7%
Brazil	Carauari	2017	62.8%	71.2%	53.7%
Brazil	Caraúbas	2000	67.0%	71.5%	61.9%
Brazil	Caraúbas	2000	79.8%	82.8%	76.3%
Brazil	Caraúbas	2017	67.9%	72.3%	62.9%
Brazil	Caraúbas	2017	80.5%	83.4%	77.0%
Brazil	Caraúbas do Piauí	2000	60.4%	65.1%	55.9%
Brazil	Caraúbas do Piauí	2017	61.4%	66.0%	57.0%
Brazil	Caravalhopolis	2000	85.2%	88.1%	81.0%
Brazil	Caravalhopolis	2017	85.7%	88.5%	81.6%
Brazil	Caravelas	2000	75.6%	81.3%	69.6%
Brazil	Caravelas	2017	76.3%	81.9%	70.3%
Brazil	Carazinho	2000	82.3%	85.7%	78.9%
Brazil	Carazinho	2017	82.9%	86.2%	79.5%
Brazil	Carbonita	2000	82.0%	87.0%	76.4%
Brazil	Carbonita	2017	82.6%	87.4%	77.1%

Country	Second Administrative Unit	Year	Mean	Upper	Lower
Brazil	Cardeal da Silva	2000	76.5%	81.0%	72.1%
Brazil	Cardeal da Silva	2017	77.2%	81.6%	72.9%
Brazil	Cardoso	2000	88.9%	92.0%	85.5%
Brazil	Cardoso	2017	89.3%	92.2%	86.0%
Brazil	Cardoso Moreira	2000	76.3%	79.6%	72.3%
Brazil	Cardoso Moreira	2017	77.0%	80.2%	73.0%
Brazil	Careaçu	2000	86.7%	89.6%	83.4%
Brazil	Careaçu	2017	87.2%	90.0%	84.0%
Brazil	Careiro	2000	56.7%	60.6%	52.3%
Brazil	Careiro	2017	57.7%	61.5%	53.3%
Brazil	Careiro da Várzea	2000	54.9%	58.5%	51.6%
Brazil	Careiro da Várzea	2017	55.9%	59.5%	52.6%
Brazil	Carepebus	2000	75.5%	79.4%	71.0%
Brazil	Carepebus	2017	76.2%	80.1%	71.8%
Brazil	Cariacica	2000	76.1%	78.2%	73.8%
Brazil	Cariacica	2017	77.1%	79.1%	74.9%
Brazil	Caridade	2000	70.8%	74.6%	67.0%
Brazil	Caridade	2017	71.6%	75.3%	67.8%
Brazil	Caridade do Piauí	2000	63.2%	68.4%	57.4%
Brazil	Caridade do Piauí	2017	64.1%	69.2%	58.4%
Brazil	Carinhanha	2000	78.4%	84.3%	70.6%
Brazil	Carinhanha	2017	79.1%	84.8%	71.4%
Brazil	Carira	2000	76.7%	79.8%	73.5%
Brazil	Carira	2017	77.4%	80.4%	74.2%
Brazil	Cariré	2000	67.0%	70.9%	62.5%
Brazil	Cariré	2017	67.8%	71.6%	63.4%
Brazil	Cariri do Tocantins	2000	76.6%	81.3%	72.4%
Brazil	Cariri do Tocantins	2017	77.3%	81.9%	73.1%
Brazil	Caririaçú	2000	68.6%	72.3%	64.8%
Brazil	Caririaçú	2017	69.4%	73.1%	65.7%
Brazil	Cariús	2000	68.7%	72.4%	64.3%
Brazil	Cariús	2017	69.5%	73.2%	65.1%
Brazil	Carlinda	2000	64.5%	71.1%	56.4%
Brazil	Carlinda	2017	65.4%	71.9%	57.3%
Brazil	Carlópolis	2000	89.5%	91.9%	86.0%
Brazil	Carlópolis	2017	89.8%	92.2%	86.5%
Brazil	Carlos Barbosa	2000	84.0%	86.6%	81.4%
Brazil	Carlos Barbosa	2017	84.5%	87.0%	82.0%
Brazil	Carlos Chagas	2000	79.4%	83.8%	74.3%
Brazil	Carlos Chagas	2017	80.1%	84.4%	75.0%
Brazil	Carlos Gomes	2000	77.5%	81.4%	73.3%
Brazil	Carlos Gomes	2017	78.1%	82.0%	74.0%
Brazil	Carmésia	2000	81.0%	85.0%	75.7%
Brazil	Carmésia	2017	81.7%	85.5%	76.4%
Brazil	Carmo	2000	80.4%	83.7%	76.7%
Brazil	Carmo	2017	81.1%	84.3%	77.5%
Brazil	Carmo da Cachoeira	2000	83.2%	86.7%	79.3%
Brazil	Carmo da Cachoeira	2017	83.7%	87.2%	79.9%

Country	Second Administrative Unit	Year	Mean	Upper	Lower
Brazil	Carmo da Mata	2000	80.6%	84.8%	75.6%
Brazil	Carmo da Mata	2017	81.2%	85.3%	76.3%
Brazil	Carmo de Minas	2000	85.2%	88.5%	81.8%
Brazil	Carmo de Minas	2017	85.7%	88.9%	82.4%
Brazil	Carmo do Cajuru	2000	80.3%	84.5%	76.0%
Brazil	Carmo do Cajuru	2017	80.9%	85.0%	76.7%
Brazil	Carmo do Paranaiba	2000	82.6%	88.0%	76.1%
Brazil	Carmo do Paranaiba	2017	83.2%	88.4%	76.8%
Brazil	Carmo do Rio Claro	2000	83.9%	87.3%	79.3%
Brazil	Carmo do Rio Claro	2017	84.2%	87.6%	79.7%
Brazil	Carmo do Rio Verde	2000	77.1%	81.2%	73.0%
Brazil	Carmo do Rio Verde	2017	77.8%	81.8%	73.7%
Brazil	Carmolândia	2000	72.6%	76.3%	68.8%
Brazil	Carmolândia	2017	73.4%	77.0%	69.6%
Brazil	Carmópolis	2000	72.2%	74.6%	69.5%
Brazil	Carmópolis	2017	73.0%	75.4%	70.3%
Brazil	Carmópolis de Minas	2000	81.3%	85.3%	77.2%
Brazil	Carmópolis de Minas	2017	81.9%	85.7%	77.8%
Brazil	Carnaíba	2000	66.5%	71.0%	62.4%
Brazil	Carnaíba	2017	67.4%	71.8%	63.4%
Brazil	Carnaúba dos Dantas	2000	79.5%	82.5%	75.4%
Brazil	Carnaúba dos Dantas	2017	80.1%	83.1%	76.2%
Brazil	Carnaubais	2000	82.6%	86.0%	79.4%
Brazil	Carnaubais	2017	83.2%	86.5%	80.1%
Brazil	Carnaubal	2000	71.1%	75.0%	67.2%
Brazil	Carnaubal	2017	72.0%	75.8%	68.1%
Brazil	Carnaubeira da Penha	2000	66.8%	71.8%	61.1%
Brazil	Carnaubeira da Penha	2017	67.6%	72.6%	62.0%
Brazil	Carneirinho	2000	84.2%	88.4%	79.2%
Brazil	Carneirinho	2017	84.7%	88.8%	79.9%
Brazil	Carneiros	2000	63.1%	66.5%	59.9%
Brazil	Carneiros	2017	64.0%	67.3%	60.8%
Brazil	Caroebe	2000	83.1%	86.8%	78.2%
Brazil	Caroebe	2017	83.7%	87.3%	78.9%
Brazil	Carolina	2000	72.6%	77.7%	67.8%
Brazil	Carolina	2017	73.4%	78.4%	68.7%
Brazil	Carpina	2000	71.2%	74.0%	69.0%
Brazil	Carpina	2017	72.2%	74.9%	70.0%
Brazil	Carrancas	2000	83.5%	87.0%	79.4%
Brazil	Carrancas	2017	84.0%	87.4%	79.9%
Brazil	Carrapateira	2000	70.5%	73.9%	66.3%
Brazil	Carrapateira	2017	71.3%	74.7%	67.2%
Brazil	Carrasco Bonito	2000	62.7%	67.3%	58.1%

Country	Second Administrative Unit	Year	Mean	Upper	Lower
Brazil	Carrasco Bonito	2017	63.6%	68.1%	59.0%
Brazil	Caruaru	2000	68.7%	71.4%	65.8%
Brazil	Caruaru	2017	69.5%	72.2%	66.6%
Brazil	Carutapera	2000	47.5%	54.6%	41.0%
Brazil	Carutapera	2017	48.4%	55.6%	42.0%
Brazil	Carvalhos	2000	85.1%	88.0%	81.6%
Brazil	Carvalhos	2017	85.6%	88.4%	82.2%
Brazil	Casa Branca	2000	93.0%	94.5%	90.9%
Brazil	Casa Branca	2017	93.2%	94.7%	91.2%
Brazil	Casa Grande	2000	81.2%	84.9%	77.1%
Brazil	Casa Grande	2017	81.8%	85.4%	77.8%
Brazil	Casa Nova	2000	67.6%	72.4%	62.8%
Brazil	Casa Nova	2017	68.5%	73.2%	63.7%
Brazil	Casca	2000	80.9%	84.4%	76.5%
Brazil	Casca	2017	81.5%	84.9%	77.2%
Brazil	Cascalho Rico	2000	79.2%	84.0%	73.9%
Brazil	Cascalho Rico	2017	79.9%	84.5%	74.7%
Brazil	Cascavel	2000	69.2%	72.3%	65.7%
Brazil	Cascavel	2000	86.1%	88.7%	82.9%
Brazil	Cascavel	2017	86.6%	89.1%	83.5%
Brazil	Cascavel	2017	70.0%	73.1%	66.6%
Brazil	Caseara	2000	59.4%	66.7%	51.5%
Brazil	Caseara	2017	60.3%	67.5%	52.4%
Brazil	Caseiros	2000	79.8%	84.1%	75.5%
Brazil	Caseiros	2017	80.4%	84.6%	76.2%
Brazil	Casimiro de Abreu	2000	77.1%	80.4%	73.6%
Brazil	Casimiro de Abreu	2017	78.0%	81.3%	74.5%
Brazil	Casinhas	2000	63.7%	67.2%	60.6%
Brazil	Casinhas	2017	64.6%	68.1%	61.5%
Brazil	Casserengue	2000	73.0%	76.0%	69.5%
Brazil	Casserengue	2017	73.7%	76.7%	70.4%
Brazil	Cássia	2000	86.5%	90.1%	82.0%
Brazil	Cássia	2017	86.9%	90.5%	82.6%
Brazil	Cássia dos Coqueiros	2000	89.7%	92.3%	86.5%
Brazil	Cássia dos Coqueiros	2017	90.0%	92.6%	86.9%
Brazil	Cassilândia	2000	80.1%	84.8%	74.6%
Brazil	Cassilândia	2017	80.7%	85.3%	75.4%
Brazil	Cassiterita	2000	80.3%	84.5%	75.7%
Brazil	Cassiterita	2017	80.9%	85.0%	76.4%
Brazil	Castanhal	2000	38.3%	41.6%	34.8%
Brazil	Castanhal	2017	39.2%	42.6%	35.7%
Brazil	Castanheira	2000	62.6%	71.8%	54.9%
Brazil	Castanheira	2017	63.5%	72.6%	55.8%
Brazil	Castanheiras	2000	27.1%	30.2%	23.2%
Brazil	Castanheiras	2017	27.9%	31.0%	23.8%
Brazil	Castelândia	2000	77.5%	82.0%	72.5%
Brazil	Castelândia	2017	78.2%	82.5%	73.3%
Brazil	Castelo	2000	77.6%	80.8%	74.0%
Brazil	Castelo	2017	78.3%	81.4%	74.8%
Brazil	Castelo do Piauí	2000	62.2%	66.8%	57.2%
Brazil	Castelo do Piauí	2017	63.1%	67.7%	58.1%
Brazil	Castilho	2000	86.3%	89.0%	82.9%
Brazil	Castilho	2017	86.8%	89.3%	83.5%
Brazil	Castro	2000	85.8%	88.7%	82.3%
Brazil	Castro	2017	86.2%	89.0%	82.9%

Country	Second Administrative Unit	Year	Mean	Upper	Lower
Brazil	Castro Alves	2000	79.1%	82.7%	74.3%
Brazil	Castro Alves	2017	79.7%	83.3%	75.1%
Brazil	Cataguases	2000	80.1%	83.6%	76.3%
Brazil	Cataguases	2017	80.7%	84.2%	77.0%
Brazil	Catalão	2000	79.4%	84.0%	73.8%
Brazil	Catalão	2017	80.0%	84.5%	74.6%
Brazil	Catanduva	2000	93.5%	95.0%	91.5%
Brazil	Catanduva	2017	93.9%	95.4%	92.0%
Brazil	Catanduvas	2000	77.7%	81.6%	73.3%
Brazil	Catanduvas	2000	84.6%	87.6%	80.8%
Brazil	Catanduvas	2017	78.4%	82.2%	74.1%
Brazil	Catanduvas	2017	84.6%	87.6%	80.8%
Brazil	Catarina	2000	71.1%	75.4%	66.3%
Brazil	Catarina	2017	71.9%	76.1%	67.2%
Brazil	Catas Altas	2000	80.8%	84.7%	77.5%
Brazil	Catas Altas	2017	81.4%	85.2%	78.1%
Brazil	Catas Altas da Noruega	2000	80.6%	84.2%	76.4%
Brazil	Catas Altas da Noruega	2017	81.2%	84.7%	77.1%
Brazil	Catende	2000	62.8%	66.6%	59.3%
Brazil	Catende	2017	63.8%	67.5%	60.3%
Brazil	Catigua	2000	92.7%	94.6%	90.5%
Brazil	Catigua	2017	93.0%	94.8%	90.9%
Brazil	Catingueira	2000	66.8%	70.3%	62.6%
Brazil	Catingueira	2017	67.7%	71.1%	63.5%
Brazil	Catolândia	2000	77.1%	83.4%	69.3%
Brazil	Catolândia	2017	77.6%	83.9%	69.9%
Brazil	Catolé do Rocha	2000	75.9%	79.5%	72.8%
Brazil	Catolé do Rocha	2017	76.6%	80.1%	73.6%
Brazil	Catu	2000	76.7%	80.0%	73.3%
Brazil	Catu	2017	77.4%	80.7%	74.0%
Brazil	Catuípe	2000	82.6%	86.3%	78.2%
Brazil	Catuípe	2017	83.2%	86.8%	78.9%
Brazil	Catuji	2000	82.6%	87.6%	77.4%
Brazil	Catuji	2017	83.1%	88.0%	78.1%
Brazil	Catunda	2000	69.2%	73.5%	64.8%
Brazil	Catunda	2017	70.0%	74.3%	65.7%
Brazil	Caturaí	2000	77.0%	79.7%	74.4%
Brazil	Caturaí	2017	77.7%	80.3%	75.1%
Brazil	Caturama	2000	77.0%	82.9%	70.6%
Brazil	Caturama	2017	77.7%	83.5%	71.4%
Brazil	Caturité	2000	69.8%	73.2%	66.8%
Brazil	Caturité	2017	70.7%	74.0%	67.7%
Brazil	Catuti	2000	80.2%	85.6%	73.9%
Brazil	Catuti	2017	80.8%	86.0%	74.7%
Brazil	Caucaia	2000	68.2%	70.3%	66.2%
Brazil	Caucaia	2017	69.1%	71.1%	67.1%
Brazil	Cavalcante	2000	80.6%	85.5%	75.8%
Brazil	Cavalcante	2017	81.1%	85.9%	76.4%
Brazil	Caxambu	2000	83.8%	87.2%	79.9%
Brazil	Caxambu	2017	84.4%	87.6%	80.5%
Brazil	Caxambu do Sul	2000	76.7%	80.2%	72.4%
Brazil	Caxambu do Sul	2017	77.3%	80.8%	73.2%
Brazil	Caxias	2000	61.5%	65.5%	57.2%
Brazil	Caxias	2017	62.5%	66.4%	58.2%
Brazil	Caxias do Sul	2000	83.5%	85.8%	80.5%
Brazil	Caxias do Sul	2017	84.0%	86.3%	81.1%

Country	Second Administrative Unit	Year	Mean	Upper	Lower
Brazil	Caxingó	2000	60.2%	64.6%	55.9%
Brazil	Caxingó	2017	61.1%	65.5%	56.8%
Brazil	Ceará-Mirim	2000	82.6%	84.6%	80.3%
Brazil	Ceará-Mirim	2017	83.2%	85.1%	80.9%
Brazil	Cedral	2000	92.4%	94.0%	90.2%
Brazil	Cedral	2000	58.2%	65.1%	51.6%
Brazil	Cedral	2017	59.1%	65.9%	52.6%
Brazil	Cedral	2017	92.6%	94.3%	90.5%
Brazil	Cedro	2000	70.7%	74.4%	66.4%
Brazil	Cedro	2000	70.7%	74.4%	66.8%
Brazil	Cedro	2017	71.5%	75.1%	67.6%
Brazil	Cedro	2017	71.6%	75.2%	67.3%
Brazil	Cedro de São João	2000	70.1%	73.1%	67.4%
Brazil	Cedro de São João	2017	70.9%	73.8%	68.3%
Brazil	Cedro do Abaeté	2000	80.0%	85.5%	72.8%
Brazil	Cedro do Abaeté	2017	80.6%	86.0%	73.6%
Brazil	Celso Ramos	2000	76.4%	80.7%	71.8%
Brazil	Celso Ramos	2017	77.1%	81.4%	72.6%
Brazil	Centenário	2000	78.2%	82.2%	74.1%
Brazil	Centenário	2000	74.9%	80.9%	68.3%
Brazil	Centenário	2017	78.9%	82.7%	74.9%
Brazil	Centenário	2017	75.6%	81.5%	69.1%
Brazil	Centenário do Sul	2000	86.0%	88.6%	82.8%
Brazil	Centenário do Sul	2017	86.5%	89.0%	83.3%
Brazil	Central	2000	76.5%	82.5%	68.9%
Brazil	Central	2017	77.2%	83.0%	69.8%
Brazil	Central de Minas	2000	80.0%	84.2%	75.6%
Brazil	Central de Minas	2017	80.6%	84.7%	76.4%
Brazil	Central do Maranhão	2000	59.9%	65.4%	53.6%
Brazil	Central do Maranhão	2017	60.8%	66.2%	54.5%
Brazil	Centralina	2000	83.7%	87.5%	79.7%
Brazil	Centralina	2017	84.2%	87.9%	80.3%
Brazil	Centro do Guilherme	2000	51.4%	57.7%	46.1%
Brazil	Centro do Guilherme	2017	52.4%	58.7%	47.1%
Brazil	Centro Novo do Maranhão	2000	49.2%	54.8%	44.2%
Brazil	Centro Novo do Maranhão	2017	50.1%	55.7%	45.0%
Brazil	Cerejeiras	2000	30.1%	35.6%	25.5%
Brazil	Cerejeiras	2017	30.9%	36.5%	26.2%
Brazil	Ceres	2000	77.4%	81.7%	73.3%
Brazil	Ceres	2017	78.1%	82.3%	74.1%
Brazil	Cerqueira César	2000	93.0%	94.9%	90.4%
Brazil	Cerqueira César	2017	93.2%	95.1%	90.7%
Brazil	Cerquilho	2000	93.2%	94.6%	91.6%
Brazil	Cerquilho	2017	93.5%	94.8%	91.9%
Brazil	Cerrito	2000	80.9%	85.1%	75.8%
Brazil	Cerrito	2017	81.5%	85.6%	76.5%

Country	Second Administrative Unit	Year	Mean	Upper	Lower
Brazil	Cêrro Azul	2000	85.7%	88.9%	82.4%
Brazil	Cêrro Azul	2017	86.4%	89.5%	83.2%
Brazil	Cerro Branco	2000	82.0%	85.6%	77.8%
Brazil	Cerro Branco	2017	82.5%	86.0%	78.4%
Brazil	Cerro Corá	2000	84.2%	86.9%	81.3%
Brazil	Cerro Corá	2017	84.7%	87.3%	81.9%
Brazil	Cerro Grande	2000	78.6%	82.5%	74.6%
Brazil	Cerro Grande	2017	79.2%	83.1%	75.4%
Brazil	Cerro Grande do Sul	2000	82.3%	85.7%	78.2%
Brazil	Cerro Grande do Sul	2017	82.9%	86.2%	78.8%
Brazil	Cerro Largo	2000	80.8%	85.7%	75.5%
Brazil	Cerro Largo	2017	81.4%	86.1%	76.2%
Brazil	Cerro Negro	2000	76.4%	80.9%	71.2%
Brazil	Cerro Negro	2017	77.1%	81.5%	72.1%
Brazil	Cesário Lange	2000	93.4%	94.9%	91.4%
Brazil	Cesário Lange	2017	93.7%	95.1%	91.8%
Brazil	Céu Azul	2000	84.5%	87.6%	81.1%
Brazil	Céu Azul	2017	85.0%	88.0%	81.7%
Brazil	Cezarina	2000	75.8%	79.3%	72.3%
Brazil	Cezarina	2017	76.5%	80.0%	73.1%
Brazil	Chã de Alegria	2000	65.9%	68.7%	63.5%
Brazil	Chã de Alegria	2017	66.8%	69.6%	64.4%
Brazil	Chã Grande	2000	71.6%	74.7%	68.7%
Brazil	Chã Grande	2017	72.4%	75.4%	69.5%
Brazil	Chã Preta	2000	59.7%	63.2%	56.3%
Brazil	Chã Preta	2017	60.6%	64.2%	57.3%
Brazil	Chácara	2000	80.6%	83.7%	76.9%
Brazil	Chácara	2017	81.2%	84.2%	77.6%
Brazil	Chale	2000	79.6%	83.2%	75.3%
Brazil	Chale	2017	80.3%	83.7%	76.0%
Brazil	Chapada	2000	80.8%	84.4%	76.6%
Brazil	Chapada	2017	81.3%	84.9%	77.3%
Brazil	Chapada da Natividade	2000	76.6%	81.3%	70.9%
Brazil	Chapada da Natividade	2017	77.3%	81.9%	71.7%
Brazil	Chapada de Areia	2000	72.7%	77.3%	67.8%
Brazil	Chapada de Areia	2017	73.5%	78.0%	68.6%
Brazil	Chapada do Norte	2000	82.4%	87.2%	77.1%
Brazil	Chapada do Norte	2017	82.9%	87.6%	77.8%
Brazil	Chapada dos Guimarães	2000	69.0%	73.6%	64.3%
Brazil	Chapada dos Guimarães	2017	69.9%	74.3%	65.2%
Brazil	Chapada Gaúcha	2000	82.9%	88.5%	77.1%
Brazil	Chapada Gaúcha	2017	83.5%	88.9%	77.8%
Brazil	Chapadão do Céu	2000	61.0%	65.9%	55.9%
Brazil	Chapadão do Céu	2017	62.0%	66.8%	56.9%
Brazil	Chapadão do Lageado	2000	74.6%	78.8%	69.8%

Country	Second Administrative Unit	Year	Mean	Upper	Lower
Brazil	Chapadão do Lageado	2017	75.3%	79.5%	70.6%
Brazil	Chapadão do Sul	2000	79.3%	84.8%	72.9%
Brazil	Chapadão do Sul	2017	80.4%	85.6%	74.2%
Brazil	Chapadinha	2000	78.6%	84.1%	72.5%
Brazil	Chapadinha	2017	79.2%	84.6%	73.3%
Brazil	Chapecó	2000	79.5%	82.9%	75.7%
Brazil	Chapecó	2017	80.1%	83.5%	76.5%
Brazil	Charqueada	2000	93.5%	94.7%	92.0%
Brazil	Charqueada	2017	93.7%	94.9%	92.3%
Brazil	Charqueadas	2000	83.1%	85.8%	80.2%
Brazil	Charqueadas	2017	83.7%	86.3%	80.8%
Brazil	Charrua	2000	78.5%	82.5%	74.2%
Brazil	Charrua	2017	79.1%	83.0%	74.9%
Brazil	Chaval	2000	65.9%	71.0%	60.7%
Brazil	Chaval	2017	66.7%	71.8%	61.6%
Brazil	Chavantes	2000	92.0%	94.2%	89.2%
Brazil	Chavantes	2017	92.3%	94.4%	89.6%
Brazil	Chaves	2000	36.0%	42.9%	30.4%
Brazil	Chaves	2017	37.0%	43.9%	31.3%
Brazil	Chaveslandia	2000	81.3%	85.3%	76.5%
Brazil	Chaveslandia	2017	81.8%	85.7%	77.2%
Brazil	Chiador	2000	75.8%	79.5%	72.2%
Brazil	Chiador	2017	76.3%	79.9%	72.7%
Brazil	Chiapeta	2000	82.1%	86.1%	77.5%
Brazil	Chiapeta	2017	82.7%	86.6%	78.1%
Brazil	Chopinzinho	2000	81.6%	85.5%	77.0%
Brazil	Chopinzinho	2017	82.2%	85.9%	77.7%
Brazil	Choró	2000	71.2%	74.9%	66.5%
Brazil	Choró	2017	72.0%	75.6%	67.3%
Brazil	Chorozinho	2000	70.5%	73.8%	66.8%
Brazil	Chorozinho	2017	71.3%	74.6%	67.7%
Brazil	Chorrochó	2000	66.8%	72.9%	61.0%
Brazil	Chorrochó	2017	67.7%	73.6%	61.9%
Brazil	Chuí	2000	78.1%	87.9%	65.9%
Brazil	Chuí	2017	78.7%	88.3%	66.8%
Brazil	Chupinguaia	2000	29.4%	34.4%	24.9%
Brazil	Chupinguaia	2017	30.2%	35.4%	25.6%
Brazil	Chuvisca	2000	82.1%	86.2%	77.7%
Brazil	Chuvisca	2017	82.7%	86.7%	78.4%
Brazil	Cianorte	2000	84.8%	87.5%	81.3%
Brazil	Cianorte	2017	85.3%	87.9%	81.9%
Brazil	Cícero Dantas	2000	77.4%	81.6%	72.7%
Brazil	Cícero Dantas	2017	78.0%	82.2%	73.4%
Brazil	Cidade Gaúcha	2000	84.6%	87.5%	81.1%
Brazil	Cidade Gaúcha	2017	85.1%	87.9%	81.7%
Brazil	Cidade Ocidental	2000	89.9%	91.2%	88.5%
Brazil	Cidade Ocidental	2017	90.3%	91.5%	88.9%
Brazil	Cidelândia	2000	63.4%	67.9%	58.7%
Brazil	Cidelândia	2017	64.3%	68.7%	59.6%
Brazil	Cidreira	2000	79.3%	83.9%	74.1%
Brazil	Cidreira	2017	79.9%	84.4%	74.9%
Brazil	Cipó	2000	77.5%	81.3%	72.5%
Brazil	Cipó	2017	78.2%	81.9%	73.3%
Brazil	Cipotânea	2000	80.3%	83.7%	76.5%
Brazil	Cipotânea	2017	80.9%	84.2%	77.2%

Country	Second Administrative Unit	Year	Mean	Upper	Lower
Brazil	Ciríaco	2000	80.5%	84.0%	76.0%
Brazil	Ciríaco	2017	81.1%	84.5%	76.7%
Brazil	Claraval	2000	87.3%	90.8%	83.3%
Brazil	Claraval	2017	87.7%	91.1%	83.8%
Brazil	Claro dos Poções	2000	81.4%	86.1%	75.2%
Brazil	Claro dos Poções	2017	82.0%	86.6%	76.0%
Brazil	Cláudia	2000	67.4%	73.2%	60.6%
Brazil	Cláudia	2017	68.2%	74.0%	61.6%
Brazil	Cláudio	2000	80.9%	85.2%	76.5%
Brazil	Cláudio	2017	81.5%	85.7%	77.2%
Brazil	Clementina	2000	92.1%	94.2%	89.7%
Brazil	Clementina	2017	92.4%	94.4%	90.1%
Brazil	Clevelândia	2000	78.9%	83.3%	74.7%
Brazil	Clevelândia	2017	79.6%	83.8%	75.4%
Brazil	Coaraci	2000	79.5%	83.4%	75.0%
Brazil	Coaraci	2017	80.1%	83.9%	75.7%
Brazil	Coari	2000	65.0%	71.6%	57.4%
Brazil	Coari	2017	65.9%	72.4%	58.3%
Brazil	Cocal	2000	66.2%	70.3%	61.6%
Brazil	Cocal	2017	67.0%	71.2%	62.5%
Brazil	Cocal de Telha	2000	59.9%	64.4%	55.4%
Brazil	Cocal de Telha	2017	60.8%	65.2%	56.4%
Brazil	Cocal do Sul	2000	74.4%	78.4%	70.0%
Brazil	Cocal do Sul	2017	75.1%	79.0%	70.8%
Brazil	Cocal dos Alves	2000	66.4%	70.6%	61.9%
Brazil	Cocal dos Alves	2017	67.4%	71.4%	62.9%
Brazil	Cocalinho	2000	71.7%	77.8%	64.4%
Brazil	Cocalinho	2017	72.5%	78.5%	65.0%
Brazil	Cocalzinho de Goiás	2000	86.7%	88.6%	84.3%
Brazil	Cocalzinho de Goiás	2017	87.2%	89.1%	85.0%
Brazil	Cocos	2000	79.4%	85.7%	72.0%
Brazil	Cocos	2017	80.9%	86.9%	73.9%
Brazil	Codajás	2000	60.5%	66.4%	53.9%
Brazil	Codajás	2017	61.4%	67.2%	54.8%
Brazil	Codó	2000	61.2%	65.5%	56.5%
Brazil	Codó	2017	62.1%	66.3%	57.5%
Brazil	Coelho Neto	2000	62.5%	66.5%	57.8%
Brazil	Coelho Neto	2017	63.4%	67.4%	58.8%
Brazil	Coimbra	2000	81.8%	85.0%	77.6%
Brazil	Coimbra	2017	82.4%	85.4%	78.2%
Brazil	Coité do Nóia	2000	61.0%	63.5%	58.4%
Brazil	Coité do Nóia	2017	61.9%	64.3%	59.4%
Brazil	Coivaras	2000	59.8%	64.0%	55.4%
Brazil	Coivaras	2017	60.7%	64.9%	56.4%
Brazil	Colares	2000	35.0%	39.8%	31.1%
Brazil	Colares	2017	35.9%	40.7%	31.9%
Brazil	Colatina	2000	75.9%	78.9%	72.5%
Brazil	Colatina	2017	76.6%	79.5%	73.2%
Brazil	Colíder	2000	70.8%	76.4%	64.5%
Brazil	Colíder	2017	71.6%	77.1%	65.4%
Brazil	Colina	2000	92.1%	94.2%	89.6%
Brazil	Colina	2017	92.3%	94.4%	89.9%
Brazil	Colinas	2000	61.1%	66.9%	54.9%
Brazil	Colinas	2000	79.6%	83.4%	76.1%

Country	Second Administrative Unit	Year	Mean	Upper	Lower
Brazil	Colinas	2017	62.0%	67.7%	55.9%
Brazil	Colinas	2017	80.2%	84.0%	76.8%
Brazil	Colinas do Sul	2000	80.6%	85.6%	75.3%
Brazil	Colinas do Sul	2017	81.1%	86.0%	76.0%
Brazil	Colinas do Tocantins	2000	74.8%	79.0%	70.0%
Brazil	Colinas do Tocantins	2017	75.6%	79.7%	70.9%
Brazil	Colméia	2000	73.1%	77.4%	68.2%
Brazil	Colméia	2017	73.9%	78.1%	69.0%
Brazil	Colômbia	2000	89.5%	92.5%	86.4%
Brazil	Colômbia	2017	89.9%	92.7%	86.9%
Brazil	Colombo	2000	86.2%	88.1%	84.0%
Brazil	Colombo	2017	86.7%	88.5%	84.6%
Brazil	Colônia do Gurguéia	2000	60.1%	67.0%	53.0%
Brazil	Colônia do Gurguéia	2017	61.1%	67.9%	54.0%
Brazil	Colônia do Piauí	2000	59.6%	64.7%	53.8%
Brazil	Colônia do Piauí	2017	60.5%	65.6%	54.7%
Brazil	Colônia Leopoldina	2000	61.7%	65.2%	58.7%
Brazil	Colônia Leopoldina	2017	62.5%	66.0%	59.6%
Brazil	Colorado	2000	88.5%	90.8%	85.7%
Brazil	Colorado	2000	82.0%	85.4%	77.8%
Brazil	Colorado	2017	82.6%	85.9%	78.5%
Brazil	Colorado	2017	88.8%	91.1%	86.1%
Brazil	Colorado do Oeste	2000	33.0%	38.0%	29.2%
Brazil	Colorado do Oeste	2017	33.9%	38.9%	30.0%
Brazil	Coluna	2000	81.9%	86.6%	75.9%
Brazil	Coluna	2017	82.5%	87.0%	76.6%
Brazil	Combinado	2000	76.5%	81.6%	70.0%
Brazil	Combinado	2017	77.2%	82.2%	70.9%
Brazil	Comendador Gomes	2000	86.8%	90.2%	82.6%
Brazil	Comendador Gomes	2017	87.3%	90.5%	83.1%
Brazil	Comendador Levy Gasparian	2000	77.9%	81.2%	74.4%
Brazil	Comendador Levy Gasparian	2017	78.6%	81.8%	75.1%
Brazil	Comercinho	2000	81.6%	85.8%	75.9%
Brazil	Comercinho	2017	82.2%	86.3%	76.6%
Brazil	Comodoro	2000	46.8%	53.1%	40.2%
Brazil	Comodoro	2017	47.5%	53.7%	41.0%
Brazil	Conceição	2000	68.4%	72.5%	64.3%
Brazil	Conceição	2017	69.2%	73.3%	65.2%
Brazil	Conceição da Aparecida	2000	84.4%	88.1%	80.1%
Brazil	Conceição da Aparecida	2017	84.9%	88.5%	80.7%
Brazil	Conceição da Barra	2000	76.1%	80.8%	71.5%
Brazil	Conceição da Barra	2017	76.8%	81.4%	72.3%

Piped Water Proportion by Second Administrative Unit *(continued)*

Country	Second Administrative Unit	Year	Mean	Upper	Lower
Brazil	Conceição da Feira	2000	76.4%	79.8%	73.1%
Brazil	Conceição da Feira	2017	77.3%	80.6%	74.1%
Brazil	Conceição das Alagoas	2000	87.7%	90.6%	84.1%
Brazil	Conceição das Alagoas	2017	88.1%	91.1%	84.4%
Brazil	Conceição das Pedras	2000	87.2%	90.4%	83.2%
Brazil	Conceição das Pedras	2017	87.7%	90.8%	83.7%
Brazil	Conceição de Ipanema	2000	79.7%	83.4%	75.5%
Brazil	Conceição de Ipanema	2017	80.3%	83.9%	76.2%
Brazil	Conceicao do Almeida	2000	76.7%	80.6%	72.1%
Brazil	Conceicao do Almeida	2017	77.4%	81.3%	72.9%
Brazil	Conceição do Almeida	2000	76.7%	80.4%	72.2%
Brazil	Conceição do Almeida	2017	77.4%	81.1%	73.1%
Brazil	Conceição do Araguaia	2000	36.6%	41.0%	32.5%
Brazil	Conceição do Araguaia	2017	37.5%	41.9%	33.3%
Brazil	Conceição do Canindé	2000	59.7%	65.1%	52.9%
Brazil	Conceição do Canindé	2017	60.7%	66.0%	54.0%
Brazil	Conceição do Castelo	2000	80.6%	83.8%	77.6%
Brazil	Conceição do Castelo	2017	81.2%	84.3%	78.2%
Brazil	Conceição do Coité	2000	79.2%	82.6%	75.3%
Brazil	Conceição do Coité	2017	80.6%	83.9%	76.9%
Brazil	Conceição do Jacuípe	2000	77.7%	80.8%	74.0%
Brazil	Conceição do Jacuípe	2017	78.4%	81.4%	74.7%
Brazil	Conceição do Lago-Açu	2000	61.4%	66.1%	56.1%
Brazil	Conceição do Lago-Açu	2017	62.4%	67.1%	57.2%
Brazil	Conceição do Mato Dentro	2000	80.9%	84.9%	75.7%
Brazil	Conceição do Mato Dentro	2017	81.5%	85.4%	76.5%
Brazil	Conceição do Para	2000	79.8%	84.0%	75.2%
Brazil	Conceição do Para	2017	80.4%	84.6%	75.9%
Brazil	Conceição do Rio Verde	2000	83.8%	87.1%	79.7%
Brazil	Conceição do Rio Verde	2017	85.0%	88.1%	81.2%
Brazil	Conceição do Tocantins	2000	77.3%	82.3%	71.0%

Country	Second Administrative Unit	Year	Mean	Upper	Lower
Brazil	Conceição do Tocantins	2017	77.9%	82.9%	71.7%
Brazil	Conceição dos Ouros	2000	88.6%	90.9%	85.9%
Brazil	Conceição dos Ouros	2017	88.9%	91.2%	86.3%
Brazil	Conceicao Macabu	2000	76.8%	80.2%	73.1%
Brazil	Conceicao Macabu	2017	77.5%	80.8%	73.8%
Brazil	Conchal	2000	92.0%	93.6%	90.2%
Brazil	Conchal	2017	92.3%	93.9%	90.5%
Brazil	Conchas	2000	93.1%	94.6%	91.2%
Brazil	Conchas	2017	93.4%	94.8%	91.5%
Brazil	Concórdia	2000	78.3%	81.6%	73.8%
Brazil	Concórdia	2017	79.1%	82.2%	74.7%
Brazil	Concórdia do Pará	2000	61.8%	67.4%	55.6%
Brazil	Concórdia do Pará	2017	62.6%	68.2%	56.6%
Brazil	Condado	2000	67.8%	70.9%	64.9%
Brazil	Condado	2000	70.2%	74.0%	65.9%
Brazil	Condado	2017	68.7%	71.8%	65.8%
Brazil	Condado	2017	71.0%	74.7%	66.7%
Brazil	Conde	2000	75.7%	80.4%	71.2%
Brazil	Conde	2000	65.9%	69.1%	63.0%
Brazil	Conde	2017	76.3%	80.9%	71.9%
Brazil	Conde	2017	66.8%	69.9%	63.9%
Brazil	Condeúba	2000	78.5%	83.4%	72.4%
Brazil	Condeúba	2017	79.2%	84.0%	73.2%
Brazil	Condor	2000	81.9%	85.9%	77.5%
Brazil	Condor	2017	82.5%	86.3%	78.1%
Brazil	Cônego Marinho	2000	80.5%	85.7%	73.7%
Brazil	Cônego Marinho	2017	81.1%	86.2%	74.5%
Brazil	Confins	2000	81.2%	83.9%	78.3%
Brazil	Confins	2017	81.8%	84.4%	78.9%
Brazil	Confresa	2000	65.8%	74.0%	55.8%
Brazil	Confresa	2017	66.7%	74.7%	56.7%
Brazil	Congo	2000	67.0%	71.6%	62.3%
Brazil	Congo	2017	67.9%	72.4%	63.2%
Brazil	Congonhal	2000	88.0%	90.5%	84.8%
Brazil	Congonhal	2017	88.4%	90.8%	85.3%
Brazil	Congonhas	2000	82.1%	85.5%	78.8%
Brazil	Congonhas	2017	82.7%	86.0%	79.4%
Brazil	Congonhas do Norte	2000	84.2%	87.8%	79.2%
Brazil	Congonhas do Norte	2017	84.7%	88.2%	79.8%
Brazil	Congonhinhas	2000	86.3%	89.0%	83.6%
Brazil	Congonhinhas	2017	86.7%	89.4%	84.1%
Brazil	Conquista	2000	84.9%	89.0%	79.9%
Brazil	Conquista	2017	85.9%	89.8%	81.2%
Brazil	Conselheiro Lafaiete	2000	82.3%	86.0%	78.6%
Brazil	Conselheiro Lafaiete	2017	82.9%	86.5%	79.2%
Brazil	Conselheiro Mayrinck	2000	87.1%	90.1%	84.0%
Brazil	Conselheiro Mayrinck	2017	87.6%	90.5%	84.5%

Country	Second Administrative Unit	Year	Mean	Upper	Lower
Brazil	Conselheiro Pena	2000	78.7%	82.5%	74.2%
Brazil	Conselheiro Pena	2017	79.3%	83.1%	74.9%
Brazil	Consolação	2000	90.9%	92.7%	88.7%
Brazil	Consolação	2017	91.1%	92.8%	88.9%
Brazil	Constantina	2000	78.9%	82.8%	75.1%
Brazil	Constantina	2017	79.6%	83.3%	75.8%
Brazil	Contagem	2000	82.3%	84.6%	79.7%
Brazil	Contagem	2017	82.8%	85.2%	80.4%
Brazil	Contenda	2000	83.6%	85.9%	81.2%
Brazil	Contenda	2017	84.1%	86.4%	81.8%
Brazil	Contendas do Sincorá	2000	76.2%	81.7%	70.8%
Brazil	Contendas do Sincorá	2017	76.9%	82.3%	71.6%
Brazil	Coqueiral	2000	82.2%	86.0%	77.8%
Brazil	Coqueiral	2017	82.8%	86.4%	78.4%
Brazil	Coqueiro Seco	2000	57.4%	59.9%	54.6%
Brazil	Coqueiro Seco	2017	58.3%	60.9%	55.6%
Brazil	Coqueiros do Sul	2000	81.0%	84.5%	77.7%
Brazil	Coqueiros do Sul	2017	81.6%	85.0%	78.4%
Brazil	Coração de Jesus	2000	82.0%	85.7%	77.2%
Brazil	Coração de Jesus	2017	82.6%	86.1%	78.0%
Brazil	Coração de Maria	2000	77.0%	80.3%	73.5%
Brazil	Coração de Maria	2017	77.7%	80.9%	74.3%
Brazil	Corbélia	2000	84.8%	88.3%	81.1%
Brazil	Corbélia	2017	85.3%	88.7%	81.6%
Brazil	Cordeiro	2000	80.5%	83.7%	76.9%
Brazil	Cordeiro	2017	81.1%	84.2%	77.6%
Brazil	Cordeirópolis	2000	92.8%	94.3%	91.0%
Brazil	Cordeirópolis	2017	93.1%	94.5%	91.3%
Brazil	Cordeiros	2000	79.6%	84.3%	74.0%
Brazil	Cordeiros	2017	80.2%	84.8%	74.7%
Brazil	Cordilheira Alta	2000	77.3%	81.4%	73.0%
Brazil	Cordilheira Alta	2017	78.0%	81.9%	73.8%
Brazil	Cordisburgo	2000	81.9%	86.0%	77.4%
Brazil	Cordisburgo	2017	82.5%	86.5%	78.1%
Brazil	Cordislândia	2000	82.1%	86.9%	76.0%
Brazil	Cordislândia	2017	82.7%	87.3%	76.7%
Brazil	Coreaú	2000	66.6%	70.7%	62.4%
Brazil	Coreaú	2017	67.5%	71.5%	63.3%
Brazil	Coremas	2000	67.9%	71.7%	64.3%
Brazil	Coremas	2017	68.7%	72.5%	65.2%
Brazil	Corguinho	2000	78.1%	82.0%	73.4%
Brazil	Corguinho	2017	78.9%	82.7%	74.2%
Brazil	Coribe	2000	78.9%	85.1%	70.5%
Brazil	Coribe	2017	79.5%	85.6%	71.3%
Brazil	Corinto	2000	81.8%	86.4%	75.6%
Brazil	Corinto	2017	82.3%	86.9%	76.3%
Brazil	Cornélio Procópio	2000	88.5%	90.5%	86.3%
Brazil	Cornélio Procópio	2017	88.9%	90.8%	86.7%

Country	Second Administrative Unit	Year	Mean	Upper	Lower
Brazil	Coroaci	2000	81.3%	85.9%	74.8%
Brazil	Coroaci	2017	81.9%	86.4%	75.5%
Brazil	Coroados	2000	92.4%	94.5%	89.8%
Brazil	Coroados	2017	92.7%	94.7%	90.2%
Brazil	Coroatá	2000	61.7%	66.6%	56.5%
Brazil	Coroatá	2017	62.6%	67.4%	57.4%
Brazil	Coromandel	2000	80.2%	85.1%	74.1%
Brazil	Coromandel	2017	80.9%	85.6%	74.9%
Brazil	Coronel Barros	2000	81.9%	85.9%	77.3%
Brazil	Coronel Barros	2017	82.5%	86.4%	78.0%
Brazil	Coronel Bicaco	2000	81.2%	85.1%	76.8%
Brazil	Coronel Bicaco	2017	81.7%	85.6%	77.5%
Brazil	Coronel domingos Soares	2000	81.0%	85.1%	76.7%
Brazil	Coronel domingos Soares	2017	81.6%	85.6%	77.4%
Brazil	Coronel Ezequiel	2000	79.4%	82.3%	75.9%
Brazil	Coronel Ezequiel	2017	80.0%	82.9%	76.6%
Brazil	Coronel Fabriciano	2000	81.2%	84.7%	77.2%
Brazil	Coronel Fabriciano	2017	81.8%	85.2%	77.8%
Brazil	Coronel Freitas	2000	76.9%	81.3%	72.2%
Brazil	Coronel Freitas	2017	76.6%	81.0%	71.9%
Brazil	Coronel João Pessoa	2000	74.8%	78.7%	70.8%
Brazil	Coronel João Pessoa	2017	75.6%	79.4%	71.6%
Brazil	Coronel João Sá	2000	75.8%	79.6%	71.9%
Brazil	Coronel João Sá	2017	76.5%	80.2%	72.7%
Brazil	Coronel José Dias	2000	63.2%	69.5%	56.5%
Brazil	Coronel José Dias	2017	64.1%	70.2%	57.5%
Brazil	Coronel Macedo	2000	90.8%	93.0%	88.0%
Brazil	Coronel Macedo	2017	91.1%	93.3%	88.4%
Brazil	Coronel Martins	2000	78.2%	82.6%	74.0%
Brazil	Coronel Martins	2017	78.8%	83.1%	74.7%
Brazil	Coronel Murta	2000	81.2%	85.7%	75.1%
Brazil	Coronel Murta	2017	81.8%	86.2%	75.9%
Brazil	Coronel Pacheco	2000	80.0%	83.1%	76.3%
Brazil	Coronel Pacheco	2017	80.6%	83.7%	77.0%

Country	Second Administrative Unit	Year	Mean	Upper	Lower
Brazil	Coronel Sapucaia	2000	81.9%	86.6%	76.8%
Brazil	Coronel Sapucaia	2017	82.5%	87.0%	77.5%
Brazil	Coronel Vivida	2000	81.5%	85.3%	76.9%
Brazil	Coronel Vivida	2017	82.1%	85.8%	77.6%
Brazil	Coronel Xavier Chaves	2000	81.2%	85.4%	76.7%
Brazil	Coronel Xavier Chaves	2017	81.8%	85.8%	77.4%
Brazil	Córrego Danta	2000	82.1%	87.2%	76.7%
Brazil	Córrego Danta	2017	82.6%	87.6%	77.3%
Brazil	Córrego do Bom Jesus	2000	91.4%	93.0%	89.4%
Brazil	Córrego do Bom Jesus	2017	91.7%	93.2%	89.8%
Brazil	Córrego do Ouro	2000	76.0%	80.3%	70.6%
Brazil	Córrego do Ouro	2017	76.7%	80.9%	71.4%
Brazil	Córrego Fundo	2000	82.1%	86.2%	77.4%
Brazil	Córrego Fundo	2017	82.7%	86.7%	78.0%
Brazil	Córrego Novo	2000	81.4%	85.1%	77.8%
Brazil	Córrego Novo	2017	81.9%	85.5%	78.4%
Brazil	Correia Pinto	2000	77.4%	81.9%	72.6%
Brazil	Correia Pinto	2017	78.1%	82.4%	73.4%
Brazil	Corrente	2000	67.4%	74.9%	60.2%
Brazil	Corrente	2017	68.3%	75.7%	61.2%
Brazil	Correntes	2000	60.1%	63.4%	56.3%
Brazil	Correntes	2017	61.0%	64.3%	57.3%
Brazil	Correntina	2000	78.2%	84.4%	70.6%
Brazil	Correntina	2017	78.9%	84.8%	71.4%
Brazil	Cortes	2000	65.1%	68.9%	61.8%
Brazil	Cortes	2017	65.9%	69.7%	62.7%
Brazil	Corumbá	2000	77.9%	81.8%	73.3%
Brazil	Corumbá	2017	78.6%	82.3%	74.0%
Brazil	Corumbá de Goiás	2000	83.9%	86.3%	81.3%
Brazil	Corumbá de Goiás	2017	84.4%	86.8%	81.9%
Brazil	Corumbaíba	2000	79.4%	83.3%	74.7%
Brazil	Corumbaíba	2017	80.0%	83.9%	75.4%
Brazil	Corumbataí	2000	92.8%	94.3%	91.0%
Brazil	Corumbataí	2017	93.0%	94.5%	91.3%
Brazil	Corumbataí do Sul	2000	83.8%	87.0%	79.9%
Brazil	Corumbataí do Sul	2017	84.3%	87.4%	80.6%
Brazil	Corumbiara	2000	29.4%	34.7%	25.2%
Brazil	Corumbiara	2017	30.2%	35.6%	26.0%
Brazil	Corupá	2000	72.4%	76.7%	68.8%
Brazil	Corupá	2017	73.1%	77.4%	69.7%
Brazil	Coruripe	2000	64.8%	69.2%	60.8%
Brazil	Coruripe	2017	65.7%	70.0%	61.7%
Brazil	Cosmópolis	2000	93.0%	94.4%	91.4%
Brazil	Cosmópolis	2017	93.3%	94.6%	91.7%

Country	Second Administrative Unit	Year	Mean	Upper	Lower
Brazil	Cosmorama	2000	91.3%	93.6%	88.5%
Brazil	Cosmorama	2017	91.6%	93.8%	88.9%
Brazil	Costa Marques	2000	31.0%	38.4%	24.6%
Brazil	Costa Marques	2017	31.9%	39.3%	25.3%
Brazil	Costa Rica	2000	78.7%	83.2%	72.7%
Brazil	Costa Rica	2017	79.3%	83.7%	73.5%
Brazil	Cotegipe	2000	74.3%	80.9%	65.3%
Brazil	Cotegipe	2017	75.0%	81.5%	66.3%
Brazil	Cotia	2000	94.3%	95.1%	93.4%
Brazil	Cotia	2017	94.6%	95.4%	93.7%
Brazil	Cotiporã	2000	81.7%	85.3%	78.5%
Brazil	Cotiporã	2017	82.3%	85.7%	79.1%
Brazil	Cotriguaçu	2000	66.6%	75.0%	59.1%
Brazil	Cotriguaçu	2017	67.5%	75.7%	60.1%
Brazil	Couto de Magalhães	2000	82.4%	87.3%	76.3%
Brazil	Couto de Magalhães	2017	83.0%	87.7%	77.0%
Brazil	Couto Magalhaes	2000	63.7%	69.1%	57.9%
Brazil	Couto Magalhaes	2017	64.6%	69.9%	58.8%
Brazil	Coxilha	2000	81.2%	84.6%	76.6%
Brazil	Coxilha	2017	81.8%	85.1%	77.3%
Brazil	Coxim	2000	77.9%	82.4%	71.6%
Brazil	Coxim	2017	78.5%	82.9%	72.4%
Brazil	Coxixola	2000	67.6%	72.0%	62.4%
Brazil	Coxixola	2017	68.4%	72.7%	63.2%
Brazil	Craíbas	2000	64.1%	66.8%	61.4%
Brazil	Craíbas	2017	65.0%	67.7%	62.3%
Brazil	Crateús	2000	67.0%	71.2%	62.7%
Brazil	Crateús	2017	67.8%	71.9%	63.6%
Brazil	Crato	2000	68.8%	72.3%	65.3%
Brazil	Crato	2017	69.6%	73.1%	66.2%
Brazil	Cravinhos	2000	92.9%	94.5%	90.8%
Brazil	Cravinhos	2017	93.1%	94.7%	91.1%
Brazil	Cravolândia	2000	82.2%	85.5%	77.6%
Brazil	Cravolândia	2017	82.8%	86.0%	78.2%
Brazil	Criciúma	2000	75.5%	79.5%	70.8%
Brazil	Criciúma	2017	76.2%	80.1%	71.6%
Brazil	Crisólita	2000	81.0%	85.3%	75.9%
Brazil	Crisólita	2017	81.5%	85.8%	76.5%
Brazil	Crisópolis	2000	79.2%	82.7%	75.5%
Brazil	Crisópolis	2017	79.1%	82.6%	75.5%
Brazil	Crissiumal	2000	80.6%	84.8%	75.9%
Brazil	Crissiumal	2017	81.1%	85.3%	76.5%
Brazil	Cristais	2000	81.4%	85.4%	76.6%
Brazil	Cristais	2017	82.7%	86.4%	78.1%
Brazil	Cristais Paulista	2000	87.9%	91.1%	84.1%
Brazil	Cristais Paulista	2017	88.3%	91.4%	84.6%
Brazil	Cristal	2000	81.3%	85.3%	76.8%
Brazil	Cristal	2017	81.9%	85.8%	77.5%
Brazil	Cristal do Sul	2000	78.0%	81.9%	73.5%
Brazil	Cristal do Sul	2017	78.7%	82.5%	74.3%
Brazil	Cristalândia	2000	75.7%	80.1%	70.4%
Brazil	Cristalândia	2017	76.4%	80.7%	71.2%
Brazil	Cristalândia do Piauí	2000	68.7%	76.3%	61.7%

Country	Second Administrative Unit	Year	Mean	Upper	Lower
Brazil	Cristalândia do Piauí	2017	69.5%	76.9%	62.6%
Brazil	Cristália	2000	82.3%	87.1%	76.6%
Brazil	Cristália	2017	82.9%	87.5%	77.3%
Brazil	Cristalina	2000	87.1%	89.9%	83.8%
Brazil	Cristalina	2017	87.7%	90.4%	84.5%
Brazil	Cristiano Otoni	2000	81.1%	84.8%	77.3%
Brazil	Cristiano Otoni	2017	81.7%	85.3%	77.9%
Brazil	Cristianópolis	2000	78.0%	81.4%	74.1%
Brazil	Cristianópolis	2017	78.7%	82.0%	74.9%
Brazil	Cristina	2000	88.8%	91.6%	85.8%
Brazil	Cristina	2017	89.3%	91.9%	86.3%
Brazil	Cristinápolis	2000	77.4%	80.7%	74.0%
Brazil	Cristinápolis	2017	78.1%	81.3%	74.8%
Brazil	Cristino Castro	2000	59.1%	67.0%	52.0%
Brazil	Cristino Castro	2017	60.1%	67.9%	53.0%
Brazil	Cristópolis	2000	75.9%	82.2%	67.4%
Brazil	Cristópolis	2017	76.6%	82.7%	68.2%
Brazil	Crixás	2000	77.7%	82.9%	71.9%
Brazil	Crixás	2017	78.4%	83.5%	72.7%
Brazil	Crixás do Tocantins	2000	75.7%	79.9%	71.8%
Brazil	Crixás do Tocantins	2017	76.4%	80.5%	72.5%
Brazil	Croatá	2000	70.9%	75.3%	66.6%
Brazil	Croatá	2017	71.7%	76.0%	67.4%
Brazil	Cromínia	2000	76.4%	79.7%	72.4%
Brazil	Cromínia	2017	77.1%	80.3%	73.1%
Brazil	Crucilândia	2000	80.6%	84.3%	76.5%
Brazil	Crucilândia	2017	81.2%	84.8%	77.2%
Brazil	Cruz	2000	67.4%	72.7%	61.9%
Brazil	Cruz	2017	68.2%	73.3%	62.7%
Brazil	Cruz Alta	2000	84.4%	87.9%	79.9%
Brazil	Cruz Alta	2017	84.9%	88.3%	80.5%
Brazil	Cruz das Almas	2000	77.2%	80.7%	72.9%
Brazil	Cruz das Almas	2017	78.1%	81.4%	73.8%
Brazil	Cruz do Espírito Santo	2000	67.4%	70.6%	64.5%
Brazil	Cruz do Espírito Santo	2017	68.3%	71.4%	65.4%
Brazil	Cruz Machado	2000	80.4%	84.0%	75.8%
Brazil	Cruz Machado	2017	81.0%	84.5%	76.5%
Brazil	Cruzália	2000	88.4%	90.8%	85.8%
Brazil	Cruzália	2017	88.8%	91.2%	86.3%
Brazil	Cruzeiro	2000	86.2%	88.7%	83.2%
Brazil	Cruzeiro	2017	86.9%	89.3%	84.1%
Brazil	Cruzeiro da Fortaleza	2000	81.3%	86.1%	75.6%
Brazil	Cruzeiro da Fortaleza	2017	81.9%	86.6%	76.3%
Brazil	Cruzeiro do Iguaçu	2000	81.8%	85.5%	78.2%
Brazil	Cruzeiro do Iguaçu	2017	82.4%	86.0%	78.8%

Country	Second Administrative Unit	Year	Mean	Upper	Lower
Brazil	Cruzeiro do Oeste	2000	84.5%	87.7%	81.0%
Brazil	Cruzeiro do Oeste	2017	85.0%	88.1%	81.6%
Brazil	Cruzeiro do Sul	2000	45.4%	49.5%	41.8%
Brazil	Cruzeiro do Sul	2000	81.0%	84.5%	77.7%
Brazil	Cruzeiro do Sul	2000	85.3%	88.1%	82.1%
Brazil	Cruzeiro do Sul	2017	85.8%	88.5%	82.7%
Brazil	Cruzeiro do Sul	2017	46.0%	50.0%	42.4%
Brazil	Cruzeiro do Sul	2017	81.9%	85.2%	78.6%
Brazil	Cruzeta	2000	78.4%	81.8%	74.8%
Brazil	Cruzeta	2017	79.0%	82.3%	75.5%
Brazil	Cruzília	2000	86.3%	89.1%	82.8%
Brazil	Cruzília	2017	86.8%	89.5%	83.3%
Brazil	Cruzmaltina	2000	83.3%	86.5%	79.7%
Brazil	Cruzmaltina	2017	83.8%	87.0%	80.4%
Brazil	Cubatão	2000	87.5%	89.3%	85.6%
Brazil	Cubatão	2017	87.9%	89.7%	86.0%
Brazil	Cubati	2000	74.3%	77.9%	70.3%
Brazil	Cubati	2017	74.9%	78.4%	70.9%
Brazil	Cuiaba	2000	67.9%	70.9%	64.3%
Brazil	Cuiaba	2017	68.7%	71.6%	65.2%
Brazil	Cuité	2000	77.5%	80.7%	74.0%
Brazil	Cuité	2017	78.1%	81.2%	74.7%
Brazil	Cuité de Mamanguape	2000	68.3%	71.3%	65.2%
Brazil	Cuité de Mamanguape	2017	69.2%	72.1%	66.1%
Brazil	Cuitegi	2000	68.6%	71.4%	65.3%
Brazil	Cuitegi	2017	69.4%	72.2%	66.2%
Brazil	Cujubim	2000	28.9%	32.9%	24.3%
Brazil	Cujubim	2017	29.7%	33.7%	25.0%
Brazil	Cumari	2000	77.8%	82.5%	72.5%
Brazil	Cumari	2017	78.5%	83.0%	73.2%
Brazil	Cumaru	2000	65.8%	69.2%	62.7%
Brazil	Cumaru	2017	66.5%	69.9%	63.4%
Brazil	Cumaru do Norte	2000	43.7%	51.8%	35.6%
Brazil	Cumaru do Norte	2017	44.6%	52.8%	36.5%
Brazil	Cumbe	2000	73.7%	76.6%	70.4%
Brazil	Cumbe	2017	74.5%	77.3%	71.2%
Brazil	Cunha	2000	90.6%	92.7%	87.8%
Brazil	Cunha	2017	90.9%	93.0%	88.2%
Brazil	Cunha Porã	2000	79.9%	83.9%	75.7%
Brazil	Cunha Porã	2017	80.6%	84.5%	76.5%
Brazil	Cunhataí	2000	78.3%	82.1%	74.0%
Brazil	Cunhataí	2017	78.9%	82.7%	74.8%
Brazil	Cuparaque	2000	80.1%	84.3%	76.0%
Brazil	Cuparaque	2017	80.8%	84.8%	76.7%
Brazil	Cupira	2000	64.5%	68.2%	61.4%
Brazil	Cupira	2017	65.4%	69.1%	62.3%
Brazil	Curaçá	2000	67.7%	72.8%	62.2%
Brazil	Curaçá	2017	68.5%	73.6%	63.1%
Brazil	Curimatá	2000	63.8%	72.0%	56.9%
Brazil	Curimatá	2017	64.7%	72.7%	57.8%

Country	Second Administrative Unit	Year	Mean	Upper	Lower
Brazil	Curionópolis	2000	46.5%	51.9%	40.6%
Brazil	Curionópolis	2017	47.2%	52.6%	41.4%
Brazil	Curitiba	2000	85.7%	87.5%	84.0%
Brazil	Curitiba	2017	86.2%	87.9%	84.5%
Brazil	Curitibanos	2000	77.4%	81.5%	72.5%
Brazil	Curitibanos	2017	78.1%	82.1%	73.3%
Brazil	Curiúva	2000	85.6%	88.6%	82.1%
Brazil	Curiúva	2017	86.0%	89.0%	82.6%
Brazil	Currais	2000	61.1%	68.8%	54.0%
Brazil	Currais	2017	61.9%	69.6%	54.9%
Brazil	Currais Novos	2000	83.5%	86.1%	80.6%
Brazil	Currais Novos	2017	84.0%	86.6%	81.1%
Brazil	Curral de Cima	2000	72.3%	75.0%	69.3%
Brazil	Curral de Cima	2017	73.1%	75.8%	70.2%
Brazil	Curral de dentro	2000	82.7%	87.0%	77.5%
Brazil	Curral de dentro	2017	83.3%	87.5%	78.2%
Brazil	Curral Novo do Piauí	2000	64.1%	68.9%	58.6%
Brazil	Curral Novo do Piauí	2017	65.0%	69.8%	59.6%
Brazil	Curral Velho	2000	67.7%	71.7%	63.4%
Brazil	Curral Velho	2017	68.6%	72.5%	64.3%
Brazil	Curralinho	2000	38.5%	43.6%	32.4%
Brazil	Curralinho	2017	39.4%	44.5%	33.2%
Brazil	Curralinhos	2000	59.9%	64.1%	55.3%
Brazil	Curralinhos	2017	60.8%	65.0%	56.2%
Brazil	Curuá	2000	40.4%	46.9%	33.5%
Brazil	Curuá	2017	41.4%	47.9%	34.4%
Brazil	Curuçá	2000	35.3%	40.6%	30.5%
Brazil	Curuçá	2017	36.2%	41.6%	31.3%
Brazil	Cururupu	2000	60.2%	67.4%	51.5%
Brazil	Cururupu	2017	61.1%	68.2%	52.5%
Brazil	Curvelo	2000	82.1%	86.6%	76.1%
Brazil	Curvelo	2017	82.7%	87.1%	76.8%
Brazil	Custódia	2000	67.4%	71.6%	62.8%
Brazil	Custódia	2017	68.3%	72.4%	63.8%
Brazil	Cutias	2000	47.8%	52.2%	43.5%
Brazil	Cutias	2017	48.8%	53.2%	44.5%
Brazil	Damianópolis	2000	82.1%	86.9%	76.1%
Brazil	Damianópolis	2017	82.6%	87.3%	76.8%
Brazil	Damião	2000	76.3%	79.4%	72.6%
Brazil	Damião	2017	76.9%	80.0%	73.3%
Brazil	Darcinópolis	2000	74.2%	78.4%	70.3%
Brazil	Darcinópolis	2017	74.8%	79.0%	71.0%
Brazil	Dário Meira	2000	78.5%	83.0%	73.4%
Brazil	Dário Meira	2017	79.2%	83.6%	74.2%
Brazil	Datas	2000	84.4%	88.6%	79.0%
Brazil	Datas	2017	84.9%	89.0%	79.6%
Brazil	David Canabarro	2000	80.6%	84.3%	76.3%
Brazil	David Canabarro	2017	81.2%	84.8%	77.0%
Brazil	Davinópolis	2000	65.4%	69.6%	60.9%
Brazil	Davinópolis	2000	78.1%	83.3%	72.5%
Brazil	Davinópolis	2017	78.8%	83.8%	73.3%
Brazil	Davinópolis	2017	66.3%	70.5%	61.9%
Brazil	Delfim Moreira	2000	90.7%	92.7%	88.6%

Country	Second Administrative Unit	Year	Mean	Upper	Lower
Brazil	Delfim Moreira	2017	91.1%	93.0%	89.0%
Brazil	Delfinópolis	2000	85.2%	89.3%	81.1%
Brazil	Delfinópolis	2017	85.7%	89.7%	81.7%
Brazil	Delmiro Gouveia	2000	65.2%	69.2%	61.0%
Brazil	Delmiro Gouveia	2017	66.1%	70.0%	62.0%
Brazil	Delta	2000	86.3%	89.7%	81.5%
Brazil	Delta	2017	87.8%	90.9%	83.5%
Brazil	Demerval Lobão	2000	59.1%	62.5%	55.4%
Brazil	Demerval Lobão	2017	60.0%	63.4%	56.3%
Brazil	Denise	2000	65.8%	71.4%	59.5%
Brazil	Denise	2017	66.6%	72.1%	60.4%
Brazil	Deodápolis	2000	80.0%	83.4%	76.2%
Brazil	Deodápolis	2017	80.7%	84.0%	76.9%
Brazil	Deputado Irapuan Pinheiro	2000	70.4%	75.0%	66.0%
Brazil	Deputado Irapuan Pinheiro	2017	71.2%	75.7%	66.8%
Brazil	Derrubadas	2000	79.8%	84.4%	74.7%
Brazil	Derrubadas	2017	80.3%	84.8%	75.3%
Brazil	Descalvado	2000	92.4%	94.0%	90.3%
Brazil	Descalvado	2017	92.7%	94.3%	90.7%
Brazil	Descanso	2000	79.6%	83.5%	75.6%
Brazil	Descanso	2017	80.2%	84.0%	76.3%
Brazil	Descoberto	2000	80.1%	83.5%	76.0%
Brazil	Descoberto	2017	80.7%	84.0%	76.7%
Brazil	Desterro	2000	70.6%	74.9%	66.4%
Brazil	Desterro	2017	71.4%	75.6%	67.2%
Brazil	Desterro de Entre Rios	2000	82.2%	85.9%	78.2%
Brazil	Desterro de Entre Rios	2017	82.7%	86.3%	78.8%
Brazil	Desterro de Malta	2000	75.5%	78.6%	71.6%
Brazil	Desterro de Malta	2017	76.2%	79.3%	72.5%
Brazil	Desterro do Melo	2000	80.9%	84.6%	76.6%
Brazil	Desterro do Melo	2017	81.5%	85.1%	77.3%
Brazil	Dezesseis de Novembro	2000	81.4%	86.5%	75.3%
Brazil	Dezesseis de Novembro	2017	82.0%	86.9%	76.1%
Brazil	Diadema	2000	94.8%	95.5%	94.1%
Brazil	Diadema	2017	95.0%	95.7%	94.3%
Brazil	Diamante	2000	67.7%	71.5%	63.4%
Brazil	Diamante	2017	68.6%	72.3%	64.4%
Brazil	Diamante d'Oeste	2000	83.8%	87.4%	80.1%
Brazil	Diamante d'Oeste	2017	84.3%	87.8%	80.7%
Brazil	Diamante do Norte	2000	83.2%	87.1%	79.0%
Brazil	Diamante do Norte	2017	83.7%	87.6%	79.6%
Brazil	Diamante do Sul	2000	83.4%	86.7%	79.0%

Country	Second Administrative Unit	Year	Mean	Upper	Lower
Brazil	Diamante do Sul	2017	84.0%	87.2%	79.7%
Brazil	Diamantina	2000	84.2%	88.5%	78.1%
Brazil	Diamantina	2017	84.7%	88.9%	78.8%
Brazil	Diamantino	2000	69.8%	76.4%	63.9%
Brazil	Diamantino	2017	70.7%	77.0%	64.8%
Brazil	Dianopolis	2000	77.0%	82.3%	71.6%
Brazil	Dianopolis	2017	77.7%	82.9%	72.4%
Brazil	Dias d'vila	2000	76.1%	79.8%	72.6%
Brazil	Dias d'vila	2017	76.8%	80.4%	73.3%
Brazil	Dilermano de Aguiar	2000	81.2%	85.4%	75.9%
Brazil	Dilermano de Aguiar	2017	81.7%	85.9%	76.6%
Brazil	Diogo de Vasconcelos	2000	80.2%	83.9%	76.0%
Brazil	Diogo de Vasconcelos	2017	80.8%	84.4%	76.7%
Brazil	Dionísio	2000	81.1%	84.8%	77.0%
Brazil	Dionísio	2017	80.9%	84.6%	76.8%
Brazil	Dionísio Cerqueira	2000	82.1%	86.2%	77.3%
Brazil	Dionísio Cerqueira	2017	82.6%	86.7%	78.0%
Brazil	Diorama	2000	75.4%	81.1%	69.5%
Brazil	Diorama	2017	76.1%	81.7%	70.3%
Brazil	Dirce Reis	2000	88.9%	92.0%	85.3%
Brazil	Dirce Reis	2017	89.3%	92.3%	85.8%
Brazil	Dirceu Arcoverde	2000	65.4%	72.3%	58.2%
Brazil	Dirceu Arcoverde	2017	66.3%	73.1%	59.1%
Brazil	Divina Pastora	2000	74.0%	76.0%	71.3%
Brazil	Divina Pastora	2017	74.7%	76.7%	72.0%
Brazil	Divinésia	2000	80.6%	83.7%	76.4%
Brazil	Divinésia	2017	81.2%	84.2%	77.1%
Brazil	Divino	2000	81.6%	85.3%	78.2%
Brazil	Divino	2017	82.2%	85.7%	78.8%
Brazil	Divino das Laranjeiras	2000	79.6%	84.7%	74.9%
Brazil	Divino das Laranjeiras	2017	80.2%	85.2%	75.6%
Brazil	Divino de São Lourenço	2000	80.7%	83.8%	77.1%
Brazil	Divino de São Lourenço	2017	81.3%	84.3%	77.8%
Brazil	Divinolândia	2000	90.3%	92.7%	87.3%
Brazil	Divinolândia	2017	90.6%	92.9%	87.8%
Brazil	Divinolandia de Minas	2000	83.6%	87.4%	78.6%
Brazil	Divinolandia de Minas	2017	84.1%	87.9%	79.3%
Brazil	Divinópolis	2000	81.7%	85.7%	77.7%
Brazil	Divinópolis	2017	82.3%	86.2%	78.4%
Brazil	Divinópolis de Goiás	2000	78.0%	83.3%	72.4%
Brazil	Divinópolis de Goiás	2017	78.6%	83.8%	73.2%
Brazil	Divinópolis do Tocantins	2000	70.4%	74.9%	65.7%

Country	Second Administrative Unit	Year	Mean	Upper	Lower
Brazil	Divinópolis do Tocantins	2017	71.3%	75.7%	66.7%
Brazil	Divisa Alegre	2000	80.7%	85.2%	75.2%
Brazil	Divisa Alegre	2017	81.3%	85.7%	76.0%
Brazil	Divisa Nova	2000	81.2%	85.3%	75.6%
Brazil	Divisa Nova	2017	81.8%	85.8%	76.3%
Brazil	Divisópolis	2000	85.6%	89.0%	81.5%
Brazil	Divisópolis	2017	86.1%	89.4%	82.1%
Brazil	Dobrada	2000	93.4%	94.9%	91.2%
Brazil	Dobrada	2017	93.6%	95.1%	91.5%
Brazil	Dois Córregos	2000	93.7%	95.3%	91.7%
Brazil	Dois Córregos	2017	93.9%	95.5%	92.0%
Brazil	Dois Irmãos	2000	81.6%	84.0%	78.9%
Brazil	Dois Irmãos	2017	82.2%	84.6%	79.6%
Brazil	Dois Irmãos das Missões	2000	80.7%	84.7%	76.4%
Brazil	Dois Irmãos das Missões	2017	81.3%	85.2%	77.1%
Brazil	Dois Irmãos do Buriti	2000	78.6%	82.1%	75.0%
Brazil	Dois Irmãos do Buriti	2017	79.2%	82.6%	75.7%
Brazil	Dois Lajeados	2000	81.4%	85.0%	77.8%
Brazil	Dois Lajeados	2017	82.0%	85.5%	78.4%
Brazil	Dois Riachos	2000	62.0%	65.1%	58.6%
Brazil	Dois Riachos	2017	62.9%	66.0%	59.5%
Brazil	Dois Vizinhos	2000	83.2%	86.7%	79.5%
Brazil	Dois Vizinhos	2017	83.8%	87.2%	80.2%
Brazil	Dom Aquino	2000	66.6%	72.0%	61.1%
Brazil	Dom Aquino	2017	67.6%	72.8%	62.1%
Brazil	Dom Basílio	2000	77.1%	82.2%	71.9%
Brazil	Dom Basílio	2017	77.8%	82.7%	72.7%
Brazil	Dom Bosco	2000	83.6%	89.3%	77.0%
Brazil	Dom Bosco	2017	84.1%	89.6%	77.7%
Brazil	Dom Cavati	2000	81.6%	85.5%	77.1%
Brazil	Dom Cavati	2017	82.2%	86.0%	77.8%
Brazil	Dom Eliseu	2000	52.7%	58.4%	46.4%
Brazil	Dom Eliseu	2017	53.6%	59.3%	47.2%
Brazil	Dom Expedito Lopes	2000	63.8%	68.3%	58.8%
Brazil	Dom Expedito Lopes	2017	64.8%	69.1%	59.8%
Brazil	Dom Feliciano	2000	82.4%	86.4%	78.1%
Brazil	Dom Feliciano	2017	82.9%	86.9%	78.7%
Brazil	Dom Inocêncio	2000	63.5%	70.2%	56.7%
Brazil	Dom Inocêncio	2017	64.4%	71.1%	57.6%
Brazil	Dom Joaquim	2000	80.2%	84.2%	74.3%
Brazil	Dom Joaquim	2017	80.8%	84.7%	75.0%
Brazil	Dom Macedo Costa	2000	76.3%	80.1%	71.8%
Brazil	Dom Macedo Costa	2017	77.0%	80.7%	72.5%
Brazil	Dom Pedrito	2000	84.9%	89.5%	79.9%
Brazil	Dom Pedrito	2017	86.3%	90.5%	81.6%
Brazil	Dom Pedro	2000	62.0%	68.0%	56.7%
Brazil	Dom Pedro	2017	62.9%	68.8%	57.6%
Brazil	Dom Pedro de Alcântara	2000	76.5%	81.2%	71.2%
Brazil	Dom Pedro de Alcântara	2017	77.2%	81.8%	72.0%

Country	Second Administrative Unit	Year	Mean	Upper	Lower
Brazil	Dom Silvério	2000	81.2%	85.0%	77.4%
Brazil	Dom Silvério	2017	81.8%	85.5%	78.1%
Brazil	Dom Viçoso	2000	86.1%	89.0%	83.0%
Brazil	Dom Viçoso	2017	86.5%	89.4%	83.5%
Brazil	Domingos Martins	2000	81.0%	83.3%	78.7%
Brazil	Domingos Martins	2017	81.6%	83.9%	79.3%
Brazil	Domingos Mourão	2000	64.1%	68.2%	59.2%
Brazil	Domingos Mourão	2017	64.9%	69.0%	60.1%
Brazil	Dona Emma	2000	74.5%	78.6%	70.3%
Brazil	Dona Emma	2017	75.2%	79.3%	71.1%
Brazil	Dona Eusébia	2000	79.2%	82.8%	75.2%
Brazil	Dona Eusébia	2017	79.8%	83.3%	75.9%
Brazil	Dona Francisca	2000	81.2%	84.7%	76.7%
Brazil	Dona Francisca	2017	81.9%	85.3%	77.5%
Brazil	Dona Inês	2000	74.0%	76.9%	71.2%
Brazil	Dona Inês	2017	74.8%	77.7%	72.0%
Brazil	Dores de Campos	2000	81.0%	85.2%	76.8%
Brazil	Dores de Campos	2017	81.6%	85.7%	77.5%
Brazil	Dores de Guanhães	2000	80.5%	84.9%	75.5%
Brazil	Dores de Guanhães	2017	81.1%	85.4%	76.2%
Brazil	Dores do Indaiá	2000	81.9%	86.8%	76.3%
Brazil	Dores do Indaiá	2017	82.5%	87.2%	77.1%
Brazil	Dores do Rio Preto	2000	81.9%	85.1%	78.7%
Brazil	Dores do Rio Preto	2017	82.5%	85.6%	79.4%
Brazil	Dores do Turvo	2000	80.6%	84.1%	76.9%
Brazil	Dores do Turvo	2017	81.2%	84.6%	77.6%
Brazil	Doresópolis	2000	82.1%	86.8%	76.7%
Brazil	Doresópolis	2017	82.7%	87.2%	77.4%
Brazil	Dormentes	2000	65.0%	70.3%	59.6%
Brazil	Dormentes	2017	65.8%	71.1%	60.6%
Brazil	Douradina	2000	83.2%	86.4%	79.4%
Brazil	Douradina	2000	79.8%	83.1%	76.5%
Brazil	Douradina	2017	80.4%	83.7%	77.2%
Brazil	Douradina	2017	83.7%	86.8%	80.1%
Brazil	Dourado	2000	93.3%	95.0%	91.5%
Brazil	Dourado	2017	93.5%	95.2%	91.8%
Brazil	Douradoquara	2000	78.4%	83.1%	72.8%
Brazil	Douradoquara	2017	79.0%	83.7%	73.5%
Brazil	Dourados	2000	82.1%	84.6%	79.2%
Brazil	Dourados	2017	82.9%	85.3%	80.1%
Brazil	Doutor Camargo	2000	83.0%	85.9%	79.6%
Brazil	Doutor Camargo	2017	84.7%	87.4%	81.5%
Brazil	Doutor Maurício Cardoso	2000	79.8%	84.7%	74.4%

Country	Second Administrative Unit	Year	Mean	Upper	Lower
Brazil	Doutor Maurício Cardoso	2017	80.3%	85.1%	74.9%
Brazil	Doutor Pedrinho	2000	77.1%	80.5%	73.6%
Brazil	Doutor Pedrinho	2017	77.8%	81.1%	74.4%
Brazil	Doutor Ricardo	2000	81.8%	85.2%	78.3%
Brazil	Doutor Ricardo	2017	82.4%	85.7%	79.0%
Brazil	Doutor Severiano	2000	74.1%	78.1%	70.1%
Brazil	Doutor Severiano	2017	74.9%	78.7%	70.8%
Brazil	Doutor Ulysses	2000	86.6%	89.9%	83.3%
Brazil	Doutor Ulysses	2017	87.1%	90.2%	83.8%
Brazil	Doverlândia	2000	75.1%	80.9%	67.8%
Brazil	Doverlândia	2017	74.5%	80.3%	67.0%
Brazil	Dracena	2000	88.6%	91.3%	85.3%
Brazil	Dracena	2017	89.0%	91.6%	85.7%
Brazil	Duartina	2000	93.4%	95.2%	91.5%
Brazil	Duartina	2017	93.7%	95.4%	91.8%
Brazil	Duas Barras	2000	81.5%	84.3%	78.5%
Brazil	Duas Barras	2017	82.1%	84.8%	79.1%
Brazil	Duas Estradas	2000	72.9%	75.6%	70.0%
Brazil	Duas Estradas	2017	73.6%	76.2%	70.8%
Brazil	Dueré	2000	75.3%	79.5%	71.2%
Brazil	Dueré	2017	76.0%	80.1%	72.0%
Brazil	Dulcinopolis	2000	88.3%	91.6%	84.7%
Brazil	Dulcinopolis	2017	88.7%	91.9%	85.2%
Brazil	Dumont	2000	93.2%	94.8%	90.9%
Brazil	Dumont	2017	93.4%	95.0%	91.2%
Brazil	Duque Bacelar	2000	59.9%	64.4%	55.0%
Brazil	Duque Bacelar	2017	60.8%	65.2%	55.9%
Brazil	Duque de Caxias	2000	76.9%	78.4%	75.3%
Brazil	Duque de Caxias	2017	77.6%	79.1%	76.0%
Brazil	Durandé	2000	80.5%	83.7%	76.5%
Brazil	Durandé	2017	81.1%	84.3%	77.2%
Brazil	Echaporã	2000	91.8%	94.0%	89.0%
Brazil	Echaporã	2017	92.1%	94.2%	89.4%
Brazil	Ecoporanga	2000	79.4%	83.0%	75.2%
Brazil	Ecoporanga	2017	80.0%	83.5%	75.9%
Brazil	Edealina	2000	76.0%	79.9%	71.6%
Brazil	Edealina	2017	76.7%	80.5%	72.4%
Brazil	Edéia	2000	77.2%	81.5%	72.5%
Brazil	Edéia	2017	77.8%	82.0%	73.2%
Brazil	Eirunepé	2000	60.4%	67.3%	53.0%
Brazil	Eirunepé	2017	61.2%	68.0%	53.9%
Brazil	Eldorado	2000	81.9%	85.8%	77.7%
Brazil	Eldorado	2000	88.3%	91.6%	84.6%
Brazil	Eldorado	2017	88.8%	91.9%	85.1%
Brazil	Eldorado	2017	82.4%	86.3%	78.4%
Brazil	Eldorado do Sul	2000	81.2%	83.6%	79.2%
Brazil	Eldorado do Sul	2017	81.8%	84.2%	79.9%

Country	Second Administrative Unit	Year	Mean	Upper	Lower
Brazil	Eldorado dos Carajás	2000	45.6%	51.1%	39.6%
Brazil	Eldorado dos Carajás	2017	46.5%	52.1%	40.5%
Brazil	Elesbão Veloso	2000	59.9%	64.7%	54.3%
Brazil	Elesbão Veloso	2017	61.7%	66.4%	56.2%
Brazil	Elias Fausto	2000	93.2%	94.3%	92.0%
Brazil	Elias Fausto	2017	93.4%	94.5%	92.2%
Brazil	Eliseu Martins	2000	61.1%	67.6%	53.5%
Brazil	Eliseu Martins	2017	62.0%	68.4%	54.5%
Brazil	Elisiário	2000	92.9%	94.8%	90.6%
Brazil	Elisiário	2017	93.1%	95.0%	90.9%
Brazil	Elísio Medrado	2000	79.7%	83.4%	74.5%
Brazil	Elísio Medrado	2017	80.3%	83.9%	75.2%
Brazil	Elói Mendes	2000	84.3%	87.5%	80.6%
Brazil	Elói Mendes	2017	84.8%	87.9%	81.2%
Brazil	Emas	2000	65.8%	69.7%	61.6%
Brazil	Emas	2017	66.7%	70.5%	62.5%
Brazil	Embaúba	2000	92.7%	94.2%	90.8%
Brazil	Embaúba	2017	92.9%	94.4%	91.1%
Brazil	Embu	2000	94.6%	95.4%	93.7%
Brazil	Embu	2017	94.8%	95.6%	94.0%
Brazil	Embu-Guaçu	2000	93.8%	94.9%	92.6%
Brazil	Embu-Guaçu	2017	94.1%	95.1%	93.0%
Brazil	Emilianópolis	2000	88.5%	91.2%	85.6%
Brazil	Emilianópolis	2017	88.9%	91.5%	86.1%
Brazil	Encantado	2000	80.7%	84.2%	77.0%
Brazil	Encantado	2017	81.3%	84.7%	77.7%
Brazil	Encanto	2000	74.7%	78.5%	70.8%
Brazil	Encanto	2017	75.4%	79.2%	71.6%
Brazil	Encruzilhada	2000	80.0%	84.3%	74.5%
Brazil	Encruzilhada	2017	80.5%	84.5%	75.1%
Brazil	Encruzilhada do Sul	2000	83.8%	87.8%	79.9%
Brazil	Encruzilhada do Sul	2017	84.3%	88.2%	80.6%
Brazil	Enéas Marques	2000	82.5%	86.3%	78.1%
Brazil	Enéas Marques	2017	83.0%	86.8%	78.8%
Brazil	Engenheiro Beltrão	2000	82.9%	86.1%	79.7%
Brazil	Engenheiro Beltrão	2017	83.5%	86.6%	80.3%
Brazil	Engenheiro Caldas	2000	79.9%	84.3%	75.5%
Brazil	Engenheiro Caldas	2017	80.6%	84.8%	76.3%
Brazil	Engenheiro Coelho	2000	92.4%	93.8%	90.7%
Brazil	Engenheiro Coelho	2017	92.6%	94.0%	91.1%
Brazil	Engenheiro Navarro	2000	82.5%	87.2%	77.5%
Brazil	Engenheiro Navarro	2017	83.1%	87.6%	78.2%

Country	Second Administrative Unit	Year	Mean	Upper	Lower
Brazil	Engenheiro Paulo de Front	2000	81.8%	83.9%	79.1%
Brazil	Engenheiro Paulo de Front	2017	82.3%	84.4%	79.7%
Brazil	Engenho Velho	2000	79.0%	82.4%	75.5%
Brazil	Engenho Velho	2017	79.7%	83.0%	76.2%
Brazil	Entre Folhas	2000	81.5%	85.1%	77.0%
Brazil	Entre Folhas	2017	82.1%	85.5%	77.7%
Brazil	Entre Rios	2000	76.6%	81.2%	71.9%
Brazil	Entre Rios	2000	76.7%	80.8%	72.7%
Brazil	Entre Rios	2017	77.4%	81.8%	72.7%
Brazil	Entre Rios	2017	77.4%	81.4%	73.5%
Brazil	Entre Rios de Minas	2000	82.0%	85.8%	78.5%
Brazil	Entre Rios de Minas	2017	82.6%	86.3%	79.2%
Brazil	Entre Rios do Oeste	2000	82.1%	86.2%	78.3%
Brazil	Entre Rios do Oeste	2017	82.7%	86.7%	78.9%
Brazil	Entre Rios do Sul	2000	78.7%	81.9%	75.4%
Brazil	Entre Rios do Sul	2017	79.4%	82.5%	76.2%
Brazil	Entre-Ijuís	2000	82.4%	86.4%	78.0%
Brazil	Entre-Ijuís	2017	82.9%	86.8%	78.6%
Brazil	Envira	2000	53.6%	59.8%	48.1%
Brazil	Envira	2017	53.8%	59.9%	48.8%
Brazil	Epitaciolândia	2000	42.4%	48.0%	37.9%
Brazil	Epitaciolândia	2017	43.4%	49.0%	38.8%
Brazil	Equador	2000	74.0%	77.7%	69.7%
Brazil	Equador	2017	74.7%	78.3%	70.5%
Brazil	Erebango	2000	79.8%	83.3%	75.9%
Brazil	Erebango	2017	80.4%	83.8%	76.6%
Brazil	Erechim	2000	80.8%	84.0%	77.5%
Brazil	Erechim	2017	81.4%	84.5%	78.2%
Brazil	Ererê	2000	74.5%	78.5%	70.3%
Brazil	Ererê	2017	75.2%	79.2%	71.1%
Brazil	Érico Cardoso	2000	78.7%	83.9%	72.8%
Brazil	Érico Cardoso	2017	79.3%	84.3%	73.5%
Brazil	Ermo	2000	77.6%	81.9%	73.0%
Brazil	Ermo	2017	78.3%	82.5%	73.7%
Brazil	Ernestina	2000	81.4%	84.9%	77.0%
Brazil	Ernestina	2017	82.0%	85.4%	77.8%
Brazil	Erval	2000	81.9%	86.8%	76.5%
Brazil	Erval	2017	82.5%	87.3%	77.3%
Brazil	Erval Grande	2000	77.7%	81.1%	73.5%
Brazil	Erval Grande	2017	78.3%	81.7%	74.3%
Brazil	Erval Seco	2000	79.6%	83.8%	74.9%
Brazil	Erval Seco	2017	80.1%	84.3%	75.6%
Brazil	Erval Velho	2000	76.0%	80.3%	71.3%
Brazil	Erval Velho	2017	76.7%	80.9%	72.1%
Brazil	Ervália	2000	83.4%	86.3%	79.4%
Brazil	Ervália	2017	83.9%	86.8%	80.1%
Brazil	Escada	2000	64.0%	67.0%	60.8%
Brazil	Escada	2017	64.9%	67.8%	61.7%
Brazil	Esmeralda	2000	77.9%	82.3%	74.1%
Brazil	Esmeralda	2017	78.6%	82.9%	74.8%

Country	Second Administrative Unit	Year	Mean	Upper	Lower
Brazil	Esmeraldas	2000	81.6%	84.4%	78.8%
Brazil	Esmeraldas	2017	82.4%	85.0%	79.6%
Brazil	Espera Feliz	2000	81.1%	84.4%	77.8%
Brazil	Espera Feliz	2017	81.7%	84.9%	78.5%
Brazil	Esperança	2000	70.7%	73.7%	67.7%
Brazil	Esperança	2017	71.5%	74.4%	68.5%
Brazil	Esperança do Sul	2000	79.0%	83.7%	73.7%
Brazil	Esperança do Sul	2017	79.7%	84.3%	74.5%
Brazil	Esperança Nova	2000	83.0%	86.3%	78.8%
Brazil	Esperança Nova	2017	83.5%	86.8%	79.5%
Brazil	Esperantina	2000	59.5%	63.9%	55.1%
Brazil	Esperantina	2000	53.3%	58.7%	47.8%
Brazil	Esperantina	2017	60.4%	64.7%	56.0%
Brazil	Esperantina	2017	54.3%	59.6%	48.8%
Brazil	Esperantinópolis	2000	58.8%	64.4%	53.7%
Brazil	Esperantinópolis	2017	59.7%	65.3%	54.7%
Brazil	Espigão Alto do Iguaçu	2000	83.2%	86.8%	79.2%
Brazil	Espigão Alto do Iguaçu	2017	83.7%	87.2%	79.9%
Brazil	Espigão d'Oeste	2000	31.8%	36.8%	27.3%
Brazil	Espigão d'Oeste	2017	32.6%	37.7%	28.1%
Brazil	Espinosa	2000	80.1%	85.3%	74.0%
Brazil	Espinosa	2017	80.7%	85.8%	74.8%
Brazil	Espírito Santo	2000	79.0%	81.4%	76.8%
Brazil	Espírito Santo	2017	79.6%	82.0%	77.5%
Brazil	Espírito Santo do Dourado	2000	87.4%	90.1%	84.1%
Brazil	Espírito Santo do Dourado	2017	87.9%	90.4%	84.7%
Brazil	Espírito Santo do Pinhal	2000	90.5%	92.5%	88.2%
Brazil	Espírito Santo do Pinhal	2017	90.8%	92.7%	88.6%
Brazil	Espírito Santo do Turvo	2000	92.3%	94.4%	90.0%
Brazil	Espírito Santo do Turvo	2017	92.6%	94.6%	90.4%
Brazil	Esplanada	2000	77.6%	81.9%	73.7%
Brazil	Esplanada	2017	78.4%	82.6%	74.5%
Brazil	Espumoso	2000	81.9%	85.5%	77.1%
Brazil	Espumoso	2017	82.5%	86.0%	77.8%
Brazil	Estação	2000	80.5%	82.8%	78.0%
Brazil	Estação	2017	81.8%	84.0%	79.6%
Brazil	Estância	2000	77.1%	79.9%	74.2%
Brazil	Estância	2017	77.8%	80.5%	75.0%
Brazil	Estância Velha	2000	81.0%	84.4%	77.1%
Brazil	Estância Velha	2017	81.6%	84.9%	77.8%
Brazil	Esteio	2000	82.5%	84.2%	80.7%
Brazil	Esteio	2017	83.0%	84.7%	81.3%
Brazil	Estiva	2000	89.9%	92.0%	87.6%
Brazil	Estiva	2017	90.3%	92.3%	88.0%
Brazil	Estiva Gerbi	2000	91.3%	93.0%	89.1%
Brazil	Estiva Gerbi	2017	91.6%	93.3%	89.5%

Country	Second Administrative Unit	Year	Mean	Upper	Lower
Brazil	Estreito	2000	71.4%	76.1%	66.4%
Brazil	Estreito	2017	72.2%	76.8%	67.2%
Brazil	Estrela	2000	80.6%	84.0%	77.1%
Brazil	Estrela	2017	81.3%	84.6%	77.9%
Brazil	Estrela dalva	2000	77.3%	80.8%	73.0%
Brazil	Estrela dalva	2017	78.0%	81.4%	73.7%
Brazil	Estrela de Alagoas	2000	62.5%	65.6%	59.2%
Brazil	Estrela de Alagoas	2017	63.4%	66.5%	60.1%
Brazil	Estrela do Indaiá	2000	81.0%	86.0%	74.6%
Brazil	Estrela do Indaiá	2017	81.6%	86.4%	75.4%
Brazil	Estrela do Norte	2000	86.6%	89.6%	83.2%
Brazil	Estrela do Norte	2000	76.9%	82.5%	71.0%
Brazil	Estrela do Norte	2017	87.1%	90.0%	83.8%
Brazil	Estrela do Norte	2017	77.5%	83.1%	71.8%
Brazil	Estrela do Oeste	2000	89.2%	92.4%	86.0%
Brazil	Estrela do Oeste	2017	89.6%	92.7%	86.5%
Brazil	Estrela do Sul	2000	80.3%	84.9%	74.9%
Brazil	Estrela do Sul	2017	80.9%	85.4%	75.6%
Brazil	Estrela Velha	2000	82.2%	85.8%	78.1%
Brazil	Estrela Velha	2017	82.8%	86.3%	78.8%
Brazil	Euclides da Cunha	2000	77.6%	82.6%	71.9%
Brazil	Euclides da Cunha	2017	78.3%	83.1%	72.7%
Brazil	Euclides da Cunha Paulista	2000	83.6%	87.2%	79.2%
Brazil	Euclides da Cunha Paulista	2017	84.1%	87.6%	79.9%
Brazil	Eugênio de Castro	2000	82.3%	86.2%	77.6%
Brazil	Eugênio de Castro	2017	82.8%	86.6%	78.3%
Brazil	Eugenópolis	2000	80.0%	83.5%	76.2%
Brazil	Eugenópolis	2017	80.6%	84.0%	76.9%
Brazil	Eunápolis	2000	78.3%	83.1%	72.0%
Brazil	Eunápolis	2017	79.0%	83.7%	72.8%
Brazil	Eusébio	2000	68.1%	70.1%	66.0%
Brazil	Eusébio	2017	69.6%	71.6%	67.5%
Brazil	Ewbank da Câmara	2000	79.9%	83.1%	75.8%
Brazil	Ewbank da Câmara	2017	80.5%	83.6%	76.5%
Brazil	Extrema	2000	93.5%	94.7%	92.1%
Brazil	Extrema	2017	93.8%	94.9%	92.4%
Brazil	Extremoz	2000	81.2%	83.3%	79.1%
Brazil	Extremoz	2017	81.8%	83.9%	79.8%
Brazil	Exu	2000	69.1%	73.0%	64.1%
Brazil	Exu	2017	69.9%	73.8%	65.0%
Brazil	Fagundes	2000	64.1%	67.3%	61.3%
Brazil	Fagundes	2017	65.0%	68.1%	62.2%

Country	Second Administrative Unit	Year	Mean	Upper	Lower
Brazil	Fagundes Varela	2000	81.5%	84.9%	78.1%
Brazil	Fagundes Varela	2017	82.1%	85.4%	78.7%
Brazil	Faina	2000	75.1%	79.7%	69.6%
Brazil	Faina	2017	75.8%	80.4%	70.4%
Brazil	Fama	2000	83.6%	87.1%	79.1%
Brazil	Fama	2017	84.1%	87.5%	79.8%
Brazil	Faria Lemos	2000	80.3%	83.6%	76.8%
Brazil	Faria Lemos	2017	80.9%	84.1%	77.4%
Brazil	Farias Brito	2000	69.8%	73.7%	65.6%
Brazil	Farias Brito	2017	70.6%	74.4%	66.5%
Brazil	Faro	2000	50.2%	56.6%	44.3%
Brazil	Faro	2017	51.2%	57.4%	45.3%
Brazil	Farol	2000	84.4%	87.5%	80.6%
Brazil	Farol	2017	84.9%	87.9%	81.2%
Brazil	Farroupilha	2000	84.1%	86.6%	81.3%
Brazil	Farroupilha	2017	84.7%	87.1%	81.9%
Brazil	Fartura	2000	90.9%	93.2%	87.8%
Brazil	Fartura	2017	91.3%	93.5%	88.3%
Brazil	Fartura do Piauí	2000	66.2%	72.1%	58.2%
Brazil	Fartura do Piauí	2017	67.0%	72.8%	59.2%
Brazil	Fátima	2000	77.2%	81.0%	72.9%
Brazil	Fátima	2000	75.5%	80.5%	70.9%
Brazil	Fátima	2017	76.2%	81.1%	71.7%
Brazil	Fátima	2017	77.9%	81.6%	73.6%
Brazil	Fatima do Sul	2000	80.9%	84.1%	77.5%
Brazil	Fatima do Sul	2017	81.5%	84.6%	78.2%
Brazil	Faxinal	2000	85.0%	88.2%	81.3%
Brazil	Faxinal	2017	85.5%	88.6%	81.9%
Brazil	Faxinal do Soturno	2000	81.3%	84.7%	76.8%
Brazil	Faxinal do Soturno	2017	81.9%	85.2%	77.5%
Brazil	Faxinal dos Guedes	2000	76.8%	81.2%	72.2%
Brazil	Faxinal dos Guedes	2017	77.5%	81.8%	73.0%
Brazil	Faxinalzinho	2000	77.8%	81.2%	73.7%
Brazil	Faxinalzinho	2017	78.4%	81.7%	74.4%
Brazil	Fazenda Nova	2000	75.0%	79.5%	69.4%
Brazil	Fazenda Nova	2017	75.7%	80.1%	70.3%
Brazil	Fazenda Rio Grande	2000	84.7%	86.5%	82.8%
Brazil	Fazenda Rio Grande	2017	85.2%	87.0%	83.3%
Brazil	Fazenda Vilanova	2000	81.0%	84.1%	77.5%
Brazil	Fazenda Vilanova	2017	81.5%	84.6%	78.2%
Brazil	Feijó	2000	48.1%	52.4%	43.8%
Brazil	Feijó	2017	48.9%	53.2%	44.7%
Brazil	Feira da Mata	2000	78.3%	84.5%	70.1%
Brazil	Feira da Mata	2017	78.9%	85.0%	70.9%
Brazil	Feira de Santana	2000	79.0%	81.9%	75.8%
Brazil	Feira de Santana	2017	79.6%	82.4%	76.5%
Brazil	Feira Grande	2000	63.5%	66.6%	60.9%
Brazil	Feira Grande	2017	64.4%	67.4%	61.8%

Country	Second Administrative Unit	Year	Mean	Upper	Lower
Brazil	Feira Nova	2000	74.5%	77.3%	71.4%
Brazil	Feira Nova	2000	67.2%	70.1%	64.4%
Brazil	Feira Nova	2017	75.4%	78.1%	72.4%
Brazil	Feira Nova	2017	68.0%	70.9%	65.2%
Brazil	Feira Nova do Maranhão	2000	70.3%	76.6%	62.9%
Brazil	Feira Nova do Maranhão	2017	71.1%	77.3%	63.8%
Brazil	Felício dos Santos	2000	82.6%	87.0%	77.0%
Brazil	Felício dos Santos	2017	83.1%	87.4%	77.7%
Brazil	Felipe Guerra	2000	78.9%	82.0%	75.4%
Brazil	Felipe Guerra	2017	79.5%	82.6%	76.1%
Brazil	Felisberto Caldeira	2000	82.4%	87.2%	76.8%
Brazil	Felisberto Caldeira	2017	83.0%	87.7%	77.4%
Brazil	Felisburgo	2000	82.6%	86.8%	77.4%
Brazil	Felisburgo	2017	83.2%	87.2%	78.1%
Brazil	Felixlândia	2000	81.8%	86.7%	75.3%
Brazil	Felixlândia	2017	82.3%	87.2%	76.0%
Brazil	Feliz	2000	80.0%	82.9%	77.4%
Brazil	Feliz	2017	80.6%	83.5%	78.1%
Brazil	Feliz Deserto	2000	61.6%	65.9%	57.3%
Brazil	Feliz Deserto	2017	62.5%	66.8%	58.3%
Brazil	Feliz Natal	2000	68.5%	74.4%	61.1%
Brazil	Feliz Natal	2017	70.8%	76.5%	63.7%
Brazil	Fênix	2000	81.8%	85.0%	78.0%
Brazil	Fênix	2017	82.4%	85.5%	78.7%
Brazil	Fernandes Pinheiro	2000	81.8%	85.4%	78.0%
Brazil	Fernandes Pinheiro	2017	82.4%	85.9%	78.6%
Brazil	Fernandes Tourinho	2000	80.0%	84.4%	75.5%
Brazil	Fernandes Tourinho	2017	80.6%	84.9%	76.2%
Brazil	Fernando de Noronha	2000	78.0%	91.8%	56.8%
Brazil	Fernando de Noronha	2017	78.6%	92.1%	57.8%
Brazil	Fernando Falcão	2000	61.4%	68.0%	53.8%
Brazil	Fernando Falcão	2017	62.3%	68.8%	54.8%
Brazil	Fernando Pedroza	2000	83.4%	86.2%	80.3%
Brazil	Fernando Pedroza	2017	84.0%	86.7%	80.9%
Brazil	Fernando Prestes	2000	93.2%	94.8%	91.2%
Brazil	Fernando Prestes	2017	93.5%	95.0%	91.5%
Brazil	Fernandópolis	2000	89.7%	92.7%	86.7%
Brazil	Fernandópolis	2017	90.1%	93.0%	87.2%
Brazil	Ferno	2000	92.9%	94.7%	90.8%
Brazil	Ferno	2017	93.1%	94.9%	91.1%
Brazil	Ferraz de Vascon	2000	94.7%	95.4%	93.9%
Brazil	Ferraz de Vascon	2017	94.9%	95.6%	94.1%

Country	Second Administrative Unit	Year	Mean	Upper	Lower
Brazil	Ferreira Gomes	2000	48.2%	52.3%	43.2%
Brazil	Ferreira Gomes	2017	49.1%	53.3%	44.2%
Brazil	Ferreiros	2000	70.2%	73.1%	67.4%
Brazil	Ferreiros	2017	71.1%	73.9%	68.3%
Brazil	Ferros	2000	80.8%	84.3%	76.5%
Brazil	Ferros	2017	81.4%	84.9%	77.2%
Brazil	Fervedouro	2000	82.0%	85.5%	78.4%
Brazil	Fervedouro	2017	82.6%	86.0%	79.0%
Brazil	Figueira	2000	85.5%	88.7%	82.1%
Brazil	Figueira	2017	86.0%	89.1%	82.7%
Brazil	Figueirópolis	2000	73.7%	78.3%	69.5%
Brazil	Figueirópolis	2017	74.4%	79.0%	70.3%
Brazil	Figueirópolis d'Oeste	2000	67.7%	74.9%	60.6%
Brazil	Figueirópolis d'Oeste	2017	68.5%	75.6%	61.5%
Brazil	Filadélfia	2000	75.1%	80.4%	68.3%
Brazil	Filadélfia	2000	75.4%	80.6%	70.5%
Brazil	Filadélfia	2017	75.8%	81.0%	69.2%
Brazil	Filadélfia	2017	76.1%	81.2%	71.3%
Brazil	Firmino Alves	2000	79.0%	82.7%	73.3%
Brazil	Firmino Alves	2017	79.6%	83.3%	74.1%
Brazil	Firminópolis	2000	76.3%	80.2%	71.9%
Brazil	Firminópolis	2017	77.0%	80.8%	72.7%
Brazil	Flexeiras	2000	60.5%	63.1%	57.7%
Brazil	Flexeiras	2017	61.4%	64.0%	58.7%
Brazil	Flor da Serra do Sul	2000	81.7%	85.8%	76.8%
Brazil	Flor da Serra do Sul	2017	82.3%	86.2%	77.5%
Brazil	Flor do Sertão	2000	77.7%	82.1%	73.1%
Brazil	Flor do Sertão	2017	78.3%	82.6%	73.8%
Brazil	Flora Rica	2000	88.8%	91.3%	85.7%
Brazil	Flora Rica	2017	89.1%	91.6%	86.1%
Brazil	Floraí	2000	83.9%	86.9%	80.2%
Brazil	Floraí	2017	84.4%	87.3%	80.9%
Brazil	Florânia	2000	81.1%	84.0%	77.9%
Brazil	Florânia	2017	81.7%	84.6%	78.7%
Brazil	Floreal	2000	91.0%	93.7%	87.9%
Brazil	Floreal	2017	91.3%	93.9%	88.3%
Brazil	Flores	2000	67.1%	71.4%	61.7%
Brazil	Flores	2017	68.0%	72.2%	62.6%
Brazil	Flores da Cunha	2000	81.6%	84.6%	78.0%
Brazil	Flores da Cunha	2017	82.1%	85.1%	78.6%
Brazil	Flores de Goiás	2000	82.8%	86.4%	78.0%
Brazil	Flores de Goiás	2017	83.3%	86.9%	78.6%
Brazil	Flores do Piauí	2000	62.9%	68.9%	56.3%
Brazil	Flores do Piauí	2017	63.8%	69.7%	57.3%
Brazil	Floresta	2000	83.0%	85.9%	79.7%
Brazil	Floresta	2000	64.4%	70.3%	58.4%
Brazil	Floresta	2017	83.6%	86.4%	80.3%
Brazil	Floresta	2017	65.3%	71.1%	59.4%
Brazil	Floresta Azul	2000	77.2%	81.1%	72.3%
Brazil	Floresta Azul	2017	78.0%	81.8%	73.1%

Country	Second Administrative Unit	Year	Mean	Upper	Lower
Brazil	Floresta do Araguaia	2000	49.9%	55.8%	43.9%
Brazil	Floresta do Araguaia	2017	50.9%	56.8%	44.9%
Brazil	Floresta do Piauí	2000	60.6%	65.8%	54.6%
Brazil	Floresta do Piauí	2017	61.5%	66.7%	55.6%
Brazil	Florestal	2000	80.7%	84.1%	76.9%
Brazil	Florestal	2017	82.5%	85.6%	78.9%
Brazil	Florestópolis	2000	86.5%	89.2%	83.6%
Brazil	Florestópolis	2017	87.0%	89.6%	84.1%
Brazil	Floriano	2000	61.5%	66.3%	55.6%
Brazil	Floriano	2017	62.4%	67.2%	56.6%
Brazil	Floriano Peixoto	2000	78.2%	82.2%	74.1%
Brazil	Floriano Peixoto	2017	78.9%	82.8%	74.9%
Brazil	Florianopolis	2000	72.8%	78.8%	66.5%
Brazil	Florianopolis	2017	73.6%	79.5%	67.4%
Brazil	Flórida	2000	85.1%	87.8%	82.3%
Brazil	Flórida	2017	85.6%	88.2%	82.8%
Brazil	Flórida Paulista	2000	89.6%	92.0%	86.5%
Brazil	Flórida Paulista	2017	90.0%	92.3%	87.0%
Brazil	Florínia	2000	87.9%	90.3%	85.1%
Brazil	Florínia	2017	88.3%	90.6%	85.6%
Brazil	Floriniapolis	2000	73.1%	78.7%	67.3%
Brazil	Floriniapolis	2017	73.8%	79.4%	68.1%
Brazil	Fonte Boa	2000	60.6%	68.1%	52.8%
Brazil	Fonte Boa	2017	61.4%	68.8%	53.7%
Brazil	Fontoura Xavier	2000	83.2%	86.4%	79.5%
Brazil	Fontoura Xavier	2017	83.8%	86.8%	80.1%
Brazil	Formiga	2000	82.6%	86.2%	77.6%
Brazil	Formiga	2017	83.1%	86.7%	78.3%
Brazil	Formigueiro	2000	81.7%	85.5%	77.2%
Brazil	Formigueiro	2017	82.3%	85.9%	77.9%
Brazil	Formosa	2000	89.9%	92.0%	87.5%
Brazil	Formosa	2017	90.3%	92.3%	87.9%
Brazil	Formosa da Serra Negra	2000	67.2%	73.7%	60.4%
Brazil	Formosa da Serra Negra	2017	68.1%	74.5%	61.4%
Brazil	Formosa do Oeste	2000	84.1%	87.7%	80.2%
Brazil	Formosa do Oeste	2017	83.6%	87.3%	79.7%
Brazil	Formosa do Rio Preto	2000	72.7%	79.6%	65.7%
Brazil	Formosa do Rio Preto	2017	73.5%	80.2%	66.5%
Brazil	Formosa do Sul	2000	77.6%	82.0%	73.4%
Brazil	Formosa do Sul	2017	78.3%	82.5%	74.1%
Brazil	Formoso	2000	78.5%	84.0%	73.1%
Brazil	Formoso	2000	84.0%	88.2%	78.6%
Brazil	Formoso	2017	79.2%	84.5%	73.9%
Brazil	Formoso	2017	84.5%	88.6%	79.2%

Country	Second Administrative Unit	Year	Mean	Upper	Lower
Brazil	Formoso do Araguaia	2000	74.8%	79.4%	69.9%
Brazil	Formoso do Araguaia	2017	75.5%	80.0%	70.7%
Brazil	Forquilha	2000	70.3%	74.4%	66.0%
Brazil	Forquilha	2017	71.2%	75.2%	66.9%
Brazil	Forquilhinha	2000	77.5%	81.4%	73.5%
Brazil	Forquilhinha	2017	78.2%	82.0%	74.3%
Brazil	Fortaleza	2000	69.0%	71.1%	66.8%
Brazil	Fortaleza	2017	69.8%	71.9%	67.7%
Brazil	Fortaleza de Minas	2000	86.7%	90.0%	82.7%
Brazil	Fortaleza de Minas	2017	87.1%	90.4%	83.3%
Brazil	Fortaleza do Tabocão	2000	75.3%	79.9%	71.3%
Brazil	Fortaleza do Tabocão	2017	76.1%	80.5%	72.1%
Brazil	Fortaleza dos Nogueiras	2000	68.8%	75.0%	60.5%
Brazil	Fortaleza dos Nogueiras	2017	69.6%	75.8%	61.5%
Brazil	Fortaleza dos Valos	2000	82.5%	86.0%	77.8%
Brazil	Fortaleza dos Valos	2017	83.1%	86.4%	78.4%
Brazil	Fortim	2000	71.0%	75.2%	66.2%
Brazil	Fortim	2017	71.8%	76.0%	67.1%
Brazil	Fortuna	2000	61.6%	67.5%	55.0%
Brazil	Fortuna	2017	62.5%	68.3%	56.0%
Brazil	Fortuna de Minas	2000	79.8%	83.6%	75.2%
Brazil	Fortuna de Minas	2017	80.5%	84.1%	76.0%
Brazil	Foz do Iguaçu	2000	82.8%	87.0%	78.1%
Brazil	Foz do Iguaçu	2017	83.3%	87.4%	78.6%
Brazil	Foz do Jordão	2000	81.3%	85.3%	77.0%
Brazil	Foz do Jordão	2017	81.9%	85.8%	77.7%
Brazil	Fraiburgo	2000	78.1%	82.4%	73.9%
Brazil	Fraiburgo	2017	78.7%	82.9%	74.6%
Brazil	Franca	2000	88.9%	91.8%	85.1%
Brazil	Franca	2017	89.3%	92.1%	85.6%
Brazil	Francinópolis	2000	59.4%	64.1%	54.4%
Brazil	Francinópolis	2017	60.4%	65.0%	55.4%
Brazil	Francisco Alves	2000	83.1%	86.3%	79.0%
Brazil	Francisco Alves	2017	83.6%	86.8%	79.7%
Brazil	Francisco Ayres	2000	58.8%	63.8%	53.4%
Brazil	Francisco Ayres	2017	59.8%	64.7%	54.5%
Brazil	Francisco Badaró	2000	81.9%	87.1%	76.9%
Brazil	Francisco Badaró	2017	82.5%	87.5%	77.6%
Brazil	Francisco Beltrão	2000	83.0%	86.9%	78.7%
Brazil	Francisco Beltrão	2017	83.5%	87.4%	79.4%
Brazil	Francisco Dantas	2000	78.5%	81.5%	75.4%

Country	Second Administrative Unit	Year	Mean	Upper	Lower
Brazil	Francisco Dantas	2017	79.2%	82.1%	76.1%
Brazil	Francisco Dumon	2000	81.9%	86.5%	75.6%
Brazil	Francisco Dumon	2017	82.4%	86.9%	76.3%
Brazil	Francisco Macêdo	2000	64.5%	69.0%	60.0%
Brazil	Francisco Macêdo	2017	65.4%	69.9%	60.9%
Brazil	Francisco Morato	2000	94.2%	95.0%	93.2%
Brazil	Francisco Morato	2017	94.4%	95.2%	93.4%
Brazil	Francisco Sá	2000	81.7%	85.2%	77.3%
Brazil	Francisco Sá	2017	82.3%	85.7%	77.9%
Brazil	Francisco Santos	2000	62.7%	67.2%	58.8%
Brazil	Francisco Santos	2017	63.6%	68.1%	59.7%
Brazil	Franciscópolis	2000	83.1%	87.5%	78.2%
Brazil	Franciscópolis	2017	83.6%	87.9%	78.8%
Brazil	Franco da Rocha	2000	94.1%	94.9%	93.1%
Brazil	Franco da Rocha	2017	94.3%	95.1%	93.4%
Brazil	Frecheirinha	2000	67.7%	71.7%	62.8%
Brazil	Frecheirinha	2017	68.5%	72.5%	63.7%
Brazil	Frederico Westphalen	2000	79.7%	83.4%	74.8%
Brazil	Frederico Westphalen	2017	80.4%	84.0%	75.6%
Brazil	Frei Gaspar	2000	81.8%	86.6%	76.1%
Brazil	Frei Gaspar	2017	82.4%	87.1%	76.8%
Brazil	Frei Inocêncio	2000	79.2%	84.4%	74.2%
Brazil	Frei Inocêncio	2017	79.9%	84.9%	75.0%
Brazil	Frei Lagonegro	2000	82.3%	86.7%	76.1%
Brazil	Frei Lagonegro	2017	82.8%	87.1%	76.9%
Brazil	Frei Martinho	2000	80.8%	83.5%	77.5%
Brazil	Frei Martinho	2017	81.4%	84.0%	78.2%
Brazil	Frei Miguelinho	2000	65.7%	69.0%	62.3%
Brazil	Frei Miguelinho	2017	66.6%	69.8%	63.3%
Brazil	Frei Paulo	2000	78.7%	81.1%	76.2%
Brazil	Frei Paulo	2017	79.3%	81.7%	76.9%
Brazil	Frei Rogério	2000	75.3%	80.1%	70.1%
Brazil	Frei Rogério	2017	76.0%	80.7%	70.9%
Brazil	Fronteira	2000	90.8%	93.1%	87.8%
Brazil	Fronteira	2017	91.1%	93.4%	88.3%
Brazil	Fronteira dos Vales	2000	81.3%	85.8%	76.2%
Brazil	Fronteira dos Vales	2017	81.8%	86.3%	76.8%
Brazil	Fronteiras	2000	66.8%	71.4%	62.1%
Brazil	Fronteiras	2017	67.7%	72.2%	63.0%
Brazil	Fruta de Leite	2000	81.3%	86.2%	75.7%
Brazil	Fruta de Leite	2017	81.9%	86.7%	76.3%
Brazil	Frutal	2000	89.1%	91.8%	85.7%
Brazil	Frutal	2017	89.5%	92.1%	86.2%

Country	Second Administrative Unit	Year	Mean	Upper	Lower
Brazil	Frutuoso Gomes	2000	77.8%	81.1%	74.5%
Brazil	Frutuoso Gomes	2017	78.4%	81.6%	75.1%
Brazil	Fundão	2000	75.8%	78.5%	73.1%
Brazil	Fundão	2017	76.5%	79.1%	73.8%
Brazil	Funilândia	2000	80.5%	84.2%	76.2%
Brazil	Funilândia	2017	81.1%	84.7%	76.9%
Brazil	Gabriel Monteiro	2000	91.5%	93.8%	88.9%
Brazil	Gabriel Monteiro	2017	91.8%	94.0%	89.3%
Brazil	Gado Bravo	2000	64.2%	67.8%	60.9%
Brazil	Gado Bravo	2017	65.1%	68.6%	61.8%
Brazil	Gália	2000	92.8%	94.7%	90.8%
Brazil	Gália	2017	93.1%	94.9%	91.1%
Brazil	Galiléia	2000	78.5%	83.1%	74.0%
Brazil	Galiléia	2017	79.1%	83.6%	74.6%
Brazil	Galinhos	2000	82.3%	85.6%	78.2%
Brazil	Galinhos	2017	82.8%	86.1%	78.8%
Brazil	Galvão	2000	79.2%	83.3%	75.2%
Brazil	Galvão	2017	79.9%	83.8%	75.9%
Brazil	Gameleira	2000	62.1%	65.8%	58.4%
Brazil	Gameleira	2017	63.0%	66.7%	59.4%
Brazil	Gameleiras	2000	79.5%	85.0%	73.0%
Brazil	Gameleiras	2017	80.1%	85.6%	73.7%
Brazil	Gandu	2000	76.8%	81.2%	72.6%
Brazil	Gandu	2017	77.4%	81.8%	73.3%
Brazil	Garanhuns	2000	66.3%	69.5%	62.8%
Brazil	Garanhuns	2017	67.2%	70.4%	63.8%
Brazil	Gararu	2000	68.1%	71.0%	64.5%
Brazil	Gararu	2017	68.9%	71.8%	65.3%
Brazil	Garça	2000	93.3%	95.0%	91.0%
Brazil	Garça	2017	93.5%	95.2%	91.3%
Brazil	Garibaldi	2000	84.1%	86.6%	81.4%
Brazil	Garibaldi	2017	84.6%	87.0%	82.0%
Brazil	Garopaba	2000	71.3%	76.7%	64.4%
Brazil	Garopaba	2017	72.5%	77.8%	65.8%
Brazil	Garrafão do Norte	2000	40.7%	46.4%	36.0%
Brazil	Garrafão do Norte	2017	41.7%	47.4%	36.9%
Brazil	Garruchos	2000	80.6%	86.4%	73.4%
Brazil	Garruchos	2017	81.2%	86.9%	74.1%
Brazil	Garuva	2000	76.1%	79.7%	71.9%
Brazil	Garuva	2017	76.8%	80.3%	72.7%
Brazil	Gaspar	2000	72.7%	75.8%	69.0%
Brazil	Gaspar	2017	74.0%	77.1%	70.5%
Brazil	Gastão Vidigal	2000	91.0%	93.6%	87.9%
Brazil	Gastão Vidigal	2017	91.3%	93.8%	88.3%
Brazil	Gaúcha do Norte	2000	66.2%	74.3%	58.2%
Brazil	Gaúcha do Norte	2017	67.1%	75.0%	59.2%
Brazil	Gaurama	2000	78.6%	82.3%	74.6%
Brazil	Gaurama	2017	79.3%	82.9%	75.3%
Brazil	Gavião	2000	77.0%	81.1%	71.6%
Brazil	Gavião	2017	77.6%	81.7%	72.3%
Brazil	Gavião Peixoto	2000	93.4%	95.0%	91.2%

Country	Second Administrative Unit	Year	Mean	Upper	Lower
Brazil	Gavião Peixoto	2017	93.7%	95.2%	91.5%
Brazil	Geminiano	2000	61.2%	66.0%	56.9%
Brazil	Geminiano	2017	62.1%	66.8%	57.8%
Brazil	General Câmara	2000	80.6%	83.8%	77.2%
Brazil	General Câmara	2017	81.2%	84.4%	77.9%
Brazil	General Carneiro	2000	81.0%	84.9%	76.8%
Brazil	General Carneiro	2000	69.6%	75.2%	63.0%
Brazil	General Carneiro	2017	70.4%	75.8%	63.8%
Brazil	General Carneiro	2017	81.6%	85.4%	77.5%
Brazil	General Maynard	2000	72.2%	74.6%	69.4%
Brazil	General Maynard	2017	72.9%	75.3%	70.2%
Brazil	General Salgado	2000	90.4%	93.1%	87.4%
Brazil	General Salgado	2017	90.8%	93.3%	87.8%
Brazil	General Sampaio	2000	70.5%	74.1%	66.6%
Brazil	General Sampaio	2017	71.4%	74.8%	67.4%
Brazil	Gentil	2000	81.1%	84.5%	76.2%
Brazil	Gentil	2017	81.6%	85.0%	76.8%
Brazil	Gentio do Ouro	2000	76.2%	82.3%	68.8%
Brazil	Gentio do Ouro	2017	76.9%	82.9%	69.6%
Brazil	Getulina	2000	93.2%	95.1%	90.8%
Brazil	Getulina	2017	93.4%	95.3%	91.1%
Brazil	Getúlio Vargas	2000	81.1%	84.5%	77.3%
Brazil	Getúlio Vargas	2017	81.7%	85.1%	78.0%
Brazil	Gilbués	2000	65.4%	72.1%	58.0%
Brazil	Gilbués	2017	66.3%	72.9%	59.0%
Brazil	Girau do Ponciano	2000	66.7%	69.6%	63.8%
Brazil	Girau do Ponciano	2017	67.6%	70.5%	64.7%
Brazil	Giruá	2000	82.7%	86.8%	78.1%
Brazil	Giruá	2017	83.2%	87.3%	78.8%
Brazil	Glaucilândia	2000	81.9%	86.6%	76.3%
Brazil	Glaucilândia	2017	82.6%	87.1%	77.1%
Brazil	Glicério	2000	92.1%	94.1%	89.4%
Brazil	Glicério	2017	92.4%	94.3%	89.8%
Brazil	Glória	2000	64.8%	70.4%	59.2%
Brazil	Glória	2017	65.7%	71.2%	60.1%
Brazil	Glória d'Oeste	2000	68.3%	74.8%	60.3%
Brazil	Glória d'Oeste	2017	69.1%	75.5%	61.3%
Brazil	Glória de Dourados	2000	80.9%	84.5%	76.9%
Brazil	Glória de Dourados	2017	81.5%	85.1%	77.6%
Brazil	Glória do Goitá	2000	68.2%	70.8%	65.7%

Country	Second Administrative Unit	Year	Mean	Upper	Lower
Brazil	Glória do Goitá	2017	69.0%	71.6%	66.5%
Brazil	Glorinha	2000	81.6%	84.1%	78.9%
Brazil	Glorinha	2017	82.2%	84.6%	79.6%
Brazil	Godofredo Viana	2000	45.3%	52.9%	38.2%
Brazil	Godofredo Viana	2017	47.4%	55.0%	40.2%
Brazil	Godoy Moreira	2000	82.6%	86.1%	78.2%
Brazil	Godoy Moreira	2017	83.1%	86.5%	78.9%
Brazil	Goiabeira	2000	76.1%	80.6%	71.3%
Brazil	Goiabeira	2017	76.8%	81.2%	72.1%
Brazil	Goianá	2000	79.7%	82.9%	75.8%
Brazil	Goianá	2000	65.7%	68.7%	62.3%
Brazil	Goianá	2017	80.3%	83.4%	76.5%
Brazil	Goianá	2017	66.6%	69.5%	63.2%
Brazil	Goianápolis	2000	80.0%	82.1%	77.6%
Brazil	Goianápolis	2017	80.6%	82.6%	78.3%
Brazil	Goiandira	2000	78.0%	82.7%	72.5%
Brazil	Goiandira	2017	78.7%	83.2%	73.2%
Brazil	Goianésia	2000	82.4%	85.9%	78.3%
Brazil	Goianésia	2017	82.9%	86.4%	79.0%
Brazil	Goianésia do Pará	2000	40.3%	45.9%	33.8%
Brazil	Goianésia do Pará	2017	41.4%	47.1%	34.8%
Brazil	Goiania	2000	78.6%	80.5%	76.7%
Brazil	Goiania	2017	79.2%	81.1%	77.5%
Brazil	Goianinha	2000	81.3%	83.6%	79.3%
Brazil	Goianinha	2017	82.0%	84.2%	79.9%
Brazil	Goianira	2000	77.4%	79.9%	75.2%
Brazil	Goianira	2017	78.1%	80.5%	75.9%
Brazil	Goianorte	2000	69.6%	74.8%	64.3%
Brazil	Goianorte	2017	70.3%	75.4%	65.1%
Brazil	Goiás	2000	76.6%	80.8%	72.2%
Brazil	Goiás	2017	77.3%	81.4%	73.0%
Brazil	Goiatins	2000	74.3%	79.3%	69.1%
Brazil	Goiatins	2017	75.0%	79.8%	69.9%
Brazil	Goiatuba	2000	79.3%	83.3%	75.0%
Brazil	Goiatuba	2017	79.9%	83.9%	75.7%
Brazil	Goioerê	2000	84.9%	88.4%	81.6%
Brazil	Goioerê	2017	85.4%	88.8%	82.2%
Brazil	Goioxim	2000	83.4%	87.1%	79.3%
Brazil	Goioxim	2017	83.9%	87.5%	80.0%
Brazil	Gonçalves	2000	91.3%	93.1%	89.3%
Brazil	Gonçalves	2017	91.6%	93.3%	89.6%
Brazil	Gonçalves Dias	2000	62.1%	68.1%	57.5%
Brazil	Gonçalves Dias	2017	63.0%	68.9%	58.5%
Brazil	Gongogi	2000	76.0%	80.6%	71.4%
Brazil	Gongogi	2017	75.9%	80.7%	71.3%
Brazil	Gonzaga	2000	82.6%	86.6%	78.2%
Brazil	Gonzaga	2017	83.1%	87.0%	78.8%
Brazil	Gouvea	2000	84.5%	88.9%	79.0%
Brazil	Gouvea	2017	85.1%	89.3%	79.7%
Brazil	Gouvelândia	2000	78.5%	83.3%	73.4%
Brazil	Gouvelândia	2017	79.2%	83.8%	74.2%
Brazil	Governador Archer	2000	62.7%	68.6%	57.7%

Country	Second Administrative Unit	Year	Mean	Upper	Lower
Brazil	Governador Archer	2017	63.6%	69.5%	58.6%
Brazil	Governador Celso Ramos	2000	71.2%	76.5%	65.3%
Brazil	Governador Celso Ramos	2017	72.0%	77.2%	66.2%
Brazil	Governador Dix-Sept Rosad	2000	80.5%	83.1%	77.5%
Brazil	Governador Dix-Sept Rosad	2017	81.1%	83.6%	78.1%
Brazil	Governador Edison Lobão	2000	67.4%	71.8%	62.9%
Brazil	Governador Edison Lobão	2017	68.3%	72.7%	63.9%
Brazil	Governador Eugênio Barros	2000	61.6%	67.2%	56.7%
Brazil	Governador Eugênio Barros	2017	62.6%	68.1%	57.6%
Brazil	Governador Jorge Teixeira	2000	28.8%	33.3%	24.7%
Brazil	Governador Jorge Teixeira	2017	29.6%	34.2%	25.4%
Brazil	Governador Luiz Rocha	2000	60.8%	66.6%	54.7%
Brazil	Governador Luiz Rocha	2017	63.5%	69.1%	57.6%
Brazil	Governador Mangabeira	2000	76.6%	80.0%	72.7%
Brazil	Governador Mangabeira	2017	77.2%	80.6%	73.4%
Brazil	Governador Newton Bello	2000	59.0%	63.9%	53.7%
Brazil	Governador Newton Bello	2017	59.9%	64.8%	54.7%
Brazil	Governador Nunes Freire	2000	51.9%	57.9%	46.7%
Brazil	Governador Nunes Freire	2017	52.6%	58.6%	47.5%
Brazil	Governador Valadares	2000	80.1%	84.7%	75.9%
Brazil	Governador Valadares	2017	80.8%	85.2%	76.6%
Brazil	Graça	2000	67.4%	71.8%	63.5%
Brazil	Graça	2017	68.3%	72.6%	64.5%
Brazil	Graça Aranha	2000	61.8%	68.3%	56.5%
Brazil	Graça Aranha	2017	62.7%	69.2%	57.5%
Brazil	Gracho Cardoso	2000	71.9%	74.8%	68.6%
Brazil	Gracho Cardoso	2017	72.7%	75.5%	69.5%
Brazil	Grajaú	2000	63.0%	69.2%	56.4%
Brazil	Grajaú	2017	65.1%	71.1%	58.6%
Brazil	Gramado	2000	81.9%	84.6%	78.8%
Brazil	Gramado	2017	82.5%	85.1%	79.4%
Brazil	Gramado dos Loureiros	2000	78.9%	82.5%	74.7%
Brazil	Gramado dos Loureiros	2017	79.5%	83.0%	75.5%

Country	Second Administrative Unit	Year	Mean	Upper	Lower
Brazil	Gramado Xavier	2000	83.7%	86.9%	80.0%
Brazil	Gramado Xavier	2017	84.1%	87.4%	80.5%
Brazil	Grandes Rios	2000	83.3%	86.8%	79.3%
Brazil	Grandes Rios	2017	83.9%	87.2%	79.9%
Brazil	Granito	2000	68.6%	72.8%	63.4%
Brazil	Granito	2017	69.4%	73.6%	64.3%
Brazil	Granja	2000	67.5%	71.8%	62.5%
Brazil	Granja	2017	68.4%	72.6%	63.4%
Brazil	Granjeiro	2000	68.9%	72.7%	65.2%
Brazil	Granjeiro	2017	69.8%	73.5%	66.1%
Brazil	Grão Mogol	2000	82.3%	87.1%	76.6%
Brazil	Grão Mogol	2017	82.9%	87.5%	77.3%
Brazil	Grão Pará	2000	73.4%	77.4%	69.1%
Brazil	Grão Pará	2017	74.1%	78.0%	69.9%
Brazil	Gravatá	2000	72.4%	75.4%	69.6%
Brazil	Gravatá	2017	73.2%	76.2%	70.4%
Brazil	Gravataí	2000	73.2%	77.7%	68.2%
Brazil	Gravataí	2000	82.2%	84.0%	80.2%
Brazil	Gravataí	2017	74.1%	78.4%	69.1%
Brazil	Gravataí	2017	82.7%	84.5%	80.7%
Brazil	Groaíras	2000	80.1%	83.4%	76.9%
Brazil	Groaíras	2000	68.0%	72.0%	63.1%
Brazil	Groaíras	2017	68.8%	72.7%	64.1%
Brazil	Groaíras	2017	80.7%	84.0%	77.5%
Brazil	Grupiara	2000	78.1%	83.0%	72.3%
Brazil	Grupiara	2017	78.8%	83.6%	73.1%
Brazil	Guabiju	2000	80.6%	84.3%	76.5%
Brazil	Guabiju	2017	81.2%	84.8%	77.2%
Brazil	Guabiruba	2000	70.8%	74.2%	66.6%
Brazil	Guabiruba	2017	71.6%	75.0%	67.5%
Brazil	Guaçuí	2000	79.6%	83.1%	76.2%
Brazil	Guaçuí	2017	80.3%	83.6%	77.0%
Brazil	Guadalupe	2000	61.6%	67.3%	55.8%
Brazil	Guadalupe	2017	62.5%	68.1%	56.7%
Brazil	Guaíba	2000	81.8%	84.3%	79.4%
Brazil	Guaíba	2017	82.4%	84.8%	80.0%
Brazil	Guaiçara	2000	93.1%	95.1%	90.8%
Brazil	Guaiçara	2017	93.2%	95.2%	91.0%
Brazil	Guaimbê	2000	93.0%	95.0%	90.5%
Brazil	Guaimbê	2017	93.2%	95.2%	90.8%
Brazil	Guaíra	2000	91.2%	93.8%	88.2%
Brazil	Guaíra	2000	82.5%	86.6%	78.8%
Brazil	Guaíra	2017	83.1%	87.0%	79.4%
Brazil	Guaíra	2017	91.5%	94.0%	88.6%
Brazil	Guairaçá	2000	85.3%	88.7%	81.7%
Brazil	Guairaçá	2017	85.8%	89.1%	82.3%
Brazil	Guaiúba	2000	68.9%	71.8%	65.9%
Brazil	Guaiúba	2017	69.8%	72.6%	66.7%
Brazil	Guajará	2000	46.1%	50.2%	42.2%
Brazil	Guajará	2017	46.1%	50.2%	42.2%
Brazil	Guajará-Mirim	2000	31.0%	37.5%	24.4%
Brazil	Guajará-Mirim	2017	31.8%	38.4%	25.2%
Brazil	Guajeru	2000	77.8%	83.0%	72.1%
Brazil	Guajeru	2017	78.5%	83.5%	72.9%
Brazil	Guamaré	2000	82.0%	85.5%	77.9%
Brazil	Guamaré	2017	82.7%	86.0%	78.7%
Brazil	Guamiranga	2000	82.0%	86.3%	77.8%
Brazil	Guamiranga	2017	82.6%	86.7%	78.5%

Country	Second Administrative Unit	Year	Mean	Upper	Lower
Brazil	Guanambi	2000	79.0%	84.4%	74.0%
Brazil	Guanambi	2017	79.7%	84.9%	74.7%
Brazil	Guanhães	2000	82.9%	86.7%	77.3%
Brazil	Guanhães	2017	82.5%	86.4%	76.8%
Brazil	Guapé	2000	82.3%	86.5%	77.2%
Brazil	Guapé	2017	82.9%	86.9%	77.9%
Brazil	Guapiaçu	2000	91.6%	93.8%	89.1%
Brazil	Guapiaçu	2017	91.9%	94.0%	89.4%
Brazil	Guapiara	2000	92.9%	94.6%	90.8%
Brazil	Guapiara	2017	93.2%	94.8%	91.2%
Brazil	Guapimirim	2000	75.8%	77.7%	73.3%
Brazil	Guapimirim	2017	76.5%	78.4%	74.0%
Brazil	Guapirama	2000	87.7%	90.5%	84.0%
Brazil	Guapirama	2017	88.2%	90.8%	84.5%
Brazil	Guapó	2000	76.4%	79.0%	73.6%
Brazil	Guapó	2017	77.1%	79.6%	74.3%
Brazil	Guaporé	2000	82.7%	85.9%	79.2%
Brazil	Guaporé	2017	83.3%	86.4%	79.9%
Brazil	Guaporema	2000	83.3%	86.3%	79.8%
Brazil	Guaporema	2017	83.9%	86.7%	80.4%
Brazil	Guará	2000	90.0%	92.6%	86.9%
Brazil	Guará	2017	90.3%	92.8%	87.4%
Brazil	Guarabira	2000	68.7%	71.5%	65.4%
Brazil	Guarabira	2017	69.5%	72.3%	66.3%
Brazil	Guaraçaí	2000	88.9%	91.4%	85.8%
Brazil	Guaraçaí	2017	89.2%	91.7%	86.2%
Brazil	Guaraci	2000	91.0%	93.3%	88.5%
Brazil	Guaraci	2000	86.1%	88.9%	83.2%
Brazil	Guaraci	2017	91.3%	93.6%	88.9%
Brazil	Guaraci	2017	86.6%	89.3%	83.7%
Brazil	Guaraciaba	2000	80.3%	83.9%	76.1%
Brazil	Guaraciaba	2000	81.2%	85.2%	76.7%
Brazil	Guaraciaba	2017	80.9%	84.4%	76.8%
Brazil	Guaraciaba	2017	80.9%	85.0%	76.3%
Brazil	Guaraciaba do Norte	2000	71.0%	75.0%	67.0%
Brazil	Guaraciaba do Norte	2017	71.8%	75.7%	67.8%
Brazil	Guaraciama	2000	82.2%	86.9%	76.9%
Brazil	Guaraciama	2017	82.8%	87.3%	77.6%
Brazil	Guaraíta	2000	79.8%	84.8%	74.3%
Brazil	Guaraíta	2017	80.5%	85.3%	75.0%
Brazil	Guaramiranga	2000	73.7%	77.1%	70.0%
Brazil	Guaramiranga	2017	74.4%	77.8%	70.8%
Brazil	Guaramirim	2000	75.8%	79.2%	72.0%
Brazil	Guaramirim	2017	76.8%	80.1%	73.0%
Brazil	Guaranesia	2000	87.8%	90.6%	84.4%
Brazil	Guaranesia	2017	88.2%	90.9%	84.9%
Brazil	Guarani	2000	79.2%	82.9%	75.0%
Brazil	Guarani	2017	79.9%	83.4%	75.7%
Brazil	Guarani das Missões	2000	81.8%	86.3%	76.8%
Brazil	Guarani das Missões	2017	82.4%	86.8%	77.5%
Brazil	Guarani de Goiás	2000	76.7%	81.1%	72.2%
Brazil	Guarani de Goiás	2017	77.4%	81.7%	73.0%
Brazil	Guarani do Oeste	2000	88.3%	91.5%	84.9%
Brazil	Guarani do Oeste	2017	88.7%	91.8%	85.4%

Country	Second Administrative Unit	Year	Mean	Upper	Lower
Brazil	Guaraniaçu	2000	83.9%	87.0%	80.2%
Brazil	Guaraniaçu	2017	84.4%	87.5%	80.8%
Brazil	Guarantã	2000	93.0%	95.2%	90.2%
Brazil	Guarantã	2017	93.3%	95.4%	90.6%
Brazil	Guarantã do Norte	2000	63.8%	70.9%	55.8%
Brazil	Guarantã do Norte	2017	64.7%	71.7%	56.7%
Brazil	Guarapari	2000	76.0%	79.0%	72.3%
Brazil	Guarapari	2017	76.7%	79.7%	73.1%
Brazil	Guarapuava	2000	83.5%	87.1%	79.2%
Brazil	Guarapuava	2017	84.1%	87.5%	79.9%
Brazil	Guaraqueçaba	2000	80.0%	84.3%	75.1%
Brazil	Guaraqueçaba	2017	80.6%	84.8%	75.9%
Brazil	Guarará	2000	80.7%	83.7%	77.3%
Brazil	Guarará	2017	81.4%	84.3%	78.1%
Brazil	Guararapes	2000	90.0%	91.9%	87.7%
Brazil	Guararapes	2017	90.3%	92.1%	88.1%
Brazil	Guararema	2000	90.6%	93.1%	87.2%
Brazil	Guararema	2017	90.9%	93.4%	87.6%
Brazil	Guaratinga	2000	80.2%	84.8%	74.1%
Brazil	Guaratinga	2017	80.8%	85.3%	74.9%
Brazil	Guaratinguetá	2000	93.6%	94.6%	92.3%
Brazil	Guaratinguetá	2017	93.9%	94.8%	92.6%
Brazil	Guaratuba	2000	75.8%	79.9%	71.1%
Brazil	Guaratuba	2017	76.5%	80.5%	71.9%
Brazil	Guarda-Mor	2000	79.8%	85.2%	73.4%
Brazil	Guarda-Mor	2017	80.4%	85.7%	74.2%
Brazil	Guareí	2000	93.2%	94.9%	90.9%
Brazil	Guareí	2017	93.4%	95.1%	91.2%
Brazil	Guariba	2000	92.7%	94.4%	90.4%
Brazil	Guariba	2017	93.0%	94.6%	90.8%
Brazil	Guaribas	2000	65.6%	72.2%	58.9%
Brazil	Guaribas	2017	66.5%	73.0%	59.8%
Brazil	Guarinos	2000	77.0%	81.8%	71.8%
Brazil	Guarinos	2017	77.7%	82.4%	72.6%
Brazil	Guarujá	2000	87.4%	89.6%	85.0%
Brazil	Guarujá	2017	87.8%	89.9%	85.4%
Brazil	Guarujá do Sul	2000	81.5%	85.7%	76.5%
Brazil	Guarujá do Sul	2017	82.0%	86.2%	77.2%
Brazil	Guarulhos	2000	94.4%	95.2%	93.7%
Brazil	Guarulhos	2017	94.6%	95.4%	93.9%
Brazil	Guatambú	2000	77.7%	81.0%	73.4%
Brazil	Guatambú	2017	78.3%	81.6%	74.2%
Brazil	Guatapará	2000	92.7%	94.3%	90.2%
Brazil	Guatapará	2017	92.9%	94.5%	90.5%
Brazil	Guaxupé	2000	88.2%	91.0%	84.9%
Brazil	Guaxupé	2017	88.6%	91.3%	85.4%
Brazil	Guia Branca	2000	78.9%	82.0%	75.1%
Brazil	Guia Branca	2017	79.5%	82.6%	75.8%
Brazil	Guia Lopes da Laguna	2000	79.8%	84.3%	74.9%
Brazil	Guia Lopes da Laguna	2017	80.6%	85.0%	75.8%
Brazil	Guidoval	2000	80.2%	83.7%	76.4%
Brazil	Guidoval	2017	80.8%	84.2%	77.1%
Brazil	Guimarães	2000	58.6%	64.4%	52.1%
Brazil	Guimarães	2017	59.5%	65.3%	53.0%
Brazil	Guimarania	2000	81.4%	86.1%	75.7%
Brazil	Guimarania	2017	82.0%	86.5%	76.4%

Country	Second Administrative Unit	Year	Mean	Upper	Lower
Brazil	Guiratinga	2000	72.1%	77.9%	66.1%
Brazil	Guiratinga	2017	72.9%	78.6%	66.9%
Brazil	Guiricema	2000	80.8%	84.3%	76.6%
Brazil	Guiricema	2017	81.4%	84.8%	77.3%
Brazil	Gurinhatã	2000	83.0%	87.3%	78.7%
Brazil	Gurinhatã	2017	83.5%	87.7%	79.3%
Brazil	Gurinhém	2000	67.6%	71.0%	64.1%
Brazil	Gurinhém	2017	68.4%	71.8%	65.0%
Brazil	Gurjão	2000	69.0%	73.3%	64.6%
Brazil	Gurjão	2017	69.8%	74.1%	65.5%
Brazil	Gurupá	2000	39.8%	45.3%	34.8%
Brazil	Gurupá	2017	40.7%	46.2%	35.6%
Brazil	Gurupi	2000	78.3%	82.1%	74.5%
Brazil	Gurupi	2017	79.1%	82.8%	75.5%
Brazil	Guzolandia	2000	89.4%	92.4%	86.2%
Brazil	Guzolandia	2017	89.7%	92.7%	86.6%
Brazil	Harmonia	2000	79.5%	82.2%	76.6%
Brazil	Harmonia	2017	80.2%	82.8%	77.3%
Brazil	Heitoraí	2000	76.4%	80.5%	71.6%
Brazil	Heitoraí	2017	77.1%	81.1%	72.4%
Brazil	Heliodora	2000	86.3%	89.3%	83.1%
Brazil	Heliodora	2017	86.7%	89.7%	83.6%
Brazil	Heliópolis	2000	77.2%	81.2%	73.0%
Brazil	Heliópolis	2017	77.8%	81.7%	73.8%
Brazil	Herculândia	2000	92.0%	94.4%	89.5%
Brazil	Herculândia	2017	92.3%	94.6%	89.8%
Brazil	Herval d'Oeste	2000	77.5%	81.6%	73.2%
Brazil	Herval d'Oeste	2017	76.9%	81.1%	72.5%
Brazil	Herveiras	2000	84.2%	87.2%	80.3%
Brazil	Herveiras	2017	84.6%	87.6%	80.9%
Brazil	Hidrolândia	2000	78.5%	80.7%	75.9%
Brazil	Hidrolândia	2000	67.3%	71.9%	62.8%
Brazil	Hidrolândia	2017	68.1%	72.7%	63.8%
Brazil	Hidrolândia	2017	79.6%	81.7%	77.0%
Brazil	Hidrolina	2000	78.1%	82.6%	72.6%
Brazil	Hidrolina	2017	78.7%	83.1%	73.4%
Brazil	Holambra	2000	92.2%	93.6%	90.6%
Brazil	Holambra	2017	92.5%	93.8%	90.9%
Brazil	Honório Serpa	2000	80.5%	84.5%	76.1%
Brazil	Honório Serpa	2017	81.1%	85.0%	76.9%
Brazil	Horizonte	2000	69.9%	72.8%	66.9%
Brazil	Horizonte	2017	72.5%	75.2%	69.6%
Brazil	Horizontina	2000	82.1%	86.4%	77.5%
Brazil	Horizontina	2017	82.6%	86.8%	78.2%
Brazil	Hortolândia	2000	93.5%	94.6%	92.2%
Brazil	Hortolândia	2017	93.7%	94.8%	92.5%
Brazil	Hugo Napoleão	2000	59.2%	64.0%	54.3%
Brazil	Hugo Napoleão	2017	60.1%	64.9%	55.2%
Brazil	Hulha Negra	2000	82.6%	87.6%	76.9%
Brazil	Hulha Negra	2017	83.2%	88.0%	77.6%
Brazil	Humaitá	2000	81.1%	85.3%	76.6%
Brazil	Humaitá	2000	53.5%	60.3%	46.6%
Brazil	Humaitá	2017	81.7%	85.8%	77.3%
Brazil	Humaitá	2017	54.5%	61.3%	47.5%
Brazil	Humberto Campos	2000	59.3%	65.2%	53.2%
Brazil	Humberto Campos	2017	60.3%	66.1%	54.1%

Country	Second Administrative Unit	Year	Mean	Upper	Lower
Brazil	Iacanga	2000	93.4%	95.3%	91.0%
Brazil	Iacanga	2017	93.6%	95.5%	91.4%
Brazil	Iaciara	2000	80.0%	84.6%	74.7%
Brazil	Iaciara	2017	80.6%	85.1%	75.4%
Brazil	Iacri	2000	91.4%	93.7%	88.6%
Brazil	Iacri	2017	91.7%	93.9%	89.0%
Brazil	Iaçu	2000	80.9%	84.7%	76.3%
Brazil	Iaçu	2000	77.7%	82.6%	71.5%
Brazil	Iaçu	2017	81.5%	85.2%	77.0%
Brazil	Iaçu	2017	78.4%	83.1%	72.3%
Brazil	Iaras	2000	92.7%	94.6%	90.2%
Brazil	Iaras	2017	93.0%	94.8%	90.6%
Brazil	Iati	2000	64.7%	68.3%	60.8%
Brazil	Iati	2017	65.5%	69.1%	61.7%
Brazil	Ibaiti	2000	87.4%	90.1%	84.0%
Brazil	Ibaiti	2017	87.8%	90.5%	84.5%
Brazil	Ibarama	2000	83.2%	86.5%	79.1%
Brazil	Ibarama	2017	83.7%	86.9%	79.8%
Brazil	Ibaretama	2000	70.7%	74.6%	66.6%
Brazil	Ibaretama	2017	71.5%	75.3%	67.4%
Brazil	Ibaté	2000	93.1%	94.7%	91.1%
Brazil	Ibaté	2017	93.4%	94.8%	91.4%
Brazil	Ibateguara	2000	62.6%	66.2%	59.4%
Brazil	Ibateguara	2017	63.5%	67.1%	60.3%
Brazil	Ibatiba	2000	80.8%	84.1%	77.4%
Brazil	Ibatiba	2017	81.4%	84.6%	78.1%
Brazil	Ibema	2000	84.4%	87.4%	80.7%
Brazil	Ibema	2017	84.9%	87.8%	81.3%
Brazil	Ibertioga	2000	83.9%	87.3%	79.8%
Brazil	Ibertioga	2017	84.4%	87.7%	80.4%
Brazil	Ibiá	2000	82.7%	87.2%	77.1%
Brazil	Ibiá	2017	83.2%	87.7%	77.8%
Brazil	Ibiaçá	2000	79.2%	83.5%	75.2%
Brazil	Ibiaçá	2017	79.9%	84.0%	76.0%
Brazil	Ibiaí	2000	81.1%	86.1%	74.4%
Brazil	Ibiaí	2017	81.7%	86.6%	75.1%
Brazil	Ibiam	2000	75.8%	79.8%	71.5%
Brazil	Ibiam	2017	76.5%	80.5%	72.4%
Brazil	Ibiapina	2000	70.5%	74.4%	66.6%
Brazil	Ibiapina	2017	71.3%	75.1%	67.5%
Brazil	Ibiara	2000	67.7%	71.7%	63.5%
Brazil	Ibiara	2017	68.6%	72.5%	64.4%
Brazil	Ibiassucê	2000	78.3%	83.7%	73.2%
Brazil	Ibiassucê	2017	78.1%	83.6%	72.9%
Brazil	Ibicaraí	2000	76.7%	80.5%	71.4%
Brazil	Ibicaraí	2017	77.4%	81.1%	72.2%
Brazil	Ibicaré	2000	75.6%	79.9%	71.2%
Brazil	Ibicaré	2017	76.3%	80.5%	72.0%
Brazil	Ibicoara	2000	79.5%	84.5%	73.5%
Brazil	Ibicoara	2017	80.2%	85.1%	74.3%
Brazil	Ibicuí	2000	79.3%	83.2%	74.8%
Brazil	Ibicuí	2017	80.1%	83.8%	75.7%
Brazil	Ibicuitinga	2000	71.3%	75.6%	66.9%
Brazil	Ibicuitinga	2017	72.1%	76.3%	67.8%
Brazil	Ibimirim	2000	65.0%	69.1%	60.7%
Brazil	Ibimirim	2017	65.8%	69.9%	61.6%
Brazil	Ibipeba	2000	75.7%	81.3%	68.4%
Brazil	Ibipeba	2017	76.5%	81.9%	69.3%
Brazil	Ibipitanga	2000	77.3%	82.4%	71.8%
Brazil	Ibipitanga	2017	78.0%	83.0%	72.6%
Brazil	Ibiporã	2000	86.4%	88.5%	83.7%
Brazil	Ibiporã	2017	86.8%	88.9%	84.2%

Country	Second Administrative Unit	Year	Mean	Upper	Lower
Brazil	Ibiquera	2000	78.4%	83.9%	72.8%
Brazil	Ibiquera	2017	79.0%	84.4%	73.5%
Brazil	Ibirá	2000	93.2%	94.9%	91.0%
Brazil	Ibirá	2017	93.4%	95.1%	91.3%
Brazil	Ibiracatu	2000	81.4%	86.5%	75.5%
Brazil	Ibiracatu	2017	82.0%	87.0%	76.2%
Brazil	Ibiraci	2000	87.7%	91.2%	83.9%
Brazil	Ibiraci	2017	88.1%	91.6%	84.5%
Brazil	Ibiraçu	2000	76.0%	78.7%	73.1%
Brazil	Ibiraçu	2017	76.7%	79.4%	73.9%
Brazil	Ibiraiaras	2000	80.0%	84.1%	76.0%
Brazil	Ibiraiaras	2017	80.6%	84.6%	76.7%
Brazil	Ibirajuba	2000	66.2%	69.6%	63.0%
Brazil	Ibirajuba	2017	67.1%	70.5%	63.9%
Brazil	Ibirama	2000	71.4%	75.1%	67.6%
Brazil	Ibirama	2017	72.1%	75.8%	68.4%
Brazil	Ibirapitanga	2000	76.3%	80.7%	71.6%
Brazil	Ibirapitanga	2017	77.0%	81.4%	72.4%
Brazil	Ibirapuã	2000	78.7%	83.3%	73.5%
Brazil	Ibirapuã	2017	79.4%	83.8%	74.2%
Brazil	Ibirapuitã	2000	82.0%	85.5%	78.0%
Brazil	Ibirapuitã	2017	82.5%	86.0%	78.6%
Brazil	Ibirarema	2000	90.4%	92.7%	87.4%
Brazil	Ibirarema	2017	90.7%	93.0%	87.8%
Brazil	Ibirataia	2000	76.9%	81.4%	72.1%
Brazil	Ibirataia	2017	77.6%	82.0%	72.9%
Brazil	Ibirité	2000	82.7%	85.1%	80.0%
Brazil	Ibirité	2017	83.2%	85.6%	80.5%
Brazil	Ibirubá	2000	83.4%	86.8%	79.0%
Brazil	Ibirubá	2017	84.0%	87.3%	79.6%
Brazil	Ibitiara	2000	79.1%	84.7%	73.1%
Brazil	Ibitiara	2017	79.8%	85.2%	73.9%
Brazil	Ibitinga	2000	93.8%	95.4%	91.7%
Brazil	Ibitinga	2017	94.0%	95.6%	92.0%
Brazil	Ibitirama	2000	80.6%	83.6%	77.5%
Brazil	Ibitirama	2017	81.2%	84.1%	78.1%
Brazil	Ibititá	2000	77.2%	82.7%	70.9%
Brazil	Ibititá	2017	77.9%	83.3%	71.7%
Brazil	Ibitiúra de Minas	2000	90.1%	92.3%	87.4%
Brazil	Ibitiúra de Minas	2017	90.5%	92.5%	87.8%
Brazil	Ibituruna	2000	79.8%	84.0%	75.2%
Brazil	Ibituruna	2017	80.4%	84.5%	75.9%
Brazil	Ibiúna	2000	94.6%	95.5%	93.4%
Brazil	Ibiúna	2017	94.8%	95.7%	93.7%
Brazil	Ibotirama	2000	74.8%	81.4%	65.9%
Brazil	Ibotirama	2017	75.6%	82.0%	66.8%
Brazil	Icapuí	2000	76.5%	80.5%	72.4%
Brazil	Icapuí	2017	77.7%	81.5%	73.6%
Brazil	Içara	2000	74.6%	78.8%	69.8%
Brazil	Içara	2017	75.3%	79.5%	70.6%
Brazil	Icaraí de Minas	2000	81.9%	87.3%	75.7%
Brazil	Icaraí de Minas	2017	82.5%	87.7%	76.5%
Brazil	Icaraíma	2000	82.8%	86.0%	78.5%
Brazil	Icaraíma	2017	83.3%	86.5%	79.1%
Brazil	Icatu	2000	59.0%	63.4%	53.7%
Brazil	Icatu	2017	59.9%	64.3%	54.6%
Brazil	Icém	2000	90.5%	92.9%	87.7%
Brazil	Icém	2017	90.9%	93.1%	88.1%

Country	Second Administrative Unit	Year	Mean	Upper	Lower
Brazil	Ichu	2000	78.9%	82.4%	74.8%
Brazil	Ichu	2017	79.5%	83.0%	75.5%
Brazil	Icó	2000	71.9%	75.9%	67.7%
Brazil	Icó	2017	72.7%	76.6%	68.6%
Brazil	Iconha	2000	77.9%	81.0%	74.4%
Brazil	Iconha	2017	78.5%	81.6%	75.0%
Brazil	Ielmo Marinho	2000	83.1%	85.3%	81.2%
Brazil	Ielmo Marinho	2017	83.7%	85.8%	81.7%
Brazil	Iepê	2000	88.1%	90.5%	85.5%
Brazil	Iepê	2017	88.5%	90.9%	86.0%
Brazil	Igaci	2000	60.8%	63.5%	58.2%
Brazil	Igaci	2017	61.8%	64.5%	59.3%
Brazil	Igaporã	2000	78.4%	83.6%	72.9%
Brazil	Igaporã	2017	79.0%	84.1%	73.6%
Brazil	Igaracu	2000	64.7%	67.1%	62.4%
Brazil	Igaracu	2017	66.2%	68.5%	63.9%
Brazil	Igaraçu do Tietê	2000	93.7%	95.3%	91.9%
Brazil	Igaraçu do Tietê	2017	93.9%	95.5%	92.1%
Brazil	Igarapava	2000	86.7%	90.1%	82.1%
Brazil	Igarapava	2017	87.3%	90.6%	82.8%
Brazil	Igarapé	2000	81.9%	84.9%	78.7%
Brazil	Igarapé	2017	82.5%	85.4%	79.4%
Brazil	Igarapé do Meio	2000	60.2%	65.9%	55.0%
Brazil	Igarapé do Meio	2017	61.2%	66.8%	56.0%
Brazil	Igarapé Grande	2000	60.6%	65.5%	55.9%
Brazil	Igarapé Grande	2017	61.5%	66.3%	56.8%
Brazil	Igarapé-Açu	2000	37.2%	42.1%	33.4%
Brazil	Igarapé-Açu	2017	38.2%	43.0%	34.3%
Brazil	Igarapé-Miri	2000	37.6%	42.6%	33.5%
Brazil	Igarapé-Miri	2017	38.6%	43.6%	34.4%
Brazil	Igaratá	2000	92.9%	94.1%	91.7%
Brazil	Igaratá	2017	93.4%	94.5%	92.2%
Brazil	Igaratinga	2000	80.7%	84.5%	76.7%
Brazil	Igaratinga	2017	81.3%	85.0%	77.4%
Brazil	Igrapiúna	2000	71.7%	76.9%	66.4%
Brazil	Igrapiúna	2017	72.5%	77.5%	67.2%
Brazil	Igreja Nova	2000	65.4%	68.2%	62.8%
Brazil	Igreja Nova	2017	66.3%	69.1%	63.8%
Brazil	Igrejinha	2000	81.2%	84.0%	78.2%
Brazil	Igrejinha	2017	81.8%	84.5%	78.8%
Brazil	Iguaba Grande	2000	77.7%	81.0%	73.5%
Brazil	Iguaba Grande	2017	78.6%	81.8%	74.5%
Brazil	Iguaí	2000	79.6%	83.8%	74.4%
Brazil	Iguaí	2017	80.2%	84.3%	75.1%
Brazil	Iguape	2000	88.8%	92.1%	85.0%
Brazil	Iguape	2017	89.2%	92.4%	85.5%
Brazil	Iguaraci	2000	66.4%	70.4%	62.2%
Brazil	Iguaraci	2017	67.3%	71.2%	63.1%
Brazil	Iguaraçu	2000	85.5%	88.2%	82.9%
Brazil	Iguaraçu	2017	86.0%	88.6%	83.4%
Brazil	Iguatama	2000	81.8%	86.3%	76.7%
Brazil	Iguatama	2017	82.4%	86.8%	77.4%

Country	Second Administrative Unit	Year	Mean	Upper	Lower
Brazil	Iguatemi	2000	82.6%	86.5%	78.1%
Brazil	Iguatemi	2017	83.2%	87.0%	78.9%
Brazil	Iguatu	2000	83.9%	87.7%	79.8%
Brazil	Iguatu	2000	69.7%	73.6%	65.9%
Brazil	Iguatu	2017	84.5%	88.2%	80.5%
Brazil	Iguatu	2017	70.6%	74.4%	66.8%
Brazil	Ijaci	2000	81.0%	84.8%	76.4%
Brazil	Ijaci	2017	81.6%	85.3%	77.1%
Brazil	Ijuí	2000	83.4%	86.9%	79.1%
Brazil	Ijuí	2017	83.9%	87.4%	79.7%
Brazil	Ilha das Flores	2000	66.7%	70.2%	63.2%
Brazil	Ilha das Flores	2017	67.6%	71.0%	64.1%
Brazil	Ilha Grande	2000	61.3%	66.2%	56.0%
Brazil	Ilha Grande	2017	62.2%	67.1%	56.9%
Brazil	Ilha Solteira	2000	85.4%	88.8%	80.9%
Brazil	Ilha Solteira	2017	86.4%	89.6%	82.2%
Brazil	Ilhabela	2000	90.4%	93.0%	87.5%
Brazil	Ilhabela	2017	90.8%	93.3%	88.0%
Brazil	Ilhéus	2000	74.6%	79.0%	69.2%
Brazil	Ilhéus	2017	75.3%	79.6%	70.1%
Brazil	Ilhota	2000	73.2%	76.4%	69.4%
Brazil	Ilhota	2017	74.0%	77.2%	70.3%
Brazil	Ilicínea	2000	83.0%	86.8%	78.1%
Brazil	Ilicínea	2017	83.5%	87.2%	78.7%
Brazil	Ilópolis	2000	83.4%	86.9%	79.7%
Brazil	Ilópolis	2017	84.0%	87.4%	80.3%
Brazil	Imaculada	2000	69.5%	73.7%	65.3%
Brazil	Imaculada	2017	70.3%	74.5%	66.2%
Brazil	Imaruí	2000	71.0%	76.2%	65.1%
Brazil	Imaruí	2017	71.8%	76.9%	66.0%
Brazil	Imbaú	2000	84.2%	87.9%	80.4%
Brazil	Imbaú	2017	84.7%	88.3%	81.0%
Brazil	Imbé	2000	78.7%	83.0%	73.6%
Brazil	Imbé	2017	79.4%	83.6%	74.3%
Brazil	Imbé de Minas	2000	81.3%	85.0%	77.0%
Brazil	Imbé de Minas	2017	82.0%	85.5%	77.7%
Brazil	Imbituba	2000	71.7%	76.8%	65.3%
Brazil	Imbituba	2017	72.5%	77.5%	66.2%
Brazil	Imbituva	2000	82.6%	86.2%	78.4%
Brazil	Imbituva	2017	83.2%	86.7%	79.0%
Brazil	Imbuia	2000	76.6%	80.3%	71.9%
Brazil	Imbuia	2017	77.3%	80.9%	72.7%
Brazil	Imigrante	2000	81.2%	84.2%	78.2%
Brazil	Imigrante	2017	81.7%	84.7%	78.8%
Brazil	Imperatriz	2000	66.1%	70.4%	61.7%
Brazil	Imperatriz	2017	67.0%	71.2%	62.6%
Brazil	Inácio Martins	2000	83.0%	86.5%	78.9%
Brazil	Inácio Martins	2017	83.5%	87.0%	79.6%
Brazil	Inaciolândia	2000	79.4%	83.8%	74.2%
Brazil	Inaciolândia	2017	80.0%	84.3%	74.9%
Brazil	Inajá	2000	85.5%	88.6%	82.1%
Brazil	Inajá	2000	64.4%	68.6%	60.0%
Brazil	Inajá	2017	86.0%	89.0%	82.6%
Brazil	Inajá	2017	65.2%	69.4%	60.8%
Brazil	Inconfidentes	2000	89.5%	91.6%	87.0%
Brazil	Inconfidentes	2017	89.9%	91.9%	87.5%
Brazil	Indaiabira	2000	81.4%	86.3%	75.5%
Brazil	Indaiabira	2017	82.0%	86.7%	76.2%

Country	Second Administrative Unit	Year	Mean	Upper	Lower
Brazil	Indaial	2000	71.8%	75.5%	68.7%
Brazil	Indaial	2017	72.6%	76.2%	69.5%
Brazil	Indaiatuba	2000	93.5%	94.5%	92.2%
Brazil	Indaiatuba	2017	93.8%	94.7%	92.5%
Brazil	Independência	2000	71.2%	75.4%	67.0%
Brazil	Independência	2000	82.2%	86.5%	77.2%
Brazil	Independência	2017	71.0%	75.2%	66.8%
Brazil	Independência	2017	82.8%	86.9%	77.9%
Brazil	Indiana	2000	89.5%	92.3%	86.5%
Brazil	Indiana	2017	89.9%	92.5%	87.0%
Brazil	Indianópolis	2000	82.2%	86.3%	77.5%
Brazil	Indianópolis	2000	83.2%	86.2%	79.6%
Brazil	Indianópolis	2017	82.8%	86.8%	78.2%
Brazil	Indianópolis	2017	83.8%	86.6%	80.2%
Brazil	Indiaporã	2000	87.7%	91.1%	84.3%
Brazil	Indiaporã	2017	88.1%	91.4%	84.8%
Brazil	Indiara	2000	76.1%	80.1%	71.5%
Brazil	Indiara	2017	76.8%	80.8%	72.3%
Brazil	Indiaroba	2000	75.7%	79.3%	71.8%
Brazil	Indiaroba	2017	76.4%	79.9%	72.6%
Brazil	Indiavaí	2000	67.6%	73.8%	60.9%
Brazil	Indiavaí	2017	68.4%	74.5%	61.7%
Brazil	Ingá	2000	67.2%	70.2%	64.2%
Brazil	Ingá	2017	68.0%	71.0%	65.1%
Brazil	Ingaí	2000	81.6%	85.0%	76.8%
Brazil	Ingaí	2017	82.2%	85.4%	77.5%
Brazil	Ingazeira	2000	67.4%	71.2%	63.7%
Brazil	Ingazeira	2017	68.2%	72.0%	64.6%
Brazil	Inhacor	2000	81.8%	86.1%	76.9%
Brazil	Inhacor	2017	82.4%	86.5%	77.7%
Brazil	Inhambupe	2000	78.4%	81.9%	74.6%
Brazil	Inhambupe	2017	79.1%	82.6%	75.3%
Brazil	Inhangapi	2000	36.7%	40.1%	33.1%
Brazil	Inhangapi	2017	37.6%	41.0%	33.9%
Brazil	Inhapi	2000	63.9%	67.0%	60.1%
Brazil	Inhapi	2017	64.8%	67.9%	61.1%
Brazil	Inhapim	2000	81.2%	85.0%	77.0%
Brazil	Inhapim	2017	82.5%	86.1%	78.5%
Brazil	Inhaúma	2000	80.3%	84.0%	75.7%
Brazil	Inhaúma	2017	80.9%	84.6%	76.4%
Brazil	Inhuma	2000	62.3%	67.3%	57.5%
Brazil	Inhuma	2017	63.2%	68.2%	58.5%
Brazil	Inhumas	2000	78.1%	80.8%	75.3%
Brazil	Inhumas	2017	78.8%	81.4%	76.0%
Brazil	Inimutaba	2000	81.0%	85.8%	74.7%
Brazil	Inimutaba	2017	81.6%	86.3%	75.5%
Brazil	Inocência	2000	81.0%	85.2%	75.3%
Brazil	Inocência	2017	81.5%	85.7%	76.0%
Brazil	Inúbia Paulista	2000	90.9%	93.3%	87.8%
Brazil	Inúbia Paulista	2017	91.2%	93.5%	88.2%
Brazil	Iomerê	2000	76.6%	80.9%	72.6%
Brazil	Iomerê	2017	77.3%	81.5%	73.3%
Brazil	Ipaba	2000	80.0%	84.2%	75.4%
Brazil	Ipaba	2017	80.6%	84.7%	76.1%
Brazil	Ipameri	2000	79.8%	84.0%	74.6%
Brazil	Ipameri	2017	80.5%	84.6%	75.3%
Brazil	Ipanema	2000	78.8%	82.7%	74.0%
Brazil	Ipanema	2017	79.4%	83.3%	74.7%
Brazil	Ipanguaçu	2000	82.0%	84.9%	78.8%
Brazil	Ipanguaçu	2017	82.5%	85.4%	79.5%

Country	Second Administrative Unit	Year	Mean	Upper	Lower
Brazil	Ipaporanga	2000	67.1%	72.4%	62.1%
Brazil	Ipaporanga	2017	68.0%	73.2%	63.0%
Brazil	Ipatinga	2000	80.3%	84.0%	76.0%
Brazil	Ipatinga	2017	81.4%	84.9%	77.3%
Brazil	Ipaucu	2000	91.4%	93.5%	88.4%
Brazil	Ipaucu	2017	91.7%	93.8%	88.8%
Brazil	Ipaumirim	2000	70.3%	74.2%	66.7%
Brazil	Ipaumirim	2017	71.1%	75.0%	67.6%
Brazil	Ipê	2000	80.4%	83.7%	76.6%
Brazil	Ipê	2017	81.0%	84.3%	77.3%
Brazil	Ipecaetá	2000	77.5%	81.2%	73.5%
Brazil	Ipecaetá	2017	78.2%	81.8%	74.3%
Brazil	Iperó	2000	93.7%	95.0%	92.2%
Brazil	Iperó	2017	93.9%	95.2%	92.5%
Brazil	Ipeúna	2000	92.9%	94.3%	91.2%
Brazil	Ipeúna	2017	93.1%	94.5%	91.5%
Brazil	Ipiaçu	2000	80.5%	85.4%	75.8%
Brazil	Ipiaçu	2017	81.1%	85.8%	76.5%
Brazil	Ipiaú	2000	76.3%	80.8%	71.4%
Brazil	Ipiaú	2017	77.0%	81.4%	72.2%
Brazil	Ipiguá	2000	91.6%	93.7%	89.2%
Brazil	Ipiguá	2017	91.9%	93.9%	89.6%
Brazil	Ipirá	2000	78.6%	82.8%	74.4%
Brazil	Ipirá	2000	76.2%	80.0%	71.0%
Brazil	Ipirá	2017	79.3%	83.4%	75.2%
Brazil	Ipirá	2017	76.9%	80.6%	71.8%
Brazil	Ipiranga	2000	83.0%	86.8%	79.2%
Brazil	Ipiranga	2017	83.5%	87.2%	79.9%
Brazil	Ipiranga do Piauí	2000	62.4%	67.2%	57.5%
Brazil	Ipiranga do Piauí	2017	63.3%	68.0%	58.4%
Brazil	Ipiranga do Sul	2000	80.0%	83.5%	75.9%
Brazil	Ipiranga do Sul	2017	80.6%	84.1%	76.6%
Brazil	Ipixuna	2000	53.9%	60.6%	47.7%
Brazil	Ipixuna	2017	54.8%	61.5%	48.6%
Brazil	Ipixuna do Pará	2000	39.0%	43.9%	34.3%
Brazil	Ipixuna do Pará	2017	40.0%	44.8%	35.2%
Brazil	Ipojuca	2000	60.9%	64.4%	57.3%
Brazil	Ipojuca	2017	61.8%	65.3%	58.2%
Brazil	Iporá	2000	83.4%	86.7%	79.3%
Brazil	Iporá	2000	75.9%	81.2%	70.6%
Brazil	Iporá	2017	83.9%	87.1%	79.9%
Brazil	Iporá	2017	76.6%	81.8%	71.4%
Brazil	Iporã	2000	82.8%	86.1%	78.9%
Brazil	Iporã	2017	83.4%	86.5%	79.5%
Brazil	Iporã do Oeste	2000	79.7%	83.6%	75.4%
Brazil	Iporã do Oeste	2017	80.3%	84.1%	76.1%
Brazil	Iporanga	2000	87.7%	90.7%	84.6%
Brazil	Iporanga	2017	88.1%	91.0%	85.1%
Brazil	Ipú	2000	66.5%	71.4%	62.3%
Brazil	Ipú	2017	67.3%	72.2%	63.3%
Brazil	Ipuã	2000	89.5%	92.3%	86.5%
Brazil	Ipuã	2017	89.9%	92.5%	86.9%
Brazil	Ipuaçu	2000	76.9%	81.3%	72.5%
Brazil	Ipuaçu	2017	77.6%	81.9%	73.3%

Country	Second Administrative Unit	Year	Mean	Upper	Lower
Brazil	Ipubi	2000	66.1%	70.8%	61.6%
Brazil	Ipubi	2017	66.9%	71.6%	62.5%
Brazil	Ipueira	2000	72.3%	76.4%	67.8%
Brazil	Ipueira	2017	73.0%	77.1%	68.7%
Brazil	Ipueiras	2000	74.9%	79.5%	70.0%
Brazil	Ipueiras	2000	67.3%	72.3%	62.8%
Brazil	Ipueiras	2017	68.1%	73.1%	63.7%
Brazil	Ipueiras	2017	75.7%	80.2%	70.8%
Brazil	Ipuiúna	2000	89.7%	91.7%	86.6%
Brazil	Ipuiúna	2017	90.8%	92.7%	88.0%
Brazil	Ipumirim	2000	76.3%	79.8%	71.2%
Brazil	Ipumirim	2017	77.0%	80.4%	72.0%
Brazil	Ipupiara	2000	75.8%	82.2%	68.1%
Brazil	Ipupiara	2017	76.5%	82.7%	68.9%
Brazil	Iracema	2000	76.1%	80.2%	71.6%
Brazil	Iracema	2000	86.1%	88.4%	83.0%
Brazil	Iracema	2017	87.3%	89.4%	84.4%
Brazil	Iracema	2017	76.7%	80.7%	72.4%
Brazil	Iracema do Oeste	2000	84.2%	88.0%	80.2%
Brazil	Iracema do Oeste	2017	84.8%	88.4%	80.9%
Brazil	Iracemápolis	2000	93.6%	94.9%	92.0%
Brazil	Iracemápolis	2017	93.8%	95.1%	92.3%
Brazil	Iraceminha	2000	77.9%	82.3%	73.4%
Brazil	Iraceminha	2017	78.6%	82.8%	74.2%
Brazil	Iraí	2000	76.7%	80.8%	71.8%
Brazil	Iraí	2017	77.3%	81.4%	72.5%
Brazil	Iraí de Minas	2000	81.6%	85.9%	76.2%
Brazil	Iraí de Minas	2017	82.2%	86.3%	76.9%
Brazil	Irajuba	2000	81.6%	85.6%	75.9%
Brazil	Irajuba	2017	82.2%	86.1%	76.6%
Brazil	Iramaia	2000	76.7%	81.5%	70.9%
Brazil	Iramaia	2017	77.3%	82.1%	71.7%
Brazil	Iranduba	2000	56.5%	59.0%	54.0%
Brazil	Iranduba	2017	57.4%	59.9%	55.0%
Brazil	Irani	2000	78.1%	81.8%	74.0%
Brazil	Irani	2017	78.7%	82.3%	74.7%
Brazil	Irapuã	2000	93.0%	94.7%	90.9%
Brazil	Irapuã	2017	93.3%	94.9%	91.2%
Brazil	Irapuru	2000	89.3%	91.6%	86.1%
Brazil	Irapuru	2017	89.7%	91.9%	86.6%
Brazil	Iraquara	2000	78.3%	83.4%	72.4%
Brazil	Iraquara	2017	79.0%	83.9%	73.1%
Brazil	Irará	2000	78.3%	81.7%	74.6%
Brazil	Irará	2017	78.9%	82.2%	75.4%
Brazil	Irati	2000	77.9%	82.0%	74.0%
Brazil	Irati	2000	83.1%	86.4%	79.3%
Brazil	Irati	2017	78.5%	82.5%	74.7%
Brazil	Irati	2017	83.6%	86.9%	80.0%
Brazil	Irauçuba	2000	71.7%	75.3%	67.1%
Brazil	Irauçuba	2017	73.1%	76.6%	68.6%
Brazil	Irecê	2000	78.1%	83.8%	72.4%
Brazil	Irecê	2017	78.8%	84.3%	73.2%
Brazil	Iretama	2000	84.6%	87.8%	80.9%
Brazil	Iretama	2017	85.1%	88.2%	81.5%
Brazil	Irineópolis	2000	78.1%	81.9%	73.5%
Brazil	Irineópolis	2017	78.8%	82.4%	74.3%
Brazil	Irituia	2000	38.0%	42.8%	34.2%
Brazil	Irituia	2017	39.2%	44.0%	35.4%
Brazil	Irupi	2000	81.0%	84.4%	77.7%
Brazil	Irupi	2017	82.4%	85.5%	79.3%

Country	Second Administrative Unit	Year	Mean	Upper	Lower
Brazil	Isaías Coelho	2000	59.4%	65.1%	53.3%
Brazil	Isaías Coelho	2017	60.3%	66.0%	54.3%
Brazil	Israelândia	2000	74.5%	79.3%	68.5%
Brazil	Israelândia	2017	75.3%	79.9%	69.3%
Brazil	Itá	2000	76.3%	80.0%	72.2%
Brazil	Itá	2017	77.0%	80.6%	72.9%
Brazil	Itaara	2000	84.1%	87.6%	79.9%
Brazil	Itaara	2017	84.7%	88.0%	80.5%
Brazil	Itabaiana	2000	77.8%	79.8%	75.6%
Brazil	Itabaiana	2000	67.2%	70.2%	64.4%
Brazil	Itabaiana	2017	68.5%	71.5%	65.8%
Brazil	Itabaiana	2017	78.4%	80.4%	76.4%
Brazil	Itabaianinha	2000	78.2%	81.1%	75.4%
Brazil	Itabaianinha	2017	78.9%	81.6%	76.1%
Brazil	Itabela	2000	77.2%	82.2%	71.2%
Brazil	Itabela	2017	77.9%	82.8%	72.0%
Brazil	Itaberá	2000	91.0%	93.4%	88.0%
Brazil	Itaberá	2017	91.3%	93.7%	88.4%
Brazil	Itaberaba	2000	77.1%	82.1%	71.1%
Brazil	Itaberaba	2017	77.8%	82.7%	71.9%
Brazil	Itaberaí	2000	76.3%	80.0%	72.5%
Brazil	Itaberaí	2017	77.0%	80.6%	73.2%
Brazil	Itabi	2000	69.6%	72.7%	66.0%
Brazil	Itabi	2017	70.5%	73.5%	66.8%
Brazil	Itabira	2000	83.2%	86.2%	79.8%
Brazil	Itabira	2017	83.8%	86.6%	80.4%
Brazil	Itabirinha de Mantena	2000	80.8%	83.9%	77.3%
Brazil	Itabirinha de Mantena	2017	81.4%	84.5%	77.9%
Brazil	Itabirito	2000	81.5%	84.3%	78.8%
Brazil	Itabirito	2017	82.1%	84.8%	79.3%
Brazil	Itaboraí	2000	78.1%	80.2%	75.6%
Brazil	Itaboraí	2017	78.9%	81.0%	76.5%
Brazil	Itabuna	2000	76.9%	81.1%	71.7%
Brazil	Itabuna	2017	77.6%	81.7%	72.5%
Brazil	Itacajá	2000	76.2%	80.9%	70.9%
Brazil	Itacajá	2017	76.9%	81.5%	71.7%
Brazil	Itacarambi	2000	80.7%	84.2%	77.5%
Brazil	Itacarambi	2017	81.3%	84.7%	78.1%
Brazil	Itacarambira	2000	81.1%	86.7%	74.8%
Brazil	Itacarambira	2017	81.7%	87.1%	75.5%
Brazil	Itacaré	2000	72.7%	78.1%	67.1%
Brazil	Itacaré	2017	73.3%	78.5%	67.9%
Brazil	Itacoatiara	2000	59.5%	64.0%	54.4%
Brazil	Itacoatiara	2017	60.4%	64.9%	55.4%
Brazil	Itacuruba	2000	65.3%	71.1%	58.8%
Brazil	Itacuruba	2017	66.1%	71.8%	59.7%
Brazil	Itacurubi	2000	81.3%	86.6%	75.5%
Brazil	Itacurubi	2017	81.9%	87.1%	76.2%
Brazil	Itaeté	2000	75.7%	81.1%	68.9%
Brazil	Itaeté	2017	76.4%	81.7%	69.8%
Brazil	Itagi	2000	79.6%	84.0%	75.6%
Brazil	Itagi	2017	80.2%	84.4%	76.3%
Brazil	Itagibá	2000	77.1%	81.9%	72.6%
Brazil	Itagibá	2017	77.8%	82.4%	73.3%
Brazil	Itagimirim	2000	79.9%	84.6%	74.1%
Brazil	Itagimirim	2017	80.5%	85.2%	74.8%
Brazil	Itaguaçu	2000	79.0%	82.2%	75.5%
Brazil	Itaguaçu	2017	79.6%	82.8%	76.2%
Brazil	Itaguaçu da Bahia	2000	73.2%	79.6%	64.7%

Country	Second Administrative Unit	Year	Mean	Upper	Lower
Brazil	Itaguaçu da Bahia	2017	73.9%	80.2%	65.5%
Brazil	Itaguaí	2000	77.0%	79.4%	74.6%
Brazil	Itaguaí	2017	78.0%	80.3%	75.8%
Brazil	Itaguajé	2000	85.1%	88.3%	81.3%
Brazil	Itaguajé	2017	85.6%	88.7%	81.9%
Brazil	Itaguara	2000	83.6%	87.8%	78.4%
Brazil	Itaguara	2017	84.0%	88.2%	79.0%
Brazil	Itaguari	2000	76.8%	80.9%	72.5%
Brazil	Itaguari	2017	77.5%	81.5%	73.3%
Brazil	Itaguaru	2000	77.2%	81.4%	72.7%
Brazil	Itaguaru	2017	77.9%	82.0%	73.4%
Brazil	Itaguatins	2000	67.6%	71.6%	63.2%
Brazil	Itaguatins	2017	68.4%	72.4%	64.1%
Brazil	Itaí	2000	93.0%	94.9%	90.5%
Brazil	Itaí	2017	93.2%	95.1%	90.8%
Brazil	Itaíba	2000	62.6%	66.4%	58.6%
Brazil	Itaíba	2017	63.5%	67.3%	59.5%
Brazil	Itaiçaba	2000	73.4%	77.3%	69.2%
Brazil	Itaiçaba	2017	74.2%	78.0%	70.1%
Brazil	Itainópolis	2000	59.7%	64.5%	54.5%
Brazil	Itainópolis	2017	60.6%	65.5%	55.5%
Brazil	Itaiópolis	2000	78.5%	82.1%	75.0%
Brazil	Itaiópolis	2017	79.2%	82.7%	75.8%
Brazil	Itaipava do Grajaú	2000	60.3%	65.9%	55.1%
Brazil	Itaipava do Grajaú	2017	61.2%	66.8%	56.0%
Brazil	Itaipé	2000	82.2%	85.8%	78.2%
Brazil	Itaipé	2017	82.8%	86.3%	78.8%
Brazil	Itaipulândia	2000	82.2%	86.5%	78.2%
Brazil	Itaipulândia	2017	82.8%	87.0%	78.9%
Brazil	Itaitinga	2000	69.0%	71.1%	66.6%
Brazil	Itaitinga	2017	69.9%	71.9%	67.5%
Brazil	Itaituba	2000	46.0%	54.8%	38.3%
Brazil	Itaituba	2017	46.9%	55.8%	39.2%
Brazil	Itajá	2000	79.9%	84.7%	74.3%
Brazil	Itajá	2000	81.6%	84.6%	78.3%
Brazil	Itajá	2017	82.2%	85.1%	78.9%
Brazil	Itajá	2017	80.6%	85.2%	75.1%
Brazil	Itajaí	2000	74.5%	78.1%	70.7%
Brazil	Itajaí	2017	75.2%	78.8%	71.4%
Brazil	Itajobi	2000	93.0%	94.9%	90.6%
Brazil	Itajobi	2017	93.3%	95.1%	90.9%
Brazil	Itaju	2000	93.4%	95.1%	91.1%
Brazil	Itaju	2017	93.6%	95.3%	91.4%
Brazil	Itaju do Colônia	2000	79.0%	83.0%	73.3%
Brazil	Itaju do Colônia	2017	79.7%	83.6%	74.2%
Brazil	Itajubá	2000	88.1%	90.4%	85.7%
Brazil	Itajubá	2017	88.5%	90.7%	86.2%
Brazil	Itajuípe	2000	75.8%	79.8%	70.7%
Brazil	Itajuípe	2017	76.4%	80.4%	71.3%
Brazil	Italva	2000	77.4%	81.1%	73.4%
Brazil	Italva	2017	78.1%	81.7%	74.2%
Brazil	Itamaraju	2000	77.6%	83.2%	71.6%
Brazil	Itamaraju	2017	78.9%	84.2%	73.1%
Brazil	Itamarandiba	2000	86.0%	88.8%	82.0%
Brazil	Itamarandiba	2017	86.4%	89.2%	82.5%
Brazil	Itamarati	2000	58.0%	67.1%	48.1%
Brazil	Itamarati	2017	59.0%	68.0%	49.1%

Country	Second Administrative Unit	Year	Mean	Upper	Lower
Brazil	Itamarati de Minas	2000	83.6%	87.7%	78.3%
Brazil	Itamarati de Minas	2017	84.2%	88.1%	79.0%
Brazil	Itamari	2000	76.9%	81.3%	71.9%
Brazil	Itamari	2017	77.6%	81.9%	72.7%
Brazil	Itambacuri	2000	79.9%	82.6%	76.7%
Brazil	Itambacuri	2017	80.4%	83.1%	77.3%
Brazil	Itambaraca	2000	88.9%	91.1%	86.2%
Brazil	Itambaraca	2017	89.3%	91.4%	86.7%
Brazil	Itambaracá	2000	63.4%	65.9%	60.3%
Brazil	Itambaracá	2017	64.3%	66.7%	61.2%
Brazil	Itambé	2000	78.8%	83.8%	73.0%
Brazil	Itambé	2000	83.3%	86.4%	79.7%
Brazil	Itambé	2017	79.5%	84.3%	73.8%
Brazil	Itambé	2017	83.8%	86.9%	80.3%
Brazil	Itambé do Mato Dentro	2000	81.9%	86.9%	76.7%
Brazil	Itambé do Mato Dentro	2017	82.4%	87.4%	77.4%
Brazil	Itamogi	2000	80.5%	84.3%	76.4%
Brazil	Itamogi	2017	81.1%	84.8%	77.2%
Brazil	Itamonte	2000	89.0%	91.8%	85.5%
Brazil	Itamonte	2017	89.1%	91.9%	85.6%
Brazil	Itanagra	2000	74.5%	79.2%	69.8%
Brazil	Itanagra	2017	75.2%	79.9%	70.6%
Brazil	Itanhaém	2000	90.7%	92.7%	88.4%
Brazil	Itanhaém	2017	91.0%	93.0%	88.8%
Brazil	Itanhandu	2000	86.8%	89.1%	83.9%
Brazil	Itanhandu	2017	87.2%	89.5%	84.4%
Brazil	Itanhém	2000	80.6%	84.9%	74.8%
Brazil	Itanhém	2017	81.2%	85.4%	75.6%
Brazil	Itanhomi	2000	83.6%	85.9%	81.0%
Brazil	Itanhomi	2017	84.3%	86.5%	81.7%
Brazil	Itaobim	2000	79.1%	83.3%	74.8%
Brazil	Itaobim	2017	79.8%	83.8%	75.5%
Brazil	Itaóca	2000	87.4%	90.5%	84.0%
Brazil	Itaóca	2017	87.8%	90.9%	84.6%
Brazil	Itaocara	2000	77.8%	81.4%	74.0%
Brazil	Itaocara	2017	78.5%	82.0%	74.7%
Brazil	Itapaci	2000	77.9%	82.4%	72.8%
Brazil	Itapaci	2017	78.5%	83.0%	73.6%
Brazil	Itapagipe	2000	80.6%	85.3%	73.3%
Brazil	Itapagipe	2017	81.2%	85.8%	74.1%
Brazil	Itapajé	2000	73.2%	76.6%	69.4%
Brazil	Itapajé	2017	73.9%	77.3%	70.2%
Brazil	Itaparica	2000	71.9%	75.3%	68.6%
Brazil	Itaparica	2017	72.7%	76.0%	69.5%
Brazil	Itapé	2000	76.1%	80.2%	70.8%
Brazil	Itapé	2017	76.8%	80.8%	71.6%
Brazil	Itapebi	2000	79.0%	84.2%	73.3%
Brazil	Itapebi	2017	79.7%	84.7%	74.1%
Brazil	Itapecerica	2000	83.5%	86.2%	80.6%
Brazil	Itapecerica	2017	84.4%	86.9%	81.8%
Brazil	Itapecerica da Serra	2000	94.5%	95.4%	93.5%
Brazil	Itapecerica da Serra	2017	94.8%	95.6%	93.8%
Brazil	Itapecuru Mirim	2000	62.4%	66.9%	58.2%
Brazil	Itapecuru Mirim	2017	63.3%	67.8%	59.1%

Country	Second Administrative Unit	Year	Mean	Upper	Lower
Brazil	Itapejara d'Oeste	2000	82.4%	86.1%	78.1%
Brazil	Itapejara d'Oeste	2017	83.0%	86.6%	78.8%
Brazil	Itapema	2000	72.9%	77.2%	67.8%
Brazil	Itapema	2017	75.0%	79.1%	70.1%
Brazil	Itapemirim	2000	75.1%	78.5%	71.2%
Brazil	Itapemirim	2017	75.8%	79.1%	72.0%
Brazil	Itaperuçu	2000	85.9%	88.4%	83.3%
Brazil	Itaperuçu	2017	86.4%	88.8%	83.9%
Brazil	Itaperuna	2000	76.8%	80.8%	73.0%
Brazil	Itaperuna	2017	77.5%	81.4%	73.7%
Brazil	Itapetim	2000	71.0%	75.7%	66.9%
Brazil	Itapetim	2017	71.9%	76.4%	67.8%
Brazil	Itapetinga	2000	80.5%	84.7%	74.9%
Brazil	Itapetinga	2017	81.1%	85.2%	75.7%
Brazil	Itapetininga	2000	93.9%	95.5%	91.8%
Brazil	Itapetininga	2017	94.1%	95.6%	92.1%
Brazil	Itapeva	2000	92.2%	94.3%	89.6%
Brazil	Itapeva	2000	81.3%	85.3%	76.3%
Brazil	Itapeva	2017	92.5%	94.5%	90.0%
Brazil	Itapeva	2017	81.8%	85.8%	77.0%
Brazil	Itapevi	2000	94.3%	95.1%	93.3%
Brazil	Itapevi	2017	94.5%	95.3%	93.5%
Brazil	Itapicuru	2000	78.7%	81.8%	75.1%
Brazil	Itapicuru	2017	79.3%	82.4%	75.8%
Brazil	Itapipoca	2000	69.8%	73.7%	66.1%
Brazil	Itapipoca	2017	70.6%	74.4%	66.9%
Brazil	Itapira	2000	94.3%	95.5%	93.0%
Brazil	Itapira	2017	94.5%	95.6%	93.2%
Brazil	Itapiranga	2000	70.3%	74.2%	66.0%
Brazil	Itapiranga	2017	70.8%	74.7%	66.6%
Brazil	Itapirapuã	2000	74.1%	79.1%	68.3%
Brazil	Itapirapuã	2017	74.8%	79.7%	69.1%
Brazil	Itapirapuã Paulista	2000	87.6%	90.7%	84.0%
Brazil	Itapirapuã Paulista	2017	88.0%	91.0%	84.5%
Brazil	Itapiratins	2000	73.6%	78.4%	69.0%
Brazil	Itapiratins	2017	74.4%	79.2%	69.8%
Brazil	Itapissuma	2000	63.6%	66.3%	60.9%
Brazil	Itapissuma	2017	64.5%	67.2%	61.8%
Brazil	Itapitanga	2000	78.9%	83.3%	74.5%
Brazil	Itapitanga	2017	79.6%	83.9%	75.3%
Brazil	Itapiúna	2000	69.9%	74.1%	66.2%
Brazil	Itapiúna	2017	70.7%	74.9%	67.1%
Brazil	Itapoá	2000	73.9%	78.0%	69.1%
Brazil	Itapoá	2017	75.1%	79.0%	70.4%
Brazil	Itápolis	2000	93.8%	95.3%	91.7%
Brazil	Itápolis	2017	94.0%	95.5%	92.0%
Brazil	Itaporã	2000	80.9%	83.7%	78.0%
Brazil	Itaporã	2017	81.4%	84.2%	78.6%
Brazil	Itaporã do Tocantins	2000	73.0%	77.4%	68.3%
Brazil	Itaporã do Tocantins	2017	73.7%	78.0%	69.1%
Brazil	Itaporanga	2000	90.4%	92.6%	87.4%
Brazil	Itaporanga	2000	69.4%	73.0%	65.0%
Brazil	Itaporanga	2017	90.7%	92.9%	87.8%
Brazil	Itaporanga	2017	70.3%	73.8%	65.9%
Brazil	Itaporanga dajuda	2000	76.5%	78.3%	74.5%

Country	Second Administrative Unit	Year	Mean	Upper	Lower
Brazil	Itaporanga dajuda	2017	77.2%	78.9%	75.3%
Brazil	Itapororoca	2000	70.9%	73.6%	67.8%
Brazil	Itapororoca	2017	71.7%	74.4%	68.6%
Brazil	Itapuã do Oeste	2000	27.0%	31.4%	23.0%
Brazil	Itapuã do Oeste	2017	27.8%	32.3%	23.8%
Brazil	Itapuca	2000	82.3%	85.7%	78.6%
Brazil	Itapuca	2017	82.9%	86.2%	79.2%
Brazil	Itapuí	2000	93.2%	95.0%	91.2%
Brazil	Itapuí	2017	93.5%	95.2%	91.5%
Brazil	Itapura	2000	84.7%	87.7%	80.6%
Brazil	Itapura	2017	85.2%	88.1%	81.2%
Brazil	Itapuranga	2000	76.0%	80.4%	71.6%
Brazil	Itapuranga	2017	76.7%	81.0%	72.4%
Brazil	Itaquaquecetuba	2000	94.5%	95.3%	93.6%
Brazil	Itaquaquecetuba	2017	94.7%	95.4%	93.8%
Brazil	Itaquara	2000	82.3%	86.0%	77.4%
Brazil	Itaquara	2017	82.8%	86.4%	78.0%
Brazil	Itaqui	2000	85.5%	90.3%	79.2%
Brazil	Itaqui	2017	86.0%	90.7%	79.9%
Brazil	Itaquiraí	2000	81.9%	85.5%	77.8%
Brazil	Itaquiraí	2017	82.4%	86.0%	78.4%
Brazil	Itaquitinga	2000	67.1%	70.1%	64.5%
Brazil	Itaquitinga	2017	68.0%	71.0%	65.4%
Brazil	Itarana	2000	79.9%	82.8%	76.8%
Brazil	Itarana	2017	80.5%	83.4%	77.6%
Brazil	Itarantim	2000	81.0%	85.4%	75.7%
Brazil	Itarantim	2017	81.6%	85.9%	76.4%
Brazil	Itararé	2000	89.9%	92.8%	86.7%
Brazil	Itararé	2017	90.2%	93.0%	87.2%
Brazil	Itariri	2000	90.7%	93.0%	87.8%
Brazil	Itariri	2017	91.1%	93.4%	88.4%
Brazil	Itaruma	2000	79.4%	84.1%	73.7%
Brazil	Itaruma	2017	80.0%	84.6%	74.4%
Brazil	Itarumã	2000	67.0%	72.6%	61.4%
Brazil	Itarumã	2017	67.9%	73.4%	62.4%
Brazil	Itatiaia	2000	83.0%	86.1%	79.7%
Brazil	Itatiaia	2017	83.5%	86.5%	80.3%
Brazil	Itatiaiuçu	2000	87.3%	89.1%	85.4%
Brazil	Itatiaiuçu	2017	87.8%	89.5%	85.9%
Brazil	Itatiba	2000	93.3%	94.4%	92.0%
Brazil	Itatiba	2017	93.5%	94.6%	92.2%
Brazil	Itatiba do Sul	2000	76.8%	80.5%	72.8%
Brazil	Itatiba do Sul	2017	77.4%	81.0%	73.4%
Brazil	Itatim	2000	80.3%	84.3%	75.7%
Brazil	Itatim	2017	80.8%	84.8%	76.4%
Brazil	Itatinga	2000	93.2%	95.1%	91.1%
Brazil	Itatinga	2017	93.5%	95.3%	91.4%
Brazil	Itatira	2000	71.8%	76.4%	66.5%
Brazil	Itatira	2017	72.6%	77.1%	67.4%
Brazil	Itatuba	2000	65.2%	68.5%	62.3%
Brazil	Itatuba	2017	66.1%	69.3%	63.2%
Brazil	Itaú	2000	77.3%	80.8%	73.5%
Brazil	Itaú	2017	77.9%	81.4%	74.1%
Brazil	Itaú de Minas	2000	81.2%	84.6%	77.4%
Brazil	Itaú de Minas	2017	81.8%	85.1%	78.1%
Brazil	Itaúba	2000	68.5%	74.1%	62.2%
Brazil	Itaúba	2017	69.4%	74.7%	62.5%
Brazil	Itaubal	2000	46.7%	50.6%	41.7%
Brazil	Itaubal	2017	47.6%	51.6%	42.6%

Country	Second Administrative Unit	Year	Mean	Upper	Lower
Brazil	Itauçu	2000	77.3%	80.5%	73.9%
Brazil	Itauçu	2017	78.0%	81.1%	74.6%
Brazil	Itaueira	2000	61.7%	67.3%	55.1%
Brazil	Itaueira	2017	62.6%	68.2%	56.1%
Brazil	Itaúna	2000	81.3%	84.8%	77.1%
Brazil	Itaúna	2017	81.9%	85.3%	77.8%
Brazil	Itaúna do Sul	2000	84.3%	88.0%	80.3%
Brazil	Itaúna do Sul	2017	84.8%	88.4%	80.9%
Brazil	Itaverava	2000	82.0%	85.6%	78.2%
Brazil	Itaverava	2017	82.6%	86.1%	78.9%
Brazil	Itinga	2000	81.1%	85.8%	74.0%
Brazil	Itinga	2017	81.7%	86.3%	74.7%
Brazil	Itinga do Maranhão	2000	54.6%	60.3%	48.0%
Brazil	Itinga do Maranhão	2017	55.4%	61.1%	48.8%
Brazil	Itiquira	2000	75.3%	80.1%	69.7%
Brazil	Itiquira	2017	76.1%	80.9%	70.5%
Brazil	Itirapina	2000	93.9%	95.3%	92.3%
Brazil	Itirapina	2017	94.1%	95.4%	92.6%
Brazil	Itirapuã	2000	88.1%	91.3%	83.7%
Brazil	Itirapuã	2017	88.5%	91.6%	84.2%
Brazil	Itiruçu	2000	81.6%	85.7%	76.0%
Brazil	Itiruçu	2017	82.1%	86.2%	76.7%
Brazil	Itiúba	2000	75.5%	81.1%	68.3%
Brazil	Itiúba	2017	76.2%	81.7%	69.2%
Brazil	Itobi	2000	91.9%	93.7%	89.6%
Brazil	Itobi	2017	92.2%	94.0%	89.9%
Brazil	Itororó	2000	79.4%	83.1%	73.7%
Brazil	Itororó	2017	80.0%	83.6%	74.4%
Brazil	Itu	2000	93.5%	94.6%	92.3%
Brazil	Itu	2017	93.7%	94.8%	92.6%
Brazil	Ituaçu	2000	77.6%	82.2%	72.4%
Brazil	Ituaçu	2017	78.3%	82.8%	73.2%
Brazil	Ituberá	2000	72.0%	77.1%	66.4%
Brazil	Ituberá	2017	72.7%	77.8%	67.3%
Brazil	Itueta	2000	77.0%	81.0%	73.0%
Brazil	Itueta	2017	77.7%	81.6%	73.8%
Brazil	Ituiutaba	2000	83.5%	87.7%	78.7%
Brazil	Ituiutaba	2017	84.0%	88.1%	79.4%
Brazil	Itumbiara	2000	81.4%	85.1%	77.2%
Brazil	Itumbiara	2017	81.9%	85.6%	77.8%
Brazil	Itumirim	2000	80.7%	84.6%	76.0%
Brazil	Itumirim	2017	81.3%	85.1%	76.7%
Brazil	Itupeva	2000	93.3%	94.4%	92.2%
Brazil	Itupeva	2017	93.6%	94.6%	92.5%
Brazil	Itupiranga	2000	38.7%	44.5%	32.7%
Brazil	Itupiranga	2017	39.6%	45.6%	33.5%
Brazil	Ituporanga	2000	74.9%	79.1%	70.6%
Brazil	Ituporanga	2017	75.6%	79.7%	71.4%
Brazil	Iturama	2000	87.2%	90.5%	83.3%
Brazil	Iturama	2017	87.6%	90.8%	83.9%
Brazil	Itutinga	2000	80.7%	84.7%	75.8%
Brazil	Itutinga	2017	81.3%	85.2%	76.5%
Brazil	Ituverava	2000	88.8%	91.6%	85.3%
Brazil	Ituverava	2017	89.2%	91.9%	85.8%
Brazil	Iuiú	2000	78.9%	85.0%	72.1%
Brazil	Iuiú	2017	79.6%	85.5%	72.8%
Brazil	Iúna	2000	80.4%	83.7%	77.0%
Brazil	Iúna	2017	81.0%	84.2%	77.7%
Brazil	Ivaí	2000	82.6%	86.9%	78.4%
Brazil	Ivaí	2017	83.1%	87.3%	79.0%

Country	Second Administrative Unit	Year	Mean	Upper	Lower
Brazil	Ivaiporã	2000	83.9%	87.4%	80.3%
Brazil	Ivaiporã	2017	84.4%	87.8%	80.9%
Brazil	Ivaté	2000	82.7%	85.1%	80.2%
Brazil	Ivaté	2017	83.3%	85.5%	80.8%
Brazil	Ivatuva	2000	83.2%	86.3%	79.4%
Brazil	Ivatuva	2017	83.7%	86.8%	80.0%
Brazil	Ivinhema	2000	81.5%	85.0%	77.6%
Brazil	Ivinhema	2017	82.1%	85.5%	78.3%
Brazil	Ivolândia	2000	76.3%	81.2%	71.5%
Brazil	Ivolândia	2017	77.0%	81.8%	72.3%
Brazil	Ivorá	2000	82.7%	85.9%	77.9%
Brazil	Ivorá	2017	83.3%	86.5%	78.7%
Brazil	Ivoti	2000	80.5%	83.0%	77.8%
Brazil	Ivoti	2017	81.2%	83.6%	78.6%
Brazil	Jaboatão dos Guararapes	2000	65.2%	67.1%	63.0%
Brazil	Jaboatão dos Guararapes	2017	66.1%	68.0%	63.9%
Brazil	Jaborá	2000	76.5%	80.2%	71.8%
Brazil	Jaborá	2017	77.3%	80.9%	72.6%
Brazil	Jaborandi	2000	78.6%	84.8%	70.6%
Brazil	Jaborandi	2000	91.5%	93.9%	88.8%
Brazil	Jaborandi	2017	79.3%	85.2%	71.5%
Brazil	Jaborandi	2017	91.8%	94.1%	89.2%
Brazil	Jaboticaba	2000	79.9%	83.8%	76.0%
Brazil	Jaboticaba	2017	80.5%	84.4%	76.8%
Brazil	Jaboticatubas	2000	80.9%	84.3%	77.2%
Brazil	Jaboticatubas	2017	81.5%	84.8%	77.8%
Brazil	Jabuti	2000	87.2%	90.1%	83.6%
Brazil	Jabuti	2017	87.6%	90.4%	84.2%
Brazil	Jabuticabal	2000	93.3%	94.9%	91.3%
Brazil	Jabuticabal	2017	93.6%	95.0%	91.6%
Brazil	Jaçanã	2000	78.5%	81.5%	74.9%
Brazil	Jaçanã	2017	79.2%	82.1%	75.6%
Brazil	Jacaraci	2000	78.4%	83.5%	72.4%
Brazil	Jacaraci	2017	79.0%	84.1%	73.2%
Brazil	Jacaraú	2000	75.6%	78.4%	73.1%
Brazil	Jacaraú	2017	76.3%	79.0%	73.8%
Brazil	Jacaré dos Homens	2000	63.3%	66.5%	60.0%
Brazil	Jacaré dos Homens	2017	64.2%	67.4%	60.9%
Brazil	Jacareacanga	2000	54.9%	63.7%	44.9%
Brazil	Jacareacanga	2017	55.7%	64.5%	45.7%
Brazil	Jacareí	2000	93.7%	94.7%	92.5%
Brazil	Jacareí	2017	94.0%	94.9%	92.8%
Brazil	Jacarezinho	2000	91.1%	93.2%	88.3%
Brazil	Jacarezinho	2017	91.4%	93.4%	88.7%
Brazil	Jaci	2000	92.4%	94.1%	90.2%
Brazil	Jaci	2017	92.6%	94.4%	90.5%
Brazil	Jaciara	2000	67.1%	72.3%	61.6%
Brazil	Jaciara	2017	68.0%	73.1%	62.6%
Brazil	Jacinto	2000	80.1%	85.5%	74.3%
Brazil	Jacinto	2017	80.8%	86.0%	75.1%
Brazil	Jacinto Machado	2000	78.2%	82.4%	73.6%
Brazil	Jacinto Machado	2017	78.8%	82.9%	74.4%
Brazil	Jacobina	2000	78.5%	82.7%	74.0%
Brazil	Jacobina	2017	79.2%	83.3%	74.8%
Brazil	Jacobina do Piauí	2000	61.5%	66.1%	55.4%

Country	Second Administrative Unit	Year	Mean	Upper	Lower
Brazil	Jacobina do Piauí	2017	62.4%	67.0%	56.4%
Brazil	Jacuí	2000	86.8%	90.1%	82.8%
Brazil	Jacuí	2017	87.3%	90.4%	83.4%
Brazil	Jacuípe	2000	62.1%	65.5%	58.7%
Brazil	Jacuípe	2017	63.0%	66.4%	59.6%
Brazil	Jacundá	2000	39.7%	45.9%	33.3%
Brazil	Jacundá	2017	40.6%	46.9%	34.2%
Brazil	Jacupiranga	2000	88.0%	91.3%	84.1%
Brazil	Jacupiranga	2017	88.4%	91.6%	84.6%
Brazil	Jacutinga	2000	90.1%	92.2%	87.6%
Brazil	Jacutinga	2000	79.3%	82.6%	75.7%
Brazil	Jacutinga	2017	79.9%	83.2%	76.4%
Brazil	Jacutinga	2017	90.5%	92.4%	88.0%
Brazil	Jaguapitã	2000	86.1%	88.5%	83.2%
Brazil	Jaguapitã	2017	86.6%	88.9%	83.7%
Brazil	Jaguaquara	2000	82.1%	85.9%	77.0%
Brazil	Jaguaquara	2017	82.7%	86.4%	77.7%
Brazil	Jaguaraçu	2000	82.2%	85.9%	78.5%
Brazil	Jaguaraçu	2017	81.9%	85.6%	78.1%
Brazil	Jaguarão	2000	81.0%	87.3%	73.2%
Brazil	Jaguarão	2017	80.1%	86.6%	72.0%
Brazil	Jaguarari	2000	74.5%	79.8%	68.3%
Brazil	Jaguarari	2017	75.3%	80.5%	69.1%
Brazil	Jaguaré	2000	75.8%	79.8%	71.3%
Brazil	Jaguaré	2017	76.5%	80.4%	72.1%
Brazil	Jaguaretama	2000	70.3%	75.3%	65.7%
Brazil	Jaguaretama	2017	71.1%	76.0%	66.5%
Brazil	Jaguari	2000	82.0%	86.2%	76.8%
Brazil	Jaguari	2017	82.6%	86.6%	77.5%
Brazil	Jaguariaíva	2000	87.5%	90.1%	84.3%
Brazil	Jaguariaíva	2017	87.9%	90.5%	84.8%
Brazil	Jaguaribara	2000	71.8%	76.4%	66.9%
Brazil	Jaguaribara	2017	72.6%	77.0%	67.8%
Brazil	Jaguaribe	2000	72.4%	76.6%	67.6%
Brazil	Jaguaribe	2017	73.1%	77.3%	68.4%
Brazil	Jaguaripe	2000	74.6%	78.3%	70.8%
Brazil	Jaguaripe	2017	75.3%	79.0%	71.6%
Brazil	Jaguariúna	2000	92.6%	94.0%	91.2%
Brazil	Jaguariúna	2017	92.9%	94.2%	91.5%
Brazil	Jaguaruana	2000	74.6%	78.4%	70.6%
Brazil	Jaguaruana	2017	75.4%	79.1%	71.4%
Brazil	Jaguaruna	2000	74.4%	78.8%	69.0%
Brazil	Jaguaruna	2017	75.1%	79.5%	69.8%
Brazil	Jaíba	2000	80.4%	85.8%	74.5%
Brazil	Jaíba	2017	81.0%	86.3%	75.2%
Brazil	Jaicós	2000	61.3%	65.6%	56.7%
Brazil	Jaicós	2017	62.2%	66.5%	57.7%
Brazil	Jales	2000	89.5%	92.5%	86.5%
Brazil	Jales	2017	89.9%	92.7%	86.9%
Brazil	Jambeiro	2000	93.3%	94.6%	91.9%
Brazil	Jambeiro	2017	93.6%	94.8%	92.2%
Brazil	Jampruca	2000	80.1%	85.4%	75.3%
Brazil	Jampruca	2017	80.7%	85.9%	76.0%
Brazil	Janaúba	2000	83.2%	87.8%	77.6%
Brazil	Janaúba	2017	83.8%	88.2%	78.2%
Brazil	Jandaia	2000	76.5%	80.7%	71.9%
Brazil	Jandaia	2017	77.2%	81.3%	72.7%
Brazil	Jandaia do Sul	2000	84.6%	87.1%	81.6%
Brazil	Jandaia do Sul	2017	85.1%	87.6%	82.2%

Country	Second Administrative Unit	Year	Mean	Upper	Lower
Brazil	Jandaíra	2000	76.3%	79.9%	72.1%
Brazil	Jandaíra	2000	85.2%	88.0%	82.3%
Brazil	Jandaíra	2017	76.9%	80.5%	72.8%
Brazil	Jandaíra	2017	85.7%	88.4%	82.9%
Brazil	Jandira	2000	94.3%	95.1%	93.4%
Brazil	Jandira	2017	94.5%	95.3%	93.6%
Brazil	Janduís	2000	79.1%	82.5%	75.6%
Brazil	Janduís	2017	79.8%	83.1%	76.3%
Brazil	Jangada	2000	67.4%	72.3%	62.4%
Brazil	Jangada	2017	68.2%	73.1%	63.3%
Brazil	Janiópolis	2000	84.5%	87.7%	80.6%
Brazil	Janiópolis	2017	85.0%	88.2%	81.2%
Brazil	Januária	2000	80.6%	85.9%	73.9%
Brazil	Januária	2017	81.2%	86.3%	74.7%
Brazil	Januário Cicco	2000	81.6%	83.8%	79.6%
Brazil	Januário Cicco	2017	82.2%	84.3%	80.2%
Brazil	Japaraíba	2000	81.1%	85.5%	76.0%
Brazil	Japaraíba	2017	81.7%	85.9%	76.7%
Brazil	Japaratinga	2000	62.5%	66.6%	57.1%
Brazil	Japaratinga	2017	63.5%	67.5%	58.0%
Brazil	Japaratuba	2000	72.9%	75.3%	70.1%
Brazil	Japaratuba	2017	73.7%	76.0%	70.9%
Brazil	Japeri	2000	76.2%	78.2%	73.7%
Brazil	Japeri	2017	76.9%	78.9%	74.5%
Brazil	Japi	2000	78.0%	80.9%	75.0%
Brazil	Japi	2017	78.7%	81.5%	75.7%
Brazil	Japira	2000	86.8%	89.6%	83.2%
Brazil	Japira	2017	87.2%	89.9%	83.8%
Brazil	Japoatã	2000	71.2%	73.9%	68.0%
Brazil	Japoatã	2017	72.0%	74.6%	68.8%
Brazil	Japonvar	2000	81.9%	86.8%	76.0%
Brazil	Japonvar	2017	82.5%	87.2%	76.7%
Brazil	Japorã	2000	81.5%	85.7%	76.7%
Brazil	Japorã	2017	82.1%	86.2%	77.4%
Brazil	Japurá	2000	84.8%	87.6%	81.4%
Brazil	Japurá	2000	54.7%	62.6%	46.3%
Brazil	Japurá	2017	85.3%	88.0%	82.0%
Brazil	Japurá	2017	55.7%	63.5%	47.3%
Brazil	Jaqueira	2000	64.6%	68.1%	61.4%
Brazil	Jaqueira	2017	65.4%	68.9%	62.2%
Brazil	Jaquirana	2000	80.0%	84.1%	75.2%
Brazil	Jaquirana	2017	80.6%	84.6%	75.9%
Brazil	Jaraguá	2000	79.0%	82.7%	74.5%
Brazil	Jaraguá	2017	79.6%	83.3%	75.2%
Brazil	Jaraguá do Sul	2000	74.1%	77.6%	70.7%
Brazil	Jaraguá do Sul	2017	74.8%	78.3%	71.5%
Brazil	Jaraguari	2000	79.0%	82.1%	75.4%
Brazil	Jaraguari	2017	79.6%	82.6%	76.1%
Brazil	Jaramataia	2000	64.1%	67.1%	60.9%
Brazil	Jaramataia	2017	64.9%	67.9%	61.8%
Brazil	Jardim	2000	69.2%	73.0%	64.9%
Brazil	Jardim	2000	80.1%	84.5%	75.1%
Brazil	Jardim	2017	70.1%	73.8%	65.8%
Brazil	Jardim	2017	82.1%	86.2%	77.5%
Brazil	Jardim Alegre	2000	84.4%	87.7%	80.8%
Brazil	Jardim Alegre	2017	84.1%	87.5%	80.6%
Brazil	Jardim de Angicos	2000	84.8%	87.4%	81.8%

Country	Second Administrative Unit	Year	Mean	Upper	Lower
Brazil	Jardim de Angicos	2017	85.3%	87.9%	82.5%
Brazil	Jardim do Mulato	2000	59.2%	63.6%	54.3%
Brazil	Jardim do Mulato	2017	60.1%	64.5%	55.2%
Brazil	Jardim do Seridó	2000	78.0%	81.3%	74.5%
Brazil	Jardim do Seridó	2017	78.6%	81.9%	75.2%
Brazil	Jardim Olinda	2000	84.7%	88.1%	80.6%
Brazil	Jardim Olinda	2017	85.1%	88.4%	81.2%
Brazil	Jardim-Piranhas	2000	77.3%	80.5%	73.6%
Brazil	Jardim-Piranhas	2017	77.9%	81.1%	74.3%
Brazil	Jardinópolis	2000	77.6%	81.9%	73.2%
Brazil	Jardinópolis	2000	91.6%	93.5%	89.1%
Brazil	Jardinópolis	2017	78.2%	82.4%	74.0%
Brazil	Jardinópolis	2017	91.9%	93.7%	89.5%
Brazil	Jari	2000	83.1%	87.1%	78.3%
Brazil	Jari	2017	83.7%	87.5%	79.0%
Brazil	Jarinu	2000	93.2%	94.3%	91.9%
Brazil	Jarinu	2017	93.4%	94.4%	92.1%
Brazil	Jaru	2000	30.0%	33.8%	26.8%
Brazil	Jaru	2017	30.9%	34.7%	27.6%
Brazil	Jataí	2000	79.5%	84.2%	73.4%
Brazil	Jataí	2017	80.1%	84.7%	74.1%
Brazil	Jataizinho	2000	86.7%	88.7%	84.2%
Brazil	Jataizinho	2017	87.1%	89.1%	84.7%
Brazil	Jataúba	2000	67.6%	71.1%	63.4%
Brazil	Jataúba	2017	68.4%	72.0%	64.2%
Brazil	Jateí	2000	80.2%	83.6%	76.1%
Brazil	Jateí	2017	80.8%	84.1%	76.8%
Brazil	Jati	2000	67.4%	71.3%	62.8%
Brazil	Jati	2017	68.2%	72.0%	63.7%
Brazil	Jatobá	2000	64.2%	69.6%	59.2%
Brazil	Jatobá	2000	61.8%	67.5%	55.6%
Brazil	Jatobá	2017	65.1%	70.4%	60.1%
Brazil	Jatobá	2017	62.8%	68.4%	56.6%
Brazil	Jatobá do Piauí	2000	60.5%	64.7%	55.6%
Brazil	Jatobá do Piauí	2017	61.4%	65.5%	56.5%
Brazil	Jaú	2000	93.8%	95.5%	91.9%
Brazil	Jaú	2017	94.1%	95.7%	92.2%
Brazil	Jaú do Tocantins	2000	77.9%	82.1%	72.7%
Brazil	Jaú do Tocantins	2017	78.5%	82.7%	73.4%
Brazil	Jaupaci	2000	74.4%	79.3%	68.6%
Brazil	Jaupaci	2017	75.1%	79.9%	69.4%
Brazil	Jauru	2000	68.7%	76.0%	61.3%
Brazil	Jauru	2017	69.6%	76.8%	62.2%
Brazil	Jeceaba	2000	81.0%	84.6%	77.7%
Brazil	Jeceaba	2017	81.7%	85.1%	78.3%
Brazil	Jenipapo de Minas	2000	82.4%	87.1%	76.7%
Brazil	Jenipapo de Minas	2017	82.9%	87.5%	77.4%
Brazil	Jenipapo dos Vieiras	2000	61.8%	68.7%	55.8%

Country	Second Administrative Unit	Year	Mean	Upper	Lower
Brazil	Jenipapo dos Vieiras	2017	62.7%	69.5%	56.8%
Brazil	Jequeri	2000	81.2%	85.2%	77.0%
Brazil	Jequeri	2017	81.8%	85.7%	77.7%
Brazil	Jequié	2000	79.1%	83.8%	74.6%
Brazil	Jequié	2017	79.8%	84.4%	75.4%
Brazil	Jequitaí	2000	81.5%	86.9%	74.5%
Brazil	Jequitaí	2017	82.0%	87.3%	75.2%
Brazil	Jequitibá	2000	80.9%	84.6%	76.2%
Brazil	Jequitibá	2017	81.5%	85.1%	76.9%
Brazil	Jequitinhonha	2000	79.5%	84.2%	73.2%
Brazil	Jequitinhonha	2017	80.1%	84.7%	73.9%
Brazil	Jeremoabo	2000	78.5%	81.9%	74.6%
Brazil	Jeremoabo	2017	79.1%	82.4%	75.2%
Brazil	Jericó	2000	74.4%	78.0%	70.7%
Brazil	Jericó	2017	75.1%	78.6%	71.5%
Brazil	Jeriquara	2000	88.2%	91.4%	84.2%
Brazil	Jeriquara	2017	88.6%	91.7%	84.7%
Brazil	Jerônimo Monteiro	2000	77.5%	81.0%	74.0%
Brazil	Jerônimo Monteiro	2017	78.2%	81.6%	74.8%
Brazil	Jerumenha	2000	60.2%	65.9%	53.9%
Brazil	Jerumenha	2017	61.1%	66.7%	54.9%
Brazil	Jesuânia	2000	86.3%	89.4%	82.5%
Brazil	Jesuânia	2017	86.7%	89.8%	83.0%
Brazil	Jesuítas	2000	84.8%	88.3%	81.0%
Brazil	Jesuítas	2017	85.3%	88.7%	81.6%
Brazil	Jesúpolis	2000	77.5%	81.1%	73.5%
Brazil	Jesúpolis	2017	78.2%	81.7%	74.3%
Brazil	Ji-Paraná	2000	30.0%	33.5%	26.8%
Brazil	Ji-Paraná	2017	30.8%	34.4%	27.5%
Brazil	Jijoca de Jericoacoara	2000	66.8%	72.4%	60.4%
Brazil	Jijoca de Jericoacoara	2017	67.7%	73.2%	61.3%
Brazil	Jiquiriçá	2000	72.6%	77.4%	67.7%
Brazil	Jiquiriçá	2017	73.4%	78.0%	68.6%
Brazil	Jitaúna	2000	76.7%	81.5%	71.9%
Brazil	Jitaúna	2017	77.4%	82.1%	72.7%
Brazil	Joaçaba	2000	77.4%	81.4%	72.7%
Brazil	Joaçaba	2017	78.1%	82.0%	73.5%
Brazil	Joaíma	2000	80.8%	85.8%	75.0%
Brazil	Joaíma	2017	81.4%	86.2%	75.8%
Brazil	Joanésia	2000	81.1%	85.1%	76.2%
Brazil	Joanésia	2017	81.7%	85.6%	76.9%
Brazil	Joanópolis	2000	93.2%	94.4%	91.7%
Brazil	Joanópolis	2017	93.4%	94.7%	92.0%
Brazil	João Alfredo	2000	63.2%	67.0%	60.0%
Brazil	João Alfredo	2017	64.1%	67.9%	61.0%
Brazil	João Câmara	2000	86.0%	88.6%	83.3%
Brazil	João Câmara	2017	86.5%	89.0%	83.8%
Brazil	João Costa	2000	61.8%	67.6%	55.7%
Brazil	João Costa	2017	62.7%	68.5%	56.7%
Brazil	João Dias	2000	75.9%	79.6%	72.6%
Brazil	João Dias	2017	76.6%	80.2%	73.4%
Brazil	João Dourado	2000	78.4%	84.0%	72.9%
Brazil	João Dourado	2017	78.1%	83.6%	72.5%
Brazil	João Lisboa	2000	65.3%	69.6%	60.4%
Brazil	João Lisboa	2017	66.2%	70.5%	61.2%
Brazil	João Monlevade	2000	82.9%	86.4%	79.8%

Country	Second Administrative Unit	Year	Mean	Upper	Lower
Brazil	João Monlevade	2017	83.5%	86.9%	80.4%
Brazil	João Neiva	2000	76.4%	79.4%	73.4%
Brazil	João Neiva	2017	77.1%	80.0%	74.2%
Brazil	João Pessoa	2000	66.3%	68.7%	63.7%
Brazil	João Pessoa	2017	67.2%	69.6%	64.6%
Brazil	João Pinheiro	2000	80.7%	86.2%	73.9%
Brazil	João Pinheiro	2017	81.3%	86.6%	74.6%
Brazil	João Ramalho	2000	90.7%	93.1%	88.2%
Brazil	João Ramalho	2017	91.0%	93.4%	88.6%
Brazil	Joaquim Felício	2000	81.3%	85.9%	75.1%
Brazil	Joaquim Felício	2017	81.8%	86.4%	75.8%
Brazil	Joaquim Gomes	2000	60.6%	63.5%	57.8%
Brazil	Joaquim Gomes	2017	61.5%	64.4%	58.8%
Brazil	Joaquim Nabuco	2000	63.9%	67.7%	60.5%
Brazil	Joaquim Nabuco	2017	64.8%	68.5%	61.4%
Brazil	Joaquim Pires	2000	60.1%	64.7%	55.6%
Brazil	Joaquim Pires	2017	61.0%	65.6%	56.5%
Brazil	Joaquim Távora	2000	88.3%	90.8%	84.7%
Brazil	Joaquim Távora	2017	88.7%	91.1%	85.2%
Brazil	Joca Marques	2000	59.7%	64.2%	54.6%
Brazil	Joca Marques	2017	60.6%	65.0%	55.5%
Brazil	Jóia	2000	83.0%	86.6%	78.6%
Brazil	Jóia	2017	83.6%	87.0%	79.3%
Brazil	Joinvile	2000	75.9%	79.2%	71.6%
Brazil	Joinvile	2017	76.6%	79.9%	72.4%
Brazil	Jordânia	2000	82.6%	86.9%	77.3%
Brazil	Jordânia	2017	83.2%	87.3%	78.0%
Brazil	Jordão	2000	37.6%	43.3%	32.1%
Brazil	Jordão	2017	38.4%	44.2%	32.8%
Brazil	José Boiteux	2000	73.4%	77.6%	69.3%
Brazil	José Boiteux	2017	74.3%	78.4%	70.2%
Brazil	José Bonifácio	2000	93.2%	95.0%	91.1%
Brazil	José Bonifácio	2017	93.4%	95.2%	91.4%
Brazil	José da Penha	2000	76.1%	79.8%	72.5%
Brazil	José da Penha	2017	76.9%	80.4%	73.3%
Brazil	José de Freitas	2000	61.6%	65.6%	58.1%
Brazil	José de Freitas	2017	62.5%	66.5%	59.1%
Brazil	José Gonçalves de Minas	2000	81.5%	86.2%	75.5%
Brazil	José Gonçalves de Minas	2017	82.1%	86.7%	76.2%
Brazil	José Raydan	2000	82.3%	87.1%	76.8%
Brazil	José Raydan	2017	82.8%	87.5%	77.5%
Brazil	Joselândia	2000	58.7%	64.4%	53.6%
Brazil	Joselândia	2017	59.6%	65.3%	54.6%
Brazil	Josenópolis	2000	81.3%	86.3%	76.2%
Brazil	Josenópolis	2017	81.8%	86.8%	76.8%
Brazil	Joviânia	2000	77.1%	81.4%	72.8%
Brazil	Joviânia	2017	77.7%	82.0%	73.6%
Brazil	Juara	2000	67.1%	74.6%	59.3%
Brazil	Juara	2017	68.2%	75.4%	60.5%

Country	Second Administrative Unit	Year	Mean	Upper	Lower
Brazil	Juarez Távora	2000	66.6%	69.6%	63.3%
Brazil	Juarez Távora	2017	67.5%	70.5%	64.2%
Brazil	Juarina	2000	63.8%	68.9%	58.3%
Brazil	Juarina	2017	64.5%	69.6%	59.1%
Brazil	Juatuba	2000	80.7%	83.8%	77.1%
Brazil	Juatuba	2017	81.3%	84.3%	77.7%
Brazil	Juazeirinho	2000	71.8%	75.6%	67.5%
Brazil	Juazeirinho	2017	72.6%	76.3%	68.4%
Brazil	Juazeiro	2000	75.1%	79.3%	70.5%
Brazil	Juazeiro	2017	75.8%	80.0%	71.3%
Brazil	Juazeiro do Norte	2000	70.3%	73.5%	67.1%
Brazil	Juazeiro do Norte	2017	71.1%	74.2%	68.0%
Brazil	Juazeiro do Piauí	2000	61.5%	66.0%	56.1%
Brazil	Juazeiro do Piauí	2017	62.5%	66.9%	57.1%
Brazil	Jucás	2000	67.8%	71.7%	63.3%
Brazil	Jucás	2017	68.7%	72.5%	64.2%
Brazil	Jucati	2000	65.9%	69.1%	62.3%
Brazil	Jucati	2017	66.7%	70.0%	63.3%
Brazil	Jucuruçu	2000	81.3%	85.5%	75.7%
Brazil	Jucuruçu	2017	81.9%	85.9%	76.4%
Brazil	Jucurutu	2000	78.9%	82.3%	75.5%
Brazil	Jucurutu	2017	80.1%	83.3%	76.8%
Brazil	Juína	2000	62.0%	71.3%	52.4%
Brazil	Juína	2017	62.8%	72.0%	53.4%
Brazil	Juiz de Fora	2000	81.7%	84.8%	78.5%
Brazil	Juiz de Fora	2017	82.3%	85.3%	79.2%
Brazil	Júlio Borges	2000	66.4%	73.8%	58.4%
Brazil	Júlio Borges	2017	67.3%	74.5%	59.4%
Brazil	Júlio de Castilhos	2000	84.2%	87.6%	80.0%
Brazil	Júlio de Castilhos	2017	85.7%	88.7%	81.7%
Brazil	Júlio Mesquita	2000	92.9%	94.9%	90.4%
Brazil	Júlio Mesquita	2017	93.2%	95.1%	90.7%
Brazil	Jumirim	2000	92.9%	94.4%	91.1%
Brazil	Jumirim	2017	93.2%	94.6%	91.4%
Brazil	Junco	2000	80.2%	83.5%	76.9%
Brazil	Junco	2017	80.8%	84.1%	77.6%
Brazil	Junco do Maranhão	2000	48.1%	55.0%	42.4%
Brazil	Junco do Maranhão	2017	49.0%	55.8%	43.1%
Brazil	Junco do Seridó	2000	73.2%	76.9%	68.8%
Brazil	Junco do Seridó	2017	74.0%	77.6%	69.6%
Brazil	Jundiá	2000	61.5%	64.7%	58.3%
Brazil	Jundiá	2017	62.5%	65.6%	59.3%
Brazil	Jundiaí	2000	94.2%	95.1%	93.2%
Brazil	Jundiaí	2017	94.4%	95.2%	93.4%
Brazil	Jundiaí do Sul	2000	87.8%	90.5%	84.3%
Brazil	Jundiaí do Sul	2017	88.2%	90.8%	84.8%
Brazil	Junqueiro	2000	60.8%	63.8%	58.1%
Brazil	Junqueiro	2017	61.7%	64.7%	59.0%
Brazil	Junqueirópolis	2000	89.7%	92.1%	86.6%
Brazil	Junqueirópolis	2017	90.0%	92.3%	87.0%

Country	Second Administrative Unit	Year	Mean	Upper	Lower
Brazil	Jupiá	2000	66.2%	69.2%	62.7%
Brazil	Jupiá	2000	80.1%	84.2%	76.1%
Brazil	Jupiá	2017	67.0%	70.1%	63.6%
Brazil	Jupiá	2017	80.8%	84.7%	76.8%
Brazil	Juquiá	2000	89.9%	92.7%	86.7%
Brazil	Juquiá	2017	90.2%	93.0%	87.2%
Brazil	Juquitiba	2000	93.5%	94.7%	91.8%
Brazil	Juquitiba	2017	93.7%	94.9%	92.1%
Brazil	Juramento	2000	82.6%	87.3%	76.7%
Brazil	Juramento	2017	83.1%	87.7%	77.3%
Brazil	Juranda	2000	83.7%	87.3%	79.5%
Brazil	Juranda	2017	84.2%	87.7%	80.1%
Brazil	Jurema	2000	64.8%	70.6%	57.6%
Brazil	Jurema	2000	67.7%	71.2%	64.4%
Brazil	Jurema	2017	68.6%	72.0%	65.3%
Brazil	Jurema	2017	65.6%	71.3%	58.7%
Brazil	Juripiranga	2000	69.0%	72.0%	66.0%
Brazil	Juripiranga	2017	69.9%	72.8%	66.9%
Brazil	Juru	2000	67.0%	71.0%	62.9%
Brazil	Juru	2017	67.9%	71.8%	63.8%
Brazil	Juruá	2000	56.4%	64.1%	50.1%
Brazil	Juruá	2017	58.9%	66.4%	52.4%
Brazil	Juruaia	2000	86.4%	89.5%	82.4%
Brazil	Juruaia	2017	87.6%	90.5%	84.0%
Brazil	Juruena	2000	64.9%	73.6%	56.2%
Brazil	Juruena	2017	65.8%	74.4%	57.2%
Brazil	Juruti	2000	45.8%	52.2%	38.7%
Brazil	Juruti	2017	47.1%	53.6%	39.9%
Brazil	Juscimeira	2000	69.3%	74.3%	64.1%
Brazil	Juscimeira	2017	70.1%	75.1%	65.0%
Brazil	Jussara	2000	75.1%	80.0%	69.7%
Brazil	Jussara	2000	84.0%	86.9%	80.6%
Brazil	Jussara	2000	76.5%	82.6%	69.2%
Brazil	Jussara	2017	77.2%	83.1%	70.0%
Brazil	Jussara	2017	75.2%	80.0%	69.7%
Brazil	Jussara	2017	84.5%	87.4%	81.2%
Brazil	Jussari	2000	77.5%	81.7%	72.0%
Brazil	Jussari	2017	78.1%	82.3%	72.8%
Brazil	Jussiape	2000	77.7%	82.8%	72.0%
Brazil	Jussiape	2017	78.3%	83.3%	72.7%
Brazil	Jutaí	2000	58.5%	66.7%	50.2%
Brazil	Jutaí	2017	59.4%	67.6%	51.2%
Brazil	Juti	2000	80.3%	84.1%	75.6%
Brazil	Juti	2017	80.9%	84.6%	76.3%
Brazil	Juvenília	2000	78.8%	85.3%	71.0%
Brazil	Juvenília	2017	79.5%	85.7%	71.9%
Brazil	Kaloré	2000	83.8%	86.7%	80.2%
Brazil	Kaloré	2017	84.3%	87.2%	80.8%
Brazil	Lábrea	2000	55.0%	62.9%	47.1%
Brazil	Lábrea	2017	55.9%	63.8%	48.1%
Brazil	Lacerdópolis	2000	75.9%	80.0%	71.2%
Brazil	Lacerdópolis	2017	76.6%	80.7%	72.0%
Brazil	Ladainha	2000	82.7%	87.5%	77.1%
Brazil	Ladainha	2017	83.3%	87.9%	77.8%
Brazil	Lafaiete Coutinho	2000	80.1%	84.6%	74.9%
Brazil	Lafaiete Coutinho	2017	80.7%	85.1%	75.7%
Brazil	Lagamar	2000	79.3%	85.0%	72.1%
Brazil	Lagamar	2017	79.9%	85.5%	72.9%
Brazil	Lagarto	2000	77.5%	79.9%	75.3%
Brazil	Lagarto	2017	78.4%	80.7%	76.2%

Country	Second Administrative Unit	Year	Mean	Upper	Lower
Brazil	Lages	2000	79.5%	83.5%	74.8%
Brazil	Lages	2017	79.9%	83.9%	75.4%
Brazil	Lago da Pedra	2000	60.6%	65.6%	55.6%
Brazil	Lago da Pedra	2017	61.6%	66.5%	56.6%
Brazil	Lago do Junco	2000	60.5%	66.0%	55.0%
Brazil	Lago do Junco	2017	61.4%	66.8%	56.0%
Brazil	Lago dos Rodrigues	2000	60.3%	66.2%	54.6%
Brazil	Lago dos Rodrigues	2017	61.2%	67.0%	55.6%
Brazil	Lago Verde	2000	60.3%	65.1%	54.8%
Brazil	Lago Verde	2017	62.4%	67.1%	56.9%
Brazil	Lagoa	2000	74.8%	78.4%	71.0%
Brazil	Lagoa	2017	75.5%	79.1%	71.8%
Brazil	Lagoa Alegre	2000	60.2%	65.2%	55.8%
Brazil	Lagoa Alegre	2017	61.1%	66.1%	56.7%
Brazil	Lagoa da Canoa	2000	64.5%	67.5%	61.6%
Brazil	Lagoa da Canoa	2017	65.3%	68.3%	62.5%
Brazil	Lagoa da Confusão	2000	70.9%	76.1%	64.3%
Brazil	Lagoa da Confusão	2017	72.1%	77.1%	65.8%
Brazil	Lagoa da Prata	2000	81.1%	85.5%	76.0%
Brazil	Lagoa da Prata	2017	81.6%	86.0%	76.7%
Brazil	Lagoa de Anta	2000	79.2%	81.8%	76.6%
Brazil	Lagoa de Anta	2017	79.8%	82.3%	77.2%
Brazil	Lagoa de dentro	2000	73.4%	76.0%	70.3%
Brazil	Lagoa de dentro	2017	74.1%	76.7%	71.1%
Brazil	Lagoa de Pedras	2000	80.6%	82.8%	78.4%
Brazil	Lagoa de Pedras	2017	81.2%	83.4%	79.0%
Brazil	Lagoa de São Francisco	2000	64.6%	69.0%	59.4%
Brazil	Lagoa de São Francisco	2017	65.5%	69.9%	60.4%
Brazil	Lagoa do Barro do Piauí	2000	64.4%	70.4%	57.9%
Brazil	Lagoa do Barro do Piauí	2017	65.2%	71.2%	58.8%
Brazil	Lagoa do Carro	2000	72.5%	75.3%	70.2%
Brazil	Lagoa do Carro	2017	73.4%	76.1%	71.1%
Brazil	Lagoa do Itaenga	2000	71.0%	73.7%	68.5%
Brazil	Lagoa do Itaenga	2017	71.9%	74.4%	69.4%
Brazil	Lagoa do Mato	2000	61.3%	67.3%	54.7%
Brazil	Lagoa do Mato	2017	63.5%	69.3%	57.1%
Brazil	Lagoa do Ouro	2000	60.1%	63.5%	56.3%

Country	Second Administrative Unit	Year	Mean	Upper	Lower
Brazil	Lagoa do Ouro	2017	61.0%	64.4%	57.3%
Brazil	Lagoa do Piauí	2000	59.1%	63.0%	55.0%
Brazil	Lagoa do Piauí	2017	60.1%	64.0%	55.9%
Brazil	Lagoa do Sítio	2000	62.4%	68.4%	57.0%
Brazil	Lagoa do Sítio	2017	63.3%	69.2%	57.9%
Brazil	Lagoa do Tocantins	2000	77.2%	82.3%	70.9%
Brazil	Lagoa do Tocantins	2017	77.9%	82.9%	71.7%
Brazil	Lagoa dos Gatos	2000	65.3%	69.2%	62.1%
Brazil	Lagoa dos Gatos	2017	66.1%	69.9%	62.9%
Brazil	Lagoa dos Patos	2000	80.9%	86.1%	73.9%
Brazil	Lagoa dos Patos	2017	81.5%	86.6%	74.7%
Brazil	Lagoa dos Três Cantos	2000	81.9%	85.8%	77.3%
Brazil	Lagoa dos Três Cantos	2017	82.4%	86.3%	78.0%
Brazil	Lagoa dourada	2000	81.8%	85.9%	77.9%
Brazil	Lagoa dourada	2017	82.4%	86.4%	78.6%
Brazil	Lagoa Formosa	2000	81.0%	86.2%	74.4%
Brazil	Lagoa Formosa	2017	81.6%	86.7%	75.1%
Brazil	Lagoa Grande	2000	65.5%	70.8%	59.3%
Brazil	Lagoa Grande	2000	79.7%	85.2%	73.0%
Brazil	Lagoa Grande	2017	66.3%	71.6%	60.2%
Brazil	Lagoa Grande	2017	80.4%	85.8%	73.9%
Brazil	Lagoa Grande do Maranhão	2000	60.4%	65.7%	55.1%
Brazil	Lagoa Grande do Maranhão	2017	61.3%	66.6%	56.1%
Brazil	Lagoa Mirim	2000	78.6%	85.3%	70.6%
Brazil	Lagoa Mirim	2017	79.4%	85.7%	71.9%
Brazil	Lagoa Nova	2000	84.2%	86.8%	81.3%
Brazil	Lagoa Nova	2017	84.7%	87.2%	81.9%
Brazil	Lagoa Real	2000	77.6%	82.9%	72.5%
Brazil	Lagoa Real	2017	78.3%	83.4%	73.3%
Brazil	Lagoa Salgada	2000	81.1%	83.2%	78.8%
Brazil	Lagoa Salgada	2017	81.7%	83.8%	79.5%
Brazil	Lagoa Santa	2000	81.3%	84.1%	77.9%
Brazil	Lagoa Santa	2017	82.5%	85.2%	79.4%
Brazil	Lagoa Seca	2000	68.2%	71.1%	65.5%
Brazil	Lagoa Seca	2017	69.1%	71.9%	66.4%
Brazil	Lagoa Vermelha	2000	80.3%	83.9%	76.2%
Brazil	Lagoa Vermelha	2017	80.9%	84.4%	76.9%
Brazil	Lagoão	2000	83.7%	86.8%	80.1%
Brazil	Lagoão	2017	84.3%	87.3%	80.8%
Brazil	Lagoas de Velhos	2000	82.6%	85.2%	80.2%
Brazil	Lagoas de Velhos	2017	83.1%	85.7%	80.8%

Country	Second Administrative Unit	Year	Mean	Upper	Lower
Brazil	Lagoinha	2000	91.3%	93.2%	89.1%
Brazil	Lagoinha	2017	91.6%	93.5%	89.4%
Brazil	Lagoinha do Piauí	2000	60.3%	64.6%	55.1%
Brazil	Lagoinha do Piauí	2017	61.1%	65.5%	56.0%
Brazil	Laguna	2000	72.2%	77.7%	66.5%
Brazil	Laguna	2017	73.1%	78.5%	67.5%
Brazil	Laguna Carapã	2000	81.3%	84.6%	77.5%
Brazil	Laguna Carapã	2017	81.9%	85.1%	78.2%
Brazil	Laje	2000	76.7%	80.5%	72.1%
Brazil	Laje	2017	77.4%	81.1%	72.9%
Brazil	Laje do Muriaé	2000	77.7%	81.2%	73.6%
Brazil	Laje do Muriaé	2017	78.4%	81.8%	74.4%
Brazil	Lajeado do Bugre	2000	82.9%	86.4%	79.5%
Brazil	Lajeado do Bugre	2017	83.5%	86.8%	80.1%
Brazil	Lajeado Grande	2000	76.8%	81.4%	72.6%
Brazil	Lajeado Grande	2017	77.5%	82.0%	73.3%
Brazil	Lajeado Novo	2000	68.1%	73.7%	62.6%
Brazil	Lajeado Novo	2017	68.9%	74.4%	63.5%
Brazil	Lajedao	2000	81.1%	85.5%	75.9%
Brazil	Lajedao	2017	81.7%	86.0%	76.6%
Brazil	Lajedão	2000	80.8%	84.2%	77.6%
Brazil	Lajedão	2000	74.1%	78.2%	70.2%
Brazil	Lajedão	2017	74.9%	78.9%	71.1%
Brazil	Lajedão	2017	81.4%	84.7%	78.3%
Brazil	Lajedinho	2000	79.0%	84.1%	73.5%
Brazil	Lajedinho	2017	79.7%	84.6%	74.2%
Brazil	Lajedo	2000	67.0%	70.2%	63.9%
Brazil	Lajedo	2017	67.9%	71.1%	64.8%
Brazil	Lajedo do Tabocal	2000	81.6%	85.8%	76.3%
Brazil	Lajedo do Tabocal	2017	82.3%	86.3%	77.1%
Brazil	Lajes	2000	84.8%	87.5%	82.0%
Brazil	Lajes	2017	85.3%	87.9%	82.5%
Brazil	Lajes Pintadas	2000	81.4%	84.4%	78.4%
Brazil	Lajes Pintadas	2017	82.0%	84.9%	79.0%
Brazil	Lajinha	2000	80.0%	83.6%	76.5%
Brazil	Lajinha	2017	80.6%	84.1%	77.2%
Brazil	Lamarão	2000	78.5%	82.0%	74.4%
Brazil	Lamarão	2017	79.1%	82.6%	75.1%
Brazil	Lambari	2000	86.2%	89.4%	82.7%
Brazil	Lambari	2017	86.7%	89.8%	83.3%
Brazil	Lambari d'Oeste	2000	67.2%	73.0%	60.0%
Brazil	Lambari d'Oeste	2017	68.0%	73.7%	60.9%
Brazil	Lamim	2000	81.0%	84.4%	77.0%
Brazil	Lamim	2017	81.6%	84.9%	77.7%
Brazil	Landri Sales	2000	61.5%	67.7%	54.7%
Brazil	Landri Sales	2017	62.4%	68.6%	55.6%

Country	Second Administrative Unit	Year	Mean	Upper	Lower
Brazil	Lapão	2000	77.0%	82.7%	71.2%
Brazil	Lapão	2017	77.7%	83.2%	72.0%
Brazil	Laranja da Terra	2000	78.8%	82.4%	75.3%
Brazil	Laranja da Terra	2017	79.5%	83.0%	76.0%
Brazil	Laranjal	2000	83.3%	87.1%	78.9%
Brazil	Laranjal	2000	78.1%	81.8%	73.6%
Brazil	Laranjal	2017	83.8%	87.5%	79.6%
Brazil	Laranjal	2017	78.8%	82.4%	74.4%
Brazil	Laranjal do Jari	2000	47.2%	53.7%	40.7%
Brazil	Laranjal do Jari	2017	48.2%	54.6%	41.7%
Brazil	Laranjal Paulista	2000	92.9%	94.4%	91.1%
Brazil	Laranjal Paulista	2017	93.2%	94.6%	91.4%
Brazil	Laranjeiras	2000	75.4%	77.2%	73.4%
Brazil	Laranjeiras	2017	76.1%	77.9%	74.1%
Brazil	Laranjeiras do Sul	2000	83.8%	87.7%	79.4%
Brazil	Laranjeiras do Sul	2017	84.5%	88.2%	80.3%
Brazil	Lassance	2000	80.9%	86.4%	75.5%
Brazil	Lassance	2017	81.6%	86.9%	76.3%
Brazil	Lastro	2000	74.8%	78.2%	71.5%
Brazil	Lastro	2017	75.7%	79.0%	72.5%
Brazil	Laurentino	2000	73.4%	77.3%	69.2%
Brazil	Laurentino	2017	74.1%	78.0%	70.0%
Brazil	Lauro de Freitas	2000	77.6%	80.3%	74.4%
Brazil	Lauro de Freitas	2017	78.3%	80.9%	75.1%
Brazil	Lauro Muller	2000	74.6%	78.8%	70.0%
Brazil	Lauro Muller	2017	75.3%	79.5%	70.8%
Brazil	Lavandeira	2000	76.6%	81.5%	70.1%
Brazil	Lavandeira	2017	77.3%	82.1%	70.9%
Brazil	Lavínia	2000	90.2%	92.4%	87.5%
Brazil	Lavínia	2017	90.4%	92.6%	87.8%
Brazil	Lavras	2000	82.8%	86.3%	78.4%
Brazil	Lavras	2017	83.4%	86.8%	79.0%
Brazil	Lavras da Mangabeira	2000	68.3%	72.1%	64.6%
Brazil	Lavras da Mangabeira	2017	69.2%	72.9%	65.5%
Brazil	Lavras do Sul	2000	84.2%	88.6%	79.7%
Brazil	Lavras do Sul	2017	84.7%	89.0%	80.4%
Brazil	Lavrinhas	2000	85.8%	88.4%	83.0%
Brazil	Lavrinhas	2017	86.3%	88.8%	83.5%
Brazil	Leandro Ferreira	2000	80.0%	84.3%	75.5%
Brazil	Leandro Ferreira	2017	80.6%	84.8%	76.2%
Brazil	Lebon Régis	2000	78.3%	82.7%	73.8%
Brazil	Lebon Régis	2017	78.9%	83.3%	74.5%
Brazil	Leme	2000	92.6%	94.1%	90.7%
Brazil	Leme	2017	92.9%	94.3%	91.0%
Brazil	Leme do Prado	2000	82.3%	87.1%	76.5%
Brazil	Leme do Prado	2017	82.9%	87.5%	77.2%

Country	Second Administrative Unit	Year	Mean	Upper	Lower
Brazil	Lençóis	2000	75.4%	80.8%	68.8%
Brazil	Lençóis	2017	76.2%	81.4%	69.7%
Brazil	Lençóis Paulista	2000	93.8%	95.4%	91.8%
Brazil	Lençóis Paulista	2017	94.0%	95.6%	92.1%
Brazil	Leoberto Leal	2000	76.0%	79.5%	71.6%
Brazil	Leoberto Leal	2017	76.8%	80.2%	72.5%
Brazil	Leopoldina	2000	78.7%	82.3%	74.7%
Brazil	Leopoldina	2017	79.4%	82.9%	75.4%
Brazil	Leopoldo de Bulhões	2000	79.2%	81.5%	76.6%
Brazil	Leopoldo de Bulhões	2017	79.8%	82.0%	77.2%
Brazil	Leópolis	2000	87.5%	89.7%	85.0%
Brazil	Leópolis	2017	87.9%	90.0%	85.5%
Brazil	Liberato Salzano	2000	77.9%	81.6%	73.9%
Brazil	Liberato Salzano	2017	78.5%	82.1%	74.6%
Brazil	Liberdade	2000	84.5%	87.7%	81.2%
Brazil	Liberdade	2017	85.0%	88.1%	81.8%
Brazil	Licínio de Almeida	2000	78.4%	83.7%	72.7%
Brazil	Licínio de Almeida	2017	79.0%	84.2%	73.4%
Brazil	Lidianópolis	2000	83.2%	86.5%	79.7%
Brazil	Lidianópolis	2017	83.7%	86.9%	80.3%
Brazil	Lima Campos	2000	60.8%	66.2%	55.1%
Brazil	Lima Campos	2017	61.7%	67.0%	56.0%
Brazil	Lima Duarte	2000	82.0%	85.1%	78.3%
Brazil	Lima Duarte	2017	82.6%	85.6%	79.0%
Brazil	Limeira	2000	93.6%	94.9%	92.1%
Brazil	Limeira	2017	93.9%	95.2%	92.4%
Brazil	Limeira do Oeste	2000	83.8%	87.7%	79.4%
Brazil	Limeira do Oeste	2017	84.3%	88.1%	80.0%
Brazil	Limoeiro	2000	66.4%	70.1%	63.5%
Brazil	Limoeiro	2017	67.2%	70.9%	64.4%
Brazil	Limoeiro de Anadia	2000	61.0%	63.7%	58.7%
Brazil	Limoeiro de Anadia	2017	62.0%	64.6%	59.6%
Brazil	Limoeiro do Ajuru	2000	37.4%	43.0%	32.5%
Brazil	Limoeiro do Ajuru	2017	38.3%	43.9%	33.4%
Brazil	Limoeiro do Norte	2000	76.3%	79.9%	72.7%
Brazil	Limoeiro do Norte	2017	77.0%	80.5%	73.5%
Brazil	Lindoeste	2000	84.0%	87.1%	80.7%
Brazil	Lindoeste	2017	84.5%	87.5%	81.3%
Brazil	Lindóia	2000	91.2%	93.0%	89.3%
Brazil	Lindóia	2017	91.5%	93.2%	89.6%
Brazil	Lindóia do Sul	2000	76.7%	80.1%	72.0%
Brazil	Lindóia do Sul	2017	77.3%	80.7%	72.8%
Brazil	Lindolfo Collor	2000	79.9%	82.3%	77.2%
Brazil	Lindolfo Collor	2017	80.6%	82.9%	77.9%

Country	Second Administrative Unit	Year	Mean	Upper	Lower
Brazil	Linha Nova	2000	81.7%	84.4%	79.1%
Brazil	Linha Nova	2017	82.3%	85.0%	79.8%
Brazil	Linhares	2000	75.8%	79.5%	71.2%
Brazil	Linhares	2017	76.5%	80.1%	71.9%
Brazil	Lins	2000	93.6%	95.3%	91.4%
Brazil	Lins	2017	93.8%	95.5%	91.6%
Brazil	Livramento	2000	69.6%	74.1%	64.9%
Brazil	Livramento	2017	70.5%	74.9%	65.8%
Brazil	Livramento do Brumado	2000	78.0%	83.2%	72.5%
Brazil	Livramento do Brumado	2017	79.2%	84.1%	73.9%
Brazil	Lizarda	2000	74.0%	80.0%	66.8%
Brazil	Lizarda	2017	74.8%	80.6%	67.7%
Brazil	Loanda	2000	84.7%	88.0%	81.2%
Brazil	Loanda	2017	85.2%	88.4%	81.8%
Brazil	Lobato	2000	85.7%	88.2%	82.8%
Brazil	Lobato	2017	86.2%	88.6%	83.4%
Brazil	Logradouro	2000	76.6%	79.2%	74.0%
Brazil	Logradouro	2017	77.2%	79.8%	74.7%
Brazil	Londrina	2000	86.7%	88.7%	84.1%
Brazil	Londrina	2017	87.2%	89.1%	84.6%
Brazil	Lontra	2000	81.8%	86.9%	76.1%
Brazil	Lontra	2017	82.4%	87.4%	76.8%
Brazil	Lontras	2000	73.1%	76.6%	69.6%
Brazil	Lontras	2017	73.8%	77.3%	70.4%
Brazil	Lorena	2000	88.1%	90.6%	85.3%
Brazil	Lorena	2017	88.5%	90.9%	85.8%
Brazil	Loreto	2000	63.0%	70.8%	55.1%
Brazil	Loreto	2017	63.9%	71.5%	56.1%
Brazil	Lourdes	2000	90.9%	93.5%	87.8%
Brazil	Lourdes	2017	91.2%	93.7%	88.2%
Brazil	Louveira	2000	93.7%	94.7%	92.6%
Brazil	Louveira	2017	94.0%	94.9%	92.8%
Brazil	Lucas do Rio Verde	2000	69.6%	76.2%	64.1%
Brazil	Lucas do Rio Verde	2017	70.5%	76.9%	65.0%
Brazil	Lucélia	2000	91.2%	93.4%	88.3%
Brazil	Lucélia	2017	91.5%	93.6%	88.7%
Brazil	Lucena	2000	67.8%	71.0%	64.0%
Brazil	Lucena	2017	68.6%	71.8%	64.9%
Brazil	Lucianópolis	2000	92.8%	94.7%	90.6%
Brazil	Lucianópolis	2017	93.0%	94.9%	91.0%
Brazil	Luciára	2000	67.2%	75.3%	58.2%
Brazil	Luciára	2017	67.9%	75.9%	59.1%
Brazil	Lucrécia	2000	76.9%	80.1%	73.3%
Brazil	Lucrécia	2017	77.6%	80.8%	74.1%
Brazil	Luís Antônio	2000	93.7%	95.1%	91.8%
Brazil	Luís Antônio	2017	93.9%	95.3%	92.1%
Brazil	Luís Correia	2000	64.3%	68.8%	60.0%
Brazil	Luís Correia	2017	65.2%	69.7%	61.0%
Brazil	Luís Domingues	2000	47.7%	55.5%	40.6%
Brazil	Luís Domingues	2017	50.0%	57.7%	42.8%
Brazil	Luís Gomes	2000	75.8%	79.6%	72.0%
Brazil	Luís Gomes	2017	76.5%	80.2%	72.7%
Brazil	Luisburgo	2000	81.6%	84.9%	77.5%
Brazil	Luisburgo	2017	82.2%	85.4%	78.2%
Brazil	Luisiania	2000	92.0%	94.3%	89.7%
Brazil	Luisiania	2017	92.2%	94.5%	90.1%

Country	Second Administrative Unit	Year	Mean	Upper	Lower
Brazil	Luislândia	2000	81.6%	86.5%	75.4%
Brazil	Luislândia	2017	82.1%	86.9%	76.1%
Brazil	Luiz Alves	2000	75.4%	78.4%	71.4%
Brazil	Luiz Alves	2017	76.1%	79.1%	72.2%
Brazil	Luiziânia	2000	84.0%	87.3%	80.2%
Brazil	Luiziânia	2017	84.5%	87.7%	80.8%
Brazil	Luminárias	2000	82.1%	85.5%	77.6%
Brazil	Luminárias	2017	82.7%	86.0%	78.3%
Brazil	Lunardelli	2000	83.3%	86.6%	79.7%
Brazil	Lunardelli	2017	83.8%	87.0%	80.3%
Brazil	Lupercio	2000	92.5%	94.3%	90.1%
Brazil	Lupercio	2017	92.7%	94.6%	90.4%
Brazil	Lupionópolis	2000	86.2%	88.7%	82.9%
Brazil	Lupionópolis	2017	86.7%	89.1%	83.5%
Brazil	Lutécia	2000	91.5%	93.8%	89.1%
Brazil	Lutécia	2017	91.8%	94.1%	89.5%
Brazil	Luz	2000	81.9%	86.5%	76.2%
Brazil	Luz	2017	82.5%	86.9%	76.9%
Brazil	Luzerna	2000	76.8%	80.9%	72.1%
Brazil	Luzerna	2017	77.5%	81.5%	72.9%
Brazil	Luziânia	2000	88.4%	89.8%	86.6%
Brazil	Luziânia	2017	88.9%	90.3%	87.2%
Brazil	Luzilândia	2000	61.3%	65.6%	56.6%
Brazil	Luzilândia	2017	62.2%	66.5%	57.5%
Brazil	Luzinópolis	2000	72.4%	76.4%	68.1%
Brazil	Luzinópolis	2017	73.2%	77.1%	68.9%
Brazil	Macaé	2000	76.3%	79.7%	72.6%
Brazil	Macaé	2017	77.6%	80.9%	74.1%
Brazil	Macaíba	2000	82.1%	83.7%	80.4%
Brazil	Macaíba	2017	82.7%	84.3%	81.1%
Brazil	Macajuba	2000	78.9%	83.4%	73.6%
Brazil	Macajuba	2017	79.6%	83.9%	74.4%
Brazil	Maçambara	2000	81.5%	86.1%	75.5%
Brazil	Maçambara	2000	78.5%	80.7%	76.3%
Brazil	Maçambara	2017	82.1%	86.5%	76.2%
Brazil	Maçambara	2017	79.1%	81.2%	77.0%
Brazil	Macapa	2000	43.3%	45.1%	41.7%
Brazil	Macapa	2017	44.4%	46.2%	42.8%
Brazil	Macaparana	2000	67.3%	70.6%	64.4%
Brazil	Macaparana	2017	68.1%	71.3%	65.2%
Brazil	Macarani	2000	81.1%	85.6%	75.5%
Brazil	Macarani	2017	81.7%	86.1%	76.2%
Brazil	Macatuba	2000	94.9%	96.3%	93.3%
Brazil	Macatuba	2017	95.1%	96.4%	93.5%
Brazil	Macau	2000	82.1%	85.3%	78.1%
Brazil	Macau	2017	82.6%	85.8%	78.8%
Brazil	Macaubal	2000	91.9%	94.1%	89.0%
Brazil	Macaubal	2017	92.2%	94.3%	89.4%
Brazil	Macaúbas	2000	77.4%	82.6%	71.6%
Brazil	Macaúbas	2017	78.1%	83.2%	72.4%
Brazil	Macedonia	2000	89.3%	92.4%	86.0%
Brazil	Macedonia	2017	89.6%	92.7%	86.5%
Brazil	Maceió (capital)	2000	60.2%	62.7%	57.8%
Brazil	Maceió (capital)	2017	61.2%	63.6%	58.7%
Brazil	Machacalis	2000	79.7%	84.2%	74.3%
Brazil	Machacalis	2017	80.3%	84.7%	75.0%
Brazil	Machadinho	2000	77.0%	80.6%	72.2%
Brazil	Machadinho	2000	33.8%	39.7%	28.3%
Brazil	Machadinho	2017	77.7%	81.2%	73.0%
Brazil	Machadinho	2017	34.6%	40.6%	29.1%

Country	Second Administrative Unit	Year	Mean	Upper	Lower
Brazil	Machado	2000	84.9%	88.0%	80.3%
Brazil	Machado	2017	85.4%	88.4%	80.9%
Brazil	Machados	2000	66.3%	69.5%	63.2%
Brazil	Machados	2017	67.2%	70.4%	64.2%
Brazil	Macieira	2000	78.5%	82.4%	74.7%
Brazil	Macieira	2017	79.0%	82.8%	75.3%
Brazil	Macuco	2000	79.5%	83.0%	75.6%
Brazil	Macuco	2017	80.1%	83.5%	76.3%
Brazil	Macururé	2000	69.2%	75.1%	62.6%
Brazil	Macururé	2017	70.1%	75.9%	63.5%
Brazil	Madalena	2000	70.6%	75.2%	65.2%
Brazil	Madalena	2017	71.4%	75.9%	66.1%
Brazil	Madeiro	2000	59.5%	64.4%	54.6%
Brazil	Madeiro	2017	60.4%	65.2%	55.5%
Brazil	Madre de deus	2000	74.8%	77.8%	72.2%
Brazil	Madre de deus	2017	75.5%	78.4%	73.0%
Brazil	Madre de deus de Minas	2000	81.2%	85.5%	76.5%
Brazil	Madre de deus de Minas	2017	81.8%	86.0%	77.2%
Brazil	Mãe d'Água	2000	69.9%	73.7%	65.8%
Brazil	Mãe d'Água	2017	70.7%	74.4%	66.6%
Brazil	Maetinga	2000	78.7%	83.4%	73.2%
Brazil	Maetinga	2017	79.2%	83.7%	73.9%
Brazil	Mafra	2000	80.2%	83.4%	76.5%
Brazil	Mafra	2017	80.8%	83.8%	77.2%
Brazil	Magalhães Barata	2000	35.3%	39.9%	30.6%
Brazil	Magalhães Barata	2017	36.1%	40.8%	31.4%
Brazil	Magalhães de Almeida	2000	59.7%	64.4%	54.9%
Brazil	Magalhães de Almeida	2017	60.7%	65.3%	55.9%
Brazil	Magda	2000	91.0%	93.6%	88.1%
Brazil	Magda	2017	91.3%	93.9%	88.5%
Brazil	Magé	2000	75.3%	77.3%	73.3%
Brazil	Magé	2017	76.0%	78.0%	74.0%
Brazil	Maiquinique	2000	80.8%	85.2%	75.2%
Brazil	Maiquinique	2017	81.4%	85.7%	75.9%
Brazil	Mairi	2000	79.7%	84.1%	75.5%
Brazil	Mairi	2017	80.4%	84.6%	76.2%
Brazil	Mairinque	2000	94.2%	95.3%	93.1%
Brazil	Mairinque	2017	94.4%	95.4%	93.3%
Brazil	Mairiporã	2000	93.9%	94.8%	93.1%
Brazil	Mairiporã	2017	94.2%	95.0%	93.3%
Brazil	Mairipotaba	2000	76.1%	79.8%	72.3%
Brazil	Mairipotaba	2017	76.8%	80.5%	73.1%
Brazil	Major Gercino	2000	74.1%	78.0%	69.3%
Brazil	Major Gercino	2017	74.9%	78.7%	70.1%
Brazil	Major Isidoro	2000	63.2%	66.3%	59.9%
Brazil	Major Isidoro	2017	64.1%	67.2%	60.8%
Brazil	Major Sales	2000	76.5%	80.1%	72.9%
Brazil	Major Sales	2017	77.2%	80.7%	73.7%
Brazil	Major Vieira	2000	77.4%	81.1%	73.4%
Brazil	Major Vieira	2017	78.1%	81.7%	74.2%
Brazil	Malacacheta	2000	82.9%	87.6%	77.2%
Brazil	Malacacheta	2017	83.5%	88.1%	77.9%
Brazil	Malhada	2000	78.7%	84.5%	71.5%
Brazil	Malhada	2017	79.3%	85.0%	72.3%
Brazil	Malhada de Pedras	2000	77.2%	82.4%	71.7%

Country	Second Administrative Unit	Year	Mean	Upper	Lower
Brazil	Malhada de Pedras	2017	77.9%	82.9%	72.5%
Brazil	Malhada dos Bois	2000	72.2%	74.8%	69.1%
Brazil	Malhada dos Bois	2017	73.0%	75.5%	69.9%
Brazil	Malhador	2000	75.5%	77.6%	73.0%
Brazil	Malhador	2017	76.2%	78.2%	73.7%
Brazil	Mallet	2000	80.2%	84.5%	75.3%
Brazil	Mallet	2017	80.8%	85.0%	76.0%
Brazil	Malta	2000	70.9%	74.8%	66.7%
Brazil	Malta	2017	71.7%	75.6%	67.7%
Brazil	Mamanguape	2000	71.8%	74.7%	69.1%
Brazil	Mamanguape	2017	71.7%	74.6%	68.9%
Brazil	Mambaí	2000	81.8%	86.9%	75.8%
Brazil	Mambaí	2017	82.4%	87.3%	76.5%
Brazil	Mamborê	2000	84.4%	87.7%	80.3%
Brazil	Mamborê	2017	84.9%	88.2%	80.9%
Brazil	Mamonas	2000	79.6%	85.1%	73.2%
Brazil	Mamonas	2017	80.2%	85.5%	73.9%
Brazil	Mampituba	2000	78.4%	82.6%	73.7%
Brazil	Mampituba	2017	79.1%	83.1%	74.4%
Brazil	Manacapuru	2000	58.2%	62.3%	53.7%
Brazil	Manacapuru	2017	59.2%	63.2%	54.7%
Brazil	Manaíra	2000	67.6%	71.7%	63.2%
Brazil	Manaíra	2017	68.5%	72.4%	64.1%
Brazil	Manaquiri	2000	56.3%	60.2%	52.2%
Brazil	Manaquiri	2017	57.2%	61.2%	53.1%
Brazil	Manari	2000	63.2%	66.9%	59.0%
Brazil	Manari	2017	64.2%	67.8%	60.0%
Brazil	Mâncio Lima	2000	44.0%	48.3%	39.7%
Brazil	Mâncio Lima	2017	45.0%	49.4%	40.6%
Brazil	Mandaguaçu	2000	85.3%	87.7%	82.1%
Brazil	Mandaguaçu	2017	85.7%	88.1%	82.7%
Brazil	Mandaguari	2000	86.1%	88.4%	83.1%
Brazil	Mandaguari	2017	86.6%	88.8%	83.6%
Brazil	Mandirituba	2000	82.7%	85.0%	79.9%
Brazil	Mandirituba	2017	83.3%	85.5%	80.5%
Brazil	Manduri	2000	93.0%	94.9%	90.7%
Brazil	Manduri	2017	93.3%	95.1%	91.1%
Brazil	Manfrinópolis	2000	81.6%	85.7%	76.4%
Brazil	Manfrinópolis	2017	82.2%	86.2%	77.2%
Brazil	Manga	2000	80.2%	86.4%	73.5%
Brazil	Manga	2017	80.8%	86.8%	74.2%
Brazil	Mangaratiba	2000	77.2%	80.6%	74.0%
Brazil	Mangaratiba	2000	81.4%	83.9%	78.6%
Brazil	Mangaratiba	2017	81.9%	84.4%	79.2%
Brazil	Mangaratiba	2017	78.0%	81.3%	74.9%
Brazil	Mangueirinha	2000	81.3%	85.4%	76.7%
Brazil	Mangueirinha	2017	81.9%	85.9%	77.4%
Brazil	Manhuaçu	2000	82.2%	85.4%	78.2%
Brazil	Manhuaçu	2017	83.5%	86.5%	79.8%
Brazil	Manhumirim	2000	80.6%	83.7%	76.7%
Brazil	Manhumirim	2017	81.2%	84.2%	77.3%
Brazil	Manicore	2000	58.5%	64.7%	50.2%
Brazil	Manicore	2017	59.4%	65.5%	51.2%
Brazil	Manoel Emídio	2000	60.3%	67.3%	52.3%
Brazil	Manoel Emídio	2017	61.2%	68.1%	53.3%
Brazil	Manoel Ribas	2000	84.1%	87.7%	80.1%
Brazil	Manoel Ribas	2017	84.7%	88.2%	80.7%

Country	Second Administrative Unit	Year	Mean	Upper	Lower
Brazil	Manoel Urbano	2000	44.4%	50.2%	39.0%
Brazil	Manoel Urbano	2017	47.1%	53.0%	41.8%
Brazil	Manoel Viana	2000	82.2%	86.4%	76.3%
Brazil	Manoel Viana	2017	82.7%	86.9%	77.0%
Brazil	Manoel Vitorino	2000	78.6%	83.3%	73.7%
Brazil	Manoel Vitorino	2017	79.3%	83.9%	74.5%
Brazil	Mansidão	2000	69.1%	76.2%	61.5%
Brazil	Mansidão	2017	70.0%	77.0%	62.4%
Brazil	Mantena	2000	80.1%	83.4%	76.1%
Brazil	Mantena	2017	80.7%	83.9%	76.8%
Brazil	Mantenópolis	2000	80.7%	84.5%	76.7%
Brazil	Mantenópolis	2017	81.3%	85.0%	77.4%
Brazil	Maquiné	2000	78.6%	82.6%	74.0%
Brazil	Maquiné	2017	79.1%	83.1%	74.5%
Brazil	Mar de Espanha	2000	79.7%	82.9%	76.3%
Brazil	Mar de Espanha	2017	80.4%	83.5%	77.0%
Brazil	Mar Vermelho	2000	59.6%	62.7%	56.8%
Brazil	Mar Vermelho	2017	60.5%	63.6%	57.7%
Brazil	Mara Rosa	2000	77.2%	82.5%	71.6%
Brazil	Mara Rosa	2017	77.8%	83.0%	72.4%
Brazil	Maraã	2000	57.8%	64.8%	50.0%
Brazil	Maraã	2017	58.8%	65.7%	51.0%
Brazil	Marabá	2000	43.4%	48.6%	39.3%
Brazil	Marabá	2017	44.6%	49.8%	40.4%
Brazil	Marabá Paulista	2000	86.4%	89.7%	82.7%
Brazil	Marabá Paulista	2017	86.8%	90.1%	83.3%
Brazil	Maracaçumé	2000	49.2%	55.9%	43.9%
Brazil	Maracaçumé	2017	50.2%	56.9%	45.0%
Brazil	Maracaí	2000	89.5%	92.0%	87.0%
Brazil	Maracaí	2017	89.9%	92.3%	87.5%
Brazil	Maracajá	2000	76.8%	81.0%	72.3%
Brazil	Maracajá	2017	77.5%	81.6%	73.1%
Brazil	Maracaju	2000	80.8%	84.2%	77.4%
Brazil	Maracaju	2017	81.4%	84.7%	78.1%
Brazil	Maracanã	2000	35.5%	40.4%	30.7%
Brazil	Maracanã	2017	36.4%	41.3%	31.5%
Brazil	Maracanaú	2000	69.4%	71.5%	67.4%
Brazil	Maracanaú	2017	70.2%	72.3%	68.3%
Brazil	Maracás	2000	79.9%	84.4%	74.5%
Brazil	Maracás	2017	80.6%	84.9%	75.2%
Brazil	Maragogi	2000	63.2%	67.3%	57.9%
Brazil	Maragogi	2017	63.9%	68.0%	58.7%
Brazil	Maragogipe	2000	74.7%	78.4%	70.8%
Brazil	Maragogipe	2017	75.7%	79.2%	71.8%
Brazil	Maraial	2000	64.5%	67.4%	61.8%
Brazil	Maraial	2017	65.2%	68.1%	62.5%
Brazil	Marajá do Sena	2000	61.0%	65.7%	56.1%
Brazil	Marajá do Sena	2017	61.9%	66.6%	57.1%
Brazil	Maranguape	2000	69.1%	71.4%	66.7%
Brazil	Maranguape	2017	70.2%	72.5%	67.8%
Brazil	Maranhãozinho	2000	52.6%	58.1%	47.4%
Brazil	Maranhãozinho	2017	53.8%	59.4%	48.6%

Country	Second Administrative Unit	Year	Mean	Upper	Lower
Brazil	Marapanim	2000	35.1%	39.6%	30.6%
Brazil	Marapanim	2017	36.0%	40.6%	31.4%
Brazil	Marapoama	2000	93.0%	94.8%	90.7%
Brazil	Marapoama	2017	93.2%	95.0%	91.0%
Brazil	Maratá	2000	81.1%	83.9%	78.2%
Brazil	Maratá	2017	81.7%	84.4%	78.9%
Brazil	Marataízes	2000	74.9%	78.4%	70.8%
Brazil	Marataízes	2017	75.7%	79.1%	71.6%
Brazil	Maraú	2000	81.5%	84.9%	77.1%
Brazil	Maraú	2000	70.8%	76.7%	65.4%
Brazil	Maraú	2017	82.1%	85.4%	77.8%
Brazil	Maraú	2017	71.6%	77.3%	66.2%
Brazil	Maravilha	2000	78.8%	82.8%	74.7%
Brazil	Maravilha	2000	61.6%	64.9%	58.1%
Brazil	Maravilha	2017	81.0%	84.7%	77.1%
Brazil	Maravilha	2017	62.5%	65.8%	59.0%
Brazil	Maravilhas	2000	80.8%	85.1%	75.7%
Brazil	Maravilhas	2017	81.4%	85.6%	76.4%
Brazil	Marcação	2000	69.1%	72.5%	65.9%
Brazil	Marcação	2017	69.9%	73.2%	66.8%
Brazil	Marcelândia	2000	66.7%	73.4%	58.6%
Brazil	Marcelândia	2017	67.5%	74.0%	59.5%
Brazil	Marcelino Vieira	2000	76.0%	79.4%	72.6%
Brazil	Marcelino Vieira	2017	76.7%	80.0%	73.4%
Brazil	Marcionilio Dias	2000	75.5%	79.5%	70.8%
Brazil	Marcionilio Dias	2017	76.2%	80.1%	71.6%
Brazil	Marcionílio Souza	2000	75.7%	81.3%	69.8%
Brazil	Marcionílio Souza	2017	76.4%	81.9%	70.6%
Brazil	Marco	2000	68.4%	72.7%	63.1%
Brazil	Marco	2017	69.2%	73.5%	64.0%
Brazil	Marcolândia	2000	64.9%	69.3%	60.1%
Brazil	Marcolândia	2017	65.8%	70.1%	61.0%
Brazil	Marcos Parente	2000	62.2%	68.5%	55.4%
Brazil	Marcos Parente	2017	63.1%	69.4%	56.3%
Brazil	Marechal Cândido Rondon	2000	84.4%	87.8%	80.8%
Brazil	Marechal Cândido Rondon	2017	84.9%	88.3%	81.4%
Brazil	Marechal deodoro	2000	60.3%	63.1%	57.5%
Brazil	Marechal deodoro	2017	61.2%	64.0%	58.4%
Brazil	Marechal Floriano	2000	81.3%	83.6%	78.9%
Brazil	Marechal Floriano	2017	81.9%	84.2%	79.6%
Brazil	Marechal Thaumaturgo	2000	43.6%	50.1%	37.3%
Brazil	Marechal Thaumaturgo	2017	44.3%	50.8%	38.0%
Brazil	Marema	2000	75.9%	80.6%	71.0%
Brazil	Marema	2017	76.7%	81.3%	71.8%
Brazil	Mari	2000	67.6%	71.2%	64.3%
Brazil	Mari	2017	68.4%	71.9%	65.2%

Country	Second Administrative Unit	Year	Mean	Upper	Lower
Brazil	Maria da Fé	2000	89.6%	92.1%	86.8%
Brazil	Maria da Fé	2017	90.0%	92.4%	87.3%
Brazil	Maria Helena	2000	83.3%	86.4%	79.7%
Brazil	Maria Helena	2017	83.8%	86.8%	80.3%
Brazil	Marialva	2000	85.8%	88.1%	82.7%
Brazil	Marialva	2017	86.3%	88.4%	83.3%
Brazil	Mariana	2000	80.7%	84.4%	77.1%
Brazil	Mariana	2017	81.3%	84.9%	77.8%
Brazil	Mariana Pimentel	2000	82.3%	85.5%	79.0%
Brazil	Mariana Pimentel	2017	82.8%	86.0%	79.6%
Brazil	Mariano Moro	2000	76.6%	80.1%	72.5%
Brazil	Mariano Moro	2017	77.2%	80.7%	73.2%
Brazil	Marianópolis do Tocantins	2000	66.7%	72.8%	59.9%
Brazil	Marianópolis do Tocantins	2017	67.5%	73.6%	60.8%
Brazil	Mariápolis	2000	89.5%	92.2%	86.2%
Brazil	Mariápolis	2017	89.8%	92.5%	86.7%
Brazil	Maribondo	2000	58.2%	61.7%	55.4%
Brazil	Maribondo	2017	59.2%	62.6%	56.3%
Brazil	Maricá	2000	77.5%	79.7%	74.7%
Brazil	Maricá	2017	78.4%	80.6%	75.7%
Brazil	Marilac	2000	80.0%	85.1%	74.6%
Brazil	Marilac	2017	80.6%	85.6%	75.4%
Brazil	Marilândia	2000	79.0%	81.5%	75.8%
Brazil	Marilândia	2017	79.6%	82.1%	76.5%
Brazil	Marilândia do Sul	2000	84.6%	87.5%	81.5%
Brazil	Marilândia do Sul	2017	85.1%	87.9%	82.1%
Brazil	Marilena	2000	83.6%	87.2%	79.6%
Brazil	Marilena	2017	84.1%	87.6%	80.2%
Brazil	Marília	2000	93.0%	94.8%	90.3%
Brazil	Marília	2017	93.2%	95.0%	90.6%
Brazil	Mariluz	2000	85.0%	88.5%	81.6%
Brazil	Mariluz	2017	85.5%	88.9%	82.2%
Brazil	Maringá	2000	85.5%	88.0%	82.5%
Brazil	Maringá	2017	86.1%	88.5%	83.2%
Brazil	Marinópolis	2000	88.0%	91.3%	84.1%
Brazil	Marinópolis	2017	88.4%	91.6%	84.6%
Brazil	Mário Campos	2000	82.4%	85.0%	79.6%
Brazil	Mário Campos	2017	83.0%	85.5%	80.2%
Brazil	Mariópolis	2000	80.5%	84.6%	76.3%
Brazil	Mariópolis	2017	81.1%	85.1%	77.0%
Brazil	Maripá	2000	84.0%	87.3%	80.4%
Brazil	Maripá	2017	84.5%	87.8%	81.0%
Brazil	Maripá de Minas	2000	80.4%	83.6%	76.9%
Brazil	Maripá de Minas	2017	81.0%	84.1%	77.6%
Brazil	Marituba	2000	36.7%	39.2%	34.3%
Brazil	Marituba	2017	37.5%	40.0%	35.1%
Brazil	Marizópolis	2000	70.3%	74.1%	66.6%
Brazil	Marizópolis	2017	71.1%	74.9%	67.4%
Brazil	Marliéria	2000	81.0%	84.8%	77.0%
Brazil	Marliéria	2017	81.0%	84.8%	77.0%
Brazil	Marmeleiro	2000	81.6%	85.9%	76.8%
Brazil	Marmeleiro	2017	82.2%	86.4%	77.5%

Country	Second Administrative Unit	Year	Mean	Upper	Lower
Brazil	Marmelópolis	2000	89.8%	92.0%	87.5%
Brazil	Marmelópolis	2017	90.1%	92.3%	87.9%
Brazil	Marques de Souza	2000	81.4%	85.0%	77.8%
Brazil	Marques de Souza	2017	82.0%	85.5%	78.4%
Brazil	Marquinho	2000	82.9%	86.6%	78.5%
Brazil	Marquinho	2017	83.5%	87.1%	79.2%
Brazil	Martinho Campos	2000	79.9%	84.8%	73.7%
Brazil	Martinho Campos	2017	80.5%	85.3%	74.5%
Brazil	Martinópole	2000	69.7%	74.2%	64.5%
Brazil	Martinópole	2017	70.6%	75.0%	65.5%
Brazil	Martinópolis	2000	90.0%	92.7%	87.2%
Brazil	Martinópolis	2017	90.3%	93.0%	87.6%
Brazil	Martins	2000	79.0%	82.0%	75.7%
Brazil	Martins	2017	79.6%	82.5%	76.5%
Brazil	Martins Soares	2000	81.3%	84.8%	77.7%
Brazil	Martins Soares	2017	81.9%	85.3%	78.4%
Brazil	Maruim	2000	73.6%	75.7%	70.9%
Brazil	Maruim	2017	74.4%	76.4%	71.7%
Brazil	Marumbi	2000	84.3%	87.1%	81.0%
Brazil	Marumbi	2017	84.8%	87.5%	81.6%
Brazil	Marzagão	2000	77.4%	81.4%	72.9%
Brazil	Marzagão	2017	78.1%	82.0%	73.7%
Brazil	Mascote	2000	77.8%	82.9%	71.9%
Brazil	Mascote	2017	78.4%	83.3%	72.6%
Brazil	Massapê	2000	68.6%	72.3%	64.7%
Brazil	Massapê	2017	69.4%	73.1%	65.6%
Brazil	Massapê do Piauí	2000	61.1%	65.8%	56.1%
Brazil	Massapê do Piauí	2017	61.9%	66.7%	57.1%
Brazil	Massaranduba	2000	66.3%	69.2%	63.0%
Brazil	Massaranduba	2000	76.1%	79.2%	72.6%
Brazil	Massaranduba	2017	67.1%	70.0%	63.9%
Brazil	Massaranduba	2017	76.8%	79.9%	73.4%
Brazil	Mata	2000	81.6%	86.0%	76.3%
Brazil	Mata	2017	82.2%	86.5%	77.0%
Brazil	Mata de São João	2000	76.4%	79.7%	72.9%
Brazil	Mata de São João	2017	77.1%	80.3%	73.6%
Brazil	Mata Grande	2000	63.5%	67.1%	59.2%
Brazil	Mata Grande	2017	64.5%	68.0%	60.1%
Brazil	Mata Roma	2000	61.6%	66.2%	56.7%
Brazil	Mata Roma	2017	62.5%	67.1%	57.6%
Brazil	Mata Verde	2000	81.8%	86.2%	76.4%
Brazil	Mata Verde	2017	82.4%	86.7%	77.2%
Brazil	Matão	2000	94.1%	95.4%	92.3%
Brazil	Matão	2017	94.3%	95.6%	92.5%
Brazil	Mataraca	2000	73.1%	76.4%	69.9%
Brazil	Mataraca	2017	75.6%	78.7%	72.6%
Brazil	Mateira	2000	80.5%	84.7%	75.6%
Brazil	Mateira	2017	81.1%	85.2%	76.3%
Brazil	Mateiros	2000	72.4%	79.6%	64.0%
Brazil	Mateiros	2017	73.1%	80.3%	64.9%
Brazil	Matelândia	2000	84.0%	87.4%	80.7%
Brazil	Matelândia	2017	84.5%	87.9%	81.3%

Country	Second Administrative Unit	Year	Mean	Upper	Lower
Brazil	Materlândia	2000	81.9%	86.6%	75.9%
Brazil	Materlândia	2017	82.5%	87.0%	76.6%
Brazil	Mates do Norte	2000	61.0%	65.8%	55.8%
Brazil	Mates do Norte	2017	61.9%	66.7%	56.8%
Brazil	Mateus Leme	2000	81.0%	84.2%	77.2%
Brazil	Mateus Leme	2017	81.6%	84.7%	77.9%
Brazil	Mathias Lobato	2000	79.2%	84.4%	74.2%
Brazil	Mathias Lobato	2017	79.9%	84.9%	74.9%
Brazil	Matias Barbosa	2000	81.0%	84.1%	77.7%
Brazil	Matias Barbosa	2017	81.6%	84.6%	78.4%
Brazil	Matias Cardoso	2000	80.1%	86.2%	73.1%
Brazil	Matias Cardoso	2017	80.6%	86.5%	73.7%
Brazil	Matias Olímpio	2000	60.7%	65.3%	56.0%
Brazil	Matias Olímpio	2017	61.7%	66.2%	57.0%
Brazil	Matina	2000	77.6%	83.1%	71.5%
Brazil	Matina	2017	78.3%	83.6%	72.3%
Brazil	Matinha	2000	60.9%	65.6%	56.1%
Brazil	Matinha	2017	61.8%	66.5%	57.1%
Brazil	Matinhas	2000	66.1%	69.0%	62.9%
Brazil	Matinhas	2017	66.9%	69.8%	63.7%
Brazil	Matinhos	2000	76.7%	81.0%	71.9%
Brazil	Matinhos	2017	77.4%	81.6%	72.7%
Brazil	Matipó	2000	81.6%	85.0%	77.4%
Brazil	Matipó	2017	82.2%	85.5%	78.1%
Brazil	Mato Castelhano	2000	81.1%	84.4%	76.4%
Brazil	Mato Castelhano	2017	81.7%	84.9%	77.1%
Brazil	Mato Grosso	2000	75.4%	79.0%	72.0%
Brazil	Mato Grosso	2000	59.0%	68.0%	49.0%
Brazil	Mato Grosso	2017	60.6%	69.5%	50.8%
Brazil	Mato Grosso	2017	76.2%	79.6%	72.8%
Brazil	Mato Leitão	2000	80.3%	83.8%	75.9%
Brazil	Mato Leitão	2017	80.9%	84.4%	76.6%
Brazil	Mato Rico	2000	83.9%	87.1%	79.9%
Brazil	Mato Rico	2017	84.5%	87.6%	80.6%
Brazil	Mato Verde	2000	80.3%	86.1%	74.4%
Brazil	Mato Verde	2017	81.0%	86.6%	75.2%
Brazil	Matões	2000	60.2%	64.7%	55.5%
Brazil	Matões	2017	61.1%	65.6%	56.5%
Brazil	Matos Costa	2000	80.5%	84.3%	76.5%
Brazil	Matos Costa	2017	81.0%	84.8%	77.1%
Brazil	Matozinhos	2000	81.1%	84.3%	77.7%
Brazil	Matozinhos	2017	81.7%	84.9%	78.4%
Brazil	Matrinchã	2000	73.8%	79.3%	67.2%
Brazil	Matrinchã	2017	74.6%	80.0%	68.1%
Brazil	Matriz de Camaragibe	2000	61.2%	64.0%	57.9%
Brazil	Matriz de Camaragibe	2017	62.1%	64.9%	58.9%
Brazil	Matupá	2000	65.3%	71.9%	57.6%
Brazil	Matupá	2017	66.2%	72.8%	58.6%

Country	Second Administrative Unit	Year	Mean	Upper	Lower
Brazil	Maturéia	2000	74.1%	78.0%	69.8%
Brazil	Maturéia	2017	74.9%	78.7%	70.7%
Brazil	Matutina	2000	81.0%	86.2%	74.3%
Brazil	Matutina	2017	81.6%	86.6%	75.0%
Brazil	Mauá	2000	94.7%	95.5%	94.0%
Brazil	Mauá	2017	94.9%	95.6%	94.2%
Brazil	Mauá da Serra	2000	85.5%	88.5%	82.1%
Brazil	Mauá da Serra	2017	86.0%	88.9%	82.7%
Brazil	Maués	2000	58.1%	59.7%	56.3%
Brazil	Maués	2017	59.1%	60.6%	57.3%
Brazil	Maurilândia	2000	78.1%	82.4%	73.3%
Brazil	Maurilândia	2017	78.8%	83.0%	74.0%
Brazil	Maurilândia do Tocantins	2000	69.3%	73.9%	64.2%
Brazil	Maurilândia do Tocantins	2017	70.2%	74.7%	65.3%
Brazil	Maxaranguape	2000	82.6%	85.3%	79.4%
Brazil	Maxaranguape	2017	83.2%	85.8%	80.0%
Brazil	Maximiliano de Almaeida	2000	77.2%	81.0%	72.9%
Brazil	Maximiliano de Almaeida	2017	77.9%	81.6%	73.7%
Brazil	Mazagão	2000	43.9%	47.0%	40.7%
Brazil	Mazagão	2017	44.8%	47.9%	41.6%
Brazil	Me do Rio	2000	39.3%	44.4%	34.8%
Brazil	Me do Rio	2017	40.3%	45.4%	35.7%
Brazil	Medeiros	2000	83.2%	87.7%	77.7%
Brazil	Medeiros	2017	83.7%	88.1%	78.4%
Brazil	Medeiros Neto	2000	79.3%	83.8%	73.7%
Brazil	Medeiros Neto	2017	79.9%	84.3%	74.5%
Brazil	Medianeira	2000	84.5%	87.8%	81.4%
Brazil	Medianeira	2017	85.1%	88.2%	82.0%
Brazil	Medicilândia	2000	41.5%	51.1%	32.6%
Brazil	Medicilândia	2017	42.5%	52.0%	33.4%
Brazil	Medina	2000	82.6%	87.0%	76.9%
Brazil	Medina	2017	83.2%	87.5%	77.6%
Brazil	Meleiro	2000	79.7%	83.5%	75.8%
Brazil	Meleiro	2017	80.3%	84.0%	76.5%
Brazil	Melgaco	2000	38.8%	45.7%	33.6%
Brazil	Melgaco	2017	39.7%	46.7%	34.4%
Brazil	Melgaço	2000	40.6%	47.7%	34.5%
Brazil	Melgaço	2017	41.6%	48.6%	35.4%
Brazil	Mendes	2000	82.2%	84.3%	79.6%
Brazil	Mendes	2017	82.8%	84.8%	80.2%
Brazil	Mendes Pimentel	2000	79.5%	84.8%	75.2%
Brazil	Mendes Pimentel	2017	80.1%	85.3%	75.9%
Brazil	Mendonça	2000	92.9%	94.7%	90.8%
Brazil	Mendonça	2017	93.1%	94.9%	91.1%
Brazil	Mercedes	2000	82.6%	86.4%	78.8%
Brazil	Mercedes	2017	83.2%	86.8%	79.4%
Brazil	Mercês	2000	80.5%	84.0%	76.4%
Brazil	Mercês	2017	81.2%	84.5%	77.1%
Brazil	Meridiano	2000	90.2%	93.4%	87.1%
Brazil	Meridiano	2017	90.5%	93.6%	87.6%
Brazil	Meruoca	2000	71.5%	75.1%	67.9%
Brazil	Meruoca	2017	72.3%	75.7%	68.7%
Brazil	Mesópolis	2000	86.7%	90.6%	82.8%
Brazil	Mesópolis	2017	87.2%	90.9%	83.3%
Brazil	Mesquita	2000	80.2%	84.5%	75.3%
Brazil	Mesquita	2017	80.8%	85.0%	75.9%

Country	Second Administrative Unit	Year	Mean	Upper	Lower
Brazil	Messias	2000	62.2%	64.7%	59.5%
Brazil	Messias	2017	63.1%	65.6%	60.4%
Brazil	Miguel Alves	2000	60.6%	64.8%	56.4%
Brazil	Miguel Alves	2017	61.6%	65.6%	57.3%
Brazil	Miguel Calmon	2000	80.5%	84.7%	75.8%
Brazil	Miguel Calmon	2017	81.1%	85.1%	76.5%
Brazil	Miguel Leão	2000	59.9%	65.0%	55.0%
Brazil	Miguel Leão	2017	60.8%	65.9%	56.0%
Brazil	Miguel Pereira	2000	81.7%	83.9%	79.2%
Brazil	Miguel Pereira	2017	82.2%	84.3%	79.8%
Brazil	Miguelópolis	2000	88.7%	91.7%	85.3%
Brazil	Miguelópolis	2017	89.0%	92.0%	85.8%
Brazil	Milagres	2000	66.9%	70.4%	63.4%
Brazil	Milagres	2000	81.4%	85.3%	76.8%
Brazil	Milagres	2017	67.8%	71.2%	64.3%
Brazil	Milagres	2017	82.0%	85.8%	77.5%
Brazil	Milagres do Maranhão	2000	59.9%	64.6%	54.6%
Brazil	Milagres do Maranhão	2017	60.8%	65.5%	55.6%
Brazil	Milhã	2000	70.3%	74.9%	65.7%
Brazil	Milhã	2017	71.1%	75.6%	66.6%
Brazil	Milton Brandão	2000	65.0%	69.3%	60.1%
Brazil	Milton Brandão	2017	65.8%	70.1%	61.0%
Brazil	Mimoso de Goiás	2000	87.7%	90.6%	84.4%
Brazil	Mimoso de Goiás	2017	88.1%	90.9%	84.9%
Brazil	Mimoso do Sul	2000	77.6%	80.9%	74.0%
Brazil	Mimoso do Sul	2017	78.2%	81.5%	74.7%
Brazil	Minaçu	2000	77.6%	83.5%	71.6%
Brazil	Minaçu	2017	78.3%	84.0%	72.4%
Brazil	Minador do Negrão	2000	63.1%	66.6%	59.3%
Brazil	Minador do Negrão	2017	64.0%	67.4%	60.3%
Brazil	Minas do Leão	2000	81.6%	85.4%	77.7%
Brazil	Minas do Leão	2017	82.2%	85.8%	78.4%
Brazil	Minas Novas	2000	83.1%	87.7%	77.8%
Brazil	Minas Novas	2017	83.6%	88.1%	78.4%
Brazil	Minduri	2000	83.9%	87.2%	80.0%
Brazil	Minduri	2017	84.4%	87.6%	80.6%
Brazil	Mineiros	2000	77.2%	83.0%	71.0%
Brazil	Mineiros	2017	77.9%	83.6%	71.8%
Brazil	Mineiros do Tietê	2000	93.5%	95.2%	91.6%
Brazil	Mineiros do Tietê	2017	93.8%	95.4%	91.9%
Brazil	Ministro Andreazza	2000	30.8%	34.4%	27.9%
Brazil	Ministro Andreazza	2017	31.6%	35.3%	28.6%
Brazil	Mira Estrela	2000	87.8%	91.2%	84.3%
Brazil	Mira Estrela	2017	88.2%	91.5%	84.8%

Country	Second Administrative Unit	Year	Mean	Upper	Lower
Brazil	Mirabela	2000	81.9%	86.9%	76.1%
Brazil	Mirabela	2017	82.5%	87.3%	76.8%
Brazil	Miracatu	2000	90.7%	93.0%	87.9%
Brazil	Miracatu	2017	91.0%	93.3%	88.3%
Brazil	Miracema	2000	77.8%	81.4%	73.4%
Brazil	Miracema	2017	78.4%	82.0%	74.2%
Brazil	Miracema do Tocantins	2000	74.0%	78.3%	70.0%
Brazil	Miracema do Tocantins	2017	74.7%	79.0%	70.8%
Brazil	Mirador	2000	60.2%	66.4%	53.2%
Brazil	Mirador	2000	83.3%	86.6%	79.8%
Brazil	Mirador	2017	61.2%	67.3%	54.3%
Brazil	Mirador	2017	83.9%	87.0%	80.4%
Brazil	Miradouro	2000	81.3%	84.8%	77.5%
Brazil	Miradouro	2017	81.9%	85.3%	78.2%
Brazil	Miraguaí	2000	80.4%	84.4%	76.0%
Brazil	Miraguaí	2017	81.1%	85.0%	76.7%
Brazil	Miraí	2000	81.0%	84.3%	77.4%
Brazil	Miraí	2017	81.6%	84.8%	78.1%
Brazil	Miraíma	2000	69.4%	73.1%	64.8%
Brazil	Miraíma	2017	70.2%	73.7%	65.5%
Brazil	Miranda	2000	79.7%	84.2%	73.5%
Brazil	Miranda	2017	80.0%	84.6%	74.0%
Brazil	Miranda do Norte	2000	62.2%	66.9%	58.2%
Brazil	Miranda do Norte	2017	63.1%	67.8%	59.1%
Brazil	Mirandiba	2000	66.7%	71.8%	61.6%
Brazil	Mirandiba	2017	67.5%	72.5%	62.5%
Brazil	Mirandópolis	2000	90.4%	92.6%	87.8%
Brazil	Mirandópolis	2017	90.7%	92.9%	88.2%
Brazil	Mirangaba	2000	78.0%	82.8%	72.7%
Brazil	Mirangaba	2017	78.7%	83.3%	73.5%
Brazil	Miranorte	2000	73.9%	78.7%	69.7%
Brazil	Miranorte	2017	74.6%	79.3%	70.4%
Brazil	Mirante	2000	77.6%	83.4%	71.8%
Brazil	Mirante	2017	78.3%	83.9%	72.6%
Brazil	Mirante da Serra	2000	29.7%	33.6%	26.0%
Brazil	Mirante da Serra	2017	30.6%	34.5%	26.8%
Brazil	Mirante do Paranapanema	2000	86.5%	89.7%	83.2%
Brazil	Mirante do Paranapanema	2017	87.0%	90.0%	83.7%
Brazil	Miraselva	2000	86.7%	89.3%	83.8%
Brazil	Miraselva	2017	87.1%	89.7%	84.3%
Brazil	Mirassol	2000	92.3%	94.0%	90.1%
Brazil	Mirassol	2017	92.6%	94.2%	90.4%
Brazil	Mirassol d'Oeste	2000	71.0%	76.5%	63.7%
Brazil	Mirassol d'Oeste	2017	71.8%	77.3%	64.6%
Brazil	Mirassolândia	2000	91.4%	93.5%	88.8%
Brazil	Mirassolândia	2017	91.7%	93.7%	89.2%
Brazil	Miravânia	2000	79.6%	85.6%	72.0%
Brazil	Miravânia	2017	80.2%	86.1%	72.8%
Brazil	Mirim doce	2000	73.7%	78.2%	68.5%
Brazil	Mirim doce	2017	74.5%	78.9%	69.3%

Country	Second Administrative Unit	Year	Mean	Upper	Lower
Brazil	Mirinzal	2000	59.7%	65.8%	52.6%
Brazil	Mirinzal	2017	60.6%	66.7%	53.6%
Brazil	Missal	2000	82.8%	86.9%	78.9%
Brazil	Missal	2017	83.3%	87.4%	79.6%
Brazil	Misso Velha	2000	68.1%	71.3%	65.0%
Brazil	Misso Velha	2017	68.9%	72.1%	65.9%
Brazil	Mocajuba	2000	38.2%	44.4%	32.9%
Brazil	Mocajuba	2017	39.1%	45.4%	33.7%
Brazil	Mococa	2000	89.7%	92.0%	86.8%
Brazil	Mococa	2017	90.1%	92.2%	87.2%
Brazil	Modelo	2000	78.4%	82.3%	73.9%
Brazil	Modelo	2017	79.0%	82.9%	74.6%
Brazil	Moeda	2000	81.3%	84.4%	78.2%
Brazil	Moeda	2017	81.9%	84.9%	78.9%
Brazil	Moema	2000	81.0%	85.1%	76.0%
Brazil	Moema	2017	81.6%	85.6%	76.7%
Brazil	Mogi das Cruzes	2000	94.4%	95.2%	93.3%
Brazil	Mogi das Cruzes	2017	94.6%	95.4%	93.6%
Brazil	Mogi Guaçu	2000	92.1%	93.7%	90.1%
Brazil	Mogi Guaçu	2017	92.4%	93.9%	90.5%
Brazil	Mogi Mirim	2000	92.6%	94.1%	91.0%
Brazil	Mogi Mirim	2017	92.9%	94.3%	91.3%
Brazil	Moiporá	2000	76.2%	81.0%	71.2%
Brazil	Moiporá	2017	76.9%	81.6%	72.0%
Brazil	Moita Bonita	2000	76.0%	78.3%	73.5%
Brazil	Moita Bonita	2017	76.8%	79.0%	74.2%
Brazil	Moju	2000	38.9%	42.7%	34.9%
Brazil	Moju	2017	39.8%	43.7%	35.8%
Brazil	Mombaça	2000	69.8%	73.9%	65.5%
Brazil	Mombaça	2000	93.1%	94.4%	91.5%
Brazil	Mombaça	2017	70.7%	74.6%	66.4%
Brazil	Mombaça	2017	93.4%	94.6%	91.8%
Brazil	Monção	2000	60.3%	64.7%	55.3%
Brazil	Monção	2017	61.2%	65.6%	56.3%
Brazil	Monções	2000	91.4%	93.9%	88.6%
Brazil	Monções	2017	91.7%	94.1%	89.0%
Brazil	Mondaí	2000	76.9%	81.0%	72.0%
Brazil	Mondaí	2017	77.7%	81.6%	72.8%
Brazil	Mongaguá	2000	89.4%	91.4%	86.9%
Brazil	Mongaguá	2017	89.7%	91.7%	87.3%
Brazil	Mongeiro	2000	67.8%	70.8%	64.9%
Brazil	Mongeiro	2017	67.4%	70.4%	64.4%
Brazil	Monjolos	2000	81.7%	86.6%	75.5%
Brazil	Monjolos	2017	82.3%	87.1%	76.2%
Brazil	Monsenhor Gil	2000	59.0%	63.6%	54.0%
Brazil	Monsenhor Gil	2017	59.9%	64.5%	54.9%
Brazil	Monsenhor Hipólito	2000	63.5%	68.2%	59.8%
Brazil	Monsenhor Hipólito	2017	64.5%	69.1%	60.8%
Brazil	Monsenhor Paulo	2000	84.8%	87.7%	81.1%
Brazil	Monsenhor Paulo	2017	85.3%	88.1%	81.7%
Brazil	Monsenhor Tabosa	2000	71.9%	76.4%	67.5%
Brazil	Monsenhor Tabosa	2017	72.6%	77.1%	68.3%

Country	Second Administrative Unit	Year	Mean	Upper	Lower
Brazil	Montadas	2000	70.8%	73.8%	67.9%
Brazil	Montadas	2017	71.6%	74.6%	68.8%
Brazil	Montalvânia	2000	79.4%	85.5%	71.9%
Brazil	Montalvânia	2017	80.1%	86.0%	72.7%
Brazil	Montanha	2000	79.3%	83.5%	75.3%
Brazil	Montanha	2017	79.9%	84.1%	76.0%
Brazil	Montanhas	2000	75.7%	78.3%	73.3%
Brazil	Montanhas	2017	76.5%	78.9%	74.1%
Brazil	Montauri	2000	81.2%	84.5%	77.4%
Brazil	Montauri	2017	81.9%	85.0%	78.1%
Brazil	Monte Alegre	2000	80.9%	83.0%	78.7%
Brazil	Monte Alegre	2000	40.2%	47.3%	33.3%
Brazil	Monte Alegre	2017	81.8%	83.9%	79.7%
Brazil	Monte Alegre	2017	41.2%	48.2%	34.4%
Brazil	Monte Alegre de Goiás	2000	77.4%	82.5%	71.8%
Brazil	Monte Alegre de Goiás	2017	78.0%	83.1%	72.5%
Brazil	Monte Alegre de Minas	2000	82.0%	86.1%	77.2%
Brazil	Monte Alegre de Minas	2017	82.6%	86.6%	77.8%
Brazil	Monte Alegre de Sergipe	2000	71.8%	74.6%	67.9%
Brazil	Monte Alegre de Sergipe	2017	72.6%	75.4%	68.8%
Brazil	Monte Alegre do Piauí	2000	64.4%	71.3%	57.3%
Brazil	Monte Alegre do Piauí	2017	65.3%	72.0%	58.2%
Brazil	Monte Alegre do Sul	2000	92.0%	93.5%	90.2%
Brazil	Monte Alegre do Sul	2017	92.3%	93.7%	90.6%
Brazil	Monte Alegre dos Campos	2000	79.6%	83.4%	75.5%
Brazil	Monte Alegre dos Campos	2017	80.2%	83.9%	76.2%
Brazil	Monte Alto	2000	93.7%	95.0%	91.8%
Brazil	Monte Alto	2017	93.9%	95.2%	92.1%
Brazil	Monte Aprazível	2000	92.0%	94.1%	89.2%
Brazil	Monte Aprazível	2017	92.3%	94.3%	89.6%
Brazil	Monte Azul	2000	79.9%	85.4%	74.0%
Brazil	Monte Azul	2017	80.5%	85.9%	74.8%
Brazil	Monte Azul Paulista	2000	92.7%	94.4%	90.8%
Brazil	Monte Azul Paulista	2017	93.0%	94.6%	91.1%
Brazil	Monte Belo	2000	85.5%	88.7%	81.7%
Brazil	Monte Belo	2017	86.0%	89.1%	82.3%
Brazil	Monte Belo do Sul	2000	81.9%	85.2%	78.9%
Brazil	Monte Belo do Sul	2017	82.5%	85.6%	79.5%
Brazil	Monte Carlo	2000	78.2%	82.5%	73.9%
Brazil	Monte Carlo	2017	78.8%	83.1%	74.7%
Brazil	Monte Carmelo	2000	81.2%	85.8%	75.8%
Brazil	Monte Carmelo	2017	81.8%	86.2%	76.5%

Country	Second Administrative Unit	Year	Mean	Upper	Lower
Brazil	Monte Castelo	2000	76.9%	80.9%	72.7%
Brazil	Monte Castelo	2000	87.5%	90.1%	83.9%
Brazil	Monte Castelo	2017	77.5%	81.5%	73.5%
Brazil	Monte Castelo	2017	87.9%	90.4%	84.5%
Brazil	Monte das Gameleiras	2000	80.6%	83.1%	77.8%
Brazil	Monte das Gameleiras	2017	81.1%	83.6%	78.4%
Brazil	Monte do Carmo	2000	76.9%	80.7%	72.9%
Brazil	Monte do Carmo	2017	77.5%	81.3%	73.7%
Brazil	Monte Formoso	2000	82.5%	86.9%	77.1%
Brazil	Monte Formoso	2017	83.1%	87.3%	77.8%
Brazil	Monte Horebe	2000	70.3%	74.2%	66.3%
Brazil	Monte Horebe	2017	71.1%	75.0%	67.2%
Brazil	Monte Mor	2000	93.3%	94.4%	92.0%
Brazil	Monte Mor	2017	93.5%	94.6%	92.2%
Brazil	Monte Negro	2000	28.5%	32.4%	24.8%
Brazil	Monte Negro	2017	29.2%	33.2%	25.6%
Brazil	Monte Santo	2000	75.5%	80.3%	69.6%
Brazil	Monte Santo	2017	76.2%	80.9%	70.4%
Brazil	Monte Santo de Minas	2000	89.3%	91.9%	86.0%
Brazil	Monte Santo de Minas	2017	89.6%	92.2%	86.4%
Brazil	Monte Santo do Tocantins	2000	73.4%	78.0%	68.9%
Brazil	Monte Santo do Tocantins	2017	74.1%	78.7%	69.7%
Brazil	Monte Sião	2000	90.5%	92.5%	88.3%
Brazil	Monte Sião	2017	90.8%	92.7%	88.7%
Brazil	Monteiro	2000	66.1%	70.0%	61.8%
Brazil	Monteiro	2017	66.9%	70.8%	62.8%
Brazil	Monteiro Lobato	2000	91.6%	93.0%	89.9%
Brazil	Monteiro Lobato	2017	91.9%	93.3%	90.3%
Brazil	Monteirópolis	2000	62.9%	66.2%	59.8%
Brazil	Monteirópolis	2017	63.8%	67.0%	60.7%
Brazil	Montenegro	2000	80.9%	83.5%	78.2%
Brazil	Montenegro	2017	81.5%	84.0%	78.8%
Brazil	Montes Altos	2000	69.3%	75.1%	63.9%
Brazil	Montes Altos	2017	70.1%	75.8%	64.8%
Brazil	Montes Claros	2000	83.5%	87.7%	78.2%
Brazil	Montes Claros	2017	84.0%	88.1%	78.9%
Brazil	Montes Claros de Goiás	2000	73.5%	79.0%	67.1%
Brazil	Montes Claros de Goiás	2017	74.2%	79.7%	68.0%
Brazil	Montezuma	2000	81.8%	86.9%	75.7%
Brazil	Montezuma	2017	82.4%	87.3%	76.4%
Brazil	Montividiu	2000	77.7%	82.4%	71.9%
Brazil	Montividiu	2017	78.4%	83.0%	72.8%
Brazil	Montividiu do Norte	2000	78.5%	83.1%	73.0%
Brazil	Montividiu do Norte	2017	79.1%	83.6%	73.7%
Brazil	Morada Nova	2000	71.9%	75.7%	67.8%
Brazil	Morada Nova	2017	72.7%	76.4%	68.6%

Country	Second Administrative Unit	Year	Mean	Upper	Lower
Brazil	Morada Nova de Minas	2000	80.4%	85.8%	72.6%
Brazil	Morada Nova de Minas	2017	81.0%	86.3%	73.4%
Brazil	Moraújo	2000	66.3%	70.4%	62.0%
Brazil	Moraújo	2017	67.2%	71.3%	62.9%
Brazil	Moreira Sales	2000	84.1%	87.7%	80.1%
Brazil	Moreira Sales	2017	84.6%	88.1%	80.7%
Brazil	Moreno	2000	65.7%	68.0%	63.3%
Brazil	Moreno	2017	66.5%	68.8%	64.2%
Brazil	Mormaço	2000	81.7%	85.3%	77.3%
Brazil	Mormaço	2017	82.3%	85.8%	77.9%
Brazil	Morpará	2000	73.3%	80.4%	64.7%
Brazil	Morpará	2017	74.1%	81.0%	65.6%
Brazil	Morretes	2000	77.7%	81.1%	73.2%
Brazil	Morretes	2017	78.4%	81.7%	74.0%
Brazil	Morrinhos	2000	76.7%	80.4%	72.3%
Brazil	Morrinhos	2000	68.3%	72.4%	63.5%
Brazil	Morrinhos	2017	77.4%	81.0%	73.0%
Brazil	Morrinhos	2017	69.2%	73.2%	64.4%
Brazil	Morrinhos do Sul	2000	77.4%	81.7%	72.5%
Brazil	Morrinhos do Sul	2017	78.0%	82.3%	73.2%
Brazil	Morro Agudo	2000	90.8%	93.2%	88.2%
Brazil	Morro Agudo	2017	91.1%	93.5%	88.6%
Brazil	Morro Agudo de Goiás	2000	77.1%	81.7%	72.2%
Brazil	Morro Agudo de Goiás	2017	77.8%	82.2%	73.0%
Brazil	Morro Cabeça No Tempo	2000	65.9%	72.4%	58.6%
Brazil	Morro Cabeça No Tempo	2017	66.7%	73.2%	59.6%
Brazil	Morro da Fumaça	2000	72.7%	76.9%	68.0%
Brazil	Morro da Fumaça	2017	73.5%	77.6%	68.8%
Brazil	Morro da Garça	2000	81.1%	86.1%	74.8%
Brazil	Morro da Garça	2017	81.7%	86.6%	75.5%
Brazil	Morro do Chapéu	2000	79.6%	84.5%	75.1%
Brazil	Morro do Chapéu	2017	80.2%	84.9%	75.8%
Brazil	Morro do Chapéu do Piauí	2000	60.8%	65.1%	56.5%
Brazil	Morro do Chapéu do Piauí	2017	61.7%	66.0%	57.5%
Brazil	Morro do Pilar	2000	80.3%	84.1%	75.6%
Brazil	Morro do Pilar	2017	80.9%	84.6%	76.4%
Brazil	Morro Grande	2000	78.2%	82.4%	74.0%
Brazil	Morro Grande	2017	78.9%	83.0%	74.7%
Brazil	Morro Redondo	2000	82.2%	85.6%	77.4%
Brazil	Morro Redondo	2017	82.8%	86.1%	78.1%

Country	Second Administrative Unit	Year	Mean	Upper	Lower
Brazil	Morro Reuter	2000	82.9%	85.3%	80.4%
Brazil	Morro Reuter	2017	83.4%	85.8%	81.0%
Brazil	Morros	2000	60.0%	64.4%	54.8%
Brazil	Morros	2017	61.0%	65.3%	55.8%
Brazil	Mortugaba	2000	79.5%	84.7%	72.5%
Brazil	Mortugaba	2017	80.1%	85.2%	73.2%
Brazil	Morungaba	2000	92.3%	93.7%	90.8%
Brazil	Morungaba	2017	92.6%	93.9%	91.1%
Brazil	Mosquito	2000	71.9%	76.1%	67.5%
Brazil	Mosquito	2017	72.7%	76.8%	68.3%
Brazil	Mossâmedes	2000	76.0%	80.3%	71.7%
Brazil	Mossâmedes	2017	76.7%	80.9%	72.5%
Brazil	Mossoró	2000	81.1%	83.4%	78.3%
Brazil	Mossoró	2017	81.9%	84.2%	79.2%
Brazil	Mostardas	2000	81.9%	87.0%	76.1%
Brazil	Mostardas	2017	82.4%	87.4%	76.8%
Brazil	Motuca	2000	94.0%	95.4%	91.7%
Brazil	Motuca	2017	94.2%	95.5%	92.0%
Brazil	Mozarlândia	2000	75.1%	81.2%	67.5%
Brazil	Mozarlândia	2017	75.9%	81.9%	68.5%
Brazil	Muaná	2000	36.0%	41.4%	31.1%
Brazil	Muaná	2017	36.9%	42.3%	31.9%
Brazil	Mucajaí	2000	85.6%	87.6%	83.4%
Brazil	Mucajaí	2017	86.1%	88.1%	84.0%
Brazil	Mucambo	2000	65.0%	69.4%	60.8%
Brazil	Mucambo	2017	65.9%	70.3%	61.8%
Brazil	Mucugê	2000	79.5%	84.5%	73.9%
Brazil	Mucugê	2017	80.2%	85.0%	74.7%
Brazil	Muçum	2000	78.6%	82.4%	74.7%
Brazil	Muçum	2017	79.3%	83.0%	75.5%
Brazil	Mucuri	2000	76.4%	81.4%	71.0%
Brazil	Mucuri	2017	77.1%	81.9%	71.7%
Brazil	Mucurici	2000	79.5%	83.5%	75.0%
Brazil	Mucurici	2017	80.1%	84.1%	75.7%
Brazil	Muitos Capões	2000	79.4%	83.6%	75.3%
Brazil	Muitos Capões	2017	80.0%	84.2%	76.0%
Brazil	Muliterno	2000	80.2%	84.0%	76.0%
Brazil	Muliterno	2017	80.8%	84.5%	76.7%
Brazil	Mulungu	2000	72.9%	76.7%	69.1%
Brazil	Mulungu	2000	67.8%	70.8%	64.2%
Brazil	Mulungu	2017	74.8%	78.4%	71.3%
Brazil	Mulungu	2017	68.6%	71.6%	65.1%
Brazil	Mulungu do Morro	2000	78.8%	84.0%	73.3%
Brazil	Mulungu do Morro	2017	79.5%	84.5%	74.1%
Brazil	Mundo Novo	2000	81.6%	86.0%	77.4%
Brazil	Mundo Novo	2000	78.6%	83.7%	74.0%
Brazil	Mundo Novo	2000	74.2%	80.6%	66.4%
Brazil	Mundo Novo	2017	79.2%	84.2%	74.7%
Brazil	Mundo Novo	2017	74.9%	81.2%	67.3%
Brazil	Mundo Novo	2017	82.1%	86.4%	78.1%
Brazil	Munhoz	2000	93.4%	94.7%	92.0%
Brazil	Munhoz	2017	93.6%	94.9%	92.3%
Brazil	Munhoz de Melo	2000	85.7%	88.4%	83.1%
Brazil	Munhoz de Melo	2017	86.2%	88.8%	83.6%
Brazil	Muniz Ferreira	2000	76.1%	79.9%	71.7%

Country	Second Administrative Unit	Year	Mean	Upper	Lower
Brazil	Muniz Ferreira	2017	76.7%	80.5%	72.5%
Brazil	Muniz Freire	2000	80.4%	83.6%	77.2%
Brazil	Muniz Freire	2017	81.0%	84.1%	77.9%
Brazil	Muquém de São Francisco	2000	75.5%	82.1%	66.2%
Brazil	Muquém de São Francisco	2017	76.1%	82.7%	67.0%
Brazil	Muqui	2000	78.0%	81.5%	74.5%
Brazil	Muqui	2017	78.7%	82.0%	75.3%
Brazil	Muriaé	2000	79.8%	83.3%	76.2%
Brazil	Muriaé	2017	80.4%	83.9%	76.9%
Brazil	Muribeca	2000	73.0%	75.6%	69.8%
Brazil	Muribeca	2017	73.8%	76.3%	70.6%
Brazil	Murici	2000	61.6%	64.8%	58.8%
Brazil	Murici	2017	62.5%	65.7%	59.7%
Brazil	Murici dos Portelas	2000	59.7%	64.5%	55.2%
Brazil	Murici dos Portelas	2017	60.6%	65.4%	56.2%
Brazil	Muricilândia	2000	68.1%	72.8%	63.4%
Brazil	Muricilândia	2017	69.2%	73.9%	64.6%
Brazil	Muritiba	2000	76.4%	80.0%	72.4%
Brazil	Muritiba	2017	77.1%	80.6%	73.1%
Brazil	Murutinga do Sul	2000	88.4%	90.9%	85.4%
Brazil	Murutinga do Sul	2017	88.8%	91.2%	85.9%
Brazil	Mutuípe	2000	78.0%	81.6%	73.5%
Brazil	Mutuípe	2017	78.7%	82.1%	74.3%
Brazil	Mutum	2000	78.2%	81.7%	74.6%
Brazil	Mutum	2017	78.9%	82.3%	75.4%
Brazil	Mutunópolis	2000	76.4%	81.9%	70.6%
Brazil	Mutunópolis	2017	77.1%	82.5%	71.3%
Brazil	Muzambinho	2000	86.9%	89.8%	83.3%
Brazil	Muzambinho	2017	87.4%	90.2%	83.9%
Brazil	Nacip Raydan	2000	81.4%	85.7%	76.0%
Brazil	Nacip Raydan	2017	82.0%	86.2%	76.7%
Brazil	Nantes	2000	88.0%	90.8%	85.4%
Brazil	Nantes	2017	88.4%	91.1%	85.9%
Brazil	Nanuque	2000	80.1%	84.3%	74.9%
Brazil	Nanuque	2017	80.7%	84.8%	75.6%
Brazil	Naque	2000	78.2%	82.4%	73.0%
Brazil	Naque	2017	78.8%	82.9%	73.7%
Brazil	Narandiba	2000	87.8%	90.8%	84.7%
Brazil	Narandiba	2017	88.3%	91.1%	85.3%
Brazil	Natal	2000	82.0%	83.7%	80.0%
Brazil	Natal	2017	82.5%	84.2%	80.7%
Brazil	Natalândia	2000	84.3%	89.5%	77.3%
Brazil	Natalândia	2017	84.8%	89.9%	77.9%
Brazil	Natércia	2000	87.5%	90.3%	84.2%
Brazil	Natércia	2017	87.9%	90.7%	84.8%
Brazil	Natividade	2000	76.9%	81.5%	71.4%
Brazil	Natividade	2000	79.4%	83.1%	76.0%
Brazil	Natividade	2017	80.0%	83.7%	76.7%
Brazil	Natividade	2017	77.6%	82.1%	72.3%
Brazil	Natividade da Serra	2000	92.8%	94.4%	90.7%
Brazil	Natividade da Serra	2017	93.0%	94.6%	91.0%
Brazil	Natuba	2000	66.2%	69.5%	63.3%
Brazil	Natuba	2017	67.2%	70.4%	64.3%

Country	Second Administrative Unit	Year	Mean	Upper	Lower
Brazil	Navegantes	2000	73.6%	77.4%	69.5%
Brazil	Navegantes	2017	74.3%	78.1%	70.3%
Brazil	Naviraí	2000	82.1%	85.5%	78.2%
Brazil	Naviraí	2017	82.7%	86.0%	78.8%
Brazil	Nazaré	2000	73.3%	77.3%	69.0%
Brazil	Nazaré	2000	75.8%	79.4%	71.9%
Brazil	Nazaré	2017	74.1%	78.0%	69.8%
Brazil	Nazaré	2017	76.5%	80.0%	72.6%
Brazil	Nazaré da Mata	2000	67.3%	70.4%	64.6%
Brazil	Nazaré da Mata	2017	68.2%	71.2%	65.5%
Brazil	Nazaré do Piauí	2000	60.1%	65.1%	54.0%
Brazil	Nazaré do Piauí	2017	61.0%	66.0%	54.9%
Brazil	Nazaré Paulista	2000	93.5%	94.5%	92.3%
Brazil	Nazaré Paulista	2017	93.7%	94.7%	92.6%
Brazil	Nazareno	2000	80.2%	84.6%	75.4%
Brazil	Nazareno	2017	80.8%	85.1%	76.1%
Brazil	Nazarezinho	2000	70.5%	74.2%	66.5%
Brazil	Nazarezinho	2017	71.4%	75.0%	67.4%
Brazil	Nazário	2000	77.0%	80.1%	73.6%
Brazil	Nazário	2017	77.6%	80.7%	74.4%
Brazil	Neópolis	2000	69.0%	71.6%	66.4%
Brazil	Neópolis	2017	69.8%	72.4%	67.2%
Brazil	Nepomuceno	2000	82.2%	85.9%	77.8%
Brazil	Nepomuceno	2017	82.8%	86.4%	78.5%
Brazil	Nerópolis	2000	78.0%	80.3%	75.7%
Brazil	Nerópolis	2017	78.6%	80.9%	76.4%
Brazil	Neves Paulista	2000	92.2%	94.1%	89.8%
Brazil	Neves Paulista	2017	92.5%	94.3%	90.2%
Brazil	Nhamundá	2000	51.2%	57.8%	45.5%
Brazil	Nhamundá	2017	52.3%	58.8%	46.6%
Brazil	Nhandeara	2000	91.3%	93.8%	88.3%
Brazil	Nhandeara	2017	91.6%	94.0%	88.7%
Brazil	Nicolau Vergueiro	2000	81.4%	84.9%	77.1%
Brazil	Nicolau Vergueiro	2017	82.0%	85.4%	77.8%
Brazil	Nilo Peçanha	2000	73.1%	77.6%	68.4%
Brazil	Nilo Peçanha	2017	73.8%	78.2%	69.1%
Brazil	Nilópolis	2000	79.4%	80.6%	78.1%
Brazil	Nilópolis	2017	80.0%	81.2%	78.8%
Brazil	Nina Rodrigues	2000	60.6%	65.7%	54.7%
Brazil	Nina Rodrigues	2017	61.5%	66.6%	55.6%
Brazil	Ninheira	2000	79.5%	84.4%	74.2%
Brazil	Ninheira	2017	80.1%	84.9%	75.0%
Brazil	Nioaque	2000	79.3%	83.1%	74.6%
Brazil	Nioaque	2017	79.9%	83.6%	75.4%
Brazil	Nipoã	2000	92.3%	94.3%	89.9%
Brazil	Nipoã	2017	92.6%	94.5%	90.3%
Brazil	Niquelândia	2000	82.4%	86.6%	77.5%
Brazil	Niquelândia	2017	83.5%	87.5%	78.9%
Brazil	Nísia Floresta	2000	79.4%	81.6%	76.9%
Brazil	Nísia Floresta	2017	80.1%	82.1%	77.6%

Country	Second Administrative Unit	Year	Mean	Upper	Lower
Brazil	Niterói	2000	77.6%	79.4%	75.5%
Brazil	Niterói	2017	78.2%	80.1%	76.2%
Brazil	Nobres	2000	67.0%	73.0%	60.5%
Brazil	Nobres	2017	67.9%	73.8%	61.5%
Brazil	Nonoai	2000	78.4%	81.7%	74.5%
Brazil	Nonoai	2017	79.0%	82.2%	75.3%
Brazil	Nordestina	2000	76.9%	81.7%	71.2%
Brazil	Nordestina	2017	77.6%	82.3%	71.9%
Brazil	Normandia	2000	85.4%	88.4%	81.0%
Brazil	Normandia	2017	85.9%	88.8%	81.7%
Brazil	Nortelândia	2000	67.2%	73.0%	60.1%
Brazil	Nortelândia	2017	68.0%	73.7%	61.0%
Brazil	Nossa Senhora Aprecido	2000	76.7%	79.2%	74.3%
Brazil	Nossa Senhora Aprecido	2017	77.4%	79.8%	75.0%
Brazil	Nossa Senhora da Glória	2000	75.5%	78.4%	72.4%
Brazil	Nossa Senhora da Glória	2017	76.2%	79.1%	73.3%
Brazil	Nossa Senhora das Dores	2000	74.6%	77.0%	71.6%
Brazil	Nossa Senhora das Dores	2017	75.3%	77.7%	72.4%
Brazil	Nossa Senhora das Graças	2000	85.4%	88.1%	82.1%
Brazil	Nossa Senhora das Graças	2017	85.9%	88.5%	82.7%
Brazil	Nossa Senhora de Lourdes	2000	68.5%	71.6%	65.0%
Brazil	Nossa Senhora de Lourdes	2017	69.3%	72.4%	65.9%
Brazil	Nossa Senhora de Nazaré	2000	59.3%	63.7%	54.9%
Brazil	Nossa Senhora de Nazaré	2017	60.2%	64.6%	55.9%
Brazil	Nossa Senhora do Livramento	2000	68.5%	72.4%	64.4%
Brazil	Nossa Senhora do Livramento	2017	69.3%	73.1%	65.3%
Brazil	Nossa Senhora do Socorro	2000	76.3%	78.0%	74.3%
Brazil	Nossa Senhora do Socorro	2017	77.0%	78.7%	75.0%
Brazil	Nossa Senhora dos Remédios	2000	60.3%	65.3%	55.7%
Brazil	Nossa Senhora dos Remédios	2017	61.3%	66.2%	56.7%
Brazil	Nova Aliança	2000	92.7%	94.5%	90.5%
Brazil	Nova Aliança	2017	93.0%	94.7%	90.9%
Brazil	Nova Aliança do Ivaí	2000	83.7%	86.7%	80.0%
Brazil	Nova Aliança do Ivaí	2017	84.3%	87.2%	80.7%
Brazil	Nova Alvorada	2000	81.4%	84.8%	77.4%
Brazil	Nova Alvorada	2017	82.0%	85.2%	78.0%
Brazil	Nova Alvorada do Sul	2000	79.7%	83.2%	76.3%

Country	Second Administrative Unit	Year	Mean	Upper	Lower
Brazil	Nova Alvorada do Sul	2017	80.3%	83.7%	77.0%
Brazil	Nova América	2000	77.0%	81.6%	71.1%
Brazil	Nova América	2017	77.7%	82.1%	71.9%
Brazil	Nova América da Colina	2000	86.8%	89.1%	84.4%
Brazil	Nova América da Colina	2017	87.3%	89.5%	84.9%
Brazil	Nova Andradina	2000	82.8%	86.3%	79.0%
Brazil	Nova Andradina	2017	83.4%	86.8%	79.6%
Brazil	Nova Araçá	2000	81.0%	84.5%	77.3%
Brazil	Nova Araçá	2017	81.6%	85.0%	78.0%
Brazil	Nova Aurora	2000	77.3%	81.7%	71.7%
Brazil	Nova Aurora	2000	83.9%	87.8%	80.1%
Brazil	Nova Aurora	2017	77.9%	82.3%	72.5%
Brazil	Nova Aurora	2017	84.4%	88.2%	80.7%
Brazil	Nova Bandeirantes	2000	67.6%	74.8%	59.9%
Brazil	Nova Bandeirantes	2017	69.5%	76.5%	62.0%
Brazil	Nova Bassano	2000	81.1%	84.4%	77.5%
Brazil	Nova Bassano	2017	81.7%	84.9%	78.1%
Brazil	Nova Belém	2000	81.3%	85.2%	77.3%
Brazil	Nova Belém	2017	81.8%	85.6%	78.0%
Brazil	Nova Boa Vista	2000	80.3%	83.9%	76.5%
Brazil	Nova Boa Vista	2017	80.9%	84.4%	77.2%
Brazil	Nova Brasilândia	2000	68.4%	75.0%	61.4%
Brazil	Nova Brasilândia	2017	69.2%	75.7%	62.3%
Brazil	Nova Brasilândia d'Oeste	2000	29.7%	33.3%	25.8%
Brazil	Nova Brasilândia d'Oeste	2017	30.5%	34.1%	26.5%
Brazil	Nova Bréscia	2000	83.0%	86.4%	79.6%
Brazil	Nova Bréscia	2017	83.5%	86.9%	80.2%
Brazil	Nova Campina	2000	91.0%	93.5%	88.1%
Brazil	Nova Campina	2017	91.3%	93.7%	88.4%
Brazil	Nova Canaã	2000	80.6%	84.5%	75.5%
Brazil	Nova Canaã	2017	81.2%	85.1%	76.3%
Brazil	Nova Canaã do Norte	2000	68.7%	74.8%	61.7%
Brazil	Nova Canaã do Norte	2017	69.5%	75.6%	62.6%
Brazil	Nova Canaã Paulista	2000	87.4%	90.9%	83.9%
Brazil	Nova Canaã Paulista	2017	87.8%	91.2%	84.4%
Brazil	Nova Candelária	2000	79.6%	84.1%	74.9%
Brazil	Nova Candelária	2017	80.3%	84.6%	75.7%
Brazil	Nova Cantu	2000	83.8%	87.5%	80.0%
Brazil	Nova Cantu	2017	84.3%	87.9%	80.6%
Brazil	Nova Castilho	2000	90.4%	93.2%	87.4%
Brazil	Nova Castilho	2017	90.7%	93.4%	87.8%

Country	Second Administrative Unit	Year	Mean	Upper	Lower
Brazil	Nova Colinas	2000	67.8%	74.0%	58.6%
Brazil	Nova Colinas	2017	68.6%	74.6%	59.4%
Brazil	Nova Crixás	2000	74.0%	80.2%	66.3%
Brazil	Nova Crixás	2017	74.7%	80.8%	67.2%
Brazil	Nova Cruz	2000	77.3%	79.9%	74.6%
Brazil	Nova Cruz	2017	78.0%	80.5%	75.4%
Brazil	Nova Era	2000	82.2%	85.8%	78.4%
Brazil	Nova Era	2017	82.8%	86.3%	79.1%
Brazil	Nova Erechim	2000	76.6%	81.0%	72.0%
Brazil	Nova Erechim	2017	77.3%	81.7%	72.8%
Brazil	Nova Esperança	2000	84.3%	87.2%	80.8%
Brazil	Nova Esperança	2017	84.8%	87.6%	81.4%
Brazil	Nova Esperança do Piriá	2000	42.2%	47.9%	36.8%
Brazil	Nova Esperança do Piriá	2017	43.2%	48.9%	37.8%
Brazil	Nova Esperança do Sudoeste	2000	82.3%	86.0%	78.2%
Brazil	Nova Esperança do Sudoeste	2017	82.8%	86.5%	78.8%
Brazil	Nova Esperança do Sul	2000	82.7%	87.0%	77.5%
Brazil	Nova Esperança do Sul	2017	83.3%	87.4%	78.2%
Brazil	Nova Europa	2000	93.2%	94.9%	90.9%
Brazil	Nova Europa	2017	93.4%	95.1%	91.3%
Brazil	Nova Fátima	2000	78.7%	82.6%	73.6%
Brazil	Nova Fátima	2000	87.9%	90.3%	85.4%
Brazil	Nova Fátima	2017	79.3%	83.1%	74.4%
Brazil	Nova Fátima	2017	88.3%	90.6%	85.8%
Brazil	Nova Floresta	2000	78.2%	81.3%	74.6%
Brazil	Nova Floresta	2017	78.8%	81.9%	75.3%
Brazil	Nova Friburgo	2000	84.8%	86.9%	82.5%
Brazil	Nova Friburgo	2017	85.3%	87.4%	83.1%
Brazil	Nova Glória	2000	78.3%	82.7%	73.5%
Brazil	Nova Glória	2017	78.9%	83.3%	74.2%
Brazil	Nova Granada	2000	91.1%	93.5%	88.7%
Brazil	Nova Granada	2017	91.5%	93.7%	89.1%
Brazil	Nova Guarita	2000	65.4%	71.6%	58.1%
Brazil	Nova Guarita	2017	66.2%	72.3%	59.0%
Brazil	Nova Guataporanga	2000	87.5%	90.3%	84.0%
Brazil	Nova Guataporanga	2017	87.9%	90.7%	84.5%
Brazil	Nova Hartz	2000	80.7%	83.5%	77.9%
Brazil	Nova Hartz	2017	81.3%	84.0%	78.6%
Brazil	Nova Ibiá	2000	76.9%	81.2%	71.9%
Brazil	Nova Ibiá	2017	77.5%	81.8%	72.7%
Brazil	Nova Iguaçu	2000	78.2%	79.6%	76.7%
Brazil	Nova Iguaçu	2017	78.8%	80.1%	77.3%
Brazil	Nova Iguaçu de Goiás	2000	77.0%	81.9%	71.3%
Brazil	Nova Iguaçu de Goiás	2017	77.7%	82.5%	72.1%

Country	Second Administrative Unit	Year	Mean	Upper	Lower
Brazil	Nova Independência	2000	87.5%	90.2%	84.4%
Brazil	Nova Independência	2017	88.0%	90.6%	84.9%
Brazil	Nova Iorque	2000	60.0%	66.9%	53.1%
Brazil	Nova Iorque	2017	61.0%	67.7%	54.0%
Brazil	Nova Ipixuna	2000	41.7%	47.3%	36.1%
Brazil	Nova Ipixuna	2017	42.7%	48.2%	37.1%
Brazil	Nova Itaberaba	2000	76.1%	80.5%	71.5%
Brazil	Nova Itaberaba	2017	76.9%	81.2%	72.4%
Brazil	Nova Itarana	2000	81.7%	85.5%	76.7%
Brazil	Nova Itarana	2017	82.3%	86.0%	77.4%
Brazil	Nova Lacerda	2000	54.9%	64.3%	47.0%
Brazil	Nova Lacerda	2017	55.9%	65.3%	48.1%
Brazil	Nova Laranjeiras	2000	83.2%	86.9%	78.9%
Brazil	Nova Laranjeiras	2017	83.8%	87.3%	79.5%
Brazil	Nova Lima	2000	83.1%	85.5%	80.4%
Brazil	Nova Lima	2017	83.5%	86.0%	80.9%
Brazil	Nova Londrina	2000	84.6%	88.1%	80.5%
Brazil	Nova Londrina	2017	85.1%	88.5%	81.1%
Brazil	Nova Luzitânia	2000	90.9%	93.5%	87.9%
Brazil	Nova Luzitânia	2017	91.2%	93.7%	88.3%
Brazil	Nova Mamoré	2000	30.6%	36.2%	25.6%
Brazil	Nova Mamoré	2017	31.4%	37.1%	26.4%
Brazil	Nova Marilândia	2000	67.8%	73.5%	60.6%
Brazil	Nova Marilândia	2017	68.6%	74.2%	61.4%
Brazil	Nova Maringá	2000	67.8%	75.1%	60.7%
Brazil	Nova Maringá	2017	68.7%	75.8%	61.7%
Brazil	Nova Módica	2000	81.5%	86.5%	76.8%
Brazil	Nova Módica	2017	82.1%	86.9%	77.5%
Brazil	Nova Monte Verde	2000	68.0%	75.0%	59.4%
Brazil	Nova Monte Verde	2017	68.8%	75.7%	60.4%
Brazil	Nova Mutum	2000	69.0%	75.6%	62.6%
Brazil	Nova Mutum	2017	70.9%	77.3%	64.7%
Brazil	Nova Odessa	2000	93.3%	94.5%	91.8%
Brazil	Nova Odessa	2017	93.6%	94.7%	92.1%
Brazil	Nova Olímpia	2000	66.1%	71.5%	60.5%
Brazil	Nova Olímpia	2000	83.8%	86.8%	80.4%
Brazil	Nova Olímpia	2017	67.0%	72.3%	61.5%
Brazil	Nova Olímpia	2017	84.3%	87.2%	81.0%
Brazil	Nova Olinda	2000	67.0%	71.0%	62.6%
Brazil	Nova Olinda	2000	74.5%	77.9%	70.7%
Brazil	Nova Olinda	2000	69.0%	72.4%	64.7%
Brazil	Nova Olinda	2017	75.2%	78.6%	71.5%
Brazil	Nova Olinda	2017	67.9%	71.8%	63.5%
Brazil	Nova Olinda	2017	69.8%	73.2%	65.6%
Brazil	Nova Olinda do Maranhão	2000	57.3%	64.0%	51.0%
Brazil	Nova Olinda do Maranhão	2017	58.2%	64.8%	52.0%

Country	Second Administrative Unit	Year	Mean	Upper	Lower
Brazil	Nova Olinda do Norte	2000	57.5%	62.4%	52.0%
Brazil	Nova Olinda do Norte	2017	58.4%	63.3%	52.9%
Brazil	Nova Pádua	2000	82.1%	85.6%	78.0%
Brazil	Nova Pádua	2017	82.8%	86.1%	78.8%
Brazil	Nova Palma	2000	81.8%	83.7%	79.0%
Brazil	Nova Palma	2017	82.3%	84.2%	79.7%
Brazil	Nova Palmeira	2000	76.6%	80.0%	72.3%
Brazil	Nova Palmeira	2017	77.3%	80.6%	73.0%
Brazil	Nova Petrópolis	2000	81.5%	84.1%	78.6%
Brazil	Nova Petrópolis	2017	82.1%	84.6%	79.3%
Brazil	Nova Ponte	2000	82.7%	86.6%	78.1%
Brazil	Nova Ponte	2017	83.3%	87.0%	78.7%
Brazil	Nova Porteirinha	2000	83.2%	87.7%	77.4%
Brazil	Nova Porteirinha	2017	83.8%	88.2%	78.1%
Brazil	Nova Prata	2000	81.0%	83.8%	77.6%
Brazil	Nova Prata	2017	81.6%	84.3%	78.4%
Brazil	Nova Prata do Iguaçu	2000	83.4%	86.9%	79.9%
Brazil	Nova Prata do Iguaçu	2017	83.9%	87.3%	80.5%
Brazil	Nova Ramada	2000	82.2%	86.0%	77.2%
Brazil	Nova Ramada	2017	82.7%	86.5%	77.9%
Brazil	Nova Redenção	2000	75.4%	81.2%	68.8%
Brazil	Nova Redenção	2017	76.1%	81.8%	69.6%
Brazil	Nova Resende	2000	86.4%	89.6%	82.4%
Brazil	Nova Resende	2017	86.8%	89.9%	82.9%
Brazil	Nova Roma	2000	78.9%	83.9%	73.0%
Brazil	Nova Roma	2017	79.5%	84.5%	73.8%
Brazil	Nova Roma do Sul	2000	81.3%	84.7%	77.9%
Brazil	Nova Roma do Sul	2017	81.9%	85.2%	78.6%
Brazil	Nova Rosalândia	2000	74.9%	79.3%	70.3%
Brazil	Nova Rosalândia	2017	75.6%	79.9%	71.1%
Brazil	Nova Russas	2000	68.3%	73.1%	63.5%
Brazil	Nova Russas	2017	69.2%	73.9%	64.4%
Brazil	Nova Santa Bárbara	2000	85.4%	88.1%	82.6%
Brazil	Nova Santa Bárbara	2017	85.9%	88.5%	83.2%
Brazil	Nova Santa Rita	2000	81.6%	83.7%	79.5%
Brazil	Nova Santa Rita	2000	61.2%	66.6%	54.8%
Brazil	Nova Santa Rita	2017	82.6%	84.6%	80.6%
Brazil	Nova Santa Rita	2017	62.1%	67.3%	55.8%
Brazil	Nova Santa Rosa	2000	83.5%	87.0%	79.8%

Country	Second Administrative Unit	Year	Mean	Upper	Lower
Brazil	Nova Santa Rosa	2017	84.1%	87.4%	80.4%
Brazil	Nova Serrana	2000	81.9%	85.7%	77.7%
Brazil	Nova Serrana	2017	82.5%	86.2%	78.4%
Brazil	Nova Soure	2000	78.3%	81.8%	74.2%
Brazil	Nova Soure	2017	79.0%	82.4%	75.0%
Brazil	Nova Tebas	2000	83.5%	86.8%	79.5%
Brazil	Nova Tebas	2017	84.0%	87.3%	80.1%
Brazil	Nova Timboteua	2000	36.9%	41.3%	33.2%
Brazil	Nova Timboteua	2017	37.8%	42.3%	34.0%
Brazil	Nova Trento	2000	72.2%	76.2%	67.5%
Brazil	Nova Trento	2017	73.5%	77.3%	68.9%
Brazil	Nova Ubiratã	2000	68.1%	74.1%	61.3%
Brazil	Nova Ubiratã	2017	68.9%	74.8%	62.2%
Brazil	Nova União	2000	81.5%	84.8%	77.9%
Brazil	Nova União	2000	29.7%	33.1%	26.1%
Brazil	Nova União	2017	82.1%	85.3%	78.6%
Brazil	Nova União	2017	30.6%	34.0%	26.9%
Brazil	Nova Venécia	2000	77.6%	81.3%	74.3%
Brazil	Nova Venécia	2017	78.2%	81.9%	75.0%
Brazil	Nova Veneza	2000	76.4%	80.4%	71.9%
Brazil	Nova Veneza	2000	78.7%	81.1%	76.4%
Brazil	Nova Veneza	2017	77.0%	81.0%	72.6%
Brazil	Nova Veneza	2017	79.3%	81.7%	77.1%
Brazil	Nova Viçosa	2000	77.1%	81.9%	71.5%
Brazil	Nova Viçosa	2017	77.8%	82.5%	72.3%
Brazil	Nova Xavantina	2000	72.0%	78.2%	65.1%
Brazil	Nova Xavantina	2017	72.8%	78.8%	65.9%
Brazil	Novais	2000	92.7%	94.3%	90.8%
Brazil	Novais	2017	93.0%	94.5%	91.1%
Brazil	Novo Acordo	2000	74.1%	79.2%	68.4%
Brazil	Novo Acordo	2017	74.8%	79.9%	69.3%
Brazil	Novo Airão	2000	60.6%	66.2%	55.2%
Brazil	Novo Airão	2017	63.1%	68.5%	57.7%
Brazil	Novo Alegre	2000	76.8%	81.8%	70.2%
Brazil	Novo Alegre	2017	77.5%	82.3%	71.0%
Brazil	Novo Aripuanã	2000	60.2%	68.2%	51.5%
Brazil	Novo Aripuanã	2017	61.1%	69.0%	52.4%
Brazil	Novo Barreiro	2000	79.5%	83.5%	75.9%
Brazil	Novo Barreiro	2017	80.1%	84.0%	76.6%
Brazil	Novo Brazil	2000	74.9%	79.7%	69.4%
Brazil	Novo Brazil	2017	75.6%	80.3%	70.2%
Brazil	Novo Cabrais	2000	80.8%	84.5%	76.3%
Brazil	Novo Cabrais	2017	81.5%	85.1%	77.1%
Brazil	Novo Cruzeiro	2000	83.8%	88.3%	78.4%
Brazil	Novo Cruzeiro	2017	84.4%	88.7%	79.1%
Brazil	Novo Gama	2000	90.3%	91.4%	88.9%
Brazil	Novo Gama	2017	90.6%	91.7%	89.2%
Brazil	Novo Hamburgo	2000	81.6%	83.7%	79.3%
Brazil	Novo Hamburgo	2017	82.2%	84.2%	80.0%
Brazil	Novo Horizonte	2000	79.7%	83.7%	75.8%
Brazil	Novo Horizonte	2000	93.6%	95.3%	91.5%

Country	Second Administrative Unit	Year	Mean	Upper	Lower
Brazil	Novo Horizonte	2000	79.5%	84.7%	74.3%
Brazil	Novo Horizonte	2017	80.0%	85.1%	75.0%
Brazil	Novo Horizonte	2017	80.3%	84.3%	76.5%
Brazil	Novo Horizonte	2017	93.8%	95.5%	91.8%
Brazil	Novo Horizonte do Norte	2000	68.7%	75.8%	60.9%
Brazil	Novo Horizonte do Norte	2017	69.7%	76.7%	62.0%
Brazil	Novo Horizonte do Oeste	2000	28.6%	31.9%	24.6%
Brazil	Novo Horizonte do Oeste	2017	29.4%	32.7%	25.3%
Brazil	Novo Horizonte do Sul	2000	81.0%	84.7%	77.0%
Brazil	Novo Horizonte do Sul	2017	81.6%	85.2%	77.7%
Brazil	Novo Itacolomi	2000	83.8%	86.7%	80.3%
Brazil	Novo Itacolomi	2017	84.3%	87.1%	80.9%
Brazil	Novo Jardim	2000	75.1%	80.8%	68.7%
Brazil	Novo Jardim	2017	75.8%	81.4%	69.6%
Brazil	Novo Lino	2000	62.0%	65.4%	59.1%
Brazil	Novo Lino	2017	62.9%	66.2%	60.0%
Brazil	Novo Machado	2000	79.8%	84.4%	73.9%
Brazil	Novo Machado	2017	80.3%	84.9%	74.5%
Brazil	Novo Mundo	2000	62.4%	69.1%	54.4%
Brazil	Novo Mundo	2017	63.4%	70.0%	55.4%
Brazil	Novo Oriente	2000	68.0%	72.5%	63.0%
Brazil	Novo Oriente	2017	68.8%	73.3%	64.0%
Brazil	Novo Oriente de Minas	2000	82.5%	86.8%	77.7%
Brazil	Novo Oriente de Minas	2017	83.1%	87.3%	78.4%
Brazil	Novo Oriente do Piauí	2000	60.7%	65.5%	55.2%
Brazil	Novo Oriente do Piauí	2017	61.6%	66.4%	56.2%
Brazil	Novo Planalto	2000	75.0%	80.4%	68.5%
Brazil	Novo Planalto	2017	75.8%	81.1%	69.3%
Brazil	Novo Progresso	2000	44.5%	53.8%	34.2%
Brazil	Novo Progresso	2017	45.5%	54.8%	35.1%
Brazil	Novo Repartimento	2000	39.0%	44.4%	32.3%
Brazil	Novo Repartimento	2017	39.9%	45.4%	33.1%
Brazil	Novo Santo Antônio	2000	60.2%	64.5%	55.6%

Country	Second Administrative Unit	Year	Mean	Upper	Lower
Brazil	Novo Santo Antônio	2017	61.1%	65.4%	56.6%
Brazil	Novo São Joaquim	2000	69.2%	75.8%	62.1%
Brazil	Novo São Joaquim	2017	70.0%	76.5%	63.0%
Brazil	Novo Tiradentes	2000	78.4%	82.3%	74.3%
Brazil	Novo Tiradentes	2017	79.1%	82.8%	75.1%
Brazil	Novo Triunfo	2000	75.2%	80.2%	70.3%
Brazil	Novo Triunfo	2017	76.0%	80.8%	71.1%
Brazil	Novorizonte	2000	81.2%	86.4%	75.6%
Brazil	Novorizonte	2017	81.7%	86.8%	76.3%
Brazil	Nuporanga	2000	91.2%	93.3%	88.3%
Brazil	Nuporanga	2017	91.6%	93.6%	88.7%
Brazil	Óbidos	2000	42.2%	49.7%	35.1%
Brazil	Óbidos	2017	43.2%	50.6%	36.0%
Brazil	Ocara	2000	72.5%	75.9%	68.8%
Brazil	Ocara	2017	73.3%	76.6%	69.6%
Brazil	Ocauçu	2000	92.3%	94.2%	89.9%
Brazil	Ocauçu	2017	92.6%	94.4%	90.3%
Brazil	Oeiras	2000	60.1%	64.9%	54.7%
Brazil	Oeiras	2017	61.4%	66.1%	56.0%
Brazil	Oeiras do Pará	2000	39.2%	45.1%	34.1%
Brazil	Oeiras do Pará	2017	40.1%	46.0%	35.0%
Brazil	Oiapoque	2000	48.6%	56.7%	41.0%
Brazil	Oiapoque	2017	49.5%	57.6%	41.9%
Brazil	Olaria	2000	82.4%	85.5%	78.4%
Brazil	Olaria	2017	82.9%	86.0%	79.1%
Brazil	Óleo	2000	92.3%	94.5%	89.8%
Brazil	Óleo	2017	92.5%	94.7%	90.2%
Brazil	Olho d'Água	2000	65.7%	69.2%	61.8%
Brazil	Olho d'Água	2017	66.6%	70.0%	62.7%
Brazil	Olho d'Água das Cunhãs	2000	61.4%	66.2%	56.6%
Brazil	Olho d'Água das Cunhãs	2017	62.3%	67.1%	57.5%
Brazil	Olho d'Água das Flores	2000	61.7%	64.9%	58.7%
Brazil	Olho d'Água das Flores	2017	62.6%	65.8%	59.6%
Brazil	Olho d'Água do Casado	2000	67.7%	71.1%	64.0%
Brazil	Olho d'Água do Casado	2017	68.6%	72.0%	64.9%
Brazil	Olho d'água do Piauí	2000	59.9%	64.2%	55.2%
Brazil	Olho d'água do Piauí	2017	60.8%	65.1%	56.1%
Brazil	Olho d'Água Grande	2000	66.3%	69.3%	63.3%
Brazil	Olho d'Água Grande	2017	67.1%	70.2%	64.2%
Brazil	Olho-d'Água do Borges	2000	78.7%	81.8%	75.1%
Brazil	Olho-d'Água do Borges	2017	79.3%	82.3%	75.8%
Brazil	Olhos-d'Água	2000	81.6%	86.3%	76.2%
Brazil	Olhos-d'Água	2017	82.2%	86.7%	76.9%

Country	Second Administrative Unit	Year	Mean	Upper	Lower
Brazil	Olímpia	2000	91.9%	93.8%	89.5%
Brazil	Olímpia	2017	92.1%	94.0%	89.8%
Brazil	Olímpio Noronha	2000	87.1%	90.4%	83.7%
Brazil	Olímpio Noronha	2017	87.5%	90.7%	84.2%
Brazil	Olinda	2000	66.5%	68.4%	64.5%
Brazil	Olinda	2017	67.3%	69.2%	65.4%
Brazil	Olinda Nova do Maranhão	2000	60.8%	65.7%	55.9%
Brazil	Olinda Nova do Maranhão	2017	61.7%	66.6%	56.8%
Brazil	Olindina	2000	78.5%	82.2%	74.2%
Brazil	Olindina	2017	79.2%	82.8%	75.0%
Brazil	Olivedos	2000	72.4%	75.8%	68.2%
Brazil	Olivedos	2017	73.2%	76.6%	69.1%
Brazil	Oliveira	2000	80.8%	84.9%	76.4%
Brazil	Oliveira	2017	81.4%	85.4%	77.1%
Brazil	Oliveira de Fátima	2000	75.3%	79.8%	71.3%
Brazil	Oliveira de Fátima	2017	76.0%	80.4%	72.1%
Brazil	Oliveira Fortes	2000	81.2%	84.7%	77.0%
Brazil	Oliveira Fortes	2017	81.9%	85.3%	77.8%
Brazil	Olivença	2000	62.3%	65.1%	59.3%
Brazil	Olivença	2017	63.2%	66.0%	60.2%
Brazil	Oliveria dos Brejinhos	2000	75.8%	82.5%	68.7%
Brazil	Oliveria dos Brejinhos	2017	76.6%	83.1%	69.5%
Brazil	Onça de Pitangui	2000	80.8%	84.7%	76.5%
Brazil	Onça de Pitangui	2017	81.4%	85.2%	77.2%
Brazil	Onda Verde	2000	91.5%	93.7%	89.1%
Brazil	Onda Verde	2017	91.8%	93.9%	89.5%
Brazil	Oratórios	2000	81.0%	84.8%	76.9%
Brazil	Oratórios	2017	81.6%	85.3%	77.6%
Brazil	Oriente	2000	92.5%	94.5%	89.9%
Brazil	Oriente	2017	92.8%	94.7%	90.2%
Brazil	Orindiúva	2000	89.6%	92.1%	86.3%
Brazil	Orindiúva	2017	89.9%	92.4%	86.8%
Brazil	Oriximiná	2000	44.2%	51.8%	36.4%
Brazil	Oriximiná	2017	45.2%	52.8%	37.3%
Brazil	Orizânia	2000	81.8%	85.1%	78.0%
Brazil	Orizânia	2017	82.4%	85.6%	78.7%
Brazil	Orizona	2000	79.8%	83.3%	75.5%
Brazil	Orizona	2017	80.4%	83.8%	76.2%
Brazil	Orlandia	2000	91.3%	93.4%	88.6%
Brazil	Orlandia	2017	91.6%	93.6%	89.0%
Brazil	Orleaes	2000	73.1%	77.2%	68.8%
Brazil	Orleaes	2017	73.9%	77.9%	69.6%
Brazil	Orobó	2000	65.8%	68.5%	62.6%
Brazil	Orobó	2017	66.9%	69.6%	63.7%
Brazil	Orós	2000	70.1%	74.6%	66.0%
Brazil	Orós	2017	71.0%	75.3%	66.8%
Brazil	Ortigueira	2000	84.3%	87.5%	80.3%
Brazil	Ortigueira	2017	84.9%	87.9%	81.0%
Brazil	Osasco	2000	94.3%	95.1%	93.5%
Brazil	Osasco	2017	94.5%	95.3%	93.7%

Country	Second Administrative Unit	Year	Mean	Upper	Lower
Brazil	Oscar Bressane	2000	91.4%	93.6%	88.5%
Brazil	Oscar Bressane	2017	91.7%	93.8%	88.8%
Brazil	Osório	2000	79.4%	83.5%	74.8%
Brazil	Osório	2017	80.0%	84.0%	75.5%
Brazil	Osvaldo Cruz	2000	91.1%	93.5%	88.4%
Brazil	Osvaldo Cruz	2017	91.4%	93.7%	88.8%
Brazil	Otacílio Costa	2000	76.5%	81.2%	71.3%
Brazil	Otacílio Costa	2017	77.2%	81.8%	72.1%
Brazil	Ourém	2000	39.8%	44.8%	35.5%
Brazil	Ourém	2017	40.7%	45.7%	36.4%
Brazil	Ouriçangas	2000	78.4%	81.8%	74.9%
Brazil	Ouriçangas	2017	79.1%	82.4%	75.7%
Brazil	Ouricuri	2000	67.3%	71.8%	63.0%
Brazil	Ouricuri	2017	68.1%	72.6%	63.9%
Brazil	Ourilândia do Norte	2000	39.1%	46.7%	32.5%
Brazil	Ourilândia do Norte	2017	40.1%	47.7%	33.4%
Brazil	Ourinhos	2000	91.3%	93.5%	88.3%
Brazil	Ourinhos	2017	91.7%	93.8%	88.7%
Brazil	Ourizona	2000	83.8%	86.5%	80.4%
Brazil	Ourizona	2017	84.3%	87.0%	81.0%
Brazil	Ouro	2000	76.7%	80.6%	72.2%
Brazil	Ouro	2017	77.4%	81.3%	73.0%
Brazil	Ouro Branco	2000	83.7%	86.9%	81.0%
Brazil	Ouro Branco	2000	75.8%	79.7%	71.6%
Brazil	Ouro Branco	2000	62.0%	65.6%	58.2%
Brazil	Ouro Branco	2017	76.5%	80.3%	72.3%
Brazil	Ouro Branco	2017	84.2%	87.4%	81.5%
Brazil	Ouro Branco	2017	62.9%	66.4%	59.1%
Brazil	Ouro Fino	2000	89.4%	91.5%	86.8%
Brazil	Ouro Fino	2017	89.8%	91.8%	87.3%
Brazil	Ouro Preto	2000	83.2%	86.5%	80.2%
Brazil	Ouro Preto	2017	83.7%	86.9%	80.8%
Brazil	Ouro Preto do Oeste	2000	31.6%	34.7%	28.0%
Brazil	Ouro Preto do Oeste	2017	32.4%	35.6%	28.8%
Brazil	Ouro Velho	2000	66.9%	71.1%	62.7%
Brazil	Ouro Velho	2017	67.8%	71.9%	63.6%
Brazil	Ouro Verde	2000	76.7%	80.9%	71.9%
Brazil	Ouro Verde	2000	87.3%	90.2%	83.8%
Brazil	Ouro Verde	2017	77.3%	81.5%	72.6%
Brazil	Ouro Verde	2017	87.7%	90.6%	84.3%
Brazil	Ouro Verde de Goiás	2000	78.2%	80.9%	75.6%
Brazil	Ouro Verde de Goiás	2017	78.8%	81.5%	76.3%
Brazil	Ouro Verde de Minas	2000	82.0%	86.3%	76.7%
Brazil	Ouro Verde de Minas	2017	82.6%	86.8%	77.4%
Brazil	Ouro Verde do Oeste	2000	84.8%	88.1%	81.5%
Brazil	Ouro Verde do Oeste	2017	85.2%	88.5%	82.1%
Brazil	Ouroeste	2000	87.8%	91.0%	84.4%
Brazil	Ouroeste	2017	88.3%	91.4%	84.9%
Brazil	Ourolândia	2000	77.1%	82.7%	71.2%
Brazil	Ourolândia	2017	77.7%	83.2%	71.9%

Country	Second Administrative Unit	Year	Mean	Upper	Lower
Brazil	Ouvidor	2000	78.0%	82.8%	72.1%
Brazil	Ouvidor	2017	78.6%	83.3%	72.9%
Brazil	Pacaembu	2000	89.5%	91.9%	86.4%
Brazil	Pacaembu	2017	89.9%	92.2%	86.8%
Brazil	Pacajá	2000	39.1%	45.9%	31.5%
Brazil	Pacajá	2017	40.0%	46.9%	32.3%
Brazil	Pacajús	2000	71.0%	73.8%	67.8%
Brazil	Pacajús	2017	71.8%	74.5%	68.6%
Brazil	Pacaraima	2000	89.2%	92.1%	85.6%
Brazil	Pacaraima	2017	89.6%	92.4%	86.1%
Brazil	Pacatuba	2000	68.8%	72.0%	65.4%
Brazil	Pacatuba	2000	69.9%	72.0%	67.7%
Brazil	Pacatuba	2017	69.7%	72.8%	66.3%
Brazil	Pacatuba	2017	70.7%	72.8%	68.5%
Brazil	Paço do Lumiar	2000	60.4%	64.0%	56.1%
Brazil	Paço do Lumiar	2017	61.4%	65.0%	57.2%
Brazil	Pacoti	2000	72.7%	75.8%	69.5%
Brazil	Pacoti	2017	73.4%	76.4%	70.2%
Brazil	Pacujá	2000	65.3%	69.5%	61.0%
Brazil	Pacujá	2017	66.1%	70.3%	61.9%
Brazil	Padre Bernardo	2000	88.7%	91.0%	86.1%
Brazil	Padre Bernardo	2017	89.1%	91.4%	86.6%
Brazil	Padre Carvalho	2000	80.9%	86.1%	75.0%
Brazil	Padre Carvalho	2017	81.5%	86.6%	75.7%
Brazil	Padre Marcos	2000	63.4%	68.1%	58.7%
Brazil	Padre Marcos	2017	64.3%	68.9%	59.6%
Brazil	Padre Paraíso	2000	82.9%	87.4%	76.2%
Brazil	Padre Paraíso	2017	83.5%	87.8%	76.9%
Brazil	Paes Landim	2000	59.9%	65.6%	54.1%
Brazil	Paes Landim	2017	60.8%	66.5%	55.0%
Brazil	Pai Pedro	2000	80.3%	85.8%	74.6%
Brazil	Pai Pedro	2017	80.9%	86.2%	75.4%
Brazil	Paial	2000	75.5%	79.4%	71.2%
Brazil	Paial	2017	76.0%	79.8%	71.7%
Brazil	Paiçandu	2000	84.6%	87.1%	81.3%
Brazil	Paiçandu	2017	85.1%	87.5%	81.9%
Brazil	Paim Filho	2000	77.8%	81.7%	73.5%
Brazil	Paim Filho	2017	78.5%	82.3%	74.2%
Brazil	Paineiras	2000	79.9%	85.4%	72.9%
Brazil	Paineiras	2017	80.5%	85.9%	73.6%
Brazil	Painel	2000	79.6%	83.8%	74.6%
Brazil	Painel	2017	80.2%	84.4%	75.3%
Brazil	Pains	2000	81.9%	86.0%	76.9%
Brazil	Pains	2017	82.5%	86.5%	77.6%
Brazil	Paiva	2000	80.1%	83.4%	75.9%
Brazil	Paiva	2017	80.7%	84.0%	76.6%
Brazil	Pajeú do Piauí	2000	61.9%	68.3%	55.5%
Brazil	Pajeú do Piauí	2017	62.8%	69.1%	56.4%
Brazil	Palestina	2000	64.0%	67.5%	60.0%
Brazil	Palestina	2000	90.4%	92.9%	87.5%
Brazil	Palestina	2017	64.9%	68.4%	60.9%
Brazil	Palestina	2017	90.7%	93.2%	88.0%
Brazil	Palestina de Goiás	2000	75.8%	81.4%	69.5%

Country	Second Administrative Unit	Year	Mean	Upper	Lower
Brazil	Palestina de Goiás	2017	76.5%	82.0%	70.3%
Brazil	Palestina do Pará	2000	62.0%	66.7%	57.0%
Brazil	Palestina do Pará	2017	62.9%	67.6%	58.0%
Brazil	Palhano	2000	73.5%	77.4%	69.5%
Brazil	Palhano	2017	74.3%	78.1%	70.4%
Brazil	Palhoça	2000	73.1%	78.3%	68.0%
Brazil	Palhoça	2017	73.9%	79.0%	68.9%
Brazil	Palma	2000	81.7%	85.5%	76.7%
Brazil	Palma	2017	82.0%	85.7%	77.4%
Brazil	Palma Sola	2000	81.2%	85.6%	76.5%
Brazil	Palma Sola	2017	81.8%	86.1%	77.2%
Brazil	Palmácia	2000	71.0%	74.0%	67.6%
Brazil	Palmácia	2017	71.8%	74.7%	68.4%
Brazil	Palmares	2000	61.3%	64.8%	57.9%
Brazil	Palmares	2017	62.2%	65.6%	58.8%
Brazil	Palmares do Sul	2000	81.0%	85.4%	76.0%
Brazil	Palmares do Sul	2017	81.6%	85.9%	76.7%
Brazil	Palmares Paulista	2000	94.1%	95.4%	92.4%
Brazil	Palmares Paulista	2017	94.4%	95.5%	92.7%
Brazil	Palmas	2000	76.3%	78.9%	73.7%
Brazil	Palmas	2000	81.1%	84.7%	77.3%
Brazil	Palmas	2017	77.0%	79.6%	74.5%
Brazil	Palmas	2017	81.7%	85.2%	78.0%
Brazil	Palmas de Monte Alto	2000	77.8%	83.5%	72.4%
Brazil	Palmas de Monte Alto	2017	78.5%	84.0%	73.2%
Brazil	Palmeira	2000	82.1%	85.0%	78.6%
Brazil	Palmeira	2000	76.4%	80.9%	71.3%
Brazil	Palmeira	2017	77.1%	81.6%	72.1%
Brazil	Palmeira	2017	82.6%	85.5%	79.3%
Brazil	Palmeira das Missões	2000	81.2%	85.0%	77.4%
Brazil	Palmeira das Missões	2017	81.8%	85.5%	78.0%
Brazil	Palmeira do Oeste	2000	88.8%	91.9%	85.4%
Brazil	Palmeira do Oeste	2017	89.2%	92.2%	85.9%
Brazil	Palmeira do Piauí	2000	60.1%	67.8%	53.0%
Brazil	Palmeira do Piauí	2017	61.0%	68.6%	53.9%
Brazil	Palmeira dos índios	2000	61.2%	64.2%	58.4%
Brazil	Palmeira dos índios	2017	62.1%	65.1%	59.3%
Brazil	Palmeirais	2000	59.3%	64.0%	53.9%
Brazil	Palmeirais	2017	61.0%	65.6%	55.6%
Brazil	Palmeirândia	2000	60.4%	65.6%	55.4%
Brazil	Palmeirândia	2017	61.4%	66.5%	56.3%
Brazil	Palmeirante	2000	79.0%	83.8%	73.1%
Brazil	Palmeirante	2017	79.7%	84.4%	73.9%
Brazil	Palmeiras	2000	78.5%	83.8%	72.9%
Brazil	Palmeiras	2017	79.2%	84.3%	73.7%

Country	Second Administrative Unit	Year	Mean	Upper	Lower
Brazil	Palmeiras de Goiás	2000	76.1%	80.3%	72.4%
Brazil	Palmeiras de Goiás	2017	76.8%	80.9%	73.1%
Brazil	Palmeirina	2000	62.2%	65.5%	58.4%
Brazil	Palmeirina	2017	63.1%	66.3%	59.3%
Brazil	Palmeirópolis	2000	73.5%	77.4%	68.8%
Brazil	Palmeirópolis	2017	74.3%	78.1%	69.7%
Brazil	Palmelo	2000	78.7%	82.5%	74.2%
Brazil	Palmelo	2017	79.3%	83.0%	74.9%
Brazil	Palminópolis	2000	76.4%	80.5%	72.0%
Brazil	Palminópolis	2017	77.1%	81.1%	72.8%
Brazil	Palmital	2000	90.1%	92.4%	87.3%
Brazil	Palmital	2000	84.0%	87.5%	80.0%
Brazil	Palmital	2017	90.4%	92.7%	87.7%
Brazil	Palmital	2017	84.5%	87.9%	80.6%
Brazil	Palmitinhos	2000	79.4%	83.2%	74.5%
Brazil	Palmitinhos	2017	80.1%	83.7%	75.2%
Brazil	Palmitos	2000	77.3%	81.3%	73.0%
Brazil	Palmitos	2017	78.7%	82.6%	74.6%
Brazil	Palmópolis	2000	77.2%	80.9%	72.4%
Brazil	Palmópolis	2017	77.9%	81.5%	73.2%
Brazil	Palotina	2000	83.0%	86.4%	78.8%
Brazil	Palotina	2017	83.6%	86.9%	79.4%
Brazil	Panamá	2000	77.9%	82.3%	73.5%
Brazil	Panamá	2017	78.6%	82.9%	74.3%
Brazil	Panambi	2000	82.9%	86.8%	78.6%
Brazil	Panambi	2017	83.4%	87.3%	79.2%
Brazil	Pancas	2000	78.6%	81.8%	75.1%
Brazil	Pancas	2017	79.2%	82.4%	75.8%
Brazil	Panelas	2000	69.0%	72.4%	65.9%
Brazil	Panelas	2017	69.8%	73.2%	66.7%
Brazil	Panorama	2000	85.5%	88.7%	81.3%
Brazil	Panorama	2017	86.0%	89.1%	81.9%
Brazil	Pantano Grande	2000	81.4%	85.5%	76.8%
Brazil	Pantano Grande	2017	81.9%	86.0%	77.5%
Brazil	Pão de Açúcar	2000	64.8%	68.1%	60.7%
Brazil	Pão de Açúcar	2017	65.7%	69.0%	61.6%
Brazil	Papagaios	2000	81.0%	85.4%	76.0%
Brazil	Papagaios	2017	81.6%	85.9%	76.7%
Brazil	Papanduva	2000	77.4%	81.3%	73.5%
Brazil	Papanduva	2017	78.0%	81.9%	74.3%
Brazil	Paquetá	2000	61.7%	66.6%	56.1%
Brazil	Paquetá	2017	62.6%	67.5%	57.1%
Brazil	Pará de Minas	2000	81.8%	85.3%	77.5%
Brazil	Pará de Minas	2017	82.4%	85.8%	78.2%
Brazil	Paracambi	2000	76.9%	79.2%	74.1%
Brazil	Paracambi	2017	77.6%	79.9%	74.8%
Brazil	Paracatu	2000	82.8%	87.9%	77.0%
Brazil	Paracatu	2017	83.3%	88.3%	77.7%
Brazil	Paracuru	2000	68.6%	72.3%	64.4%
Brazil	Paracuru	2017	69.4%	73.0%	65.3%
Brazil	Paragominas	2000	43.5%	50.2%	37.0%
Brazil	Paragominas	2017	45.3%	51.8%	38.8%
Brazil	Paraguaçu	2000	85.4%	88.3%	81.4%
Brazil	Paraguaçu	2017	85.9%	88.7%	82.0%
Brazil	Paraguaçu Paulista	2000	90.8%	93.1%	88.5%
Brazil	Paraguaçu Paulista	2017	91.1%	93.3%	88.9%

Country	Second Administrative Unit	Year	Mean	Upper	Lower
Brazil	Paraí	2000	80.9%	84.4%	77.2%
Brazil	Paraí	2017	81.5%	84.9%	77.9%
Brazil	Paraíba do Sul	2000	78.0%	81.1%	74.6%
Brazil	Paraíba do Sul	2017	78.7%	81.7%	75.4%
Brazil	Paraibano	2000	64.1%	70.5%	57.6%
Brazil	Paraibano	2017	65.0%	71.3%	58.6%
Brazil	Paraibuna	2000	93.3%	94.6%	91.8%
Brazil	Paraibuna	2017	93.5%	94.8%	92.0%
Brazil	Paraipaba	2000	70.1%	73.9%	65.9%
Brazil	Paraipaba	2017	70.9%	74.7%	66.8%
Brazil	Paraíso	2000	80.2%	84.5%	75.8%
Brazil	Paraíso	2000	93.0%	94.5%	91.0%
Brazil	Paraíso	2017	80.9%	85.0%	76.5%
Brazil	Paraíso	2017	93.2%	94.7%	91.3%
Brazil	Paraíso do Norte	2000	83.5%	86.7%	80.0%
Brazil	Paraíso do Norte	2017	84.1%	87.1%	80.6%
Brazil	Paraíso do Sul	2000	81.8%	85.2%	77.3%
Brazil	Paraíso do Sul	2017	82.3%	85.6%	77.9%
Brazil	Paraíso do Tocantins	2000	76.0%	79.9%	72.1%
Brazil	Paraíso do Tocantins	2017	76.7%	80.5%	72.9%
Brazil	Paraisópolis	2000	90.6%	92.6%	88.2%
Brazil	Paraisópolis	2017	90.9%	92.9%	88.6%
Brazil	Parambu	2000	69.5%	73.9%	64.2%
Brazil	Parambu	2017	70.3%	74.6%	65.1%
Brazil	Paramirim	2000	77.8%	83.2%	71.7%
Brazil	Paramirim	2017	78.4%	83.7%	72.5%
Brazil	Paramoti	2000	70.7%	74.3%	66.5%
Brazil	Paramoti	2017	71.5%	75.0%	67.4%
Brazil	Paraná	2000	76.5%	80.0%	73.0%
Brazil	Paraná	2000	76.2%	81.3%	70.3%
Brazil	Paraná	2017	76.9%	81.9%	71.2%
Brazil	Paraná	2017	77.2%	80.6%	73.8%
Brazil	Paranacity	2000	86.6%	89.2%	83.6%
Brazil	Paranacity	2017	87.0%	89.5%	84.1%
Brazil	Paranaguá	2000	77.1%	80.8%	72.7%
Brazil	Paranaguá	2017	77.8%	81.4%	73.4%
Brazil	Paranaíba	2000	83.3%	87.8%	78.7%
Brazil	Paranaíba	2017	83.8%	88.2%	79.4%
Brazil	Paranaíta	2000	69.6%	77.0%	61.4%
Brazil	Paranaíta	2017	70.4%	77.7%	62.4%
Brazil	Paranaparema	2000	93.2%	95.0%	90.8%
Brazil	Paranaparema	2017	93.4%	95.2%	91.2%
Brazil	Paranapoema	2000	84.8%	88.1%	81.0%
Brazil	Paranapoema	2017	85.4%	88.6%	81.6%
Brazil	Paranapuã	2000	87.9%	91.3%	84.3%
Brazil	Paranapuã	2017	88.3%	91.6%	84.9%
Brazil	Paranatama	2000	65.2%	68.6%	61.7%
Brazil	Paranatama	2017	66.0%	69.5%	62.6%
Brazil	Paranatinga	2000	63.4%	70.8%	53.9%
Brazil	Paranatinga	2017	64.3%	71.6%	54.9%
Brazil	Paranavaí	2000	85.6%	88.4%	82.3%
Brazil	Paranavaí	2017	86.1%	88.8%	82.9%
Brazil	Paranhos	2000	81.5%	86.8%	75.2%
Brazil	Paranhos	2017	82.1%	87.3%	75.9%
Brazil	Paraopeba	2000	81.5%	85.6%	76.6%
Brazil	Paraopeba	2017	82.1%	86.1%	77.3%
Brazil	Parapuã	2000	90.9%	93.3%	88.1%
Brazil	Parapuã	2017	91.2%	93.5%	88.5%

Country	Second Administrative Unit	Year	Mean	Upper	Lower
Brazil	Parari	2000	67.9%	72.1%	62.7%
Brazil	Parari	2017	68.9%	73.0%	63.7%
Brazil	Parati	2000	83.1%	86.9%	78.4%
Brazil	Parati	2017	83.6%	87.3%	79.0%
Brazil	Paratinga	2000	75.8%	81.7%	67.7%
Brazil	Paratinga	2017	76.5%	82.2%	68.5%
Brazil	Paraú	2000	80.5%	83.5%	77.1%
Brazil	Paraú	2017	81.1%	84.1%	77.8%
Brazil	Parauapebas	2000	40.8%	46.6%	35.9%
Brazil	Parauapebas	2017	41.8%	47.6%	36.8%
Brazil	Paraúna	2000	76.9%	81.6%	71.8%
Brazil	Paraúna	2017	77.6%	82.1%	72.6%
Brazil	Parazinho	2000	85.7%	88.6%	82.1%
Brazil	Parazinho	2017	86.2%	89.0%	82.7%
Brazil	Pardinho	2000	93.5%	95.2%	91.4%
Brazil	Pardinho	2017	93.8%	95.4%	91.7%
Brazil	Pareci Novo	2000	79.7%	82.3%	76.8%
Brazil	Pareci Novo	2017	80.3%	82.9%	77.5%
Brazil	Parecis	2000	29.5%	34.3%	25.0%
Brazil	Parecis	2017	30.4%	35.2%	25.8%
Brazil	Parelhas	2000	75.8%	79.2%	71.7%
Brazil	Parelhas	2017	76.5%	79.8%	72.5%
Brazil	Pariconha	2000	65.5%	69.1%	61.6%
Brazil	Pariconha	2017	66.4%	70.0%	62.5%
Brazil	Parintins	2000	56.2%	61.2%	51.3%
Brazil	Parintins	2017	57.1%	62.1%	52.2%
Brazil	Paripiranga	2000	78.7%	81.5%	76.1%
Brazil	Paripiranga	2017	79.4%	82.1%	76.8%
Brazil	Paripueira	2000	61.2%	64.4%	57.8%
Brazil	Paripueira	2017	62.2%	65.3%	58.8%
Brazil	Pariquera-Açu	2000	87.9%	91.3%	83.9%
Brazil	Pariquera-Açu	2017	88.3%	91.6%	84.4%
Brazil	Parisi	2000	90.2%	93.3%	87.0%
Brazil	Parisi	2017	90.5%	93.6%	87.4%
Brazil	Parnaguá	2000	65.9%	72.7%	59.0%
Brazil	Parnaguá	2017	68.1%	74.6%	61.4%
Brazil	Parnaíba	2000	63.4%	68.1%	58.4%
Brazil	Parnaíba	2017	64.3%	69.0%	59.4%
Brazil	Parnamirim	2000	66.7%	71.2%	61.4%
Brazil	Parnamirim	2000	81.9%	83.8%	79.9%
Brazil	Parnamirim	2017	67.5%	72.0%	62.3%
Brazil	Parnamirim	2017	82.5%	84.3%	80.5%
Brazil	Parnarama	2000	59.8%	64.8%	54.3%
Brazil	Parnarama	2017	60.8%	65.7%	55.3%
Brazil	Parobé	2000	81.3%	83.7%	78.8%
Brazil	Parobé	2017	81.9%	84.2%	79.5%
Brazil	Passa e Fica	2000	78.4%	81.1%	75.9%
Brazil	Passa e Fica	2017	79.1%	81.7%	76.6%
Brazil	Passa Quatro	2000	88.2%	90.6%	85.6%
Brazil	Passa Quatro	2017	88.5%	90.8%	86.0%
Brazil	Passa Sete	2000	84.1%	87.3%	80.4%
Brazil	Passa Sete	2017	84.7%	87.8%	81.1%
Brazil	Passa Tempo	2000	80.8%	84.8%	76.5%
Brazil	Passa Tempo	2017	81.4%	85.3%	77.2%
Brazil	Passa Vinte	2000	82.4%	85.6%	79.3%
Brazil	Passa Vinte	2017	83.0%	86.1%	79.9%
Brazil	Passabém	2000	79.5%	81.8%	77.2%
Brazil	Passabém	2000	70.3%	74.7%	65.6%
Brazil	Passabém	2000	81.0%	84.6%	77.0%
Brazil	Passabém	2017	80.1%	82.4%	77.9%

Country	Second Administrative Unit	Year	Mean	Upper	Lower
Brazil	Passabém	2017	71.1%	75.4%	66.5%
Brazil	Passabém	2017	81.7%	85.1%	77.7%
Brazil	Passagem Franca	2000	62.4%	68.3%	55.5%
Brazil	Passagem Franca	2017	63.3%	69.2%	56.5%
Brazil	Passagem Franca do Piauí	2000	58.3%	62.8%	52.5%
Brazil	Passagem Franca do Piauí	2017	59.2%	63.7%	53.5%
Brazil	Passira	2000	65.0%	68.5%	61.9%
Brazil	Passira	2017	65.8%	69.3%	62.8%
Brazil	Passo de Camaragibe	2000	61.0%	64.2%	57.3%
Brazil	Passo de Camaragibe	2017	61.9%	65.1%	58.2%
Brazil	Passo de Torres	2000	74.4%	79.7%	68.9%
Brazil	Passo de Torres	2017	75.2%	80.4%	69.9%
Brazil	Passo do Sobrado	2000	80.6%	84.6%	76.1%
Brazil	Passo do Sobrado	2017	81.2%	85.1%	76.8%
Brazil	Passo Fundo	2000	82.9%	86.1%	78.9%
Brazil	Passo Fundo	2017	83.5%	86.6%	79.7%
Brazil	Passos	2000	86.5%	90.2%	82.1%
Brazil	Passos	2017	86.9%	90.5%	82.7%
Brazil	Passos Maia	2000	78.3%	82.5%	73.5%
Brazil	Passos Maia	2017	78.9%	83.0%	74.2%
Brazil	Pastos Bons	2000	61.7%	68.1%	54.7%
Brazil	Pastos Bons	2017	62.6%	68.9%	55.6%
Brazil	Patis	2000	81.9%	86.6%	76.0%
Brazil	Patis	2017	82.5%	87.1%	76.7%
Brazil	Pato Bragado	2000	82.3%	86.3%	78.5%
Brazil	Pato Bragado	2017	82.8%	86.8%	79.1%
Brazil	Pato Branco	2000	82.4%	86.2%	78.2%
Brazil	Pato Branco	2017	83.0%	86.6%	78.9%
Brazil	Patos	2000	69.3%	73.4%	65.2%
Brazil	Patos	2017	70.1%	74.2%	66.1%
Brazil	Patos de Minas	2000	81.3%	86.5%	74.2%
Brazil	Patos de Minas	2017	81.8%	87.0%	75.0%
Brazil	Patos do Piauí	2000	60.2%	65.4%	55.0%
Brazil	Patos do Piauí	2017	61.2%	66.3%	56.0%
Brazil	Patrocínio	2000	82.9%	87.2%	77.7%
Brazil	Patrocínio	2017	83.5%	87.7%	78.4%
Brazil	Patrocínio do Muriaé	2000	78.0%	81.5%	74.2%
Brazil	Patrocínio do Muriaé	2017	78.7%	82.1%	74.9%
Brazil	Patrocínio Paulista	2000	88.4%	91.3%	84.4%
Brazil	Patrocínio Paulista	2017	88.8%	91.6%	84.9%
Brazil	Patu	2000	81.2%	84.3%	78.1%
Brazil	Patu	2017	81.8%	84.8%	78.7%
Brazil	Paty do Alferes	2000	81.6%	83.7%	78.6%

Country	Second Administrative Unit	Year	Mean	Upper	Lower
Brazil	Paty do Alferes	2017	81.8%	83.9%	78.9%
Brazil	Pau Brazil	2000	79.6%	84.2%	74.4%
Brazil	Pau Brazil	2017	80.2%	84.7%	75.1%
Brazil	Pau d'Arco	2000	57.5%	63.0%	51.6%
Brazil	Pau d'Arco	2000	44.3%	51.0%	37.5%
Brazil	Pau d'Arco	2017	58.4%	63.9%	52.6%
Brazil	Pau d'Arco	2017	45.3%	51.9%	38.4%
Brazil	Pau dos Ferros	2000	75.8%	79.3%	72.2%
Brazil	Pau dos Ferros	2017	76.5%	79.9%	72.9%
Brazil	Paudalho	2000	67.2%	69.8%	65.0%
Brazil	Paudalho	2017	68.1%	70.7%	66.0%
Brazil	Pauini	2000	56.0%	64.6%	47.9%
Brazil	Pauini	2017	56.9%	65.4%	48.8%
Brazil	Paula Cândido	2000	81.8%	84.9%	78.0%
Brazil	Paula Cândido	2017	82.4%	85.3%	78.6%
Brazil	Paula Freitas	2000	78.8%	82.9%	73.6%
Brazil	Paula Freitas	2017	79.4%	83.5%	74.3%
Brazil	Paulicéia	2000	85.3%	88.3%	81.2%
Brazil	Paulicéia	2017	85.8%	88.7%	81.8%
Brazil	Paulínia	2000	92.9%	94.1%	91.4%
Brazil	Paulínia	2017	93.2%	94.4%	91.8%
Brazil	Paulino Neves	2000	60.3%	66.2%	54.4%
Brazil	Paulino Neves	2017	61.2%	67.0%	55.4%
Brazil	Paulista	2000	66.1%	68.2%	63.9%
Brazil	Paulista	2000	73.9%	77.4%	70.0%
Brazil	Paulista	2017	67.0%	69.0%	64.8%
Brazil	Paulista	2017	74.7%	78.1%	70.8%
Brazil	Paulistana	2000	64.5%	69.5%	58.3%
Brazil	Paulistana	2017	65.3%	70.3%	59.2%
Brazil	Paulistânia	2000	92.8%	94.8%	90.7%
Brazil	Paulistânia	2017	93.1%	95.0%	91.0%
Brazil	Paulistas	2000	81.7%	86.0%	75.9%
Brazil	Paulistas	2017	82.3%	86.4%	76.6%
Brazil	Paulo Afonso	2000	66.0%	70.8%	61.0%
Brazil	Paulo Afonso	2017	66.9%	71.6%	62.0%
Brazil	Paulo de Faria	2000	88.7%	91.3%	84.7%
Brazil	Paulo de Faria	2017	89.1%	91.6%	85.2%
Brazil	Paulo Frontin	2000	79.2%	83.4%	74.0%
Brazil	Paulo Frontin	2017	79.8%	83.9%	74.8%
Brazil	Paulo Jacinto	2000	58.3%	61.6%	55.5%
Brazil	Paulo Jacinto	2017	59.3%	62.5%	56.4%
Brazil	Paulo Lopez	2000	71.4%	76.8%	64.7%
Brazil	Paulo Lopez	2017	72.2%	77.5%	65.7%
Brazil	Paulo Ramos	2000	60.7%	65.8%	55.6%
Brazil	Paulo Ramos	2017	61.6%	66.7%	56.6%
Brazil	Pavão	2000	80.8%	85.4%	75.4%
Brazil	Pavão	2017	81.4%	85.8%	76.2%
Brazil	Paverama	2000	81.0%	84.1%	77.8%
Brazil	Paverama	2017	81.6%	84.7%	78.5%
Brazil	Pavussu	2000	62.1%	67.8%	55.5%
Brazil	Pavussu	2017	63.0%	68.6%	56.5%
Brazil	Pé de Serra	2000	80.4%	84.0%	75.9%
Brazil	Pé de Serra	2017	81.1%	84.6%	76.7%
Brazil	Peabiru	2000	84.2%	87.3%	81.1%
Brazil	Peabiru	2017	84.7%	87.7%	81.7%
Brazil	Peçanha	2000	82.8%	86.9%	77.9%
Brazil	Peçanha	2017	83.3%	87.3%	78.5%

Country	Second Administrative Unit	Year	Mean	Upper	Lower
Brazil	Pederneiras	2000	93.6%	95.3%	91.6%
Brazil	Pederneiras	2017	93.8%	95.5%	91.9%
Brazil	Pedra	2000	66.8%	70.5%	63.1%
Brazil	Pedra	2017	67.7%	71.4%	64.0%
Brazil	Pedra Azul	2000	80.7%	85.1%	74.7%
Brazil	Pedra Azul	2017	81.3%	85.6%	75.4%
Brazil	Pedra Bela	2000	92.6%	94.1%	91.0%
Brazil	Pedra Bela	2017	92.9%	94.3%	91.3%
Brazil	Pedra Bonita	2000	82.8%	86.1%	78.8%
Brazil	Pedra Bonita	2017	83.3%	86.6%	79.4%
Brazil	Pedra Branca	2000	66.6%	70.8%	62.2%
Brazil	Pedra Branca	2000	71.0%	75.2%	66.3%
Brazil	Pedra Branca	2017	71.8%	76.0%	67.2%
Brazil	Pedra Branca	2017	67.5%	71.6%	63.1%
Brazil	Pedra Branca do Amaparí	2000	47.7%	52.9%	42.6%
Brazil	Pedra Branca do Amaparí	2017	48.7%	54.0%	43.5%
Brazil	Pedra do Anta	2000	81.1%	84.7%	77.0%
Brazil	Pedra do Anta	2017	81.7%	85.2%	77.7%
Brazil	Pedra do Indaiá	2000	81.4%	85.4%	76.8%
Brazil	Pedra do Indaiá	2017	82.0%	85.9%	77.5%
Brazil	Pedra dourada	2000	81.2%	84.7%	77.6%
Brazil	Pedra dourada	2017	81.8%	85.1%	78.2%
Brazil	Pedra Grande	2000	85.0%	88.3%	80.3%
Brazil	Pedra Grande	2017	85.5%	88.7%	81.0%
Brazil	Pedra Lavadra	2000	75.3%	78.7%	71.1%
Brazil	Pedra Lavadra	2017	76.0%	79.3%	71.9%
Brazil	Pedra Mole	2000	76.7%	79.6%	73.9%
Brazil	Pedra Mole	2017	77.4%	80.2%	74.6%
Brazil	Pedra Preta	2000	69.2%	73.9%	64.4%
Brazil	Pedra Preta	2000	84.9%	87.6%	82.2%
Brazil	Pedra Preta	2017	85.4%	88.0%	82.8%
Brazil	Pedra Preta	2017	70.0%	74.6%	65.3%
Brazil	Pedralva	2000	87.5%	90.5%	84.0%
Brazil	Pedralva	2017	88.0%	90.8%	84.5%
Brazil	Pedranópolis	2000	89.6%	92.7%	86.3%
Brazil	Pedranópolis	2017	90.0%	92.9%	86.8%
Brazil	Pedrão	2000	77.5%	80.6%	74.1%
Brazil	Pedrão	2017	78.2%	81.3%	74.9%
Brazil	Pedras de Fogo	2000	67.6%	70.4%	64.8%
Brazil	Pedras de Fogo	2017	68.4%	71.2%	65.7%
Brazil	Pedras de Maria da Cruz	2000	80.8%	86.2%	74.2%
Brazil	Pedras de Maria da Cruz	2017	81.4%	86.7%	75.0%
Brazil	Pedras Grandes	2000	74.0%	77.9%	70.2%
Brazil	Pedras Grandes	2017	74.8%	78.6%	71.0%
Brazil	Pedregulho	2000	88.7%	91.9%	84.3%
Brazil	Pedregulho	2017	89.0%	92.2%	84.8%

Country	Second Administrative Unit	Year	Mean	Upper	Lower
Brazil	Pedreira	2000	92.6%	93.9%	90.9%
Brazil	Pedreira	2017	92.8%	94.1%	91.2%
Brazil	Pedreiras	2000	60.0%	65.4%	54.9%
Brazil	Pedreiras	2017	60.9%	66.3%	55.9%
Brazil	Pedrinhas	2000	77.5%	80.3%	75.0%
Brazil	Pedrinhas	2017	78.2%	80.9%	75.7%
Brazil	Pedrinhas Paulista	2000	87.9%	90.2%	85.2%
Brazil	Pedrinhas Paulista	2017	88.4%	90.6%	85.7%
Brazil	Pedrinópolis	2000	82.5%	86.5%	77.6%
Brazil	Pedrinópolis	2017	83.0%	86.9%	78.2%
Brazil	Pedro Afonso	2000	73.8%	78.3%	68.7%
Brazil	Pedro Afonso	2017	74.6%	79.0%	69.5%
Brazil	Pedro Alexandre	2000	73.4%	76.9%	69.4%
Brazil	Pedro Alexandre	2017	74.1%	77.5%	70.2%
Brazil	Pedro Avelino	2000	83.7%	86.6%	80.4%
Brazil	Pedro Avelino	2017	84.8%	87.5%	81.7%
Brazil	Pedro Canário	2000	76.2%	80.9%	71.0%
Brazil	Pedro Canário	2017	76.9%	81.5%	71.8%
Brazil	Pedro de Toledo	2000	90.9%	93.1%	88.4%
Brazil	Pedro de Toledo	2017	91.2%	93.3%	88.8%
Brazil	Pedro do Rosário	2000	60.3%	65.5%	55.3%
Brazil	Pedro do Rosário	2017	61.2%	66.3%	56.2%
Brazil	Pedro Gomes	2000	77.1%	82.8%	71.7%
Brazil	Pedro Gomes	2017	77.8%	83.3%	72.5%
Brazil	Pedro Laurentino	2000	61.4%	66.7%	55.0%
Brazil	Pedro Laurentino	2017	62.3%	67.6%	56.0%
Brazil	Pedro Leopoldo	2000	81.3%	84.1%	78.4%
Brazil	Pedro Leopoldo	2017	82.0%	84.6%	79.1%
Brazil	Pedro Li	2000	66.9%	71.3%	62.2%
Brazil	Pedro Li	2017	67.8%	72.1%	63.1%
Brazil	Pedro Osório	2000	80.6%	85.2%	74.9%
Brazil	Pedro Osório	2017	81.2%	85.6%	75.6%
Brazil	Pedro Régis	2000	74.1%	76.8%	71.2%
Brazil	Pedro Régis	2017	74.8%	77.5%	72.0%
Brazil	Pedro Teixeira	2000	80.6%	83.8%	76.6%
Brazil	Pedro Teixeira	2017	81.2%	84.3%	77.3%
Brazil	Pedro Velho	2000	75.7%	78.4%	73.1%
Brazil	Pedro Velho	2017	76.4%	79.1%	73.8%
Brazil	Peixe	2000	75.6%	79.7%	70.5%
Brazil	Peixe	2017	76.3%	80.3%	71.4%
Brazil	Peixe Boi	2000	37.2%	42.0%	33.6%
Brazil	Peixe Boi	2017	38.2%	43.0%	34.5%
Brazil	Peixoto de Azevedo	2000	62.9%	70.0%	55.3%
Brazil	Peixoto de Azevedo	2017	63.8%	70.8%	56.3%
Brazil	Pejuçara	2000	82.9%	86.8%	78.7%
Brazil	Pejuçara	2017	83.5%	87.2%	79.3%
Brazil	Pelotas	2000	82.6%	86.0%	77.6%
Brazil	Pelotas	2017	83.1%	86.5%	78.2%

Country	Second Administrative Unit	Year	Mean	Upper	Lower
Brazil	Penaforte	2000	67.5%	71.5%	62.6%
Brazil	Penaforte	2017	68.4%	72.3%	63.5%
Brazil	Penalva	2000	61.0%	65.0%	56.2%
Brazil	Penalva	2017	61.9%	65.8%	57.2%
Brazil	Penápolis	2000	92.8%	94.7%	90.4%
Brazil	Penápolis	2017	93.0%	94.9%	90.8%
Brazil	Pendências	2000	83.3%	86.6%	80.0%
Brazil	Pendências	2017	83.8%	87.1%	80.6%
Brazil	Penedo	2000	70.2%	72.9%	67.5%
Brazil	Penedo	2017	70.9%	73.5%	68.2%
Brazil	Penha	2000	71.4%	75.4%	66.6%
Brazil	Penha	2017	72.2%	76.1%	67.5%
Brazil	Pentecoste	2000	71.2%	74.4%	67.8%
Brazil	Pentecoste	2017	72.0%	75.1%	68.6%
Brazil	Pequeri	2000	79.4%	82.6%	75.9%
Brazil	Pequeri	2017	80.1%	83.2%	76.6%
Brazil	Pequi	2000	80.7%	84.4%	76.1%
Brazil	Pequi	2017	81.3%	84.9%	76.9%
Brazil	Pequizeiro	2000	70.5%	75.2%	65.9%
Brazil	Pequizeiro	2017	71.4%	75.9%	66.7%
Brazil	Perdigão	2000	81.2%	85.0%	76.5%
Brazil	Perdigão	2017	81.8%	85.6%	77.3%
Brazil	Perdizes	2000	82.6%	86.9%	77.0%
Brazil	Perdizes	2017	83.2%	87.3%	77.8%
Brazil	Perdões	2000	81.4%	85.2%	76.6%
Brazil	Perdões	2017	82.0%	85.7%	77.3%
Brazil	Pereira Barreto	2000	88.8%	91.6%	85.5%
Brazil	Pereira Barreto	2017	89.2%	91.9%	86.0%
Brazil	Pereiras	2000	93.2%	94.6%	91.5%
Brazil	Pereiras	2017	93.5%	94.9%	91.8%
Brazil	Pereiro	2000	74.7%	78.9%	70.5%
Brazil	Pereiro	2017	75.5%	79.5%	71.4%
Brazil	Peri-Mirim	2000	60.6%	65.6%	55.6%
Brazil	Peri-Mirim	2017	61.5%	66.4%	56.6%
Brazil	Periquito	2000	78.6%	83.3%	73.5%
Brazil	Periquito	2017	79.2%	83.8%	74.3%
Brazil	Peritiba	2000	76.7%	80.3%	71.9%
Brazil	Peritiba	2017	77.4%	80.9%	72.6%
Brazil	Peritoró	2000	60.9%	65.6%	55.2%
Brazil	Peritoró	2017	61.8%	66.5%	56.1%
Brazil	Perobal	2000	83.3%	87.0%	79.7%
Brazil	Perobal	2017	83.8%	87.4%	80.3%
Brazil	Pérola	2000	83.6%	86.7%	79.8%
Brazil	Pérola	2017	84.2%	87.2%	80.4%
Brazil	Pérola d'Oeste	2000	82.7%	86.5%	78.8%
Brazil	Pérola d'Oeste	2017	83.2%	86.9%	79.4%
Brazil	Perolândia	2000	77.7%	83.0%	71.6%
Brazil	Perolândia	2017	78.3%	83.6%	72.4%
Brazil	Peruíbe	2000	91.1%	93.2%	88.3%
Brazil	Peruíbe	2017	91.3%	93.4%	88.7%
Brazil	Pescador	2000	81.4%	86.3%	76.3%
Brazil	Pescador	2017	82.0%	86.8%	77.0%
Brazil	Pesqueira	2000	66.0%	69.8%	61.7%
Brazil	Pesqueira	2017	66.9%	70.7%	62.6%
Brazil	Petrolândia	2000	64.8%	69.9%	59.7%
Brazil	Petrolândia	2000	74.2%	78.8%	69.3%
Brazil	Petrolândia	2017	74.9%	79.4%	70.1%
Brazil	Petrolândia	2017	67.5%	72.4%	62.6%
Brazil	Petrolina	2000	72.1%	76.5%	67.2%
Brazil	Petrolina	2017	73.3%	77.5%	68.5%

Country	Second Administrative Unit	Year	Mean	Upper	Lower
Brazil	Petrolina de Goiás	2000	77.8%	81.0%	74.6%
Brazil	Petrolina de Goiás	2017	78.5%	81.6%	75.3%
Brazil	Petrópolis	2000	82.2%	83.9%	80.0%
Brazil	Petrópolis	2017	82.8%	84.4%	80.6%
Brazil	Piaçabuçu	2000	64.2%	67.9%	60.4%
Brazil	Piaçabuçu	2017	65.1%	68.7%	61.3%
Brazil	Piacatu	2000	91.3%	93.6%	88.8%
Brazil	Piacatu	2017	91.6%	93.8%	89.2%
Brazil	Piancó	2000	65.6%	69.2%	61.2%
Brazil	Piancó	2017	66.5%	70.0%	62.1%
Brazil	Piatã	2000	80.3%	85.2%	75.2%
Brazil	Piatã	2017	80.9%	85.7%	76.0%
Brazil	Piau	2000	80.0%	83.1%	76.0%
Brazil	Piau	2017	80.6%	83.6%	76.7%
Brazil	Picada Café	2000	80.4%	83.3%	77.4%
Brazil	Picada Café	2017	81.0%	83.8%	78.2%
Brazil	Piçarra	2000	57.5%	62.4%	52.5%
Brazil	Piçarra	2017	58.5%	63.4%	53.5%
Brazil	Piçarras	2000	73.4%	77.1%	68.7%
Brazil	Piçarras	2017	74.8%	78.3%	70.2%
Brazil	Picos	2000	61.8%	66.4%	57.3%
Brazil	Picos	2017	63.1%	67.7%	58.7%
Brazil	Picuí	2000	78.8%	81.9%	75.0%
Brazil	Picuí	2017	79.5%	82.4%	75.7%
Brazil	Piedade	2000	94.3%	95.7%	93.1%
Brazil	Piedade	2017	94.5%	95.8%	93.2%
Brazil	Piedade de Caratinga	2000	82.6%	86.0%	78.6%
Brazil	Piedade de Caratinga	2017	84.3%	87.4%	80.5%
Brazil	Piedade do Ponte Nova	2000	80.7%	84.4%	76.4%
Brazil	Piedade do Ponte Nova	2017	81.3%	84.9%	77.1%
Brazil	Piedade do Rio Grande	2000	82.3%	86.1%	77.8%
Brazil	Piedade do Rio Grande	2017	82.8%	86.6%	78.4%
Brazil	Piedade dos Gerais	2000	80.5%	84.2%	76.4%
Brazil	Piedade dos Gerais	2017	81.1%	84.8%	77.1%
Brazil	Piên	2000	80.7%	83.6%	77.4%
Brazil	Piên	2017	81.3%	84.1%	78.1%
Brazil	Pilão Arcado	2000	69.4%	75.5%	60.9%
Brazil	Pilão Arcado	2017	70.2%	76.2%	61.8%
Brazil	Pilar	2000	67.2%	70.4%	64.1%
Brazil	Pilar	2000	57.2%	59.8%	54.6%
Brazil	Pilar	2017	58.1%	60.7%	55.5%
Brazil	Pilar	2017	68.0%	71.2%	64.9%
Brazil	Pilar de Goiás	2000	77.8%	82.1%	72.4%
Brazil	Pilar de Goiás	2017	78.5%	82.7%	73.2%
Brazil	Pilar do Sul	2000	93.8%	95.6%	92.0%
Brazil	Pilar do Sul	2017	94.1%	95.7%	92.3%
Brazil	Pilões	2000	76.1%	79.5%	72.4%
Brazil	Pilões	2000	67.0%	70.0%	63.7%
Brazil	Pilões	2017	76.8%	80.1%	73.2%
Brazil	Pilões	2017	67.8%	70.8%	64.6%
Brazil	Pilõezinhos	2000	67.9%	70.8%	64.8%
Brazil	Pilõezinhos	2017	68.7%	71.6%	65.7%

Country	Second Administrative Unit	Year	Mean	Upper	Lower
Brazil	Pimenta	2000	81.6%	86.2%	76.6%
Brazil	Pimenta	2017	83.4%	87.6%	78.8%
Brazil	Pimenta Bueno	2000	28.9%	33.3%	24.9%
Brazil	Pimenta Bueno	2017	29.7%	34.2%	25.7%
Brazil	Pimenteiras	2000	63.9%	69.1%	58.7%
Brazil	Pimenteiras	2017	64.8%	70.0%	59.6%
Brazil	Pimenteiras do Oeste	2000	30.5%	36.5%	25.3%
Brazil	Pimenteiras do Oeste	2017	31.3%	37.5%	26.0%
Brazil	Pindaí	2000	78.9%	84.0%	73.0%
Brazil	Pindaí	2017	79.5%	84.5%	73.8%
Brazil	Pindamonhangaba	2000	91.3%	93.1%	89.0%
Brazil	Pindamonhangaba	2017	91.6%	93.3%	89.3%
Brazil	Pindaré-Mirim	2000	60.3%	64.6%	55.5%
Brazil	Pindaré-Mirim	2017	61.2%	65.5%	56.5%
Brazil	Pindoba	2000	57.9%	61.3%	54.9%
Brazil	Pindoba	2017	58.9%	62.2%	55.8%
Brazil	Pindobaçu	2000	76.0%	81.1%	68.8%
Brazil	Pindobaçu	2017	76.7%	81.7%	69.7%
Brazil	Pindorama	2000	93.5%	95.1%	91.7%
Brazil	Pindorama	2017	93.8%	95.4%	92.1%
Brazil	Pindorama do Tocantins	2000	76.6%	81.9%	71.0%
Brazil	Pindorama do Tocantins	2017	77.4%	82.5%	71.7%
Brazil	Pindoretama	2000	68.4%	71.3%	65.4%
Brazil	Pindoretama	2017	69.3%	72.1%	66.3%
Brazil	Pingo d'Água	2000	80.1%	83.7%	75.8%
Brazil	Pingo d'Água	2017	80.7%	84.2%	76.5%
Brazil	Pinhais	2000	86.4%	88.3%	84.4%
Brazil	Pinhais	2017	86.8%	88.7%	84.9%
Brazil	Pinhal	2000	89.9%	92.1%	87.4%
Brazil	Pinhal	2000	78.3%	82.1%	74.1%
Brazil	Pinhal	2017	90.2%	92.4%	87.9%
Brazil	Pinhal	2017	79.0%	82.7%	74.8%
Brazil	Pinhal de São Bento	2000	81.8%	85.8%	77.3%
Brazil	Pinhal de São Bento	2017	82.4%	86.3%	78.0%
Brazil	Pinhal Grande	2000	83.1%	86.2%	78.8%
Brazil	Pinhal Grande	2017	83.6%	86.7%	79.5%
Brazil	Pinhalão	2000	86.9%	89.8%	83.3%
Brazil	Pinhalão	2017	87.3%	90.1%	83.8%
Brazil	Pinhalzinho	2000	79.5%	83.3%	75.0%
Brazil	Pinhalzinho	2000	91.9%	93.5%	90.3%
Brazil	Pinhalzinho	2017	92.2%	93.7%	90.6%
Brazil	Pinhalzinho	2017	80.2%	83.9%	75.8%
Brazil	Pinhão	2000	77.7%	80.6%	74.8%
Brazil	Pinhão	2000	81.6%	85.3%	77.1%
Brazil	Pinhão	2017	82.2%	85.8%	77.8%
Brazil	Pinhão	2017	78.4%	81.2%	75.5%
Brazil	Pinheiral	2000	79.9%	82.5%	76.9%
Brazil	Pinheiral	2017	80.5%	83.1%	77.6%
Brazil	Pinheirinho do Vale	2000	77.9%	82.0%	72.8%

Country	Second Administrative Unit	Year	Mean	Upper	Lower
Brazil	Pinheirinho do Vale	2017	78.5%	82.5%	73.5%
Brazil	Pinheiro	2000	60.2%	65.1%	55.2%
Brazil	Pinheiro	2017	61.3%	66.2%	56.4%
Brazil	Pinheiro Machado	2000	82.9%	87.5%	77.6%
Brazil	Pinheiro Machado	2017	83.4%	87.9%	78.3%
Brazil	Pinheiro Preto	2000	75.8%	80.2%	71.7%
Brazil	Pinheiro Preto	2017	76.5%	80.8%	72.5%
Brazil	Pinheiros	2000	77.4%	82.1%	73.3%
Brazil	Pinheiros	2017	78.6%	83.0%	74.6%
Brazil	Pintadas	2000	79.0%	83.2%	74.6%
Brazil	Pintadas	2017	79.6%	83.7%	75.3%
Brazil	Pintópolis	2000	82.3%	87.6%	75.7%
Brazil	Pintópolis	2017	82.8%	88.0%	76.4%
Brazil	Pio IX	2000	66.9%	71.3%	62.3%
Brazil	Pio IX	2017	67.7%	72.0%	63.2%
Brazil	Pio XII	2000	60.4%	65.3%	55.0%
Brazil	Pio XII	2017	61.3%	66.2%	55.8%
Brazil	Piquerobi	2000	87.8%	90.4%	84.7%
Brazil	Piquerobi	2017	88.3%	90.8%	85.2%
Brazil	Piquet Carneiro	2000	69.9%	74.6%	65.7%
Brazil	Piquet Carneiro	2017	70.7%	75.3%	66.6%
Brazil	Piquete	2000	87.5%	89.9%	84.7%
Brazil	Piquete	2017	87.9%	90.3%	85.2%
Brazil	Piracaia	2000	93.3%	94.4%	91.9%
Brazil	Piracaia	2017	93.5%	94.6%	92.2%
Brazil	Piracanjuba	2000	77.9%	81.1%	74.4%
Brazil	Piracanjuba	2017	78.6%	81.7%	75.1%
Brazil	Piracema	2000	80.4%	84.3%	75.8%
Brazil	Piracema	2017	81.0%	84.8%	76.5%
Brazil	Piracicaba	2000	93.4%	94.7%	91.9%
Brazil	Piracicaba	2017	93.7%	94.9%	92.2%
Brazil	Piracununga	2000	92.6%	94.0%	90.6%
Brazil	Piracununga	2017	92.8%	94.2%	90.9%
Brazil	Piracuruca	2000	61.0%	65.5%	56.3%
Brazil	Piracuruca	2017	61.9%	66.4%	57.3%
Brazil	Piraí	2000	78.2%	80.8%	75.3%
Brazil	Piraí	2017	78.8%	81.4%	76.0%
Brazil	Piraí do Norte	2000	75.5%	80.0%	70.5%
Brazil	Piraí do Norte	2017	76.3%	80.7%	71.3%
Brazil	Piraí do Sul	2000	87.6%	90.3%	84.8%
Brazil	Piraí do Sul	2017	88.0%	90.7%	85.3%
Brazil	Pirajuba	2000	87.8%	90.8%	83.8%
Brazil	Pirajuba	2017	88.2%	91.1%	84.3%
Brazil	Pirajui	2000	92.1%	93.8%	89.8%
Brazil	Pirajui	2017	92.4%	94.1%	90.3%
Brazil	Pirajuí	2000	93.1%	95.0%	90.8%
Brazil	Pirajuí	2017	93.4%	95.2%	91.1%
Brazil	Pirambu	2000	71.3%	74.0%	68.0%
Brazil	Pirambu	2017	72.1%	74.7%	68.8%
Brazil	Piranga	2000	80.6%	84.0%	76.7%
Brazil	Piranga	2017	81.7%	85.0%	77.9%
Brazil	Pirangi	2000	93.0%	94.6%	91.0%
Brazil	Pirangi	2017	93.2%	94.8%	91.4%
Brazil	Piranguçu	2000	89.2%	91.6%	86.4%
Brazil	Piranguçu	2017	89.5%	91.8%	86.8%

Country	Second Administrative Unit	Year	Mean	Upper	Lower
Brazil	Piranguinho	2000	87.8%	90.6%	84.7%
Brazil	Piranguinho	2017	88.2%	91.0%	85.2%
Brazil	Piranhas	2000	66.3%	70.0%	62.3%
Brazil	Piranhas	2000	74.3%	79.8%	68.2%
Brazil	Piranhas	2017	75.0%	80.4%	69.0%
Brazil	Piranhas	2017	67.2%	70.9%	63.3%
Brazil	Pirapemas	2000	61.4%	66.4%	56.3%
Brazil	Pirapemas	2017	62.3%	67.2%	57.2%
Brazil	Pirapetinga	2000	77.6%	81.0%	73.5%
Brazil	Pirapetinga	2017	78.2%	81.6%	74.2%
Brazil	Pirapó	2000	80.5%	86.1%	74.0%
Brazil	Pirapó	2017	81.1%	86.5%	74.7%
Brazil	Pirapora	2000	82.3%	87.4%	75.9%
Brazil	Pirapora	2017	82.8%	87.8%	76.6%
Brazil	Pirapora do Bom Jesus	2000	93.1%	94.1%	91.9%
Brazil	Pirapora do Bom Jesus	2017	93.3%	94.3%	92.1%
Brazil	Pirapozinho	2000	88.5%	91.4%	85.6%
Brazil	Pirapozinho	2017	88.9%	91.7%	86.0%
Brazil	Piraquara	2000	85.8%	87.9%	83.6%
Brazil	Piraquara	2017	86.2%	88.3%	84.1%
Brazil	Piraquê	2000	71.6%	75.1%	67.3%
Brazil	Piraquê	2017	72.3%	75.8%	68.2%
Brazil	Piratini	2000	82.4%	86.4%	77.7%
Brazil	Piratini	2017	83.0%	86.8%	78.4%
Brazil	Piratininga	2000	93.2%	95.2%	90.9%
Brazil	Piratininga	2017	93.5%	95.3%	91.3%
Brazil	Piratuba	2000	77.1%	80.6%	72.3%
Brazil	Piratuba	2017	77.8%	81.2%	73.1%
Brazil	Pirauba	2000	81.0%	84.2%	77.4%
Brazil	Pirauba	2017	81.6%	84.7%	78.0%
Brazil	Pirenópolis	2000	80.2%	83.0%	76.9%
Brazil	Pirenópolis	2017	80.9%	83.6%	77.6%
Brazil	Pires do Rio	2000	79.8%	83.7%	74.9%
Brazil	Pires do Rio	2017	80.4%	84.2%	75.7%
Brazil	Pires Ferreira	2000	65.1%	69.4%	60.8%
Brazil	Pires Ferreira	2017	65.9%	70.1%	61.7%
Brazil	Piripá	2000	79.0%	84.0%	73.1%
Brazil	Piripá	2017	79.7%	84.6%	73.9%
Brazil	Piripiri	2000	62.7%	67.5%	57.9%
Brazil	Piripiri	2017	63.6%	68.3%	58.8%
Brazil	Piritiba	2000	79.0%	83.6%	74.5%
Brazil	Piritiba	2017	79.7%	84.1%	75.3%
Brazil	Pirpirituba	2000	70.7%	73.3%	67.8%
Brazil	Pirpirituba	2017	71.6%	74.1%	68.7%
Brazil	Pitanga	2000	84.8%	88.1%	80.7%
Brazil	Pitanga	2017	85.3%	88.6%	81.3%
Brazil	Pitangueiras	2000	86.0%	88.4%	83.1%
Brazil	Pitangueiras	2000	92.8%	94.5%	90.7%
Brazil	Pitangueiras	2017	86.4%	88.7%	83.6%
Brazil	Pitangueiras	2017	93.1%	94.7%	91.0%
Brazil	Pitangui	2000	81.5%	85.7%	77.3%
Brazil	Pitangui	2017	82.1%	86.1%	78.0%
Brazil	Pitimbu	2000	64.5%	67.8%	61.1%
Brazil	Pitimbu	2017	65.4%	68.7%	62.1%
Brazil	Piui	2000	82.9%	87.1%	77.7%
Brazil	Piui	2017	83.5%	87.6%	78.4%
Brazil	Pium	2000	68.3%	73.0%	62.8%
Brazil	Pium	2017	68.1%	72.7%	62.5%
Brazil	Piúma	2000	77.3%	80.4%	73.1%
Brazil	Piúma	2017	78.0%	81.1%	73.9%

Country	Second Administrative Unit	Year	Mean	Upper	Lower
Brazil	Placas	2000	38.7%	44.8%	32.2%
Brazil	Placas	2017	39.6%	45.7%	32.9%
Brazil	Plácido de Castro	2000	39.3%	43.6%	34.5%
Brazil	Plácido de Castro	2017	40.2%	44.6%	35.4%
Brazil	Planaltina	2000	91.8%	93.3%	90.0%
Brazil	Planaltina	2017	92.1%	93.5%	90.4%
Brazil	Planaltina do Paraná	2000	83.8%	87.2%	80.3%
Brazil	Planaltina do Paraná	2017	84.3%	87.6%	80.9%
Brazil	Planaltino	2000	79.8%	84.5%	74.2%
Brazil	Planaltino	2017	80.4%	85.0%	75.0%
Brazil	Planalto	2000	83.2%	86.7%	79.4%
Brazil	Planalto	2000	79.2%	82.6%	75.2%
Brazil	Planalto	2000	83.1%	87.2%	78.6%
Brazil	Planalto	2000	92.2%	94.4%	89.6%
Brazil	Planalto	2017	83.7%	87.1%	80.0%
Brazil	Planalto	2017	83.7%	87.7%	79.3%
Brazil	Planalto	2017	92.4%	94.6%	90.0%
Brazil	Planalto	2017	79.8%	83.2%	75.9%
Brazil	Planalto Alegre	2000	77.7%	81.2%	73.1%
Brazil	Planalto Alegre	2017	78.3%	81.8%	73.9%
Brazil	Planalto da Serra	2000	68.1%	75.4%	60.1%
Brazil	Planalto da Serra	2017	68.9%	76.1%	61.0%
Brazil	Planura	2000	89.2%	92.2%	86.0%
Brazil	Planura	2017	89.6%	92.5%	86.4%
Brazil	Platina	2000	90.7%	93.0%	88.1%
Brazil	Platina	2017	91.1%	93.3%	88.5%
Brazil	Poá	2000	94.6%	95.4%	93.8%
Brazil	Poá	2017	94.8%	95.5%	94.0%
Brazil	Poção	2000	67.3%	71.1%	63.2%
Brazil	Poção	2017	68.2%	71.9%	64.1%
Brazil	Poção de Pedras	2000	60.8%	66.0%	55.6%
Brazil	Poção de Pedras	2017	61.7%	66.9%	56.6%
Brazil	Pocinhos	2000	72.7%	75.9%	69.1%
Brazil	Pocinhos	2017	73.5%	76.6%	69.9%
Brazil	Poço Branco	2000	84.2%	86.6%	81.6%
Brazil	Poço Branco	2017	84.7%	87.1%	82.2%
Brazil	Poço Dantas	2000	75.1%	79.3%	71.1%
Brazil	Poço Dantas	2017	75.8%	80.0%	71.8%
Brazil	Poço das Antas	2000	81.6%	84.3%	78.4%
Brazil	Poço das Antas	2017	82.1%	84.7%	78.9%
Brazil	Poço das Trincheiras	2000	61.3%	64.2%	57.9%
Brazil	Poço das Trincheiras	2017	62.2%	65.1%	58.9%
Brazil	Poço de José de Moura	2000	70.0%	74.0%	66.2%
Brazil	Poço de José de Moura	2017	70.9%	74.7%	67.1%
Brazil	Poço Fundo	2000	85.9%	88.8%	81.7%
Brazil	Poço Fundo	2017	86.4%	89.1%	82.3%

Country	Second Administrative Unit	Year	Mean	Upper	Lower
Brazil	Poço Redondo	2000	69.1%	72.6%	65.0%
Brazil	Poço Redondo	2017	69.9%	73.3%	65.8%
Brazil	Poço Verde	2000	79.6%	82.7%	76.1%
Brazil	Poço Verde	2017	80.3%	83.3%	76.9%
Brazil	Poções	2000	83.6%	87.4%	79.6%
Brazil	Poções	2017	84.1%	87.8%	80.2%
Brazil	Poconé	2000	69.2%	75.0%	63.3%
Brazil	Poconé	2017	70.1%	75.7%	64.2%
Brazil	Poços de Caldas	2000	91.5%	93.4%	88.9%
Brazil	Poços de Caldas	2017	91.8%	93.7%	89.2%
Brazil	Pocrane	2000	78.2%	82.5%	74.1%
Brazil	Pocrane	2017	78.9%	83.0%	74.8%
Brazil	Pojuca	2000	76.2%	79.8%	72.4%
Brazil	Pojuca	2017	76.9%	80.4%	73.1%
Brazil	Poloni	2000	91.9%	94.2%	89.0%
Brazil	Poloni	2017	92.2%	94.4%	89.4%
Brazil	Pombal	2000	71.3%	74.3%	68.4%
Brazil	Pombal	2017	72.3%	75.2%	69.3%
Brazil	Pombos	2000	69.8%	72.9%	67.0%
Brazil	Pombos	2017	70.7%	73.7%	67.9%
Brazil	Pomerode	2000	72.5%	76.0%	69.1%
Brazil	Pomerode	2017	73.2%	76.7%	69.9%
Brazil	Pompéia	2000	92.9%	94.9%	90.6%
Brazil	Pompéia	2017	93.2%	95.1%	90.9%
Brazil	Pompéu	2000	81.7%	86.3%	76.4%
Brazil	Pompéu	2017	82.3%	86.8%	77.0%
Brazil	Pongaí	2000	93.1%	95.1%	90.5%
Brazil	Pongaí	2017	93.3%	95.3%	90.9%
Brazil	Ponta Alta	2000	75.6%	80.3%	70.4%
Brazil	Ponta Alta	2017	76.3%	80.9%	71.2%
Brazil	Ponta de Pedras	2000	36.2%	40.8%	32.4%
Brazil	Ponta de Pedras	2017	37.1%	41.7%	33.3%
Brazil	Ponta Grossa	2000	85.1%	87.6%	81.9%
Brazil	Ponta Grossa	2017	85.7%	88.1%	82.5%
Brazil	Ponta Porã	2000	83.8%	87.3%	79.6%
Brazil	Ponta Porã	2017	84.4%	87.7%	80.2%
Brazil	Pontal	2000	94.0%	95.5%	92.0%
Brazil	Pontal	2017	94.3%	95.7%	92.3%
Brazil	Pontal do Araguaia	2000	72.3%	78.2%	66.2%
Brazil	Pontal do Araguaia	2017	73.0%	78.8%	67.1%
Brazil	Pontal do Paraná	2000	74.7%	78.9%	69.8%
Brazil	Pontal do Paraná	2017	75.4%	79.6%	70.6%
Brazil	Pontalina	2000	77.8%	81.1%	74.0%
Brazil	Pontalina	2017	78.4%	81.7%	74.7%
Brazil	Pontalinda	2000	89.1%	92.3%	85.5%
Brazil	Pontalinda	2017	89.5%	92.5%	86.0%
Brazil	Pontão	2000	80.9%	84.3%	76.9%
Brazil	Pontão	2017	81.5%	84.8%	77.6%
Brazil	Ponte Alta do Bom Jesus	2000	75.4%	81.0%	68.7%
Brazil	Ponte Alta do Bom Jesus	2017	76.1%	81.6%	69.5%
Brazil	Ponte Alta do Norte	2000	75.1%	80.5%	69.1%

Country	Second Administrative Unit	Year	Mean	Upper	Lower
Brazil	Ponte Alta do Norte	2000	76.5%	80.9%	71.6%
Brazil	Ponte Alta do Norte	2017	75.8%	81.1%	69.9%
Brazil	Ponte Alta do Norte	2017	77.2%	81.5%	72.4%
Brazil	Ponte Branca	2000	71.8%	78.7%	63.9%
Brazil	Ponte Branca	2017	72.5%	79.4%	64.8%
Brazil	Ponte Nova	2000	81.4%	85.3%	77.0%
Brazil	Ponte Nova	2017	82.0%	85.8%	77.7%
Brazil	Ponte Preta	2000	79.1%	82.6%	75.6%
Brazil	Ponte Preta	2017	79.7%	83.1%	76.4%
Brazil	Ponte Serrada	2000	79.1%	82.9%	75.0%
Brazil	Ponte Serrada	2017	79.7%	83.5%	75.7%
Brazil	Pontes e Lacerda	2000	64.3%	72.3%	55.6%
Brazil	Pontes e Lacerda	2017	65.1%	72.9%	56.5%
Brazil	Pontes Gestral	2000	89.6%	92.4%	86.0%
Brazil	Pontes Gestral	2017	90.0%	92.6%	86.4%
Brazil	Ponto Belo	2000	79.1%	83.1%	74.7%
Brazil	Ponto Belo	2017	79.8%	83.6%	75.4%
Brazil	Ponto Chique	2000	81.8%	87.4%	75.2%
Brazil	Ponto Chique	2017	82.3%	87.8%	76.0%
Brazil	Ponto dos Volantes	2000	82.7%	87.3%	75.9%
Brazil	Ponto dos Volantes	2017	83.3%	87.7%	76.6%
Brazil	Ponto Novo	2000	75.7%	81.2%	69.2%
Brazil	Ponto Novo	2017	76.4%	81.8%	70.1%
Brazil	Populina	2000	86.7%	90.3%	82.7%
Brazil	Populina	2017	87.1%	90.6%	83.3%
Brazil	Poranga	2000	68.2%	73.3%	63.5%
Brazil	Poranga	2017	69.0%	74.1%	64.4%
Brazil	Porangaba	2000	93.2%	94.7%	91.1%
Brazil	Porangaba	2017	93.4%	94.9%	91.4%
Brazil	Porangatu	2000	77.9%	82.7%	71.5%
Brazil	Porangatu	2017	78.7%	83.4%	72.5%
Brazil	Porciúncula	2000	78.7%	82.2%	75.0%
Brazil	Porciúncula	2017	79.3%	82.7%	75.7%
Brazil	Porecatu	2000	86.8%	89.8%	83.8%
Brazil	Porecatu	2017	87.3%	90.1%	84.3%
Brazil	Portalegre	2000	78.1%	81.3%	74.8%
Brazil	Portalegre	2017	78.7%	81.9%	75.5%
Brazil	Portao	2000	80.5%	82.7%	78.2%
Brazil	Portao	2017	81.3%	83.3%	79.0%
Brazil	Porteiras	2000	68.1%	71.7%	64.0%
Brazil	Porteiras	2017	69.0%	72.4%	64.8%
Brazil	Porteirinha	2000	80.9%	85.9%	75.1%
Brazil	Porteirinha	2017	81.6%	86.4%	75.8%
Brazil	Porteiro	2000	76.3%	80.9%	71.4%
Brazil	Porteiro	2017	77.0%	81.5%	72.2%
Brazil	Portel	2000	39.6%	46.8%	33.6%
Brazil	Portel	2017	40.5%	47.7%	34.5%
Brazil	Portelândia	2000	76.5%	82.6%	71.1%
Brazil	Portelândia	2017	77.2%	83.2%	71.9%
Brazil	Porto	2000	59.3%	64.2%	54.7%
Brazil	Porto	2017	60.3%	65.1%	55.6%
Brazil	Porto Acre	2000	42.0%	45.3%	38.9%
Brazil	Porto Acre	2017	42.9%	46.2%	39.8%

Country	Second Administrative Unit	Year	Mean	Upper	Lower
Brazil	Porto Alegre	2000	82.3%	84.4%	80.4%
Brazil	Porto Alegre	2017	82.9%	84.9%	81.0%
Brazil	Porto Alegre do Norte	2000	68.2%	76.2%	57.5%
Brazil	Porto Alegre do Norte	2017	69.0%	76.8%	58.4%
Brazil	Porto Alegre do Piauí	2000	60.1%	67.4%	52.8%
Brazil	Porto Alegre do Piauí	2017	61.0%	68.3%	53.8%
Brazil	Porto Alegre do Tocantins	2000	76.6%	82.0%	70.9%
Brazil	Porto Alegre do Tocantins	2017	77.3%	82.5%	71.7%
Brazil	Porto Amazonas	2000	81.6%	84.5%	78.2%
Brazil	Porto Amazonas	2017	82.1%	85.0%	78.8%
Brazil	Porto Barreiro	2000	82.2%	85.8%	77.8%
Brazil	Porto Barreiro	2017	82.7%	86.2%	78.4%
Brazil	Porto Belo	2000	72.7%	77.4%	67.6%
Brazil	Porto Belo	2017	73.7%	78.3%	68.8%
Brazil	Porto Calvo	2000	64.7%	68.0%	60.8%
Brazil	Porto Calvo	2017	65.6%	68.9%	61.7%
Brazil	Porto da Folha	2000	69.6%	72.6%	65.6%
Brazil	Porto da Folha	2017	70.5%	73.5%	66.6%
Brazil	Porto de Moz	2000	38.4%	45.3%	31.7%
Brazil	Porto de Moz	2017	40.1%	47.1%	33.3%
Brazil	Porto de Pedras	2000	63.7%	67.0%	59.4%
Brazil	Porto de Pedras	2017	64.7%	68.0%	60.6%
Brazil	Porto do Mangue	2000	82.0%	85.3%	78.0%
Brazil	Porto do Mangue	2017	82.6%	85.8%	78.7%
Brazil	Porto dos Gaúchos	2000	66.9%	74.4%	59.8%
Brazil	Porto dos Gaúchos	2017	67.7%	75.1%	60.8%
Brazil	Porto Esperidião	2000	67.8%	75.0%	59.6%
Brazil	Porto Esperidião	2017	68.6%	75.7%	60.5%
Brazil	Porto Estrela	2000	67.4%	72.6%	61.1%
Brazil	Porto Estrela	2017	68.3%	73.4%	62.1%
Brazil	Porto Feliz	2000	93.5%	94.7%	92.2%
Brazil	Porto Feliz	2017	93.7%	94.9%	92.5%
Brazil	Porto Ferreira	2000	92.7%	94.3%	90.8%
Brazil	Porto Ferreira	2017	93.0%	94.5%	91.1%
Brazil	Porto Firme	2000	80.6%	83.9%	76.7%
Brazil	Porto Firme	2017	81.2%	84.4%	77.4%
Brazil	Porto Franco	2000	71.8%	76.1%	66.9%
Brazil	Porto Franco	2017	72.6%	76.9%	67.8%
Brazil	Porto Grande	2000	49.0%	53.3%	44.5%
Brazil	Porto Grande	2017	50.0%	54.3%	45.5%
Brazil	Porto Lucena	2000	80.5%	86.1%	74.2%
Brazil	Porto Lucena	2017	81.1%	86.5%	75.0%
Brazil	Porto Mauá	2000	79.9%	85.0%	73.5%
Brazil	Porto Mauá	2017	80.5%	85.5%	74.2%

Country	Second Administrative Unit	Year	Mean	Upper	Lower
Brazil	Porto Murtinho	2000	77.6%	85.2%	66.7%
Brazil	Porto Murtinho	2017	78.3%	85.6%	67.6%
Brazil	Porto Nacional	2000	75.2%	79.4%	71.3%
Brazil	Porto Nacional	2017	75.9%	80.0%	72.1%
Brazil	Porto Real	2000	81.3%	84.2%	78.0%
Brazil	Porto Real	2017	81.9%	84.7%	78.6%
Brazil	Porto Real do Colégio	2000	66.9%	69.6%	64.1%
Brazil	Porto Real do Colégio	2017	67.7%	70.4%	65.0%
Brazil	Porto Rico	2000	82.2%	86.0%	78.4%
Brazil	Porto Rico	2017	82.7%	86.4%	79.0%
Brazil	Porto Rico do Maranhão	2000	59.0%	66.1%	51.4%
Brazil	Porto Rico do Maranhão	2017	60.0%	67.0%	52.4%
Brazil	Porto Seguro	2000	76.1%	81.6%	69.9%
Brazil	Porto Seguro	2017	76.8%	82.2%	70.7%
Brazil	Porto União	2000	79.6%	83.8%	74.7%
Brazil	Porto União	2017	80.3%	84.4%	75.5%
Brazil	Porto Velho	2000	30.4%	32.9%	28.1%
Brazil	Porto Velho	2017	31.2%	33.8%	28.9%
Brazil	Porto Vera Cruz	2000	80.1%	85.8%	73.5%
Brazil	Porto Vera Cruz	2017	80.7%	86.3%	74.3%
Brazil	Porto Vitória	2000	79.8%	84.0%	74.9%
Brazil	Porto Vitória	2017	80.4%	84.5%	75.7%
Brazil	Porto Walter	2000	42.3%	46.6%	37.8%
Brazil	Porto Walter	2017	43.3%	47.6%	38.6%
Brazil	Porto Xavier	2000	81.1%	86.6%	74.5%
Brazil	Porto Xavier	2017	81.7%	87.0%	75.1%
Brazil	Posse	2000	81.2%	85.7%	75.9%
Brazil	Posse	2017	81.8%	86.2%	76.6%
Brazil	Poté	2000	82.9%	87.5%	77.6%
Brazil	Poté	2017	83.4%	87.9%	78.3%
Brazil	Potengi	2000	68.3%	72.4%	63.7%
Brazil	Potengi	2017	70.7%	74.6%	66.3%
Brazil	Potim	2000	89.5%	91.9%	86.9%
Brazil	Potim	2017	89.8%	92.1%	87.2%
Brazil	Potiraguá	2000	79.1%	84.0%	73.7%
Brazil	Potiraguá	2017	79.7%	84.5%	74.5%
Brazil	Potirendaba	2000	92.8%	94.5%	90.6%
Brazil	Potirendaba	2017	93.1%	94.7%	90.9%
Brazil	Potiretama	2000	76.1%	79.7%	71.5%
Brazil	Potiretama	2017	76.8%	80.4%	72.3%
Brazil	Pouso Alegre	2000	89.1%	91.4%	86.5%
Brazil	Pouso Alegre	2017	89.4%	91.7%	87.0%
Brazil	Pouso Alto	2000	85.6%	88.7%	82.4%
Brazil	Pouso Alto	2017	86.1%	89.1%	83.0%
Brazil	Pouso Novo	2000	82.5%	85.9%	78.8%
Brazil	Pouso Novo	2017	83.0%	86.3%	79.4%
Brazil	Pouso Redondo	2000	73.9%	78.1%	69.2%
Brazil	Pouso Redondo	2017	74.6%	78.8%	70.1%
Brazil	Poxoréo	2000	67.9%	73.7%	62.2%
Brazil	Poxoréo	2017	69.4%	75.0%	63.6%

Country	Second Administrative Unit	Year	Mean	Upper	Lower
Brazil	Pracinha	2000	89.7%	92.4%	86.4%
Brazil	Pracinha	2017	90.0%	92.6%	86.9%
Brazil	Pracuúba	2000	47.4%	53.6%	41.1%
Brazil	Pracuúba	2017	48.4%	54.5%	42.1%
Brazil	Prado	2000	76.9%	82.8%	69.9%
Brazil	Prado	2017	77.6%	83.3%	70.8%
Brazil	Prado Ferreira	2000	86.5%	88.9%	83.4%
Brazil	Prado Ferreira	2017	86.9%	89.2%	84.0%
Brazil	Pradópolis	2000	92.5%	94.2%	90.0%
Brazil	Pradópolis	2017	92.7%	94.4%	90.3%
Brazil	Prados	2000	80.7%	85.0%	76.3%
Brazil	Prados	2017	81.3%	85.5%	77.0%
Brazil	Praia Grande	2000	77.0%	81.1%	72.1%
Brazil	Praia Grande	2000	87.0%	89.2%	84.8%
Brazil	Praia Grande	2017	77.7%	81.7%	72.8%
Brazil	Praia Grande	2017	87.8%	89.9%	85.8%
Brazil	Praia Norte	2000	64.7%	69.0%	60.4%
Brazil	Praia Norte	2017	65.5%	69.7%	61.2%
Brazil	Prainha	2000	38.3%	45.3%	30.8%
Brazil	Prainha	2017	39.2%	46.2%	31.7%
Brazil	Pranchita	2000	82.5%	86.6%	78.3%
Brazil	Pranchita	2017	83.1%	87.1%	79.0%
Brazil	Prata	2000	66.4%	70.8%	62.0%
Brazil	Prata	2000	84.5%	88.2%	79.6%
Brazil	Prata	2017	67.3%	71.6%	62.9%
Brazil	Prata	2017	85.0%	88.6%	80.2%
Brazil	Prata do Piauí	2000	58.1%	63.2%	52.6%
Brazil	Prata do Piauí	2017	59.0%	64.1%	53.5%
Brazil	Pratânia	2000	93.9%	95.5%	91.9%
Brazil	Pratânia	2017	94.1%	95.6%	92.2%
Brazil	Pratápolis	2000	87.1%	90.8%	82.9%
Brazil	Pratápolis	2017	87.6%	91.1%	83.4%
Brazil	Pratinha	2000	84.7%	88.9%	79.9%
Brazil	Pratinha	2017	85.2%	89.3%	80.5%
Brazil	Presidente Alves	2000	93.1%	95.0%	90.6%
Brazil	Presidente Alves	2017	93.3%	95.2%	90.9%
Brazil	Presidente Bernardes	2000	88.7%	91.3%	86.0%
Brazil	Presidente Bernardes	2000	80.3%	83.5%	76.6%
Brazil	Presidente Bernardes	2017	89.1%	91.6%	86.5%
Brazil	Presidente Bernardes	2017	80.9%	84.1%	77.3%
Brazil	Presidente Castelo Branco	2000	84.8%	87.4%	81.4%
Brazil	Presidente Castelo Branco	2000	76.2%	79.8%	71.2%
Brazil	Presidente Castelo Branco	2017	76.9%	80.5%	72.1%
Brazil	Presidente Castelo Branco	2017	85.3%	87.8%	82.0%
Brazil	Presidente Dutra	2000	76.7%	82.5%	70.2%
Brazil	Presidente Dutra	2000	60.8%	66.8%	56.5%

Country	Second Administrative Unit	Year	Mean	Upper	Lower
Brazil	Presidente Dutra	2017	77.4%	83.1%	71.1%
Brazil	Presidente Dutra	2017	63.1%	69.0%	58.8%
Brazil	Presidente Epitácio	2000	86.1%	88.9%	82.3%
Brazil	Presidente Epitácio	2017	86.6%	89.3%	82.8%
Brazil	Presidente Figueiredo	2000	61.2%	66.8%	55.9%
Brazil	Presidente Figueiredo	2017	62.1%	67.7%	56.8%
Brazil	Presidente Getúlio	2000	73.4%	77.2%	69.4%
Brazil	Presidente Getúlio	2017	74.1%	77.8%	70.2%
Brazil	Presidente Jânio Quadros	2000	78.8%	83.5%	73.1%
Brazil	Presidente Jânio Quadros	2017	79.4%	84.1%	73.8%
Brazil	Presidente Juscelino	2000	82.4%	84.7%	80.3%
Brazil	Presidente Juscelino	2000	81.3%	86.1%	75.1%
Brazil	Presidente Juscelino	2000	60.6%	65.1%	55.8%
Brazil	Presidente Juscelino	2017	82.9%	85.2%	80.9%
Brazil	Presidente Juscelino	2017	81.9%	86.6%	75.8%
Brazil	Presidente Juscelino	2017	61.5%	65.9%	56.8%
Brazil	Presidente Kennedy	2000	74.9%	79.4%	70.2%
Brazil	Presidente Kennedy	2000	74.8%	78.3%	70.9%
Brazil	Presidente Kennedy	2017	75.5%	79.0%	71.7%
Brazil	Presidente Kennedy	2017	75.6%	80.0%	71.0%
Brazil	Presidente Kubitschek	2000	84.4%	88.0%	79.3%
Brazil	Presidente Kubitschek	2017	84.9%	88.4%	79.9%
Brazil	Presidente Lucena	2000	80.1%	82.8%	77.4%
Brazil	Presidente Lucena	2017	80.7%	83.3%	78.0%
Brazil	Presidente Médici	2000	27.9%	31.0%	24.7%
Brazil	Presidente Médici	2000	53.5%	59.5%	47.7%
Brazil	Presidente Médici	2017	28.7%	31.9%	25.5%
Brazil	Presidente Médici	2017	54.5%	60.5%	48.7%
Brazil	Presidente Nereu	2000	75.2%	78.8%	71.6%
Brazil	Presidente Nereu	2017	76.0%	79.5%	72.4%
Brazil	Presidente Olegário	2000	80.4%	86.0%	73.5%

Country	Second Administrative Unit	Year	Mean	Upper	Lower
Brazil	Presidente Olegário	2017	81.0%	86.4%	74.3%
Brazil	Presidente Prudente	2000	89.9%	92.5%	87.5%
Brazil	Presidente Prudente	2017	90.3%	92.7%	88.0%
Brazil	Presidente Sarney	2000	57.7%	63.2%	52.1%
Brazil	Presidente Sarney	2017	58.6%	64.1%	53.1%
Brazil	Presidente Tancredo Neves	2000	76.4%	80.1%	71.8%
Brazil	Presidente Tancredo Neves	2017	77.1%	80.7%	72.6%
Brazil	Presidente Vargas	2000	60.5%	65.8%	55.2%
Brazil	Presidente Vargas	2017	61.5%	66.7%	56.2%
Brazil	Presidente Venceslau	2000	88.0%	90.4%	84.7%
Brazil	Presidente Venceslau	2017	88.4%	90.7%	85.2%
Brazil	Primavera	2000	66.9%	69.9%	63.7%
Brazil	Primavera	2000	37.2%	41.9%	32.6%
Brazil	Primavera	2017	67.7%	70.7%	64.6%
Brazil	Primavera	2017	38.1%	42.9%	33.4%
Brazil	Primavera de Rondônia	2000	27.7%	31.9%	23.8%
Brazil	Primavera de Rondônia	2017	28.5%	32.7%	24.6%
Brazil	Primavera do Leste	2000	70.2%	75.5%	64.4%
Brazil	Primavera do Leste	2017	71.0%	76.2%	65.3%
Brazil	Primeira Cruz	2000	59.4%	64.9%	52.7%
Brazil	Primeira Cruz	2017	60.3%	65.8%	53.7%
Brazil	Primeiro de Maio	2000	86.6%	89.0%	84.0%
Brazil	Primeiro de Maio	2017	87.1%	89.4%	84.5%
Brazil	Princesa	2000	81.2%	85.6%	76.5%
Brazil	Princesa	2017	81.8%	86.2%	77.2%
Brazil	Princesa Isabel	2000	67.5%	71.5%	62.8%
Brazil	Princesa Isabel	2017	68.3%	72.3%	63.7%
Brazil	Professor Jamil	2000	75.9%	79.0%	72.3%
Brazil	Professor Jamil	2017	76.6%	79.6%	73.1%
Brazil	Progresso	2000	83.1%	86.5%	79.7%
Brazil	Progresso	2017	83.7%	87.0%	80.4%
Brazil	Promissão	2000	93.2%	95.2%	90.9%
Brazil	Promissão	2017	93.5%	95.4%	91.2%
Brazil	Propriá	2000	68.9%	71.9%	66.0%
Brazil	Propriá	2017	69.7%	72.7%	66.9%
Brazil	Protásio Alves	2000	80.7%	83.9%	76.8%
Brazil	Protásio Alves	2017	81.3%	84.5%	77.5%
Brazil	Prudente de Morais	2000	80.8%	84.3%	77.2%

Country	Second Administrative Unit	Year	Mean	Upper	Lower
Brazil	Prudente de Morais	2017	81.4%	84.8%	77.9%
Brazil	Prudentópolis	2000	82.3%	86.1%	78.3%
Brazil	Prudentópolis	2017	82.9%	86.6%	79.0%
Brazil	Pugmil	2000	74.7%	78.5%	70.5%
Brazil	Pugmil	2017	75.4%	79.2%	71.3%
Brazil	Pureza	2000	84.6%	87.2%	81.5%
Brazil	Pureza	2017	85.1%	87.6%	82.1%
Brazil	Putinga	2000	82.7%	86.0%	79.1%
Brazil	Putinga	2017	83.2%	86.4%	79.7%
Brazil	Puxinanã	2000	70.8%	73.9%	68.3%
Brazil	Puxinanã	2017	71.6%	74.7%	69.1%
Brazil	Quadra	2000	93.4%	95.0%	91.4%
Brazil	Quadra	2017	93.6%	95.2%	91.7%
Brazil	Quaraí	2000	81.9%	88.1%	72.7%
Brazil	Quaraí	2017	82.4%	88.5%	73.5%
Brazil	Quartel Geral	2000	80.4%	85.5%	73.4%
Brazil	Quartel Geral	2017	81.0%	86.0%	74.2%
Brazil	Quarto Centenário	2000	83.3%	87.1%	79.1%
Brazil	Quarto Centenário	2017	83.9%	87.5%	79.7%
Brazil	Quatá	2000	91.1%	93.4%	88.6%
Brazil	Quatá	2017	91.4%	93.6%	89.0%
Brazil	Quatiguá	2000	88.4%	90.9%	85.0%
Brazil	Quatiguá	2017	88.8%	91.2%	85.5%
Brazil	Quatipuru	2000	37.4%	42.7%	32.2%
Brazil	Quatipuru	2017	38.3%	43.7%	33.1%
Brazil	Quatis	2000	81.4%	84.3%	78.2%
Brazil	Quatis	2017	81.9%	84.8%	78.8%
Brazil	Quatro Barras	2000	86.1%	88.4%	83.5%
Brazil	Quatro Barras	2017	86.6%	88.8%	84.0%
Brazil	Quatro Pontes	2000	83.9%	87.5%	80.3%
Brazil	Quatro Pontes	2017	84.4%	88.0%	80.9%
Brazil	Quebrangulo	2000	58.4%	61.9%	55.2%
Brazil	Quebrangulo	2017	59.4%	62.8%	56.1%
Brazil	Quedas do Iguaçu	2000	82.9%	86.2%	79.0%
Brazil	Quedas do Iguaçu	2017	83.4%	86.7%	79.6%
Brazil	Queimada Nova	2000	64.4%	70.1%	59.0%
Brazil	Queimada Nova	2017	65.4%	70.9%	60.0%
Brazil	Queimadas	2000	76.0%	81.5%	70.4%
Brazil	Queimadas	2000	69.2%	72.4%	66.4%
Brazil	Queimadas	2017	76.7%	82.0%	71.2%
Brazil	Queimadas	2017	70.1%	73.3%	67.5%
Brazil	Queimados	2000	76.2%	78.0%	74.1%
Brazil	Queimados	2017	76.8%	78.6%	74.8%
Brazil	Queiroz	2000	92.3%	94.5%	89.6%
Brazil	Queiroz	2017	92.5%	94.7%	90.0%
Brazil	Queluz	2000	84.9%	87.6%	81.7%
Brazil	Queluz	2017	85.4%	88.0%	82.3%
Brazil	Queluzita	2000	81.2%	85.2%	77.4%
Brazil	Queluzita	2017	81.8%	85.7%	78.0%
Brazil	Querência	2000	68.5%	76.7%	59.3%
Brazil	Querência	2017	70.9%	78.7%	62.1%
Brazil	Querência do Norte	2000	83.3%	86.8%	79.5%
Brazil	Querência do Norte	2017	83.9%	87.2%	80.1%

Country	Second Administrative Unit	Year	Mean	Upper	Lower
Brazil	Quevedos	2000	83.1%	87.2%	78.8%
Brazil	Quevedos	2017	83.7%	87.6%	79.5%
Brazil	Quijingue	2000	77.1%	81.4%	71.6%
Brazil	Quijingue	2017	77.8%	82.0%	72.4%
Brazil	Quilombo	2000	77.3%	81.8%	72.9%
Brazil	Quilombo	2017	78.0%	82.3%	73.7%
Brazil	Quinta do Sol	2000	83.1%	86.3%	79.4%
Brazil	Quinta do Sol	2017	83.6%	86.8%	80.0%
Brazil	Quinze de Novembro	2000	81.8%	85.3%	77.0%
Brazil	Quinze de Novembro	2017	82.4%	85.8%	77.6%
Brazil	Quipapá	2000	66.1%	69.6%	62.9%
Brazil	Quipapá	2017	66.9%	70.5%	63.8%
Brazil	Quirinópolis	2000	78.9%	83.2%	74.0%
Brazil	Quirinópolis	2017	80.2%	84.3%	75.5%
Brazil	Quissamã	2000	75.1%	79.3%	70.7%
Brazil	Quissamã	2017	75.9%	80.0%	71.5%
Brazil	Quitana	2000	92.2%	94.4%	89.4%
Brazil	Quitana	2017	92.4%	94.6%	89.8%
Brazil	Quitandinha	2000	81.8%	84.5%	79.0%
Brazil	Quitandinha	2017	82.3%	85.0%	79.6%
Brazil	Quiterianópolis	2000	68.9%	73.8%	63.7%
Brazil	Quiterianópolis	2017	69.7%	74.5%	64.6%
Brazil	Quixabá	2000	67.0%	71.2%	62.2%
Brazil	Quixabá	2000	69.1%	73.7%	64.2%
Brazil	Quixabá	2017	67.9%	72.0%	63.1%
Brazil	Quixabá	2017	70.0%	74.4%	65.1%
Brazil	Quixabeira	2000	78.2%	82.5%	73.6%
Brazil	Quixabeira	2017	78.9%	83.1%	74.3%
Brazil	Quixada	2000	74.2%	78.1%	70.3%
Brazil	Quixada	2017	75.0%	78.8%	71.2%
Brazil	Quixelô	2000	71.4%	75.5%	67.3%
Brazil	Quixelô	2017	72.2%	76.2%	68.2%
Brazil	Quixeramobim	2000	69.5%	73.8%	65.3%
Brazil	Quixeramobim	2017	70.4%	74.5%	66.1%
Brazil	Quixeré	2000	75.9%	79.5%	72.0%
Brazil	Quixeré	2017	76.6%	80.1%	72.8%
Brazil	Rafael Fernandes	2000	75.4%	78.8%	71.8%
Brazil	Rafael Fernandes	2017	76.1%	79.5%	72.6%
Brazil	Rafael Godeiro	2000	78.8%	82.0%	75.2%
Brazil	Rafael Godeiro	2017	79.4%	82.5%	75.9%
Brazil	Rafael Jambeiro	2000	78.1%	82.1%	73.5%
Brazil	Rafael Jambeiro	2017	78.7%	82.6%	74.2%
Brazil	Rafard	2000	93.6%	94.7%	92.2%
Brazil	Rafard	2017	93.9%	94.9%	92.4%
Brazil	Ramilândia	2000	83.6%	87.1%	80.2%
Brazil	Ramilândia	2017	84.1%	87.5%	80.8%
Brazil	Rancharia	2000	90.6%	93.1%	88.2%
Brazil	Rancharia	2017	90.9%	93.3%	88.6%
Brazil	Rancho Alegre	2000	86.7%	88.9%	83.9%
Brazil	Rancho Alegre	2017	87.1%	89.3%	84.4%
Brazil	Rancho Alegre d'Oeste	2000	83.4%	87.0%	79.0%

Country	Second Administrative Unit	Year	Mean	Upper	Lower
Brazil	Rancho Alegre d'Oeste	2017	84.0%	87.4%	79.6%
Brazil	Rancho Queimado	2000	77.1%	81.0%	72.1%
Brazil	Rancho Queimado	2017	77.8%	81.6%	72.9%
Brazil	Raposa	2000	60.9%	65.0%	55.7%
Brazil	Raposa	2017	61.8%	65.9%	56.7%
Brazil	Raposos	2000	82.4%	85.1%	79.7%
Brazil	Raposos	2017	83.0%	85.6%	80.3%
Brazil	Raul Soares	2000	80.5%	84.1%	76.7%
Brazil	Raul Soares	2017	81.1%	84.6%	77.4%
Brazil	Realeza	2000	82.1%	85.4%	78.2%
Brazil	Realeza	2017	82.6%	85.9%	78.9%
Brazil	Rebouças	2000	81.9%	85.0%	78.0%
Brazil	Rebouças	2017	82.4%	85.5%	78.7%
Brazil	Recife	2000	65.4%	67.3%	63.5%
Brazil	Recife	2017	66.3%	68.1%	64.4%
Brazil	Recreio	2000	77.4%	81.1%	73.4%
Brazil	Recreio	2017	78.1%	81.7%	74.2%
Brazil	Recursolândia	2000	74.7%	80.6%	67.8%
Brazil	Recursolândia	2017	75.4%	81.2%	68.6%
Brazil	Redenção	2000	49.6%	56.4%	42.2%
Brazil	Redenção	2000	70.2%	73.5%	66.8%
Brazil	Redenção	2017	71.7%	74.8%	68.3%
Brazil	Redenção	2017	50.7%	57.4%	43.2%
Brazil	Redenção da Serra	2000	92.8%	94.3%	91.2%
Brazil	Redenção da Serra	2017	93.0%	94.5%	91.5%
Brazil	Redenção do Gurguéia	2000	60.3%	67.5%	52.3%
Brazil	Redenção do Gurguéia	2017	61.3%	68.4%	53.3%
Brazil	Redentora	2000	80.7%	85.1%	76.3%
Brazil	Redentora	2017	81.3%	85.6%	77.0%
Brazil	Reduto	2000	81.1%	84.4%	77.0%
Brazil	Reduto	2017	82.2%	85.3%	78.2%
Brazil	Regeneração	2000	59.3%	63.9%	54.6%
Brazil	Regeneração	2017	60.2%	64.8%	55.6%
Brazil	Regente Feijó	2000	90.0%	92.5%	87.4%
Brazil	Regente Feijó	2017	90.4%	92.8%	87.8%
Brazil	Reginópolis	2000	93.2%	95.1%	90.8%
Brazil	Reginópolis	2017	93.8%	95.6%	91.7%
Brazil	Registro	2000	89.2%	92.3%	85.7%
Brazil	Registro	2017	89.6%	92.6%	86.2%
Brazil	Relvado	2000	82.4%	85.8%	78.8%
Brazil	Relvado	2017	82.9%	86.3%	79.5%
Brazil	Remanso	2000	67.4%	74.2%	59.9%
Brazil	Remanso	2017	68.3%	75.0%	60.8%
Brazil	Remígio	2000	68.3%	71.3%	65.1%
Brazil	Remígio	2017	69.1%	72.1%	65.9%
Brazil	Renascença	2000	81.3%	85.3%	76.3%
Brazil	Renascença	2017	81.9%	85.8%	77.0%
Brazil	Reriutaba	2000	66.4%	70.3%	62.3%
Brazil	Reriutaba	2017	67.4%	71.2%	63.3%
Brazil	Resende	2000	83.3%	86.3%	80.2%
Brazil	Resende	2017	83.9%	86.8%	80.8%
Brazil	Resende Costa	2000	83.6%	87.5%	79.8%
Brazil	Resende Costa	2017	84.1%	87.9%	80.4%

Country	Second Administrative Unit	Year	Mean	Upper	Lower
Brazil	Reserva	2000	83.9%	87.6%	79.2%
Brazil	Reserva	2017	84.4%	88.0%	79.8%
Brazil	Reserva do Cabaçal	2000	69.9%	75.8%	62.5%
Brazil	Reserva do Cabaçal	2017	70.6%	76.4%	63.4%
Brazil	Reserva do Iguaçu	2000	81.5%	85.6%	76.7%
Brazil	Reserva do Iguaçu	2017	82.1%	86.1%	77.4%
Brazil	Resplendor	2000	76.8%	80.8%	72.4%
Brazil	Resplendor	2017	77.6%	81.4%	73.2%
Brazil	Ressaquinha	2000	83.9%	87.0%	80.1%
Brazil	Ressaquinha	2017	84.3%	87.4%	80.6%
Brazil	Restinga	2000	88.9%	91.6%	85.0%
Brazil	Restinga	2017	89.3%	91.9%	85.5%
Brazil	Restinga Seca	2000	81.2%	84.7%	76.2%
Brazil	Restinga Seca	2017	81.7%	85.2%	76.9%
Brazil	Retirolândia	2000	79.3%	82.8%	74.8%
Brazil	Retirolândia	2017	79.9%	83.3%	75.5%
Brazil	Riachão	2000	71.2%	77.3%	63.3%
Brazil	Riachão	2017	72.0%	77.9%	64.2%
Brazil	Riachão das Neves	2000	73.4%	80.2%	66.3%
Brazil	Riachão das Neves	2017	74.1%	80.9%	67.2%
Brazil	Riachão do Bacamarte	2000	77.2%	79.9%	74.6%
Brazil	Riachão do Bacamarte	2017	77.9%	80.6%	75.4%
Brazil	Riachao do dantas	2000	77.8%	80.6%	75.2%
Brazil	Riachao do dantas	2017	78.5%	81.2%	75.9%
Brazil	Riachao do Jacuipe	2000	78.6%	82.4%	74.0%
Brazil	Riachao do Jacuipe	2017	79.2%	82.9%	74.8%
Brazil	Riachão do Poço	2000	68.4%	72.0%	64.7%
Brazil	Riachão do Poço	2017	69.3%	72.8%	65.6%
Brazil	Riachinho	2000	70.6%	74.6%	66.1%
Brazil	Riachinho	2000	83.6%	89.1%	77.9%
Brazil	Riachinho	2017	71.5%	75.4%	67.1%
Brazil	Riachinho	2017	84.6%	89.9%	79.2%
Brazil	Riacho	2000	66.0%	69.1%	63.0%
Brazil	Riacho	2017	66.9%	70.0%	63.9%
Brazil	Riacho da Cruz	2000	77.3%	80.6%	73.7%
Brazil	Riacho da Cruz	2017	77.9%	81.2%	74.5%
Brazil	Riacho das Almas	2000	66.9%	69.9%	63.9%
Brazil	Riacho das Almas	2017	67.7%	70.7%	64.7%
Brazil	Riacho de Santana	2000	77.0%	82.5%	70.8%
Brazil	Riacho de Santana	2000	75.5%	79.1%	71.9%
Brazil	Riacho de Santana	2017	76.2%	79.7%	72.7%

Country	Second Administrative Unit	Year	Mean	Upper	Lower
Brazil	Riacho de Santana	2017	77.7%	83.1%	71.6%
Brazil	Riacho de Santo Antônio	2000	67.1%	70.7%	63.7%
Brazil	Riacho de Santo Antônio	2017	67.9%	71.5%	64.6%
Brazil	Riacho dos Machados	2000	82.2%	86.8%	76.6%
Brazil	Riacho dos Machados	2017	82.7%	87.2%	77.3%
Brazil	Riacho Frio	2000	65.0%	72.1%	57.8%
Brazil	Riacho Frio	2017	65.9%	72.8%	58.7%
Brazil	Riachuelo	2000	83.8%	86.4%	81.1%
Brazil	Riachuelo	2000	74.3%	76.3%	71.8%
Brazil	Riachuelo	2017	84.3%	86.8%	81.7%
Brazil	Riachuelo	2017	75.0%	77.0%	72.6%
Brazil	Rialma	2000	77.7%	81.9%	73.5%
Brazil	Rialma	2017	78.3%	82.5%	74.2%
Brazil	Rianápolis	2000	77.9%	82.2%	73.9%
Brazil	Rianápolis	2017	78.6%	82.8%	74.7%
Brazil	Ribamar Fiquene	2000	68.4%	73.4%	63.4%
Brazil	Ribamar Fiquene	2017	69.3%	74.1%	64.3%
Brazil	Ribas do Rio Pardo	2000	78.5%	82.5%	73.8%
Brazil	Ribas do Rio Pardo	2017	79.1%	83.0%	74.6%
Brazil	Ribeira	2000	87.2%	90.3%	83.5%
Brazil	Ribeira	2017	87.6%	90.6%	84.0%
Brazil	Ribeira do Amparo	2000	77.8%	81.3%	73.6%
Brazil	Ribeira do Amparo	2017	78.4%	81.8%	74.3%
Brazil	Ribeira do Piauí	2000	59.9%	66.0%	53.6%
Brazil	Ribeira do Piauí	2017	60.9%	66.9%	54.6%
Brazil	Ribeira do Pombal	2000	77.4%	81.6%	72.9%
Brazil	Ribeira do Pombal	2017	78.1%	82.2%	73.7%
Brazil	Ribeirão	2000	62.5%	66.2%	59.1%
Brazil	Ribeirão	2017	63.4%	67.0%	60.0%
Brazil	Ribeirão Bonito	2000	93.3%	94.9%	91.5%
Brazil	Ribeirão Bonito	2017	93.5%	95.1%	91.8%
Brazil	Ribeirão Branco	2000	91.8%	94.1%	89.3%
Brazil	Ribeirão Branco	2017	92.1%	94.3%	89.6%
Brazil	Ribeirão Cascalheira	2000	72.0%	80.5%	63.7%
Brazil	Ribeirão Cascalheira	2017	72.6%	80.8%	64.7%
Brazil	Ribeirão Claro	2000	90.4%	92.7%	87.3%
Brazil	Ribeirão Claro	2017	90.7%	92.9%	87.7%
Brazil	Ribeirão Corrente	2000	88.8%	91.6%	85.4%

Country	Second Administrative Unit	Year	Mean	Upper	Lower
Brazil	Ribeirão Corrente	2017	89.2%	91.9%	85.8%
Brazil	Ribeirão das Neves	2000	82.0%	84.4%	79.4%
Brazil	Ribeirão das Neves	2017	82.7%	85.0%	80.2%
Brazil	Ribeirão do Largo	2000	79.6%	84.4%	73.8%
Brazil	Ribeirão do Largo	2017	80.0%	84.7%	74.3%
Brazil	Ribeirão do Pinhal	2000	87.4%	90.1%	84.6%
Brazil	Ribeirão do Pinhal	2017	87.9%	90.5%	85.1%
Brazil	Ribeirão do Sul	2000	91.3%	93.7%	88.4%
Brazil	Ribeirão do Sul	2017	91.6%	93.9%	88.8%
Brazil	Ribeirão dos índios	2000	88.3%	91.0%	85.4%
Brazil	Ribeirão dos índios	2017	88.7%	91.3%	85.9%
Brazil	Ribeirão Grande	2000	92.5%	94.5%	90.0%
Brazil	Ribeirão Grande	2017	92.7%	94.7%	90.4%
Brazil	Ribeirão Pires	2000	94.2%	95.0%	93.2%
Brazil	Ribeirão Pires	2017	94.5%	95.3%	93.6%
Brazil	Ribeirao Preto	2000	94.3%	95.6%	92.5%
Brazil	Ribeirao Preto	2017	94.5%	95.8%	92.8%
Brazil	Ribeirão Vermelho	2000	81.4%	85.2%	77.1%
Brazil	Ribeirão Vermelho	2017	81.9%	85.7%	77.8%
Brazil	Ribeirãozinho	2000	71.8%	78.2%	64.7%
Brazil	Ribeirãozinho	2017	72.6%	78.9%	65.5%
Brazil	Ribeiro Gonçalves	2000	62.0%	69.8%	53.8%
Brazil	Ribeiro Gonçalves	2017	62.8%	70.6%	54.7%
Brazil	Ribeirópolis	2000	77.9%	80.2%	75.3%
Brazil	Ribeirópolis	2017	78.6%	80.8%	76.0%
Brazil	Ricaho dos Cavalos	2000	74.5%	78.1%	70.9%
Brazil	Ricaho dos Cavalos	2017	75.2%	78.7%	71.7%
Brazil	Rifaina	2000	85.8%	90.1%	80.8%
Brazil	Rifaina	2017	86.2%	90.4%	81.4%
Brazil	Rincão	2000	93.0%	94.6%	90.6%
Brazil	Rincão	2017	93.3%	94.8%	90.9%
Brazil	Rinópolis	2000	91.8%	94.0%	89.4%
Brazil	Rinópolis	2017	92.1%	94.2%	89.7%
Brazil	Rio Acima	2000	80.9%	84.1%	78.0%
Brazil	Rio Acima	2017	81.5%	84.6%	78.6%
Brazil	Rio Azul	2000	80.8%	84.7%	76.3%
Brazil	Rio Azul	2017	81.4%	85.2%	77.0%
Brazil	Rio Bananal	2000	77.5%	80.7%	73.4%
Brazil	Rio Bananal	2017	78.1%	81.3%	74.2%
Brazil	Rio Bom	2000	84.3%	87.2%	80.9%
Brazil	Rio Bom	2017	84.8%	87.6%	81.5%

Country	Second Administrative Unit	Year	Mean	Upper	Lower
Brazil	Rio Bonito	2000	77.9%	80.3%	74.8%
Brazil	Rio Bonito	2017	78.6%	80.9%	75.5%
Brazil	Rio Bonito do Iguaçu	2000	82.5%	86.2%	78.1%
Brazil	Rio Bonito do Iguaçu	2017	83.1%	86.7%	78.8%
Brazil	Rio Branco	2000	66.8%	73.0%	59.1%
Brazil	Rio Branco	2000	40.6%	42.3%	38.9%
Brazil	Rio Branco	2017	67.7%	73.8%	60.1%
Brazil	Rio Branco	2017	41.5%	43.3%	39.8%
Brazil	Rio Branco do Ivaí	2000	83.3%	87.1%	78.9%
Brazil	Rio Branco do Ivaí	2017	83.9%	87.6%	79.5%
Brazil	Rio Branco do Sul	2000	86.1%	88.6%	83.7%
Brazil	Rio Branco do Sul	2017	86.5%	88.9%	84.1%
Brazil	Rio Brilhante	2000	79.4%	83.0%	75.8%
Brazil	Rio Brilhante	2017	80.1%	83.6%	76.5%
Brazil	Rio Casca	2000	80.1%	83.9%	75.8%
Brazil	Rio Casca	2017	80.7%	84.4%	76.5%
Brazil	Rio Claro	2000	83.8%	86.0%	81.4%
Brazil	Rio Claro	2000	93.3%	94.7%	91.7%
Brazil	Rio Claro	2017	84.3%	86.4%	82.0%
Brazil	Rio Claro	2017	93.5%	94.9%	92.0%
Brazil	Rio Crespo	2000	27.6%	31.9%	23.4%
Brazil	Rio Crespo	2017	28.4%	32.8%	24.1%
Brazil	Rio da Conceição	2000	75.5%	81.3%	69.5%
Brazil	Rio da Conceição	2017	76.2%	81.9%	70.4%
Brazil	Rio das Antas	2000	76.3%	81.0%	72.3%
Brazil	Rio das Antas	2017	77.0%	81.6%	73.1%
Brazil	Rio das Flores	2000	80.4%	83.4%	77.1%
Brazil	Rio das Flores	2017	81.0%	83.9%	77.8%
Brazil	Rio das Ostras	2000	76.8%	80.5%	73.0%
Brazil	Rio das Ostras	2017	77.4%	81.2%	73.8%
Brazil	Rio das Pedras	2000	93.7%	94.8%	92.2%
Brazil	Rio das Pedras	2017	93.9%	95.0%	92.5%
Brazil	Rio de Contas	2000	77.3%	82.4%	71.6%
Brazil	Rio de Contas	2017	78.0%	82.9%	72.4%
Brazil	Rio de Janeiro	2000	79.1%	80.3%	77.9%
Brazil	Rio de Janeiro	2017	79.7%	80.9%	78.6%
Brazil	Rio do Antônio	2000	77.6%	83.1%	72.1%
Brazil	Rio do Antônio	2017	78.2%	83.6%	72.8%
Brazil	Rio do Campo	2000	75.4%	79.9%	69.4%
Brazil	Rio do Campo	2017	76.1%	80.6%	70.3%
Brazil	Rio do Fogo	2000	83.2%	86.0%	79.6%
Brazil	Rio do Fogo	2017	83.7%	86.5%	80.3%
Brazil	Rio do Oeste	2000	73.0%	77.2%	68.6%
Brazil	Rio do Oeste	2017	73.8%	77.9%	69.4%
Brazil	Rio do Pires	2000	77.5%	83.0%	71.6%
Brazil	Rio do Pires	2017	78.1%	83.5%	72.4%
Brazil	Rio do Prado	2000	82.6%	86.4%	77.2%
Brazil	Rio do Prado	2017	83.1%	86.8%	77.9%
Brazil	Rio do Sul	2000	75.4%	79.2%	71.5%
Brazil	Rio do Sul	2017	76.1%	79.8%	72.2%

Country	Second Administrative Unit	Year	Mean	Upper	Lower
Brazil	Rio doce	2000	79.3%	83.4%	74.9%
Brazil	Rio doce	2017	79.9%	83.9%	75.6%
Brazil	Rio dos Bois	2000	74.0%	78.9%	69.3%
Brazil	Rio dos Bois	2017	74.7%	79.5%	70.2%
Brazil	Rio dos Cedros	2000	73.9%	77.6%	70.3%
Brazil	Rio dos Cedros	2017	74.7%	78.3%	71.2%
Brazil	Rio dos índios	2000	77.5%	81.2%	73.1%
Brazil	Rio dos índios	2017	78.2%	81.8%	73.9%
Brazil	Rio Espera	2000	81.0%	84.2%	76.9%
Brazil	Rio Espera	2017	81.5%	84.7%	77.6%
Brazil	Rio Formoso	2000	60.5%	64.3%	56.3%
Brazil	Rio Formoso	2017	61.4%	65.2%	57.3%
Brazil	Rio Fortuna	2000	73.3%	77.2%	68.3%
Brazil	Rio Fortuna	2017	74.0%	77.9%	69.2%
Brazil	Rio Grande	2000	80.7%	85.6%	75.0%
Brazil	Rio Grande	2017	81.3%	86.1%	75.7%
Brazil	Rio Grande da Serra	2000	93.8%	95.0%	92.1%
Brazil	Rio Grande da Serra	2017	94.0%	95.2%	92.4%
Brazil	Rio Grande do Piauí	2000	62.1%	67.8%	55.5%
Brazil	Rio Grande do Piauí	2017	63.0%	68.6%	56.5%
Brazil	Rio Largo	2000	64.2%	66.9%	62.2%
Brazil	Rio Largo	2017	65.0%	67.7%	63.0%
Brazil	Rio Manso	2000	80.9%	84.2%	77.1%
Brazil	Rio Manso	2017	81.5%	84.7%	77.8%
Brazil	Rio Maria	2000	42.5%	49.4%	34.9%
Brazil	Rio Maria	2017	43.6%	50.4%	35.9%
Brazil	Rio Negrinho	2000	80.5%	83.6%	77.1%
Brazil	Rio Negrinho	2017	81.1%	84.1%	77.8%
Brazil	Rio Negro	2000	80.5%	83.8%	76.9%
Brazil	Rio Negro	2000	77.7%	82.4%	72.1%
Brazil	Rio Negro	2017	81.2%	84.3%	77.6%
Brazil	Rio Negro	2017	78.4%	83.0%	72.9%
Brazil	Rio Novo	2000	79.3%	82.6%	75.2%
Brazil	Rio Novo	2017	79.9%	83.2%	75.9%
Brazil	Rio Novo do Sul	2000	77.3%	80.3%	73.5%
Brazil	Rio Novo do Sul	2017	78.0%	80.9%	74.3%
Brazil	Rio Paranaiba	2000	81.6%	86.8%	74.5%
Brazil	Rio Paranaiba	2017	82.1%	87.3%	75.2%
Brazil	Rio Paranaíba	2000	81.5%	86.0%	76.3%
Brazil	Rio Paranaíba	2017	82.1%	86.5%	77.0%
Brazil	Rio Pardo	2000	82.2%	85.4%	78.0%
Brazil	Rio Pardo	2017	82.7%	85.9%	78.6%
Brazil	Rio Pardo de Minas	2000	80.8%	84.6%	77.2%
Brazil	Rio Pardo de Minas	2017	81.5%	85.2%	78.0%
Brazil	Rio Pomba	2000	80.1%	83.5%	76.0%
Brazil	Rio Pomba	2017	80.7%	84.0%	76.7%
Brazil	Rio Preto	2000	81.1%	84.3%	77.8%
Brazil	Rio Preto	2017	81.7%	84.8%	78.4%
Brazil	Rio Preto da Eva	2000	56.8%	60.9%	52.6%
Brazil	Rio Preto da Eva	2017	57.8%	61.8%	53.6%

Country	Second Administrative Unit	Year	Mean	Upper	Lower
Brazil	Rio Quente	2000	78.0%	81.6%	73.4%
Brazil	Rio Quente	2017	78.7%	82.2%	74.2%
Brazil	Rio Real	2000	78.6%	81.9%	75.0%
Brazil	Rio Real	2017	80.2%	83.3%	76.8%
Brazil	Rio Rufino	2000	77.9%	81.9%	72.8%
Brazil	Rio Rufino	2017	78.5%	82.4%	73.5%
Brazil	Rio Sono	2000	74.9%	79.4%	69.7%
Brazil	Rio Sono	2017	75.5%	80.0%	70.3%
Brazil	Rio Tinto	2000	70.7%	73.8%	67.9%
Brazil	Rio Tinto	2017	71.7%	74.7%	68.9%
Brazil	Rio Verde	2000	78.3%	82.8%	73.8%
Brazil	Rio Verde	2017	78.9%	83.3%	74.5%
Brazil	Rio Verde de Mato Grosso	2000	79.8%	84.5%	74.1%
Brazil	Rio Verde de Mato Grosso	2017	80.4%	85.0%	74.9%
Brazil	Rio Vermelho	2000	81.9%	86.3%	76.1%
Brazil	Rio Vermelho	2017	82.5%	86.8%	76.8%
Brazil	Riolândia	2000	88.5%	91.2%	84.9%
Brazil	Riolândia	2017	88.9%	91.5%	85.4%
Brazil	Riozinho	2000	82.0%	85.3%	78.4%
Brazil	Riozinho	2017	82.5%	85.8%	79.0%
Brazil	Riqueza	2000	77.4%	81.6%	72.9%
Brazil	Riqueza	2017	78.0%	82.2%	73.6%
Brazil	Ritápolis	2000	81.9%	86.0%	77.6%
Brazil	Ritápolis	2017	82.4%	86.4%	78.2%
Brazil	Riversul	2000	90.1%	92.5%	87.2%
Brazil	Riversul	2017	90.0%	92.5%	87.2%
Brazil	Roca Sales	2000	78.9%	82.6%	75.0%
Brazil	Roca Sales	2017	79.8%	83.4%	75.9%
Brazil	Rochedo	2000	77.4%	81.2%	73.6%
Brazil	Rochedo	2017	78.1%	81.8%	74.2%
Brazil	Rochedo de Minas	2000	80.3%	83.5%	76.5%
Brazil	Rochedo de Minas	2017	80.9%	84.1%	77.2%
Brazil	Rodeio	2000	73.3%	76.9%	70.1%
Brazil	Rodeio	2017	74.0%	77.5%	70.9%
Brazil	Rodeio Bonito	2000	77.6%	81.4%	73.4%
Brazil	Rodeio Bonito	2017	78.3%	82.0%	74.2%
Brazil	Rodeiro	2000	79.7%	83.1%	75.5%
Brazil	Rodeiro	2017	80.3%	83.7%	76.2%
Brazil	Rodelas	2000	67.4%	73.1%	61.4%
Brazil	Rodelas	2017	68.3%	74.0%	62.2%
Brazil	Rodolfo Fernandes	2000	77.1%	80.8%	72.9%
Brazil	Rodolfo Fernandes	2017	77.8%	81.4%	73.7%
Brazil	Rodrigues Alves	2000	43.4%	46.6%	40.0%
Brazil	Rodrigues Alves	2017	44.3%	47.6%	40.9%
Brazil	Rolândia	2000	86.6%	88.7%	83.9%
Brazil	Rolândia	2017	87.2%	89.2%	84.6%
Brazil	Rolante	2000	80.1%	83.2%	76.6%
Brazil	Rolante	2017	80.7%	83.8%	77.3%
Brazil	Rolim de Moura	2000	28.8%	32.6%	24.6%
Brazil	Rolim de Moura	2017	29.6%	33.4%	25.4%
Brazil	Romaria	2000	82.0%	86.5%	77.1%
Brazil	Romaria	2017	82.6%	87.0%	77.8%

Country	Second Administrative Unit	Year	Mean	Upper	Lower
Brazil	Romelândia	2000	78.6%	82.7%	74.1%
Brazil	Romelândia	2017	79.3%	83.2%	74.9%
Brazil	Roncador	2000	84.6%	87.6%	80.7%
Brazil	Roncador	2017	85.1%	88.1%	81.4%
Brazil	Ronda Alta	2000	80.2%	83.7%	76.6%
Brazil	Ronda Alta	2017	80.8%	84.2%	77.3%
Brazil	Rondinha	2000	79.6%	83.1%	76.1%
Brazil	Rondinha	2017	80.2%	83.6%	76.8%
Brazil	Rondon	2000	83.3%	86.4%	79.8%
Brazil	Rondon	2017	83.9%	86.8%	80.4%
Brazil	Rondon do Pará	2000	51.9%	57.8%	45.8%
Brazil	Rondon do Pará	2017	52.9%	58.8%	46.8%
Brazil	Rondonópolis	2000	68.9%	73.9%	64.0%
Brazil	Rondonópolis	2017	69.7%	74.6%	64.9%
Brazil	Roque Gonzales	2000	80.8%	86.1%	74.7%
Brazil	Roque Gonzales	2017	81.4%	86.6%	75.5%
Brazil	Rorainópolis	2000	84.8%	87.8%	81.3%
Brazil	Rorainópolis	2017	85.9%	88.7%	82.7%
Brazil	Rosana	2000	82.9%	86.6%	78.7%
Brazil	Rosana	2017	83.5%	87.1%	79.3%
Brazil	Rosário	2000	59.5%	63.8%	54.9%
Brazil	Rosário	2017	60.5%	64.7%	55.8%
Brazil	Rosário da Limeira	2000	81.2%	84.8%	77.3%
Brazil	Rosário da Limeira	2017	81.8%	85.3%	77.9%
Brazil	Rosário do Catete	2000	72.8%	75.1%	69.9%
Brazil	Rosário do Catete	2017	73.5%	75.8%	70.7%
Brazil	Rosário do Ivaí	2000	83.1%	86.9%	77.8%
Brazil	Rosário do Ivaí	2017	83.6%	87.4%	78.5%
Brazil	Rosário do Sul	2000	82.9%	87.6%	77.9%
Brazil	Rosário do Sul	2017	83.5%	88.0%	78.6%
Brazil	Rosário Oeste	2000	67.7%	72.8%	61.7%
Brazil	Rosário Oeste	2017	68.6%	73.6%	62.8%
Brazil	Roseira	2000	89.8%	92.1%	87.2%
Brazil	Roseira	2017	90.1%	92.4%	87.6%
Brazil	Roteiro	2000	59.5%	63.0%	56.0%
Brazil	Roteiro	2017	59.3%	62.9%	55.8%
Brazil	Rubelita	2000	81.0%	85.6%	75.0%
Brazil	Rubelita	2017	81.6%	86.0%	75.8%
Brazil	Rubiácea	2000	90.9%	93.2%	88.1%
Brazil	Rubiácea	2017	91.2%	93.4%	88.5%
Brazil	Rubiataba	2000	78.3%	82.3%	73.4%
Brazil	Rubiataba	2017	78.9%	82.9%	74.1%
Brazil	Rubim	2000	81.1%	85.5%	75.5%
Brazil	Rubim	2017	81.7%	85.9%	76.2%
Brazil	Rubinéia	2000	85.9%	90.0%	81.7%
Brazil	Rubinéia	2017	86.4%	90.4%	82.4%
Brazil	Rurópolis	2000	41.9%	48.7%	34.4%
Brazil	Rurópolis	2017	42.8%	49.7%	35.3%
Brazil	Russas	2000	75.1%	78.5%	71.3%
Brazil	Russas	2017	75.8%	79.2%	72.1%
Brazil	Ruy Barbosa	2000	84.2%	86.8%	81.4%
Brazil	Ruy Barbosa	2000	78.6%	83.3%	73.6%

Country	Second Administrative Unit	Year	Mean	Upper	Lower
Brazil	Ruy Barbosa	2017	84.7%	87.2%	82.0%
Brazil	Ruy Barbosa	2017	79.3%	83.9%	74.4%
Brazil	Sabará	2000	82.0%	84.5%	79.2%
Brazil	Sabará	2017	82.5%	85.0%	79.9%
Brazil	Sabáudia	2000	85.7%	87.9%	82.7%
Brazil	Sabáudia	2017	86.2%	88.3%	83.2%
Brazil	Sabino	2000	92.8%	94.9%	90.5%
Brazil	Sabino	2017	93.1%	95.1%	90.9%
Brazil	Sabinópolis	2000	82.0%	86.2%	76.5%
Brazil	Sabinópolis	2017	82.6%	86.7%	77.2%
Brazil	Saboeiro	2000	68.7%	72.4%	64.3%
Brazil	Saboeiro	2017	69.6%	73.2%	65.3%
Brazil	Sacramento	2000	85.6%	89.6%	80.9%
Brazil	Sacramento	2017	86.1%	90.0%	81.4%
Brazil	Sagrada Família	2000	78.9%	82.8%	75.0%
Brazil	Sagrada Família	2017	79.6%	83.4%	75.8%
Brazil	Sagres	2000	90.2%	92.7%	87.1%
Brazil	Sagres	2017	90.4%	92.8%	87.3%
Brazil	Sairé	2000	69.4%	72.8%	66.4%
Brazil	Sairé	2017	70.2%	73.4%	67.2%
Brazil	Saldanha Marinho	2000	82.4%	85.9%	77.5%
Brazil	Saldanha Marinho	2017	82.9%	86.4%	78.2%
Brazil	Sales	2000	92.9%	94.7%	90.7%
Brazil	Sales	2017	93.1%	94.9%	91.0%
Brazil	Sales Oliveira	2000	90.6%	92.8%	87.7%
Brazil	Sales Oliveira	2017	90.9%	93.1%	88.2%
Brazil	Salesópolis	2000	93.8%	94.9%	92.5%
Brazil	Salesópolis	2017	94.1%	95.1%	92.8%
Brazil	Salete	2000	74.6%	79.0%	69.3%
Brazil	Salete	2017	75.4%	79.7%	70.1%
Brazil	Salgadinho	2000	72.0%	76.2%	67.9%
Brazil	Salgadinho	2000	62.7%	66.3%	59.4%
Brazil	Salgadinho	2017	63.5%	67.2%	60.3%
Brazil	Salgadinho	2017	72.9%	77.0%	68.8%
Brazil	Salgado	2000	77.4%	79.4%	75.4%
Brazil	Salgado	2017	78.1%	80.0%	76.1%
Brazil	Salgado de São Félix	2000	66.0%	69.0%	63.2%
Brazil	Salgado de São Félix	2017	66.8%	69.9%	64.0%
Brazil	Salgado Filho	2000	81.2%	85.3%	76.1%
Brazil	Salgado Filho	2017	81.8%	85.8%	76.8%
Brazil	Salgueiro	2000	66.9%	71.1%	62.3%
Brazil	Salgueiro	2017	67.8%	71.9%	63.3%
Brazil	Salidao	2000	67.0%	71.5%	62.7%
Brazil	Salidao	2017	68.4%	72.7%	64.3%
Brazil	Salinas	2000	80.9%	85.7%	74.4%
Brazil	Salinas	2017	81.7%	86.3%	75.4%
Brazil	Salinas da Margarida	2000	74.2%	77.7%	70.9%
Brazil	Salinas da Margarida	2017	74.9%	78.4%	71.7%
Brazil	Salinópolis	2000	35.1%	41.2%	29.7%
Brazil	Salinópolis	2017	36.0%	42.2%	30.5%
Brazil	Salitre	2000	66.8%	71.1%	62.2%
Brazil	Salitre	2017	67.6%	71.9%	63.1%
Brazil	Salmourão	2000	90.7%	93.1%	87.9%
Brazil	Salmourão	2017	91.0%	93.3%	88.3%

Country	Second Administrative Unit	Year	Mean	Upper	Lower
Brazil	Saloá	2000	64.1%	67.7%	60.4%
Brazil	Saloá	2017	65.0%	68.5%	61.3%
Brazil	Saltinho	2000	93.1%	94.4%	91.5%
Brazil	Saltinho	2000	79.4%	83.4%	75.4%
Brazil	Saltinho	2017	80.0%	84.0%	76.2%
Brazil	Saltinho	2017	93.3%	94.6%	91.8%
Brazil	Salto	2000	93.8%	94.8%	92.6%
Brazil	Salto	2017	94.0%	95.0%	92.8%
Brazil	Salto da Divisa	2000	81.0%	85.9%	75.5%
Brazil	Salto da Divisa	2017	81.6%	86.4%	76.2%
Brazil	Salto do Céu	2000	69.4%	75.5%	62.1%
Brazil	Salto do Céu	2017	70.1%	76.1%	62.9%
Brazil	Salto do Itararé	2000	89.5%	92.0%	86.3%
Brazil	Salto do Itararé	2017	89.9%	92.3%	86.8%
Brazil	Salto do Jacuí	2000	82.6%	86.2%	78.3%
Brazil	Salto do Jacuí	2017	83.1%	86.7%	78.9%
Brazil	Salto do Londra	2000	82.4%	86.2%	78.5%
Brazil	Salto do Londra	2017	83.0%	86.6%	79.2%
Brazil	Salto do Pirapora	2000	93.8%	95.3%	92.3%
Brazil	Salto do Pirapora	2017	94.1%	95.5%	92.6%
Brazil	Salto Grande	2000	91.2%	93.4%	88.1%
Brazil	Salto Grande	2017	91.5%	93.6%	88.5%
Brazil	Salto Veloso	2000	77.9%	81.6%	74.1%
Brazil	Salto Veloso	2017	78.5%	82.2%	74.8%
Brazil	Salvador	2000	76.1%	78.7%	73.0%
Brazil	Salvador	2017	76.8%	79.3%	73.8%
Brazil	Salvador das Missões	2000	81.1%	86.2%	75.3%
Brazil	Salvador das Missões	2017	81.7%	86.6%	76.0%
Brazil	Salvador do Sul	2000	83.8%	86.3%	81.1%
Brazil	Salvador do Sul	2017	84.3%	86.8%	81.7%
Brazil	Salvaterra	2000	35.7%	40.3%	31.1%
Brazil	Salvaterra	2017	36.6%	41.2%	32.0%
Brazil	Sambaíba	2000	62.3%	70.7%	54.5%
Brazil	Sambaíba	2017	63.2%	71.5%	55.5%
Brazil	Sampaio	2000	62.4%	67.0%	58.3%
Brazil	Sampaio	2017	63.2%	67.8%	59.1%
Brazil	San Antonio do Itambe	2000	81.8%	86.3%	75.7%
Brazil	San Antonio do Itambe	2017	82.3%	86.8%	76.4%
Brazil	San Antonio do Rio Abai	2000	80.2%	84.2%	75.3%
Brazil	San Antonio do Rio Abai	2017	80.8%	84.8%	76.0%
Brazil	Sananduva	2000	78.5%	82.4%	74.5%
Brazil	Sananduva	2017	79.1%	83.0%	75.2%
Brazil	Sanclerlândia	2000	75.5%	79.8%	70.5%
Brazil	Sanclerlândia	2017	76.2%	80.4%	71.3%
Brazil	Sandolândia	2000	73.8%	79.4%	66.5%
Brazil	Sandolândia	2017	74.6%	80.0%	67.3%

Country	Second Administrative Unit	Year	Mean	Upper	Lower
Brazil	Sandovalina	2000	86.4%	89.6%	83.2%
Brazil	Sandovalina	2017	86.9%	90.0%	83.8%
Brazil	Sangão	2000	73.0%	77.5%	67.6%
Brazil	Sangão	2017	73.7%	78.2%	68.4%
Brazil	Sanharó	2000	66.3%	69.8%	62.5%
Brazil	Sanharó	2017	67.2%	70.6%	63.4%
Brazil	Santa Adélia	2000	93.2%	94.9%	91.1%
Brazil	Santa Adélia	2017	93.5%	95.1%	91.4%
Brazil	Santa Albertina	2000	86.5%	90.7%	82.3%
Brazil	Santa Albertina	2017	86.9%	91.1%	82.9%
Brazil	Santa Amélia	2000	88.0%	90.2%	85.2%
Brazil	Santa Amélia	2017	88.4%	90.5%	85.7%
Brazil	Santa Bárbara	2000	82.0%	85.6%	78.8%
Brazil	Santa Bárbara	2000	78.1%	81.6%	74.2%
Brazil	Santa Bárbara	2017	82.5%	86.1%	79.4%
Brazil	Santa Bárbara	2017	78.8%	82.2%	74.9%
Brazil	Santa Bárbara d'Oeste	2000	93.4%	94.6%	91.9%
Brazil	Santa Bárbara d'Oeste	2017	93.6%	94.8%	92.2%
Brazil	Santa Bárbara de Goiás	2000	75.8%	79.1%	72.9%
Brazil	Santa Bárbara de Goiás	2017	76.5%	79.8%	73.7%
Brazil	Santa Bárbara do Leste	2000	80.8%	84.2%	76.5%
Brazil	Santa Bárbara do Leste	2017	81.4%	84.8%	77.3%
Brazil	Santa Bárbara do Monte Verde	2000	82.1%	85.0%	78.8%
Brazil	Santa Bárbara do Monte Verde	2017	82.7%	85.6%	79.4%
Brazil	Santa Bárbara do Pará	2000	35.4%	37.9%	32.5%
Brazil	Santa Bárbara do Pará	2017	36.3%	38.9%	33.4%
Brazil	Santa Bárbara do Sul	2000	82.6%	86.2%	78.1%
Brazil	Santa Bárbara do Sul	2017	83.2%	86.7%	78.8%
Brazil	Santa Bárbara do Tugúrio	2000	81.3%	84.5%	77.8%
Brazil	Santa Bárbara do Tugúrio	2017	81.9%	85.0%	78.5%
Brazil	Santa Branca	2000	93.4%	94.6%	92.1%
Brazil	Santa Branca	2017	93.7%	94.8%	92.4%
Brazil	Santa Brígida	2000	68.8%	72.7%	64.0%
Brazil	Santa Brígida	2017	69.6%	73.4%	64.9%
Brazil	Santa Carmem	2000	69.2%	75.3%	63.0%
Brazil	Santa Carmem	2017	70.0%	76.0%	63.9%
Brazil	Santa Cecília	2000	77.5%	81.5%	72.7%
Brazil	Santa Cecília	2000	65.0%	68.5%	61.5%
Brazil	Santa Cecília	2017	78.2%	82.1%	73.5%
Brazil	Santa Cecília	2017	65.9%	69.4%	62.5%
Brazil	Santa Cecília do Pavão	2000	85.7%	88.4%	82.8%

Country	Second Administrative Unit	Year	Mean	Upper	Lower
Brazil	Santa Cecília do Pavão	2017	86.2%	88.8%	83.3%
Brazil	Santa Clara do Oeste	2000	86.0%	90.4%	81.6%
Brazil	Santa Clara do Oeste	2017	86.5%	90.7%	82.2%
Brazil	Santa Clara do Sul	2000	81.3%	85.0%	77.2%
Brazil	Santa Clara do Sul	2017	82.0%	85.6%	78.0%
Brazil	Santa Cruz	2000	81.5%	83.5%	79.1%
Brazil	Santa Cruz	2000	65.7%	70.9%	60.8%
Brazil	Santa Cruz	2017	66.6%	71.7%	61.7%
Brazil	Santa Cruz	2017	81.1%	83.3%	78.5%
Brazil	Santa Cruz Cabrália	2000	76.1%	81.4%	69.8%
Brazil	Santa Cruz Cabrália	2017	76.8%	82.0%	70.6%
Brazil	Santa Cruz da Baixa Verde	2000	66.9%	71.6%	62.0%
Brazil	Santa Cruz da Baixa Verde	2017	67.8%	72.4%	62.9%
Brazil	Santa Cruz da Conceição	2000	92.4%	93.9%	90.4%
Brazil	Santa Cruz da Conceição	2017	92.6%	94.1%	90.8%
Brazil	Santa Cruz da Esperança	2000	90.5%	92.6%	87.9%
Brazil	Santa Cruz da Esperança	2017	90.8%	92.9%	88.3%
Brazil	Santa Cruz da Vitória	2000	80.3%	83.9%	75.5%
Brazil	Santa Cruz da Vitória	2017	81.0%	84.4%	76.2%
Brazil	Santa Cruz das Palmeiras	2000	91.4%	93.1%	89.1%
Brazil	Santa Cruz das Palmeiras	2017	91.7%	93.4%	89.5%
Brazil	Santa Cruz de Goiás	2000	78.1%	81.9%	73.7%
Brazil	Santa Cruz de Goiás	2017	78.8%	82.5%	74.5%
Brazil	Santa Cruz de Minas	2000	82.9%	86.9%	79.0%
Brazil	Santa Cruz de Minas	2017	83.5%	87.3%	79.6%
Brazil	Santa Cruz de Monte Caste	2000	83.1%	86.7%	79.0%
Brazil	Santa Cruz de Monte Caste	2017	83.7%	87.2%	79.7%
Brazil	Santa Cruz de Salinas	2000	81.9%	86.1%	76.4%
Brazil	Santa Cruz de Salinas	2017	82.5%	86.6%	77.1%
Brazil	Santa Cruz do Arari	2000	37.4%	46.1%	30.1%
Brazil	Santa Cruz do Arari	2017	38.4%	47.1%	31.0%
Brazil	Santa Cruz do Capibaribe	2000	66.6%	70.6%	62.7%
Brazil	Santa Cruz do Capibaribe	2017	67.5%	71.4%	63.6%

Country	Second Administrative Unit	Year	Mean	Upper	Lower
Brazil	Santa Cruz do Escalvado	2000	78.8%	83.0%	74.2%
Brazil	Santa Cruz do Escalvado	2017	79.5%	83.6%	75.0%
Brazil	Santa Cruz do Piaui	2000	59.6%	64.8%	54.0%
Brazil	Santa Cruz do Piaui	2017	60.5%	65.6%	55.0%
Brazil	Santa Cruz do Rio Pardo	2000	91.5%	93.7%	88.4%
Brazil	Santa Cruz do Rio Pardo	2017	91.8%	93.9%	88.8%
Brazil	Santa Cruz do Sul	2000	81.6%	85.0%	77.0%
Brazil	Santa Cruz do Sul	2017	82.1%	85.5%	77.6%
Brazil	Santa Cruz dos Milagres	2000	59.4%	64.4%	53.4%
Brazil	Santa Cruz dos Milagres	2017	60.3%	65.3%	54.3%
Brazil	Santa Efigênia de Minas	2000	82.7%	86.7%	78.6%
Brazil	Santa Efigênia de Minas	2017	83.3%	87.1%	79.3%
Brazil	Santa Ernestina	2000	93.8%	95.3%	91.9%
Brazil	Santa Ernestina	2017	94.0%	95.4%	92.2%
Brazil	Santa Fé	2000	85.3%	88.2%	82.3%
Brazil	Santa Fé	2017	85.7%	88.6%	82.9%
Brazil	Santa Fé de Goiás	2000	61.1%	67.8%	55.4%
Brazil	Santa Fé de Goiás	2017	62.0%	68.7%	56.4%
Brazil	Santa Fé de Minas	2000	82.4%	87.8%	75.9%
Brazil	Santa Fé de Minas	2017	82.9%	88.2%	76.6%
Brazil	Santa Fé do Araguaia	2000	66.5%	71.6%	61.2%
Brazil	Santa Fé do Araguaia	2017	67.4%	72.4%	62.1%
Brazil	Santa Fé do Sul	2000	86.9%	91.0%	83.0%
Brazil	Santa Fé do Sul	2017	87.3%	91.3%	83.5%
Brazil	Santa Filomena	2000	65.4%	70.7%	60.1%
Brazil	Santa Filomena	2000	63.6%	71.2%	56.1%
Brazil	Santa Filomena	2017	66.2%	71.5%	61.0%
Brazil	Santa Filomena	2017	64.5%	72.0%	56.8%
Brazil	Santa Filomena do Maranhão	2000	74.2%	80.2%	67.5%
Brazil	Santa Filomena do Maranhão	2017	74.9%	80.8%	68.4%
Brazil	Santa Gertrudes	2000	92.9%	94.4%	91.2%

Country	Second Administrative Unit	Year	Mean	Upper	Lower
Brazil	Santa Gertrudes	2017	93.1%	94.6%	91.5%
Brazil	Santa Helena	2000	82.0%	86.2%	78.1%
Brazil	Santa Helena	2000	79.7%	83.8%	75.6%
Brazil	Santa Helena	2000	71.0%	74.8%	67.4%
Brazil	Santa Helena	2000	57.2%	62.8%	51.1%
Brazil	Santa Helena	2017	80.3%	84.3%	76.3%
Brazil	Santa Helena	2017	82.6%	86.7%	78.7%
Brazil	Santa Helena	2017	71.8%	75.5%	68.3%
Brazil	Santa Helena	2017	58.2%	63.7%	52.1%
Brazil	Santa Helena de Goiás	2000	78.3%	82.6%	73.8%
Brazil	Santa Helena de Goiás	2017	79.0%	83.1%	74.5%
Brazil	Santa Helena de Minas	2000	80.5%	85.3%	75.0%
Brazil	Santa Helena de Minas	2017	81.1%	85.8%	75.7%
Brazil	Santa Inês	2000	85.9%	88.8%	82.3%
Brazil	Santa Inês	2000	83.1%	86.2%	78.4%
Brazil	Santa Inês	2000	61.4%	65.6%	56.5%
Brazil	Santa Inês	2000	67.9%	72.3%	63.8%
Brazil	Santa Inês	2017	86.4%	89.2%	82.9%
Brazil	Santa Inês	2017	83.6%	86.7%	79.0%
Brazil	Santa Inês	2017	62.3%	66.4%	57.4%
Brazil	Santa Inês	2017	68.7%	73.0%	64.6%
Brazil	Santa Isabel	2000	78.5%	82.8%	74.4%
Brazil	Santa Isabel	2000	93.6%	94.6%	92.5%
Brazil	Santa Isabel	2017	79.2%	83.4%	75.2%
Brazil	Santa Isabel	2017	93.8%	94.8%	92.8%
Brazil	Santa Isabel do Ivaí	2000	85.2%	88.4%	81.6%
Brazil	Santa Isabel do Ivaí	2017	85.7%	88.8%	82.1%
Brazil	Santa Isabel do Oeste	2000	82.1%	85.7%	78.5%
Brazil	Santa Isabel do Oeste	2017	82.7%	86.2%	79.1%
Brazil	Santa Isabel do Pará	2000	36.8%	40.0%	34.1%
Brazil	Santa Isabel do Pará	2017	37.7%	41.0%	35.0%
Brazil	Santa Isabel do Rio Negro	2000	65.2%	75.4%	53.7%
Brazil	Santa Isabel do Rio Negro	2017	66.6%	76.6%	55.3%
Brazil	Santa Juliana	2000	83.2%	87.3%	78.1%
Brazil	Santa Juliana	2017	83.7%	87.7%	78.8%
Brazil	Santa Leopoldina	2000	78.5%	80.8%	76.2%
Brazil	Santa Leopoldina	2017	79.2%	81.5%	76.9%
Brazil	Santa Lucia	2000	93.1%	94.6%	90.8%
Brazil	Santa Lucia	2017	93.4%	94.8%	91.2%
Brazil	Santa Lúcia	2000	83.3%	86.6%	79.6%
Brazil	Santa Lúcia	2017	83.8%	87.0%	80.3%
Brazil	Santa Luz	2000	59.9%	68.4%	52.9%
Brazil	Santa Luz	2017	60.9%	69.2%	53.9%
Brazil	Santa Luzia	2000	60.6%	65.7%	56.0%
Brazil	Santa Luzia	2000	81.8%	84.3%	79.2%
Brazil	Santa Luzia	2000	77.2%	81.9%	71.3%
Brazil	Santa Luzia	2000	74.9%	78.7%	70.7%

Country	Second Administrative Unit	Year	Mean	Upper	Lower
Brazil	Santa Luzia	2017	75.6%	79.3%	71.5%
Brazil	Santa Luzia	2017	61.6%	66.6%	57.0%
Brazil	Santa Luzia	2017	77.8%	82.5%	72.2%
Brazil	Santa Luzia	2017	82.6%	85.0%	80.0%
Brazil	Santa Luzia d'Oeste	2000	29.3%	33.4%	25.1%
Brazil	Santa Luzia d'Oeste	2017	30.1%	34.4%	25.9%
Brazil	Santa Luzia do Itanhy	2000	76.0%	79.2%	72.9%
Brazil	Santa Luzia do Itanhy	2017	76.7%	79.9%	73.7%
Brazil	Santa Luzia do Norte	2000	55.7%	58.1%	53.2%
Brazil	Santa Luzia do Norte	2017	56.7%	59.0%	54.2%
Brazil	Santa Luzia do Pará	2000	40.0%	44.6%	35.5%
Brazil	Santa Luzia do Pará	2017	40.9%	45.5%	36.4%
Brazil	Santa Luzia do Paruá	2000	55.6%	61.7%	49.1%
Brazil	Santa Luzia do Paruá	2017	56.5%	62.6%	50.1%
Brazil	Santa Margarida	2000	82.0%	85.3%	77.8%
Brazil	Santa Margarida	2017	82.6%	85.8%	78.4%
Brazil	Santa Maria	2000	83.5%	85.7%	81.2%
Brazil	Santa Maria	2000	81.7%	85.5%	76.9%
Brazil	Santa Maria	2017	84.0%	86.2%	81.8%
Brazil	Santa Maria	2017	82.3%	86.0%	77.6%
Brazil	Santa Maria da Boa Vista	2000	66.3%	71.9%	60.2%
Brazil	Santa Maria da Boa Vista	2017	67.1%	72.7%	61.1%
Brazil	Santa Maria da Serra	2000	93.1%	94.7%	91.2%
Brazil	Santa Maria da Serra	2017	93.4%	94.9%	91.5%
Brazil	Santa Maria da Vitória	2000	78.3%	84.7%	71.2%
Brazil	Santa Maria da Vitória	2017	79.0%	85.2%	72.0%
Brazil	Santa Maria das Barreiras	2000	51.9%	58.8%	44.4%
Brazil	Santa Maria das Barreiras	2017	52.7%	59.5%	45.2%
Brazil	Santa Maria de Itabira	2000	81.5%	84.9%	77.5%
Brazil	Santa Maria de Itabira	2017	82.0%	85.3%	78.1%
Brazil	Santa Maria de Jetibá	2000	81.9%	84.4%	79.4%
Brazil	Santa Maria de Jetibá	2017	82.4%	84.9%	80.1%
Brazil	Santa Maria do Cambucá	2000	66.6%	70.1%	63.0%
Brazil	Santa Maria do Cambucá	2017	67.5%	71.0%	64.0%
Brazil	Santa Maria do Herval	2000	82.9%	85.6%	80.1%

Country	Second Administrative Unit	Year	Mean	Upper	Lower
Brazil	Santa Maria do Herval	2017	83.4%	86.0%	80.7%
Brazil	Santa Maria do Oeste	2000	83.6%	86.7%	80.1%
Brazil	Santa Maria do Oeste	2017	84.2%	86.9%	80.9%
Brazil	Santa Maria do Pará	2000	39.1%	43.7%	35.2%
Brazil	Santa Maria do Pará	2017	40.0%	44.6%	36.1%
Brazil	Santa Maria do Salto	2000	83.3%	87.8%	78.3%
Brazil	Santa Maria do Salto	2017	83.9%	88.3%	79.0%
Brazil	Santa Maria do Suaçuí	2000	81.8%	86.4%	76.5%
Brazil	Santa Maria do Suaçuí	2017	82.4%	86.9%	77.2%
Brazil	Santa Maria do Tocantins	2000	76.2%	81.4%	71.2%
Brazil	Santa Maria do Tocantins	2017	76.9%	82.0%	72.0%
Brazil	Santa Maria Madalena	2000	79.5%	82.5%	76.1%
Brazil	Santa Maria Madalena	2017	80.1%	82.9%	76.7%
Brazil	Santa Mariana	2000	83.7%	87.6%	79.5%
Brazil	Santa Mariana	2017	84.3%	88.0%	80.1%
Brazil	Santa Mercedes	2000	87.0%	89.8%	83.4%
Brazil	Santa Mercedes	2017	87.4%	90.2%	83.9%
Brazil	Santa Mônica	2000	88.1%	90.3%	85.4%
Brazil	Santa Mônica	2017	88.5%	90.7%	85.9%
Brazil	Santa Quitéria	2000	70.3%	74.5%	66.2%
Brazil	Santa Quitéria	2017	71.1%	75.2%	67.1%
Brazil	Santa Quitéria do Maranhão	2000	60.9%	65.2%	55.9%
Brazil	Santa Quitéria do Maranhão	2017	61.8%	66.1%	56.9%
Brazil	Santa Rita	2000	60.1%	64.6%	55.7%
Brazil	Santa Rita	2000	67.4%	70.1%	64.6%
Brazil	Santa Rita	2017	61.1%	65.5%	56.7%
Brazil	Santa Rita	2017	68.2%	70.8%	65.4%
Brazil	Santa Rita de Araguaia	2000	74.9%	80.5%	68.3%
Brazil	Santa Rita de Araguaia	2017	75.6%	81.1%	69.1%
Brazil	Santa Rita de Caldas	2000	89.8%	91.8%	87.1%
Brazil	Santa Rita de Caldas	2017	90.2%	92.1%	87.5%
Brazil	Santa Rita de Cássia	2000	70.9%	77.7%	63.0%
Brazil	Santa Rita de Cássia	2017	71.7%	78.4%	63.9%

Country	Second Administrative Unit	Year	Mean	Upper	Lower
Brazil	Santa Rita de Jacutinga	2000	81.1%	84.4%	77.5%
Brazil	Santa Rita de Jacutinga	2017	81.7%	84.9%	78.1%
Brazil	Santa Rita de Minas	2000	83.0%	86.4%	78.7%
Brazil	Santa Rita de Minas	2017	83.5%	86.9%	79.4%
Brazil	Santa Rita do Ibitipoca	2000	87.4%	89.9%	84.5%
Brazil	Santa Rita do Ibitipoca	2017	87.9%	90.4%	85.1%
Brazil	Santa Rita do Novo destino	2000	80.6%	84.6%	76.0%
Brazil	Santa Rita do Novo destino	2017	81.2%	85.1%	76.7%
Brazil	Santa Rita do Oeste	2000	86.8%	91.1%	82.9%
Brazil	Santa Rita do Oeste	2017	87.3%	91.4%	83.4%
Brazil	Santa Rita do Pardo	2000	82.3%	85.7%	78.7%
Brazil	Santa Rita do Pardo	2017	82.9%	86.1%	79.3%
Brazil	Santa Rita do Passa Quatro	2000	91.8%	93.6%	89.4%
Brazil	Santa Rita do Passa Quatro	2017	92.1%	93.8%	89.7%
Brazil	Santa Rita do Sapucaí	2000	82.9%	86.3%	78.8%
Brazil	Santa Rita do Sapucaí	2017	83.5%	86.8%	79.4%
Brazil	Santa Rita do Tocantins	2000	74.9%	79.0%	71.0%
Brazil	Santa Rita do Tocantins	2017	75.6%	79.6%	71.7%
Brazil	Santa Rita Itueto	2000	79.0%	82.7%	75.0%
Brazil	Santa Rita Itueto	2017	79.6%	83.3%	75.7%
Brazil	Santa Rosa	2000	82.3%	86.8%	77.2%
Brazil	Santa Rosa	2017	83.4%	87.6%	78.6%
Brazil	Santa Rosa da Serra	2000	81.5%	86.4%	75.0%
Brazil	Santa Rosa da Serra	2017	82.1%	86.8%	75.8%
Brazil	Santa Rosa de Goiás	2000	77.4%	80.9%	73.6%
Brazil	Santa Rosa de Goiás	2017	78.0%	81.4%	74.3%
Brazil	Santa Rosa de Lima	2000	75.2%	79.3%	70.3%
Brazil	Santa Rosa de Lima	2000	74.7%	76.9%	72.1%
Brazil	Santa Rosa de Lima	2017	75.4%	77.6%	72.9%
Brazil	Santa Rosa de Lima	2017	76.3%	80.2%	71.5%
Brazil	Santa Rosa de Viterbo	2000	92.1%	94.0%	89.7%
Brazil	Santa Rosa de Viterbo	2017	93.0%	94.7%	90.8%

Country	Second Administrative Unit	Year	Mean	Upper	Lower
Brazil	Santa Rosa do Piauí	2000	60.5%	65.2%	55.2%
Brazil	Santa Rosa do Piauí	2017	61.4%	66.1%	56.1%
Brazil	Santa Rosa do Purus	2000	42.9%	48.9%	36.4%
Brazil	Santa Rosa do Purus	2017	43.9%	49.9%	37.3%
Brazil	Santa Rosa do Sul	2000	75.0%	80.0%	69.8%
Brazil	Santa Rosa do Sul	2017	75.7%	80.6%	70.6%
Brazil	Santa Rosa do Tocantins	2000	76.9%	81.5%	71.4%
Brazil	Santa Rosa do Tocantins	2017	77.7%	82.1%	72.2%
Brazil	Santa Salete	2000	88.1%	91.7%	84.5%
Brazil	Santa Salete	2017	88.5%	92.0%	85.0%
Brazil	Santa Teresa	2000	80.4%	83.0%	77.5%
Brazil	Santa Teresa	2017	81.1%	83.5%	78.2%
Brazil	Santa Teresinha	2000	79.3%	83.4%	74.5%
Brazil	Santa Teresinha	2000	69.1%	72.9%	64.7%
Brazil	Santa Teresinha	2017	80.0%	84.0%	75.2%
Brazil	Santa Teresinha	2017	69.9%	73.7%	65.6%
Brazil	Santa Tereza	2000	79.3%	82.8%	75.9%
Brazil	Santa Tereza	2017	79.9%	83.3%	76.6%
Brazil	Santa Tereza de Goiás	2000	76.8%	82.2%	70.6%
Brazil	Santa Tereza de Goiás	2017	77.5%	82.8%	71.4%
Brazil	Santa Tereza do Oeste	2000	85.9%	88.8%	82.8%
Brazil	Santa Tereza do Oeste	2017	86.4%	89.2%	83.4%
Brazil	Santa Tereza do Tocantins	2000	74.8%	79.3%	69.6%
Brazil	Santa Tereza do Tocantins	2017	75.5%	79.9%	70.4%
Brazil	Santa Terezinha	2000	71.4%	75.6%	67.2%
Brazil	Santa Terezinha	2000	75.9%	80.3%	71.0%
Brazil	Santa Terezinha	2000	64.1%	71.7%	55.2%
Brazil	Santa Terezinha	2017	76.5%	80.9%	71.8%
Brazil	Santa Terezinha	2017	72.2%	76.3%	68.1%
Brazil	Santa Terezinha	2017	65.0%	72.4%	56.2%
Brazil	Santa Terezinha de Goiás	2000	76.4%	81.2%	70.3%
Brazil	Santa Terezinha de Goiás	2017	77.0%	81.8%	71.1%
Brazil	Santa Terezinha de Itaipu	2000	82.7%	86.7%	78.2%
Brazil	Santa Terezinha de Itaipu	2017	83.3%	87.1%	78.8%

Country	Second Administrative Unit	Year	Mean	Upper	Lower
Brazil	Santa Terezinha do Progresso	2000	78.7%	82.9%	74.6%
Brazil	Santa Terezinha do Progresso	2017	79.4%	83.5%	75.4%
Brazil	Santa Terezinha do Tocantins	2000	73.6%	77.3%	69.3%
Brazil	Santa Terezinha do Tocantins	2017	74.4%	78.0%	70.2%
Brazil	Santa Vitória	2000	80.6%	85.3%	76.0%
Brazil	Santa Vitória	2017	81.2%	85.8%	76.7%
Brazil	Santa Vitória do Palmar	2000	80.2%	88.5%	69.9%
Brazil	Santa Vitória do Palmar	2017	80.8%	88.9%	70.7%
Brazil	Santaluz	2000	77.0%	81.4%	72.1%
Brazil	Santaluz	2017	77.7%	82.0%	72.9%
Brazil	Santana	2000	75.6%	79.4%	71.5%
Brazil	Santana	2000	44.1%	46.2%	42.0%
Brazil	Santana	2000	76.5%	83.9%	68.6%
Brazil	Santana	2017	45.1%	47.2%	43.0%
Brazil	Santana	2017	76.3%	80.0%	72.2%
Brazil	Santana	2017	77.2%	84.4%	69.4%
Brazil	Santana da Boa Vista	2000	81.4%	85.6%	76.6%
Brazil	Santana da Boa Vista	2017	82.0%	86.0%	77.3%
Brazil	Santana da Ponte Pensa	2000	87.4%	91.2%	83.7%
Brazil	Santana da Ponte Pensa	2017	87.9%	91.5%	84.2%
Brazil	Santana da Vargem	2000	83.0%	86.4%	78.9%
Brazil	Santana da Vargem	2017	83.5%	86.9%	79.5%
Brazil	Santana de Cataguases	2000	79.6%	83.2%	75.7%
Brazil	Santana de Cataguases	2017	80.2%	83.7%	76.4%
Brazil	Santana de Mangueira	2000	67.8%	72.0%	63.5%
Brazil	Santana de Mangueira	2017	68.7%	72.8%	64.4%
Brazil	Santana de Parnaíba	2000	93.9%	94.7%	92.9%
Brazil	Santana de Parnaíba	2017	94.1%	94.9%	93.1%
Brazil	Santana de Pirapama	2000	81.3%	85.3%	75.9%
Brazil	Santana de Pirapama	2017	81.9%	85.8%	76.7%
Brazil	Santana do Acaraú	2000	69.0%	73.1%	65.3%
Brazil	Santana do Acaraú	2017	69.9%	73.8%	66.2%
Brazil	Santana do Araguaia	2000	57.5%	64.3%	49.8%
Brazil	Santana do Araguaia	2017	58.5%	65.3%	50.8%

Country	Second Administrative Unit	Year	Mean	Upper	Lower
Brazil	Santana do Cariri	2000	69.3%	73.1%	65.2%
Brazil	Santana do Cariri	2017	70.2%	73.9%	66.1%
Brazil	Santana do deserto	2000	78.4%	81.8%	74.8%
Brazil	Santana do deserto	2017	79.0%	82.4%	75.5%
Brazil	Santana do Garambéu	2000	81.6%	85.0%	77.4%
Brazil	Santana do Garambéu	2017	82.2%	85.5%	78.1%
Brazil	Santana do Ipanema	2000	62.2%	65.1%	59.1%
Brazil	Santana do Ipanema	2017	63.1%	66.0%	60.0%
Brazil	Santana do Itararé	2000	89.2%	91.9%	86.1%
Brazil	Santana do Itararé	2017	89.6%	92.1%	86.5%
Brazil	Santana do Jacaré	2000	81.0%	84.7%	76.0%
Brazil	Santana do Jacaré	2017	81.6%	85.2%	76.7%
Brazil	Santana do Livramento	2000	82.6%	88.0%	75.5%
Brazil	Santana do Livramento	2017	83.2%	88.5%	76.2%
Brazil	Santana do Manhuaçu	2000	81.0%	84.5%	76.8%
Brazil	Santana do Manhuaçu	2017	81.6%	85.0%	77.5%
Brazil	Santana do Maranhão	2000	60.8%	65.4%	55.3%
Brazil	Santana do Maranhão	2017	61.8%	66.3%	56.2%
Brazil	Santana do Matos	2000	81.3%	84.0%	78.1%
Brazil	Santana do Matos	2017	82.0%	84.6%	78.9%
Brazil	Santana do Mundaú	2000	60.1%	63.5%	56.7%
Brazil	Santana do Mundaú	2017	61.1%	64.4%	57.6%
Brazil	Santana do Paraíso	2000	79.1%	83.0%	74.4%
Brazil	Santana do Paraíso	2017	79.8%	83.7%	75.3%
Brazil	Santana do Piauí	2000	61.9%	66.9%	57.3%
Brazil	Santana do Piauí	2017	62.9%	67.8%	58.3%
Brazil	Santana do Riacho	2000	82.8%	86.0%	78.7%
Brazil	Santana do Riacho	2017	83.4%	86.5%	79.5%
Brazil	Santana do São Francisco	2000	68.0%	70.7%	65.2%
Brazil	Santana do São Francisco	2017	68.9%	71.5%	66.1%
Brazil	Santana dos Garrotes	2000	66.9%	70.9%	62.5%

Country	Second Administrative Unit	Year	Mean	Upper	Lower
Brazil	Santana dos Garrotes	2017	67.8%	71.7%	63.4%
Brazil	Santana dos Montes	2000	81.1%	84.6%	77.1%
Brazil	Santana dos Montes	2017	81.7%	85.1%	77.8%
Brazil	Santanópolis	2000	78.0%	81.6%	74.3%
Brazil	Santanópolis	2017	78.6%	82.1%	75.0%
Brazil	Santarém	2000	74.9%	78.8%	71.4%
Brazil	Santarém	2000	39.3%	45.3%	34.2%
Brazil	Santarém	2017	40.3%	46.3%	35.1%
Brazil	Santarém	2017	75.6%	79.4%	72.2%
Brazil	Santarém Novo	2000	36.2%	40.8%	31.8%
Brazil	Santarém Novo	2017	37.1%	41.8%	32.7%
Brazil	Santiago	2000	83.7%	87.6%	78.6%
Brazil	Santiago	2017	84.2%	88.0%	79.2%
Brazil	Santiago do Sul	2000	77.4%	82.0%	73.0%
Brazil	Santiago do Sul	2017	78.1%	82.6%	73.7%
Brazil	Santo Afonso	2000	68.5%	73.8%	61.7%
Brazil	Santo Afonso	2017	69.3%	74.5%	62.6%
Brazil	Santo Amaro	2000	74.7%	77.7%	71.0%
Brazil	Santo Amaro	2017	75.4%	78.4%	71.8%
Brazil	Santo Amaro da Imperatriz	2000	72.0%	77.1%	66.7%
Brazil	Santo Amaro da Imperatriz	2017	72.8%	77.8%	67.5%
Brazil	Santo Amaro das Brotas	2000	73.1%	75.3%	70.6%
Brazil	Santo Amaro das Brotas	2017	73.9%	76.0%	71.4%
Brazil	Santo Amaro do Maranhão	2000	59.9%	66.2%	52.3%
Brazil	Santo Amaro do Maranhão	2017	60.8%	67.0%	53.3%
Brazil	Santo Anastácio	2000	89.5%	92.0%	86.8%
Brazil	Santo Anastácio	2017	89.9%	92.2%	87.2%
Brazil	Santo André	2000	69.1%	73.5%	64.9%
Brazil	Santo André	2000	94.7%	95.5%	94.0%
Brazil	Santo André	2017	70.0%	74.3%	65.8%
Brazil	Santo André	2017	94.9%	95.6%	94.2%
Brazil	Santo Ángelo	2000	82.7%	86.4%	78.2%
Brazil	Santo Ángelo	2017	83.2%	86.9%	78.8%
Brazil	Santo Antônio	2000	79.2%	81.5%	76.9%
Brazil	Santo Antônio	2017	79.9%	82.1%	77.5%
Brazil	Santo Antônio da Alegria	2000	90.3%	93.1%	87.1%
Brazil	Santo Antônio da Alegria	2017	90.7%	93.3%	87.5%
Brazil	Santo Antônio da Barra	2000	76.8%	81.2%	71.5%
Brazil	Santo Antônio da Barra	2017	77.5%	81.8%	72.3%
Brazil	Santo Antônio da Patrulha	2000	81.5%	84.4%	78.0%
Brazil	Santo Antônio da Patrulha	2017	82.1%	84.9%	78.6%

Country	Second Administrative Unit	Year	Mean	Upper	Lower
Brazil	Santo Antonio da Platina	2000	89.5%	91.8%	86.6%
Brazil	Santo Antonio da Platina	2017	89.9%	92.1%	87.0%
Brazil	Santo Antônio das Missões	2000	82.5%	87.5%	77.0%
Brazil	Santo Antônio das Missões	2017	83.1%	87.9%	77.7%
Brazil	Santo Antônio de Goiás	2000	77.9%	80.3%	75.7%
Brazil	Santo Antônio de Goiás	2017	78.5%	80.8%	76.4%
Brazil	Santo Antônio de Jesus	2000	77.4%	81.0%	72.6%
Brazil	Santo Antônio de Jesus	2017	78.1%	81.6%	73.4%
Brazil	Santo Antônio de Lisboa	2000	60.2%	64.9%	54.8%
Brazil	Santo Antônio de Lisboa	2017	61.1%	65.8%	55.8%
Brazil	Santo Antônio de Pádua	2000	76.9%	80.7%	72.5%
Brazil	Santo Antônio de Pádua	2017	77.6%	81.3%	73.3%
Brazil	Santo Antônio de Posse	2000	89.1%	91.8%	85.4%
Brazil	Santo Antônio de Posse	2017	89.5%	92.1%	85.9%
Brazil	Santo Antônio do Amparo	2000	80.7%	84.3%	76.6%
Brazil	Santo Antônio do Amparo	2017	81.3%	84.8%	77.3%
Brazil	Santo Antônio do Aracanguá	2000	92.0%	93.4%	90.2%
Brazil	Santo Antônio do Aracanguá	2017	92.3%	93.6%	90.6%
Brazil	Santo Antônio do Aventureiro	2000	80.5%	82.9%	77.2%
Brazil	Santo Antônio do Aventureiro	2017	81.1%	83.5%	77.8%
Brazil	Santo Antonio do Caiuá	2000	85.3%	88.3%	81.4%
Brazil	Santo Antonio do Caiuá	2017	85.8%	88.7%	82.0%
Brazil	Santo Antônio do Descoberto	2000	87.9%	89.4%	86.4%
Brazil	Santo Antônio do Descoberto	2017	88.5%	90.0%	87.0%
Brazil	Santo Antônio do Grama	2000	80.4%	83.8%	76.6%
Brazil	Santo Antônio do Grama	2017	81.0%	84.3%	77.3%
Brazil	Santo Antônio do Içá	2000	57.3%	64.4%	49.9%
Brazil	Santo Antônio do Içá	2017	58.3%	65.3%	51.0%
Brazil	Santo Antônio do Jacinto	2000	81.7%	84.4%	78.8%
Brazil	Santo Antônio do Jacinto	2017	82.3%	84.9%	79.5%

Country	Second Administrative Unit	Year	Mean	Upper	Lower
Brazil	Santo Antônio do Leverger	2000	67.9%	71.7%	64.4%
Brazil	Santo Antônio do Leverger	2017	69.3%	73.0%	65.8%
Brazil	Santo Antônio do Monte	2000	83.0%	87.4%	78.4%
Brazil	Santo Antônio do Monte	2017	83.5%	87.8%	79.1%
Brazil	Santo Antônio do Palma	2000	81.1%	84.5%	76.4%
Brazil	Santo Antônio do Palma	2017	81.7%	85.0%	77.1%
Brazil	Santo Antonio do Paraíso	2000	86.4%	88.9%	83.6%
Brazil	Santo Antonio do Paraíso	2017	86.8%	89.3%	84.1%
Brazil	Santo Antônio do Pinhal	2000	91.3%	93.0%	89.4%
Brazil	Santo Antônio do Pinhal	2017	91.6%	93.2%	89.7%
Brazil	Santo Antônio do Planalto	2000	81.7%	85.3%	77.3%
Brazil	Santo Antônio do Planalto	2017	82.3%	85.8%	78.0%
Brazil	Santo Antônio do Retiro	2000	81.1%	85.2%	76.7%
Brazil	Santo Antônio do Retiro	2017	81.7%	85.7%	77.4%
Brazil	Santo Antônio do Tauá	2000	35.6%	38.8%	32.4%
Brazil	Santo Antônio do Tauá	2017	36.6%	39.7%	33.2%
Brazil	Santo Antônio dos Lopes	2000	60.1%	65.5%	55.5%
Brazil	Santo Antônio dos Lopes	2017	61.0%	66.3%	56.4%
Brazil	Santo Antônio dos Milagres	2000	62.0%	66.7%	58.1%
Brazil	Santo Antônio dos Milagres	2017	62.9%	67.6%	59.1%
Brazil	Santo Augusto	2000	83.7%	87.3%	79.3%
Brazil	Santo Augusto	2017	84.2%	87.7%	80.0%
Brazil	Santo Cristo	2000	82.1%	86.8%	76.5%
Brazil	Santo Cristo	2017	82.7%	87.3%	77.2%
Brazil	Santo Estêvão	2000	77.3%	81.1%	73.3%
Brazil	Santo Estêvão	2017	78.1%	81.8%	74.2%
Brazil	Santo Expedito	2000	88.8%	91.5%	85.9%
Brazil	Santo Expedito	2017	89.2%	91.8%	86.4%
Brazil	Santo Expedito do Sul	2000	77.9%	81.7%	73.4%
Brazil	Santo Expedito do Sul	2017	78.6%	82.2%	74.1%
Brazil	Santo Hipólito	2000	81.5%	86.6%	74.9%
Brazil	Santo Hipólito	2017	82.0%	87.0%	75.6%
Brazil	Santo Inácio	2000	85.6%	88.5%	81.9%
Brazil	Santo Inácio	2017	86.1%	88.9%	82.5%
Brazil	Santo Inácio do Piauí	2000	60.5%	65.8%	54.7%

Country	Second Administrative Unit	Year	Mean	Upper	Lower
Brazil	Santo Inácio do Piauí	2017	61.4%	66.7%	55.6%
Brazil	Santópolis do Aguapeí	2000	91.7%	93.8%	89.3%
Brazil	Santópolis do Aguapeí	2017	92.0%	94.1%	89.7%
Brazil	Santos	2000	87.3%	89.4%	85.1%
Brazil	Santos	2017	87.7%	89.8%	85.6%
Brazil	Santos Dumont	2000	80.7%	84.2%	76.7%
Brazil	Santos Dumont	2017	81.3%	84.7%	77.4%
Brazil	São Antonio de Sudoeste	2000	82.2%	86.4%	77.9%
Brazil	São Antonio de Sudoeste	2017	82.7%	86.8%	78.6%
Brazil	São Benedito	2000	69.6%	73.7%	65.6%
Brazil	São Benedito	2017	70.4%	74.4%	66.5%
Brazil	São Benedito do Rio Preto	2000	61.8%	67.2%	55.6%
Brazil	São Benedito do Rio Preto	2017	62.7%	68.0%	56.6%
Brazil	São Benedito do Sul	2000	64.6%	68.0%	61.4%
Brazil	São Benedito do Sul	2017	65.5%	68.8%	62.3%
Brazil	São Bentinho	2000	69.2%	72.7%	65.3%
Brazil	São Bentinho	2017	70.1%	73.5%	66.2%
Brazil	São Bento	2000	60.4%	65.4%	55.2%
Brazil	São Bento	2000	74.3%	77.7%	70.3%
Brazil	São Bento	2017	61.3%	66.2%	56.1%
Brazil	São Bento	2017	75.0%	78.3%	71.1%
Brazil	São Bento Abade	2000	82.7%	86.2%	77.8%
Brazil	São Bento Abade	2017	84.6%	87.7%	80.1%
Brazil	São Bento do Norte	2000	83.7%	87.2%	78.9%
Brazil	São Bento do Norte	2017	84.2%	87.6%	79.5%
Brazil	São Bento do Sapucaí	2000	90.6%	92.5%	88.4%
Brazil	São Bento do Sapucaí	2017	90.9%	92.8%	88.8%
Brazil	São Bento do Sul	2000	80.9%	84.2%	77.9%
Brazil	São Bento do Sul	2017	81.5%	84.8%	78.6%
Brazil	São Bento do Tocantins	2000	70.7%	74.9%	65.8%
Brazil	São Bento do Tocantins	2017	71.5%	75.6%	66.6%
Brazil	São Bento do Trairí	2000	79.8%	82.5%	77.0%
Brazil	São Bento do Trairí	2017	80.5%	83.1%	77.7%
Brazil	São Bento do Una	2000	67.8%	71.0%	64.7%
Brazil	São Bento do Una	2017	68.6%	71.8%	65.6%
Brazil	São Bernardino	2000	79.9%	84.2%	75.6%

Country	Second Administrative Unit	Year	Mean	Upper	Lower
Brazil	São Bernardino	2017	80.5%	84.8%	76.3%
Brazil	São Bernardo	2000	59.8%	64.3%	54.7%
Brazil	São Bernardo	2017	60.7%	65.2%	55.7%
Brazil	São Bernardo do Campo	2000	94.5%	95.3%	93.7%
Brazil	São Bernardo do Campo	2017	94.7%	95.5%	93.9%
Brazil	São Bonifácio	2000	75.9%	80.1%	70.8%
Brazil	São Bonifácio	2017	76.7%	80.8%	71.7%
Brazil	São Borja	2000	81.6%	87.0%	75.0%
Brazil	São Borja	2017	82.1%	87.4%	75.6%
Brazil	São Brás	2000	67.3%	70.2%	64.2%
Brazil	São Brás	2017	68.2%	71.0%	65.1%
Brazil	São Brás do Suaçuí	2000	81.0%	84.8%	77.6%
Brazil	São Brás do Suaçuí	2017	81.6%	85.3%	78.3%
Brazil	São Braz do Piauí	2000	64.5%	70.9%	57.6%
Brazil	São Braz do Piauí	2017	65.4%	71.7%	58.6%
Brazil	São Caetano de Odivelas	2000	34.7%	39.8%	30.4%
Brazil	São Caetano de Odivelas	2017	35.6%	40.7%	31.3%
Brazil	São Caetano do Sul	2000	94.8%	95.6%	94.2%
Brazil	São Caetano do Sul	2017	95.0%	95.7%	94.4%
Brazil	São Caitano	2000	67.9%	70.9%	64.7%
Brazil	São Caitano	2017	68.8%	71.7%	65.6%
Brazil	São Carlos	2000	93.7%	95.1%	91.8%
Brazil	São Carlos	2000	76.5%	80.1%	72.1%
Brazil	São Carlos	2017	77.1%	80.8%	72.9%
Brazil	São Carlos	2017	93.9%	95.3%	92.2%
Brazil	São Carlos do Ivaí	2000	83.6%	86.5%	80.3%
Brazil	São Carlos do Ivaí	2017	84.1%	86.9%	80.9%
Brazil	São Cristóvão	2000	75.7%	77.3%	73.8%
Brazil	São Cristóvão	2017	76.4%	78.0%	74.5%
Brazil	São Cristóvão do Sul	2000	76.9%	81.3%	72.1%
Brazil	São Cristóvão do Sul	2017	77.6%	81.9%	72.9%
Brazil	São Desidério	2000	75.7%	82.0%	68.9%
Brazil	São Desidério	2017	76.4%	82.5%	69.7%
Brazil	São Domingos	2000	78.6%	83.8%	72.8%
Brazil	São Domingos	2000	77.9%	82.5%	72.9%
Brazil	São Domingos	2000	78.7%	82.5%	73.9%
Brazil	São Domingos	2000	77.7%	79.9%	75.5%
Brazil	São Domingos	2017	79.3%	84.3%	73.6%
Brazil	São Domingos	2017	78.6%	83.1%	73.6%
Brazil	São Domingos	2017	78.4%	80.5%	76.2%
Brazil	São Domingos	2017	79.4%	83.0%	74.6%
Brazil	São Domingos das Dores	2000	81.4%	85.1%	77.1%
Brazil	São Domingos das Dores	2017	82.1%	85.7%	78.0%
Brazil	São Domingos de Pombal	2000	69.6%	73.3%	65.9%

Country	Second Administrative Unit	Year	Mean	Upper	Lower
Brazil	São Domingos de Pombal	2017	70.4%	74.1%	66.8%
Brazil	São Domingos do Araguaia	2000	53.0%	58.3%	47.9%
Brazil	São Domingos do Araguaia	2017	54.0%	59.2%	48.8%
Brazil	São Domingos do Azeitão	2000	62.0%	69.4%	53.2%
Brazil	São Domingos do Azeitão	2017	64.9%	72.0%	56.3%
Brazil	São Domingos do Capim	2000	37.6%	41.8%	33.9%
Brazil	São Domingos do Capim	2017	38.5%	42.7%	34.7%
Brazil	São Domingos do Cariri	2000	66.3%	70.9%	61.5%
Brazil	São Domingos do Cariri	2017	67.2%	71.7%	62.4%
Brazil	São Domingos do Maranhão	2000	62.5%	68.3%	56.9%
Brazil	São Domingos do Maranhão	2017	63.4%	69.2%	57.9%
Brazil	São Domingos do Norte	2000	78.8%	81.6%	75.1%
Brazil	São Domingos do Norte	2017	79.4%	82.2%	75.7%
Brazil	São Domingos do Prata	2000	82.0%	85.5%	78.4%
Brazil	São Domingos do Prata	2017	82.5%	86.0%	79.1%
Brazil	São Domingos do Sul	2000	80.6%	84.1%	76.4%
Brazil	São Domingos do Sul	2017	81.2%	84.6%	77.1%
Brazil	São Felipe	2000	76.9%	80.6%	72.5%
Brazil	São Felipe	2017	77.5%	81.2%	73.3%
Brazil	São Felipe d'Oeste	2000	30.6%	34.6%	26.3%
Brazil	São Felipe d'Oeste	2017	31.1%	35.2%	26.8%
Brazil	São Félix	2000	76.0%	79.5%	72.1%
Brazil	São Félix	2017	76.8%	80.2%	73.0%
Brazil	São Félix de Balsas	2000	60.8%	68.9%	52.7%
Brazil	São Félix de Balsas	2017	61.7%	69.7%	53.8%
Brazil	São Félix de Minas	2000	81.2%	86.3%	76.5%
Brazil	São Félix de Minas	2017	81.7%	86.7%	77.2%
Brazil	São Félix do Coribe	2000	78.5%	84.9%	71.0%
Brazil	São Félix do Coribe	2017	79.1%	85.4%	71.8%
Brazil	São Félix do Piauí	2000	58.4%	63.4%	52.9%
Brazil	São Félix do Piauí	2017	59.3%	64.3%	53.9%
Brazil	São Félix do Tocantins	2000	72.2%	79.7%	64.5%
Brazil	São Félix do Tocantins	2017	73.1%	80.4%	65.4%

Country	Second Administrative Unit	Year	Mean	Upper	Lower
Brazil	São Félix do Xingu	2000	37.9%	46.1%	30.9%
Brazil	São Félix do Xingu	2017	38.9%	47.1%	31.8%
Brazil	São Félix Xingu	2000	69.2%	76.4%	60.6%
Brazil	São Félix Xingu	2017	70.1%	77.1%	61.7%
Brazil	São Fernando	2000	76.4%	79.8%	72.6%
Brazil	São Fernando	2017	77.1%	80.4%	73.3%
Brazil	São Fidélis	2000	76.1%	79.9%	71.9%
Brazil	São Fidélis	2017	76.8%	80.6%	72.7%
Brazil	São Francisco	2000	74.7%	77.8%	71.1%
Brazil	São Francisco	2000	88.5%	91.8%	84.9%
Brazil	São Francisco	2000	81.8%	87.3%	76.0%
Brazil	São Francisco	2000	71.6%	74.3%	68.5%
Brazil	São Francisco	2017	75.3%	78.4%	71.8%
Brazil	São Francisco	2017	82.4%	87.7%	76.7%
Brazil	São Francisco	2017	72.4%	75.1%	69.4%
Brazil	São Francisco	2017	88.9%	92.1%	85.4%
Brazil	São Francisco de Assis	2000	81.2%	85.5%	75.2%
Brazil	São Francisco de Assis	2017	81.8%	86.0%	75.9%
Brazil	São Francisco de Assis do Piauí	2000	62.6%	68.0%	55.9%
Brazil	São Francisco de Assis do Piauí	2017	63.5%	68.8%	57.0%
Brazil	São Francisco de Goias	2000	77.8%	81.3%	74.1%
Brazil	São Francisco de Goias	2017	78.5%	81.9%	74.8%
Brazil	São Francisco de Itabapoana	2000	74.3%	78.0%	69.7%
Brazil	São Francisco de Itabapoana	2017	75.0%	78.7%	70.5%
Brazil	São Francisco de Oliveira	2000	80.8%	84.9%	76.0%
Brazil	São Francisco de Oliveira	2017	81.4%	85.4%	76.7%
Brazil	São Francisco de Paula	2000	80.7%	83.9%	77.3%
Brazil	São Francisco de Paula	2017	81.3%	84.4%	78.0%
Brazil	São Francisco de Sales	2000	87.4%	90.5%	83.6%
Brazil	São Francisco de Sales	2017	87.9%	90.8%	84.1%
Brazil	São Francisco do Brejão	2000	65.7%	70.5%	60.4%
Brazil	São Francisco do Brejão	2017	66.6%	71.3%	61.3%
Brazil	São Francisco do Conde	2000	73.9%	77.0%	70.9%
Brazil	São Francisco do Conde	2017	74.7%	77.8%	71.8%
Brazil	São Francisco do Glória	2000	81.2%	84.6%	77.3%
Brazil	São Francisco do Glória	2017	81.8%	85.1%	78.1%

Country	Second Administrative Unit	Year	Mean	Upper	Lower
Brazil	São Francisco do Guaporé	2000	29.1%	33.4%	25.0%
Brazil	São Francisco do Guaporé	2017	29.9%	34.3%	25.8%
Brazil	São Francisco do Maranhão	2000	60.0%	64.5%	55.0%
Brazil	São Francisco do Maranhão	2017	61.5%	65.9%	56.5%
Brazil	São Francisco do Oeste	2000	75.8%	79.2%	71.9%
Brazil	São Francisco do Oeste	2017	76.5%	79.9%	72.7%
Brazil	São Francisco do Pará	2000	36.8%	40.4%	33.5%
Brazil	São Francisco do Pará	2017	38.1%	41.7%	34.8%
Brazil	São Francisco do Piauí	2000	60.1%	65.5%	53.9%
Brazil	São Francisco do Piauí	2017	61.0%	66.5%	55.0%
Brazil	São Francisco do Sul	2000	72.7%	76.8%	67.7%
Brazil	São Francisco do Sul	2017	73.6%	77.6%	68.7%
Brazil	São Gabriel	2000	76.2%	82.0%	69.8%
Brazil	São Gabriel	2000	83.0%	87.5%	78.2%
Brazil	São Gabriel	2017	76.9%	82.5%	70.6%
Brazil	São Gabriel	2017	83.6%	87.9%	78.8%
Brazil	São Gabriel da Palha	2000	77.7%	80.7%	73.6%
Brazil	São Gabriel da Palha	2017	78.3%	81.3%	74.4%
Brazil	São Gabriel de Cahoeira	2000	59.2%	69.1%	48.9%
Brazil	São Gabriel de Cahoeira	2017	60.2%	69.9%	49.9%
Brazil	São Gabriel do Oeste	2000	80.3%	84.8%	75.2%
Brazil	São Gabriel do Oeste	2017	81.0%	85.3%	75.9%
Brazil	São Geraldo	2000	80.7%	83.8%	76.4%
Brazil	São Geraldo	2017	81.3%	84.3%	77.1%
Brazil	São Geraldo da Piedade	2000	80.1%	84.4%	75.8%
Brazil	São Geraldo da Piedade	2017	80.7%	84.9%	76.5%
Brazil	São Geraldo do Araguaia	2000	62.0%	66.8%	57.1%
Brazil	São Geraldo do Araguaia	2017	62.7%	67.5%	57.9%
Brazil	São Geraldo do Baixio	2000	79.3%	83.4%	74.9%
Brazil	São Geraldo do Baixio	2017	79.9%	83.9%	75.7%
Brazil	São Gonçalo	2000	78.2%	79.9%	76.3%
Brazil	São Gonçalo	2017	78.9%	80.5%	77.0%
Brazil	São Gonçalo do Abaeté	2000	80.5%	86.2%	73.6%
Brazil	São Gonçalo do Abaeté	2017	81.1%	86.7%	74.3%
Brazil	São Gonçalo do Amarante	2000	82.3%	84.0%	80.4%

Country	Second Administrative Unit	Year	Mean	Upper	Lower
Brazil	São Gonçalo do Amarante	2000	68.5%	72.1%	64.9%
Brazil	São Gonçalo do Amarante	2017	83.0%	84.6%	81.2%
Brazil	São Gonçalo do Amarante	2017	69.4%	73.0%	65.9%
Brazil	São Gonçalo do Gurguéia	2000	66.9%	74.0%	59.4%
Brazil	São Gonçalo do Gurguéia	2017	67.8%	74.8%	60.4%
Brazil	São Gonçalo do Pará	2000	80.0%	84.4%	75.6%
Brazil	São Gonçalo do Pará	2017	80.6%	84.9%	76.4%
Brazil	São Gonçalo do Piauí	2000	60.5%	65.1%	55.0%
Brazil	São Gonçalo do Piauí	2017	61.4%	65.9%	55.9%
Brazil	São Gonçalo do Rio Abaixo	2000	81.3%	85.1%	77.8%
Brazil	São Gonçalo do Rio Abaixo	2017	81.8%	85.5%	78.4%
Brazil	São Gonçalo do Sapucaí	2000	85.5%	88.7%	81.9%
Brazil	São Gonçalo do Sapucaí	2017	86.0%	89.0%	82.5%
Brazil	São Gonçalo dos Campos	2000	77.8%	80.9%	74.6%
Brazil	São Gonçalo dos Campos	2017	78.5%	81.5%	75.4%
Brazil	São Gotardo	2000	81.3%	86.5%	74.5%
Brazil	São Gotardo	2017	81.9%	86.9%	75.2%
Brazil	São Jerônimo	2000	82.9%	85.7%	79.8%
Brazil	São Jerônimo	2017	83.4%	86.2%	80.5%
Brazil	São Jerônimo da Serra	2000	85.6%	88.6%	82.6%
Brazil	São Jerônimo da Serra	2017	86.1%	89.0%	83.1%
Brazil	São João	2000	68.1%	70.6%	65.2%
Brazil	São João	2000	82.4%	86.0%	78.0%
Brazil	São João	2017	68.9%	71.4%	66.1%
Brazil	São João	2017	83.0%	86.5%	78.7%
Brazil	São João Batista	2000	60.6%	65.4%	55.4%
Brazil	São João Batista	2000	70.2%	74.5%	65.5%
Brazil	São João Batista	2017	61.5%	66.3%	56.4%
Brazil	São João Batista	2017	71.0%	75.2%	66.3%
Brazil	São João Batista do Glória	2000	85.2%	89.1%	80.2%
Brazil	São João Batista do Glória	2017	85.6%	89.5%	80.8%
Brazil	São João d'Aliança	2000	86.1%	89.2%	81.9%
Brazil	São João d'Aliança	2017	87.1%	90.0%	83.1%
Brazil	São João da Baliza	2000	85.0%	88.0%	80.6%

Country	Second Administrative Unit	Year	Mean	Upper	Lower
Brazil	São João da Baliza	2017	85.5%	88.4%	81.1%
Brazil	São João da Barra	2000	74.5%	78.6%	69.3%
Brazil	São João da Barra	2017	76.0%	79.9%	71.0%
Brazil	São João da Boa Vista	2000	90.6%	92.8%	87.9%
Brazil	São João da Boa Vista	2017	90.9%	93.0%	88.4%
Brazil	São João da Canabrava	2000	61.7%	67.2%	56.8%
Brazil	São João da Canabrava	2017	62.6%	67.9%	57.7%
Brazil	São João da Fronteira	2000	66.0%	70.0%	61.5%
Brazil	São João da Fronteira	2017	67.0%	70.9%	62.5%
Brazil	São João da Lagoa	2000	81.3%	86.1%	74.1%
Brazil	São João da Lagoa	2017	81.9%	86.5%	74.9%
Brazil	São João da Mata	2000	87.6%	90.2%	84.2%
Brazil	São João da Mata	2017	88.0%	90.5%	84.7%
Brazil	São João da Paraúna	2000	76.8%	81.2%	72.1%
Brazil	São João da Paraúna	2017	77.4%	81.8%	72.9%
Brazil	São João da Ponta	2000	35.4%	40.1%	30.9%
Brazil	São João da Ponta	2017	36.3%	41.1%	31.7%
Brazil	São João da Ponte	2000	81.8%	86.5%	76.1%
Brazil	São João da Ponte	2017	82.4%	87.0%	76.8%
Brazil	São João da Serra	2000	60.0%	64.5%	54.7%
Brazil	São João da Serra	2017	60.9%	65.4%	55.7%
Brazil	São João da Urtiga	2000	77.9%	81.8%	73.8%
Brazil	São João da Urtiga	2017	78.6%	82.4%	74.6%
Brazil	São João da Varjota	2000	62.1%	67.0%	56.4%
Brazil	São João da Varjota	2017	63.0%	67.9%	57.4%
Brazil	São João das Duas Ponte	2000	89.6%	92.8%	86.5%
Brazil	São João das Duas Ponte	2017	90.0%	93.1%	86.9%
Brazil	São João das Missões	2000	80.0%	85.9%	72.7%
Brazil	São João das Missões	2017	80.6%	86.4%	73.5%
Brazil	São João de Iracema	2000	90.1%	92.9%	86.8%
Brazil	São João de Iracema	2017	90.4%	93.2%	87.2%

Country	Second Administrative Unit	Year	Mean	Upper	Lower
Brazil	São João de Meriti	2000	78.3%	79.5%	76.9%
Brazil	São João de Meriti	2017	78.9%	80.2%	77.6%
Brazil	São João de Pirabas	2000	36.5%	42.1%	31.3%
Brazil	São João de Pirabas	2017	37.2%	42.9%	32.0%
Brazil	São João del Rei	2000	82.8%	86.7%	78.7%
Brazil	São João del Rei	2017	83.3%	87.2%	79.3%
Brazil	São João do Araguaia	2000	49.4%	55.1%	44.8%
Brazil	São João do Araguaia	2017	50.3%	56.0%	45.7%
Brazil	São João do Arraial	2000	60.4%	64.8%	56.0%
Brazil	São João do Arraial	2017	61.3%	65.7%	57.0%
Brazil	São João do Belm	2000	69.5%	73.3%	65.8%
Brazil	São João do Belm	2017	70.3%	74.0%	66.7%
Brazil	São João do Belmonte	2000	66.3%	70.5%	61.8%
Brazil	São João do Belmonte	2017	67.1%	71.3%	62.7%
Brazil	São João do Caiuá	2000	85.0%	88.3%	81.2%
Brazil	São João do Caiuá	2017	85.5%	88.7%	81.8%
Brazil	São João do Cariri	2000	67.9%	72.6%	63.9%
Brazil	São João do Cariri	2017	68.8%	73.3%	64.8%
Brazil	São João do Carú	2000	56.4%	62.7%	50.0%
Brazil	São João do Carú	2017	57.4%	63.7%	51.0%
Brazil	São João do Itaperiú	2000	73.4%	76.5%	68.8%
Brazil	São João do Itaperiú	2017	74.2%	77.2%	69.7%
Brazil	São João do Ivaí	2000	82.2%	85.4%	78.6%
Brazil	São João do Ivaí	2017	82.8%	85.9%	79.2%
Brazil	São João do Jaguaribe	2000	72.7%	76.8%	68.0%
Brazil	São João do Jaguaribe	2017	73.5%	77.5%	68.9%
Brazil	São João do Manhuaçu	2000	81.8%	85.2%	78.0%
Brazil	São João do Manhuaçu	2017	82.4%	85.7%	78.7%
Brazil	São João do Manteninha	2000	79.5%	83.1%	75.3%
Brazil	São João do Manteninha	2017	80.2%	83.7%	76.0%
Brazil	São João do Oeste	2000	79.6%	83.8%	75.2%

Country	Second Administrative Unit	Year	Mean	Upper	Lower
Brazil	São João do Oeste	2017	80.2%	84.3%	75.9%
Brazil	São João do Oriente	2000	80.3%	84.5%	75.5%
Brazil	São João do Oriente	2017	80.9%	85.0%	76.3%
Brazil	São João do Pacuí	2000	81.4%	86.3%	74.8%
Brazil	São João do Pacuí	2017	82.0%	86.7%	75.5%
Brazil	São João do Paraíso	2000	80.2%	85.0%	73.7%
Brazil	São João do Paraíso	2000	71.9%	77.3%	65.5%
Brazil	São João do Paraíso	2017	80.8%	85.5%	74.4%
Brazil	São João do Paraíso	2017	72.8%	78.0%	66.5%
Brazil	São João do Pau d'Alho	2000	87.2%	90.1%	83.6%
Brazil	São João do Pau d'Alho	2017	87.6%	90.4%	84.1%
Brazil	São João do Polêsine	2000	81.8%	85.1%	77.0%
Brazil	São João do Polêsine	2017	82.4%	85.6%	77.7%
Brazil	São João do Rio do Peixe	2000	70.2%	73.9%	66.5%
Brazil	São João do Rio do Peixe	2017	71.0%	74.7%	67.4%
Brazil	São João do Sabugi	2000	71.9%	75.9%	67.5%
Brazil	São João do Sabugi	2017	72.6%	76.6%	68.3%
Brazil	São João do Soter	2000	59.9%	65.1%	55.0%
Brazil	São João do Soter	2017	60.9%	65.9%	56.0%
Brazil	São João do Sul	2000	76.9%	81.1%	71.6%
Brazil	São João do Sul	2017	77.6%	81.6%	72.4%
Brazil	São João do Tigre	2000	66.6%	70.4%	62.3%
Brazil	São João do Tigre	2017	67.4%	71.2%	63.2%
Brazil	São João do Triunfo	2000	80.7%	83.8%	76.9%
Brazil	São João do Triunfo	2017	81.3%	84.3%	77.6%
Brazil	São João dos Patos	2000	62.4%	68.9%	56.2%
Brazil	São João dos Patos	2017	63.4%	69.8%	57.2%
Brazil	São João Evangelista	2000	82.4%	86.6%	76.8%
Brazil	São João Evangelista	2017	83.0%	87.1%	77.5%
Brazil	São João Nepomuceno	2000	79.7%	82.9%	75.4%
Brazil	São João Nepomuceno	2017	80.3%	83.4%	76.1%

Country	Second Administrative Unit	Year	Mean	Upper	Lower
Brazil	São João Piaui	2000	60.8%	66.5%	54.6%
Brazil	São João Piaui	2017	61.7%	67.4%	55.6%
Brazil	São Joaquim	2000	81.5%	85.4%	77.1%
Brazil	São Joaquim	2017	82.1%	85.9%	77.7%
Brazil	São Joaquim da Barra	2000	90.5%	92.8%	87.6%
Brazil	São Joaquim da Barra	2017	90.8%	93.1%	88.0%
Brazil	São Joaquim de Bicas	2000	81.7%	84.8%	78.5%
Brazil	São Joaquim de Bicas	2017	82.3%	85.3%	79.2%
Brazil	São Joaquin do Monte	2000	68.0%	71.1%	65.0%
Brazil	São Joaquin do Monte	2017	68.9%	72.0%	65.9%
Brazil	São Jorge	2000	80.4%	84.2%	76.3%
Brazil	São Jorge	2017	81.0%	84.7%	77.0%
Brazil	São Jorge d'Oeste	2000	81.9%	85.6%	78.0%
Brazil	São Jorge d'Oeste	2017	82.5%	86.0%	78.6%
Brazil	São Jorge do Ivaí	2000	83.3%	86.4%	80.0%
Brazil	São Jorge do Ivaí	2017	83.9%	86.9%	80.6%
Brazil	São Jorge do Patrocínio	2000	82.9%	86.5%	78.8%
Brazil	São Jorge do Patrocínio	2017	83.4%	87.0%	79.4%
Brazil	São José	2000	72.6%	78.0%	66.8%
Brazil	São José	2017	73.4%	78.7%	67.7%
Brazil	São José da Barra	2000	83.5%	87.9%	78.1%
Brazil	São José da Barra	2017	84.1%	88.4%	78.8%
Brazil	São José da Bela Vista	2000	90.2%	92.5%	86.7%
Brazil	São José da Bela Vista	2017	90.5%	92.8%	87.1%
Brazil	São José da Boa Vista	2000	88.3%	91.0%	85.1%
Brazil	São José da Boa Vista	2017	88.7%	91.3%	85.6%
Brazil	São José da Coroa Grande	2000	64.3%	68.8%	59.0%
Brazil	São José da Coroa Grande	2017	63.4%	68.0%	58.0%
Brazil	São José da Lagoa Tapada	2000	70.1%	73.5%	65.9%
Brazil	São José da Lagoa Tapada	2017	70.8%	74.3%	66.7%
Brazil	São José da Laje	2000	64.1%	67.2%	60.8%
Brazil	São José da Laje	2017	63.1%	66.1%	59.8%
Brazil	São José da Lapa	2000	81.7%	84.3%	78.7%
Brazil	São José da Lapa	2017	82.2%	84.8%	79.4%

Country	Second Administrative Unit	Year	Mean	Upper	Lower
Brazil	São José da Safira	2000	80.3%	85.1%	75.1%
Brazil	São José da Safira	2017	80.9%	85.6%	75.9%
Brazil	São José da Tapera	2000	63.3%	66.9%	59.9%
Brazil	São José da Tapera	2017	64.2%	67.7%	60.8%
Brazil	São José da Varginha	2000	80.2%	83.9%	75.7%
Brazil	São José da Varginha	2017	80.8%	84.4%	76.4%
Brazil	São José da Vitória	2000	76.9%	81.3%	71.3%
Brazil	São José da Vitória	2017	77.6%	81.9%	72.1%
Brazil	São José das Missões	2000	79.0%	82.7%	75.2%
Brazil	São José das Missões	2017	79.7%	83.3%	75.9%
Brazil	São José das Palmeiras	2000	83.3%	87.0%	79.7%
Brazil	São José das Palmeiras	2017	83.8%	87.4%	80.3%
Brazil	São José de Caiana	2000	69.9%	73.4%	65.8%
Brazil	São José de Caiana	2017	70.8%	74.2%	66.7%
Brazil	São José de Espinharas	2000	71.9%	75.5%	67.7%
Brazil	São José de Espinharas	2017	72.7%	76.2%	68.6%
Brazil	São José de Mipibu	2000	80.2%	82.4%	77.8%
Brazil	São José de Mipibu	2017	80.8%	83.0%	78.4%
Brazil	São José de Piranhas	2000	69.9%	73.6%	66.0%
Brazil	São José de Piranhas	2017	70.7%	74.4%	66.9%
Brazil	São José de Princesa	2000	68.0%	72.2%	63.0%
Brazil	São José de Princesa	2017	68.8%	73.0%	63.9%
Brazil	São José de Ribamar	2000	61.4%	64.9%	57.1%
Brazil	São José de Ribamar	2017	62.3%	65.8%	58.1%
Brazil	São José de Ubá	2000	76.5%	80.3%	72.7%
Brazil	São José de Ubá	2017	77.3%	81.0%	73.5%
Brazil	São José do Alegre	2000	87.6%	90.5%	84.3%
Brazil	São José do Alegre	2017	88.0%	90.8%	84.8%
Brazil	São José do Barreiro	2000	85.2%	87.7%	82.3%
Brazil	São José do Barreiro	2017	85.7%	88.1%	82.9%
Brazil	São José do Belmonte	2000	77.6%	81.3%	74.0%

Country	Second Administrative Unit	Year	Mean	Upper	Lower
Brazil	São José do Belmonte	2017	78.3%	81.9%	74.8%
Brazil	São José do Bonfim	2000	69.8%	73.5%	65.5%
Brazil	São José do Bonfim	2017	70.6%	74.3%	66.3%
Brazil	São José do Calçado	2000	80.2%	83.6%	76.6%
Brazil	São José do Calçado	2017	80.7%	84.1%	77.3%
Brazil	São José do Campestre	2000	80.1%	82.5%	77.5%
Brazil	São José do Campestre	2017	80.8%	83.1%	78.2%
Brazil	São José do Cedro	2000	81.0%	85.1%	76.4%
Brazil	São José do Cedro	2017	81.5%	85.6%	77.1%
Brazil	São José do Cerrito	2000	76.1%	80.4%	71.0%
Brazil	São José do Cerrito	2017	76.8%	81.0%	71.8%
Brazil	São José do Divino	2000	59.4%	63.5%	55.0%
Brazil	São José do Divino	2000	81.4%	86.3%	77.0%
Brazil	São José do Divino	2017	60.4%	64.4%	55.9%
Brazil	São José do Divino	2017	82.0%	86.7%	77.6%
Brazil	São José do Goiabal	2000	79.8%	83.7%	75.8%
Brazil	São José do Goiabal	2017	80.2%	84.0%	76.3%
Brazil	São José do Herval	2000	82.7%	86.2%	78.9%
Brazil	São José do Herval	2017	83.2%	86.6%	79.5%
Brazil	São José do Hortêncio	2000	79.9%	82.5%	77.2%
Brazil	São José do Hortêncio	2017	80.5%	83.0%	77.9%
Brazil	São José do Inhacorá	2000	80.1%	84.7%	75.0%
Brazil	São José do Inhacorá	2017	80.7%	85.1%	75.7%
Brazil	São José do Jacuípe	2000	77.6%	82.0%	72.6%
Brazil	São José do Jacuípe	2017	78.2%	82.6%	73.4%
Brazil	São José do Jacuri	2000	81.6%	86.6%	75.6%
Brazil	São José do Jacuri	2017	82.2%	87.0%	76.3%
Brazil	São José do Mantimento	2000	79.7%	83.1%	75.2%
Brazil	São José do Mantimento	2017	80.4%	83.7%	76.0%
Brazil	São José do Ouro	2000	77.9%	81.6%	73.3%
Brazil	São José do Ouro	2017	78.5%	82.2%	74.0%

Country	Second Administrative Unit	Year	Mean	Upper	Lower
Brazil	São José do Peixe	2000	59.8%	65.2%	52.7%
Brazil	São José do Peixe	2017	60.8%	66.1%	53.7%
Brazil	São José do Piauí	2000	62.0%	67.1%	57.2%
Brazil	São José do Piauí	2017	62.9%	67.9%	58.2%
Brazil	São José do Povo	2000	67.9%	73.4%	63.0%
Brazil	São José do Povo	2017	68.7%	74.1%	63.9%
Brazil	São José do Rio Claro	2000	69.4%	75.0%	62.7%
Brazil	São José do Rio Claro	2017	70.2%	75.7%	63.6%
Brazil	São José do Rio Pardo	2000	90.1%	92.5%	87.3%
Brazil	São José do Rio Pardo	2017	90.4%	92.7%	87.8%
Brazil	São José do Rio Preto	2000	93.0%	94.5%	90.8%
Brazil	São José do Rio Preto	2017	93.1%	94.7%	91.1%
Brazil	São José do Sabugi	2000	76.1%	79.7%	72.2%
Brazil	São José do Sabugi	2017	76.8%	80.3%	73.0%
Brazil	São José do Seridó	2000	77.1%	80.6%	73.3%
Brazil	São José do Seridó	2017	77.8%	81.2%	74.1%
Brazil	São José do Vale do Rio Preto	2000	80.8%	83.7%	78.0%
Brazil	São José do Vale do Rio Preto	2017	81.8%	84.6%	79.2%
Brazil	São José do Xingu	2000	62.6%	71.2%	51.7%
Brazil	São José do Xingu	2017	63.4%	71.9%	52.6%
Brazil	São José dos Ausentes	2000	81.7%	85.8%	77.5%
Brazil	São José dos Ausentes	2017	82.3%	86.3%	78.2%
Brazil	São José dos Basílios	2000	61.1%	66.9%	56.0%
Brazil	São José dos Basílios	2017	62.0%	67.8%	57.0%
Brazil	São José dos Campos	2000	93.4%	94.5%	92.2%
Brazil	São José dos Campos	2017	93.6%	94.7%	92.4%
Brazil	São José dos Cordeiros	2000	68.5%	73.1%	63.9%
Brazil	São José dos Cordeiros	2017	69.4%	73.8%	64.8%
Brazil	São José dos Pinhais	2000	85.9%	87.7%	84.1%
Brazil	São José dos Pinhais	2017	86.4%	88.2%	84.7%

Country	Second Administrative Unit	Year	Mean	Upper	Lower
Brazil	São José dos Quatro Marcos	2000	69.9%	75.4%	62.9%
Brazil	São José dos Quatro Marcos	2017	70.7%	76.1%	63.8%
Brazil	São José dos Ramos	2000	67.4%	70.8%	64.2%
Brazil	São José dos Ramos	2017	68.3%	71.7%	65.1%
Brazil	São Juliao	2000	64.8%	69.4%	60.2%
Brazil	São Juliao	2017	65.7%	70.2%	61.1%
Brazil	São Leopoldo	2000	81.9%	83.8%	79.8%
Brazil	São Leopoldo	2017	82.5%	84.4%	80.4%
Brazil	São Lourenço	2000	85.2%	88.4%	81.6%
Brazil	São Lourenço	2017	85.7%	88.8%	82.2%
Brazil	São Lourenço da Mata	2000	65.2%	67.4%	63.0%
Brazil	São Lourenço da Mata	2017	66.7%	68.8%	64.6%
Brazil	São Lourenço da Serra	2000	93.3%	94.4%	91.8%
Brazil	São Lourenço da Serra	2017	93.5%	94.6%	92.1%
Brazil	São Lourenço do Oeste	2000	80.8%	85.2%	76.6%
Brazil	São Lourenço do Oeste	2017	81.4%	85.7%	77.3%
Brazil	São Lourenço do Piauí	2000	64.8%	71.2%	58.0%
Brazil	São Lourenço do Piauí	2017	65.7%	71.9%	59.0%
Brazil	São Lourenço do Sul	2000	81.1%	84.7%	76.3%
Brazil	São Lourenço do Sul	2017	81.7%	85.2%	77.0%
Brazil	São Ludgero	2000	73.1%	77.2%	68.9%
Brazil	São Ludgero	2017	73.8%	77.9%	69.7%
Brazil	São Luis	2000	61.2%	64.6%	57.2%
Brazil	São Luis	2017	62.1%	65.5%	58.2%
Brazil	São Luís de Montes Belos	2000	76.9%	80.8%	71.9%
Brazil	São Luís de Montes Belos	2017	77.6%	81.4%	72.7%
Brazil	São Luis do Piauí	2000	62.7%	68.1%	57.9%
Brazil	São Luis do Piauí	2017	63.6%	68.9%	58.9%
Brazil	São Luís do Quitunde	2000	61.6%	64.4%	58.2%
Brazil	São Luís do Quitunde	2017	62.5%	65.3%	59.2%
Brazil	São Luis Gonzaga do Maranhao	2000	60.0%	65.1%	54.8%
Brazil	São Luis Gonzaga do Maranhao	2017	60.9%	66.0%	55.8%
Brazil	São Luiz	2000	85.8%	89.0%	81.2%
Brazil	São Luiz	2017	86.3%	89.4%	81.8%
Brazil	São Luiz do Curu	2000	71.2%	74.4%	67.2%

Country	Second Administrative Unit	Year	Mean	Upper	Lower
Brazil	São Luiz do Curu	2017	72.0%	75.1%	68.0%
Brazil	São Luiz do Norte	2000	78.8%	83.7%	73.7%
Brazil	São Luiz do Norte	2017	79.4%	84.3%	74.5%
Brazil	São Luiz do Paraitinga	2000	92.5%	94.2%	90.2%
Brazil	São Luiz do Paraitinga	2017	92.7%	94.4%	90.5%
Brazil	São Luiz Gonzaga	2000	81.7%	86.6%	76.7%
Brazil	São Luiz Gonzaga	2017	82.2%	87.0%	77.2%
Brazil	São Mamede	2000	73.9%	78.1%	69.7%
Brazil	São Mamede	2017	74.6%	78.8%	70.5%
Brazil	São Manoel do Paraná	2000	83.3%	86.4%	79.6%
Brazil	São Manoel do Paraná	2017	83.8%	86.8%	80.2%
Brazil	São Manuel	2000	94.8%	96.2%	93.2%
Brazil	São Manuel	2017	95.0%	96.4%	93.4%
Brazil	São Marcos	2000	82.1%	85.0%	78.2%
Brazil	São Marcos	2017	82.7%	85.5%	78.8%
Brazil	São Martinho	2000	81.3%	85.2%	76.3%
Brazil	São Martinho	2000	73.0%	77.5%	67.4%
Brazil	São Martinho	2017	73.8%	78.2%	68.4%
Brazil	São Martinho	2017	81.9%	85.7%	77.0%
Brazil	São Martinho da Serra	2000	83.2%	86.9%	78.9%
Brazil	São Martinho da Serra	2017	83.9%	87.5%	79.7%
Brazil	São Mateus	2000	76.3%	80.6%	71.3%
Brazil	São Mateus	2017	77.0%	81.3%	72.1%
Brazil	São Mateus do Maranhão	2000	60.7%	65.5%	55.8%
Brazil	São Mateus do Maranhão	2017	61.6%	66.4%	56.8%
Brazil	São Mateus do Sul	2000	79.2%	82.8%	75.1%
Brazil	São Mateus do Sul	2017	79.9%	83.4%	75.8%
Brazil	São Miguel	2000	74.8%	78.9%	70.5%
Brazil	São Miguel	2017	75.5%	79.5%	71.3%
Brazil	São Miguel Arcanjo	2000	93.7%	95.4%	91.8%
Brazil	São Miguel Arcanjo	2017	93.9%	95.5%	92.1%
Brazil	São Miguel da Baixa Grande	2000	58.9%	63.9%	53.7%
Brazil	São Miguel da Baixa Grande	2017	59.8%	64.9%	54.7%
Brazil	São Miguel da Boa Vista	2000	78.1%	82.1%	73.7%
Brazil	São Miguel da Boa Vista	2017	78.7%	82.7%	74.5%
Brazil	São Miguel das Matas	2000	78.5%	82.2%	73.9%
Brazil	São Miguel das Matas	2017	79.1%	82.7%	74.7%
Brazil	São Miguel das Misses	2000	82.2%	86.3%	77.4%

Country	Second Administrative Unit	Year	Mean	Upper	Lower
Brazil	São Miguel das Misses	2017	82.7%	86.8%	78.1%
Brazil	São Miguel de Touros	2000	85.2%	88.2%	81.1%
Brazil	São Miguel de Touros	2017	85.6%	88.6%	81.7%
Brazil	São Miguel do Aleixo	2000	75.5%	77.9%	72.7%
Brazil	São Miguel do Aleixo	2017	76.2%	78.5%	73.4%
Brazil	São Miguel do Anta	2000	82.2%	85.4%	78.4%
Brazil	São Miguel do Anta	2017	82.8%	85.9%	79.0%
Brazil	São Miguel do Araguaia	2000	74.4%	81.0%	67.7%
Brazil	São Miguel do Araguaia	2017	75.1%	81.5%	68.6%
Brazil	São Miguel do Fidalgo	2000	60.2%	65.9%	54.1%
Brazil	São Miguel do Fidalgo	2017	61.2%	66.8%	55.1%
Brazil	São Miguel do Guamá	2000	37.4%	41.8%	33.9%
Brazil	São Miguel do Guamá	2017	38.4%	42.8%	34.8%
Brazil	São Miguel do Guaporé	2000	29.1%	33.1%	25.8%
Brazil	São Miguel do Guaporé	2017	29.9%	34.0%	26.4%
Brazil	São Miguel do Iguaçu	2000	83.1%	86.8%	79.1%
Brazil	São Miguel do Iguaçu	2017	83.6%	87.2%	79.8%
Brazil	São Miguel do Oeste	2000	81.9%	85.8%	78.0%
Brazil	São Miguel do Oeste	2017	82.5%	86.3%	78.7%
Brazil	São Miguel do Passa Quatro	2000	78.3%	81.7%	74.9%
Brazil	São Miguel do Passa Quatro	2017	79.0%	82.2%	75.6%
Brazil	São Miguel do Tocantins	2000	66.5%	70.3%	62.1%
Brazil	São Miguel do Tocantins	2017	67.2%	71.1%	62.9%
Brazil	São Miguel dos Campos	2000	65.6%	68.7%	62.6%
Brazil	São Miguel dos Campos	2017	66.5%	69.5%	63.4%
Brazil	São Miguel dos Milagres	2000	62.0%	65.9%	57.3%
Brazil	São Miguel dos Milagres	2017	62.9%	66.8%	58.2%
Brazil	São Miguel Taipu	2000	67.0%	70.6%	63.9%
Brazil	São Miguel Taipu	2017	67.9%	71.4%	64.7%
Brazil	São Miguel Tapuio	2000	65.1%	70.2%	60.0%
Brazil	São Miguel Tapuio	2017	66.0%	70.9%	60.9%

Country	Second Administrative Unit	Year	Mean	Upper	Lower
Brazil	São Nicolau	2000	80.7%	86.3%	74.3%
Brazil	São Nicolau	2017	81.3%	86.7%	75.0%
Brazil	São Patrício	2000	76.7%	81.3%	72.4%
Brazil	São Patrício	2017	77.4%	81.9%	73.2%
Brazil	São Paulo	2000	94.5%	95.3%	93.9%
Brazil	São Paulo	2017	94.7%	95.4%	94.1%
Brazil	São Paulo das Missões	2000	81.1%	86.3%	75.1%
Brazil	São Paulo das Missões	2017	81.7%	86.8%	75.8%
Brazil	São Paulo de Olivença	2000	61.3%	67.7%	53.7%
Brazil	São Paulo de Olivença	2017	62.2%	68.5%	54.7%
Brazil	São Paulo do Potengi	2000	84.3%	86.4%	81.9%
Brazil	São Paulo do Potengi	2017	84.8%	86.8%	82.5%
Brazil	São Pedro	2000	83.3%	85.3%	81.1%
Brazil	São Pedro	2000	93.5%	94.9%	91.7%
Brazil	São Pedro	2017	83.8%	85.8%	81.7%
Brazil	São Pedro	2017	93.7%	95.0%	92.0%
Brazil	São Pedro da Água Branca	2000	51.3%	57.1%	45.1%
Brazil	São Pedro da Água Branca	2017	52.3%	58.1%	46.0%
Brazil	São Pedro da Aldeia	2000	77.8%	81.2%	73.7%
Brazil	São Pedro da Aldeia	2017	78.6%	81.9%	74.6%
Brazil	São Pedro da Cipa	2000	67.5%	72.7%	62.0%
Brazil	São Pedro da Cipa	2017	68.2%	73.4%	62.8%
Brazil	São Pedro da Serra	2000	84.5%	87.0%	81.8%
Brazil	São Pedro da Serra	2017	85.0%	87.4%	82.4%
Brazil	São Pedro da União	2000	86.4%	89.8%	82.6%
Brazil	São Pedro da União	2017	86.9%	90.1%	83.1%
Brazil	São Pedro de Alcântara	2000	73.8%	78.5%	68.4%
Brazil	São Pedro de Alcântara	2017	74.6%	79.2%	69.3%
Brazil	São Pedro do Butiá	2000	81.1%	86.1%	75.4%
Brazil	São Pedro do Butiá	2017	81.7%	86.5%	76.1%
Brazil	São Pedro do Iguaçu	2000	84.2%	87.6%	80.9%
Brazil	São Pedro do Iguaçu	2017	84.7%	88.0%	81.5%
Brazil	São Pedro do Ivaí	2000	83.0%	86.0%	79.4%
Brazil	São Pedro do Ivaí	2017	83.5%	86.5%	80.0%
Brazil	São Pedro do Paraná	2000	82.7%	86.4%	78.9%
Brazil	São Pedro do Paraná	2017	83.2%	86.8%	79.6%

Country	Second Administrative Unit	Year	Mean	Upper	Lower
Brazil	São Pedro do Piauí	2000	63.2%	67.4%	57.8%
Brazil	São Pedro do Piauí	2017	64.2%	68.3%	58.8%
Brazil	São Pedro do Suaçuí	2000	81.1%	86.0%	75.6%
Brazil	São Pedro do Suaçuí	2017	81.7%	86.4%	76.3%
Brazil	São Pedro do Sul	2000	81.7%	85.6%	76.8%
Brazil	São Pedro do Sul	2017	82.3%	86.1%	77.5%
Brazil	São Pedro do Turvo	2000	91.6%	93.9%	88.9%
Brazil	São Pedro do Turvo	2017	91.9%	94.1%	89.3%
Brazil	São Pedro dos Crentes	2000	70.0%	76.4%	62.3%
Brazil	São Pedro dos Crentes	2017	70.8%	77.1%	63.3%
Brazil	São Pedro dos Ferros	2000	81.4%	84.9%	77.3%
Brazil	São Pedro dos Ferros	2017	81.9%	85.4%	78.0%
Brazil	São Rafael	2000	80.6%	83.5%	77.1%
Brazil	São Rafael	2017	81.2%	84.0%	77.8%
Brazil	São Raimundo das Mangabeiras	2000	63.0%	71.1%	54.5%
Brazil	São Raimundo das Mangabeiras	2017	63.9%	71.9%	55.4%
Brazil	São Raimundo do Doca Bezerra	2000	59.4%	65.6%	54.1%
Brazil	São Raimundo do Doca Bezerra	2017	60.3%	66.5%	55.1%
Brazil	São Raimundo Nonato	2000	65.6%	71.8%	59.5%
Brazil	São Raimundo Nonato	2017	66.5%	72.6%	60.5%
Brazil	São Roberto	2000	58.9%	64.4%	53.8%
Brazil	São Roberto	2017	59.9%	65.3%	54.8%
Brazil	São Romão	2000	82.4%	87.9%	76.4%
Brazil	São Romão	2017	83.0%	88.3%	77.1%
Brazil	São Roque	2000	94.3%	95.3%	93.2%
Brazil	São Roque	2017	94.5%	95.5%	93.4%
Brazil	São Roque de Minas	2000	83.9%	88.4%	79.3%
Brazil	São Roque de Minas	2017	84.5%	88.8%	79.9%
Brazil	São Roque do Canaã	2000	79.3%	82.2%	76.1%
Brazil	São Roque do Canaã	2017	79.9%	82.7%	76.7%
Brazil	São Salvador do Tocantins	2000	76.0%	81.3%	70.2%
Brazil	São Salvador do Tocantins	2017	76.7%	81.9%	71.0%
Brazil	São Sebastião	2000	61.8%	65.1%	59.1%
Brazil	São Sebastião	2000	90.7%	93.1%	88.0%

Country	Second Administrative Unit	Year	Mean	Upper	Lower
Brazil	São Sebastião	2017	62.7%	66.0%	60.0%
Brazil	São Sebastião	2017	91.0%	93.4%	88.4%
Brazil	São Sebastião da Amoreira	2000	87.0%	89.3%	84.5%
Brazil	São Sebastião da Amoreira	2017	87.5%	89.7%	85.0%
Brazil	São Sebastião da Bela Vista	2000	87.4%	90.3%	84.5%
Brazil	São Sebastião da Bela Vista	2017	87.9%	90.6%	85.0%
Brazil	São Sebastião da Boa Vista	2000	38.6%	44.2%	33.5%
Brazil	São Sebastião da Boa Vista	2017	39.5%	45.2%	34.4%
Brazil	São Sebastião da Grama	2000	90.3%	92.7%	87.5%
Brazil	São Sebastião da Grama	2017	90.6%	93.0%	88.0%
Brazil	São Sebastião de Lagoa de Roça	2000	67.8%	70.8%	64.8%
Brazil	São Sebastião de Lagoa de Roça	2017	68.6%	71.6%	65.7%
Brazil	São Sebastião do Alto	2000	77.8%	81.5%	74.0%
Brazil	São Sebastião do Alto	2017	78.4%	82.0%	74.8%
Brazil	São Sebastião do Anta	2000	81.1%	84.8%	76.9%
Brazil	São Sebastião do Anta	2017	82.7%	86.2%	78.8%
Brazil	São Sebastião do Caí	2000	79.9%	82.4%	77.1%
Brazil	São Sebastião do Caí	2017	81.1%	83.5%	78.5%
Brazil	São Sebastião do Maranhão	2000	81.9%	86.6%	76.1%
Brazil	São Sebastião do Maranhão	2017	82.5%	87.0%	76.8%
Brazil	São Sebastião do Oeste	2000	80.9%	85.4%	76.4%
Brazil	São Sebastião do Oeste	2017	81.5%	85.9%	77.1%
Brazil	São Sebastião do Paraíso	2000	88.6%	91.4%	84.8%
Brazil	São Sebastião do Paraíso	2017	88.9%	91.7%	85.3%
Brazil	São Sebastião do Passé	2000	76.4%	79.2%	73.1%
Brazil	São Sebastião do Passé	2017	78.4%	81.0%	75.3%
Brazil	São Sebastião do Rio Preto	2000	80.3%	84.1%	75.8%
Brazil	São Sebastião do Rio Preto	2017	80.9%	84.6%	76.6%
Brazil	São Sebastião do Rio Verde	2000	85.8%	89.0%	82.7%
Brazil	São Sebastião do Rio Verde	2017	86.3%	89.4%	83.3%
Brazil	São Sebastião do Tocantins	2000	58.5%	63.6%	53.0%

Country	Second Administrative Unit	Year	Mean	Upper	Lower
Brazil	São Sebastião do Tocantins	2017	59.5%	64.5%	54.1%
Brazil	São Sebastião do Uatumã	2000	62.1%	68.2%	56.1%
Brazil	São Sebastião do Uatumã	2017	63.0%	69.0%	57.1%
Brazil	São Sebastião do Umbuzeiro	2000	65.0%	69.0%	60.4%
Brazil	São Sebastião do Umbuzeiro	2017	65.9%	69.8%	61.3%
Brazil	São Sebastio da Vargem Alegre	2000	81.7%	85.1%	78.0%
Brazil	São Sebastio da Vargem Alegre	2017	82.3%	85.5%	78.6%
Brazil	São Sepé	2000	81.2%	85.4%	76.4%
Brazil	São Sepé	2017	81.8%	85.9%	77.1%
Brazil	São Simão	2000	80.8%	84.8%	75.8%
Brazil	São Simão	2000	92.7%	94.2%	90.6%
Brazil	São Simão	2017	81.4%	85.3%	76.5%
Brazil	São Simão	2017	93.0%	94.4%	90.9%
Brazil	São Thomé das Letras	2000	85.0%	88.1%	80.9%
Brazil	São Thomé das Letras	2017	85.5%	88.5%	81.5%
Brazil	São Tiago	2000	81.6%	85.6%	77.6%
Brazil	São Tiago	2017	82.2%	86.1%	78.3%
Brazil	São Tomás de Aquino	2000	87.9%	91.1%	84.1%
Brazil	São Tomás de Aquino	2017	88.3%	91.4%	84.6%
Brazil	São Tomé	2000	83.6%	86.5%	80.1%
Brazil	São Tomé	2000	82.4%	85.1%	79.3%
Brazil	São Tomé	2017	83.0%	85.7%	80.0%
Brazil	São Tomé	2017	84.1%	86.9%	80.8%
Brazil	São Valentim	2000	79.4%	82.5%	76.0%
Brazil	São Valentim	2017	80.0%	83.0%	76.7%
Brazil	São Valentim do Sul	2000	81.9%	85.4%	77.2%
Brazil	São Valentim do Sul	2017	82.5%	85.9%	77.9%
Brazil	São Valério da Natividade	2000	76.0%	80.8%	70.4%
Brazil	São Valério da Natividade	2017	76.7%	81.4%	71.2%
Brazil	São Valério do Sul	2000	81.5%	85.7%	76.5%
Brazil	São Valério do Sul	2017	82.1%	86.1%	77.2%
Brazil	São Vendelino	2000	79.7%	82.6%	76.9%
Brazil	São Vendelino	2017	80.4%	83.3%	77.6%
Brazil	São Vicente	2000	82.8%	85.4%	79.9%
Brazil	São Vicente	2000	87.7%	89.8%	85.4%
Brazil	São Vicente	2017	83.4%	85.9%	80.5%
Brazil	São Vicente	2017	88.2%	90.3%	86.1%
Brazil	São Vicente de Minas	2000	82.7%	86.1%	79.0%
Brazil	São Vicente de Minas	2017	83.3%	86.5%	79.7%
Brazil	São Vicente Ferrer	2000	69.6%	72.6%	66.8%

Country	Second Administrative Unit	Year	Mean	Upper	Lower
Brazil	São Vicente Ferrer	2000	60.3%	65.5%	55.1%
Brazil	São Vicente Ferrer	2017	70.4%	73.3%	67.6%
Brazil	São Vicente Ferrer	2017	61.2%	66.4%	56.1%
Brazil	Sapé	2000	69.4%	72.7%	66.3%
Brazil	Sapé	2017	70.2%	73.4%	67.1%
Brazil	Sapeaçu	2000	76.6%	80.4%	71.9%
Brazil	Sapeaçu	2017	77.3%	81.0%	72.7%
Brazil	Sapezal	2000	80.4%	85.7%	74.4%
Brazil	Sapezal	2000	63.1%	70.6%	55.3%
Brazil	Sapezal	2017	64.0%	71.5%	56.2%
Brazil	Sapezal	2017	81.0%	86.2%	75.2%
Brazil	Sapiranga	2000	81.6%	84.1%	79.2%
Brazil	Sapiranga	2017	82.1%	84.6%	79.8%
Brazil	Sapopema	2000	84.8%	88.1%	81.4%
Brazil	Sapopema	2017	85.3%	88.5%	82.0%
Brazil	Sapucaí-Mirim	2000	91.0%	92.7%	89.0%
Brazil	Sapucaí-Mirim	2017	91.2%	92.9%	89.2%
Brazil	Sapucaia	2000	48.7%	55.2%	41.4%
Brazil	Sapucaia	2000	77.8%	81.3%	74.4%
Brazil	Sapucaia	2017	78.5%	81.9%	75.1%
Brazil	Sapucaia	2017	49.7%	56.1%	42.3%
Brazil	Sapucaia do Sul	2000	82.5%	84.2%	80.6%
Brazil	Sapucaia do Sul	2017	83.0%	84.7%	81.2%
Brazil	Saquarema	2000	78.1%	81.0%	74.8%
Brazil	Saquarema	2017	79.0%	81.8%	75.9%
Brazil	Sarandi	2000	85.8%	88.1%	83.0%
Brazil	Sarandi	2000	80.4%	83.9%	76.8%
Brazil	Sarandi	2017	81.1%	84.5%	77.5%
Brazil	Sarandi	2017	86.3%	88.5%	83.6%
Brazil	Sarapuí	2000	93.6%	95.3%	91.6%
Brazil	Sarapuí	2017	93.8%	95.4%	91.9%
Brazil	Sardoá	2000	82.7%	86.5%	78.4%
Brazil	Sardoá	2017	83.2%	86.9%	79.0%
Brazil	Sarutaiá	2000	91.2%	93.3%	88.1%
Brazil	Sarutaiá	2017	91.5%	93.5%	88.5%
Brazil	Sarzedo	2000	81.9%	84.5%	78.9%
Brazil	Sarzedo	2017	82.4%	85.0%	79.5%
Brazil	Sátiro Dias	2000	79.8%	83.2%	75.7%
Brazil	Sátiro Dias	2017	80.4%	83.7%	76.5%
Brazil	Satuba	2000	59.2%	61.5%	57.0%
Brazil	Satuba	2017	60.1%	62.4%	57.9%
Brazil	Satubinha	2000	60.7%	65.9%	54.8%
Brazil	Satubinha	2017	61.7%	66.8%	55.8%
Brazil	Saubara	2000	73.6%	77.1%	70.4%
Brazil	Saubara	2017	74.3%	77.7%	71.2%
Brazil	Saudade do Iguaçu	2000	82.1%	85.6%	77.3%
Brazil	Saudade do Iguaçu	2017	82.7%	86.0%	78.0%
Brazil	Saudades	2000	76.6%	80.5%	71.9%
Brazil	Saudades	2017	77.3%	81.1%	72.7%
Brazil	Saúde	2000	76.3%	81.1%	70.4%
Brazil	Saúde	2017	77.0%	81.8%	71.2%
Brazil	Schroeder	2000	72.7%	76.7%	69.0%
Brazil	Schroeder	2017	73.4%	77.4%	69.7%

Country	Second Administrative Unit	Year	Mean	Upper	Lower
Brazil	Seabra	2000	79.8%	84.8%	74.8%
Brazil	Seabra	2017	80.4%	85.3%	75.5%
Brazil	Seara	2000	77.1%	80.7%	72.7%
Brazil	Seara	2017	77.7%	81.2%	73.4%
Brazil	Sebastianópolis do Sul	2000	91.5%	94.0%	88.8%
Brazil	Sebastianópolis do Sul	2017	91.8%	94.2%	89.2%
Brazil	Sebastião Barros	2000	68.6%	75.1%	60.9%
Brazil	Sebastião Barros	2017	69.4%	75.7%	61.9%
Brazil	Sebastião Laranjeiras	2000	78.2%	83.8%	71.9%
Brazil	Sebastião Laranjeiras	2017	78.9%	84.4%	72.7%
Brazil	Sebastião Leal	2000	62.4%	68.5%	55.0%
Brazil	Sebastião Leal	2017	63.3%	69.3%	55.9%
Brazil	Seberi	2000	79.7%	83.6%	75.5%
Brazil	Seberi	2017	80.2%	84.1%	76.2%
Brazil	Sede Nova	2000	81.4%	85.4%	76.1%
Brazil	Sede Nova	2017	81.9%	85.9%	76.8%
Brazil	Segredo	2000	83.2%	86.3%	79.3%
Brazil	Segredo	2017	83.7%	86.8%	80.0%
Brazil	Selbach	2000	81.8%	85.7%	77.1%
Brazil	Selbach	2017	82.4%	86.2%	77.7%
Brazil	Selvíria	2000	84.4%	88.0%	80.1%
Brazil	Selvíria	2017	84.9%	88.4%	80.7%
Brazil	Sem-Peixe	2000	80.3%	84.1%	76.4%
Brazil	Sem-Peixe	2017	80.9%	84.7%	77.1%
Brazil	Sena Madureira	2000	44.5%	49.2%	39.9%
Brazil	Sena Madureira	2017	45.4%	50.1%	40.9%
Brazil	Senador Alexandre Costa	2000	63.3%	68.8%	58.7%
Brazil	Senador Alexandre Costa	2017	64.2%	69.7%	59.7%
Brazil	Senador Amaral	2000	93.2%	94.5%	91.7%
Brazil	Senador Amaral	2017	93.5%	94.7%	92.0%
Brazil	Senador Canedo	2000	77.5%	79.3%	75.0%
Brazil	Senador Canedo	2017	78.4%	80.2%	76.0%
Brazil	Senador Cortes	2000	80.6%	84.0%	77.1%
Brazil	Senador Cortes	2017	81.2%	84.5%	77.8%
Brazil	Senador Elói de Souza	2000	82.6%	85.0%	80.4%
Brazil	Senador Elói de Souza	2017	83.2%	85.5%	81.1%
Brazil	Senador Firmino	2000	80.5%	83.8%	76.9%
Brazil	Senador Firmino	2017	81.9%	84.9%	78.5%
Brazil	Senador Georgino Avelino	2000	79.1%	81.5%	76.3%

Piped Water Proportion by Second Administrative Unit *(continued)*

Country	Second Administrative Unit	Year	Mean	Upper	Lower
Brazil	Senador Georgino Avelino	2017	79.7%	82.0%	76.9%
Brazil	Senador Guiomard	2000	39.6%	41.8%	37.4%
Brazil	Senador Guiomard	2017	40.6%	42.8%	38.5%
Brazil	Senador José Bento	2000	88.7%	90.9%	85.5%
Brazil	Senador José Bento	2017	89.1%	91.3%	86.1%
Brazil	Senador José Porfírio	2000	38.4%	46.1%	30.1%
Brazil	Senador José Porfírio	2017	39.2%	46.8%	30.9%
Brazil	Senador La Rocque	2000	66.1%	70.8%	61.3%
Brazil	Senador La Rocque	2017	66.9%	71.6%	62.2%
Brazil	Senador Modestino Gonçalves	2000	82.6%	86.9%	77.0%
Brazil	Senador Modestino Gonçalves	2017	83.1%	87.3%	77.6%
Brazil	Senador Pompeu	2000	68.9%	73.6%	64.3%
Brazil	Senador Pompeu	2017	69.8%	74.4%	65.2%
Brazil	Senador Rui Palmeira	2000	63.5%	66.8%	59.9%
Brazil	Senador Rui Palmeira	2017	64.3%	67.6%	60.8%
Brazil	Senador Sá	2000	69.3%	73.4%	64.7%
Brazil	Senador Sá	2017	70.1%	74.2%	65.6%
Brazil	Senador Salgado Filho	2000	82.2%	86.5%	77.8%
Brazil	Senador Salgado Filho	2017	82.8%	86.9%	78.5%
Brazil	Sengés	2000	88.6%	91.6%	85.4%
Brazil	Sengés	2017	89.0%	91.9%	85.9%
Brazil	Senhor do Bonfim	2000	75.6%	81.4%	69.4%
Brazil	Senhor do Bonfim	2017	76.3%	82.0%	70.2%
Brazil	Senhora de Oliveira	2000	81.0%	84.2%	77.0%
Brazil	Senhora de Oliveira	2017	81.5%	84.8%	77.7%
Brazil	Senhora do Porto	2000	81.4%	85.6%	75.7%
Brazil	Senhora do Porto	2017	82.0%	86.1%	76.4%
Brazil	Senhora dos Remédios	2000	81.1%	84.6%	76.8%
Brazil	Senhora dos Remédios	2017	81.7%	85.1%	77.5%
Brazil	Sentinela do Sul	2000	82.7%	86.4%	79.0%
Brazil	Sentinela do Sul	2017	83.2%	86.8%	79.6%
Brazil	Sento Sé	2000	69.3%	75.4%	62.5%

Country	Second Administrative Unit	Year	Mean	Upper	Lower
Brazil	Sento Sé	2017	70.1%	76.1%	63.4%
Brazil	Serafina Corrêa	2000	82.6%	85.7%	78.9%
Brazil	Serafina Corrêa	2017	83.2%	86.2%	79.6%
Brazil	Sericita	2000	82.5%	86.2%	78.5%
Brazil	Sericita	2017	83.0%	86.7%	79.1%
Brazil	Seridó	2000	73.2%	76.9%	69.1%
Brazil	Seridó	2017	73.9%	77.6%	69.9%
Brazil	Seringueiras	2000	30.5%	36.6%	24.9%
Brazil	Seringueiras	2017	31.4%	37.5%	25.6%
Brazil	Sério	2000	84.1%	87.5%	80.4%
Brazil	Sério	2017	84.6%	87.9%	80.9%
Brazil	Seritinga	2000	84.3%	87.6%	80.6%
Brazil	Seritinga	2017	84.8%	88.0%	81.2%
Brazil	Seropédica	2000	76.7%	78.7%	74.6%
Brazil	Seropédica	2017	77.5%	79.5%	75.4%
Brazil	Serra	2000	76.7%	79.0%	73.7%
Brazil	Serra	2017	77.3%	79.6%	74.5%
Brazil	Serra Alta	2000	78.4%	82.5%	74.0%
Brazil	Serra Alta	2017	79.1%	83.0%	74.7%
Brazil	Serra Azul	2000	91.0%	93.2%	88.7%
Brazil	Serra Azul	2017	92.0%	93.9%	89.9%
Brazil	Serra Azul de Minas	2000	82.1%	86.6%	76.1%
Brazil	Serra Azul de Minas	2017	82.7%	87.0%	76.8%
Brazil	Serra Branca	2000	67.5%	72.3%	62.8%
Brazil	Serra Branca	2017	68.4%	73.0%	63.7%
Brazil	Serra da Raiz	2000	72.6%	75.2%	69.9%
Brazil	Serra da Raiz	2017	73.3%	76.0%	70.7%
Brazil	Serra da Saudad	2000	80.9%	86.1%	74.1%
Brazil	Serra da Saudad	2017	81.5%	86.6%	74.8%
Brazil	Serra de São Bento	2000	80.5%	83.0%	77.8%
Brazil	Serra de São Bento	2017	81.1%	83.5%	78.5%
Brazil	Serra do Mel	2000	83.1%	85.7%	80.0%
Brazil	Serra do Mel	2017	83.6%	86.2%	80.6%
Brazil	Serra do Navio	2000	47.7%	53.4%	41.7%
Brazil	Serra do Navio	2017	48.7%	54.4%	42.7%
Brazil	Serra do Ramalho	2000	75.9%	81.7%	68.2%
Brazil	Serra do Ramalho	2017	76.6%	82.3%	69.0%
Brazil	Serra do Salitre	2000	83.2%	87.8%	78.0%
Brazil	Serra do Salitre	2017	83.8%	88.2%	78.7%
Brazil	Serra dos Aimorés	2000	79.7%	84.1%	74.9%
Brazil	Serra dos Aimorés	2017	80.3%	84.6%	75.6%
Brazil	Serra dourada	2000	76.4%	83.4%	67.4%
Brazil	Serra dourada	2017	77.1%	83.9%	68.2%
Brazil	Serra Grande	2000	69.6%	73.0%	65.8%
Brazil	Serra Grande	2017	70.4%	73.7%	66.7%
Brazil	Serra Negra	2000	92.3%	93.8%	90.6%

Country	Second Administrative Unit	Year	Mean	Upper	Lower
Brazil	Serra Negra	2017	92.6%	94.0%	91.0%
Brazil	Serra Negra do Norte	2000	74.2%	77.8%	70.5%
Brazil	Serra Negra do Norte	2017	74.9%	78.5%	71.3%
Brazil	Serra Preta	2000	78.3%	82.2%	74.4%
Brazil	Serra Preta	2017	79.0%	82.8%	75.1%
Brazil	Serra Redonda	2000	66.0%	69.0%	62.7%
Brazil	Serra Redonda	2017	66.9%	69.8%	63.6%
Brazil	Serra Talhada	2000	66.6%	71.0%	61.9%
Brazil	Serra Talhada	2017	67.4%	71.8%	62.8%
Brazil	Serrana	2000	92.1%	94.0%	89.8%
Brazil	Serrana	2017	92.4%	94.2%	90.1%
Brazil	Serrania	2000	81.4%	86.4%	76.1%
Brazil	Serrania	2017	82.0%	86.8%	76.9%
Brazil	Serrano do Maranhão	2000	59.7%	66.2%	51.8%
Brazil	Serrano do Maranhão	2017	60.6%	67.1%	52.8%
Brazil	Serranópolis	2000	78.4%	83.8%	72.1%
Brazil	Serranópolis	2017	79.0%	84.3%	72.9%
Brazil	Serranópolis de Minas	2000	85.1%	88.3%	80.6%
Brazil	Serranópolis de Minas	2017	86.1%	89.1%	81.8%
Brazil	Serranópolis do Iguaçu	2000	82.5%	86.1%	78.6%
Brazil	Serranópolis do Iguaçu	2017	82.9%	86.5%	79.1%
Brazil	Serranos	2000	84.2%	87.5%	80.5%
Brazil	Serranos	2017	84.7%	87.9%	81.1%
Brazil	Serraria	2000	67.7%	70.8%	64.4%
Brazil	Serraria	2017	68.6%	71.6%	65.3%
Brazil	Serrinha	2000	80.3%	82.5%	77.8%
Brazil	Serrinha	2000	78.9%	82.4%	74.7%
Brazil	Serrinha	2017	80.9%	83.1%	78.4%
Brazil	Serrinha	2017	79.5%	82.9%	75.4%
Brazil	Serrinha dos Pintos	2000	79.0%	82.0%	76.0%
Brazil	Serrinha dos Pintos	2017	79.7%	82.5%	76.7%
Brazil	Serrita	2000	68.0%	72.0%	63.3%
Brazil	Serrita	2017	68.8%	72.8%	64.3%
Brazil	Serro	2000	82.5%	86.9%	76.9%
Brazil	Serro	2017	83.1%	87.4%	77.6%
Brazil	Serrolândia	2000	79.0%	83.2%	74.6%
Brazil	Serrolândia	2017	79.6%	83.7%	75.3%
Brazil	Sertaneja	2000	87.1%	89.5%	84.5%
Brazil	Sertaneja	2017	87.6%	89.8%	85.0%
Brazil	Sertânia	2000	65.4%	69.2%	61.3%
Brazil	Sertânia	2017	66.3%	70.0%	62.3%
Brazil	Sertanópolis	2000	86.3%	88.7%	83.6%
Brazil	Sertanópolis	2017	86.7%	89.1%	84.2%
Brazil	Sertão	2000	80.1%	83.7%	76.2%
Brazil	Sertão	2017	80.8%	84.3%	76.9%
Brazil	Sertão Santana	2000	82.3%	85.8%	78.7%
Brazil	Sertão Santana	2017	82.9%	86.2%	79.4%
Brazil	Sertaozinho	2000	93.7%	95.2%	91.7%

Country	Second Administrative Unit	Year	Mean	Upper	Lower
Brazil	Sertaozinho	2017	93.9%	95.4%	92.0%
Brazil	Sertãozinho	2000	71.7%	74.2%	68.7%
Brazil	Sertãozinho	2017	72.5%	75.0%	69.5%
Brazil	Sete Barras	2000	88.6%	91.8%	85.0%
Brazil	Sete Barras	2017	89.0%	92.1%	85.5%
Brazil	Sete de Setembro	2000	82.1%	86.4%	77.3%
Brazil	Sete de Setembro	2017	82.7%	86.8%	77.9%
Brazil	Sete Lagoas	2000	82.4%	85.7%	78.8%
Brazil	Sete Lagoas	2017	83.0%	86.1%	79.5%
Brazil	Sete Quedas	2000	81.8%	86.4%	76.0%
Brazil	Sete Quedas	2017	82.3%	86.8%	76.6%
Brazil	Setubinha	2000	83.5%	87.8%	77.7%
Brazil	Setubinha	2017	84.0%	88.2%	78.4%
Brazil	Severiano de Almeida	2000	77.0%	80.5%	72.6%
Brazil	Severiano de Almeida	2017	77.7%	81.1%	73.4%
Brazil	Severiano Melo	2000	77.7%	81.2%	73.5%
Brazil	Severiano Melo	2017	78.3%	81.8%	74.3%
Brazil	Severínia	2000	92.3%	94.1%	90.0%
Brazil	Severínia	2017	93.3%	94.8%	91.3%
Brazil	Siderópolis	2000	75.0%	79.0%	70.3%
Brazil	Siderópolis	2017	75.6%	79.6%	71.0%
Brazil	Sidrolândia	2000	79.9%	83.1%	76.7%
Brazil	Sidrolândia	2017	80.6%	83.6%	77.4%
Brazil	Sigefredo Pacheco	2000	61.7%	65.9%	56.5%
Brazil	Sigefredo Pacheco	2017	62.6%	66.8%	57.4%
Brazil	Silva Jardim	2000	76.4%	79.0%	73.2%
Brazil	Silva Jardim	2017	77.1%	79.6%	74.0%
Brazil	Silvânia	2000	80.5%	83.0%	77.8%
Brazil	Silvânia	2017	81.1%	83.6%	78.5%
Brazil	Silvanópolis	2000	76.4%	81.4%	71.3%
Brazil	Silvanópolis	2017	77.1%	82.0%	72.1%
Brazil	Silveira Martins	2000	82.8%	86.0%	78.3%
Brazil	Silveira Martins	2017	83.3%	86.5%	79.0%
Brazil	Silveirânia	2000	80.4%	84.0%	76.6%
Brazil	Silveirânia	2017	81.0%	84.5%	77.3%
Brazil	Silveiras	2000	87.7%	90.0%	85.0%
Brazil	Silveiras	2017	88.1%	90.3%	85.5%
Brazil	Silves	2000	58.8%	64.0%	52.9%
Brazil	Silves	2017	59.7%	65.0%	53.8%
Brazil	Silvianópolis	2000	86.8%	89.7%	83.4%
Brazil	Silvianópolis	2017	87.2%	90.1%	83.9%
Brazil	Simão Dias	2000	78.0%	80.6%	75.4%
Brazil	Simão Dias	2017	78.6%	81.2%	76.2%
Brazil	Simão Pereira	2000	79.5%	82.9%	76.2%
Brazil	Simão Pereira	2017	80.1%	83.4%	76.9%
Brazil	Simões	2000	65.8%	70.4%	61.0%
Brazil	Simões	2017	66.6%	71.2%	61.9%
Brazil	Simões Filho	2000	76.1%	79.0%	73.0%
Brazil	Simões Filho	2017	76.4%	79.4%	73.2%
Brazil	Simolândia	2000	83.0%	88.0%	78.0%
Brazil	Simolândia	2017	83.6%	88.4%	78.7%
Brazil	Simonésia	2000	81.5%	84.9%	77.2%

Country	Second Administrative Unit	Year	Mean	Upper	Lower
Brazil	Simonésia	2017	82.1%	85.4%	77.9%
Brazil	Simplício Mendes	2000	61.0%	66.2%	54.9%
Brazil	Simplício Mendes	2017	61.9%	67.1%	55.9%
Brazil	Sinimbu	2000	82.7%	86.2%	78.6%
Brazil	Sinimbu	2017	83.3%	86.7%	79.3%
Brazil	Sinop	2000	71.4%	77.5%	65.1%
Brazil	Sinop	2017	72.3%	78.3%	66.2%
Brazil	Siqueira Campos	2000	88.7%	91.1%	85.3%
Brazil	Siqueira Campos	2017	89.0%	91.4%	85.8%
Brazil	Sirinhaém	2000	60.0%	63.9%	55.8%
Brazil	Sirinhaém	2017	60.9%	64.8%	56.8%
Brazil	Siriri	2000	73.6%	76.0%	70.5%
Brazil	Siriri	2017	74.4%	76.7%	71.3%
Brazil	Sítio d'Abadia	2000	83.4%	87.9%	77.9%
Brazil	Sítio d'Abadia	2017	84.0%	88.3%	78.7%
Brazil	Sítio do Mato	2000	75.4%	82.0%	67.5%
Brazil	Sítio do Mato	2017	76.1%	82.6%	68.4%
Brazil	Sítio do Quinto	2000	74.8%	79.3%	69.9%
Brazil	Sítio do Quinto	2017	75.5%	79.9%	70.6%
Brazil	Sitio dos Moreiras	2000	68.8%	72.5%	63.9%
Brazil	Sitio dos Moreiras	2017	69.6%	73.3%	64.8%
Brazil	Sítio Novo	2000	83.4%	86.0%	80.8%
Brazil	Sítio Novo	2000	67.4%	74.1%	60.7%
Brazil	Sítio Novo	2017	83.9%	86.5%	81.4%
Brazil	Sítio Novo	2017	68.2%	74.8%	61.5%
Brazil	Sítio Novo do Tocantins	2000	68.2%	72.0%	64.3%
Brazil	Sítio Novo do Tocantins	2017	69.1%	72.8%	65.2%
Brazil	Sobradinho	2000	68.4%	73.5%	63.3%
Brazil	Sobradinho	2000	83.8%	86.9%	79.9%
Brazil	Sobradinho	2017	84.3%	87.3%	80.5%
Brazil	Sobradinho	2017	69.3%	74.2%	64.2%
Brazil	Sobrado	2000	67.3%	70.9%	63.9%
Brazil	Sobrado	2017	68.1%	71.7%	64.8%
Brazil	Sobral	2000	70.4%	73.8%	66.8%
Brazil	Sobral	2017	71.2%	74.6%	67.7%
Brazil	Sobrália	2000	79.7%	84.2%	75.2%
Brazil	Sobrália	2017	80.4%	84.7%	75.9%
Brazil	Socorro	2000	91.8%	93.3%	90.0%
Brazil	Socorro	2017	92.1%	93.5%	90.3%
Brazil	Socorro do Piauí	2000	60.9%	66.8%	54.6%
Brazil	Socorro do Piauí	2017	61.9%	67.7%	55.7%
Brazil	Solânea	2000	71.5%	74.5%	68.4%
Brazil	Solânea	2017	72.4%	75.3%	69.3%
Brazil	Soledade	2000	83.2%	86.2%	79.5%
Brazil	Soledade	2000	71.2%	75.1%	67.1%
Brazil	Soledade	2017	72.0%	75.9%	67.9%
Brazil	Soledade	2017	83.7%	86.7%	80.2%
Brazil	Soledade de Minas	2000	84.5%	87.9%	80.7%
Brazil	Soledade de Minas	2017	85.0%	88.3%	81.3%

Country	Second Administrative Unit	Year	Mean	Upper	Lower
Brazil	Solonópole	2000	70.3%	74.8%	65.7%
Brazil	Solonópole	2017	71.1%	75.6%	66.6%
Brazil	Sombrio	2000	74.9%	79.9%	69.9%
Brazil	Sombrio	2017	75.6%	80.6%	70.7%
Brazil	Sonora	2000	76.4%	81.5%	70.2%
Brazil	Sonora	2017	77.1%	82.1%	71.0%
Brazil	Sooretama	2000	76.5%	80.3%	72.1%
Brazil	Sooretama	2017	77.2%	80.9%	72.9%
Brazil	Sorocaba	2000	94.0%	95.2%	92.8%
Brazil	Sorocaba	2017	94.3%	95.4%	93.0%
Brazil	Sossêgo	2000	75.3%	78.6%	71.5%
Brazil	Sossêgo	2017	76.1%	79.3%	72.3%
Brazil	Soure	2000	34.9%	40.1%	30.1%
Brazil	Soure	2017	35.8%	41.0%	30.9%
Brazil	Sousa	2000	72.2%	75.8%	68.8%
Brazil	Sousa	2017	73.0%	76.5%	69.6%
Brazil	Souto Soares	2000	78.7%	83.8%	73.6%
Brazil	Souto Soares	2017	79.3%	84.4%	74.3%
Brazil	Sucupira	2000	77.0%	81.2%	72.3%
Brazil	Sucupira	2017	77.7%	81.8%	73.0%
Brazil	Sucupira do Norte	2000	61.6%	67.7%	55.2%
Brazil	Sucupira do Norte	2017	62.5%	68.5%	56.1%
Brazil	Sucupira do Riachão	2000	62.1%	67.8%	55.8%
Brazil	Sucupira do Riachão	2017	63.0%	68.7%	56.8%
Brazil	Sud Mennucci	2000	88.3%	91.4%	84.7%
Brazil	Sud Mennucci	2017	88.7%	91.7%	85.2%
Brazil	Sul Brazil	2000	77.7%	81.9%	73.6%
Brazil	Sul Brazil	2017	78.3%	82.4%	74.2%
Brazil	Sulina	2000	82.0%	85.7%	77.4%
Brazil	Sulina	2017	82.5%	86.2%	78.1%
Brazil	Sumaré	2000	93.4%	94.6%	92.1%
Brazil	Sumaré	2017	93.7%	94.8%	92.4%
Brazil	Sumé	2000	67.4%	71.9%	62.6%
Brazil	Sumé	2017	68.2%	72.6%	63.5%
Brazil	Sumidouro	2000	82.6%	85.3%	79.8%
Brazil	Sumidouro	2017	83.2%	85.8%	80.5%
Brazil	Surubim	2000	65.5%	69.0%	62.1%
Brazil	Surubim	2017	66.4%	69.9%	63.0%
Brazil	Sussuapara	2000	61.1%	66.0%	56.7%
Brazil	Sussuapara	2017	62.0%	66.9%	57.6%
Brazil	Suzanápolis	2000	94.6%	95.3%	93.6%
Brazil	Suzanápolis	2017	94.8%	95.5%	93.8%
Brazil	Suzano	2000	94.4%	95.2%	93.5%
Brazil	Suzano	2017	94.6%	95.3%	93.8%
Brazil	Tabaí	2000	80.1%	83.2%	76.4%
Brazil	Tabaí	2017	80.7%	83.8%	77.1%
Brazil	Tabaporã	2000	68.4%	75.7%	61.3%
Brazil	Tabaporã	2017	69.3%	76.4%	62.3%
Brazil	Tabapuã	2000	92.7%	94.3%	90.6%
Brazil	Tabapuã	2017	93.0%	94.5%	90.9%
Brazil	Tabatinga	2000	59.0%	64.9%	53.3%
Brazil	Tabatinga	2000	93.2%	94.9%	91.0%
Brazil	Tabatinga	2017	59.9%	65.8%	54.3%
Brazil	Tabatinga	2017	93.5%	95.1%	91.3%
Brazil	Tabira	2000	67.5%	71.6%	63.0%
Brazil	Tabira	2017	68.3%	72.4%	63.9%
Brazil	Taboão da Serra	2000	94.5%	95.3%	93.7%

Country	Second Administrative Unit	Year	Mean	Upper	Lower
Brazil	Taboão da Serra	2017	94.7%	95.5%	94.0%
Brazil	Tabocas do Brejo Velho	2000	76.5%	83.2%	67.5%
Brazil	Tabocas do Brejo Velho	2017	77.2%	83.7%	68.3%
Brazil	Taboleiro Grande	2000	76.9%	80.5%	73.2%
Brazil	Taboleiro Grande	2017	77.6%	81.1%	73.9%
Brazil	Tabuleiro	2000	79.5%	82.9%	75.3%
Brazil	Tabuleiro	2017	80.1%	83.4%	76.1%
Brazil	Tabuleiro do Norte	2000	74.6%	78.4%	70.7%
Brazil	Tabuleiro do Norte	2017	75.4%	79.1%	71.5%
Brazil	Tacaimbó	2000	66.7%	69.9%	63.4%
Brazil	Tacaimbó	2017	67.5%	70.7%	64.3%
Brazil	Tacaratu	2000	64.8%	69.1%	60.5%
Brazil	Tacaratu	2017	65.8%	70.1%	61.6%
Brazil	Taciba	2000	88.7%	91.6%	86.0%
Brazil	Taciba	2017	89.0%	91.9%	86.4%
Brazil	Tacima	2000	76.2%	78.6%	73.8%
Brazil	Tacima	2017	77.0%	79.4%	74.6%
Brazil	Tacuru	2000	80.9%	85.1%	75.5%
Brazil	Tacuru	2017	81.5%	85.6%	76.3%
Brazil	Taguaí	2000	90.8%	93.2%	87.8%
Brazil	Taguaí	2017	91.2%	93.4%	88.2%
Brazil	Taguatinga	2000	76.0%	81.5%	69.5%
Brazil	Taguatinga	2017	76.7%	82.1%	70.4%
Brazil	Taiaçu	2000	93.0%	94.6%	91.0%
Brazil	Taiaçu	2017	93.3%	94.8%	91.3%
Brazil	Tailândia	2000	41.1%	46.4%	35.6%
Brazil	Tailândia	2017	42.1%	47.4%	36.6%
Brazil	Taió	2000	73.1%	77.5%	68.0%
Brazil	Taió	2017	73.9%	78.2%	68.8%
Brazil	Taiobeiras	2000	81.0%	86.0%	75.0%
Brazil	Taiobeiras	2017	81.6%	86.5%	75.7%
Brazil	Taipas do Tocantins	2000	75.6%	81.1%	69.1%
Brazil	Taipas do Tocantins	2017	76.3%	81.7%	69.9%
Brazil	Taipu	2000	83.6%	85.9%	81.1%
Brazil	Taipu	2017	84.1%	86.4%	81.7%
Brazil	Taiúva	2000	92.8%	94.4%	90.7%
Brazil	Taiúva	2017	93.1%	94.6%	91.0%
Brazil	Talismã	2000	76.0%	80.8%	70.2%
Brazil	Talismã	2017	76.7%	81.5%	71.0%
Brazil	Tamandaré	2000	64.3%	68.4%	60.1%
Brazil	Tamandaré	2017	65.2%	69.2%	61.0%
Brazil	Tamarana	2000	84.5%	87.3%	80.9%
Brazil	Tamarana	2017	85.0%	87.7%	81.5%
Brazil	Tambaú	2000	91.5%	93.5%	89.0%
Brazil	Tambaú	2017	91.8%	93.7%	89.3%
Brazil	Tambe	2000	70.0%	72.9%	67.2%
Brazil	Tambe	2017	70.9%	73.7%	68.1%
Brazil	Tamboara	2000	83.9%	86.8%	80.1%
Brazil	Tamboara	2017	84.4%	87.2%	80.8%
Brazil	Tamboril	2000	69.5%	73.6%	65.1%
Brazil	Tamboril	2017	70.3%	74.3%	65.9%
Brazil	Tamboril do Piauí	2000	61.5%	68.0%	54.7%

Country	Second Administrative Unit	Year	Mean	Upper	Lower
Brazil	Tamboril do Piauí	2017	62.4%	68.8%	55.7%
Brazil	Tanabi	2000	91.9%	93.9%	89.3%
Brazil	Tanabi	2017	92.2%	94.2%	89.6%
Brazil	Tangará	2000	75.4%	79.7%	71.1%
Brazil	Tangará	2000	81.4%	84.2%	78.9%
Brazil	Tangará	2017	82.0%	84.7%	79.5%
Brazil	Tangará	2017	76.1%	80.4%	72.0%
Brazil	Tangará da Serra	2000	69.7%	74.9%	64.0%
Brazil	Tangará da Serra	2017	70.5%	75.7%	64.9%
Brazil	Tanguá	2000	77.2%	79.6%	74.1%
Brazil	Tanguá	2017	77.9%	80.2%	74.9%
Brazil	Tanhaçu	2000	76.1%	81.4%	70.5%
Brazil	Tanhaçu	2017	76.8%	82.0%	71.3%
Brazil	Tanque d'Arca	2000	60.0%	62.7%	57.5%
Brazil	Tanque d'Arca	2017	60.9%	63.6%	58.5%
Brazil	Tanque do Piauí	2000	62.9%	67.4%	57.8%
Brazil	Tanque do Piauí	2017	63.8%	68.2%	58.7%
Brazil	Tanque Novo	2000	78.9%	84.0%	73.3%
Brazil	Tanque Novo	2017	79.6%	84.6%	74.1%
Brazil	Tanquinho	2000	77.3%	81.0%	73.3%
Brazil	Tanquinho	2017	78.0%	81.6%	74.1%
Brazil	Taparuba	2000	79.2%	83.2%	74.9%
Brazil	Taparuba	2017	79.9%	83.8%	75.8%
Brazil	Tapauá	2000	58.2%	66.0%	49.9%
Brazil	Tapauá	2017	59.1%	66.8%	50.8%
Brazil	Tapejara	2000	79.6%	83.6%	75.4%
Brazil	Tapejara	2000	84.1%	87.2%	80.6%
Brazil	Tapejara	2017	84.6%	87.7%	81.2%
Brazil	Tapejara	2017	80.2%	84.1%	76.1%
Brazil	Tapera	2000	82.6%	86.1%	77.9%
Brazil	Tapera	2017	83.1%	86.5%	78.5%
Brazil	Taperoá	2000	72.6%	77.3%	67.8%
Brazil	Taperoá	2000	70.4%	74.9%	65.9%
Brazil	Taperoá	2017	71.2%	75.6%	66.8%
Brazil	Taperoá	2017	74.0%	78.5%	69.4%
Brazil	Tapes	2000	82.3%	86.1%	78.3%
Brazil	Tapes	2017	82.9%	86.6%	79.0%
Brazil	Tapira	2000	86.1%	89.7%	81.8%
Brazil	Tapira	2000	83.3%	86.2%	79.9%
Brazil	Tapira	2017	83.9%	86.7%	80.5%
Brazil	Tapira	2017	86.6%	90.1%	82.3%
Brazil	Tapiraí	2000	82.6%	87.5%	77.2%
Brazil	Tapiraí	2000	93.3%	95.0%	91.2%
Brazil	Tapiraí	2017	93.5%	95.2%	91.5%
Brazil	Tapiraí	2017	83.2%	87.9%	77.9%
Brazil	Tapiramutá	2000	78.3%	83.6%	73.3%
Brazil	Tapiramutá	2017	79.0%	84.1%	74.1%
Brazil	Tapiratiba	2000	88.1%	91.0%	84.7%
Brazil	Tapiratiba	2017	88.5%	91.3%	85.2%
Brazil	Tapurah	2000	69.0%	74.8%	64.0%
Brazil	Tapurah	2017	69.8%	75.5%	64.9%
Brazil	Taquara	2000	80.1%	82.7%	77.4%
Brazil	Taquara	2017	80.7%	83.3%	78.2%
Brazil	Taquaraçu de Minas	2000	81.1%	84.1%	77.8%

Country	Second Administrative Unit	Year	Mean	Upper	Lower
Brazil	Taquaraçu de Minas	2017	81.7%	84.7%	78.4%
Brazil	Taquaral	2000	92.8%	94.4%	90.8%
Brazil	Taquaral	2017	93.1%	94.6%	91.1%
Brazil	Taquaral de Goiás	2000	77.5%	81.3%	73.6%
Brazil	Taquaral de Goiás	2017	78.2%	81.8%	74.3%
Brazil	Taquarana	2000	60.6%	63.3%	58.2%
Brazil	Taquarana	2017	61.5%	64.2%	59.1%
Brazil	Taquari	2000	80.9%	84.0%	77.0%
Brazil	Taquari	2017	81.4%	84.5%	77.7%
Brazil	Taquaritinga	2000	93.3%	94.8%	91.5%
Brazil	Taquaritinga	2017	93.6%	95.0%	91.8%
Brazil	Taquaritinga do Norte	2000	67.5%	70.9%	64.1%
Brazil	Taquaritinga do Norte	2017	68.3%	71.7%	64.8%
Brazil	Taquarituba	2000	91.3%	93.6%	88.5%
Brazil	Taquarituba	2017	91.6%	93.8%	88.9%
Brazil	Taquarivaí	2000	92.0%	94.2%	89.2%
Brazil	Taquarivaí	2017	92.3%	94.4%	89.5%
Brazil	Taquaruçu do Sul	2000	79.9%	83.8%	75.1%
Brazil	Taquaruçu do Sul	2017	80.5%	84.3%	75.8%
Brazil	Taquarussu	2000	81.7%	85.4%	77.6%
Brazil	Taquarussu	2017	82.2%	85.8%	78.3%
Brazil	Tarabai	2000	89.1%	91.8%	86.1%
Brazil	Tarabai	2017	89.5%	92.1%	86.5%
Brazil	Tarauacá	2000	46.5%	50.8%	42.1%
Brazil	Tarauacá	2017	47.4%	51.8%	43.0%
Brazil	Tarrafas	2000	69.4%	73.4%	65.2%
Brazil	Tarrafas	2017	70.2%	74.1%	66.1%
Brazil	Tartarugalzinho	2000	48.9%	53.9%	43.6%
Brazil	Tartarugalzinho	2017	49.9%	54.9%	44.5%
Brazil	Tarumã	2000	89.8%	92.2%	87.6%
Brazil	Tarumã	2017	90.2%	92.5%	88.0%
Brazil	Tarumirim	2000	80.6%	84.5%	76.3%
Brazil	Tarumirim	2017	81.2%	85.0%	77.0%
Brazil	Tasso Fragoso	2000	64.9%	72.8%	56.8%
Brazil	Tasso Fragoso	2017	65.9%	73.8%	58.0%
Brazil	Tatuí	2000	94.1%	95.4%	92.4%
Brazil	Tatuí	2017	94.3%	95.5%	92.7%
Brazil	Tauá	2000	70.2%	74.8%	65.6%
Brazil	Tauá	2017	71.0%	75.5%	66.4%
Brazil	Taubaté	2000	92.2%	93.9%	90.2%
Brazil	Taubaté	2017	92.5%	94.1%	90.6%
Brazil	Tavares	2000	67.2%	71.4%	62.6%
Brazil	Tavares	2000	80.9%	86.0%	75.1%
Brazil	Tavares	2017	81.5%	86.5%	75.8%
Brazil	Tavares	2017	68.1%	72.2%	63.5%
Brazil	Tefé	2000	62.7%	68.7%	56.2%
Brazil	Tefé	2017	63.6%	69.5%	57.1%
Brazil	Teixeira	2000	69.9%	74.0%	65.7%
Brazil	Teixeira	2017	70.7%	74.8%	66.6%
Brazil	Teixeira de Freitas	2000	78.5%	83.8%	72.9%
Brazil	Teixeira de Freitas	2017	79.2%	84.3%	73.7%
Brazil	Teixeiras	2000	80.9%	84.3%	76.9%
Brazil	Teixeiras	2017	81.5%	84.8%	77.6%

Country	Second Administrative Unit	Year	Mean	Upper	Lower
Brazil	Teixeirópolis	2000	28.0%	31.2%	24.8%
Brazil	Teixeirópolis	2017	28.8%	32.1%	25.6%
Brazil	Tejuçuoca	2000	70.6%	74.5%	66.6%
Brazil	Tejuçuoca	2017	71.5%	75.2%	67.4%
Brazil	Tejupa	2000	91.6%	93.8%	88.7%
Brazil	Tejupa	2017	91.9%	94.1%	89.1%
Brazil	Telêmaco Borba	2000	85.3%	88.3%	81.7%
Brazil	Telêmaco Borba	2017	85.8%	88.7%	82.3%
Brazil	Telha	2000	68.5%	71.5%	65.6%
Brazil	Telha	2017	69.4%	72.3%	66.5%
Brazil	Tenente Ananias	2000	76.6%	79.9%	73.3%
Brazil	Tenente Ananias	2017	77.4%	80.6%	74.1%
Brazil	Tenente Laurentino Cruz	2000	82.8%	85.4%	79.9%
Brazil	Tenente Laurentino Cruz	2017	83.4%	85.9%	80.5%
Brazil	Tenente Portela	2000	80.2%	84.2%	75.4%
Brazil	Tenente Portela	2017	80.8%	84.8%	76.1%
Brazil	Tenório	2000	73.3%	76.9%	69.0%
Brazil	Tenório	2017	74.1%	77.6%	69.8%
Brazil	Teodoro Sampaio	2000	76.7%	80.0%	72.9%
Brazil	Teodoro Sampaio	2000	85.4%	88.7%	81.7%
Brazil	Teodoro Sampaio	2017	77.4%	80.6%	73.6%
Brazil	Teodoro Sampaio	2017	85.9%	89.1%	82.3%
Brazil	Teofilândia	2000	78.8%	82.4%	74.7%
Brazil	Teofilândia	2017	79.4%	83.0%	75.4%
Brazil	Teófilo Otoni	2000	82.3%	87.0%	76.9%
Brazil	Teófilo Otoni	2017	82.9%	87.5%	77.6%
Brazil	Teolândia	2000	76.3%	80.1%	71.9%
Brazil	Teolândia	2017	77.5%	81.2%	73.2%
Brazil	Teotônio Vilela	2000	60.4%	63.8%	57.3%
Brazil	Teotônio Vilela	2017	61.3%	64.7%	58.2%
Brazil	Terenos	2000	78.5%	81.2%	75.8%
Brazil	Terenos	2017	79.1%	81.8%	76.5%
Brazil	Teresina	2000	60.9%	63.4%	58.5%
Brazil	Teresina	2017	61.8%	64.3%	59.5%
Brazil	Teresina de Goiás	2000	80.5%	85.0%	74.5%
Brazil	Teresina de Goiás	2017	81.0%	85.4%	75.2%
Brazil	Teresópolis	2000	83.8%	85.7%	81.3%
Brazil	Teresópolis	2017	84.3%	86.3%	82.0%
Brazil	Terezinha	2000	62.5%	66.0%	58.8%
Brazil	Terezinha	2017	63.4%	66.9%	59.7%
Brazil	Terezópolis de Goiás	2000	78.6%	80.9%	76.4%
Brazil	Terezópolis de Goiás	2017	79.2%	81.5%	77.0%
Brazil	Terra Alta	2000	36.3%	40.5%	31.8%
Brazil	Terra Alta	2017	37.2%	41.5%	32.6%

Country	Second Administrative Unit	Year	Mean	Upper	Lower
Brazil	Terra Boa	2000	83.6%	86.8%	80.3%
Brazil	Terra Boa	2017	84.1%	87.2%	80.9%
Brazil	Terra de Areia	2000	77.8%	82.3%	72.5%
Brazil	Terra de Areia	2017	78.4%	82.9%	73.3%
Brazil	Terra Nova	2000	65.9%	71.2%	61.0%
Brazil	Terra Nova	2000	76.2%	79.4%	72.4%
Brazil	Terra Nova	2017	66.8%	72.0%	62.0%
Brazil	Terra Nova	2017	76.9%	80.1%	73.2%
Brazil	Terra Nova do Norte	2000	67.6%	73.5%	60.1%
Brazil	Terra Nova do Norte	2017	68.4%	74.3%	61.0%
Brazil	Terra Rica	2000	84.7%	88.4%	80.7%
Brazil	Terra Rica	2017	85.2%	88.9%	81.3%
Brazil	Terra Roxa	2000	83.9%	87.4%	80.2%
Brazil	Terra Roxa	2000	91.7%	93.9%	89.3%
Brazil	Terra Roxa	2017	92.0%	94.1%	89.7%
Brazil	Terra Roxa	2017	84.4%	87.8%	80.8%
Brazil	Terra Santa	2000	48.0%	54.9%	41.4%
Brazil	Terra Santa	2017	48.9%	55.8%	42.3%
Brazil	Tesouro	2000	69.4%	75.7%	62.7%
Brazil	Tesouro	2017	70.2%	76.4%	63.7%
Brazil	Teutônia	2000	81.1%	84.1%	77.9%
Brazil	Teutônia	2017	81.7%	84.7%	78.5%
Brazil	Texeira Soares	2000	82.2%	85.3%	78.4%
Brazil	Texeira Soares	2017	83.0%	86.0%	79.3%
Brazil	Theobroma	2000	29.9%	33.6%	26.3%
Brazil	Theobroma	2017	30.7%	34.5%	27.1%
Brazil	Tianguá	2000	73.4%	77.0%	68.9%
Brazil	Tianguá	2017	74.1%	77.6%	69.7%
Brazil	Tibaji	2000	84.3%	87.6%	80.7%
Brazil	Tibaji	2017	84.8%	88.0%	81.3%
Brazil	Tibau	2000	78.4%	81.9%	74.9%
Brazil	Tibau	2017	79.0%	82.5%	75.6%
Brazil	Tibau do Sul	2000	78.4%	81.1%	75.5%
Brazil	Tibau do Sul	2017	79.0%	81.7%	76.1%
Brazil	Tietê	2000	93.0%	94.4%	91.3%
Brazil	Tietê	2017	93.3%	94.6%	91.6%
Brazil	Tigrinhos	2000	79.2%	83.1%	75.0%
Brazil	Tigrinhos	2017	79.9%	83.7%	75.8%
Brazil	Tijucas	2000	71.2%	75.9%	65.6%
Brazil	Tijucas	2017	72.9%	77.4%	67.5%
Brazil	Tijucas do Sul	2000	82.6%	85.0%	79.7%
Brazil	Tijucas do Sul	2017	83.2%	85.5%	80.3%
Brazil	Timbaúba	2000	69.3%	72.4%	66.5%
Brazil	Timbaúba	2017	70.1%	73.1%	67.3%
Brazil	Timbaúba dos Batistas	2000	75.4%	78.8%	71.3%
Brazil	Timbaúba dos Batistas	2017	76.1%	79.5%	72.1%
Brazil	Timbé do Sul	2000	76.9%	81.6%	72.0%
Brazil	Timbé do Sul	2017	77.5%	82.1%	72.8%
Brazil	Timbiras	2000	62.4%	67.1%	57.5%
Brazil	Timbiras	2017	63.3%	68.0%	58.4%
Brazil	Timbó	2000	71.6%	75.2%	68.2%
Brazil	Timbó	2017	72.4%	75.9%	69.0%
Brazil	Timbó Grande	2000	78.1%	82.3%	74.0%
Brazil	Timbó Grande	2017	78.7%	82.9%	74.7%
Brazil	Timburi	2000	90.6%	92.9%	87.7%
Brazil	Timburi	2017	91.0%	93.1%	88.1%

Country	Second Administrative Unit	Year	Mean	Upper	Lower
Brazil	Timon	2000	60.8%	63.5%	58.2%
Brazil	Timon	2017	61.8%	64.4%	59.2%
Brazil	Timóteo	2000	81.4%	84.9%	77.4%
Brazil	Timóteo	2017	81.9%	85.4%	78.1%
Brazil	Tiradentes	2000	81.3%	85.6%	77.0%
Brazil	Tiradentes	2017	81.8%	86.0%	77.7%
Brazil	Tiradentes do Sul	2000	78.0%	82.9%	72.5%
Brazil	Tiradentes do Sul	2017	78.7%	83.4%	73.3%
Brazil	Tiros	2000	81.4%	86.4%	74.7%
Brazil	Tiros	2017	81.5%	86.5%	74.8%
Brazil	Tobias Barreto	2000	79.5%	82.3%	76.4%
Brazil	Tobias Barreto	2017	80.1%	82.9%	77.1%
Brazil	Tocantínia	2000	74.3%	78.4%	70.0%
Brazil	Tocantínia	2017	75.1%	79.1%	70.9%
Brazil	Tocantinópolis	2000	70.0%	74.3%	65.2%
Brazil	Tocantinópolis	2017	70.9%	75.1%	66.2%
Brazil	Tocantins	2000	81.7%	84.9%	78.0%
Brazil	Tocantins	2017	82.2%	85.4%	78.6%
Brazil	Tocos do Moji	2000	90.1%	92.2%	87.7%
Brazil	Tocos do Moji	2017	90.5%	92.5%	88.2%
Brazil	Toledo	2000	86.4%	89.1%	83.1%
Brazil	Toledo	2000	92.9%	94.3%	91.3%
Brazil	Toledo	2017	86.8%	89.5%	83.7%
Brazil	Toledo	2017	93.1%	94.5%	91.6%
Brazil	Tomar do Geru	2000	78.4%	81.3%	75.1%
Brazil	Tomar do Geru	2017	79.0%	81.9%	75.8%
Brazil	Tomazina	2000	87.6%	90.2%	84.2%
Brazil	Tomazina	2017	88.0%	90.6%	84.8%
Brazil	Tombos	2000	79.7%	83.1%	76.3%
Brazil	Tombos	2017	80.3%	83.7%	77.1%
Brazil	Tomé-Açu	2000	36.5%	41.2%	31.7%
Brazil	Tomé-Açu	2017	37.4%	42.2%	32.5%
Brazil	Tonantins	2000	55.9%	64.7%	46.6%
Brazil	Tonantins	2017	56.8%	65.6%	47.5%
Brazil	Toritama	2000	67.6%	70.9%	64.4%
Brazil	Toritama	2017	68.4%	71.7%	65.3%
Brazil	Torixoréu	2000	71.5%	77.8%	64.7%
Brazil	Torixoréu	2017	72.3%	78.5%	65.6%
Brazil	Toropi	2000	81.7%	85.8%	76.5%
Brazil	Toropi	2017	82.3%	86.3%	77.3%
Brazil	Torre de Pedra	2000	93.1%	94.8%	91.0%
Brazil	Torre de Pedra	2017	93.4%	94.9%	91.3%
Brazil	Torres	2000	76.8%	81.8%	71.5%
Brazil	Torres	2017	77.8%	82.6%	72.6%
Brazil	Torrinha	2000	93.2%	94.7%	91.2%
Brazil	Torrinha	2017	93.4%	94.9%	91.6%
Brazil	Touros	2000	84.3%	87.4%	80.6%
Brazil	Touros	2017	84.9%	87.8%	81.2%
Brazil	Trabiju	2000	93.5%	95.1%	91.8%
Brazil	Trabiju	2017	93.7%	95.3%	92.1%
Brazil	Tracuateua	2000	38.1%	43.1%	33.5%
Brazil	Tracuateua	2017	39.1%	44.1%	34.4%
Brazil	Tracunhaém	2000	67.6%	70.7%	65.2%
Brazil	Tracunhaém	2017	69.6%	72.6%	67.3%

Country	Second Administrative Unit	Year	Mean	Upper	Lower
Brazil	Traipu	2000	66.4%	69.3%	63.1%
Brazil	Traipu	2017	67.3%	70.1%	64.0%
Brazil	Trairão	2000	44.4%	53.1%	36.1%
Brazil	Trairão	2017	45.3%	54.1%	36.9%
Brazil	Trairi	2000	67.5%	71.6%	62.9%
Brazil	Trairi	2017	68.4%	72.4%	63.8%
Brazil	Trajano de Morais	2000	81.6%	84.6%	78.6%
Brazil	Trajano de Morais	2017	82.2%	85.1%	79.2%
Brazil	Tramandaí	2000	79.8%	84.4%	74.9%
Brazil	Tramandaí	2017	80.5%	84.9%	75.6%
Brazil	Travesseiro	2000	80.7%	84.5%	76.8%
Brazil	Travesseiro	2017	81.3%	85.0%	77.5%
Brazil	Tremedal	2000	78.7%	83.8%	72.6%
Brazil	Tremedal	2017	79.4%	84.3%	73.4%
Brazil	Tremembé	2000	91.3%	93.0%	89.2%
Brazil	Tremembé	2017	91.6%	93.3%	89.6%
Brazil	Três Arroios	2000	78.0%	81.4%	73.9%
Brazil	Três Arroios	2017	78.6%	82.0%	74.6%
Brazil	Três Barras do Paraná	2000	84.0%	87.1%	80.5%
Brazil	Três Barras do Paraná	2017	84.5%	87.6%	81.0%
Brazil	Três Cachoeiras	2000	76.7%	81.2%	71.8%
Brazil	Três Cachoeiras	2017	77.7%	82.0%	72.9%
Brazil	Três Corações	2000	84.6%	87.7%	80.6%
Brazil	Três Corações	2017	85.1%	88.1%	81.2%
Brazil	Três Coroas	2000	80.9%	83.9%	77.5%
Brazil	Três Coroas	2017	81.5%	84.4%	78.2%
Brazil	Três de Maio	2000	81.2%	85.8%	76.3%
Brazil	Três de Maio	2017	81.8%	86.3%	77.0%
Brazil	Três Forquilhas	2000	78.4%	82.6%	73.6%
Brazil	Três Forquilhas	2017	79.0%	83.2%	74.3%
Brazil	Três Fronteiras	2000	86.8%	90.9%	82.9%
Brazil	Três Fronteiras	2017	87.2%	91.2%	83.5%
Brazil	Três Lagoas	2000	85.5%	88.4%	82.0%
Brazil	Três Lagoas	2017	86.0%	88.8%	82.6%
Brazil	Três Marias	2000	81.9%	86.9%	75.5%
Brazil	Três Marias	2017	82.5%	87.4%	76.2%
Brazil	Três Palmeiras	2000	79.5%	82.8%	76.1%
Brazil	Três Palmeiras	2017	80.1%	83.4%	76.8%
Brazil	Três Passos	2000	81.2%	85.4%	76.8%
Brazil	Três Passos	2017	81.8%	85.9%	77.5%
Brazil	Três Pontas	2000	84.0%	87.5%	80.0%
Brazil	Três Pontas	2017	84.5%	87.9%	80.6%
Brazil	Três Ranchos	2000	77.9%	83.2%	72.2%
Brazil	Três Ranchos	2017	78.5%	83.7%	73.0%
Brazil	Três Rios	2000	76.9%	80.0%	73.3%
Brazil	Três Rios	2017	77.6%	80.6%	74.1%
Brazil	Treviso	2000	76.6%	80.4%	72.1%
Brazil	Treviso	2017	77.3%	81.0%	72.8%
Brazil	Treze de Maio	2000	73.7%	77.9%	69.3%
Brazil	Treze de Maio	2017	74.4%	78.5%	70.0%

Country	Second Administrative Unit	Year	Mean	Upper	Lower
Brazil	Treze Tílias	2000	76.3%	80.3%	72.3%
Brazil	Treze Tílias	2017	77.0%	80.9%	73.0%
Brazil	Trindade	2000	78.3%	80.6%	76.3%
Brazil	Trindade	2000	67.9%	73.0%	63.5%
Brazil	Trindade	2017	68.8%	73.8%	64.4%
Brazil	Trindade	2017	79.1%	81.3%	77.1%
Brazil	Trindade do Sul	2000	79.2%	82.7%	75.7%
Brazil	Trindade do Sul	2017	79.9%	83.2%	76.4%
Brazil	Triunfo	2000	72.6%	76.2%	68.9%
Brazil	Triunfo	2000	80.8%	83.6%	77.8%
Brazil	Triunfo	2000	68.4%	73.0%	63.1%
Brazil	Triunfo	2017	73.4%	76.9%	69.7%
Brazil	Triunfo	2017	69.3%	73.8%	64.0%
Brazil	Triunfo	2017	81.4%	84.1%	78.5%
Brazil	Triunfo Potiguar	2000	80.3%	83.3%	77.0%
Brazil	Triunfo Potiguar	2017	80.9%	83.8%	77.7%
Brazil	Trizidela do Vale	2000	59.9%	65.3%	54.8%
Brazil	Trizidela do Vale	2017	60.9%	66.2%	55.7%
Brazil	Trombas	2000	78.7%	84.0%	73.0%
Brazil	Trombas	2017	79.3%	84.5%	73.8%
Brazil	Trombudo Central	2000	72.8%	77.1%	68.2%
Brazil	Trombudo Central	2017	73.6%	77.8%	69.1%
Brazil	Tubarão	2000	73.8%	78.2%	68.8%
Brazil	Tubarão	2017	75.0%	79.3%	70.2%
Brazil	Tucano	2000	77.4%	81.4%	72.9%
Brazil	Tucano	2017	78.1%	82.0%	73.7%
Brazil	Tucumã	2000	39.0%	46.9%	31.9%
Brazil	Tucumã	2017	39.9%	47.9%	32.8%
Brazil	Tucunduva	2000	80.3%	84.9%	75.0%
Brazil	Tucunduva	2017	80.9%	85.4%	75.7%
Brazil	Tucuruí	2000	38.5%	43.2%	32.7%
Brazil	Tucuruí	2017	39.4%	44.1%	33.5%
Brazil	Tufilândia	2000	60.0%	65.4%	55.4%
Brazil	Tufilândia	2017	60.9%	66.3%	56.4%
Brazil	Tuiuti	2000	92.2%	93.7%	90.6%
Brazil	Tuiuti	2017	92.5%	93.9%	90.9%
Brazil	Tumiritinga	2000	78.1%	82.7%	73.5%
Brazil	Tumiritinga	2017	78.7%	83.2%	74.3%
Brazil	Tunápolis	2000	79.6%	83.8%	75.5%
Brazil	Tunápolis	2017	80.2%	84.2%	76.1%
Brazil	Tunas	2000	82.5%	85.9%	78.3%
Brazil	Tunas	2017	83.1%	86.4%	79.0%
Brazil	Tunas do Paraná	2000	87.1%	89.9%	84.0%
Brazil	Tunas do Paraná	2017	87.5%	90.3%	84.5%
Brazil	Tuneiras do Oeste	2000	84.2%	87.4%	80.8%
Brazil	Tuneiras do Oeste	2017	84.8%	87.8%	81.4%
Brazil	Tuntum	2000	61.9%	67.2%	56.7%
Brazil	Tuntum	2017	62.8%	68.1%	57.7%
Brazil	Tupã	2000	92.2%	94.4%	89.7%
Brazil	Tupã	2017	92.5%	94.6%	90.0%

Country	Second Administrative Unit	Year	Mean	Upper	Lower
Brazil	Tupaciguara	2000	81.7%	85.5%	77.0%
Brazil	Tupaciguara	2017	82.3%	86.0%	77.6%
Brazil	Tupanatinga	2000	63.2%	67.3%	58.7%
Brazil	Tupanatinga	2017	64.0%	68.2%	59.6%
Brazil	Tupanci do Sul	2000	78.5%	82.3%	74.2%
Brazil	Tupanci do Sul	2017	79.1%	82.9%	75.0%
Brazil	Tupanciretã	2000	84.4%	87.9%	80.1%
Brazil	Tupanciretã	2017	84.9%	88.3%	80.7%
Brazil	Tupandi	2000	82.4%	85.1%	79.7%
Brazil	Tupandi	2017	83.0%	85.6%	80.4%
Brazil	Tuparendi	2000	82.2%	86.8%	77.3%
Brazil	Tuparendi	2017	82.8%	87.2%	78.0%
Brazil	Tuparetama	2000	67.6%	71.0%	63.4%
Brazil	Tuparetama	2017	68.4%	71.8%	64.3%
Brazil	Tupãssi	2000	84.6%	88.1%	80.8%
Brazil	Tupãssi	2017	85.1%	88.5%	81.4%
Brazil	Tupi Paulista	2000	88.8%	91.3%	85.6%
Brazil	Tupi Paulista	2017	89.2%	91.6%	86.1%
Brazil	Tupirama	2000	75.1%	79.3%	70.9%
Brazil	Tupirama	2017	75.8%	79.9%	71.7%
Brazil	Tupiratins	2000	73.6%	78.7%	68.8%
Brazil	Tupiratins	2017	74.3%	79.3%	69.6%
Brazil	Turiaçu	2000	52.8%	59.7%	46.5%
Brazil	Turiaçu	2017	53.9%	60.8%	47.7%
Brazil	Turilândia	2000	57.0%	62.8%	50.7%
Brazil	Turilândia	2017	58.0%	63.7%	51.7%
Brazil	Turiúba	2000	91.3%	93.7%	88.5%
Brazil	Turiúba	2017	91.6%	94.0%	88.9%
Brazil	Turmalina	2000	88.1%	91.3%	84.4%
Brazil	Turmalina	2000	82.2%	87.0%	77.1%
Brazil	Turmalina	2017	82.7%	87.4%	77.8%
Brazil	Turmalina	2017	88.5%	91.6%	84.9%
Brazil	Turuçu	2000	80.9%	84.5%	76.1%
Brazil	Turuçu	2017	81.5%	85.0%	76.8%
Brazil	Tururu	2000	70.2%	73.6%	65.8%
Brazil	Tururu	2017	72.1%	75.3%	67.8%
Brazil	Turvânia	2000	76.2%	79.8%	71.8%
Brazil	Turvânia	2017	76.9%	80.5%	72.6%
Brazil	Turvelândia	2000	76.9%	81.3%	72.0%
Brazil	Turvelândia	2017	77.6%	81.9%	72.8%
Brazil	Turvo	2000	79.5%	83.2%	75.4%
Brazil	Turvo	2000	83.5%	87.8%	79.5%
Brazil	Turvo	2017	80.1%	83.8%	76.1%
Brazil	Turvo	2017	84.0%	88.2%	80.1%
Brazil	Turvolandia	2000	85.9%	88.8%	82.5%
Brazil	Turvolandia	2017	86.3%	89.2%	83.1%
Brazil	Tutoia	2000	60.2%	65.5%	55.2%
Brazil	Tutoia	2017	61.1%	66.3%	56.2%
Brazil	Tutóia	2000	60.1%	65.7%	54.6%
Brazil	Tutóia	2017	61.0%	66.6%	55.6%
Brazil	Uarini	2000	57.4%	63.9%	50.4%
Brazil	Uarini	2017	58.4%	64.8%	51.4%
Brazil	Uauá	2000	74.5%	79.9%	68.4%
Brazil	Uauá	2017	75.2%	80.5%	69.2%
Brazil	Ubá	2000	80.7%	83.9%	76.9%
Brazil	Ubá	2017	81.3%	84.5%	77.6%
Brazil	Ubaí	2000	81.7%	87.1%	75.2%
Brazil	Ubaí	2017	82.3%	87.6%	75.9%
Brazil	Ubaíra	2000	79.4%	83.0%	74.3%
Brazil	Ubaíra	2017	80.0%	83.5%	75.0%

Country	Second Administrative Unit	Year	Mean	Upper	Lower
Brazil	Ubaitaba	2000	75.4%	80.2%	70.3%
Brazil	Ubaitaba	2017	76.3%	80.9%	71.4%
Brazil	Ubajara	2000	69.4%	73.3%	65.2%
Brazil	Ubajara	2017	70.2%	74.1%	66.1%
Brazil	Ubaporanga	2000	82.7%	86.2%	78.5%
Brazil	Ubaporanga	2017	83.2%	86.7%	79.1%
Brazil	Ubarana	2000	92.7%	94.8%	90.5%
Brazil	Ubarana	2017	93.0%	95.0%	90.8%
Brazil	Ubatã	2000	76.8%	81.2%	72.4%
Brazil	Ubatã	2017	77.5%	81.8%	73.2%
Brazil	Ubatuba	2000	88.3%	91.3%	84.9%
Brazil	Ubatuba	2017	88.7%	91.6%	85.3%
Brazil	Uberaba	2000	86.6%	89.7%	82.1%
Brazil	Uberaba	2017	87.1%	90.0%	82.7%
Brazil	Uberlândia	2000	82.6%	86.2%	78.3%
Brazil	Uberlândia	2017	83.2%	86.7%	79.0%
Brazil	Ubirajara	2000	92.4%	94.4%	90.0%
Brazil	Ubirajara	2017	92.7%	94.6%	90.4%
Brazil	Ubiratã	2000	83.1%	86.8%	78.7%
Brazil	Ubiratã	2017	83.6%	87.3%	79.4%
Brazil	Ubiretama	2000	81.4%	85.9%	76.2%
Brazil	Ubiretama	2017	82.0%	86.4%	77.0%
Brazil	Uchoa	2000	92.5%	94.2%	90.2%
Brazil	Uchoa	2017	92.7%	94.4%	90.6%
Brazil	Uibaí	2000	75.2%	81.1%	68.1%
Brazil	Uibaí	2017	76.0%	81.7%	69.0%
Brazil	Uiramutã	2000	88.2%	91.8%	83.7%
Brazil	Uiramutã	2017	88.6%	92.1%	84.2%
Brazil	Uirapuru	2000	75.5%	81.3%	67.8%
Brazil	Uirapuru	2017	76.2%	81.9%	68.7%
Brazil	Uiraúna	2000	75.5%	79.0%	72.1%
Brazil	Uiraúna	2017	76.2%	79.6%	72.9%
Brazil	Ulianópolis	2000	50.4%	57.0%	43.7%
Brazil	Ulianópolis	2017	51.4%	57.9%	44.8%
Brazil	Umari	2000	71.9%	75.7%	68.5%
Brazil	Umari	2017	72.7%	76.4%	69.4%
Brazil	Umarizal	2000	77.7%	80.7%	74.1%
Brazil	Umarizal	2017	78.4%	81.3%	74.9%
Brazil	Umbauba	2000	79.2%	82.2%	76.1%
Brazil	Umbauba	2017	79.9%	82.8%	76.9%
Brazil	Umburanas	2000	75.4%	81.6%	68.4%
Brazil	Umburanas	2017	76.1%	82.2%	69.2%
Brazil	Umburatiba	2000	79.1%	83.6%	73.6%
Brazil	Umburatiba	2017	79.7%	84.1%	74.3%
Brazil	Umbuzeiro	2000	63.6%	67.4%	60.6%
Brazil	Umbuzeiro	2017	64.6%	68.2%	61.6%
Brazil	Umirim	2000	71.6%	74.8%	67.6%
Brazil	Umirim	2017	72.4%	75.5%	68.5%
Brazil	Umuarama	2000	85.1%	88.3%	81.8%
Brazil	Umuarama	2017	85.6%	88.7%	82.4%
Brazil	Una	2000	75.9%	80.4%	70.1%
Brazil	Una	2017	76.6%	81.0%	71.0%
Brazil	Unaí	2000	86.3%	90.5%	81.1%
Brazil	Unaí	2017	86.7%	90.8%	81.7%
Brazil	União	2000	60.3%	64.1%	56.8%
Brazil	União	2017	61.3%	65.0%	57.7%
Brazil	União da Serra	2000	81.1%	84.6%	77.3%
Brazil	União da Serra	2017	81.7%	85.1%	78.0%
Brazil	União da Vitória	2000	80.1%	84.4%	75.3%

Country	Second Administrative Unit	Year	Mean	Upper	Lower
Brazil	União da Vitória	2017	80.7%	84.9%	76.1%
Brazil	União de Minas	2000	84.3%	88.1%	80.3%
Brazil	União de Minas	2017	84.8%	88.5%	80.9%
Brazil	União do Oeste	2000	77.6%	82.0%	73.2%
Brazil	União do Oeste	2017	78.3%	82.5%	74.0%
Brazil	União do Sul	2000	66.2%	73.3%	58.3%
Brazil	União do Sul	2017	67.0%	74.1%	59.2%
Brazil	União dos Palmares	2000	62.5%	65.2%	59.2%
Brazil	União dos Palmares	2017	63.4%	66.1%	60.2%
Brazil	União Paulista	2000	92.1%	94.3%	89.4%
Brazil	União Paulista	2017	92.4%	94.5%	89.8%
Brazil	Uniflor	2000	84.4%	87.2%	81.3%
Brazil	Uniflor	2017	84.9%	87.7%	81.9%
Brazil	Unistalda	2000	82.7%	86.8%	76.9%
Brazil	Unistalda	2017	83.3%	87.2%	77.6%
Brazil	Upanema	2000	81.0%	83.8%	77.7%
Brazil	Upanema	2017	81.6%	84.3%	78.3%
Brazil	Uraí	2000	86.9%	88.9%	84.1%
Brazil	Uraí	2017	87.3%	89.3%	84.6%
Brazil	Urandi	2000	78.9%	83.9%	72.7%
Brazil	Urandi	2017	79.5%	84.4%	73.5%
Brazil	Urânia	2000	89.0%	92.2%	85.7%
Brazil	Urânia	2017	89.4%	92.5%	86.2%
Brazil	Urbano Santos	2000	62.5%	68.0%	55.9%
Brazil	Urbano Santos	2017	63.4%	68.9%	56.9%
Brazil	urea	2000	78.0%	81.9%	73.9%
Brazil	urea	2017	78.7%	82.4%	74.7%
Brazil	Uru	2000	93.1%	95.0%	90.7%
Brazil	Uru	2017	93.4%	95.2%	91.0%
Brazil	Uruaçu	2000	80.6%	85.1%	76.4%
Brazil	Uruaçu	2017	81.2%	85.6%	77.1%
Brazil	Uruana	2000	78.3%	82.1%	74.5%
Brazil	Uruana	2017	79.0%	82.7%	75.2%
Brazil	Uruana de Minas	2000	85.0%	90.1%	78.9%
Brazil	Uruana de Minas	2017	85.5%	90.4%	79.5%
Brazil	Uruará	2000	40.2%	47.7%	32.9%
Brazil	Uruará	2017	41.1%	48.6%	33.8%
Brazil	Urubici	2000	78.2%	82.1%	73.4%
Brazil	Urubici	2017	79.0%	82.7%	74.2%
Brazil	Uruburetama	2000	72.0%	75.4%	68.0%
Brazil	Uruburetama	2017	72.8%	76.2%	69.0%
Brazil	Urucânia	2000	80.9%	84.7%	76.6%
Brazil	Urucânia	2017	81.5%	85.2%	77.3%
Brazil	Urucará	2000	59.1%	64.7%	53.5%
Brazil	Urucará	2017	60.0%	65.6%	54.4%
Brazil	Uruçuca	2000	74.7%	79.1%	69.7%
Brazil	Uruçuca	2017	75.4%	79.8%	70.6%
Brazil	Uruçuí	2000	61.7%	68.4%	53.6%
Brazil	Uruçuí	2017	62.5%	69.2%	54.5%

Country	Second Administrative Unit	Year	Mean	Upper	Lower
Brazil	Urucuia	2000	83.0%	88.7%	77.4%
Brazil	Urucuia	2017	83.9%	89.4%	78.5%
Brazil	Urucurituba	2000	59.9%	64.5%	53.7%
Brazil	Urucurituba	2017	60.9%	65.4%	54.6%
Brazil	Uruguaiana	2000	82.4%	89.2%	74.5%
Brazil	Uruguaiana	2017	82.9%	89.5%	75.2%
Brazil	Uruoca	2000	67.7%	72.3%	62.8%
Brazil	Uruoca	2017	68.6%	73.1%	63.7%
Brazil	Urupá	2000	27.9%	31.1%	24.6%
Brazil	Urupá	2017	28.6%	31.9%	25.4%
Brazil	Urupema	2000	80.8%	84.8%	76.4%
Brazil	Urupema	2017	81.4%	85.3%	77.1%
Brazil	Urupês	2000	92.9%	94.6%	90.7%
Brazil	Urupês	2017	93.2%	94.8%	91.1%
Brazil	Urussanga	2000	73.1%	77.3%	68.9%
Brazil	Urussanga	2017	73.8%	78.0%	69.8%
Brazil	Urutaí	2000	78.7%	82.9%	73.5%
Brazil	Urutaí	2017	79.3%	83.5%	74.3%
Brazil	Utinga	2000	79.8%	84.8%	75.0%
Brazil	Utinga	2017	80.4%	85.3%	75.7%
Brazil	Vacaria	2000	81.2%	85.0%	77.1%
Brazil	Vacaria	2017	81.8%	85.5%	77.7%
Brazil	Vale do Anari	2000	31.9%	36.3%	26.9%
Brazil	Vale do Anari	2017	32.9%	37.3%	27.7%
Brazil	Vale do Paraíso	2000	30.4%	33.8%	26.7%
Brazil	Vale do Paraíso	2017	31.2%	34.7%	27.5%
Brazil	Vale do Sol	2000	81.0%	84.5%	76.4%
Brazil	Vale do Sol	2017	81.6%	85.0%	77.2%
Brazil	Vale Real	2000	81.3%	84.1%	78.6%
Brazil	Vale Real	2017	81.9%	84.7%	79.2%
Brazil	Vale Verde	2000	81.0%	84.7%	76.9%
Brazil	Vale Verde	2017	81.6%	85.2%	77.6%
Brazil	Valença	2000	73.0%	77.7%	68.3%
Brazil	Valença	2017	73.7%	78.4%	69.2%
Brazil	Valença do Piauí	2000	61.3%	67.0%	56.1%
Brazil	Valença do Piauí	2017	62.2%	67.9%	57.1%
Brazil	Valencia	2000	81.3%	84.3%	78.3%
Brazil	Valencia	2017	81.9%	84.8%	79.0%
Brazil	Valente	2000	79.0%	82.7%	74.1%
Brazil	Valente	2017	79.6%	83.3%	74.9%
Brazil	Valentim Gentil	2000	90.6%	93.6%	87.6%
Brazil	Valentim Gentil	2017	90.9%	93.8%	88.0%
Brazil	Valinhos	2000	93.3%	94.4%	92.0%
Brazil	Valinhos	2017	93.6%	94.6%	92.3%
Brazil	Valparaíso	2000	90.5%	93.0%	87.6%
Brazil	Valparaíso	2017	90.8%	93.2%	88.0%
Brazil	Valparaíso de Goiás	2000	90.7%	91.8%	89.5%
Brazil	Valparaíso de Goiás	2017	91.0%	92.1%	89.8%
Brazil	Vanini	2000	80.6%	84.2%	76.3%
Brazil	Vanini	2017	81.2%	84.7%	77.0%
Brazil	Vargeão	2000	76.9%	81.3%	72.0%
Brazil	Vargeão	2017	77.6%	81.9%	72.9%
Brazil	Vargem	2000	92.3%	93.7%	90.7%
Brazil	Vargem	2000	75.4%	79.8%	69.8%

Country	Second Administrative Unit	Year	Mean	Upper	Lower
Brazil	Vargem	2017	76.1%	80.4%	70.6%
Brazil	Vargem	2017	92.6%	93.9%	91.0%
Brazil	Vargem Alegre	2000	81.2%	84.9%	76.8%
Brazil	Vargem Alegre	2017	81.8%	85.3%	77.4%
Brazil	Vargem Alta	2000	80.1%	82.6%	76.9%
Brazil	Vargem Alta	2017	80.7%	83.1%	77.6%
Brazil	Vargem Bonita	2000	77.6%	81.7%	73.1%
Brazil	Vargem Bonita	2000	83.7%	88.1%	78.7%
Brazil	Vargem Bonita	2017	84.3%	88.6%	79.5%
Brazil	Vargem Bonita	2017	78.2%	82.2%	73.8%
Brazil	Vargem Grande	2000	60.3%	65.3%	54.9%
Brazil	Vargem Grande	2017	61.3%	66.2%	55.9%
Brazil	Vargem Grande do Rio Pardo	2000	81.5%	86.4%	74.6%
Brazil	Vargem Grande do Rio Pardo	2017	82.1%	86.8%	75.3%
Brazil	Vargem Grande do Sul	2000	91.3%	93.3%	88.8%
Brazil	Vargem Grande do Sul	2017	91.6%	93.5%	89.2%
Brazil	Vargem Grande Paulista	2000	94.0%	95.0%	93.0%
Brazil	Vargem Grande Paulista	2017	94.4%	95.3%	93.3%
Brazil	Varginha	2000	84.7%	87.7%	80.9%
Brazil	Varginha	2017	85.2%	88.1%	81.5%
Brazil	Varjao	2000	75.7%	79.1%	72.0%
Brazil	Varjao	2017	76.4%	79.7%	72.7%
Brazil	Varjão de Minas	2000	80.6%	86.7%	73.0%
Brazil	Varjão de Minas	2017	81.2%	87.1%	73.8%
Brazil	Varjota	2000	66.0%	70.6%	61.5%
Brazil	Varjota	2017	66.9%	71.4%	62.4%
Brazil	Varre-Sai	2000	80.2%	83.6%	77.0%
Brazil	Varre-Sai	2017	80.8%	84.1%	77.7%
Brazil	Várzea	2000	78.7%	80.6%	76.8%
Brazil	Várzea	2017	79.2%	81.1%	77.4%
Brazil	Várzea Alegre	2000	69.4%	73.0%	65.5%
Brazil	Várzea Alegre	2017	70.9%	74.3%	67.1%
Brazil	Várzea Branca	2000	65.1%	70.6%	57.3%
Brazil	Várzea Branca	2017	65.8%	71.2%	58.2%
Brazil	Várzea da Palma	2000	81.1%	86.6%	75.1%
Brazil	Várzea da Palma	2017	82.9%	87.9%	77.2%
Brazil	Várzea da Roça	2000	78.9%	83.2%	74.6%
Brazil	Várzea da Roça	2017	79.6%	83.7%	75.3%

Country	Second Administrative Unit	Year	Mean	Upper	Lower
Brazil	Várzea do Poço	2000	79.1%	83.4%	74.7%
Brazil	Várzea do Poço	2017	79.8%	84.0%	75.5%
Brazil	Várzea Grande	2000	68.3%	71.2%	65.3%
Brazil	Várzea Grande	2000	61.4%	65.9%	56.2%
Brazil	Várzea Grande	2017	69.1%	72.0%	66.2%
Brazil	Várzea Grande	2017	62.4%	66.9%	57.2%
Brazil	Várzea Nova	2000	80.4%	85.1%	75.7%
Brazil	Várzea Nova	2017	81.0%	85.6%	76.4%
Brazil	Várzea Paulista	2000	94.4%	95.3%	93.4%
Brazil	Várzea Paulista	2017	94.6%	95.5%	93.7%
Brazil	Varzedo	2000	78.9%	82.6%	74.5%
Brazil	Varzedo	2017	79.6%	83.2%	75.3%
Brazil	Varzelândia	2000	81.6%	86.6%	75.8%
Brazil	Varzelândia	2017	81.9%	86.8%	76.1%
Brazil	Vassouras	2000	80.4%	83.1%	77.4%
Brazil	Vassouras	2017	81.1%	83.6%	78.1%
Brazil	Venâncio Aires	2000	80.7%	84.2%	76.4%
Brazil	Venâncio Aires	2017	82.0%	85.3%	78.0%
Brazil	Venceslau Bras	2000	88.7%	91.3%	85.6%
Brazil	Venceslau Bras	2017	89.1%	91.6%	86.1%
Brazil	Venda Nova do Imigrante	2000	80.9%	84.0%	78.0%
Brazil	Venda Nova do Imigrante	2017	81.5%	84.5%	78.7%
Brazil	Venha-Ver	2000	76.1%	80.0%	72.2%
Brazil	Venha-Ver	2017	76.7%	80.5%	72.8%
Brazil	Ventania	2000	86.2%	89.0%	82.7%
Brazil	Ventania	2017	86.6%	89.4%	83.2%
Brazil	Venturosa	2000	65.8%	69.9%	61.4%
Brazil	Venturosa	2017	66.7%	70.7%	62.3%
Brazil	Vera	2000	67.9%	74.9%	61.8%
Brazil	Vera	2017	68.7%	75.6%	62.7%
Brazil	Vera Cruz	2000	73.7%	77.0%	70.1%
Brazil	Vera Cruz	2000	81.1%	84.8%	76.3%
Brazil	Vera Cruz	2000	81.7%	83.7%	79.5%
Brazil	Vera Cruz	2000	93.4%	95.1%	91.0%
Brazil	Vera Cruz	2017	82.6%	84.6%	80.6%
Brazil	Vera Cruz	2017	81.7%	85.3%	77.0%
Brazil	Vera Cruz	2017	74.5%	77.7%	71.0%
Brazil	Vera Cruz	2017	93.6%	95.3%	91.3%
Brazil	Vera Cruz do Oeste	2000	84.8%	88.0%	81.4%
Brazil	Vera Cruz do Oeste	2017	85.3%	88.4%	82.0%
Brazil	Vera Mendes	2000	59.4%	65.0%	53.9%
Brazil	Vera Mendes	2017	60.4%	65.9%	54.9%
Brazil	Veranópolis	2000	81.9%	85.2%	78.7%
Brazil	Veranópolis	2017	82.5%	85.7%	79.3%
Brazil	Verdejante	2000	67.8%	71.9%	63.1%
Brazil	Verdejante	2017	68.6%	72.7%	64.0%

Country	Second Administrative Unit	Year	Mean	Upper	Lower
Brazil	Verdelândia	2000	81.2%	86.0%	75.4%
Brazil	Verdelândia	2017	81.8%	86.5%	76.1%
Brazil	Verê	2000	81.8%	85.5%	77.4%
Brazil	Verê	2017	82.3%	86.0%	78.0%
Brazil	Vereda	2000	80.3%	84.7%	74.6%
Brazil	Vereda	2017	80.9%	85.2%	75.3%
Brazil	Veredinha	2000	82.3%	87.1%	76.8%
Brazil	Veredinha	2017	82.9%	87.5%	77.5%
Brazil	Veríssimo	2000	86.0%	89.1%	82.0%
Brazil	Veríssimo	2017	86.5%	89.5%	82.6%
Brazil	Vermelho Novo	2000	82.1%	85.8%	78.3%
Brazil	Vermelho Novo	2017	82.7%	86.2%	79.0%
Brazil	Vertente do Lério	2000	64.3%	67.8%	60.7%
Brazil	Vertente do Lério	2017	65.2%	68.7%	61.7%
Brazil	Vertentes	2000	68.7%	72.0%	65.5%
Brazil	Vertentes	2017	69.5%	72.7%	66.3%
Brazil	Vespasiano	2000	81.7%	84.1%	78.7%
Brazil	Vespasiano	2017	82.7%	85.1%	79.9%
Brazil	Vespasiano Correa	2000	82.3%	85.6%	78.8%
Brazil	Vespasiano Correa	2017	82.9%	86.2%	79.5%
Brazil	Viadutos	2000	77.1%	80.9%	72.6%
Brazil	Viadutos	2017	77.8%	81.5%	73.4%
Brazil	Viamão	2000	83.0%	85.1%	81.0%
Brazil	Viamão	2017	83.6%	85.6%	81.6%
Brazil	Viana	2000	60.7%	64.9%	56.1%
Brazil	Viana	2000	75.8%	77.9%	73.2%
Brazil	Viana	2017	76.4%	78.6%	73.9%
Brazil	Viana	2017	61.6%	65.8%	57.1%
Brazil	Vianópolis	2000	80.6%	83.6%	77.3%
Brazil	Vianópolis	2017	81.3%	84.2%	78.0%
Brazil	Vicência	2000	67.0%	70.2%	64.1%
Brazil	Vicência	2017	67.9%	71.0%	65.0%
Brazil	Vicente Dutra	2000	77.1%	81.2%	72.2%
Brazil	Vicente Dutra	2017	77.7%	81.7%	72.9%
Brazil	Vicentina	2000	79.9%	83.4%	76.2%
Brazil	Vicentina	2017	80.5%	83.9%	76.9%
Brazil	Vicentinópolis	2000	77.0%	81.1%	72.4%
Brazil	Vicentinópolis	2017	77.7%	81.7%	73.2%
Brazil	Viçosa	2000	78.4%	81.6%	75.1%
Brazil	Viçosa	2000	59.4%	62.7%	56.5%
Brazil	Viçosa	2000	81.9%	85.0%	77.9%
Brazil	Viçosa	2017	79.0%	82.2%	75.8%
Brazil	Viçosa	2017	60.3%	63.6%	57.4%
Brazil	Viçosa	2017	82.5%	85.5%	78.6%
Brazil	Viçosa do Ceará	2000	69.6%	73.5%	65.1%
Brazil	Viçosa do Ceará	2017	70.4%	74.3%	66.0%
Brazil	Victor Graeff	2000	81.5%	85.2%	77.1%
Brazil	Victor Graeff	2017	82.1%	85.7%	77.7%
Brazil	Victorino Freire	2000	61.3%	66.5%	56.5%
Brazil	Victorino Freire	2017	62.2%	67.4%	57.5%
Brazil	Vidal Ramos	2000	75.0%	78.6%	70.7%
Brazil	Vidal Ramos	2017	75.8%	79.3%	71.6%

Country	Second Administrative Unit	Year	Mean	Upper	Lower
Brazil	Videira	2000	77.1%	81.7%	73.3%
Brazil	Videira	2017	77.8%	82.3%	74.0%
Brazil	Vieiras	2000	81.4%	84.8%	77.8%
Brazil	Vieiras	2017	82.0%	85.3%	78.5%
Brazil	Vieirópolis	2000	74.7%	77.9%	71.2%
Brazil	Vieirópolis	2017	75.4%	78.6%	72.0%
Brazil	Vigia	2000	35.0%	40.0%	30.9%
Brazil	Vigia	2017	35.9%	40.9%	31.7%
Brazil	Vila Alta	2000	82.8%	86.0%	78.2%
Brazil	Vila Alta	2017	83.3%	86.4%	78.8%
Brazil	Vila Boa	2000	85.4%	89.1%	80.7%
Brazil	Vila Boa	2017	85.9%	89.5%	81.3%
Brazil	Vila Flor	2000	75.7%	78.8%	72.5%
Brazil	Vila Flor	2017	76.5%	79.5%	73.3%
Brazil	Vila Flores	2000	81.4%	84.8%	78.2%
Brazil	Vila Flores	2017	82.0%	85.3%	78.9%
Brazil	Vila Lângaro	2000	80.1%	83.9%	75.7%
Brazil	Vila Lângaro	2017	80.7%	84.4%	76.4%
Brazil	Vila Maria	2000	80.8%	84.1%	76.4%
Brazil	Vila Maria	2017	81.4%	84.7%	77.2%
Brazil	Vila Nova do Piauí	2000	63.6%	68.0%	59.5%
Brazil	Vila Nova do Piauí	2017	64.5%	68.8%	60.4%
Brazil	Vila Nova do Sul	2000	81.9%	86.6%	76.9%
Brazil	Vila Nova do Sul	2017	82.4%	87.1%	77.6%
Brazil	Vila Nova dos Martírios	2000	57.7%	62.6%	52.4%
Brazil	Vila Nova dos Martírios	2017	59.1%	63.8%	53.9%
Brazil	Vila Pavão	2000	78.6%	82.5%	74.8%
Brazil	Vila Pavão	2017	79.2%	83.0%	75.5%
Brazil	Vila Propício	2000	83.8%	86.7%	80.3%
Brazil	Vila Propício	2017	84.3%	87.1%	81.0%
Brazil	Vila Rica	2000	61.6%	70.0%	52.5%
Brazil	Vila Rica	2017	62.5%	70.8%	53.5%
Brazil	Vila Valério	2000	78.7%	81.8%	75.2%
Brazil	Vila Valério	2017	79.3%	82.3%	75.9%
Brazil	Vila Velha	2000	75.8%	78.1%	73.2%
Brazil	Vila Velha	2017	76.5%	78.7%	73.9%
Brazil	Vilhena	2000	38.0%	43.0%	32.8%
Brazil	Vilhena	2017	38.9%	44.0%	33.7%
Brazil	Vinhedo	2000	93.6%	94.5%	92.4%
Brazil	Vinhedo	2017	93.8%	94.7%	92.7%
Brazil	Viradouro	2000	91.9%	93.9%	89.5%
Brazil	Viradouro	2017	92.2%	94.2%	89.9%
Brazil	Virgem da Lapa	2000	81.6%	86.3%	75.4%
Brazil	Virgem da Lapa	2017	82.2%	86.8%	76.1%
Brazil	Virgínia	2000	88.1%	90.5%	85.5%
Brazil	Virgínia	2017	88.5%	90.8%	85.9%
Brazil	Virginópolis	2000	81.6%	85.9%	75.9%
Brazil	Virginópolis	2017	82.2%	86.4%	76.6%
Brazil	Virgolândia	2000	81.5%	86.1%	76.0%
Brazil	Virgolândia	2017	82.1%	86.5%	76.7%
Brazil	Virmond	2000	82.6%	86.4%	78.4%
Brazil	Virmond	2017	83.1%	86.8%	79.1%
Brazil	Visconde do Rio Branco	2000	81.0%	84.2%	77.1%

Country	Second Administrative Unit	Year	Mean	Upper	Lower
Brazil	Visconde do Rio Branco	2017	81.6%	84.7%	77.7%
Brazil	Viseu	2000	44.4%	49.9%	39.5%
Brazil	Viseu	2017	45.3%	50.9%	40.4%
Brazil	Vista Alegre	2000	79.3%	83.0%	74.3%
Brazil	Vista Alegre	2017	79.9%	83.5%	75.1%
Brazil	Vista Alegre do Alto	2000	93.1%	94.7%	91.2%
Brazil	Vista Alegre do Alto	2017	93.3%	94.9%	91.5%
Brazil	Vista Alegre do Prata	2000	81.2%	84.4%	77.8%
Brazil	Vista Alegre do Prata	2017	81.8%	85.0%	78.5%
Brazil	Vista Gaúcha	2000	77.9%	82.2%	72.8%
Brazil	Vista Gaúcha	2017	78.5%	82.8%	73.5%
Brazil	Vitor Meireles	2000	75.0%	79.5%	70.5%
Brazil	Vitor Meireles	2017	75.8%	80.1%	71.4%
Brazil	Vitoria	2000	76.7%	78.8%	74.1%
Brazil	Vitoria	2017	77.3%	79.4%	74.9%
Brazil	Vitória Brasil	2000	88.7%	92.0%	85.3%
Brazil	Vitória Brasil	2017	89.1%	92.3%	85.8%
Brazil	Vitória da Conquista	2000	81.6%	85.5%	76.9%
Brazil	Vitória da Conquista	2017	82.2%	86.0%	77.6%
Brazil	Vitória das Misses	2000	81.5%	85.7%	76.4%
Brazil	Vitória das Misses	2017	82.1%	86.2%	77.2%
Brazil	Vitória de Santo Antão	2000	67.1%	69.8%	64.5%
Brazil	Vitória de Santo Antão	2017	68.1%	70.8%	65.7%
Brazil	Vitória do Jari	2000	46.8%	52.3%	41.2%
Brazil	Vitória do Jari	2017	47.8%	53.3%	42.2%
Brazil	Vitória do Mearim	2000	60.4%	64.9%	55.5%
Brazil	Vitória do Mearim	2017	61.3%	65.8%	56.4%
Brazil	Vitória do Xingu	2000	39.6%	48.3%	30.4%
Brazil	Vitória do Xingu	2017	41.7%	50.5%	32.3%
Brazil	Vitorino	2000	81.9%	86.1%	77.9%
Brazil	Vitorino	2017	82.5%	86.6%	78.6%
Brazil	Volta Grande	2000	78.0%	81.5%	73.8%
Brazil	Volta Grande	2017	78.7%	82.1%	74.6%
Brazil	Volta Redonda	2000	81.7%	84.2%	79.1%
Brazil	Volta Redonda	2017	82.3%	84.7%	79.8%
Brazil	Votorantim	2000	94.2%	95.4%	92.9%
Brazil	Votorantim	2017	94.4%	95.6%	93.2%
Brazil	Votuporanga	2000	91.2%	93.9%	88.5%
Brazil	Votuporanga	2017	91.5%	94.1%	88.8%
Brazil	Wagner	2000	78.9%	84.3%	72.9%
Brazil	Wagner	2017	79.5%	84.8%	73.7%
Brazil	Wall Ferraz	2000	59.7%	65.1%	54.0%
Brazil	Wall Ferraz	2017	60.7%	66.0%	55.0%
Brazil	Wanderlândia	2000	74.2%	77.6%	70.4%
Brazil	Wanderlândia	2017	75.0%	78.3%	71.2%

Country	Second Administrative Unit	Year	Mean	Upper	Lower
Brazil	Wenceslau Braz	2000	89.3%	91.7%	86.8%
Brazil	Wenceslau Braz	2017	89.7%	92.0%	87.3%
Brazil	Wenceslau Guimarães	2000	76.6%	80.6%	72.4%
Brazil	Wenceslau Guimarães	2017	77.6%	81.4%	73.5%
Brazil	Witmarsum	2000	74.4%	78.7%	69.9%
Brazil	Witmarsum	2017	75.1%	79.3%	70.7%
Brazil	Xambioá	2000	66.0%	70.5%	60.8%
Brazil	Xambioá	2017	67.0%	71.4%	61.9%
Brazil	Xambrê	2000	83.2%	86.1%	79.8%
Brazil	Xambrê	2017	83.7%	86.5%	80.4%
Brazil	Xangri-lá	2000	78.8%	83.1%	73.8%
Brazil	Xangri-lá	2017	79.4%	83.6%	74.5%
Brazil	Xanxerê	2000	77.1%	81.6%	72.5%
Brazil	Xanxerê	2017	77.8%	82.2%	73.2%
Brazil	Xapuri	2000	39.8%	44.4%	35.7%
Brazil	Xapuri	2017	40.9%	45.6%	36.7%
Brazil	Xavantina	2000	77.0%	81.0%	72.0%
Brazil	Xavantina	2017	77.6%	81.5%	72.7%
Brazil	Xaxim	2000	78.2%	82.3%	73.9%
Brazil	Xaxim	2017	78.4%	82.4%	74.1%
Brazil	Xexéu	2000	62.4%	66.2%	59.2%
Brazil	Xexéu	2017	63.3%	67.0%	60.2%
Brazil	Xinguara	2000	50.0%	56.2%	42.2%
Brazil	Xinguara	2017	51.0%	57.2%	43.2%
Brazil	Xique-Xique	2000	73.2%	80.0%	64.7%
Brazil	Xique-Xique	2017	74.0%	80.6%	65.6%
Brazil	Zabelê	2000	65.1%	69.1%	60.5%
Brazil	Zabelê	2017	66.0%	69.9%	61.4%
Brazil	Zacarias	2000	92.1%	94.2%	89.4%
Brazil	Zacarias	2017	92.4%	94.4%	89.8%
Brazil	Zé Doca	2000	60.7%	66.3%	55.5%
Brazil	Zé Doca	2017	61.6%	67.1%	56.5%
Brazil	Zortéa	2000	76.3%	80.2%	71.7%
Brazil	Zortéa	2017	77.0%	80.8%	72.5%
Colombia	Abejorral	2000	73.1%	99.8%	12.9%
Colombia	Abejorral	2017	51.4%	96.9%	3.5%
Colombia	Abrego	2000	62.4%	93.5%	19.0%
Colombia	Abrego	2017	39.9%	79.4%	8.4%
Colombia	Abriaquí	2000	88.7%	95.4%	70.8%
Colombia	Abriaquí	2017	67.2%	81.1%	41.6%
Colombia	Acacías	2000	88.9%	95.6%	80.3%
Colombia	Acacías	2017	53.3%	66.7%	36.8%
Colombia	Acandí	2000	68.3%	88.9%	53.4%
Colombia	Acandí	2017	45.5%	62.5%	34.9%
Colombia	Acevedo	2000	84.9%	88.9%	80.8%
Colombia	Acevedo	2017	44.7%	49.8%	38.3%
Colombia	Achí	2000	38.7%	51.4%	27.8%
Colombia	Achí	2017	20.6%	30.5%	13.5%
Colombia	Agrado	2000	20.2%	74.6%	1.9%
Colombia	Agrado	2017	9.4%	38.7%	0.3%
Colombia	Agua de Dios	2000	93.6%	97.3%	79.6%
Colombia	Agua de Dios	2017	61.7%	86.2%	26.5%
Colombia	Aguachica	2000	84.0%	92.2%	71.4%
Colombia	Aguachica	2017	36.4%	52.6%	25.9%
Colombia	Aguada	2000	71.0%	100.0%	0.3%
Colombia	Aguada	2017	53.1%	100.0%	0.0%
Colombia	Aguadas	2000	59.0%	99.1%	3.6%
Colombia	Aguadas	2017	38.8%	94.5%	0.5%

Country	Second Administrative Unit	Year	Mean	Upper	Lower
Colombia	Aguazul	2000	65.6%	84.0%	48.6%
Colombia	Aguazul	2017	26.3%	47.2%	13.6%
Colombia	Agustín Codazzi	2000	74.4%	88.0%	62.1%
Colombia	Agustín Codazzi	2017	43.4%	59.5%	31.7%
Colombia	Aipe	2000	84.5%	98.6%	57.3%
Colombia	Aipe	2017	59.8%	88.7%	18.8%
Colombia	Albán	2000	95.8%	100.0%	71.6%
Colombia	Albán	2000	86.4%	98.9%	66.1%
Colombia	Albán	2017	86.8%	99.8%	38.4%
Colombia	Albán	2017	60.0%	86.2%	49.2%
Colombia	Albania	2000	67.4%	96.7%	21.8%
Colombia	Albania	2000	76.0%	83.7%	71.3%
Colombia	Albania	2017	55.1%	57.4%	53.1%
Colombia	Albania	2017	33.7%	76.5%	6.8%
Colombia	Alcalá	2000	97.3%	97.9%	96.4%
Colombia	Alcalá	2017	93.1%	95.7%	86.4%
Colombia	Aldana	2000	19.0%	22.7%	16.7%
Colombia	Aldana	2017	4.2%	5.5%	3.0%
Colombia	Alejandría	2000	49.1%	99.8%	1.7%
Colombia	Alejandría	2017	28.6%	97.8%	0.1%
Colombia	Algeciras	2000	79.2%	88.3%	70.3%
Colombia	Algeciras	2017	59.8%	68.3%	49.5%
Colombia	Almaguer	2000	55.7%	96.3%	7.2%
Colombia	Almaguer	2017	27.0%	82.2%	0.9%
Colombia	Almeida	2000	72.3%	97.0%	31.2%
Colombia	Almeida	2017	32.6%	77.1%	6.7%
Colombia	Alpujarra	2000	66.0%	99.5%	7.7%
Colombia	Alpujarra	2017	45.1%	94.2%	2.7%
Colombia	Altamira	2000	86.2%	98.4%	64.4%
Colombia	Altamira	2017	57.0%	80.3%	28.9%
Colombia	Alto Baudó	2000	30.3%	45.3%	19.1%
Colombia	Alto Baudó	2017	8.0%	17.2%	2.7%
Colombia	Altos del Rosario	2000	81.1%	95.2%	51.5%
Colombia	Altos del Rosario	2017	48.1%	79.1%	9.7%
Colombia	Alvarado	2000	84.0%	97.0%	56.1%
Colombia	Alvarado	2017	55.9%	82.4%	26.3%
Colombia	Amagá	2000	94.2%	98.1%	88.2%
Colombia	Amagá	2017	59.3%	75.2%	38.2%
Colombia	Amalfi	2000	72.4%	96.4%	40.4%
Colombia	Amalfi	2017	40.1%	80.0%	10.5%
Colombia	Ambalema	2000	91.0%	97.5%	74.7%
Colombia	Ambalema	2017	66.0%	82.4%	37.0%
Colombia	Anapoima	2000	45.3%	99.7%	0.7%
Colombia	Anapoima	2017	22.3%	96.9%	0.0%
Colombia	Ancuyá	2000	93.6%	95.3%	90.1%
Colombia	Ancuyá	2017	50.7%	61.7%	33.2%
Colombia	Andalucía	2000	79.4%	85.6%	69.6%
Colombia	Andalucía	2017	54.5%	62.2%	44.6%
Colombia	Andes	2000	86.1%	95.9%	67.8%
Colombia	Andes	2017	58.4%	79.9%	28.2%
Colombia	Angelópolis	2000	98.9%	99.6%	97.3%
Colombia	Angelópolis	2017	86.7%	93.7%	77.0%
Colombia	Angostura	2000	88.7%	95.9%	69.1%
Colombia	Angostura	2017	69.7%	86.7%	37.8%
Colombia	Anolaima	2000	95.5%	99.1%	81.0%
Colombia	Anolaima	2017	87.3%	97.9%	53.3%
Colombia	Anorí	2000	58.5%	96.9%	15.7%
Colombia	Anorí	2017	39.9%	84.0%	3.7%

Country	Second Administrative Unit	Year	Mean	Upper	Lower
Colombia	Anserma	2000	89.0%	93.0%	84.6%
Colombia	Anserma	2017	58.9%	68.0%	47.0%
Colombia	Ansermanuevo	2000	96.1%	99.8%	83.0%
Colombia	Ansermanuevo	2017	83.6%	97.2%	54.0%
Colombia	Anzá	2000	67.0%	100.0%	4.7%
Colombia	Anzá	2017	49.9%	99.6%	0.8%
Colombia	Anzoátegui	2000	81.7%	99.3%	44.6%
Colombia	Anzoátegui	2017	60.5%	95.9%	14.2%
Colombia	Apartadó	2000	93.0%	95.6%	88.9%
Colombia	Apartadó	2017	55.0%	62.5%	46.8%
Colombia	Apía	2000	56.9%	62.6%	52.4%
Colombia	Apía	2017	25.3%	29.0%	21.6%
Colombia	Apulo	2000	41.1%	84.6%	13.6%
Colombia	Apulo	2017	13.4%	64.7%	3.4%
Colombia	Aquitania	2000	76.2%	89.5%	63.4%
Colombia	Aquitania	2017	42.3%	56.4%	32.0%
Colombia	Aracataca	2000	89.5%	94.3%	83.4%
Colombia	Aracataca	2017	49.7%	59.4%	37.1%
Colombia	Aranzazú	2000	99.0%	99.5%	96.9%
Colombia	Aranzazú	2017	87.8%	93.1%	69.7%
Colombia	Aratoca	2000	55.9%	98.8%	14.7%
Colombia	Aratoca	2017	31.6%	88.0%	8.1%
Colombia	Arauca	2000	81.2%	88.8%	61.3%
Colombia	Arauca	2017	52.1%	62.0%	38.3%
Colombia	Arauquita	2000	85.5%	99.4%	51.6%
Colombia	Arauquita	2017	67.3%	94.9%	26.7%
Colombia	Arbeláez	2000	58.2%	95.0%	11.1%
Colombia	Arbeláez	2017	27.4%	74.4%	0.9%
Colombia	Arboleda	2000	100.0%	100.0%	100.0%
Colombia	Arboleda	2017	100.0%	100.0%	99.9%
Colombia	Arboledas	2000	66.2%	100.0%	5.1%
Colombia	Arboledas	2017	50.8%	100.0%	1.1%
Colombia	Arboletes	2000	71.1%	99.1%	26.2%
Colombia	Arboletes	2017	50.6%	91.9%	8.0%
Colombia	Arcabuco	2000	46.9%	81.2%	14.1%
Colombia	Arcabuco	2017	22.0%	55.6%	1.9%
Colombia	Argelia	2000	53.9%	99.3%	8.1%
Colombia	Argelia	2000	54.6%	99.5%	2.1%
Colombia	Argelia	2000	89.7%	99.6%	54.1%
Colombia	Argelia	2017	32.7%	92.0%	3.1%
Colombia	Argelia	2017	33.6%	94.1%	0.2%
Colombia	Argelia	2017	68.4%	95.7%	24.5%
Colombia	Ariguaní	2000	40.6%	58.1%	28.2%
Colombia	Ariguaní	2017	18.5%	29.8%	11.2%
Colombia	Arjona	2000	78.1%	97.9%	33.3%
Colombia	Arjona	2017	45.7%	82.9%	9.6%
Colombia	Armenia	2000	98.5%	98.9%	98.1%
Colombia	Armenia	2000	46.7%	75.8%	17.5%
Colombia	Armenia	2017	84.2%	87.2%	80.8%
Colombia	Armenia	2017	27.4%	56.7%	2.5%
Colombia	Armero	2000	86.7%	94.8%	75.1%
Colombia	Armero	2017	61.4%	74.1%	49.8%
Colombia	Astrea	2000	51.2%	98.8%	0.8%
Colombia	Astrea	2017	36.0%	92.3%	0.1%
Colombia	Ataco	2000	75.9%	83.9%	64.3%
Colombia	Ataco	2017	49.6%	60.5%	37.5%
Colombia	Ayapel	2000	56.8%	85.9%	29.4%
Colombia	Ayapel	2017	37.1%	62.5%	13.8%
Colombia	Bagadó	2000	51.9%	76.5%	27.5%
Colombia	Bagadó	2017	24.8%	48.2%	8.7%
Colombia	Bahía Solano	2000	58.7%	97.8%	5.1%
Colombia	Bahía Solano	2017	43.9%	93.6%	0.7%

Country	Second Administrative Unit	Year	Mean	Upper	Lower
Colombia	Bajo Baudó	2000	57.5%	81.2%	29.8%
Colombia	Bajo Baudó	2017	41.1%	65.3%	13.4%
Colombia	Balboa	2000	83.8%	100.0%	39.3%
Colombia	Balboa	2000	80.2%	96.3%	55.1%
Colombia	Balboa	2017	47.3%	70.8%	23.4%
Colombia	Balboa	2017	67.8%	99.6%	33.2%
Colombia	Baranoa	2000	91.4%	95.8%	85.6%
Colombia	Baranoa	2017	45.7%	57.1%	34.7%
Colombia	Baraya	2000	87.1%	99.7%	25.3%
Colombia	Baraya	2017	71.5%	97.4%	8.9%
Colombia	Barbacoas	2000	67.9%	85.4%	45.6%
Colombia	Barbacoas	2017	47.9%	70.7%	21.9%
Colombia	Barbosa	2000	93.6%	96.7%	88.8%
Colombia	Barbosa	2000	62.7%	85.2%	41.3%
Colombia	Barbosa	2017	59.9%	69.2%	49.7%
Colombia	Barbosa	2017	22.4%	36.5%	5.0%
Colombia	Barichara	2000	74.0%	100.0%	1.3%
Colombia	Barichara	2017	54.5%	99.9%	0.5%
Colombia	Barranca de Upía	2000	58.4%	93.2%	17.8%
Colombia	Barranca de Upía	2017	30.3%	76.3%	1.7%
Colombia	Barrancabermeja	2000	75.9%	88.7%	64.0%
Colombia	Barrancabermeja	2017	35.7%	49.7%	25.1%
Colombia	Barrancas	2000	89.7%	96.9%	67.1%
Colombia	Barrancas	2017	69.7%	85.5%	42.9%
Colombia	Barranco de Loba	2000	84.6%	97.6%	64.1%
Colombia	Barranco de Loba	2017	60.3%	86.0%	17.6%
Colombia	Barranco Minas	2000	38.9%	53.5%	26.3%
Colombia	Barranco Minas	2017	24.9%	38.3%	13.6%
Colombia	Barranquilla	2000	96.9%	97.5%	96.1%
Colombia	Barranquilla	2017	70.1%	72.4%	67.7%
Colombia	Becerril	2000	67.0%	97.1%	17.9%
Colombia	Becerril	2017	45.7%	87.6%	8.4%
Colombia	Belalcázar	2000	49.3%	90.5%	32.5%
Colombia	Belalcázar	2017	33.6%	66.0%	16.4%
Colombia	Belén	2000	67.2%	98.4%	12.2%
Colombia	Belén	2000	87.3%	100.0%	4.4%
Colombia	Belén	2017	23.0%	77.8%	1.0%
Colombia	Belén	2017	78.4%	100.0%	0.4%
Colombia	Belén de los Andaquies	2000	62.4%	93.7%	27.1%
Colombia	Belén de los Andaquies	2017	36.8%	74.0%	7.5%
Colombia	Belén de Umbría	2000	81.5%	84.0%	79.0%
Colombia	Belén de Umbría	2017	27.2%	30.6%	23.5%
Colombia	Bello	2000	97.6%	97.8%	97.3%
Colombia	Bello	2017	69.7%	71.9%	67.7%
Colombia	Belmira	2000	86.8%	100.0%	42.0%
Colombia	Belmira	2017	74.7%	100.0%	19.9%
Colombia	Beltrán	2000	86.9%	99.9%	68.5%
Colombia	Beltrán	2017	76.5%	98.1%	58.0%
Colombia	Berbeo	2000	68.5%	100.0%	0.4%
Colombia	Berbeo	2017	52.4%	99.9%	0.0%
Colombia	Betania	2000	94.0%	98.2%	76.7%
Colombia	Betania	2017	78.7%	90.0%	45.8%

Country	Second Administrative Unit	Year	Mean	Upper	Lower
Colombia	Beteitiva	2000	89.0%	100.0%	19.6%
Colombia	Beteitiva	2017	75.4%	100.0%	3.3%
Colombia	Betulia	2000	26.4%	46.7%	1.4%
Colombia	Betulia	2000	63.8%	98.5%	16.1%
Colombia	Betulia	2017	10.7%	25.8%	0.1%
Colombia	Betulia	2017	32.4%	86.0%	2.4%
Colombia	Bituima	2000	26.2%	35.4%	21.3%
Colombia	Bituima	2017	15.1%	16.6%	13.4%
Colombia	Boavita	2000	48.4%	66.4%	30.7%
Colombia	Boavita	2017	8.6%	20.5%	3.5%
Colombia	Bochalema	2000	77.2%	99.3%	36.2%
Colombia	Bochalema	2017	50.4%	89.5%	8.8%
Colombia	Bojacá	2000	86.3%	89.6%	82.2%
Colombia	Bojacá	2017	30.9%	35.6%	26.3%
Colombia	Bojayá	2000	45.7%	73.5%	16.6%
Colombia	Bojayá	2017	32.0%	58.7%	5.9%
Colombia	Bolívar	2000	85.8%	99.6%	71.3%
Colombia	Bolívar	2000	66.9%	86.3%	46.2%
Colombia	Bolívar	2000	70.2%	96.1%	43.4%
Colombia	Bolívar	2000	74.2%	79.3%	71.4%
Colombia	Bolívar	2017	52.0%	85.4%	19.1%
Colombia	Bolívar	2017	35.5%	59.9%	18.2%
Colombia	Bolívar	2017	71.4%	94.8%	44.0%
Colombia	Bolívar	2017	48.3%	55.3%	40.4%
Colombia	Bosconia	2000	30.3%	88.8%	3.1%
Colombia	Bosconia	2017	12.0%	65.7%	0.9%
Colombia	Boyacá	2000	34.3%	77.6%	10.7%
Colombia	Boyacá	2017	5.8%	36.7%	0.9%
Colombia	Briceño	2000	96.4%	98.2%	91.7%
Colombia	Briceño	2000	88.3%	100.0%	34.9%
Colombia	Briceño	2017	80.7%	89.3%	52.5%
Colombia	Briceño	2017	71.4%	99.6%	11.9%
Colombia	Bucaramanga	2000	83.2%	86.3%	80.2%
Colombia	Bucaramanga	2017	46.5%	49.4%	43.8%
Colombia	Bucarasica	2000	85.9%	99.9%	37.2%
Colombia	Bucarasica	2017	66.0%	98.2%	13.1%
Colombia	Buenaventura	2000	90.1%	94.3%	84.4%
Colombia	Buenaventura	2017	65.0%	73.0%	56.2%
Colombia	Buenavista	2000	47.7%	75.5%	8.8%
Colombia	Buenavista	2000	86.8%	89.0%	84.9%
Colombia	Buenavista	2000	99.9%	100.0%	99.6%
Colombia	Buenavista	2000	58.7%	80.5%	33.0%
Colombia	Buenavista	2017	20.7%	55.1%	0.6%
Colombia	Buenavista	2017	59.3%	62.1%	56.4%
Colombia	Buenavista	2017	98.9%	99.9%	94.3%
Colombia	Buenavista	2017	43.8%	65.9%	24.6%
Colombia	Buenos Aires	2000	67.6%	89.0%	55.4%
Colombia	Buenos Aires	2017	45.8%	57.1%	38.2%
Colombia	Buesaco	2000	98.0%	100.0%	85.3%
Colombia	Buesaco	2017	93.0%	100.0%	58.4%
Colombia	Bugalagrande	2000	87.6%	96.8%	71.6%
Colombia	Bugalagrande	2017	70.2%	83.9%	35.6%
Colombia	Buriticá	2000	79.8%	99.0%	36.1%
Colombia	Buriticá	2017	53.8%	89.7%	11.9%
Colombia	Busbanza	2000	97.8%	99.6%	90.4%
Colombia	Busbanza	2017	77.1%	92.3%	43.5%
Colombia	Cabrera	2000	76.4%	99.9%	15.2%
Colombia	Cabrera	2000	75.7%	100.0%	1.1%
Colombia	Cabrera	2017	47.2%	98.2%	1.3%
Colombia	Cabrera	2017	66.7%	100.0%	0.1%
Colombia	Cabuyaro	2000	58.6%	90.9%	27.4%
Colombia	Cabuyaro	2017	33.4%	66.2%	10.0%

Country	Second Administrative Unit	Year	Mean	Upper	Lower
Colombia	Cacahual	2000	48.4%	61.3%	34.5%
Colombia	Cacahual	2017	15.5%	26.8%	6.3%
Colombia	Cáceres	2000	56.1%	70.8%	39.4%
Colombia	Cáceres	2017	31.6%	49.3%	14.4%
Colombia	Cachipay	2000	95.3%	100.0%	71.2%
Colombia	Cachipay	2017	81.1%	99.6%	18.5%
Colombia	Cáchira	2000	73.5%	99.6%	27.2%
Colombia	Cáchira	2017	53.5%	96.2%	7.4%
Colombia	Cácota	2000	37.7%	95.0%	0.6%
Colombia	Cácota	2017	22.1%	85.5%	0.0%
Colombia	Caicedo	2000	71.5%	100.0%	0.9%
Colombia	Caicedo	2017	56.8%	100.0%	0.1%
Colombia	Caicedonia	2000	93.2%	96.6%	87.7%
Colombia	Caicedonia	2017	63.0%	72.1%	52.3%
Colombia	Caimito	2000	74.3%	92.5%	34.1%
Colombia	Caimito	2017	49.0%	80.0%	14.9%
Colombia	Cajamarca	2000	85.9%	95.4%	72.8%
Colombia	Cajamarca	2017	56.9%	80.0%	33.6%
Colombia	Cajibío	2000	88.9%	98.9%	69.9%
Colombia	Cajibío	2017	64.7%	92.0%	33.4%
Colombia	Cajicá	2000	97.4%	98.5%	95.6%
Colombia	Cajicá	2017	75.9%	85.0%	60.3%
Colombia	Calamar	2000	94.0%	99.2%	72.6%
Colombia	Calamar	2000	53.4%	71.2%	33.0%
Colombia	Calamar	2017	76.8%	93.9%	38.8%
Colombia	Calamar	2017	38.1%	55.3%	19.0%
Colombia	Calarcá	2000	97.4%	99.4%	95.0%
Colombia	Calarcá	2017	88.7%	92.2%	84.2%
Colombia	Caldas	2000	75.2%	96.0%	30.8%
Colombia	Caldas	2000	94.4%	96.0%	91.3%
Colombia	Caldas	2017	32.8%	76.3%	5.6%
Colombia	Caldas	2017	52.1%	57.7%	44.3%
Colombia	Caldonó	2000	95.8%	97.4%	90.9%
Colombia	Caldonó	2017	80.0%	86.4%	67.2%
Colombia	California	2000	68.7%	100.0%	0.2%
Colombia	California	2017	54.4%	100.0%	0.0%
Colombia	Calima	2000	67.7%	100.0%	2.8%
Colombia	Calima	2017	52.3%	99.8%	2.1%
Colombia	Caloto	2000	84.5%	96.2%	57.0%
Colombia	Caloto	2017	44.8%	69.8%	25.0%
Colombia	Campamento	2000	85.9%	97.3%	64.8%
Colombia	Campamento	2017	53.1%	77.3%	31.9%
Colombia	Campo de la Cruz	2000	97.6%	100.0%	70.4%
Colombia	Campo de la Cruz	2017	92.7%	100.0%	20.2%
Colombia	Campoalegre	2000	49.3%	83.9%	20.9%
Colombia	Campoalegre	2017	25.9%	48.8%	9.4%
Colombia	Campohermoso	2000	63.1%	99.9%	1.8%
Colombia	Campohermoso	2017	45.6%	98.8%	0.3%
Colombia	Canalete	2000	67.3%	96.1%	23.9%
Colombia	Canalete	2017	35.1%	74.5%	3.6%
Colombia	Cañasgordas	2000	95.0%	99.0%	78.7%
Colombia	Cañasgordas	2017	75.3%	88.8%	47.5%
Colombia	Candelaria	2000	95.2%	96.3%	94.0%
Colombia	Candelaria	2000	99.2%	99.9%	90.6%
Colombia	Candelaria	2017	94.6%	99.3%	67.6%
Colombia	Candelaria	2017	75.6%	79.6%	71.3%
Colombia	Cantagallo	2000	74.6%	99.7%	17.9%
Colombia	Cantagallo	2017	58.3%	97.7%	4.2%
Colombia	Caparrapí	2000	72.3%	99.5%	28.8%
Colombia	Caparrapí	2017	49.2%	92.4%	11.2%

Country	Second Administrative Unit	Year	Mean	Upper	Lower
Colombia	Capitanejo	2000	77.5%	88.2%	60.3%
Colombia	Capitanejo	2017	28.9%	47.6%	17.2%
Colombia	Cáqueza	2000	51.4%	58.5%	45.1%
Colombia	Cáqueza	2017	15.0%	19.8%	11.2%
Colombia	Caracolí	2000	87.3%	100.0%	33.2%
Colombia	Caracolí	2017	74.5%	99.8%	12.0%
Colombia	Caramanta	2000	73.6%	99.8%	17.1%
Colombia	Caramanta	2017	44.7%	96.9%	3.4%
Colombia	Carcasí	2000	38.4%	93.0%	1.3%
Colombia	Carcasí	2017	21.8%	83.3%	0.2%
Colombia	Carepa	2000	92.9%	97.0%	84.3%
Colombia	Carepa	2017	55.7%	68.9%	41.3%
Colombia	Carmen de Apicalá	2000	92.2%	99.6%	55.0%
Colombia	Carmen de Apicalá	2017	73.5%	94.9%	39.3%
Colombia	Carmen de Carupa	2000	96.7%	100.0%	68.2%
Colombia	Carmen de Carupa	2017	90.5%	99.8%	47.7%
Colombia	Carolina del Principe	2000	96.9%	100.0%	79.3%
Colombia	Carolina del Principe	2017	87.4%	99.8%	47.3%
Colombia	Cartagena de Indias	2000	94.4%	95.6%	92.2%
Colombia	Cartagena de Indias	2017	61.5%	65.6%	55.9%
Colombia	Cartagena del Chairá	2000	66.8%	77.3%	54.4%
Colombia	Cartagena del Chairá	2017	46.6%	59.2%	33.3%
Colombia	Cartago	2000	79.3%	81.3%	76.9%
Colombia	Cartago	2017	75.9%	78.1%	72.3%
Colombia	Carurú	2000	16.3%	29.8%	6.4%
Colombia	Carurú	2017	7.5%	17.4%	1.2%
Colombia	Casabianca	2000	83.9%	97.8%	61.1%
Colombia	Casabianca	2017	51.4%	81.7%	21.6%
Colombia	Castilla la Nueva	2000	72.5%	98.8%	19.6%
Colombia	Castilla la Nueva	2017	44.6%	93.4%	4.9%
Colombia	Caucasia	2000	77.7%	88.8%	64.7%
Colombia	Caucasia	2017	36.6%	52.0%	22.0%
Colombia	Cepitá	2000	51.6%	99.5%	4.4%
Colombia	Cepitá	2017	29.0%	89.5%	3.7%
Colombia	Cereté	2000	90.8%	93.2%	87.0%
Colombia	Cereté	2017	60.2%	69.0%	42.1%
Colombia	Cerinza	2000	97.3%	100.0%	72.1%
Colombia	Cerinza	2017	91.7%	100.0%	42.5%
Colombia	Cerrito	2000	57.1%	100.0%	0.2%
Colombia	Cerrito	2017	43.7%	100.0%	0.0%
Colombia	Cerro de San Antonio	2000	90.2%	100.0%	54.7%
Colombia	Cerro de San Antonio	2017	77.6%	99.3%	33.0%
Colombia	Chachagüí	2000	98.1%	100.0%	78.2%
Colombia	Chachagüí	2017	91.4%	99.5%	35.8%
Colombia	Chaguaní	2000	55.7%	90.4%	6.2%
Colombia	Chaguaní	2017	37.9%	88.4%	1.0%
Colombia	Chalán	2000	53.3%	95.0%	12.0%
Colombia	Chalán	2017	20.0%	72.6%	1.8%

Country	Second Administrative Unit	Year	Mean	Upper	Lower
Colombia	Chámeza	2000	62.1%	97.9%	6.2%
Colombia	Chámeza	2017	34.4%	84.7%	2.6%
Colombia	Chaparral	2000	76.8%	86.6%	69.6%
Colombia	Chaparral	2017	43.4%	53.4%	35.6%
Colombia	Charalá	2000	71.4%	99.7%	8.3%
Colombia	Charalá	2017	54.1%	97.6%	1.9%
Colombia	Charta	2000	89.1%	100.0%	53.7%
Colombia	Charta	2017	78.2%	99.6%	33.7%
Colombia	Chía	2000	96.7%	98.1%	94.7%
Colombia	Chía	2017	75.7%	80.9%	69.4%
Colombia	Chigorodó	2000	84.2%	93.0%	70.1%
Colombia	Chigorodó	2017	42.4%	62.9%	21.9%
Colombia	Chimá	2000	11.2%	14.8%	9.1%
Colombia	Chimá	2000	65.7%	100.0%	0.3%
Colombia	Chimá	2017	50.3%	100.0%	0.0%
Colombia	Chimá	2017	2.1%	3.6%	1.2%
Colombia	Chimichagua	2000	68.9%	91.4%	32.7%
Colombia	Chimichagua	2017	47.0%	80.8%	14.5%
Colombia	Chinácota	2000	77.9%	96.3%	40.1%
Colombia	Chinácota	2017	35.3%	78.0%	7.3%
Colombia	Chinavita	2000	79.3%	100.0%	14.3%
Colombia	Chinavita	2017	58.1%	99.8%	2.3%
Colombia	Chinchiná	2000	91.7%	99.7%	67.4%
Colombia	Chinchiná	2017	75.8%	96.7%	44.3%
Colombia	Chinú	2000	87.9%	91.1%	82.0%
Colombia	Chinú	2017	63.0%	72.5%	51.8%
Colombia	Chipaque	2000	91.9%	99.6%	66.3%
Colombia	Chipaque	2017	71.9%	93.4%	33.8%
Colombia	Chipatá	2000	93.8%	99.9%	56.1%
Colombia	Chipatá	2017	78.2%	98.6%	27.6%
Colombia	Chiquinquirá	2000	91.5%	93.1%	89.7%
Colombia	Chiquinquirá	2017	41.4%	48.4%	35.2%
Colombia	Chíquiza	2000	69.4%	100.0%	5.2%
Colombia	Chíquiza	2017	40.1%	99.5%	0.4%
Colombia	Chiriguaná	2000	70.9%	82.5%	56.2%
Colombia	Chiriguaná	2017	36.8%	53.8%	18.0%
Colombia	Chiscas	2000	25.4%	77.0%	1.1%
Colombia	Chiscas	2017	16.5%	63.2%	0.3%
Colombia	Chita	2000	47.4%	98.5%	1.3%
Colombia	Chita	2017	31.2%	93.0%	0.1%
Colombia	Chitagá	2000	39.6%	95.4%	0.5%
Colombia	Chitagá	2017	28.1%	86.9%	0.0%
Colombia	Chitaraque	2000	83.2%	99.9%	26.6%
Colombia	Chitaraque	2017	59.8%	98.9%	5.4%
Colombia	Chivatá	2000	61.7%	76.4%	48.8%
Colombia	Chivatá	2017	12.5%	21.0%	7.9%
Colombia	Chivolo	2000	43.2%	93.0%	10.9%
Colombia	Chivolo	2017	25.6%	71.7%	2.0%
Colombia	Chivor	2000	47.0%	100.0%	0.0%
Colombia	Chivor	2017	31.4%	99.9%	0.0%
Colombia	Choachí	2000	98.0%	99.9%	89.5%
Colombia	Choachí	2017	90.0%	98.5%	65.6%
Colombia	Chocontá	2000	56.2%	95.3%	7.8%
Colombia	Chocontá	2017	50.9%	93.1%	1.0%
Colombia	Cicuco	2000	89.4%	94.3%	83.8%
Colombia	Cicuco	2017	59.6%	68.1%	50.8%
Colombia	Ciénaga	2000	29.0%	99.7%	0.0%
Colombia	Ciénaga	2000	84.1%	92.0%	75.4%
Colombia	Ciénaga	2017	14.5%	95.3%	0.0%
Colombia	Ciénaga	2017	49.4%	59.0%	39.9%
Colombia	Ciénaga de Oro	2000	38.5%	76.1%	10.7%

Country	Second Administrative Unit	Year	Mean	Upper	Lower
Colombia	Ciénaga de Oro	2017	14.5%	47.5%	1.9%
Colombia	Cimitarra	2000	69.5%	89.9%	32.2%
Colombia	Cimitarra	2017	43.5%	67.8%	15.5%
Colombia	Circasia	2000	98.8%	99.6%	96.5%
Colombia	Circasia	2017	83.6%	92.6%	70.5%
Colombia	Cisneros	2000	87.4%	99.7%	43.4%
Colombia	Cisneros	2017	56.4%	93.8%	12.6%
Colombia	Cocorná	2000	81.0%	93.7%	65.4%
Colombia	Cocorná	2017	41.1%	60.1%	25.1%
Colombia	Coello	2000	88.7%	99.6%	66.6%
Colombia	Coello	2017	70.3%	95.2%	33.0%
Colombia	Cogua	2000	93.4%	98.5%	83.9%
Colombia	Cogua	2017	67.7%	78.3%	57.5%
Colombia	Colombia	2000	77.6%	99.2%	42.5%
Colombia	Colombia	2017	60.7%	93.1%	21.6%
Colombia	Colón	2000	72.3%	97.6%	29.9%
Colombia	Colón	2000	81.6%	100.0%	20.7%
Colombia	Colón	2017	29.3%	75.1%	7.2%
Colombia	Colón	2017	59.5%	99.8%	4.2%
Colombia	Colosó	2000	39.0%	94.8%	1.1%
Colombia	Colosó	2017	13.8%	68.7%	0.1%
Colombia	Cómbita	2000	84.7%	87.3%	80.9%
Colombia	Cómbita	2017	37.0%	41.5%	31.8%
Colombia	Concepción	2000	64.0%	99.5%	8.5%
Colombia	Concepción	2000	60.5%	99.5%	5.2%
Colombia	Concepción	2017	31.7%	96.0%	0.3%
Colombia	Concepción	2017	39.0%	93.3%	3.5%
Colombia	Concordia	2000	77.2%	98.6%	28.1%
Colombia	Concordia	2017	55.3%	91.7%	11.7%
Colombia	Condoto	2000	56.5%	72.2%	41.0%
Colombia	Condoto	2017	30.5%	49.5%	15.8%
Colombia	Confines	2000	75.0%	100.0%	1.2%
Colombia	Confines	2017	57.6%	100.0%	0.1%
Colombia	Consacá	2000	99.0%	99.9%	89.5%
Colombia	Consacá	2017	95.2%	99.7%	72.6%
Colombia	Contadero	2000	94.4%	96.5%	92.1%
Colombia	Contadero	2017	51.2%	60.0%	41.9%
Colombia	Contratación	2000	66.6%	100.0%	0.2%
Colombia	Contratación	2017	50.9%	100.0%	0.0%
Colombia	Convención	2000	61.5%	82.2%	38.9%
Colombia	Convención	2017	30.3%	55.0%	13.3%
Colombia	Copacabana	2000	99.5%	99.6%	99.4%
Colombia	Copacabana	2017	92.9%	94.3%	90.9%
Colombia	Coper	2000	55.5%	79.5%	22.8%
Colombia	Coper	2017	27.5%	53.9%	3.4%
Colombia	Córdoba	2000	95.4%	98.6%	83.1%
Colombia	Córdoba	2000	98.2%	100.0%	87.4%
Colombia	Córdoba	2000	61.7%	91.2%	10.5%
Colombia	Córdoba	2017	44.0%	79.9%	6.7%
Colombia	Córdoba	2017	70.1%	83.5%	39.6%
Colombia	Córdoba	2017	89.2%	99.5%	58.2%
Colombia	Corinto	2000	66.7%	77.5%	60.1%
Colombia	Corinto	2017	25.6%	37.7%	16.3%
Colombia	Coromoro	2000	70.9%	99.7%	18.6%
Colombia	Coromoro	2017	51.5%	95.8%	5.9%
Colombia	Corozal	2000	94.4%	97.0%	83.8%
Colombia	Corozal	2017	72.9%	80.3%	59.9%
Colombia	Corrales	2000	98.2%	98.8%	97.0%
Colombia	Corrales	2017	77.6%	84.5%	68.1%
Colombia	Cota	2000	88.5%	92.5%	84.5%
Colombia	Cota	2017	64.7%	69.0%	59.2%

Country	Second Administrative Unit	Year	Mean	Upper	Lower
Colombia	Covarachía	2000	60.5%	97.4%	12.3%
Colombia	Covarachía	2017	26.1%	80.8%	1.1%
Colombia	Coyaima	2000	51.8%	90.9%	22.4%
Colombia	Coyaima	2017	28.2%	69.1%	5.6%
Colombia	Cravo Norte	2000	71.8%	96.8%	28.5%
Colombia	Cravo Norte	2017	60.0%	90.7%	19.7%
Colombia	Cuaspud	2000	59.1%	64.2%	54.2%
Colombia	Cuaspud	2017	11.3%	14.0%	8.5%
Colombia	Cubará	2000	65.8%	97.9%	15.7%
Colombia	Cubará	2017	54.1%	93.5%	6.7%
Colombia	Cucaita	2000	95.5%	99.0%	89.6%
Colombia	Cucaita	2017	59.7%	84.7%	33.0%
Colombia	Cucunubá	2000	94.4%	98.0%	82.9%
Colombia	Cucunubá	2017	66.9%	79.6%	46.9%
Colombia	Cucutilla	2000	59.3%	100.0%	0.2%
Colombia	Cucutilla	2017	47.7%	100.0%	0.0%
Colombia	Cuítiva	2000	77.1%	100.0%	10.0%
Colombia	Cuítiva	2017	54.2%	99.5%	1.3%
Colombia	Cumaral	2000	63.6%	70.0%	57.6%
Colombia	Cumaral	2017	32.2%	40.6%	22.3%
Colombia	Cumaribo	2000	70.1%	79.8%	59.2%
Colombia	Cumaribo	2017	47.5%	58.0%	36.6%
Colombia	Cumbal	2000	59.7%	87.7%	25.6%
Colombia	Cumbal	2017	34.2%	70.7%	4.9%
Colombia	Cumbitara	2000	55.1%	99.4%	1.9%
Colombia	Cumbitara	2017	30.9%	92.8%	0.1%
Colombia	Cunday	2000	98.1%	100.0%	85.4%
Colombia	Cunday	2017	94.3%	99.8%	70.6%
Colombia	Curillo	2000	70.6%	96.3%	36.8%
Colombia	Curillo	2017	40.5%	77.9%	6.7%
Colombia	Curití	2000	44.1%	95.8%	1.1%
Colombia	Curití	2017	21.3%	87.4%	0.1%
Colombia	Curumaní	2000	82.0%	96.9%	41.2%
Colombia	Curumaní	2017	48.7%	80.7%	10.2%
Colombia	Dabeiba	2000	77.0%	96.3%	42.3%
Colombia	Dabeiba	2017	54.2%	81.9%	19.3%
Colombia	Dagua	2000	65.4%	96.2%	31.4%
Colombia	Dagua	2017	52.2%	87.2%	19.4%
Colombia	Dolores	2000	74.1%	100.0%	15.4%
Colombia	Dolores	2017	59.6%	99.7%	6.3%
Colombia	Don Matías	2000	95.8%	99.9%	79.5%
Colombia	Don Matías	2017	86.0%	99.2%	55.1%
Colombia	Dosquebradas	2000	97.2%	97.5%	96.9%
Colombia	Dosquebradas	2017	73.4%	76.4%	70.5%
Colombia	Duitama	2000	93.5%	96.1%	88.5%
Colombia	Duitama	2017	57.2%	63.8%	50.3%
Colombia	Durania	2000	81.7%	100.0%	19.3%
Colombia	Durania	2017	61.8%	99.8%	3.1%
Colombia	Ebéjico	2000	94.2%	100.0%	65.1%
Colombia	Ebéjico	2017	85.6%	99.9%	37.1%
Colombia	El Águila	2000	79.6%	100.0%	9.9%
Colombia	El Águila	2017	65.2%	99.9%	2.0%
Colombia	El Bagre	2000	50.7%	77.6%	27.6%
Colombia	El Bagre	2017	26.3%	49.7%	10.8%
Colombia	El Banco	2000	73.7%	81.3%	64.6%
Colombia	El Banco	2017	28.2%	37.7%	17.7%
Colombia	El Cairo	2000	72.2%	100.0%	3.0%
Colombia	El Cairo	2017	54.2%	100.0%	0.2%
Colombia	El Calvario	2000	78.5%	99.9%	22.8%
Colombia	El Calvario	2017	61.2%	98.7%	11.7%
Colombia	El Cantón del San Pablo	2000	58.6%	90.2%	25.6%

Country	Second Administrative Unit	Year	Mean	Upper	Lower
Colombia	El Cantón del San Pablo	2017	24.7%	62.2%	3.8%
Colombia	El Carmen	2000	60.9%	95.6%	21.9%
Colombia	El Carmen	2017	44.7%	83.2%	11.9%
Colombia	El Carmen de Atrato	2000	57.2%	84.1%	26.4%
Colombia	El Carmen de Atrato	2017	31.4%	60.3%	5.9%
Colombia	El Carmen de Bolívar	2000	22.7%	43.6%	9.7%
Colombia	El Carmen de Bolívar	2017	11.8%	26.2%	1.7%
Colombia	El Carmen de Chucurí	2000	69.8%	98.8%	20.2%
Colombia	El Carmen de Chucurí	2017	47.5%	90.7%	5.7%
Colombia	El Carmen de Viboral	2000	95.5%	98.1%	90.7%
Colombia	El Carmen de Viboral	2017	70.7%	81.2%	56.5%
Colombia	El Castillo	2000	64.8%	80.8%	50.7%
Colombia	El Castillo	2017	19.9%	35.6%	12.6%
Colombia	El Cerrito	2000	66.8%	78.9%	43.0%
Colombia	El Cerrito	2017	46.3%	61.9%	31.4%
Colombia	El Charco	2000	51.7%	78.8%	25.4%
Colombia	El Charco	2017	27.9%	51.8%	8.7%
Colombia	El Cocuy	2000	77.6%	99.8%	29.1%
Colombia	El Cocuy	2017	52.9%	96.3%	8.7%
Colombia	El Colegio	2000	72.2%	95.7%	38.6%
Colombia	El Colegio	2017	26.0%	60.4%	7.8%
Colombia	El Copey	2000	89.2%	95.8%	78.6%
Colombia	El Copey	2017	73.3%	84.4%	61.3%
Colombia	El Doncello	2000	57.7%	76.9%	37.0%
Colombia	El Doncello	2017	24.7%	42.9%	11.1%
Colombia	El Dorado	2000	83.2%	98.6%	51.9%
Colombia	El Dorado	2017	54.1%	86.5%	24.9%
Colombia	El Dovio	2000	72.7%	99.9%	34.7%
Colombia	El Dovio	2017	51.4%	98.0%	15.8%
Colombia	El Encanto	2000	49.8%	72.8%	26.1%
Colombia	El Encanto	2017	30.9%	51.1%	13.5%
Colombia	El Espino	2000	17.6%	81.1%	0.4%
Colombia	El Espino	2017	6.9%	63.7%	0.0%
Colombia	El Guacamayo	2000	67.3%	100.0%	0.8%
Colombia	El Guacamayo	2017	48.9%	99.8%	0.0%
Colombia	El Guamo	2000	79.5%	98.9%	47.3%
Colombia	El Guamo	2017	63.3%	92.8%	25.9%
Colombia	El Litoral del San Juan	2000	51.1%	76.4%	29.6%
Colombia	El Litoral del San Juan	2017	31.8%	54.1%	14.9%
Colombia	El Molino	2000	99.1%	100.0%	93.6%
Colombia	El Molino	2017	97.8%	100.0%	65.7%
Colombia	El Paso	2000	53.2%	99.4%	5.7%
Colombia	El Paso	2017	38.4%	95.9%	1.0%
Colombia	El Paujíl	2000	42.8%	59.4%	20.7%
Colombia	El Paujíl	2017	22.2%	38.4%	8.0%
Colombia	El Peñon	2000	49.7%	99.1%	5.8%
Colombia	El Peñon	2000	65.1%	98.6%	20.1%
Colombia	El Peñon	2017	34.6%	94.4%	0.4%
Colombia	El Peñon	2017	31.2%	83.5%	2.1%
Colombia	El Piñón	2000	84.1%	96.0%	65.4%
Colombia	El Piñón	2017	63.3%	84.0%	23.6%

Country	Second Administrative Unit	Year	Mean	Upper	Lower
Colombia	El Playón	2000	78.2%	100.0%	9.9%
Colombia	El Playón	2017	60.1%	99.8%	1.3%
Colombia	El Retorno	2000	61.3%	75.1%	48.2%
Colombia	El Retorno	2017	36.7%	52.3%	23.9%
Colombia	El Rosario	2000	43.5%	98.9%	1.1%
Colombia	El Rosario	2017	25.9%	92.9%	0.1%
Colombia	El Santuario	2000	54.8%	93.2%	14.3%
Colombia	El Santuario	2017	14.9%	48.2%	2.6%
Colombia	El Tablón de Gomez	2000	82.8%	99.1%	56.7%
Colombia	El Tablón de Gomez	2017	70.1%	95.6%	35.7%
Colombia	El Tambo	2000	97.9%	99.7%	85.2%
Colombia	El Tambo	2000	66.2%	94.7%	29.5%
Colombia	El Tambo	2017	83.0%	94.9%	49.7%
Colombia	El Tambo	2017	49.0%	83.2%	13.5%
Colombia	El Tarra	2000	54.3%	100.0%	0.2%
Colombia	El Tarra	2017	40.3%	99.1%	0.0%
Colombia	El Zulia	2000	66.0%	89.4%	40.3%
Colombia	El Zulia	2017	48.9%	79.3%	24.1%
Colombia	Elías	2000	36.6%	84.4%	4.5%
Colombia	Elías	2017	16.1%	66.6%	0.5%
Colombia	Encino	2000	73.6%	100.0%	7.2%
Colombia	Encino	2017	59.3%	99.7%	2.7%
Colombia	Enciso	2000	89.8%	96.9%	78.6%
Colombia	Enciso	2017	45.4%	67.9%	30.6%
Colombia	Entrerríos	2000	91.1%	100.0%	33.1%
Colombia	Entrerríos	2017	80.1%	100.0%	10.2%
Colombia	Envigado	2000	98.4%	98.7%	97.8%
Colombia	Envigado	2017	76.6%	80.8%	71.2%
Colombia	Espinal	2000	93.2%	95.9%	89.7%
Colombia	Espinal	2017	63.3%	70.8%	53.6%
Colombia	Facatativá	2000	92.7%	94.9%	88.6%
Colombia	Facatativá	2017	44.8%	51.2%	38.0%
Colombia	Falán	2000	94.5%	96.6%	91.3%
Colombia	Falán	2017	56.1%	64.6%	46.8%
Colombia	Filadelfia	2000	98.1%	98.8%	97.0%
Colombia	Filadelfia	2017	79.4%	86.5%	65.9%
Colombia	Filandia	2000	99.3%	99.9%	96.0%
Colombia	Filandia	2017	92.5%	98.7%	72.5%
Colombia	Firavitoba	2000	80.4%	99.6%	37.9%
Colombia	Firavitoba	2017	58.5%	94.4%	18.2%
Colombia	Flandes	2000	91.8%	99.5%	64.7%
Colombia	Flandes	2017	57.3%	91.4%	20.9%
Colombia	Florencia	2000	77.3%	91.0%	58.5%
Colombia	Florencia	2000	75.8%	79.3%	71.9%
Colombia	Florencia	2017	32.1%	49.2%	18.7%
Colombia	Florencia	2017	39.5%	43.6%	35.7%
Colombia	Floresta	2000	93.1%	94.8%	86.2%
Colombia	Floresta	2017	51.3%	57.7%	39.6%
Colombia	Florián	2000	96.4%	100.0%	70.5%
Colombia	Florián	2017	91.3%	100.0%	52.8%
Colombia	Florida	2000	61.1%	76.5%	39.3%
Colombia	Florida	2017	20.2%	44.9%	7.5%
Colombia	Floridablanca	2000	94.7%	96.1%	92.8%
Colombia	Floridablanca	2017	59.9%	64.5%	55.6%
Colombia	Fómeque	2000	94.3%	99.4%	72.2%
Colombia	Fómeque	2017	83.1%	94.7%	52.4%
Colombia	Fonseca	2000	90.9%	100.0%	62.9%
Colombia	Fonseca	2017	82.0%	99.7%	47.4%
Colombia	Fortul	2000	62.6%	76.6%	39.7%
Colombia	Fortul	2017	43.4%	65.5%	17.3%

Country	Second Administrative Unit	Year	Mean	Upper	Lower
Colombia	Fosca	2000	41.8%	60.5%	18.6%
Colombia	Fosca	2017	14.6%	33.8%	2.5%
Colombia	Francisco Pizarro	2000	77.5%	99.2%	25.4%
Colombia	Francisco Pizarro	2017	63.4%	96.0%	8.1%
Colombia	Fredonia	2000	83.3%	99.4%	33.4%
Colombia	Fredonia	2017	57.0%	94.6%	12.6%
Colombia	Fresno	2000	88.5%	93.1%	79.9%
Colombia	Fresno	2017	45.6%	55.4%	35.9%
Colombia	Frontino	2000	71.4%	93.8%	52.2%
Colombia	Frontino	2017	54.5%	82.7%	38.0%
Colombia	Fuente de Oro	2000	42.9%	56.6%	31.9%
Colombia	Fuente de Oro	2017	12.4%	18.9%	7.7%
Colombia	Fundación	2000	81.4%	91.9%	65.6%
Colombia	Fundación	2017	45.9%	63.4%	25.0%
Colombia	Funes	2000	50.1%	92.6%	16.3%
Colombia	Funes	2017	31.8%	73.8%	9.7%
Colombia	Funza	2000	92.8%	94.1%	91.0%
Colombia	Funza	2017	49.2%	53.6%	44.7%
Colombia	Fúquene	2000	77.0%	100.0%	9.4%
Colombia	Fúquene	2017	56.3%	99.5%	1.2%
Colombia	Fusagasugá	2000	85.4%	89.7%	80.3%
Colombia	Fusagasugá	2017	45.1%	50.2%	40.2%
Colombia	Gachalá	2000	69.0%	96.5%	32.6%
Colombia	Gachalá	2017	44.2%	77.2%	10.1%
Colombia	Gachancipá	2000	96.9%	99.8%	79.6%
Colombia	Gachancipá	2017	81.2%	97.4%	28.5%
Colombia	Gachantivá	2000	56.0%	98.5%	1.8%
Colombia	Gachantivá	2017	26.5%	86.1%	0.1%
Colombia	Gachetá	2000	54.2%	100.0%	0.1%
Colombia	Gachetá	2017	42.7%	100.0%	0.0%
Colombia	Galán	2000	72.7%	100.0%	4.9%
Colombia	Galán	2017	54.7%	99.9%	0.8%
Colombia	Galapa	2000	91.4%	93.2%	89.4%
Colombia	Galapa	2017	48.5%	53.7%	43.4%
Colombia	Galeras	2000	97.5%	100.0%	80.6%
Colombia	Galeras	2017	87.9%	99.8%	37.4%
Colombia	Gama	2000	58.4%	100.0%	0.7%
Colombia	Gama	2017	42.0%	99.4%	0.0%
Colombia	Gamarra	2000	79.2%	97.8%	36.8%
Colombia	Gamarra	2017	47.1%	83.4%	7.9%
Colombia	Gámbita	2000	61.4%	97.2%	13.8%
Colombia	Gámbita	2017	39.8%	86.0%	2.2%
Colombia	Gámeza	2000	79.3%	99.7%	35.7%
Colombia	Gámeza	2017	59.9%	96.0%	21.6%
Colombia	Garagoa	2000	90.9%	96.7%	76.5%
Colombia	Garagoa	2017	48.9%	72.0%	27.2%
Colombia	Garzón	2000	57.0%	62.5%	53.1%
Colombia	Garzón	2017	45.2%	47.0%	43.9%
Colombia	Génova	2000	75.4%	79.6%	72.8%
Colombia	Génova	2017	60.3%	65.1%	45.4%
Colombia	Gigante	2000	70.1%	96.4%	20.6%
Colombia	Gigante	2017	62.6%	94.1%	13.5%
Colombia	Ginebra	2000	33.7%	44.3%	9.9%
Colombia	Ginebra	2017	17.2%	33.3%	3.1%
Colombia	Giraldo	2000	94.2%	100.0%	58.0%
Colombia	Giraldo	2017	82.6%	100.0%	16.4%
Colombia	Girardot	2000	91.7%	98.2%	79.2%
Colombia	Girardot	2017	61.1%	79.9%	40.4%
Colombia	Girardota	2000	99.2%	99.7%	97.0%
Colombia	Girardota	2017	88.2%	94.2%	74.8%

Country	Second Administrative Unit	Year	Mean	Upper	Lower
Colombia	Girón	2000	80.6%	86.3%	72.9%
Colombia	Girón	2017	38.9%	45.9%	32.5%
Colombia	Gómez Plata	2000	92.4%	99.5%	53.5%
Colombia	Gómez Plata	2017	81.9%	96.2%	38.5%
Colombia	González	2000	52.6%	65.6%	42.8%
Colombia	González	2017	7.3%	10.6%	5.1%
Colombia	Gramalote	2000	84.4%	96.4%	66.1%
Colombia	Gramalote	2017	42.1%	69.7%	17.6%
Colombia	Granada	2000	67.4%	74.7%	60.7%
Colombia	Granada	2000	43.1%	84.5%	25.8%
Colombia	Granada	2017	22.1%	27.9%	14.8%
Colombia	Granada	2017	11.3%	28.9%	6.3%
Colombia	Guaca	2000	81.3%	100.0%	12.8%
Colombia	Guaca	2017	65.8%	99.5%	2.5%
Colombia	Guacamayas	2000	40.9%	58.1%	29.7%
Colombia	Guacamayas	2017	8.2%	12.9%	5.9%
Colombia	Guacarí	2000	80.4%	86.3%	76.2%
Colombia	Guacarí	2017	52.9%	58.2%	48.0%
Colombia	Guachetá	2000	77.1%	99.8%	10.1%
Colombia	Guachetá	2017	55.9%	98.7%	2.5%
Colombia	Guachucal	2000	20.7%	61.1%	1.4%
Colombia	Guachucal	2017	10.5%	50.7%	0.1%
Colombia	Guadalajara de Buga	2000	92.9%	98.8%	87.7%
Colombia	Guadalajara de Buga	2017	80.6%	90.0%	68.6%
Colombia	Guadalupe	2000	80.0%	98.9%	36.8%
Colombia	Guadalupe	2000	80.7%	88.7%	74.1%
Colombia	Guadalupe	2000	87.8%	98.3%	60.7%
Colombia	Guadalupe	2017	50.1%	87.0%	11.7%
Colombia	Guadalupe	2017	59.4%	84.5%	27.4%
Colombia	Guadalupe	2017	49.3%	58.1%	39.1%
Colombia	Guaduas	2000	88.6%	98.0%	64.3%
Colombia	Guaduas	2017	71.3%	90.4%	44.4%
Colombia	Guaitarilla	2000	91.0%	98.5%	69.6%
Colombia	Guaitarilla	2017	64.0%	82.8%	46.8%
Colombia	Gualmatán	2000	94.2%	97.7%	88.7%
Colombia	Gualmatán	2017	57.5%	69.3%	48.5%
Colombia	Guamal	2000	70.8%	98.7%	31.7%
Colombia	Guamal	2000	79.5%	99.6%	31.4%
Colombia	Guamal	2017	54.2%	96.0%	6.9%
Colombia	Guamal	2017	44.1%	91.2%	11.8%
Colombia	Guamo	2000	84.9%	87.4%	81.6%
Colombia	Guamo	2017	55.5%	61.5%	48.9%
Colombia	Guapí	2000	45.2%	68.6%	23.8%
Colombia	Guapí	2017	30.8%	57.1%	12.9%
Colombia	Guapotá	2000	80.0%	100.0%	10.6%
Colombia	Guapotá	2017	56.0%	99.3%	1.2%
Colombia	Guarandá	2000	23.1%	34.1%	13.0%
Colombia	Guarandá	2017	9.4%	17.8%	2.1%
Colombia	Guarne	2000	99.8%	99.9%	99.7%
Colombia	Guarne	2017	97.0%	98.2%	94.9%
Colombia	Guasca	2000	58.4%	92.7%	16.0%
Colombia	Guasca	2017	38.0%	84.9%	2.9%
Colombia	Guatapé	2000	14.8%	84.6%	0.0%
Colombia	Guatapé	2017	6.3%	60.1%	0.0%
Colombia	Guataquí	2000	48.1%	99.7%	0.3%
Colombia	Guataquí	2017	28.3%	96.7%	0.0%
Colombia	Guatavita	2000	77.3%	100.0%	9.5%
Colombia	Guatavita	2017	61.6%	99.8%	1.3%
Colombia	Guateque	2000	69.5%	100.0%	0.4%
Colombia	Guateque	2017	51.8%	100.0%	0.0%

Country	Second Administrative Unit	Year	Mean	Upper	Lower
Colombia	Guática	2000	82.1%	99.8%	23.4%
Colombia	Guática	2017	51.7%	95.2%	3.0%
Colombia	Guavatá	2000	62.6%	81.6%	55.2%
Colombia	Guavatá	2017	21.2%	26.1%	17.7%
Colombia	Guayabal de Síquima	2000	98.6%	99.4%	96.8%
Colombia	Guayabal de Síquima	2017	91.8%	96.6%	83.8%
Colombia	Guayabetal	2000	37.5%	99.4%	0.2%
Colombia	Guayabetal	2017	24.7%	93.7%	0.0%
Colombia	Guayatá	2000	61.4%	100.0%	0.0%
Colombia	Guayatá	2017	46.0%	100.0%	0.0%
Colombia	Güepsa	2000	90.6%	100.0%	31.7%
Colombia	Güepsa	2017	72.7%	99.8%	5.1%
Colombia	Guicán	2000	63.6%	99.3%	16.6%
Colombia	Guicán	2017	46.9%	94.7%	4.4%
Colombia	Gutiérrez	2000	83.4%	99.8%	46.0%
Colombia	Gutiérrez	2017	68.7%	97.3%	19.3%
Colombia	Hacarí	2000	33.9%	99.1%	0.0%
Colombia	Hacarí	2017	19.7%	94.4%	0.0%
Colombia	Hatillo de Loba	2000	99.1%	99.8%	96.3%
Colombia	Hatillo de Loba	2017	91.7%	98.3%	73.0%
Colombia	Hato	2000	65.8%	100.0%	0.9%
Colombia	Hato	2017	46.9%	99.9%	0.1%
Colombia	Hato Corozal	2000	55.9%	72.4%	41.0%
Colombia	Hato Corozal	2017	26.6%	44.8%	13.4%
Colombia	Heliconia	2000	97.9%	100.0%	86.8%
Colombia	Heliconia	2017	93.7%	100.0%	71.3%
Colombia	Herrán	2000	69.9%	99.8%	5.0%
Colombia	Herrán	2017	48.0%	97.9%	0.8%
Colombia	Herveo	2000	69.2%	98.2%	21.3%
Colombia	Herveo	2017	50.8%	89.1%	11.1%
Colombia	Hispania	2000	96.2%	98.1%	91.3%
Colombia	Hispania	2017	61.9%	73.7%	48.8%
Colombia	Hobo	2000	65.4%	89.9%	12.8%
Colombia	Hobo	2017	54.9%	90.3%	2.7%
Colombia	Honda	2000	77.6%	92.1%	40.9%
Colombia	Honda	2017	60.1%	79.6%	25.4%
Colombia	Ibagué	2000	92.4%	94.7%	89.7%
Colombia	Ibagué	2017	53.5%	56.6%	50.1%
Colombia	Icononzo	2000	67.2%	100.0%	7.1%
Colombia	Icononzo	2017	45.6%	99.5%	1.2%
Colombia	Iles	2000	70.7%	97.0%	43.0%
Colombia	Iles	2017	23.7%	65.4%	11.9%
Colombia	Imués	2000	64.7%	94.0%	38.5%
Colombia	Imués	2017	21.2%	48.2%	7.2%
Colombia	Inzá	2000	82.0%	96.7%	48.4%
Colombia	Inzá	2017	52.9%	80.1%	22.6%
Colombia	Ipiales	2000	79.8%	87.3%	71.8%
Colombia	Ipiales	2017	30.6%	42.1%	22.5%
Colombia	Iquira	2000	91.2%	99.5%	57.3%
Colombia	Iquira	2017	73.0%	95.2%	30.1%
Colombia	Isnos	2000	94.3%	95.3%	93.0%
Colombia	Isnos	2017	54.8%	60.0%	49.3%
Colombia	Istmina	2000	53.3%	67.5%	37.3%
Colombia	Istmina	2017	23.3%	39.7%	7.5%
Colombia	Itagüí	2000	98.0%	98.3%	97.5%
Colombia	Itagüí	2017	73.2%	76.7%	67.4%
Colombia	Ituango	2000	70.9%	96.3%	44.7%
Colombia	Ituango	2017	49.7%	78.6%	19.2%

Country	Second Administrative Unit	Year	Mean	Upper	Lower
Colombia	Izá	2000	76.0%	100.0%	10.6%
Colombia	Izá	2017	53.4%	99.4%	0.9%
Colombia	Jambaló	2000	87.5%	91.3%	81.6%
Colombia	Jambaló	2017	52.4%	61.1%	41.0%
Colombia	Jamundí	2000	94.6%	97.5%	88.8%
Colombia	Jamundí	2017	74.2%	81.0%	63.7%
Colombia	Jardín	2000	69.3%	98.6%	23.7%
Colombia	Jardín	2017	38.4%	88.8%	9.2%
Colombia	Jenesano	2000	60.4%	94.4%	27.3%
Colombia	Jenesano	2017	19.3%	60.4%	4.7%
Colombia	Jericó	2000	75.6%	83.5%	63.9%
Colombia	Jericó	2000	39.7%	100.0%	0.0%
Colombia	Jericó	2017	25.1%	99.6%	0.0%
Colombia	Jericó	2017	31.2%	39.3%	22.2%
Colombia	Jerusalén	2000	37.0%	96.2%	0.8%
Colombia	Jerusalén	2017	19.3%	80.6%	0.1%
Colombia	Jesús María	2000	50.3%	84.2%	15.1%
Colombia	Jesús María	2017	30.3%	58.6%	7.9%
Colombia	Jordán	2000	74.0%	99.7%	16.2%
Colombia	Jordán	2017	49.8%	94.1%	5.3%
Colombia	Juan de Acosta	2000	28.2%	98.0%	0.1%
Colombia	Juan de Acosta	2017	13.7%	86.4%	0.0%
Colombia	Junín	2000	60.0%	100.0%	1.7%
Colombia	Junín	2017	47.2%	99.8%	0.2%
Colombia	Juradó	2000	74.9%	100.0%	13.9%
Colombia	Juradó	2017	69.2%	100.0%	7.2%
Colombia	La Argentina	2000	84.2%	99.5%	61.7%
Colombia	La Argentina	2017	69.8%	92.7%	49.7%
Colombia	La Belleza	2000	86.8%	99.5%	66.3%
Colombia	La Belleza	2017	68.9%	93.6%	36.5%
Colombia	La Calera	2000	89.4%	91.2%	86.9%
Colombia	La Calera	2017	38.1%	44.1%	31.9%
Colombia	La Capilla	2000	86.6%	100.0%	27.6%
Colombia	La Capilla	2017	68.8%	99.5%	18.1%
Colombia	La Ceja	2000	90.4%	98.1%	71.4%
Colombia	La Ceja	2017	54.0%	83.9%	27.0%
Colombia	La Celia	2000	58.4%	99.6%	2.4%
Colombia	La Celia	2017	33.6%	94.7%	0.2%
Colombia	La Chorrera	2000	49.3%	65.2%	34.0%
Colombia	La Chorrera	2017	31.2%	47.4%	18.3%
Colombia	La Cruz	2000	63.5%	97.7%	21.5%
Colombia	La Cruz	2017	31.7%	78.9%	9.2%
Colombia	La Cumbre	2000	20.0%	71.4%	0.3%
Colombia	La Cumbre	2017	8.6%	54.3%	0.0%
Colombia	La Dorada	2000	92.0%	94.0%	87.3%
Colombia	La Dorada	2017	70.8%	78.6%	58.3%
Colombia	La Esperanza	2000	75.9%	98.9%	17.3%
Colombia	La Esperanza	2017	52.7%	92.2%	6.1%
Colombia	La Estrella	2000	98.5%	98.9%	98.0%
Colombia	La Estrella	2017	80.1%	85.2%	72.9%
Colombia	La Florida	2000	99.5%	100.0%	97.6%
Colombia	La Florida	2017	97.0%	100.0%	79.9%
Colombia	La Gloria	2000	56.1%	99.6%	1.5%
Colombia	La Gloria	2017	40.0%	94.9%	0.2%
Colombia	La Guadalupe	2000	42.3%	86.0%	5.5%
Colombia	La Guadalupe	2017	25.7%	65.7%	0.6%
Colombia	La Jagua de Ibirico	2000	74.6%	97.8%	41.5%
Colombia	La Jagua de Ibirico	2017	46.6%	81.5%	17.6%

Country	Second Administrative Unit	Year	Mean	Upper	Lower
Colombia	La Llanada	2000	85.0%	99.8%	45.8%
Colombia	La Llanada	2017	67.1%	97.8%	15.5%
Colombia	La Macarena	2000	59.7%	74.9%	44.6%
Colombia	La Macarena	2017	37.0%	50.9%	25.0%
Colombia	La Merced	2000	85.8%	93.3%	76.5%
Colombia	La Merced	2017	35.0%	46.3%	25.8%
Colombia	La Mesa	2000	85.5%	95.4%	73.3%
Colombia	La Mesa	2017	40.6%	54.9%	26.9%
Colombia	La Montañita	2000	73.8%	82.8%	66.4%
Colombia	La Montañita	2017	58.2%	64.0%	53.6%
Colombia	La Palma	2000	49.9%	99.9%	0.6%
Colombia	La Palma	2017	30.3%	98.5%	0.0%
Colombia	La Paz	2000	75.4%	88.9%	57.3%
Colombia	La Paz	2000	70.2%	97.1%	19.5%
Colombia	La Paz	2017	49.1%	64.9%	31.1%
Colombia	La Paz	2017	45.4%	87.0%	3.3%
Colombia	La Pedrera	2000	49.1%	70.5%	26.1%
Colombia	La Pedrera	2017	31.7%	52.5%	15.2%
Colombia	La Peña	2000	66.5%	99.5%	9.8%
Colombia	La Peña	2017	41.5%	93.8%	2.2%
Colombia	La Plata	2000	87.4%	94.1%	70.8%
Colombia	La Plata	2017	73.0%	83.4%	56.9%
Colombia	La Playa de Belén	2000	38.2%	90.3%	4.6%
Colombia	La Playa de Belén	2017	17.8%	70.8%	0.5%
Colombia	La Primavera	2000	65.0%	76.5%	54.2%
Colombia	La Primavera	2017	41.6%	52.4%	31.1%
Colombia	La Salina	2000	50.3%	99.9%	0.0%
Colombia	La Salina	2017	32.8%	99.1%	0.0%
Colombia	La Sierra	2000	68.0%	98.0%	29.7%
Colombia	La Sierra	2017	40.1%	80.8%	7.6%
Colombia	La Tebaida	2000	89.3%	92.7%	85.8%
Colombia	La Tebaida	2017	82.8%	86.3%	73.5%
Colombia	La Tola	2000	67.4%	89.0%	37.6%
Colombia	La Tola	2017	40.5%	61.9%	10.0%
Colombia	La Unión de Sucre	2000	33.7%	39.7%	30.9%
Colombia	La Unión de Sucre	2000	76.8%	97.3%	55.0%
Colombia	La Unión de Sucre	2000	95.4%	99.3%	82.4%
Colombia	La Unión de Sucre	2000	72.9%	98.5%	16.3%
Colombia	La Unión de Sucre	2017	53.2%	79.1%	31.0%
Colombia	La Unión de Sucre	2017	47.3%	93.3%	4.0%
Colombia	La Unión de Sucre	2017	15.4%	17.1%	11.1%
Colombia	La Unión de Sucre	2017	77.6%	89.5%	59.6%
Colombia	La Uribe	2000	55.9%	80.4%	26.4%
Colombia	La Uribe	2017	37.0%	61.6%	14.2%
Colombia	La Uvita	2000	50.9%	89.9%	18.9%
Colombia	La Uvita	2017	17.7%	55.6%	1.8%
Colombia	La Vega	2000	78.0%	99.7%	15.2%
Colombia	La Vega	2000	75.1%	90.1%	55.3%
Colombia	La Vega	2017	38.2%	54.3%	23.6%
Colombia	La Vega	2017	53.3%	96.9%	1.9%
Colombia	La Victoria	2000	77.0%	97.3%	49.8%
Colombia	La Victoria	2000	24.5%	55.6%	3.0%

Piped Water Proportion by Second Administrative Unit *(continued)*

Country	Second Administrative Unit	Year	Mean	Upper	Lower
Colombia	La Victoria	2017	50.6%	71.9%	31.4%
Colombia	La Victoria	2017	8.3%	37.4%	0.4%
Colombia	La Virginia	2000	99.7%	99.9%	99.0%
Colombia	La Virginia	2017	96.7%	99.1%	91.9%
Colombia	Labateca	2000	61.3%	77.3%	55.3%
Colombia	Labateca	2017	24.5%	30.9%	20.7%
Colombia	Labranzagrande	2000	15.7%	54.6%	0.2%
Colombia	Labranzagrande	2017	9.2%	48.6%	0.0%
Colombia	Landázuri	2000	74.8%	83.4%	60.3%
Colombia	Landázuri	2017	54.8%	65.1%	40.9%
Colombia	Lebrija	2000	86.5%	90.0%	82.6%
Colombia	Lebrija	2017	76.5%	79.1%	72.9%
Colombia	Leiva	2000	60.5%	95.8%	15.8%
Colombia	Leiva	2017	34.3%	84.7%	4.1%
Colombia	Lejanías	2000	75.2%	89.5%	50.8%
Colombia	Lejanías	2017	35.0%	55.4%	16.2%
Colombia	Lenguazaque	2000	85.8%	97.3%	52.7%
Colombia	Lenguazaque	2017	63.6%	90.9%	16.3%
Colombia	Lérida	2000	83.9%	98.5%	46.1%
Colombia	Lérida	2017	45.2%	82.4%	15.2%
Colombia	Leticia	2000	38.6%	46.9%	29.6%
Colombia	Leticia	2017	13.7%	22.7%	5.3%
Colombia	Líbano	2000	86.2%	93.8%	73.2%
Colombia	Líbano	2017	44.6%	61.5%	30.6%
Colombia	Liborina	2000	70.9%	100.0%	1.2%
Colombia	Liborina	2017	55.7%	100.0%	0.1%
Colombia	Linares	2000	96.5%	98.1%	88.7%
Colombia	Linares	2017	69.4%	83.6%	40.4%
Colombia	Lloró	2000	76.0%	98.6%	32.3%
Colombia	Lloró	2017	57.5%	93.1%	12.3%
Colombia	López de Micay	2000	47.1%	82.3%	16.7%
Colombia	López de Micay	2017	33.2%	68.8%	5.5%
Colombia	Los Andes	2000	70.9%	95.7%	34.6%
Colombia	Los Andes	2017	42.0%	78.7%	14.4%
Colombia	Los Córdobas	2000	67.9%	99.0%	11.5%
Colombia	Los Córdobas	2017	46.3%	92.4%	1.3%
Colombia	Los Palmitos	2000	94.2%	99.0%	75.4%
Colombia	Los Palmitos	2017	69.7%	87.0%	41.0%
Colombia	Los Patios	2000	82.8%	99.5%	31.8%
Colombia	Los Patios	2017	58.2%	96.0%	6.1%
Colombia	Los Santos	2000	98.0%	100.0%	87.5%
Colombia	Los Santos	2017	93.4%	99.4%	73.3%
Colombia	Lourdes	2000	88.0%	99.5%	41.4%
Colombia	Lourdes	2017	61.9%	93.4%	11.0%
Colombia	Luruaco	2000	43.8%	82.7%	21.3%
Colombia	Luruaco	2017	16.8%	47.2%	9.4%
Colombia	Macanal	2000	73.5%	94.7%	44.8%
Colombia	Macanal	2017	30.9%	69.3%	7.7%
Colombia	Macaravita	2000	2.1%	5.9%	0.6%
Colombia	Macaravita	2017	0.2%	0.4%	0.0%
Colombia	Maceo	2000	83.4%	100.0%	17.8%
Colombia	Maceo	2017	68.9%	99.7%	4.9%
Colombia	Machetá	2000	63.8%	100.0%	0.5%
Colombia	Machetá	2017	50.4%	100.0%	0.0%
Colombia	Madrid	2000	87.2%	90.2%	83.5%
Colombia	Madrid	2017	37.1%	40.5%	33.2%
Colombia	Magüí	2000	51.1%	72.9%	23.9%
Colombia	Magüí	2017	32.9%	54.5%	13.2%
Colombia	Mahates	2000	55.6%	85.7%	23.7%
Colombia	Mahates	2017	21.4%	51.8%	5.8%

Country	Second Administrative Unit	Year	Mean	Upper	Lower
Colombia	Maicao	2000	59.3%	66.3%	50.0%
Colombia	Maicao	2017	24.8%	30.8%	18.9%
Colombia	Majagual	2000	62.8%	83.2%	34.7%
Colombia	Majagual	2017	33.7%	55.9%	9.3%
Colombia	Málaga	2000	86.0%	96.8%	63.6%
Colombia	Málaga	2017	44.3%	61.0%	28.1%
Colombia	Malambo	2000	95.0%	96.2%	91.9%
Colombia	Malambo	2017	58.9%	63.8%	53.5%
Colombia	Mallama	2000	71.6%	100.0%	12.9%
Colombia	Mallama	2017	57.4%	99.8%	3.3%
Colombia	Manatí	2000	99.7%	100.0%	98.1%
Colombia	Manatí	2017	97.3%	99.8%	82.3%
Colombia	Manaure	2000	71.3%	86.8%	48.0%
Colombia	Manaure	2000	64.1%	69.3%	59.9%
Colombia	Manaure	2017	71.8%	85.6%	35.6%
Colombia	Manaure	2017	41.7%	46.3%	37.5%
Colombia	Maní	2000	70.5%	92.6%	36.8%
Colombia	Maní	2017	49.6%	80.6%	16.5%
Colombia	Manizales	2000	98.6%	98.9%	98.1%
Colombia	Manizales	2017	79.6%	82.9%	75.5%
Colombia	Manta	2000	64.7%	100.0%	0.0%
Colombia	Manta	2017	48.4%	100.0%	0.0%
Colombia	Manzanares	2000	86.3%	99.9%	23.5%
Colombia	Manzanares	2017	61.6%	98.5%	4.3%
Colombia	Mapiripán	2000	60.7%	75.3%	44.2%
Colombia	Mapiripán	2017	40.3%	55.9%	23.8%
Colombia	Margarita	2000	75.6%	100.0%	16.6%
Colombia	Margarita	2017	59.0%	99.1%	11.2%
Colombia	María la Baja	2000	52.2%	98.9%	7.7%
Colombia	María la Baja	2017	27.8%	89.3%	1.3%
Colombia	Marinilla	2000	84.4%	95.3%	69.5%
Colombia	Marinilla	2017	39.9%	53.1%	30.6%
Colombia	Maripí	2000	60.5%	68.8%	52.3%
Colombia	Maripí	2017	13.4%	17.8%	10.1%
Colombia	Marmato	2000	95.0%	99.2%	84.1%
Colombia	Marmato	2017	68.8%	86.9%	46.1%
Colombia	Marquetalia	2000	83.4%	100.0%	21.6%
Colombia	Marquetalia	2017	57.5%	99.5%	2.3%
Colombia	Marsella	2000	54.2%	68.9%	47.4%
Colombia	Marsella	2017	38.0%	47.2%	30.8%
Colombia	Marulanda	2000	92.2%	100.0%	42.9%
Colombia	Marulanda	2017	82.7%	99.8%	23.3%
Colombia	Matanza	2000	69.7%	93.7%	32.9%
Colombia	Matanza	2017	52.3%	86.4%	24.8%
Colombia	Medellín	2000	97.4%	97.6%	97.2%
Colombia	Medellín	2017	66.1%	68.4%	64.1%
Colombia	Medina	2000	70.8%	90.5%	49.1%
Colombia	Medina	2017	42.2%	63.2%	22.6%
Colombia	Melgar	2000	75.8%	99.9%	10.8%
Colombia	Melgar	2017	52.0%	98.2%	1.2%
Colombia	Mercaderes	2000	53.5%	76.4%	37.4%
Colombia	Mercaderes	2017	23.4%	43.0%	13.1%
Colombia	Mesetas	2000	48.7%	58.7%	39.3%
Colombia	Mesetas	2017	31.9%	41.9%	21.3%
Colombia	Milán	2000	36.6%	46.6%	29.3%
Colombia	Milán	2017	18.6%	27.5%	12.6%
Colombia	Miraflores	2000	67.4%	100.0%	1.8%
Colombia	Miraflores	2000	49.5%	68.7%	29.3%
Colombia	Miraflores	2017	49.2%	99.9%	0.2%
Colombia	Miraflores	2017	34.0%	52.5%	17.0%
Colombia	Miranda	2000	86.5%	95.8%	75.4%
Colombia	Miranda	2017	44.8%	63.3%	31.6%

Country	Second Administrative Unit	Year	Mean	Upper	Lower
Colombia	Mirití-Paraná	2000	48.5%	75.0%	21.2%
Colombia	Mirití-Paraná	2017	32.9%	58.0%	9.8%
Colombia	Mistrato	2000	64.3%	99.0%	26.2%
Colombia	Mistrato	2017	47.5%	92.7%	17.3%
Colombia	Mitú	2000	25.7%	36.1%	16.1%
Colombia	Mitú	2017	13.1%	24.1%	5.2%
Colombia	Mogotes	2000	71.6%	84.4%	52.0%
Colombia	Mogotes	2017	30.8%	47.0%	14.2%
Colombia	Molagavita	2000	65.5%	98.8%	16.0%
Colombia	Molagavita	2017	32.6%	85.9%	2.1%
Colombia	Momil	2000	71.9%	96.5%	25.5%
Colombia	Momil	2017	40.4%	81.4%	3.1%
Colombia	Mompós	2000	70.0%	86.0%	53.1%
Colombia	Mompós	2017	58.0%	72.1%	42.7%
Colombia	Mongua	2000	39.4%	82.0%	7.4%
Colombia	Mongua	2017	16.2%	58.3%	1.3%
Colombia	Monguí	2000	61.3%	73.3%	52.0%
Colombia	Monguí	2017	23.5%	27.1%	20.6%
Colombia	Moniquirá	2000	61.7%	88.0%	30.0%
Colombia	Moniquirá	2017	25.4%	48.8%	6.6%
Colombia	Moñitos	2000	68.8%	97.8%	12.9%
Colombia	Moñitos	2017	43.0%	87.6%	2.0%
Colombia	Montebello	2000	80.7%	99.8%	23.9%
Colombia	Montebello	2017	52.2%	96.5%	4.5%
Colombia	Montecristo	2000	59.5%	90.8%	24.6%
Colombia	Montecristo	2017	38.7%	73.2%	9.3%
Colombia	Montelíbano	2000	75.5%	93.0%	38.5%
Colombia	Montelíbano	2017	54.0%	80.3%	20.5%
Colombia	Montenegro	2000	98.5%	98.8%	98.1%
Colombia	Montenegro	2017	89.2%	91.7%	85.4%
Colombia	Montería	2000	70.6%	85.5%	51.8%
Colombia	Montería	2017	40.8%	59.0%	17.4%
Colombia	Monterrey	2000	73.7%	94.4%	43.0%
Colombia	Monterrey	2017	30.4%	62.3%	8.2%
Colombia	Morales	2000	79.1%	91.5%	62.0%
Colombia	Morales	2000	66.6%	92.2%	35.4%
Colombia	Morales	2017	38.3%	58.0%	19.2%
Colombia	Morales	2017	47.0%	75.0%	17.9%
Colombia	Morelia	2000	29.3%	85.3%	0.1%
Colombia	Morelia	2017	16.5%	53.5%	0.0%
Colombia	Morroa	2000	92.0%	97.9%	82.2%
Colombia	Morroa	2017	68.3%	76.5%	62.2%
Colombia	Mosquera	2000	90.6%	92.0%	89.1%
Colombia	Mosquera	2000	46.8%	99.4%	3.9%
Colombia	Mosquera	2017	43.2%	46.8%	39.4%
Colombia	Mosquera	2017	31.9%	95.4%	0.7%
Colombia	Motavita	2000	66.9%	99.8%	7.4%
Colombia	Motavita	2017	35.2%	95.4%	0.7%
Colombia	Murillo	2000	68.2%	100.0%	2.4%
Colombia	Murillo	2017	54.8%	100.0%	0.2%
Colombia	Murindó	2000	62.5%	100.0%	1.2%
Colombia	Murindó	2017	53.7%	100.0%	0.1%
Colombia	Mutatá	2000	45.0%	97.1%	2.1%
Colombia	Mutatá	2017	32.6%	90.4%	0.7%
Colombia	Mutiscua	2000	61.9%	100.0%	0.0%
Colombia	Mutiscua	2017	49.5%	100.0%	0.0%
Colombia	Muzo	2000	70.7%	77.0%	64.1%
Colombia	Muzo	2017	20.0%	25.7%	14.8%
Colombia	Nariño	2000	68.7%	98.6%	20.5%
Colombia	Nariño	2000	66.4%	99.9%	8.6%
Colombia	Nariño	2017	35.5%	91.7%	2.1%
Colombia	Nariño	2017	45.9%	98.0%	1.3%

Country	Second Administrative Unit	Year	Mean	Upper	Lower
Colombia	Nátaga	2000	95.4%	100.0%	53.1%
Colombia	Nátaga	2017	83.9%	99.1%	24.8%
Colombia	Natagaima	2000	71.6%	99.6%	10.4%
Colombia	Natagaima	2017	53.5%	96.0%	3.8%
Colombia	Nechí	2000	42.8%	73.6%	16.9%
Colombia	Nechí	2017	21.4%	54.9%	3.0%
Colombia	Necoclí	2000	75.6%	98.8%	42.0%
Colombia	Necoclí	2017	55.5%	93.8%	18.4%
Colombia	Neira	2000	97.0%	98.2%	95.4%
Colombia	Neira	2017	80.4%	84.7%	73.9%
Colombia	Neiva	2000	95.8%	97.5%	92.2%
Colombia	Neiva	2017	70.3%	74.5%	65.3%
Colombia	Nemocón	2000	99.7%	99.9%	99.0%
Colombia	Nemocón	2017	95.0%	98.5%	85.2%
Colombia	Nilo	2000	75.8%	99.1%	21.6%
Colombia	Nilo	2017	44.6%	90.8%	4.5%
Colombia	Nimaima	2000	81.6%	95.4%	59.1%
Colombia	Nimaima	2017	40.0%	62.6%	19.2%
Colombia	Nobsa	2000	94.8%	95.6%	93.9%
Colombia	Nobsa	2017	48.7%	54.0%	43.4%
Colombia	Nocaima	2000	78.0%	98.0%	42.2%
Colombia	Nocaima	2017	38.7%	73.4%	7.0%
Colombia	Novita	2000	48.8%	68.4%	36.3%
Colombia	Novita	2017	24.5%	42.5%	15.4%
Colombia	Nuevo Colón	2000	96.0%	96.9%	94.8%
Colombia	Nuevo Colón	2017	59.9%	66.4%	52.7%
Colombia	Nunchía	2000	71.4%	94.0%	42.5%
Colombia	Nunchía	2017	41.3%	67.2%	16.0%
Colombia	Nuquí	2000	40.8%	86.8%	5.1%
Colombia	Nuquí	2017	23.5%	68.1%	0.8%
Colombia	Obando	2000	64.6%	71.9%	57.5%
Colombia	Obando	2017	35.1%	44.3%	26.8%
Colombia	Ocamonte	2000	75.0%	100.0%	0.6%
Colombia	Ocamonte	2017	58.0%	100.0%	0.0%
Colombia	Ocaña	2000	89.6%	91.2%	87.7%
Colombia	Ocaña	2017	64.5%	68.7%	60.3%
Colombia	Oiba	2000	86.5%	97.7%	45.9%
Colombia	Oiba	2017	52.7%	81.6%	16.4%
Colombia	Oicatá	2000	85.2%	87.2%	82.7%
Colombia	Oicatá	2017	31.5%	34.8%	28.0%
Colombia	Olaya	2000	78.0%	100.0%	2.6%
Colombia	Olaya	2017	59.0%	99.9%	0.2%
Colombia	Olaya Herrera	2000	59.2%	88.9%	19.2%
Colombia	Olaya Herrera	2017	37.4%	72.7%	4.5%
Colombia	Onzaga	2000	57.7%	99.2%	2.3%
Colombia	Onzaga	2017	39.1%	95.3%	0.3%
Colombia	Oporapa	2000	46.9%	96.5%	5.1%
Colombia	Oporapa	2017	25.5%	87.2%	0.7%
Colombia	Orito	2000	61.9%	79.4%	48.2%
Colombia	Orito	2017	32.5%	49.3%	22.4%
Colombia	Orocué	2000	63.3%	92.1%	22.9%
Colombia	Orocué	2017	45.6%	80.3%	13.7%
Colombia	Ortega	2000	53.7%	87.0%	13.4%
Colombia	Ortega	2017	28.0%	72.4%	3.5%
Colombia	Ospina	2000	88.8%	97.4%	71.9%
Colombia	Ospina	2017	55.1%	79.5%	17.7%
Colombia	Otanche	2000	87.4%	100.0%	41.1%
Colombia	Otanche	2017	72.9%	99.5%	14.5%
Colombia	Ovejas	2000	93.1%	97.8%	86.4%
Colombia	Ovejas	2017	76.5%	86.7%	64.6%
Colombia	Pachavita	2000	93.5%	99.9%	59.5%
Colombia	Pachavita	2017	74.9%	98.9%	23.4%

Country	Second Administrative Unit	Year	Mean	Upper	Lower
Colombia	Pacho	2000	69.1%	98.1%	17.1%
Colombia	Pacho	2017	41.2%	89.1%	3.5%
Colombia	Pacoa	2000	37.1%	56.6%	20.1%
Colombia	Pacoa	2017	23.2%	40.8%	9.1%
Colombia	Pácora	2000	80.5%	92.8%	55.8%
Colombia	Pácora	2017	45.3%	63.7%	29.1%
Colombia	Padilla	2000	89.0%	93.0%	82.4%
Colombia	Padilla	2017	41.0%	50.9%	32.1%
Colombia	Páez	2000	82.4%	94.9%	65.4%
Colombia	Páez	2000	68.5%	100.0%	0.6%
Colombia	Páez	2017	53.9%	99.9%	0.1%
Colombia	Páez	2017	69.2%	83.9%	54.0%
Colombia	Paicol	2000	90.5%	99.4%	76.8%
Colombia	Paicol	2017	79.3%	95.1%	58.9%
Colombia	Pailitas	2000	92.6%	99.6%	60.1%
Colombia	Pailitas	2017	76.6%	94.6%	27.5%
Colombia	Paime	2000	49.3%	99.3%	0.1%
Colombia	Paime	2017	30.0%	94.7%	0.0%
Colombia	Paipa	2000	86.0%	99.6%	55.0%
Colombia	Paipa	2017	67.3%	96.4%	30.2%
Colombia	Pajarito	2000	31.5%	98.9%	0.1%
Colombia	Pajarito	2017	23.5%	97.4%	0.0%
Colombia	Palermo	2000	96.3%	99.3%	85.9%
Colombia	Palermo	2017	83.2%	94.0%	63.1%
Colombia	Palestina	2000	49.3%	88.0%	14.0%
Colombia	Palestina	2000	95.4%	99.6%	81.2%
Colombia	Palestina	2017	24.6%	68.6%	3.9%
Colombia	Palestina	2017	83.2%	98.0%	60.2%
Colombia	Palmar	2000	70.4%	100.0%	0.7%
Colombia	Palmar	2017	44.5%	99.8%	0.0%
Colombia	Palmar de Varela	2000	76.1%	99.5%	30.7%
Colombia	Palmar de Varela	2017	46.7%	92.9%	20.4%
Colombia	Palmas del Socorro	2000	74.5%	100.0%	1.3%
Colombia	Palmas del Socorro	2017	54.7%	100.0%	0.1%
Colombia	Palmira	2000	93.7%	94.8%	93.1%
Colombia	Palmira	2017	76.9%	78.8%	74.8%
Colombia	Pamplona	2000	73.9%	98.3%	9.1%
Colombia	Pamplona	2017	48.6%	96.5%	1.9%
Colombia	Pamplonita	2000	72.1%	99.7%	9.4%
Colombia	Pamplonita	2017	44.0%	95.9%	2.8%
Colombia	Pana Pana	2000	43.0%	65.4%	19.7%
Colombia	Pana Pana	2017	27.7%	46.4%	9.4%
Colombia	Pandi	2000	59.5%	100.0%	0.1%
Colombia	Pandi	2017	41.9%	100.0%	0.0%
Colombia	Panqueba	2000	70.1%	97.0%	47.8%
Colombia	Panqueba	2017	34.7%	74.2%	13.0%
Colombia	Papunahua	2000	39.6%	68.2%	13.0%
Colombia	Papunahua	2017	23.9%	51.4%	4.4%
Colombia	Páramo	2000	83.6%	99.6%	25.0%
Colombia	Páramo	2017	54.0%	95.8%	3.0%
Colombia	Paratebueno	2000	62.1%	80.4%	40.6%
Colombia	Paratebueno	2017	29.1%	51.9%	9.5%
Colombia	Pasca	2000	3.2%	11.8%	0.0%
Colombia	Pasca	2017	1.2%	5.8%	0.0%
Colombia	Patía	2000	64.7%	93.1%	27.5%
Colombia	Patía	2017	35.1%	71.1%	7.0%
Colombia	Pauna	2000	84.8%	94.4%	59.1%
Colombia	Pauna	2017	59.8%	80.2%	25.4%

Country	Second Administrative Unit	Year	Mean	Upper	Lower
Colombia	Paya	2000	46.5%	97.4%	2.5%
Colombia	Paya	2017	32.2%	91.0%	0.4%
Colombia	Paz de Ariporo	2000	62.1%	72.4%	50.0%
Colombia	Paz de Ariporo	2017	35.7%	47.1%	25.5%
Colombia	Paz de Río	2000	72.9%	100.0%	0.0%
Colombia	Paz de Río	2017	58.6%	100.0%	0.0%
Colombia	Pedraza	2000	75.4%	99.9%	34.8%
Colombia	Pedraza	2017	62.2%	98.8%	22.5%
Colombia	Pelaya	2000	62.2%	99.4%	8.1%
Colombia	Pelaya	2017	40.8%	94.0%	2.3%
Colombia	Peñol	2000	32.2%	89.8%	0.8%
Colombia	Peñol	2017	13.2%	64.2%	0.1%
Colombia	Pensilvania	2000	89.8%	99.7%	53.1%
Colombia	Pensilvania	2017	73.9%	97.0%	23.3%
Colombia	Pequé	2000	83.5%	98.9%	57.0%
Colombia	Pequé	2017	54.8%	87.2%	25.9%
Colombia	Pereira	2000	94.0%	95.0%	92.6%
Colombia	Pereira	2017	72.9%	75.2%	70.2%
Colombia	Pesca	2000	38.8%	98.3%	0.8%
Colombia	Pesca	2017	21.8%	88.0%	0.0%
Colombia	Piedecuesta	2000	93.4%	95.5%	89.7%
Colombia	Piedecuesta	2017	51.3%	56.1%	46.5%
Colombia	Piedras	2000	50.3%	86.2%	8.6%
Colombia	Piedras	2017	29.5%	68.1%	0.6%
Colombia	Piendamó	2000	95.8%	97.4%	92.6%
Colombia	Piendamó	2017	67.5%	76.4%	57.6%
Colombia	Pijao	2000	99.2%	100.0%	92.2%
Colombia	Pijao	2017	95.3%	99.6%	72.2%
Colombia	Pinchote	2000	88.0%	95.4%	73.5%
Colombia	Pinchote	2017	45.6%	58.7%	33.8%
Colombia	Pinillos	2000	73.7%	94.0%	47.6%
Colombia	Pinillos	2017	52.2%	82.3%	19.9%
Colombia	Piojó	2000	22.2%	81.0%	0.6%
Colombia	Piojó	2017	8.0%	49.3%	0.0%
Colombia	Pisba	2000	41.1%	100.0%	0.0%
Colombia	Pisba	2017	29.6%	99.7%	0.0%
Colombia	Pital	2000	22.8%	59.7%	6.5%
Colombia	Pital	2017	15.2%	42.7%	2.1%
Colombia	Pitalito	2000	83.7%	85.9%	82.0%
Colombia	Pitalito	2017	44.6%	48.2%	41.1%
Colombia	Pivijay	2000	84.6%	99.0%	61.2%
Colombia	Pivijay	2017	69.3%	94.1%	39.4%
Colombia	Planadas	2000	77.7%	96.0%	59.6%
Colombia	Planadas	2017	64.4%	85.3%	47.1%
Colombia	Planeta Rica	2000	60.1%	82.4%	38.3%
Colombia	Planeta Rica	2017	29.7%	54.1%	10.3%
Colombia	Plato	2000	76.3%	95.7%	50.2%
Colombia	Plato	2017	55.3%	83.8%	25.7%
Colombia	Policarpa	2000	59.0%	91.3%	26.6%
Colombia	Policarpa	2017	27.1%	63.5%	7.4%
Colombia	Polonuevo	2000	94.3%	99.2%	69.3%
Colombia	Polonuevo	2017	63.2%	88.8%	14.6%
Colombia	Ponedera	2000	98.5%	99.1%	97.2%
Colombia	Ponedera	2017	81.0%	86.8%	72.0%
Colombia	Popayán	2000	92.5%	94.1%	89.6%
Colombia	Popayán	2017	50.4%	56.1%	42.5%
Colombia	Pore	2000	66.8%	94.2%	37.9%
Colombia	Pore	2017	33.3%	67.9%	7.2%
Colombia	Potosí	2000	82.5%	91.6%	65.7%
Colombia	Potosí	2017	32.1%	46.2%	15.6%

Country	Second Administrative Unit	Year	Mean	Upper	Lower
Colombia	Pradera	2000	74.0%	78.0%	70.2%
Colombia	Pradera	2017	39.7%	44.2%	35.1%
Colombia	Prado	2000	98.4%	99.8%	94.2%
Colombia	Prado	2017	87.6%	96.8%	62.5%
Colombia	Providencia	2000	96.2%	99.0%	83.4%
Colombia	Providencia	2017	76.5%	94.3%	25.0%
Colombia	Pueblo Nuevo	2000	21.4%	78.2%	0.3%
Colombia	Pueblo Nuevo	2017	13.9%	58.0%	0.0%
Colombia	Pueblo Rico	2000	31.1%	80.6%	3.5%
Colombia	Pueblo Rico	2017	17.8%	61.5%	1.5%
Colombia	Pueblo Viejo	2000	48.9%	72.1%	34.5%
Colombia	Pueblo Viejo	2017	19.5%	29.8%	9.8%
Colombia	Pueblorrico	2000	64.1%	92.4%	15.4%
Colombia	Pueblorrico	2017	27.5%	57.4%	2.3%
Colombia	Puente Nacional	2000	12.3%	44.7%	3.1%
Colombia	Puente Nacional	2017	3.2%	11.4%	0.3%
Colombia	Puerres	2000	94.9%	99.2%	79.8%
Colombia	Puerres	2017	76.4%	89.3%	44.4%
Colombia	Puerto Asís	2000	48.6%	60.0%	35.8%
Colombia	Puerto Asís	2017	18.9%	30.1%	9.7%
Colombia	Puerto Berrío	2000	89.5%	99.0%	67.0%
Colombia	Puerto Berrío	2017	69.5%	88.0%	43.6%
Colombia	Puerto Boyacá	2000	89.0%	95.5%	80.1%
Colombia	Puerto Boyacá	2017	51.8%	64.4%	38.7%
Colombia	Puerto Caicedo	2000	35.6%	59.4%	23.0%
Colombia	Puerto Caicedo	2017	9.8%	22.7%	3.6%
Colombia	Puerto Carreño	2000	74.6%	84.1%	60.0%
Colombia	Puerto Carreño	2017	39.2%	51.0%	28.0%
Colombia	Puerto Colombia	2000	42.2%	64.7%	21.9%
Colombia	Puerto Colombia	2000	98.5%	99.5%	95.6%
Colombia	Puerto Colombia	2017	81.5%	90.3%	58.4%
Colombia	Puerto Colombia	2017	25.1%	43.7%	8.7%
Colombia	Puerto Concordia	2000	61.5%	76.5%	38.4%
Colombia	Puerto Concordia	2017	41.3%	61.2%	16.3%
Colombia	Puerto Escondido	2000	62.9%	99.7%	5.5%
Colombia	Puerto Escondido	2017	43.5%	96.7%	0.6%
Colombia	Puerto Gaitán	2000	66.2%	82.2%	47.9%
Colombia	Puerto Gaitán	2017	49.2%	70.0%	25.1%
Colombia	Puerto Guzmán	2000	61.4%	95.8%	19.9%
Colombia	Puerto Guzmán	2017	46.1%	87.3%	8.0%
Colombia	Puerto Inírida	2000	38.2%	48.2%	28.9%
Colombia	Puerto Inírida	2017	16.3%	23.9%	9.3%
Colombia	Puerto Leguízamo	2000	54.5%	84.9%	21.1%

Country	Second Administrative Unit	Year	Mean	Upper	Lower
Colombia	Puerto Leguízamo	2017	42.7%	73.8%	10.8%
Colombia	Puerto Libertador	2000	72.9%	89.1%	48.1%
Colombia	Puerto Libertador	2017	59.4%	80.3%	26.6%
Colombia	Puerto Lleras	2000	71.1%	92.1%	45.2%
Colombia	Puerto Lleras	2017	45.0%	68.7%	24.8%
Colombia	Puerto López	2000	73.6%	88.7%	53.1%
Colombia	Puerto López	2017	43.9%	65.1%	22.5%
Colombia	Puerto Nare	2000	85.0%	99.0%	53.2%
Colombia	Puerto Nare	2017	57.6%	90.5%	22.4%
Colombia	Puerto Nariño	2000	14.5%	22.4%	7.1%
Colombia	Puerto Nariño	2017	3.8%	9.2%	0.8%
Colombia	Puerto Parra	2000	62.8%	99.9%	3.2%
Colombia	Puerto Parra	2017	49.0%	99.4%	0.3%
Colombia	Puerto Rico	2000	69.9%	88.6%	46.0%
Colombia	Puerto Rico	2000	66.9%	82.6%	53.1%
Colombia	Puerto Rico	2017	30.7%	48.3%	17.8%
Colombia	Puerto Rico	2017	41.5%	61.8%	20.2%
Colombia	Puerto Rondón	2000	70.7%	86.7%	50.2%
Colombia	Puerto Rondón	2017	50.2%	76.9%	24.5%
Colombia	Puerto Salgar	2000	95.1%	96.8%	90.6%
Colombia	Puerto Salgar	2017	57.7%	65.0%	43.8%
Colombia	Puerto Santander	2000	48.1%	74.5%	20.9%
Colombia	Puerto Santander	2000	98.2%	100.0%	78.4%
Colombia	Puerto Santander	2017	34.5%	62.5%	7.8%
Colombia	Puerto Santander	2017	93.3%	99.8%	58.0%
Colombia	Puerto Tejada	2000	91.4%	93.0%	89.6%
Colombia	Puerto Tejada	2017	58.6%	64.6%	52.2%
Colombia	Puerto Triunfo	2000	94.1%	98.7%	83.9%
Colombia	Puerto Triunfo	2017	69.0%	85.9%	33.4%
Colombia	Puerto Wilches	2000	77.1%	91.3%	42.2%
Colombia	Puerto Wilches	2017	57.1%	81.0%	16.3%
Colombia	Pulí	2000	43.5%	99.9%	0.4%
Colombia	Pulí	2017	26.3%	98.2%	0.0%
Colombia	Pupiales	2000	65.4%	74.8%	57.8%
Colombia	Pupiales	2017	30.4%	40.8%	23.5%
Colombia	Puracé	2000	95.9%	100.0%	53.5%
Colombia	Puracé	2017	90.3%	100.0%	39.2%
Colombia	Purificación	2000	97.1%	98.1%	95.9%
Colombia	Purificación	2017	81.0%	88.5%	72.7%
Colombia	Purísima	2000	51.2%	95.2%	9.4%
Colombia	Purísima	2017	20.3%	69.6%	0.8%
Colombia	Quebradanegra	2000	70.3%	99.5%	17.5%
Colombia	Quebradanegra	2017	36.8%	90.2%	2.7%
Colombia	Quetame	2000	9.8%	30.6%	2.3%
Colombia	Quetame	2017	3.2%	15.8%	0.6%
Colombia	Quibdó	2000	48.0%	55.9%	39.6%
Colombia	Quibdó	2017	26.9%	34.6%	19.4%
Colombia	Quimbaya	2000	98.9%	99.4%	98.1%
Colombia	Quimbaya	2017	87.3%	92.4%	77.1%

Country	Second Administrative Unit	Year	Mean	Upper	Lower
Colombia	Quinchía	2000	97.5%	100.0%	87.3%
Colombia	Quinchía	2017	89.7%	99.5%	57.8%
Colombia	Quípama	2000	97.1%	98.3%	95.2%
Colombia	Quípama	2017	81.3%	89.0%	68.0%
Colombia	Quipile	2000	43.7%	91.1%	3.0%
Colombia	Quipile	2017	22.9%	79.7%	0.2%
Colombia	Ragonvalia	2000	65.5%	99.6%	3.9%
Colombia	Ragonvalia	2017	41.1%	95.5%	0.3%
Colombia	Ramiriquí	2000	38.4%	98.5%	0.4%
Colombia	Ramiriquí	2017	22.4%	84.8%	0.0%
Colombia	Ráquira	2000	54.4%	98.7%	3.4%
Colombia	Ráquira	2017	28.5%	89.4%	0.2%
Colombia	Recetor	2000	43.7%	81.9%	10.5%
Colombia	Recetor	2017	18.6%	63.1%	1.8%
Colombia	Remedios	2000	69.9%	97.3%	20.9%
Colombia	Remedios	2017	49.3%	90.3%	6.3%
Colombia	Remolino	2000	93.6%	99.9%	76.3%
Colombia	Remolino	2017	84.8%	97.9%	66.5%
Colombia	Repelón	2000	83.8%	96.4%	72.3%
Colombia	Repelón	2017	72.0%	82.8%	66.3%
Colombia	Restrepo	2000	44.4%	92.1%	1.6%
Colombia	Restrepo	2000	77.7%	80.2%	75.4%
Colombia	Restrepo	2017	17.9%	19.9%	16.1%
Colombia	Restrepo	2017	19.5%	74.7%	0.1%
Colombia	Retiro	2000	98.8%	99.4%	97.6%
Colombia	Retiro	2017	84.7%	90.1%	78.1%
Colombia	Ricaurte	2000	93.7%	96.9%	87.3%
Colombia	Ricaurte	2000	71.7%	95.6%	34.6%
Colombia	Ricaurte	2017	50.8%	81.8%	16.7%
Colombia	Ricaurte	2017	58.4%	75.2%	41.0%
Colombia	Río de Oro	2000	82.5%	91.6%	69.8%
Colombia	Río de Oro	2017	35.6%	60.3%	18.5%
Colombia	Río Viejo	2000	52.1%	96.8%	1.3%
Colombia	Río Viejo	2017	32.7%	87.0%	0.1%
Colombia	Rioblanco	2000	61.2%	85.7%	38.0%
Colombia	Rioblanco	2017	49.1%	74.7%	20.8%
Colombia	Riofrío	2000	83.2%	100.0%	17.2%
Colombia	Riofrío	2017	71.5%	100.0%	2.3%
Colombia	Riohacha	2000	80.1%	86.5%	72.9%
Colombia	Riohacha	2017	46.4%	54.9%	38.4%
Colombia	Rionegro	2000	92.9%	95.8%	88.8%
Colombia	Rionegro	2000	40.6%	53.2%	20.2%
Colombia	Rionegro	2017	55.2%	61.6%	47.8%
Colombia	Rionegro	2017	30.9%	48.8%	12.2%
Colombia	Riosucio	2000	59.6%	84.2%	35.7%
Colombia	Riosucio	2000	91.1%	93.3%	88.9%
Colombia	Riosucio	2017	44.6%	74.2%	21.8%
Colombia	Riosucio	2017	74.9%	78.6%	69.4%
Colombia	Risaralda	2000	67.2%	87.0%	56.8%
Colombia	Risaralda	2017	25.3%	36.8%	18.9%
Colombia	Rivera	2000	74.7%	97.9%	47.3%
Colombia	Rivera	2017	58.1%	88.6%	38.9%
Colombia	Roberto Payán	2000	63.4%	93.9%	31.0%
Colombia	Roberto Payán	2017	37.1%	70.5%	10.7%
Colombia	Roldanillo	2000	96.5%	98.4%	90.3%
Colombia	Roldanillo	2017	88.9%	95.8%	69.2%
Colombia	Roncesvalles	2000	40.1%	92.6%	6.9%
Colombia	Roncesvalles	2017	25.6%	80.0%	2.5%
Colombia	Rondón	2000	41.5%	99.8%	0.0%
Colombia	Rondón	2017	26.5%	97.7%	0.0%

Country	Second Administrative Unit	Year	Mean	Upper	Lower
Colombia	Rosas	2000	50.7%	99.2%	7.0%
Colombia	Rosas	2017	26.2%	89.1%	0.9%
Colombia	Rovira	2000	61.8%	98.8%	11.6%
Colombia	Rovira	2017	40.7%	91.6%	4.7%
Colombia	Sabana de Torres	2000	75.0%	90.6%	35.2%
Colombia	Sabana de Torres	2017	52.7%	80.1%	13.6%
Colombia	Sabanagrande	2000	95.1%	99.9%	65.0%
Colombia	Sabanagrande	2017	75.1%	99.1%	25.4%
Colombia	Sabanalarga	2000	65.4%	98.0%	21.2%
Colombia	Sabanalarga	2000	92.8%	95.0%	89.0%
Colombia	Sabanalarga	2000	68.6%	99.9%	18.0%
Colombia	Sabanalarga	2017	47.0%	55.7%	37.8%
Colombia	Sabanalarga	2017	45.2%	98.0%	2.3%
Colombia	Sabanalarga	2017	35.0%	85.2%	2.7%
Colombia	Sabaneta	2000	98.4%	98.8%	97.9%
Colombia	Sabaneta	2017	78.2%	82.3%	73.2%
Colombia	Saboyá	2000	81.7%	85.8%	77.3%
Colombia	Saboyá	2017	20.8%	25.4%	17.3%
Colombia	Sácama	2000	47.7%	92.0%	7.2%
Colombia	Sácama	2017	25.1%	67.6%	1.1%
Colombia	Sáchica	2000	80.5%	95.1%	56.5%
Colombia	Sáchica	2017	30.5%	56.2%	14.5%
Colombia	Saladoblanco	2000	81.3%	98.0%	57.5%
Colombia	Saladoblanco	2017	55.0%	80.0%	31.7%
Colombia	Salamina	2000	83.3%	87.0%	78.4%
Colombia	Salamina	2000	92.9%	95.0%	89.6%
Colombia	Salamina	2017	56.0%	60.6%	49.5%
Colombia	Salamina	2017	61.1%	66.3%	50.0%
Colombia	Salazar de las Palmas	2000	84.0%	97.4%	62.5%
Colombia	Salazar de las Palmas	2017	51.7%	74.3%	26.8%
Colombia	Saldaña	2000	82.4%	97.0%	70.1%
Colombia	Saldaña	2017	50.9%	65.8%	39.7%
Colombia	Salento	2000	94.9%	98.4%	90.7%
Colombia	Salento	2017	72.6%	84.2%	58.8%
Colombia	Salgar	2000	85.8%	96.5%	75.2%
Colombia	Salgar	2017	59.8%	74.7%	34.2%
Colombia	Samacá	2000	80.2%	98.4%	44.8%
Colombia	Samacá	2017	39.9%	83.4%	10.9%
Colombia	Samaná	2000	66.5%	98.3%	33.0%
Colombia	Samaná	2017	46.9%	87.7%	17.8%
Colombia	Samaniego	2000	93.7%	97.4%	85.7%
Colombia	Samaniego	2017	65.1%	79.2%	39.9%
Colombia	Sampués	2000	93.5%	95.6%	91.3%
Colombia	Sampués	2017	81.2%	84.8%	74.6%
Colombia	San Agustín	2000	55.6%	69.5%	36.8%
Colombia	San Agustín	2017	47.2%	61.1%	23.6%
Colombia	San Alberto	2000	68.2%	91.9%	32.0%
Colombia	San Alberto	2017	42.7%	74.4%	12.7%
Colombia	San Andrés de Cuerquia	2000	89.2%	99.8%	31.7%
Colombia	San Andrés de Cuerquia	2000	62.3%	99.8%	2.5%
Colombia	San Andrés de Cuerquia	2017	69.6%	97.6%	9.5%
Colombia	San Andrés de Cuerquia	2017	43.2%	97.0%	0.2%
Colombia	San Andrés de Sotavento	2000	41.9%	48.2%	36.3%

Country	Second Administrative Unit	Year	Mean	Upper	Lower
Colombia	San Andrés de Sotavento	2017	13.9%	19.1%	10.2%
Colombia	San Antero	2000	77.5%	99.6%	7.0%
Colombia	San Antero	2017	52.8%	94.5%	2.3%
Colombia	San Antonio	2000	56.6%	98.0%	6.6%
Colombia	San Antonio	2017	34.5%	90.2%	1.1%
Colombia	San Antonio de Palmito	2000	84.6%	97.7%	51.6%
Colombia	San Antonio de Palmito	2017	53.6%	84.8%	11.4%
Colombia	San Antonio del Tequendama	2000	98.8%	99.6%	97.3%
Colombia	San Antonio del Tequendama	2017	85.2%	92.9%	72.5%
Colombia	San Benito	2000	78.0%	100.0%	3.5%
Colombia	San Benito	2017	60.6%	100.0%	0.2%
Colombia	San Benito Abad	2000	71.1%	96.3%	33.8%
Colombia	San Benito Abad	2017	53.0%	84.4%	17.8%
Colombia	San Bernardino de Sahagún	2000	58.5%	86.2%	26.3%
Colombia	San Bernardino de Sahagún	2017	28.2%	64.0%	5.3%
Colombia	San Bernardo	2000	62.6%	100.0%	0.2%
Colombia	San Bernardo	2000	66.6%	98.0%	26.0%
Colombia	San Bernardo	2017	49.0%	100.0%	0.0%
Colombia	San Bernardo	2017	22.0%	78.3%	3.6%
Colombia	San Bernardo del Viento	2000	75.9%	83.9%	63.7%
Colombia	San Bernardo del Viento	2017	45.6%	57.5%	31.9%
Colombia	San Calixto	2000	50.4%	88.8%	10.8%
Colombia	San Calixto	2017	24.9%	65.3%	2.4%
Colombia	San Carlos	2000	62.3%	74.5%	36.1%
Colombia	San Carlos	2000	69.5%	98.9%	8.1%
Colombia	San Carlos	2017	43.1%	60.8%	12.3%
Colombia	San Carlos	2017	49.5%	95.1%	1.6%
Colombia	San Carlos de Guaroa	2000	61.5%	87.4%	39.9%
Colombia	San Carlos de Guaroa	2017	36.2%	65.3%	12.0%
Colombia	San Cayetano	2000	83.7%	100.0%	33.8%
Colombia	San Cayetano	2000	91.0%	100.0%	37.1%
Colombia	San Cayetano	2017	73.0%	99.8%	28.5%
Colombia	San Cayetano	2017	77.4%	99.8%	8.9%
Colombia	San Diego	2000	86.1%	99.2%	32.8%
Colombia	San Diego	2017	76.3%	95.0%	18.9%
Colombia	San Eduardo	2000	66.8%	99.9%	2.5%
Colombia	San Eduardo	2017	45.3%	98.7%	0.4%
Colombia	San Estanislao de Kostka	2000	98.7%	99.5%	94.9%
Colombia	San Estanislao de Kostka	2017	91.2%	95.6%	79.0%
Colombia	San Felipe	2000	39.9%	66.1%	15.6%
Colombia	San Felipe	2017	23.3%	45.9%	5.1%
Colombia	San Fernando	2000	89.4%	97.7%	68.2%
Colombia	San Fernando	2017	60.1%	79.7%	35.2%

Country	Second Administrative Unit	Year	Mean	Upper	Lower
Colombia	San Francisco	2000	83.2%	99.2%	27.5%
Colombia	San Francisco	2000	45.9%	98.7%	0.6%
Colombia	San Francisco	2000	66.6%	93.9%	31.1%
Colombia	San Francisco	2017	29.7%	90.3%	0.0%
Colombia	San Francisco	2017	58.4%	93.6%	10.8%
Colombia	San Francisco	2017	29.4%	69.7%	7.2%
Colombia	San Gil	2000	71.5%	99.1%	24.2%
Colombia	San Gil	2017	35.3%	89.5%	3.8%
Colombia	San Jacinto	2000	72.9%	90.2%	53.4%
Colombia	San Jacinto	2017	35.3%	57.1%	16.7%
Colombia	San Jerónimo	2000	99.0%	100.0%	92.9%
Colombia	San Jerónimo	2017	95.4%	100.0%	70.2%
Colombia	San Joaquín	2000	57.1%	96.1%	8.4%
Colombia	San Joaquín	2017	27.7%	77.4%	0.8%
Colombia	San José de Cúcuta	2000	92.0%	95.3%	88.2%
Colombia	San José de Cúcuta	2017	65.3%	71.0%	61.4%
Colombia	San José de la Montaña	2000	83.9%	99.9%	27.7%
Colombia	San José de la Montaña	2017	63.1%	99.1%	6.5%
Colombia	San José de Miranda	2000	85.7%	93.6%	69.5%
Colombia	San José de Miranda	2017	38.8%	58.5%	20.6%
Colombia	San Jose de Ocune	2000	59.3%	69.7%	48.9%
Colombia	San Jose de Ocune	2017	37.9%	49.3%	28.3%
Colombia	San José de Pare	2000	84.9%	91.7%	63.6%
Colombia	San José de Pare	2017	35.8%	49.4%	12.9%
Colombia	San José del Fragua	2000	57.6%	86.0%	23.3%
Colombia	San José del Fragua	2017	29.6%	60.1%	3.7%
Colombia	San José del Guaviare	2000	54.7%	60.9%	49.2%
Colombia	San José del Guaviare	2017	30.5%	35.8%	25.7%
Colombia	San José del Palmar	2000	71.9%	97.6%	18.2%
Colombia	San José del Palmar	2017	45.5%	86.7%	4.5%
Colombia	San Juan de Arama	2000	78.1%	95.2%	50.1%
Colombia	San Juan de Arama	2017	59.0%	85.4%	26.5%
Colombia	San Juan de Betulia	2000	98.2%	99.8%	85.4%
Colombia	San Juan de Betulia	2017	88.0%	96.0%	66.2%
Colombia	San Juan de Pasto	2000	88.8%	94.9%	84.4%
Colombia	San Juan de Pasto	2017	61.4%	67.7%	55.8%
Colombia	San Juan de Río Seco	2000	50.0%	67.6%	36.1%
Colombia	San Juan de Río Seco	2017	43.9%	61.3%	19.5%

Country	Second Administrative Unit	Year	Mean	Upper	Lower
Colombia	San Juan de Urabá	2000	72.9%	99.4%	24.6%
Colombia	San Juan de Urabá	2017	50.1%	93.2%	7.9%
Colombia	San Juan del Cesar	2000	29.5%	53.0%	15.8%
Colombia	San Juan del Cesar	2017	25.5%	41.6%	15.5%
Colombia	San Juan Nepomuceno	2000	17.5%	29.8%	10.6%
Colombia	San Juan Nepomuceno	2017	10.4%	19.9%	4.2%
Colombia	San Juanito	2000	85.6%	100.0%	16.3%
Colombia	San Juanito	2017	74.3%	100.0%	3.3%
Colombia	San Lorenzo	2000	98.7%	99.9%	95.7%
Colombia	San Lorenzo	2017	91.6%	98.5%	80.6%
Colombia	San Luis	2000	56.5%	95.4%	13.9%
Colombia	San Luis	2017	27.7%	75.3%	3.9%
Colombia	San Luís	2000	81.2%	90.9%	72.0%
Colombia	San Luís	2017	48.8%	57.9%	39.1%
Colombia	San Luis de Cubarral	2000	62.7%	91.6%	31.2%
Colombia	San Luis de Cubarral	2017	37.0%	76.2%	12.4%
Colombia	San Luis de Gaceno	2000	48.4%	99.9%	0.1%
Colombia	San Luis de Gaceno	2017	32.3%	98.7%	0.0%
Colombia	San Luis de Palenque	2000	56.4%	90.4%	20.4%
Colombia	San Luis de Palenque	2017	43.8%	83.7%	6.5%
Colombia	San Marcos	2000	74.3%	82.0%	65.0%
Colombia	San Marcos	2017	42.1%	56.2%	27.9%
Colombia	San Martín	2000	73.9%	87.1%	52.6%
Colombia	San Martín	2000	64.8%	89.9%	32.6%
Colombia	San Martín	2017	42.3%	62.2%	23.1%
Colombia	San Martín	2017	29.9%	66.4%	6.9%
Colombia	San Martín de Loba	2000	72.7%	87.0%	53.4%
Colombia	San Martín de Loba	2017	38.9%	57.5%	25.0%
Colombia	San Mateo	2000	66.7%	75.1%	58.3%
Colombia	San Mateo	2017	16.3%	22.5%	12.3%
Colombia	San Miguel	2000	60.3%	70.6%	53.5%
Colombia	San Miguel	2017	24.2%	33.6%	16.0%
Colombia	San Miguel de Mocoa	2000	89.2%	94.1%	82.3%
Colombia	San Miguel de Mocoa	2017	46.9%	55.0%	38.6%
Colombia	San Miguel de Sema	2000	70.3%	85.5%	56.9%
Colombia	San Miguel de Sema	2017	28.8%	35.1%	23.7%
Colombia	San Onofre	2000	84.0%	97.8%	56.6%
Colombia	San Onofre	2017	63.1%	86.0%	34.2%
Colombia	San Pablo	2000	96.2%	97.9%	93.9%
Colombia	San Pablo	2000	83.8%	98.0%	56.9%
Colombia	San Pablo	2017	69.3%	76.7%	58.9%
Colombia	San Pablo	2017	65.0%	90.8%	19.2%
Colombia	San Pablo de Borbur	2000	88.5%	97.6%	58.1%

Country	Second Administrative Unit	Year	Mean	Upper	Lower
Colombia	San Pablo de Borbur	2017	55.6%	85.4%	14.2%
Colombia	San Pedro	2000	99.6%	100.0%	96.2%
Colombia	San Pedro	2000	62.3%	79.4%	44.3%
Colombia	San Pedro	2017	98.6%	100.0%	91.4%
Colombia	San Pedro	2017	37.1%	62.0%	14.2%
Colombia	San Pedro de Cartago	2000	98.5%	99.4%	96.8%
Colombia	San Pedro de Cartago	2017	83.6%	90.3%	74.9%
Colombia	San Pedro de los Milagros	2000	99.1%	100.0%	89.3%
Colombia	San Pedro de los Milagros	2017	97.4%	100.0%	76.0%
Colombia	San Pedro de Urabá	2000	73.0%	82.5%	60.0%
Colombia	San Pedro de Urabá	2017	41.1%	54.1%	27.1%
Colombia	San Pelayo	2000	30.0%	52.4%	11.3%
Colombia	San Pelayo	2017	13.0%	33.4%	1.3%
Colombia	San Rafael	2000	51.8%	99.8%	2.3%
Colombia	San Rafael	2017	35.2%	96.3%	0.2%
Colombia	San Roque	2000	86.2%	98.5%	52.0%
Colombia	San Roque	2017	54.3%	82.5%	20.5%
Colombia	San Sebastián	2000	73.3%	99.6%	23.2%
Colombia	San Sebastián	2017	56.4%	97.8%	13.2%
Colombia	San Sebastián de Buenavista	2000	78.7%	99.9%	22.6%
Colombia	San Sebastián de Buenavista	2017	61.4%	98.9%	7.1%
Colombia	San Sebastian de Mariquita	2000	90.9%	95.0%	86.3%
Colombia	San Sebastian de Mariquita	2017	56.0%	61.9%	49.7%
Colombia	San Vicente	2000	91.0%	99.8%	68.0%
Colombia	San Vicente	2017	76.5%	96.9%	43.3%
Colombia	San Vicente de Chucurí	2000	60.4%	76.2%	42.0%
Colombia	San Vicente de Chucurí	2017	35.5%	56.6%	14.9%
Colombia	San Vicente del Caguán	2000	74.5%	83.6%	66.4%
Colombia	San Vicente del Caguán	2017	50.6%	59.1%	42.1%
Colombia	San Zenón	2000	92.3%	99.4%	71.3%
Colombia	San Zenón	2017	70.2%	92.3%	25.0%
Colombia	Sandoná	2000	99.5%	99.8%	98.4%
Colombia	Sandoná	2017	95.0%	98.3%	85.7%
Colombia	Santa Ana	2000	62.1%	86.7%	33.1%
Colombia	Santa Ana	2017	42.7%	70.2%	15.1%
Colombia	Santa Bárbara	2000	98.4%	100.0%	88.4%
Colombia	Santa Bárbara	2000	78.4%	98.2%	47.2%
Colombia	Santa Bárbara	2000	45.7%	86.9%	9.6%
Colombia	Santa Bárbara	2017	46.4%	80.0%	12.8%
Colombia	Santa Bárbara	2017	94.8%	99.8%	75.7%
Colombia	Santa Bárbara	2017	29.0%	71.4%	3.0%
Colombia	Santa Catalina	2000	28.2%	56.8%	10.6%
Colombia	Santa Catalina	2017	6.7%	22.8%	1.1%
Colombia	Santa Cruz	2000	89.9%	99.8%	59.2%
Colombia	Santa Cruz	2017	73.8%	96.3%	24.1%

Country	Second Administrative Unit	Year	Mean	Upper	Lower
Colombia	Santa Cruz de Lorica	2000	63.0%	86.0%	34.3%
Colombia	Santa Cruz de Lorica	2017	27.4%	57.8%	4.9%
Colombia	Santa Fe de Antioquia	2000	83.5%	99.9%	31.4%
Colombia	Santa Fe de Antioquia	2017	65.9%	98.1%	17.3%
Colombia	Santa Helena del Opón	2000	61.3%	99.7%	3.3%
Colombia	Santa Helena del Opón	2017	44.7%	97.7%	0.4%
Colombia	Santa Isabel	2000	76.1%	99.7%	27.4%
Colombia	Santa Isabel	2017	56.7%	97.4%	12.9%
Colombia	Santa Lucía	2000	98.2%	100.0%	87.5%
Colombia	Santa Lucía	2017	92.1%	100.0%	36.9%
Colombia	Santa María	2000	84.9%	100.0%	16.5%
Colombia	Santa María	2000	40.2%	98.7%	0.0%
Colombia	Santa María	2017	73.2%	100.0%	7.9%
Colombia	Santa María	2017	25.5%	92.5%	0.0%
Colombia	Santa Marta (Dist. Esp.)	2000	86.1%	88.2%	83.9%
Colombia	Santa Marta (Dist. Esp.)	2017	34.5%	38.0%	31.3%
Colombia	Santa Rita	2000	60.0%	77.8%	43.9%
Colombia	Santa Rita	2017	40.0%	56.8%	24.9%
Colombia	Santa Rosa	2000	79.4%	92.1%	60.8%
Colombia	Santa Rosa	2000	55.5%	85.8%	23.2%
Colombia	Santa Rosa	2017	40.1%	71.8%	14.3%
Colombia	Santa Rosa	2017	24.1%	41.1%	11.5%
Colombia	Santa Rosa de Cabal	2000	94.4%	97.0%	87.6%
Colombia	Santa Rosa de Cabal	2017	54.7%	63.9%	45.4%
Colombia	Santa Rosa de Osos	2000	88.6%	98.8%	46.4%
Colombia	Santa Rosa de Osos	2017	66.4%	89.0%	25.6%
Colombia	Santa Rosa de Viterbo	2000	97.9%	98.5%	96.7%
Colombia	Santa Rosa de Viterbo	2017	72.8%	77.6%	66.5%
Colombia	Santa Rosa del Sur	2000	39.4%	59.2%	22.3%
Colombia	Santa Rosa del Sur	2017	25.2%	44.1%	12.7%
Colombia	Santa Rosalía	2000	63.9%	81.6%	46.2%
Colombia	Santa Rosalía	2017	37.4%	52.8%	23.3%
Colombia	Santa Sofía	2000	57.4%	90.4%	18.4%
Colombia	Santa Sofía	2017	17.2%	49.6%	2.4%
Colombia	Santafé de Bogotá	2000	98.3%	98.7%	97.7%
Colombia	Santafé de Bogotá	2017	85.3%	86.3%	84.1%
Colombia	Santana	2000	79.8%	100.0%	8.4%
Colombia	Santana	2017	61.0%	100.0%	0.6%
Colombia	Santander de Quilichao	2000	79.9%	86.5%	71.2%
Colombia	Santander de Quilichao	2017	43.4%	51.2%	34.7%
Colombia	Santiago	2000	75.0%	99.9%	11.1%
Colombia	Santiago	2000	74.8%	96.3%	43.1%

Country	Second Administrative Unit	Year	Mean	Upper	Lower
Colombia	Santiago	2017	52.1%	98.6%	0.8%
Colombia	Santiago	2017	45.3%	78.4%	13.3%
Colombia	Santiago de Cali	2000	98.9%	99.1%	98.8%
Colombia	Santiago de Cali	2017	83.8%	85.4%	81.8%
Colombia	Santo Domingo	2000	79.2%	99.3%	34.5%
Colombia	Santo Domingo	2017	50.0%	90.0%	8.4%
Colombia	Santo Domingo de Silos	2000	65.5%	100.0%	1.0%
Colombia	Santo Domingo de Silos	2017	54.3%	100.0%	0.1%
Colombia	Santo Tomás	2000	81.3%	99.9%	24.0%
Colombia	Santo Tomás	2017	52.3%	98.4%	2.9%
Colombia	Santuario	2000	80.2%	87.6%	66.9%
Colombia	Santuario	2017	33.4%	49.7%	22.1%
Colombia	Sapuyes	2000	65.2%	97.5%	7.9%
Colombia	Sapuyes	2017	47.5%	93.6%	1.2%
Colombia	Saravena	2000	92.2%	96.8%	85.0%
Colombia	Saravena	2017	66.7%	76.1%	56.4%
Colombia	Sardinata	2000	70.8%	87.4%	46.9%
Colombia	Sardinata	2017	47.2%	67.9%	24.8%
Colombia	Sasaima	2000	81.7%	99.8%	28.1%
Colombia	Sasaima	2017	60.6%	97.7%	6.2%
Colombia	Sativanorte	2000	58.2%	100.0%	0.0%
Colombia	Sativanorte	2017	44.9%	100.0%	0.0%
Colombia	Sativasur	2000	64.4%	100.0%	0.0%
Colombia	Sativasur	2017	50.8%	100.0%	0.0%
Colombia	Segovia	2000	76.7%	96.4%	47.5%
Colombia	Segovia	2017	47.2%	76.8%	15.1%
Colombia	Sesquilé	2000	94.6%	100.0%	59.1%
Colombia	Sesquilé	2017	89.4%	100.0%	23.5%
Colombia	Sevilla	2000	74.4%	90.3%	57.2%
Colombia	Sevilla	2017	51.8%	76.4%	19.9%
Colombia	Siachoque	2000	27.0%	71.6%	5.1%
Colombia	Siachoque	2017	6.4%	34.0%	0.3%
Colombia	Sibaté	2000	61.1%	90.3%	20.9%
Colombia	Sibaté	2017	30.2%	80.0%	3.5%
Colombia	Sibundoy	2000	85.9%	98.3%	46.0%
Colombia	Sibundoy	2017	48.0%	84.2%	6.2%
Colombia	Silvania	2000	83.3%	87.1%	79.7%
Colombia	Silvania	2017	35.6%	44.0%	28.3%
Colombia	Silvia	2000	51.7%	74.0%	42.4%
Colombia	Silvia	2017	29.5%	39.8%	22.7%
Colombia	Simacota	2000	61.5%	98.6%	17.3%
Colombia	Simacota	2017	46.3%	91.9%	6.0%
Colombia	Simijaca	2000	94.6%	97.1%	88.9%
Colombia	Simijaca	2017	67.3%	77.6%	53.8%
Colombia	Simití	2000	69.5%	93.8%	43.6%
Colombia	Simití	2017	57.2%	86.2%	30.3%
Colombia	Sincé	2000	92.5%	98.7%	80.7%
Colombia	Sincé	2017	74.1%	86.6%	46.2%
Colombia	Sincelejo	2000	94.9%	95.5%	94.2%
Colombia	Sincelejo	2017	63.3%	65.8%	60.2%
Colombia	Sipí	2000	37.8%	88.1%	3.0%
Colombia	Sipí	2017	23.8%	77.4%	0.3%
Colombia	Soacha	2000	90.8%	97.4%	82.6%
Colombia	Soacha	2017	65.8%	75.0%	57.4%

Country	Second Administrative Unit	Year	Mean	Upper	Lower
Colombia	Soatá	2000	55.3%	96.0%	3.6%
Colombia	Soatá	2017	17.2%	66.5%	0.1%
Colombia	Socha	2000	69.9%	99.3%	6.9%
Colombia	Socha	2017	51.8%	96.6%	1.0%
Colombia	Socorro	2000	83.0%	99.3%	41.4%
Colombia	Socorro	2017	47.3%	91.5%	8.8%
Colombia	Socotá	2000	54.0%	99.5%	1.3%
Colombia	Socotá	2017	40.4%	94.6%	0.1%
Colombia	Sogamoso	2000	93.9%	96.4%	90.5%
Colombia	Sogamoso	2017	57.1%	67.2%	46.4%
Colombia	Solano	2000	54.0%	67.1%	41.3%
Colombia	Solano	2017	39.6%	56.0%	25.8%
Colombia	Soledad	2000	96.0%	96.3%	95.8%
Colombia	Soledad	2017	57.4%	59.5%	55.4%
Colombia	Somondoco	2000	69.4%	99.7%	4.1%
Colombia	Somondoco	2017	45.2%	98.0%	1.0%
Colombia	Sonsón	2000	73.0%	94.2%	44.6%
Colombia	Sonsón	2017	43.7%	74.7%	16.8%
Colombia	Sopetrán	2000	91.1%	99.6%	54.1%
Colombia	Sopetrán	2017	66.1%	91.7%	26.9%
Colombia	Soplaviento	2000	99.1%	99.7%	96.9%
Colombia	Soplaviento	2017	93.7%	96.6%	85.1%
Colombia	Sopó	2000	92.7%	95.5%	88.4%
Colombia	Sopó	2017	55.4%	67.3%	39.7%
Colombia	Sora	2000	84.7%	99.7%	40.9%
Colombia	Sora	2017	47.1%	93.8%	5.7%
Colombia	Soracá	2000	19.5%	70.5%	6.4%
Colombia	Soracá	2017	5.8%	15.8%	3.2%
Colombia	Sotaquirá	2000	83.1%	97.9%	38.7%
Colombia	Sotaquirá	2017	49.9%	80.1%	15.5%
Colombia	Sotará	2000	88.4%	94.0%	76.7%
Colombia	Sotará	2017	65.8%	77.9%	46.9%
Colombia	Suaita	2000	72.1%	99.6%	12.5%
Colombia	Suaita	2017	48.4%	95.0%	3.6%
Colombia	Suan	2000	97.6%	100.0%	72.8%
Colombia	Suan	2017	89.6%	99.8%	35.8%
Colombia	Suárez	2000	92.2%	96.1%	83.1%
Colombia	Suárez	2000	45.8%	97.9%	8.5%
Colombia	Suárez	2017	22.0%	85.9%	1.2%
Colombia	Suárez	2017	56.9%	70.9%	45.7%
Colombia	Suaza	2000	52.1%	87.9%	14.3%
Colombia	Suaza	2017	26.2%	76.3%	2.2%
Colombia	Subachoque	2000	97.7%	99.2%	93.2%
Colombia	Subachoque	2017	86.0%	91.5%	78.4%
Colombia	Sucre	2000	48.5%	94.0%	4.5%
Colombia	Sucre	2000	65.5%	90.4%	27.9%
Colombia	Sucre	2017	33.1%	82.4%	1.0%
Colombia	Sucre	2017	39.9%	73.7%	5.2%
Colombia	Suesca	2000	92.4%	98.1%	84.2%
Colombia	Suesca	2017	91.1%	96.5%	61.5%
Colombia	Supatá	2000	79.2%	100.0%	7.7%
Colombia	Supatá	2017	60.3%	99.9%	0.7%
Colombia	Supía	2000	98.9%	99.4%	98.1%
Colombia	Supía	2017	84.3%	90.2%	76.5%
Colombia	Suratá	2000	70.9%	100.0%	1.7%
Colombia	Suratá	2017	55.1%	100.0%	0.1%
Colombia	Susa	2000	86.9%	98.4%	49.4%
Colombia	Susa	2017	60.5%	88.7%	18.8%
Colombia	Susacón	2000	53.1%	99.9%	0.0%
Colombia	Susacón	2017	37.1%	98.5%	0.0%
Colombia	Sutamarchán	2000	65.9%	86.3%	40.8%
Colombia	Sutamarchán	2017	14.6%	38.1%	4.6%

Country	Second Administrative Unit	Year	Mean	Upper	Lower
Colombia	Sutatausa	2000	94.1%	99.0%	72.3%
Colombia	Sutatausa	2017	66.8%	86.8%	28.6%
Colombia	Sutatenza	2000	79.4%	100.0%	15.9%
Colombia	Sutatenza	2017	57.8%	99.7%	2.3%
Colombia	Tabio	2000	97.9%	99.3%	94.6%
Colombia	Tabio	2017	79.2%	92.8%	51.1%
Colombia	Tadó	2000	54.4%	61.8%	45.5%
Colombia	Tadó	2017	17.0%	23.4%	11.8%
Colombia	Talaigua Nuevo	2000	43.9%	98.3%	3.1%
Colombia	Talaigua Nuevo	2017	22.6%	84.6%	0.3%
Colombia	Tamalameque	2000	46.9%	93.0%	7.3%
Colombia	Tamalameque	2017	29.5%	77.5%	1.3%
Colombia	Támara	2000	46.4%	83.4%	6.7%
Colombia	Támara	2017	28.0%	70.2%	0.9%
Colombia	Tame	2000	61.7%	74.6%	50.7%
Colombia	Tame	2017	46.0%	57.5%	36.0%
Colombia	Támesis	2000	37.4%	79.8%	10.4%
Colombia	Támesis	2017	16.2%	56.9%	1.6%
Colombia	Taminango	2000	90.6%	99.5%	62.5%
Colombia	Taminango	2017	70.7%	92.9%	42.6%
Colombia	Tangua	2000	34.2%	93.2%	1.2%
Colombia	Tangua	2017	17.9%	88.0%	0.1%
Colombia	Taraira	2000	45.6%	80.7%	8.6%
Colombia	Taraira	2017	33.2%	68.1%	4.1%
Colombia	Tarapacá	2000	39.9%	56.6%	26.5%
Colombia	Tarapacá	2017	24.6%	42.1%	13.2%
Colombia	Tarazá	2000	68.9%	91.9%	48.3%
Colombia	Tarazá	2017	45.6%	67.4%	25.7%
Colombia	Tarquí	2000	78.8%	99.7%	25.1%
Colombia	Tarquí	2017	59.8%	97.8%	11.0%
Colombia	Tarso	2000	95.8%	100.0%	75.9%
Colombia	Tarso	2017	84.8%	99.4%	38.9%
Colombia	Tasco	2000	78.5%	99.8%	16.1%
Colombia	Tasco	2017	59.3%	97.6%	3.3%
Colombia	Tauramena	2000	55.3%	70.3%	39.0%
Colombia	Tauramena	2017	37.3%	47.5%	26.4%
Colombia	Tausa	2000	89.2%	93.4%	82.5%
Colombia	Tausa	2017	55.9%	66.4%	43.5%
Colombia	Tello	2000	98.9%	100.0%	91.3%
Colombia	Tello	2017	97.1%	100.0%	85.0%
Colombia	Tena	2000	95.2%	96.6%	93.0%
Colombia	Tena	2017	51.9%	60.2%	42.3%
Colombia	Tenerife	2000	65.9%	78.3%	50.5%
Colombia	Tenerife	2017	41.8%	57.1%	22.3%
Colombia	Tenjo	2000	61.1%	70.0%	53.5%
Colombia	Tenjo	2017	30.7%	33.7%	27.8%
Colombia	Tenza	2000	87.9%	99.9%	41.3%
Colombia	Tenza	2017	67.7%	99.1%	15.8%
Colombia	Teorama	2000	80.9%	93.8%	61.3%
Colombia	Teorama	2017	46.1%	70.2%	23.1%
Colombia	Teruel	2000	95.3%	99.7%	79.4%
Colombia	Teruel	2017	84.5%	97.4%	53.6%
Colombia	Tesalia	2000	96.4%	100.0%	76.4%
Colombia	Tesalia	2017	88.4%	99.7%	55.8%
Colombia	Tibacuy	2000	78.1%	98.2%	44.9%
Colombia	Tibacuy	2017	39.7%	89.5%	9.9%
Colombia	Tibaná	2000	82.8%	98.4%	52.7%
Colombia	Tibaná	2017	51.2%	87.0%	21.7%
Colombia	Tibasosa	2000	98.0%	99.1%	95.5%
Colombia	Tibasosa	2017	81.4%	88.3%	68.4%

Country	Second Administrative Unit	Year	Mean	Upper	Lower
Colombia	Tibirita	2000	67.6%	100.0%	0.1%
Colombia	Tibirita	2017	50.1%	100.0%	0.0%
Colombia	Tibú	2000	55.9%	74.4%	39.8%
Colombia	Tibú	2017	39.3%	58.3%	23.8%
Colombia	Tierralta	2000	51.0%	79.5%	23.2%
Colombia	Tierralta	2017	29.9%	59.8%	9.3%
Colombia	Timaná	2000	51.8%	70.6%	33.9%
Colombia	Timaná	2017	29.3%	52.7%	19.2%
Colombia	Timbío	2000	87.7%	98.0%	59.3%
Colombia	Timbío	2017	69.3%	84.8%	45.6%
Colombia	Timbiquí	2000	48.5%	74.7%	22.1%
Colombia	Timbiquí	2017	28.5%	52.9%	8.4%
Colombia	Tinjacá	2000	53.8%	93.1%	19.1%
Colombia	Tinjacá	2017	12.8%	50.8%	1.9%
Colombia	Tipacoque	2000	53.9%	97.6%	5.3%
Colombia	Tipacoque	2017	21.4%	76.7%	0.3%
Colombia	Titiribí	2000	86.2%	91.9%	76.9%
Colombia	Titiribí	2017	52.8%	65.9%	36.7%
Colombia	Toca	2000	5.3%	27.8%	1.1%
Colombia	Toca	2017	1.1%	9.9%	0.1%
Colombia	Tocaima	2000	81.7%	89.6%	74.1%
Colombia	Tocaima	2017	57.3%	64.9%	45.5%
Colombia	Tocancipá	2000	87.0%	92.7%	77.8%
Colombia	Tocancipá	2017	33.6%	48.8%	21.0%
Colombia	Toguí	2000	89.2%	98.2%	61.5%
Colombia	Toguí	2017	52.3%	79.9%	12.3%
Colombia	Toledo	2000	88.9%	99.3%	53.9%
Colombia	Toledo	2000	72.1%	83.9%	55.4%
Colombia	Toledo	2017	59.8%	91.1%	11.9%
Colombia	Toledo	2017	35.8%	48.1%	19.8%
Colombia	Tolú	2000	87.5%	97.1%	64.2%
Colombia	Tolú	2017	58.2%	80.6%	32.6%
Colombia	Toluviejo	2000	54.1%	92.0%	13.7%
Colombia	Toluviejo	2017	21.0%	72.5%	3.2%
Colombia	Tona	2000	91.5%	99.3%	64.0%
Colombia	Tona	2017	66.9%	90.7%	35.5%
Colombia	Topagá	2000	93.0%	94.8%	90.9%
Colombia	Topagá	2017	52.7%	57.4%	48.1%
Colombia	Topaipí	2000	45.3%	99.3%	0.9%
Colombia	Topaipí	2017	24.5%	93.0%	0.0%
Colombia	Toribío	2000	63.1%	94.1%	33.0%
Colombia	Toribío	2017	23.9%	61.6%	10.8%
Colombia	Toro	2000	36.8%	44.0%	30.9%
Colombia	Toro	2017	19.6%	24.7%	14.5%
Colombia	Tota	2000	58.1%	99.2%	11.9%
Colombia	Tota	2017	34.5%	91.2%	3.4%
Colombia	Totoró	2000	93.3%	99.6%	74.8%
Colombia	Totoró	2017	78.2%	94.4%	51.7%
Colombia	Trinidad	2000	62.5%	84.5%	37.3%
Colombia	Trinidad	2017	38.1%	68.5%	10.8%
Colombia	Trujillo	2000	78.9%	100.0%	12.3%
Colombia	Trujillo	2017	60.1%	99.9%	2.1%
Colombia	Tubará	2000	77.7%	99.8%	33.8%
Colombia	Tubará	2017	61.0%	97.0%	18.2%
Colombia	Tuluá	2000	91.6%	98.2%	84.9%
Colombia	Tuluá	2017	82.5%	90.8%	71.7%
Colombia	Tumaco	2000	57.9%	67.5%	48.7%
Colombia	Tumaco	2017	22.4%	32.3%	15.7%
Colombia	Tunja	2000	85.3%	89.1%	81.5%
Colombia	Tunja	2017	31.7%	36.3%	26.9%
Colombia	Tunungua	2000	99.8%	100.0%	98.6%
Colombia	Tunungua	2017	98.4%	100.0%	86.4%

Country	Second Administrative Unit	Year	Mean	Upper	Lower
Colombia	Túquerres	2000	94.6%	99.9%	64.5%
Colombia	Túquerres	2017	90.0%	99.3%	71.4%
Colombia	Turbaco	2000	75.2%	93.2%	36.6%
Colombia	Turbaco	2017	29.8%	63.6%	7.6%
Colombia	Turbaná	2000	88.4%	97.9%	67.7%
Colombia	Turbaná	2017	52.0%	77.3%	25.8%
Colombia	Turbo	2000	64.3%	73.6%	50.9%
Colombia	Turbo	2017	41.4%	55.2%	25.4%
Colombia	Turmequé	2000	95.0%	99.2%	82.2%
Colombia	Turmequé	2017	66.7%	89.5%	44.9%
Colombia	Tuta	2000	63.2%	77.8%	50.6%
Colombia	Tuta	2017	27.1%	44.4%	15.0%
Colombia	Tutazá	2000	79.5%	100.0%	0.3%
Colombia	Tutazá	2017	68.1%	100.0%	0.0%
Colombia	Ubalá	2000	53.5%	99.9%	0.7%
Colombia	Ubalá	2017	35.2%	98.7%	0.0%
Colombia	Ubaque	2000	95.4%	99.9%	78.8%
Colombia	Ubaque	2017	83.2%	99.1%	53.9%
Colombia	Ulloa	2000	98.8%	99.4%	97.8%
Colombia	Ulloa	2017	97.2%	97.9%	95.7%
Colombia	Umbita	2000	89.7%	100.0%	45.5%
Colombia	Umbita	2017	69.7%	99.5%	17.7%
Colombia	Une	2000	84.9%	98.8%	54.0%
Colombia	Une	2017	59.3%	80.0%	37.6%
Colombia	Unguía	2000	80.0%	90.9%	69.0%
Colombia	Unguía	2017	61.2%	75.9%	40.1%
Colombia	Uramita	2000	91.0%	100.0%	39.9%
Colombia	Uramita	2017	78.8%	100.0%	9.5%
Colombia	Uribia	2000	35.4%	52.3%	19.6%
Colombia	Uribia	2017	18.7%	32.0%	6.9%
Colombia	Urrao	2000	68.4%	93.8%	28.3%
Colombia	Urrao	2017	48.3%	81.9%	12.7%
Colombia	Urumita	2000	73.1%	75.7%	70.0%
Colombia	Urumita	2017	45.6%	49.3%	41.4%
Colombia	Usiacurí	2000	77.4%	97.5%	60.8%
Colombia	Usiacurí	2017	43.2%	76.3%	16.9%
Colombia	Utica	2000	63.8%	100.0%	0.9%
Colombia	Utica	2017	43.6%	99.4%	0.1%
Colombia	Valdivia	2000	92.8%	99.3%	81.0%
Colombia	Valdivia	2017	74.1%	90.3%	51.5%
Colombia	Valencia	2000	37.6%	66.8%	13.5%
Colombia	Valencia	2017	13.8%	39.0%	1.2%
Colombia	Valle de San José	2000	80.7%	99.9%	11.2%
Colombia	Valle de San José	2017	54.3%	98.4%	1.5%
Colombia	Valle de San Juan	2000	59.7%	97.9%	11.3%
Colombia	Valle de San Juan	2017	33.1%	85.3%	2.2%
Colombia	Valle del Guamuez	2000	68.9%	86.1%	44.7%
Colombia	Valle del Guamuez	2017	42.9%	61.7%	14.8%
Colombia	Valledupar	2000	85.5%	91.3%	77.5%
Colombia	Valledupar	2017	60.4%	67.3%	52.6%
Colombia	Valparaíso	2000	44.8%	99.0%	0.7%
Colombia	Valparaíso	2000	58.7%	87.5%	28.5%
Colombia	Valparaíso	2017	24.5%	93.4%	0.1%
Colombia	Valparaíso	2017	31.7%	61.6%	6.8%
Colombia	Vegachí	2000	60.5%	100.0%	1.0%
Colombia	Vegachí	2017	46.3%	99.7%	0.1%

Country	Second Administrative Unit	Year	Mean	Upper	Lower
Colombia	Vélez	2000	89.0%	95.2%	77.4%
Colombia	Vélez	2017	62.4%	75.4%	44.7%
Colombia	Venadillo	2000	87.4%	99.5%	52.0%
Colombia	Venadillo	2017	61.7%	94.8%	11.6%
Colombia	Venecia	2000	71.7%	100.0%	0.0%
Colombia	Venecia	2000	96.1%	99.4%	86.3%
Colombia	Venecia	2017	61.3%	100.0%	0.0%
Colombia	Venecia	2017	79.5%	93.8%	36.7%
Colombia	Ventaquemada	2000	91.0%	95.8%	80.9%
Colombia	Ventaquemada	2017	58.8%	73.1%	33.6%
Colombia	Vergara	2000	83.8%	99.9%	26.3%
Colombia	Vergara	2017	57.4%	97.8%	4.1%
Colombia	Versalles	2000	65.7%	99.2%	27.3%
Colombia	Versalles	2017	36.9%	93.0%	9.3%
Colombia	Vetas	2000	66.2%	100.0%	0.0%
Colombia	Vetas	2017	54.7%	100.0%	0.0%
Colombia	Vianí	2000	12.5%	25.0%	4.7%
Colombia	Vianí	2017	3.2%	9.5%	1.2%
Colombia	Victoria	2000	67.5%	84.6%	50.3%
Colombia	Victoria	2017	56.3%	73.4%	31.2%
Colombia	Vigía del Fuerte	2000	60.1%	99.3%	8.6%
Colombia	Vigía del Fuerte	2017	46.7%	96.9%	1.8%
Colombia	Vijes	2000	84.5%	97.4%	67.4%
Colombia	Vijes	2017	64.6%	82.3%	49.6%
Colombia	Villa Caro	2000	77.4%	100.0%	4.2%
Colombia	Villa Caro	2017	63.6%	99.9%	1.1%
Colombia	Villa de Leyva	2000	67.8%	97.8%	13.1%
Colombia	Villa de Leyva	2017	34.0%	88.1%	1.6%
Colombia	Villa de San Diego de Ubaté	2000	81.1%	100.0%	12.5%
Colombia	Villa de San Diego de Ubaté	2017	56.3%	99.7%	1.6%
Colombia	Villa del Rosario	2000	95.7%	97.3%	91.8%
Colombia	Villa del Rosario	2017	70.3%	75.9%	63.6%
Colombia	Villagarzón	2000	81.5%	90.8%	68.7%
Colombia	Villagarzón	2017	35.4%	42.8%	29.4%
Colombia	Villagómez	2000	65.4%	100.0%	2.7%
Colombia	Villagómez	2017	43.2%	99.2%	0.1%
Colombia	Villahermosa	2000	77.2%	95.6%	51.7%
Colombia	Villahermosa	2017	44.3%	72.3%	16.6%
Colombia	Villamaría	2000	98.2%	99.8%	93.8%
Colombia	Villamaría	2017	85.9%	96.2%	70.7%
Colombia	Villanueva	2000	75.5%	94.6%	39.1%
Colombia	Villanueva	2000	70.3%	100.0%	1.7%
Colombia	Villanueva	2000	93.8%	99.5%	70.3%
Colombia	Villanueva	2000	89.5%	95.5%	52.5%
Colombia	Villanueva	2017	36.0%	71.4%	8.4%
Colombia	Villanueva	2017	56.4%	73.1%	18.4%
Colombia	Villanueva	2017	51.1%	100.0%	0.1%
Colombia	Villanueva	2017	72.7%	94.2%	20.4%
Colombia	Villapinzón	2000	63.4%	98.9%	16.2%
Colombia	Villapinzón	2017	35.5%	95.7%	3.0%
Colombia	Villarrica	2000	92.8%	100.0%	31.6%
Colombia	Villarrica	2017	88.1%	100.0%	22.4%
Colombia	Villavicencio	2000	73.2%	77.2%	69.0%
Colombia	Villavicencio	2017	20.0%	23.7%	17.0%

Piped Water Proportion by Second Administrative Unit *(continued)*

Country	Second Administrative Unit	Year	Mean	Upper	Lower
Colombia	Villavieja	2000	77.3%	99.6%	34.2%
Colombia	Villavieja	2017	59.9%	96.7%	17.8%
Colombia	Villeta	2000	59.6%	97.2%	14.2%
Colombia	Villeta	2017	22.2%	75.9%	3.2%
Colombia	Viotá	2000	84.9%	96.6%	63.2%
Colombia	Viotá	2017	39.2%	67.0%	18.7%
Colombia	Viracachá	2000	19.6%	90.6%	0.0%
Colombia	Viracachá	2017	8.2%	65.0%	0.0%
Colombia	Vista Hermosa	2000	67.0%	85.0%	44.9%
Colombia	Vista Hermosa	2017	33.5%	54.4%	16.6%
Colombia	Viterbo	2000	82.6%	89.5%	77.3%
Colombia	Viterbo	2017	36.6%	42.2%	31.5%
Colombia	Yacopí	2000	79.8%	94.5%	65.7%
Colombia	Yacopí	2017	56.3%	72.9%	40.5%
Colombia	Yacuanquer	2000	73.1%	99.9%	19.5%
Colombia	Yacuanquer	2017	55.2%	98.8%	7.0%
Colombia	Yaguará	2000	87.9%	100.0%	22.0%
Colombia	Yaguará	2017	75.3%	100.0%	5.8%
Colombia	Yalí	2000	59.7%	100.0%	1.6%
Colombia	Yalí	2017	45.2%	99.7%	0.1%
Colombia	Yarumal	2000	94.4%	98.7%	81.8%
Colombia	Yarumal	2017	70.8%	88.0%	43.1%
Colombia	Yavaraté	2000	32.3%	66.3%	5.9%
Colombia	Yavaraté	2017	19.5%	53.1%	0.9%
Colombia	Yolombó	2000	77.8%	96.4%	47.2%
Colombia	Yolombó	2017	51.7%	79.1%	22.0%
Colombia	Yondó	2000	55.8%	91.2%	21.6%
Colombia	Yondó	2017	37.8%	77.5%	8.2%
Colombia	Yopal	2000	67.4%	78.7%	57.2%
Colombia	Yopal	2017	30.7%	36.7%	24.3%
Colombia	Yotoco	2000	87.0%	100.0%	31.3%
Colombia	Yotoco	2017	73.7%	99.9%	13.4%
Colombia	Yumbo	2000	82.5%	88.5%	77.7%
Colombia	Yumbo	2017	55.2%	57.7%	52.3%
Colombia	Zambrano	2000	92.2%	99.9%	59.1%
Colombia	Zambrano	2017	79.3%	99.0%	33.9%
Colombia	Zapatoca	2000	76.4%	96.8%	5.7%
Colombia	Zapatoca	2017	64.1%	94.8%	2.3%
Colombia	Zaragoza	2000	76.8%	93.7%	50.0%
Colombia	Zaragoza	2017	45.6%	68.4%	24.7%
Colombia	Zarzal	2000	88.4%	98.7%	54.5%
Colombia	Zarzal	2017	74.3%	97.8%	32.3%
Colombia	Zetaquirá	2000	62.9%	98.6%	7.4%
Colombia	Zetaquirá	2017	40.4%	92.0%	1.6%
Colombia	Zipacón	2000	98.2%	99.8%	89.6%
Colombia	Zipacón	2017	82.1%	96.8%	31.9%
Colombia	Zipaquirá	2000	89.0%	96.2%	78.3%
Colombia	Zipaquirá	2017	53.9%	66.5%	43.2%
Dominican Republic	Altamira	2000	55.8%	94.1%	5.4%
Dominican Republic	Altamira	2017	22.7%	61.1%	0.2%
Dominican Republic	Arenoso	2000	47.5%	96.4%	2.1%
Dominican Republic	Arenoso	2017	16.9%	71.0%	0.0%
Dominican Republic	Azua de Compostela	2000	54.2%	92.7%	14.9%
Dominican Republic	Azua de Compostela	2017	6.7%	45.0%	0.2%

Country	Second Administrative Unit	Year	Mean	Upper	Lower
Dominican Republic	Bajos de Haina	2000	22.6%	66.0%	0.4%
Dominican Republic	Bajos de Haina	2017	7.7%	40.2%	0.0%
Dominican Republic	Baní	2000	58.3%	85.6%	27.4%
Dominican Republic	Baní	2017	14.6%	39.4%	3.1%
Dominican Republic	Banica	2000	52.2%	95.0%	11.8%
Dominican Republic	Banica	2017	23.4%	71.1%	0.5%
Dominican Republic	Bayaguana	2000	47.9%	83.9%	11.4%
Dominican Republic	Bayaguana	2017	16.4%	53.1%	1.6%
Dominican Republic	Boca Chica	2000	47.0%	97.7%	1.5%
Dominican Republic	Boca Chica	2017	15.1%	77.2%	0.0%
Dominican Republic	Bohechio	2000	55.0%	93.9%	12.2%
Dominican Republic	Bohechio	2017	25.3%	77.2%	1.3%
Dominican Republic	Bonao	2000	47.8%	80.3%	13.5%
Dominican Republic	Bonao	2017	8.1%	24.3%	0.4%
Dominican Republic	Cabral	2000	68.6%	99.8%	10.8%
Dominican Republic	Cabral	2017	34.0%	92.6%	1.7%
Dominican Republic	Cabrera	2000	46.5%	90.9%	3.1%
Dominican Republic	Cabrera	2017	17.2%	53.9%	0.0%
Dominican Republic	Cambita Garabito	2000	53.4%	95.3%	7.4%
Dominican Republic	Cambita Garabito	2017	18.7%	67.9%	0.4%
Dominican Republic	Castañuela	2000	55.5%	95.0%	13.9%
Dominican Republic	Castañuela	2017	17.4%	44.6%	10.4%
Dominican Republic	Castillo	2000	53.1%	98.7%	5.4%
Dominican Republic	Castillo	2017	20.2%	85.6%	0.1%
Dominican Republic	Cayetano Germosén	2000	46.6%	100.0%	0.1%
Dominican Republic	Cayetano Germosén	2017	22.0%	96.9%	0.0%
Dominican Republic	Cevicos	2000	49.4%	88.2%	10.2%
Dominican Republic	Cevicos	2017	19.3%	65.1%	0.6%
Dominican Republic	Comendador	2000	49.0%	79.9%	14.9%
Dominican Republic	Comendador	2017	12.1%	46.8%	0.3%
Dominican Republic	Concepción de la Vega	2000	40.0%	68.6%	15.0%

Piped Water Proportion by Second Administrative Unit *(continued)*

Country	Second Administrative Unit	Year	Mean	Upper	Lower
Dominican Republic	Concepción de la Vega	2017	9.6%	26.2%	0.6%
Dominican Republic	Constanza	2000	63.5%	93.5%	23.4%
Dominican Republic	Constanza	2017	27.2%	75.4%	2.5%
Dominican Republic	Consuelo	2000	48.2%	96.8%	1.4%
Dominican Republic	Consuelo	2017	17.9%	73.3%	0.0%
Dominican Republic	Cotuí	2000	44.6%	70.2%	15.5%
Dominican Republic	Cotuí	2017	15.4%	37.5%	3.1%
Dominican Republic	Cristobal	2000	57.9%	99.1%	5.9%
Dominican Republic	Cristobal	2017	25.2%	76.1%	0.1%
Dominican Republic	Dajabón	2000	53.3%	96.1%	5.7%
Dominican Republic	Dajabón	2017	19.6%	84.9%	0.0%
Dominican Republic	Distrito Nacional	2000	23.9%	69.4%	1.9%
Dominican Republic	Distrito Nacional	2017	3.9%	21.5%	0.0%
Dominican Republic	Duvergé	2000	57.0%	92.2%	14.8%
Dominican Republic	Duvergé	2017	23.7%	61.2%	0.9%
Dominican Republic	El Cercado	2000	55.6%	97.0%	10.1%
Dominican Republic	El Cercado	2017	25.3%	81.4%	0.3%
Dominican Republic	El Factor	2000	55.5%	98.0%	5.2%
Dominican Republic	El Factor	2017	22.5%	80.8%	0.1%
Dominican Republic	El Llano	2000	59.3%	98.1%	11.0%
Dominican Republic	El Llano	2017	19.8%	80.9%	0.1%
Dominican Republic	El Peñón	2000	56.2%	100.0%	0.7%
Dominican Republic	El Peñón	2017	24.1%	97.5%	0.0%
Dominican Republic	El Pino	2000	56.3%	94.6%	16.1%
Dominican Republic	El Pino	2017	10.5%	44.0%	0.1%
Dominican Republic	El Valle	2000	49.3%	97.8%	2.6%
Dominican Republic	El Valle	2017	18.1%	75.0%	0.0%
Dominican Republic	Enriquillo	2000	55.7%	97.2%	7.8%
Dominican Republic	Enriquillo	2017	22.6%	79.6%	0.6%
Dominican Republic	Esperalvillo	2000	37.5%	91.0%	0.6%
Dominican Republic	Esperalvillo	2017	9.0%	44.7%	0.0%

Country	Second Administrative Unit	Year	Mean	Upper	Lower
Dominican Republic	Esperanza	2000	9.7%	37.0%	0.3%
Dominican Republic	Esperanza	2017	1.9%	12.0%	0.0%
Dominican Republic	Estebania	2000	52.3%	94.6%	5.5%
Dominican Republic	Estebania	2017	19.6%	64.0%	0.2%
Dominican Republic	Fantino	2000	54.8%	99.7%	3.0%
Dominican Republic	Fantino	2017	22.1%	87.8%	0.1%
Dominican Republic	Fundación	2000	50.2%	99.8%	0.4%
Dominican Republic	Fundación	2017	21.6%	85.4%	0.0%
Dominican Republic	Galvan	2000	67.9%	93.8%	35.1%
Dominican Republic	Galvan	2017	21.4%	48.2%	4.0%
Dominican Republic	Gaspar Hernández	2000	49.7%	91.5%	7.4%
Dominican Republic	Gaspar Hernández	2017	18.4%	59.5%	0.2%
Dominican Republic	Guananico	2000	40.2%	82.0%	2.2%
Dominican Republic	Guananico	2017	9.5%	45.4%	0.0%
Dominican Republic	Guayabal	2000	54.7%	94.6%	10.8%
Dominican Republic	Guayabal	2017	21.9%	67.3%	0.2%
Dominican Republic	Guayacanes	2000	60.0%	99.0%	7.5%
Dominican Republic	Guayacanes	2017	23.1%	80.6%	0.1%
Dominican Republic	Guaymate	2000	54.5%	91.6%	10.6%
Dominican Republic	Guaymate	2017	20.8%	60.4%	0.6%
Dominican Republic	Guayubín	2000	56.6%	88.8%	22.4%
Dominican Republic	Guayubín	2017	35.0%	71.3%	9.5%
Dominican Republic	Guerra	2000	46.6%	92.7%	4.0%
Dominican Republic	Guerra	2017	17.4%	71.2%	0.1%
Dominican Republic	Hato Mayor del Rey	2000	64.2%	92.0%	36.3%
Dominican Republic	Hato Mayor del Rey	2017	45.2%	72.8%	26.5%
Dominican Republic	Hondo Valle	2000	59.0%	99.5%	1.9%
Dominican Republic	Hondo Valle	2017	25.4%	93.0%	0.0%
Dominican Republic	Hostos	2000	49.6%	98.1%	2.1%
Dominican Republic	Hostos	2017	19.0%	76.5%	0.0%
Dominican Republic	Imbert	2000	57.3%	97.3%	10.4%

Country	Second Administrative Unit	Year	Mean	Upper	Lower
Dominican Republic	Imbert	2017	20.2%	70.9%	0.7%
Dominican Republic	Jamao al Norte	2000	52.2%	94.9%	5.0%
Dominican Republic	Jamao al Norte	2017	19.3%	74.3%	0.0%
Dominican Republic	Janico	2000	54.0%	89.3%	17.5%
Dominican Republic	Janico	2017	23.0%	63.6%	1.3%
Dominican Republic	Jaquimeyes	2000	54.3%	97.2%	8.9%
Dominican Republic	Jaquimeyes	2017	23.3%	81.3%	0.2%
Dominican Republic	Jarabacoa	2000	56.0%	90.1%	14.1%
Dominican Republic	Jarabacoa	2017	23.1%	72.8%	1.7%
Dominican Republic	Jima Abajo	2000	43.4%	97.2%	1.0%
Dominican Republic	Jima Abajo	2017	15.3%	80.0%	0.0%
Dominican Republic	Jimaní	2000	55.8%	93.4%	18.0%
Dominican Republic	Jimaní	2017	24.2%	69.1%	1.4%
Dominican Republic	Juan de Herrera	2000	66.7%	99.5%	20.0%
Dominican Republic	Juan de Herrera	2017	17.4%	64.3%	1.2%
Dominican Republic	Juan Santiago	2000	56.0%	98.5%	4.5%
Dominican Republic	Juan Santiago	2017	21.7%	74.3%	0.1%
Dominican Republic	La Cienaga	2000	59.6%	97.9%	8.1%
Dominican Republic	La Cienaga	2017	25.9%	79.0%	0.1%
Dominican Republic	La Descubierta	2000	57.7%	97.0%	9.9%
Dominican Republic	La Descubierta	2017	22.1%	83.6%	0.2%
Dominican Republic	La Isabela	2000	49.0%	96.7%	5.7%
Dominican Republic	La Isabela	2017	20.1%	75.4%	0.1%
Dominican Republic	La Laguna de Nisibón	2000	52.1%	90.8%	8.4%
Dominican Republic	La Laguna de Nisibón	2017	20.8%	66.4%	0.2%
Dominican Republic	La Mata	2000	88.3%	98.8%	70.7%
Dominican Republic	La Mata	2017	69.2%	91.3%	34.6%
Dominican Republic	La Romana	2000	36.7%	82.3%	6.5%
Dominican Republic	La Romana	2017	6.9%	33.5%	0.1%
Dominican Republic	Laguna Salada	2000	36.6%	87.4%	3.6%
Dominican Republic	Laguna Salada	2017	8.8%	58.0%	0.0%

Piped Water Proportion by Second Administrative Unit *(continued)*

Country	Second Administrative Unit	Year	Mean	Upper	Lower
Dominican Republic	Las Charcas	2000	51.7%	94.9%	9.8%
Dominican Republic	Las Charcas	2017	19.1%	60.1%	0.1%
Dominican Republic	Las Guaranas	2000	63.2%	99.8%	3.2%
Dominican Republic	Las Guaranas	2017	26.9%	91.1%	0.0%
Dominican Republic	Las Matas de Farfan	2000	61.7%	90.2%	25.8%
Dominican Republic	Las Matas de Farfan	2017	21.6%	64.8%	2.3%
Dominican Republic	Las Matas de Santa Cruz	2000	82.3%	99.7%	23.0%
Dominican Republic	Las Matas de Santa Cruz	2017	76.3%	95.7%	40.0%
Dominican Republic	Las Salinas	2000	57.2%	99.9%	1.5%
Dominican Republic	Las Salinas	2017	25.7%	89.8%	0.0%
Dominican Republic	Las Terrenas	2000	63.3%	98.8%	8.9%
Dominican Republic	Las Terrenas	2017	29.0%	83.7%	2.1%
Dominican Republic	Las Yayas de Viajama	2000	51.9%	89.4%	10.5%
Dominican Republic	Las Yayas de Viajama	2017	17.1%	48.0%	0.7%
Dominican Republic	Licey al Medio	2000	41.4%	95.6%	0.7%
Dominican Republic	Licey al Medio	2017	6.8%	52.4%	0.0%
Dominican Republic	Loma de Cabrera	2000	48.2%	80.1%	16.1%
Dominican Republic	Loma de Cabrera	2017	11.2%	37.5%	0.2%
Dominican Republic	Los Alcarrizos	2000	41.1%	98.3%	0.2%
Dominican Republic	Los Alcarrizos	2017	13.2%	79.2%	0.0%
Dominican Republic	Los Almácigos	2000	50.1%	95.7%	4.0%
Dominican Republic	Los Almácigos	2017	15.5%	70.6%	0.0%
Dominican Republic	Los Cacaos	2000	56.7%	96.2%	11.0%
Dominican Republic	Los Cacaos	2017	25.4%	75.7%	0.3%
Dominican Republic	Los Hidalgos	2000	28.8%	93.2%	0.0%
Dominican Republic	Los Hidalgos	2017	8.8%	57.9%	0.0%
Dominican Republic	Los Llanos	2000	54.9%	83.6%	19.6%
Dominican Republic	Los Llanos	2017	24.8%	55.4%	3.2%
Dominican Republic	Los Rios	2000	56.9%	97.5%	7.2%
Dominican Republic	Los Rios	2017	24.7%	84.1%	0.2%
Dominican Republic	Luperon	2000	47.8%	91.4%	3.9%

Country	Second Administrative Unit	Year	Mean	Upper	Lower
Dominican Republic	Luperon	2017	17.2%	58.2%	0.3%
Dominican Republic	Maimón	2000	16.9%	70.2%	0.0%
Dominican Republic	Maimón	2017	4.1%	38.9%	0.0%
Dominican Republic	Mao	2000	55.8%	86.3%	16.2%
Dominican Republic	Mao	2017	10.5%	33.4%	0.4%
Dominican Republic	Mella	2000	57.8%	94.7%	15.3%
Dominican Republic	Mella	2017	24.8%	68.7%	0.6%
Dominican Republic	Miches	2000	46.7%	91.2%	8.0%
Dominican Republic	Miches	2017	15.0%	58.9%	0.4%
Dominican Republic	Moca	2000	59.7%	89.9%	27.5%
Dominican Republic	Moca	2017	13.1%	45.2%	0.4%
Dominican Republic	Monción	2000	50.4%	98.4%	3.1%
Dominican Republic	Monción	2017	18.2%	80.1%	0.1%
Dominican Republic	Monte Plata	2000	27.6%	53.2%	9.0%
Dominican Republic	Monte Plata	2017	7.5%	20.4%	0.4%
Dominican Republic	Montellano	2000	48.0%	96.8%	2.1%
Dominican Republic	Montellano	2017	16.6%	80.1%	0.0%
Dominican Republic	Nagua	2000	58.3%	85.9%	15.4%
Dominican Republic	Nagua	2017	20.7%	59.4%	0.8%
Dominican Republic	Neyba	2000	82.0%	99.8%	43.2%
Dominican Republic	Neyba	2017	45.4%	90.9%	4.2%
Dominican Republic	Nigua	2000	58.0%	99.7%	2.1%
Dominican Republic	Nigua	2017	20.7%	89.1%	0.0%
Dominican Republic	Nizao	2000	50.4%	99.5%	0.1%
Dominican Republic	Nizao	2017	18.1%	87.9%	0.0%
Dominican Republic	Padre Las Casas	2000	56.1%	91.6%	14.4%
Dominican Republic	Padre Las Casas	2017	23.0%	61.6%	1.2%
Dominican Republic	Paraiso	2000	56.6%	99.9%	4.3%
Dominican Republic	Paraiso	2017	24.1%	93.6%	0.1%
Dominican Republic	Partido	2000	53.6%	96.8%	6.3%
Dominican Republic	Partido	2017	15.8%	77.1%	0.0%

Country	Second Administrative Unit	Year	Mean	Upper	Lower
Dominican Republic	Pedernales	2000	57.3%	82.4%	27.8%
Dominican Republic	Pedernales	2017	23.2%	52.7%	4.6%
Dominican Republic	Pedro Brand	2000	48.0%	91.3%	5.5%
Dominican Republic	Pedro Brand	2017	19.3%	67.8%	0.1%
Dominican Republic	Pedro Santana	2000	54.7%	85.0%	18.6%
Dominican Republic	Pedro Santana	2017	22.2%	60.2%	1.2%
Dominican Republic	Pepillo Salcedo	2000	51.5%	96.7%	2.3%
Dominican Republic	Pepillo Salcedo	2017	18.8%	72.9%	0.0%
Dominican Republic	Peralta	2000	51.7%	94.4%	6.6%
Dominican Republic	Peralta	2017	16.4%	71.2%	0.0%
Dominican Republic	Piedra Blanca	2000	27.8%	71.1%	3.0%
Dominican Republic	Piedra Blanca	2017	9.7%	41.3%	0.0%
Dominican Republic	Pimentel	2000	60.8%	97.5%	7.6%
Dominican Republic	Pimentel	2017	24.2%	85.1%	0.1%
Dominican Republic	Polo	2000	63.2%	99.0%	10.5%
Dominican Republic	Polo	2017	30.5%	86.8%	1.3%
Dominican Republic	Postrer Rio	2000	57.2%	98.2%	11.5%
Dominican Republic	Postrer Rio	2017	23.4%	81.0%	0.1%
Dominican Republic	Pueblo Viejo	2000	57.6%	93.7%	15.9%
Dominican Republic	Pueblo Viejo	2017	16.8%	56.4%	0.3%
Dominican Republic	Puñal	2000	40.3%	99.1%	0.2%
Dominican Republic	Puñal	2017	9.5%	71.9%	0.0%
Dominican Republic	Quisquella	2000	63.1%	94.5%	15.2%
Dominican Republic	Quisquella	2017	21.8%	67.8%	0.8%
Dominican Republic	Ramón Santana	2000	52.1%	95.1%	10.4%
Dominican Republic	Ramón Santana	2017	19.7%	66.2%	0.1%
Dominican Republic	Rancho Arriba	2000	71.8%	99.2%	17.8%
Dominican Republic	Rancho Arriba	2017	36.5%	87.7%	2.7%
Dominican Republic	Restauración	2000	52.7%	94.5%	5.8%
Dominican Republic	Restauración	2017	19.6%	67.8%	0.1%
Dominican Republic	Rio San Juan	2000	46.2%	89.4%	5.3%

Country	Second Administrative Unit	Year	Mean	Upper	Lower
Dominican Republic	Rio San Juan	2017	16.4%	61.3%	0.2%
Dominican Republic	Sabana de la Mar	2000	46.1%	87.3%	10.8%
Dominican Republic	Sabana de la Mar	2017	17.0%	70.9%	0.6%
Dominican Republic	Sabana Grande de Boyá	2000	46.4%	90.6%	9.0%
Dominican Republic	Sabana Grande de Boyá	2017	15.9%	56.3%	0.6%
Dominican Republic	Sabana Grande de Palenque	2000	49.6%	100.0%	0.0%
Dominican Republic	Sabana Grande de Palenque	2017	21.6%	91.3%	0.0%
Dominican Republic	Sabana Iglesia	2000	49.1%	99.3%	1.2%
Dominican Republic	Sabana Iglesia	2017	23.9%	88.0%	0.0%
Dominican Republic	Sabana Larga	2000	70.5%	99.2%	30.9%
Dominican Republic	Sabana Larga	2017	29.6%	79.5%	8.4%
Dominican Republic	Sabana Yegua	2000	59.2%	99.3%	1.2%
Dominican Republic	Sabana Yegua	2017	18.7%	93.7%	0.0%
Dominican Republic	Salcedo	2000	64.1%	90.0%	34.8%
Dominican Republic	Salcedo	2017	42.0%	68.0%	4.2%
Dominican Republic	Salvaleón de Higüey	2000	48.1%	69.6%	25.2%
Dominican Republic	Salvaleón de Higüey	2017	9.9%	24.8%	2.4%
Dominican Republic	San Cristóbal	2000	48.3%	75.6%	12.6%
Dominican Republic	San Cristóbal	2017	12.2%	44.5%	0.3%
Dominican Republic	San Felipe de Puerto Plata	2000	50.1%	92.0%	7.8%
Dominican Republic	San Felipe de Puerto Plata	2017	15.3%	64.4%	0.6%
Dominican Republic	San Fernando de Monte Cristi	2000	56.3%	88.6%	20.7%
Dominican Republic	San Fernando de Monte Cristi	2017	23.1%	60.9%	1.9%
Dominican Republic	San Francisco de Macorís	2000	50.8%	90.6%	10.7%
Dominican Republic	San Francisco de Macorís	2017	13.1%	48.0%	0.7%
Dominican Republic	San Gregorio de Yaguate	2000	54.2%	98.3%	1.7%
Dominican Republic	San Gregorio de Yaguate	2017	20.5%	76.4%	0.0%
Dominican Republic	San Ignacio de Sabaneta	2000	51.2%	85.4%	22.2%

Country	Second Administrative Unit	Year	Mean	Upper	Lower
Dominican Republic	San Ignacio de Sabaneta	2017	15.2%	48.7%	2.2%
Dominican Republic	San José de Las Matas	2000	54.7%	81.5%	25.8%
Dominican Republic	San José de Las Matas	2017	19.8%	52.1%	4.0%
Dominican Republic	San José de Ocoa	2000	61.9%	94.3%	23.3%
Dominican Republic	San José de Ocoa	2017	29.2%	72.6%	7.3%
Dominican Republic	San Juan de la Maguana	2000	62.8%	91.1%	27.7%
Dominican Republic	San Juan de la Maguana	2017	19.0%	49.3%	4.8%
Dominican Republic	San Pedro de Macorís	2000	46.3%	92.1%	5.1%
Dominican Republic	San Pedro de Macorís	2017	11.6%	67.4%	0.1%
Dominican Republic	San Rafael del Yuma	2000	49.2%	81.4%	20.1%
Dominican Republic	San Rafael del Yuma	2017	19.2%	43.7%	2.2%
Dominican Republic	Sánchez	2000	44.9%	85.8%	9.4%
Dominican Republic	Sánchez	2017	16.0%	53.3%	1.5%
Dominican Republic	Santa Bárbara de Samaná	2000	46.2%	87.7%	11.2%
Dominican Republic	Santa Bárbara de Samaná	2017	15.9%	50.2%	0.4%
Dominican Republic	Santa Cruz de Barahona	2000	37.8%	95.5%	5.1%
Dominican Republic	Santa Cruz de Barahona	2017	10.6%	48.3%	0.7%
Dominican Republic	Santa Cruz del Seybo	2000	66.5%	84.6%	45.7%
Dominican Republic	Santa Cruz del Seybo	2017	41.7%	61.0%	21.5%
Dominican Republic	Santiago de los Caballeros	2000	42.4%	65.2%	18.5%
Dominican Republic	Santiago de los Caballeros	2017	5.5%	17.5%	0.3%
Dominican Republic	Santo Domingo Este	2000	11.4%	42.1%	0.1%
Dominican Republic	Santo Domingo Este	2017	3.5%	25.1%	0.0%
Dominican Republic	Santo Domingo Norte	2000	29.9%	59.3%	3.0%
Dominican Republic	Santo Domingo Norte	2017	9.0%	40.9%	0.1%
Dominican Republic	Santo Domingo Oeste	2000	33.3%	90.3%	0.2%
Dominican Republic	Santo Domingo Oeste	2017	6.7%	46.2%	0.0%
Dominican Republic	Sosua	2000	50.5%	91.3%	9.8%

Country	Second Administrative Unit	Year	Mean	Upper	Lower
Dominican Republic	Sosua	2017	18.3%	63.6%	0.4%
Dominican Republic	Tamayo	2000	55.4%	96.1%	8.4%
Dominican Republic	Tamayo	2017	23.8%	72.7%	1.4%
Dominican Republic	Tamboril	2000	48.4%	99.2%	0.3%
Dominican Republic	Tamboril	2017	18.6%	88.2%	0.0%
Dominican Republic	Tenares	2000	66.0%	98.9%	31.0%
Dominican Republic	Tenares	2017	41.8%	75.6%	3.3%
Dominican Republic	Vallejuelo	2000	57.3%	98.9%	9.0%
Dominican Republic	Vallejuelo	2017	25.1%	83.4%	0.6%
Dominican Republic	Vicente Noble	2000	55.4%	96.8%	6.8%
Dominican Republic	Vicente Noble	2017	24.2%	75.1%	0.5%
Dominican Republic	Villa Altagracia	2000	48.7%	91.7%	7.9%
Dominican Republic	Villa Altagracia	2017	20.0%	66.1%	0.5%
Dominican Republic	Villa Bisonó	2000	42.8%	98.1%	0.8%
Dominican Republic	Villa Bisonó	2017	17.6%	84.9%	0.0%
Dominican Republic	Villa Gonzalez	2000	48.3%	93.6%	5.2%
Dominican Republic	Villa Gonzalez	2017	16.0%	72.4%	0.0%
Dominican Republic	Villa Hermosa	2000	42.3%	88.9%	2.1%
Dominican Republic	Villa Hermosa	2017	9.7%	59.3%	0.0%
Dominican Republic	Villa Jaragua	2000	65.0%	99.4%	12.7%
Dominican Republic	Villa Jaragua	2017	33.7%	89.9%	1.3%
Dominican Republic	Villa Rivas	2000	48.8%	92.8%	10.8%
Dominican Republic	Villa Rivas	2017	18.7%	64.9%	0.2%
Dominican Republic	Villa Tabara Arriba	2000	52.8%	91.9%	10.1%
Dominican Republic	Villa Tabara Arriba	2017	18.6%	68.5%	0.4%
Dominican Republic	Villa Tapia	2000	77.0%	99.9%	34.7%
Dominican Republic	Villa Tapia	2017	57.0%	95.8%	3.4%
Dominican Republic	Villa Vázquez	2000	52.7%	93.4%	7.0%
Dominican Republic	Villa Vázquez	2017	25.4%	77.5%	2.0%
Dominican Republic	Yamasá	2000	27.3%	58.2%	6.0%
Dominican Republic	Yamasá	2017	6.3%	22.4%	0.1%

Country	Second Administrative Unit	Year	Mean	Upper	Lower
Ecuador	24 De Mayo	2000	48.8%	96.8%	1.8%
Ecuador	24 De Mayo	2017	48.4%	96.8%	1.7%
Ecuador	Aguarico	2000	54.7%	99.6%	0.6%
Ecuador	Aguarico	2017	54.6%	99.7%	0.4%
Ecuador	Alausí	2000	89.8%	98.0%	76.4%
Ecuador	Alausí	2017	90.2%	97.8%	78.4%
Ecuador	Alfredo Baquerizo Moreno	2000	67.2%	95.3%	23.6%
Ecuador	Alfredo Baquerizo Moreno	2017	66.7%	96.1%	20.8%
Ecuador	Ambato	2000	94.7%	96.1%	91.8%
Ecuador	Ambato	2017	94.8%	96.1%	92.0%
Ecuador	Antonio Ante	2000	65.2%	100.0%	0.1%
Ecuador	Antonio Ante	2017	65.2%	100.0%	0.1%
Ecuador	Arajuno	2000	60.9%	98.0%	16.3%
Ecuador	Arajuno	2017	60.8%	98.4%	14.3%
Ecuador	Archidona	2000	68.4%	91.0%	38.9%
Ecuador	Archidona	2017	69.4%	90.2%	38.2%
Ecuador	Arenillas	2000	86.5%	97.9%	65.3%
Ecuador	Arenillas	2017	87.8%	97.6%	69.1%
Ecuador	Atacames	2000	66.2%	84.2%	42.4%
Ecuador	Atacames	2017	69.4%	83.6%	53.2%
Ecuador	Atahualpa	2000	96.2%	99.5%	81.8%
Ecuador	Atahualpa	2017	96.3%	99.4%	83.0%
Ecuador	Azogues	2000	96.3%	98.0%	94.1%
Ecuador	Azogues	2017	95.9%	97.8%	93.5%
Ecuador	Baba	2000	51.8%	69.8%	33.4%
Ecuador	Baba	2017	50.1%	67.3%	33.2%
Ecuador	Babahoyo	2000	81.8%	92.8%	60.8%
Ecuador	Babahoyo	2017	82.3%	92.4%	62.8%
Ecuador	Balao	2000	93.3%	99.5%	77.1%
Ecuador	Balao	2017	93.2%	99.5%	78.6%
Ecuador	Balsas	2000	96.0%	99.2%	86.8%
Ecuador	Balsas	2017	96.3%	99.2%	89.0%
Ecuador	Balzar	2000	76.9%	96.9%	47.0%
Ecuador	Balzar	2017	77.2%	97.0%	50.2%
Ecuador	Baños de Agua Santa	2000	95.3%	99.2%	84.5%
Ecuador	Baños de Agua Santa	2017	95.2%	99.1%	84.8%
Ecuador	Biblián	2000	98.1%	99.1%	96.2%
Ecuador	Biblián	2017	98.0%	99.0%	96.1%
Ecuador	Bolívar	2000	80.5%	91.1%	64.1%
Ecuador	Bolívar	2000	27.7%	57.2%	5.8%
Ecuador	Bolívar	2017	80.3%	91.1%	64.4%
Ecuador	Bolívar	2017	26.5%	55.6%	4.9%
Ecuador	Buena Fé	2000	66.2%	91.7%	38.4%
Ecuador	Buena Fé	2017	66.2%	92.1%	38.8%
Ecuador	Caluma	2000	89.0%	97.7%	72.4%
Ecuador	Caluma	2017	87.7%	97.7%	67.2%
Ecuador	Calvas	2000	86.8%	99.0%	63.0%
Ecuador	Calvas	2017	86.0%	99.0%	61.1%
Ecuador	Camilo Ponce Enriquez	2000	82.0%	100.0%	17.1%
Ecuador	Camilo Ponce Enriquez	2017	82.1%	100.0%	17.5%
Ecuador	Cañar	2000	89.0%	96.8%	74.8%
Ecuador	Cañar	2017	88.9%	96.8%	74.7%
Ecuador	Carlos Julio Arosemena Tola	2000	79.4%	100.0%	18.9%

Country	Second Administrative Unit	Year	Mean	Upper	Lower
Ecuador	Carlos Julio Arosemena Tola	2017	81.0%	100.0%	14.6%
Ecuador	Cascales	2000	65.4%	99.1%	16.8%
Ecuador	Cascales	2017	67.5%	98.7%	23.9%
Ecuador	Catamayo	2000	94.4%	98.8%	84.9%
Ecuador	Catamayo	2017	94.4%	98.7%	85.1%
Ecuador	Cayambe	2000	86.7%	99.5%	36.3%
Ecuador	Cayambe	2017	86.5%	99.4%	37.0%
Ecuador	Celica	2000	70.0%	100.0%	3.3%
Ecuador	Celica	2017	69.6%	100.0%	3.1%
Ecuador	Centinela del Cóndor	2000	87.7%	99.9%	38.1%
Ecuador	Centinela del Cóndor	2017	86.6%	99.9%	31.8%
Ecuador	Cevallos	2000	84.6%	89.4%	77.8%
Ecuador	Cevallos	2017	84.1%	89.0%	77.1%
Ecuador	Chaguarpamba	2000	94.9%	99.4%	74.8%
Ecuador	Chaguarpamba	2017	94.4%	99.4%	72.3%
Ecuador	Chambo	2000	64.7%	97.9%	16.3%
Ecuador	Chambo	2017	64.6%	97.7%	16.4%
Ecuador	Chilla	2000	82.0%	100.0%	26.8%
Ecuador	Chilla	2017	81.2%	100.0%	24.5%
Ecuador	Chillanes	2000	81.0%	95.3%	54.7%
Ecuador	Chillanes	2017	80.8%	95.0%	54.4%
Ecuador	Chimbo	2000	89.5%	93.9%	83.2%
Ecuador	Chimbo	2017	88.8%	93.7%	81.4%
Ecuador	Chinchipe	2000	73.4%	99.9%	15.2%
Ecuador	Chinchipe	2017	73.7%	99.9%	13.0%
Ecuador	Chone	2000	62.1%	88.8%	25.2%
Ecuador	Chone	2017	61.9%	88.3%	26.0%
Ecuador	Chordeleg	2000	84.7%	99.9%	31.9%
Ecuador	Chordeleg	2017	80.3%	99.9%	28.2%
Ecuador	Chunchi	2000	89.1%	99.5%	46.0%
Ecuador	Chunchi	2017	88.9%	99.5%	45.9%
Ecuador	Colimes	2000	52.6%	99.5%	1.2%
Ecuador	Colimes	2017	52.8%	99.3%	1.4%
Ecuador	Colta	2000	97.5%	99.6%	90.9%
Ecuador	Colta	2017	97.4%	99.6%	90.3%
Ecuador	Coronel Marcelino Maridueña	2000	85.0%	99.8%	47.8%
Ecuador	Coronel Marcelino Maridueña	2017	86.1%	99.8%	49.7%
Ecuador	Cotacachi	2000	64.7%	99.8%	5.9%
Ecuador	Cotacachi	2017	64.3%	99.8%	6.5%
Ecuador	Cuenca	2000	76.8%	98.6%	20.5%
Ecuador	Cuenca	2017	76.2%	98.5%	19.8%
Ecuador	Cumanda	2000	89.5%	99.6%	62.2%
Ecuador	Cumanda	2017	89.7%	99.5%	62.5%
Ecuador	Cuyabeno	2000	58.5%	99.9%	1.9%
Ecuador	Cuyabeno	2017	58.3%	99.9%	1.7%
Ecuador	Daule	2000	52.1%	68.1%	34.4%
Ecuador	Daule	2017	50.0%	67.0%	31.6%
Ecuador	Déleg	2000	97.1%	98.8%	92.9%
Ecuador	Déleg	2017	97.1%	98.8%	92.6%
Ecuador	Durán	2000	94.1%	99.0%	82.0%
Ecuador	Durán	2017	94.7%	99.1%	84.2%
Ecuador	Echeandía	2000	88.1%	97.5%	67.5%
Ecuador	Echeandía	2017	86.5%	97.7%	59.1%
Ecuador	El Carmen	2000	51.7%	78.6%	24.8%

Country	Second Administrative Unit	Year	Mean	Upper	Lower
Ecuador	El Carmen	2017	51.4%	78.2%	25.5%
Ecuador	El Chaco	2000	85.4%	97.7%	64.2%
Ecuador	El Chaco	2017	87.5%	98.3%	67.5%
Ecuador	El Empalme	2000	61.1%	88.7%	25.6%
Ecuador	El Empalme	2017	60.4%	87.6%	26.8%
Ecuador	El Guabo	2000	92.2%	98.0%	80.7%
Ecuador	El Guabo	2017	92.1%	97.9%	81.2%
Ecuador	El Pan	2000	82.7%	100.0%	24.3%
Ecuador	El Pan	2017	82.5%	100.0%	26.4%
Ecuador	El Pangui	2000	52.4%	99.9%	0.2%
Ecuador	El Pangui	2017	52.1%	99.8%	0.2%
Ecuador	El Tambo	2000	97.4%	98.5%	95.8%
Ecuador	El Tambo	2017	97.3%	98.4%	95.9%
Ecuador	El Triunfo	2000	83.7%	98.8%	48.4%
Ecuador	El Triunfo	2017	84.1%	98.9%	47.2%
Ecuador	Eloy Alfaro	2000	52.8%	75.4%	23.3%
Ecuador	Eloy Alfaro	2017	52.8%	75.7%	23.5%
Ecuador	Esmeraldas	2000	78.3%	94.1%	54.2%
Ecuador	Esmeraldas	2017	78.2%	93.6%	54.9%
Ecuador	Espejo	2000	95.7%	98.9%	88.4%
Ecuador	Espejo	2017	96.1%	98.9%	90.2%
Ecuador	Espíndola	2000	65.6%	100.0%	0.1%
Ecuador	Espíndola	2017	65.4%	100.0%	0.1%
Ecuador	Flavio Alfaro	2000	36.1%	68.3%	5.6%
Ecuador	Flavio Alfaro	2017	35.3%	66.2%	5.3%
Ecuador	General Antonio Elizalde	2000	85.8%	99.9%	37.2%
Ecuador	General Antonio Elizalde	2017	87.5%	99.8%	46.1%
Ecuador	Girón	2000	57.2%	99.0%	4.2%
Ecuador	Girón	2017	56.3%	99.0%	3.8%
Ecuador	Gonzalo Pizarro	2000	65.3%	99.8%	7.1%
Ecuador	Gonzalo Pizarro	2017	65.3%	99.8%	5.9%
Ecuador	Gonzanamá	2000	84.3%	98.8%	52.5%
Ecuador	Gonzanamá	2017	85.1%	98.9%	54.3%
Ecuador	Guachapala	2000	96.7%	99.5%	88.2%
Ecuador	Guachapala	2017	96.9%	99.5%	88.8%
Ecuador	Gualaceo	2000	71.6%	97.7%	23.7%
Ecuador	Gualaceo	2017	72.3%	97.8%	25.1%
Ecuador	Gualaquiza	2000	78.8%	94.4%	60.7%
Ecuador	Gualaquiza	2017	79.8%	94.3%	63.1%
Ecuador	Guamote	2000	81.4%	97.8%	56.8%
Ecuador	Guamote	2017	81.9%	97.8%	59.1%
Ecuador	Guano	2000	97.2%	98.3%	94.5%
Ecuador	Guano	2017	97.1%	98.2%	94.1%
Ecuador	Guaranda	2000	92.2%	97.8%	80.5%
Ecuador	Guaranda	2017	91.9%	97.7%	80.6%
Ecuador	Guayaquil	2000	81.0%	94.3%	60.0%
Ecuador	Guayaquil	2017	81.8%	93.7%	64.2%
Ecuador	Huamboya	2000	68.5%	100.0%	0.1%
Ecuador	Huamboya	2017	68.5%	100.0%	0.1%
Ecuador	Huaquillas	2000	96.5%	99.2%	88.8%
Ecuador	Huaquillas	2017	96.8%	99.2%	90.8%
Ecuador	Ibarra	2000	63.6%	99.3%	5.5%
Ecuador	Ibarra	2017	63.2%	99.2%	4.9%
Ecuador	Isabela	2000	49.0%	100.0%	0.0%
Ecuador	Isabela	2017	49.0%	100.0%	0.0%
Ecuador	Isidro Ayora	2000	68.2%	96.7%	23.5%
Ecuador	Isidro Ayora	2017	68.4%	96.7%	24.6%
Ecuador	Jama	2000	40.9%	99.7%	0.1%

Country	Second Administrative Unit	Year	Mean	Upper	Lower
Ecuador	Jama	2017	40.8%	99.7%	0.1%
Ecuador	Jaramijó	2000	58.4%	99.2%	6.2%
Ecuador	Jaramijó	2017	60.7%	99.3%	6.2%
Ecuador	Jipijapa	2000	51.8%	79.9%	27.4%
Ecuador	Jipijapa	2017	50.5%	78.7%	27.4%
Ecuador	Junín	2000	54.9%	74.2%	29.6%
Ecuador	Junín	2017	56.7%	75.9%	32.8%
Ecuador	La Concordia	2000	65.6%	100.0%	0.4%
Ecuador	La Concordia	2017	65.0%	100.0%	0.4%
Ecuador	La Joya de los Sachas	2000	60.4%	98.3%	7.1%
Ecuador	La Joya de los Sachas	2017	59.9%	98.2%	6.6%
Ecuador	La Libertad	2000	79.9%	99.9%	17.2%
Ecuador	La Libertad	2017	86.0%	99.9%	27.5%
Ecuador	La Maná	2000	86.2%	96.7%	72.3%
Ecuador	La Maná	2017	85.9%	96.5%	72.0%
Ecuador	La Troncal	2000	92.8%	96.1%	86.2%
Ecuador	La Troncal	2017	92.7%	96.1%	85.1%
Ecuador	Lago Agrio	2000	56.1%	82.8%	31.7%
Ecuador	Lago Agrio	2017	55.3%	81.8%	31.3%
Ecuador	Las Lajas	2000	77.3%	96.5%	46.3%
Ecuador	Las Lajas	2017	77.3%	96.4%	46.3%
Ecuador	Las Naves	2000	80.1%	86.5%	73.5%
Ecuador	Las Naves	2017	77.3%	84.6%	70.1%
Ecuador	Latacunga	2000	97.6%	98.8%	95.8%
Ecuador	Latacunga	2017	97.5%	98.7%	95.6%
Ecuador	Limón Indanza	2000	71.2%	100.0%	7.5%
Ecuador	Limón Indanza	2017	70.3%	100.0%	5.3%
Ecuador	Logroño	2000	60.3%	99.9%	4.7%
Ecuador	Logroño	2017	60.5%	99.9%	3.9%
Ecuador	Loja	2000	93.0%	99.3%	80.5%
Ecuador	Loja	2017	92.9%	99.3%	80.8%
Ecuador	Lomas de Sargentillo	2000	81.0%	93.3%	63.0%
Ecuador	Lomas de Sargentillo	2017	80.7%	93.2%	62.5%
Ecuador	Loreto	2000	51.9%	94.9%	3.2%
Ecuador	Loreto	2017	51.6%	94.4%	3.1%
Ecuador	Macará	2000	68.1%	100.0%	5.4%
Ecuador	Macará	2017	68.2%	100.0%	5.7%
Ecuador	Machala	2000	89.4%	97.6%	67.7%
Ecuador	Machala	2017	89.6%	97.3%	69.3%
Ecuador	Manta	2000	71.0%	95.7%	35.5%
Ecuador	Manta	2017	70.8%	95.9%	35.3%
Ecuador	Marcabelí	2000	83.3%	99.8%	36.2%
Ecuador	Marcabelí	2017	84.5%	99.8%	36.4%
Ecuador	Mejía	2000	88.7%	99.9%	39.5%
Ecuador	Mejía	2000	94.1%	99.0%	81.3%
Ecuador	Mejía	2017	87.8%	99.9%	38.6%
Ecuador	Mejía	2017	94.4%	99.1%	81.2%
Ecuador	Mera	2000	95.1%	99.6%	76.3%
Ecuador	Mera	2017	94.8%	99.6%	73.7%
Ecuador	Milagro	2000	87.8%	98.3%	70.6%
Ecuador	Milagro	2017	88.2%	98.3%	71.7%
Ecuador	Mira	2000	87.7%	98.8%	63.8%
Ecuador	Mira	2017	88.5%	98.7%	69.0%
Ecuador	Mocache	2000	52.4%	86.2%	18.9%
Ecuador	Mocache	2017	52.4%	84.1%	21.6%
Ecuador	Mocha	2000	95.4%	96.8%	93.4%

Country	Second Administrative Unit	Year	Mean	Upper	Lower
Ecuador	Mocha	2017	95.2%	96.6%	93.2%
Ecuador	Montalvo	2000	79.2%	99.8%	21.0%
Ecuador	Montalvo	2017	79.2%	99.8%	21.4%
Ecuador	Montecristi	2000	47.2%	72.8%	20.7%
Ecuador	Montecristi	2017	47.7%	73.0%	20.9%
Ecuador	Montúfar	2000	99.0%	99.5%	97.8%
Ecuador	Montúfar	2017	99.0%	99.5%	97.7%
Ecuador	Morona	2000	81.3%	95.1%	65.6%
Ecuador	Morona	2017	80.8%	94.1%	64.8%
Ecuador	Muisne	2000	41.3%	68.8%	16.7%
Ecuador	Muisne	2017	40.1%	69.5%	15.3%
Ecuador	Nabón	2000	60.5%	99.2%	8.4%
Ecuador	Nabón	2017	60.2%	99.2%	8.1%
Ecuador	Nangaritza	2000	79.9%	99.8%	32.6%
Ecuador	Nangaritza	2017	80.1%	99.8%	30.8%
Ecuador	Naranjal	2000	79.3%	97.2%	52.7%
Ecuador	Naranjal	2017	80.9%	97.1%	57.3%
Ecuador	Naranjito	2000	90.7%	99.3%	64.8%
Ecuador	Naranjito	2017	90.9%	99.3%	65.3%
Ecuador	Nobol	2000	74.5%	97.7%	30.5%
Ecuador	Nobol	2017	75.3%	97.3%	33.6%
Ecuador	Olmedo	2000	93.2%	99.5%	74.8%
Ecuador	Olmedo	2000	56.4%	100.0%	0.0%
Ecuador	Olmedo	2017	92.6%	99.5%	72.9%
Ecuador	Olmedo	2017	56.1%	100.0%	0.0%
Ecuador	Oña	2000	68.2%	97.9%	18.0%
Ecuador	Oña	2017	68.9%	97.6%	21.5%
Ecuador	Orellana	2000	68.9%	92.3%	24.0%
Ecuador	Orellana	2017	69.1%	92.4%	25.0%
Ecuador	Otavalo	2000	69.4%	100.0%	3.8%
Ecuador	Otavalo	2017	69.4%	100.0%	3.9%
Ecuador	Pablo Sexto	2000	60.5%	98.8%	8.1%
Ecuador	Pablo Sexto	2017	61.4%	98.5%	9.1%
Ecuador	Paján	2000	35.3%	75.2%	3.7%
Ecuador	Paján	2017	34.3%	74.0%	3.5%
Ecuador	Palanda	2000	67.2%	100.0%	4.3%
Ecuador	Palanda	2017	67.1%	100.0%	3.5%
Ecuador	Palenque	2000	43.2%	85.6%	9.7%
Ecuador	Palenque	2017	42.9%	85.7%	9.6%
Ecuador	Palestina	2000	47.4%	94.0%	5.7%
Ecuador	Palestina	2017	47.2%	94.1%	5.6%
Ecuador	Pallatanga	2000	85.2%	97.8%	60.9%
Ecuador	Pallatanga	2017	85.1%	97.7%	61.6%
Ecuador	Palora	2000	75.1%	99.9%	16.3%
Ecuador	Palora	2017	75.1%	99.9%	16.4%
Ecuador	Paltas	2000	85.7%	98.0%	66.2%
Ecuador	Paltas	2017	84.8%	97.8%	64.3%
Ecuador	Pangua	2000	86.7%	97.2%	63.2%
Ecuador	Pangua	2017	86.7%	96.9%	65.8%
Ecuador	Paquisha	2000	80.9%	100.0%	27.7%
Ecuador	Paquisha	2017	82.3%	100.0%	32.2%
Ecuador	Pasaje	2000	98.4%	99.2%	96.9%
Ecuador	Pasaje	2017	98.3%	99.1%	96.8%
Ecuador	Pastaza	2000	88.4%	97.3%	72.5%
Ecuador	Pastaza	2017	88.6%	97.4%	72.4%
Ecuador	Patate	2000	96.7%	99.1%	90.2%
Ecuador	Patate	2017	96.5%	99.1%	89.3%
Ecuador	Paute	2000	89.7%	98.2%	70.4%
Ecuador	Paute	2017	90.5%	98.2%	72.8%
Ecuador	Pedernales	2000	51.1%	88.6%	15.3%
Ecuador	Pedernales	2017	50.3%	88.1%	14.6%
Ecuador	Pedro Carbo	2000	28.5%	63.9%	5.5%

Country	Second Administrative Unit	Year	Mean	Upper	Lower
Ecuador	Pedro Carbo	2017	27.7%	60.5%	5.4%
Ecuador	Pedro Moncayo	2000	84.8%	99.7%	35.2%
Ecuador	Pedro Moncayo	2017	84.0%	99.7%	32.8%
Ecuador	Pedro Vicente Maldonado	2000	83.3%	99.6%	43.8%
Ecuador	Pedro Vicente Maldonado	2017	83.0%	99.6%	41.3%
Ecuador	Penipe	2000	93.1%	99.9%	70.3%
Ecuador	Penipe	2017	93.1%	99.9%	70.7%
Ecuador	Pichincha	2000	55.9%	99.8%	1.9%
Ecuador	Pichincha	2017	55.7%	99.8%	2.3%
Ecuador	Pimampiro	2000	62.4%	97.4%	11.7%
Ecuador	Pimampiro	2017	61.7%	97.6%	10.6%
Ecuador	Piñas	2000	93.9%	98.6%	85.4%
Ecuador	Piñas	2017	94.3%	98.7%	86.5%
Ecuador	Pindal	2000	64.2%	100.0%	3.7%
Ecuador	Pindal	2017	63.7%	100.0%	3.6%
Ecuador	Playas	2000	86.3%	99.7%	56.4%
Ecuador	Playas	2017	87.9%	99.6%	62.5%
Ecuador	Portovelo	2000	94.5%	99.0%	83.5%
Ecuador	Portovelo	2017	94.2%	99.0%	82.6%
Ecuador	Portoviejo	2000	73.5%	90.3%	50.1%
Ecuador	Portoviejo	2017	74.4%	90.6%	51.7%
Ecuador	Pucará	2000	75.9%	97.4%	26.2%
Ecuador	Pucará	2017	75.6%	96.6%	27.1%
Ecuador	Pueblo Viejo	2000	61.1%	81.1%	25.1%
Ecuador	Pueblo Viejo	2017	61.5%	82.3%	23.8%
Ecuador	Puerto López	2000	68.3%	100.0%	1.1%
Ecuador	Puerto López	2017	68.2%	100.0%	1.1%
Ecuador	Puerto Quito	2000	75.4%	99.4%	41.1%
Ecuador	Puerto Quito	2017	75.5%	99.3%	40.4%
Ecuador	Pujilí	2000	88.8%	96.1%	75.7%
Ecuador	Pujilí	2017	88.5%	95.8%	76.1%
Ecuador	Putumayo	2000	56.7%	99.9%	0.3%
Ecuador	Putumayo	2017	56.6%	99.9%	0.3%
Ecuador	Puyango	2000	83.9%	98.7%	61.9%
Ecuador	Puyango	2017	83.8%	98.7%	62.2%
Ecuador	Quero	2000	96.3%	97.6%	94.2%
Ecuador	Quero	2017	96.2%	97.6%	94.2%
Ecuador	Quevedo	2000	71.3%	89.4%	48.8%
Ecuador	Quevedo	2017	71.1%	89.3%	47.5%
Ecuador	Quijos	2000	80.9%	98.6%	36.9%
Ecuador	Quijos	2017	81.1%	98.4%	37.1%
Ecuador	Quilanga	2000	69.4%	100.0%	0.8%
Ecuador	Quilanga	2017	69.3%	100.0%	0.7%
Ecuador	Quinindé	2000	59.4%	84.8%	31.2%
Ecuador	Quinindé	2017	60.0%	84.8%	32.6%
Ecuador	Quinsaloma	2000	55.2%	86.6%	16.6%
Ecuador	Quinsaloma	2017	53.1%	84.8%	17.2%
Ecuador	Quito	2000	97.2%	99.2%	92.1%
Ecuador	Quito	2017	97.2%	99.2%	92.3%
Ecuador	Río Verde	2000	41.6%	73.3%	16.4%
Ecuador	Río Verde	2017	41.7%	73.3%	17.6%
Ecuador	Riobamba	2000	93.6%	98.4%	87.1%
Ecuador	Riobamba	2017	93.4%	98.3%	86.4%
Ecuador	Rocafuerte	2000	79.7%	97.5%	53.7%
Ecuador	Rocafuerte	2017	79.6%	97.3%	54.8%
Ecuador	Rumiñahui	2000	98.5%	99.5%	95.5%
Ecuador	Rumiñahui	2017	98.5%	99.5%	95.9%
Ecuador	Salcedo	2000	94.5%	96.4%	91.5%

Piped Water Proportion by Second Administrative Unit *(continued)*

Country	Second Administrative Unit	Year	Mean	Upper	Lower
Ecuador	Salcedo	2017	94.3%	96.3%	91.6%
Ecuador	Salinas	2000	83.0%	99.2%	45.7%
Ecuador	Salinas	2017	83.1%	99.1%	46.6%
Ecuador	Samborondón	2000	72.4%	98.5%	38.5%
Ecuador	Samborondón	2017	73.1%	98.7%	39.7%
Ecuador	San Cristóbal	2000	51.2%	100.0%	0.0%
Ecuador	San Cristóbal	2017	51.3%	100.0%	0.0%
Ecuador	San Fernando	2000	62.8%	100.0%	1.5%
Ecuador	San Fernando	2017	62.1%	100.0%	1.4%
Ecuador	San Jacinto de Yaguachi	2000	85.5%	99.2%	47.7%
Ecuador	San Jacinto de Yaguachi	2017	85.7%	99.3%	49.1%
Ecuador	San Juan Bosco	2000	75.3%	100.0%	8.8%
Ecuador	San Juan Bosco	2017	74.4%	100.0%	7.7%
Ecuador	San Lorenzo	2000	75.3%	97.3%	41.3%
Ecuador	San Lorenzo	2017	77.4%	97.0%	46.0%
Ecuador	San Miguel	2000	86.1%	90.2%	79.4%
Ecuador	San Miguel	2017	85.8%	90.2%	78.1%
Ecuador	San Miguel de los Bancos	2000	75.9%	98.5%	36.0%
Ecuador	San Miguel de los Bancos	2017	75.7%	98.6%	34.7%
Ecuador	San Miguel de Urcuquí	2000	69.0%	99.9%	7.6%
Ecuador	San Miguel de Urcuquí	2017	68.8%	99.9%	6.9%
Ecuador	San Pedro de Huaca	2000	97.8%	98.6%	96.4%
Ecuador	San Pedro de Huaca	2017	97.5%	98.3%	96.2%
Ecuador	San Pedro de Pelileo	2000	98.4%	98.9%	97.1%
Ecuador	San Pedro de Pelileo	2017	98.4%	98.9%	97.4%
Ecuador	San Vicente	2000	55.7%	91.5%	14.5%
Ecuador	San Vicente	2017	56.6%	92.5%	14.1%
Ecuador	Santa Ana	2000	65.3%	90.7%	36.7%
Ecuador	Santa Ana	2017	65.0%	90.7%	35.7%
Ecuador	Santa Clara	2000	87.9%	99.9%	58.4%
Ecuador	Santa Clara	2017	89.6%	99.8%	65.9%
Ecuador	Santa Cruz	2000	51.0%	100.0%	0.0%
Ecuador	Santa Cruz	2017	51.0%	100.0%	0.0%
Ecuador	Santa Elena	2000	69.8%	93.0%	44.6%
Ecuador	Santa Elena	2017	69.9%	92.6%	44.5%
Ecuador	Santa Isabel	2000	81.5%	96.1%	57.3%
Ecuador	Santa Isabel	2017	80.7%	95.8%	56.0%
Ecuador	Santa Lucia	2000	25.4%	48.0%	6.9%
Ecuador	Santa Lucia	2017	23.9%	44.2%	6.6%
Ecuador	Santa Rosa	2000	95.7%	99.6%	88.2%
Ecuador	Santa Rosa	2017	96.1%	99.6%	89.4%
Ecuador	Santiago	2000	71.9%	98.9%	21.2%
Ecuador	Santiago	2017	71.2%	99.1%	18.6%
Ecuador	Santiago de Pillaro	2000	95.9%	98.6%	89.0%
Ecuador	Santiago de Pillaro	2017	95.8%	98.6%	88.6%
Ecuador	Santo Domingo	2000	69.3%	90.5%	43.3%
Ecuador	Santo Domingo	2017	70.4%	90.7%	46.4%

Country	Second Administrative Unit	Year	Mean	Upper	Lower
Ecuador	Saquisili	2000	90.3%	95.4%	83.5%
Ecuador	Saquisili	2017	89.9%	95.2%	83.0%
Ecuador	Saquisilí	2000	96.9%	98.8%	88.1%
Ecuador	Saquisilí	2017	96.7%	98.7%	88.1%
Ecuador	Saraguro	2000	85.4%	97.0%	65.6%
Ecuador	Saraguro	2017	84.7%	96.6%	64.6%
Ecuador	Sevilla de Oro	2000	77.6%	100.0%	10.0%
Ecuador	Sevilla de Oro	2017	77.6%	100.0%	10.6%
Ecuador	Shushufindi	2000	73.4%	93.1%	48.5%
Ecuador	Shushufindi	2017	72.3%	93.1%	46.4%
Ecuador	Sigchos	2000	79.1%	97.2%	47.1%
Ecuador	Sigchos	2017	78.9%	97.2%	46.7%
Ecuador	Sigsig	2000	79.3%	92.6%	53.7%
Ecuador	Sigsig	2017	79.3%	92.8%	52.1%
Ecuador	Simon Bolivar	2000	73.6%	100.0%	9.2%
Ecuador	Simon Bolivar	2017	73.2%	100.0%	8.7%
Ecuador	Sozoranga	2000	77.0%	99.3%	28.9%
Ecuador	Sozoranga	2017	76.9%	99.3%	29.2%
Ecuador	Sucre	2000	69.1%	94.7%	38.0%
Ecuador	Sucre	2017	68.8%	94.9%	38.1%
Ecuador	Sucúa	2000	74.6%	98.3%	46.6%
Ecuador	Sucúa	2017	75.9%	98.1%	47.0%
Ecuador	Sucumbíos	2000	86.1%	99.6%	49.6%
Ecuador	Sucumbíos	2017	84.5%	99.6%	44.6%
Ecuador	Suscal	2000	89.0%	95.0%	66.8%
Ecuador	Suscal	2017	88.7%	94.5%	67.9%
Ecuador	Taisha	2000	43.2%	85.7%	3.3%
Ecuador	Taisha	2017	42.9%	87.2%	3.3%
Ecuador	Tena	2000	79.2%	92.0%	60.3%
Ecuador	Tena	2017	78.8%	91.4%	59.8%
Ecuador	Tisaleo	2000	93.9%	96.0%	91.0%
Ecuador	Tisaleo	2017	93.6%	95.8%	90.6%
Ecuador	Tiwintza	2000	55.5%	100.0%	0.0%
Ecuador	Tiwintza	2017	55.3%	100.0%	0.0%
Ecuador	Tosagua	2000	58.9%	82.0%	24.3%
Ecuador	Tosagua	2017	58.7%	81.3%	26.2%
Ecuador	Tulcán	2000	94.0%	99.0%	83.6%
Ecuador	Tulcán	2017	93.9%	98.9%	83.3%
Ecuador	Urbina Jado	2000	43.1%	95.2%	1.2%
Ecuador	Urbina Jado	2017	42.7%	95.3%	1.1%
Ecuador	Urdaneta	2000	78.8%	99.8%	18.2%
Ecuador	Urdaneta	2017	78.4%	99.8%	17.6%
Ecuador	Valencia	2000	67.0%	93.9%	29.9%
Ecuador	Valencia	2017	66.6%	92.2%	32.9%
Ecuador	Ventanas	2000	62.5%	81.2%	41.9%
Ecuador	Ventanas	2017	62.5%	80.9%	42.6%
Ecuador	Vinces	2000	55.5%	69.4%	41.7%
Ecuador	Vinces	2017	55.0%	68.7%	42.6%
Ecuador	Yacuambi	2000	76.1%	98.0%	43.3%
Ecuador	Yacuambi	2017	77.0%	97.9%	46.6%
Ecuador	Yantzaza	2000	84.5%	99.6%	53.5%
Ecuador	Yantzaza	2017	84.4%	99.6%	51.7%
Ecuador	Zamora	2000	86.6%	98.0%	62.0%
Ecuador	Zamora	2017	86.1%	98.0%	62.7%
Ecuador	Zapotillo	2000	57.5%	94.2%	12.7%
Ecuador	Zapotillo	2017	57.2%	94.2%	12.6%
Ecuador	Zaruma	2000	87.2%	95.1%	77.1%
Ecuador	Zaruma	2017	86.8%	94.8%	76.4%
El Salvador	Acajutla	2000	33.2%	54.6%	12.9%
El Salvador	Acajutla	2017	82.7%	94.5%	59.2%
El Salvador	Agua Caliente	2000	61.3%	84.8%	31.7%
El Salvador	Agua Caliente	2017	93.9%	99.0%	82.7%

Country	Second Administrative Unit	Year	Mean	Upper	Lower
El Salvador	Aguilares	2000	37.3%	56.1%	23.7%
El Salvador	Aguilares	2017	88.1%	93.3%	78.0%
El Salvador	Ahuachapán	2000	49.3%	70.3%	29.8%
El Salvador	Ahuachapán	2017	88.4%	97.4%	76.5%
El Salvador	Alegría	2000	82.7%	92.9%	70.9%
El Salvador	Alegría	2017	98.0%	99.5%	92.4%
El Salvador	Anamorós	2000	16.9%	43.8%	2.2%
El Salvador	Anamorós	2017	56.6%	80.5%	26.6%
El Salvador	Antiguo Cuscatlán	2000	85.9%	94.5%	69.0%
El Salvador	Antiguo Cuscatlán	2017	98.5%	99.7%	92.6%
El Salvador	Apaneca	2000	91.4%	99.3%	72.0%
El Salvador	Apaneca	2017	98.6%	100.0%	93.0%
El Salvador	Apastepeque	2000	35.1%	47.3%	22.0%
El Salvador	Apastepeque	2017	86.6%	91.6%	73.0%
El Salvador	Apopa	2000	20.2%	22.7%	18.1%
El Salvador	Apopa	2017	57.6%	64.8%	51.7%
El Salvador	Arambala	2000	32.1%	55.8%	12.2%
El Salvador	Arambala	2017	85.3%	95.3%	65.9%
El Salvador	Arcatao	2000	78.4%	98.2%	34.6%
El Salvador	Arcatao	2017	97.6%	99.9%	87.4%
El Salvador	Armenia	2000	67.7%	82.3%	52.5%
El Salvador	Armenia	2017	96.0%	98.8%	87.3%
El Salvador	Atiquizaya	2000	61.9%	70.2%	49.6%
El Salvador	Atiquizaya	2017	96.0%	97.3%	92.1%
El Salvador	Ayutuxtepeque	2000	66.1%	68.2%	63.7%
El Salvador	Ayutuxtepeque	2017	95.6%	96.1%	95.0%
El Salvador	Azacualpa	2000	50.2%	92.9%	5.6%
El Salvador	Azacualpa	2017	89.0%	99.6%	50.8%
El Salvador	Berlín	2000	77.2%	93.8%	56.4%
El Salvador	Berlín	2017	95.5%	99.6%	85.0%
El Salvador	Bolívar	2000	25.0%	53.0%	11.2%
El Salvador	Bolívar	2017	70.8%	91.9%	40.6%
El Salvador	Cacaopera	2000	59.1%	88.5%	28.2%
El Salvador	Cacaopera	2017	89.5%	99.0%	64.2%
El Salvador	California	2000	80.3%	90.2%	65.7%
El Salvador	California	2017	98.4%	99.3%	96.7%
El Salvador	Caluco	2000	56.0%	90.9%	14.0%
El Salvador	Caluco	2017	90.1%	99.3%	53.5%
El Salvador	Candelaria	2000	67.2%	70.4%	64.3%
El Salvador	Candelaria	2017	96.4%	97.1%	95.7%
El Salvador	Candelaria de la Frontera	2000	54.9%	76.4%	35.7%
El Salvador	Candelaria de la Frontera	2017	91.6%	98.0%	81.5%
El Salvador	Carolina	2000	19.4%	34.2%	8.4%
El Salvador	Carolina	2017	77.5%	88.9%	61.9%
El Salvador	Chalatenango	2000	59.8%	83.5%	34.4%
El Salvador	Chalatenango	2017	93.5%	99.0%	80.4%
El Salvador	Chalchuapa	2000	69.6%	77.8%	59.9%
El Salvador	Chalchuapa	2017	88.7%	97.3%	81.3%
El Salvador	Chapeltique	2000	44.3%	67.0%	20.7%
El Salvador	Chapeltique	2017	88.8%	96.8%	71.1%
El Salvador	Chilanga	2000	42.4%	49.3%	35.1%
El Salvador	Chilanga	2017	90.7%	92.0%	87.3%
El Salvador	Chiltiupán	2000	66.4%	93.4%	35.9%
El Salvador	Chiltiupán	2017	91.4%	99.6%	65.2%
El Salvador	Chinameca	2000	59.1%	72.3%	45.6%
El Salvador	Chinameca	2017	95.1%	97.9%	89.8%
El Salvador	Chirilagua	2000	54.3%	80.0%	27.2%
El Salvador	Chirilagua	2017	88.4%	98.3%	69.3%

Country	Second Administrative Unit	Year	Mean	Upper	Lower
El Salvador	Cinquera	2000	75.3%	89.0%	60.7%
El Salvador	Cinquera	2017	97.3%	99.4%	91.1%
El Salvador	Citalá	2000	96.9%	99.9%	84.2%
El Salvador	Citalá	2017	99.8%	100.0%	98.7%
El Salvador	Ciudad Arce	2000	50.7%	71.1%	30.2%
El Salvador	Ciudad Arce	2017	93.5%	97.7%	85.6%
El Salvador	Ciudad Barrios	2000	38.8%	49.7%	29.3%
El Salvador	Ciudad Barrios	2017	89.9%	93.3%	83.7%
El Salvador	Coatepeque	2000	39.3%	69.3%	14.8%
El Salvador	Coatepeque	2017	82.3%	96.1%	59.3%
El Salvador	Cojutepeque	2000	71.9%	75.3%	68.2%
El Salvador	Cojutepeque	2017	97.1%	97.4%	96.8%
El Salvador	Colón	2000	60.7%	67.0%	53.9%
El Salvador	Colón	2017	96.4%	97.5%	94.7%
El Salvador	Comacarán	2000	41.5%	67.0%	13.6%
El Salvador	Comacarán	2017	88.7%	95.2%	66.5%
El Salvador	Comalapa	2000	93.1%	96.7%	87.7%
El Salvador	Comalapa	2017	99.6%	99.8%	99.3%
El Salvador	Comasagua	2000	67.8%	79.3%	56.3%
El Salvador	Comasagua	2017	96.9%	98.5%	92.9%
El Salvador	Concepción Batres	2000	37.0%	65.0%	17.7%
El Salvador	Concepción Batres	2017	78.9%	94.8%	52.9%
El Salvador	Concepción de Ataco	2000	81.6%	88.7%	75.4%
El Salvador	Concepción de Ataco	2017	97.7%	99.1%	94.7%
El Salvador	Concepción de Oriente	2000	29.7%	59.4%	7.4%
El Salvador	Concepción de Oriente	2017	77.5%	94.1%	46.7%
El Salvador	Concepción Quezaltepeque	2000	76.9%	84.3%	68.1%
El Salvador	Concepción Quezaltepeque	2017	98.4%	99.1%	96.5%
El Salvador	Conchagua	2000	44.5%	80.1%	14.6%
El Salvador	Conchagua	2017	82.1%	97.8%	48.6%
El Salvador	Corinto	2000	43.5%	92.2%	3.1%
El Salvador	Corinto	2017	81.3%	99.5%	23.9%
El Salvador	Cuisnahuat	2000	55.2%	88.6%	20.0%
El Salvador	Cuisnahuat	2017	87.8%	99.2%	52.3%
El Salvador	Cuscatancingo	2000	45.8%	48.8%	43.0%
El Salvador	Cuscatancingo	2017	88.5%	90.2%	86.4%
El Salvador	Cuyultitán	2000	46.8%	71.0%	30.6%
El Salvador	Cuyultitán	2017	92.4%	98.3%	79.4%
El Salvador	Delgado	2000	56.7%	58.9%	54.3%
El Salvador	Delgado	2017	84.5%	87.7%	81.5%
El Salvador	Delicias de Concepción	2000	67.4%	77.5%	52.6%
El Salvador	Delicias de Concepción	2017	97.2%	98.4%	93.4%
El Salvador	Dolores	2000	39.4%	72.5%	15.7%
El Salvador	Dolores	2017	81.9%	97.2%	54.3%
El Salvador	Dulce Nombre de María	2000	88.1%	94.0%	76.6%
El Salvador	Dulce Nombre de María	2017	98.9%	99.6%	96.3%

Country	Second Administrative Unit	Year	Mean	Upper	Lower
El Salvador	El Carmen	2000	34.0%	63.3%	8.2%
El Salvador	El Carmen	2000	23.6%	25.7%	21.6%
El Salvador	El Carmen	2017	80.9%	82.9%	78.6%
El Salvador	El Carmen	2017	80.3%	92.6%	53.0%
El Salvador	El Carrizal	2000	91.4%	98.5%	70.4%
El Salvador	El Carrizal	2017	99.4%	99.9%	97.4%
El Salvador	El Congo	2000	46.5%	72.8%	18.5%
El Salvador	El Congo	2017	88.8%	97.7%	70.0%
El Salvador	El Divisadero	2000	39.8%	56.7%	22.8%
El Salvador	El Divisadero	2017	90.3%	94.3%	81.3%
El Salvador	El Paisnal	2000	37.9%	62.6%	17.6%
El Salvador	El Paisnal	2017	86.0%	96.8%	63.7%
El Salvador	El Paraíso	2000	45.4%	52.6%	38.4%
El Salvador	El Paraíso	2017	94.0%	95.5%	92.1%
El Salvador	El Porvenir	2000	62.7%	77.2%	49.5%
El Salvador	El Porvenir	2017	90.5%	96.8%	80.8%
El Salvador	El Refugio	2000	79.4%	83.8%	75.2%
El Salvador	El Refugio	2017	97.9%	98.5%	97.4%
El Salvador	El Rosario	2000	15.3%	44.9%	1.9%
El Salvador	El Rosario	2000	23.8%	27.7%	20.2%
El Salvador	El Rosario	2000	51.2%	65.1%	37.5%
El Salvador	El Rosario	2017	85.9%	87.7%	83.8%
El Salvador	El Rosario	2017	51.4%	66.1%	23.4%
El Salvador	El Rosario	2017	89.7%	95.6%	76.0%
El Salvador	El Sauce	2000	28.3%	52.8%	9.1%
El Salvador	El Sauce	2017	73.9%	92.4%	50.8%
El Salvador	El Tránsito	2000	47.5%	84.1%	13.4%
El Salvador	El Tránsito	2017	85.5%	98.4%	52.5%
El Salvador	El Triunfo	2000	83.9%	98.1%	53.8%
El Salvador	El Triunfo	2017	97.9%	99.9%	90.4%
El Salvador	Embalse Cerron Grande	2000	47.1%	70.9%	23.1%
El Salvador	Embalse Cerron Grande	2017	87.2%	96.9%	67.4%
El Salvador	Ereguayquín	2000	29.6%	72.5%	1.7%
El Salvador	Ereguayquín	2017	69.2%	88.7%	22.5%
El Salvador	Estanzuelas	2000	79.5%	97.0%	52.3%
El Salvador	Estanzuelas	2017	97.3%	99.8%	87.2%
El Salvador	Guacotecti	2000	90.2%	94.8%	82.2%
El Salvador	Guacotecti	2017	99.4%	99.7%	98.6%
El Salvador	Guadalupe	2000	76.0%	88.3%	63.1%
El Salvador	Guadalupe	2017	97.0%	99.2%	92.1%
El Salvador	Gualococti	2000	29.6%	40.2%	19.4%
El Salvador	Gualococti	2017	84.6%	89.2%	76.0%
El Salvador	Guatajiagua	2000	38.5%	61.9%	18.3%
El Salvador	Guatajiagua	2017	87.4%	95.2%	70.3%
El Salvador	Guaymango	2000	50.9%	62.4%	37.4%
El Salvador	Guaymango	2017	88.6%	93.2%	78.0%
El Salvador	Guazapa	2000	36.1%	54.4%	16.8%
El Salvador	Guazapa	2017	76.3%	92.9%	56.0%
El Salvador	Huizúcar	2000	65.1%	85.2%	30.6%
El Salvador	Huizúcar	2017	95.5%	98.9%	83.4%
El Salvador	Ilobasco	2000	36.9%	48.2%	26.1%
El Salvador	Ilobasco	2017	88.2%	93.1%	79.9%
El Salvador	Ilopango	2000	66.1%	71.2%	60.4%
El Salvador	Ilopango	2017	97.1%	97.5%	96.6%
El Salvador	Intipucá	2000	72.7%	93.5%	44.0%
El Salvador	Intipucá	2017	95.2%	99.6%	84.4%
El Salvador	Izalco	2000	39.7%	56.3%	22.9%
El Salvador	Izalco	2017	87.4%	92.8%	74.3%
El Salvador	Jayaque	2000	86.1%	94.5%	71.0%
El Salvador	Jayaque	2017	98.8%	99.6%	95.7%

Country	Second Administrative Unit	Year	Mean	Upper	Lower
El Salvador	Jerusalén	2000	91.8%	96.0%	86.5%
El Salvador	Jerusalén	2017	99.5%	99.8%	99.2%
El Salvador	Jicalapa	2000	43.5%	87.8%	8.4%
El Salvador	Jicalapa	2017	81.1%	99.1%	44.4%
El Salvador	Jiquilisco	2000	31.8%	55.3%	14.0%
El Salvador	Jiquilisco	2017	74.7%	92.5%	54.7%
El Salvador	Joateca	2000	64.2%	96.8%	23.0%
El Salvador	Joateca	2017	92.0%	99.8%	58.9%
El Salvador	Jocoaitique	2000	23.3%	42.9%	14.5%
El Salvador	Jocoaitique	2017	75.8%	92.4%	49.5%
El Salvador	Jocoro	2000	18.7%	26.5%	13.1%
El Salvador	Jocoro	2017	77.1%	83.3%	69.4%
El Salvador	Juayúa	2000	76.9%	86.0%	65.6%
El Salvador	Juayúa	2017	92.8%	95.1%	89.8%
El Salvador	Jucuapa	2000	86.7%	96.4%	62.2%
El Salvador	Jucuapa	2017	98.8%	99.8%	95.6%
El Salvador	Jucuarán	2000	65.4%	88.1%	40.6%
El Salvador	Jucuarán	2017	93.1%	99.1%	80.4%
El Salvador	Jujutla	2000	43.8%	74.9%	19.0%
El Salvador	Jujutla	2017	84.1%	97.1%	61.5%
El Salvador	Jutiapa	2000	33.2%	48.6%	16.7%
El Salvador	Jutiapa	2017	85.9%	91.8%	75.9%
El Salvador	La Laguna	2000	94.5%	97.7%	89.7%
El Salvador	La Laguna	2017	99.7%	99.9%	99.4%
El Salvador	La Libertad	2000	43.7%	65.3%	20.0%
El Salvador	La Libertad	2017	85.7%	94.9%	67.0%
El Salvador	La Palma	2000	93.9%	99.5%	80.9%
El Salvador	La Palma	2017	99.1%	100.0%	95.2%
El Salvador	La Reina	2000	69.5%	86.9%	46.9%
El Salvador	La Reina	2017	95.8%	99.1%	86.0%
El Salvador	La Unión	2000	43.0%	77.9%	15.1%
El Salvador	La Unión	2017	79.9%	97.0%	47.1%
El Salvador	Lago de Coatepeque	2000	48.5%	91.9%	3.7%
El Salvador	Lago de Coatepeque	2017	86.1%	99.6%	36.2%
El Salvador	Lago de Guija	2000	52.3%	94.4%	8.1%
El Salvador	Lago de Guija	2017	87.1%	99.5%	50.6%
El Salvador	Lago de Llopango	2000	72.8%	78.6%	64.4%
El Salvador	Lago de Llopango	2017	97.7%	98.3%	96.9%
El Salvador	Las Vueltas	2000	88.5%	96.1%	73.4%
El Salvador	Las Vueltas	2017	98.6%	99.8%	94.7%
El Salvador	Lislique	2000	41.6%	87.1%	4.1%
El Salvador	Lislique	2017	80.8%	99.0%	31.3%
El Salvador	Lolotique	2000	84.0%	95.4%	62.1%
El Salvador	Lolotique	2017	98.2%	99.7%	92.9%
El Salvador	Lolotiquillo	2000	23.6%	25.8%	21.2%
El Salvador	Lolotiquillo	2017	84.9%	86.1%	83.7%
El Salvador	Masahuat	2000	34.3%	53.9%	18.0%
El Salvador	Masahuat	2017	85.5%	95.1%	70.4%
El Salvador	Meanguera	2000	69.9%	80.4%	55.7%
El Salvador	Meanguera	2017	93.1%	94.9%	89.8%
El Salvador	Meanguera del Golfo	2000	25.0%	59.7%	1.0%
El Salvador	Meanguera del Golfo	2017	52.7%	68.8%	15.4%
El Salvador	Mejicanos	2000	67.7%	70.5%	64.8%
El Salvador	Mejicanos	2017	97.6%	97.8%	97.3%
El Salvador	Mercedes La Ceiba	2000	94.1%	97.1%	88.6%

Country	Second Administrative Unit	Year	Mean	Upper	Lower
El Salvador	Mercedes La Ceiba	2017	99.7%	99.8%	99.3%
El Salvador	Mercedes Umaña	2000	84.1%	95.3%	63.8%
El Salvador	Mercedes Umaña	2017	98.2%	99.7%	93.7%
El Salvador	Metapán	2000	57.6%	80.5%	31.5%
El Salvador	Metapán	2017	91.5%	98.3%	77.8%
El Salvador	Moncagua	2000	44.8%	70.0%	23.1%
El Salvador	Moncagua	2017	89.4%	96.7%	76.9%
El Salvador	Monte San Juan	2000	55.3%	58.9%	51.8%
El Salvador	Monte San Juan	2017	92.9%	93.7%	91.9%
El Salvador	Nahuizalco	2000	67.0%	70.7%	61.4%
El Salvador	Nahuizalco	2017	89.4%	90.3%	88.6%
El Salvador	Nahulingo	2000	20.5%	25.7%	15.7%
El Salvador	Nahulingo	2017	80.8%	83.6%	77.5%
El Salvador	Nejapa	2000	52.1%	56.1%	48.4%
El Salvador	Nejapa	2017	75.2%	77.9%	72.1%
El Salvador	Nombre de Jesús	2000	56.2%	67.8%	42.3%
El Salvador	Nombre de Jesús	2017	95.3%	97.8%	89.1%
El Salvador	Nueva Concepción	2000	42.5%	67.9%	21.6%
El Salvador	Nueva Concepción	2017	83.3%	96.4%	65.1%
El Salvador	Nueva Esparta	2000	44.2%	78.6%	9.1%
El Salvador	Nueva Esparta	2017	80.8%	97.6%	45.4%
El Salvador	Nueva Granada	2000	78.4%	97.1%	47.4%
El Salvador	Nueva Granada	2017	96.5%	99.8%	83.5%
El Salvador	Nueva Guadalupe	2000	73.6%	91.1%	46.4%
El Salvador	Nueva Guadalupe	2017	97.9%	99.5%	93.3%
El Salvador	Nueva San Salvador	2000	85.3%	90.5%	76.9%
El Salvador	Nueva San Salvador	2017	98.7%	99.4%	95.3%
El Salvador	Nueva Trinidad	2000	71.2%	97.6%	25.1%
El Salvador	Nueva Trinidad	2017	95.0%	99.9%	76.7%
El Salvador	Nuevo Cuscatlán	2000	78.8%	98.5%	25.7%
El Salvador	Nuevo Cuscatlán	2017	97.8%	99.9%	86.9%
El Salvador	Nuevo Edén de San Juan	2000	25.5%	55.2%	6.3%
El Salvador	Nuevo Edén de San Juan	2017	72.8%	91.0%	46.4%
El Salvador	Ojos de Agua	2000	86.9%	96.3%	70.5%
El Salvador	Ojos de Agua	2017	99.1%	99.8%	97.0%
El Salvador	Olocuilta	2000	42.9%	61.7%	26.7%
El Salvador	Olocuilta	2017	89.9%	95.1%	78.7%
El Salvador	Opico	2000	45.5%	72.0%	22.6%
El Salvador	Opico	2017	88.9%	97.3%	67.4%

Country	Second Administrative Unit	Year	Mean	Upper	Lower
El Salvador	Oratorio de Concepción	2000	28.6%	37.6%	17.9%
El Salvador	Oratorio de Concepción	2017	85.6%	88.2%	79.4%
El Salvador	Osicala	2000	68.0%	73.8%	58.6%
El Salvador	Osicala	2017	94.8%	95.5%	93.6%
El Salvador	Ozatlán	2000	30.7%	71.0%	7.0%
El Salvador	Ozatlán	2017	78.9%	97.0%	50.3%
El Salvador	Panchimalco	2000	35.1%	42.6%	28.1%
El Salvador	Panchimalco	2017	84.2%	87.6%	78.8%
El Salvador	Paraíso de Osorio	2000	64.7%	68.9%	56.9%
El Salvador	Paraíso de Osorio	2017	71.2%	75.8%	68.9%
El Salvador	Pasaquina	2000	51.2%	72.2%	28.9%
El Salvador	Pasaquina	2017	85.1%	96.7%	63.2%
El Salvador	Perquín	2000	43.2%	74.1%	18.1%
El Salvador	Perquín	2017	85.5%	98.0%	57.4%
El Salvador	Polorós	2000	41.4%	77.6%	8.8%
El Salvador	Polorós	2017	82.8%	97.3%	48.4%
El Salvador	Potonico	2000	40.3%	78.4%	8.1%
El Salvador	Potonico	2017	83.8%	98.2%	45.7%
El Salvador	Puerto El Triunfo	2000	15.3%	32.9%	4.2%
El Salvador	Puerto El Triunfo	2017	67.5%	87.1%	40.5%
El Salvador	Quelepa	2000	21.4%	33.2%	12.9%
El Salvador	Quelepa	2017	78.3%	84.2%	67.7%
El Salvador	Quezaltepeque	2000	52.3%	67.3%	39.6%
El Salvador	Quezaltepeque	2017	92.5%	97.2%	82.2%
El Salvador	Rosario de Mora	2000	32.8%	59.5%	14.0%
El Salvador	Rosario de Mora	2017	81.9%	93.9%	58.9%
El Salvador	Sacacoyo	2000	87.3%	93.8%	76.4%
El Salvador	Sacacoyo	2017	99.2%	99.7%	98.4%
El Salvador	Salcoatitán	2000	79.8%	88.6%	58.4%
El Salvador	Salcoatitán	2017	98.3%	99.2%	95.5%
El Salvador	San Agustín	2000	53.7%	84.8%	20.7%
El Salvador	San Agustín	2017	87.7%	98.9%	56.4%
El Salvador	San Alejo	2000	30.1%	53.6%	11.0%
El Salvador	San Alejo	2017	73.5%	89.0%	52.6%
El Salvador	San Antonio	2000	18.2%	37.5%	6.3%
El Salvador	San Antonio	2017	78.3%	91.8%	56.8%
El Salvador	San Antonio de la Cruz	2000	85.9%	98.1%	65.0%
El Salvador	San Antonio de la Cruz	2017	95.9%	99.9%	77.6%
El Salvador	San Antonio del Monte	2000	56.2%	66.8%	42.5%
El Salvador	San Antonio del Monte	2017	94.3%	95.7%	92.1%
El Salvador	San Antonio Los Ranchos	2000	57.0%	97.2%	8.3%
El Salvador	San Antonio Los Ranchos	2017	91.0%	99.9%	56.0%
El Salvador	San Antonio Masahuat	2000	79.7%	96.5%	41.7%
El Salvador	San Antonio Masahuat	2017	96.9%	99.8%	86.0%
El Salvador	San Antonio Pajonal	2000	58.6%	93.6%	13.6%

Country	Second Administrative Unit	Year	Mean	Upper	Lower
El Salvador	San Antonio Pajonal	2017	89.7%	99.6%	50.9%
El Salvador	San Bartolomé Perulapía	2000	25.5%	28.4%	22.3%
El Salvador	San Bartolomé Perulapía	2017	86.4%	87.5%	84.9%
El Salvador	San Buenaventura	2000	88.2%	97.9%	58.1%
El Salvador	San Buenaventura	2017	98.9%	99.8%	95.0%
El Salvador	San Carlos	2000	69.5%	92.6%	36.4%
El Salvador	San Carlos	2017	95.7%	99.5%	81.9%
El Salvador	San Cayetano Istepeque	2000	22.8%	27.6%	18.4%
El Salvador	San Cayetano Istepeque	2017	88.1%	90.4%	85.2%
El Salvador	San Cristóbal	2000	98.4%	99.3%	96.4%
El Salvador	San Cristóbal	2017	99.9%	99.9%	99.8%
El Salvador	San Dionisio	2000	61.5%	82.9%	35.6%
El Salvador	San Dionisio	2017	92.5%	98.5%	76.3%
El Salvador	San Emigdio	2000	83.1%	93.1%	63.6%
El Salvador	San Emigdio	2017	99.0%	99.6%	97.4%
El Salvador	San Esteban Catarina	2000	18.1%	27.2%	12.0%
El Salvador	San Esteban Catarina	2017	75.6%	83.4%	66.7%
El Salvador	San Fernando	2000	97.0%	100.0%	86.3%
El Salvador	San Fernando	2000	33.7%	89.8%	0.5%
El Salvador	San Fernando	2017	99.6%	100.0%	96.7%
El Salvador	San Fernando	2017	74.0%	99.1%	10.3%
El Salvador	San Francisco Chinameca	2000	50.0%	83.4%	14.4%
El Salvador	San Francisco Chinameca	2017	90.5%	98.9%	66.4%
El Salvador	San Francisco Gotera	2000	35.4%	44.8%	24.8%
El Salvador	San Francisco Gotera	2017	89.3%	92.3%	83.4%
El Salvador	San Francisco Javier	2000	47.5%	84.4%	19.0%
El Salvador	San Francisco Javier	2017	86.5%	98.8%	58.7%
El Salvador	San Francisco Lempa	2000	47.5%	93.5%	2.8%
El Salvador	San Francisco Lempa	2017	83.8%	99.7%	34.4%
El Salvador	San Francisco Menéndez	2000	34.3%	62.8%	13.8%
El Salvador	San Francisco Menéndez	2017	80.2%	95.5%	58.8%
El Salvador	San Francisco Morazán	2000	73.0%	90.8%	46.7%
El Salvador	San Francisco Morazán	2017	95.9%	99.5%	81.9%
El Salvador	San Gerardo	2000	19.7%	41.1%	5.5%
El Salvador	San Gerardo	2017	68.8%	82.2%	49.1%
El Salvador	San Ignacio	2000	98.2%	99.8%	92.1%
El Salvador	San Ignacio	2017	99.8%	100.0%	99.0%
El Salvador	San Ildefonso	2000	62.1%	86.7%	33.0%
El Salvador	San Ildefonso	2017	92.4%	99.1%	75.9%

Country	Second Administrative Unit	Year	Mean	Upper	Lower
El Salvador	San Isidro	2000	23.8%	69.6%	2.4%
El Salvador	San Isidro	2000	38.5%	44.7%	27.9%
El Salvador	San Isidro	2017	56.6%	59.3%	53.4%
El Salvador	San Isidro	2017	72.8%	97.4%	23.7%
El Salvador	San Isidro Labrador	2000	59.9%	96.0%	19.6%
El Salvador	San Isidro Labrador	2017	92.4%	99.8%	61.4%
El Salvador	San Jorge	2000	33.8%	49.9%	20.6%
El Salvador	San Jorge	2017	84.7%	91.0%	73.6%
El Salvador	San José	2000	52.1%	61.4%	41.2%
El Salvador	San José	2017	86.4%	92.2%	79.3%
El Salvador	San José Cancasque	2000	52.3%	97.1%	5.1%
El Salvador	San José Cancasque	2017	87.3%	99.9%	38.2%
El Salvador	San José Guayabal	2000	73.0%	85.0%	56.9%
El Salvador	San José Guayabal	2017	95.6%	97.2%	89.9%
El Salvador	San José Las Flores	2000	69.1%	98.3%	20.1%
El Salvador	San José Las Flores	2017	92.3%	99.9%	61.9%
El Salvador	San José Villanueva	2000	78.1%	88.3%	60.7%
El Salvador	San José Villanueva	2017	98.1%	99.2%	95.2%
El Salvador	San Juan Nonualco	2000	21.3%	35.4%	11.9%
El Salvador	San Juan Nonualco	2017	74.7%	87.2%	60.1%
El Salvador	San Juan Talpa	2000	75.8%	84.0%	67.3%
El Salvador	San Juan Talpa	2017	96.8%	98.6%	92.7%
El Salvador	San Juan Tepezontes	2000	86.2%	97.0%	66.3%
El Salvador	San Juan Tepezontes	2017	97.7%	99.8%	86.3%
El Salvador	San Julián	2000	79.2%	91.7%	57.0%
El Salvador	San Julián	2017	97.4%	99.5%	91.8%
El Salvador	San Lorenzo	2000	13.6%	16.0%	11.7%
El Salvador	San Lorenzo	2000	60.9%	73.5%	44.1%
El Salvador	San Lorenzo	2017	74.9%	78.7%	70.6%
El Salvador	San Lorenzo	2017	95.3%	97.9%	89.9%
El Salvador	San Luis de la Reina	2000	31.8%	58.3%	12.0%
El Salvador	San Luis de la Reina	2017	83.7%	94.0%	66.5%
El Salvador	San Luis del Carmen	2000	51.9%	97.5%	3.7%
El Salvador	San Luis del Carmen	2017	88.7%	99.9%	42.3%
El Salvador	San Luis La Herradura	2000	42.4%	83.0%	6.7%
El Salvador	San Luis La Herradura	2017	79.5%	98.2%	40.3%
El Salvador	San Luis Talpa	2000	50.4%	63.6%	40.2%
El Salvador	San Luis Talpa	2017	84.6%	93.7%	71.5%

Country	Second Administrative Unit	Year	Mean	Upper	Lower
El Salvador	San Marcos	2000	50.4%	58.2%	39.1%
El Salvador	San Marcos	2017	94.1%	95.1%	90.1%
El Salvador	San Martín	2000	36.1%	38.1%	34.1%
El Salvador	San Martín	2017	91.1%	91.6%	90.6%
El Salvador	San Matías	2000	44.2%	84.6%	9.1%
El Salvador	San Matías	2017	85.5%	98.8%	48.4%
El Salvador	San Miguel	2000	29.9%	45.7%	16.2%
El Salvador	San Miguel	2017	79.5%	87.7%	66.8%
El Salvador	San Miguel de Mercedes	2000	54.0%	94.7%	7.9%
El Salvador	San Miguel de Mercedes	2017	90.4%	99.7%	53.8%
El Salvador	San Miguel Tepezontes	2000	77.2%	95.8%	46.0%
El Salvador	San Miguel Tepezontes	2017	96.3%	99.8%	77.2%
El Salvador	San Pablo Tacachico	2000	31.5%	58.0%	9.8%
El Salvador	San Pablo Tacachico	2017	81.2%	94.9%	58.8%
El Salvador	San Pedro Masahuat	2000	55.1%	77.9%	35.4%
El Salvador	San Pedro Masahuat	2017	89.5%	97.9%	68.9%
El Salvador	San Pedro Nonualco	2000	20.2%	29.0%	12.5%
El Salvador	San Pedro Nonualco	2017	39.3%	50.5%	31.2%
El Salvador	San Pedro Perulapán	2000	42.9%	50.8%	34.2%
El Salvador	San Pedro Perulapán	2017	92.9%	94.7%	88.1%
El Salvador	San Pedro Puxtla	2000	68.3%	75.2%	60.1%
El Salvador	San Pedro Puxtla	2017	97.2%	98.0%	96.2%
El Salvador	San Rafael	2000	60.4%	70.3%	46.4%
El Salvador	San Rafael	2000	25.6%	45.9%	11.5%
El Salvador	San Rafael	2017	96.0%	97.5%	93.1%
El Salvador	San Rafael	2017	80.6%	90.7%	58.2%
El Salvador	San Rafael Cedros	2000	24.8%	28.1%	22.0%
El Salvador	San Rafael Cedros	2017	83.0%	84.7%	81.3%
El Salvador	San Rafael Obrajuelo	2000	18.3%	23.4%	14.5%
El Salvador	San Rafael Obrajuelo	2017	80.3%	84.1%	76.1%
El Salvador	San Ramón	2000	96.1%	97.2%	94.6%
El Salvador	San Ramón	2017	99.8%	99.9%	99.7%
El Salvador	San Salvador	2000	69.4%	73.5%	65.7%
El Salvador	San Salvador	2017	97.4%	97.8%	96.9%
El Salvador	San Sebastián	2000	29.3%	35.9%	25.0%
El Salvador	San Sebastián	2017	85.8%	89.8%	78.6%
El Salvador	San Sebastián Salitrillo	2000	70.0%	87.9%	51.6%
El Salvador	San Sebastián Salitrillo	2017	96.2%	99.3%	87.2%
El Salvador	San Simón	2000	35.7%	55.9%	18.9%
El Salvador	San Simón	2017	87.2%	94.0%	74.3%
El Salvador	San Vicente	2000	34.9%	47.8%	21.1%
El Salvador	San Vicente	2017	80.7%	91.3%	67.1%

Country	Second Administrative Unit	Year	Mean	Upper	Lower
El Salvador	Santa Ana	2000	34.3%	44.1%	23.6%
El Salvador	Santa Ana	2017	84.1%	90.9%	74.6%
El Salvador	Santa Catarina Masahuat	2000	69.5%	75.7%	62.1%
El Salvador	Santa Catarina Masahuat	2017	97.2%	98.0%	95.9%
El Salvador	Santa Clara	2000	42.0%	80.2%	9.0%
El Salvador	Santa Clara	2017	82.6%	97.9%	48.6%
El Salvador	Santa Cruz Analquito	2000	84.4%	91.7%	72.8%
El Salvador	Santa Cruz Analquito	2017	99.1%	99.5%	98.2%
El Salvador	Santa Cruz Michapa	2000	55.3%	58.6%	52.2%
El Salvador	Santa Cruz Michapa	2017	95.2%	95.6%	94.8%
El Salvador	Santa Elena	2000	50.0%	70.9%	33.0%
El Salvador	Santa Elena	2017	90.3%	97.6%	73.2%
El Salvador	Santa Isabel Ishuatán	2000	42.7%	65.3%	19.2%
El Salvador	Santa Isabel Ishuatán	2017	84.6%	94.2%	66.8%
El Salvador	Santa María	2000	29.7%	58.9%	12.2%
El Salvador	Santa María	2017	75.4%	88.9%	42.5%
El Salvador	Santa María Ostuma	2000	28.4%	34.3%	23.7%
El Salvador	Santa María Ostuma	2017	43.8%	53.9%	37.5%
El Salvador	Santa Rita	2000	73.1%	93.0%	48.2%
El Salvador	Santa Rita	2017	95.0%	99.5%	76.7%
El Salvador	Santa Rosa de Lima	2000	39.7%	61.6%	22.0%
El Salvador	Santa Rosa de Lima	2017	80.6%	94.6%	65.9%
El Salvador	Santa Rosa Guachipilín	2000	43.9%	69.2%	15.8%
El Salvador	Santa Rosa Guachipilín	2017	89.8%	97.4%	69.3%
El Salvador	Santiago de la Frontera	2000	87.5%	97.4%	68.9%
El Salvador	Santiago de la Frontera	2017	99.0%	99.9%	96.4%
El Salvador	Santiago de María	2000	94.9%	98.6%	88.2%
El Salvador	Santiago de María	2017	99.7%	99.9%	99.0%
El Salvador	Santiago Nonualco	2000	35.4%	54.8%	18.6%
El Salvador	Santiago Nonualco	2017	82.1%	90.0%	66.2%
El Salvador	Santiago Texacuangos	2000	53.7%	63.0%	44.1%
El Salvador	Santiago Texacuangos	2017	94.8%	96.8%	88.8%
El Salvador	Santo Domingo	2000	65.0%	73.4%	54.8%
El Salvador	Santo Domingo	2000	59.0%	64.8%	48.7%
El Salvador	Santo Domingo	2017	95.7%	96.8%	94.2%
El Salvador	Santo Domingo	2017	93.9%	94.6%	92.9%

Country	Second Administrative Unit	Year	Mean	Upper	Lower
El Salvador	Santo Tomás	2000	27.2%	29.9%	24.7%
El Salvador	Santo Tomás	2017	86.0%	87.2%	84.8%
El Salvador	Sensembra	2000	49.5%	70.7%	30.4%
El Salvador	Sensembra	2017	93.1%	97.9%	82.1%
El Salvador	Sensuntepeque	2000	62.0%	80.2%	45.3%
El Salvador	Sensuntepeque	2017	92.3%	98.3%	80.1%
El Salvador	Sesori	2000	40.0%	70.7%	13.6%
El Salvador	Sesori	2017	83.4%	97.1%	60.2%
El Salvador	Sociedad	2000	21.4%	44.0%	6.0%
El Salvador	Sociedad	2017	69.6%	87.7%	44.2%
El Salvador	Sonsonate	2000	53.3%	70.6%	38.1%
El Salvador	Sonsonate	2017	91.2%	96.1%	80.4%
El Salvador	Sonzacate	2000	55.9%	65.4%	44.0%
El Salvador	Sonzacate	2017	94.7%	96.2%	92.7%
El Salvador	Soyapango	2000	44.6%	48.8%	41.0%
El Salvador	Soyapango	2017	93.9%	94.8%	93.2%
El Salvador	Suchitoto	2000	56.4%	83.6%	24.3%
El Salvador	Suchitoto	2017	89.3%	98.6%	70.4%
El Salvador	Tacuba	2000	40.7%	55.9%	25.4%
El Salvador	Tacuba	2017	87.2%	93.2%	74.8%
El Salvador	Talnique	2000	85.3%	94.1%	70.3%
El Salvador	Talnique	2017	98.9%	99.6%	97.5%
El Salvador	Tamanique	2000	61.7%	92.7%	25.5%
El Salvador	Tamanique	2017	91.4%	99.4%	63.6%
El Salvador	Tapalhuaca	2000	91.1%	99.2%	69.9%
El Salvador	Tapalhuaca	2017	99.0%	100.0%	96.1%
El Salvador	Tecapán	2000	65.0%	82.8%	43.1%
El Salvador	Tecapán	2017	92.0%	97.8%	75.5%
El Salvador	Tecoluca	2000	34.0%	58.0%	12.0%
El Salvador	Tecoluca	2017	76.5%	92.2%	53.9%
El Salvador	Tejutepeque	2000	32.1%	36.3%	27.3%
El Salvador	Tejutepeque	2017	88.5%	89.8%	86.4%
El Salvador	Tejutla	2000	67.4%	86.9%	41.3%
El Salvador	Tejutla	2017	94.0%	99.2%	74.9%
El Salvador	Tenancingo	2000	69.9%	82.0%	52.0%
El Salvador	Tenancingo	2017	96.5%	98.4%	90.5%
El Salvador	Teotepeque	2000	35.5%	58.4%	17.4%
El Salvador	Teotepeque	2017	76.4%	91.0%	54.2%
El Salvador	Tepecoyo	2000	91.7%	97.4%	83.3%
El Salvador	Tepecoyo	2017	99.1%	99.9%	96.0%
El Salvador	Tepetitán	2000	48.2%	51.6%	43.4%
El Salvador	Tepetitán	2017	91.7%	93.3%	89.9%
El Salvador	Texistepeque	2000	36.8%	63.3%	15.8%
El Salvador	Texistepeque	2017	80.5%	92.7%	63.2%
El Salvador	Tonacatepeque	2000	54.3%	57.0%	51.8%
El Salvador	Tonacatepeque	2017	83.5%	87.6%	80.2%
El Salvador	Torola	2000	29.2%	56.2%	9.2%
El Salvador	Torola	2017	84.9%	95.3%	65.1%
El Salvador	Turín	2000	55.0%	65.2%	42.3%
El Salvador	Turín	2017	95.9%	97.4%	93.7%
El Salvador	Uluazapa	2000	74.0%	91.6%	47.2%
El Salvador	Uluazapa	2017	95.1%	99.4%	83.8%
El Salvador	Usulután	2000	36.1%	46.7%	27.8%
El Salvador	Usulután	2017	86.9%	91.5%	79.8%
El Salvador	Verapaz	2000	95.1%	97.9%	87.0%
El Salvador	Verapaz	2017	99.7%	99.9%	99.1%
El Salvador	Victoria	2000	43.0%	61.4%	25.8%
El Salvador	Victoria	2017	87.3%	95.2%	74.1%
El Salvador	Yamabal	2000	50.5%	85.2%	20.8%
El Salvador	Yamabal	2017	87.9%	98.8%	65.1%
El Salvador	Yayantique	2000	55.8%	81.4%	31.1%
El Salvador	Yayantique	2017	92.7%	98.5%	76.5%

Country	Second Administrative Unit	Year	Mean	Upper	Lower
El Salvador	Yoloaiquín	2000	60.2%	67.7%	51.5%
El Salvador	Yoloaiquín	2017	96.6%	97.6%	94.9%
El Salvador	Yucuaiquín	2000	37.9%	53.1%	27.0%
El Salvador	Yucuaiquín	2017	88.6%	91.8%	81.4%
El Salvador	Zacatecoluca	2000	25.4%	52.2%	9.5%
El Salvador	Zacatecoluca	2017	73.1%	90.9%	50.7%
El Salvador	Zaragoza	2000	86.9%	96.0%	69.4%
El Salvador	Zaragoza	2017	98.5%	99.8%	93.8%
Guatemala	Acatenango	2000	87.2%	98.6%	57.9%
Guatemala	Acatenango	2017	81.7%	96.9%	49.7%
Guatemala	Agua Blanca	2000	76.7%	93.2%	39.4%
Guatemala	Agua Blanca	2017	67.1%	88.7%	29.7%
Guatemala	Aguacatán	2000	76.3%	96.0%	48.6%
Guatemala	Aguacatán	2017	67.6%	92.2%	38.5%
Guatemala	Almolonga	2000	93.5%	95.4%	90.4%
Guatemala	Almolonga	2017	81.6%	87.0%	74.6%
Guatemala	Alotenango	2000	92.0%	95.8%	84.4%
Guatemala	Alotenango	2017	85.5%	91.8%	74.4%
Guatemala	Amatitlán	2000	60.8%	67.0%	55.4%
Guatemala	Amatitlán	2017	46.6%	51.0%	43.0%
Guatemala	Antigua Guatemala	2000	79.3%	80.6%	77.9%
Guatemala	Antigua Guatemala	2017	66.5%	68.1%	64.8%
Guatemala	Asunción Mita	2000	80.4%	96.5%	57.5%
Guatemala	Asunción Mita	2017	73.7%	93.2%	51.8%
Guatemala	Atescatempa	2000	86.1%	94.0%	67.8%
Guatemala	Atescatempa	2017	77.6%	89.3%	55.5%
Guatemala	Ayutla	2000	60.5%	87.0%	26.0%
Guatemala	Ayutla	2017	47.4%	77.0%	16.5%
Guatemala	Barberena	2000	73.8%	88.1%	49.7%
Guatemala	Barberena	2017	61.3%	79.9%	37.0%
Guatemala	Cabañas	2000	87.0%	95.9%	71.0%
Guatemala	Cabañas	2017	78.9%	91.8%	60.8%
Guatemala	Cabricán	2000	96.7%	99.3%	89.9%
Guatemala	Cabricán	2017	94.7%	98.5%	86.5%
Guatemala	Cajolá	2000	94.2%	96.3%	90.8%
Guatemala	Cajolá	2017	88.1%	92.1%	82.4%
Guatemala	Camotán	2000	85.2%	96.4%	69.8%
Guatemala	Camotán	2017	78.9%	93.0%	63.2%
Guatemala	Canillá	2000	70.9%	98.2%	23.8%
Guatemala	Canillá	2017	62.9%	96.4%	17.8%
Guatemala	Cantel	2000	92.6%	94.1%	90.6%
Guatemala	Cantel	2017	86.9%	89.4%	83.3%
Guatemala	Casillas	2000	54.0%	74.4%	36.3%
Guatemala	Casillas	2017	43.8%	66.6%	26.7%
Guatemala	Catarina	2000	44.9%	66.6%	23.6%
Guatemala	Catarina	2017	27.2%	45.8%	13.2%
Guatemala	Chahal	2000	69.1%	89.9%	39.6%
Guatemala	Chahal	2017	58.8%	85.6%	28.8%
Guatemala	Chajul	2000	77.7%	92.2%	58.6%
Guatemala	Chajul	2017	70.9%	87.7%	50.2%
Guatemala	Champerico	2000	54.2%	71.8%	33.8%
Guatemala	Champerico	2017	43.2%	61.5%	24.0%
Guatemala	Chiantla	2000	76.7%	91.7%	51.9%
Guatemala	Chiantla	2017	69.6%	86.5%	43.0%
Guatemala	Chicacao	2000	85.7%	95.0%	73.7%
Guatemala	Chicacao	2017	80.3%	91.1%	69.0%
Guatemala	Chicaman	2000	69.1%	82.9%	52.3%
Guatemala	Chicaman	2017	61.8%	76.4%	45.4%

Country	Second Administrative Unit	Year	Mean	Upper	Lower
Guatemala	Chiché	2000	64.7%	73.5%	48.0%
Guatemala	Chiché	2017	52.8%	64.8%	37.2%
Guatemala	Chichicastenango	2000	93.3%	97.8%	79.4%
Guatemala	Chichicastenango	2017	88.4%	95.6%	70.8%
Guatemala	Chimaltenango	2000	81.2%	82.6%	79.2%
Guatemala	Chimaltenango	2017	67.3%	69.6%	64.7%
Guatemala	Chinautla	2000	63.9%	72.8%	51.7%
Guatemala	Chinautla	2017	47.1%	58.0%	35.0%
Guatemala	Chinique	2000	75.9%	98.1%	30.2%
Guatemala	Chinique	2017	66.5%	96.0%	22.0%
Guatemala	Chiquimula	2000	74.3%	79.6%	67.0%
Guatemala	Chiquimula	2017	64.4%	70.8%	56.7%
Guatemala	Chiquimulilla	2000	70.9%	85.8%	53.7%
Guatemala	Chiquimulilla	2017	61.1%	78.8%	42.6%
Guatemala	Chisec	2000	66.1%	79.1%	51.5%
Guatemala	Chisec	2017	57.5%	71.5%	42.8%
Guatemala	Chuarrancho	2000	67.2%	97.5%	21.5%
Guatemala	Chuarrancho	2017	57.6%	94.8%	14.4%
Guatemala	Ciudad Vieja	2000	89.7%	91.2%	88.2%
Guatemala	Ciudad Vieja	2017	81.4%	83.7%	78.9%
Guatemala	Coatepeque	2000	33.6%	43.0%	25.2%
Guatemala	Coatepeque	2017	24.3%	32.3%	17.5%
Guatemala	Cobán	2000	64.7%	74.2%	53.6%
Guatemala	Cobán	2017	52.4%	63.1%	41.1%
Guatemala	Colomba	2000	81.8%	87.7%	70.8%
Guatemala	Colomba	2017	74.5%	81.7%	62.4%
Guatemala	Colotenango	2000	93.1%	98.0%	86.0%
Guatemala	Colotenango	2017	89.2%	96.4%	77.5%
Guatemala	Comalapa	2000	82.3%	84.5%	79.3%
Guatemala	Comalapa	2017	69.5%	72.8%	65.5%
Guatemala	Comapa	2000	77.3%	92.5%	48.0%
Guatemala	Comapa	2017	68.1%	86.1%	37.9%
Guatemala	Comitancillo	2000	91.3%	94.9%	82.3%
Guatemala	Comitancillo	2017	88.0%	92.6%	74.9%
Guatemala	Concepción	2000	86.9%	98.9%	63.7%
Guatemala	Concepción	2017	80.2%	97.7%	51.1%
Guatemala	Concepción Chiquirichapa	2000	93.7%	96.1%	89.0%
Guatemala	Concepción Chiquirichapa	2017	87.2%	91.5%	80.6%
Guatemala	Concepción Huista	2000	83.3%	98.2%	52.2%
Guatemala	Concepción Huista	2017	76.1%	96.3%	41.6%
Guatemala	Concepción Las Minas	2000	52.6%	65.1%	36.6%
Guatemala	Concepción Las Minas	2017	42.9%	57.6%	27.4%
Guatemala	Concepción Tutuapa	2000	87.3%	98.7%	67.1%
Guatemala	Concepción Tutuapa	2017	81.3%	97.3%	56.1%
Guatemala	Conguaco	2000	88.0%	98.4%	66.7%
Guatemala	Conguaco	2017	83.5%	96.9%	62.3%
Guatemala	Cubulco	2000	75.4%	88.7%	59.3%
Guatemala	Cubulco	2017	66.1%	81.5%	48.5%
Guatemala	Cuilapa	2000	85.1%	92.7%	68.6%
Guatemala	Cuilapa	2017	77.2%	87.5%	59.7%
Guatemala	Cuilco	2000	78.6%	94.9%	57.5%
Guatemala	Cuilco	2017	72.1%	90.6%	49.1%
Guatemala	Cunén	2000	81.1%	96.9%	53.0%
Guatemala	Cunén	2017	73.5%	94.5%	41.7%

Country	Second Administrative Unit	Year	Mean	Upper	Lower
Guatemala	Cuyotenango	2000	51.6%	67.7%	32.0%
Guatemala	Cuyotenango	2017	38.6%	57.5%	21.3%
Guatemala	Dolores	2000	72.2%	85.1%	58.1%
Guatemala	Dolores	2017	63.6%	78.2%	48.5%
Guatemala	El Adelanto	2000	91.9%	97.0%	75.4%
Guatemala	El Adelanto	2017	86.0%	94.5%	62.7%
Guatemala	El Asintal	2000	45.0%	58.8%	33.3%
Guatemala	El Asintal	2017	31.7%	45.0%	22.4%
Guatemala	El Estor	2000	67.3%	81.7%	51.2%
Guatemala	El Estor	2017	59.2%	75.1%	42.9%
Guatemala	El Jícaro	2000	81.6%	88.5%	69.2%
Guatemala	El Jícaro	2017	72.9%	83.0%	56.9%
Guatemala	El Palmar	2000	75.5%	95.3%	35.0%
Guatemala	El Palmar	2017	66.8%	91.9%	27.9%
Guatemala	El Progreso	2000	47.1%	69.7%	27.4%
Guatemala	El Progreso	2017	34.6%	54.6%	19.9%
Guatemala	El Quetzal	2000	70.4%	85.6%	46.8%
Guatemala	El Quetzal	2017	59.3%	78.1%	35.9%
Guatemala	El Rodeo	2000	56.3%	92.1%	12.8%
Guatemala	El Rodeo	2017	46.8%	86.6%	8.0%
Guatemala	El Tejar	2000	81.9%	83.9%	79.5%
Guatemala	El Tejar	2017	68.7%	71.3%	65.8%
Guatemala	El Tumbador	2000	65.7%	87.0%	38.1%
Guatemala	El Tumbador	2017	55.2%	80.1%	28.5%
Guatemala	Escuintla	2000	61.1%	70.0%	52.1%
Guatemala	Escuintla	2017	47.9%	58.0%	39.9%
Guatemala	Esquipulas	2000	70.4%	85.3%	52.2%
Guatemala	Esquipulas	2017	61.3%	77.9%	42.7%
Guatemala	Esquipulas Palo Gordo	2000	85.5%	91.1%	77.2%
Guatemala	Esquipulas Palo Gordo	2017	75.2%	83.4%	64.9%
Guatemala	Estanzuela	2000	67.8%	75.5%	59.3%
Guatemala	Estanzuela	2017	54.4%	62.0%	46.1%
Guatemala	Flores	2000	47.5%	56.5%	38.3%
Guatemala	Flores	2017	39.8%	50.1%	30.2%
Guatemala	Flores Costa Cuca	2000	83.9%	89.4%	72.9%
Guatemala	Flores Costa Cuca	2017	78.0%	84.1%	68.8%
Guatemala	Fraijanes	2000	61.8%	92.2%	20.0%
Guatemala	Fraijanes	2017	50.3%	85.9%	11.9%
Guatemala	Fray Bartolomé de las Casas	2000	68.2%	86.0%	46.7%
Guatemala	Fray Bartolomé de las Casas	2017	59.8%	80.2%	36.0%
Guatemala	Génova	2000	67.9%	91.8%	36.6%
Guatemala	Génova	2017	62.2%	85.5%	35.9%
Guatemala	Granados	2000	82.0%	95.3%	62.3%
Guatemala	Granados	2017	76.1%	91.3%	56.5%
Guatemala	Gualán	2000	72.0%	82.1%	59.4%
Guatemala	Gualán	2017	63.1%	74.5%	49.9%
Guatemala	Guanagazapa	2000	71.4%	89.3%	46.7%
Guatemala	Guanagazapa	2017	61.8%	83.9%	37.1%
Guatemala	Guastatoya	2000	79.0%	84.5%	70.0%
Guatemala	Guastatoya	2017	67.4%	74.5%	56.5%
Guatemala	Guazacapán	2000	65.0%	90.5%	31.3%
Guatemala	Guazacapán	2017	53.5%	84.3%	21.9%
Guatemala	Huehuetenango	2000	63.2%	84.6%	32.3%
Guatemala	Huehuetenango	2017	51.3%	78.0%	19.9%

Country	Second Administrative Unit	Year	Mean	Upper	Lower
Guatemala	Huitán	2000	96.4%	98.6%	86.4%
Guatemala	Huitán	2017	94.0%	97.6%	76.8%
Guatemala	Huité	2000	83.8%	90.8%	72.8%
Guatemala	Huité	2017	74.1%	83.9%	59.9%
Guatemala	Ipala	2000	81.8%	96.9%	47.2%
Guatemala	Ipala	2017	73.6%	94.6%	35.8%
Guatemala	Ixcán	2000	64.3%	82.5%	42.6%
Guatemala	Ixcán	2017	55.3%	75.5%	32.8%
Guatemala	Ixchiguan	2000	92.5%	97.6%	81.9%
Guatemala	Ixchiguan	2017	88.9%	95.8%	77.4%
Guatemala	Iztapa	2000	67.4%	92.9%	25.5%
Guatemala	Iztapa	2017	56.3%	86.4%	19.5%
Guatemala	Jacaltenango	2000	81.1%	98.0%	49.6%
Guatemala	Jacaltenango	2017	74.5%	96.4%	38.7%
Guatemala	Jalapa	2000	74.6%	83.9%	62.1%
Guatemala	Jalapa	2017	65.0%	77.0%	54.0%
Guatemala	Jalpatagua	2000	82.1%	96.0%	58.6%
Guatemala	Jalpatagua	2017	74.8%	92.4%	48.6%
Guatemala	Jerez	2000	87.0%	98.3%	56.3%
Guatemala	Jerez	2017	79.8%	96.7%	48.7%
Guatemala	Jocotán	2000	79.7%	93.4%	57.1%
Guatemala	Jocotán	2017	69.5%	87.9%	44.3%
Guatemala	Jocotenango	2000	77.3%	78.9%	75.4%
Guatemala	Jocotenango	2017	63.0%	64.9%	60.7%
Guatemala	Joyabaj	2000	80.2%	94.7%	58.3%
Guatemala	Joyabaj	2017	72.3%	90.2%	49.4%
Guatemala	Jutiapa	2000	56.0%	62.2%	47.8%
Guatemala	Jutiapa	2017	44.8%	52.1%	35.4%
Guatemala	La Democracia	2000	62.4%	87.9%	31.3%
Guatemala	La Democracia	2000	70.0%	84.7%	46.9%
Guatemala	La Democracia	2017	62.1%	80.4%	36.0%
Guatemala	La Democracia	2017	51.0%	80.3%	22.3%
Guatemala	La Esperanza	2000	88.4%	92.1%	83.7%
Guatemala	La Esperanza	2017	78.8%	85.5%	70.7%
Guatemala	La Gomera	2000	61.7%	84.0%	36.6%
Guatemala	La Gomera	2017	52.0%	76.3%	27.9%
Guatemala	La Libertad	2000	80.7%	94.8%	49.2%
Guatemala	La Libertad	2000	66.1%	74.9%	55.3%
Guatemala	La Libertad	2017	74.3%	93.3%	40.3%
Guatemala	La Libertad	2017	57.5%	67.5%	44.7%
Guatemala	La Reforma	2000	58.2%	77.4%	26.0%
Guatemala	La Reforma	2017	51.6%	74.4%	20.0%
Guatemala	La Unión	2000	79.2%	89.1%	67.4%
Guatemala	La Unión	2017	71.2%	82.1%	59.3%
Guatemala	Lanquín	2000	63.2%	90.4%	22.2%
Guatemala	Lanquín	2017	54.0%	84.5%	15.9%
Guatemala	Livingston	2000	65.6%	80.3%	48.7%
Guatemala	Livingston	2017	54.9%	71.4%	37.7%
Guatemala	Los Amates	2000	73.1%	88.0%	53.3%
Guatemala	Los Amates	2017	65.1%	82.2%	44.3%
Guatemala	Magdalena Milpas Altas	2000	90.3%	92.2%	88.1%
Guatemala	Magdalena Milpas Altas	2017	80.2%	83.6%	76.6%
Guatemala	Malacatán	2000	63.9%	82.6%	43.0%
Guatemala	Malacatán	2017	52.1%	71.8%	33.0%
Guatemala	Malacatancito	2000	67.5%	86.5%	46.7%
Guatemala	Malacatancito	2017	53.4%	77.1%	33.1%

Country	Second Administrative Unit	Year	Mean	Upper	Lower
Guatemala	Masagua	2000	60.4%	81.8%	31.3%
Guatemala	Masagua	2017	49.8%	75.2%	21.0%
Guatemala	Mataquescuintla	2000	93.3%	98.0%	82.0%
Guatemala	Mataquescuintla	2017	89.4%	96.4%	75.8%
Guatemala	Mazatenango	2000	38.8%	44.0%	32.7%
Guatemala	Mazatenango	2017	23.9%	28.1%	19.7%
Guatemala	Melchor de Mencos	2000	68.1%	84.6%	48.5%
Guatemala	Melchor de Mencos	2017	59.1%	77.2%	39.8%
Guatemala	Mixco	2000	58.2%	60.8%	55.5%
Guatemala	Mixco	2017	42.8%	45.2%	40.4%
Guatemala	Momostenango	2000	72.0%	78.7%	63.7%
Guatemala	Momostenango	2017	61.7%	70.0%	53.1%
Guatemala	Monjas	2000	79.1%	89.7%	65.8%
Guatemala	Monjas	2017	69.9%	82.3%	53.0%
Guatemala	Morales	2000	57.5%	69.6%	45.8%
Guatemala	Morales	2017	46.7%	59.0%	34.9%
Guatemala	Morazán	2000	79.7%	92.2%	65.2%
Guatemala	Morazán	2017	71.2%	86.5%	54.6%
Guatemala	Moyuta	2000	66.9%	88.6%	35.0%
Guatemala	Moyuta	2017	59.6%	83.6%	29.4%
Guatemala	NA	2000	74.8%	88.5%	56.0%
Guatemala	NA	2000	48.3%	59.4%	40.5%
Guatemala	NA	2017	67.4%	84.5%	44.6%
Guatemala	NA	2017	32.5%	40.6%	26.9%
Guatemala	Nahualá	2000	95.5%	97.0%	91.8%
Guatemala	Nahualá	2017	91.0%	93.3%	85.6%
Guatemala	Nebaj	2000	78.6%	92.7%	60.3%
Guatemala	Nebaj	2017	71.4%	88.1%	52.3%
Guatemala	Nentón	2000	78.0%	92.8%	58.9%
Guatemala	Nentón	2017	70.7%	88.8%	49.0%
Guatemala	Nueva Concepción	2000	45.9%	65.9%	27.3%
Guatemala	Nueva Concepción	2017	36.1%	56.5%	19.1%
Guatemala	Nueva Santa Rosa	2000	54.7%	61.7%	48.5%
Guatemala	Nueva Santa Rosa	2017	43.6%	49.8%	38.6%
Guatemala	Nuevo Progreso	2000	55.2%	80.8%	25.5%
Guatemala	Nuevo Progreso	2017	45.7%	72.1%	17.8%
Guatemala	Nuevo San Carlos	2000	60.6%	64.3%	54.5%
Guatemala	Nuevo San Carlos	2017	46.5%	50.2%	40.5%
Guatemala	Ocos	2000	63.1%	89.8%	22.3%
Guatemala	Ocos	2017	53.8%	85.9%	15.4%
Guatemala	Olintepeque	2000	80.9%	83.3%	78.2%
Guatemala	Olintepeque	2017	64.1%	67.7%	60.2%
Guatemala	Olopa	2000	71.1%	88.2%	47.6%
Guatemala	Olopa	2017	58.2%	78.4%	34.2%
Guatemala	Oratorio	2000	57.4%	76.9%	36.9%
Guatemala	Oratorio	2017	50.2%	71.1%	29.0%
Guatemala	Ostuncalco	2000	94.7%	97.8%	85.8%
Guatemala	Ostuncalco	2017	90.0%	95.0%	77.0%
Guatemala	Pachalúm	2000	82.5%	99.1%	35.6%
Guatemala	Pachalúm	2017	75.5%	98.1%	26.6%
Guatemala	Pajapita	2000	46.7%	75.6%	21.0%
Guatemala	Pajapita	2017	35.7%	66.0%	11.9%

Country	Second Administrative Unit	Year	Mean	Upper	Lower
Guatemala	Palencia	2000	75.7%	88.0%	56.2%
Guatemala	Palencia	2017	64.8%	80.5%	44.7%
Guatemala	Palestina de Los Altos	2000	95.5%	98.4%	84.5%
Guatemala	Palestina de Los Altos	2017	88.9%	95.6%	74.3%
Guatemala	Palín	2000	61.5%	84.7%	36.3%
Guatemala	Palín	2017	53.6%	79.0%	29.9%
Guatemala	Panajachel	2000	34.3%	61.8%	20.4%
Guatemala	Panajachel	2017	26.6%	45.7%	16.8%
Guatemala	Panzós	2000	72.4%	86.9%	55.0%
Guatemala	Panzós	2017	62.3%	78.9%	42.0%
Guatemala	Parramos	2000	84.4%	88.4%	79.4%
Guatemala	Parramos	2017	71.3%	77.8%	64.1%
Guatemala	Pasaco	2000	69.2%	93.2%	31.6%
Guatemala	Pasaco	2017	60.0%	88.5%	22.3%
Guatemala	Pastores	2000	77.4%	79.6%	75.1%
Guatemala	Pastores	2017	62.3%	65.0%	59.5%
Guatemala	Patulul	2000	48.2%	62.1%	37.4%
Guatemala	Patulul	2017	40.0%	54.4%	31.5%
Guatemala	Patzicía	2000	77.6%	85.8%	60.4%
Guatemala	Patzicía	2017	64.8%	76.1%	47.7%
Guatemala	Patzité	2000	90.4%	94.3%	80.0%
Guatemala	Patzité	2017	81.3%	87.5%	69.5%
Guatemala	Patzún	2000	80.0%	88.8%	61.2%
Guatemala	Patzún	2017	69.3%	81.0%	49.6%
Guatemala	Petapa	2000	67.6%	70.9%	64.3%
Guatemala	Petapa	2017	50.8%	54.3%	47.4%
Guatemala	Pochuta	2000	92.3%	99.6%	71.7%
Guatemala	Pochuta	2017	88.0%	99.1%	62.1%
Guatemala	Poptún	2000	71.4%	84.7%	53.9%
Guatemala	Poptún	2017	61.2%	75.7%	44.7%
Guatemala	Pueblo Nuevo	2000	70.2%	82.1%	44.2%
Guatemala	Pueblo Nuevo	2017	57.0%	75.6%	27.0%
Guatemala	Pueblo Nuevo Viñas	2000	80.9%	97.2%	59.0%
Guatemala	Pueblo Nuevo Viñas	2017	74.4%	95.1%	50.2%
Guatemala	Puerto Barrios	2000	47.3%	58.8%	35.4%
Guatemala	Puerto Barrios	2017	36.4%	47.3%	24.9%
Guatemala	Purulhá	2000	77.0%	91.1%	57.4%
Guatemala	Purulhá	2017	69.4%	86.2%	48.9%
Guatemala	Quetzaltenango	2000	82.0%	84.8%	77.3%
Guatemala	Quetzaltenango	2017	70.6%	74.2%	64.7%
Guatemala	Quezada	2000	59.8%	79.1%	22.7%
Guatemala	Quezada	2017	50.4%	73.0%	16.5%
Guatemala	Quezaltepeque	2000	72.5%	78.6%	62.8%
Guatemala	Quezaltepeque	2017	60.5%	67.2%	52.1%
Guatemala	Rabinal	2000	90.2%	95.6%	78.9%
Guatemala	Rabinal	2017	82.8%	90.7%	69.4%
Guatemala	Retalhuleu	2000	52.6%	64.2%	38.3%
Guatemala	Retalhuleu	2017	39.9%	50.8%	27.9%
Guatemala	Río Blanco	2000	98.1%	99.7%	93.3%
Guatemala	Río Blanco	2017	96.5%	99.2%	89.5%
Guatemala	Río Bravo	2000	76.5%	85.5%	61.2%
Guatemala	Río Bravo	2017	68.0%	78.6%	51.8%
Guatemala	Río Hondo	2000	77.8%	89.5%	60.1%
Guatemala	Río Hondo	2017	69.4%	84.1%	49.2%
Guatemala	Sacapulas	2000	80.5%	94.5%	60.7%
Guatemala	Sacapulas	2017	73.1%	90.1%	51.2%

Country	Second Administrative Unit	Year	Mean	Upper	Lower
Guatemala	Salamá	2000	70.4%	79.3%	61.2%
Guatemala	Salamá	2017	60.5%	69.5%	51.4%
Guatemala	Salcajá	2000	76.8%	79.1%	74.5%
Guatemala	Salcajá	2017	63.0%	66.0%	60.1%
Guatemala	Samayac	2000	48.2%	52.3%	44.2%
Guatemala	Samayac	2017	35.0%	38.2%	31.6%
Guatemala	San Agustín Acasaguastlán	2000	81.1%	87.1%	70.2%
Guatemala	San Agustín Acasaguastlán	2017	71.8%	79.2%	60.5%
Guatemala	San Andrés	2000	58.3%	70.0%	46.1%
Guatemala	San Andrés	2017	50.7%	62.0%	39.0%
Guatemala	San Andrés Itzapa	2000	82.6%	85.7%	77.9%
Guatemala	San Andrés Itzapa	2017	69.3%	73.7%	63.0%
Guatemala	San Andrés Sajcabajá	2000	73.1%	93.1%	41.9%
Guatemala	San Andrés Sajcabajá	2017	64.6%	87.7%	30.9%
Guatemala	San Andrés Semetabaj	2000	79.1%	88.6%	69.8%
Guatemala	San Andrés Semetabaj	2017	73.4%	82.0%	61.2%
Guatemala	San Andrés Villa Seca	2000	54.1%	68.4%	39.2%
Guatemala	San Andrés Villa Seca	2017	43.6%	60.1%	29.0%
Guatemala	San Andrés Xecul	2000	75.2%	77.4%	72.7%
Guatemala	San Andrés Xecul	2017	58.4%	61.8%	54.7%
Guatemala	San Antonio Aguas Calientes	2000	77.4%	80.0%	74.8%
Guatemala	San Antonio Aguas Calientes	2017	63.5%	66.9%	60.2%
Guatemala	San Antonio Huista	2000	87.4%	98.0%	67.9%
Guatemala	San Antonio Huista	2017	80.9%	96.3%	56.7%
Guatemala	San Antonio Ilotenango	2000	70.0%	77.6%	57.6%
Guatemala	San Antonio Ilotenango	2017	51.8%	59.1%	42.7%
Guatemala	San Antonio La Paz	2000	69.3%	77.4%	57.9%
Guatemala	San Antonio La Paz	2017	58.7%	68.6%	42.2%
Guatemala	San Antonio Palopó	2000	64.5%	81.3%	49.4%
Guatemala	San Antonio Palopó	2017	49.4%	68.4%	36.2%
Guatemala	San Antonio Sacatepéquez	2000	92.5%	99.5%	70.0%
Guatemala	San Antonio Sacatepéquez	2017	88.8%	98.3%	68.0%
Guatemala	San Antonio Suchitepéquez	2000	66.8%	75.1%	59.0%

Country	Second Administrative Unit	Year	Mean	Upper	Lower
Guatemala	San Antonio Suchitepéquez	2017	50.3%	60.1%	42.2%
Guatemala	San Bartolo	2000	57.3%	76.3%	40.1%
Guatemala	San Bartolo	2017	45.2%	64.5%	26.9%
Guatemala	San Bartolomé Jocotenango	2000	69.7%	98.2%	18.7%
Guatemala	San Bartolomé Jocotenango	2017	60.9%	96.6%	12.9%
Guatemala	San Bartolomé Milpas Altas	2000	85.6%	89.2%	82.1%
Guatemala	San Bartolomé Milpas Altas	2017	74.2%	79.5%	69.1%
Guatemala	San Benito	2000	79.0%	86.4%	70.5%
Guatemala	San Benito	2017	67.5%	76.0%	55.9%
Guatemala	San Bernardino	2000	48.7%	52.4%	45.3%
Guatemala	San Bernardino	2017	33.0%	36.1%	30.2%
Guatemala	San Carlos Alzatate	2000	98.0%	99.6%	90.8%
Guatemala	San Carlos Alzatate	2017	96.5%	99.2%	86.0%
Guatemala	San Carlos Sija	2000	77.4%	86.6%	66.8%
Guatemala	San Carlos Sija	2017	71.4%	81.5%	60.3%
Guatemala	San Cristóbal Acasaguastlán	2000	84.9%	92.0%	74.6%
Guatemala	San Cristóbal Acasaguastlán	2017	77.7%	87.0%	66.6%
Guatemala	San Cristobal Cucho	2000	96.8%	98.7%	88.2%
Guatemala	San Cristobal Cucho	2017	93.8%	97.2%	82.6%
Guatemala	San Cristóbal Totonicapán	2000	84.0%	85.6%	81.9%
Guatemala	San Cristóbal Totonicapán	2017	71.9%	74.7%	68.5%
Guatemala	San Cristóbal Verapaz	2000	67.2%	91.4%	36.9%
Guatemala	San Cristóbal Verapaz	2017	58.3%	85.8%	28.8%
Guatemala	San Diego	2000	80.3%	94.0%	52.8%
Guatemala	San Diego	2017	70.6%	88.8%	38.6%
Guatemala	San Felipe	2000	50.7%	61.5%	28.8%
Guatemala	San Felipe	2017	40.3%	55.3%	17.5%
Guatemala	San Francisco	2000	81.4%	93.5%	61.9%
Guatemala	San Francisco	2017	75.3%	88.5%	56.8%
Guatemala	San Francisco El Alto	2000	89.5%	90.6%	88.2%
Guatemala	San Francisco El Alto	2017	79.5%	81.5%	77.0%
Guatemala	San Francisco La Unión	2000	85.2%	90.7%	76.4%
Guatemala	San Francisco La Unión	2017	71.5%	79.6%	59.3%
Guatemala	San Francisco Zapotitlán	2000	65.2%	75.2%	46.1%

Country	Second Administrative Unit	Year	Mean	Upper	Lower
Guatemala	San Francisco Zapotitlán	2017	49.6%	63.7%	29.1%
Guatemala	San Gabriel	2000	30.4%	35.0%	26.3%
Guatemala	San Gabriel	2017	17.3%	20.3%	14.5%
Guatemala	San Gaspar Ixchil	2000	85.1%	94.1%	71.4%
Guatemala	San Gaspar Ixchil	2017	77.1%	90.6%	61.7%
Guatemala	San Ildefonso Ixtahuacán	2000	49.8%	66.6%	33.4%
Guatemala	San Ildefonso Ixtahuacán	2017	42.8%	59.4%	26.2%
Guatemala	San Jacinto	2000	87.7%	91.6%	83.1%
Guatemala	San Jacinto	2017	77.9%	83.3%	72.2%
Guatemala	San Jerónimo	2000	65.4%	75.4%	48.9%
Guatemala	San Jerónimo	2017	53.9%	67.5%	34.8%
Guatemala	San José	2000	65.8%	87.1%	37.1%
Guatemala	San José	2000	53.7%	71.7%	32.6%
Guatemala	San José	2017	54.2%	78.8%	26.4%
Guatemala	San José	2017	43.1%	61.8%	24.7%
Guatemala	San José Acatempa	2000	71.7%	90.1%	51.2%
Guatemala	San José Acatempa	2017	64.5%	85.7%	44.1%
Guatemala	San José Chacayá	2000	78.6%	81.9%	75.2%
Guatemala	San José Chacayá	2017	64.1%	68.4%	59.5%
Guatemala	San José del Golfo	2000	53.7%	74.3%	32.4%
Guatemala	San José del Golfo	2017	42.0%	64.3%	22.2%
Guatemala	San José El Idolo	2000	57.8%	91.3%	14.9%
Guatemala	San José El Idolo	2017	48.8%	86.5%	10.2%
Guatemala	San José La Arada	2000	81.6%	97.6%	37.3%
Guatemala	San José La Arada	2017	72.8%	95.8%	25.7%
Guatemala	San José Ojetenam	2000	86.1%	99.7%	50.7%
Guatemala	San José Ojetenam	2017	79.1%	99.1%	38.3%
Guatemala	San José Pinula	2000	82.9%	93.7%	61.7%
Guatemala	San José Pinula	2017	75.1%	88.2%	51.8%
Guatemala	San José Poaquil	2000	92.5%	99.9%	71.0%
Guatemala	San José Poaquil	2017	88.7%	99.6%	60.7%
Guatemala	San Juan Atitán	2000	89.5%	99.2%	55.7%
Guatemala	San Juan Atitán	2017	85.2%	98.3%	46.7%
Guatemala	San Juan Bautista	2000	37.8%	52.4%	33.0%
Guatemala	San Juan Bautista	2017	25.2%	34.7%	21.5%
Guatemala	San Juan Chamelco	2000	74.6%	85.9%	60.0%

Country	Second Administrative Unit	Year	Mean	Upper	Lower
Guatemala	San Juan Chamelco	2017	63.1%	76.6%	48.1%
Guatemala	San Juan Cotzal	2000	71.2%	94.9%	32.9%
Guatemala	San Juan Cotzal	2017	62.2%	90.0%	22.4%
Guatemala	San Juan Ermita	2000	86.8%	89.8%	83.3%
Guatemala	San Juan Ermita	2017	76.5%	80.8%	71.0%
Guatemala	San Juan Ixcoy	2000	76.5%	97.1%	40.4%
Guatemala	San Juan Ixcoy	2017	67.6%	94.4%	28.5%
Guatemala	San Juan La Laguna	2000	94.8%	97.9%	77.2%
Guatemala	San Juan La Laguna	2017	90.5%	95.6%	69.1%
Guatemala	San Juan Sacatepéquez	2000	58.1%	64.0%	50.0%
Guatemala	San Juan Sacatepéquez	2017	46.1%	54.0%	37.0%
Guatemala	San Juan Tecuaco	2000	77.1%	96.2%	39.8%
Guatemala	San Juan Tecuaco	2017	67.5%	92.6%	28.9%
Guatemala	San Lorenzo	2000	63.9%	86.0%	35.6%
Guatemala	San Lorenzo	2000	84.9%	99.4%	34.8%
Guatemala	San Lorenzo	2017	77.8%	98.8%	24.5%
Guatemala	San Lorenzo	2017	53.9%	77.0%	27.2%
Guatemala	San Lucas Sacatepéquez	2000	79.3%	83.2%	74.3%
Guatemala	San Lucas Sacatepéquez	2017	66.1%	71.5%	59.3%
Guatemala	San Lucas Tolimán	2000	87.1%	93.4%	63.8%
Guatemala	San Lucas Tolimán	2017	78.8%	90.8%	45.3%
Guatemala	San Luis	2000	61.6%	85.0%	34.0%
Guatemala	San Luis	2000	70.1%	81.0%	55.8%
Guatemala	San Luis	2017	62.3%	74.6%	47.2%
Guatemala	San Luis	2017	52.4%	77.7%	24.8%
Guatemala	San Luis Jilotepeque	2000	91.0%	97.0%	79.4%
Guatemala	San Luis Jilotepeque	2017	86.1%	94.3%	73.6%
Guatemala	San Manuel Chaparrón	2000	80.6%	89.7%	65.2%
Guatemala	San Manuel Chaparrón	2017	70.1%	82.1%	54.6%
Guatemala	San Marcos	2000	85.3%	94.7%	66.8%
Guatemala	San Marcos	2017	76.7%	89.8%	55.1%
Guatemala	San Marcos La Laguna	2000	92.3%	93.6%	90.5%
Guatemala	San Marcos La Laguna	2017	83.3%	85.7%	80.2%
Guatemala	San Martín Jilotepeque	2000	84.1%	93.8%	63.1%
Guatemala	San Martín Jilotepeque	2017	75.9%	88.6%	52.4%
Guatemala	San Martín Sacatepéquez	2000	94.1%	99.4%	77.0%

Country	Second Administrative Unit	Year	Mean	Upper	Lower
Guatemala	San Martín Sacatepéquez	2017	90.3%	99.1%	64.4%
Guatemala	San Martín Zapotitlán	2000	31.0%	38.4%	24.1%
Guatemala	San Martín Zapotitlán	2017	17.8%	22.8%	13.6%
Guatemala	San Mateo	2000	92.5%	94.8%	89.4%
Guatemala	San Mateo	2017	85.1%	89.5%	79.2%
Guatemala	San Mateo Ixtatán	2000	71.0%	93.8%	41.4%
Guatemala	San Mateo Ixtatán	2017	62.8%	89.0%	31.8%
Guatemala	San Miguel Acatán	2000	91.7%	98.5%	78.5%
Guatemala	San Miguel Acatán	2017	86.1%	97.1%	68.9%
Guatemala	San Miguel Chicaj	2000	89.8%	93.6%	82.0%
Guatemala	San Miguel Chicaj	2017	84.3%	90.3%	73.1%
Guatemala	San Miguel Dueñas	2000	76.6%	84.0%	67.2%
Guatemala	San Miguel Dueñas	2017	65.1%	73.8%	54.7%
Guatemala	San Miguel Ixtahuacán	2000	76.1%	96.2%	46.4%
Guatemala	San Miguel Ixtahuacán	2017	66.1%	93.8%	32.0%
Guatemala	San Miguel Panán	2000	74.8%	79.0%	70.3%
Guatemala	San Miguel Panán	2017	59.4%	64.9%	53.8%
Guatemala	San Miguel Sigüilá	2000	88.7%	92.5%	83.7%
Guatemala	San Miguel Sigüilá	2017	79.3%	85.6%	72.0%
Guatemala	San Pablo	2000	76.3%	95.4%	38.3%
Guatemala	San Pablo	2017	67.2%	91.2%	30.4%
Guatemala	San Pablo Jocopilas	2000	73.5%	77.8%	68.5%
Guatemala	San Pablo Jocopilas	2017	59.0%	63.9%	53.3%
Guatemala	San Pablo La Laguna	2000	93.9%	95.3%	92.1%
Guatemala	San Pablo La Laguna	2017	88.2%	90.8%	84.9%
Guatemala	San Pedro Ayampuc	2000	52.3%	66.0%	34.8%
Guatemala	San Pedro Ayampuc	2017	37.2%	50.0%	22.4%
Guatemala	San Pedro Carchá	2000	62.0%	75.2%	46.0%
Guatemala	San Pedro Carchá	2017	51.7%	66.2%	36.3%
Guatemala	San Pedro Jocopilas	2000	72.2%	90.6%	45.9%
Guatemala	San Pedro Jocopilas	2017	62.2%	82.6%	37.6%
Guatemala	San Pedro La Laguna	2000	82.2%	91.5%	55.5%
Guatemala	San Pedro La Laguna	2017	71.4%	83.4%	45.5%

Country	Second Administrative Unit	Year	Mean	Upper	Lower
Guatemala	San Pedro Necta	2000	70.9%	93.4%	25.2%
Guatemala	San Pedro Necta	2017	64.3%	93.5%	17.5%
Guatemala	San Pedro Pinula	2000	65.2%	86.7%	38.7%
Guatemala	San Pedro Pinula	2017	55.5%	81.1%	29.1%
Guatemala	San Pedro Sacatepéquez	2000	88.9%	97.4%	68.8%
Guatemala	San Pedro Sacatepéquez	2000	79.9%	82.9%	76.5%
Guatemala	San Pedro Sacatepéquez	2017	62.5%	66.2%	57.6%
Guatemala	San Pedro Sacatepéquez	2017	82.2%	94.9%	61.3%
Guatemala	San Rafael La Independencia	2000	93.4%	99.8%	64.1%
Guatemala	San Rafael La Independencia	2017	88.9%	99.5%	49.6%
Guatemala	San Rafaél Las Flores	2000	66.3%	89.6%	33.2%
Guatemala	San Rafaél Las Flores	2017	56.0%	85.1%	25.0%
Guatemala	San Rafael Petzal	2000	96.9%	98.9%	91.2%
Guatemala	San Rafael Petzal	2017	95.0%	98.4%	84.9%
Guatemala	San Rafaél Pie de la Cuesta	2000	83.8%	97.1%	57.3%
Guatemala	San Rafaél Pie de la Cuesta	2017	76.9%	94.4%	49.1%
Guatemala	San Raymundo	2000	38.1%	58.8%	11.6%
Guatemala	San Raymundo	2017	29.8%	52.5%	5.8%
Guatemala	San Sebastián	2000	32.8%	38.7%	28.2%
Guatemala	San Sebastián	2017	19.3%	23.5%	16.0%
Guatemala	San Sebastián Coatán	2000	90.6%	99.7%	68.2%
Guatemala	San Sebastián Coatán	2017	86.9%	99.4%	61.7%
Guatemala	San Sebastián Huehuetenango	2000	91.6%	95.6%	85.2%
Guatemala	San Sebastián Huehuetenango	2017	86.2%	92.0%	79.7%
Guatemala	San Sibinal	2000	81.2%	99.5%	42.5%
Guatemala	San Sibinal	2017	75.0%	98.9%	33.4%
Guatemala	San Vicente Pacaya	2000	51.8%	83.7%	11.2%
Guatemala	San Vicente Pacaya	2017	41.7%	78.1%	6.6%
Guatemala	Sanarate	2000	66.7%	73.9%	60.0%
Guatemala	Sanarate	2017	55.8%	63.4%	48.3%
Guatemala	Sansare	2000	71.5%	82.6%	56.7%
Guatemala	Sansare	2017	60.5%	72.6%	43.0%
Guatemala	Santa Ana	2000	52.2%	69.5%	35.6%
Guatemala	Santa Ana	2017	45.1%	63.6%	28.5%

Country	Second Administrative Unit	Year	Mean	Upper	Lower
Guatemala	Santa Ana Huista	2000	81.0%	96.3%	49.7%
Guatemala	Santa Ana Huista	2017	74.8%	93.7%	42.4%
Guatemala	Santa Apolonia	2000	88.2%	91.6%	76.8%
Guatemala	Santa Apolonia	2017	79.8%	84.7%	66.9%
Guatemala	Santa Bárbara	2000	82.6%	87.9%	75.2%
Guatemala	Santa Bárbara	2000	74.7%	95.9%	39.6%
Guatemala	Santa Bárbara	2017	74.6%	80.4%	66.4%
Guatemala	Santa Bárbara	2017	65.5%	91.5%	29.2%
Guatemala	Santa Catarina Barahona	2000	83.0%	87.2%	78.4%
Guatemala	Santa Catarina Barahona	2017	63.8%	71.0%	56.2%
Guatemala	Santa Catarina Ixtahuacan	2000	95.7%	97.1%	89.3%
Guatemala	Santa Catarina Ixtahuacan	2017	91.9%	94.2%	82.2%
Guatemala	Santa Catarina Mita	2000	83.8%	93.6%	67.3%
Guatemala	Santa Catarina Mita	2017	74.6%	88.2%	54.8%
Guatemala	Santa Catarina Palopó	2000	29.1%	60.7%	11.0%
Guatemala	Santa Catarina Palopó	2017	19.2%	43.3%	8.1%
Guatemala	Santa Catarina Pinula	2000	54.2%	60.4%	48.7%
Guatemala	Santa Catarina Pinula	2017	39.3%	44.5%	34.6%
Guatemala	Santa Clara La Laguna	2000	97.4%	98.7%	93.4%
Guatemala	Santa Clara La Laguna	2017	94.6%	97.2%	86.3%
Guatemala	Santa Cruz Balanyá	2000	82.5%	88.0%	75.4%
Guatemala	Santa Cruz Balanyá	2017	69.4%	77.2%	60.6%
Guatemala	Santa Cruz Barillas	2000	70.7%	84.3%	53.1%
Guatemala	Santa Cruz Barillas	2017	62.1%	77.8%	44.2%
Guatemala	Santa Cruz del Quiché	2000	51.0%	56.9%	44.0%
Guatemala	Santa Cruz del Quiché	2017	35.5%	40.9%	29.8%
Guatemala	Santa Cruz El Chol	2000	86.4%	99.1%	55.2%
Guatemala	Santa Cruz El Chol	2017	81.5%	98.2%	50.4%
Guatemala	Santa Cruz La Laguna	2000	70.8%	77.2%	64.6%
Guatemala	Santa Cruz La Laguna	2017	49.2%	55.1%	43.7%
Guatemala	Santa Cruz Muluá	2000	46.5%	52.2%	39.6%
Guatemala	Santa Cruz Muluá	2017	30.6%	35.6%	25.4%

Country	Second Administrative Unit	Year	Mean	Upper	Lower
Guatemala	Santa Cruz Naranjo	2000	81.7%	86.9%	75.1%
Guatemala	Santa Cruz Naranjo	2017	69.9%	76.4%	61.9%
Guatemala	Santa Cruz Verapaz	2000	78.1%	90.8%	57.8%
Guatemala	Santa Cruz Verapaz	2017	66.0%	82.8%	44.2%
Guatemala	Santa Eulalia	2000	73.2%	92.6%	46.4%
Guatemala	Santa Eulalia	2017	65.9%	88.3%	38.7%
Guatemala	Santa Lucía Cotzumalguapa	2000	54.0%	75.0%	32.9%
Guatemala	Santa Lucía Cotzumalguapa	2017	41.7%	63.1%	21.8%
Guatemala	Santa Lucía La Reforma	2000	48.7%	67.5%	31.3%
Guatemala	Santa Lucía La Reforma	2017	35.4%	51.7%	23.8%
Guatemala	Santa Lucía Milpas Altas	2000	86.7%	89.6%	83.5%
Guatemala	Santa Lucía Milpas Altas	2017	71.3%	77.1%	65.9%
Guatemala	Santa Lucía Utatlán	2000	93.0%	94.3%	91.6%
Guatemala	Santa Lucía Utatlán	2017	85.7%	88.2%	83.3%
Guatemala	Santa María Cahabón	2000	73.4%	93.5%	43.6%
Guatemala	Santa María Cahabón	2017	65.3%	88.7%	34.9%
Guatemala	Santa María Chiquimula	2000	87.9%	94.7%	73.9%
Guatemala	Santa María Chiquimula	2017	80.7%	90.1%	65.4%
Guatemala	Santa María de Jesús	2000	88.8%	93.3%	83.7%
Guatemala	Santa María de Jesús	2017	82.6%	88.1%	76.5%
Guatemala	Santa María Ixhuatán	2000	83.3%	94.5%	64.0%
Guatemala	Santa María Ixhuatán	2017	77.8%	90.9%	59.9%
Guatemala	Santa María Visitación	2000	98.6%	99.9%	93.4%
Guatemala	Santa María Visitación	2017	97.0%	99.8%	84.6%
Guatemala	Santa Rosa de Lima	2000	47.5%	73.5%	25.1%
Guatemala	Santa Rosa de Lima	2017	39.6%	64.2%	17.5%
Guatemala	Santiago Atitlán	2000	84.2%	88.3%	78.9%
Guatemala	Santiago Atitlán	2017	78.7%	83.2%	72.1%
Guatemala	Santiago Chimaltenango	2000	75.2%	98.5%	22.9%
Guatemala	Santiago Chimaltenango	2017	67.2%	97.0%	16.9%
Guatemala	Santiago Sacatepéquez	2000	82.9%	85.5%	80.5%

Country	Second Administrative Unit	Year	Mean	Upper	Lower
Guatemala	Santiago Sacatepéquez	2017	70.7%	74.3%	67.4%
Guatemala	Santo Domingo Suchitepequez	2000	45.9%	60.9%	29.6%
Guatemala	Santo Domingo Suchitepequez	2017	33.4%	47.4%	20.3%
Guatemala	Santo Domingo Xenacoj	2000	87.5%	90.1%	84.4%
Guatemala	Santo Domingo Xenacoj	2017	76.8%	81.2%	72.0%
Guatemala	Santo Tomás La Unión	2000	92.5%	94.4%	89.0%
Guatemala	Santo Tomás La Unión	2017	86.4%	89.5%	80.3%
Guatemala	Sayaxché	2000	66.9%	77.6%	53.3%
Guatemala	Sayaxché	2017	58.0%	69.7%	44.5%
Guatemala	Senahú	2000	73.4%	86.3%	54.4%
Guatemala	Senahú	2017	63.9%	80.3%	44.1%
Guatemala	Sibilia	2000	94.2%	97.5%	84.7%
Guatemala	Sibilia	2017	87.9%	94.1%	71.1%
Guatemala	Sipacapa	2000	74.8%	98.0%	41.4%
Guatemala	Sipacapa	2017	64.1%	95.8%	26.6%
Guatemala	Siquinalá	2000	60.3%	74.7%	43.4%
Guatemala	Siquinalá	2017	44.7%	61.2%	30.1%
Guatemala	Sololá	2000	71.7%	80.6%	56.6%
Guatemala	Sololá	2017	60.0%	70.1%	45.2%
Guatemala	Soloma	2000	89.8%	98.1%	73.2%
Guatemala	Soloma	2017	83.2%	95.5%	64.1%
Guatemala	Sumpango	2000	90.2%	91.6%	89.0%
Guatemala	Sumpango	2017	82.2%	84.2%	79.9%
Guatemala	Tacaná	2000	85.4%	97.1%	65.4%
Guatemala	Tacaná	2017	78.4%	94.6%	54.7%
Guatemala	Tactic	2000	83.7%	95.4%	64.5%
Guatemala	Tactic	2017	74.9%	90.7%	54.0%
Guatemala	Tajumulco	2000	86.5%	97.6%	62.8%
Guatemala	Tajumulco	2017	80.8%	95.7%	53.4%
Guatemala	Tamahú	2000	67.8%	98.0%	26.6%
Guatemala	Tamahú	2017	59.1%	96.2%	19.0%
Guatemala	Taxisco	2000	55.9%	77.3%	34.3%
Guatemala	Taxisco	2017	46.6%	69.2%	27.0%
Guatemala	Tecpán Guatemala	2000	77.3%	85.2%	67.5%
Guatemala	Tecpán Guatemala	2017	66.0%	76.8%	55.6%
Guatemala	Tectitán	2000	78.2%	97.2%	48.3%
Guatemala	Tectitán	2017	70.9%	94.6%	39.0%
Guatemala	Teculután	2000	55.6%	64.8%	45.4%
Guatemala	Teculután	2017	41.8%	52.9%	31.3%
Guatemala	Tejutla	2000	84.2%	93.5%	65.6%
Guatemala	Tejutla	2017	75.6%	88.8%	53.9%
Guatemala	Tiquisate	2000	62.1%	74.8%	47.1%
Guatemala	Tiquisate	2017	51.2%	65.1%	35.6%
Guatemala	Todos Santos Cuchumatán	2000	80.9%	97.7%	49.0%
Guatemala	Todos Santos Cuchumatán	2017	74.8%	95.8%	41.7%

Country	Second Administrative Unit	Year	Mean	Upper	Lower
Guatemala	Totonicapán	2000	93.1%	96.1%	86.7%
Guatemala	Totonicapán	2017	88.3%	92.2%	82.1%
Guatemala	Tucurú	2000	77.3%	96.2%	47.7%
Guatemala	Tucurú	2017	68.8%	93.2%	39.6%
Guatemala	Uspantán	2000	79.4%	93.0%	62.4%
Guatemala	Uspantán	2017	72.7%	88.3%	54.4%
Guatemala	Usumatlán	2000	76.2%	82.9%	66.2%
Guatemala	Usumatlán	2017	63.1%	71.6%	52.2%
Guatemala	Villa Canales	2000	67.8%	77.3%	52.3%
Guatemala	Villa Canales	2017	56.1%	68.5%	40.2%
Guatemala	Villa Nueva	2000	67.6%	69.6%	65.7%
Guatemala	Villa Nueva	2017	50.3%	52.2%	48.1%
Guatemala	Yepocapa	2000	89.5%	97.1%	77.3%
Guatemala	Yepocapa	2017	84.0%	94.7%	68.7%
Guatemala	Yupiltepeque	2000	90.8%	97.4%	65.6%
Guatemala	Yupiltepeque	2017	84.9%	94.9%	57.9%
Guatemala	Zacapa	2000	76.3%	82.3%	66.8%
Guatemala	Zacapa	2017	65.2%	73.3%	53.2%
Guatemala	Zacualpa	2000	76.7%	96.6%	45.7%
Guatemala	Zacualpa	2017	68.9%	94.5%	35.1%
Guatemala	Zapotitlán	2000	82.0%	99.1%	40.0%
Guatemala	Zapotitlán	2017	74.5%	98.3%	28.3%
Guatemala	Zaragoza	2000	83.2%	87.4%	75.2%
Guatemala	Zaragoza	2017	69.7%	75.7%	59.7%
Guatemala	ZONA 1	2000	56.1%	58.3%	53.5%
Guatemala	ZONA 1	2017	38.1%	40.2%	36.1%
Guatemala	ZONA 10	2000	49.4%	54.4%	45.0%
Guatemala	ZONA 10	2017	32.0%	35.9%	28.5%
Guatemala	ZONA 11	2000	46.4%	50.4%	42.6%
Guatemala	ZONA 11	2017	29.8%	32.7%	27.0%
Guatemala	ZONA 12	2000	58.9%	61.7%	55.8%
Guatemala	ZONA 12	2017	42.8%	45.3%	40.4%
Guatemala	ZONA 13	2000	49.6%	53.9%	45.3%
Guatemala	ZONA 13	2017	32.4%	35.5%	29.3%
Guatemala	ZONA 14	2000	48.2%	52.5%	43.7%
Guatemala	ZONA 14	2017	30.9%	34.4%	27.7%
Guatemala	ZONA 15	2000	55.0%	58.6%	51.3%
Guatemala	ZONA 15	2017	37.5%	40.4%	34.1%
Guatemala	ZONA 16	2000	67.0%	70.1%	63.8%
Guatemala	ZONA 16	2017	49.6%	53.3%	46.1%
Guatemala	ZONA 17	2000	62.5%	64.8%	60.1%
Guatemala	ZONA 17	2017	44.7%	47.1%	42.7%
Guatemala	ZONA 18	2000	61.0%	63.7%	58.6%
Guatemala	ZONA 18	2017	43.2%	45.4%	40.7%
Guatemala	ZONA 19	2000	44.9%	48.6%	41.8%
Guatemala	ZONA 19	2017	28.3%	30.8%	26.0%
Guatemala	ZONA 2	2000	59.3%	61.3%	57.2%
Guatemala	ZONA 2	2017	42.1%	44.2%	40.1%
Guatemala	ZONA 22	2000	68.3%	70.6%	66.2%
Guatemala	ZONA 22	2017	52.0%	54.0%	49.9%
Guatemala	ZONA 24	2000	66.8%	71.5%	61.7%
Guatemala	ZONA 24	2017	51.6%	56.4%	46.8%
Guatemala	ZONA 25	2000	65.8%	69.9%	61.2%
Guatemala	ZONA 25	2017	51.9%	56.1%	47.5%
Guatemala	ZONA 3	2000	54.7%	56.9%	52.0%
Guatemala	ZONA 3	2017	36.8%	38.7%	35.0%
Guatemala	ZONA 4	2000	50.2%	54.9%	45.9%
Guatemala	ZONA 4	2017	32.8%	36.5%	29.3%
Guatemala	ZONA 5	2000	57.1%	59.2%	54.4%
Guatemala	ZONA 5	2017	39.3%	41.1%	37.4%
Guatemala	ZONA 6	2000	61.0%	63.3%	58.7%
Guatemala	ZONA 6	2017	43.0%	45.1%	41.0%

Country	Second Administrative Unit	Year	Mean	Upper	Lower
Guatemala	ZONA 7	2000	49.0%	51.5%	46.1%
Guatemala	ZONA 7	2017	31.9%	33.8%	30.0%
Guatemala	ZONA 8	2000	50.1%	54.9%	45.8%
Guatemala	ZONA 8	2017	32.7%	36.4%	29.3%
Guatemala	ZONA 9	2000	49.7%	54.8%	45.3%
Guatemala	ZONA 9	2017	32.3%	36.3%	28.7%
Guatemala	Zunil	2000	88.0%	99.7%	45.7%
Guatemala	Zunil	2017	82.0%	99.5%	33.2%
Guatemala	Zunilito	2000	79.4%	96.9%	40.5%
Guatemala	Zunilito	2017	70.1%	94.6%	29.3%
Guyana	Abary / Mahaicony	2000	80.0%	96.2%	51.9%
Guyana	Abary / Mahaicony	2017	58.6%	88.1%	26.2%
Guyana	Agatash	2000	50.7%	54.8%	46.7%
Guyana	Agatash	2017	22.2%	26.0%	18.6%
Guyana	Aishalton - Karaudanawa, Achiwib	2000	24.2%	37.5%	13.6%
Guyana	Aishalton - Karaudanawa, Achiwib	2017	13.2%	23.0%	6.1%
Guyana	Amsterdam (Demerara River) / Vriesland	2000	49.5%	57.3%	44.0%
Guyana	Amsterdam (Demerara River) / Vriesland	2017	25.6%	33.1%	21.6%
Guyana	Anna Regina	2000	11.1%	15.6%	7.9%
Guyana	Anna Regina	2017	3.0%	4.4%	2.1%
Guyana	Arau	2000	31.0%	66.2%	7.3%
Guyana	Arau	2017	17.0%	44.9%	2.2%
Guyana	Barima / Amakura	2000	26.7%	36.2%	19.4%
Guyana	Barima / Amakura	2017	12.7%	19.8%	8.1%
Guyana	Bartica	2000	59.5%	64.6%	54.2%
Guyana	Bartica	2017	25.6%	30.2%	21.3%
Guyana	Bel Air / Woodlands	2000	74.9%	80.2%	66.8%
Guyana	Bel Air / Woodlands	2017	41.8%	49.5%	34.7%
Guyana	Berbice River Settlements	2000	30.9%	52.2%	17.0%
Guyana	Berbice River Settlements	2017	17.9%	31.6%	8.9%
Guyana	Black Bush Polder land Development Scheme	2000	79.7%	90.2%	56.6%
Guyana	Black Bush Polder land Development Scheme	2017	52.1%	67.4%	36.7%
Guyana	Blankenburg / Hague	2000	57.5%	62.1%	52.4%
Guyana	Blankenburg / Hague	2017	24.7%	28.5%	21.4%
Guyana	Bonasika / Boerasirie	2000	25.2%	33.6%	19.4%

Country	Second Administrative Unit	Year	Mean	Upper	Lower
Guyana	Bonasika / Boerasirie	2017	8.1%	12.7%	5.5%
Guyana	Borlam (No.37) / Kintyre	2000	55.9%	62.0%	50.3%
Guyana	Borlam (No.37) / Kintyre	2017	28.9%	34.5%	24.7%
Guyana	Bush Lot / Adventure	2000	85.1%	88.5%	81.0%
Guyana	Bush Lot / Adventure	2017	58.0%	64.9%	50.9%
Guyana	Canal No. 2 (part) + The Belle + Little Alliance	2000	56.7%	61.2%	51.1%
Guyana	Canal No. 2 (part) + The Belle + Little Alliance	2017	23.3%	26.4%	19.8%
Guyana	Canals Polder	2000	54.8%	59.1%	50.1%
Guyana	Canals Polder	2017	22.4%	25.5%	19.5%
Guyana	Cane Field / Enterprise	2000	59.8%	61.4%	58.2%
Guyana	Cane Field / Enterprise	2017	31.3%	33.7%	29.3%
Guyana	Cane Grove Land Development Scheme	2000	66.6%	70.9%	61.4%
Guyana	Cane Grove Land Development Scheme	2017	46.2%	54.2%	37.5%
Guyana	Chance / Hamlet	2000	78.0%	82.7%	73.2%
Guyana	Chance / Hamlet	2017	47.6%	53.4%	42.3%
Guyana	Charity / Urasara	2000	18.6%	31.4%	6.8%
Guyana	Charity / Urasara	2017	9.0%	18.9%	2.6%
Guyana	Chenapau River	2000	13.9%	42.1%	2.4%
Guyana	Chenapau River	2017	5.5%	26.2%	0.5%
Guyana	City of Georgetown	2000	33.5%	35.8%	31.4%
Guyana	City of Georgetown	2017	11.2%	11.9%	10.4%
Guyana	Coomaka Lands	2000	53.6%	66.9%	41.5%
Guyana	Coomaka Lands	2017	27.5%	41.3%	16.6%
Guyana	Corentyne River	2000	68.3%	71.0%	64.5%
Guyana	Corentyne River	2017	42.3%	47.9%	37.5%
Guyana	Cornelia Ida / Stewartville	2000	53.9%	57.9%	49.6%
Guyana	Cornelia Ida / Stewartville	2017	22.6%	25.7%	19.7%

Country	Second Administrative Unit	Year	Mean	Upper	Lower
Guyana	Corriverton	2000	65.9%	88.0%	26.7%
Guyana	Corriverton	2017	42.9%	80.2%	9.4%
Guyana	Demerara Conservancy	2000	59.2%	70.6%	46.5%
Guyana	Demerara Conservancy	2017	33.1%	47.4%	21.3%
Guyana	Diamond / Golden Grove	2000	53.3%	58.6%	48.2%
Guyana	Diamond / Golden Grove	2017	22.5%	28.6%	17.8%
Guyana	East Bank Berbice	2000	22.8%	31.5%	15.5%
Guyana	East Bank Berbice	2017	12.1%	17.5%	7.4%
Guyana	Eccles / Ramsburg	2000	57.2%	60.8%	53.5%
Guyana	Eccles / Ramsburg	2017	23.4%	25.8%	21.1%
Guyana	Enfield / New Doe Park	2000	80.9%	91.5%	69.0%
Guyana	Enfield / New Doe Park	2017	55.9%	74.0%	41.5%
Guyana	Enmore / Hope	2000	55.3%	60.8%	50.7%
Guyana	Enmore / Hope	2017	25.5%	30.6%	21.7%
Guyana	Farm / Woodlands	2000	37.3%	56.4%	26.5%
Guyana	Farm / Woodlands	2017	24.1%	38.2%	17.2%
Guyana	Foulis / Buxton	2000	43.5%	47.6%	39.6%
Guyana	Foulis / Buxton	2017	18.8%	21.3%	16.8%
Guyana	Fyrish / Gibraltar	2000	22.6%	34.5%	13.6%
Guyana	Fyrish / Gibraltar	2017	6.4%	10.3%	3.7%
Guyana	Gelderland / No. 3	2000	77.7%	82.2%	70.9%
Guyana	Gelderland / No. 3	2017	46.4%	53.3%	39.3%
Guyana	Good Hope / Hydronie	2000	35.9%	45.3%	28.7%
Guyana	Good Hope / Hydronie	2017	11.4%	15.9%	8.5%
Guyana	Good Hope / Pomona	2000	5.1%	10.3%	1.7%
Guyana	Good Hope / Pomona	2017	2.4%	9.0%	0.5%
Guyana	Good Success / Caledonia	2000	49.3%	59.6%	38.7%
Guyana	Good Success / Caledonia	2017	21.6%	31.9%	14.4%
Guyana	Grove / Haslington	2000	51.4%	57.6%	45.4%
Guyana	Grove / Haslington	2017	20.9%	25.9%	17.2%
Guyana	Hampshire / Kilcoy	2000	85.7%	90.4%	80.1%
Guyana	Hampshire / Kilcoy	2017	59.7%	71.4%	48.3%

Country	Second Administrative Unit	Year	Mean	Upper	Lower
Guyana	Herstelling / Little Diamond	2000	51.0%	54.5%	47.2%
Guyana	Herstelling / Little Diamond	2017	19.5%	21.5%	17.5%
Guyana	Hogstye / Lancaster	2000	88.7%	90.5%	86.6%
Guyana	Hogstye / Lancaster	2017	65.7%	70.2%	61.0%
Guyana	Ireng / Sawariwau (Including St. Ignatius)	2000	43.5%	52.8%	33.3%
Guyana	Ireng / Sawariwau (Including St. Ignatius)	2017	21.0%	29.3%	13.8%
Guyana	Ituni	2000	30.7%	62.4%	11.7%
Guyana	Ituni	2017	15.9%	43.5%	3.9%
Guyana	Jackson Creek / Crabwood Creek	2000	46.1%	60.9%	33.3%
Guyana	Jackson Creek / Crabwood Creek	2017	27.4%	39.6%	14.3%
Guyana	Jawalla, Kubenang River	2000	19.9%	40.6%	3.1%
Guyana	Jawalla, Kubenang River	2017	9.5%	26.7%	0.7%
Guyana	John / Port Mourant	2000	85.9%	90.2%	80.8%
Guyana	John / Port Mourant	2017	61.7%	70.2%	54.1%
Guyana	Joppa / Macedonia	2000	85.3%	87.4%	82.9%
Guyana	Joppa / Macedonia	2017	57.4%	61.8%	53.0%
Guyana	Kaibarupai	2000	26.7%	59.1%	8.1%
Guyana	Kaibarupai	2017	11.5%	37.9%	2.2%
Guyana	Kamarang	2000	23.0%	38.7%	10.9%
Guyana	Kamarang	2017	11.3%	22.8%	3.8%
Guyana	Karambaru to Kukui River + Phillipi	2000	19.7%	35.9%	8.6%
Guyana	Karambaru to Kukui River + Phillipi	2017	9.4%	19.5%	2.9%
Guyana	Klein Pouderoyen / Best	2000	44.1%	49.2%	39.7%
Guyana	Klein Pouderoyen / Best	2017	15.1%	17.8%	13.0%
Guyana	Kopanang, Waipa, Kenepai	2000	27.8%	49.7%	10.8%
Guyana	Kopanang, Waipa, Kenepai	2017	17.6%	34.8%	9.2%

Country	Second Administrative Unit	Year	Mean	Upper	Lower
Guyana	Kwakwani	2000	45.5%	79.7%	20.6%
Guyana	Kwakwani	2017	30.1%	61.8%	14.0%
Guyana	La Bonne Intention / Better Hope	2000	44.0%	47.0%	41.1%
Guyana	La Bonne Intention / Better Hope	2017	16.6%	18.7%	14.8%
Guyana	La Reconnaissance / Mon Repos	2000	43.2%	46.4%	40.2%
Guyana	La Reconnaissance / Mon Repos	2017	16.8%	18.8%	14.9%
Guyana	Leguan (Essequibo Islands)	2000	38.1%	69.6%	14.0%
Guyana	Leguan (Essequibo Islands)	2017	16.4%	45.0%	4.0%
Guyana	Linden	2000	76.2%	80.1%	72.7%
Guyana	Linden	2017	46.5%	51.2%	42.5%
Guyana	Lower West Demerara	2000	29.6%	62.3%	8.1%
Guyana	Lower West Demerara	2017	15.8%	44.8%	2.5%
Guyana	Mabaruma / Kumaka / Hosororo	2000	41.0%	52.9%	30.7%
Guyana	Mabaruma / Kumaka / Hosororo	2017	20.3%	34.0%	11.9%
Guyana	Mabura Hills	2000	33.6%	54.3%	16.8%
Guyana	Mabura Hills	2017	19.4%	34.1%	8.5%
Guyana	Madhia + Kurubrong River + Mona Falls	2000	21.4%	33.5%	12.5%
Guyana	Madhia + Kurubrong River + Mona Falls	2017	10.1%	19.4%	4.4%
Guyana	Makouria River	2000	60.1%	65.4%	54.7%
Guyana	Makouria River	2017	25.9%	30.5%	21.6%
Guyana	Maripari River + Kurukabaru	2000	23.6%	40.7%	10.5%
Guyana	Maripari River + Kurukabaru	2017	12.9%	26.3%	4.6%
Guyana	Marudi	2000	23.5%	46.2%	9.0%
Guyana	Marudi	2017	11.8%	28.6%	3.1%
Guyana	Matthews Ridge / Arakaka (Matakai) / Port Kaituma	2000	22.1%	43.4%	8.0%
Guyana	Matthews Ridge / Arakaka (Matakai) / Port Kaituma	2017	10.4%	24.2%	2.8%

Country	Second Administrative Unit	Year	Mean	Upper	Lower
Guyana	Meer Zorgen / Malgre Tout	2000	51.8%	54.9%	49.3%
Guyana	Meer Zorgen / Malgre Tout	2017	20.2%	21.7%	18.7%
Guyana	Mocha / Arcadia	2000	66.9%	74.7%	57.8%
Guyana	Mocha / Arcadia	2017	31.8%	39.3%	23.7%
Guyana	Monkey Mountain	2000	39.5%	74.7%	12.0%
Guyana	Monkey Mountain	2017	22.5%	57.8%	4.1%
Guyana	Naarstigheid / Union	2000	81.7%	86.7%	73.4%
Guyana	Naarstigheid / Union	2017	55.1%	62.1%	47.1%
Guyana	New Amsterdam	2000	78.2%	80.5%	75.4%
Guyana	New Amsterdam	2017	45.1%	48.9%	41.1%
Guyana	Nismes / La Grange	2000	59.4%	61.9%	56.8%
Guyana	Nismes / La Grange	2017	25.0%	26.8%	23.3%
Guyana	No. 38 / Ordnance Fortlands	2000	66.2%	68.6%	63.6%
Guyana	No. 38 / Ordnance Fortlands	2017	34.5%	37.2%	31.9%
Guyana	No.51 Village / Good Hope	2000	85.9%	89.1%	82.1%
Guyana	No.51 Village / Good Hope	2017	58.8%	65.5%	52.1%
Guyana	No.74 Village / No.52 Village	2000	83.3%	89.0%	73.9%
Guyana	No.74 Village / No.52 Village	2017	56.9%	66.4%	46.8%
Guyana	Nouvelle Flanders / La Jalousie	2000	53.9%	58.2%	49.2%
Guyana	Nouvelle Flanders / La Jalousie	2017	22.5%	27.3%	18.0%
Guyana	Paradise / Evergreen (including Somerset and Berks)	2000	20.3%	29.4%	13.4%
Guyana	Paradise / Evergreen (including Somerset and Berks)	2017	6.2%	11.6%	3.3%
Guyana	Paramakatoi	2000	25.9%	44.6%	10.8%
Guyana	Paramakatoi	2017	10.6%	28.0%	3.3%
Guyana	Parika / Mora	2000	9.5%	21.6%	4.3%
Guyana	Parika / Mora	2017	2.5%	5.9%	1.2%
Guyana	Paruima	2000	26.2%	41.7%	10.9%
Guyana	Paruima	2017	13.5%	26.8%	4.3%

Country	Second Administrative Unit	Year	Mean	Upper	Lower
Guyana	Patentia / Toevlugt	2000	50.7%	54.3%	46.9%
Guyana	Patentia / Toevlugt	2017	18.9%	21.1%	17.0%
Guyana	Plaisance / Industry	2000	42.4%	45.4%	39.5%
Guyana	Plaisance / Industry	2017	16.2%	18.3%	14.2%
Guyana	Rest of Region 1	2000	26.1%	38.9%	14.0%
Guyana	Rest of Region 1	2017	13.7%	24.9%	5.8%
Guyana	Rest of Region 10	2000	32.9%	47.4%	21.1%
Guyana	Rest of Region 10	2017	18.3%	27.3%	11.1%
Guyana	Rest of Region 7	2000	27.1%	37.7%	19.3%
Guyana	Rest of Region 7	2017	13.8%	18.1%	10.5%
Guyana	Rest of Region 8	2000	22.8%	28.8%	16.4%
Guyana	Rest of Region 8	2017	12.0%	16.1%	8.0%
Guyana	Rest of Region 9	2000	29.9%	39.4%	21.5%
Guyana	Rest of Region 9	2017	16.4%	23.7%	10.7%
Guyana	Rising Sun / Profit	2000	72.2%	81.6%	59.6%
Guyana	Rising Sun / Profit	2017	53.5%	65.1%	41.1%
Guyana	Riverstown / Annandale	2000	6.0%	10.3%	2.8%
Guyana	Riverstown / Annandale	2017	2.9%	6.7%	0.9%
Guyana	Rose Hall	2000	86.1%	90.4%	80.7%
Guyana	Rose Hall	2017	60.6%	71.1%	50.3%
Guyana	Rosignol / Zeelust	2000	80.4%	82.5%	78.0%
Guyana	Rosignol / Zeelust	2017	48.7%	52.3%	45.2%
Guyana	Sand Creek - Dadanawa, Catunarib, Sawariwau	2000	30.8%	46.3%	18.0%
Guyana	Sand Creek - Dadanawa, Catunarib, Sawariwau	2017	17.5%	29.5%	8.2%
Guyana	Soesdyke-Linden highway (including Timehri)	2000	31.5%	43.7%	20.5%
Guyana	Soesdyke-Linden highway (including Timehri)	2017	14.2%	23.9%	7.4%

Piped Water Proportion by Second Administrative Unit *(continued)*

Country	Second Administrative Unit	Year	Mean	Upper	Lower
Guyana	Sparta / Bonasika and Rest of Essequibo Islands	2000	21.4%	47.8%	4.3%
Guyana	Sparta / Bonasika and Rest of Essequibo Islands	2017	10.3%	29.9%	1.0%
Guyana	St. Cuthberts / Orange Nassau (Mahaica River)	2000	51.2%	61.9%	40.7%
Guyana	St. Cuthberts / Orange Nassau (Mahaica River)	2017	30.3%	41.9%	19.1%
Guyana	St. Francis Mission	2000	36.9%	56.8%	17.3%
Guyana	St. Francis Mission	2017	20.9%	36.5%	6.8%
Guyana	Supernaam River, Bethany and Mashabo Villages	2000	32.4%	58.6%	12.8%
Guyana	Supernaam River, Bethany and Mashabo Villages	2017	16.7%	41.8%	4.9%
Guyana	Tarlogie / Maida	2000	84.4%	86.7%	82.2%
Guyana	Tarlogie / Maida	2017	56.1%	60.6%	51.4%
Guyana	Te Huist Coverden / Soesdyke	2000	34.8%	48.1%	23.1%
Guyana	Te Huist Coverden / Soesdyke	2017	12.5%	22.8%	7.1%
Guyana	Tempe / Seafield	2000	94.7%	96.7%	91.6%
Guyana	Tempe / Seafield	2017	82.8%	87.7%	75.8%
Guyana	Toka - Jakaretinga	2000	36.1%	56.0%	17.6%
Guyana	Toka - Jakaretinga	2017	21.7%	37.8%	7.6%
Guyana	Triumph / Beterverwagting	2000	41.7%	45.1%	38.7%
Guyana	Triumph / Beterverwagting	2017	15.3%	17.4%	13.6%
Guyana	Uitvlugt / Tuschen	2000	31.9%	36.5%	27.7%
Guyana	Uitvlugt / Tuschen	2017	9.6%	12.2%	7.8%
Guyana	Vereeniging / Unity	2000	64.9%	69.1%	60.2%

Country	Second Administrative Unit	Year	Mean	Upper	Lower
Guyana	Vereeniging / Unity	2017	30.4%	34.5%	26.5%
Guyana	Vergenoegen / Greenwich Park	2000	26.4%	34.4%	20.3%
Guyana	Vergenoegen / Greenwich Park	2017	8.2%	11.4%	5.9%
Guyana	Waini	2000	28.8%	40.8%	18.1%
Guyana	Waini	2017	16.0%	25.5%	8.3%
Guyana	Wakenaam (Essequibo Islands)	2000	14.5%	40.3%	1.4%
Guyana	Wakenaam (Essequibo Islands)	2017	5.9%	24.5%	0.4%
Guyana	Waramadan	2000	22.8%	47.2%	7.8%
Guyana	Waramadan	2017	8.6%	21.7%	2.2%
Guyana	West bank Berbice	2000	76.3%	79.7%	72.7%
Guyana	West bank Berbice	2017	45.2%	49.9%	40.3%
Guyana	Whim / Bloomfield	2000	87.8%	90.3%	84.0%
Guyana	Whim / Bloomfield	2017	64.8%	69.0%	59.6%
Guyana	Woodley Park / Bath	2000	73.3%	78.9%	64.0%
Guyana	Woodley Park / Bath	2017	39.4%	46.8%	32.2%
Guyana	Yakarinta - Wowetta, Surama	2000	29.2%	42.5%	17.5%
Guyana	Yakarinta - Wowetta, Surama	2017	16.6%	27.2%	8.0%
Guyana	Yarong Paru - Good Hope	2000	28.0%	44.5%	13.7%
Guyana	Yarong Paru - Good Hope	2017	15.0%	28.8%	5.6%
Guyana	Zorg-en-Vlygt / Aberdeen	2000	6.8%	21.5%	1.6%
Guyana	Zorg-en-Vlygt / Aberdeen	2017	2.1%	8.4%	0.3%
Haiti	Anse d'Hainault	2000	37.4%	41.0%	34.1%
Haiti	Anse d'Hainault	2017	19.3%	22.1%	16.5%
Haiti	Aquin	2000	54.1%	57.8%	49.5%
Haiti	Aquin	2017	36.8%	40.5%	31.9%
Haiti	Bainet	2000	34.1%	37.7%	29.7%
Haiti	Bainet	2017	24.7%	29.3%	18.2%
Haiti	Belle-Anse	2000	57.7%	62.9%	51.1%
Haiti	Belle-Anse	2017	46.4%	52.4%	37.6%
Haiti	Borgne	2000	31.1%	45.9%	23.1%
Haiti	Borgne	2017	21.5%	33.2%	15.7%
Haiti	Cerca La Source	2000	29.8%	41.5%	19.3%
Haiti	Cerca La Source	2017	16.8%	27.8%	7.8%
Haiti	Corail	2000	16.3%	21.2%	13.9%
Haiti	Corail	2017	8.1%	11.2%	6.9%

Country	Second Administrative Unit	Year	Mean	Upper	Lower
Haiti	Croix-des-Bouquets	2000	28.0%	31.3%	25.0%
Haiti	Croix-des-Bouquets	2017	11.9%	14.6%	9.9%
Haiti	Dessalines	2000	25.9%	30.1%	22.8%
Haiti	Dessalines	2017	11.5%	14.3%	9.2%
Haiti	Fort-Liberté	2000	40.1%	48.7%	33.5%
Haiti	Fort-Liberté	2017	18.5%	25.2%	15.0%
Haiti	Grande-Rivière du Nord	2000	21.1%	23.6%	18.8%
Haiti	Grande-Rivière du Nord	2017	8.9%	10.1%	7.8%
Haiti	Gros-Morne	2000	30.7%	39.1%	20.5%
Haiti	Gros-Morne	2017	25.6%	38.2%	9.5%
Haiti	Hinche	2000	39.9%	45.0%	35.4%
Haiti	Hinche	2017	23.8%	27.6%	19.8%
Haiti	Jacmel	2000	54.8%	56.7%	52.2%
Haiti	Jacmel	2017	32.8%	36.0%	29.0%
Haiti	Jérémie	2000	28.4%	30.5%	26.8%
Haiti	Jérémie	2017	15.7%	17.8%	14.1%
Haiti	l'Acul-du-Nord	2000	44.1%	45.8%	42.6%
Haiti	l'Acul-du-Nord	2017	22.8%	24.1%	21.6%
Haiti	l'Anse-à-Veau	2000	30.9%	32.6%	29.5%
Haiti	l'Anse-à-Veau	2017	14.2%	15.3%	13.2%
Haiti	l'Arcahaie	2000	39.9%	55.8%	25.8%
Haiti	l'Arcahaie	2017	24.6%	38.1%	14.8%
Haiti	La Gonâve	2000	29.2%	47.7%	15.8%
Haiti	La Gonâve	2017	18.5%	38.9%	7.1%
Haiti	Lascahobas	2000	43.1%	46.7%	39.3%
Haiti	Lascahobas	2017	27.7%	30.9%	24.3%
Haiti	le Cap-Haïtien	2000	41.3%	42.5%	39.9%
Haiti	le Cap-Haïtien	2017	22.0%	23.0%	20.9%
Haiti	le Limbé	2000	67.6%	76.4%	61.4%
Haiti	le Limbé	2017	59.7%	64.9%	55.8%
Haiti	le Trou-du-Nord	2000	29.0%	33.1%	25.7%
Haiti	le Trou-du-Nord	2017	18.2%	21.5%	16.0%
Haiti	Léogâne	2000	45.5%	51.9%	41.5%
Haiti	Léogâne	2017	40.5%	45.5%	36.5%
Haiti	les Cayes	2000	50.2%	53.1%	45.6%
Haiti	les Cayes	2017	33.2%	35.7%	29.2%
Haiti	les Chardonnières	2000	43.5%	50.6%	38.7%
Haiti	les Chardonnières	2017	29.7%	35.1%	25.2%
Haiti	les Côteaux	2000	49.8%	63.1%	32.1%
Haiti	les Côteaux	2017	33.9%	52.4%	15.8%
Haiti	les Gonaïves	2000	23.8%	29.3%	18.5%
Haiti	les Gonaïves	2017	11.8%	16.9%	7.1%
Haiti	Marmelade	2000	26.0%	40.0%	17.0%
Haiti	Marmelade	2017	13.5%	26.4%	6.7%
Haiti	Miragoâne	2000	64.9%	66.4%	63.5%
Haiti	Miragoâne	2017	42.6%	44.1%	41.3%
Haiti	Mirebalais	2000	34.9%	40.9%	30.0%
Haiti	Mirebalais	2017	16.6%	21.6%	12.9%
Haiti	Môle Saint-Nicolas	2000	47.6%	53.3%	41.4%

Country	Second Administrative Unit	Year	Mean	Upper	Lower
Haiti	Môle Saint-Nicolas	2017	27.6%	33.4%	22.0%
Haiti	Ouanaminthe	2000	30.5%	35.2%	28.5%
Haiti	Ouanaminthe	2017	11.1%	14.8%	10.1%
Haiti	Plaisance	2000	39.8%	55.0%	29.0%
Haiti	Plaisance	2017	20.6%	38.4%	13.0%
Haiti	Port-au-Prince	2000	62.8%	63.7%	62.0%
Haiti	Port-au-Prince	2017	32.2%	32.9%	31.4%
Haiti	Port-de-Paix	2000	51.9%	55.5%	49.6%
Haiti	Port-de-Paix	2017	32.5%	35.1%	30.8%
Haiti	Port-Salut	2000	45.2%	57.3%	32.8%
Haiti	Port-Salut	2017	26.3%	39.4%	13.6%
Haiti	Saint-Louis du Nord	2000	55.0%	57.5%	53.2%
Haiti	Saint-Louis du Nord	2017	28.6%	30.8%	27.3%
Haiti	Saint-Marc	2000	48.3%	53.0%	42.5%
Haiti	Saint-Marc	2017	30.4%	34.5%	25.6%
Haiti	Saint-Raphaël	2000	34.0%	42.5%	30.0%
Haiti	Saint-Raphaël	2017	16.1%	21.9%	13.7%
Haiti	Vallières	2000	30.5%	39.0%	23.4%
Haiti	Vallières	2017	13.3%	21.7%	8.0%
Honduras	Aguaqueterique	2000	73.0%	94.2%	39.3%
Honduras	Aguaqueterique	2017	52.5%	82.7%	22.8%
Honduras	Ahuas	2000	58.6%	87.1%	26.8%
Honduras	Ahuas	2017	43.3%	77.3%	15.0%
Honduras	Ajuterique	2000	84.8%	87.6%	81.9%
Honduras	Ajuterique	2017	66.9%	71.1%	59.5%
Honduras	Alauca	2000	80.4%	92.2%	57.3%
Honduras	Alauca	2017	62.2%	81.5%	34.4%
Honduras	Alianza	2000	82.8%	91.9%	72.1%
Honduras	Alianza	2017	63.7%	77.4%	50.0%
Honduras	Alubarén	2000	92.8%	97.3%	84.8%
Honduras	Alubarén	2017	80.7%	89.7%	70.1%
Honduras	Amapala	2000	86.0%	94.0%	74.3%
Honduras	Amapala	2017	66.0%	80.1%	49.9%
Honduras	Apacilagua	2000	58.6%	85.5%	27.0%
Honduras	Apacilagua	2017	40.8%	70.1%	13.6%
Honduras	Arada	2000	56.0%	77.4%	29.2%
Honduras	Arada	2017	43.2%	75.1%	17.6%
Honduras	Aramecina	2000	81.5%	98.6%	54.0%
Honduras	Aramecina	2017	68.6%	94.5%	39.8%
Honduras	Arenal	2000	65.4%	93.8%	27.5%
Honduras	Arenal	2017	49.6%	85.6%	13.1%
Honduras	Arizona	2000	57.4%	81.3%	35.7%
Honduras	Arizona	2017	37.1%	60.0%	16.8%
Honduras	Atima	2000	64.1%	85.9%	39.3%
Honduras	Atima	2017	44.0%	67.6%	24.5%
Honduras	Azacualpa	2000	72.1%	86.7%	52.9%
Honduras	Azacualpa	2017	44.2%	64.7%	25.0%
Honduras	Balfate	2000	60.6%	77.2%	42.1%
Honduras	Balfate	2017	44.5%	63.5%	27.5%
Honduras	Belen	2000	84.1%	97.4%	57.7%
Honduras	Belen	2017	74.2%	95.2%	47.1%
Honduras	Belén Gualcho	2000	85.0%	91.4%	74.8%
Honduras	Belén Gualcho	2017	59.9%	72.6%	47.2%
Honduras	Bonito Oriental	2000	80.5%	89.8%	68.7%

Country	Second Administrative Unit	Year	Mean	Upper	Lower
Honduras	Bonito Oriental	2017	62.5%	74.3%	48.8%
Honduras	Brus Laguna	2000	61.5%	75.4%	45.1%
Honduras	Brus Laguna	2017	45.2%	60.3%	29.8%
Honduras	Cabañas	2000	85.2%	98.8%	62.3%
Honduras	Cabañas	2000	73.6%	91.2%	51.5%
Honduras	Cabañas	2017	74.7%	95.7%	48.0%
Honduras	Cabañas	2017	50.0%	74.3%	25.2%
Honduras	Camasca	2000	98.8%	99.5%	94.6%
Honduras	Camasca	2017	95.7%	97.8%	90.7%
Honduras	Campamento	2000	65.2%	87.1%	37.5%
Honduras	Campamento	2017	44.4%	71.1%	19.2%
Honduras	Candelaria	2000	49.4%	72.3%	25.4%
Honduras	Candelaria	2017	24.3%	46.7%	8.2%
Honduras	Cane	2000	74.8%	77.2%	71.8%
Honduras	Cane	2017	44.5%	47.8%	40.1%
Honduras	Caridad	2000	92.2%	96.4%	85.4%
Honduras	Caridad	2017	78.4%	86.3%	66.7%
Honduras	Catacamas	2000	61.6%	68.2%	54.0%
Honduras	Catacamas	2017	44.1%	50.6%	36.9%
Honduras	Cedros	2000	70.5%	86.4%	47.2%
Honduras	Cedros	2017	53.3%	72.9%	28.2%
Honduras	Ceguaca	2000	74.7%	89.5%	47.3%
Honduras	Ceguaca	2017	57.1%	80.0%	25.9%
Honduras	Chinacla	2000	98.1%	98.4%	97.7%
Honduras	Chinacla	2017	93.2%	94.1%	91.9%
Honduras	Chinda	2000	61.7%	84.1%	31.7%
Honduras	Chinda	2017	43.2%	73.9%	21.3%
Honduras	Choloma	2000	45.6%	49.8%	40.9%
Honduras	Choloma	2017	23.0%	26.0%	19.0%
Honduras	Choluteca	2000	75.6%	84.6%	65.6%
Honduras	Choluteca	2017	60.4%	68.4%	52.2%
Honduras	Cololaca	2000	86.4%	96.0%	72.4%
Honduras	Cololaca	2017	71.1%	85.7%	54.4%
Honduras	Colomoncagua	2000	75.4%	89.3%	53.7%
Honduras	Colomoncagua	2017	58.4%	78.5%	36.4%
Honduras	Comayagua	2000	79.4%	82.8%	74.6%
Honduras	Comayagua	2017	54.6%	59.7%	48.6%
Honduras	Concepción	2000	82.1%	93.3%	69.6%
Honduras	Concepción	2000	55.6%	59.9%	52.2%
Honduras	Concepción	2000	54.2%	71.4%	26.3%
Honduras	Concepción	2017	31.8%	54.6%	9.0%
Honduras	Concepción	2017	46.5%	50.6%	44.4%
Honduras	Concepción	2017	64.9%	78.2%	53.0%
Honduras	Concepción de Maria	2000	92.7%	96.3%	85.2%
Honduras	Concepción de Maria	2017	83.6%	90.0%	72.8%
Honduras	Concepción del Norte	2000	42.5%	78.8%	9.6%
Honduras	Concepción del Norte	2017	28.9%	70.0%	3.6%
Honduras	Concepción del Sur	2000	59.9%	89.9%	12.4%
Honduras	Concepción del Sur	2017	41.9%	75.3%	3.5%
Honduras	Concordia	2000	67.9%	95.2%	28.4%
Honduras	Concordia	2017	51.8%	88.6%	15.1%
Honduras	Copán Ruinas	2000	72.8%	84.8%	58.4%
Honduras	Copán Ruinas	2017	53.9%	70.2%	41.6%
Honduras	Corquín	2000	76.6%	90.4%	54.0%
Honduras	Corquín	2017	51.5%	73.6%	29.5%

Country	Second Administrative Unit	Year	Mean	Upper	Lower
Honduras	Cucuyagua	2000	60.9%	68.1%	52.7%
Honduras	Cucuyagua	2017	48.8%	57.7%	39.5%
Honduras	Curarén	2000	79.9%	96.5%	53.0%
Honduras	Curarén	2017	63.5%	86.6%	35.7%
Honduras	Danlí	2000	70.7%	80.1%	60.0%
Honduras	Danlí	2017	47.1%	58.9%	35.5%
Honduras	Distrito Central	2000	73.5%	78.9%	67.7%
Honduras	Distrito Central	2017	48.1%	53.9%	41.7%
Honduras	Dolores	2000	89.0%	93.2%	77.4%
Honduras	Dolores	2000	76.5%	94.3%	51.4%
Honduras	Dolores	2017	68.9%	75.9%	59.4%
Honduras	Dolores	2017	53.3%	77.7%	34.3%
Honduras	Dolores Merendon	2000	74.2%	94.9%	44.9%
Honduras	Dolores Merendon	2017	51.3%	80.2%	23.0%
Honduras	Dulce Nombre	2000	84.2%	90.9%	79.6%
Honduras	Dulce Nombre	2017	61.1%	68.8%	53.4%
Honduras	Dulce Nombre de Culmí	2000	69.4%	82.6%	54.0%
Honduras	Dulce Nombre de Culmí	2017	54.2%	70.1%	37.1%
Honduras	Duyure	2000	70.8%	96.8%	20.7%
Honduras	Duyure	2017	52.5%	93.4%	6.9%
Honduras	El Corpus	2000	76.8%	86.4%	60.6%
Honduras	El Corpus	2017	59.2%	71.7%	43.3%
Honduras	El Negrito	2000	74.7%	85.9%	60.4%
Honduras	El Negrito	2017	52.5%	69.0%	35.6%
Honduras	El Nispero	2000	85.5%	92.4%	64.8%
Honduras	El Nispero	2017	78.3%	89.3%	50.4%
Honduras	El Paraíso	2000	79.6%	87.8%	67.2%
Honduras	El Paraíso	2000	61.5%	76.8%	43.1%
Honduras	El Paraíso	2017	58.0%	70.0%	44.5%
Honduras	El Paraíso	2017	38.1%	53.9%	24.2%
Honduras	El Porvenir	2000	78.8%	95.0%	55.9%
Honduras	El Porvenir	2000	74.6%	86.1%	58.9%
Honduras	El Porvenir	2017	62.6%	85.0%	37.7%
Honduras	El Porvenir	2017	45.8%	59.7%	30.2%
Honduras	El Progreso	2000	65.9%	73.8%	56.3%
Honduras	El Progreso	2017	37.1%	45.8%	29.2%
Honduras	El Rosario	2000	87.1%	98.3%	67.0%
Honduras	El Rosario	2000	89.7%	99.7%	63.8%
Honduras	El Rosario	2017	78.9%	98.4%	45.6%
Honduras	El Rosario	2017	75.1%	93.5%	51.3%
Honduras	El Triunfo	2000	65.5%	87.7%	41.4%
Honduras	El Triunfo	2017	41.7%	64.6%	24.8%
Honduras	Erandique	2000	80.0%	90.9%	60.5%
Honduras	Erandique	2017	57.0%	74.1%	36.3%
Honduras	Esparta	2000	76.1%	92.7%	54.2%
Honduras	Esparta	2017	60.4%	81.5%	37.5%
Honduras	Esquías	2000	81.0%	97.0%	49.7%
Honduras	Esquías	2017	68.4%	91.8%	33.9%
Honduras	Esquipulas del Norte	2000	72.5%	92.7%	50.3%
Honduras	Esquipulas del Norte	2017	58.1%	83.1%	34.0%
Honduras	Florida	2000	76.7%	89.0%	57.5%
Honduras	Florida	2017	55.1%	71.2%	36.2%
Honduras	Fraternidad	2000	84.5%	91.4%	72.6%
Honduras	Fraternidad	2017	62.4%	75.7%	46.6%

Country	Second Administrative Unit	Year	Mean	Upper	Lower
Honduras	Goascorán	2000	64.8%	71.1%	58.9%
Honduras	Goascorán	2017	45.2%	51.8%	38.3%
Honduras	Gracias	2000	84.8%	90.1%	78.7%
Honduras	Gracias	2017	72.5%	79.0%	63.2%
Honduras	Guaimaca	2000	61.8%	77.6%	43.9%
Honduras	Guaimaca	2017	41.1%	60.5%	20.9%
Honduras	Guajiquiro	2000	85.6%	97.6%	68.4%
Honduras	Guajiquiro	2017	71.7%	90.5%	52.3%
Honduras	Gualaco	2000	65.5%	84.8%	41.6%
Honduras	Gualaco	2017	50.5%	73.8%	29.2%
Honduras	Gualala	2000	78.1%	97.7%	39.3%
Honduras	Gualala	2017	60.6%	91.6%	27.9%
Honduras	Gualcince	2000	73.9%	84.5%	48.9%
Honduras	Gualcince	2017	58.4%	76.8%	29.4%
Honduras	Guanaja	2000	72.2%	85.9%	50.7%
Honduras	Guanaja	2017	44.0%	59.9%	25.3%
Honduras	Guarita	2000	75.1%	98.3%	36.9%
Honduras	Guarita	2017	59.4%	92.9%	21.3%
Honduras	Guarizama	2000	70.8%	97.1%	27.9%
Honduras	Guarizama	2017	58.3%	92.7%	16.7%
Honduras	Guata	2000	87.5%	96.0%	75.7%
Honduras	Guata	2017	77.9%	89.9%	62.9%
Honduras	Guayape	2000	76.2%	97.8%	36.7%
Honduras	Guayape	2017	62.0%	92.9%	22.8%
Honduras	Guinope	2000	79.3%	99.0%	41.8%
Honduras	Guinope	2017	64.9%	95.9%	24.0%
Honduras	Humuya	2000	68.5%	90.1%	31.8%
Honduras	Humuya	2017	41.4%	75.3%	12.7%
Honduras	Ilama	2000	67.6%	92.7%	36.8%
Honduras	Ilama	2017	51.6%	84.7%	22.4%
Honduras	Intibucá	2000	80.3%	88.8%	71.6%
Honduras	Intibucá	2017	57.0%	67.2%	48.8%
Honduras	Iriona	2000	74.9%	87.8%	58.4%
Honduras	Iriona	2017	61.3%	77.6%	43.0%
Honduras	Jacaleapa	2000	62.2%	83.5%	39.0%
Honduras	Jacaleapa	2017	40.4%	61.7%	19.4%
Honduras	Jano	2000	73.6%	95.3%	45.4%
Honduras	Jano	2017	60.6%	90.6%	27.2%
Honduras	Jesús de Otoro	2000	87.6%	92.2%	80.8%
Honduras	Jesús de Otoro	2017	70.3%	78.2%	59.6%
Honduras	Jocón	2000	70.6%	93.1%	42.7%
Honduras	Jocón	2017	51.9%	78.8%	24.6%
Honduras	José Santos Guardiola	2000	31.9%	48.4%	16.8%
Honduras	José Santos Guardiola	2017	16.5%	26.2%	6.4%
Honduras	Juan Francisco Bulnes	2000	69.9%	88.2%	49.4%
Honduras	Juan Francisco Bulnes	2017	50.5%	72.4%	29.7%
Honduras	Jutiapa	2000	74.6%	92.9%	50.5%
Honduras	Jutiapa	2017	58.9%	83.8%	33.3%
Honduras	Juticalpa	2000	67.3%	75.6%	56.7%
Honduras	Juticalpa	2017	41.8%	52.8%	31.3%
Honduras	La Campa	2000	90.7%	98.7%	73.1%
Honduras	La Campa	2017	80.3%	94.7%	58.7%
Honduras	La Ceiba	2000	61.4%	66.9%	55.7%
Honduras	La Ceiba	2017	30.6%	35.1%	26.2%
Honduras	La Encarnación	2000	73.2%	79.7%	66.9%

Country	Second Administrative Unit	Year	Mean	Upper	Lower
Honduras	La Encarnación	2017	47.7%	54.5%	42.2%
Honduras	La Esperanza	2000	47.0%	58.2%	36.2%
Honduras	La Esperanza	2017	31.7%	45.4%	23.8%
Honduras	La Iguala	2000	88.5%	97.8%	69.3%
Honduras	La Iguala	2017	78.6%	93.9%	55.3%
Honduras	La Jigua	2000	61.2%	77.1%	41.9%
Honduras	La Jigua	2017	30.4%	46.1%	16.4%
Honduras	La Labor	2000	86.1%	93.0%	71.4%
Honduras	La Labor	2017	63.2%	74.9%	50.2%
Honduras	La Libertad	2000	92.8%	99.1%	59.2%
Honduras	La Libertad	2000	87.1%	97.3%	72.6%
Honduras	La Libertad	2017	74.2%	91.5%	54.0%
Honduras	La Libertad	2017	82.0%	95.8%	46.9%
Honduras	La Lima	2000	61.0%	70.1%	52.6%
Honduras	La Lima	2017	30.6%	38.1%	24.5%
Honduras	La Masica	2000	79.7%	90.1%	65.0%
Honduras	La Masica	2017	59.5%	76.6%	42.1%
Honduras	La Paz	2000	59.7%	62.3%	57.7%
Honduras	La Paz	2017	41.9%	44.6%	39.6%
Honduras	La Trinidad	2000	91.9%	99.0%	70.5%
Honduras	La Trinidad	2017	81.2%	96.7%	47.4%
Honduras	La Unión	2000	69.9%	89.7%	39.8%
Honduras	La Unión	2000	54.4%	72.4%	31.9%
Honduras	La Unión	2000	91.0%	97.4%	71.9%
Honduras	La Unión	2017	50.0%	78.3%	20.6%
Honduras	La Unión	2017	37.7%	57.9%	16.9%
Honduras	La Unión	2017	77.6%	90.5%	58.8%
Honduras	La Venta	2000	80.9%	98.8%	43.2%
Honduras	La Venta	2017	68.4%	95.0%	30.4%
Honduras	La Virtud	2000	56.2%	67.4%	41.9%
Honduras	La Virtud	2017	28.3%	41.4%	17.3%
Honduras	Lamaní	2000	59.5%	85.6%	27.1%
Honduras	Lamaní	2017	43.5%	74.3%	12.7%
Honduras	Langue	2000	89.8%	92.0%	86.8%
Honduras	Langue	2017	76.0%	80.9%	69.8%
Honduras	Las Flores	2000	86.1%	89.6%	79.4%
Honduras	Las Flores	2017	65.0%	71.9%	52.3%
Honduras	Las Lajas	2000	90.9%	99.6%	56.2%
Honduras	Las Lajas	2017	81.8%	98.5%	37.9%
Honduras	Las Vegas	2000	74.7%	90.9%	38.6%
Honduras	Las Vegas	2017	54.7%	82.6%	19.1%
Honduras	Lauterique	2000	81.6%	95.2%	62.4%
Honduras	Lauterique	2017	63.6%	80.0%	45.1%
Honduras	Lejamaní	2000	34.1%	49.7%	26.9%
Honduras	Lejamaní	2017	24.9%	31.2%	22.6%
Honduras	Lepaera	2000	90.6%	96.7%	79.6%
Honduras	Lepaera	2017	77.6%	88.3%	64.3%
Honduras	Lepaterique	2000	73.0%	93.9%	44.0%
Honduras	Lepaterique	2017	56.5%	85.4%	25.9%
Honduras	Limón	2000	77.5%	92.4%	58.1%
Honduras	Limón	2017	60.6%	80.8%	38.0%
Honduras	Liure	2000	14.3%	37.9%	1.3%
Honduras	Liure	2017	6.4%	20.8%	0.4%
Honduras	Lucerna	2000	78.8%	84.5%	71.4%
Honduras	Lucerna	2017	53.3%	60.8%	45.4%
Honduras	Macuelizo	2000	82.2%	90.9%	69.1%
Honduras	Macuelizo	2017	58.8%	73.7%	42.6%
Honduras	Magdalena	2000	98.7%	99.3%	97.3%
Honduras	Magdalena	2017	94.1%	96.7%	89.0%
Honduras	Mangulile	2000	62.8%	86.9%	31.2%
Honduras	Mangulile	2017	45.6%	74.8%	13.6%

Country	Second Administrative Unit	Year	Mean	Upper	Lower
Honduras	Manto	2000	85.9%	98.0%	67.9%
Honduras	Manto	2017	76.0%	94.8%	50.7%
Honduras	Mapulaca	2000	56.7%	69.2%	42.5%
Honduras	Mapulaca	2017	23.7%	34.6%	14.4%
Honduras	Maraita	2000	77.8%	98.3%	40.9%
Honduras	Maraita	2017	63.5%	93.1%	25.9%
Honduras	Marale	2000	77.6%	95.8%	53.1%
Honduras	Marale	2017	66.7%	90.6%	33.6%
Honduras	Marcala	2000	88.9%	93.9%	82.3%
Honduras	Marcala	2017	76.6%	83.1%	70.2%
Honduras	Marcovia	2000	66.3%	80.7%	51.8%
Honduras	Marcovia	2017	51.6%	69.5%	38.0%
Honduras	Masaguara	2000	89.2%	95.1%	80.5%
Honduras	Masaguara	2017	73.9%	83.9%	64.1%
Honduras	Meámbar	2000	77.0%	95.9%	44.3%
Honduras	Meámbar	2017	62.4%	88.4%	28.9%
Honduras	Mercedes	2000	40.5%	45.2%	34.6%
Honduras	Mercedes	2017	24.3%	29.0%	18.8%
Honduras	Mercedes de Oriente	2000	72.3%	99.4%	18.9%
Honduras	Mercedes de Oriente	2017	57.3%	97.1%	13.3%
Honduras	Minas de Oro	2000	80.8%	96.1%	57.2%
Honduras	Minas de Oro	2017	67.5%	89.2%	41.2%
Honduras	Morazán	2000	64.8%	79.1%	50.6%
Honduras	Morazán	2017	40.3%	53.9%	27.3%
Honduras	Morocelí	2000	75.7%	90.0%	53.2%
Honduras	Morocelí	2017	55.1%	75.4%	30.9%
Honduras	Morolica	2000	70.0%	91.3%	42.8%
Honduras	Morolica	2017	53.8%	80.7%	23.9%
Honduras	Nacaome	2000	73.6%	79.2%	68.0%
Honduras	Nacaome	2017	48.9%	55.1%	43.3%
Honduras	Namasigue	2000	83.6%	93.6%	68.9%
Honduras	Namasigue	2017	67.9%	81.8%	51.9%
Honduras	Naranjito	2000	81.3%	94.0%	59.1%
Honduras	Naranjito	2017	59.4%	79.5%	34.8%
Honduras	Nueva Arcadia	2000	70.9%	77.7%	62.5%
Honduras	Nueva Arcadia	2017	37.1%	45.1%	28.6%
Honduras	Nueva Armenia	2000	70.8%	92.8%	37.1%
Honduras	Nueva Armenia	2017	52.3%	83.0%	17.2%
Honduras	Nueva Frontera	2000	77.9%	95.1%	51.2%
Honduras	Nueva Frontera	2017	55.1%	82.4%	24.4%
Honduras	Nuevo Celilac	2000	63.5%	89.6%	29.7%
Honduras	Nuevo Celilac	2017	43.8%	74.0%	12.7%
Honduras	Ocotepeque	2000	59.9%	72.9%	35.6%
Honduras	Ocotepeque	2017	35.9%	57.7%	18.3%
Honduras	Ojo de Agua	2000	83.1%	93.2%	66.8%
Honduras	Ojo de Agua	2017	60.1%	78.5%	40.3%
Honduras	Ojojona	2000	51.1%	71.2%	25.8%
Honduras	Ojojona	2017	39.2%	63.2%	14.1%
Honduras	Olanchito	2000	64.2%	76.3%	51.5%
Honduras	Olanchito	2017	40.1%	54.9%	28.6%
Honduras	Omoa	2000	76.3%	91.2%	58.8%
Honduras	Omoa	2017	61.5%	80.2%	44.1%
Honduras	Opatoro	2000	90.8%	98.7%	72.2%
Honduras	Opatoro	2017	79.7%	94.7%	53.7%

Country	Second Administrative Unit	Year	Mean	Upper	Lower
Honduras	Orica	2000	82.5%	96.1%	65.1%
Honduras	Orica	2017	66.0%	87.2%	44.1%
Honduras	Orocuina	2000	51.4%	78.0%	31.6%
Honduras	Orocuina	2017	40.9%	66.7%	24.2%
Honduras	Oropolí	2000	71.6%	98.6%	21.3%
Honduras	Oropolí	2017	57.5%	95.2%	10.0%
Honduras	Patuca	2000	67.7%	87.3%	44.0%
Honduras	Patuca	2017	52.2%	75.0%	28.5%
Honduras	Pespire	2000	71.6%	87.4%	53.4%
Honduras	Pespire	2017	49.4%	67.7%	32.3%
Honduras	Petoa	2000	72.6%	93.0%	41.2%
Honduras	Petoa	2017	52.9%	78.0%	21.9%
Honduras	Pimienta	2000	53.2%	61.3%	44.7%
Honduras	Pimienta	2017	23.1%	29.8%	17.1%
Honduras	Piraera	2000	86.2%	98.4%	58.9%
Honduras	Piraera	2017	74.2%	93.4%	46.0%
Honduras	Potrerillos	2000	62.5%	80.5%	42.8%
Honduras	Potrerillos	2000	86.2%	98.9%	60.4%
Honduras	Potrerillos	2017	74.2%	95.4%	43.5%
Honduras	Potrerillos	2017	32.3%	49.1%	23.1%
Honduras	Protección	2000	81.4%	94.3%	53.3%
Honduras	Protección	2017	60.3%	82.1%	31.7%
Honduras	Puerto Cortés	2000	68.3%	78.3%	58.3%
Honduras	Puerto Cortés	2017	40.3%	49.1%	31.5%
Honduras	Puerto Lempira	2000	61.4%	71.5%	49.1%
Honduras	Puerto Lempira	2017	49.3%	60.2%	35.8%
Honduras	Quimistán	2000	64.6%	81.2%	46.8%
Honduras	Quimistán	2017	46.7%	67.3%	27.0%
Honduras	Ramón Villeda Morales	2000	47.7%	71.6%	24.4%
Honduras	Ramón Villeda Morales	2017	33.4%	56.5%	12.4%
Honduras	Reitoca	2000	65.2%	86.0%	46.1%
Honduras	Reitoca	2017	43.8%	67.2%	24.3%
Honduras	Roatán	2000	20.9%	30.0%	14.3%
Honduras	Roatán	2017	8.6%	14.0%	4.5%
Honduras	Sabá	2000	58.7%	72.3%	45.2%
Honduras	Sabá	2017	31.9%	47.5%	19.5%
Honduras	Sabanagrande	2000	83.4%	97.3%	52.2%
Honduras	Sabanagrande	2017	70.0%	92.9%	35.3%
Honduras	Salamá	2000	76.5%	95.1%	49.3%
Honduras	Salamá	2017	60.9%	87.5%	34.5%
Honduras	San Agustín	2000	76.0%	99.7%	28.2%
Honduras	San Agustín	2017	62.9%	98.5%	18.5%
Honduras	San Andrés	2000	79.5%	93.1%	59.7%
Honduras	San Andrés	2017	60.6%	79.6%	40.2%
Honduras	San Antonio	2000	87.6%	91.3%	80.7%
Honduras	San Antonio	2000	88.1%	100.0%	49.7%
Honduras	San Antonio	2017	66.1%	73.3%	57.6%
Honduras	San Antonio	2017	78.2%	99.8%	29.8%
Honduras	San Antonio de Cortés	2000	39.9%	66.6%	21.9%
Honduras	San Antonio de Cortés	2017	24.5%	45.4%	13.4%
Honduras	San Antonio de Flores	2000	79.6%	98.7%	37.6%
Honduras	San Antonio de Flores	2000	78.3%	94.6%	50.3%

Country	Second Administrative Unit	Year	Mean	Upper	Lower
Honduras	San Antonio de Flores	2017	58.6%	86.2%	25.4%
Honduras	San Antonio de Flores	2017	58.8%	94.6%	16.8%
Honduras	San Antonio de Oriente	2000	76.8%	87.9%	60.6%
Honduras	San Antonio de Oriente	2017	61.0%	74.4%	47.3%
Honduras	San Antonio del Norte	2000	73.9%	95.8%	39.8%
Honduras	San Antonio del Norte	2017	52.9%	85.5%	17.5%
Honduras	San Buenaventura	2000	72.4%	97.2%	21.1%
Honduras	San Buenaventura	2017	54.5%	89.7%	8.1%
Honduras	San Esteban	2000	69.7%	87.4%	46.7%
Honduras	San Esteban	2017	54.2%	77.7%	32.2%
Honduras	San Fernando	2000	82.9%	87.9%	77.1%
Honduras	San Fernando	2017	53.6%	61.9%	46.3%
Honduras	San Francisco	2000	63.7%	73.4%	52.0%
Honduras	San Francisco	2000	79.3%	90.6%	58.9%
Honduras	San Francisco	2017	53.2%	72.2%	31.0%
Honduras	San Francisco	2017	46.1%	57.5%	32.3%
Honduras	San Francisco de Becerra	2000	53.4%	73.6%	31.4%
Honduras	San Francisco de Becerra	2017	31.9%	53.6%	12.9%
Honduras	San Francisco de Coray	2000	93.3%	95.7%	87.5%
Honduras	San Francisco de Coray	2017	77.6%	83.4%	68.2%
Honduras	San Francisco de la Paz	2000	62.7%	85.0%	33.6%
Honduras	San Francisco de la Paz	2017	40.6%	68.8%	21.1%
Honduras	San Francisco de Ojuera	2000	66.7%	92.6%	30.4%
Honduras	San Francisco de Ojuera	2017	48.4%	82.6%	14.8%
Honduras	San Francisco de Opalaca	2000	59.4%	79.6%	35.7%
Honduras	San Francisco de Opalaca	2017	39.0%	60.2%	19.7%
Honduras	San Francisco de Yojoa	2000	77.4%	96.8%	38.0%
Honduras	San Francisco de Yojoa	2017	59.8%	92.6%	25.6%
Honduras	San Francisco del Valle	2000	77.5%	79.5%	75.6%
Honduras	San Francisco del Valle	2017	53.2%	56.3%	50.2%
Honduras	San Ignacio	2000	85.4%	95.7%	69.1%
Honduras	San Ignacio	2017	67.7%	86.1%	45.9%
Honduras	San Isidro	2000	59.4%	87.7%	26.9%
Honduras	San Isidro	2000	94.9%	99.6%	74.8%
Honduras	San Isidro	2017	40.1%	79.1%	14.7%
Honduras	San Isidro	2017	87.7%	98.2%	61.2%
Honduras	San Jerónimo	2000	81.6%	98.2%	51.0%
Honduras	San Jerónimo	2000	88.3%	95.3%	67.6%
Honduras	San Jerónimo	2017	69.2%	82.7%	48.9%
Honduras	San Jerónimo	2017	68.1%	94.8%	34.8%

Country	Second Administrative Unit	Year	Mean	Upper	Lower
Honduras	San Jorge	2000	82.5%	94.5%	57.2%
Honduras	San Jorge	2017	60.1%	82.2%	32.2%
Honduras	San José	2000	89.7%	95.1%	76.6%
Honduras	San José	2000	97.3%	98.1%	94.7%
Honduras	San José	2000	86.5%	95.7%	73.4%
Honduras	San José	2017	90.6%	92.3%	87.8%
Honduras	San José	2017	71.2%	85.1%	52.4%
Honduras	San José	2017	68.8%	82.5%	54.5%
Honduras	San José de Colinas	2000	85.6%	98.4%	57.9%
Honduras	San José de Colinas	2017	73.4%	94.4%	41.6%
Honduras	San José de Comayagua	2000	79.1%	98.7%	39.2%
Honduras	San José de Comayagua	2017	63.0%	94.3%	23.6%
Honduras	San José del Potrero	2000	76.6%	98.0%	40.0%
Honduras	San José del Potrero	2017	61.9%	92.3%	25.2%
Honduras	San Juan	2000	87.3%	97.7%	68.5%
Honduras	San Juan	2000	69.9%	99.6%	12.0%
Honduras	San Juan	2017	74.5%	91.4%	55.2%
Honduras	San Juan	2017	54.3%	98.3%	6.8%
Honduras	San Juan de Flores	2000	70.9%	95.1%	39.3%
Honduras	San Juan de Flores	2017	52.8%	86.9%	22.0%
Honduras	San Juan de Opoa	2000	73.2%	89.2%	65.6%
Honduras	San Juan de Opoa	2017	48.6%	69.7%	40.2%
Honduras	San Juan Guarita	2000	68.8%	99.8%	9.5%
Honduras	San Juan Guarita	2017	55.4%	99.2%	2.9%
Honduras	San Lorenzo	2000	79.2%	82.1%	71.4%
Honduras	San Lorenzo	2017	56.6%	61.8%	40.7%
Honduras	San Lucas	2000	80.4%	95.4%	54.1%
Honduras	San Lucas	2017	64.6%	87.9%	36.1%
Honduras	San Luis	2000	84.6%	95.5%	66.9%
Honduras	San Luis	2000	91.3%	99.6%	62.7%
Honduras	San Luis	2017	84.8%	98.2%	52.3%
Honduras	San Luis	2017	71.5%	85.8%	52.0%
Honduras	San Manuel	2000	59.1%	71.6%	47.6%
Honduras	San Manuel	2017	28.2%	39.4%	22.0%
Honduras	San Manuel Colohete	2000	81.6%	97.2%	55.2%
Honduras	San Manuel Colohete	2017	66.7%	88.9%	38.5%
Honduras	San Marcos	2000	63.8%	83.2%	39.0%
Honduras	San Marcos	2000	72.5%	76.0%	68.0%
Honduras	San Marcos	2017	52.2%	57.8%	46.0%
Honduras	San Marcos	2017	43.9%	71.5%	19.3%
Honduras	San Marcos de Caiquín	2000	78.8%	98.6%	42.6%
Honduras	San Marcos de Caiquín	2017	63.2%	93.0%	26.5%
Honduras	San Marcos de Colón	2000	73.7%	91.6%	50.2%
Honduras	San Marcos de Colón	2017	53.2%	78.8%	30.5%

Country	Second Administrative Unit	Year	Mean	Upper	Lower
Honduras	San Marcos de la Sierra	2000	43.9%	65.4%	19.3%
Honduras	San Marcos de la Sierra	2017	24.6%	48.6%	5.3%
Honduras	San Matías	2000	64.2%	89.6%	35.8%
Honduras	San Matías	2017	41.2%	69.8%	16.7%
Honduras	San Miguelito	2000	95.0%	99.5%	77.0%
Honduras	San Miguelito	2000	67.0%	83.3%	45.6%
Honduras	San Miguelito	2017	87.8%	97.6%	65.5%
Honduras	San Miguelito	2017	45.0%	63.5%	24.8%
Honduras	San Nicolás	2000	64.2%	86.5%	37.8%
Honduras	San Nicolás	2000	55.9%	79.1%	34.8%
Honduras	San Nicolás	2017	30.7%	56.3%	13.9%
Honduras	San Nicolás	2017	37.8%	66.0%	18.0%
Honduras	San Pedro	2000	68.0%	70.7%	55.4%
Honduras	San Pedro	2017	59.5%	64.2%	36.2%
Honduras	San Pedro de Tutule	2000	97.9%	98.7%	96.7%
Honduras	San Pedro de Tutule	2017	90.8%	94.2%	86.0%
Honduras	San Pedro Sula	2000	45.3%	49.2%	41.6%
Honduras	San Pedro Sula	2017	17.1%	20.0%	14.9%
Honduras	San Pedro Zacapa	2000	80.4%	97.7%	46.1%
Honduras	San Pedro Zacapa	2017	65.6%	92.0%	27.6%
Honduras	San Rafael	2000	79.7%	99.1%	35.3%
Honduras	San Rafael	2017	64.8%	96.8%	16.1%
Honduras	San Sebastian	2000	86.1%	95.7%	68.5%
Honduras	San Sebastian	2017	71.3%	85.1%	51.8%
Honduras	San Sebastián	2000	58.4%	68.8%	42.7%
Honduras	San Sebastián	2017	41.4%	53.2%	27.9%
Honduras	San Vicente Centenario	2000	21.9%	43.9%	9.4%
Honduras	San Vicente Centenario	2017	9.3%	34.1%	2.7%
Honduras	Santa Ana	2000	82.1%	93.4%	60.5%
Honduras	Santa Ana	2000	56.9%	78.7%	29.3%
Honduras	Santa Ana	2017	60.9%	78.5%	38.5%
Honduras	Santa Ana	2017	33.2%	59.9%	11.2%
Honduras	Santa Ana de Yusguare	2000	94.1%	96.4%	90.3%
Honduras	Santa Ana de Yusguare	2017	78.6%	84.6%	72.3%
Honduras	Santa Bárbara	2000	64.6%	72.2%	55.8%
Honduras	Santa Bárbara	2017	43.7%	53.4%	33.1%
Honduras	Santa Cruz	2000	74.8%	97.5%	35.3%
Honduras	Santa Cruz	2017	59.4%	91.8%	19.6%
Honduras	Santa Cruz de Yojoa	2000	80.9%	90.3%	68.1%
Honduras	Santa Cruz de Yojoa	2017	61.5%	74.2%	48.4%
Honduras	Santa Elena	2000	64.4%	80.0%	48.0%
Honduras	Santa Elena	2017	41.8%	55.6%	30.3%
Honduras	Santa Fé	2000	62.4%	80.5%	35.5%
Honduras	Santa Fé	2000	68.9%	97.3%	27.4%
Honduras	Santa Fé	2017	37.1%	61.3%	12.6%
Honduras	Santa Fé	2017	53.9%	90.6%	14.5%
Honduras	Santa Lucía	2000	98.4%	99.9%	85.3%
Honduras	Santa Lucía	2000	87.7%	92.2%	80.0%

Country	Second Administrative Unit	Year	Mean	Upper	Lower
Honduras	Santa Lucía	2017	94.7%	99.6%	65.9%
Honduras	Santa Lucía	2017	64.4%	76.3%	50.3%
Honduras	Santa María	2000	93.4%	98.2%	78.6%
Honduras	Santa María	2017	81.5%	92.1%	65.0%
Honduras	Santa Maria del Real	2000	82.1%	88.4%	73.0%
Honduras	Santa Maria del Real	2017	61.4%	68.6%	53.9%
Honduras	Santa Rita	2000	86.1%	96.3%	67.6%
Honduras	Santa Rita	2000	72.6%	86.1%	46.2%
Honduras	Santa Rita	2000	39.5%	79.6%	10.5%
Honduras	Santa Rita	2017	71.5%	87.4%	49.1%
Honduras	Santa Rita	2017	43.4%	59.5%	24.4%
Honduras	Santa Rita	2017	22.5%	58.0%	3.2%
Honduras	Santa Rosa de Aguán	2000	62.0%	86.9%	23.6%
Honduras	Santa Rosa de Aguán	2017	44.2%	72.6%	11.3%
Honduras	Santa Rosa de Copán	2000	49.5%	61.7%	37.5%
Honduras	Santa Rosa de Copán	2017	31.5%	45.1%	20.4%
Honduras	Santiago de Puringla	2000	95.5%	99.4%	86.6%
Honduras	Santiago de Puringla	2017	90.1%	97.1%	79.7%
Honduras	Sensenti	2000	74.2%	79.7%	67.4%
Honduras	Sensenti	2017	48.6%	54.8%	42.3%
Honduras	Siguatepeque	2000	77.5%	84.6%	65.8%
Honduras	Siguatepeque	2017	50.2%	61.2%	38.5%
Honduras	Silca	2000	77.4%	96.9%	43.4%
Honduras	Silca	2017	63.7%	87.8%	34.1%
Honduras	Sinuapa	2000	33.1%	41.7%	19.6%
Honduras	Sinuapa	2017	21.2%	31.2%	11.2%
Honduras	Soledad	2000	54.5%	81.2%	25.0%
Honduras	Soledad	2017	36.0%	70.6%	9.1%
Honduras	Sonaguera	2000	73.0%	80.6%	63.5%
Honduras	Sonaguera	2017	45.3%	53.2%	37.2%
Honduras	Sulaco	2000	73.2%	96.9%	40.7%
Honduras	Sulaco	2017	57.8%	89.0%	28.4%
Honduras	Talanga	2000	64.6%	82.2%	42.0%
Honduras	Talanga	2017	39.3%	58.0%	20.7%
Honduras	Talgua	2000	92.4%	94.5%	88.8%
Honduras	Talgua	2017	77.2%	83.2%	68.0%
Honduras	Tambla	2000	84.5%	99.5%	41.9%
Honduras	Tambla	2017	71.6%	97.6%	25.3%
Honduras	Tatumbla	2000	88.5%	99.5%	62.1%
Honduras	Tatumbla	2017	77.2%	97.8%	41.5%
Honduras	Taulabe	2000	70.8%	89.7%	45.0%
Honduras	Taulabe	2017	52.6%	78.7%	29.2%
Honduras	Tela	2000	65.2%	72.6%	57.4%
Honduras	Tela	2017	39.7%	48.0%	30.0%
Honduras	Teupasenti	2000	74.8%	90.6%	54.4%
Honduras	Teupasenti	2017	56.2%	76.4%	35.1%
Honduras	Texiguat	2000	50.0%	75.9%	31.2%
Honduras	Texiguat	2017	35.0%	56.8%	20.3%
Honduras	Tocoa	2000	57.6%	63.3%	51.5%
Honduras	Tocoa	2017	28.7%	34.2%	23.2%
Honduras	Tomalá	2000	87.0%	99.9%	32.1%
Honduras	Tomalá	2017	76.4%	99.6%	19.8%
Honduras	Trinidad	2000	47.6%	70.2%	26.2%
Honduras	Trinidad	2017	29.3%	47.2%	16.7%

Country	Second Administrative Unit	Year	Mean	Upper	Lower
Honduras	Trinidad de Copán	2000	58.9%	77.5%	39.6%
Honduras	Trinidad de Copán	2017	36.5%	54.1%	21.7%
Honduras	Trojes	2000	66.1%	81.4%	47.8%
Honduras	Trojes	2017	45.0%	61.8%	29.2%
Honduras	Trujillo	2000	66.0%	78.0%	49.6%
Honduras	Trujillo	2017	43.7%	56.6%	30.6%
Honduras	Utila	2000	36.3%	65.0%	8.4%
Honduras	Utila	2017	19.5%	37.4%	2.0%
Honduras	Vado Ancho	2000	53.4%	91.9%	12.0%
Honduras	Vado Ancho	2017	33.5%	82.1%	5.6%
Honduras	Valladolid	2000	78.8%	82.7%	67.0%
Honduras	Valladolid	2017	71.5%	80.7%	45.3%
Honduras	Valle de Ángeles	2000	82.5%	93.8%	60.2%
Honduras	Valle de Ángeles	2017	57.9%	78.9%	33.8%
Honduras	Vallecillo	2000	66.6%	95.8%	25.0%
Honduras	Vallecillo	2017	49.5%	88.7%	13.4%
Honduras	Veracruz	2000	91.1%	93.9%	85.9%
Honduras	Veracruz	2017	69.9%	78.4%	58.0%
Honduras	Victoria	2000	71.4%	88.1%	48.5%
Honduras	Victoria	2017	54.4%	77.7%	29.3%
Honduras	Villa de San Antonio	2000	56.2%	70.4%	35.6%
Honduras	Villa de San Antonio	2017	35.7%	52.9%	18.8%
Honduras	Villa de San Francisco	2000	74.9%	93.6%	44.5%
Honduras	Villa de San Francisco	2017	50.3%	79.8%	23.7%
Honduras	Villanueva	2000	44.4%	53.3%	36.3%
Honduras	Villanueva	2017	18.7%	24.7%	13.9%
Honduras	Virginia	2000	38.7%	66.0%	16.0%
Honduras	Virginia	2017	20.5%	39.1%	5.1%
Honduras	Wampusirpi	2000	61.3%	83.7%	37.0%
Honduras	Wampusirpi	2017	45.5%	71.1%	21.7%
Honduras	Yamaranguila	2000	75.0%	85.4%	61.1%
Honduras	Yamaranguila	2017	55.9%	70.2%	40.8%
Honduras	Yarula	2000	73.3%	91.5%	52.4%
Honduras	Yarula	2017	57.4%	82.4%	35.4%
Honduras	Yauyupe	2000	89.3%	99.9%	62.8%
Honduras	Yauyupe	2017	83.3%	99.2%	49.9%
Honduras	Yocón	2000	74.5%	93.4%	46.1%
Honduras	Yocón	2017	59.3%	83.2%	31.2%
Honduras	Yorito	2000	67.7%	93.5%	33.9%
Honduras	Yorito	2017	49.4%	84.0%	17.9%
Honduras	Yoro	2000	76.1%	85.0%	67.2%
Honduras	Yoro	2017	62.3%	73.1%	51.0%
Honduras	Yuscarán	2000	71.3%	93.6%	36.2%
Honduras	Yuscarán	2017	54.8%	86.6%	18.6%
Mexico	Abala	2000	94.0%	99.5%	76.4%
Mexico	Abala	2017	99.3%	100.0%	96.1%
Mexico	Abasolo	2000	83.0%	97.0%	58.6%
Mexico	Abasolo	2000	95.2%	99.9%	70.1%
Mexico	Abasolo	2000	93.4%	98.9%	81.9%
Mexico	Abasolo	2000	79.4%	99.3%	34.3%
Mexico	Abasolo	2017	98.9%	99.9%	94.1%
Mexico	Abasolo	2017	97.6%	99.7%	87.2%
Mexico	Abasolo	2017	99.6%	100.0%	97.8%
Mexico	Abasolo	2017	95.0%	100.0%	64.0%

Country	Second Administrative Unit	Year	Mean	Upper	Lower
Mexico	Abejones	2000	73.9%	99.7%	15.0%
Mexico	Abejones	2017	92.1%	100.0%	41.1%
Mexico	Acacoyagua	2000	42.0%	64.1%	24.6%
Mexico	Acacoyagua	2017	89.5%	95.8%	79.4%
Mexico	Acajete	2000	59.6%	70.1%	40.3%
Mexico	Acajete	2000	94.0%	97.7%	86.9%
Mexico	Acajete	2017	99.6%	99.9%	99.0%
Mexico	Acajete	2017	93.3%	96.2%	88.5%
Mexico	Acala	2000	83.0%	95.2%	60.5%
Mexico	Acala	2017	97.1%	99.7%	88.0%
Mexico	Acambaro	2000	87.0%	94.4%	74.6%
Mexico	Acambaro	2017	97.2%	99.4%	93.0%
Mexico	Acambay	2000	90.4%	98.6%	73.3%
Mexico	Acambay	2017	98.4%	99.9%	92.8%
Mexico	Acanceh	2000	86.0%	99.8%	34.2%
Mexico	Acanceh	2017	97.9%	100.0%	87.5%
Mexico	Acapetahua	2000	49.3%	73.4%	24.2%
Mexico	Acapetahua	2017	87.6%	95.6%	68.2%
Mexico	Acaponeta	2000	85.9%	96.8%	59.0%
Mexico	Acaponeta	2017	97.4%	99.7%	87.1%
Mexico	Acapulco De Juarez	2000	57.3%	67.9%	44.3%
Mexico	Acapulco De Juarez	2017	87.4%	91.3%	79.6%
Mexico	Acateno	2000	33.7%	54.7%	10.4%
Mexico	Acateno	2017	79.6%	94.2%	50.4%
Mexico	Acatepec	2000	69.9%	90.7%	38.1%
Mexico	Acatepec	2017	92.4%	99.1%	76.5%
Mexico	Acatic	2000	82.7%	99.4%	23.2%
Mexico	Acatic	2017	96.4%	100.0%	68.5%
Mexico	Acatlan	2000	80.8%	99.8%	22.1%
Mexico	Acatlan	2000	42.9%	57.9%	23.9%
Mexico	Acatlan	2000	92.4%	99.2%	74.7%
Mexico	Acatlan	2017	96.9%	100.0%	80.6%
Mexico	Acatlan	2017	86.7%	93.6%	68.2%
Mexico	Acatlan	2017	99.0%	99.9%	95.2%
Mexico	Acatlan De Juarez	2000	82.4%	96.6%	60.6%
Mexico	Acatlan De Juarez	2017	97.8%	99.7%	92.4%
Mexico	Acatlan De Perez Figueroa	2000	74.7%	95.4%	39.2%
Mexico	Acatlan De Perez Figueroa	2017	94.3%	99.7%	73.0%
Mexico	Acatzingo	2000	89.7%	93.7%	81.7%
Mexico	Acatzingo	2017	98.7%	99.4%	96.1%
Mexico	Acaxochitlan	2000	78.1%	91.8%	55.9%
Mexico	Acaxochitlan	2017	96.7%	99.1%	90.4%
Mexico	Acayucan	2000	62.1%	86.8%	29.1%
Mexico	Acayucan	2017	92.6%	98.8%	76.8%
Mexico	Acolman	2000	94.6%	99.8%	78.1%
Mexico	Acolman	2017	98.8%	100.0%	90.7%
Mexico	Aconchi	2000	97.7%	99.9%	88.5%
Mexico	Aconchi	2017	99.8%	100.0%	98.7%
Mexico	Acteopan	2000	77.3%	94.1%	45.9%
Mexico	Acteopan	2017	96.8%	99.6%	82.4%
Mexico	Actopan	2000	71.0%	90.6%	44.2%
Mexico	Actopan	2000	79.5%	99.0%	26.1%
Mexico	Actopan	2017	93.7%	98.8%	80.3%
Mexico	Actopan	2017	95.4%	99.9%	67.1%

Country	Second Administrative Unit	Year	Mean	Upper	Lower
Mexico	Acuamanala De Miguel Hidalgo	2000	99.7%	99.9%	99.3%
Mexico	Acuamanala De Miguel Hidalgo	2017	100.0%	100.0%	100.0%
Mexico	Acuitzio	2000	80.4%	99.8%	24.9%
Mexico	Acuitzio	2017	95.9%	100.0%	72.5%
Mexico	Acula	2000	76.0%	99.2%	17.4%
Mexico	Acula	2017	95.4%	99.9%	66.3%
Mexico	Aculco	2000	87.9%	98.7%	65.2%
Mexico	Aculco	2017	97.6%	99.9%	88.8%
Mexico	Acultzingo	2000	89.7%	97.9%	67.0%
Mexico	Acultzingo	2017	99.0%	99.9%	95.2%
Mexico	Acuna	2000	95.3%	98.6%	87.6%
Mexico	Acuna	2017	99.6%	99.9%	98.5%
Mexico	Agua Blanca De Iturbide	2000	95.8%	99.5%	85.4%
Mexico	Agua Blanca De Iturbide	2017	99.5%	100.0%	97.0%
Mexico	Agua Dulce	2000	65.8%	97.6%	6.9%
Mexico	Agua Dulce	2017	88.0%	99.3%	26.6%
Mexico	Agua Prieta	2000	98.2%	99.6%	94.8%
Mexico	Agua Prieta	2017	99.9%	100.0%	99.6%
Mexico	Agualeguas	2000	76.0%	97.8%	34.0%
Mexico	Agualeguas	2017	94.4%	99.9%	72.0%
Mexico	Aguascalientes	2000	89.4%	95.5%	81.7%
Mexico	Aguascalientes	2017	97.1%	99.6%	93.5%
Mexico	Aguililla	2000	76.0%	97.4%	34.9%
Mexico	Aguililla	2017	94.1%	99.7%	62.2%
Mexico	Ahome	2000	90.9%	95.3%	84.0%
Mexico	Ahome	2017	98.9%	99.6%	97.4%
Mexico	Ahuacatlan	2000	91.9%	97.9%	74.4%
Mexico	Ahuacatlan	2000	42.5%	90.0%	6.6%
Mexico	Ahuacatlan	2017	99.0%	99.9%	96.3%
Mexico	Ahuacatlan	2017	79.3%	99.2%	32.6%
Mexico	Ahuacuotzingo	2000	66.5%	89.2%	40.2%
Mexico	Ahuacuotzingo	2017	92.2%	98.6%	78.3%
Mexico	Ahualulco	2000	47.8%	69.6%	23.8%
Mexico	Ahualulco	2017	81.6%	94.8%	60.7%
Mexico	Ahualulco De Mercado	2000	80.8%	99.5%	15.3%
Mexico	Ahualulco De Mercado	2017	95.9%	100.0%	65.9%
Mexico	Ahuatlan	2000	78.2%	99.6%	24.3%
Mexico	Ahuatlan	2017	94.7%	100.0%	63.8%
Mexico	Ahuazotepec	2000	74.9%	99.8%	15.0%
Mexico	Ahuazotepec	2017	93.7%	100.0%	49.7%
Mexico	Ahuehuetitla	2000	71.5%	99.3%	12.6%
Mexico	Ahuehuetitla	2017	93.6%	99.9%	49.3%
Mexico	Ahumada	2000	83.2%	97.3%	42.9%
Mexico	Ahumada	2017	97.6%	99.7%	89.9%
Mexico	Ajacuba	2000	90.0%	99.7%	61.1%
Mexico	Ajacuba	2017	98.5%	100.0%	89.0%
Mexico	Ajalpan	2000	74.3%	92.1%	44.0%
Mexico	Ajalpan	2017	95.8%	99.2%	82.7%
Mexico	Ajuchitlan Del Progreso	2000	72.5%	92.7%	33.4%
Mexico	Ajuchitlan Del Progreso	2017	92.9%	98.7%	74.7%
Mexico	Akil	2000	96.6%	99.2%	89.2%
Mexico	Akil	2017	99.7%	99.9%	98.9%

Country	Second Administrative Unit	Year	Mean	Upper	Lower
Mexico	Alamos	2000	67.5%	77.5%	57.2%
Mexico	Alamos	2017	95.7%	98.2%	91.5%
Mexico	Alaquines	2000	65.2%	94.8%	27.0%
Mexico	Alaquines	2017	89.0%	99.0%	69.9%
Mexico	Albino Zertuche	2000	84.9%	99.9%	25.3%
Mexico	Albino Zertuche	2017	97.2%	100.0%	80.3%
Mexico	Alcozauca De Guerrero	2000	69.1%	86.9%	47.2%
Mexico	Alcozauca De Guerrero	2017	92.7%	98.5%	75.6%
Mexico	Aldama	2000	87.1%	96.3%	67.4%
Mexico	Aldama	2000	72.9%	99.7%	6.9%
Mexico	Aldama	2000	82.9%	93.2%	69.6%
Mexico	Aldama	2017	91.0%	100.0%	39.0%
Mexico	Aldama	2017	98.8%	99.7%	95.9%
Mexico	Aldama	2017	96.8%	99.1%	92.2%
Mexico	Alfajayucan	2000	78.2%	98.4%	30.8%
Mexico	Alfajayucan	2017	94.5%	99.9%	71.7%
Mexico	Aljojuca	2000	78.1%	99.8%	13.4%
Mexico	Aljojuca	2017	95.1%	100.0%	60.2%
Mexico	Allende	2000	83.7%	97.7%	49.1%
Mexico	Allende	2000	92.4%	98.4%	77.8%
Mexico	Allende	2000	93.0%	97.6%	86.0%
Mexico	Allende	2017	97.5%	99.8%	89.6%
Mexico	Allende	2017	99.4%	99.8%	98.1%
Mexico	Allende	2017	99.3%	99.9%	96.5%
Mexico	Almoloya	2000	77.6%	99.2%	26.0%
Mexico	Almoloya	2017	94.0%	99.9%	60.5%
Mexico	Almoloya De Alquisiras	2000	77.1%	89.2%	58.8%
Mexico	Almoloya De Alquisiras	2017	97.1%	99.2%	90.2%
Mexico	Almoloya De Juarez	2000	65.9%	82.9%	48.4%
Mexico	Almoloya De Juarez	2017	91.2%	98.3%	81.5%
Mexico	Almoloya Del Rio	2000	75.1%	91.9%	58.1%
Mexico	Almoloya Del Rio	2017	98.0%	99.6%	95.0%
Mexico	Alpatlahuac	2000	87.3%	99.9%	41.3%
Mexico	Alpatlahuac	2017	97.8%	100.0%	86.6%
Mexico	Alpoyeca	2000	78.2%	99.4%	22.0%
Mexico	Alpoyeca	2017	95.1%	99.9%	65.9%
Mexico	Altamira	2000	94.3%	97.6%	89.3%
Mexico	Altamira	2017	99.0%	99.8%	97.3%
Mexico	Altamirano	2000	83.8%	96.4%	67.9%
Mexico	Altamirano	2017	95.8%	99.7%	84.8%
Mexico	Altar	2000	89.6%	99.3%	56.7%
Mexico	Altar	2017	98.8%	99.9%	93.5%
Mexico	Altepexi	2000	88.3%	94.7%	78.0%
Mexico	Altepexi	2017	98.7%	99.6%	94.9%
Mexico	Alto Lucero De Gutierrez Barrios	2000	75.1%	96.2%	38.0%
Mexico	Alto Lucero De Gutierrez Barrios	2017	93.3%	99.7%	71.4%
Mexico	Altotonga	2000	69.6%	86.6%	47.6%
Mexico	Altotonga	2017	90.4%	98.8%	80.1%

Country	Second Administrative Unit	Year	Mean	Upper	Lower
Mexico	Altzayanca	2000	86.0%	99.0%	51.5%
Mexico	Altzayanca	2017	97.8%	99.9%	85.1%
Mexico	Alvarado	2000	76.4%	94.2%	40.8%
Mexico	Alvarado	2017	93.7%	99.5%	69.0%
Mexico	Alvaro Obregon	2000	93.6%	95.1%	91.7%
Mexico	Alvaro Obregon	2000	68.5%	98.6%	17.6%
Mexico	Alvaro Obregon	2017	90.0%	99.9%	49.6%
Mexico	Alvaro Obregon	2017	99.5%	99.6%	99.3%
Mexico	Amacueca	2000	76.4%	99.6%	15.9%
Mexico	Amacueca	2017	94.3%	100.0%	57.7%
Mexico	Amacuzac	2000	87.5%	95.2%	78.5%
Mexico	Amacuzac	2017	98.4%	99.7%	95.0%
Mexico	Amanalco	2000	61.6%	97.6%	9.3%
Mexico	Amanalco	2017	87.1%	99.8%	43.0%
Mexico	Amatan	2000	70.8%	97.1%	23.6%
Mexico	Amatan	2017	91.7%	99.8%	59.8%
Mexico	Amatenango De La Frontera	2000	70.5%	95.8%	31.0%
Mexico	Amatenango De La Frontera	2017	93.2%	99.7%	72.1%
Mexico	Amatenango Del Valle	2000	75.3%	99.0%	18.3%
Mexico	Amatenango Del Valle	2017	95.0%	99.9%	68.8%
Mexico	Amatepec	2000	76.2%	98.0%	41.9%
Mexico	Amatepec	2017	94.7%	99.8%	80.8%
Mexico	Amatitan	2000	79.9%	99.9%	11.5%
Mexico	Amatitan	2017	95.8%	100.0%	67.5%
Mexico	Amatitlan	2000	72.9%	99.0%	19.3%
Mexico	Amatitlan	2017	92.5%	99.9%	52.5%
Mexico	Amatlan De Canas	2000	74.3%	95.2%	40.7%
Mexico	Amatlan De Canas	2017	94.9%	99.7%	76.7%
Mexico	Amatlan De Los Reyes	2000	60.2%	74.1%	46.1%
Mexico	Amatlan De Los Reyes	2017	93.6%	97.1%	87.9%
Mexico	Amaxac De Guerrero	2000	95.2%	97.4%	92.7%
Mexico	Amaxac De Guerrero	2017	99.7%	99.8%	99.6%
Mexico	Amealco De Bonfil	2000	78.5%	92.6%	59.2%
Mexico	Amealco De Bonfil	2017	96.2%	99.4%	87.3%
Mexico	Ameca	2000	87.0%	98.6%	49.9%
Mexico	Ameca	2017	97.5%	99.9%	83.7%
Mexico	Amecameca	2000	86.4%	99.8%	31.0%
Mexico	Amecameca	2017	97.4%	100.0%	81.6%
Mexico	Amixtlan	2000	64.4%	83.1%	41.6%
Mexico	Amixtlan	2017	95.0%	98.7%	85.3%
Mexico	Amozoc	2000	96.6%	99.4%	87.9%
Mexico	Amozoc	2017	99.8%	100.0%	99.0%
Mexico	Anahuac	2000	96.8%	99.5%	84.1%
Mexico	Anahuac	2017	99.5%	99.9%	98.4%

Country	Second Administrative Unit	Year	Mean	Upper	Lower
Mexico	Angamacutiro	2000	79.1%	97.8%	35.9%
Mexico	Angamacutiro	2017	95.9%	99.9%	72.4%
Mexico	Angangueo	2000	79.9%	99.8%	25.8%
Mexico	Angangueo	2017	96.7%	100.0%	82.5%
Mexico	Angel Albino Corzo	2000	62.0%	93.0%	23.7%
Mexico	Angel Albino Corzo	2017	90.2%	99.2%	62.6%
Mexico	Angel R. Cabada	2000	63.4%	83.4%	39.4%
Mexico	Angel R. Cabada	2017	92.8%	98.3%	80.6%
Mexico	Angostura	2000	86.5%	97.6%	63.3%
Mexico	Angostura	2017	97.8%	99.8%	87.9%
Mexico	Animas Trujano	2000	55.4%	76.1%	34.0%
Mexico	Animas Trujano	2017	94.9%	98.2%	89.4%
Mexico	Antiguo Morelos	2000	87.0%	98.6%	64.2%
Mexico	Antiguo Morelos	2017	97.9%	99.9%	90.5%
Mexico	Apan	2000	80.5%	98.5%	26.5%
Mexico	Apan	2017	95.0%	99.9%	62.8%
Mexico	Apaseo El Alto	2000	82.1%	95.5%	56.8%
Mexico	Apaseo El Alto	2017	97.1%	99.6%	87.5%
Mexico	Apaseo El Grande	2000	82.5%	97.1%	50.9%
Mexico	Apaseo El Grande	2017	96.7%	99.8%	87.3%
Mexico	Apatzingan	2000	65.4%	76.1%	50.1%
Mexico	Apatzingan	2017	96.1%	97.4%	92.4%
Mexico	Apaxco	2000	94.7%	99.8%	68.4%
Mexico	Apaxco	2017	99.4%	100.0%	94.8%
Mexico	Apaxtla	2000	77.9%	97.8%	32.6%
Mexico	Apaxtla	2017	95.2%	99.9%	75.5%
Mexico	Apazapan	2000	73.1%	98.9%	20.4%
Mexico	Apazapan	2017	92.5%	99.9%	57.8%
Mexico	Apetatitlan De Antonio Carvajal	2000	98.1%	98.8%	96.9%
Mexico	Apetatitlan De Antonio Carvajal	2017	99.9%	99.9%	99.8%
Mexico	Apizaco	2000	98.3%	99.3%	96.8%
Mexico	Apizaco	2017	99.9%	100.0%	99.8%
Mexico	Apodaca	2000	95.9%	99.0%	89.4%
Mexico	Apodaca	2017	99.5%	99.9%	98.1%
Mexico	Aporo	2000	75.5%	99.8%	8.1%
Mexico	Aporo	2017	92.9%	100.0%	44.1%
Mexico	Apozol	2000	97.1%	99.7%	87.9%
Mexico	Apozol	2017	99.7%	100.0%	98.2%
Mexico	Apulco	2000	73.5%	97.7%	26.1%
Mexico	Apulco	2017	94.5%	99.8%	73.0%
Mexico	Aquila	2000	79.0%	92.5%	58.0%
Mexico	Aquila	2000	82.4%	99.9%	23.7%
Mexico	Aquila	2017	95.6%	100.0%	67.6%
Mexico	Aquila	2017	95.7%	99.2%	87.4%
Mexico	Aquiles Serdan	2000	86.6%	97.8%	60.9%

Country	Second Administrative Unit	Year	Mean	Upper	Lower
Mexico	Aquiles Serdan	2017	97.7%	99.9%	87.4%
Mexico	Aquismon	2000	55.9%	81.5%	29.9%
Mexico	Aquismon	2017	87.2%	96.7%	62.6%
Mexico	Aquixtla	2000	70.5%	98.9%	14.2%
Mexico	Aquixtla	2017	90.8%	99.9%	48.4%
Mexico	Aramberri	2000	85.0%	93.8%	72.6%
Mexico	Aramberri	2017	97.4%	99.4%	93.1%
Mexico	Arandas	2000	76.6%	98.7%	25.1%
Mexico	Arandas	2017	94.4%	99.9%	57.6%
Mexico	Arcelia	2000	64.8%	94.8%	25.3%
Mexico	Arcelia	2017	91.7%	99.5%	58.9%
Mexico	Ario	2000	80.0%	96.2%	41.9%
Mexico	Ario	2017	96.5%	99.7%	83.0%
Mexico	Arivechi	2000	78.6%	99.0%	28.3%
Mexico	Arivechi	2017	95.1%	99.9%	69.4%
Mexico	Arizpe	2000	84.9%	98.3%	47.9%
Mexico	Arizpe	2017	97.1%	99.9%	79.1%
Mexico	Armadillo De Los Infante	2000	77.1%	96.9%	50.5%
Mexico	Armadillo De Los Infante	2017	94.5%	99.8%	77.0%
Mexico	Armeria	2000	95.9%	99.7%	85.5%
Mexico	Armeria	2017	99.4%	100.0%	95.4%
Mexico	Arriaga	2000	72.7%	94.3%	33.5%
Mexico	Arriaga	2017	95.5%	99.5%	80.6%
Mexico	Arroyo Seco	2000	78.1%	93.5%	54.4%
Mexico	Arroyo Seco	2017	95.2%	99.5%	80.3%
Mexico	Arteaga	2000	90.8%	97.6%	78.0%
Mexico	Arteaga	2000	78.7%	94.7%	41.5%
Mexico	Arteaga	2017	98.4%	99.8%	93.6%
Mexico	Arteaga	2017	95.3%	99.4%	80.6%
Mexico	Ascension	2000	92.9%	97.8%	83.4%
Mexico	Ascension	2017	99.2%	99.8%	97.8%
Mexico	Asientos	2000	87.0%	97.3%	65.1%
Mexico	Asientos	2017	97.8%	99.8%	89.1%
Mexico	Astacinga	2000	73.2%	99.5%	10.5%
Mexico	Astacinga	2017	94.1%	100.0%	62.4%
Mexico	Asuncion Cacalotepec	2000	65.9%	99.3%	7.6%
Mexico	Asuncion Cacalotepec	2017	89.0%	100.0%	40.5%
Mexico	Asuncion Cuyotepeji	2000	91.3%	99.9%	55.6%
Mexico	Asuncion Cuyotepeji	2017	99.0%	100.0%	94.1%
Mexico	Asuncion Ixtaltepec	2000	64.4%	81.5%	43.0%
Mexico	Asuncion Ixtaltepec	2017	90.0%	98.5%	69.1%
Mexico	Asuncion Nochixtlan	2000	68.1%	88.9%	38.4%
Mexico	Asuncion Nochixtlan	2017	94.5%	98.8%	80.0%
Mexico	Asuncion Ocotlan	2000	8.7%	39.0%	0.5%
Mexico	Asuncion Ocotlan	2017	48.7%	91.2%	8.2%
Mexico	Asuncion Tlacolulita	2000	77.8%	99.3%	27.9%
Mexico	Asuncion Tlacolulita	2017	95.6%	100.0%	75.5%

Country	Second Administrative Unit	Year	Mean	Upper	Lower
Mexico	Atarjea	2000	61.1%	82.5%	31.5%
Mexico	Atarjea	2017	90.9%	98.7%	75.3%
Mexico	Atemajac De Brizuela	2000	84.0%	99.8%	16.3%
Mexico	Atemajac De Brizuela	2017	96.6%	100.0%	69.0%
Mexico	Atempan	2000	75.7%	84.7%	62.9%
Mexico	Atempan	2017	97.6%	99.0%	94.2%
Mexico	Atenango Del Rio	2000	69.8%	91.6%	32.6%
Mexico	Atenango Del Rio	2017	94.7%	99.3%	78.8%
Mexico	Atenco	2000	96.1%	97.8%	93.0%
Mexico	Atenco	2017	99.7%	99.9%	99.4%
Mexico	Atengo	2000	82.9%	99.5%	30.9%
Mexico	Atengo	2017	96.8%	100.0%	75.4%
Mexico	Atenguillo	2000	80.7%	99.2%	27.6%
Mexico	Atenguillo	2017	95.7%	99.9%	67.8%
Mexico	Atexcal	2000	78.4%	99.5%	26.6%
Mexico	Atexcal	2017	93.9%	100.0%	49.9%
Mexico	Atil	2000	80.4%	99.9%	18.1%
Mexico	Atil	2017	96.2%	100.0%	69.8%
Mexico	Atitalaquia	2000	98.6%	99.7%	95.7%
Mexico	Atitalaquia	2017	99.9%	100.0%	99.7%
Mexico	Atizapan	2000	58.9%	89.1%	31.6%
Mexico	Atizapan	2017	96.4%	99.6%	89.6%
Mexico	Atizapan De Zaragoza	2000	86.4%	93.0%	74.5%
Mexico	Atizapan De Zaragoza	2017	98.9%	99.6%	96.8%
Mexico	Atlacomulco	2000	85.3%	96.6%	65.5%
Mexico	Atlacomulco	2017	97.9%	99.8%	92.1%
Mexico	Atlahuilco	2000	72.8%	92.5%	40.3%
Mexico	Atlahuilco	2017	95.3%	99.5%	77.4%
Mexico	Atlamajalcingo Del Monte	2000	65.5%	98.5%	10.1%
Mexico	Atlamajalcingo Del Monte	2017	89.9%	99.8%	47.3%
Mexico	Atlangatepec	2000	92.5%	99.8%	60.6%
Mexico	Atlangatepec	2017	98.5%	100.0%	86.9%
Mexico	Atlapexco	2000	75.8%	90.2%	42.5%
Mexico	Atlapexco	2017	94.4%	97.7%	83.2%
Mexico	Atlatlahucan	2000	92.7%	99.9%	62.0%
Mexico	Atlatlahucan	2017	99.1%	100.0%	94.8%
Mexico	Atlautla	2000	82.7%	99.8%	27.7%
Mexico	Atlautla	2017	95.8%	100.0%	71.1%
Mexico	Atlequizayan	2000	64.3%	72.2%	54.5%
Mexico	Atlequizayan	2017	96.4%	97.6%	94.1%
Mexico	Atlixco	2000	76.9%	87.5%	58.6%
Mexico	Atlixco	2017	97.5%	98.8%	93.4%
Mexico	Atlixtac	2000	60.5%	78.9%	34.9%
Mexico	Atlixtac	2017	87.5%	94.5%	74.5%
Mexico	Atolinga	2000	79.8%	99.3%	27.4%
Mexico	Atolinga	2017	95.8%	100.0%	71.0%
Mexico	Atotonilco De Tula	2000	87.3%	92.0%	79.7%
Mexico	Atotonilco De Tula	2017	98.5%	99.6%	94.3%
Mexico	Atotonilco El Alto	2000	80.3%	99.2%	29.2%
Mexico	Atotonilco El Alto	2017	96.5%	100.0%	79.0%

Country	Second Administrative Unit	Year	Mean	Upper	Lower
Mexico	Atotonilco El Grande	2000	73.5%	98.8%	27.4%
Mexico	Atotonilco El Grande	2017	91.7%	99.9%	53.4%
Mexico	Atoyac	2000	90.3%	98.2%	75.7%
Mexico	Atoyac	2000	78.0%	98.0%	36.6%
Mexico	Atoyac	2017	98.2%	99.9%	92.0%
Mexico	Atoyac	2017	96.0%	99.9%	82.0%
Mexico	Atoyac De Alvarez	2000	74.4%	93.2%	43.1%
Mexico	Atoyac De Alvarez	2017	95.4%	99.4%	86.9%
Mexico	Atoyatempan	2000	69.4%	99.4%	11.6%
Mexico	Atoyatempan	2017	91.8%	100.0%	53.5%
Mexico	Atzacan	2000	45.8%	57.6%	32.3%
Mexico	Atzacan	2017	80.6%	86.7%	75.0%
Mexico	Atzala	2000	68.8%	74.1%	62.3%
Mexico	Atzala	2017	97.5%	98.1%	96.7%
Mexico	Atzalan	2000	69.4%	92.9%	37.6%
Mexico	Atzalan	2017	91.4%	99.4%	69.8%
Mexico	Atzitzihuacan	2000	76.6%	97.7%	30.8%
Mexico	Atzitzihuacan	2017	95.5%	99.8%	74.9%
Mexico	Atzitzintla	2000	69.3%	98.4%	13.8%
Mexico	Atzitzintla	2017	91.9%	99.9%	51.8%
Mexico	Autlan De Navarro	2000	91.8%	96.1%	83.0%
Mexico	Autlan De Navarro	2017	99.3%	99.8%	98.0%
Mexico	Axapusco	2000	87.8%	98.4%	68.0%
Mexico	Axapusco	2017	98.0%	99.9%	90.4%
Mexico	Axochiapan	2000	75.1%	99.4%	17.9%
Mexico	Axochiapan	2017	93.6%	100.0%	55.9%
Mexico	Axtla De Terrazas	2000	11.3%	20.4%	4.2%
Mexico	Axtla De Terrazas	2017	38.3%	58.2%	22.1%
Mexico	Axutla	2000	80.4%	99.7%	21.6%
Mexico	Axutla	2017	96.5%	100.0%	72.9%
Mexico	Ayahualulco	2000	36.7%	75.8%	11.4%
Mexico	Ayahualulco	2017	65.0%	97.7%	21.2%
Mexico	Ayala	2000	91.3%	98.9%	76.4%
Mexico	Ayala	2017	98.4%	99.9%	92.0%
Mexico	Ayapango	2000	89.5%	99.9%	49.0%
Mexico	Ayapango	2017	97.9%	100.0%	82.8%
Mexico	Ayoquezco De Aldama	2000	80.8%	99.2%	28.9%
Mexico	Ayoquezco De Aldama	2017	97.2%	99.9%	85.6%
Mexico	Ayotlan	2000	83.5%	95.7%	62.5%
Mexico	Ayotlan	2017	97.6%	99.7%	89.3%
Mexico	Ayotoxco De Guerrero	2000	71.6%	98.5%	17.7%
Mexico	Ayotoxco De Guerrero	2017	91.3%	99.9%	50.0%
Mexico	Ayotzintepec	2000	57.3%	95.4%	8.6%
Mexico	Ayotzintepec	2017	84.9%	99.7%	28.3%
Mexico	Ayutla	2000	81.7%	98.6%	33.8%
Mexico	Ayutla	2017	96.5%	99.9%	79.6%
Mexico	Ayutla De Los Libres	2000	68.0%	90.6%	35.0%
Mexico	Ayutla De Los Libres	2017	92.1%	98.9%	66.3%

Country	Second Administrative Unit	Year	Mean	Upper	Lower
Mexico	Azcapotzalco	2000	99.4%	99.6%	99.2%
Mexico	Azcapotzalco	2017	100.0%	100.0%	99.9%
Mexico	Azoyu	2000	73.8%	96.0%	24.7%
Mexico	Azoyu	2017	93.7%	99.8%	61.6%
Mexico	Baca	2000	85.3%	99.8%	35.9%
Mexico	Baca	2017	97.9%	100.0%	87.6%
Mexico	Bacadehuachi	2000	82.8%	99.7%	20.8%
Mexico	Bacadehuachi	2017	97.3%	100.0%	80.2%
Mexico	Bacanora	2000	80.8%	99.1%	31.5%
Mexico	Bacanora	2017	96.2%	99.9%	81.7%
Mexico	Bacerac	2000	82.0%	98.7%	26.3%
Mexico	Bacerac	2017	96.3%	99.9%	78.1%
Mexico	Bachiniva	2000	83.2%	97.4%	57.2%
Mexico	Bachiniva	2017	97.1%	99.8%	87.5%
Mexico	Bacoachi	2000	83.7%	99.7%	26.9%
Mexico	Bacoachi	2017	94.5%	100.0%	37.0%
Mexico	Bacum	2000	95.6%	99.4%	84.5%
Mexico	Bacum	2017	99.4%	99.9%	96.5%
Mexico	Badiraguato	2000	54.2%	64.1%	40.2%
Mexico	Badiraguato	2017	78.9%	86.7%	68.6%
Mexico	Bahia De Banderas	2000	72.2%	89.4%	54.0%
Mexico	Bahia De Banderas	2017	94.1%	99.2%	82.5%
Mexico	Balancan	2000	69.2%	81.9%	55.9%
Mexico	Balancan	2017	91.1%	97.6%	81.5%
Mexico	Balleza	2000	81.8%	91.4%	66.7%
Mexico	Balleza	2017	96.7%	99.1%	91.5%
Mexico	Banamichi	2000	96.3%	99.9%	80.5%
Mexico	Banamichi	2017	99.7%	100.0%	97.9%
Mexico	Banderilla	2000	91.1%	94.5%	86.5%
Mexico	Banderilla	2017	99.4%	99.7%	99.1%
Mexico	Batopilas	2000	76.3%	91.5%	50.6%
Mexico	Batopilas	2017	94.9%	99.0%	86.8%
Mexico	Baviacora	2000	95.0%	99.8%	82.2%
Mexico	Baviacora	2017	99.4%	100.0%	96.5%
Mexico	Bavispe	2000	82.7%	99.3%	24.9%
Mexico	Bavispe	2017	96.3%	99.9%	74.0%
Mexico	Bejucal De Ocampo	2000	69.0%	99.0%	21.6%
Mexico	Bejucal De Ocampo	2017	91.7%	99.9%	58.6%
Mexico	Bella Vista	2000	69.1%	95.0%	29.6%
Mexico	Bella Vista	2017	93.4%	99.6%	64.2%
Mexico	Benemerito De Las Americas	2000	70.3%	91.9%	33.6%
Mexico	Benemerito De Las Americas	2017	92.6%	99.2%	72.4%
Mexico	Benito Juarez	2000	99.0%	99.4%	98.6%
Mexico	Benito Juarez	2000	94.3%	99.7%	77.3%
Mexico	Benito Juarez	2000	90.3%	99.0%	70.6%
Mexico	Benito Juarez	2000	69.9%	83.4%	54.9%
Mexico	Benito Juarez	2000	92.3%	99.7%	62.1%
Mexico	Benito Juarez	2000	72.5%	97.4%	24.2%
Mexico	Benito Juarez	2000	79.0%	88.2%	67.4%
Mexico	Benito Juarez	2017	95.6%	98.7%	90.4%
Mexico	Benito Juarez	2017	99.9%	100.0%	99.9%
Mexico	Benito Juarez	2017	98.8%	100.0%	92.6%
Mexico	Benito Juarez	2017	92.8%	99.8%	68.7%
Mexico	Benito Juarez	2017	98.8%	100.0%	90.1%

Country	Second Administrative Unit	Year	Mean	Upper	Lower
Mexico	Benito Juarez	2017	99.6%	100.0%	97.5%
Mexico	Benito Juarez	2017	93.1%	98.9%	86.0%
Mexico	Benjamin Hill	2000	93.4%	99.6%	72.6%
Mexico	Benjamin Hill	2017	98.3%	100.0%	87.9%
Mexico	Berriozabal	2000	68.3%	86.2%	46.6%
Mexico	Berriozabal	2017	92.8%	98.4%	82.2%
Mexico	Boca Del Rio	2000	88.4%	91.2%	84.4%
Mexico	Boca Del Rio	2017	95.2%	96.6%	93.8%
Mexico	Bochil	2000	82.9%	92.1%	69.9%
Mexico	Bochil	2017	97.1%	99.2%	92.9%
Mexico	Bocoyna	2000	45.1%	53.8%	37.6%
Mexico	Bocoyna	2017	85.4%	90.5%	80.2%
Mexico	Bokoba	2000	92.4%	99.7%	63.5%
Mexico	Bokoba	2017	99.4%	100.0%	96.9%
Mexico	Bolanos	2000	73.7%	95.6%	39.5%
Mexico	Bolanos	2017	93.1%	99.6%	71.6%
Mexico	Brisenas	2000	97.9%	99.8%	90.8%
Mexico	Brisenas	2017	99.8%	100.0%	98.6%
Mexico	Buctzotz	2000	75.4%	99.3%	21.8%
Mexico	Buctzotz	2017	94.3%	100.0%	64.9%
Mexico	Buenaventura	2000	93.6%	98.0%	83.6%
Mexico	Buenaventura	2017	99.1%	99.9%	96.3%
Mexico	Buenavista	2000	79.4%	97.0%	47.9%
Mexico	Buenavista	2017	96.2%	99.8%	80.6%
Mexico	Buenavista De Cuellar	2000	65.0%	95.3%	11.3%
Mexico	Buenavista De Cuellar	2017	92.5%	99.0%	65.5%
Mexico	Burgos	2000	75.0%	94.2%	44.3%
Mexico	Burgos	2017	93.3%	99.4%	66.0%
Mexico	Bustamante	2000	96.4%	99.8%	81.6%
Mexico	Bustamante	2000	87.2%	96.7%	72.1%
Mexico	Bustamante	2017	99.7%	100.0%	98.4%
Mexico	Bustamante	2017	97.8%	99.7%	91.8%
Mexico	Cabo Corrientes	2000	77.6%	96.6%	42.0%
Mexico	Cabo Corrientes	2017	94.9%	99.7%	76.1%
Mexico	Caborca	2000	92.3%	96.3%	85.4%
Mexico	Caborca	2017	98.9%	99.7%	97.3%
Mexico	Cacahoatan	2000	71.6%	86.8%	50.0%
Mexico	Cacahoatan	2017	95.9%	98.9%	85.8%
Mexico	Cacalchen	2000	87.6%	99.9%	24.6%
Mexico	Cacalchen	2017	97.4%	100.0%	77.8%
Mexico	Cadereyta De Montes	2000	87.4%	97.4%	65.3%
Mexico	Cadereyta De Montes	2017	97.8%	99.8%	92.8%
Mexico	Cadereyta Jimenez	2000	83.1%	91.9%	68.2%
Mexico	Cadereyta Jimenez	2017	96.3%	98.7%	92.4%
Mexico	Cajeme	2000	98.5%	99.3%	96.7%
Mexico	Cajeme	2017	99.8%	99.9%	99.4%
Mexico	Calakmul	2000	71.2%	80.4%	57.7%
Mexico	Calakmul	2017	94.7%	97.3%	89.4%
Mexico	Calcahualco	2000	78.9%	98.0%	41.6%
Mexico	Calcahualco	2017	96.4%	99.9%	83.1%
Mexico	Calera	2000	92.1%	96.5%	82.9%
Mexico	Calera	2017	99.3%	99.7%	97.6%
Mexico	Calihuala	2000	77.2%	99.8%	12.7%
Mexico	Calihuala	2017	93.9%	100.0%	51.7%

Country	Second Administrative Unit	Year	Mean	Upper	Lower
Mexico	Calimaya	2000	88.9%	99.5%	54.0%
Mexico	Calimaya	2017	98.4%	100.0%	88.9%
Mexico	Calkini	2000	82.9%	91.3%	71.2%
Mexico	Calkini	2017	94.7%	98.5%	87.7%
Mexico	Calnali	2000	68.8%	97.3%	17.1%
Mexico	Calnali	2017	90.2%	99.8%	55.7%
Mexico	Calotmul	2000	81.3%	99.2%	25.0%
Mexico	Calotmul	2017	95.9%	100.0%	75.4%
Mexico	Calpan	2000	93.1%	96.4%	85.7%
Mexico	Calpan	2017	99.4%	99.7%	98.8%
Mexico	Calpulalpan	2000	94.5%	98.4%	87.9%
Mexico	Calpulalpan	2017	99.2%	99.9%	96.8%
Mexico	Caltepec	2000	76.6%	98.1%	30.6%
Mexico	Caltepec	2017	93.7%	99.9%	67.8%
Mexico	Calvillo	2000	90.0%	98.5%	68.1%
Mexico	Calvillo	2017	98.3%	99.9%	90.7%
Mexico	Camargo	2000	81.9%	99.0%	31.4%
Mexico	Camargo	2000	91.0%	97.5%	76.1%
Mexico	Camargo	2017	96.2%	99.9%	76.7%
Mexico	Camargo	2017	99.0%	99.8%	96.6%
Mexico	Camaron De Tejeda	2000	70.1%	98.5%	21.2%
Mexico	Camaron De Tejeda	2017	92.9%	99.9%	63.8%
Mexico	Camerino Z. Mendoza	2000	38.4%	50.6%	28.5%
Mexico	Camerino Z. Mendoza	2017	88.0%	93.7%	80.1%
Mexico	Camocuautla	2000	92.4%	98.1%	80.8%
Mexico	Camocuautla	2017	99.6%	99.9%	98.8%
Mexico	Campeche	2000	60.4%	71.2%	48.7%
Mexico	Campeche	2017	83.5%	90.0%	76.1%
Mexico	Canada Morelos	2000	80.0%	98.5%	28.9%
Mexico	Canada Morelos	2017	96.1%	99.9%	73.0%
Mexico	Canadas De Obregon	2000	81.8%	99.6%	28.2%
Mexico	Canadas De Obregon	2017	96.5%	100.0%	75.7%
Mexico	Cananea	2000	97.1%	99.5%	89.0%
Mexico	Cananea	2017	99.7%	100.0%	98.8%
Mexico	Canatlan	2000	86.0%	95.8%	67.8%
Mexico	Canatlan	2017	97.5%	99.5%	92.7%
Mexico	Candela	2000	84.2%	99.2%	48.7%
Mexico	Candela	2017	97.0%	99.9%	84.0%
Mexico	Candelaria	2000	59.4%	69.3%	49.2%
Mexico	Candelaria	2017	87.8%	94.2%	80.9%
Mexico	Candelaria Loxicha	2000	65.4%	87.9%	20.3%
Mexico	Candelaria Loxicha	2017	93.5%	98.0%	73.1%
Mexico	Canelas	2000	82.7%	95.5%	61.9%
Mexico	Canelas	2017	97.1%	99.5%	91.0%
Mexico	Canitas De Felipe Pescador	2000	91.5%	98.6%	70.0%
Mexico	Canitas De Felipe Pescador	2017	99.1%	99.9%	96.0%
Mexico	Cansahcab	2000	95.8%	99.9%	82.7%
Mexico	Cansahcab	2017	99.2%	100.0%	93.1%
Mexico	Cantamayec	2000	76.9%	99.6%	23.0%
Mexico	Cantamayec	2017	94.7%	100.0%	62.9%

Country	Second Administrative Unit	Year	Mean	Upper	Lower
Mexico	Capulalpam De Mendez	2000	72.7%	96.8%	16.1%
Mexico	Capulalpam De Mendez	2017	95.7%	99.8%	71.4%
Mexico	Capulhuac	2000	47.1%	86.6%	15.8%
Mexico	Capulhuac	2017	89.0%	99.2%	65.4%
Mexico	Caracuaro	2000	79.8%	96.6%	45.2%
Mexico	Caracuaro	2017	95.6%	99.7%	78.3%
Mexico	Carbo	2000	91.6%	99.6%	55.5%
Mexico	Carbo	2017	98.9%	100.0%	94.3%
Mexico	Cardenas	2000	73.5%	80.6%	64.9%
Mexico	Cardenas	2000	60.6%	77.8%	39.2%
Mexico	Cardenas	2017	87.9%	96.4%	76.2%
Mexico	Cardenas	2017	91.3%	97.0%	87.3%
Mexico	Cardonal	2000	76.4%	99.0%	33.3%
Mexico	Cardonal	2017	94.0%	99.9%	66.6%
Mexico	Carichi	2000	74.9%	87.4%	56.8%
Mexico	Carichi	2017	95.4%	98.5%	89.1%
Mexico	Carlos A. Carrillo	2000	67.4%	93.9%	25.8%
Mexico	Carlos A. Carrillo	2017	93.4%	99.3%	74.8%
Mexico	Carmen	2000	96.7%	99.4%	90.1%
Mexico	Carmen	2000	75.5%	83.4%	64.2%
Mexico	Carmen	2017	99.8%	100.0%	99.4%
Mexico	Carmen	2017	96.2%	98.1%	93.3%
Mexico	Carrillo Puerto	2000	73.8%	99.3%	19.4%
Mexico	Carrillo Puerto	2017	93.0%	100.0%	58.3%
Mexico	Casas	2000	77.7%	93.4%	53.7%
Mexico	Casas	2017	95.0%	99.4%	84.8%
Mexico	Casas Grandes	2000	86.6%	95.4%	70.4%
Mexico	Casas Grandes	2017	98.1%	99.6%	93.4%
Mexico	Casimiro Castillo	2000	76.6%	99.1%	13.8%
Mexico	Casimiro Castillo	2017	94.2%	99.9%	56.5%
Mexico	Castanos	2000	80.6%	99.0%	22.4%
Mexico	Castanos	2017	96.3%	99.9%	78.2%
Mexico	Castillo De Teayo	2000	90.5%	99.9%	62.0%
Mexico	Castillo De Teayo	2017	98.2%	100.0%	89.4%
Mexico	Catazaja	2000	68.3%	93.5%	29.7%
Mexico	Catazaja	2017	90.9%	99.4%	65.5%
Mexico	Catemaco	2000	46.2%	60.1%	28.6%
Mexico	Catemaco	2017	90.7%	96.0%	81.5%
Mexico	Catorce	2000	78.3%	96.6%	46.2%
Mexico	Catorce	2017	94.9%	99.7%	78.2%
Mexico	Caxhuacan	2000	50.4%	54.5%	46.3%
Mexico	Caxhuacan	2017	92.5%	94.3%	90.2%
Mexico	Cazones	2000	74.4%	99.1%	28.6%
Mexico	Cazones	2017	93.5%	99.9%	72.0%
Mexico	Cedral	2000	94.4%	99.0%	86.2%
Mexico	Cedral	2017	98.9%	99.9%	96.3%
Mexico	Celaya	2000	88.5%	94.5%	77.6%
Mexico	Celaya	2017	98.2%	99.5%	95.1%
Mexico	Celestun	2000	76.3%	99.9%	3.6%
Mexico	Celestun	2017	92.7%	100.0%	35.7%

Country	Second Administrative Unit	Year	Mean	Upper	Lower
Mexico	Cenotillo	2000	74.9%	99.3%	12.0%
Mexico	Cenotillo	2017	93.0%	100.0%	39.7%
Mexico	Centla	2000	61.5%	82.0%	33.3%
Mexico	Centla	2017	90.6%	97.5%	75.7%
Mexico	Centro	2000	78.7%	86.5%	67.4%
Mexico	Centro	2017	96.6%	98.7%	92.5%
Mexico	Cerralvo	2000	94.7%	98.6%	85.6%
Mexico	Cerralvo	2017	99.4%	99.9%	97.7%
Mexico	Cerritos	2000	78.7%	94.6%	44.5%
Mexico	Cerritos	2017	97.1%	99.7%	89.0%
Mexico	Cerro Azul	2000	97.3%	99.9%	86.7%
Mexico	Cerro Azul	2017	99.8%	100.0%	98.8%
Mexico	Cerro De San Pedro	2000	27.4%	45.1%	18.0%
Mexico	Cerro De San Pedro	2017	71.5%	91.0%	47.4%
Mexico	Chacaltianguis	2000	51.4%	75.2%	23.8%
Mexico	Chacaltianguis	2017	78.1%	92.3%	56.8%
Mexico	Chacsinkin	2000	84.8%	99.6%	41.3%
Mexico	Chacsinkin	2017	98.9%	100.0%	91.9%
Mexico	Chahuites	2000	79.1%	99.7%	8.5%
Mexico	Chahuites	2017	95.7%	99.9%	64.2%
Mexico	Chalcatongo De Hidalgo	2000	63.8%	85.0%	37.4%
Mexico	Chalcatongo De Hidalgo	2017	95.7%	99.2%	86.7%
Mexico	Chalchicomula De Sesma	2000	74.1%	97.2%	28.3%
Mexico	Chalchicomula De Sesma	2017	94.6%	99.8%	67.1%
Mexico	Chalchihuitan	2000	52.3%	75.0%	27.0%
Mexico	Chalchihuitan	2017	88.5%	97.2%	68.4%
Mexico	Chalchihuites	2000	79.9%	98.3%	32.1%
Mexico	Chalchihuites	2017	95.4%	99.8%	72.9%
Mexico	Chalco	2000	96.2%	98.5%	91.3%
Mexico	Chalco	2017	99.6%	99.9%	97.5%
Mexico	Chalma	2000	51.7%	86.2%	12.1%
Mexico	Chalma	2017	85.0%	97.9%	50.4%
Mexico	Champoton	2000	67.7%	87.9%	41.8%
Mexico	Champoton	2017	90.9%	98.6%	67.9%
Mexico	Chamula	2000	76.9%	86.1%	60.6%
Mexico	Chamula	2017	97.2%	98.6%	91.4%
Mexico	Chanal	2000	75.9%	98.7%	26.1%
Mexico	Chanal	2017	95.2%	99.9%	70.3%
Mexico	Chankom	2000	80.3%	98.0%	41.3%
Mexico	Chankom	2017	95.9%	99.8%	76.9%
Mexico	Chapa De Mota	2000	83.8%	99.0%	36.8%
Mexico	Chapa De Mota	2017	97.5%	99.9%	83.0%
Mexico	Chapab	2000	75.3%	99.7%	22.8%
Mexico	Chapab	2017	94.8%	100.0%	65.9%
Mexico	Chapala	2000	82.1%	98.5%	42.8%
Mexico	Chapala	2017	96.6%	99.9%	79.0%
Mexico	Chapantongo	2000	78.2%	99.2%	33.7%
Mexico	Chapantongo	2017	94.9%	99.9%	75.1%
Mexico	Chapulco	2000	95.0%	99.5%	82.7%
Mexico	Chapulco	2017	99.5%	100.0%	97.8%
Mexico	Chapulhuacan	2000	76.4%	90.1%	53.0%
Mexico	Chapulhuacan	2017	96.7%	99.2%	87.7%
Mexico	Chapultenango	2000	55.7%	95.9%	5.5%
Mexico	Chapultenango	2017	84.0%	99.5%	31.3%

Country	Second Administrative Unit	Year	Mean	Upper	Lower
Mexico	Chapultepec	2000	94.4%	98.2%	87.0%
Mexico	Chapultepec	2017	99.9%	100.0%	99.6%
Mexico	Charapan	2000	80.5%	99.1%	41.2%
Mexico	Charapan	2017	96.2%	99.9%	81.2%
Mexico	Charcas	2000	69.5%	81.9%	49.4%
Mexico	Charcas	2017	95.6%	98.9%	85.4%
Mexico	Charo	2000	22.7%	46.0%	4.7%
Mexico	Charo	2017	43.6%	75.9%	18.8%
Mexico	Chavinda	2000	69.1%	98.9%	16.7%
Mexico	Chavinda	2017	91.2%	99.9%	54.2%
Mexico	Chemax	2000	91.3%	97.3%	80.7%
Mexico	Chemax	2017	98.5%	99.8%	94.6%
Mexico	Chenalho	2000	56.7%	94.5%	15.9%
Mexico	Chenalho	2017	85.5%	99.6%	45.2%
Mexico	Cheran	2000	75.0%	97.8%	28.1%
Mexico	Cheran	2017	94.3%	99.9%	66.1%
Mexico	Chiapa De Corzo	2000	56.4%	73.9%	36.8%
Mexico	Chiapa De Corzo	2017	92.1%	96.9%	80.9%
Mexico	Chiapilla	2000	75.0%	98.5%	23.8%
Mexico	Chiapilla	2017	94.5%	99.9%	60.2%
Mexico	Chiautempan	2000	98.3%	98.9%	97.3%
Mexico	Chiautempan	2017	99.9%	99.9%	99.8%
Mexico	Chiautla	2000	82.7%	94.3%	63.9%
Mexico	Chiautla	2000	89.7%	99.9%	44.3%
Mexico	Chiautla	2017	97.2%	99.6%	87.7%
Mexico	Chiautla	2017	99.0%	100.0%	92.2%
Mexico	Chiautzingo	2000	80.3%	85.6%	73.9%
Mexico	Chiautzingo	2017	98.6%	99.0%	98.1%
Mexico	Chichimila	2000	74.0%	97.0%	29.5%
Mexico	Chichimila	2017	93.5%	99.7%	75.6%
Mexico	Chichiquila	2000	50.6%	68.2%	32.1%
Mexico	Chichiquila	2017	91.6%	96.2%	81.6%
Mexico	Chicoasen	2000	73.4%	99.7%	9.1%
Mexico	Chicoasen	2017	91.8%	100.0%	43.0%
Mexico	Chicoloapan	2000	95.2%	96.7%	90.6%
Mexico	Chicoloapan	2017	99.6%	99.7%	99.4%
Mexico	Chicomuselo	2000	76.1%	95.5%	41.1%
Mexico	Chicomuselo	2017	94.4%	99.6%	73.9%
Mexico	Chiconamel	2000	27.0%	47.5%	8.7%
Mexico	Chiconamel	2017	74.8%	87.4%	53.7%
Mexico	Chiconcuac	2000	90.3%	99.8%	44.9%
Mexico	Chiconcuac	2017	99.3%	100.0%	95.6%
Mexico	Chiconcuautla	2000	92.0%	95.8%	85.5%
Mexico	Chiconcuautla	2017	99.3%	99.7%	98.5%
Mexico	Chiconquiaco	2000	72.7%	98.6%	20.8%
Mexico	Chiconquiaco	2017	92.4%	99.9%	60.8%
Mexico	Chicontepec	2000	66.0%	87.3%	37.0%
Mexico	Chicontepec	2017	91.5%	98.2%	75.5%
Mexico	Chicxulub Pueblo	2000	79.1%	99.8%	16.7%
Mexico	Chicxulub Pueblo	2017	96.4%	100.0%	75.1%
Mexico	Chietla	2000	60.6%	73.5%	39.2%
Mexico	Chietla	2017	94.7%	97.0%	86.7%
Mexico	Chigmecatitlan	2000	81.1%	99.9%	7.8%
Mexico	Chigmecatitlan	2017	95.6%	100.0%	54.7%
Mexico	Chignahuapan	2000	78.4%	96.8%	39.2%
Mexico	Chignahuapan	2017	93.2%	99.7%	62.4%
Mexico	Chignautla	2000	66.2%	83.9%	38.4%
Mexico	Chignautla	2017	97.7%	99.1%	92.9%

Country	Second Administrative Unit	Year	Mean	Upper	Lower
Mexico	Chihuahua	2000	95.0%	96.5%	92.5%
Mexico	Chihuahua	2017	99.6%	99.8%	99.2%
Mexico	Chikindzonot	2000	76.6%	99.0%	23.5%
Mexico	Chikindzonot	2017	93.9%	99.9%	58.5%
Mexico	Chila	2000	81.7%	99.8%	26.0%
Mexico	Chila	2017	95.8%	100.0%	70.5%
Mexico	Chila De La Sal	2000	96.0%	99.7%	84.3%
Mexico	Chila De La Sal	2017	99.7%	100.0%	98.7%
Mexico	Chilapa De Alvarez	2000	52.5%	69.4%	36.0%
Mexico	Chilapa De Alvarez	2017	90.9%	95.6%	82.3%
Mexico	Chilchota	2000	91.9%	97.5%	78.9%
Mexico	Chilchota	2017	99.3%	99.9%	96.9%
Mexico	Chilchotla	2000	74.6%	83.8%	61.3%
Mexico	Chilchotla	2017	93.8%	97.4%	88.5%
Mexico	Chilcuautla	2000	92.5%	99.7%	73.5%
Mexico	Chilcuautla	2017	98.9%	100.0%	93.2%
Mexico	Chilon	2000	65.1%	84.6%	43.9%
Mexico	Chilon	2017	89.2%	97.5%	75.2%
Mexico	Chilpancingo De Los Bravo	2000	58.5%	69.8%	47.7%
Mexico	Chilpancingo De Los Bravo	2017	90.9%	95.7%	83.3%
Mexico	Chimalhuacan	2000	95.5%	96.3%	94.5%
Mexico	Chimalhuacan	2017	99.8%	99.8%	99.7%
Mexico	Chimaltitan	2000	82.4%	98.7%	53.0%
Mexico	Chimaltitan	2017	96.3%	99.9%	81.9%
Mexico	China	2000	86.3%	98.0%	36.5%
Mexico	China	2017	97.1%	99.7%	84.3%
Mexico	Chinameca	2000	57.5%	94.8%	9.4%
Mexico	Chinameca	2017	89.2%	99.4%	47.4%
Mexico	Chinampa De Gorostiza	2000	50.4%	67.9%	33.2%
Mexico	Chinampa De Gorostiza	2017	91.1%	96.9%	82.7%
Mexico	Chinantla	2000	71.8%	98.7%	14.6%
Mexico	Chinantla	2017	94.6%	99.9%	61.9%
Mexico	Chinicuila	2000	75.5%	95.2%	45.8%
Mexico	Chinicuila	2017	94.0%	99.4%	78.4%
Mexico	Chinipas	2000	79.4%	92.9%	52.8%
Mexico	Chinipas	2017	95.3%	99.3%	82.8%
Mexico	Chiquihuitlan De Benito Juarez	2000	47.6%	90.9%	4.5%
Mexico	Chiquihuitlan De Benito Juarez	2017	86.2%	99.3%	36.9%
Mexico	Chiquilistlan	2000	90.8%	98.1%	76.1%
Mexico	Chiquilistlan	2017	99.1%	99.9%	96.5%
Mexico	Chocaman	2000	66.3%	73.6%	61.2%
Mexico	Chocaman	2017	77.0%	91.3%	64.5%
Mexico	Chochola	2000	79.5%	99.7%	18.7%
Mexico	Chochola	2017	95.1%	100.0%	65.3%
Mexico	Choix	2000	86.9%	95.0%	73.2%
Mexico	Choix	2017	97.2%	99.6%	90.6%
Mexico	Chontla	2000	86.1%	98.8%	55.8%
Mexico	Chontla	2017	97.4%	99.9%	88.7%
Mexico	Chucandiro	2000	81.8%	99.8%	29.1%
Mexico	Chucandiro	2017	96.1%	100.0%	74.0%

Country	Second Administrative Unit	Year	Mean	Upper	Lower
Mexico	Chumatlan	2000	26.5%	69.3%	0.4%
Mexico	Chumatlan	2017	62.9%	95.2%	6.8%
Mexico	Chumayel	2000	88.8%	99.8%	35.3%
Mexico	Chumayel	2017	97.9%	100.0%	83.5%
Mexico	Churintzio	2000	81.9%	99.6%	29.7%
Mexico	Churintzio	2017	96.4%	100.0%	72.2%
Mexico	Churumuco	2000	77.2%	96.6%	43.8%
Mexico	Churumuco	2017	94.2%	99.6%	69.4%
Mexico	Cienega De Flores	2000	83.6%	99.9%	24.6%
Mexico	Cienega De Flores	2017	97.0%	100.0%	74.6%
Mexico	Cienega De Zimatlan	2000	56.4%	99.5%	2.3%
Mexico	Cienega De Zimatlan	2017	86.0%	100.0%	24.3%
Mexico	Cihuatlan	2000	80.3%	99.3%	37.9%
Mexico	Cihuatlan	2017	96.2%	100.0%	77.3%
Mexico	Cintalapa	2000	74.6%	91.5%	49.4%
Mexico	Cintalapa	2017	95.9%	99.1%	89.4%
Mexico	Citlaltepetl	2000	95.7%	99.7%	82.2%
Mexico	Citlaltepetl	2017	99.6%	100.0%	98.1%
Mexico	Ciudad Del Maiz	2000	78.0%	87.2%	67.3%
Mexico	Ciudad Del Maiz	2017	95.8%	98.9%	89.7%
Mexico	Ciudad Fernandez	2000	85.0%	89.8%	79.6%
Mexico	Ciudad Fernandez	2017	98.5%	99.3%	96.9%
Mexico	Ciudad Ixtepec	2000	70.4%	99.0%	3.8%
Mexico	Ciudad Ixtepec	2017	90.9%	99.9%	25.9%
Mexico	Ciudad Madero	2000	95.6%	97.4%	92.7%
Mexico	Ciudad Madero	2017	98.7%	99.7%	96.1%
Mexico	Ciudad Valles	2000	77.7%	87.4%	64.6%
Mexico	Ciudad Valles	2017	96.8%	98.9%	90.0%
Mexico	Coacalco De Berriozabal	2000	99.0%	99.8%	97.0%
Mexico	Coacalco De Berriozabal	2017	99.9%	100.0%	99.8%
Mexico	Coacoatzintla	2000	96.0%	99.7%	85.4%
Mexico	Coacoatzintla	2017	99.6%	100.0%	97.9%
Mexico	Coahuayana	2000	78.1%	99.4%	19.4%
Mexico	Coahuayana	2017	94.7%	100.0%	61.5%
Mexico	Coahuayutla De Jose Maria Izazaga	2000	77.5%	93.4%	56.2%
Mexico	Coahuayutla De Jose Maria Izazaga	2017	94.9%	99.2%	85.7%
Mexico	Coahuitlan	2000	70.3%	99.0%	4.9%
Mexico	Coahuitlan	2017	90.6%	99.9%	40.9%
Mexico	Coalcoman De Vazquez Pallares	2000	81.0%	96.3%	41.0%
Mexico	Coalcoman De Vazquez Pallares	2017	96.3%	99.6%	83.9%

Country	Second Administrative Unit	Year	Mean	Upper	Lower
Mexico	Coapilla	2000	73.4%	97.5%	21.5%
Mexico	Coapilla	2017	93.9%	99.9%	63.3%
Mexico	Coatecas Altas	2000	71.3%	99.0%	10.8%
Mexico	Coatecas Altas	2017	93.5%	99.9%	61.6%
Mexico	Coatepec	2000	54.7%	59.3%	44.6%
Mexico	Coatepec	2000	86.4%	97.6%	61.1%
Mexico	Coatepec	2017	99.2%	99.9%	97.1%
Mexico	Coatepec	2017	56.4%	71.3%	50.1%
Mexico	Coatepec Harinas	2000	78.3%	99.1%	25.0%
Mexico	Coatepec Harinas	2017	94.7%	99.9%	65.5%
Mexico	Coatlan Del Rio	2000	76.8%	92.7%	58.9%
Mexico	Coatlan Del Rio	2017	96.4%	99.6%	89.1%
Mexico	Coatzacoalcos	2000	58.0%	76.0%	40.3%
Mexico	Coatzacoalcos	2017	93.6%	98.0%	80.4%
Mexico	Coatzingo	2000	77.0%	99.9%	8.8%
Mexico	Coatzingo	2017	93.2%	100.0%	38.5%
Mexico	Coatzintla	2000	67.6%	76.9%	52.4%
Mexico	Coatzintla	2017	96.7%	97.8%	93.6%
Mexico	Cochoapa El Grande	2000	76.2%	96.8%	37.3%
Mexico	Cochoapa El Grande	2017	93.3%	99.7%	61.3%
Mexico	Cocotitlan	2000	98.5%	99.7%	95.3%
Mexico	Cocotitlan	2017	99.9%	100.0%	99.7%
Mexico	Cocula	2000	88.0%	97.9%	69.7%
Mexico	Cocula	2000	85.1%	99.3%	32.8%
Mexico	Cocula	2017	97.6%	99.9%	86.1%
Mexico	Cocula	2017	96.8%	100.0%	78.0%
Mexico	Coeneo	2000	63.0%	90.0%	18.9%
Mexico	Coeneo	2017	86.5%	98.9%	57.7%
Mexico	Coetzala	2000	36.9%	81.2%	9.9%
Mexico	Coetzala	2017	85.0%	98.6%	57.1%
Mexico	Cohetzala	2000	77.0%	99.4%	25.7%
Mexico	Cohetzala	2017	95.2%	99.9%	73.6%
Mexico	Cohuecan	2000	72.6%	87.9%	50.7%
Mexico	Cohuecan	2017	96.5%	98.7%	89.4%
Mexico	Coicoyan De Las Flores	2000	73.6%	99.0%	23.2%
Mexico	Coicoyan De Las Flores	2017	93.4%	99.9%	70.3%
Mexico	Cojumatlan De Regules	2000	80.9%	99.7%	18.0%
Mexico	Cojumatlan De Regules	2017	95.8%	100.0%	71.0%
Mexico	Colima	2000	97.3%	98.6%	95.1%
Mexico	Colima	2017	99.7%	99.9%	98.9%
Mexico	Colipa	2000	70.5%	90.9%	42.7%
Mexico	Colipa	2017	93.5%	98.9%	81.6%
Mexico	Colon	2000	89.8%	98.4%	66.5%
Mexico	Colon	2017	98.3%	99.9%	90.7%
Mexico	Colotlan	2000	83.8%	99.0%	20.3%
Mexico	Colotlan	2017	96.4%	99.9%	72.8%
Mexico	Comala	2000	95.9%	98.9%	88.6%
Mexico	Comala	2017	99.6%	99.9%	97.7%
Mexico	Comalcalco	2000	50.9%	73.0%	31.5%
Mexico	Comalcalco	2017	88.6%	96.4%	74.1%

Country	Second Administrative Unit	Year	Mean	Upper	Lower
Mexico	Comapa	2000	75.1%	93.8%	44.8%
Mexico	Comapa	2017	94.9%	99.6%	80.1%
Mexico	Comitan De Dominguez	2000	88.0%	95.2%	73.1%
Mexico	Comitan De Dominguez	2017	98.2%	99.5%	94.6%
Mexico	Comondu	2000	93.2%	96.6%	86.6%
Mexico	Comondu	2017	98.8%	99.5%	97.3%
Mexico	Comonfort	2000	75.2%	88.6%	58.4%
Mexico	Comonfort	2017	96.2%	99.1%	87.0%
Mexico	Compostela	2000	77.3%	95.8%	46.8%
Mexico	Compostela	2017	95.6%	99.6%	80.0%
Mexico	Concepcion Buenavista	2000	74.3%	99.1%	21.2%
Mexico	Concepcion Buenavista	2017	93.3%	99.9%	55.4%
Mexico	Concepcion De Buenos Aires	2000	78.8%	99.7%	17.2%
Mexico	Concepcion De Buenos Aires	2017	95.2%	100.0%	62.9%
Mexico	Concepcion Del Oro	2000	81.0%	95.7%	43.6%
Mexico	Concepcion Del Oro	2017	97.1%	99.6%	87.1%
Mexico	Concepcion Papalo	2000	87.3%	96.5%	65.5%
Mexico	Concepcion Papalo	2017	98.2%	99.7%	91.4%
Mexico	Concordia	2000	88.0%	96.3%	75.7%
Mexico	Concordia	2017	97.8%	99.6%	93.1%
Mexico	Coneto De Comonfort	2000	80.5%	97.3%	45.5%
Mexico	Coneto De Comonfort	2017	96.4%	99.8%	82.6%
Mexico	Conkal	2000	85.1%	99.8%	32.6%
Mexico	Conkal	2017	97.3%	100.0%	83.3%
Mexico	Constancia Del Rosario	2000	69.5%	95.6%	33.0%
Mexico	Constancia Del Rosario	2017	95.3%	99.7%	83.0%
Mexico	Contepec	2000	79.9%	95.9%	52.2%
Mexico	Contepec	2017	96.6%	99.7%	85.4%
Mexico	Contla De Juan Cuamatzi	2000	98.1%	98.8%	97.0%
Mexico	Contla De Juan Cuamatzi	2017	99.9%	99.9%	99.8%
Mexico	Copainala	2000	88.6%	98.9%	66.6%
Mexico	Copainala	2017	97.7%	99.9%	89.8%
Mexico	Copala	2000	88.8%	98.8%	65.9%
Mexico	Copala	2017	98.0%	99.9%	90.4%
Mexico	Copalillo	2000	68.8%	93.3%	34.3%
Mexico	Copalillo	2017	93.1%	99.1%	75.9%
Mexico	Copanatoyac	2000	23.6%	43.8%	7.0%
Mexico	Copanatoyac	2017	70.0%	87.0%	41.1%
Mexico	Copandaro	2000	82.9%	95.8%	53.8%
Mexico	Copandaro	2017	97.1%	99.6%	84.2%
Mexico	Coquimatlan	2000	96.3%	99.7%	87.4%
Mexico	Coquimatlan	2017	99.4%	100.0%	96.8%

Country	Second Administrative Unit	Year	Mean	Upper	Lower
Mexico	Cordoba	2000	79.6%	83.9%	75.0%
Mexico	Cordoba	2017	98.0%	98.6%	96.4%
Mexico	Coronado	2000	82.7%	97.3%	48.0%
Mexico	Coronado	2017	97.1%	99.8%	86.8%
Mexico	Coronango	2000	62.8%	67.3%	57.1%
Mexico	Coronango	2017	95.1%	96.1%	94.0%
Mexico	Coroneo	2000	79.8%	99.5%	23.2%
Mexico	Coroneo	2017	95.7%	100.0%	70.2%
Mexico	Corregidora	2000	93.9%	98.0%	86.5%
Mexico	Corregidora	2017	99.2%	99.9%	96.7%
Mexico	Cortazar	2000	88.9%	94.0%	81.0%
Mexico	Cortazar	2017	98.8%	99.5%	97.0%
Mexico	Cosala	2000	83.3%	94.8%	59.2%
Mexico	Cosala	2017	96.3%	99.5%	88.4%
Mexico	Cosamaloapan De Carpio	2000	62.3%	76.9%	46.2%
Mexico	Cosamaloapan De Carpio	2017	83.5%	95.8%	71.0%
Mexico	Cosautlan De Carvajal	2000	87.2%	99.9%	33.7%
Mexico	Cosautlan De Carvajal	2017	98.0%	100.0%	84.7%
Mexico	Coscomatepec	2000	75.2%	83.7%	66.5%
Mexico	Coscomatepec	2017	82.5%	96.9%	74.3%
Mexico	Cosio	2000	88.8%	99.6%	51.9%
Mexico	Cosio	2017	98.5%	100.0%	89.6%
Mexico	Cosolapa	2000	68.6%	99.4%	16.7%
Mexico	Cosolapa	2017	92.4%	100.0%	57.2%
Mexico	Cosoleacaque	2000	55.8%	66.9%	43.5%
Mexico	Cosoleacaque	2017	92.6%	96.4%	84.6%
Mexico	Cosoltepec	2000	81.5%	99.8%	21.3%
Mexico	Cosoltepec	2017	95.9%	100.0%	73.6%
Mexico	Cotaxtla	2000	77.1%	97.3%	36.9%
Mexico	Cotaxtla	2017	94.7%	99.8%	73.2%
Mexico	Cotija	2000	81.3%	99.4%	22.6%
Mexico	Cotija	2017	96.1%	100.0%	72.5%
Mexico	Coxcatlan	2000	70.8%	87.7%	51.3%
Mexico	Coxcatlan	2000	50.2%	95.9%	4.1%
Mexico	Coxcatlan	2017	95.6%	99.1%	84.1%
Mexico	Coxcatlan	2017	82.7%	99.4%	33.6%
Mexico	Coxquihui	2000	32.3%	72.1%	5.4%
Mexico	Coxquihui	2017	66.7%	93.3%	25.9%
Mexico	Coyame Del Sotol	2000	82.9%	96.5%	52.0%
Mexico	Coyame Del Sotol	2017	95.4%	99.5%	75.0%
Mexico	Coyoacan	2000	99.3%	99.6%	98.9%
Mexico	Coyoacan	2017	100.0%	100.0%	99.9%
Mexico	Coyomeapan	2000	62.4%	77.4%	46.6%
Mexico	Coyomeapan	2017	94.5%	97.8%	87.5%
Mexico	Coyotepec	2000	89.1%	99.7%	53.3%
Mexico	Coyotepec	2000	79.7%	99.5%	26.6%
Mexico	Coyotepec	2017	97.8%	100.0%	77.9%
Mexico	Coyotepec	2017	96.0%	100.0%	76.7%
Mexico	Coyuca De Benitez	2000	69.2%	90.5%	37.0%
Mexico	Coyuca De Benitez	2017	92.0%	99.1%	74.3%
Mexico	Coyuca De Catalan	2000	62.8%	84.4%	41.2%
Mexico	Coyuca De Catalan	2017	90.0%	97.4%	73.0%

Country	Second Administrative Unit	Year	Mean	Upper	Lower
Mexico	Coyutla	2000	56.6%	87.1%	18.8%
Mexico	Coyutla	2017	86.5%	99.0%	47.3%
Mexico	Cozumel	2000	87.5%	95.0%	75.3%
Mexico	Cozumel	2017	98.6%	99.7%	94.1%
Mexico	Cruillas	2000	74.3%	95.5%	41.9%
Mexico	Cruillas	2017	93.7%	99.5%	75.6%
Mexico	Cuajimalpa De Morelos	2000	95.5%	98.5%	89.8%
Mexico	Cuajimalpa De Morelos	2017	99.6%	99.9%	98.3%
Mexico	Cuajinicuilapa	2000	70.4%	93.2%	33.2%
Mexico	Cuajinicuilapa	2017	92.9%	99.5%	71.6%
Mexico	Cualac	2000	77.4%	97.2%	23.4%
Mexico	Cualac	2017	95.6%	99.8%	70.0%
Mexico	Cuapiaxtla	2000	91.8%	99.6%	53.1%
Mexico	Cuapiaxtla	2017	98.8%	100.0%	90.1%
Mexico	Cuapiaxtla De Madero	2000	86.3%	95.9%	52.0%
Mexico	Cuapiaxtla De Madero	2017	98.4%	99.6%	90.0%
Mexico	Cuatrocienegas	2000	89.4%	98.5%	45.3%
Mexico	Cuatrocienegas	2017	98.4%	99.8%	91.0%
Mexico	Cuauhtemoc	2000	86.6%	98.6%	56.2%
Mexico	Cuauhtemoc	2000	99.4%	99.7%	98.9%
Mexico	Cuauhtemoc	2000	85.3%	95.4%	58.9%
Mexico	Cuauhtemoc	2000	87.9%	93.7%	80.0%
Mexico	Cuauhtemoc	2017	98.8%	99.5%	97.4%
Mexico	Cuauhtemoc	2017	98.2%	99.9%	89.0%
Mexico	Cuauhtemoc	2017	97.8%	99.7%	83.8%
Mexico	Cuauhtemoc	2017	100.0%	100.0%	99.9%
Mexico	Cuautempan	2000	59.6%	99.1%	3.6%
Mexico	Cuautempan	2017	87.2%	99.9%	27.9%
Mexico	Cuautepec	2000	54.4%	67.6%	41.8%
Mexico	Cuautepec	2017	92.1%	97.6%	81.8%
Mexico	Cuautepec De Hinojosa	2000	83.5%	92.1%	71.9%
Mexico	Cuautepec De Hinojosa	2017	96.9%	99.3%	90.0%
Mexico	Cuautinchan	2000	84.9%	99.5%	48.1%
Mexico	Cuautinchan	2017	97.4%	100.0%	87.2%
Mexico	Cuautitlan	2000	90.5%	96.3%	78.6%
Mexico	Cuautitlan	2017	99.4%	99.8%	98.3%
Mexico	Cuautitlan De Garcia Barragan	2000	81.8%	97.0%	57.1%
Mexico	Cuautitlan De Garcia Barragan	2017	96.2%	99.7%	84.6%
Mexico	Cuautitlan Izcalli	2000	89.1%	93.9%	81.0%
Mexico	Cuautitlan Izcalli	2017	99.2%	99.6%	98.2%
Mexico	Cuautla	2000	94.8%	97.6%	89.7%
Mexico	Cuautla	2000	81.4%	98.4%	37.6%
Mexico	Cuautla	2017	99.6%	99.8%	99.1%
Mexico	Cuautla	2017	95.9%	99.9%	77.7%
Mexico	Cuautlancingo	2000	49.5%	54.2%	45.1%
Mexico	Cuautlancingo	2017	92.5%	94.0%	90.7%
Mexico	Cuaxomulco	2000	99.5%	100.0%	97.3%
Mexico	Cuaxomulco	2017	100.0%	100.0%	99.9%
Mexico	Cuayuca De Andrade	2000	80.4%	99.7%	21.0%

Country	Second Administrative Unit	Year	Mean	Upper	Lower
Mexico	Cuayuca De Andrade	2017	95.6%	100.0%	64.5%
Mexico	Cucurpe	2000	83.6%	99.4%	29.7%
Mexico	Cucurpe	2017	96.9%	99.9%	78.5%
Mexico	Cuencame	2000	79.3%	88.7%	66.9%
Mexico	Cuencame	2017	96.7%	98.8%	92.5%
Mexico	Cueramaro	2000	96.1%	99.7%	83.9%
Mexico	Cueramaro	2017	99.7%	100.0%	98.5%
Mexico	Cuernavaca	2000	88.8%	93.3%	81.5%
Mexico	Cuernavaca	2017	98.7%	99.5%	96.0%
Mexico	Cuetzala Del Progreso	2000	81.2%	98.8%	35.1%
Mexico	Cuetzala Del Progreso	2017	96.2%	99.9%	79.2%
Mexico	Cuetzalan Del Progreso	2000	68.3%	97.4%	22.0%
Mexico	Cuetzalan Del Progreso	2017	92.2%	99.8%	63.4%
Mexico	Cuichapa	2000	49.9%	91.5%	9.1%
Mexico	Cuichapa	2017	85.9%	99.3%	43.7%
Mexico	Cuilapam De Guerrero	2000	65.9%	77.6%	53.6%
Mexico	Cuilapam De Guerrero	2017	95.8%	97.5%	92.4%
Mexico	Cuitlahuac	2000	78.7%	99.1%	26.0%
Mexico	Cuitlahuac	2017	95.3%	99.9%	77.7%
Mexico	Cuitzeo	2000	77.9%	97.5%	36.7%
Mexico	Cuitzeo	2017	96.1%	99.8%	79.9%
Mexico	Culiacan	2000	92.3%	94.5%	89.2%
Mexico	Culiacan	2017	99.1%	99.5%	98.2%
Mexico	Cumpas	2000	90.8%	99.4%	66.1%
Mexico	Cumpas	2017	98.5%	100.0%	90.0%
Mexico	Cuncunul	2000	77.1%	99.8%	19.9%
Mexico	Cuncunul	2017	95.2%	100.0%	74.0%
Mexico	Cunduacan	2000	60.1%	82.5%	39.2%
Mexico	Cunduacan	2017	89.6%	98.3%	73.9%
Mexico	Cuquio	2000	46.5%	60.7%	30.3%
Mexico	Cuquio	2017	85.9%	93.1%	73.2%
Mexico	Cusihuiriachi	2000	76.5%	96.7%	46.4%
Mexico	Cusihuiriachi	2017	95.3%	99.6%	82.3%
Mexico	Cutzamala De Pinzon	2000	76.1%	93.5%	49.7%
Mexico	Cutzamala De Pinzon	2017	94.8%	99.4%	82.8%
Mexico	Cuyamecalco Villa De Zaragoza	2000	67.6%	96.5%	10.4%
Mexico	Cuyamecalco Villa De Zaragoza	2017	91.3%	99.7%	46.4%
Mexico	Cuyoaco	2000	79.4%	98.9%	40.7%
Mexico	Cuyoaco	2017	95.6%	99.9%	79.2%
Mexico	Cuzama	2000	87.5%	99.8%	40.4%
Mexico	Cuzama	2017	98.5%	100.0%	88.4%
Mexico	Degollado	2000	90.0%	99.2%	72.4%
Mexico	Degollado	2017	97.9%	99.9%	87.9%
Mexico	Del Nayar	2000	70.8%	81.8%	55.6%
Mexico	Del Nayar	2017	92.3%	95.8%	86.7%
Mexico	Delicias	2000	90.6%	96.1%	76.2%
Mexico	Delicias	2017	98.8%	99.7%	95.3%
Mexico	Divisaderos	2000	83.8%	99.9%	16.4%
Mexico	Divisaderos	2017	97.1%	100.0%	76.7%

Country	Second Administrative Unit	Year	Mean	Upper	Lower
Mexico	Doctor Mora	2000	87.3%	98.1%	66.6%
Mexico	Doctor Mora	2017	98.3%	99.8%	91.9%
Mexico	Dolores Hidalgo Cuna De La Independenc	2000	86.9%	94.8%	74.3%
Mexico	Dolores Hidalgo Cuna De La Independenc	2017	97.5%	99.6%	92.3%
Mexico	Domingo Arenas	2000	91.0%	95.5%	83.1%
Mexico	Domingo Arenas	2017	99.4%	99.7%	98.8%
Mexico	Donato Guerra	2000	58.0%	84.0%	30.6%
Mexico	Donato Guerra	2017	90.8%	98.6%	71.5%
Mexico	Dr. Arroyo	2000	83.9%	93.3%	71.7%
Mexico	Dr. Arroyo	2017	97.6%	99.3%	94.1%
Mexico	Dr. Belisario Dominguez	2000	84.3%	99.1%	50.7%
Mexico	Dr. Belisario Dominguez	2017	97.0%	99.9%	82.0%
Mexico	Dr. Coss	2000	85.6%	98.7%	58.1%
Mexico	Dr. Coss	2017	96.9%	99.9%	81.7%
Mexico	Dr. Gonzalez	2000	64.3%	94.5%	24.5%
Mexico	Dr. Gonzalez	2017	90.5%	99.4%	59.8%
Mexico	Durango	2000	93.1%	96.1%	87.7%
Mexico	Durango	2017	99.3%	99.7%	98.6%
Mexico	Dzan	2000	78.2%	98.4%	32.4%
Mexico	Dzan	2017	95.9%	99.9%	76.8%
Mexico	Dzemul	2000	80.9%	99.8%	17.4%
Mexico	Dzemul	2017	97.4%	100.0%	81.7%
Mexico	Dzidzantun	2000	82.9%	99.9%	12.8%
Mexico	Dzidzantun	2017	95.8%	100.0%	63.2%
Mexico	Dzilam De Bravo	2000	72.0%	99.7%	5.4%
Mexico	Dzilam De Bravo	2017	93.3%	100.0%	45.6%
Mexico	Dzilam Gonzalez	2000	88.4%	99.6%	30.9%
Mexico	Dzilam Gonzalez	2017	98.1%	100.0%	87.1%
Mexico	Dzitas	2000	74.2%	99.3%	26.4%
Mexico	Dzitas	2017	93.6%	100.0%	56.1%
Mexico	Dzoncauich	2000	75.0%	99.9%	9.4%
Mexico	Dzoncauich	2017	96.2%	100.0%	69.2%
Mexico	Ebano	2000	73.0%	94.0%	39.2%
Mexico	Ebano	2017	94.0%	99.5%	74.6%
Mexico	Ecatepec De Morelos	2000	96.6%	97.3%	95.7%
Mexico	Ecatepec De Morelos	2017	99.8%	99.8%	99.7%
Mexico	Ecatzingo	2000	86.7%	99.7%	45.7%
Mexico	Ecatzingo	2017	97.1%	100.0%	72.9%
Mexico	Ecuandureo	2000	76.5%	96.0%	36.2%
Mexico	Ecuandureo	2017	93.4%	99.6%	77.5%
Mexico	Eduardo Neri	2000	46.1%	81.8%	24.5%
Mexico	Eduardo Neri	2017	73.8%	94.9%	42.1%
Mexico	Ejutla	2000	81.0%	99.4%	40.8%
Mexico	Ejutla	2017	96.3%	100.0%	81.6%

Country	Second Administrative Unit	Year	Mean	Upper	Lower
Mexico	El Arenal	2000	75.4%	99.6%	16.1%
Mexico	El Arenal	2000	87.2%	96.7%	67.8%
Mexico	El Arenal	2017	98.2%	99.7%	90.2%
Mexico	El Arenal	2017	95.5%	100.0%	67.2%
Mexico	El Barrio De La Soledad	2000	80.6%	93.0%	61.2%
Mexico	El Barrio De La Soledad	2017	97.9%	99.5%	92.7%
Mexico	El Bosque	2000	58.6%	93.3%	12.1%
Mexico	El Bosque	2017	89.0%	99.4%	48.0%
Mexico	El Carmen Tequexquitla	2000	97.2%	99.8%	88.2%
Mexico	El Carmen Tequexquitla	2017	99.7%	100.0%	98.5%
Mexico	El Espinal	2000	61.2%	66.3%	55.5%
Mexico	El Espinal	2017	95.9%	97.1%	93.8%
Mexico	El Fuerte	2000	88.7%	94.2%	82.0%
Mexico	El Fuerte	2017	98.3%	99.5%	95.9%
Mexico	El Grullo	2000	88.2%	96.5%	72.6%
Mexico	El Grullo	2017	99.0%	99.8%	96.6%
Mexico	El Higo	2000	82.3%	92.6%	67.1%
Mexico	El Higo	2017	96.1%	99.4%	90.2%
Mexico	El Limon	2000	80.8%	99.5%	22.2%
Mexico	El Limon	2017	96.4%	100.0%	73.4%
Mexico	El Llano	2000	83.9%	98.6%	47.4%
Mexico	El Llano	2017	96.5%	99.9%	80.1%
Mexico	El Mante	2000	90.9%	95.5%	84.3%
Mexico	El Mante	2017	98.9%	99.7%	96.5%
Mexico	El Marques	2000	94.0%	98.9%	82.6%
Mexico	El Marques	2017	99.0%	99.9%	94.5%
Mexico	El Naranjo	2000	69.3%	90.2%	38.2%
Mexico	El Naranjo	2017	93.8%	99.2%	81.0%
Mexico	El Oro	2000	66.4%	97.0%	18.5%
Mexico	El Oro	2000	82.5%	91.8%	66.6%
Mexico	El Oro	2017	97.1%	99.1%	92.2%
Mexico	El Oro	2017	90.9%	99.7%	59.9%
Mexico	El Plateado De Joaquin Amaro	2000	82.6%	99.2%	45.8%
Mexico	El Plateado De Joaquin Amaro	2017	97.2%	100.0%	82.1%
Mexico	El Porvenir	2000	55.2%	80.3%	30.1%
Mexico	El Porvenir	2017	92.4%	98.6%	67.8%
Mexico	El Salto	2000	97.8%	99.0%	95.0%
Mexico	El Salto	2017	99.9%	99.9%	99.6%
Mexico	El Salvador	2000	83.0%	99.4%	48.4%
Mexico	El Salvador	2017	96.4%	100.0%	74.0%
Mexico	El Tule	2000	85.9%	98.1%	56.3%
Mexico	El Tule	2017	98.3%	99.8%	90.5%
Mexico	Elota	2000	88.9%	97.0%	74.3%
Mexico	Elota	2017	98.3%	99.7%	93.7%
Mexico	Eloxochitlan	2000	71.9%	88.2%	52.7%
Mexico	Eloxochitlan	2000	76.1%	99.5%	17.4%
Mexico	Eloxochitlan	2017	95.2%	98.6%	86.0%
Mexico	Eloxochitlan	2017	94.7%	100.0%	59.0%
Mexico	Eloxochitlan De Flores Magon	2000	77.7%	91.9%	50.1%
Mexico	Eloxochitlan De Flores Magon	2017	95.9%	98.7%	83.4%

Country	Second Administrative Unit	Year	Mean	Upper	Lower
Mexico	Emiliano Zapata	2000	84.5%	99.0%	47.2%
Mexico	Emiliano Zapata	2000	88.8%	93.0%	82.2%
Mexico	Emiliano Zapata	2000	89.5%	97.4%	76.2%
Mexico	Emiliano Zapata	2000	59.6%	80.4%	27.6%
Mexico	Emiliano Zapata	2000	73.0%	99.7%	7.6%
Mexico	Emiliano Zapata	2017	99.2%	99.6%	98.2%
Mexico	Emiliano Zapata	2017	97.6%	99.8%	91.0%
Mexico	Emiliano Zapata	2017	97.9%	99.9%	89.5%
Mexico	Emiliano Zapata	2017	92.8%	100.0%	38.6%
Mexico	Emiliano Zapata	2017	89.9%	98.1%	62.1%
Mexico	Empalme	2000	96.9%	99.4%	90.8%
Mexico	Empalme	2017	99.6%	100.0%	98.5%
Mexico	Encarnacion De Diaz	2000	94.1%	98.5%	86.8%
Mexico	Encarnacion De Diaz	2017	99.1%	99.9%	96.1%
Mexico	Ensenada	2000	86.5%	91.2%	80.3%
Mexico	Ensenada	2017	98.5%	99.2%	97.2%
Mexico	Epatlan	2000	83.1%	99.9%	23.2%
Mexico	Epatlan	2017	96.8%	100.0%	75.1%
Mexico	Epazoyucan	2000	96.2%	99.9%	80.0%
Mexico	Epazoyucan	2017	99.7%	100.0%	97.8%
Mexico	Epitacio Huerta	2000	78.6%	98.0%	38.6%
Mexico	Epitacio Huerta	2017	95.1%	99.8%	78.5%
Mexico	Erongaricuaro	2000	74.7%	98.6%	22.8%
Mexico	Erongaricuaro	2017	93.3%	99.9%	59.5%
Mexico	Escarcega	2000	84.5%	91.1%	78.2%
Mexico	Escarcega	2017	96.9%	99.0%	93.6%
Mexico	Escobedo	2000	83.3%	98.9%	44.5%
Mexico	Escobedo	2017	96.7%	99.9%	82.5%
Mexico	Escuinapa	2000	77.6%	95.8%	42.5%
Mexico	Escuinapa	2017	95.9%	99.7%	85.5%
Mexico	Escuintla	2000	61.5%	93.8%	20.6%
Mexico	Escuintla	2017	90.4%	99.3%	58.8%
Mexico	Espanita	2000	96.9%	99.6%	88.4%
Mexico	Espanita	2017	99.5%	100.0%	97.7%
Mexico	Esperanza	2000	75.0%	97.7%	24.4%
Mexico	Esperanza	2017	95.3%	99.8%	77.8%
Mexico	Espinal	2000	59.7%	88.4%	27.6%
Mexico	Espinal	2017	89.3%	98.8%	66.7%
Mexico	Espita	2000	94.0%	99.0%	80.1%
Mexico	Espita	2017	99.1%	99.9%	96.3%
Mexico	Etchojoa	2000	96.1%	99.6%	87.1%
Mexico	Etchojoa	2017	99.4%	100.0%	96.2%
Mexico	Etzatlan	2000	83.5%	99.3%	32.8%
Mexico	Etzatlan	2017	97.0%	100.0%	76.9%
Mexico	Ezequiel Montes	2000	90.0%	99.4%	58.0%
Mexico	Ezequiel Montes	2017	98.8%	99.9%	92.7%

Country	Second Administrative Unit	Year	Mean	Upper	Lower
Mexico	Felipe Carrillo Puerto	2000	72.9%	82.0%	62.7%
Mexico	Felipe Carrillo Puerto	2017	93.2%	96.9%	87.0%
Mexico	Filomeno Mata	2000	39.9%	79.4%	6.5%
Mexico	Filomeno Mata	2017	86.3%	97.7%	49.0%
Mexico	Florencio Villarreal	2000	72.3%	92.2%	36.9%
Mexico	Florencio Villarreal	2017	95.6%	99.1%	80.5%
Mexico	Fortin	2000	87.8%	92.1%	83.3%
Mexico	Fortin	2017	96.0%	98.8%	92.8%
Mexico	Francisco I. Madero	2000	87.3%	94.8%	70.8%
Mexico	Francisco I. Madero	2000	95.9%	98.7%	87.4%
Mexico	Francisco I. Madero	2017	98.7%	99.5%	95.9%
Mexico	Francisco I. Madero	2017	99.5%	99.9%	98.1%
Mexico	Francisco Leon	2000	64.8%	96.3%	13.5%
Mexico	Francisco Leon	2017	89.2%	99.8%	50.5%
Mexico	Francisco Z. Mena	2000	81.0%	98.7%	45.8%
Mexico	Francisco Z. Mena	2017	96.0%	99.9%	83.7%
Mexico	Fresnillo	2000	88.0%	92.2%	82.9%
Mexico	Fresnillo	2017	98.6%	99.3%	97.2%
Mexico	Fresnillo De Trujano	2000	68.2%	96.5%	16.7%
Mexico	Fresnillo De Trujano	2017	91.9%	99.8%	47.6%
Mexico	Frontera	2000	96.4%	98.3%	93.1%
Mexico	Frontera	2017	99.8%	99.9%	99.5%
Mexico	Frontera Comalapa	2000	68.9%	82.2%	53.1%
Mexico	Frontera Comalapa	2017	94.1%	98.0%	85.0%
Mexico	Frontera Hidalgo	2000	82.5%	99.2%	46.6%
Mexico	Frontera Hidalgo	2017	96.8%	99.9%	84.6%
Mexico	Fronteras	2000	92.3%	99.5%	60.5%
Mexico	Fronteras	2017	99.1%	100.0%	95.4%
Mexico	Gabriel Zamora	2000	84.8%	94.9%	66.3%
Mexico	Gabriel Zamora	2017	97.5%	99.6%	91.4%
Mexico	Galeana	2000	82.3%	90.4%	71.6%
Mexico	Galeana	2000	86.3%	98.7%	53.5%
Mexico	Galeana	2017	96.6%	99.0%	92.4%
Mexico	Galeana	2017	97.8%	99.9%	82.6%
Mexico	Garcia	2000	96.6%	98.7%	92.9%
Mexico	Garcia	2017	99.7%	99.9%	98.9%
Mexico	Genaro Codina	2000	80.0%	98.0%	35.9%
Mexico	Genaro Codina	2017	96.4%	99.8%	79.1%

Country	Second Administrative Unit	Year	Mean	Upper	Lower
Mexico	General Bravo	2000	90.1%	98.4%	61.8%
Mexico	General Bravo	2017	98.6%	99.8%	94.3%
Mexico	General Canuto A. Neri	2000	78.3%	98.8%	36.5%
Mexico	General Canuto A. Neri	2017	95.6%	100.0%	77.5%
Mexico	General Cepeda	2000	84.1%	93.9%	65.1%
Mexico	General Cepeda	2017	97.2%	99.1%	92.0%
Mexico	General Enrique Estrada	2000	85.8%	97.5%	53.2%
Mexico	General Enrique Estrada	2017	98.5%	99.7%	94.2%
Mexico	General Felipe Angeles	2000	87.4%	99.9%	37.8%
Mexico	General Felipe Angeles	2017	97.4%	100.0%	78.7%
Mexico	General Francisco R. Murguia	2000	75.0%	88.4%	58.4%
Mexico	General Francisco R. Murguia	2017	95.7%	98.8%	90.4%
Mexico	General Heliodoro Castillo	2000	49.3%	65.8%	30.9%
Mexico	General Heliodoro Castillo	2017	71.4%	83.1%	54.0%
Mexico	General Panfilo Natera	2000	93.4%	98.3%	81.8%
Mexico	General Panfilo Natera	2017	99.1%	99.9%	96.0%
Mexico	General Plutarco Elias Calles	2000	94.0%	99.3%	74.2%
Mexico	General Plutarco Elias Calles	2017	99.1%	99.9%	95.6%
Mexico	Gomez Farias	2000	85.8%	98.9%	56.2%
Mexico	Gomez Farias	2000	80.7%	99.5%	25.7%
Mexico	Gomez Farias	2000	86.1%	97.6%	62.8%
Mexico	Gomez Farias	2017	96.6%	100.0%	71.0%
Mexico	Gomez Farias	2017	97.7%	99.9%	88.4%
Mexico	Gomez Farias	2017	98.2%	99.8%	92.1%
Mexico	Gomez Palacio	2000	94.6%	96.2%	92.0%
Mexico	Gomez Palacio	2017	99.5%	99.7%	99.0%
Mexico	Gonzalez	2000	85.3%	95.9%	66.6%
Mexico	Gonzalez	2017	96.8%	99.6%	88.3%
Mexico	Gral. Escobedo	2000	98.4%	99.1%	96.9%
Mexico	Gral. Escobedo	2017	99.9%	99.9%	99.7%
Mexico	Gral. Simon Bolivar	2000	88.9%	97.1%	77.0%
Mexico	Gral. Simon Bolivar	2017	98.1%	99.8%	93.3%

Country	Second Administrative Unit	Year	Mean	Upper	Lower
Mexico	Gral. Teran	2000	80.3%	92.1%	60.2%
Mexico	Gral. Teran	2017	97.2%	99.2%	91.5%
Mexico	Gral. Trevino	2000	75.0%	99.7%	9.4%
Mexico	Gral. Trevino	2017	93.3%	100.0%	41.9%
Mexico	Gral. Zaragoza	2000	75.4%	94.0%	41.9%
Mexico	Gral. Zaragoza	2017	95.1%	99.5%	78.2%
Mexico	Gral. Zuazua	2000	87.6%	99.6%	46.0%
Mexico	Gral. Zuazua	2017	97.8%	100.0%	88.1%
Mexico	Gran Morelos	2000	92.3%	99.1%	74.6%
Mexico	Gran Morelos	2017	98.8%	99.9%	93.1%
Mexico	Granados	2000	80.4%	100.0%	8.5%
Mexico	Granados	2017	95.3%	100.0%	61.4%
Mexico	Guachinango	2000	79.2%	97.2%	46.3%
Mexico	Guachinango	2017	94.9%	99.8%	73.8%
Mexico	Guachochi	2000	52.3%	57.6%	45.4%
Mexico	Guachochi	2017	87.5%	90.8%	83.2%
Mexico	Guadalajara	2000	87.4%	92.7%	78.2%
Mexico	Guadalajara	2017	97.6%	99.2%	94.4%
Mexico	Guadalcazar	2000	80.4%	91.8%	64.6%
Mexico	Guadalcazar	2017	96.1%	99.2%	89.7%
Mexico	Guadalupe	2000	98.4%	99.1%	97.6%
Mexico	Guadalupe	2000	92.8%	98.8%	78.9%
Mexico	Guadalupe	2000	66.4%	93.8%	22.1%
Mexico	Guadalupe	2000	96.2%	98.9%	91.1%
Mexico	Guadalupe	2017	93.9%	99.5%	72.7%
Mexico	Guadalupe	2017	99.9%	99.9%	99.8%
Mexico	Guadalupe	2017	99.1%	99.9%	95.7%
Mexico	Guadalupe	2017	99.5%	99.9%	98.0%
Mexico	Guadalupe De Ramirez	2000	67.0%	99.6%	8.9%
Mexico	Guadalupe De Ramirez	2017	89.3%	100.0%	45.5%
Mexico	Guadalupe Etla	2000	55.9%	64.0%	47.8%
Mexico	Guadalupe Etla	2017	95.6%	96.8%	92.6%
Mexico	Guadalupe Victoria	2000	69.2%	80.2%	55.0%
Mexico	Guadalupe Victoria	2000	82.8%	94.5%	65.0%
Mexico	Guadalupe Victoria	2017	95.3%	99.0%	87.1%
Mexico	Guadalupe Victoria	2017	94.5%	97.3%	89.1%
Mexico	Guadalupe Y Calvo	2000	50.0%	57.7%	42.6%
Mexico	Guadalupe Y Calvo	2017	84.9%	89.0%	80.6%
Mexico	Guanacevi	2000	75.7%	91.5%	52.4%
Mexico	Guanacevi	2017	94.3%	98.8%	83.0%
Mexico	Guanajuato	2000	71.7%	83.1%	58.4%
Mexico	Guanajuato	2017	91.2%	97.9%	83.7%
Mexico	Guasave	2000	80.2%	89.3%	68.0%
Mexico	Guasave	2017	95.2%	98.0%	89.8%
Mexico	Guaymas	2000	93.9%	97.4%	86.3%
Mexico	Guaymas	2017	99.4%	99.8%	98.7%
Mexico	Guazapares	2000	82.8%	93.6%	67.3%
Mexico	Guazapares	2017	96.7%	99.3%	91.1%
Mexico	Guelatao De Juarez	2000	75.4%	99.8%	4.4%

Country	Second Administrative Unit	Year	Mean	Upper	Lower
Mexico	Guelatao De Juarez	2017	94.3%	100.0%	42.3%
Mexico	Guemez	2000	89.2%	97.9%	70.6%
Mexico	Guemez	2017	98.2%	99.8%	93.7%
Mexico	Guerrero	2000	80.7%	98.2%	45.5%
Mexico	Guerrero	2000	84.8%	92.9%	73.1%
Mexico	Guerrero	2000	84.6%	97.6%	54.9%
Mexico	Guerrero	2017	95.6%	99.8%	72.9%
Mexico	Guerrero	2017	97.5%	99.8%	88.9%
Mexico	Guerrero	2017	97.8%	99.3%	95.0%
Mexico	Guevea De Humboldt	2000	67.0%	93.3%	28.2%
Mexico	Guevea De Humboldt	2017	92.0%	99.6%	58.3%
Mexico	Gustavo A. Madero	2000	98.9%	99.2%	98.6%
Mexico	Gustavo A. Madero	2017	99.9%	100.0%	99.9%
Mexico	Gustavo Diaz Ordaz	2000	81.0%	99.5%	27.9%
Mexico	Gustavo Diaz Ordaz	2017	96.1%	100.0%	73.3%
Mexico	Gutierrez Zamora	2000	56.5%	69.0%	42.3%
Mexico	Gutierrez Zamora	2017	93.5%	97.9%	85.2%
Mexico	Halacho	2000	33.2%	72.0%	10.3%
Mexico	Halacho	2017	71.3%	97.3%	33.6%
Mexico	Hecelchakan	2000	77.8%	93.4%	49.0%
Mexico	Hecelchakan	2017	96.1%	99.5%	80.9%
Mexico	Hermenegildo Galeana	2000	57.4%	90.5%	13.4%
Mexico	Hermenegildo Galeana	2017	90.8%	99.2%	59.1%
Mexico	Hermosillo	2000	98.0%	98.9%	97.1%
Mexico	Hermosillo	2017	99.7%	99.9%	99.2%
Mexico	Heroica Ciudad De Ejutla De Crespo	2000	64.1%	87.3%	25.4%
Mexico	Heroica Ciudad De Ejutla De Crespo	2017	92.4%	98.8%	68.4%
Mexico	Heroica Ciudad De Huajuapan De Leon	2000	66.7%	90.2%	30.2%
Mexico	Heroica Ciudad De Huajuapan De Leon	2017	95.6%	99.2%	83.4%
Mexico	Heroica Ciudad De Tlaxiaco	2000	60.9%	73.8%	45.9%
Mexico	Heroica Ciudad De Tlaxiaco	2017	95.1%	97.4%	90.8%
Mexico	Hidalgo	2000	64.0%	75.7%	51.1%
Mexico	Hidalgo	2000	82.2%	99.5%	18.1%
Mexico	Hidalgo	2000	81.9%	95.8%	60.8%
Mexico	Hidalgo	2000	97.4%	99.9%	87.2%
Mexico	Hidalgo	2000	85.9%	97.4%	56.3%
Mexico	Hidalgo	2017	93.6%	97.5%	87.8%

Country	Second Administrative Unit	Year	Mean	Upper	Lower
Mexico	Hidalgo	2017	95.9%	99.7%	86.5%
Mexico	Hidalgo	2017	96.3%	100.0%	70.4%
Mexico	Hidalgo	2017	97.6%	99.7%	88.8%
Mexico	Hidalgo	2017	99.8%	100.0%	99.1%
Mexico	Hidalgo Del Parral	2000	92.9%	96.9%	86.7%
Mexico	Hidalgo Del Parral	2017	99.6%	99.8%	99.0%
Mexico	Hidalgotitlan	2000	66.0%	90.5%	39.8%
Mexico	Hidalgotitlan	2017	88.7%	98.7%	63.5%
Mexico	Higueras	2000	80.9%	99.7%	13.0%
Mexico	Higueras	2017	96.4%	100.0%	67.3%
Mexico	Hocaba	2000	85.8%	99.9%	34.7%
Mexico	Hocaba	2017	97.7%	100.0%	82.7%
Mexico	Hoctun	2000	96.5%	99.9%	85.7%
Mexico	Hoctun	2017	99.6%	100.0%	97.9%
Mexico	Homun	2000	92.9%	98.7%	77.5%
Mexico	Homun	2017	99.3%	99.9%	97.1%
Mexico	Honey	2000	76.4%	99.7%	16.4%
Mexico	Honey	2017	94.5%	100.0%	57.9%
Mexico	Hopelchen	2000	81.3%	89.1%	72.5%
Mexico	Hopelchen	2017	95.9%	98.7%	91.0%
Mexico	Hostotipaquillo	2000	79.2%	98.4%	31.6%
Mexico	Hostotipaquillo	2017	95.2%	99.9%	71.4%
Mexico	Huachinera	2000	79.3%	98.9%	28.8%
Mexico	Huachinera	2017	94.7%	99.9%	66.5%
Mexico	Huajicori	2000	80.7%	95.2%	57.9%
Mexico	Huajicori	2017	96.3%	99.3%	89.3%
Mexico	Hualahuises	2000	87.0%	99.8%	33.9%
Mexico	Hualahuises	2017	98.1%	100.0%	82.8%
Mexico	Huamantla	2000	96.6%	98.5%	93.0%
Mexico	Huamantla	2017	99.6%	99.9%	99.0%
Mexico	Huamuxtitlan	2000	60.4%	79.5%	36.6%
Mexico	Huamuxtitlan	2017	93.3%	98.1%	79.9%
Mexico	Huandacareo	2000	97.3%	99.8%	88.8%
Mexico	Huandacareo	2017	99.8%	100.0%	99.1%
Mexico	Huanimaro	2000	94.5%	99.8%	77.1%
Mexico	Huanimaro	2017	99.4%	100.0%	96.1%
Mexico	Huaniqueo	2000	82.5%	99.7%	38.6%
Mexico	Huaniqueo	2017	96.7%	100.0%	83.3%
Mexico	Huanusco	2000	80.4%	99.2%	38.4%
Mexico	Huanusco	2017	95.7%	99.9%	76.3%
Mexico	Huaquechula	2000	77.9%	98.7%	31.5%
Mexico	Huaquechula	2017	95.0%	99.9%	71.1%
Mexico	Huasabas	2000	84.3%	99.9%	20.2%
Mexico	Huasabas	2017	97.1%	100.0%	79.9%
Mexico	Huasca De Ocampo	2000	80.1%	99.0%	25.4%
Mexico	Huasca De Ocampo	2017	94.1%	99.9%	60.3%
Mexico	Huatabampo	2000	91.8%	97.0%	82.5%
Mexico	Huatabampo	2017	98.9%	99.7%	96.9%
Mexico	Huatlatlauca	2000	81.1%	99.6%	26.0%
Mexico	Huatlatlauca	2017	96.4%	100.0%	79.7%
Mexico	Huatusco	2000	86.5%	91.8%	78.0%
Mexico	Huatusco	2017	98.8%	99.3%	98.0%
Mexico	Huauchinango	2000	88.5%	95.0%	75.0%
Mexico	Huauchinango	2017	98.7%	99.6%	96.3%
Mexico	Huautepec	2000	54.7%	85.7%	23.2%
Mexico	Huautepec	2017	91.3%	98.9%	62.1%
Mexico	Huautla	2000	77.0%	97.7%	31.1%
Mexico	Huautla	2017	95.3%	99.9%	76.3%

Country	Second Administrative Unit	Year	Mean	Upper	Lower
Mexico	Huautla De Jimenez	2000	45.1%	52.5%	38.8%
Mexico	Huautla De Jimenez	2017	90.4%	94.3%	83.4%
Mexico	Huayacocotla	2000	77.5%	97.7%	40.6%
Mexico	Huayacocotla	2017	94.4%	99.8%	76.5%
Mexico	Huazalingo	2000	76.2%	98.9%	25.5%
Mexico	Huazalingo	2017	94.5%	99.9%	67.2%
Mexico	Huehuetan	2000	72.0%	98.8%	20.8%
Mexico	Huehuetan	2017	92.8%	99.9%	58.7%
Mexico	Huehuetla	2000	72.0%	92.8%	35.7%
Mexico	Huehuetla	2000	30.0%	33.9%	26.7%
Mexico	Huehuetla	2017	74.3%	78.9%	69.9%
Mexico	Huehuetla	2017	92.5%	99.6%	61.9%
Mexico	Huehuetlan	2000	72.1%	91.2%	25.7%
Mexico	Huehuetlan	2017	94.3%	99.2%	66.5%
Mexico	Huehuetlan El Chico	2000	80.4%	99.4%	29.0%
Mexico	Huehuetlan El Chico	2017	95.7%	100.0%	72.2%
Mexico	Huehuetlan El Grande	2000	78.1%	99.6%	22.1%
Mexico	Huehuetlan El Grande	2017	94.9%	100.0%	69.8%
Mexico	Huehuetoca	2000	89.3%	99.9%	48.2%
Mexico	Huehuetoca	2017	98.4%	100.0%	84.7%
Mexico	Huejotitan	2000	83.7%	98.9%	52.8%
Mexico	Huejotitan	2017	97.1%	99.9%	85.7%
Mexico	Huejotzingo	2000	70.7%	74.4%	67.2%
Mexico	Huejotzingo	2017	97.3%	97.8%	96.8%
Mexico	Huejucar	2000	80.0%	99.1%	26.2%
Mexico	Huejucar	2017	95.8%	99.9%	70.7%
Mexico	Huejuquilla El Alto	2000	79.6%	98.9%	24.5%
Mexico	Huejuquilla El Alto	2017	95.1%	99.9%	64.8%
Mexico	Huejutla De Reyes	2000	54.4%	69.6%	32.9%
Mexico	Huejutla De Reyes	2017	87.1%	92.7%	69.7%
Mexico	Huepac	2000	94.2%	99.9%	65.9%
Mexico	Huepac	2017	98.8%	100.0%	87.3%
Mexico	Huetamo	2000	58.4%	77.2%	36.9%
Mexico	Huetamo	2017	91.4%	96.7%	82.1%
Mexico	Hueyapan	2000	83.8%	95.7%	66.4%
Mexico	Hueyapan	2017	97.9%	99.7%	93.7%
Mexico	Hueyapan De Ocampo	2000	68.8%	93.9%	30.5%
Mexico	Hueyapan De Ocampo	2017	91.7%	99.4%	69.0%
Mexico	Hueyotlipan	2000	97.8%	99.4%	94.4%
Mexico	Hueyotlipan	2017	99.8%	100.0%	99.1%
Mexico	Hueypoxtla	2000	95.6%	99.9%	76.2%
Mexico	Hueypoxtla	2017	99.6%	100.0%	96.7%
Mexico	Hueytamalco	2000	69.0%	95.5%	26.6%
Mexico	Hueytamalco	2017	91.6%	99.7%	67.2%
Mexico	Hueytlalpan	2000	70.9%	77.4%	62.0%
Mexico	Hueytlalpan	2017	98.0%	98.6%	97.0%
Mexico	Huhi	2000	79.6%	99.8%	16.5%
Mexico	Huhi	2017	96.2%	100.0%	73.1%
Mexico	Huichapan	2000	82.5%	96.9%	57.0%
Mexico	Huichapan	2017	97.0%	99.7%	86.6%

Country	Second Administrative Unit	Year	Mean	Upper	Lower
Mexico	Huiloapan	2000	52.3%	68.8%	35.4%
Mexico	Huiloapan	2017	94.4%	96.8%	89.9%
Mexico	Huimanguillo	2000	54.5%	73.8%	34.7%
Mexico	Huimanguillo	2017	84.0%	93.2%	69.3%
Mexico	Huimilpan	2000	82.8%	96.4%	60.3%
Mexico	Huimilpan	2017	97.3%	99.8%	88.2%
Mexico	Huiramba	2000	69.2%	97.8%	9.1%
Mexico	Huiramba	2017	91.6%	99.7%	54.7%
Mexico	Huitiupan	2000	77.0%	90.2%	54.3%
Mexico	Huitiupan	2017	96.6%	99.1%	89.7%
Mexico	Huitzilac	2000	63.8%	79.0%	47.5%
Mexico	Huitzilac	2017	95.2%	98.2%	88.4%
Mexico	Huitzilan De Serdan	2000	76.8%	98.7%	27.2%
Mexico	Huitzilan De Serdan	2017	96.1%	99.9%	72.7%
Mexico	Huitziltepec	2000	67.6%	98.9%	10.7%
Mexico	Huitziltepec	2017	91.6%	99.9%	52.8%
Mexico	Huitzuco De Los Figueroa	2000	53.3%	83.4%	23.5%
Mexico	Huitzuco De Los Figueroa	2017	83.9%	97.0%	50.6%
Mexico	Huixquilucan	2000	91.4%	94.9%	85.5%
Mexico	Huixquilucan	2017	99.1%	99.7%	97.2%
Mexico	Huixtan	2000	73.1%	97.0%	25.9%
Mexico	Huixtan	2017	93.8%	99.8%	67.6%
Mexico	Huixtla	2000	56.9%	68.7%	43.1%
Mexico	Huixtla	2017	93.6%	96.3%	87.2%
Mexico	Hunucma	2000	89.8%	94.8%	82.6%
Mexico	Hunucma	2017	98.9%	99.6%	96.3%
Mexico	Ignacio De La Llave	2000	75.4%	99.0%	18.8%
Mexico	Ignacio De La Llave	2017	93.9%	99.9%	51.3%
Mexico	Ignacio Zaragoza	2000	86.1%	98.1%	53.4%
Mexico	Ignacio Zaragoza	2017	98.3%	99.8%	94.2%
Mexico	Iguala De La Independencia	2000	66.6%	80.2%	45.0%
Mexico	Iguala De La Independencia	2017	94.6%	97.5%	88.0%
Mexico	Igualapa	2000	61.8%	84.1%	31.7%
Mexico	Igualapa	2017	93.0%	98.1%	78.2%
Mexico	Ilamatlan	2000	68.5%	98.9%	17.9%
Mexico	Ilamatlan	2017	90.9%	99.9%	59.2%
Mexico	Iliatenco	2000	69.2%	98.5%	13.7%
Mexico	Iliatenco	2017	90.2%	99.8%	43.7%
Mexico	Imuris	2000	93.1%	99.6%	63.9%
Mexico	Imuris	2017	98.9%	100.0%	93.6%
Mexico	Indaparapeo	2000	71.7%	96.0%	29.6%
Mexico	Indaparapeo	2017	95.2%	99.6%	77.3%
Mexico	Inde	2000	80.4%	96.0%	55.7%
Mexico	Inde	2017	95.4%	99.6%	83.6%
Mexico	Irapuato	2000	89.7%	94.0%	82.2%
Mexico	Irapuato	2017	98.7%	99.6%	95.5%
Mexico	Irimbo	2000	71.5%	92.7%	37.1%
Mexico	Irimbo	2017	96.3%	99.6%	82.1%
Mexico	Isidro Fabela	2000	82.0%	96.8%	46.1%
Mexico	Isidro Fabela	2017	97.6%	99.8%	85.8%

Country	Second Administrative Unit	Year	Mean	Upper	Lower
Mexico	Isla	2000	69.4%	92.3%	23.1%
Mexico	Isla	2017	90.9%	98.8%	57.3%
Mexico	Isla Mujeres	2000	46.2%	95.3%	10.0%
Mexico	Isla Mujeres	2017	84.6%	99.4%	42.0%
Mexico	Iturbide	2000	56.5%	89.0%	25.1%
Mexico	Iturbide	2017	89.1%	99.0%	58.0%
Mexico	Ixcamilpa De Guerrero	2000	78.9%	99.4%	34.6%
Mexico	Ixcamilpa De Guerrero	2017	95.8%	100.0%	78.4%
Mexico	Ixcaquixtla	2000	77.3%	99.4%	23.8%
Mexico	Ixcaquixtla	2017	92.8%	100.0%	55.8%
Mexico	Ixcateopan De Cuauhtemoc	2000	81.2%	99.4%	33.6%
Mexico	Ixcateopan De Cuauhtemoc	2017	95.7%	100.0%	75.1%
Mexico	Ixcatepec	2000	91.7%	99.5%	73.2%
Mexico	Ixcatepec	2017	98.5%	100.0%	92.4%
Mexico	Ixhuacan De Los Reyes	2000	56.7%	90.0%	17.8%
Mexico	Ixhuacan De Los Reyes	2017	82.2%	99.1%	44.2%
Mexico	Ixhuatan	2000	52.1%	75.2%	27.6%
Mexico	Ixhuatan	2017	90.5%	96.8%	76.1%
Mexico	Ixhuatlan De Madero	2000	58.6%	79.9%	32.6%
Mexico	Ixhuatlan De Madero	2017	89.8%	97.6%	71.8%
Mexico	Ixhuatlan Del Cafe	2000	93.8%	98.9%	82.8%
Mexico	Ixhuatlan Del Cafe	2017	99.3%	99.9%	97.0%
Mexico	Ixhuatlan Del Sureste	2000	72.3%	94.7%	30.3%
Mexico	Ixhuatlan Del Sureste	2017	95.0%	99.3%	81.2%
Mexico	Ixhuatlancillo	2000	84.2%	88.7%	79.2%
Mexico	Ixhuatlancillo	2017	98.6%	99.2%	97.5%
Mexico	Ixil	2000	81.1%	99.6%	22.8%
Mexico	Ixil	2017	96.7%	100.0%	77.7%
Mexico	Ixmatlahuacan	2000	73.9%	98.3%	20.5%
Mexico	Ixmatlahuacan	2017	95.0%	99.8%	71.5%
Mexico	Ixmiquilpan	2000	79.2%	93.9%	56.6%
Mexico	Ixmiquilpan	2017	97.5%	99.6%	91.3%
Mexico	Ixpantepec Nieves	2000	73.0%	98.6%	17.1%
Mexico	Ixpantepec Nieves	2017	94.7%	99.9%	63.2%
Mexico	Ixtacamaxtitlan	2000	75.3%	93.6%	44.6%
Mexico	Ixtacamaxtitlan	2017	94.8%	99.5%	79.6%
Mexico	Ixtacomitan	2000	53.0%	97.5%	2.6%
Mexico	Ixtacomitan	2017	83.1%	99.8%	19.2%
Mexico	Ixtacuixtla De Mariano Matamoros	2000	75.9%	81.3%	68.6%
Mexico	Ixtacuixtla De Mariano Matamoros	2017	97.8%	98.5%	96.4%
Mexico	Ixtaczoquitlan	2000	76.5%	84.3%	62.2%
Mexico	Ixtaczoquitlan	2017	94.6%	96.9%	88.8%
Mexico	Ixtapa	2000	77.2%	97.8%	25.3%
Mexico	Ixtapa	2017	94.7%	99.8%	73.4%

Country	Second Administrative Unit	Year	Mean	Upper	Lower
Mexico	Ixtapaluca	2000	97.6%	99.5%	91.5%
Mexico	Ixtapaluca	2017	99.8%	100.0%	99.1%
Mexico	Ixtapan De La Sal	2000	75.4%	95.9%	36.4%
Mexico	Ixtapan De La Sal	2017	96.7%	99.7%	84.1%
Mexico	Ixtapan Del Oro	2000	79.3%	99.7%	21.5%
Mexico	Ixtapan Del Oro	2017	96.3%	100.0%	75.0%
Mexico	Ixtapangajoya	2000	69.1%	90.4%	46.5%
Mexico	Ixtapangajoya	2017	92.7%	99.2%	76.1%
Mexico	Ixtenco	2000	99.1%	99.9%	95.8%
Mexico	Ixtenco	2017	99.9%	100.0%	99.7%
Mexico	Ixtepec	2000	61.3%	66.9%	54.3%
Mexico	Ixtepec	2017	96.4%	97.1%	95.2%
Mexico	Ixtlahuaca	2000	74.9%	93.0%	52.7%
Mexico	Ixtlahuaca	2017	94.1%	99.5%	80.7%
Mexico	Ixtlahuacan	2000	82.7%	96.1%	66.6%
Mexico	Ixtlahuacan	2017	97.4%	99.7%	89.8%
Mexico	Ixtlahuacan De Los Membrillos	2000	80.6%	98.5%	38.3%
Mexico	Ixtlahuacan De Los Membrillos	2017	96.9%	99.9%	83.2%
Mexico	Ixtlahuacan Del Rio	2000	49.7%	71.5%	25.0%
Mexico	Ixtlahuacan Del Rio	2017	84.5%	94.3%	66.2%
Mexico	Ixtlan	2000	80.3%	94.1%	54.5%
Mexico	Ixtlan	2017	95.9%	99.3%	85.9%
Mexico	Ixtlan De Juarez	2000	68.1%	91.0%	40.4%
Mexico	Ixtlan De Juarez	2017	92.6%	99.2%	76.3%
Mexico	Ixtlan Del Rio	2000	89.2%	94.8%	78.0%
Mexico	Ixtlan Del Rio	2017	99.0%	99.6%	97.1%
Mexico	Izamal	2000	83.8%	94.0%	63.4%
Mexico	Izamal	2017	98.4%	99.6%	94.0%
Mexico	Iztacalco	2000	99.5%	99.7%	99.2%
Mexico	Iztacalco	2017	100.0%	100.0%	100.0%
Mexico	Iztapalapa	2000	99.5%	99.7%	99.3%
Mexico	Iztapalapa	2017	100.0%	100.0%	100.0%
Mexico	Izucar De Matamoros	2000	91.6%	96.2%	83.9%
Mexico	Izucar De Matamoros	2017	99.1%	99.8%	97.2%
Mexico	Jacala De Ledezma	2000	91.2%	99.5%	71.8%
Mexico	Jacala De Ledezma	2017	98.6%	100.0%	91.7%
Mexico	Jacona	2000	88.6%	93.1%	81.2%
Mexico	Jacona	2017	99.1%	99.5%	97.5%
Mexico	Jala	2000	90.4%	96.6%	71.8%
Mexico	Jala	2017	99.2%	99.8%	96.8%
Mexico	Jalacingo	2000	88.6%	99.1%	68.6%
Mexico	Jalacingo	2017	98.3%	100.0%	92.7%
Mexico	Jalapa	2000	54.5%	80.9%	24.0%
Mexico	Jalapa	2017	86.0%	97.8%	65.5%
Mexico	Jalcomulco	2000	77.7%	99.7%	11.4%
Mexico	Jalcomulco	2017	95.5%	100.0%	56.9%

Country	Second Administrative Unit	Year	Mean	Upper	Lower
Mexico	Jalostotitlan	2000	80.8%	99.4%	26.9%
Mexico	Jalostotitlan	2017	94.8%	100.0%	61.6%
Mexico	Jalpa	2000	90.6%	97.5%	78.2%
Mexico	Jalpa	2017	99.1%	99.9%	96.7%
Mexico	Jalpa De Mendez	2000	62.7%	77.8%	44.7%
Mexico	Jalpa De Mendez	2017	94.3%	98.0%	86.7%
Mexico	Jalpan	2000	72.0%	97.9%	25.5%
Mexico	Jalpan	2017	92.5%	99.9%	63.2%
Mexico	Jalpan De Serra	2000	80.1%	91.9%	63.8%
Mexico	Jalpan De Serra	2017	97.1%	99.5%	91.3%
Mexico	Jaltenco	2000	98.5%	99.2%	97.4%
Mexico	Jaltenco	2017	99.9%	100.0%	99.9%
Mexico	Jaltipan	2000	64.8%	97.1%	14.3%
Mexico	Jaltipan	2017	91.0%	99.8%	56.3%
Mexico	Jaltocan	2000	46.9%	63.0%	31.9%
Mexico	Jaltocan	2017	91.4%	95.3%	83.5%
Mexico	Jamapa	2000	57.8%	96.9%	12.7%
Mexico	Jamapa	2017	81.5%	98.6%	38.8%
Mexico	Jamay	2000	95.6%	99.5%	77.7%
Mexico	Jamay	2017	99.6%	100.0%	98.0%
Mexico	Janos	2000	85.8%	95.2%	69.5%
Mexico	Janos	2017	97.7%	99.6%	91.2%
Mexico	Jantetelco	2000	24.8%	41.8%	10.3%
Mexico	Jantetelco	2017	76.1%	86.7%	59.6%
Mexico	Jaral Del Progreso	2000	87.8%	99.9%	34.7%
Mexico	Jaral Del Progreso	2017	97.1%	100.0%	70.5%
Mexico	Jaumave	2000	85.7%	96.0%	66.7%
Mexico	Jaumave	2017	97.5%	99.7%	90.9%
Mexico	Jerecuaro	2000	72.7%	92.0%	45.8%
Mexico	Jerecuaro	2017	94.1%	99.2%	80.0%
Mexico	Jerez	2000	85.2%	91.1%	78.0%
Mexico	Jerez	2017	98.2%	99.4%	95.6%
Mexico	Jesus Carranza	2000	70.1%	91.3%	37.2%
Mexico	Jesus Carranza	2017	92.5%	99.1%	70.2%
Mexico	Jesus Maria	2000	91.5%	97.0%	82.5%
Mexico	Jesus Maria	2000	82.2%	98.7%	37.2%
Mexico	Jesus Maria	2017	96.4%	99.9%	78.6%
Mexico	Jesus Maria	2017	98.3%	99.8%	95.6%
Mexico	Jilotepec	2000	93.1%	98.2%	83.4%
Mexico	Jilotepec	2000	82.4%	96.6%	52.6%
Mexico	Jilotepec	2017	99.5%	99.9%	98.5%
Mexico	Jilotepec	2017	96.9%	99.8%	86.2%
Mexico	Jilotlan De Los Dolores	2000	78.3%	95.9%	51.9%
Mexico	Jilotlan De Los Dolores	2017	94.5%	99.6%	80.7%
Mexico	Jilotzingo	2000	67.9%	82.4%	47.7%
Mexico	Jilotzingo	2017	96.5%	98.9%	89.6%
Mexico	Jimenez	2000	87.6%	99.6%	44.0%
Mexico	Jimenez	2000	97.0%	98.9%	93.2%
Mexico	Jimenez	2000	93.3%	98.0%	82.6%
Mexico	Jimenez	2000	59.5%	73.3%	44.5%
Mexico	Jimenez	2017	97.6%	100.0%	81.6%
Mexico	Jimenez	2017	86.3%	95.1%	76.2%

Country	Second Administrative Unit	Year	Mean	Upper	Lower
Mexico	Jimenez	2017	99.0%	99.8%	96.4%
Mexico	Jimenez	2017	99.6%	99.9%	99.1%
Mexico	Jimenez Del Teul	2000	75.7%	97.2%	30.2%
Mexico	Jimenez Del Teul	2017	95.1%	99.7%	77.7%
Mexico	Jiquilpan	2000	88.9%	95.5%	76.1%
Mexico	Jiquilpan	2017	98.2%	99.5%	95.3%
Mexico	Jiquipilas	2000	74.1%	95.1%	44.7%
Mexico	Jiquipilas	2017	93.3%	99.6%	71.0%
Mexico	Jiquipilco	2000	81.2%	99.5%	32.6%
Mexico	Jiquipilco	2017	96.3%	100.0%	76.1%
Mexico	Jitotol	2000	54.8%	88.0%	16.7%
Mexico	Jitotol	2017	87.6%	98.6%	57.2%
Mexico	Jiutepec	2000	90.3%	93.5%	84.1%
Mexico	Jiutepec	2017	99.3%	99.6%	98.8%
Mexico	Jocotepec	2000	86.9%	99.1%	50.2%
Mexico	Jocotepec	2017	97.4%	99.9%	85.8%
Mexico	Jocotitlan	2000	63.6%	84.6%	35.4%
Mexico	Jocotitlan	2017	88.0%	98.1%	71.9%
Mexico	Jojutla	2000	98.3%	99.4%	94.6%
Mexico	Jojutla	2017	99.9%	100.0%	99.6%
Mexico	Jolalpan	2000	77.9%	99.1%	27.4%
Mexico	Jolalpan	2017	93.7%	99.9%	54.3%
Mexico	Jonacatepec	2000	49.5%	93.4%	5.8%
Mexico	Jonacatepec	2017	83.8%	99.3%	30.0%
Mexico	Jonotla	2000	67.7%	87.0%	41.8%
Mexico	Jonotla	2017	92.9%	99.1%	68.3%
Mexico	Jonuta	2000	54.9%	72.5%	36.5%
Mexico	Jonuta	2017	83.1%	94.3%	69.2%
Mexico	Jopala	2000	63.4%	90.4%	20.2%
Mexico	Jopala	2017	91.9%	98.5%	71.8%
Mexico	Joquicingo	2000	71.5%	87.4%	44.1%
Mexico	Joquicingo	2017	96.0%	99.2%	79.9%
Mexico	Jose Azueta	2000	77.4%	95.6%	35.1%
Mexico	Jose Azueta	2000	58.9%	80.1%	27.5%
Mexico	Jose Azueta	2017	95.9%	99.6%	77.1%
Mexico	Jose Azueta	2017	85.3%	94.8%	72.8%
Mexico	Jose Joaquin De Herrera	2000	70.2%	99.1%	12.9%
Mexico	Jose Joaquin De Herrera	2017	91.5%	99.9%	53.3%
Mexico	Jose Maria Morelos	2000	78.5%	89.8%	61.8%
Mexico	Jose Maria Morelos	2017	94.3%	98.7%	86.2%
Mexico	Jose Sixto Verduzco	2000	85.4%	99.4%	44.9%
Mexico	Jose Sixto Verduzco	2017	97.5%	100.0%	84.7%
Mexico	Juan Aldama	2000	84.5%	93.8%	66.3%
Mexico	Juan Aldama	2017	98.2%	99.7%	93.0%
Mexico	Juan C. Bonilla	2000	57.9%	67.3%	45.7%
Mexico	Juan C. Bonilla	2017	96.4%	97.6%	94.2%
Mexico	Juan Galindo	2000	61.1%	68.5%	54.3%
Mexico	Juan Galindo	2017	95.3%	97.0%	92.9%
Mexico	Juan N. Mendez	2000	76.7%	99.0%	26.0%
Mexico	Juan N. Mendez	2017	93.7%	100.0%	67.1%

Country	Second Administrative Unit	Year	Mean	Upper	Lower
Mexico	Juan R. Escudero	2000	86.2%	94.6%	72.1%
Mexico	Juan R. Escudero	2017	96.9%	99.7%	86.1%
Mexico	Juan Rodriguez Clara	2000	72.1%	91.6%	44.5%
Mexico	Juan Rodriguez Clara	2017	94.4%	99.3%	79.9%
Mexico	Juanacatlan	2000	77.7%	90.2%	59.8%
Mexico	Juanacatlan	2017	96.6%	99.6%	81.7%
Mexico	Juarez	2000	84.4%	98.8%	44.4%
Mexico	Juarez	2000	79.8%	87.2%	72.6%
Mexico	Juarez	2000	78.2%	99.8%	15.6%
Mexico	Juarez	2000	84.0%	89.0%	78.9%
Mexico	Juarez	2000	58.9%	89.8%	24.1%
Mexico	Juarez	2017	96.5%	98.7%	93.3%
Mexico	Juarez	2017	94.2%	100.0%	59.9%
Mexico	Juarez	2017	94.2%	98.2%	87.8%
Mexico	Juarez	2017	87.3%	98.8%	53.3%
Mexico	Juarez	2017	97.3%	99.9%	87.1%
Mexico	Juarez Hidalgo	2000	86.4%	94.8%	71.8%
Mexico	Juarez Hidalgo	2017	98.4%	99.7%	93.8%
Mexico	Juchipila	2000	97.1%	99.5%	89.6%
Mexico	Juchipila	2017	99.8%	100.0%	99.1%
Mexico	Juchique De Ferrer	2000	74.5%	99.4%	18.2%
Mexico	Juchique De Ferrer	2017	93.5%	100.0%	65.0%
Mexico	Juchitan	2000	74.7%	99.0%	13.8%
Mexico	Juchitan	2017	93.5%	99.9%	56.5%
Mexico	Juchitan De Zaragoza	2000	81.9%	92.1%	61.3%
Mexico	Juchitan De Zaragoza	2017	98.0%	99.3%	94.9%
Mexico	Juchitepec	2000	96.7%	99.8%	84.9%
Mexico	Juchitepec	2017	99.6%	100.0%	97.0%
Mexico	Juchitlan	2000	75.1%	99.7%	18.4%
Mexico	Juchitlan	2017	94.1%	100.0%	68.2%
Mexico	Julimes	2000	84.4%	99.3%	43.3%
Mexico	Julimes	2017	97.6%	99.9%	82.3%
Mexico	Jungapeo	2000	49.8%	70.8%	27.9%
Mexico	Jungapeo	2017	89.4%	97.2%	72.1%
Mexico	Kanasin	2000	89.3%	92.6%	85.3%
Mexico	Kanasin	2017	99.3%	99.6%	99.0%
Mexico	Kantunil	2000	91.6%	99.7%	66.9%
Mexico	Kantunil	2017	97.7%	100.0%	79.0%
Mexico	Kaua	2000	77.9%	99.6%	17.0%
Mexico	Kaua	2017	95.0%	100.0%	63.3%
Mexico	Kinchil	2000	80.2%	99.7%	19.1%
Mexico	Kinchil	2017	96.3%	100.0%	75.9%
Mexico	Kopoma	2000	78.6%	99.9%	17.5%
Mexico	Kopoma	2017	95.0%	100.0%	68.5%
Mexico	La Antigua	2000	82.3%	99.8%	36.0%
Mexico	La Antigua	2017	96.5%	100.0%	73.8%
Mexico	La Barca	2000	91.7%	97.7%	79.5%
Mexico	La Barca	2017	98.8%	99.8%	94.7%
Mexico	La Colorada	2000	80.0%	96.6%	56.3%
Mexico	La Colorada	2017	95.3%	99.7%	81.3%
Mexico	La Compania	2000	75.5%	99.4%	16.3%
Mexico	La Compania	2017	95.2%	100.0%	62.3%

Country	Second Administrative Unit	Year	Mean	Upper	Lower
Mexico	La Concordia	2000	79.5%	92.0%	62.0%
Mexico	La Concordia	2017	95.4%	98.6%	87.0%
Mexico	La Cruz	2000	81.2%	99.8%	29.1%
Mexico	La Cruz	2017	96.0%	100.0%	74.7%
Mexico	La Grandeza	2000	57.9%	98.4%	6.5%
Mexico	La Grandeza	2017	86.1%	99.9%	37.8%
Mexico	La Huacana	2000	79.2%	95.9%	52.5%
Mexico	La Huacana	2017	95.3%	99.6%	84.5%
Mexico	La Huerta	2000	79.8%	95.8%	53.2%
Mexico	La Huerta	2017	95.9%	99.6%	88.2%
Mexico	La Independencia	2000	71.7%	90.7%	45.0%
Mexico	La Independencia	2017	94.5%	99.1%	79.4%
Mexico	La Libertad	2000	77.4%	94.8%	50.0%
Mexico	La Libertad	2017	93.6%	99.5%	78.7%
Mexico	La Magdalena Contreras	2000	80.7%	85.4%	74.8%
Mexico	La Magdalena Contreras	2017	98.5%	99.0%	97.9%
Mexico	La Magdalena Tlaltelulco	2000	99.3%	99.6%	98.7%
Mexico	La Magdalena Tlaltelulco	2017	100.0%	100.0%	99.9%
Mexico	La Magdalena Tlatlauquitepec	2000	77.7%	99.9%	10.9%
Mexico	La Magdalena Tlatlauquitepec	2017	93.8%	100.0%	50.9%
Mexico	La Manzanilla De La Paz	2000	82.4%	99.6%	27.6%
Mexico	La Manzanilla De La Paz	2017	94.9%	100.0%	50.6%
Mexico	La Mision	2000	84.3%	99.1%	42.1%
Mexico	La Mision	2017	96.9%	99.9%	83.1%
Mexico	La Paz	2000	96.1%	97.3%	94.6%
Mexico	La Paz	2000	93.7%	97.0%	88.3%
Mexico	La Paz	2017	99.7%	99.8%	99.6%
Mexico	La Paz	2017	99.3%	99.8%	98.3%
Mexico	La Pe	2000	71.9%	99.4%	12.9%
Mexico	La Pe	2017	93.1%	100.0%	56.1%
Mexico	La Perla	2000	50.5%	83.5%	18.5%
Mexico	La Perla	2017	76.6%	96.1%	39.4%
Mexico	La Piedad	2000	87.7%	96.7%	67.4%
Mexico	La Piedad	2017	98.5%	99.7%	94.8%
Mexico	La Reforma	2000	68.7%	97.8%	16.3%
Mexico	La Reforma	2017	91.0%	99.8%	48.3%
Mexico	La Trinidad Vista Hermosa	2000	73.9%	99.9%	4.1%
Mexico	La Trinidad Vista Hermosa	2017	92.2%	100.0%	30.3%
Mexico	La Trinitaria	2000	74.6%	91.3%	48.5%
Mexico	La Trinitaria	2017	94.5%	99.4%	81.4%
Mexico	La Union De Isidoro Montes De Oca	2000	76.2%	92.7%	50.4%

Country	Second Administrative Unit	Year	Mean	Upper	Lower
Mexico	La Union De Isidoro Montes De Oca	2017	95.0%	99.2%	79.9%
Mexico	La Yesca	2000	76.5%	91.5%	55.0%
Mexico	La Yesca	2017	93.9%	98.8%	81.1%
Mexico	Lafragua	2000	58.8%	67.6%	39.2%
Mexico	Lafragua	2017	70.8%	91.7%	62.6%
Mexico	Lagos De Moreno	2000	91.9%	96.6%	85.0%
Mexico	Lagos De Moreno	2017	98.8%	99.7%	96.8%
Mexico	Lagunillas	2000	78.1%	98.5%	20.7%
Mexico	Lagunillas	2000	78.5%	97.7%	43.0%
Mexico	Lagunillas	2017	96.0%	99.9%	74.5%
Mexico	Lagunillas	2017	95.4%	99.8%	80.3%
Mexico	Lamadrid	2000	88.4%	99.8%	41.6%
Mexico	Lamadrid	2017	98.4%	100.0%	90.9%
Mexico	Lampazos De Naranjo	2000	86.3%	99.5%	36.8%
Mexico	Lampazos De Naranjo	2017	98.0%	99.9%	88.6%
Mexico	Landa De Matamoros	2000	80.0%	98.4%	53.1%
Mexico	Landa De Matamoros	2017	95.4%	99.9%	84.2%
Mexico	Landero Y Coss	2000	73.7%	99.7%	6.4%
Mexico	Landero Y Coss	2017	94.3%	100.0%	43.5%
Mexico	Larrainzar	2000	70.9%	97.5%	20.0%
Mexico	Larrainzar	2017	91.4%	99.8%	54.7%
Mexico	Las Choapas	2000	66.8%	88.7%	26.0%
Mexico	Las Choapas	2017	90.0%	98.1%	52.7%
Mexico	Las Margaritas	2000	75.9%	89.6%	53.7%
Mexico	Las Margaritas	2017	94.4%	98.6%	84.1%
Mexico	Las Minas	2000	61.3%	94.0%	20.2%
Mexico	Las Minas	2017	89.4%	99.6%	49.9%
Mexico	Las Rosas	2000	75.7%	98.7%	13.3%
Mexico	Las Rosas	2017	94.6%	99.9%	60.4%
Mexico	Las Vigas De Ramirez	2000	75.0%	99.6%	12.6%
Mexico	Las Vigas De Ramirez	2017	94.7%	100.0%	58.8%
Mexico	Lazaro Cardenas	2000	88.8%	97.0%	71.3%
Mexico	Lazaro Cardenas	2000	75.4%	99.7%	6.5%
Mexico	Lazaro Cardenas	2000	84.8%	96.1%	64.9%
Mexico	Lazaro Cardenas	2017	97.0%	99.6%	86.6%
Mexico	Lazaro Cardenas	2017	98.6%	99.7%	94.7%
Mexico	Lazaro Cardenas	2017	94.4%	100.0%	51.5%
Mexico	Leon	2000	74.8%	80.5%	67.7%
Mexico	Leon	2017	89.6%	92.3%	86.3%
Mexico	Leonardo Bravo	2000	59.2%	84.4%	16.4%

Country	Second Administrative Unit	Year	Mean	Upper	Lower
Mexico	Leonardo Bravo	2017	82.9%	95.5%	54.4%
Mexico	Lerdo	2000	93.7%	95.8%	91.3%
Mexico	Lerdo	2017	99.5%	99.7%	98.9%
Mexico	Lerdo De Tejada	2000	73.4%	99.6%	8.8%
Mexico	Lerdo De Tejada	2017	92.9%	100.0%	36.2%
Mexico	Lerma	2000	67.6%	77.8%	52.5%
Mexico	Lerma	2017	93.4%	96.5%	88.7%
Mexico	Libres	2000	93.0%	99.3%	78.2%
Mexico	Libres	2017	98.9%	100.0%	94.7%
Mexico	Linares	2000	91.4%	96.3%	82.9%
Mexico	Linares	2017	98.8%	99.6%	97.2%
Mexico	Llera	2000	72.1%	83.9%	54.6%
Mexico	Llera	2017	95.1%	98.1%	87.1%
Mexico	Lolotla	2000	88.3%	99.7%	58.9%
Mexico	Lolotla	2017	97.9%	100.0%	87.7%
Mexico	Loma Bonita	2000	60.1%	86.8%	29.7%
Mexico	Loma Bonita	2017	92.8%	98.6%	79.3%
Mexico	Lopez	2000	84.1%	99.6%	31.4%
Mexico	Lopez	2017	97.2%	100.0%	83.3%
Mexico	Loreto	2000	84.4%	95.3%	64.1%
Mexico	Loreto	2000	93.0%	97.6%	85.5%
Mexico	Loreto	2017	98.3%	99.5%	96.1%
Mexico	Loreto	2017	99.1%	99.8%	96.5%
Mexico	Los Aldamas	2000	85.4%	99.5%	50.1%
Mexico	Los Aldamas	2017	96.0%	100.0%	70.1%
Mexico	Los Cabos	2000	95.2%	97.6%	89.7%
Mexico	Los Cabos	2017	99.6%	99.8%	98.5%
Mexico	Los Herreras	2000	96.2%	99.6%	86.1%
Mexico	Los Herreras	2017	99.4%	100.0%	95.9%
Mexico	Los Ramones	2000	81.8%	95.6%	59.1%
Mexico	Los Ramones	2017	96.2%	99.7%	87.2%
Mexico	Los Reyes	2000	70.9%	96.3%	20.8%
Mexico	Los Reyes	2000	69.0%	89.7%	45.8%
Mexico	Los Reyes	2017	91.5%	99.8%	39.2%
Mexico	Los Reyes	2017	94.0%	99.3%	77.8%
Mexico	Los Reyes De Juarez	2000	90.9%	97.1%	70.6%
Mexico	Los Reyes De Juarez	2017	99.2%	99.7%	97.6%
Mexico	Luis Moya	2000	94.6%	97.2%	88.6%
Mexico	Luis Moya	2017	99.6%	99.8%	98.6%
Mexico	Luvianos	2000	70.7%	97.7%	23.6%
Mexico	Luvianos	2017	92.5%	99.8%	59.4%
Mexico	Macuspana	2000	42.9%	58.6%	29.1%
Mexico	Macuspana	2017	82.2%	90.4%	70.0%
Mexico	Madera	2000	93.1%	97.0%	85.1%
Mexico	Madera	2017	98.9%	99.6%	97.7%
Mexico	Madero	2000	79.4%	96.6%	39.3%
Mexico	Madero	2017	95.3%	99.7%	77.1%
Mexico	Magdalena	2000	85.6%	99.7%	29.6%
Mexico	Magdalena	2000	65.3%	99.8%	2.5%
Mexico	Magdalena	2000	97.3%	99.7%	88.8%
Mexico	Magdalena	2017	88.8%	100.0%	29.0%
Mexico	Magdalena	2017	97.4%	100.0%	81.0%
Mexico	Magdalena	2017	99.8%	100.0%	99.2%
Mexico	Magdalena Apasco	2000	48.3%	73.2%	22.6%
Mexico	Magdalena Apasco	2017	91.2%	97.8%	74.6%

Country	Second Administrative Unit	Year	Mean	Upper	Lower
Mexico	Magdalena Jaltepec	2000	73.6%	98.7%	26.1%
Mexico	Magdalena Jaltepec	2017	92.8%	99.9%	66.9%
Mexico	Magdalena Mixtepec	2000	31.8%	82.2%	1.4%
Mexico	Magdalena Mixtepec	2017	67.9%	97.9%	12.9%
Mexico	Magdalena Ocotlan	2000	28.5%	74.1%	1.5%
Mexico	Magdalena Ocotlan	2017	65.6%	97.6%	17.7%
Mexico	Magdalena Penasco	2000	73.5%	98.1%	21.4%
Mexico	Magdalena Penasco	2017	95.8%	99.7%	79.3%
Mexico	Magdalena Teitipac	2000	80.7%	99.8%	17.4%
Mexico	Magdalena Teitipac	2017	96.7%	100.0%	75.2%
Mexico	Magdalena Tequisistlan	2000	76.4%	98.4%	15.0%
Mexico	Magdalena Tequisistlan	2017	93.5%	99.8%	48.8%
Mexico	Magdalena Tlacotepec	2000	78.1%	99.9%	8.5%
Mexico	Magdalena Tlacotepec	2017	95.4%	100.0%	55.4%
Mexico	Magdalena Yodocono De Porfirio Diaz	2000	71.4%	99.6%	12.1%
Mexico	Magdalena Yodocono De Porfirio Diaz	2017	93.2%	100.0%	61.2%
Mexico	Magdalena Zahuatlan	2000	66.3%	96.8%	20.0%
Mexico	Magdalena Zahuatlan	2017	90.4%	99.9%	36.0%
Mexico	Maguarichi	2000	81.9%	96.8%	45.6%
Mexico	Maguarichi	2017	96.4%	99.7%	81.9%
Mexico	Mainero	2000	79.2%	99.1%	24.6%
Mexico	Mainero	2017	94.9%	99.9%	67.1%
Mexico	Malinalco	2000	73.4%	97.6%	22.5%
Mexico	Malinalco	2017	93.3%	99.8%	67.7%
Mexico	Malinaltepec	2000	47.6%	73.5%	14.4%
Mexico	Malinaltepec	2017	74.8%	88.6%	49.7%
Mexico	Maltrata	2000	77.4%	98.2%	22.3%
Mexico	Maltrata	2017	95.6%	99.9%	69.9%
Mexico	Mama	2000	92.4%	99.9%	61.6%
Mexico	Mama	2017	99.2%	100.0%	94.3%
Mexico	Mani	2000	79.4%	98.9%	26.2%
Mexico	Mani	2017	95.7%	99.9%	64.8%
Mexico	Manlio Fabio Altamirano	2000	71.5%	91.3%	40.1%
Mexico	Manlio Fabio Altamirano	2017	95.0%	99.3%	83.8%
Mexico	Manuel Benavides	2000	90.6%	97.0%	80.9%
Mexico	Manuel Benavides	2017	98.1%	99.7%	92.4%
Mexico	Manuel Doblado	2000	90.8%	98.8%	76.8%

Country	Second Administrative Unit	Year	Mean	Upper	Lower
Mexico	Manuel Doblado	2017	98.2%	99.9%	92.3%
Mexico	Manzanillo	2000	88.6%	97.8%	64.6%
Mexico	Manzanillo	2017	98.3%	99.8%	91.5%
Mexico	Mapastepec	2000	48.6%	63.3%	32.0%
Mexico	Mapastepec	2017	90.0%	95.6%	80.3%
Mexico	Mapimi	2000	84.7%	94.2%	65.8%
Mexico	Mapimi	2017	98.3%	99.5%	95.6%
Mexico	Maravatio	2000	75.5%	89.6%	53.9%
Mexico	Maravatio	2017	96.4%	99.2%	88.4%
Mexico	Maravilla Tenejapa	2000	69.6%	97.5%	24.6%
Mexico	Maravilla Tenejapa	2017	92.0%	99.7%	67.4%
Mexico	Marcos Castellanos	2000	81.5%	98.8%	37.9%
Mexico	Marcos Castellanos	2017	96.6%	99.9%	80.4%
Mexico	Mariano Escobedo	2000	68.9%	86.8%	43.7%
Mexico	Mariano Escobedo	2017	95.4%	98.6%	86.1%
Mexico	Marin	2000	84.9%	99.0%	42.0%
Mexico	Marin	2017	96.7%	99.9%	74.5%
Mexico	Mariscala De Juarez	2000	66.9%	94.0%	16.9%
Mexico	Mariscala De Juarez	2017	92.8%	99.6%	58.9%
Mexico	Marquelia	2000	80.2%	98.8%	28.1%
Mexico	Marquelia	2017	94.4%	99.9%	68.8%
Mexico	Marques De Comillas	2000	69.4%	92.8%	36.3%
Mexico	Marques De Comillas	2017	92.6%	99.4%	78.3%
Mexico	Martinez De La Torre	2000	72.3%	88.0%	46.7%
Mexico	Martinez De La Torre	2017	96.7%	98.9%	90.7%
Mexico	Martir De Cuilapan	2000	76.5%	95.3%	38.7%
Mexico	Martir De Cuilapan	2017	94.3%	99.7%	62.1%
Mexico	Martires De Tacubaya	2000	74.1%	99.8%	12.2%
Mexico	Martires De Tacubaya	2017	93.4%	100.0%	53.2%
Mexico	Mascota	2000	80.5%	96.4%	50.7%
Mexico	Mascota	2017	96.4%	99.7%	85.2%
Mexico	Matachi	2000	81.7%	99.2%	33.1%
Mexico	Matachi	2017	96.8%	99.9%	81.4%
Mexico	Matamoros	2000	87.9%	95.6%	73.1%
Mexico	Matamoros	2000	83.1%	97.4%	48.7%
Mexico	Matamoros	2000	91.3%	96.3%	81.9%
Mexico	Matamoros	2017	98.2%	99.7%	91.3%
Mexico	Matamoros	2017	99.1%	99.7%	97.5%
Mexico	Matamoros	2017	96.0%	99.8%	82.5%
Mexico	Matehuala	2000	78.5%	91.2%	57.0%
Mexico	Matehuala	2017	97.6%	99.2%	94.5%
Mexico	Matias Romero Avendano	2000	72.7%	83.6%	58.1%

Country	Second Administrative Unit	Year	Mean	Upper	Lower
Mexico	Matias Romero Avendano	2017	95.3%	98.3%	87.9%
Mexico	Matlapa	2000	62.1%	75.6%	46.8%
Mexico	Matlapa	2017	94.2%	97.2%	87.4%
Mexico	Maxcanu	2000	86.9%	97.4%	63.7%
Mexico	Maxcanu	2017	97.9%	99.8%	87.8%
Mexico	Mayapan	2000	82.8%	100.0%	11.1%
Mexico	Mayapan	2017	96.3%	100.0%	64.8%
Mexico	Mazamitla	2000	85.3%	99.4%	30.6%
Mexico	Mazamitla	2017	97.4%	100.0%	82.3%
Mexico	Mazapa De Madero	2000	68.0%	99.3%	13.8%
Mexico	Mazapa De Madero	2017	89.7%	100.0%	41.2%
Mexico	Mazapil	2000	77.7%	86.7%	66.7%
Mexico	Mazapil	2017	95.1%	98.2%	88.7%
Mexico	Mazapiltepec De Juarez	2000	90.0%	97.5%	71.9%
Mexico	Mazapiltepec De Juarez	2017	99.2%	99.8%	97.3%
Mexico	Mazatan	2000	88.3%	99.7%	37.4%
Mexico	Mazatan	2000	74.9%	98.7%	26.1%
Mexico	Mazatan	2017	98.6%	100.0%	91.5%
Mexico	Mazatan	2017	94.2%	99.9%	70.7%
Mexico	Mazatecochco De Jose Maria Morelos	2000	99.0%	99.8%	96.6%
Mexico	Mazatecochco De Jose Maria Morelos	2017	99.9%	100.0%	99.8%
Mexico	Mazatepec	2000	63.1%	92.7%	26.0%
Mexico	Mazatepec	2017	93.8%	99.5%	76.3%
Mexico	Mazatlan	2000	95.9%	98.1%	91.4%
Mexico	Mazatlan	2017	99.5%	99.8%	98.6%
Mexico	Mazatlan Villa De Flores	2000	55.8%	75.5%	27.8%
Mexico	Mazatlan Villa De Flores	2017	92.6%	97.0%	80.0%
Mexico	Mecatlan	2000	28.9%	49.8%	12.5%
Mexico	Mecatlan	2017	77.5%	91.1%	55.9%
Mexico	Mecayapan	2000	71.5%	96.5%	18.9%
Mexico	Mecayapan	2017	92.3%	99.7%	63.3%
Mexico	Medellin	2000	45.8%	56.9%	34.0%
Mexico	Medellin	2017	69.0%	80.6%	53.6%
Mexico	Melchor Ocampo	2000	90.2%	94.9%	83.1%
Mexico	Melchor Ocampo	2000	77.6%	95.9%	44.0%
Mexico	Melchor Ocampo	2000	97.5%	99.6%	90.7%
Mexico	Melchor Ocampo	2017	99.3%	99.7%	98.6%
Mexico	Melchor Ocampo	2017	99.8%	100.0%	99.0%
Mexico	Melchor Ocampo	2017	94.6%	99.7%	77.6%
Mexico	Mendez	2000	81.1%	95.1%	54.5%
Mexico	Mendez	2017	95.5%	99.4%	82.7%
Mexico	Meoqui	2000	79.3%	92.0%	58.2%

Country	Second Administrative Unit	Year	Mean	Upper	Lower
Mexico	Meoqui	2017	97.9%	99.5%	94.4%
Mexico	Merida	2000	87.4%	91.3%	82.9%
Mexico	Merida	2017	99.0%	99.5%	98.3%
Mexico	Mesones Hidalgo	2000	67.0%	97.4%	19.5%
Mexico	Mesones Hidalgo	2017	92.9%	99.9%	64.5%
Mexico	Metapa	2000	95.3%	99.4%	84.0%
Mexico	Metapa	2017	99.6%	100.0%	98.1%
Mexico	Metepec	2000	93.1%	95.8%	87.6%
Mexico	Metepec	2000	82.2%	91.8%	63.9%
Mexico	Metepec	2017	98.1%	99.4%	92.9%
Mexico	Metepec	2017	99.5%	99.7%	99.1%
Mexico	Metlatonoc	2000	70.5%	89.6%	46.1%
Mexico	Metlatonoc	2017	92.7%	98.9%	79.3%
Mexico	Metztitlan	2000	77.6%	97.2%	39.3%
Mexico	Metztitlan	2017	94.8%	99.8%	71.3%
Mexico	Mexicali	2000	95.9%	97.7%	92.1%
Mexico	Mexicali	2017	99.6%	99.8%	99.2%
Mexico	Mexicaltzingo	2000	96.5%	99.5%	83.1%
Mexico	Mexicaltzingo	2017	99.7%	100.0%	98.7%
Mexico	Mexquitic De Carmona	2000	57.6%	75.0%	42.7%
Mexico	Mexquitic De Carmona	2017	89.7%	95.9%	75.1%
Mexico	Mexticacan	2000	75.9%	98.7%	20.6%
Mexico	Mexticacan	2017	94.9%	99.9%	68.5%
Mexico	Mezquital	2000	81.8%	89.7%	69.9%
Mexico	Mezquital	2017	96.2%	98.5%	91.1%
Mexico	Mezquital Del Oro	2000	80.2%	99.4%	20.6%
Mexico	Mezquital Del Oro	2017	95.0%	100.0%	61.2%
Mexico	Mezquitic	2000	56.5%	73.9%	36.3%
Mexico	Mezquitic	2017	82.6%	93.7%	67.8%
Mexico	Miacatlan	2000	70.0%	89.1%	48.6%
Mexico	Miacatlan	2017	95.6%	99.2%	87.7%
Mexico	Miahuatlan	2000	78.8%	99.8%	23.6%
Mexico	Miahuatlan	2017	95.2%	100.0%	63.5%
Mexico	Miahuatlan De Porfirio Diaz	2000	61.5%	72.1%	48.2%
Mexico	Miahuatlan De Porfirio Diaz	2017	93.8%	97.2%	81.6%
Mexico	Mier	2000	67.3%	97.7%	18.7%
Mexico	Mier	2017	94.2%	99.9%	70.5%
Mexico	Mier Y Noriega	2000	83.7%	96.9%	60.8%
Mexico	Mier Y Noriega	2017	96.9%	99.7%	88.9%
Mexico	Miguel Aleman	2000	82.4%	96.5%	57.0%
Mexico	Miguel Aleman	2017	97.8%	99.8%	93.2%
Mexico	Miguel Auza	2000	91.0%	96.8%	81.7%
Mexico	Miguel Auza	2017	98.4%	99.8%	93.4%
Mexico	Miguel Hidalgo	2000	98.5%	98.9%	98.0%
Mexico	Miguel Hidalgo	2017	99.9%	99.9%	99.9%
Mexico	Milpa Alta	2000	94.7%	99.6%	78.7%

Country	Second Administrative Unit	Year	Mean	Upper	Lower
Mexico	Milpa Alta	2017	99.5%	100.0%	97.5%
Mexico	Mina	2000	95.8%	99.2%	84.8%
Mexico	Mina	2017	99.2%	99.9%	97.1%
Mexico	Minatitlan	2000	70.4%	76.3%	63.7%
Mexico	Minatitlan	2000	95.7%	99.6%	87.5%
Mexico	Minatitlan	2017	99.3%	100.0%	96.6%
Mexico	Minatitlan	2017	94.9%	96.9%	91.5%
Mexico	Mineral De La Reforma	2000	91.3%	97.9%	79.5%
Mexico	Mineral De La Reforma	2017	99.2%	99.9%	95.8%
Mexico	Mineral Del Chico	2000	87.0%	99.5%	66.7%
Mexico	Mineral Del Chico	2017	96.0%	100.0%	82.5%
Mexico	Mineral Del Monte	2000	60.1%	97.8%	8.7%
Mexico	Mineral Del Monte	2017	88.6%	99.9%	45.2%
Mexico	Miquihuana	2000	81.5%	98.5%	44.8%
Mexico	Miquihuana	2017	96.1%	99.9%	76.0%
Mexico	Misantla	2000	28.0%	40.6%	14.0%
Mexico	Misantla	2017	61.8%	84.0%	41.0%
Mexico	Mitontic	2000	62.9%	98.8%	8.7%
Mexico	Mitontic	2017	88.1%	99.9%	35.7%
Mexico	Mixistlan De La Reforma	2000	67.1%	99.8%	1.5%
Mexico	Mixistlan De La Reforma	2017	89.6%	100.0%	16.5%
Mexico	Mixquiahuala De Juarez	2000	97.9%	99.6%	89.2%
Mexico	Mixquiahuala De Juarez	2017	99.8%	100.0%	99.1%
Mexico	Mixtla	2000	83.7%	97.7%	49.4%
Mexico	Mixtla	2017	98.6%	99.8%	93.3%
Mexico	Mixtla De Altamirano	2000	74.6%	99.5%	14.9%
Mexico	Mixtla De Altamirano	2017	94.0%	100.0%	55.8%
Mexico	Mixtlan	2000	82.3%	98.5%	51.2%
Mexico	Mixtlan	2017	96.8%	99.9%	85.6%
Mexico	Mochitlan	2000	76.7%	97.8%	32.6%
Mexico	Mochitlan	2017	95.0%	99.8%	77.0%
Mexico	Mococha	2000	80.1%	99.9%	19.0%
Mexico	Mococha	2017	96.1%	100.0%	63.2%
Mexico	Mocorito	2000	83.0%	94.1%	66.6%
Mexico	Mocorito	2017	96.1%	99.3%	88.7%
Mexico	Moctezuma	2000	87.0%	99.9%	19.6%
Mexico	Moctezuma	2000	76.8%	91.1%	58.3%
Mexico	Moctezuma	2017	97.7%	100.0%	78.6%
Mexico	Moctezuma	2017	95.7%	99.3%	85.9%
Mexico	Molango De Escamilla	2000	93.5%	99.6%	79.7%
Mexico	Molango De Escamilla	2017	99.2%	100.0%	95.0%
Mexico	Molcaxac	2000	70.6%	99.0%	17.3%
Mexico	Molcaxac	2017	92.7%	99.9%	56.9%
Mexico	Moloacan	2000	45.5%	84.4%	3.2%
Mexico	Moloacan	2017	75.0%	96.6%	19.7%
Mexico	Momax	2000	83.3%	99.7%	28.4%
Mexico	Momax	2017	96.7%	100.0%	79.7%
Mexico	Monclova	2000	97.2%	98.4%	95.3%

Country	Second Administrative Unit	Year	Mean	Upper	Lower
Mexico	Monclova	2017	99.8%	99.9%	99.7%
Mexico	Monjas	2000	69.7%	97.3%	17.5%
Mexico	Monjas	2017	94.0%	99.8%	66.9%
Mexico	Monte Escobedo	2000	78.5%	95.0%	45.1%
Mexico	Monte Escobedo	2017	95.7%	99.5%	83.3%
Mexico	Montecristo De Guerrero	2000	69.9%	98.3%	16.8%
Mexico	Montecristo De Guerrero	2017	92.5%	99.9%	56.3%
Mexico	Montemorelos	2000	88.9%	95.5%	78.5%
Mexico	Montemorelos	2017	98.9%	99.7%	97.5%
Mexico	Monterrey	2000	98.1%	99.0%	96.9%
Mexico	Monterrey	2017	99.9%	99.9%	99.6%
Mexico	Morelia	2000	66.5%	78.4%	49.1%
Mexico	Morelia	2017	83.7%	86.3%	77.5%
Mexico	Morelos	2000	64.0%	82.7%	40.1%
Mexico	Morelos	2000	96.3%	99.6%	72.1%
Mexico	Morelos	2000	78.0%	93.8%	49.0%
Mexico	Morelos	2000	91.1%	98.4%	72.7%
Mexico	Morelos	2000	93.7%	99.0%	72.5%
Mexico	Morelos	2017	99.6%	99.9%	98.3%
Mexico	Morelos	2017	89.4%	96.8%	78.3%
Mexico	Morelos	2017	96.2%	99.6%	84.0%
Mexico	Morelos	2017	99.6%	100.0%	96.3%
Mexico	Morelos	2017	99.1%	99.9%	95.3%
Mexico	Moris	2000	77.8%	96.1%	42.6%
Mexico	Moris	2017	95.0%	99.7%	77.6%
Mexico	Moroleon	2000	97.9%	99.7%	90.2%
Mexico	Moroleon	2017	99.8%	100.0%	99.3%
Mexico	Motozintla	2000	77.3%	94.6%	51.4%
Mexico	Motozintla	2017	94.8%	99.6%	80.1%
Mexico	Motul	2000	82.1%	88.6%	74.9%
Mexico	Motul	2017	98.2%	99.1%	95.7%
Mexico	Moyahua De Estrada	2000	78.6%	99.1%	31.9%
Mexico	Moyahua De Estrada	2017	95.1%	99.9%	73.5%
Mexico	Mugica	2000	79.1%	98.6%	29.6%
Mexico	Mugica	2017	96.9%	99.9%	80.3%
Mexico	Mulege	2000	63.2%	70.6%	54.7%
Mexico	Mulege	2017	92.3%	94.9%	88.6%
Mexico	Muna	2000	97.2%	99.5%	89.3%
Mexico	Muna	2017	99.8%	100.0%	99.0%
Mexico	Munoz De Domingo Arenas	2000	98.5%	99.9%	90.1%
Mexico	Munoz De Domingo Arenas	2017	99.9%	100.0%	99.1%
Mexico	Muxupip	2000	94.6%	98.2%	86.1%
Mexico	Muxupip	2017	99.5%	99.8%	99.0%
Mexico	Muzquiz	2000	94.3%	98.3%	81.9%
Mexico	Muzquiz	2017	99.6%	99.9%	98.7%
Mexico	Nacajuca	2000	78.8%	90.3%	59.2%
Mexico	Nacajuca	2017	96.6%	99.3%	85.5%
Mexico	Naco	2000	89.8%	99.7%	43.7%
Mexico	Naco	2017	98.8%	100.0%	92.8%
Mexico	Nacori Chico	2000	79.5%	97.8%	37.7%
Mexico	Nacori Chico	2017	96.0%	99.8%	83.5%
Mexico	Nacozari De Garcia	2000	94.4%	99.9%	65.0%

Country	Second Administrative Unit	Year	Mean	Upper	Lower
Mexico	Nacozari De Garcia	2017	99.2%	100.0%	94.0%
Mexico	Nadadores	2000	92.5%	99.0%	65.9%
Mexico	Nadadores	2017	99.0%	99.9%	94.0%
Mexico	Nahuatzen	2000	83.5%	98.0%	50.8%
Mexico	Nahuatzen	2017	97.4%	99.9%	87.9%
Mexico	Namiquipa	2000	56.5%	65.6%	46.9%
Mexico	Namiquipa	2017	90.9%	95.2%	84.0%
Mexico	Nanacamilpa De Mariano Arista	2000	98.1%	99.9%	90.2%
Mexico	Nanacamilpa De Mariano Arista	2017	99.9%	100.0%	99.3%
Mexico	Nanchital De Lazaro Cardenas Del Rio	2000	82.9%	89.2%	75.2%
Mexico	Nanchital De Lazaro Cardenas Del Rio	2017	98.7%	99.2%	97.9%
Mexico	Naolinco	2000	82.9%	99.8%	37.6%
Mexico	Naolinco	2017	97.1%	100.0%	82.3%
Mexico	Naranjal	2000	62.6%	94.5%	18.3%
Mexico	Naranjal	2017	91.5%	99.6%	66.4%
Mexico	Naranjos Amatlan	2000	74.3%	84.0%	63.7%
Mexico	Naranjos Amatlan	2017	96.4%	98.4%	93.4%
Mexico	Natividad	2000	73.1%	98.3%	13.5%
Mexico	Natividad	2017	96.1%	99.9%	72.5%
Mexico	Nativitas	2000	84.4%	87.5%	80.7%
Mexico	Nativitas	2017	98.8%	99.1%	98.4%
Mexico	Naucalpan De Juarez	2000	90.5%	95.8%	81.3%
Mexico	Naucalpan De Juarez	2017	98.9%	99.7%	96.7%
Mexico	Naupan	2000	77.1%	99.5%	24.6%
Mexico	Naupan	2017	95.2%	100.0%	66.7%
Mexico	Nautla	2000	55.6%	82.7%	24.4%
Mexico	Nautla	2017	87.6%	97.9%	67.8%
Mexico	Nauzontla	2000	68.0%	99.6%	6.1%
Mexico	Nauzontla	2017	90.6%	100.0%	42.4%
Mexico	Nava	2000	88.4%	97.9%	64.4%
Mexico	Nava	2017	98.9%	99.8%	94.8%
Mexico	Navojoa	2000	88.4%	91.5%	83.8%
Mexico	Navojoa	2017	99.1%	99.4%	98.1%
Mexico	Navolato	2000	88.4%	95.2%	76.1%
Mexico	Navolato	2017	98.3%	99.7%	94.6%
Mexico	Nazareno Etla	2000	55.3%	61.9%	48.9%
Mexico	Nazareno Etla	2017	95.1%	96.4%	93.5%
Mexico	Nazas	2000	87.4%	98.2%	57.2%
Mexico	Nazas	2017	98.0%	99.8%	90.0%
Mexico	Nealtican	2000	86.6%	92.7%	76.0%
Mexico	Nealtican	2017	98.9%	99.5%	97.9%
Mexico	Nejapa De Madero	2000	75.6%	98.2%	23.0%
Mexico	Nejapa De Madero	2017	93.8%	99.8%	58.4%
Mexico	Nextlalpan	2000	93.0%	96.0%	89.0%
Mexico	Nextlalpan	2017	99.6%	99.8%	99.3%
Mexico	Nezahualcoyotl	2000	98.4%	98.9%	97.9%
Mexico	Nezahualcoyotl	2017	99.9%	99.9%	99.9%

Country	Second Administrative Unit	Year	Mean	Upper	Lower
Mexico	Nicolas Bravo	2000	88.9%	98.7%	61.5%
Mexico	Nicolas Bravo	2017	98.1%	99.9%	84.3%
Mexico	Nicolas Flores	2000	78.3%	99.4%	23.4%
Mexico	Nicolas Flores	2017	94.7%	100.0%	65.8%
Mexico	Nicolas Romero	2000	87.5%	94.4%	74.4%
Mexico	Nicolas Romero	2017	98.7%	99.7%	93.5%
Mexico	Nicolas Ruiz	2000	97.9%	99.7%	91.7%
Mexico	Nicolas Ruiz	2017	99.9%	100.0%	99.3%
Mexico	Nochistlan De Mejia	2000	89.8%	95.1%	82.4%
Mexico	Nochistlan De Mejia	2017	98.1%	99.6%	94.2%
Mexico	Nocupetaro	2000	77.9%	98.0%	33.5%
Mexico	Nocupetaro	2017	94.8%	99.8%	76.3%
Mexico	Nogales	2000	39.6%	51.8%	27.9%
Mexico	Nogales	2000	97.6%	99.3%	93.4%
Mexico	Nogales	2017	89.3%	93.9%	81.5%
Mexico	Nogales	2017	99.8%	100.0%	99.4%
Mexico	Nombre De Dios	2000	85.1%	96.1%	67.3%
Mexico	Nombre De Dios	2017	97.1%	99.7%	87.5%
Mexico	Nonoava	2000	82.1%	97.5%	36.8%
Mexico	Nonoava	2017	96.4%	99.8%	83.0%
Mexico	Nopala De Villagran	2000	77.4%	98.4%	32.1%
Mexico	Nopala De Villagran	2017	94.9%	99.9%	77.6%
Mexico	Nopaltepec	2000	86.6%	98.9%	48.1%
Mexico	Nopaltepec	2017	97.7%	99.9%	82.6%
Mexico	Nopalucan	2000	88.5%	99.8%	43.8%
Mexico	Nopalucan	2017	98.2%	100.0%	85.8%
Mexico	Noria De Angeles	2000	89.0%	99.2%	67.3%
Mexico	Noria De Angeles	2017	97.8%	100.0%	86.9%
Mexico	Nuevo Casas Grandes	2000	93.2%	97.1%	87.4%
Mexico	Nuevo Casas Grandes	2017	99.4%	99.8%	98.8%
Mexico	Nuevo Ideal	2000	81.7%	94.3%	65.7%
Mexico	Nuevo Ideal	2017	96.2%	99.4%	89.2%
Mexico	Nuevo Laredo	2000	78.8%	89.3%	63.0%
Mexico	Nuevo Laredo	2017	95.4%	99.0%	87.6%
Mexico	Nuevo Morelos	2000	77.1%	99.5%	14.4%
Mexico	Nuevo Morelos	2017	93.0%	100.0%	37.4%
Mexico	Nuevo Parangaricutiro	2000	94.7%	99.6%	80.4%
Mexico	Nuevo Parangaricutiro	2017	99.5%	100.0%	97.8%
Mexico	Nuevo Urecho	2000	76.9%	99.1%	24.0%
Mexico	Nuevo Urecho	2017	94.5%	100.0%	67.9%
Mexico	Nuevo Zoquiapam	2000	75.4%	99.7%	13.1%
Mexico	Nuevo Zoquiapam	2017	93.6%	100.0%	53.2%

Country	Second Administrative Unit	Year	Mean	Upper	Lower
Mexico	Numaran	2000	77.2%	99.8%	29.6%
Mexico	Numaran	2017	94.3%	100.0%	71.8%
Mexico	Oaxaca De Juarez	2000	71.3%	75.2%	67.0%
Mexico	Oaxaca De Juarez	2017	97.6%	98.0%	97.1%
Mexico	Ocampo	2000	71.2%	85.2%	52.5%
Mexico	Ocampo	2000	80.9%	96.5%	55.4%
Mexico	Ocampo	2000	82.5%	97.0%	58.6%
Mexico	Ocampo	2000	21.8%	28.9%	16.2%
Mexico	Ocampo	2000	78.4%	99.7%	28.8%
Mexico	Ocampo	2000	72.7%	87.7%	55.9%
Mexico	Ocampo	2017	95.3%	98.8%	85.9%
Mexico	Ocampo	2017	96.8%	99.8%	90.7%
Mexico	Ocampo	2017	96.0%	99.7%	83.8%
Mexico	Ocampo	2017	51.0%	70.2%	34.6%
Mexico	Ocampo	2017	96.6%	100.0%	83.8%
Mexico	Ocampo	2017	95.1%	98.9%	88.0%
Mexico	Ocosingo	2000	68.4%	77.3%	56.8%
Mexico	Ocosingo	2017	91.3%	95.9%	84.8%
Mexico	Ocotepec	2000	68.9%	97.0%	17.6%
Mexico	Ocotepec	2000	79.2%	99.9%	16.2%
Mexico	Ocotepec	2017	94.7%	99.8%	70.4%
Mexico	Ocotepec	2017	95.0%	100.0%	62.8%
Mexico	Ocotlan	2000	68.9%	81.3%	50.4%
Mexico	Ocotlan	2017	95.9%	97.7%	93.2%
Mexico	Ocotlan De Morelos	2000	14.2%	28.0%	6.3%
Mexico	Ocotlan De Morelos	2017	52.9%	78.5%	26.3%
Mexico	Ocoyoacac	2000	56.1%	91.5%	19.1%
Mexico	Ocoyoacac	2017	92.2%	99.5%	70.2%
Mexico	Ocoyucan	2000	15.2%	18.4%	12.8%
Mexico	Ocoyucan	2017	61.4%	75.5%	48.0%
Mexico	Ocozocoautla De Espinosa	2000	79.3%	86.6%	70.2%
Mexico	Ocozocoautla De Espinosa	2017	95.3%	97.9%	90.8%
Mexico	Ocuilan	2000	75.1%	98.5%	22.3%
Mexico	Ocuilan	2017	93.6%	99.9%	63.6%
Mexico	Ocuituco	2000	95.8%	98.3%	91.2%
Mexico	Ocuituco	2017	99.7%	99.9%	99.1%
Mexico	Ojinaga	2000	86.2%	95.9%	64.1%
Mexico	Ojinaga	2017	98.4%	99.6%	93.8%
Mexico	Ojocaliente	2000	87.1%	97.2%	58.2%
Mexico	Ojocaliente	2017	97.9%	99.8%	87.5%
Mexico	Ojuelos De Jalisco	2000	82.5%	96.8%	49.3%
Mexico	Ojuelos De Jalisco	2017	96.8%	99.8%	88.2%
Mexico	Olinala	2000	73.8%	93.9%	35.9%
Mexico	Olinala	2017	94.9%	99.6%	78.8%
Mexico	Olintla	2000	33.3%	42.4%	23.9%
Mexico	Olintla	2017	82.0%	86.4%	76.9%
Mexico	Oluta	2000	67.7%	99.4%	4.4%
Mexico	Oluta	2017	92.0%	100.0%	41.4%
Mexico	Omealca	2000	64.7%	88.1%	30.9%
Mexico	Omealca	2017	92.5%	98.8%	77.8%
Mexico	Ometepec	2000	65.4%	76.9%	50.4%
Mexico	Ometepec	2017	95.2%	97.9%	90.1%
Mexico	Omitlan De Juarez	2000	64.9%	98.8%	8.6%

Country	Second Administrative Unit	Year	Mean	Upper	Lower
Mexico	Omitlan De Juarez	2017	87.4%	99.9%	43.5%
Mexico	Onavas	2000	79.4%	99.5%	10.8%
Mexico	Onavas	2017	94.3%	100.0%	60.8%
Mexico	Opichen	2000	72.6%	99.5%	10.5%
Mexico	Opichen	2017	91.7%	100.0%	38.9%
Mexico	Opodepe	2000	86.1%	99.2%	49.7%
Mexico	Opodepe	2017	97.8%	99.9%	87.0%
Mexico	Oquitoa	2000	78.8%	99.8%	6.3%
Mexico	Oquitoa	2017	94.1%	100.0%	43.6%
Mexico	Oriental	2000	87.5%	99.4%	56.3%
Mexico	Oriental	2017	96.9%	100.0%	86.6%
Mexico	Orizaba	2000	74.4%	78.2%	69.9%
Mexico	Orizaba	2017	97.9%	98.4%	97.3%
Mexico	Ostuacan	2000	57.9%	89.8%	14.8%
Mexico	Ostuacan	2017	85.6%	99.0%	44.1%
Mexico	Osumacinta	2000	65.7%	97.8%	7.0%
Mexico	Osumacinta	2017	89.5%	99.8%	43.2%
Mexico	Otaez	2000	77.9%	94.6%	48.9%
Mexico	Otaez	2017	95.5%	99.4%	82.1%
Mexico	Otatitlan	2000	38.3%	85.5%	0.5%
Mexico	Otatitlan	2017	63.6%	96.0%	6.4%
Mexico	Oteapan	2000	58.4%	99.1%	3.9%
Mexico	Oteapan	2017	88.0%	99.9%	34.9%
Mexico	Othon P. Blanco	2000	73.2%	82.6%	64.3%
Mexico	Othon P. Blanco	2017	95.5%	98.1%	90.7%
Mexico	Otumba	2000	90.5%	99.8%	47.3%
Mexico	Otumba	2017	98.2%	100.0%	87.3%
Mexico	Otzoloapan	2000	77.1%	99.1%	25.7%
Mexico	Otzoloapan	2017	94.8%	99.9%	72.2%
Mexico	Otzolotepec	2000	93.4%	99.9%	60.8%
Mexico	Otzolotepec	2017	98.8%	100.0%	90.1%
Mexico	Oxchuc	2000	69.0%	92.1%	28.4%
Mexico	Oxchuc	2017	93.1%	99.3%	72.4%
Mexico	Oxkutzcab	2000	81.2%	98.6%	42.4%
Mexico	Oxkutzcab	2017	96.5%	99.9%	82.5%
Mexico	Ozuluama De Mascarenas	2000	69.7%	89.2%	40.4%
Mexico	Ozuluama De Mascarenas	2017	91.0%	98.6%	73.1%
Mexico	Ozumba	2000	85.8%	99.9%	30.7%
Mexico	Ozumba	2017	96.8%	100.0%	77.1%
Mexico	Pabellon De Arteaga	2000	98.9%	99.6%	97.1%
Mexico	Pabellon De Arteaga	2017	99.9%	100.0%	99.8%
Mexico	Pachuca De Soto	2000	93.7%	97.8%	88.0%
Mexico	Pachuca De Soto	2017	99.2%	99.8%	97.4%
Mexico	Pacula	2000	82.8%	99.3%	38.1%
Mexico	Pacula	2017	96.1%	100.0%	76.8%
Mexico	Padilla	2000	84.1%	99.3%	42.6%
Mexico	Padilla	2017	96.9%	99.9%	80.9%
Mexico	Pahuatlan	2000	77.2%	99.4%	18.2%
Mexico	Pahuatlan	2017	95.9%	100.0%	68.0%
Mexico	Pajacuaran	2000	82.6%	99.7%	38.1%
Mexico	Pajacuaran	2017	96.6%	100.0%	77.2%
Mexico	Pajapan	2000	68.8%	98.1%	16.6%
Mexico	Pajapan	2017	91.3%	99.8%	48.4%

Country	Second Administrative Unit	Year	Mean	Upper	Lower
Mexico	Palenque	2000	69.0%	82.5%	51.7%
Mexico	Palenque	2017	91.1%	96.3%	84.3%
Mexico	Palizada	2000	82.5%	90.2%	67.0%
Mexico	Palizada	2017	94.4%	97.6%	89.9%
Mexico	Palmar De Bravo	2000	79.7%	91.1%	58.0%
Mexico	Palmar De Bravo	2017	97.5%	99.2%	92.0%
Mexico	Palmillas	2000	83.4%	98.7%	48.1%
Mexico	Palmillas	2017	97.5%	99.9%	86.2%
Mexico	Panaba	2000	82.7%	98.9%	41.8%
Mexico	Panaba	2017	95.8%	99.9%	64.7%
Mexico	Panindicuaro	2000	83.6%	98.8%	34.8%
Mexico	Panindicuaro	2017	97.1%	99.9%	81.5%
Mexico	Panotla	2000	98.4%	99.5%	95.1%
Mexico	Panotla	2017	99.9%	100.0%	99.7%
Mexico	Pantelho	2000	58.5%	81.8%	35.9%
Mexico	Pantelho	2017	91.6%	98.2%	78.5%
Mexico	Pantepec	2000	47.5%	66.5%	21.8%
Mexico	Pantepec	2000	69.9%	96.8%	23.4%
Mexico	Pantepec	2017	92.6%	99.8%	60.9%
Mexico	Pantepec	2017	90.9%	96.6%	74.4%
Mexico	Panuco	2000	68.9%	85.2%	44.8%
Mexico	Panuco	2000	87.4%	98.5%	62.8%
Mexico	Panuco	2017	98.2%	99.9%	91.3%
Mexico	Panuco	2017	93.8%	97.9%	85.5%
Mexico	Panuco De Coronado	2000	91.3%	98.4%	73.5%
Mexico	Panuco De Coronado	2017	98.3%	99.9%	93.4%
Mexico	Papalotla	2000	88.8%	100.0%	17.6%
Mexico	Papalotla	2017	97.6%	100.0%	77.7%
Mexico	Papalotla De Xicohtencatl	2000	94.7%	97.2%	90.2%
Mexico	Papalotla De Xicohtencatl	2017	99.6%	99.8%	99.3%
Mexico	Papantla	2000	51.0%	70.1%	32.6%
Mexico	Papantla	2017	88.0%	96.0%	74.1%
Mexico	Paracho	2000	76.0%	98.6%	19.0%
Mexico	Paracho	2017	93.8%	99.9%	56.9%
Mexico	Paracuaro	2000	79.6%	96.5%	51.0%
Mexico	Paracuaro	2017	96.1%	99.8%	83.3%
Mexico	Paraiso	2000	74.6%	89.4%	49.6%
Mexico	Paraiso	2017	95.6%	99.2%	85.1%
Mexico	Paras	2000	81.8%	99.3%	29.4%
Mexico	Paras	2017	96.0%	99.9%	75.3%
Mexico	Parras	2000	90.8%	96.3%	81.3%
Mexico	Parras	2017	99.0%	99.7%	97.7%
Mexico	Paso De Ovejas	2000	80.2%	98.4%	36.4%
Mexico	Paso De Ovejas	2017	96.0%	99.9%	77.8%
Mexico	Paso Del Macho	2000	44.3%	64.7%	23.7%
Mexico	Paso Del Macho	2017	84.7%	95.2%	65.9%
Mexico	Patzcuaro	2000	89.7%	95.3%	82.1%
Mexico	Patzcuaro	2017	97.9%	99.6%	93.7%
Mexico	Pedro Ascencio Alquisiras	2000	75.8%	98.3%	26.3%
Mexico	Pedro Ascencio Alquisiras	2017	93.6%	99.9%	62.2%

Country	Second Administrative Unit	Year	Mean	Upper	Lower
Mexico	Pedro Escobedo	2000	93.3%	99.1%	78.2%
Mexico	Pedro Escobedo	2017	99.2%	99.9%	94.8%
Mexico	Penamiller	2000	75.8%	93.7%	48.8%
Mexico	Penamiller	2017	94.5%	99.4%	78.5%
Mexico	Penjamillo	2000	72.4%	91.6%	43.7%
Mexico	Penjamillo	2017	94.9%	99.4%	75.6%
Mexico	Penjamo	2000	78.3%	95.2%	53.8%
Mexico	Penjamo	2017	95.9%	99.6%	84.1%
Mexico	Penon Blanco	2000	81.7%	93.8%	60.5%
Mexico	Penon Blanco	2017	96.9%	99.6%	88.3%
Mexico	Periban	2000	70.8%	94.0%	29.3%
Mexico	Periban	2017	95.3%	99.8%	73.8%
Mexico	Perote	2000	68.6%	84.7%	48.6%
Mexico	Perote	2017	94.6%	98.7%	88.1%
Mexico	Pesqueria	2000	81.5%	88.6%	74.0%
Mexico	Pesqueria	2017	98.1%	99.2%	95.6%
Mexico	Petatlan	2000	80.2%	97.4%	36.6%
Mexico	Petatlan	2017	95.2%	99.7%	72.7%
Mexico	Petlalcingo	2000	92.0%	99.8%	70.7%
Mexico	Petlalcingo	2017	98.9%	100.0%	94.6%
Mexico	Peto	2000	75.1%	96.2%	26.7%
Mexico	Peto	2017	95.8%	99.6%	80.0%
Mexico	Piaxtla	2000	71.7%	94.8%	38.5%
Mexico	Piaxtla	2017	94.9%	99.5%	80.2%
Mexico	Pichucalco	2000	62.0%	86.1%	34.9%
Mexico	Pichucalco	2017	91.9%	98.6%	79.7%
Mexico	Piedras Negras	2000	91.7%	97.6%	82.6%
Mexico	Piedras Negras	2017	99.0%	99.8%	96.4%
Mexico	Pihuamo	2000	79.9%	98.6%	29.7%
Mexico	Pihuamo	2017	95.7%	99.9%	75.2%
Mexico	Pijijiapan	2000	55.8%	70.0%	40.1%
Mexico	Pijijiapan	2017	90.7%	95.7%	82.2%
Mexico	Pilcaya	2000	43.7%	74.8%	8.1%
Mexico	Pilcaya	2017	69.3%	84.6%	35.4%
Mexico	Pinal De Amoles	2000	57.4%	70.1%	41.2%
Mexico	Pinal De Amoles	2017	80.3%	92.1%	69.4%
Mexico	Pinos	2000	70.5%	81.9%	56.9%
Mexico	Pinos	2017	92.0%	96.8%	84.2%
Mexico	Pinotepa De Don Luis	2000	69.7%	99.2%	8.3%
Mexico	Pinotepa De Don Luis	2017	92.1%	99.9%	41.0%
Mexico	Pisaflores	2000	67.5%	96.7%	18.5%
Mexico	Pisaflores	2017	92.9%	99.7%	66.6%
Mexico	Pitiquito	2000	89.8%	98.9%	68.0%
Mexico	Pitiquito	2017	97.3%	99.8%	80.7%
Mexico	Platon Sanchez	2000	56.9%	96.3%	10.9%
Mexico	Platon Sanchez	2017	86.0%	99.6%	36.8%
Mexico	Playa Vicente	2000	74.6%	91.6%	49.1%
Mexico	Playa Vicente	2017	94.6%	99.3%	84.8%
Mexico	Playas De Rosarito	2000	94.2%	98.0%	87.3%
Mexico	Playas De Rosarito	2017	99.5%	99.9%	98.7%

Country	Second Administrative Unit	Year	Mean	Upper	Lower
Mexico	Pluma Hidalgo	2000	73.8%	99.7%	14.0%
Mexico	Pluma Hidalgo	2017	94.2%	100.0%	63.2%
Mexico	Poanas	2000	86.4%	93.2%	76.0%
Mexico	Poanas	2017	98.0%	99.6%	93.0%
Mexico	Polotitlan	2000	77.3%	99.5%	26.6%
Mexico	Polotitlan	2017	94.1%	100.0%	68.0%
Mexico	Poncitlan	2000	82.3%	92.6%	63.1%
Mexico	Poncitlan	2017	96.5%	99.1%	88.5%
Mexico	Poza Rica De Hidalgo	2000	49.7%	63.8%	36.2%
Mexico	Poza Rica De Hidalgo	2017	92.8%	96.7%	81.8%
Mexico	Praxedis G. Guerrero	2000	86.4%	99.6%	50.4%
Mexico	Praxedis G. Guerrero	2017	97.4%	100.0%	82.1%
Mexico	Progreso	2000	87.0%	97.2%	66.6%
Mexico	Progreso	2000	83.4%	95.3%	65.9%
Mexico	Progreso	2017	97.8%	99.7%	91.7%
Mexico	Progreso	2017	98.0%	99.8%	91.5%
Mexico	Progreso De Obregon	2000	97.2%	99.2%	92.1%
Mexico	Progreso De Obregon	2017	99.8%	99.9%	99.5%
Mexico	Puebla	2000	67.6%	72.6%	62.8%
Mexico	Puebla	2017	93.5%	96.0%	90.8%
Mexico	Pueblo Nuevo	2000	83.2%	99.6%	27.6%
Mexico	Pueblo Nuevo	2000	80.1%	87.8%	70.0%
Mexico	Pueblo Nuevo	2017	97.2%	100.0%	75.3%
Mexico	Pueblo Nuevo	2017	95.9%	97.7%	93.3%
Mexico	Pueblo Nuevo Solistahuacan	2000	60.7%	95.5%	16.4%
Mexico	Pueblo Nuevo Solistahuacan	2017	87.9%	99.6%	48.3%
Mexico	Pueblo Viejo	2000	97.3%	99.3%	92.9%
Mexico	Pueblo Viejo	2017	99.5%	99.9%	98.4%
Mexico	Puente De Ixtla	2000	88.1%	93.4%	80.4%
Mexico	Puente De Ixtla	2017	98.4%	99.5%	95.3%
Mexico	Puente Nacional	2000	74.3%	93.2%	46.4%
Mexico	Puente Nacional	2017	93.3%	99.3%	72.0%
Mexico	Puerto Penasco	2000	94.2%	99.7%	64.1%
Mexico	Puerto Penasco	2017	99.5%	100.0%	95.5%
Mexico	Puerto Vallarta	2000	87.9%	93.8%	80.4%
Mexico	Puerto Vallarta	2017	98.0%	99.6%	94.7%
Mexico	Pungarabato	2000	71.0%	96.5%	33.6%
Mexico	Pungarabato	2017	94.7%	99.8%	77.4%
Mexico	Purepero	2000	85.1%	96.8%	58.7%
Mexico	Purepero	2017	98.4%	99.8%	93.1%
Mexico	Purisima Del Rincon	2000	91.4%	98.1%	80.3%
Mexico	Purisima Del Rincon	2017	98.8%	99.9%	94.4%

Country	Second Administrative Unit	Year	Mean	Upper	Lower
Mexico	Puruandiro	2000	91.1%	98.3%	76.3%
Mexico	Puruandiro	2017	98.3%	99.9%	92.8%
Mexico	Putla Villa De Guerrero	2000	70.3%	86.0%	51.1%
Mexico	Putla Villa De Guerrero	2017	94.8%	98.6%	84.5%
Mexico	Quecholac	2000	94.6%	99.8%	73.8%
Mexico	Quecholac	2017	99.3%	100.0%	94.5%
Mexico	Quechultenango	2000	71.1%	93.6%	33.3%
Mexico	Quechultenango	2017	94.1%	99.4%	72.5%
Mexico	Querendaro	2000	75.0%	96.9%	31.6%
Mexico	Querendaro	2017	96.2%	99.8%	81.3%
Mexico	Queretaro	2000	82.1%	89.5%	75.6%
Mexico	Queretaro	2017	91.5%	97.2%	85.7%
Mexico	Quimixtlan	2000	66.0%	96.4%	26.7%
Mexico	Quimixtlan	2017	90.5%	99.7%	62.9%
Mexico	Quintana Roo	2000	79.1%	99.9%	12.9%
Mexico	Quintana Roo	2017	96.2%	100.0%	73.5%
Mexico	Quiriego	2000	83.9%	96.9%	59.7%
Mexico	Quiriego	2017	96.5%	99.7%	86.7%
Mexico	Quiroga	2000	77.9%	99.2%	18.4%
Mexico	Quiroga	2017	94.9%	100.0%	64.8%
Mexico	Quitupan	2000	73.6%	97.5%	36.2%
Mexico	Quitupan	2017	93.1%	99.8%	68.9%
Mexico	Rafael Delgado	2000	56.7%	84.3%	28.2%
Mexico	Rafael Delgado	2017	93.2%	98.8%	81.5%
Mexico	Rafael Lara Grajales	2000	89.4%	100.0%	37.5%
Mexico	Rafael Lara Grajales	2017	98.5%	100.0%	88.9%
Mexico	Rafael Lucio	2000	85.4%	93.2%	70.9%
Mexico	Rafael Lucio	2017	98.9%	99.5%	97.5%
Mexico	Ramos Arizpe	2000	97.2%	98.7%	95.1%
Mexico	Ramos Arizpe	2017	99.7%	99.9%	99.1%
Mexico	Rayon	2000	61.6%	88.3%	25.2%
Mexico	Rayon	2000	83.7%	99.9%	26.1%
Mexico	Rayon	2000	40.3%	61.1%	20.3%
Mexico	Rayon	2000	94.6%	98.6%	81.1%
Mexico	Rayon	2017	97.9%	100.0%	86.0%
Mexico	Rayon	2017	99.7%	100.0%	98.8%
Mexico	Rayon	2017	88.8%	96.2%	73.7%
Mexico	Rayon	2017	85.5%	97.3%	55.1%
Mexico	Rayones	2000	82.6%	97.8%	54.5%
Mexico	Rayones	2017	96.7%	99.8%	85.2%
Mexico	Reforma	2000	80.9%	95.0%	56.9%
Mexico	Reforma	2017	97.7%	99.6%	93.2%
Mexico	Reforma De Pineda	2000	70.3%	98.8%	15.7%
Mexico	Reforma De Pineda	2017	94.1%	99.9%	61.1%
Mexico	Reyes Etla	2000	52.5%	62.4%	42.9%
Mexico	Reyes Etla	2017	93.5%	96.7%	86.5%
Mexico	Reynosa	2000	75.8%	83.6%	69.2%
Mexico	Reynosa	2017	96.2%	98.6%	90.3%
Mexico	Rincon De Romos	2000	96.9%	99.7%	89.4%
Mexico	Rincon De Romos	2017	99.5%	100.0%	96.8%
Mexico	Rio Blanco	2000	70.7%	76.5%	65.3%
Mexico	Rio Blanco	2017	97.7%	98.3%	97.0%

Country	Second Administrative Unit	Year	Mean	Upper	Lower
Mexico	Rio Bravo	2000	87.9%	93.3%	77.6%
Mexico	Rio Bravo	2017	97.5%	99.4%	90.8%
Mexico	Rio Grande	2000	92.8%	97.8%	81.4%
Mexico	Rio Grande	2017	98.8%	99.8%	93.5%
Mexico	Rio Lagartos	2000	70.4%	99.6%	8.6%
Mexico	Rio Lagartos	2017	92.0%	100.0%	37.3%
Mexico	Rioverde	2000	84.7%	91.0%	77.3%
Mexico	Rioverde	2017	97.8%	99.3%	94.9%
Mexico	Riva Palacio	2000	81.9%	96.9%	54.4%
Mexico	Riva Palacio	2017	96.6%	99.7%	88.8%
Mexico	Rodeo	2000	79.4%	95.5%	53.3%
Mexico	Rodeo	2017	96.7%	99.7%	89.6%
Mexico	Rojas De Cuauhtemoc	2000	57.1%	95.3%	10.6%
Mexico	Rojas De Cuauhtemoc	2017	91.4%	99.7%	52.3%
Mexico	Romita	2000	70.6%	95.3%	34.7%
Mexico	Romita	2017	92.5%	99.5%	73.9%
Mexico	Rosales	2000	60.7%	88.5%	38.1%
Mexico	Rosales	2017	91.2%	98.9%	72.6%
Mexico	Rosamorada	2000	77.0%	90.4%	58.0%
Mexico	Rosamorada	2017	95.1%	99.3%	84.9%
Mexico	Rosario	2000	76.9%	95.8%	38.2%
Mexico	Rosario	2000	86.1%	96.1%	58.3%
Mexico	Rosario	2000	83.2%	98.8%	45.6%
Mexico	Rosario	2017	97.4%	99.6%	89.5%
Mexico	Rosario	2017	96.9%	99.9%	80.5%
Mexico	Rosario	2017	94.3%	99.6%	70.5%
Mexico	Ruiz	2000	81.6%	97.8%	39.0%
Mexico	Ruiz	2017	96.7%	99.8%	82.4%
Mexico	Sabanilla	2000	82.4%	99.4%	37.5%
Mexico	Sabanilla	2017	96.3%	100.0%	75.2%
Mexico	Sabinas	2000	95.2%	98.8%	86.9%
Mexico	Sabinas	2017	99.6%	99.9%	98.9%
Mexico	Sabinas Hidalgo	2000	90.1%	97.3%	76.4%
Mexico	Sabinas Hidalgo	2017	98.9%	99.8%	96.0%
Mexico	Sacalum	2000	92.6%	99.3%	52.7%
Mexico	Sacalum	2017	99.1%	100.0%	93.1%
Mexico	Sacramento	2000	88.7%	99.8%	34.4%
Mexico	Sacramento	2017	98.5%	100.0%	89.6%
Mexico	Sahuaripa	2000	88.1%	97.3%	59.2%
Mexico	Sahuaripa	2017	98.0%	99.7%	92.8%
Mexico	Sahuayo	2000	89.0%	94.8%	79.6%
Mexico	Sahuayo	2017	99.0%	99.6%	97.5%
Mexico	Sain Alto	2000	82.8%	94.8%	65.3%
Mexico	Sain Alto	2017	96.8%	99.6%	89.2%
Mexico	Salamanca	2000	91.4%	97.9%	76.9%
Mexico	Salamanca	2017	98.8%	99.9%	94.8%
Mexico	Salina Cruz	2000	83.6%	94.3%	63.2%
Mexico	Salina Cruz	2017	98.3%	99.4%	95.7%
Mexico	Salinas	2000	74.9%	93.5%	38.9%
Mexico	Salinas	2017	95.5%	99.2%	87.0%
Mexico	Salinas Victoria	2000	96.9%	99.4%	90.4%
Mexico	Salinas Victoria	2017	99.6%	100.0%	97.5%
Mexico	Saltabarranca	2000	76.5%	96.7%	22.5%
Mexico	Saltabarranca	2017	96.3%	99.7%	76.0%
Mexico	Saltillo	2000	96.7%	98.1%	94.2%
Mexico	Saltillo	2017	99.7%	99.9%	99.1%

Country	Second Administrative Unit	Year	Mean	Upper	Lower
Mexico	Salto De Agua	2000	69.1%	90.5%	40.6%
Mexico	Salto De Agua	2017	91.4%	99.1%	76.7%
Mexico	Salvador Alvarado	2000	92.5%	97.2%	85.1%
Mexico	Salvador Alvarado	2017	99.3%	99.8%	98.2%
Mexico	Salvador Escalante	2000	89.8%	98.9%	70.8%
Mexico	Salvador Escalante	2017	98.1%	99.9%	90.9%
Mexico	Salvatierra	2000	84.9%	98.1%	62.1%
Mexico	Salvatierra	2017	97.4%	99.8%	88.1%
Mexico	Samahil	2000	81.0%	99.7%	26.5%
Mexico	Samahil	2017	96.8%	100.0%	74.8%
Mexico	San Agustin Amatengo	2000	70.8%	97.3%	17.8%
Mexico	San Agustin Amatengo	2017	94.9%	99.8%	73.9%
Mexico	San Agustin Atenango	2000	72.2%	99.8%	6.5%
Mexico	San Agustin Atenango	2017	92.8%	100.0%	43.3%
Mexico	San Agustin Chayuco	2000	71.8%	99.1%	19.6%
Mexico	San Agustin Chayuco	2017	92.9%	99.9%	63.0%
Mexico	San Agustin De Las Juntas	2000	66.2%	73.1%	58.6%
Mexico	San Agustin De Las Juntas	2017	96.3%	97.7%	94.2%
Mexico	San Agustin Etla	2000	50.4%	85.1%	16.4%
Mexico	San Agustin Etla	2017	95.4%	99.3%	82.6%
Mexico	San Agustin Loxicha	2000	61.1%	87.8%	30.3%
Mexico	San Agustin Loxicha	2017	92.2%	98.7%	73.6%
Mexico	San Agustin Metzquititlan	2000	68.4%	87.8%	43.1%
Mexico	San Agustin Metzquititlan	2017	94.9%	99.0%	84.9%
Mexico	San Agustin Tlacotepec	2000	75.1%	99.9%	4.6%
Mexico	San Agustin Tlacotepec	2017	93.2%	100.0%	43.2%
Mexico	San Agustin Tlaxiaca	2000	97.4%	99.9%	89.0%
Mexico	San Agustin Tlaxiaca	2017	99.6%	100.0%	97.9%
Mexico	San Agustin Yatareni	2000	79.2%	83.7%	73.4%
Mexico	San Agustin Yatareni	2017	98.4%	98.9%	97.5%
Mexico	San Andres Cabecera Nueva	2000	73.7%	98.5%	21.7%
Mexico	San Andres Cabecera Nueva	2017	92.9%	99.9%	60.1%
Mexico	San Andres Cholula	2000	34.1%	36.9%	31.5%

Country	Second Administrative Unit	Year	Mean	Upper	Lower
Mexico	San Andres Cholula	2017	84.3%	88.6%	80.5%
Mexico	San Andres Dinicuiti	2000	82.4%	99.8%	27.8%
Mexico	San Andres Dinicuiti	2017	97.5%	100.0%	83.1%
Mexico	San Andres Duraznal	2000	65.2%	78.1%	47.3%
Mexico	San Andres Duraznal	2017	96.2%	97.9%	91.1%
Mexico	San Andres Huaxpaltepec	2000	63.3%	98.3%	11.5%
Mexico	San Andres Huaxpaltepec	2017	89.3%	99.9%	49.1%
Mexico	San Andres Huayapam	2000	81.2%	96.0%	52.1%
Mexico	San Andres Huayapam	2017	98.3%	99.7%	94.3%
Mexico	San Andres Ixtlahuaca	2000	67.2%	93.9%	19.6%
Mexico	San Andres Ixtlahuaca	2017	94.7%	99.8%	66.8%
Mexico	San Andres Lagunas	2000	75.9%	99.6%	8.6%
Mexico	San Andres Lagunas	2017	94.8%	100.0%	57.4%
Mexico	San Andres Nuxino	2000	78.4%	99.8%	15.7%
Mexico	San Andres Nuxino	2017	94.7%	100.0%	61.9%
Mexico	San Andres Paxtlan	2000	73.6%	99.7%	8.8%
Mexico	San Andres Paxtlan	2017	93.7%	100.0%	49.6%
Mexico	San Andres Sinaxtla	2000	60.9%	85.1%	24.5%
Mexico	San Andres Sinaxtla	2017	94.5%	98.9%	74.2%
Mexico	San Andres Solaga	2000	73.5%	96.4%	32.8%
Mexico	San Andres Solaga	2017	95.4%	99.8%	75.4%
Mexico	San Andres Tenejapan	2000	54.5%	95.6%	8.7%
Mexico	San Andres Tenejapan	2017	84.6%	99.8%	29.0%
Mexico	San Andres Teotilalpam	2000	66.8%	92.2%	28.4%
Mexico	San Andres Teotilalpam	2017	90.4%	99.5%	49.2%
Mexico	San Andres Tepetlapa	2000	76.8%	99.9%	14.4%
Mexico	San Andres Tepetlapa	2017	93.3%	100.0%	55.1%
Mexico	San Andres Tuxtla	2000	70.5%	82.0%	55.2%
Mexico	San Andres Tuxtla	2017	94.3%	97.9%	86.1%
Mexico	San Andres Yaa	2000	73.0%	99.9%	8.3%
Mexico	San Andres Yaa	2017	94.5%	100.0%	64.8%

Country	Second Administrative Unit	Year	Mean	Upper	Lower
Mexico	San Andres Zabache	2000	81.3%	100.0%	9.8%
Mexico	San Andres Zabache	2017	96.0%	100.0%	63.4%
Mexico	San Andres Zautla	2000	51.9%	68.9%	33.4%
Mexico	San Andres Zautla	2017	93.5%	97.3%	81.8%
Mexico	San Antonino Castillo Velasco	2000	19.2%	74.7%	0.6%
Mexico	San Antonino Castillo Velasco	2017	59.0%	97.9%	5.6%
Mexico	San Antonino El Alto	2000	78.4%	99.8%	23.3%
Mexico	San Antonino El Alto	2017	94.9%	100.0%	68.0%
Mexico	San Antonino Monte Verde	2000	79.8%	99.9%	19.2%
Mexico	San Antonino Monte Verde	2017	95.9%	100.0%	71.5%
Mexico	San Antonio	2000	32.5%	42.4%	21.0%
Mexico	San Antonio	2017	82.4%	88.6%	74.1%
Mexico	San Antonio Acutla	2000	70.3%	99.8%	5.0%
Mexico	San Antonio Acutla	2017	92.2%	100.0%	39.6%
Mexico	San Antonio Canada	2000	75.7%	98.1%	30.4%
Mexico	San Antonio Canada	2017	95.9%	99.9%	73.6%
Mexico	San Antonio De La Cal	2000	69.9%	76.5%	62.0%
Mexico	San Antonio De La Cal	2017	97.4%	98.1%	96.4%
Mexico	San Antonio Huitepec	2000	77.8%	98.1%	26.7%
Mexico	San Antonio Huitepec	2017	96.2%	99.9%	78.4%
Mexico	San Antonio La Isla	2000	98.0%	99.8%	91.4%
Mexico	San Antonio La Isla	2017	99.9%	100.0%	99.5%
Mexico	San Antonio Nanahuatipam	2000	75.3%	99.2%	18.8%
Mexico	San Antonio Nanahuatipam	2017	95.3%	100.0%	69.4%
Mexico	San Antonio Sinicahua	2000	76.2%	99.8%	8.3%
Mexico	San Antonio Sinicahua	2017	94.2%	100.0%	54.5%
Mexico	San Antonio Tepetlapa	2000	68.0%	99.0%	6.4%
Mexico	San Antonio Tepetlapa	2017	90.8%	99.9%	34.6%
Mexico	San Baltazar Chichicapam	2000	74.9%	100.0%	2.6%
Mexico	San Baltazar Chichicapam	2017	92.3%	100.0%	29.8%

Country	Second Administrative Unit	Year	Mean	Upper	Lower
Mexico	San Baltazar Loxicha	2000	71.9%	99.0%	10.0%
Mexico	San Baltazar Loxicha	2017	94.3%	99.9%	63.3%
Mexico	San Baltazar Yatzachi El Bajo	2000	76.8%	97.2%	32.5%
Mexico	San Baltazar Yatzachi El Bajo	2017	96.3%	99.8%	73.2%
Mexico	San Bartolo Coyotepec	2000	60.5%	96.9%	10.0%
Mexico	San Bartolo Coyotepec	2017	91.9%	99.7%	61.2%
Mexico	San Bartolo Soyaltepec	2000	73.4%	99.1%	26.5%
Mexico	San Bartolo Soyaltepec	2017	94.0%	99.9%	66.0%
Mexico	San Bartolo Tutotepec	2000	68.7%	84.0%	45.0%
Mexico	San Bartolo Tutotepec	2017	93.9%	98.4%	83.1%
Mexico	San Bartolo Yautepec	2000	79.0%	99.9%	15.5%
Mexico	San Bartolo Yautepec	2017	95.5%	100.0%	73.1%
Mexico	San Bartolome Ayautla	2000	52.1%	98.8%	1.9%
Mexico	San Bartolome Ayautla	2017	81.7%	99.9%	18.7%
Mexico	San Bartolome Loxicha	2000	73.0%	99.2%	11.3%
Mexico	San Bartolome Loxicha	2017	93.3%	99.9%	54.8%
Mexico	San Bartolome Quialana	2000	79.0%	99.7%	14.5%
Mexico	San Bartolome Quialana	2017	96.0%	100.0%	64.5%
Mexico	San Bartolome Yucuane	2000	76.2%	99.8%	13.4%
Mexico	San Bartolome Yucuane	2017	93.9%	100.0%	54.3%
Mexico	San Bartolome Zoogocho	2000	76.6%	92.9%	49.2%
Mexico	San Bartolome Zoogocho	2017	97.7%	99.5%	92.8%
Mexico	San Bernardo	2000	78.2%	95.8%	48.5%
Mexico	San Bernardo	2017	94.8%	99.5%	79.4%
Mexico	San Bernardo Mixtepec	2000	72.5%	97.6%	15.7%
Mexico	San Bernardo Mixtepec	2017	93.5%	99.9%	62.9%

Country	Second Administrative Unit	Year	Mean	Upper	Lower
Mexico	San Blas	2000	76.2%	93.2%	49.2%
Mexico	San Blas	2017	95.1%	99.4%	84.9%
Mexico	San Blas Atempa	2000	76.3%	96.8%	36.7%
Mexico	San Blas Atempa	2017	95.2%	99.7%	74.7%
Mexico	San Buenaventura	2000	92.6%	98.3%	77.4%
Mexico	San Buenaventura	2017	99.3%	99.9%	97.7%
Mexico	San Carlos	2000	76.8%	91.4%	54.6%
Mexico	San Carlos	2017	93.7%	98.9%	79.4%
Mexico	San Carlos Yautepec	2000	74.2%	90.6%	55.6%
Mexico	San Carlos Yautepec	2017	94.1%	99.0%	85.0%
Mexico	San Ciro De Acosta	2000	90.1%	98.4%	57.1%
Mexico	San Ciro De Acosta	2017	98.8%	99.9%	94.3%
Mexico	San Cristobal Amatlan	2000	76.7%	99.9%	13.5%
Mexico	San Cristobal Amatlan	2017	93.0%	100.0%	43.8%
Mexico	San Cristobal Amoltepec	2000	72.1%	99.0%	20.5%
Mexico	San Cristobal Amoltepec	2017	94.9%	99.9%	74.5%
Mexico	San Cristobal De La Barranca	2000	78.0%	98.8%	28.4%
Mexico	San Cristobal De La Barranca	2017	95.4%	99.9%	76.8%
Mexico	San Cristobal De Las Casas	2000	74.4%	79.6%	66.5%
Mexico	San Cristobal De Las Casas	2017	97.5%	98.2%	95.0%
Mexico	San Cristobal Lachirioag	2000	70.1%	99.5%	9.1%
Mexico	San Cristobal Lachirioag	2017	92.8%	100.0%	55.7%
Mexico	San Cristobal Suchixtlahuaca	2000	72.4%	99.4%	5.5%
Mexico	San Cristobal Suchixtlahuaca	2017	92.9%	100.0%	27.5%
Mexico	San Damian Texoloc	2000	97.9%	98.9%	95.8%
Mexico	San Damian Texoloc	2017	99.9%	99.9%	99.7%
Mexico	San Diego De Alejandria	2000	89.0%	99.3%	49.1%
Mexico	San Diego De Alejandria	2017	98.2%	100.0%	87.7%
Mexico	San Diego De La Union	2000	80.3%	97.5%	51.5%
Mexico	San Diego De La Union	2017	95.8%	99.8%	80.4%
Mexico	San Diego La Mesa Tochimiltzingo	2000	82.0%	99.6%	28.7%

Country	Second Administrative Unit	Year	Mean	Upper	Lower
Mexico	San Diego La Mesa Tochimiltzingo	2017	96.5%	100.0%	77.7%
Mexico	San Dimas	2000	69.2%	84.3%	51.4%
Mexico	San Dimas	2017	93.1%	97.3%	82.5%
Mexico	San Dionisio Del Mar	2000	75.2%	98.0%	22.1%
Mexico	San Dionisio Del Mar	2017	94.5%	99.9%	65.1%
Mexico	San Dionisio Ocotepec	2000	76.7%	95.1%	41.6%
Mexico	San Dionisio Ocotepec	2017	94.8%	99.7%	69.8%
Mexico	San Dionisio Ocotlan	2000	13.4%	35.4%	3.3%
Mexico	San Dionisio Ocotlan	2017	49.3%	83.4%	23.9%
Mexico	San Esteban Atatlahuca	2000	71.4%	99.5%	7.0%
Mexico	San Esteban Atatlahuca	2017	91.7%	99.9%	41.9%
Mexico	San Felipe	2000	87.5%	95.9%	73.8%
Mexico	San Felipe	2000	72.6%	99.8%	2.6%
Mexico	San Felipe	2017	90.7%	100.0%	22.1%
Mexico	San Felipe	2017	97.5%	99.6%	93.1%
Mexico	San Felipe De Jesus	2000	95.9%	99.9%	80.6%
Mexico	San Felipe De Jesus	2017	99.7%	100.0%	98.6%
Mexico	San Felipe Del Progreso	2000	58.8%	75.9%	40.2%
Mexico	San Felipe Del Progreso	2017	89.7%	97.1%	77.1%
Mexico	San Felipe Jalapa De Diaz	2000	18.1%	27.1%	11.3%
Mexico	San Felipe Jalapa De Diaz	2017	67.4%	76.1%	56.7%
Mexico	San Felipe Orizatlan	2000	64.0%	73.8%	54.1%
Mexico	San Felipe Orizatlan	2017	84.9%	90.3%	75.9%
Mexico	San Felipe Tejalapam	2000	73.0%	91.0%	41.3%
Mexico	San Felipe Tejalapam	2017	96.7%	99.4%	85.1%
Mexico	San Felipe Teotlalcingo	2000	89.9%	96.6%	74.9%
Mexico	San Felipe Teotlalcingo	2017	99.0%	99.6%	97.4%
Mexico	San Felipe Tepatlan	2000	57.8%	94.3%	11.8%
Mexico	San Felipe Tepatlan	2017	89.3%	99.4%	46.4%
Mexico	San Felipe Usila	2000	55.9%	95.8%	10.8%
Mexico	San Felipe Usila	2017	85.1%	99.6%	39.6%
Mexico	San Fernando	2000	65.2%	92.5%	18.6%
Mexico	San Fernando	2000	80.0%	92.5%	57.6%
Mexico	San Fernando	2017	96.5%	99.2%	91.0%

Piped Water Proportion by Second Administrative Unit *(continued)*

Country	Second Administrative Unit	Year	Mean	Upper	Lower
Mexico	San Fernando	2017	91.8%	99.4%	51.3%
Mexico	San Francisco Cahuacua	2000	76.7%	98.8%	31.3%
Mexico	San Francisco Cahuacua	2017	95.1%	99.9%	75.2%
Mexico	San Francisco Cajonos	2000	76.6%	99.2%	19.3%
Mexico	San Francisco Cajonos	2017	96.1%	99.9%	75.7%
Mexico	San Francisco Chapulapa	2000	48.3%	72.0%	23.3%
Mexico	San Francisco Chapulapa	2017	91.8%	97.7%	76.3%
Mexico	San Francisco Chindua	2000	64.3%	97.4%	8.6%
Mexico	San Francisco Chindua	2017	92.9%	99.8%	55.5%
Mexico	San Francisco De Borja	2000	82.2%	99.4%	33.5%
Mexico	San Francisco De Borja	2017	96.5%	99.9%	77.7%
Mexico	San Francisco De Conchos	2000	79.7%	99.3%	30.3%
Mexico	San Francisco De Conchos	2017	95.6%	100.0%	72.7%
Mexico	San Francisco De Los Romo	2000	72.9%	91.6%	58.1%
Mexico	San Francisco De Los Romo	2017	93.6%	99.4%	81.9%
Mexico	San Francisco Del Mar	2000	71.2%	98.2%	18.9%
Mexico	San Francisco Del Mar	2017	92.4%	99.8%	63.0%
Mexico	San Francisco Del Oro	2000	86.0%	99.0%	40.6%
Mexico	San Francisco Del Oro	2017	98.5%	99.9%	91.4%
Mexico	San Francisco Del Rincon	2000	94.4%	98.1%	86.2%
Mexico	San Francisco Del Rincon	2017	99.2%	99.9%	96.8%
Mexico	San Francisco Huehuetlan	2000	95.2%	99.5%	84.2%
Mexico	San Francisco Huehuetlan	2017	99.7%	100.0%	98.7%
Mexico	San Francisco Ixhuatan	2000	71.3%	98.9%	17.6%
Mexico	San Francisco Ixhuatan	2017	93.2%	99.9%	63.4%
Mexico	San Francisco Jaltepetongo	2000	62.8%	98.1%	7.0%
Mexico	San Francisco Jaltepetongo	2017	90.1%	99.9%	40.1%
Mexico	San Francisco Lachigolo	2000	63.2%	96.3%	11.4%
Mexico	San Francisco Lachigolo	2017	92.0%	99.8%	49.7%
Mexico	San Francisco Logueche	2000	83.1%	99.9%	23.1%
Mexico	San Francisco Logueche	2017	96.7%	100.0%	75.7%
Mexico	San Francisco Nuxano	2000	71.3%	99.6%	9.3%

Country	Second Administrative Unit	Year	Mean	Upper	Lower
Mexico	San Francisco Nuxano	2017	93.7%	100.0%	54.2%
Mexico	San Francisco Ozolotepec	2000	78.4%	99.9%	9.5%
Mexico	San Francisco Ozolotepec	2017	95.0%	100.0%	56.4%
Mexico	San Francisco Sola	2000	80.4%	99.5%	32.8%
Mexico	San Francisco Sola	2017	97.0%	100.0%	80.5%
Mexico	San Francisco Telixtlahuaca	2000	69.9%	87.9%	42.7%
Mexico	San Francisco Telixtlahuaca	2017	96.4%	99.3%	87.1%
Mexico	San Francisco Teopan	2000	72.9%	99.9%	4.1%
Mexico	San Francisco Teopan	2017	91.2%	100.0%	35.3%
Mexico	San Francisco Tetlanohcan	2000	99.8%	100.0%	99.3%
Mexico	San Francisco Tetlanohcan	2017	100.0%	100.0%	100.0%
Mexico	San Francisco Tlapancingo	2000	77.2%	99.7%	25.3%
Mexico	San Francisco Tlapancingo	2017	95.6%	100.0%	77.0%
Mexico	San Gabriel	2000	81.6%	98.6%	37.2%
Mexico	San Gabriel	2017	96.5%	99.9%	80.5%
Mexico	San Gabriel Chilac	2000	95.3%	98.3%	88.5%
Mexico	San Gabriel Chilac	2017	99.7%	99.9%	99.2%
Mexico	San Gabriel Mixtepec	2000	66.0%	94.2%	14.0%
Mexico	San Gabriel Mixtepec	2017	91.0%	99.5%	43.4%
Mexico	San Gregorio Atzompa	2000	45.1%	51.5%	38.5%
Mexico	San Gregorio Atzompa	2017	94.4%	95.5%	92.9%
Mexico	San Ignacio	2000	79.1%	94.4%	50.9%
Mexico	San Ignacio	2017	95.3%	99.2%	81.1%
Mexico	San Ignacio Rio Muerto	2000	93.8%	99.6%	79.5%
Mexico	San Ignacio Rio Muerto	2017	99.1%	100.0%	95.5%
Mexico	San Ildefonso Amatlan	2000	71.9%	97.5%	20.3%
Mexico	San Ildefonso Amatlan	2017	94.7%	99.9%	63.3%
Mexico	San Ildefonso Sola	2000	73.7%	99.0%	24.2%
Mexico	San Ildefonso Sola	2017	95.4%	99.9%	78.3%
Mexico	San Ildefonso Villa Alta	2000	69.8%	98.7%	14.3%
Mexico	San Ildefonso Villa Alta	2017	91.8%	99.9%	55.9%
Mexico	San Jacinto Amilpas	2000	72.0%	77.1%	66.9%
Mexico	San Jacinto Amilpas	2017	97.7%	98.2%	97.1%

Country	Second Administrative Unit	Year	Mean	Upper	Lower
Mexico	San Jacinto Tlacotepec	2000	78.7%	99.8%	18.1%
Mexico	San Jacinto Tlacotepec	2017	95.2%	100.0%	60.4%
Mexico	San Javier	2000	85.9%	99.8%	25.5%
Mexico	San Javier	2017	97.8%	100.0%	80.9%
Mexico	San Jeronimo Coatlan	2000	82.1%	98.2%	51.8%
Mexico	San Jeronimo Coatlan	2017	95.6%	99.8%	78.3%
Mexico	San Jeronimo Silacayoapilla	2000	75.2%	99.5%	7.7%
Mexico	San Jeronimo Silacayoapilla	2017	94.3%	100.0%	52.8%
Mexico	San Jeronimo Sosola	2000	74.4%	98.6%	28.2%
Mexico	San Jeronimo Sosola	2017	93.5%	99.9%	65.5%
Mexico	San Jeronimo Taviche	2000	71.3%	93.8%	27.2%
Mexico	San Jeronimo Taviche	2017	97.0%	99.2%	91.6%
Mexico	San Jeronimo Tecoatl	2000	96.3%	98.7%	91.9%
Mexico	San Jeronimo Tecoatl	2017	99.7%	99.9%	99.3%
Mexico	San Jeronimo Tecuanipan	2000	60.2%	68.0%	51.9%
Mexico	San Jeronimo Tecuanipan	2017	96.6%	97.5%	95.3%
Mexico	San Jeronimo Tlacochahuaya	2000	74.5%	99.4%	8.8%
Mexico	San Jeronimo Tlacochahuaya	2017	93.2%	99.9%	55.7%
Mexico	San Jeronimo Xayacatlan	2000	79.6%	99.8%	11.5%
Mexico	San Jeronimo Xayacatlan	2017	94.5%	100.0%	59.3%
Mexico	San Jeronimo Zacualpan	2000	98.8%	99.4%	97.9%
Mexico	San Jeronimo Zacualpan	2017	99.9%	100.0%	99.9%
Mexico	San Joaquin	2000	78.8%	96.5%	47.0%
Mexico	San Joaquin	2017	96.3%	99.7%	84.9%
Mexico	San Jorge Nuchita	2000	71.4%	98.6%	14.5%
Mexico	San Jorge Nuchita	2017	93.8%	100.0%	55.0%
Mexico	San Jose Ayuquila	2000	77.7%	99.9%	9.6%
Mexico	San Jose Ayuquila	2017	93.9%	100.0%	39.9%
Mexico	San Jose Chiapa	2000	90.3%	99.8%	45.9%
Mexico	San Jose Chiapa	2017	98.2%	100.0%	86.7%
Mexico	San Jose Chiltepec	2000	64.8%	97.7%	13.9%
Mexico	San Jose Chiltepec	2017	90.5%	99.9%	51.8%

Country	Second Administrative Unit	Year	Mean	Upper	Lower
Mexico	San Jose De Gracia	2000	96.5%	99.7%	88.1%
Mexico	San Jose De Gracia	2017	99.6%	100.0%	98.0%
Mexico	San Jose Del Penasco	2000	69.1%	85.9%	46.9%
Mexico	San Jose Del Penasco	2017	96.3%	98.6%	90.9%
Mexico	San Jose Del Progreso	2000	44.0%	86.8%	4.6%
Mexico	San Jose Del Progreso	2017	76.5%	98.9%	25.4%
Mexico	San Jose Del Rincon	2000	78.6%	97.4%	41.8%
Mexico	San Jose Del Rincon	2017	94.9%	99.7%	78.9%
Mexico	San Jose Estancia Grande	2000	65.2%	98.9%	2.2%
Mexico	San Jose Estancia Grande	2017	89.6%	99.9%	23.0%
Mexico	San Jose Independencia	2000	62.9%	99.2%	5.9%
Mexico	San Jose Independencia	2017	88.6%	100.0%	44.7%
Mexico	San Jose Iturbide	2000	93.0%	99.2%	79.7%
Mexico	San Jose Iturbide	2017	99.0%	100.0%	94.3%
Mexico	San Jose Lachiguiri	2000	81.9%	99.9%	14.0%
Mexico	San Jose Lachiguiri	2017	95.7%	100.0%	56.6%
Mexico	San Jose Miahuatlan	2000	87.3%	99.6%	39.7%
Mexico	San Jose Miahuatlan	2017	98.0%	100.0%	89.0%
Mexico	San Jose Teacalco	2000	99.8%	100.0%	98.8%
Mexico	San Jose Teacalco	2017	100.0%	100.0%	99.9%
Mexico	San Jose Tenango	2000	44.2%	84.6%	9.0%
Mexico	San Jose Tenango	2017	80.7%	98.3%	41.7%
Mexico	San Juan Achiutla	2000	76.9%	99.5%	10.6%
Mexico	San Juan Achiutla	2017	94.8%	100.0%	55.3%
Mexico	San Juan Atenco	2000	79.5%	99.7%	22.7%
Mexico	San Juan Atenco	2017	94.8%	100.0%	68.7%
Mexico	San Juan Atepec	2000	75.3%	99.8%	8.7%
Mexico	San Juan Atepec	2017	94.2%	100.0%	50.6%
Mexico	San Juan Atzompa	2000	82.8%	99.9%	20.7%
Mexico	San Juan Atzompa	2017	97.0%	100.0%	79.0%
Mexico	San Juan Bautista Atatlahuca	2000	75.6%	99.3%	30.9%

Piped Water Proportion by Second Administrative Unit *(continued)*

Country	Second Administrative Unit	Year	Mean	Upper	Lower
Mexico	San Juan Bautista Atatlahuca	2017	92.5%	100.0%	44.2%
Mexico	San Juan Bautista Coixtlahuaca	2000	75.5%	98.6%	15.5%
Mexico	San Juan Bautista Coixtlahuaca	2017	93.2%	99.9%	50.8%
Mexico	San Juan Bautista Cuicatlan	2000	68.8%	90.8%	33.7%
Mexico	San Juan Bautista Cuicatlan	2017	93.3%	99.2%	69.6%
Mexico	San Juan Bautista Guelache	2000	45.7%	84.1%	10.5%
Mexico	San Juan Bautista Guelache	2017	87.9%	98.8%	50.2%
Mexico	San Juan Bautista Jayacatlan	2000	76.8%	99.7%	15.0%
Mexico	San Juan Bautista Jayacatlan	2017	94.8%	100.0%	61.9%
Mexico	San Juan Bautista Lo De Soto	2000	72.4%	99.8%	15.5%
Mexico	San Juan Bautista Lo De Soto	2017	91.8%	100.0%	42.7%
Mexico	San Juan Bautista Suchitepec	2000	82.4%	99.9%	16.9%
Mexico	San Juan Bautista Suchitepec	2017	96.7%	100.0%	74.9%
Mexico	San Juan Bautista Tlachichilco	2000	65.2%	96.1%	19.2%
Mexico	San Juan Bautista Tlachichilco	2017	93.2%	99.7%	65.5%
Mexico	San Juan Bautista Tlacoatzintepec	2000	62.2%	99.6%	2.0%
Mexico	San Juan Bautista Tlacoatzintepec	2017	87.6%	100.0%	23.6%
Mexico	San Juan Bautista Tuxtepec	2000	71.0%	77.9%	60.0%
Mexico	San Juan Bautista Tuxtepec	2017	91.0%	95.8%	83.2%
Mexico	San Juan Bautista Valle Nacional	2000	53.2%	90.7%	16.7%
Mexico	San Juan Bautista Valle Nacional	2017	86.8%	99.0%	55.7%

Country	Second Administrative Unit	Year	Mean	Upper	Lower
Mexico	San Juan Cacahuatepec	2000	83.7%	99.7%	26.5%
Mexico	San Juan Cacahuatepec	2017	96.3%	100.0%	70.8%
Mexico	San Juan Cancuc	2000	58.0%	93.8%	13.4%
Mexico	San Juan Cancuc	2017	86.5%	99.3%	55.2%
Mexico	San Juan Chicomezuchil	2000	76.8%	99.8%	6.3%
Mexico	San Juan Chicomezuchil	2017	94.3%	100.0%	46.2%
Mexico	San Juan Chilateca	2000	19.9%	76.5%	0.4%
Mexico	San Juan Chilateca	2017	59.4%	98.0%	5.4%
Mexico	San Juan Cieneguilla	2000	77.1%	99.3%	23.3%
Mexico	San Juan Cieneguilla	2017	95.0%	100.0%	70.6%
Mexico	San Juan Coatzospam	2000	59.2%	91.3%	17.2%
Mexico	San Juan Coatzospam	2017	91.2%	99.4%	66.5%
Mexico	San Juan Colorado	2000	70.7%	99.4%	13.5%
Mexico	San Juan Colorado	2017	92.2%	100.0%	45.4%
Mexico	San Juan Comaltepec	2000	58.8%	92.2%	23.7%
Mexico	San Juan Comaltepec	2017	90.2%	99.5%	61.0%
Mexico	San Juan Cotzocon	2000	70.8%	91.0%	45.6%
Mexico	San Juan Cotzocon	2017	92.3%	99.0%	75.5%
Mexico	San Juan De Guadalupe	2000	84.2%	95.5%	67.0%
Mexico	San Juan De Guadalupe	2017	97.1%	99.6%	89.6%
Mexico	San Juan De Los Cues	2000	67.1%	88.8%	31.4%
Mexico	San Juan De Los Cues	2017	95.2%	98.6%	86.4%
Mexico	San Juan De Los Lagos	2000	89.6%	96.9%	75.8%
Mexico	San Juan De Los Lagos	2017	98.6%	99.8%	95.1%
Mexico	San Juan De Sabinas	2000	97.7%	99.4%	94.1%
Mexico	San Juan De Sabinas	2017	99.8%	100.0%	99.5%
Mexico	San Juan Del Estado	2000	64.4%	97.4%	16.9%
Mexico	San Juan Del Estado	2017	92.1%	99.9%	55.9%
Mexico	San Juan Del Rio	2000	90.7%	96.8%	79.9%
Mexico	San Juan Del Rio	2000	76.7%	87.3%	60.3%
Mexico	San Juan Del Rio	2000	84.1%	99.9%	14.7%

Country	Second Administrative Unit	Year	Mean	Upper	Lower
Mexico	San Juan Del Rio	2017	95.3%	98.8%	84.3%
Mexico	San Juan Del Rio	2017	96.7%	100.0%	70.3%
Mexico	San Juan Del Rio	2017	98.9%	99.8%	95.8%
Mexico	San Juan Diuxi	2000	75.2%	99.8%	13.4%
Mexico	San Juan Diuxi	2017	94.0%	100.0%	58.2%
Mexico	San Juan Evangelista	2000	72.2%	93.6%	38.2%
Mexico	San Juan Evangelista	2017	92.5%	99.3%	74.1%
Mexico	San Juan Evangelista Analco	2000	76.0%	99.8%	10.7%
Mexico	San Juan Evangelista Analco	2017	95.4%	100.0%	65.1%
Mexico	San Juan Guelavia	2000	79.0%	99.9%	11.1%
Mexico	San Juan Guelavia	2017	94.4%	100.0%	56.2%
Mexico	San Juan Guichicovi	2000	73.7%	89.3%	50.5%
Mexico	San Juan Guichicovi	2017	93.5%	98.4%	79.0%
Mexico	San Juan Huactzinco	2000	99.1%	99.7%	97.7%
Mexico	San Juan Huactzinco	2017	99.9%	100.0%	99.9%
Mexico	San Juan Ihualtepec	2000	78.5%	99.6%	12.4%
Mexico	San Juan Ihualtepec	2017	94.0%	100.0%	50.9%
Mexico	San Juan Juquila Mixes	2000	63.8%	95.0%	17.9%
Mexico	San Juan Juquila Mixes	2017	90.9%	99.6%	61.9%
Mexico	San Juan Juquila Vijanos	2000	66.0%	96.9%	17.8%
Mexico	San Juan Juquila Vijanos	2017	94.1%	99.8%	73.1%
Mexico	San Juan Lachao	2000	60.8%	95.0%	15.6%
Mexico	San Juan Lachao	2017	90.3%	99.0%	61.3%
Mexico	San Juan Lachigalla	2000	77.7%	99.8%	14.0%
Mexico	San Juan Lachigalla	2017	95.0%	100.0%	64.9%
Mexico	San Juan Lajarcia	2000	80.2%	99.8%	17.2%
Mexico	San Juan Lajarcia	2017	96.4%	100.0%	66.5%
Mexico	San Juan Lalana	2000	48.3%	70.0%	23.4%
Mexico	San Juan Lalana	2017	83.2%	93.4%	59.7%

Country	Second Administrative Unit	Year	Mean	Upper	Lower
Mexico	San Juan Mazatlan	2000	69.4%	89.2%	42.4%
Mexico	San Juan Mazatlan	2017	91.4%	98.7%	74.9%
Mexico	San Juan Mixtepec - Distr. 08 -	2000	81.4%	98.8%	37.2%
Mexico	San Juan Mixtepec - Distr. 08 -	2017	96.5%	99.9%	80.6%
Mexico	San Juan Mixtepec - Distr. 26 -	2000	80.2%	99.9%	5.4%
Mexico	San Juan Mixtepec - Distr. 26 -	2017	95.0%	100.0%	47.8%
Mexico	San Juan Numi	2000	77.7%	98.9%	34.0%
Mexico	San Juan Numi	2017	95.1%	99.9%	71.4%
Mexico	San Juan Ozolotepec	2000	77.3%	99.2%	26.2%
Mexico	San Juan Ozolotepec	2017	94.1%	99.9%	66.9%
Mexico	San Juan Petlapa	2000	62.2%	97.1%	10.7%
Mexico	San Juan Petlapa	2017	87.5%	99.7%	43.9%
Mexico	San Juan Quiahije	2000	77.3%	99.6%	11.2%
Mexico	San Juan Quiahije	2017	95.1%	100.0%	65.6%
Mexico	San Juan Quiotepec	2000	77.4%	99.5%	19.7%
Mexico	San Juan Quiotepec	2017	95.1%	100.0%	65.8%
Mexico	San Juan Sayultepec	2000	59.0%	88.5%	17.6%
Mexico	San Juan Sayultepec	2017	93.8%	99.0%	72.4%
Mexico	San Juan Tabaa	2000	68.1%	99.7%	6.2%
Mexico	San Juan Tabaa	2017	91.3%	100.0%	41.2%
Mexico	San Juan Tamazola	2000	73.1%	96.9%	34.2%
Mexico	San Juan Tamazola	2017	94.0%	99.7%	74.5%
Mexico	San Juan Teita	2000	72.0%	99.1%	12.1%
Mexico	San Juan Teita	2017	92.2%	100.0%	53.8%
Mexico	San Juan Teitipac	2000	73.1%	99.8%	6.0%
Mexico	San Juan Teitipac	2017	92.0%	100.0%	37.0%
Mexico	San Juan Tepeuxila	2000	76.4%	98.8%	33.9%
Mexico	San Juan Tepeuxila	2017	94.9%	99.9%	74.6%
Mexico	San Juan Teposcolula	2000	74.8%	99.5%	17.1%

Country	Second Administrative Unit	Year	Mean	Upper	Lower
Mexico	San Juan Teposcolula	2017	94.5%	100.0%	69.4%
Mexico	San Juan Yaee	2000	65.5%	96.8%	12.4%
Mexico	San Juan Yaee	2017	92.6%	99.7%	56.9%
Mexico	San Juan Yatzona	2000	68.2%	99.7%	6.5%
Mexico	San Juan Yatzona	2017	91.3%	100.0%	43.1%
Mexico	San Juan Yucuita	2000	65.6%	83.1%	41.6%
Mexico	San Juan Yucuita	2017	96.5%	98.7%	90.9%
Mexico	San Juanito De Escobedo	2000	85.0%	99.3%	37.6%
Mexico	San Juanito De Escobedo	2017	97.8%	100.0%	85.5%
Mexico	San Julian	2000	81.3%	99.8%	16.4%
Mexico	San Julian	2017	95.5%	100.0%	53.7%
Mexico	San Lorenzo	2000	69.5%	99.8%	10.2%
Mexico	San Lorenzo	2017	91.0%	100.0%	49.6%
Mexico	San Lorenzo Albarradas	2000	78.7%	99.6%	23.4%
Mexico	San Lorenzo Albarradas	2017	95.3%	100.0%	58.6%
Mexico	San Lorenzo Axocomanitla	2000	98.9%	99.6%	97.8%
Mexico	San Lorenzo Axocomanitla	2017	99.9%	100.0%	99.9%
Mexico	San Lorenzo Cacaotepec	2000	68.8%	75.3%	61.3%
Mexico	San Lorenzo Cacaotepec	2017	97.1%	97.7%	96.3%
Mexico	San Lorenzo Cuaunecuiltitla	2000	89.1%	99.2%	59.2%
Mexico	San Lorenzo Cuaunecuiltitla	2017	98.5%	100.0%	90.4%
Mexico	San Lorenzo Texmelucan	2000	68.5%	96.2%	15.2%
Mexico	San Lorenzo Texmelucan	2017	94.1%	99.4%	67.4%
Mexico	San Lorenzo Victoria	2000	71.3%	98.1%	16.6%
Mexico	San Lorenzo Victoria	2017	95.2%	99.8%	73.7%
Mexico	San Lucas	2000	72.6%	93.1%	39.9%
Mexico	San Lucas	2000	80.0%	97.6%	24.3%
Mexico	San Lucas	2017	94.8%	99.4%	79.4%
Mexico	San Lucas	2017	97.2%	99.9%	79.4%
Mexico	San Lucas Camotlan	2000	74.8%	99.5%	12.8%
Mexico	San Lucas Camotlan	2017	94.5%	100.0%	56.6%
Mexico	San Lucas Ojitlan	2000	52.7%	66.4%	37.1%
Mexico	San Lucas Ojitlan	2017	90.1%	94.8%	80.6%
Mexico	San Lucas Quiavini	2000	72.3%	96.5%	14.5%
Mexico	San Lucas Quiavini	2017	95.4%	100.0%	65.3%

Country	Second Administrative Unit	Year	Mean	Upper	Lower
Mexico	San Lucas Tecopilco	2000	99.3%	99.9%	96.0%
Mexico	San Lucas Tecopilco	2017	99.9%	100.0%	99.6%
Mexico	San Lucas Zoquiapam	2000	79.3%	82.9%	74.3%
Mexico	San Lucas Zoquiapam	2017	98.3%	98.7%	97.8%
Mexico	San Luis Acatlan	2000	68.6%	87.6%	43.1%
Mexico	San Luis Acatlan	2017	93.8%	98.6%	81.6%
Mexico	San Luis Amatlan	2000	77.2%	98.9%	35.3%
Mexico	San Luis Amatlan	2017	95.1%	99.9%	74.1%
Mexico	San Luis De La Paz	2000	92.0%	97.9%	76.4%
Mexico	San Luis De La Paz	2017	98.9%	99.8%	96.4%
Mexico	San Luis Del Cordero	2000	82.7%	99.8%	24.4%
Mexico	San Luis Del Cordero	2017	96.0%	100.0%	71.3%
Mexico	San Luis Potosi	2000	76.5%	81.0%	71.8%
Mexico	San Luis Potosi	2017	93.9%	97.0%	88.8%
Mexico	San Luis Rio Colorado	2000	85.8%	93.5%	75.0%
Mexico	San Luis Rio Colorado	2017	98.5%	99.6%	96.6%
Mexico	San Marcial Ozolotepec	2000	74.7%	99.7%	13.5%
Mexico	San Marcial Ozolotepec	2017	94.2%	100.0%	65.6%
Mexico	San Marcos	2000	72.5%	91.5%	44.7%
Mexico	San Marcos	2000	81.2%	99.5%	28.5%
Mexico	San Marcos	2017	92.8%	99.1%	74.4%
Mexico	San Marcos	2017	95.6%	100.0%	63.4%
Mexico	San Marcos Arteaga	2000	75.3%	97.7%	21.7%
Mexico	San Marcos Arteaga	2017	93.9%	99.8%	65.5%
Mexico	San Martin Chalchicuautla	2000	47.2%	63.1%	24.2%
Mexico	San Martin Chalchicuautla	2017	74.5%	87.7%	53.1%
Mexico	San Martin De Bolanos	2000	82.2%	99.5%	22.0%
Mexico	San Martin De Bolanos	2017	95.9%	100.0%	59.1%
Mexico	San Martin De Las Piramides	2000	96.4%	99.9%	73.1%
Mexico	San Martin De Las Piramides	2017	99.6%	100.0%	96.7%
Mexico	San Martin De Los Cansecos	2000	64.1%	99.8%	2.0%

Piped Water Proportion by Second Administrative Unit *(continued)*

Country	Second Administrative Unit	Year	Mean	Upper	Lower
Mexico	San Martin De Los Cansecos	2017	89.4%	100.0%	25.7%
Mexico	San Martin Hidalgo	2000	92.5%	98.8%	73.9%
Mexico	San Martin Hidalgo	2017	99.1%	99.9%	94.9%
Mexico	San Martin Huamelulpam	2000	76.7%	99.8%	12.4%
Mexico	San Martin Huamelulpam	2017	94.6%	100.0%	53.0%
Mexico	San Martin Itunyoso	2000	76.7%	99.7%	10.4%
Mexico	San Martin Itunyoso	2017	93.4%	100.0%	53.5%
Mexico	San Martin Lachila	2000	81.2%	99.9%	11.7%
Mexico	San Martin Lachila	2017	96.0%	100.0%	65.9%
Mexico	San Martin Peras	2000	77.4%	99.2%	20.3%
Mexico	San Martin Peras	2017	95.2%	99.9%	71.8%
Mexico	San Martin Texmelucan	2000	81.7%	84.2%	78.3%
Mexico	San Martin Texmelucan	2017	98.3%	98.7%	97.6%
Mexico	San Martin Tilcajete	2000	38.5%	96.8%	1.4%
Mexico	San Martin Tilcajete	2017	74.6%	99.8%	17.1%
Mexico	San Martin Totoltepec	2000	81.8%	100.0%	14.7%
Mexico	San Martin Totoltepec	2017	96.6%	100.0%	73.2%
Mexico	San Martin Toxpalan	2000	68.9%	89.1%	43.7%
Mexico	San Martin Toxpalan	2017	96.6%	99.2%	91.0%
Mexico	San Martin Zacatepec	2000	59.5%	82.3%	29.9%
Mexico	San Martin Zacatepec	2017	93.8%	98.8%	78.0%
Mexico	San Mateo Atenco	2000	49.6%	56.5%	43.4%
Mexico	San Mateo Atenco	2017	84.1%	91.3%	76.1%
Mexico	San Mateo Cajonos	2000	76.3%	99.8%	13.7%
Mexico	San Mateo Cajonos	2017	94.4%	100.0%	61.3%
Mexico	San Mateo Del Mar	2000	70.6%	97.7%	21.1%
Mexico	San Mateo Del Mar	2017	90.7%	99.8%	54.9%
Mexico	San Mateo Etlatongo	2000	64.8%	86.7%	38.4%
Mexico	San Mateo Etlatongo	2017	94.6%	98.7%	86.0%
Mexico	San Mateo Nejapam	2000	75.8%	99.5%	13.4%
Mexico	San Mateo Nejapam	2017	94.5%	100.0%	60.3%

Country	Second Administrative Unit	Year	Mean	Upper	Lower
Mexico	San Mateo Penasco	2000	77.4%	99.8%	11.1%
Mexico	San Mateo Penasco	2017	95.5%	100.0%	65.3%
Mexico	San Mateo Pinas	2000	73.6%	99.5%	17.7%
Mexico	San Mateo Pinas	2017	92.8%	100.0%	62.6%
Mexico	San Mateo Rio Hondo	2000	76.1%	98.0%	36.6%
Mexico	San Mateo Rio Hondo	2017	94.9%	99.9%	77.2%
Mexico	San Mateo Sindihui	2000	50.3%	83.0%	15.8%
Mexico	San Mateo Sindihui	2017	88.4%	96.1%	76.0%
Mexico	San Mateo Tlapiltepec	2000	73.7%	100.0%	4.8%
Mexico	San Mateo Tlapiltepec	2017	92.6%	100.0%	43.3%
Mexico	San Mateo Yoloxochitlan	2000	67.4%	74.1%	60.2%
Mexico	San Mateo Yoloxochitlan	2017	94.8%	96.1%	93.1%
Mexico	San Matias Tlalancaleca	2000	88.1%	95.9%	73.9%
Mexico	San Matias Tlalancaleca	2017	99.3%	99.8%	97.8%
Mexico	San Melchor Betaza	2000	75.3%	99.8%	6.0%
Mexico	San Melchor Betaza	2017	95.3%	100.0%	63.6%
Mexico	San Miguel Achiutla	2000	74.8%	99.6%	12.0%
Mexico	San Miguel Achiutla	2017	94.2%	100.0%	58.5%
Mexico	San Miguel Ahuehuetitlan	2000	77.3%	99.7%	17.9%
Mexico	San Miguel Ahuehuetitlan	2017	95.6%	100.0%	59.9%
Mexico	San Miguel Aloapam	2000	79.2%	99.9%	11.0%
Mexico	San Miguel Aloapam	2017	95.4%	100.0%	55.3%
Mexico	San Miguel Amatitlan	2000	70.6%	97.1%	17.3%
Mexico	San Miguel Amatitlan	2017	91.8%	99.8%	48.2%
Mexico	San Miguel Amatlan	2000	77.3%	99.2%	28.1%
Mexico	San Miguel Amatlan	2017	94.9%	99.9%	64.6%
Mexico	San Miguel Chicahua	2000	70.5%	99.7%	4.8%
Mexico	San Miguel Chicahua	2017	91.7%	100.0%	34.7%
Mexico	San Miguel Chimalapa	2000	76.8%	96.9%	30.1%
Mexico	San Miguel Chimalapa	2017	94.4%	99.7%	72.7%

Country	Second Administrative Unit	Year	Mean	Upper	Lower
Mexico	San Miguel Coatlan	2000	74.1%	99.6%	10.7%
Mexico	San Miguel Coatlan	2017	93.7%	100.0%	52.7%
Mexico	San Miguel De Allende	2000	83.8%	92.0%	75.3%
Mexico	San Miguel De Allende	2017	97.0%	99.3%	92.3%
Mexico	San Miguel De Horcasitas	2000	93.5%	99.4%	79.0%
Mexico	San Miguel De Horcasitas	2017	99.0%	100.0%	94.9%
Mexico	San Miguel Del Puerto	2000	66.9%	88.0%	36.9%
Mexico	San Miguel Del Puerto	2017	94.7%	99.2%	83.0%
Mexico	San Miguel Del Rio	2000	75.8%	99.7%	9.9%
Mexico	San Miguel Del Rio	2017	93.9%	100.0%	43.6%
Mexico	San Miguel Ejutla	2000	64.8%	98.2%	10.6%
Mexico	San Miguel Ejutla	2017	93.7%	99.9%	57.8%
Mexico	San Miguel El Alto	2000	83.5%	99.1%	28.4%
Mexico	San Miguel El Alto	2017	96.6%	99.9%	83.7%
Mexico	San Miguel El Grande	2000	68.4%	95.2%	20.8%
Mexico	San Miguel El Grande	2017	93.8%	99.7%	70.2%
Mexico	San Miguel Huautla	2000	73.5%	99.9%	9.4%
Mexico	San Miguel Huautla	2017	93.3%	100.0%	53.3%
Mexico	San Miguel Ixitlan	2000	78.5%	99.8%	19.3%
Mexico	San Miguel Ixitlan	2017	94.3%	100.0%	59.6%
Mexico	San Miguel Mixtepec	2000	80.2%	99.8%	28.0%
Mexico	San Miguel Mixtepec	2017	96.1%	100.0%	74.8%
Mexico	San Miguel Panixtlahuaca	2000	72.4%	99.1%	8.2%
Mexico	San Miguel Panixtlahuaca	2017	93.7%	99.8%	55.2%
Mexico	San Miguel Peras	2000	71.1%	99.4%	19.8%
Mexico	San Miguel Peras	2017	92.8%	100.0%	62.9%
Mexico	San Miguel Piedras	2000	75.1%	97.1%	24.2%
Mexico	San Miguel Piedras	2017	95.7%	99.8%	75.3%
Mexico	San Miguel Quetzaltepec	2000	70.3%	98.3%	16.9%
Mexico	San Miguel Quetzaltepec	2017	92.4%	99.9%	46.8%

Country	Second Administrative Unit	Year	Mean	Upper	Lower
Mexico	San Miguel Santa Flor	2000	79.6%	90.0%	65.7%
Mexico	San Miguel Santa Flor	2017	96.0%	99.0%	88.9%
Mexico	San Miguel Soyaltepec	2000	78.9%	93.9%	56.0%
Mexico	San Miguel Soyaltepec	2017	95.2%	99.5%	81.7%
Mexico	San Miguel Suchixtepec	2000	74.9%	99.9%	8.3%
Mexico	San Miguel Suchixtepec	2017	93.9%	100.0%	49.7%
Mexico	San Miguel Tecomatlan	2000	60.1%	97.0%	10.7%
Mexico	San Miguel Tecomatlan	2017	92.1%	99.8%	61.8%
Mexico	San Miguel Tenango	2000	73.0%	95.9%	35.5%
Mexico	San Miguel Tenango	2017	95.4%	99.7%	83.5%
Mexico	San Miguel Tequixtepec	2000	74.6%	99.3%	21.6%
Mexico	San Miguel Tequixtepec	2017	94.0%	99.9%	58.7%
Mexico	San Miguel Tilquiapam	2000	83.2%	93.3%	53.1%
Mexico	San Miguel Tilquiapam	2017	98.4%	99.5%	94.2%
Mexico	San Miguel Tlacamama	2000	52.0%	82.3%	19.8%
Mexico	San Miguel Tlacamama	2017	89.6%	97.8%	62.6%
Mexico	San Miguel Tlacotepec	2000	80.0%	87.6%	69.0%
Mexico	San Miguel Tlacotepec	2017	98.2%	99.3%	95.5%
Mexico	San Miguel Totolapan	2000	58.6%	83.1%	27.8%
Mexico	San Miguel Totolapan	2017	88.1%	96.2%	66.5%
Mexico	San Miguel Tulancingo	2000	68.4%	99.5%	12.5%
Mexico	San Miguel Tulancingo	2017	92.9%	100.0%	63.1%
Mexico	San Miguel Xoxtla	2000	85.8%	89.5%	80.9%
Mexico	San Miguel Xoxtla	2017	99.0%	99.3%	98.7%
Mexico	San Miguel Yotao	2000	61.7%	79.4%	36.6%
Mexico	San Miguel Yotao	2017	95.3%	97.9%	87.3%
Mexico	San Nicolas	2000	75.4%	99.7%	8.9%
Mexico	San Nicolas	2000	79.9%	98.7%	32.9%
Mexico	San Nicolas	2017	94.6%	100.0%	54.1%
Mexico	San Nicolas	2017	95.2%	99.9%	75.2%
Mexico	San Nicolas Buenos Aires	2000	76.9%	98.3%	36.2%
Mexico	San Nicolas Buenos Aires	2017	94.9%	99.8%	75.3%
Mexico	San Nicolas De Los Garza	2000	99.1%	99.4%	98.6%

Country	Second Administrative Unit	Year	Mean	Upper	Lower
Mexico	San Nicolas De Los Garza	2017	99.9%	100.0%	99.9%
Mexico	San Nicolas De Los Ranchos	2000	97.0%	99.8%	87.7%
Mexico	San Nicolas De Los Ranchos	2017	99.7%	100.0%	98.8%
Mexico	San Nicolas Hidalgo	2000	62.4%	99.8%	2.2%
Mexico	San Nicolas Hidalgo	2017	86.6%	100.0%	22.8%
Mexico	San Nicolas Tolentino	2000	81.3%	99.0%	44.6%
Mexico	San Nicolas Tolentino	2017	96.0%	99.9%	79.2%
Mexico	San Pablo Anicano	2000	48.3%	74.8%	22.4%
Mexico	San Pablo Anicano	2017	91.7%	97.3%	81.7%
Mexico	San Pablo Coatlan	2000	79.9%	99.4%	35.7%
Mexico	San Pablo Coatlan	2017	95.7%	100.0%	79.5%
Mexico	San Pablo Cuatro Venados	2000	55.9%	95.8%	11.6%
Mexico	San Pablo Cuatro Venados	2017	83.5%	99.1%	40.5%
Mexico	San Pablo Del Monte	2000	83.4%	88.4%	75.6%
Mexico	San Pablo Del Monte	2017	98.8%	99.2%	97.6%
Mexico	San Pablo Etla	2000	62.1%	69.2%	55.0%
Mexico	San Pablo Etla	2017	96.4%	97.3%	95.1%
Mexico	San Pablo Huitzo	2000	66.7%	76.9%	51.7%
Mexico	San Pablo Huitzo	2017	96.1%	98.7%	85.8%
Mexico	San Pablo Huixtepec	2000	77.2%	93.7%	54.7%
Mexico	San Pablo Huixtepec	2017	97.4%	99.6%	89.6%
Mexico	San Pablo Macuiltianguis	2000	79.2%	99.8%	13.4%
Mexico	San Pablo Macuiltianguis	2017	95.9%	100.0%	62.8%
Mexico	San Pablo Tijaltepec	2000	68.2%	97.9%	14.4%
Mexico	San Pablo Tijaltepec	2017	93.4%	99.9%	61.9%
Mexico	San Pablo Villa De Mitla	2000	71.8%	98.2%	19.3%
Mexico	San Pablo Villa De Mitla	2017	95.5%	99.9%	74.4%
Mexico	San Pablo Yaganiza	2000	76.7%	99.9%	7.6%
Mexico	San Pablo Yaganiza	2017	94.9%	100.0%	57.5%

Country	Second Administrative Unit	Year	Mean	Upper	Lower
Mexico	San Pedro	2000	82.2%	90.2%	70.5%
Mexico	San Pedro	2017	97.3%	99.0%	94.2%
Mexico	San Pedro Amuzgos	2000	78.5%	99.9%	7.1%
Mexico	San Pedro Amuzgos	2017	96.1%	100.0%	60.9%
Mexico	San Pedro Apostol	2000	18.1%	64.9%	1.5%
Mexico	San Pedro Apostol	2017	62.1%	96.2%	12.2%
Mexico	San Pedro Atoyac	2000	70.0%	99.7%	5.5%
Mexico	San Pedro Atoyac	2017	90.7%	100.0%	34.1%
Mexico	San Pedro Cajonos	2000	76.8%	99.9%	6.0%
Mexico	San Pedro Cajonos	2017	95.0%	100.0%	57.3%
Mexico	San Pedro Cholula	2000	46.0%	51.8%	38.9%
Mexico	San Pedro Cholula	2017	93.3%	94.5%	91.6%
Mexico	San Pedro Comitancillo	2000	78.9%	100.0%	7.1%
Mexico	San Pedro Comitancillo	2017	95.2%	100.0%	54.4%
Mexico	San Pedro Coxcaltepec Cantaros	2000	71.9%	98.0%	11.7%
Mexico	San Pedro Coxcaltepec Cantaros	2017	91.3%	99.9%	40.0%
Mexico	San Pedro De La Cueva	2000	87.0%	99.4%	40.1%
Mexico	San Pedro De La Cueva	2017	97.8%	99.9%	83.3%
Mexico	San Pedro Del Gallo	2000	81.3%	98.0%	47.0%
Mexico	San Pedro Del Gallo	2017	95.3%	99.8%	73.2%
Mexico	San Pedro El Alto	2000	75.4%	99.8%	10.5%
Mexico	San Pedro El Alto	2017	94.4%	100.0%	58.9%
Mexico	San Pedro Garza Garcia	2000	99.4%	99.7%	98.6%
Mexico	San Pedro Garza Garcia	2017	100.0%	100.0%	99.9%
Mexico	San Pedro Huamelula	2000	77.4%	98.5%	37.7%
Mexico	San Pedro Huamelula	2017	95.0%	99.9%	76.2%
Mexico	San Pedro Huilotepec	2000	64.8%	97.9%	4.6%
Mexico	San Pedro Huilotepec	2017	92.6%	99.8%	45.6%
Mexico	San Pedro Ixcatlan	2000	33.7%	55.8%	14.4%
Mexico	San Pedro Ixcatlan	2017	82.0%	90.6%	61.3%
Mexico	San Pedro Ixtlahuaca	2000	61.9%	85.0%	29.7%

Country	Second Administrative Unit	Year	Mean	Upper	Lower
Mexico	San Pedro Ixtlahuaca	2017	96.1%	99.3%	82.4%
Mexico	San Pedro Jaltepetongo	2000	66.7%	93.4%	15.3%
Mexico	San Pedro Jaltepetongo	2017	90.7%	99.7%	37.7%
Mexico	San Pedro Jicayan	2000	65.1%	95.6%	15.7%
Mexico	San Pedro Jicayan	2017	93.0%	99.7%	62.8%
Mexico	San Pedro Jocotipac	2000	73.8%	99.8%	15.0%
Mexico	San Pedro Jocotipac	2017	94.6%	100.0%	69.6%
Mexico	San Pedro Juchatengo	2000	96.0%	100.0%	80.4%
Mexico	San Pedro Juchatengo	2017	99.3%	100.0%	96.9%
Mexico	San Pedro Lagunillas	2000	88.1%	98.9%	61.1%
Mexico	San Pedro Lagunillas	2017	97.4%	99.9%	87.0%
Mexico	San Pedro Martir	2000	9.4%	38.4%	0.3%
Mexico	San Pedro Martir	2017	41.2%	85.9%	5.1%
Mexico	San Pedro Martir Quiechapa	2000	77.4%	99.8%	20.5%
Mexico	San Pedro Martir Quiechapa	2017	94.9%	100.0%	68.6%
Mexico	San Pedro Martir Yucuxaco	2000	78.1%	99.7%	15.8%
Mexico	San Pedro Martir Yucuxaco	2017	95.9%	100.0%	69.8%
Mexico	San Pedro Mixtepec - Distr. 22 -	2000	76.2%	92.8%	42.6%
Mexico	San Pedro Mixtepec - Distr. 22 -	2017	96.1%	99.4%	85.5%
Mexico	San Pedro Mixtepec - Distr. 26 -	2000	82.9%	99.7%	17.3%
Mexico	San Pedro Mixtepec - Distr. 26 -	2017	96.4%	100.0%	75.2%
Mexico	San Pedro Molinos	2000	76.7%	99.8%	11.4%
Mexico	San Pedro Molinos	2017	95.1%	100.0%	62.6%
Mexico	San Pedro Nopala	2000	72.1%	99.8%	5.2%
Mexico	San Pedro Nopala	2017	92.1%	100.0%	42.3%
Mexico	San Pedro Ocopetatillo	2000	93.0%	99.6%	72.0%
Mexico	San Pedro Ocopetatillo	2017	99.5%	100.0%	97.7%

Country	Second Administrative Unit	Year	Mean	Upper	Lower
Mexico	San Pedro Ocotepec	2000	66.4%	99.7%	7.7%
Mexico	San Pedro Ocotepec	2017	90.6%	100.0%	47.8%
Mexico	San Pedro Pochutla	2000	64.9%	90.2%	31.5%
Mexico	San Pedro Pochutla	2017	93.0%	98.8%	75.4%
Mexico	San Pedro Quiatoni	2000	78.7%	94.4%	49.7%
Mexico	San Pedro Quiatoni	2017	95.9%	99.7%	81.6%
Mexico	San Pedro Sochiapam	2000	65.7%	97.2%	15.9%
Mexico	San Pedro Sochiapam	2017	91.8%	99.8%	59.9%
Mexico	San Pedro Tapanatepec	2000	72.9%	91.8%	41.4%
Mexico	San Pedro Tapanatepec	2017	95.2%	99.4%	80.5%
Mexico	San Pedro Taviche	2000	74.1%	99.9%	9.9%
Mexico	San Pedro Taviche	2017	92.7%	100.0%	49.5%
Mexico	San Pedro Teozacoalco	2000	69.7%	98.0%	11.2%
Mexico	San Pedro Teozacoalco	2017	93.8%	99.9%	57.0%
Mexico	San Pedro Teutila	2000	58.3%	93.0%	11.7%
Mexico	San Pedro Teutila	2017	86.0%	99.5%	40.5%
Mexico	San Pedro Tidaa	2000	70.9%	99.7%	8.1%
Mexico	San Pedro Tidaa	2017	93.3%	100.0%	59.7%
Mexico	San Pedro Topiltepec	2000	68.4%	99.4%	3.5%
Mexico	San Pedro Topiltepec	2017	91.5%	100.0%	31.7%
Mexico	San Pedro Totolapa	2000	81.8%	99.6%	31.6%
Mexico	San Pedro Totolapa	2017	96.1%	100.0%	74.7%
Mexico	San Pedro Y San Pablo Ayutla	2000	73.4%	98.4%	23.5%
Mexico	San Pedro Y San Pablo Ayutla	2017	93.9%	99.9%	69.8%
Mexico	San Pedro Y San Pablo Teposcolula	2000	78.1%	99.7%	10.6%
Mexico	San Pedro Y San Pablo Teposcolula	2017	95.0%	100.0%	56.9%
Mexico	San Pedro Y San Pablo Tequixtepec	2000	79.5%	99.2%	39.8%
Mexico	San Pedro Y San Pablo Tequixtepec	2017	95.6%	99.9%	76.1%

Country	Second Administrative Unit	Year	Mean	Upper	Lower
Mexico	San Pedro Yaneri	2000	68.9%	98.1%	18.5%
Mexico	San Pedro Yaneri	2017	95.0%	99.9%	73.3%
Mexico	San Pedro Yeloixtlahuaca	2000	66.8%	95.1%	17.1%
Mexico	San Pedro Yeloixtlahuaca	2017	93.7%	99.7%	70.2%
Mexico	San Pedro Yolox	2000	75.2%	98.7%	23.9%
Mexico	San Pedro Yolox	2017	93.8%	99.9%	67.8%
Mexico	San Pedro Yucunama	2000	73.9%	99.9%	5.2%
Mexico	San Pedro Yucunama	2017	92.6%	100.0%	39.3%
Mexico	San Rafael	2000	60.2%	92.1%	16.9%
Mexico	San Rafael	2017	91.2%	99.3%	61.9%
Mexico	San Raymundo Jalpan	2000	72.9%	88.2%	55.0%
Mexico	San Raymundo Jalpan	2017	97.5%	99.1%	94.9%
Mexico	San Salvador	2000	81.8%	97.9%	50.6%
Mexico	San Salvador	2017	96.9%	99.8%	84.7%
Mexico	San Salvador El Seco	2000	82.0%	98.9%	37.8%
Mexico	San Salvador El Seco	2017	97.0%	99.9%	82.7%
Mexico	San Salvador El Verde	2000	94.5%	98.3%	85.1%
Mexico	San Salvador El Verde	2017	99.6%	99.9%	99.0%
Mexico	San Salvador Huixcolotla	2000	97.0%	99.9%	85.5%
Mexico	San Salvador Huixcolotla	2017	99.8%	100.0%	99.1%
Mexico	San Sebastian Abasolo	2000	66.1%	99.4%	5.9%
Mexico	San Sebastian Abasolo	2017	90.5%	99.9%	38.3%
Mexico	San Sebastian Coatlan	2000	80.4%	99.3%	41.4%
Mexico	San Sebastian Coatlan	2017	96.1%	100.0%	80.8%
Mexico	San Sebastian Del Oeste	2000	79.1%	97.4%	47.5%
Mexico	San Sebastian Del Oeste	2017	94.4%	99.8%	69.3%
Mexico	San Sebastian Ixcapa	2000	72.4%	99.3%	6.3%
Mexico	San Sebastian Ixcapa	2017	93.1%	100.0%	44.8%
Mexico	San Sebastian Nicananduta	2000	76.7%	99.9%	5.5%
Mexico	San Sebastian Nicananduta	2017	94.0%	100.0%	46.3%
Mexico	San Sebastian Rio Hondo	2000	83.9%	98.4%	42.0%
Mexico	San Sebastian Rio Hondo	2017	97.8%	99.9%	87.0%

Country	Second Administrative Unit	Year	Mean	Upper	Lower
Mexico	San Sebastian Tecomaxtlahuaca	2000	79.6%	95.4%	58.2%
Mexico	San Sebastian Tecomaxtlahuaca	2017	96.3%	99.7%	82.4%
Mexico	San Sebastian Teitipac	2000	69.9%	99.7%	3.2%
Mexico	San Sebastian Teitipac	2017	90.6%	100.0%	30.7%
Mexico	San Sebastian Tlacotepec	2000	71.5%	95.9%	34.2%
Mexico	San Sebastian Tlacotepec	2017	93.2%	99.7%	70.4%
Mexico	San Sebastian Tutla	2000	77.7%	82.3%	72.4%
Mexico	San Sebastian Tutla	2017	98.2%	98.7%	97.6%
Mexico	San Simon Almolongas	2000	76.0%	98.9%	22.5%
Mexico	San Simon Almolongas	2017	95.4%	99.9%	68.3%
Mexico	San Simon De Guerrero	2000	74.3%	99.4%	14.7%
Mexico	San Simon De Guerrero	2017	93.3%	100.0%	58.3%
Mexico	San Simon Zahuatlan	2000	67.1%	98.7%	9.9%
Mexico	San Simon Zahuatlan	2017	91.2%	99.9%	50.5%
Mexico	San Vicente Coatlan	2000	80.6%	99.9%	8.9%
Mexico	San Vicente Coatlan	2017	95.4%	100.0%	57.5%
Mexico	San Vicente Lachixio	2000	73.7%	97.5%	15.7%
Mexico	San Vicente Lachixio	2017	93.4%	99.8%	59.4%
Mexico	San Vicente Nunu	2000	75.3%	99.2%	26.8%
Mexico	San Vicente Nunu	2017	94.0%	99.9%	62.9%
Mexico	San Vicente Tancuayalab	2000	76.9%	98.5%	26.2%
Mexico	San Vicente Tancuayalab	2017	94.7%	99.9%	68.4%
Mexico	Sanahcat	2000	90.2%	99.6%	55.6%
Mexico	Sanahcat	2017	99.1%	100.0%	95.4%
Mexico	Sanctorum De Lazaro Cardenas	2000	98.3%	99.7%	94.3%
Mexico	Sanctorum De Lazaro Cardenas	2017	99.8%	100.0%	99.0%
Mexico	Santa Ana	2000	94.6%	98.5%	85.0%
Mexico	Santa Ana	2000	75.6%	96.6%	39.2%
Mexico	Santa Ana	2017	99.4%	99.9%	97.0%
Mexico	Santa Ana	2017	97.0%	99.7%	88.5%
Mexico	Santa Ana Ateixtlahuaca	2000	82.9%	99.5%	40.8%
Mexico	Santa Ana Ateixtlahuaca	2017	96.1%	100.0%	71.8%

Country	Second Administrative Unit	Year	Mean	Upper	Lower
Mexico	Santa Ana Cuauhtemoc	2000	51.1%	90.9%	7.3%
Mexico	Santa Ana Cuauhtemoc	2017	88.0%	99.0%	48.8%
Mexico	Santa Ana Del Valle	2000	91.2%	98.6%	69.7%
Mexico	Santa Ana Del Valle	2017	99.3%	99.9%	96.3%
Mexico	Santa Ana Maya	2000	81.0%	99.8%	25.0%
Mexico	Santa Ana Maya	2017	95.9%	100.0%	66.3%
Mexico	Santa Ana Nopalucan	2000	87.3%	90.5%	83.3%
Mexico	Santa Ana Nopalucan	2017	99.0%	99.3%	98.6%
Mexico	Santa Ana Tavela	2000	66.4%	90.8%	33.4%
Mexico	Santa Ana Tavela	2017	96.3%	99.3%	88.8%
Mexico	Santa Ana Tlapacoyan	2000	81.8%	96.9%	49.6%
Mexico	Santa Ana Tlapacoyan	2017	98.0%	99.8%	91.1%
Mexico	Santa Ana Yareni	2000	73.1%	99.9%	7.9%
Mexico	Santa Ana Yareni	2017	93.1%	100.0%	52.0%
Mexico	Santa Ana Zegache	2000	58.5%	84.9%	25.9%
Mexico	Santa Ana Zegache	2017	91.5%	99.0%	71.5%
Mexico	Santa Apolonia Teacalco	2000	95.5%	97.1%	93.3%
Mexico	Santa Apolonia Teacalco	2017	99.7%	99.8%	99.6%
Mexico	Santa Barbara	2000	82.2%	96.7%	47.4%
Mexico	Santa Barbara	2017	98.0%	99.8%	91.9%
Mexico	Santa Catalina Quieri	2000	78.4%	99.8%	14.1%
Mexico	Santa Catalina Quieri	2017	93.9%	100.0%	56.3%
Mexico	Santa Catarina	2000	73.6%	94.3%	38.7%
Mexico	Santa Catarina	2000	80.1%	99.7%	19.7%
Mexico	Santa Catarina	2000	99.0%	99.7%	96.2%
Mexico	Santa Catarina	2017	95.6%	100.0%	67.5%
Mexico	Santa Catarina	2017	93.4%	99.6%	71.3%
Mexico	Santa Catarina	2017	99.9%	100.0%	99.7%
Mexico	Santa Catarina Ayometla	2000	98.1%	99.4%	95.5%
Mexico	Santa Catarina Ayometla	2017	99.9%	100.0%	99.7%
Mexico	Santa Catarina Cuixtla	2000	64.3%	88.8%	31.4%

Country	Second Administrative Unit	Year	Mean	Upper	Lower
Mexico	Santa Catarina Cuixtla	2017	95.4%	99.2%	84.5%
Mexico	Santa Catarina Ixtepeji	2000	73.8%	98.8%	26.4%
Mexico	Santa Catarina Ixtepeji	2017	93.4%	99.9%	68.1%
Mexico	Santa Catarina Juquila	2000	70.6%	95.9%	26.8%
Mexico	Santa Catarina Juquila	2017	93.3%	99.7%	62.5%
Mexico	Santa Catarina Lachatao	2000	76.4%	99.0%	35.1%
Mexico	Santa Catarina Lachatao	2017	94.2%	99.9%	68.2%
Mexico	Santa Catarina Loxicha	2000	64.4%	88.7%	20.5%
Mexico	Santa Catarina Loxicha	2017	93.7%	99.1%	74.0%
Mexico	Santa Catarina Mechoacan	2000	59.4%	91.9%	20.5%
Mexico	Santa Catarina Mechoacan	2017	90.7%	99.9%	40.0%
Mexico	Santa Catarina Minas	2000	57.3%	78.7%	40.9%
Mexico	Santa Catarina Minas	2017	88.1%	98.5%	68.9%
Mexico	Santa Catarina Quiane	2000	60.2%	99.6%	3.8%
Mexico	Santa Catarina Quiane	2017	89.3%	100.0%	32.8%
Mexico	Santa Catarina Quioquitani	2000	81.1%	100.0%	9.8%
Mexico	Santa Catarina Quioquitani	2017	94.0%	100.0%	41.2%
Mexico	Santa Catarina Tayata	2000	74.8%	99.4%	12.4%
Mexico	Santa Catarina Tayata	2017	94.0%	100.0%	52.8%
Mexico	Santa Catarina Ticua	2000	73.3%	99.7%	7.4%
Mexico	Santa Catarina Ticua	2017	94.6%	100.0%	57.4%
Mexico	Santa Catarina Tlaltempan	2000	80.1%	99.7%	13.4%
Mexico	Santa Catarina Tlaltempan	2017	95.2%	100.0%	57.7%
Mexico	Santa Catarina Yosonotu	2000	72.4%	99.5%	8.7%
Mexico	Santa Catarina Yosonotu	2017	93.4%	100.0%	50.9%
Mexico	Santa Catarina Zapoquila	2000	80.7%	99.7%	14.6%
Mexico	Santa Catarina Zapoquila	2017	96.3%	100.0%	67.5%

Country	Second Administrative Unit	Year	Mean	Upper	Lower
Mexico	Santa Clara	2000	91.1%	98.2%	78.7%
Mexico	Santa Clara	2017	98.8%	99.8%	95.4%
Mexico	Santa Cruz	2000	86.5%	99.6%	43.8%
Mexico	Santa Cruz	2017	97.1%	100.0%	77.4%
Mexico	Santa Cruz Acatepec	2000	78.2%	83.5%	71.0%
Mexico	Santa Cruz Acatepec	2017	96.6%	97.5%	95.5%
Mexico	Santa Cruz Amilpas	2000	78.1%	82.7%	72.9%
Mexico	Santa Cruz Amilpas	2017	98.3%	98.7%	97.8%
Mexico	Santa Cruz De Bravo	2000	77.8%	99.8%	11.3%
Mexico	Santa Cruz De Bravo	2017	93.3%	100.0%	48.8%
Mexico	Santa Cruz De Juventino Rosas	2000	85.5%	95.7%	70.1%
Mexico	Santa Cruz De Juventino Rosas	2017	97.1%	99.6%	90.2%
Mexico	Santa Cruz Itundujia	2000	52.0%	79.0%	21.5%
Mexico	Santa Cruz Itundujia	2017	84.5%	97.5%	59.2%
Mexico	Santa Cruz Mixtepec	2000	80.0%	98.1%	39.2%
Mexico	Santa Cruz Mixtepec	2017	96.3%	99.9%	79.2%
Mexico	Santa Cruz Nundaco	2000	80.0%	90.5%	67.9%
Mexico	Santa Cruz Nundaco	2017	98.1%	99.3%	94.8%
Mexico	Santa Cruz Papalutla	2000	73.0%	99.9%	4.3%
Mexico	Santa Cruz Papalutla	2017	91.6%	100.0%	31.9%
Mexico	Santa Cruz Quilehtla	2000	99.2%	99.8%	98.0%
Mexico	Santa Cruz Quilehtla	2017	99.9%	100.0%	99.9%
Mexico	Santa Cruz Tacache De Mina	2000	66.8%	99.5%	6.2%
Mexico	Santa Cruz Tacache De Mina	2017	91.9%	100.0%	47.3%
Mexico	Santa Cruz Tacahua	2000	74.7%	99.5%	9.3%
Mexico	Santa Cruz Tacahua	2017	93.2%	100.0%	41.6%
Mexico	Santa Cruz Tayata	2000	75.6%	99.9%	6.0%
Mexico	Santa Cruz Tayata	2017	93.5%	100.0%	46.7%
Mexico	Santa Cruz Tlaxcala	2000	95.6%	97.5%	93.4%
Mexico	Santa Cruz Tlaxcala	2017	99.6%	99.8%	99.4%
Mexico	Santa Cruz Xitla	2000	71.5%	90.1%	41.0%

Country	Second Administrative Unit	Year	Mean	Upper	Lower
Mexico	Santa Cruz Xitla	2017	96.2%	99.3%	87.4%
Mexico	Santa Cruz Xoxocotlan	2000	61.2%	65.2%	57.6%
Mexico	Santa Cruz Xoxocotlan	2017	96.0%	96.6%	95.2%
Mexico	Santa Cruz Zenzontepec	2000	75.1%	97.6%	30.8%
Mexico	Santa Cruz Zenzontepec	2017	94.7%	99.7%	75.5%
Mexico	Santa Elena	2000	75.3%	99.3%	18.4%
Mexico	Santa Elena	2017	94.7%	99.9%	64.9%
Mexico	Santa Gertrudis	2000	78.1%	91.1%	55.0%
Mexico	Santa Gertrudis	2017	97.6%	99.0%	92.8%
Mexico	Santa Ines Ahuatempan	2000	79.1%	99.7%	14.0%
Mexico	Santa Ines Ahuatempan	2017	95.4%	100.0%	60.7%
Mexico	Santa Ines De Zaragoza	2000	77.0%	99.8%	10.3%
Mexico	Santa Ines De Zaragoza	2017	92.9%	100.0%	44.4%
Mexico	Santa Ines Del Monte	2000	7.8%	22.1%	1.5%
Mexico	Santa Ines Del Monte	2017	40.6%	65.6%	17.2%
Mexico	Santa Ines Yatzeche	2000	78.6%	93.5%	59.9%
Mexico	Santa Ines Yatzeche	2017	98.3%	99.6%	96.0%
Mexico	Santa Isabel	2000	84.7%	98.3%	48.5%
Mexico	Santa Isabel	2017	97.8%	99.9%	88.5%
Mexico	Santa Isabel Cholula	2000	55.4%	64.6%	43.9%
Mexico	Santa Isabel Cholula	2017	93.7%	95.5%	90.9%
Mexico	Santa Isabel Xiloxoxtla	2000	99.5%	99.8%	99.0%
Mexico	Santa Isabel Xiloxoxtla	2017	100.0%	100.0%	99.9%
Mexico	Santa Lucia Del Camino	2000	78.5%	82.7%	74.0%
Mexico	Santa Lucia Del Camino	2017	98.4%	98.7%	97.9%
Mexico	Santa Lucia Miahuatlan	2000	75.0%	99.7%	9.3%
Mexico	Santa Lucia Miahuatlan	2017	93.8%	100.0%	54.0%
Mexico	Santa Lucia Monteverde	2000	72.3%	99.3%	11.0%
Mexico	Santa Lucia Monteverde	2017	91.8%	99.9%	55.3%
Mexico	Santa Lucia Ocotlan	2000	19.8%	74.9%	2.2%
Mexico	Santa Lucia Ocotlan	2017	60.1%	98.0%	17.1%
Mexico	Santa Magdalena Jicotlan	2000	73.3%	99.9%	8.3%

Country	Second Administrative Unit	Year	Mean	Upper	Lower
Mexico	Santa Magdalena Jicotlan	2017	91.7%	100.0%	41.9%
Mexico	Santa Maria Alotepec	2000	69.6%	99.0%	14.8%
Mexico	Santa Maria Alotepec	2017	91.5%	99.9%	55.0%
Mexico	Santa Maria Apazco	2000	66.7%	86.9%	40.5%
Mexico	Santa Maria Apazco	2017	95.3%	99.0%	87.1%
Mexico	Santa Maria Atzompa	2000	68.1%	76.0%	58.8%
Mexico	Santa Maria Atzompa	2017	97.3%	98.2%	95.7%
Mexico	Santa Maria Camotlan	2000	96.6%	99.8%	83.4%
Mexico	Santa Maria Camotlan	2017	99.7%	100.0%	98.6%
Mexico	Santa Maria Chachoapam	2000	68.3%	85.4%	44.3%
Mexico	Santa Maria Chachoapam	2017	95.9%	98.9%	86.7%
Mexico	Santa Maria Chilchotla	2000	56.2%	96.3%	14.9%
Mexico	Santa Maria Chilchotla	2017	85.3%	99.7%	49.5%
Mexico	Santa Maria Chimalapa	2000	66.0%	86.2%	41.1%
Mexico	Santa Maria Chimalapa	2017	90.8%	97.8%	77.0%
Mexico	Santa Maria Colotepec	2000	78.6%	99.1%	27.3%
Mexico	Santa Maria Colotepec	2017	95.0%	99.9%	73.3%
Mexico	Santa Maria Cortijo	2000	72.9%	99.7%	7.8%
Mexico	Santa Maria Cortijo	2017	92.7%	100.0%	42.1%
Mexico	Santa Maria Coyotepec	2000	54.4%	73.8%	32.6%
Mexico	Santa Maria Coyotepec	2017	94.4%	97.8%	87.6%
Mexico	Santa Maria De La Paz	2000	79.2%	99.5%	24.6%
Mexico	Santa Maria De La Paz	2017	95.6%	100.0%	71.4%
Mexico	Santa Maria De Los Angeles	2000	77.6%	98.7%	27.2%
Mexico	Santa Maria De Los Angeles	2017	94.6%	99.9%	66.4%
Mexico	Santa Maria Del Oro	2000	80.3%	98.4%	43.0%
Mexico	Santa Maria Del Oro	2000	83.0%	94.7%	63.3%
Mexico	Santa Maria Del Oro	2017	95.0%	99.9%	78.6%
Mexico	Santa Maria Del Oro	2017	97.2%	99.6%	90.0%
Mexico	Santa Maria Del Rio	2000	76.7%	94.3%	52.0%

Country	Second Administrative Unit	Year	Mean	Upper	Lower
Mexico	Santa Maria Del Rio	2017	95.3%	99.5%	80.4%
Mexico	Santa Maria Del Rosario	2000	72.7%	99.6%	10.0%
Mexico	Santa Maria Del Rosario	2017	92.6%	100.0%	52.7%
Mexico	Santa Maria Del Tule	2000	73.8%	96.0%	38.1%
Mexico	Santa Maria Del Tule	2017	95.2%	99.8%	72.6%
Mexico	Santa Maria Ecatepec	2000	76.3%	96.8%	43.3%
Mexico	Santa Maria Ecatepec	2017	93.8%	99.7%	77.7%
Mexico	Santa Maria Guelace	2000	63.3%	98.7%	6.4%
Mexico	Santa Maria Guelace	2017	91.8%	99.9%	48.6%
Mexico	Santa Maria Guienagati	2000	69.7%	98.3%	19.3%
Mexico	Santa Maria Guienagati	2017	93.5%	99.8%	53.4%
Mexico	Santa Maria Huatulco	2000	74.6%	98.5%	31.7%
Mexico	Santa Maria Huatulco	2017	93.6%	99.9%	65.0%
Mexico	Santa Maria Huazolotitlan	2000	65.3%	97.2%	22.6%
Mexico	Santa Maria Huazolotitlan	2017	89.9%	99.8%	58.1%
Mexico	Santa Maria Ipalapa	2000	69.5%	99.5%	6.5%
Mexico	Santa Maria Ipalapa	2017	90.1%	100.0%	41.5%
Mexico	Santa Maria Ixcatlan	2000	72.2%	99.2%	20.0%
Mexico	Santa Maria Ixcatlan	2017	93.0%	99.9%	63.4%
Mexico	Santa Maria Jacatepec	2000	68.9%	89.9%	36.6%
Mexico	Santa Maria Jacatepec	2017	92.7%	99.0%	75.3%
Mexico	Santa Maria Jalapa Del Marques	2000	76.4%	99.2%	17.2%
Mexico	Santa Maria Jalapa Del Marques	2017	95.2%	99.9%	67.6%
Mexico	Santa Maria Jaltianguis	2000	75.2%	99.7%	19.5%
Mexico	Santa Maria Jaltianguis	2017	94.3%	100.0%	55.6%
Mexico	Santa Maria La Asuncion	2000	41.4%	76.8%	10.2%
Mexico	Santa Maria La Asuncion	2017	89.4%	98.3%	63.8%
Mexico	Santa Maria Lachixio	2000	77.0%	99.9%	7.4%
Mexico	Santa Maria Lachixio	2017	94.3%	100.0%	52.8%
Mexico	Santa Maria Mixtequilla	2000	77.7%	99.7%	18.9%

Country	Second Administrative Unit	Year	Mean	Upper	Lower
Mexico	Santa Maria Mixtequilla	2017	95.9%	100.0%	71.5%
Mexico	Santa Maria Nativitas	2000	73.5%	99.8%	11.1%
Mexico	Santa Maria Nativitas	2017	93.7%	100.0%	51.9%
Mexico	Santa Maria Nduayaco	2000	73.0%	99.8%	5.7%
Mexico	Santa Maria Nduayaco	2017	92.2%	100.0%	44.1%
Mexico	Santa Maria Ozolotepec	2000	78.4%	99.6%	30.0%
Mexico	Santa Maria Ozolotepec	2017	94.9%	100.0%	71.5%
Mexico	Santa Maria Papalo	2000	78.1%	98.4%	18.8%
Mexico	Santa Maria Papalo	2017	96.1%	99.8%	70.5%
Mexico	Santa Maria Penoles	2000	74.9%	97.7%	30.4%
Mexico	Santa Maria Penoles	2017	95.3%	99.7%	76.7%
Mexico	Santa Maria Petapa	2000	72.8%	81.0%	59.5%
Mexico	Santa Maria Petapa	2017	97.3%	98.7%	92.5%
Mexico	Santa Maria Quiegolani	2000	82.3%	99.8%	14.9%
Mexico	Santa Maria Quiegolani	2017	96.7%	100.0%	74.4%
Mexico	Santa Maria Sola	2000	41.5%	86.2%	3.4%
Mexico	Santa Maria Sola	2017	81.5%	98.3%	28.5%
Mexico	Santa Maria Tataltepec	2000	71.2%	100.0%	4.5%
Mexico	Santa Maria Tataltepec	2017	90.0%	100.0%	36.7%
Mexico	Santa Maria Tecomavaca	2000	70.9%	98.6%	19.2%
Mexico	Santa Maria Tecomavaca	2017	93.0%	100.0%	48.9%
Mexico	Santa Maria Temaxcalapa	2000	68.5%	99.7%	9.4%
Mexico	Santa Maria Temaxcalapa	2017	92.4%	100.0%	57.3%
Mexico	Santa Maria Temaxcaltepec	2000	68.0%	81.7%	47.6%
Mexico	Santa Maria Temaxcaltepec	2017	94.5%	96.2%	90.2%
Mexico	Santa Maria Teopoxco	2000	97.8%	99.6%	93.1%
Mexico	Santa Maria Teopoxco	2017	99.8%	100.0%	99.5%
Mexico	Santa Maria Tepantlali	2000	65.9%	98.5%	6.9%
Mexico	Santa Maria Tepantlali	2017	90.4%	99.9%	36.0%
Mexico	Santa Maria Texcatitlan	2000	71.7%	99.8%	9.7%

Country	Second Administrative Unit	Year	Mean	Upper	Lower
Mexico	Santa Maria Texcatitlan	2017	94.4%	100.0%	62.9%
Mexico	Santa Maria Tlahuitoltepec	2000	68.7%	97.7%	20.9%
Mexico	Santa Maria Tlahuitoltepec	2017	93.9%	99.7%	61.8%
Mexico	Santa Maria Tlalixtac	2000	48.0%	84.9%	10.0%
Mexico	Santa Maria Tlalixtac	2017	88.8%	98.8%	52.3%
Mexico	Santa Maria Tonameca	2000	59.6%	86.6%	29.2%
Mexico	Santa Maria Tonameca	2017	91.0%	98.8%	67.8%
Mexico	Santa Maria Totolapilla	2000	71.3%	98.4%	17.4%
Mexico	Santa Maria Totolapilla	2017	95.5%	99.9%	71.6%
Mexico	Santa Maria Xadani	2000	48.1%	97.6%	2.4%
Mexico	Santa Maria Xadani	2017	77.8%	99.8%	11.8%
Mexico	Santa Maria Yalina	2000	73.2%	93.0%	42.1%
Mexico	Santa Maria Yalina	2017	96.4%	99.5%	87.5%
Mexico	Santa Maria Yavesia	2000	74.0%	99.7%	8.7%
Mexico	Santa Maria Yavesia	2017	94.7%	100.0%	58.6%
Mexico	Santa Maria Yolotepec	2000	76.7%	99.7%	9.4%
Mexico	Santa Maria Yolotepec	2017	93.6%	100.0%	49.7%
Mexico	Santa Maria Yosoyua	2000	77.0%	99.8%	17.3%
Mexico	Santa Maria Yosoyua	2017	95.4%	100.0%	67.7%
Mexico	Santa Maria Yucuhiti	2000	70.9%	98.7%	16.2%
Mexico	Santa Maria Yucuhiti	2017	94.6%	99.9%	69.3%
Mexico	Santa Maria Zacatepec	2000	72.8%	95.6%	26.7%
Mexico	Santa Maria Zacatepec	2017	93.6%	99.7%	65.6%
Mexico	Santa Maria Zaniza	2000	79.8%	99.4%	23.1%
Mexico	Santa Maria Zaniza	2017	95.4%	100.0%	66.0%
Mexico	Santa Maria Zoquitlan	2000	80.5%	99.5%	27.6%
Mexico	Santa Maria Zoquitlan	2017	96.1%	100.0%	76.1%
Mexico	Santiago	2000	76.6%	84.9%	64.2%
Mexico	Santiago	2017	96.7%	98.7%	93.9%
Mexico	Santiago Amoltepec	2000	54.5%	74.9%	33.8%
Mexico	Santiago Amoltepec	2017	90.9%	97.5%	78.8%

Country	Second Administrative Unit	Year	Mean	Upper	Lower
Mexico	Santiago Apoala	2000	68.9%	97.7%	10.5%
Mexico	Santiago Apoala	2017	91.6%	99.8%	45.1%
Mexico	Santiago Apostol	2000	36.6%	60.8%	16.1%
Mexico	Santiago Apostol	2017	72.7%	89.4%	48.9%
Mexico	Santiago Astata	2000	80.3%	99.8%	12.1%
Mexico	Santiago Astata	2017	95.9%	100.0%	68.1%
Mexico	Santiago Atitlan	2000	65.7%	99.5%	11.6%
Mexico	Santiago Atitlan	2017	90.2%	100.0%	48.0%
Mexico	Santiago Ayuquililla	2000	75.9%	99.8%	7.1%
Mexico	Santiago Ayuquililla	2017	93.3%	100.0%	54.5%
Mexico	Santiago Cacaloxtepec	2000	81.6%	99.3%	28.6%
Mexico	Santiago Cacaloxtepec	2017	97.2%	100.0%	80.9%
Mexico	Santiago Camotlan	2000	62.1%	94.0%	19.5%
Mexico	Santiago Camotlan	2017	87.8%	99.6%	51.4%
Mexico	Santiago Chazumba	2000	79.7%	99.2%	28.6%
Mexico	Santiago Chazumba	2017	95.6%	99.9%	74.3%
Mexico	Santiago Choapam	2000	68.7%	95.7%	19.5%
Mexico	Santiago Choapam	2017	90.4%	99.6%	56.4%
Mexico	Santiago Comaltepec	2000	75.4%	97.9%	22.4%
Mexico	Santiago Comaltepec	2017	93.6%	99.8%	59.1%
Mexico	Santiago De Anaya	2000	75.3%	98.8%	18.1%
Mexico	Santiago De Anaya	2017	94.4%	99.9%	62.5%
Mexico	Santiago Del Rio	2000	74.2%	99.6%	11.6%
Mexico	Santiago Del Rio	2017	93.8%	100.0%	46.8%
Mexico	Santiago El Pinar	2000	68.6%	99.9%	5.5%
Mexico	Santiago El Pinar	2017	90.8%	100.0%	39.8%
Mexico	Santiago Huajolotitlan	2000	77.7%	91.1%	57.5%
Mexico	Santiago Huajolotitlan	2017	98.2%	99.4%	95.6%
Mexico	Santiago Huauclilla	2000	75.9%	99.2%	23.4%
Mexico	Santiago Huauclilla	2017	93.3%	100.0%	66.0%
Mexico	Santiago Ihuitlan Plumas	2000	76.9%	99.7%	10.4%

Country	Second Administrative Unit	Year	Mean	Upper	Lower
Mexico	Santiago Ihuitlan Plumas	2017	94.6%	100.0%	54.6%
Mexico	Santiago Ixcuintepec	2000	79.7%	99.8%	24.0%
Mexico	Santiago Ixcuintepec	2017	94.2%	100.0%	48.6%
Mexico	Santiago Ixcuintla	2000	76.7%	90.5%	57.9%
Mexico	Santiago Ixcuintla	2017	95.1%	99.2%	84.2%
Mexico	Santiago Ixtayutla	2000	39.9%	84.2%	14.7%
Mexico	Santiago Ixtayutla	2017	72.7%	98.1%	37.2%
Mexico	Santiago Jamiltepec	2000	66.2%	76.6%	49.6%
Mexico	Santiago Jamiltepec	2017	95.4%	98.0%	88.5%
Mexico	Santiago Jocotepec	2000	56.1%	82.8%	25.4%
Mexico	Santiago Jocotepec	2017	85.7%	97.1%	59.1%
Mexico	Santiago Juxtlahuaca	2000	66.0%	85.6%	39.8%
Mexico	Santiago Juxtlahuaca	2017	91.3%	98.4%	72.8%
Mexico	Santiago Lachiguiri	2000	83.0%	98.2%	51.4%
Mexico	Santiago Lachiguiri	2017	96.0%	99.8%	77.2%
Mexico	Santiago Lalopa	2000	66.7%	99.3%	11.0%
Mexico	Santiago Lalopa	2017	91.0%	100.0%	47.5%
Mexico	Santiago Laollaga	2000	85.6%	99.7%	41.5%
Mexico	Santiago Laollaga	2017	98.1%	100.0%	88.0%
Mexico	Santiago Laxopa	2000	74.4%	98.1%	26.3%
Mexico	Santiago Laxopa	2017	95.9%	99.9%	77.9%
Mexico	Santiago Llano Grande	2000	74.2%	99.6%	12.5%
Mexico	Santiago Llano Grande	2017	93.3%	100.0%	58.0%
Mexico	Santiago Maravatio	2000	88.3%	99.9%	46.9%
Mexico	Santiago Maravatio	2017	98.5%	100.0%	91.0%
Mexico	Santiago Matatlan	2000	70.0%	97.0%	20.9%
Mexico	Santiago Matatlan	2017	93.0%	99.9%	65.4%
Mexico	Santiago Miahuatlan	2000	77.4%	88.4%	61.8%
Mexico	Santiago Miahuatlan	2017	97.5%	99.1%	93.2%
Mexico	Santiago Miltepec	2000	81.3%	99.8%	32.0%
Mexico	Santiago Miltepec	2017	96.9%	100.0%	80.7%

Country	Second Administrative Unit	Year	Mean	Upper	Lower
Mexico	Santiago Minas	2000	82.7%	99.3%	37.6%
Mexico	Santiago Minas	2017	96.8%	100.0%	75.5%
Mexico	Santiago Nacaltepec	2000	74.6%	99.5%	18.7%
Mexico	Santiago Nacaltepec	2017	92.8%	100.0%	51.0%
Mexico	Santiago Nejapilla	2000	70.9%	99.8%	5.7%
Mexico	Santiago Nejapilla	2017	92.6%	100.0%	49.4%
Mexico	Santiago Niltepec	2000	78.4%	98.2%	27.6%
Mexico	Santiago Niltepec	2017	95.6%	99.9%	73.1%
Mexico	Santiago Nundiche	2000	67.7%	93.3%	20.2%
Mexico	Santiago Nundiche	2017	94.2%	99.2%	77.6%
Mexico	Santiago Nuyoo	2000	72.3%	99.5%	11.1%
Mexico	Santiago Nuyoo	2017	93.8%	100.0%	62.5%
Mexico	Santiago Papasquiaro	2000	79.9%	91.2%	59.6%
Mexico	Santiago Papasquiaro	2017	96.9%	99.0%	92.6%
Mexico	Santiago Pinotepa Nacional	2000	57.1%	82.4%	27.3%
Mexico	Santiago Pinotepa Nacional	2017	89.7%	97.5%	66.2%
Mexico	Santiago Sochiapa	2000	69.3%	96.2%	27.5%
Mexico	Santiago Sochiapa	2017	91.6%	99.7%	66.3%
Mexico	Santiago Suchilquitongo	2000	58.5%	66.4%	49.3%
Mexico	Santiago Suchilquitongo	2017	95.3%	96.8%	92.1%
Mexico	Santiago Tamazola	2000	73.3%	98.5%	28.8%
Mexico	Santiago Tamazola	2017	93.4%	99.9%	70.1%
Mexico	Santiago Tapextla	2000	77.0%	99.3%	20.9%
Mexico	Santiago Tapextla	2017	94.8%	100.0%	69.5%
Mexico	Santiago Tenango	2000	72.8%	99.6%	11.1%
Mexico	Santiago Tenango	2017	92.9%	100.0%	53.4%
Mexico	Santiago Tepetlapa	2000	75.7%	99.9%	5.2%
Mexico	Santiago Tepetlapa	2017	94.2%	100.0%	47.7%
Mexico	Santiago Tetepec	2000	73.2%	93.6%	37.7%
Mexico	Santiago Tetepec	2017	95.2%	99.4%	81.5%

Country	Second Administrative Unit	Year	Mean	Upper	Lower
Mexico	Santiago Texcalcingo	2000	89.7%	94.8%	80.1%
Mexico	Santiago Texcalcingo	2017	99.1%	99.6%	98.1%
Mexico	Santiago Textitlan	2000	80.2%	99.6%	18.6%
Mexico	Santiago Textitlan	2017	95.4%	100.0%	55.9%
Mexico	Santiago Tilantongo	2000	73.3%	99.6%	15.1%
Mexico	Santiago Tilantongo	2017	92.6%	99.9%	55.5%
Mexico	Santiago Tillo	2000	64.7%	96.1%	12.6%
Mexico	Santiago Tillo	2017	92.9%	99.7%	46.3%
Mexico	Santiago Tlazoyaltepec	2000	74.1%	99.5%	13.7%
Mexico	Santiago Tlazoyaltepec	2017	94.6%	100.0%	65.4%
Mexico	Santiago Tulantepec De Lugo Guerrero	2000	69.4%	78.3%	60.1%
Mexico	Santiago Tulantepec De Lugo Guerrero	2017	95.4%	97.7%	91.5%
Mexico	Santiago Tuxtla	2000	69.6%	96.4%	27.5%
Mexico	Santiago Tuxtla	2017	93.1%	99.7%	70.9%
Mexico	Santiago Xanica	2000	75.5%	99.6%	13.7%
Mexico	Santiago Xanica	2017	93.0%	100.0%	49.0%
Mexico	Santiago Xiacui	2000	73.2%	98.6%	13.4%
Mexico	Santiago Xiacui	2017	95.3%	99.9%	66.8%
Mexico	Santiago Yaitepec	2000	68.3%	99.3%	4.9%
Mexico	Santiago Yaitepec	2017	92.2%	99.7%	46.1%
Mexico	Santiago Yaveo	2000	73.5%	93.0%	48.9%
Mexico	Santiago Yaveo	2017	93.4%	99.3%	79.8%
Mexico	Santiago Yolomecatl	2000	74.5%	99.9%	7.1%
Mexico	Santiago Yolomecatl	2017	92.7%	100.0%	31.4%
Mexico	Santiago Yosondua	2000	74.2%	98.0%	24.6%
Mexico	Santiago Yosondua	2017	93.0%	99.9%	59.5%
Mexico	Santiago Yucuyachi	2000	75.1%	99.8%	15.0%
Mexico	Santiago Yucuyachi	2017	94.6%	100.0%	58.1%
Mexico	Santiago Zacatepec	2000	78.7%	93.4%	51.3%
Mexico	Santiago Zacatepec	2017	97.8%	99.4%	92.7%

Country	Second Administrative Unit	Year	Mean	Upper	Lower
Mexico	Santiago Zoochila	2000	77.4%	91.9%	49.8%
Mexico	Santiago Zoochila	2017	97.8%	99.5%	92.4%
Mexico	Santo Domingo	2000	79.1%	94.1%	56.1%
Mexico	Santo Domingo	2017	95.3%	99.2%	85.8%
Mexico	Santo Domingo Albarradas	2000	77.0%	99.6%	19.2%
Mexico	Santo Domingo Albarradas	2017	94.7%	100.0%	66.3%
Mexico	Santo Domingo Armenta	2000	73.0%	99.6%	6.9%
Mexico	Santo Domingo Armenta	2017	93.0%	100.0%	57.2%
Mexico	Santo Domingo Chihuitan	2000	78.6%	99.8%	11.4%
Mexico	Santo Domingo Chihuitan	2017	96.1%	100.0%	72.1%
Mexico	Santo Domingo De Morelos	2000	27.2%	55.6%	7.3%
Mexico	Santo Domingo De Morelos	2017	76.0%	89.5%	50.6%
Mexico	Santo Domingo Ingenio	2000	72.0%	98.5%	23.2%
Mexico	Santo Domingo Ingenio	2017	94.8%	99.9%	70.8%
Mexico	Santo Domingo Ixcatlan	2000	77.6%	99.9%	6.3%
Mexico	Santo Domingo Ixcatlan	2017	94.1%	100.0%	51.7%
Mexico	Santo Domingo Nuxaa	2000	77.4%	99.5%	27.8%
Mexico	Santo Domingo Nuxaa	2017	94.5%	100.0%	68.2%
Mexico	Santo Domingo Ozolotepec	2000	79.2%	99.8%	16.8%
Mexico	Santo Domingo Ozolotepec	2017	95.2%	100.0%	61.5%
Mexico	Santo Domingo Petapa	2000	76.5%	98.7%	17.9%
Mexico	Santo Domingo Petapa	2017	94.3%	99.9%	60.3%

Country	Second Administrative Unit	Year	Mean	Upper	Lower
Mexico	Santo Domingo Roayaga	2000	67.6%	97.9%	14.0%
Mexico	Santo Domingo Roayaga	2017	92.8%	99.9%	60.6%
Mexico	Santo Domingo Tehuantepec	2000	80.8%	95.6%	48.7%
Mexico	Santo Domingo Tehuantepec	2017	96.6%	99.5%	84.4%
Mexico	Santo Domingo Teojomulco	2000	77.5%	99.7%	27.5%
Mexico	Santo Domingo Teojomulco	2017	95.0%	100.0%	66.3%
Mexico	Santo Domingo Tepuxtepec	2000	74.5%	99.7%	10.2%
Mexico	Santo Domingo Tepuxtepec	2017	92.9%	100.0%	39.8%
Mexico	Santo Domingo Tlatayapam	2000	70.7%	99.8%	5.7%
Mexico	Santo Domingo Tlatayapam	2017	92.8%	100.0%	47.6%
Mexico	Santo Domingo Tomaltepec	2000	69.6%	78.8%	56.5%
Mexico	Santo Domingo Tomaltepec	2017	97.1%	98.2%	95.1%
Mexico	Santo Domingo Tonala	2000	72.7%	99.5%	10.3%
Mexico	Santo Domingo Tonala	2017	92.5%	100.0%	39.0%
Mexico	Santo Domingo Tonaltepec	2000	74.6%	99.8%	16.7%
Mexico	Santo Domingo Tonaltepec	2017	94.3%	100.0%	59.9%
Mexico	Santo Domingo Xagacia	2000	76.8%	99.8%	7.8%
Mexico	Santo Domingo Xagacia	2017	94.4%	100.0%	53.4%
Mexico	Santo Domingo Yanhuitlan	2000	74.2%	99.6%	6.0%
Mexico	Santo Domingo Yanhuitlan	2017	93.8%	100.0%	38.9%
Mexico	Santo Domingo Yodohino	2000	79.8%	99.8%	13.3%

Country	Second Administrative Unit	Year	Mean	Upper	Lower
Mexico	Santo Domingo Yodohino	2017	95.4%	100.0%	60.4%
Mexico	Santo Domingo Zanatepec	2000	69.5%	96.0%	19.9%
Mexico	Santo Domingo Zanatepec	2017	91.4%	99.7%	48.6%
Mexico	Santo Tomas	2000	81.0%	99.5%	28.2%
Mexico	Santo Tomas	2017	96.9%	100.0%	83.7%
Mexico	Santo Tomas Hueyotlipan	2000	77.8%	94.5%	40.1%
Mexico	Santo Tomas Hueyotlipan	2017	97.6%	99.6%	89.8%
Mexico	Santo Tomas Jalieza	2000	38.4%	94.3%	2.8%
Mexico	Santo Tomas Jalieza	2017	71.9%	99.6%	17.0%
Mexico	Santo Tomas Mazaltepec	2000	64.8%	87.2%	29.2%
Mexico	Santo Tomas Mazaltepec	2017	93.4%	98.2%	77.7%
Mexico	Santo Tomas Ocotepec	2000	73.4%	98.4%	24.8%
Mexico	Santo Tomas Ocotepec	2017	95.1%	99.9%	71.4%
Mexico	Santo Tomas Tamazulapan	2000	66.3%	97.0%	16.4%
Mexico	Santo Tomas Tamazulapan	2017	93.7%	99.8%	67.3%
Mexico	Santos Reyes Nopala	2000	82.0%	94.5%	60.3%
Mexico	Santos Reyes Nopala	2017	97.3%	99.5%	89.9%
Mexico	Santos Reyes Papalo	2000	83.8%	99.0%	34.3%
Mexico	Santos Reyes Papalo	2017	97.0%	99.9%	82.9%
Mexico	Santos Reyes Tepejillo	2000	73.0%	99.8%	8.6%
Mexico	Santos Reyes Tepejillo	2017	93.1%	100.0%	49.1%
Mexico	Santos Reyes Yucuna	2000	67.9%	99.1%	6.8%
Mexico	Santos Reyes Yucuna	2017	91.4%	99.9%	45.8%
Mexico	Saric	2000	83.6%	99.2%	47.3%
Mexico	Saric	2017	96.7%	99.9%	79.2%
Mexico	Satevo	2000	85.2%	96.3%	65.2%
Mexico	Satevo	2017	97.4%	99.7%	91.5%
Mexico	Saucillo	2000	85.4%	95.7%	70.8%
Mexico	Saucillo	2017	98.2%	99.7%	93.3%
Mexico	Sayula	2000	80.1%	99.7%	20.2%
Mexico	Sayula	2017	96.0%	100.0%	72.7%
Mexico	Sayula De Aleman	2000	63.4%	90.6%	26.5%
Mexico	Sayula De Aleman	2017	90.0%	99.2%	54.8%
Mexico	Senguio	2000	77.4%	99.2%	22.5%
Mexico	Senguio	2017	95.3%	99.9%	67.1%
Mexico	Seye	2000	86.2%	98.3%	61.9%

Country	Second Administrative Unit	Year	Mean	Upper	Lower
Mexico	Seye	2017	97.0%	99.9%	78.3%
Mexico	Sierra Mojada	2000	83.2%	96.6%	47.5%
Mexico	Sierra Mojada	2017	96.6%	99.5%	82.8%
Mexico	Silacayoapam	2000	73.6%	95.5%	33.9%
Mexico	Silacayoapam	2017	93.9%	99.6%	69.4%
Mexico	Silao	2000	84.9%	93.3%	74.6%
Mexico	Silao	2017	96.6%	99.4%	90.6%
Mexico	Siltepec	2000	64.7%	86.6%	39.7%
Mexico	Siltepec	2017	91.4%	98.3%	77.5%
Mexico	Simojovel	2000	69.9%	90.4%	38.4%
Mexico	Simojovel	2017	94.4%	99.2%	79.1%
Mexico	Sinaloa	2000	57.9%	69.3%	47.6%
Mexico	Sinaloa	2017	80.5%	85.4%	72.8%
Mexico	Sinanche	2000	77.3%	99.8%	6.6%
Mexico	Sinanche	2017	93.3%	100.0%	38.8%
Mexico	Singuilucan	2000	82.7%	93.8%	59.6%
Mexico	Singuilucan	2017	97.0%	99.5%	87.3%
Mexico	Sitala	2000	15.3%	36.2%	6.0%
Mexico	Sitala	2017	54.3%	82.6%	27.2%
Mexico	Sitio De Xitlapehua	2000	65.1%	94.8%	17.3%
Mexico	Sitio De Xitlapehua	2017	93.7%	99.5%	66.1%
Mexico	Sochiapa	2000	89.9%	96.8%	79.1%
Mexico	Sochiapa	2017	99.0%	99.7%	97.2%
Mexico	Socoltenango	2000	77.7%	93.4%	56.4%
Mexico	Socoltenango	2017	96.5%	99.5%	87.3%
Mexico	Soconusco	2000	66.9%	93.2%	28.3%
Mexico	Soconusco	2017	94.7%	99.3%	80.5%
Mexico	Soledad Atzompa	2000	61.8%	93.3%	16.4%
Mexico	Soledad Atzompa	2017	89.9%	99.7%	47.0%
Mexico	Soledad De Doblado	2000	76.2%	98.4%	25.4%
Mexico	Soledad De Doblado	2017	93.0%	99.9%	50.9%
Mexico	Soledad De Graciano Sanchez	2000	61.8%	71.6%	54.6%
Mexico	Soledad De Graciano Sanchez	2017	89.0%	96.1%	80.1%
Mexico	Soledad Etla	2000	66.9%	74.1%	59.5%
Mexico	Soledad Etla	2017	96.8%	97.7%	95.7%
Mexico	Solidaridad	2000	93.0%	97.3%	85.3%
Mexico	Solidaridad	2017	98.6%	99.8%	92.6%
Mexico	Solosuchiapa	2000	56.5%	91.4%	10.9%
Mexico	Solosuchiapa	2017	89.7%	99.2%	58.9%
Mexico	Soltepec	2000	84.0%	94.4%	67.0%
Mexico	Soltepec	2017	98.4%	99.7%	94.0%
Mexico	Sombrerete	2000	85.9%	94.7%	73.5%
Mexico	Sombrerete	2017	97.3%	99.4%	92.9%
Mexico	Soteapan	2000	69.9%	97.1%	21.1%
Mexico	Soteapan	2017	91.6%	99.7%	58.0%
Mexico	Soto La Marina	2000	84.7%	93.1%	72.0%
Mexico	Soto La Marina	2017	96.8%	99.1%	91.4%
Mexico	Sotuta	2000	72.6%	99.0%	19.5%
Mexico	Sotuta	2017	93.0%	99.9%	61.9%
Mexico	Soyalo	2000	72.5%	99.1%	14.4%

Country	Second Administrative Unit	Year	Mean	Upper	Lower
Mexico	Soyalo	2017	93.4%	99.9%	57.4%
Mexico	Soyaniquilpan De Juarez	2000	76.4%	99.4%	21.0%
Mexico	Soyaniquilpan De Juarez	2017	95.1%	100.0%	69.6%
Mexico	Soyopa	2000	80.8%	98.7%	48.0%
Mexico	Soyopa	2017	96.0%	99.8%	80.6%
Mexico	Suaqui Grande	2000	86.7%	99.8%	16.7%
Mexico	Suaqui Grande	2017	97.6%	100.0%	75.1%
Mexico	Suchiapa	2000	60.2%	68.3%	51.6%
Mexico	Suchiapa	2017	42.7%	45.5%	40.5%
Mexico	Suchiate	2000	73.2%	98.0%	19.7%
Mexico	Suchiate	2017	93.5%	99.9%	58.5%
Mexico	Suchil	2000	72.0%	97.0%	25.8%
Mexico	Suchil	2017	93.3%	99.7%	63.6%
Mexico	Sucila	2000	82.1%	99.9%	17.1%
Mexico	Sucila	2017	97.1%	100.0%	75.4%
Mexico	Sudzal	2000	84.3%	99.3%	32.6%
Mexico	Sudzal	2017	96.8%	100.0%	82.1%
Mexico	Sultepec	2000	75.1%	96.6%	36.3%
Mexico	Sultepec	2017	94.2%	99.8%	74.1%
Mexico	Suma	2000	95.9%	99.4%	85.9%
Mexico	Suma	2017	99.6%	100.0%	96.8%
Mexico	Sunuapa	2000	49.0%	97.7%	1.4%
Mexico	Sunuapa	2017	79.7%	99.9%	15.9%
Mexico	Susticacan	2000	79.6%	99.8%	20.0%
Mexico	Susticacan	2017	94.3%	100.0%	66.5%
Mexico	Susupuato	2000	52.1%	79.1%	23.5%
Mexico	Susupuato	2017	88.3%	97.6%	70.1%
Mexico	Tabasco	2000	85.2%	97.5%	53.9%
Mexico	Tabasco	2017	98.0%	99.8%	89.5%
Mexico	Tacambaro	2000	79.4%	90.8%	59.1%
Mexico	Tacambaro	2017	97.2%	99.3%	91.1%
Mexico	Tacotalpa	2000	66.2%	83.0%	41.2%
Mexico	Tacotalpa	2017	93.3%	98.4%	81.6%
Mexico	Tahdziu	2000	85.8%	99.5%	33.1%
Mexico	Tahdziu	2017	97.4%	100.0%	80.1%
Mexico	Tahmek	2000	79.6%	99.9%	21.3%
Mexico	Tahmek	2017	94.3%	100.0%	59.7%
Mexico	Tala	2000	85.6%	98.5%	54.5%
Mexico	Tala	2017	98.0%	99.9%	89.9%
Mexico	Talpa De Allende	2000	80.6%	96.4%	33.1%
Mexico	Talpa De Allende	2017	94.4%	99.7%	59.1%
Mexico	Tamalin	2000	60.5%	85.5%	30.3%
Mexico	Tamalin	2017	87.3%	98.6%	65.8%
Mexico	Tamasopo	2000	72.6%	92.0%	44.0%
Mexico	Tamasopo	2017	93.3%	99.3%	74.1%
Mexico	Tamazula	2000	79.4%	90.4%	61.9%
Mexico	Tamazula	2017	95.1%	98.6%	88.4%
Mexico	Tamazula De Gordiano	2000	81.4%	98.7%	35.1%
Mexico	Tamazula De Gordiano	2017	96.4%	99.9%	79.2%
Mexico	Tamazulapam Del Espiritu Santo	2000	71.4%	96.2%	24.5%
Mexico	Tamazulapam Del Espiritu Santo	2017	94.2%	99.7%	72.6%

Country	Second Administrative Unit	Year	Mean	Upper	Lower
Mexico	Tamazunchale	2000	71.7%	88.6%	53.9%
Mexico	Tamazunchale	2017	94.9%	99.0%	86.7%
Mexico	Tamiahua	2000	44.9%	75.2%	22.0%
Mexico	Tamiahua	2017	74.8%	91.8%	51.3%
Mexico	Tampacan	2000	42.4%	79.5%	6.6%
Mexico	Tampacan	2017	78.1%	97.4%	36.1%
Mexico	Tampamolon Corona	2000	42.9%	62.6%	21.4%
Mexico	Tampamolon Corona	2017	87.3%	95.5%	71.1%
Mexico	Tampico	2000	97.2%	98.7%	94.1%
Mexico	Tampico	2017	99.8%	99.9%	99.3%
Mexico	Tampico Alto	2000	76.2%	95.5%	46.6%
Mexico	Tampico Alto	2017	93.9%	99.5%	79.6%
Mexico	Tamuin	2000	76.2%	92.8%	43.2%
Mexico	Tamuin	2017	95.5%	99.2%	86.0%
Mexico	Tancanhuitz	2000	33.0%	47.7%	22.0%
Mexico	Tancanhuitz	2017	81.6%	91.6%	69.2%
Mexico	Tancitaro	2000	91.1%	99.0%	74.8%
Mexico	Tancitaro	2017	98.4%	99.9%	92.5%
Mexico	Tancoco	2000	84.7%	97.6%	57.4%
Mexico	Tancoco	2017	97.9%	99.9%	88.3%
Mexico	Tanetze De Zaragoza	2000	67.3%	88.2%	36.1%
Mexico	Tanetze De Zaragoza	2017	95.9%	98.5%	86.6%
Mexico	Tangamandapio	2000	70.9%	90.2%	42.1%
Mexico	Tangamandapio	2017	94.8%	99.4%	76.3%
Mexico	Tangancicuaro	2000	76.8%	95.6%	42.9%
Mexico	Tangancicuaro	2017	95.6%	99.7%	80.5%
Mexico	Tanhuato	2000	59.9%	93.1%	12.9%
Mexico	Tanhuato	2017	85.6%	99.1%	48.8%
Mexico	Taniche	2000	68.8%	96.9%	23.6%
Mexico	Taniche	2017	94.8%	99.8%	68.8%
Mexico	Tanlajas	2000	47.4%	58.1%	35.9%
Mexico	Tanlajas	2017	82.2%	92.0%	65.5%
Mexico	Tanquian De Escobedo	2000	94.8%	98.6%	84.8%
Mexico	Tanquian De Escobedo	2017	99.5%	99.9%	98.2%
Mexico	Tantima	2000	53.1%	76.4%	28.2%
Mexico	Tantima	2017	79.5%	95.9%	59.1%
Mexico	Tantoyuca	2000	69.5%	94.0%	27.1%
Mexico	Tantoyuca	2017	92.4%	99.3%	61.7%
Mexico	Tapachula	2000	53.6%	66.8%	40.7%
Mexico	Tapachula	2017	92.9%	96.2%	87.2%
Mexico	Tapalapa	2000	61.8%	83.0%	37.9%
Mexico	Tapalapa	2017	94.2%	98.5%	82.1%
Mexico	Tapalpa	2000	82.2%	98.7%	41.6%
Mexico	Tapalpa	2017	96.3%	99.9%	79.6%
Mexico	Tapilula	2000	44.0%	79.7%	14.7%
Mexico	Tapilula	2017	88.6%	98.8%	64.1%
Mexico	Tarandacuao	2000	69.8%	99.1%	10.0%
Mexico	Tarandacuao	2017	92.1%	99.9%	50.9%
Mexico	Taretan	2000	47.6%	88.7%	3.8%
Mexico	Taretan	2017	73.5%	97.6%	20.8%
Mexico	Tarimbaro	2000	57.1%	72.7%	39.2%
Mexico	Tarimbaro	2017	89.1%	96.4%	74.8%
Mexico	Tarimoro	2000	83.5%	95.8%	55.9%
Mexico	Tarimoro	2017	95.6%	99.6%	85.9%
Mexico	Tasquillo	2000	71.6%	84.7%	52.5%
Mexico	Tasquillo	2017	96.7%	98.8%	88.5%

Country	Second Administrative Unit	Year	Mean	Upper	Lower
Mexico	Tatahuicapan De Juarez	2000	69.5%	97.7%	24.7%
Mexico	Tatahuicapan De Juarez	2017	90.9%	99.8%	60.8%
Mexico	Tataltepec De Valdes	2000	76.4%	99.6%	24.2%
Mexico	Tataltepec De Valdes	2017	93.9%	100.0%	59.7%
Mexico	Tatatila	2000	65.4%	99.1%	12.3%
Mexico	Tatatila	2017	90.3%	99.9%	50.5%
Mexico	Taxco De Alarcon	2000	49.4%	65.5%	34.7%
Mexico	Taxco De Alarcon	2017	88.8%	94.5%	81.1%
Mexico	Teabo	2000	96.7%	99.6%	90.4%
Mexico	Teabo	2017	99.6%	100.0%	97.4%
Mexico	Teapa	2000	90.1%	97.4%	76.4%
Mexico	Teapa	2017	98.5%	99.8%	94.5%
Mexico	Tecali De Herrera	2000	82.5%	98.4%	48.8%
Mexico	Tecali De Herrera	2017	97.1%	99.9%	84.2%
Mexico	Tecalitlan	2000	83.3%	98.3%	34.4%
Mexico	Tecalitlan	2017	97.3%	99.8%	84.8%
Mexico	Tecamac	2000	94.0%	97.4%	88.9%
Mexico	Tecamac	2017	99.0%	99.8%	95.8%
Mexico	Tecamachalco	2000	90.0%	96.5%	79.0%
Mexico	Tecamachalco	2017	98.8%	99.8%	95.5%
Mexico	Tecate	2000	94.6%	98.4%	87.1%
Mexico	Tecate	2017	99.4%	99.9%	97.4%
Mexico	Techaluta De Montenegro	2000	77.8%	99.9%	15.0%
Mexico	Techaluta De Montenegro	2017	95.6%	100.0%	67.0%
Mexico	Tecoanapa	2000	70.3%	93.0%	29.1%
Mexico	Tecoanapa	2017	93.5%	99.4%	69.7%
Mexico	Tecoh	2000	83.4%	99.3%	25.9%
Mexico	Tecoh	2017	96.2%	100.0%	73.3%
Mexico	Tecolotlan	2000	74.7%	97.8%	26.1%
Mexico	Tecolotlan	2017	93.1%	99.8%	60.8%
Mexico	Tecolutla	2000	53.4%	76.9%	30.2%
Mexico	Tecolutla	2017	84.1%	96.4%	66.7%
Mexico	Tecoman	2000	95.4%	98.6%	90.0%
Mexico	Tecoman	2017	99.3%	99.9%	95.8%
Mexico	Tecomatlan	2000	74.2%	98.2%	18.5%
Mexico	Tecomatlan	2017	94.9%	99.8%	67.0%
Mexico	Tecozautla	2000	90.3%	98.9%	70.6%
Mexico	Tecozautla	2017	98.4%	99.9%	92.5%
Mexico	Tecpan De Galeana	2000	67.5%	87.3%	40.2%
Mexico	Tecpan De Galeana	2017	93.9%	98.4%	80.5%
Mexico	Tecpatan	2000	65.6%	84.9%	39.6%
Mexico	Tecpatan	2017	91.8%	98.5%	78.6%
Mexico	Tecuala	2000	74.1%	82.5%	62.4%
Mexico	Tecuala	2017	96.4%	98.3%	91.6%
Mexico	Tehuacan	2000	84.8%	91.6%	73.6%
Mexico	Tehuacan	2017	97.4%	99.3%	93.3%
Mexico	Tehuipango	2000	75.5%	94.8%	34.4%
Mexico	Tehuipango	2017	96.7%	99.7%	82.0%
Mexico	Tehuitzingo	2000	78.2%	98.5%	30.9%
Mexico	Tehuitzingo	2017	95.4%	99.9%	76.5%

Country	Second Administrative Unit	Year	Mean	Upper	Lower
Mexico	Tejupilco	2000	42.0%	63.3%	26.3%
Mexico	Tejupilco	2017	85.1%	94.4%	66.9%
Mexico	Tekal De Venegas	2000	86.9%	99.5%	41.7%
Mexico	Tekal De Venegas	2017	98.6%	100.0%	92.0%
Mexico	Tekanto	2000	97.4%	99.8%	88.9%
Mexico	Tekanto	2017	99.8%	100.0%	99.2%
Mexico	Tekax	2000	75.1%	89.9%	48.6%
Mexico	Tekax	2017	96.8%	98.9%	91.8%
Mexico	Tekit	2000	96.1%	99.7%	78.5%
Mexico	Tekit	2017	99.7%	100.0%	98.3%
Mexico	Tekom	2000	76.5%	99.5%	19.2%
Mexico	Tekom	2017	93.6%	100.0%	50.4%
Mexico	Telchac Pueblo	2000	74.3%	100.0%	3.1%
Mexico	Telchac Pueblo	2017	93.1%	100.0%	38.2%
Mexico	Telchac Puerto	2000	91.3%	99.9%	41.9%
Mexico	Telchac Puerto	2017	98.8%	100.0%	89.3%
Mexico	Teloloapan	2000	71.6%	91.8%	30.5%
Mexico	Teloloapan	2017	94.4%	99.2%	73.4%
Mexico	Temamatla	2000	99.3%	99.9%	96.7%
Mexico	Temamatla	2017	100.0%	100.0%	99.8%
Mexico	Temapache	2000	83.5%	92.4%	68.7%
Mexico	Temapache	2017	94.1%	98.4%	83.2%
Mexico	Temascalapa	2000	85.6%	95.9%	62.6%
Mexico	Temascalapa	2017	97.2%	99.6%	86.9%
Mexico	Temascalcingo	2000	78.6%	98.0%	35.8%
Mexico	Temascalcingo	2017	95.1%	99.9%	73.5%
Mexico	Temascaltepec	2000	73.7%	94.1%	36.8%
Mexico	Temascaltepec	2017	92.7%	99.5%	69.8%
Mexico	Temax	2000	84.2%	99.8%	48.6%
Mexico	Temax	2017	97.5%	100.0%	84.4%
Mexico	Temixco	2000	93.0%	95.2%	90.2%
Mexico	Temixco	2017	99.5%	99.7%	99.2%
Mexico	Temoac	2000	41.7%	48.1%	36.3%
Mexico	Temoac	2017	88.3%	90.9%	84.6%
Mexico	Temoaya	2000	87.8%	99.7%	33.6%
Mexico	Temoaya	2017	97.8%	100.0%	80.9%
Mexico	Temosachi	2000	81.1%	93.7%	63.7%
Mexico	Temosachi	2017	96.1%	99.2%	88.4%
Mexico	Temozon	2000	69.1%	98.1%	27.3%
Mexico	Temozon	2017	90.8%	99.8%	59.9%
Mexico	Tempoal	2000	67.6%	84.8%	43.6%
Mexico	Tempoal	2017	94.2%	98.2%	84.8%
Mexico	Tenabo	2000	81.4%	98.9%	22.6%
Mexico	Tenabo	2017	94.3%	99.9%	60.5%
Mexico	Tenamaxtlan	2000	83.6%	98.0%	47.2%
Mexico	Tenamaxtlan	2017	97.1%	99.9%	85.6%
Mexico	Tenampa	2000	80.6%	99.8%	18.5%
Mexico	Tenampa	2017	96.2%	100.0%	72.4%
Mexico	Tenampulco	2000	69.9%	98.4%	19.1%
Mexico	Tenampulco	2017	90.5%	99.9%	55.2%
Mexico	Tenancingo	2000	91.7%	97.2%	80.9%
Mexico	Tenancingo	2000	90.0%	99.7%	68.1%
Mexico	Tenancingo	2017	98.1%	100.0%	88.1%
Mexico	Tenancingo	2017	99.5%	99.8%	98.6%
Mexico	Tenango De Doria	2000	80.2%	98.1%	32.4%

Country	Second Administrative Unit	Year	Mean	Upper	Lower
Mexico	Tenango De Doria	2017	96.1%	99.9%	74.5%
Mexico	Tenango Del Aire	2000	98.1%	100.0%	91.3%
Mexico	Tenango Del Aire	2017	99.8%	100.0%	99.1%
Mexico	Tenango Del Valle	2000	63.9%	81.2%	41.3%
Mexico	Tenango Del Valle	2017	94.8%	98.5%	85.7%
Mexico	Tenejapa	2000	58.2%	89.2%	18.1%
Mexico	Tenejapa	2017	90.8%	99.0%	64.0%
Mexico	Tenochtitlan	2000	45.9%	97.0%	2.1%
Mexico	Tenochtitlan	2017	75.4%	99.6%	12.3%
Mexico	Tenosique	2000	56.7%	83.9%	25.6%
Mexico	Tenosique	2017	87.6%	97.7%	51.8%
Mexico	Teocaltiche	2000	85.6%	96.3%	66.2%
Mexico	Teocaltiche	2017	98.1%	99.7%	93.7%
Mexico	Teocelo	2000	96.4%	99.9%	85.6%
Mexico	Teocelo	2017	99.3%	100.0%	95.7%
Mexico	Teococuilco De Marcos Perez	2000	72.8%	99.5%	17.8%
Mexico	Teococuilco De Marcos Perez	2017	93.0%	99.9%	58.8%
Mexico	Teocuitatlan De Corona	2000	78.2%	98.8%	31.5%
Mexico	Teocuitatlan De Corona	2017	95.0%	99.9%	71.2%
Mexico	Teolocholco	2000	99.7%	99.9%	99.3%
Mexico	Teolocholco	2017	100.0%	100.0%	99.9%
Mexico	Teoloyucan	2000	85.1%	96.4%	62.9%
Mexico	Teoloyucan	2017	98.0%	99.7%	89.5%
Mexico	Teopantlan	2000	79.9%	98.9%	33.4%
Mexico	Teopantlan	2017	96.2%	99.9%	82.1%
Mexico	Teopisca	2000	82.5%	99.6%	32.4%
Mexico	Teopisca	2017	95.9%	100.0%	66.0%
Mexico	Teotihuacan	2000	89.2%	99.8%	46.6%
Mexico	Teotihuacan	2017	97.5%	100.0%	81.1%
Mexico	Teotitlan De Flores Magon	2000	71.9%	95.7%	25.3%
Mexico	Teotitlan De Flores Magon	2017	95.7%	99.8%	76.6%
Mexico	Teotitlan Del Valle	2000	78.3%	98.7%	12.3%
Mexico	Teotitlan Del Valle	2017	95.2%	99.9%	59.2%
Mexico	Teotlalco	2000	77.4%	99.6%	16.6%
Mexico	Teotlalco	2017	95.4%	100.0%	69.2%
Mexico	Teotongo	2000	72.1%	99.7%	6.5%
Mexico	Teotongo	2017	92.7%	100.0%	40.9%
Mexico	Tepache	2000	81.6%	99.9%	8.8%
Mexico	Tepache	2017	95.7%	100.0%	61.4%
Mexico	Tepakan	2000	87.6%	99.6%	39.1%
Mexico	Tepakan	2017	98.3%	100.0%	86.6%
Mexico	Tepalcatepec	2000	81.1%	98.0%	31.7%
Mexico	Tepalcatepec	2017	94.8%	99.9%	56.2%
Mexico	Tepalcingo	2000	74.8%	94.5%	44.0%
Mexico	Tepalcingo	2017	95.9%	99.6%	84.1%
Mexico	Tepanco De Lopez	2000	53.3%	95.9%	9.2%

Country	Second Administrative Unit	Year	Mean	Upper	Lower
Mexico	Tepanco De Lopez	2017	81.0%	99.7%	40.7%
Mexico	Tepango De Rodriguez	2000	77.0%	97.0%	45.0%
Mexico	Tepango De Rodriguez	2017	97.4%	99.8%	86.7%
Mexico	Tepatitlan De Morelos	2000	90.1%	97.1%	76.1%
Mexico	Tepatitlan De Morelos	2017	98.4%	99.8%	94.9%
Mexico	Tepatlaxco	2000	77.6%	99.5%	21.0%
Mexico	Tepatlaxco	2017	95.4%	100.0%	66.5%
Mexico	Tepatlaxco De Hidalgo	2000	92.5%	97.5%	82.8%
Mexico	Tepatlaxco De Hidalgo	2017	99.5%	99.8%	98.8%
Mexico	Tepeaca	2000	88.8%	94.6%	79.0%
Mexico	Tepeaca	2017	98.7%	99.6%	95.0%
Mexico	Tepeapulco	2000	79.7%	99.0%	24.9%
Mexico	Tepeapulco	2017	96.2%	99.9%	74.8%
Mexico	Tepechitlan	2000	79.2%	98.9%	26.5%
Mexico	Tepechitlan	2017	95.4%	99.9%	70.9%
Mexico	Tepecoacuilco De Trujano	2000	62.4%	87.9%	32.2%
Mexico	Tepecoacuilco De Trujano	2017	87.7%	98.6%	64.6%
Mexico	Tepehuacan De Guerrero	2000	82.5%	98.6%	50.0%
Mexico	Tepehuacan De Guerrero	2017	96.6%	99.9%	84.3%
Mexico	Tepehuanes	2000	73.1%	81.9%	61.3%
Mexico	Tepehuanes	2017	95.7%	97.5%	93.1%
Mexico	Tepeji Del Rio De Ocampo	2000	76.5%	86.8%	61.5%
Mexico	Tepeji Del Rio De Ocampo	2017	93.9%	98.6%	85.4%
Mexico	Tepelmeme Villa De Morelos	2000	77.5%	98.4%	28.4%
Mexico	Tepelmeme Villa De Morelos	2017	94.5%	99.9%	59.1%
Mexico	Tepemaxalco	2000	79.2%	99.7%	15.5%
Mexico	Tepemaxalco	2017	95.9%	100.0%	66.5%
Mexico	Tepeojuma	2000	80.2%	99.8%	22.5%
Mexico	Tepeojuma	2017	95.7%	100.0%	67.3%
Mexico	Tepetitla De Lardizabal	2000	60.9%	65.9%	54.0%
Mexico	Tepetitla De Lardizabal	2017	96.1%	96.9%	94.5%
Mexico	Tepetitlan	2000	83.0%	99.8%	36.3%
Mexico	Tepetitlan	2017	97.3%	100.0%	84.2%
Mexico	Tepetlan	2000	77.1%	99.4%	21.9%
Mexico	Tepetlan	2017	95.1%	100.0%	64.4%
Mexico	Tepetlaoxtoc	2000	85.0%	99.9%	32.6%
Mexico	Tepetlaoxtoc	2017	96.5%	100.0%	73.2%
Mexico	Tepetlixpa	2000	86.9%	99.9%	29.3%
Mexico	Tepetlixpa	2017	97.5%	100.0%	83.5%
Mexico	Tepetongo	2000	84.0%	96.9%	60.2%
Mexico	Tepetongo	2017	96.6%	99.8%	84.1%
Mexico	Tepetzintla	2000	86.8%	99.8%	36.0%
Mexico	Tepetzintla	2000	45.7%	94.9%	4.7%

Piped Water Proportion by Second Administrative Unit *(continued)*

Country	Second Administrative Unit	Year	Mean	Upper	Lower
Mexico	Tepetzintla	2017	81.1%	99.6%	32.4%
Mexico	Tepetzintla	2017	97.2%	100.0%	77.9%
Mexico	Tepexco	2000	74.5%	99.4%	12.7%
Mexico	Tepexco	2017	94.2%	100.0%	62.5%
Mexico	Tepexi De Rodriguez	2000	77.2%	95.5%	38.4%
Mexico	Tepexi De Rodriguez	2017	96.2%	99.6%	80.4%
Mexico	Tepeyahualco	2000	74.2%	97.5%	27.7%
Mexico	Tepeyahualco	2017	93.2%	99.8%	65.9%
Mexico	Tepeyahualco De Cuauhtemoc	2000	66.1%	99.6%	5.0%
Mexico	Tepeyahualco De Cuauhtemoc	2017	89.9%	100.0%	39.7%
Mexico	Tepeyanco	2000	99.2%	99.8%	97.7%
Mexico	Tepeyanco	2017	100.0%	100.0%	99.9%
Mexico	Tepezala	2000	81.9%	95.7%	54.6%
Mexico	Tepezala	2017	97.6%	99.7%	87.9%
Mexico	Tepic	2000	90.7%	97.6%	72.9%
Mexico	Tepic	2017	98.6%	99.8%	90.9%
Mexico	Tepotzotlan	2000	84.3%	96.9%	57.0%
Mexico	Tepotzotlan	2017	98.3%	99.8%	93.3%
Mexico	Tepoztlan	2000	91.9%	96.5%	82.8%
Mexico	Tepoztlan	2017	99.2%	99.8%	97.5%
Mexico	Tequila	2000	82.4%	97.9%	37.3%
Mexico	Tequila	2000	61.9%	95.7%	15.4%
Mexico	Tequila	2017	97.9%	99.8%	90.7%
Mexico	Tequila	2017	90.2%	99.7%	59.5%
Mexico	Tequisquiapan	2000	91.8%	99.4%	64.1%
Mexico	Tequisquiapan	2017	99.1%	100.0%	95.3%
Mexico	Tequixquiac	2000	96.3%	99.9%	73.5%
Mexico	Tequixquiac	2017	99.4%	100.0%	95.1%
Mexico	Terrenate	2000	71.2%	99.2%	10.7%
Mexico	Terrenate	2017	93.2%	100.0%	53.2%
Mexico	Tetecala	2000	75.1%	97.5%	25.2%
Mexico	Tetecala	2017	95.1%	99.8%	72.0%
Mexico	Tetela De Ocampo	2000	70.3%	91.9%	41.0%
Mexico	Tetela De Ocampo	2017	94.7%	99.4%	81.4%
Mexico	Tetela Del Volcan	2000	86.8%	92.2%	79.2%
Mexico	Tetela Del Volcan	2017	98.6%	99.5%	95.8%
Mexico	Teteles De Avila Castillo	2000	73.0%	95.9%	23.8%
Mexico	Teteles De Avila Castillo	2017	96.8%	99.7%	85.9%
Mexico	Tetepango	2000	97.9%	99.9%	90.7%
Mexico	Tetepango	2017	99.9%	100.0%	99.3%
Mexico	Tetipac	2000	15.6%	29.6%	2.4%
Mexico	Tetipac	2017	28.7%	42.9%	14.6%
Mexico	Tetiz	2000	83.5%	99.7%	37.4%
Mexico	Tetiz	2017	97.6%	100.0%	84.3%
Mexico	Tetla De La Solidaridad	2000	96.3%	99.6%	89.6%
Mexico	Tetla De La Solidaridad	2017	99.4%	100.0%	96.8%
Mexico	Tetlatlahuca	2000	97.3%	98.1%	96.3%
Mexico	Tetlatlahuca	2017	99.8%	99.8%	99.7%

Country	Second Administrative Unit	Year	Mean	Upper	Lower
Mexico	Teuchitlan	2000	83.5%	99.5%	35.8%
Mexico	Teuchitlan	2017	97.1%	100.0%	82.8%
Mexico	Teul De Gonzalez Ortega	2000	83.3%	97.8%	52.0%
Mexico	Teul De Gonzalez Ortega	2017	97.0%	99.8%	84.3%
Mexico	Texcaltitlan	2000	75.8%	99.2%	17.3%
Mexico	Texcaltitlan	2017	93.9%	100.0%	54.0%
Mexico	Texcalyacac	2000	78.5%	87.4%	68.3%
Mexico	Texcalyacac	2017	98.3%	99.2%	97.0%
Mexico	Texcatepec	2000	67.5%	91.6%	30.1%
Mexico	Texcatepec	2017	94.2%	99.2%	73.2%
Mexico	Texcoco	2000	97.3%	99.5%	86.4%
Mexico	Texcoco	2017	99.6%	100.0%	97.2%
Mexico	Texhuacan	2000	73.2%	99.4%	12.1%
Mexico	Texhuacan	2017	93.0%	100.0%	52.4%
Mexico	Texistepec	2000	65.8%	93.4%	22.8%
Mexico	Texistepec	2017	90.3%	99.5%	53.7%
Mexico	Teya	2000	89.1%	99.9%	36.1%
Mexico	Teya	2017	97.4%	100.0%	75.1%
Mexico	Teziutlan	2000	91.8%	95.8%	83.8%
Mexico	Teziutlan	2017	99.3%	99.7%	97.5%
Mexico	Tezoatlan De Segura Y Luna	2000	79.9%	98.7%	34.1%
Mexico	Tezoatlan De Segura Y Luna	2017	95.5%	99.9%	71.5%
Mexico	Tezonapa	2000	69.9%	89.5%	39.1%
Mexico	Tezonapa	2017	93.5%	99.1%	77.1%
Mexico	Tezontepec De Aldama	2000	98.4%	99.9%	93.7%
Mexico	Tezontepec De Aldama	2017	99.9%	100.0%	99.3%
Mexico	Tezoyuca	2000	96.1%	99.8%	82.5%
Mexico	Tezoyuca	2017	99.6%	100.0%	97.4%
Mexico	Tianguismanalco	2000	78.5%	92.9%	51.7%
Mexico	Tianguismanalco	2017	97.4%	99.1%	92.9%
Mexico	Tianguistenco	2000	56.8%	88.3%	25.9%
Mexico	Tianguistenco	2017	90.4%	99.3%	64.2%
Mexico	Tianguistengo	2000	75.1%	97.6%	33.5%
Mexico	Tianguistengo	2017	94.4%	99.7%	73.4%
Mexico	Ticul	2000	93.4%	98.4%	85.4%
Mexico	Ticul	2017	99.1%	99.9%	96.4%
Mexico	Tierra Blanca	2000	83.6%	99.5%	25.2%
Mexico	Tierra Blanca	2000	73.5%	92.2%	42.4%
Mexico	Tierra Blanca	2017	94.6%	98.6%	87.1%
Mexico	Tierra Blanca	2017	95.9%	100.0%	70.3%
Mexico	Tierra Nueva	2000	82.5%	99.7%	31.0%
Mexico	Tierra Nueva	2017	96.7%	100.0%	81.2%
Mexico	Tihuatlan	2000	59.8%	76.2%	37.4%
Mexico	Tihuatlan	2017	91.2%	96.2%	76.2%
Mexico	Tijuana	2000	96.5%	98.7%	92.0%
Mexico	Tijuana	2017	99.7%	99.9%	98.8%
Mexico	Tila	2000	84.3%	96.7%	62.9%
Mexico	Tila	2017	97.0%	99.7%	88.3%
Mexico	Tilapa	2000	83.3%	95.3%	61.2%
Mexico	Tilapa	2017	98.4%	99.7%	93.8%
Mexico	Timilpan	2000	86.1%	99.1%	53.6%
Mexico	Timilpan	2017	98.0%	99.9%	87.8%
Mexico	Timucuy	2000	95.6%	99.5%	85.9%
Mexico	Timucuy	2017	99.4%	100.0%	96.9%

Country	Second Administrative Unit	Year	Mean	Upper	Lower
Mexico	Tingambato	2000	87.7%	97.9%	64.1%
Mexico	Tingambato	2017	97.3%	99.8%	90.4%
Mexico	Tinguindin	2000	88.7%	99.6%	54.5%
Mexico	Tinguindin	2017	97.9%	100.0%	88.7%
Mexico	Tinum	2000	83.4%	99.2%	41.4%
Mexico	Tinum	2017	97.0%	99.9%	81.7%
Mexico	Tiquicheo De Nicolas Romero	2000	77.6%	95.5%	50.4%
Mexico	Tiquicheo De Nicolas Romero	2017	94.7%	99.6%	78.9%
Mexico	Tixcacalcupul	2000	72.8%	97.6%	28.9%
Mexico	Tixcacalcupul	2017	92.9%	99.8%	65.1%
Mexico	Tixkokob	2000	93.0%	98.3%	83.0%
Mexico	Tixkokob	2017	99.2%	99.9%	96.9%
Mexico	Tixmehuac	2000	79.5%	99.8%	21.0%
Mexico	Tixmehuac	2017	95.3%	100.0%	64.6%
Mexico	Tixpehual	2000	84.9%	99.6%	36.4%
Mexico	Tixpehual	2017	97.9%	100.0%	85.4%
Mexico	Tixtla De Guerrero	2000	69.4%	97.8%	15.1%
Mexico	Tixtla De Guerrero	2017	93.1%	99.7%	62.2%
Mexico	Tizapan El Alto	2000	85.3%	99.8%	31.6%
Mexico	Tizapan El Alto	2017	97.3%	100.0%	75.8%
Mexico	Tizayuca	2000	90.4%	95.3%	84.1%
Mexico	Tizayuca	2017	99.3%	99.8%	98.0%
Mexico	Tizimin	2000	86.7%	93.0%	77.2%
Mexico	Tizimin	2017	98.3%	99.3%	95.8%
Mexico	Tlachichilco	2000	66.8%	94.1%	24.8%
Mexico	Tlachichilco	2017	90.9%	99.5%	64.5%
Mexico	Tlachichuca	2000	83.8%	96.6%	59.3%
Mexico	Tlachichuca	2017	97.4%	99.8%	88.3%
Mexico	Tlacoachistlahuaca	2000	69.6%	90.8%	45.5%
Mexico	Tlacoachistlahuaca	2017	92.5%	99.2%	76.1%
Mexico	Tlacoapa	2000	52.1%	84.7%	14.9%
Mexico	Tlacoapa	2017	85.3%	98.3%	50.1%
Mexico	Tlacojalpan	2000	63.1%	99.2%	2.6%
Mexico	Tlacojalpan	2017	84.9%	99.9%	21.6%
Mexico	Tlacolula De Matamoros	2000	85.3%	97.3%	39.8%
Mexico	Tlacolula De Matamoros	2017	98.3%	99.8%	88.6%
Mexico	Tlacolulan	2000	81.3%	99.0%	32.7%
Mexico	Tlacolulan	2017	95.9%	99.9%	68.0%
Mexico	Tlacotalpan	2000	89.2%	98.6%	73.7%
Mexico	Tlacotalpan	2017	97.7%	99.9%	91.5%
Mexico	Tlacotepec De Benito Juarez	2000	88.9%	98.8%	65.3%
Mexico	Tlacotepec De Benito Juarez	2017	98.2%	99.9%	91.0%
Mexico	Tlacotepec De Mejia	2000	77.5%	98.4%	25.2%
Mexico	Tlacotepec De Mejia	2017	96.2%	99.9%	78.8%
Mexico	Tlacotepec Plumas	2000	75.8%	99.8%	8.3%
Mexico	Tlacotepec Plumas	2017	93.5%	100.0%	51.1%

Country	Second Administrative Unit	Year	Mean	Upper	Lower
Mexico	Tlacuilotepec	2000	75.0%	98.4%	29.3%
Mexico	Tlacuilotepec	2017	94.1%	99.9%	68.3%
Mexico	Tlahuac	2000	99.0%	99.9%	93.5%
Mexico	Tlahuac	2017	99.9%	100.0%	98.7%
Mexico	Tlahualilo	2000	89.7%	95.4%	80.6%
Mexico	Tlahualilo	2017	99.0%	99.7%	97.7%
Mexico	Tlahuapan	2000	93.1%	98.8%	80.4%
Mexico	Tlahuapan	2017	99.2%	99.9%	94.6%
Mexico	Tlahuelilpan	2000	99.5%	99.9%	98.7%
Mexico	Tlahuelilpan	2017	100.0%	100.0%	99.9%
Mexico	Tlahuiltepa	2000	82.5%	97.7%	52.0%
Mexico	Tlahuiltepa	2017	96.3%	99.8%	82.9%
Mexico	Tlajomulco De Zuniga	2000	82.0%	93.1%	66.7%
Mexico	Tlajomulco De Zuniga	2017	94.1%	99.4%	79.6%
Mexico	Tlalchapa	2000	78.7%	98.4%	34.1%
Mexico	Tlalchapa	2017	95.7%	99.8%	79.1%
Mexico	Tlalixcoyan	2000	73.3%	93.5%	44.9%
Mexico	Tlalixcoyan	2017	95.1%	99.4%	83.2%
Mexico	Tlalixtac De Cabrera	2000	78.3%	88.5%	59.0%
Mexico	Tlalixtac De Cabrera	2017	98.0%	99.0%	95.5%
Mexico	Tlalixtaquilla De Maldonado	2000	52.8%	76.4%	23.5%
Mexico	Tlalixtaquilla De Maldonado	2017	91.5%	97.9%	78.6%
Mexico	Tlalmanalco	2000	95.1%	98.7%	86.8%
Mexico	Tlalmanalco	2017	99.3%	99.9%	95.6%
Mexico	Tlalnelhuayocan	2000	33.5%	36.5%	30.6%
Mexico	Tlalnelhuayocan	2017	64.9%	76.6%	59.8%
Mexico	Tlalnepantla	2000	84.1%	94.7%	66.6%
Mexico	Tlalnepantla	2017	98.7%	99.7%	94.8%
Mexico	Tlalnepantla De Baz	2000	98.1%	98.6%	97.5%
Mexico	Tlalnepantla De Baz	2017	99.9%	99.9%	99.8%
Mexico	Tlalpan	2000	90.6%	94.4%	84.8%
Mexico	Tlalpan	2017	98.9%	99.6%	96.2%
Mexico	Tlalpujahua	2000	61.6%	79.1%	39.1%
Mexico	Tlalpujahua	2017	94.9%	98.1%	87.2%
Mexico	Tlaltenango	2000	80.4%	84.5%	75.0%
Mexico	Tlaltenango	2017	98.4%	98.8%	97.9%
Mexico	Tlaltenango De Sanchez Roman	2000	91.8%	96.9%	83.9%
Mexico	Tlaltenango De Sanchez Roman	2017	99.2%	99.8%	97.4%
Mexico	Tlaltetela	2000	86.6%	99.1%	60.2%
Mexico	Tlaltetela	2017	97.6%	99.9%	88.8%
Mexico	Tlaltizapan	2000	83.4%	99.5%	44.3%
Mexico	Tlaltizapan	2017	96.6%	100.0%	81.6%
Mexico	Tlanalapa	2000	73.5%	99.7%	12.5%
Mexico	Tlanalapa	2017	93.7%	100.0%	58.0%
Mexico	Tlanchinol	2000	88.6%	99.6%	63.9%
Mexico	Tlanchinol	2017	97.3%	100.0%	84.8%
Mexico	Tlanepantla	2000	77.3%	98.7%	22.3%
Mexico	Tlanepantla	2017	96.3%	99.9%	74.5%

Country	Second Administrative Unit	Year	Mean	Upper	Lower
Mexico	Tlaola	2000	49.3%	62.6%	33.1%
Mexico	Tlaola	2017	88.4%	94.6%	76.3%
Mexico	Tlapa De Comonfort	2000	53.4%	72.4%	39.7%
Mexico	Tlapa De Comonfort	2017	93.3%	97.8%	85.9%
Mexico	Tlapacoya	2000	59.3%	96.7%	7.7%
Mexico	Tlapacoya	2017	88.1%	99.8%	40.8%
Mexico	Tlapacoyan	2000	70.0%	98.4%	18.3%
Mexico	Tlapacoyan	2017	92.2%	99.9%	60.4%
Mexico	Tlapanala	2000	79.2%	99.9%	24.2%
Mexico	Tlapanala	2017	95.8%	100.0%	73.4%
Mexico	Tlapehuala	2000	69.1%	90.6%	30.8%
Mexico	Tlapehuala	2017	93.8%	99.2%	71.3%
Mexico	Tlaquepaque	2000	93.1%	94.8%	90.9%
Mexico	Tlaquepaque	2017	99.5%	99.7%	98.9%
Mexico	Tlaquilpa	2000	74.1%	99.6%	10.7%
Mexico	Tlaquilpa	2017	94.2%	100.0%	55.6%
Mexico	Tlaquiltenango	2000	93.2%	99.0%	80.7%
Mexico	Tlaquiltenango	2017	98.9%	99.9%	95.3%
Mexico	Tlatlauquitepec	2000	83.8%	97.9%	54.5%
Mexico	Tlatlauquitepec	2017	97.4%	99.9%	88.2%
Mexico	Tlatlaya	2000	79.9%	97.4%	47.5%
Mexico	Tlatlaya	2017	95.2%	99.8%	81.5%
Mexico	Tlaxcala	2000	98.7%	99.3%	97.7%
Mexico	Tlaxcala	2017	99.9%	100.0%	99.9%
Mexico	Tlaxco	2000	88.2%	98.6%	69.8%
Mexico	Tlaxco	2000	74.8%	99.4%	11.8%
Mexico	Tlaxco	2017	97.6%	99.9%	88.8%
Mexico	Tlaxco	2017	93.8%	100.0%	54.5%
Mexico	Tlaxcoapan	2000	99.3%	99.9%	97.7%
Mexico	Tlaxcoapan	2017	100.0%	100.0%	99.9%
Mexico	Tlayacapan	2000	93.5%	99.7%	69.6%
Mexico	Tlayacapan	2017	99.2%	100.0%	95.8%
Mexico	Tlazazalca	2000	79.6%	99.5%	19.7%
Mexico	Tlazazalca	2017	95.2%	100.0%	62.8%
Mexico	Tlilapan	2000	56.9%	86.2%	25.5%
Mexico	Tlilapan	2017	93.6%	99.1%	77.7%
Mexico	Tocatlan	2000	94.2%	99.9%	58.9%
Mexico	Tocatlan	2017	99.3%	100.0%	96.1%
Mexico	Tochimilco	2000	76.8%	98.1%	28.1%
Mexico	Tochimilco	2017	95.3%	99.8%	69.2%
Mexico	Tochtepec	2000	69.7%	97.4%	16.6%
Mexico	Tochtepec	2017	93.9%	99.8%	64.2%
Mexico	Tocumbo	2000	80.7%	95.8%	57.3%
Mexico	Tocumbo	2017	95.7%	99.6%	86.1%
Mexico	Tolcayuca	2000	98.4%	100.0%	90.5%
Mexico	Tolcayuca	2017	99.5%	100.0%	95.2%
Mexico	Toliman	2000	90.2%	98.4%	72.5%
Mexico	Toliman	2000	84.5%	98.2%	54.7%
Mexico	Toliman	2017	98.3%	99.9%	90.9%
Mexico	Toliman	2017	97.4%	99.9%	87.4%
Mexico	Toluca	2000	84.9%	89.8%	76.2%
Mexico	Toluca	2017	96.7%	98.8%	94.3%
Mexico	Tomatlan	2000	75.9%	91.9%	47.3%
Mexico	Tomatlan	2000	97.6%	99.5%	93.0%
Mexico	Tomatlan	2017	94.6%	99.2%	80.4%
Mexico	Tomatlan	2017	99.8%	100.0%	99.5%
Mexico	Tonala	2000	80.4%	87.0%	68.0%
Mexico	Tonala	2000	78.7%	92.6%	58.8%
Mexico	Tonala	2017	95.3%	99.3%	84.8%
Mexico	Tonala	2017	97.7%	98.9%	94.0%

Country	Second Administrative Unit	Year	Mean	Upper	Lower
Mexico	Tonanitla	2000	96.7%	98.4%	93.6%
Mexico	Tonanitla	2017	99.8%	99.9%	99.5%
Mexico	Tonatico	2000	62.5%	97.4%	8.0%
Mexico	Tonatico	2017	89.3%	99.8%	51.6%
Mexico	Tonaya	2000	81.0%	99.4%	27.4%
Mexico	Tonaya	2017	96.1%	100.0%	76.5%
Mexico	Tonayan	2000	84.6%	99.7%	41.5%
Mexico	Tonayan	2017	96.8%	100.0%	77.6%
Mexico	Tonila	2000	83.8%	99.9%	25.2%
Mexico	Tonila	2017	96.5%	100.0%	71.7%
Mexico	Topia	2000	82.7%	96.4%	60.7%
Mexico	Topia	2017	96.3%	99.6%	85.8%
Mexico	Torreon	2000	96.2%	98.1%	91.6%
Mexico	Torreon	2017	99.8%	99.9%	99.5%
Mexico	Totatiche	2000	75.4%	98.8%	23.6%
Mexico	Totatiche	2017	93.5%	99.9%	59.3%
Mexico	Totolac	2000	98.7%	99.2%	98.0%
Mexico	Totolac	2017	99.9%	100.0%	99.9%
Mexico	Totolapa	2000	93.5%	97.8%	85.3%
Mexico	Totolapa	2017	99.2%	99.9%	96.8%
Mexico	Totolapan	2000	92.0%	99.3%	64.9%
Mexico	Totolapan	2017	99.2%	100.0%	96.1%
Mexico	Totoltepec De Guerrero	2000	81.2%	99.7%	27.0%
Mexico	Totoltepec De Guerrero	2017	96.3%	100.0%	76.5%
Mexico	Totontepec Villa De Morelos	2000	65.3%	94.6%	24.2%
Mexico	Totontepec Villa De Morelos	2017	90.8%	99.6%	56.0%
Mexico	Tototlan	2000	71.2%	98.5%	18.9%
Mexico	Tototlan	2017	93.6%	99.9%	58.3%
Mexico	Totutla	2000	87.3%	99.4%	48.7%
Mexico	Totutla	2017	97.7%	100.0%	85.4%
Mexico	Trancoso	2000	88.4%	96.9%	72.4%
Mexico	Trancoso	2017	98.7%	99.8%	94.2%
Mexico	Tres Valles	2000	56.1%	87.6%	17.8%
Mexico	Tres Valles	2017	83.2%	97.9%	47.3%
Mexico	Trincheras	2000	84.2%	99.2%	33.2%
Mexico	Trincheras	2017	96.1%	99.9%	73.0%
Mexico	Trinidad Garcia De La Cadena	2000	87.7%	98.8%	54.8%
Mexico	Trinidad Garcia De La Cadena	2017	97.2%	99.9%	86.5%
Mexico	Trinidad Zaachila	2000	59.3%	96.0%	11.2%
Mexico	Trinidad Zaachila	2017	92.7%	99.7%	65.7%
Mexico	Tubutama	2000	81.4%	99.1%	41.2%
Mexico	Tubutama	2017	95.2%	99.9%	67.9%
Mexico	Tula	2000	83.0%	93.8%	64.3%
Mexico	Tula	2017	97.0%	99.4%	89.7%
Mexico	Tula De Allende	2000	74.0%	89.0%	55.6%
Mexico	Tula De Allende	2017	94.7%	99.2%	87.9%
Mexico	Tulancingo De Bravo	2000	81.1%	86.9%	72.3%

Country	Second Administrative Unit	Year	Mean	Upper	Lower
Mexico	Tulancingo De Bravo	2017	98.0%	98.9%	95.9%
Mexico	Tulcingo	2000	92.4%	99.5%	78.4%
Mexico	Tulcingo	2017	98.1%	100.0%	88.8%
Mexico	Tultepec	2000	97.7%	99.0%	95.2%
Mexico	Tultepec	2017	99.8%	99.9%	99.7%
Mexico	Tultitlan	2000	98.3%	99.0%	97.3%
Mexico	Tultitlan	2017	99.9%	99.9%	99.8%
Mexico	Tumbala	2000	61.9%	80.1%	42.5%
Mexico	Tumbala	2017	93.5%	98.2%	83.5%
Mexico	Tumbiscatio	2000	78.5%	95.5%	47.5%
Mexico	Tumbiscatio	2017	95.1%	99.5%	78.3%
Mexico	Tunkas	2000	78.7%	99.6%	19.6%
Mexico	Tunkas	2017	95.3%	100.0%	63.9%
Mexico	Turicato	2000	77.3%	95.8%	40.9%
Mexico	Turicato	2017	94.0%	99.5%	69.5%
Mexico	Tuxcacuesco	2000	78.7%	98.5%	28.2%
Mexico	Tuxcacuesco	2017	94.4%	99.9%	58.8%
Mexico	Tuxcueca	2000	79.0%	99.6%	25.4%
Mexico	Tuxcueca	2017	94.7%	100.0%	67.4%
Mexico	Tuxpam	2000	64.2%	76.2%	48.7%
Mexico	Tuxpam	2017	94.6%	98.2%	85.9%
Mexico	Tuxpan	2000	84.3%	94.6%	68.3%
Mexico	Tuxpan	2000	73.0%	78.1%	66.7%
Mexico	Tuxpan	2000	67.2%	98.8%	14.2%
Mexico	Tuxpan	2017	97.4%	98.1%	95.9%
Mexico	Tuxpan	2017	98.1%	99.7%	92.6%
Mexico	Tuxpan	2017	92.8%	99.9%	60.3%
Mexico	Tuxtilla	2000	65.7%	99.4%	2.8%
Mexico	Tuxtilla	2017	89.8%	100.0%	31.2%
Mexico	Tuxtla Chico	2000	57.8%	84.9%	30.4%
Mexico	Tuxtla Chico	2017	92.2%	98.9%	70.9%
Mexico	Tuxtla Gutierrez	2000	50.3%	52.5%	47.4%
Mexico	Tuxtla Gutierrez	2017	83.0%	85.6%	80.4%
Mexico	Tuzamapan De Galeana	2000	58.2%	80.2%	27.6%
Mexico	Tuzamapan De Galeana	2017	88.3%	97.6%	58.4%
Mexico	Tuzantan	2000	51.6%	83.8%	21.6%
Mexico	Tuzantan	2017	89.2%	98.3%	71.2%
Mexico	Tuzantla	2000	74.9%	88.6%	56.5%
Mexico	Tuzantla	2017	95.7%	99.0%	87.9%
Mexico	Tzicatlacoyan	2000	76.7%	98.7%	38.1%
Mexico	Tzicatlacoyan	2017	94.2%	99.9%	74.9%
Mexico	Tzimol	2000	80.2%	97.7%	45.8%
Mexico	Tzimol	2017	96.7%	99.8%	85.0%
Mexico	Tzintzuntzan	2000	72.0%	94.7%	22.9%
Mexico	Tzintzuntzan	2017	93.7%	99.1%	69.4%
Mexico	Tzitzio	2000	78.6%	97.3%	43.4%
Mexico	Tzitzio	2017	94.8%	99.7%	77.3%
Mexico	Tzompantepec	2000	99.5%	99.8%	98.1%
Mexico	Tzompantepec	2017	100.0%	100.0%	99.9%
Mexico	Tzucacab	2000	69.7%	98.7%	15.9%
Mexico	Tzucacab	2017	89.8%	99.9%	30.2%
Mexico	Uayma	2000	77.6%	99.3%	11.1%
Mexico	Uayma	2017	93.4%	100.0%	33.6%
Mexico	Ucu	2000	84.0%	98.7%	35.0%
Mexico	Ucu	2017	97.6%	100.0%	82.3%
Mexico	Uman	2000	85.3%	91.0%	73.8%
Mexico	Uman	2017	98.6%	99.4%	96.2%

Country	Second Administrative Unit	Year	Mean	Upper	Lower
Mexico	Union De San Antonio	2000	76.0%	92.1%	40.4%
Mexico	Union De San Antonio	2017	93.3%	97.3%	81.7%
Mexico	Union De Tula	2000	82.1%	99.3%	29.2%
Mexico	Union De Tula	2017	95.3%	99.9%	68.5%
Mexico	Union Hidalgo	2000	55.6%	78.5%	20.3%
Mexico	Union Hidalgo	2017	93.6%	98.2%	78.1%
Mexico	Union Juarez	2000	94.0%	98.8%	78.1%
Mexico	Union Juarez	2017	99.3%	99.9%	96.6%
Mexico	Ures	2000	94.7%	99.1%	84.7%
Mexico	Ures	2017	99.1%	99.9%	96.3%
Mexico	Uriangato	2000	97.2%	99.5%	89.5%
Mexico	Uriangato	2017	99.8%	100.0%	98.8%
Mexico	Urique	2000	73.6%	88.4%	56.8%
Mexico	Urique	2017	93.7%	98.1%	85.6%
Mexico	Ursulo Galvan	2000	89.8%	95.7%	74.0%
Mexico	Ursulo Galvan	2017	98.7%	99.5%	96.2%
Mexico	Uruachi	2000	68.1%	79.4%	56.1%
Mexico	Uruachi	2017	93.6%	97.3%	86.8%
Mexico	Uruapan	2000	64.6%	72.5%	54.9%
Mexico	Uruapan	2017	94.0%	96.3%	90.0%
Mexico	Uxpanapa	2000	55.8%	80.7%	29.7%
Mexico	Uxpanapa	2017	84.8%	96.3%	65.8%
Mexico	Valerio Trujano	2000	65.8%	98.4%	7.8%
Mexico	Valerio Trujano	2017	93.0%	99.9%	56.6%
Mexico	Valladolid	2000	90.0%	96.3%	79.6%
Mexico	Valladolid	2017	97.9%	99.7%	93.6%
Mexico	Valle De Bravo	2000	88.6%	97.0%	66.6%
Mexico	Valle De Bravo	2017	98.3%	99.8%	91.8%
Mexico	Valle De Chalco Solidaridad	2000	99.2%	99.8%	97.3%
Mexico	Valle De Chalco Solidaridad	2017	99.9%	100.0%	99.7%
Mexico	Valle De Guadalupe	2000	78.2%	99.5%	17.5%
Mexico	Valle De Guadalupe	2017	94.4%	100.0%	54.4%
Mexico	Valle De Juarez	2000	78.7%	99.6%	24.2%
Mexico	Valle De Juarez	2017	95.6%	100.0%	71.7%
Mexico	Valle De Santiago	2000	88.5%	97.9%	59.7%
Mexico	Valle De Santiago	2017	98.5%	99.9%	92.8%
Mexico	Valle De Zaragoza	2000	86.5%	97.6%	56.3%
Mexico	Valle De Zaragoza	2017	97.8%	99.8%	92.0%
Mexico	Valle Hermoso	2000	89.8%	98.3%	66.8%
Mexico	Valle Hermoso	2017	98.9%	99.8%	95.9%
Mexico	Vallecillo	2000	79.4%	96.9%	47.8%
Mexico	Vallecillo	2017	95.1%	99.7%	76.9%
Mexico	Valparaiso	2000	75.5%	81.9%	67.9%
Mexico	Valparaiso	2017	92.0%	96.3%	87.0%

Country	Second Administrative Unit	Year	Mean	Upper	Lower
Mexico	Vanegas	2000	77.3%	93.9%	50.0%
Mexico	Vanegas	2017	96.2%	99.5%	86.6%
Mexico	Vega De Alatorre	2000	91.8%	99.1%	72.4%
Mexico	Vega De Alatorre	2017	98.4%	99.9%	94.4%
Mexico	Venado	2000	64.1%	79.0%	43.7%
Mexico	Venado	2017	94.6%	98.0%	86.4%
Mexico	Venustiano Carranza	2000	78.4%	94.6%	48.5%
Mexico	Venustiano Carranza	2000	76.0%	99.3%	18.0%
Mexico	Venustiano Carranza	2000	99.7%	99.8%	99.6%
Mexico	Venustiano Carranza	2000	77.2%	98.3%	27.9%
Mexico	Venustiano Carranza	2017	100.0%	100.0%	100.0%
Mexico	Venustiano Carranza	2017	95.8%	99.6%	80.9%
Mexico	Venustiano Carranza	2017	93.3%	99.9%	51.8%
Mexico	Venustiano Carranza	2017	95.2%	99.9%	68.5%
Mexico	Veracruz	2000	86.1%	92.8%	74.6%
Mexico	Veracruz	2017	98.4%	99.4%	95.5%
Mexico	Vetagrande	2000	92.3%	98.9%	73.8%
Mexico	Vetagrande	2017	99.2%	99.9%	96.2%
Mexico	Vicente Guerrero	2000	90.5%	97.7%	76.3%
Mexico	Vicente Guerrero	2000	75.1%	94.8%	35.6%
Mexico	Vicente Guerrero	2017	98.9%	99.8%	96.1%
Mexico	Vicente Guerrero	2017	95.3%	99.7%	68.5%
Mexico	Victoria	2000	80.5%	97.8%	43.1%
Mexico	Victoria	2000	88.0%	95.2%	77.3%
Mexico	Victoria	2017	96.0%	99.8%	81.3%
Mexico	Victoria	2017	98.6%	99.6%	94.3%
Mexico	Viesca	2000	91.2%	97.3%	79.4%
Mexico	Viesca	2017	98.7%	99.8%	94.7%
Mexico	Villa Aldama	2000	69.1%	84.8%	45.3%
Mexico	Villa Aldama	2017	96.6%	98.9%	91.2%
Mexico	Villa Comaltitlan	2000	63.5%	82.9%	33.2%
Mexico	Villa Comaltitlan	2017	92.8%	98.9%	75.5%
Mexico	Villa Corona	2000	90.7%	98.1%	72.4%
Mexico	Villa Corona	2017	98.7%	99.9%	93.8%
Mexico	Villa Corzo	2000	70.0%	85.7%	48.9%
Mexico	Villa Corzo	2017	93.6%	98.4%	82.5%
Mexico	Villa De Allende	2000	34.1%	69.2%	9.0%
Mexico	Villa De Allende	2017	59.5%	89.0%	26.6%
Mexico	Villa De Alvarez	2000	97.6%	98.7%	95.8%
Mexico	Villa De Alvarez	2017	99.8%	99.9%	99.6%
Mexico	Villa De Arista	2000	74.4%	96.4%	31.6%

Country	Second Administrative Unit	Year	Mean	Upper	Lower
Mexico	Villa De Arista	2017	95.7%	99.6%	80.5%
Mexico	Villa De Arriaga	2000	77.5%	96.8%	40.1%
Mexico	Villa De Arriaga	2017	94.8%	99.7%	78.2%
Mexico	Villa De Chilapa De Diaz	2000	80.3%	99.7%	27.4%
Mexico	Villa De Chilapa De Diaz	2017	96.7%	100.0%	74.4%
Mexico	Villa De Cos	2000	89.0%	95.1%	79.8%
Mexico	Villa De Cos	2017	98.3%	99.5%	95.5%
Mexico	Villa De Etla	2000	54.2%	64.0%	43.8%
Mexico	Villa De Etla	2017	95.0%	96.8%	91.1%
Mexico	Villa De Guadalupe	2000	80.7%	95.8%	55.3%
Mexico	Villa De Guadalupe	2017	95.7%	99.5%	83.4%
Mexico	Villa De La Paz	2000	73.4%	98.1%	18.5%
Mexico	Villa De La Paz	2017	94.9%	99.8%	63.6%
Mexico	Villa De Ramos	2000	78.1%	90.3%	61.7%
Mexico	Villa De Ramos	2017	96.4%	99.1%	89.6%
Mexico	Villa De Reyes	2000	67.0%	85.8%	42.7%
Mexico	Villa De Reyes	2017	93.6%	98.4%	80.2%
Mexico	Villa De Tamazulapam Del Progreso	2000	72.8%	99.6%	13.8%
Mexico	Villa De Tamazulapam Del Progreso	2017	92.8%	100.0%	42.7%
Mexico	Villa De Tezontepec	2000	88.7%	99.9%	34.4%
Mexico	Villa De Tezontepec	2017	97.7%	100.0%	86.2%
Mexico	Villa De Tututepec De Melchor Ocampo	2000	67.4%	90.9%	33.3%
Mexico	Villa De Tututepec De Melchor Ocampo	2017	90.7%	99.2%	62.8%
Mexico	Villa De Zaachila	2000	60.7%	93.5%	16.2%
Mexico	Villa De Zaachila	2017	91.9%	99.2%	61.9%
Mexico	Villa Del Carbon	2000	85.7%	96.6%	69.7%
Mexico	Villa Del Carbon	2017	97.9%	99.7%	91.3%
Mexico	Villa Diaz Ordaz	2000	88.3%	98.2%	65.0%
Mexico	Villa Diaz Ordaz	2017	98.6%	99.9%	91.8%
Mexico	Villa Garcia	2000	83.7%	98.8%	42.2%
Mexico	Villa Garcia	2017	97.0%	99.9%	82.6%
Mexico	Villa Gonzalez Ortega	2000	88.5%	97.5%	60.7%

Country	Second Administrative Unit	Year	Mean	Upper	Lower
Mexico	Villa Gonzalez Ortega	2017	98.1%	99.8%	90.2%
Mexico	Villa Guerrero	2000	41.5%	74.0%	17.4%
Mexico	Villa Guerrero	2000	85.8%	98.4%	50.9%
Mexico	Villa Guerrero	2017	97.2%	99.9%	81.9%
Mexico	Villa Guerrero	2017	86.9%	97.6%	64.3%
Mexico	Villa Hidalgo	2000	83.1%	99.7%	31.7%
Mexico	Villa Hidalgo	2000	75.6%	99.5%	12.1%
Mexico	Villa Hidalgo	2000	74.8%	89.9%	48.9%
Mexico	Villa Hidalgo	2000	81.6%	95.1%	59.1%
Mexico	Villa Hidalgo	2000	80.8%	99.6%	24.3%
Mexico	Villa Hidalgo	2017	95.2%	99.1%	82.1%
Mexico	Villa Hidalgo	2017	95.6%	100.0%	62.3%
Mexico	Villa Hidalgo	2017	96.4%	100.0%	74.4%
Mexico	Villa Hidalgo	2017	93.9%	100.0%	50.3%
Mexico	Villa Hidalgo	2017	97.0%	99.7%	87.8%
Mexico	Villa Juarez	2000	68.9%	90.3%	42.8%
Mexico	Villa Juarez	2017	93.7%	99.3%	80.6%
Mexico	Villa Pesqueira	2000	88.7%	99.5%	57.0%
Mexico	Villa Pesqueira	2017	98.7%	99.9%	92.5%
Mexico	Villa Purificacion	2000	77.7%	96.0%	38.5%
Mexico	Villa Purificacion	2017	94.1%	99.6%	60.1%
Mexico	Villa Sola De Vega	2000	82.8%	94.9%	64.1%
Mexico	Villa Sola De Vega	2017	96.6%	99.3%	89.1%
Mexico	Villa Talea De Castro	2000	68.6%	99.4%	8.4%
Mexico	Villa Talea De Castro	2017	91.6%	100.0%	50.6%
Mexico	Villa Tejupam De La Union	2000	64.5%	90.6%	30.4%
Mexico	Villa Tejupam De La Union	2017	93.8%	98.6%	82.8%
Mexico	Villa Union	2000	91.3%	98.6%	66.4%
Mexico	Villa Union	2017	99.0%	99.9%	93.9%
Mexico	Villa Victoria	2000	46.7%	64.0%	30.3%
Mexico	Villa Victoria	2017	74.6%	89.0%	58.5%
Mexico	Villaflores	2000	69.1%	89.8%	39.4%
Mexico	Villaflores	2017	92.9%	98.9%	78.9%
Mexico	Villagran	2000	71.5%	89.2%	34.8%
Mexico	Villagran	2000	79.9%	96.4%	55.3%
Mexico	Villagran	2017	95.2%	98.6%	85.4%
Mexico	Villagran	2017	96.3%	99.7%	87.6%
Mexico	Villaldama	2000	91.0%	99.5%	64.6%
Mexico	Villaldama	2017	99.0%	100.0%	94.0%
Mexico	Villamar	2000	49.4%	81.0%	21.3%
Mexico	Villamar	2017	78.5%	97.7%	48.8%
Mexico	Villanueva	2000	82.0%	90.4%	68.9%
Mexico	Villanueva	2017	96.7%	99.0%	90.0%
Mexico	Vista Hermosa	2000	91.8%	96.2%	86.3%
Mexico	Vista Hermosa	2017	98.2%	99.7%	92.8%
Mexico	Xalapa	2000	65.1%	71.8%	54.7%
Mexico	Xalapa	2017	85.9%	92.0%	82.0%
Mexico	Xalatlaco	2000	44.1%	91.6%	2.5%
Mexico	Xalatlaco	2017	81.6%	99.5%	27.9%

Country	Second Administrative Unit	Year	Mean	Upper	Lower
Mexico	Xalisco	2000	96.7%	98.8%	93.2%
Mexico	Xalisco	2017	99.6%	99.9%	98.4%
Mexico	Xaloztoc	2000	96.2%	99.9%	79.9%
Mexico	Xaloztoc	2017	99.5%	100.0%	96.2%
Mexico	Xalpatlahuac	2000	48.1%	73.0%	35.0%
Mexico	Xalpatlahuac	2017	80.6%	95.9%	60.0%
Mexico	Xaltocan	2000	99.9%	100.0%	99.7%
Mexico	Xaltocan	2017	100.0%	100.0%	100.0%
Mexico	Xayacatlan De Bravo	2000	71.2%	99.8%	4.3%
Mexico	Xayacatlan De Bravo	2017	90.3%	100.0%	24.1%
Mexico	Xichu	2000	66.8%	87.0%	45.0%
Mexico	Xichu	2017	93.0%	98.7%	81.1%
Mexico	Xico	2000	96.9%	99.6%	90.4%
Mexico	Xico	2017	98.9%	99.8%	95.2%
Mexico	Xicohtzinco	2000	95.1%	96.7%	92.3%
Mexico	Xicohtzinco	2017	99.5%	99.6%	99.3%
Mexico	Xicotencatl	2000	93.4%	98.7%	82.9%
Mexico	Xicotencatl	2017	99.1%	99.9%	95.6%
Mexico	Xicotepec	2000	64.9%	73.5%	53.1%
Mexico	Xicotepec	2017	94.5%	97.1%	91.2%
Mexico	Xicotlan	2000	84.5%	99.5%	38.0%
Mexico	Xicotlan	2017	96.8%	100.0%	79.8%
Mexico	Xilitla	2000	72.3%	86.5%	51.4%
Mexico	Xilitla	2017	94.7%	98.9%	87.0%
Mexico	Xiutetelco	2000	86.7%	93.7%	76.4%
Mexico	Xiutetelco	2017	98.9%	99.7%	95.8%
Mexico	Xocchel	2000	95.4%	99.9%	80.1%
Mexico	Xocchel	2017	99.7%	100.0%	98.6%
Mexico	Xochiapulco	2000	84.3%	99.9%	33.1%
Mexico	Xochiapulco	2017	96.5%	100.0%	72.3%
Mexico	Xochiatipan	2000	66.3%	98.7%	14.0%
Mexico	Xochiatipan	2017	89.7%	99.9%	50.3%
Mexico	Xochicoatlan	2000	86.5%	98.5%	59.3%
Mexico	Xochicoatlan	2017	97.1%	99.9%	85.9%
Mexico	Xochihuehuetlan	2000	77.1%	99.3%	23.0%
Mexico	Xochihuehuetlan	2017	95.0%	99.9%	71.3%
Mexico	Xochiltepec	2000	81.7%	99.9%	18.0%
Mexico	Xochiltepec	2017	96.5%	100.0%	75.6%
Mexico	Xochimilco	2000	97.8%	99.5%	92.2%
Mexico	Xochimilco	2017	99.8%	100.0%	98.8%
Mexico	Xochistlahuaca	2000	84.6%	95.5%	64.4%
Mexico	Xochistlahuaca	2017	96.7%	98.8%	91.6%
Mexico	Xochitepec	2000	89.4%	94.0%	82.8%
Mexico	Xochitepec	2017	98.9%	99.6%	96.6%
Mexico	Xochitlan De Vicente Suarez	2000	63.1%	94.6%	14.8%
Mexico	Xochitlan De Vicente Suarez	2017	92.0%	99.6%	60.8%
Mexico	Xochitlan Todos Santos	2000	56.2%	65.3%	46.4%
Mexico	Xochitlan Todos Santos	2017	94.9%	97.4%	89.7%
Mexico	Xonacatlan	2000	92.9%	99.8%	67.8%
Mexico	Xonacatlan	2017	98.4%	100.0%	88.6%
Mexico	Xoxocotla	2000	73.9%	99.8%	10.9%
Mexico	Xoxocotla	2017	94.7%	100.0%	61.1%
Mexico	Yahualica	2000	24.2%	39.9%	9.3%
Mexico	Yahualica	2017	51.5%	74.4%	27.8%

Country	Second Administrative Unit	Year	Mean	Upper	Lower
Mexico	Yahualica De Gonzalez Gallo	2000	89.0%	97.3%	72.2%
Mexico	Yahualica De Gonzalez Gallo	2017	98.7%	99.8%	95.2%
Mexico	Yajalon	2000	70.9%	90.4%	48.4%
Mexico	Yajalon	2017	95.4%	99.1%	86.1%
Mexico	Yanga	2000	72.1%	95.8%	29.9%
Mexico	Yanga	2017	95.7%	99.7%	77.9%
Mexico	Yaonahuac	2000	85.1%	98.2%	52.6%
Mexico	Yaonahuac	2017	97.8%	99.9%	85.3%
Mexico	Yauhquemecan	2000	98.6%	99.4%	97.4%
Mexico	Yauhquemecan	2017	99.9%	100.0%	99.9%
Mexico	Yautepec	2000	86.4%	97.4%	59.6%
Mexico	Yautepec	2017	98.5%	99.8%	93.5%
Mexico	Yaxcaba	2000	75.8%	94.5%	48.4%
Mexico	Yaxcaba	2017	93.9%	99.6%	79.4%
Mexico	Yaxe	2000	74.8%	100.0%	3.1%
Mexico	Yaxe	2017	91.9%	100.0%	27.0%
Mexico	Yaxkukul	2000	85.0%	99.5%	36.2%
Mexico	Yaxkukul	2017	98.2%	100.0%	85.3%
Mexico	Yecapixtla	2000	91.4%	97.4%	64.9%
Mexico	Yecapixtla	2017	98.9%	99.8%	90.6%
Mexico	Yecora	2000	83.2%	97.9%	48.3%
Mexico	Yecora	2017	96.7%	99.8%	84.3%
Mexico	Yecuatla	2000	62.5%	81.4%	37.0%
Mexico	Yecuatla	2017	93.0%	97.2%	83.4%
Mexico	Yehualtepec	2000	63.0%	93.7%	16.8%
Mexico	Yehualtepec	2017	92.2%	99.5%	60.9%
Mexico	Yobain	2000	80.4%	99.9%	6.9%
Mexico	Yobain	2017	93.7%	100.0%	34.0%
Mexico	Yogana	2000	73.9%	98.9%	16.2%
Mexico	Yogana	2017	95.0%	99.9%	70.1%
Mexico	Yurecuaro	2000	79.3%	98.8%	25.0%
Mexico	Yurecuaro	2017	95.3%	99.9%	76.6%
Mexico	Yuriria	2000	87.3%	96.9%	69.3%
Mexico	Yuriria	2017	97.4%	99.7%	87.6%
Mexico	Yutanduchi De Guerrero	2000	64.8%	97.7%	12.4%
Mexico	Yutanduchi De Guerrero	2017	92.5%	99.9%	56.2%
Mexico	Zacapala	2000	77.0%	96.4%	43.0%
Mexico	Zacapala	2017	95.9%	99.6%	80.7%
Mexico	Zacapoaxtla	2000	81.5%	99.5%	28.6%
Mexico	Zacapoaxtla	2017	95.5%	100.0%	64.1%
Mexico	Zacapu	2000	88.8%	93.1%	82.1%
Mexico	Zacapu	2017	97.4%	99.4%	94.9%
Mexico	Zacatecas	2000	97.8%	98.7%	95.9%
Mexico	Zacatecas	2017	99.8%	99.9%	99.7%
Mexico	Zacatelco	2000	97.0%	98.2%	94.6%
Mexico	Zacatelco	2017	99.7%	99.8%	99.6%
Mexico	Zacatepec De Hidalgo	2000	95.5%	97.8%	90.5%
Mexico	Zacatepec De Hidalgo	2017	99.6%	99.8%	98.7%
Mexico	Zacatlan	2000	82.9%	93.2%	67.6%
Mexico	Zacatlan	2017	97.2%	99.5%	91.1%
Mexico	Zacazonapan	2000	74.8%	99.3%	14.8%
Mexico	Zacazonapan	2017	95.0%	100.0%	66.0%
Mexico	Zacoalco De Torres	2000	78.7%	99.2%	27.6%

Country	Second Administrative Unit	Year	Mean	Upper	Lower
Mexico	Zacoalco De Torres	2017	95.2%	99.9%	57.3%
Mexico	Zacualpan	2000	76.8%	98.6%	28.4%
Mexico	Zacualpan	2000	74.1%	98.1%	32.8%
Mexico	Zacualpan	2017	93.9%	99.9%	72.5%
Mexico	Zacualpan	2017	93.8%	99.9%	71.6%
Mexico	Zacualpan De Amilpas	2000	76.9%	84.2%	69.0%
Mexico	Zacualpan De Amilpas	2017	98.0%	98.7%	97.0%
Mexico	Zacualtipan De Angeles	2000	81.6%	98.6%	35.5%
Mexico	Zacualtipan De Angeles	2017	97.2%	99.9%	81.3%
Mexico	Zamora	2000	82.2%	92.7%	63.4%
Mexico	Zamora	2017	97.8%	99.4%	94.3%
Mexico	Zapopan	2000	79.6%	84.1%	74.0%
Mexico	Zapopan	2017	96.2%	98.2%	93.6%
Mexico	Zapotiltic	2000	81.7%	92.7%	62.8%
Mexico	Zapotiltic	2017	98.0%	99.5%	93.0%
Mexico	Zapotitlan	2000	78.4%	97.8%	39.0%
Mexico	Zapotitlan	2017	94.3%	99.8%	74.5%
Mexico	Zapotitlan De Mendez	2000	78.0%	89.3%	56.3%
Mexico	Zapotitlan De Mendez	2017	98.2%	99.3%	94.7%
Mexico	Zapotitlan De Vadillo	2000	92.0%	99.3%	72.7%
Mexico	Zapotitlan De Vadillo	2017	98.8%	99.9%	92.6%
Mexico	Zapotitlan Del Rio	2000	71.3%	98.2%	24.6%
Mexico	Zapotitlan Del Rio	2017	92.5%	99.8%	65.9%
Mexico	Zapotitlan Lagunas	2000	75.7%	99.2%	19.5%
Mexico	Zapotitlan Lagunas	2017	93.8%	99.9%	57.4%
Mexico	Zapotitlan Palmas	2000	68.0%	99.2%	7.4%
Mexico	Zapotitlan Palmas	2017	92.7%	99.9%	53.9%
Mexico	Zapotitlan Tablas	2000	30.7%	38.2%	21.9%
Mexico	Zapotitlan Tablas	2017	70.9%	82.2%	60.9%
Mexico	Zapotlan De Juarez	2000	94.1%	100.0%	58.3%
Mexico	Zapotlan De Juarez	2017	98.8%	100.0%	85.2%
Mexico	Zapotlan Del Rey	2000	77.4%	93.7%	53.2%
Mexico	Zapotlan Del Rey	2017	95.1%	99.3%	84.4%
Mexico	Zapotlan El Grande	2000	93.4%	98.0%	84.7%
Mexico	Zapotlan El Grande	2017	99.3%	99.9%	97.2%
Mexico	Zapotlanejo	2000	77.7%	95.6%	34.9%
Mexico	Zapotlanejo	2017	94.4%	99.6%	76.2%
Mexico	Zaragoza	2000	83.9%	99.9%	20.7%
Mexico	Zaragoza	2000	65.7%	92.8%	29.4%

Country	Second Administrative Unit	Year	Mean	Upper	Lower
Mexico	Zaragoza	2000	91.4%	98.6%	60.5%
Mexico	Zaragoza	2000	64.9%	95.5%	21.5%
Mexico	Zaragoza	2017	96.0%	100.0%	66.2%
Mexico	Zaragoza	2017	92.1%	99.4%	72.7%
Mexico	Zaragoza	2017	99.0%	99.9%	94.8%
Mexico	Zaragoza	2017	93.1%	99.7%	66.4%
Mexico	Zautla	2000	87.9%	99.4%	61.5%
Mexico	Zautla	2017	98.6%	100.0%	94.4%
Mexico	Zempoala	2000	91.5%	99.6%	74.4%
Mexico	Zempoala	2017	98.3%	100.0%	91.0%
Mexico	Zentla	2000	75.5%	88.1%	54.7%
Mexico	Zentla	2017	95.7%	98.8%	89.0%
Mexico	Zihuateutla	2000	61.8%	79.6%	31.1%
Mexico	Zihuateutla	2017	87.8%	96.2%	71.8%
Mexico	Zimapan	2000	79.7%	97.1%	42.9%
Mexico	Zimapan	2017	95.5%	99.8%	77.4%
Mexico	Zimatlan De Alvarez	2000	69.8%	95.6%	28.4%
Mexico	Zimatlan De Alvarez	2017	93.3%	99.5%	68.2%
Mexico	Zinacantan	2000	73.2%	89.5%	52.1%
Mexico	Zinacantan	2017	96.8%	99.2%	88.7%
Mexico	Zinacantepec	2000	67.3%	84.3%	50.0%
Mexico	Zinacantepec	2017	92.1%	98.5%	83.4%
Mexico	Zinacatepec	2000	91.0%	99.4%	54.8%
Mexico	Zinacatepec	2017	98.8%	100.0%	94.6%
Mexico	Zinaparo	2000	77.8%	99.3%	24.3%
Mexico	Zinaparo	2017	95.7%	100.0%	65.8%
Mexico	Zinapecuaro	2000	78.0%	97.1%	48.1%
Mexico	Zinapecuaro	2017	95.8%	99.8%	86.3%
Mexico	Ziracuaretiro	2000	20.5%	54.6%	2.4%
Mexico	Ziracuaretiro	2017	60.9%	89.3%	24.0%
Mexico	Zirandaro	2000	69.4%	90.6%	41.2%
Mexico	Zirandaro	2017	91.7%	99.0%	73.6%
Mexico	Zitacuaro	2000	90.2%	96.3%	78.9%
Mexico	Zitacuaro	2017	98.6%	99.7%	94.2%
Mexico	Zitlala	2000	62.7%	92.6%	23.2%
Mexico	Zitlala	2017	92.1%	99.5%	64.7%
Mexico	Zitlaltepec De Trinidad Sanchez Santos	2000	97.8%	99.9%	88.7%
Mexico	Zitlaltepec De Trinidad Sanchez Santos	2017	99.8%	100.0%	98.9%
Mexico	Zongolica	2000	63.5%	88.8%	28.4%
Mexico	Zongolica	2017	91.5%	98.9%	71.7%
Mexico	Zongozotla	2000	84.4%	97.9%	54.6%
Mexico	Zongozotla	2017	97.6%	99.9%	88.5%
Mexico	Zontecomatlan De Lopez Y Fuentes	2000	72.5%	97.1%	26.5%
Mexico	Zontecomatlan De Lopez Y Fuentes	2017	93.7%	99.8%	67.6%
Mexico	Zoquiapan	2000	69.7%	84.6%	49.0%
Mexico	Zoquiapan	2017	95.0%	98.9%	85.3%
Mexico	Zoquitlan	2000	54.9%	78.5%	28.8%
Mexico	Zoquitlan	2017	89.9%	98.0%	66.0%
Mexico	Zozocolco De Hidalgo	2000	13.4%	29.4%	3.2%

Country	Second Administrative Unit	Year	Mean	Upper	Lower
Mexico	Zozocolco De Hidalgo	2017	41.2%	75.0%	16.4%
Mexico	Zumpahuacan	2000	79.1%	97.2%	48.9%
Mexico	Zumpahuacan	2017	94.8%	99.2%	81.4%
Mexico	Zumpango	2000	90.4%	95.8%	83.0%
Mexico	Zumpango	2017	98.8%	99.7%	96.4%
Nicaragua	Achuapa	2000	71.4%	97.0%	33.7%
Nicaragua	Achuapa	2017	73.0%	98.0%	35.2%
Nicaragua	Acoyapa	2000	51.1%	77.1%	20.9%
Nicaragua	Acoyapa	2017	55.0%	80.1%	27.7%
Nicaragua	Altagracia	2000	60.4%	95.6%	15.9%
Nicaragua	Altagracia	2017	64.1%	95.4%	22.2%
Nicaragua	Belén	2000	52.1%	85.1%	9.8%
Nicaragua	Belén	2017	53.5%	85.1%	13.2%
Nicaragua	Bluefields	2000	55.0%	74.2%	33.8%
Nicaragua	Bluefields	2017	57.3%	71.4%	42.3%
Nicaragua	Boaco	2000	53.1%	82.2%	22.6%
Nicaragua	Boaco	2017	56.1%	83.9%	25.7%
Nicaragua	Bocana de Paiwas	2000	58.2%	77.9%	37.8%
Nicaragua	Bocana de Paiwas	2017	60.5%	80.1%	40.1%
Nicaragua	Bonanza	2000	57.5%	76.4%	34.3%
Nicaragua	Bonanza	2017	59.6%	76.8%	38.9%
Nicaragua	Buenos Aires	2000	9.1%	14.5%	0.3%
Nicaragua	Buenos Aires	2017	8.6%	20.8%	1.2%
Nicaragua	Camoapa	2000	54.8%	82.1%	26.2%
Nicaragua	Camoapa	2017	57.7%	82.4%	31.7%
Nicaragua	Catarina	2000	99.9%	100.0%	99.7%
Nicaragua	Catarina	2017	99.9%	100.0%	99.8%
Nicaragua	Chichigalpa	2000	53.1%	81.8%	10.6%
Nicaragua	Chichigalpa	2017	56.8%	82.9%	11.5%
Nicaragua	Chinandega	2000	50.5%	84.6%	14.4%
Nicaragua	Chinandega	2017	53.2%	86.4%	13.7%
Nicaragua	Cinco Pinos	2000	76.8%	100.0%	38.8%
Nicaragua	Cinco Pinos	2017	79.8%	100.0%	51.4%
Nicaragua	Ciudad Antigua	2000	53.5%	92.3%	15.7%
Nicaragua	Ciudad Antigua	2017	57.1%	92.5%	20.1%
Nicaragua	Ciudad Darío	2000	58.7%	88.0%	23.7%
Nicaragua	Ciudad Darío	2017	61.6%	89.3%	26.8%
Nicaragua	Ciudad Sandino	2000	55.5%	85.0%	16.2%
Nicaragua	Ciudad Sandino	2017	60.0%	86.1%	25.3%
Nicaragua	Comalapa	2000	59.1%	89.5%	33.2%
Nicaragua	Comalapa	2017	61.4%	89.9%	34.9%
Nicaragua	Condega	2000	79.9%	96.8%	57.4%
Nicaragua	Condega	2017	82.0%	97.9%	61.7%
Nicaragua	Corinto	2000	62.3%	98.7%	28.4%
Nicaragua	Corinto	2017	62.2%	98.7%	23.8%
Nicaragua	Dipilto	2000	30.1%	61.5%	0.0%
Nicaragua	Dipilto	2017	34.0%	64.6%	0.0%
Nicaragua	Diriá	2000	92.9%	100.0%	81.0%
Nicaragua	Diriá	2017	95.2%	100.0%	85.6%
Nicaragua	Diriamba	2000	63.6%	84.7%	42.8%
Nicaragua	Diriamba	2017	68.1%	89.5%	44.3%
Nicaragua	Diriomo	2000	88.7%	100.0%	73.8%
Nicaragua	Diriomo	2017	91.5%	100.0%	78.9%
Nicaragua	Dolores	2000	77.3%	100.0%	21.3%
Nicaragua	Dolores	2017	81.5%	100.0%	19.7%

Country	Second Administrative Unit	Year	Mean	Upper	Lower
Nicaragua	El Almendro	2000	47.7%	69.4%	25.2%
Nicaragua	El Almendro	2017	49.5%	72.2%	25.9%
Nicaragua	El Cuá	2000	55.8%	70.9%	41.6%
Nicaragua	El Cuá	2017	58.3%	72.5%	43.7%
Nicaragua	El Jicaral	2000	62.4%	91.6%	31.9%
Nicaragua	El Jicaral	2017	64.7%	92.6%	36.1%
Nicaragua	El Rama	2000	55.5%	68.0%	41.4%
Nicaragua	El Rama	2017	58.5%	71.6%	45.0%
Nicaragua	El Realejo	2000	42.5%	86.5%	6.2%
Nicaragua	El Realejo	2017	44.6%	89.3%	9.9%
Nicaragua	El Rosario	2000	89.2%	100.0%	43.6%
Nicaragua	El Rosario	2017	90.7%	100.0%	42.5%
Nicaragua	El Sauce	2000	61.6%	87.4%	31.7%
Nicaragua	El Sauce	2017	65.8%	89.3%	36.9%
Nicaragua	El Viejo	2000	55.5%	84.3%	25.9%
Nicaragua	El Viejo	2017	58.2%	83.8%	30.0%
Nicaragua	Esquipulas	2000	57.0%	70.1%	42.4%
Nicaragua	Esquipulas	2017	63.5%	76.6%	48.5%
Nicaragua	Estelí	2000	68.3%	87.7%	42.8%
Nicaragua	Estelí	2017	71.9%	89.4%	50.2%
Nicaragua	Granada	2000	68.8%	88.9%	34.9%
Nicaragua	Granada	2017	72.6%	91.1%	41.4%
Nicaragua	Jalapa	2000	60.7%	89.5%	29.1%
Nicaragua	Jalapa	2017	63.7%	91.2%	33.5%
Nicaragua	Jinotega	2000	57.5%	83.6%	27.3%
Nicaragua	Jinotega	2017	60.1%	84.2%	30.8%
Nicaragua	Jinotepe	2000	76.2%	99.1%	20.1%
Nicaragua	Jinotepe	2017	79.1%	99.8%	19.0%
Nicaragua	Juigalpa	2000	56.1%	84.7%	25.2%
Nicaragua	Juigalpa	2017	59.0%	85.7%	25.5%
Nicaragua	Kukra Hill	2000	53.2%	80.8%	22.6%
Nicaragua	Kukra Hill	2017	56.0%	78.9%	30.0%
Nicaragua	La Concepción	2000	66.4%	100.0%	19.9%
Nicaragua	La Concepción	2017	70.6%	100.0%	17.7%
Nicaragua	La Concordia	2000	62.0%	98.9%	20.4%
Nicaragua	La Concordia	2017	65.6%	100.0%	26.8%
Nicaragua	La Conquista	2000	85.8%	100.0%	64.5%
Nicaragua	La Conquista	2017	89.5%	100.0%	69.9%
Nicaragua	La Cruz de Río Grande	2000	54.9%	66.9%	43.3%
Nicaragua	La Cruz de Río Grande	2017	57.4%	69.5%	45.8%
Nicaragua	La Libertad	2000	55.5%	85.4%	20.4%
Nicaragua	La Libertad	2017	58.0%	86.9%	21.7%
Nicaragua	La Paz Centro	2000	56.6%	81.7%	23.5%
Nicaragua	La Paz Centro	2017	59.6%	84.2%	23.9%
Nicaragua	La Paz de Carazo	2000	99.5%	100.0%	97.1%
Nicaragua	La Paz de Carazo	2017	99.5%	100.0%	96.4%
Nicaragua	La Trinidad	2000	49.8%	77.3%	16.8%
Nicaragua	La Trinidad	2017	54.2%	79.9%	20.3%
Nicaragua	Lago de Managua	2000	57.6%	97.9%	5.4%
Nicaragua	Lago de Managua	2017	59.7%	98.3%	7.3%
Nicaragua	Lago de Nicaragua	2000	55.9%	79.1%	28.7%
Nicaragua	Lago de Nicaragua	2017	59.3%	79.9%	30.7%

Country	Second Administrative Unit	Year	Mean	Upper	Lower
Nicaragua	Laguna de Perlas	2000	54.3%	71.6%	36.1%
Nicaragua	Laguna de Perlas	2017	56.8%	72.9%	39.2%
Nicaragua	Larreynaga-Malpaisillo	2000	62.3%	85.5%	37.8%
Nicaragua	Larreynaga-Malpaisillo	2017	64.2%	86.7%	39.1%
Nicaragua	Las Sabanas	2000	46.9%	82.4%	25.1%
Nicaragua	Las Sabanas	2017	42.9%	76.6%	21.6%
Nicaragua	León	2000	49.0%	80.7%	15.7%
Nicaragua	León	2017	51.6%	84.6%	16.3%
Nicaragua	Macuelizo	2000	42.8%	76.2%	11.7%
Nicaragua	Macuelizo	2017	46.1%	79.7%	15.0%
Nicaragua	Managua	2000	67.8%	97.1%	30.4%
Nicaragua	Managua	2017	70.5%	97.6%	32.6%
Nicaragua	Masatepe	2000	71.3%	100.0%	45.9%
Nicaragua	Masatepe	2017	74.6%	100.0%	45.9%
Nicaragua	Masaya	2000	76.6%	98.8%	55.1%
Nicaragua	Masaya	2017	78.9%	98.9%	58.3%
Nicaragua	Matagalpa	2000	60.9%	88.8%	34.3%
Nicaragua	Matagalpa	2017	64.0%	89.3%	36.8%
Nicaragua	Mateare	2000	64.9%	97.2%	26.7%
Nicaragua	Mateare	2017	66.9%	98.4%	26.8%
Nicaragua	Matiguás	2000	54.8%	74.3%	32.7%
Nicaragua	Matiguás	2017	57.4%	76.8%	35.9%
Nicaragua	Morrito	2000	45.2%	67.1%	23.1%
Nicaragua	Morrito	2017	48.5%	71.4%	27.2%
Nicaragua	Moyogalpa	2000	68.8%	97.1%	11.1%
Nicaragua	Moyogalpa	2017	71.7%	97.2%	13.4%
Nicaragua	Mozonte	2000	53.5%	90.3%	12.5%
Nicaragua	Mozonte	2017	59.1%	92.2%	18.1%
Nicaragua	Muelle de los Bueyes	2000	52.0%	72.8%	30.2%
Nicaragua	Muelle de los Bueyes	2017	53.2%	73.0%	32.3%
Nicaragua	Murra	2000	47.0%	78.7%	18.4%
Nicaragua	Murra	2017	50.6%	82.0%	20.9%
Nicaragua	Muy Muy	2000	47.6%	79.9%	11.1%
Nicaragua	Muy Muy	2017	48.7%	78.6%	11.1%
Nicaragua	Nagarote	2000	55.0%	87.1%	19.4%
Nicaragua	Nagarote	2017	57.4%	90.4%	19.9%
Nicaragua	Nandaime	2000	65.1%	92.9%	36.5%
Nicaragua	Nandaime	2017	69.0%	94.1%	43.4%
Nicaragua	Nandasmo	2000	81.0%	100.0%	67.9%
Nicaragua	Nandasmo	2017	83.4%	100.0%	70.7%
Nicaragua	Nindirí	2000	68.9%	100.0%	36.0%
Nicaragua	Nindirí	2017	70.5%	100.0%	35.3%
Nicaragua	Niquinohomo	2000	100.0%	100.0%	100.0%
Nicaragua	Niquinohomo	2017	100.0%	100.0%	100.0%
Nicaragua	Nueva Guinea	2000	56.2%	73.6%	37.3%
Nicaragua	Nueva Guinea	2017	58.8%	74.4%	40.7%
Nicaragua	Ocotal	2000	69.0%	95.8%	26.2%
Nicaragua	Ocotal	2017	74.4%	97.0%	25.3%
Nicaragua	Palacagüina	2000	58.5%	81.3%	45.2%
Nicaragua	Palacagüina	2017	60.9%	85.7%	47.6%
Nicaragua	Posoltega	2000	65.7%	100.0%	7.2%
Nicaragua	Posoltega	2017	73.0%	100.0%	10.8%
Nicaragua	Potosí	2000	42.0%	61.8%	11.4%
Nicaragua	Potosí	2017	41.3%	64.3%	13.6%
Nicaragua	Prinzapolka	2000	55.6%	65.8%	43.9%
Nicaragua	Prinzapolka	2017	58.2%	67.1%	46.4%

Country	Second Administrative Unit	Year	Mean	Upper	Lower
Nicaragua	Pueblo Nuevo	2000	56.7%	79.6%	26.8%
Nicaragua	Pueblo Nuevo	2017	57.0%	76.3%	28.4%
Nicaragua	Puerto Cabezas	2000	55.8%	69.2%	39.3%
Nicaragua	Puerto Cabezas	2017	58.1%	70.4%	44.3%
Nicaragua	Puerto Morazán	2000	58.1%	94.2%	19.9%
Nicaragua	Puerto Morazán	2017	61.3%	95.1%	24.0%
Nicaragua	Quezalguaque	2000	44.6%	78.5%	2.5%
Nicaragua	Quezalguaque	2017	52.0%	83.4%	3.1%
Nicaragua	Quilalí	2000	64.7%	93.7%	30.8%
Nicaragua	Quilalí	2017	67.3%	94.4%	32.5%
Nicaragua	Rancho Grande	2000	54.5%	82.4%	25.0%
Nicaragua	Rancho Grande	2017	57.2%	84.2%	27.1%
Nicaragua	Río Blanco	2000	55.7%	72.7%	37.8%
Nicaragua	Río Blanco	2017	58.6%	73.9%	42.0%
Nicaragua	Rivas	2000	29.9%	67.7%	0.6%
Nicaragua	Rivas	2017	31.6%	66.8%	0.9%
Nicaragua	Rosita	2000	56.4%	72.0%	36.7%
Nicaragua	Rosita	2017	58.5%	72.1%	42.8%
Nicaragua	San Carlos	2000	56.6%	72.3%	40.1%
Nicaragua	San Carlos	2017	58.9%	72.6%	43.3%
Nicaragua	San Dionisio	2000	45.5%	78.6%	5.1%
Nicaragua	San Dionisio	2017	52.5%	82.7%	10.5%
Nicaragua	San Fernando	2000	47.5%	76.9%	16.5%
Nicaragua	San Fernando	2017	48.4%	77.9%	18.3%
Nicaragua	San Francisco del Norte	2000	59.4%	100.0%	10.6%
Nicaragua	San Francisco del Norte	2017	63.8%	100.0%	8.7%
Nicaragua	San Francisco Libre	2000	49.6%	80.4%	14.3%
Nicaragua	San Francisco Libre	2017	49.1%	80.6%	14.5%
Nicaragua	San Isidro	2000	55.5%	88.8%	21.4%
Nicaragua	San Isidro	2017	63.1%	89.1%	27.5%
Nicaragua	San Jorge	2000	12.5%	19.4%	0.0%
Nicaragua	San Jorge	2017	13.1%	18.8%	0.1%
Nicaragua	San José de Cusmapa	2000	36.8%	64.7%	2.7%
Nicaragua	San José de Cusmapa	2017	42.6%	80.1%	11.8%
Nicaragua	San José de los Remates	2000	62.9%	94.4%	34.3%
Nicaragua	San José de los Remates	2017	65.3%	95.6%	35.6%
Nicaragua	San Juan de Limay	2000	63.2%	88.4%	33.5%
Nicaragua	San Juan de Limay	2017	67.2%	89.7%	40.4%
Nicaragua	San Juan de Oriente	2000	100.0%	100.0%	100.0%
Nicaragua	San Juan de Oriente	2017	100.0%	100.0%	100.0%
Nicaragua	San Juan del Norte	2000	54.1%	76.1%	33.0%
Nicaragua	San Juan del Norte	2017	57.1%	77.8%	36.4%

Country	Second Administrative Unit	Year	Mean	Upper	Lower
Nicaragua	San Juan del Río Coco	2000	81.1%	99.2%	53.9%
Nicaragua	San Juan del Río Coco	2017	83.2%	99.7%	57.8%
Nicaragua	San Lorenzo	2000	47.7%	76.5%	20.2%
Nicaragua	San Lorenzo	2017	48.5%	77.8%	21.7%
Nicaragua	San Lucas	2000	56.2%	82.3%	17.5%
Nicaragua	San Lucas	2017	58.6%	84.6%	15.7%
Nicaragua	San Marcos	2000	63.0%	98.6%	23.4%
Nicaragua	San Marcos	2017	68.7%	98.8%	19.7%
Nicaragua	San Miguelito	2000	56.8%	79.7%	32.1%
Nicaragua	San Miguelito	2017	58.9%	80.9%	34.9%
Nicaragua	San Nicolás	2000	56.0%	83.2%	22.7%
Nicaragua	San Nicolás	2017	56.9%	80.8%	20.1%
Nicaragua	San Pedro de Lóvago	2000	51.8%	77.5%	25.0%
Nicaragua	San Pedro de Lóvago	2017	53.9%	78.4%	28.6%
Nicaragua	San Pedro del Norte	2000	76.1%	100.0%	19.8%
Nicaragua	San Pedro del Norte	2017	79.5%	100.0%	28.5%
Nicaragua	San Rafael del Norte	2000	60.6%	87.1%	25.7%
Nicaragua	San Rafael del Norte	2017	63.5%	88.6%	29.8%
Nicaragua	San Rafael del Sur	2000	57.3%	96.9%	10.4%
Nicaragua	San Rafael del Sur	2017	61.5%	97.3%	12.4%
Nicaragua	San Ramón	2000	54.5%	88.9%	21.8%
Nicaragua	San Ramón	2017	57.8%	91.2%	23.7%
Nicaragua	San Sebastián de Yalí	2000	73.4%	97.2%	34.8%
Nicaragua	San Sebastián de Yalí	2017	77.0%	98.4%	37.1%
Nicaragua	Santa Lucía	2000	42.8%	77.3%	6.2%
Nicaragua	Santa Lucía	2017	48.2%	82.1%	9.4%
Nicaragua	Santa María	2000	60.7%	99.2%	13.6%
Nicaragua	Santa María	2017	63.3%	100.0%	20.2%
Nicaragua	Santa María de Pantasma	2000	57.3%	83.2%	27.8%
Nicaragua	Santa María de Pantasma	2017	59.9%	86.7%	29.4%
Nicaragua	Santa Rosa del Peñón	2000	73.0%	97.2%	44.4%
Nicaragua	Santa Rosa del Peñón	2017	75.5%	96.7%	47.9%
Nicaragua	Santo Domingo	2000	56.2%	85.9%	23.2%
Nicaragua	Santo Domingo	2017	58.4%	88.4%	25.9%
Nicaragua	Santo Tomás	2000	54.7%	89.6%	15.8%
Nicaragua	Santo Tomás	2017	57.3%	90.4%	17.9%
Nicaragua	Sébaco	2000	42.4%	73.8%	6.0%
Nicaragua	Sébaco	2017	47.7%	77.8%	7.8%
Nicaragua	Siuna	2000	56.5%	69.5%	42.3%
Nicaragua	Siuna	2017	58.2%	70.1%	43.9%
Nicaragua	Somotillo	2000	57.1%	81.2%	33.0%
Nicaragua	Somotillo	2017	59.7%	84.2%	30.1%
Nicaragua	Somoto	2000	54.1%	83.9%	17.7%
Nicaragua	Somoto	2017	58.5%	85.9%	21.7%

Country	Second Administrative Unit	Year	Mean	Upper	Lower
Nicaragua	Telica	2000	38.7%	73.9%	13.6%
Nicaragua	Telica	2017	41.7%	77.5%	13.3%
Nicaragua	Telpaneca	2000	64.2%	93.2%	32.3%
Nicaragua	Telpaneca	2017	65.9%	95.4%	30.3%
Nicaragua	Terrabona	2000	45.6%	80.2%	16.8%
Nicaragua	Terrabona	2017	49.7%	83.5%	18.1%
Nicaragua	Teustepe	2000	53.9%	84.8%	23.6%
Nicaragua	Teustepe	2017	57.3%	86.2%	27.0%
Nicaragua	Ticuantepe	2000	73.0%	100.0%	27.2%
Nicaragua	Ticuantepe	2017	76.5%	100.0%	35.1%
Nicaragua	Tipitapa	2000	55.0%	85.6%	26.5%
Nicaragua	Tipitapa	2017	57.4%	88.3%	27.4%
Nicaragua	Tisma	2000	55.9%	68.5%	16.8%
Nicaragua	Tisma	2017	56.0%	66.3%	28.9%
Nicaragua	Tola	2000	54.9%	79.3%	27.0%
Nicaragua	Tola	2017	57.4%	80.5%	30.6%
Nicaragua	Totogalpa	2000	84.0%	99.0%	61.3%
Nicaragua	Totogalpa	2017	89.4%	99.3%	73.7%
Nicaragua	Tuma-La Dalia	2000	54.1%	92.1%	19.1%
Nicaragua	Tuma-La Dalia	2017	56.5%	93.0%	20.8%
Nicaragua	Villa Carlos Fonseca	2000	61.6%	87.8%	31.8%
Nicaragua	Villa Carlos Fonseca	2017	64.6%	89.7%	32.3%
Nicaragua	Villa Sandino	2000	54.7%	76.9%	28.1%
Nicaragua	Villa Sandino	2017	57.3%	79.8%	32.3%
Nicaragua	Villanueva	2000	54.6%	81.9%	22.4%
Nicaragua	Villanueva	2017	56.8%	82.7%	25.3%
Nicaragua	Waslala	2000	55.9%	77.0%	33.9%
Nicaragua	Waslala	2017	58.5%	77.9%	37.6%
Nicaragua	Waspán	2000	56.4%	66.0%	45.7%
Nicaragua	Waspán	2017	58.4%	67.8%	48.8%
Nicaragua	Wiwilí	2000	57.5%	73.0%	40.4%
Nicaragua	Wiwilí	2017	59.9%	73.2%	45.1%
Nicaragua	Yalagüina	2000	86.9%	100.0%	62.4%
Nicaragua	Yalagüina	2017	89.0%	100.0%	64.6%
Panama	Aguadulce	2000	95.4%	97.6%	91.7%
Panama	Aguadulce	2017	95.5%	97.7%	91.9%
Panama	Alanje	2000	74.6%	86.6%	58.0%
Panama	Alanje	2017	75.9%	87.3%	59.8%
Panama	Antón	2000	95.5%	97.8%	92.5%
Panama	Antón	2017	95.7%	98.0%	92.9%
Panama	Arraiján	2000	95.2%	97.2%	92.2%
Panama	Arraiján	2017	95.5%	97.4%	92.8%
Panama	Atalaya	2000	90.5%	95.7%	83.3%
Panama	Atalaya	2017	91.1%	96.0%	84.6%
Panama	Balboa	2000	84.4%	99.3%	45.9%
Panama	Balboa	2017	85.0%	99.4%	46.6%
Panama	Barú	2000	71.8%	88.3%	49.2%
Panama	Barú	2017	72.5%	88.5%	50.0%
Panama	Besiko	2000	35.3%	51.2%	20.3%
Panama	Besiko	2017	36.4%	52.2%	21.2%
Panama	Bocas del Toro	2000	50.9%	77.7%	26.2%
Panama	Bocas del Toro	2017	52.1%	78.8%	26.7%
Panama	Boquerón	2000	77.7%	90.5%	58.3%
Panama	Boquerón	2017	79.0%	91.1%	60.4%
Panama	Boquete	2000	68.9%	86.7%	43.5%
Panama	Boquete	2017	69.6%	87.2%	44.5%

Country	Second Administrative Unit	Year	Mean	Upper	Lower
Panama	Bugaba	2000	79.6%	89.6%	68.4%
Panama	Bugaba	2017	80.6%	90.1%	69.8%
Panama	Calobre	2000	92.8%	97.2%	85.4%
Panama	Calobre	2017	93.2%	97.4%	86.3%
Panama	Cañazas	2000	86.7%	92.9%	77.6%
Panama	Cañazas	2017	86.6%	92.9%	77.7%
Panama	Capira	2000	93.3%	97.1%	88.1%
Panama	Capira	2017	93.7%	97.3%	88.6%
Panama	Cémaco	2000	32.1%	44.4%	21.3%
Panama	Cémaco	2017	33.7%	45.9%	22.5%
Panama	Chagres	2000	78.0%	95.2%	49.1%
Panama	Chagres	2017	78.9%	95.5%	50.8%
Panama	Chame	2000	95.9%	98.5%	89.7%
Panama	Chame	2017	96.1%	98.6%	90.0%
Panama	Changuinola	2000	57.8%	70.7%	44.4%
Panama	Changuinola	2017	59.1%	71.9%	46.2%
Panama	Chepigana	2000	63.2%	73.4%	52.4%
Panama	Chepigana	2017	64.8%	74.8%	53.9%
Panama	Chepo	2000	87.3%	93.5%	73.9%
Panama	Chepo	2017	88.0%	93.8%	76.0%
Panama	Chimán	2000	80.2%	95.2%	56.0%
Panama	Chimán	2017	80.7%	95.3%	57.8%
Panama	Chiriquí Grande	2000	41.4%	54.4%	30.4%
Panama	Chiriquí Grande	2017	42.8%	55.7%	31.9%
Panama	Chitré	2000	96.1%	97.6%	94.1%
Panama	Chitré	2017	96.3%	97.8%	94.4%
Panama	Colón	2000	75.7%	85.9%	60.8%
Panama	Colón	2017	76.8%	86.7%	62.4%
Panama	David	2000	74.4%	82.7%	63.7%
Panama	David	2017	75.5%	83.5%	65.5%
Panama	Dolega	2000	72.4%	85.1%	54.7%
Panama	Dolega	2017	73.4%	85.7%	56.0%
Panama	Donoso	2000	78.4%	91.3%	59.1%
Panama	Donoso	2017	79.4%	91.7%	60.8%
Panama	Gualaca	2000	52.4%	73.2%	28.5%
Panama	Gualaca	2017	52.7%	73.7%	29.8%
Panama	Guararé	2000	99.2%	99.7%	98.1%
Panama	Guararé	2017	99.3%	99.7%	98.2%
Panama	Kankintú	2000	23.6%	37.0%	15.1%
Panama	Kankintú	2017	24.6%	38.6%	15.7%
Panama	Kuna Yala	2000	63.8%	83.7%	34.4%
Panama	Kuna Yala	2017	64.2%	83.5%	34.9%
Panama	Kusapín	2000	32.6%	58.3%	14.4%
Panama	Kusapín	2017	33.2%	59.4%	14.4%
Panama	La Chorrera	2000	95.2%	97.3%	92.5%
Panama	La Chorrera	2017	95.4%	97.5%	92.9%
Panama	La Mesa	2000	92.5%	96.7%	85.6%
Panama	La Mesa	2017	92.9%	96.9%	86.3%
Panama	La Pintada	2000	92.2%	96.6%	85.1%
Panama	La Pintada	2017	92.7%	96.8%	85.9%
Panama	Lago Alajuela	2000	83.9%	94.2%	66.2%
Panama	Lago Alajuela	2017	86.2%	95.4%	70.5%
Panama	Lago Bayano	2000	88.8%	96.8%	66.2%
Panama	Lago Bayano	2017	89.5%	97.2%	67.8%
Panama	Lago Gatún	2000	85.5%	95.4%	69.6%
Panama	Lago Gatún	2000	78.0%	86.7%	66.6%
Panama	Lago Gatún	2017	86.4%	95.7%	71.4%
Panama	Lago Gatún	2017	78.9%	87.4%	67.7%
Panama	Las Minas	2000	91.6%	96.5%	82.5%
Panama	Las Minas	2017	92.0%	96.7%	83.4%

Country	Second Administrative Unit	Year	Mean	Upper	Lower
Panama	Las Palmas	2000	86.4%	94.0%	70.3%
Panama	Las Palmas	2017	86.6%	94.1%	70.6%
Panama	Las Tablas	2000	99.4%	99.9%	98.3%
Panama	Las Tablas	2017	99.5%	99.9%	98.4%
Panama	Los Pozos	2000	95.4%	99.1%	88.0%
Panama	Los Pozos	2017	95.8%	99.2%	88.7%
Panama	Los Santos	2000	97.7%	98.8%	95.9%
Panama	Los Santos	2017	97.8%	98.8%	96.1%
Panama	Macaracas	2000	98.1%	99.7%	93.5%
Panama	Macaracas	2017	98.2%	99.7%	93.7%
Panama	Mironó	2000	40.2%	62.1%	23.2%
Panama	Mironó	2017	41.3%	63.1%	24.1%
Panama	Montijo	2000	91.3%	96.0%	83.8%
Panama	Montijo	2017	91.7%	96.2%	84.4%
Panama	Müna	2000	35.7%	44.5%	25.3%
Panama	Müna	2017	36.2%	44.8%	25.8%
Panama	Natá	2000	96.1%	98.1%	93.2%
Panama	Natá	2017	96.3%	98.2%	93.6%
Panama	Nole Duima	2000	42.7%	56.7%	29.3%
Panama	Nole Duima	2017	43.5%	57.3%	30.2%
Panama	Ñürüm	2000	55.2%	68.7%	43.0%
Panama	Ñürüm	2017	54.0%	67.5%	42.2%
Panama	Ocú	2000	90.9%	95.8%	82.4%
Panama	Ocú	2017	91.4%	96.0%	83.3%
Panama	Olá	2000	93.3%	98.3%	83.8%
Panama	Olá	2017	93.8%	98.5%	84.9%
Panama	Panamá	2000	94.7%	96.3%	92.7%
Panama	Panamá	2017	95.2%	96.6%	93.3%
Panama	Parita	2000	93.3%	96.2%	89.0%
Panama	Parita	2017	93.6%	96.5%	89.8%
Panama	Pedasí	2000	98.5%	99.9%	92.8%
Panama	Pedasí	2017	98.6%	99.9%	93.0%
Panama	Penonomé	2000	94.0%	96.7%	89.4%
Panama	Penonomé	2017	94.4%	97.0%	89.9%
Panama	Pesé	2000	94.8%	97.9%	89.2%
Panama	Pesé	2017	94.8%	98.0%	89.1%
Panama	Pinogana	2000	48.3%	61.5%	36.5%
Panama	Pinogana	2017	49.4%	63.0%	38.0%
Panama	Pocrí	2000	99.2%	99.9%	96.3%
Panama	Pocrí	2017	99.2%	99.9%	96.4%
Panama	Portobelo	2000	67.0%	92.7%	34.3%
Panama	Portobelo	2017	68.1%	93.3%	34.9%
Panama	Remedios	2000	73.1%	85.6%	59.0%
Panama	Remedios	2017	73.7%	85.6%	60.4%
Panama	Renacimiento	2000	81.4%	94.2%	55.1%
Panama	Renacimiento	2017	82.2%	94.5%	56.6%
Panama	Río de Jesús	2000	91.7%	97.0%	80.2%
Panama	Río de Jesús	2017	92.1%	97.2%	81.2%
Panama	Sambú	2000	72.1%	92.3%	37.3%
Panama	Sambú	2017	73.2%	92.7%	39.0%
Panama	San Carlos	2000	95.4%	98.4%	88.9%
Panama	San Carlos	2017	95.7%	98.5%	89.3%
Panama	San Félix	2000	68.4%	87.0%	47.6%
Panama	San Félix	2017	69.6%	87.6%	49.1%
Panama	San Francisco	2000	92.9%	97.5%	85.3%
Panama	San Francisco	2017	93.3%	97.7%	86.1%
Panama	San Lorenzo	2000	64.2%	85.5%	36.9%
Panama	San Lorenzo	2017	64.7%	85.4%	37.6%
Panama	San Miguelito	2000	95.8%	97.0%	94.3%
Panama	San Miguelito	2017	96.0%	97.2%	94.6%
Panama	Santa Fe	2000	86.4%	93.9%	77.3%
Panama	Santa Fe	2017	87.0%	94.2%	78.2%

Country	Second Administrative Unit	Year	Mean	Upper	Lower
Panama	Santa Isabel	2000	71.3%	96.6%	30.7%
Panama	Santa Isabel	2017	72.4%	96.8%	31.9%
Panama	Santa María	2000	91.4%	96.7%	82.2%
Panama	Santa María	2017	91.8%	96.9%	83.0%
Panama	Santiago	2000	91.7%	94.6%	88.1%
Panama	Santiago	2017	92.2%	94.9%	88.8%
Panama	Soná	2000	92.2%	96.4%	85.6%
Panama	Soná	2017	92.6%	96.6%	86.3%
Panama	Taboga	2000	92.9%	99.0%	78.6%
Panama	Taboga	2017	93.4%	99.1%	79.6%
Panama	Tolé	2000	71.9%	83.8%	51.6%
Panama	Tolé	2017	72.1%	83.8%	52.2%
Panama	Tonosí	2000	97.7%	99.8%	91.6%
Panama	Tonosí	2017	97.8%	99.8%	92.0%
Paraguay	25 de Diciembre	2000	43.3%	60.5%	26.7%
Paraguay	25 de Diciembre	2017	70.5%	86.0%	50.2%
Paraguay	3 de Febrero	2000	23.4%	56.7%	5.4%
Paraguay	3 de Febrero	2017	45.2%	82.1%	15.4%
Paraguay	Abaí	2000	36.9%	48.5%	27.0%
Paraguay	Abaí	2017	62.6%	73.7%	52.8%
Paraguay	Acahay	2000	42.4%	74.7%	16.3%
Paraguay	Acahay	2017	69.3%	91.6%	38.3%
Paraguay	Alberdi	2000	20.2%	29.3%	13.1%
Paraguay	Alberdi	2017	48.7%	62.0%	34.3%
Paraguay	Alto Verá	2000	27.8%	41.0%	15.9%
Paraguay	Alto Verá	2017	55.5%	68.7%	40.5%
Paraguay	Altos	2000	66.9%	75.4%	55.2%
Paraguay	Altos	2017	88.4%	91.8%	82.3%
Paraguay	Antequera	2000	36.6%	64.1%	14.9%
Paraguay	Antequera	2017	62.8%	87.2%	27.8%
Paraguay	Areguá	2000	77.2%	93.7%	44.9%
Paraguay	Areguá	2017	92.1%	98.4%	74.1%
Paraguay	Arroyos y Esteros	2000	52.5%	73.8%	28.0%
Paraguay	Arroyos y Esteros	2017	78.5%	91.1%	58.5%
Paraguay	Asunción	2000	53.6%	59.7%	44.6%
Paraguay	Asunción	2017	80.1%	82.7%	75.5%
Paraguay	Atyrá	2000	66.2%	73.5%	58.4%
Paraguay	Atyrá	2017	85.9%	91.3%	77.5%
Paraguay	Ayolas	2000	43.2%	56.8%	30.4%
Paraguay	Ayolas	2017	72.4%	82.3%	57.7%
Paraguay	Belén	2000	68.4%	82.3%	52.2%
Paraguay	Belén	2017	88.3%	94.3%	80.6%
Paraguay	Bella Vista	2000	28.6%	46.5%	15.1%
Paraguay	Bella Vista	2000	35.7%	50.4%	23.3%
Paraguay	Bella Vista	2017	57.8%	74.2%	39.1%
Paraguay	Bella Vista	2017	63.4%	74.6%	47.7%
Paraguay	Benjamín Aceval	2000	28.6%	41.9%	18.7%
Paraguay	Benjamín Aceval	2017	57.2%	70.7%	43.6%
Paraguay	Borja	2000	27.2%	53.7%	10.0%
Paraguay	Borja	2017	50.7%	76.6%	25.1%
Paraguay	Caacupé	2000	65.1%	76.2%	52.9%
Paraguay	Caacupé	2017	87.2%	91.8%	80.5%
Paraguay	Caaguazú	2000	26.2%	33.1%	19.3%
Paraguay	Caaguazú	2017	52.4%	58.7%	44.9%
Paraguay	Caapucú	2000	36.6%	56.0%	20.7%
Paraguay	Caapucú	2017	63.4%	79.0%	45.4%

Country	Second Administrative Unit	Year	Mean	Upper	Lower
Paraguay	Caazapá	2000	24.9%	36.0%	16.5%
Paraguay	Caazapá	2017	52.5%	64.9%	41.4%
Paraguay	Cambyreta	2000	30.7%	39.2%	22.6%
Paraguay	Cambyreta	2017	61.6%	69.2%	52.6%
Paraguay	Capiatá	2000	61.7%	68.5%	52.6%
Paraguay	Capiatá	2017	85.7%	88.5%	80.8%
Paraguay	Capitán Bado	2000	29.7%	40.9%	19.9%
Paraguay	Capitán Bado	2017	57.1%	67.6%	46.3%
Paraguay	Capitán Mauricio José Troche	2000	23.2%	54.0%	3.9%
Paraguay	Capitán Mauricio José Troche	2017	47.4%	79.7%	13.3%
Paraguay	Capitán Meza	2000	28.7%	39.6%	18.8%
Paraguay	Capitán Meza	2017	56.1%	68.3%	44.5%
Paraguay	Capitán Miranda	2000	14.2%	35.0%	3.9%
Paraguay	Capitán Miranda	2017	36.4%	67.3%	13.7%
Paraguay	Caraguatay	2000	53.2%	65.1%	43.0%
Paraguay	Caraguatay	2017	79.5%	87.5%	70.8%
Paraguay	Carapeguá	2000	39.2%	61.9%	18.5%
Paraguay	Carapeguá	2017	66.9%	85.0%	41.8%
Paraguay	Carayaó	2000	39.4%	58.1%	20.0%
Paraguay	Carayaó	2017	66.7%	82.7%	41.7%
Paraguay	Carlos Antonio López	2000	27.6%	50.8%	12.3%
Paraguay	Carlos Antonio López	2017	53.2%	74.1%	31.9%
Paraguay	Carmen del Paraná	2000	28.5%	52.4%	11.7%
Paraguay	Carmen del Paraná	2017	57.8%	81.3%	28.9%
Paraguay	Cerrito	2000	27.1%	46.4%	12.9%
Paraguay	Cerrito	2017	52.6%	75.9%	32.2%
Paraguay	Choré	2000	42.7%	46.0%	39.6%
Paraguay	Choré	2017	70.4%	72.9%	68.0%
Paraguay	Ciudad del Este	2000	12.3%	13.0%	11.7%
Paraguay	Ciudad del Este	2017	36.8%	37.6%	35.8%
Paraguay	Concepción	2000	49.9%	54.4%	45.3%
Paraguay	Concepción	2017	77.4%	80.4%	73.6%
Paraguay	Coronel Bogado	2000	27.8%	38.5%	18.5%
Paraguay	Coronel Bogado	2017	56.7%	68.0%	44.0%
Paraguay	Coronel Martínez	2000	37.7%	76.3%	7.8%
Paraguay	Coronel Martínez	2017	63.8%	92.8%	23.1%
Paraguay	Coronel Oviedo	2000	38.8%	46.2%	31.1%
Paraguay	Coronel Oviedo	2017	67.1%	72.1%	60.7%
Paraguay	Corpus Christi	2000	29.9%	43.8%	17.8%
Paraguay	Corpus Christi	2017	57.4%	69.5%	43.8%
Paraguay	Desmochados	2000	26.5%	46.9%	11.3%
Paraguay	Desmochados	2017	53.7%	74.0%	31.3%

Country	Second Administrative Unit	Year	Mean	Upper	Lower
Paraguay	Doctor Botrell	2000	39.0%	72.4%	10.7%
Paraguay	Doctor Botrell	2017	64.9%	90.4%	29.5%
Paraguay	Doctor Cecilio Báez	2000	36.7%	48.7%	25.7%
Paraguay	Doctor Cecilio Báez	2017	67.8%	74.1%	59.7%
Paraguay	Doctor J. Eulogio Estigarribia	2000	34.3%	40.3%	28.9%
Paraguay	Doctor J. Eulogio Estigarribia	2017	65.8%	70.3%	61.0%
Paraguay	Doctor Juan León Mallorquín	2000	14.4%	19.4%	10.2%
Paraguay	Doctor Juan León Mallorquín	2017	39.0%	46.9%	31.3%
Paraguay	Doctor Juan Manuel Frutos	2000	20.9%	31.7%	13.3%
Paraguay	Doctor Juan Manuel Frutos	2017	46.7%	61.3%	35.1%
Paraguay	Doctor Moisés S. Bertoni	2000	19.9%	39.8%	7.9%
Paraguay	Doctor Moisés S. Bertoni	2017	42.1%	67.4%	22.9%
Paraguay	Doctor Pedro P. Peña	2000	65.6%	71.6%	58.5%
Paraguay	Doctor Pedro P. Peña	2017	85.2%	88.6%	80.7%
Paraguay	Domingo Martínez de Irala	2000	23.0%	43.5%	7.9%
Paraguay	Domingo Martínez de Irala	2017	49.1%	70.8%	23.9%
Paraguay	Edelira	2000	23.5%	39.4%	12.1%
Paraguay	Edelira	2017	49.5%	67.6%	32.0%
Paraguay	Emboscada	2000	52.1%	84.2%	24.3%
Paraguay	Emboscada	2017	75.5%	95.0%	48.0%
Paraguay	Emboscada (Caazapa)	2000	21.0%	50.7%	5.7%
Paraguay	Emboscada (Caazapa)	2017	45.8%	77.7%	19.1%
Paraguay	Encarnación	2000	26.6%	35.8%	18.5%
Paraguay	Encarnación	2017	56.0%	66.3%	45.9%
Paraguay	Escobar	2000	40.4%	58.2%	23.9%
Paraguay	Escobar	2017	69.8%	82.2%	51.2%
Paraguay	Eusebio Ayala	2000	73.3%	84.2%	58.8%
Paraguay	Eusebio Ayala	2017	90.9%	95.3%	83.2%
Paraguay	Félix Perez Cardozo	2000	43.3%	81.5%	10.1%
Paraguay	Félix Perez Cardozo	2017	69.6%	94.6%	27.9%
Paraguay	Fernando de la Mora	2000	44.1%	46.7%	41.2%
Paraguay	Fernando de la Mora	2017	75.5%	77.7%	72.8%
Paraguay	Fram	2000	24.1%	52.1%	5.7%
Paraguay	Fram	2017	50.4%	80.1%	15.2%
Paraguay	Fuerte Olimpo	2000	46.8%	55.0%	38.8%
Paraguay	Fuerte Olimpo	2017	71.8%	78.6%	64.2%

Country	Second Administrative Unit	Year	Mean	Upper	Lower
Paraguay	Fulgencio Yegros	2000	23.0%	44.7%	10.8%
Paraguay	Fulgencio Yegros	2017	48.0%	73.4%	27.1%
Paraguay	General Artigas	2000	23.2%	41.2%	10.8%
Paraguay	General Artigas	2017	48.6%	70.3%	27.1%
Paraguay	General Bernardino Caballero	2000	41.2%	54.7%	27.6%
Paraguay	General Bernardino Caballero	2017	71.3%	82.5%	55.3%
Paraguay	General Delgado	2000	28.3%	48.4%	14.0%
Paraguay	General Delgado	2017	56.6%	73.4%	37.9%
Paraguay	General Elizardo Aquino	2000	43.5%	54.5%	34.0%
Paraguay	General Elizardo Aquino	2017	72.9%	81.3%	62.2%
Paraguay	General Eugenio A. Garay	2000	65.6%	73.3%	58.1%
Paraguay	General Eugenio A. Garay	2000	42.9%	74.6%	16.5%
Paraguay	General Eugenio A. Garay	2017	84.9%	89.7%	79.7%
Paraguay	General Eugenio A. Garay	2017	68.8%	91.2%	37.4%
Paraguay	General Francisco C. Alvarez	2000	28.2%	36.3%	21.5%
Paraguay	General Francisco C. Alvarez	2017	57.1%	63.9%	49.6%
Paraguay	General Higinio Morínigo	2000	32.8%	63.8%	9.5%
Paraguay	General Higinio Morínigo	2017	60.5%	85.3%	26.0%
Paraguay	General Isidoro Resquín	2000	48.2%	60.2%	38.3%
Paraguay	General Isidoro Resquín	2017	69.7%	79.5%	60.2%
Paraguay	General José Eduvigis Díaz	2000	29.4%	46.6%	14.7%
Paraguay	General José Eduvigis Díaz	2017	57.4%	71.0%	39.8%
Paraguay	Guarambaré	2000	45.5%	63.1%	26.9%
Paraguay	Guarambaré	2017	73.0%	86.5%	53.9%
Paraguay	Guazú Cuá	2000	34.1%	53.4%	14.6%
Paraguay	Guazú Cuá	2017	60.9%	79.0%	36.0%
Paraguay	Hernandarias	2000	13.9%	18.3%	10.6%
Paraguay	Hernandarias	2017	37.7%	44.8%	31.1%
Paraguay	Hohenau	2000	24.9%	37.9%	15.6%
Paraguay	Hohenau	2017	54.4%	68.5%	40.7%
Paraguay	Horqueta	2000	37.5%	44.9%	30.6%
Paraguay	Horqueta	2017	68.0%	73.8%	61.5%

Country	Second Administrative Unit	Year	Mean	Upper	Lower
Paraguay	Humaitá	2000	33.2%	61.7%	13.5%
Paraguay	Humaitá	2017	60.1%	84.4%	32.5%
Paraguay	Independencia	2000	38.6%	53.4%	23.7%
Paraguay	Independencia	2017	63.5%	77.6%	47.9%
Paraguay	Isla Pucú	2000	68.4%	80.1%	51.4%
Paraguay	Isla Pucú	2017	89.1%	93.9%	79.9%
Paraguay	Isla Umbú	2000	45.0%	64.6%	26.6%
Paraguay	Isla Umbú	2017	73.6%	86.8%	56.4%
Paraguay	Itá	2000	23.2%	29.1%	17.9%
Paraguay	Itá	2017	46.2%	53.8%	37.8%
Paraguay	Itacurubí de la Cordillera	2000	45.7%	83.0%	15.2%
Paraguay	Itacurubí de la Cordillera	2017	69.6%	95.3%	31.1%
Paraguay	Itacurubí del Rosario	2000	30.9%	42.4%	21.6%
Paraguay	Itacurubí del Rosario	2017	57.0%	69.5%	46.0%
Paraguay	Itakyry	2000	19.3%	28.6%	12.0%
Paraguay	Itakyry	2017	43.6%	54.7%	34.2%
Paraguay	Itanara	2000	25.3%	47.7%	8.1%
Paraguay	Itanara	2017	49.9%	72.9%	27.5%
Paraguay	Itapé	2000	28.4%	58.0%	9.3%
Paraguay	Itapé	2017	54.0%	82.7%	21.3%
Paraguay	Itauguá	2000	93.8%	96.6%	89.5%
Paraguay	Itauguá	2017	98.1%	99.0%	96.8%
Paraguay	Iturbe	2000	33.6%	63.7%	10.8%
Paraguay	Iturbe	2017	60.4%	86.8%	27.8%
Paraguay	Jesús	2000	20.6%	31.2%	12.0%
Paraguay	Jesús	2017	49.2%	63.6%	34.6%
Paraguay	Jose A. Fassardi	2000	41.1%	62.8%	17.4%
Paraguay	Jose A. Fassardi	2017	65.6%	84.2%	41.7%
Paraguay	José Domingo Ocampos	2000	17.2%	46.0%	4.3%
Paraguay	José Domingo Ocampos	2017	38.4%	71.6%	13.8%
Paraguay	José Leandro Oviedo	2000	34.1%	59.1%	17.2%
Paraguay	José Leandro Oviedo	2017	63.2%	84.5%	40.9%
Paraguay	Juan Augusto Saldívar	2000	59.7%	63.7%	55.5%
Paraguay	Juan Augusto Saldívar	2017	83.1%	85.7%	80.4%
Paraguay	Juan de Mena	2000	37.8%	63.0%	18.1%
Paraguay	Juan de Mena	2017	63.3%	84.6%	37.9%
Paraguay	Juan Emilio O'Leary	2000	17.1%	44.6%	5.5%
Paraguay	Juan Emilio O'Leary	2017	40.2%	72.8%	16.6%
Paraguay	La Colmena	2000	36.7%	45.1%	29.0%
Paraguay	La Colmena	2017	68.5%	75.7%	60.8%
Paraguay	La Pastora	2000	33.4%	65.1%	9.2%
Paraguay	La Pastora	2017	59.9%	87.8%	24.5%
Paraguay	La Paz	2000	18.2%	45.3%	3.3%
Paraguay	La Paz	2017	40.6%	74.9%	11.2%
Paraguay	La Victoria	2000	49.2%	57.6%	41.5%
Paraguay	La Victoria	2017	74.2%	80.2%	68.6%
Paraguay	Lambaré	2000	68.7%	85.1%	45.0%
Paraguay	Lambaré	2017	88.8%	95.7%	75.6%

Country	Second Administrative Unit	Year	Mean	Upper	Lower
Paraguay	Laureles	2000	30.5%	49.1%	17.2%
Paraguay	Laureles	2017	58.4%	75.0%	41.3%
Paraguay	Lima	2000	61.3%	70.3%	50.1%
Paraguay	Lima	2017	85.2%	90.0%	76.6%
Paraguay	Limpio	2000	38.4%	61.6%	18.6%
Paraguay	Limpio	2017	67.5%	85.8%	42.5%
Paraguay	Loma Grande	2000	60.3%	65.9%	53.2%
Paraguay	Loma Grande	2017	84.9%	88.1%	80.6%
Paraguay	Loreto	2000	42.2%	59.3%	27.0%
Paraguay	Loreto	2017	70.6%	83.7%	54.4%
Paraguay	Los Cedrales	2000	25.7%	49.3%	8.1%
Paraguay	Los Cedrales	2017	50.5%	72.0%	24.3%
Paraguay	Luque	2000	54.6%	60.1%	47.8%
Paraguay	Luque	2017	81.9%	84.9%	77.9%
Paraguay	Maciel	2000	25.7%	53.4%	7.4%
Paraguay	Maciel	2017	49.7%	76.6%	19.1%
Paraguay	Mariano Roque Alonso	2000	36.7%	48.5%	25.6%
Paraguay	Mariano Roque Alonso	2017	68.0%	79.0%	52.3%
Paraguay	Mariscal Francisco Solano López	2000	28.2%	44.6%	14.5%
Paraguay	Mariscal Francisco Solano López	2017	53.7%	71.7%	33.9%
Paraguay	Mariscal José Félix Estigarribia	2000	70.1%	83.2%	53.1%
Paraguay	Mariscal José Félix Estigarribia	2017	87.8%	94.1%	75.4%
Paraguay	Mayor José J. Martinez	2000	33.6%	65.3%	10.3%
Paraguay	Mayor José J. Martinez	2017	61.1%	82.8%	28.8%
Paraguay	Mayor Julio D. Otaño	2000	22.1%	37.0%	11.1%
Paraguay	Mayor Julio D. Otaño	2017	46.1%	65.0%	25.0%
Paraguay	Mayor Pablo Lagerenza	2000	53.6%	63.1%	43.9%
Paraguay	Mayor Pablo Lagerenza	2017	75.4%	81.3%	68.7%
Paraguay	Mbaracayú	2000	15.9%	23.7%	10.4%
Paraguay	Mbaracayú	2017	39.3%	50.7%	29.4%
Paraguay	Mbocayaty del Guairá	2000	40.5%	73.2%	10.2%
Paraguay	Mbocayaty del Guairá	2017	66.4%	90.4%	29.2%
Paraguay	Mbocayaty del Yhaguy	2000	46.5%	74.9%	22.9%
Paraguay	Mbocayaty del Yhaguy	2017	72.3%	91.9%	47.9%
Paraguay	Mbuyapey	2000	36.6%	60.9%	17.0%
Paraguay	Mbuyapey	2017	63.0%	83.2%	39.6%
Paraguay	Minga Guazú	2000	16.9%	19.3%	14.8%
Paraguay	Minga Guazú	2017	44.8%	48.5%	41.0%
Paraguay	Minga Porá	2000	21.4%	38.6%	10.2%
Paraguay	Minga Porá	2017	47.9%	68.2%	28.1%
Paraguay	Ñacunday	2000	22.9%	35.7%	12.8%
Paraguay	Ñacunday	2017	48.6%	65.4%	33.5%

Country	Second Administrative Unit	Year	Mean	Upper	Lower
Paraguay	Naranjal	2000	17.4%	31.5%	8.4%
Paraguay	Naranjal	2017	42.1%	62.0%	23.2%
Paraguay	Natalicio Talavera	2000	19.6%	54.5%	3.0%
Paraguay	Natalicio Talavera	2017	43.9%	82.7%	11.2%
Paraguay	Natalio	2000	33.8%	58.4%	15.7%
Paraguay	Natalio	2017	60.2%	80.3%	38.7%
Paraguay	Ñemby	2000	60.5%	66.5%	52.7%
Paraguay	Ñemby	2017	84.5%	87.8%	79.9%
Paraguay	Nueva Alborada	2000	12.5%	32.0%	3.1%
Paraguay	Nueva Alborada	2017	29.2%	57.1%	10.0%
Paraguay	Nueva Colombia	2000	76.7%	87.8%	63.4%
Paraguay	Nueva Colombia	2017	91.7%	96.3%	85.9%
Paraguay	Nueva Germania	2000	43.4%	54.0%	31.4%
Paraguay	Nueva Germania	2017	72.4%	82.4%	55.7%
Paraguay	Nueva Italia	2000	37.7%	77.1%	11.3%
Paraguay	Nueva Italia	2017	64.3%	92.1%	27.4%
Paraguay	Nueva Londres	2000	32.1%	51.8%	17.9%
Paraguay	Nueva Londres	2017	57.6%	77.3%	39.2%
Paraguay	Ñumí	2000	31.1%	63.4%	6.8%
Paraguay	Ñumí	2017	58.4%	86.6%	21.4%
Paraguay	Obligado	2000	25.3%	33.3%	19.6%
Paraguay	Obligado	2017	55.3%	63.6%	47.8%
Paraguay	Paraguarí	2000	34.3%	45.0%	24.9%
Paraguay	Paraguarí	2017	64.9%	74.1%	54.4%
Paraguay	Paso de Patria	2000	38.4%	74.7%	7.7%
Paraguay	Paso de Patria	2017	65.1%	91.4%	21.1%
Paraguay	Pedro Juan Caballero	2000	37.2%	43.7%	32.0%
Paraguay	Pedro Juan Caballero	2017	67.6%	73.2%	61.4%
Paraguay	Pilar	2000	30.2%	40.5%	20.7%
Paraguay	Pilar	2017	60.8%	69.4%	50.5%
Paraguay	Pirapó	2000	26.2%	53.7%	9.3%
Paraguay	Pirapó	2017	51.4%	79.7%	25.3%
Paraguay	Pirayú	2000	59.1%	79.3%	41.8%
Paraguay	Pirayú	2017	84.1%	93.4%	71.9%
Paraguay	Piribebuy	2000	36.0%	49.0%	25.5%
Paraguay	Piribebuy	2017	62.6%	75.9%	47.4%
Paraguay	Pozo Colorado	2000	49.6%	55.8%	42.9%
Paraguay	Pozo Colorado	2017	75.0%	79.4%	70.1%
Paraguay	Presidente Franco	2000	16.5%	18.2%	14.5%
Paraguay	Presidente Franco	2017	42.5%	44.7%	39.7%
Paraguay	Primero de Marzo	2000	66.6%	79.1%	48.9%
Paraguay	Primero de Marzo	2017	88.1%	93.3%	78.3%
Paraguay	Puerto Pinasco	2000	36.3%	45.1%	29.0%
Paraguay	Puerto Pinasco	2017	64.8%	72.2%	57.3%

Country	Second Administrative Unit	Year	Mean	Upper	Lower
Paraguay	Quiíndy	2000	35.6%	61.7%	15.5%
Paraguay	Quiíndy	2017	64.8%	86.2%	35.7%
Paraguay	Quyquyhó	2000	45.0%	52.1%	38.0%
Paraguay	Quyquyhó	2017	74.4%	79.1%	68.8%
Paraguay	R. I. 3 Corrales	2000	37.4%	52.3%	24.8%
Paraguay	R. I. 3 Corrales	2017	57.6%	74.0%	39.9%
Paraguay	Raúl Arsenio Oviedo	2000	25.9%	38.3%	16.4%
Paraguay	Raúl Arsenio Oviedo	2017	50.8%	63.8%	37.6%
Paraguay	Repatriación	2000	13.2%	24.7%	4.8%
Paraguay	Repatriación	2017	28.0%	42.7%	12.8%
Paraguay	Salto del Guairá	2000	24.0%	31.6%	16.3%
Paraguay	Salto del Guairá	2017	48.1%	59.5%	36.2%
Paraguay	San Alberto	2000	18.2%	30.2%	11.1%
Paraguay	San Alberto	2017	43.8%	59.1%	30.7%
Paraguay	San Antonio	2000	77.4%	91.3%	55.4%
Paraguay	San Antonio	2017	91.9%	97.4%	80.7%
Paraguay	San Bernardino	2000	81.3%	94.9%	57.3%
Paraguay	San Bernardino	2017	94.0%	98.7%	82.6%
Paraguay	San Carlos	2000	37.8%	55.6%	22.1%
Paraguay	San Carlos	2017	64.7%	80.2%	48.4%
Paraguay	San Cosme y Damián	2000	29.9%	49.0%	12.4%
Paraguay	San Cosme y Damián	2017	55.2%	78.2%	28.3%
Paraguay	San Cristóbal	2000	26.3%	42.2%	13.9%
Paraguay	San Cristóbal	2017	55.6%	72.6%	35.5%
Paraguay	San Estanislao	2000	44.1%	49.9%	39.1%
Paraguay	San Estanislao	2017	71.0%	75.3%	66.8%
Paraguay	San Ignacio	2000	48.5%	55.2%	41.2%
Paraguay	San Ignacio	2017	76.2%	80.1%	71.4%
Paraguay	San Joaquín	2000	23.4%	34.7%	13.9%
Paraguay	San Joaquín	2017	50.8%	63.9%	37.1%
Paraguay	San José de los Arroyos	2000	36.0%	52.8%	22.6%
Paraguay	San José de los Arroyos	2017	65.6%	79.0%	51.3%
Paraguay	San José Obrero	2000	61.7%	82.1%	32.5%
Paraguay	San José Obrero	2017	84.1%	94.5%	62.2%
Paraguay	San Juan Bautista de las Misiones	2000	46.6%	58.6%	37.7%
Paraguay	San Juan Bautista de las Misiones	2017	72.0%	83.0%	58.5%
Paraguay	San Juan Bautista del Ñeembucu	2000	25.4%	41.0%	12.7%
Paraguay	San Juan Bautista del Ñeembucu	2017	49.3%	66.4%	31.5%

Country	Second Administrative Unit	Year	Mean	Upper	Lower
Paraguay	San Juan del Paraná	2000	12.7%	35.0%	3.8%
Paraguay	San Juan del Paraná	2017	32.1%	64.8%	12.0%
Paraguay	San Juan Nepomuceno	2000	26.4%	40.1%	15.8%
Paraguay	San Juan Nepomuceno	2017	55.1%	67.9%	40.1%
Paraguay	San Lázaro	2000	47.9%	60.5%	36.3%
Paraguay	San Lázaro	2017	76.1%	84.9%	66.6%
Paraguay	San Lorenzo	2000	46.6%	48.7%	44.1%
Paraguay	San Lorenzo	2017	76.0%	77.6%	74.3%
Paraguay	San Miguel	2000	44.0%	57.2%	32.0%
Paraguay	San Miguel	2017	72.3%	82.3%	59.2%
Paraguay	San Pablo	2000	43.0%	59.5%	26.0%
Paraguay	San Pablo	2017	70.1%	83.1%	52.8%
Paraguay	San Patricio	2000	54.7%	63.0%	46.5%
Paraguay	San Patricio	2017	81.9%	86.8%	76.3%
Paraguay	San Pedro del Paraná	2000	24.6%	34.3%	17.5%
Paraguay	San Pedro del Paraná	2017	51.7%	60.5%	42.2%
Paraguay	San Pedro del Ycuamandyyú	2000	40.8%	49.2%	32.7%
Paraguay	San Pedro del Ycuamandyyú	2017	69.5%	75.8%	61.3%
Paraguay	San Rafael del Paraná	2000	23.9%	39.6%	12.9%
Paraguay	San Rafael del Paraná	2017	48.6%	66.9%	32.5%
Paraguay	San Roque González de Santa Cruz	2000	24.0%	33.7%	17.3%
Paraguay	San Roque González de Santa Cruz	2017	51.4%	66.4%	40.1%
Paraguay	San Salvador	2000	18.1%	37.1%	4.5%
Paraguay	San Salvador	2017	39.5%	66.2%	14.1%
Paraguay	Santa Elena	2000	51.3%	79.0%	23.4%
Paraguay	Santa Elena	2017	77.3%	93.8%	48.8%
Paraguay	Santa María	2000	53.1%	76.6%	29.6%
Paraguay	Santa María	2017	78.6%	93.0%	56.6%
Paraguay	Santa Rita	2000	18.4%	30.1%	8.6%
Paraguay	Santa Rita	2017	44.2%	60.3%	26.2%
Paraguay	Santa Rosa	2000	47.3%	54.0%	39.5%
Paraguay	Santa Rosa	2017	75.4%	80.2%	69.6%
Paraguay	Santa Rosa del Mbutuy	2000	40.4%	62.7%	23.9%
Paraguay	Santa Rosa del Mbutuy	2017	68.3%	84.3%	50.1%
Paraguay	Santa Rosa del Monday	2000	21.9%	38.1%	11.3%
Paraguay	Santa Rosa del Monday	2017	46.4%	62.7%	29.6%
Paraguay	Santiago	2000	42.6%	57.8%	30.0%
Paraguay	Santiago	2017	68.6%	82.8%	53.8%
Paraguay	Sapucaí	2000	40.9%	52.1%	31.6%
Paraguay	Sapucaí	2017	71.2%	80.7%	60.1%
Paraguay	Simón Bolívar	2000	36.5%	50.8%	23.7%
Paraguay	Simón Bolívar	2017	66.5%	77.4%	51.8%

Country	Second Administrative Unit	Year	Mean	Upper	Lower
Paraguay	Tabaí	2000	23.6%	40.6%	11.1%
Paraguay	Tabaí	2017	50.0%	67.4%	32.1%
Paraguay	Tacuaras	2000	34.0%	52.3%	17.4%
Paraguay	Tacuaras	2017	60.1%	77.6%	38.9%
Paraguay	Tacuatí	2000	38.7%	47.6%	31.0%
Paraguay	Tacuatí	2017	64.6%	74.1%	54.3%
Paraguay	Tebicuarymí	2000	32.4%	66.6%	9.0%
Paraguay	Tebicuarymí	2017	58.9%	87.5%	26.1%
Paraguay	Tobatí	2000	50.9%	78.4%	23.9%
Paraguay	Tobatí	2017	76.3%	93.5%	50.6%
Paraguay	Tomás Romero Pereira	2000	22.5%	38.4%	10.2%
Paraguay	Tomás Romero Pereira	2017	50.2%	70.7%	26.6%
Paraguay	Trinidad	2000	22.3%	43.4%	8.6%
Paraguay	Trinidad	2017	47.8%	71.1%	23.7%
Paraguay	Unión	2000	38.3%	55.9%	21.0%
Paraguay	Unión	2017	67.3%	80.1%	48.7%
Paraguay	Valenzuela	2000	35.6%	71.7%	9.8%
Paraguay	Valenzuela	2017	62.3%	90.6%	26.4%
Paraguay	Villa del Rosario	2000	44.9%	58.7%	31.3%
Paraguay	Villa del Rosario	2017	72.1%	82.9%	58.6%
Paraguay	Villa Elisa	2000	63.2%	81.3%	36.3%
Paraguay	Villa Elisa	2017	84.8%	93.3%	68.8%
Paraguay	Villa Florida	2000	43.0%	82.2%	11.7%
Paraguay	Villa Florida	2017	68.6%	95.2%	29.7%
Paraguay	Villa Franca	2000	29.2%	52.4%	12.3%
Paraguay	Villa Franca	2017	58.6%	78.7%	34.3%
Paraguay	Villa Hayes	2000	45.1%	53.5%	35.4%
Paraguay	Villa Hayes	2017	72.0%	78.7%	63.5%
Paraguay	Villa Oliva	2000	32.1%	48.2%	17.2%
Paraguay	Villa Oliva	2017	58.6%	76.8%	38.6%
Paraguay	Villa San Isidro Curuguaty	2000	30.4%	36.5%	24.0%
Paraguay	Villa San Isidro Curuguaty	2017	55.3%	61.5%	48.4%
Paraguay	Villa Ygatimí	2000	24.0%	34.1%	15.2%
Paraguay	Villa Ygatimí	2017	51.4%	62.0%	37.9%
Paraguay	Villalbín	2000	32.8%	56.3%	13.7%
Paraguay	Villalbín	2017	60.9%	79.9%	38.6%
Paraguay	Villarrica	2000	44.5%	75.2%	18.0%
Paraguay	Villarrica	2017	71.9%	91.8%	34.6%
Paraguay	Villeta	2000	66.9%	83.9%	48.1%
Paraguay	Villeta	2017	83.2%	93.8%	69.5%
Paraguay	Water body	2000	86.5%	97.7%	66.5%
Paraguay	Water body	2017	96.0%	99.4%	88.5%
Paraguay	Yabebyry	2000	41.3%	61.4%	24.2%
Paraguay	Yabebyry	2017	67.5%	83.0%	49.4%
Paraguay	Yaguarón	2000	36.0%	56.3%	18.3%
Paraguay	Yaguarón	2017	64.2%	79.4%	42.1%
Paraguay	Yataity del Guairá	2000	48.7%	86.4%	11.5%
Paraguay	Yataity del Guairá	2017	73.1%	96.1%	26.5%
Paraguay	Yataity del Norte	2000	43.5%	57.2%	29.8%

Country	Second Administrative Unit	Year	Mean	Upper	Lower
Paraguay	Yataity del Norte	2017	72.9%	83.2%	60.7%
Paraguay	Yatytay	2000	31.6%	54.5%	15.3%
Paraguay	Yatytay	2017	59.4%	75.8%	40.7%
Paraguay	Yby Yaù	2000	36.9%	47.4%	27.4%
Paraguay	Yby Yaù	2017	67.4%	76.0%	58.3%
Paraguay	Ybycui	2000	45.8%	64.1%	27.0%
Paraguay	Ybycui	2017	73.6%	87.7%	49.5%
Paraguay	Ybytimí	2000	35.8%	60.6%	17.4%
Paraguay	Ybytimí	2017	63.0%	84.5%	37.9%
Paraguay	Yguazú	2000	21.4%	38.5%	9.2%
Paraguay	Yguazú	2017	47.2%	66.4%	26.6%
Paraguay	Yhú	2000	28.0%	36.2%	21.1%
Paraguay	Yhú	2017	54.6%	63.2%	46.5%
Paraguay	Ypacaraí	2000	88.5%	96.8%	71.3%
Paraguay	Ypacaraí	2017	96.7%	99.2%	90.6%
Paraguay	Ypané	2000	94.5%	97.7%	88.6%
Paraguay	Ypané	2017	98.2%	99.4%	95.2%
Paraguay	Ypejhú	2000	23.4%	35.9%	13.5%
Paraguay	Ypejhú	2017	49.7%	67.1%	33.6%
Paraguay	Yuty	2000	22.6%	34.9%	13.3%
Paraguay	Yuty	2017	48.9%	63.9%	34.9%
Peru	Abancay	2000	75.6%	83.2%	64.9%
Peru	Abancay	2017	87.3%	91.5%	80.7%
Peru	Acobamba	2000	61.9%	67.1%	56.9%
Peru	Acobamba	2017	77.7%	80.7%	74.4%
Peru	Acomayo	2000	64.4%	83.3%	43.4%
Peru	Acomayo	2017	78.2%	91.7%	59.2%
Peru	Aija	2000	71.6%	89.4%	46.0%
Peru	Aija	2017	80.8%	95.9%	55.1%
Peru	Alto Amazonas	2000	32.8%	38.6%	27.0%
Peru	Alto Amazonas	2017	44.8%	50.4%	39.5%
Peru	Ambo	2000	53.0%	61.1%	45.6%
Peru	Ambo	2017	70.0%	77.1%	63.2%
Peru	Andahuaylas	2000	78.9%	83.1%	74.2%
Peru	Andahuaylas	2017	90.3%	93.1%	86.8%
Peru	Angaraes	2000	77.7%	82.9%	71.8%
Peru	Angaraes	2017	87.5%	92.8%	77.1%
Peru	Anta	2000	72.6%	81.4%	62.0%
Peru	Anta	2017	83.2%	89.7%	75.8%
Peru	Antabamba	2000	54.0%	75.8%	38.1%
Peru	Antabamba	2017	62.9%	81.3%	46.5%
Peru	Antonio Raymondi	2000	73.8%	87.8%	55.1%
Peru	Antonio Raymondi	2017	85.0%	94.7%	67.8%
Peru	Arequipa	2000	90.0%	90.8%	89.2%
Peru	Arequipa	2017	95.1%	95.9%	94.0%
Peru	Ascope	2000	67.2%	76.6%	54.0%
Peru	Ascope	2017	79.3%	87.1%	66.5%
Peru	Asunción	2000	70.4%	86.4%	48.0%
Peru	Asunción	2017	82.5%	94.9%	62.3%
Peru	Atalaya	2000	42.3%	50.0%	33.0%
Peru	Atalaya	2017	52.0%	59.2%	43.1%
Peru	Ayabaca	2000	57.5%	69.0%	45.5%
Peru	Ayabaca	2017	67.3%	78.2%	55.5%
Peru	Aymaraes	2000	61.2%	74.5%	49.9%
Peru	Aymaraes	2017	73.1%	85.0%	60.4%
Peru	Azángaro	2000	44.9%	53.8%	37.2%
Peru	Azángaro	2017	56.5%	65.0%	48.9%

Country	Second Administrative Unit	Year	Mean	Upper	Lower
Peru	Bagua	2000	58.6%	63.8%	53.3%
Peru	Bagua	2017	70.4%	76.5%	64.1%
Peru	Barranca	2000	76.0%	81.1%	68.9%
Peru	Barranca	2017	87.4%	89.9%	84.0%
Peru	Bellavista	2000	32.9%	43.8%	24.5%
Peru	Bellavista	2017	43.0%	56.7%	30.8%
Peru	Bolívar	2000	60.1%	90.7%	24.8%
Peru	Bolívar	2017	66.7%	94.6%	31.6%
Peru	Bolognesi	2000	57.6%	73.8%	40.2%
Peru	Bolognesi	2017	67.1%	82.1%	50.4%
Peru	Bongará	2000	66.3%	76.2%	56.0%
Peru	Bongará	2017	77.9%	85.6%	69.8%
Peru	Cajabamba	2000	46.1%	63.6%	30.1%
Peru	Cajabamba	2017	56.9%	71.8%	41.4%
Peru	Cajamarca	2000	75.4%	83.4%	65.5%
Peru	Cajamarca	2017	87.3%	92.8%	78.8%
Peru	Cajatambo	2000	49.9%	81.2%	17.9%
Peru	Cajatambo	2017	56.6%	86.3%	23.9%
Peru	Calca	2000	57.3%	67.5%	47.1%
Peru	Calca	2017	68.1%	77.8%	57.4%
Peru	Callao	2000	81.5%	86.7%	69.2%
Peru	Callao	2017	92.9%	94.8%	85.9%
Peru	Camaná	2000	79.5%	90.8%	61.8%
Peru	Camaná	2017	85.0%	94.1%	73.1%
Peru	Canas	2000	63.6%	76.5%	49.7%
Peru	Canas	2017	76.1%	86.5%	63.1%
Peru	Canchis	2000	72.7%	81.1%	64.6%
Peru	Canchis	2017	84.4%	90.4%	77.9%
Peru	Candarave	2000	72.7%	87.6%	52.2%
Peru	Candarave	2017	81.9%	93.3%	66.1%
Peru	Cañete	2000	76.2%	81.6%	70.7%
Peru	Cañete	2017	85.4%	90.8%	79.2%
Peru	Cangallo	2000	71.1%	82.5%	57.0%
Peru	Cangallo	2017	84.3%	92.1%	74.6%
Peru	Canta	2000	70.1%	81.5%	54.0%
Peru	Canta	2017	84.0%	92.1%	71.1%
Peru	Carabaya	2000	53.0%	68.9%	38.7%
Peru	Carabaya	2017	61.9%	76.6%	48.3%
Peru	Caravelí	2000	51.2%	63.9%	39.3%
Peru	Caravelí	2017	60.6%	75.9%	46.7%
Peru	Carhuaz	2000	79.9%	88.3%	68.4%
Peru	Carhuaz	2017	90.3%	95.0%	83.2%
Peru	Carlos Fermin Fitzcarrald	2000	61.7%	74.0%	47.2%
Peru	Carlos Fermin Fitzcarrald	2017	75.0%	84.7%	61.0%
Peru	Casma	2000	77.1%	86.9%	65.6%
Peru	Casma	2017	86.8%	94.6%	76.8%
Peru	Castilla	2000	65.8%	80.4%	50.6%
Peru	Castilla	2017	81.3%	90.1%	68.5%
Peru	Castrovirreyna	2000	55.7%	69.7%	42.7%
Peru	Castrovirreyna	2017	66.4%	79.6%	54.1%
Peru	Caylloma	2000	60.1%	72.3%	47.4%
Peru	Caylloma	2017	72.7%	82.3%	61.9%
Peru	Celendín	2000	65.1%	77.9%	51.8%
Peru	Celendín	2017	77.8%	88.0%	66.2%
Peru	Chachapoyas	2000	65.9%	74.2%	56.5%
Peru	Chachapoyas	2017	79.3%	85.7%	72.0%
Peru	Chanchamayo	2000	67.0%	75.9%	58.7%
Peru	Chanchamayo	2017	78.6%	87.1%	69.6%
Peru	Chepén	2000	71.4%	80.3%	60.5%
Peru	Chepén	2017	83.9%	90.7%	74.3%

Country	Second Administrative Unit	Year	Mean	Upper	Lower
Peru	Chiclayo	2000	81.3%	82.6%	80.0%
Peru	Chiclayo	2017	92.5%	93.2%	91.5%
Peru	Chincha	2000	78.3%	81.2%	75.9%
Peru	Chincha	2017	90.6%	92.6%	88.4%
Peru	Chincheros	2000	73.9%	80.5%	67.5%
Peru	Chincheros	2017	85.6%	90.7%	80.4%
Peru	Chota	2000	53.6%	60.9%	45.7%
Peru	Chota	2017	69.2%	75.9%	62.5%
Peru	Chucuíto	2000	54.7%	66.2%	45.3%
Peru	Chucuíto	2017	58.8%	73.4%	45.7%
Peru	Chumbivilcas	2000	65.9%	75.3%	52.3%
Peru	Chumbivilcas	2017	77.7%	85.2%	66.2%
Peru	Chupaca	2000	77.8%	82.0%	73.1%
Peru	Chupaca	2017	90.4%	92.8%	87.6%
Peru	Churcampa	2000	65.0%	69.9%	59.2%
Peru	Churcampa	2017	82.1%	85.3%	78.0%
Peru	Concepción	2000	71.5%	83.6%	60.8%
Peru	Concepción	2017	81.9%	91.5%	71.8%
Peru	Condesuyos	2000	61.9%	79.9%	40.2%
Peru	Condesuyos	2017	70.0%	87.5%	45.8%
Peru	Condorcanqui	2000	45.4%	56.2%	33.7%
Peru	Condorcanqui	2017	54.0%	63.7%	42.3%
Peru	Contralmirante Villar	2000	57.7%	73.9%	42.3%
Peru	Contralmirante Villar	2017	70.1%	85.3%	52.2%
Peru	Contumazá	2000	57.5%	75.6%	40.2%
Peru	Contumazá	2017	66.8%	83.7%	48.8%
Peru	Coronel Portillo	2000	43.0%	46.8%	39.1%
Peru	Coronel Portillo	2017	68.1%	71.4%	63.8%
Peru	Corongo	2000	64.9%	82.3%	45.6%
Peru	Corongo	2017	75.4%	92.1%	56.9%
Peru	Cotabambas	2000	64.2%	73.1%	54.0%
Peru	Cotabambas	2017	78.4%	87.3%	64.8%
Peru	Cusco	2000	83.9%	89.0%	78.7%
Peru	Cusco	2017	92.9%	96.6%	85.6%
Peru	Cutervo	2000	65.9%	74.7%	55.8%
Peru	Cutervo	2017	77.7%	84.6%	68.9%
Peru	Daniel Alcides Carrión	2000	59.5%	67.0%	51.6%
Peru	Daniel Alcides Carrión	2017	71.2%	78.9%	63.5%
Peru	Dos de Mayo	2000	46.7%	56.6%	38.5%
Peru	Dos de Mayo	2017	59.9%	70.1%	48.8%
Peru	El Collao	2000	51.3%	57.8%	45.2%
Peru	El Collao	2017	69.0%	74.8%	63.5%
Peru	El Dorado	2000	62.5%	71.9%	52.5%
Peru	El Dorado	2017	77.2%	86.5%	66.2%
Peru	Espinar	2000	47.1%	60.5%	36.2%
Peru	Espinar	2017	60.8%	74.8%	49.3%
Peru	Ferreñafe	2000	63.9%	73.6%	55.3%
Peru	Ferreñafe	2017	75.7%	84.3%	66.8%
Peru	General Sánchez Cerro	2000	71.8%	79.4%	63.5%
Peru	General Sánchez Cerro	2017	81.7%	87.9%	74.9%
Peru	Gran Chimú	2000	61.5%	81.5%	37.3%
Peru	Gran Chimú	2017	73.2%	88.7%	50.0%
Peru	Grau	2000	64.7%	74.7%	51.4%
Peru	Grau	2017	74.8%	83.9%	62.7%

Country	Second Administrative Unit	Year	Mean	Upper	Lower
Peru	Huacaybamba	2000	52.2%	76.0%	24.0%
Peru	Huacaybamba	2017	61.4%	83.5%	34.8%
Peru	Hualgayoc	2000	62.6%	75.3%	48.7%
Peru	Hualgayoc	2017	77.2%	90.0%	61.5%
Peru	Huallaga	2000	42.2%	55.5%	28.1%
Peru	Huallaga	2017	59.0%	72.2%	43.2%
Peru	Huamalíes	2000	47.0%	54.6%	40.2%
Peru	Huamalíes	2017	60.3%	69.0%	50.8%
Peru	Huamanga	2000	77.3%	79.2%	75.5%
Peru	Huamanga	2017	89.7%	91.1%	88.3%
Peru	Huanca Sancos	2000	65.5%	79.5%	51.2%
Peru	Huanca Sancos	2017	74.7%	88.4%	60.7%
Peru	Huancabamba	2000	57.2%	71.3%	39.4%
Peru	Huancabamba	2017	66.5%	80.3%	49.6%
Peru	Huancane	2000	41.4%	52.6%	31.4%
Peru	Huancane	2017	52.7%	63.7%	42.3%
Peru	Huancavelica	2000	73.1%	77.3%	68.6%
Peru	Huancavelica	2017	84.7%	88.1%	80.9%
Peru	Huancayo	2000	85.9%	87.8%	84.1%
Peru	Huancayo	2017	94.3%	95.3%	93.2%
Peru	Huanta	2000	73.8%	83.1%	63.5%
Peru	Huanta	2017	84.8%	90.2%	78.9%
Peru	Huaral	2000	73.0%	77.4%	68.0%
Peru	Huaral	2017	88.3%	91.1%	84.2%
Peru	Huaraz	2000	83.4%	87.9%	77.6%
Peru	Huaraz	2017	92.8%	95.6%	89.2%
Peru	Huari	2000	69.0%	80.9%	57.7%
Peru	Huari	2017	80.1%	90.4%	67.9%
Peru	Huarmey	2000	58.2%	81.3%	34.0%
Peru	Huarmey	2017	67.8%	89.2%	44.2%
Peru	Huarochiri	2000	59.6%	71.0%	47.9%
Peru	Huarochiri	2017	64.5%	74.1%	52.5%
Peru	Huaura	2000	71.9%	78.2%	64.8%
Peru	Huaura	2017	85.6%	89.9%	80.2%
Peru	Huaylas	2000	69.4%	80.6%	60.2%
Peru	Huaylas	2017	82.9%	91.1%	74.6%
Peru	Huaytara	2000	54.0%	65.1%	40.5%
Peru	Huaytara	2017	66.2%	77.1%	55.2%
Peru	Huenuco	2000	60.2%	63.7%	56.9%
Peru	Huenuco	2017	76.6%	79.6%	73.5%
Peru	Ica	2000	66.2%	71.8%	62.1%
Peru	Ica	2017	84.0%	88.0%	80.8%
Peru	Ilo	2000	79.2%	86.1%	69.8%
Peru	Ilo	2017	90.7%	94.5%	84.3%
Peru	Islay	2000	67.5%	81.9%	48.3%
Peru	Islay	2017	76.0%	89.5%	58.3%
Peru	Jaén	2000	66.3%	74.3%	57.3%
Peru	Jaén	2017	79.4%	85.4%	72.8%
Peru	Jauja	2000	69.9%	75.8%	61.3%
Peru	Jauja	2017	82.2%	87.4%	74.9%
Peru	Jorge Basadre	2000	57.9%	85.0%	33.3%
Peru	Jorge Basadre	2017	69.7%	91.4%	45.1%
Peru	Julcan	2000	55.7%	70.3%	41.6%
Peru	Julcan	2017	72.3%	82.2%	60.0%
Peru	Junín	2000	64.2%	78.2%	53.0%
Peru	Junín	2017	76.1%	86.4%	66.6%
Peru	La Convención	2000	65.0%	71.7%	58.9%
Peru	La Convención	2017	72.4%	80.5%	63.9%

Country	Second Administrative Unit	Year	Mean	Upper	Lower
Peru	La Mar	2000	65.3%	73.8%	55.7%
Peru	La Mar	2017	78.6%	86.0%	70.7%
Peru	La Unión	2000	58.9%	74.2%	42.3%
Peru	La Unión	2017	69.5%	84.2%	53.9%
Peru	Lago Titicaca	2000	60.0%	67.7%	52.2%
Peru	Lago Titicaca	2017	74.1%	80.0%	67.6%
Peru	Lamas	2000	44.6%	51.3%	37.1%
Peru	Lamas	2017	60.2%	67.0%	53.1%
Peru	Lambayeque	2000	67.9%	74.2%	60.3%
Peru	Lambayeque	2017	80.7%	85.7%	74.0%
Peru	Lampa	2000	40.9%	55.8%	26.9%
Peru	Lampa	2017	52.5%	68.9%	36.7%
Peru	Lauricocha	2000	53.8%	65.7%	42.1%
Peru	Lauricocha	2017	67.0%	78.8%	55.2%
Peru	Leoncio Prado	2000	40.5%	47.3%	33.8%
Peru	Leoncio Prado	2017	57.0%	64.5%	48.6%
Peru	Lima	2000	81.8%	82.4%	81.1%
Peru	Lima	2017	90.1%	90.7%	89.6%
Peru	Loreto	2000	32.2%	41.0%	24.4%
Peru	Loreto	2017	41.9%	51.2%	33.6%
Peru	Lucanas	2000	67.3%	76.5%	56.7%
Peru	Lucanas	2017	73.7%	82.7%	63.4%
Peru	Luya	2000	69.2%	77.5%	60.0%
Peru	Luya	2017	81.1%	87.7%	73.8%
Peru	Manu	2000	42.9%	52.6%	33.5%
Peru	Manu	2017	60.0%	67.9%	49.7%
Peru	Marañón	2000	56.2%	70.6%	38.0%
Peru	Marañón	2017	65.5%	77.7%	50.3%
Peru	Mariscal Cáceres	2000	43.1%	52.1%	33.1%
Peru	Mariscal Cáceres	2017	51.5%	59.3%	44.5%
Peru	Mariscal Luzuriaga	2000	55.2%	67.0%	41.6%
Peru	Mariscal Luzuriaga	2017	71.2%	80.8%	61.0%
Peru	Mariscal Nieto	2000	80.3%	84.2%	75.1%
Peru	Mariscal Nieto	2017	88.8%	91.9%	84.8%
Peru	Mariscal Ramón Castilla	2000	32.7%	41.3%	24.1%
Peru	Mariscal Ramón Castilla	2017	40.6%	48.6%	31.1%
Peru	Maynas	2000	34.8%	49.3%	24.3%
Peru	Maynas	2017	49.1%	62.9%	37.6%
Peru	Melgar	2000	53.6%	65.3%	43.1%
Peru	Melgar	2017	65.9%	75.6%	56.3%
Peru	Moho	2000	49.4%	71.1%	26.7%
Peru	Moho	2017	62.8%	83.4%	37.8%
Peru	Morropón	2000	69.4%	78.8%	59.3%
Peru	Morropón	2017	81.4%	88.7%	73.6%
Peru	Moyobamba	2000	51.0%	60.5%	38.9%
Peru	Moyobamba	2017	69.5%	76.0%	58.5%
Peru	Nazca	2000	54.4%	61.4%	48.0%
Peru	Nazca	2017	74.2%	79.4%	69.1%
Peru	Ocros	2000	61.7%	84.9%	39.0%
Peru	Ocros	2017	69.8%	91.2%	42.2%
Peru	Otuzco	2000	60.7%	71.3%	49.7%
Peru	Otuzco	2017	71.1%	80.9%	61.9%

Country	Second Administrative Unit	Year	Mean	Upper	Lower
Peru	Oxapampa	2000	59.4%	66.1%	53.7%
Peru	Oxapampa	2017	67.5%	73.2%	61.9%
Peru	Oyon	2000	67.8%	85.5%	47.0%
Peru	Oyon	2017	78.0%	91.6%	62.0%
Peru	Pacasmayo	2000	62.7%	72.1%	52.6%
Peru	Pacasmayo	2017	78.9%	86.0%	68.6%
Peru	Pachitea	2000	42.6%	53.8%	33.7%
Peru	Pachitea	2017	57.3%	66.9%	49.0%
Peru	Padre Abad	2000	42.1%	53.7%	32.5%
Peru	Padre Abad	2017	52.8%	65.3%	40.8%
Peru	Paita	2000	49.4%	68.1%	31.1%
Peru	Paita	2017	64.1%	79.1%	44.8%
Peru	Pallasca	2000	66.2%	81.6%	48.2%
Peru	Pallasca	2017	74.0%	88.4%	55.8%
Peru	Palpa	2000	77.0%	86.7%	64.8%
Peru	Palpa	2017	88.5%	94.5%	80.0%
Peru	Parinacochas	2000	66.8%	81.1%	50.7%
Peru	Parinacochas	2017	71.5%	86.8%	54.7%
Peru	Paruro	2000	70.2%	83.9%	52.2%
Peru	Paruro	2017	79.3%	90.7%	63.4%
Peru	Pasco	2000	62.8%	66.2%	59.6%
Peru	Pasco	2017	75.9%	79.2%	72.6%
Peru	Pataz	2000	62.3%	77.1%	47.3%
Peru	Pataz	2017	73.9%	85.9%	60.9%
Peru	Paucar del Sara Sara	2000	62.9%	76.3%	49.1%
Peru	Paucar del Sara Sara	2017	76.5%	86.8%	65.0%
Peru	Paucartambo	2000	64.6%	77.6%	50.8%
Peru	Paucartambo	2017	76.9%	87.4%	62.9%
Peru	Picota	2000	52.3%	61.9%	41.3%
Peru	Picota	2017	67.2%	77.4%	53.5%
Peru	Pisco	2000	74.5%	82.2%	64.9%
Peru	Pisco	2017	88.4%	92.5%	81.2%
Peru	Piura	2000	55.5%	57.9%	52.7%
Peru	Piura	2017	72.0%	74.5%	69.1%
Peru	Pomabamba	2000	63.2%	77.2%	48.1%
Peru	Pomabamba	2017	75.9%	85.6%	62.5%
Peru	Puerto Inca	2000	40.3%	54.7%	27.4%
Peru	Puerto Inca	2017	48.4%	62.8%	35.4%
Peru	Puno	2000	54.1%	59.0%	48.0%
Peru	Puno	2017	64.9%	69.7%	59.7%
Peru	Purús	2000	47.3%	91.0%	6.5%
Peru	Purús	2017	49.6%	84.7%	14.0%
Peru	Quispicanchi	2000	62.8%	75.3%	48.0%
Peru	Quispicanchi	2017	73.8%	84.6%	61.6%
Peru	Recuay	2000	67.1%	86.4%	41.1%
Peru	Recuay	2017	75.4%	92.0%	51.9%
Peru	Requena	2000	35.2%	46.9%	25.3%
Peru	Requena	2017	40.9%	50.6%	31.4%
Peru	Rioja	2000	50.3%	53.9%	46.7%
Peru	Rioja	2017	69.5%	73.3%	65.6%
Peru	Rodríguez de Mendoza	2000	65.3%	71.4%	58.3%
Peru	Rodríguez de Mendoza	2017	77.8%	84.1%	70.4%
Peru	San Antonio de Putina	2000	58.2%	72.8%	41.5%
Peru	San Antonio de Putina	2017	70.9%	81.6%	58.3%
Peru	San Ignacio	2000	64.4%	74.1%	51.4%
Peru	San Ignacio	2017	74.2%	83.4%	63.5%

Country	Second Administrative Unit	Year	Mean	Upper	Lower
Peru	San Marcos	2000	63.6%	74.2%	50.5%
Peru	San Marcos	2017	79.0%	87.4%	69.4%
Peru	San Martín	2000	48.7%	51.5%	45.8%
Peru	San Martín	2017	68.8%	71.3%	66.6%
Peru	San Miguel	2000	56.8%	69.9%	42.7%
Peru	San Miguel	2017	69.5%	81.8%	56.3%
Peru	San Pablo	2000	67.8%	79.6%	53.0%
Peru	San Pablo	2017	80.4%	90.1%	67.0%
Peru	San Román	2000	53.6%	66.9%	43.4%
Peru	San Román	2017	71.3%	79.7%	61.2%
Peru	Sánchez Carrión	2000	65.9%	75.1%	57.0%
Peru	Sánchez Carrión	2017	80.0%	88.0%	70.1%
Peru	Sandia	2000	47.6%	62.0%	33.9%
Peru	Sandia	2017	55.1%	68.9%	43.1%
Peru	Santa	2000	75.7%	83.1%	67.7%
Peru	Santa	2017	89.3%	93.3%	83.8%
Peru	Santa Cruz	2000	53.5%	63.9%	38.4%
Peru	Santa Cruz	2017	70.3%	79.9%	57.2%
Peru	Santiago de Chuco	2000	58.5%	70.7%	44.5%
Peru	Santiago de Chuco	2017	70.1%	83.2%	55.8%
Peru	Satipo	2000	61.8%	68.1%	54.3%
Peru	Satipo	2017	72.5%	79.0%	65.1%
Peru	Sechura	2000	61.5%	77.8%	41.4%
Peru	Sechura	2017	71.2%	85.1%	53.2%
Peru	Sihuas	2000	65.8%	83.6%	37.9%
Peru	Sihuas	2017	76.2%	90.4%	50.8%
Peru	Sucre	2000	53.3%	77.1%	31.2%
Peru	Sucre	2017	64.8%	85.9%	41.3%
Peru	Sullana	2000	56.0%	61.1%	51.1%
Peru	Sullana	2017	70.8%	76.2%	64.9%
Peru	Tacna	2000	83.1%	87.7%	77.7%
Peru	Tacna	2017	90.6%	93.5%	87.3%
Peru	Tahuamanu	2000	39.9%	53.2%	28.0%
Peru	Tahuamanu	2017	51.8%	64.5%	40.4%
Peru	Talara	2000	57.4%	67.3%	46.1%
Peru	Talara	2017	72.1%	80.5%	62.1%
Peru	Tambopata	2000	47.9%	51.9%	44.0%
Peru	Tambopata	2017	67.9%	70.6%	64.9%
Peru	Tarata	2000	66.4%	84.6%	45.4%
Peru	Tarata	2017	76.6%	91.8%	57.1%
Peru	Tarma	2000	73.3%	83.4%	62.7%
Peru	Tarma	2017	83.3%	91.3%	74.8%
Peru	Tayacaja	2000	59.2%	65.2%	52.4%
Peru	Tayacaja	2017	71.5%	77.4%	64.8%
Peru	Tocache	2000	34.9%	44.3%	26.5%
Peru	Tocache	2017	48.5%	57.7%	39.7%
Peru	Trujillo	2000	72.2%	73.6%	70.6%
Peru	Trujillo	2017	88.8%	89.8%	87.8%
Peru	Tumbes	2000	60.3%	64.2%	57.5%
Peru	Tumbes	2017	82.7%	85.2%	80.5%
Peru	Ucayali	2000	38.3%	47.7%	30.3%
Peru	Ucayali	2017	46.9%	56.4%	38.0%
Peru	Urubamba	2000	71.0%	78.0%	63.1%
Peru	Urubamba	2017	84.5%	89.9%	77.8%
Peru	Utcubamba	2000	62.1%	66.9%	56.9%
Peru	Utcubamba	2017	76.2%	80.4%	71.2%
Peru	Victor Fajardo	2000	66.4%	77.7%	53.9%

Country	Second Administrative Unit	Year	Mean	Upper	Lower
Peru	Victor Fajardo	2017	80.7%	88.0%	71.2%
Peru	Vilcas Huamán	2000	58.1%	70.4%	44.1%
Peru	Vilcas Huamán	2017	72.8%	82.6%	60.1%
Peru	Viru	2000	55.7%	70.3%	41.0%
Peru	Viru	2017	66.9%	80.4%	54.8%
Peru	Yarowilca	2000	66.3%	72.8%	55.4%
Peru	Yarowilca	2017	85.1%	88.5%	78.7%
Peru	Yauli	2000	77.1%	87.6%	61.9%
Peru	Yauli	2017	84.9%	92.9%	73.5%
Peru	Yauyos	2000	52.8%	76.0%	29.9%
Peru	Yauyos	2017	60.8%	82.6%	37.8%
Peru	Yungay	2000	73.7%	82.1%	65.0%
Peru	Yungay	2017	85.5%	91.4%	78.3%
Peru	Yunguyo	2000	79.5%	83.7%	74.4%
Peru	Yunguyo	2017	91.2%	93.2%	88.5%
Peru	Zarumilla	2000	47.9%	52.9%	43.4%
Peru	Zarumilla	2017	71.2%	75.1%	67.0%
**North Africa and Middle East**					
Afghanistan	Ab Band	2000	0.1%	0.7%	0.0%
Afghanistan	Ab Band	2017	16.2%	37.8%	2.5%
Afghanistan	Ab Kamari	2000	0.1%	1.3%	0.0%
Afghanistan	Ab Kamari	2017	16.4%	35.7%	2.9%
Afghanistan	Achin	2000	0.2%	2.5%	0.0%
Afghanistan	Achin	2017	22.0%	63.6%	2.4%
Afghanistan	Adraskan	2000	0.2%	0.8%	0.0%
Afghanistan	Adraskan	2017	19.7%	31.2%	11.5%
Afghanistan	Ajristan	2000	0.1%	1.0%	0.0%
Afghanistan	Ajristan	2017	18.3%	38.8%	3.2%
Afghanistan	Alasay	2000	0.2%	1.1%	0.0%
Afghanistan	Alasay	2017	15.9%	63.8%	0.1%
Afghanistan	Ali abad	2000	0.1%	0.5%	0.0%
Afghanistan	Ali abad	2017	10.3%	29.7%	0.5%
Afghanistan	Alingar	2000	0.3%	2.1%	0.0%
Afghanistan	Alingar	2017	24.3%	47.3%	11.7%
Afghanistan	Alishing	2000	0.1%	1.2%	0.0%
Afghanistan	Alishing	2017	13.4%	43.3%	1.9%
Afghanistan	Almar	2000	0.2%	1.5%	0.0%
Afghanistan	Almar	2017	18.4%	39.1%	4.7%

Country	Second Administrative Unit	Year	Mean	Upper	Lower
Afghanistan	Anar Dara	2000	0.1%	0.6%	0.0%
Afghanistan	Anar Dara	2017	15.8%	24.8%	9.1%
Afghanistan	Andar	2000	0.1%	1.4%	0.0%
Afghanistan	Andar	2017	12.4%	31.8%	0.9%
Afghanistan	Andarab	2000	0.1%	1.1%	0.0%
Afghanistan	Andarab	2017	8.6%	21.6%	1.7%
Afghanistan	Andkhoy	2000	0.0%	0.1%	0.0%
Afghanistan	Andkhoy	2017	9.2%	19.8%	2.8%
Afghanistan	Aqcha	2000	12.5%	17.8%	8.9%
Afghanistan	Aqcha	2017	35.8%	40.9%	31.2%
Afghanistan	Arghandab	2000	0.0%	0.1%	0.0%
Afghanistan	Arghandab	2000	0.4%	2.5%	0.0%
Afghanistan	Arghandab	2017	28.1%	47.5%	13.1%
Afghanistan	Arghandab	2017	4.8%	15.5%	0.8%
Afghanistan	Arghistan	2000	0.5%	3.0%	0.0%
Afghanistan	Arghistan	2017	28.4%	46.7%	15.0%
Afghanistan	Asad abad	2000	0.0%	0.2%	0.0%
Afghanistan	Asad abad	2017	11.6%	22.3%	6.3%
Afghanistan	Atghar	2000	0.2%	2.1%	0.0%
Afghanistan	Atghar	2017	24.1%	50.7%	4.1%
Afghanistan	Aybak	2000	0.1%	0.8%	0.0%
Afghanistan	Aybak	2017	11.6%	34.4%	1.0%
Afghanistan	Azro	2000	0.2%	1.8%	0.0%
Afghanistan	Azro	2017	17.4%	44.3%	1.4%
Afghanistan	Baghlan City	2000	5.4%	16.5%	0.3%
Afghanistan	Baghlan City	2017	33.9%	54.5%	17.8%
Afghanistan	Baghlani Jadid	2000	0.1%	0.8%	0.0%
Afghanistan	Baghlani Jadid	2017	12.2%	41.7%	1.0%
Afghanistan	Bagram	2000	0.0%	0.1%	0.0%
Afghanistan	Bagram	2017	5.4%	13.6%	1.3%
Afghanistan	Bagrami	2000	0.0%	0.1%	0.0%

Country	Second Administrative Unit	Year	Mean	Upper	Lower
Afghanistan	Bagrami	2017	12.5%	32.6%	4.6%
Afghanistan	Baharak	2000	10.3%	14.4%	8.0%
Afghanistan	Baharak	2017	41.4%	57.1%	27.8%
Afghanistan	Bak	2000	0.1%	0.7%	0.0%
Afghanistan	Bak	2017	17.8%	52.2%	2.0%
Afghanistan	Bakwa	2000	0.1%	0.6%	0.0%
Afghanistan	Bakwa	2017	11.9%	26.6%	3.2%
Afghanistan	Bala Buluk	2000	0.1%	0.4%	0.0%
Afghanistan	Bala Buluk	2017	12.8%	27.2%	5.2%
Afghanistan	Balkh	2000	0.3%	1.9%	0.0%
Afghanistan	Balkh	2017	41.1%	66.8%	18.6%
Afghanistan	Balkhab	2000	0.1%	0.8%	0.0%
Afghanistan	Balkhab	2017	14.8%	27.4%	4.9%
Afghanistan	Bamyan City	2000	1.1%	5.4%	0.0%
Afghanistan	Bamyan City	2017	40.2%	52.6%	27.1%
Afghanistan	Bangi	2000	0.1%	1.1%	0.0%
Afghanistan	Bangi	2017	12.8%	35.0%	0.4%
Afghanistan	Bar Kunar	2000	0.1%	0.8%	0.0%
Afghanistan	Bar Kunar	2017	16.8%	34.3%	6.8%
Afghanistan	Baraki Barak	2000	0.0%	0.1%	0.0%
Afghanistan	Baraki Barak	2017	10.5%	25.8%	3.1%
Afghanistan	Bargi Matal	2000	0.1%	1.0%	0.0%
Afghanistan	Bargi Matal	2017	15.7%	33.2%	3.0%
Afghanistan	Bati Kot	2000	0.4%	3.6%	0.0%
Afghanistan	Bati Kot	2017	29.3%	86.9%	0.3%
Afghanistan	Bilchiragh	2000	0.8%	3.6%	0.0%
Afghanistan	Bilchiragh	2017	19.9%	31.1%	9.9%
Afghanistan	Bughran	2000	0.3%	1.9%	0.0%
Afghanistan	Bughran	2017	21.3%	38.4%	9.4%
Afghanistan	Burka	2000	0.6%	4.5%	0.0%
Afghanistan	Burka	2017	35.0%	62.1%	10.7%

Country	Second Administrative Unit	Year	Mean	Upper	Lower
Afghanistan	Burmul	2000	0.3%	1.9%	0.0%
Afghanistan	Burmul	2017	30.9%	49.0%	14.3%
Afghanistan	Chaghcharan	2000	0.2%	0.8%	0.0%
Afghanistan	Chaghcharan	2017	17.4%	27.0%	9.1%
Afghanistan	Chah Ab	2000	0.1%	0.9%	0.0%
Afghanistan	Chah Ab	2017	19.9%	42.1%	6.1%
Afghanistan	Chahar Asyab	2000	1.2%	7.3%	0.0%
Afghanistan	Chahar Asyab	2017	72.3%	93.9%	34.4%
Afghanistan	Chaharikar	2000	0.0%	0.1%	0.0%
Afghanistan	Chaharikar	2017	4.3%	13.2%	1.8%
Afghanistan	Chak	2000	0.2%	1.4%	0.0%
Afghanistan	Chak	2017	14.5%	42.9%	1.5%
Afghanistan	Chakhansur	2000	0.1%	0.8%	0.0%
Afghanistan	Chakhansur	2017	18.7%	30.4%	9.8%
Afghanistan	Chal	2000	0.0%	0.3%	0.0%
Afghanistan	Chal	2017	16.2%	51.2%	0.2%
Afghanistan	Chamkani	2000	0.0%	0.1%	0.0%
Afghanistan	Chamkani	2017	22.4%	30.2%	14.9%
Afghanistan	Chapa Dara	2000	0.3%	4.4%	0.0%
Afghanistan	Chapa Dara	2017	25.7%	70.9%	1.0%
Afghanistan	Chaparhar	2000	0.0%	0.2%	0.0%
Afghanistan	Chaparhar	2017	23.1%	58.1%	2.9%
Afghanistan	Char Bolak	2000	0.0%	0.1%	0.0%
Afghanistan	Char Bolak	2017	10.1%	19.2%	2.1%
Afghanistan	Char Burjak	2000	0.1%	0.5%	0.0%
Afghanistan	Char Burjak	2017	17.0%	23.8%	11.2%
Afghanistan	Char Dara	2000	0.2%	1.7%	0.0%
Afghanistan	Char Dara	2017	16.1%	46.5%	2.8%
Afghanistan	Charkh	2000	0.0%	0.4%	0.0%
Afghanistan	Charkh	2017	8.4%	23.3%	1.0%
Afghanistan	Charkint	2000	0.2%	1.9%	0.0%

Country	Second Administrative Unit	Year	Mean	Upper	Lower
Afghanistan	Charkint	2017	19.1%	50.0%	2.7%
Afghanistan	Chawkay	2000	0.2%	1.1%	0.0%
Afghanistan	Chawkay	2017	52.3%	60.4%	41.7%
Afghanistan	Chimtal	2000	0.1%	0.6%	0.0%
Afghanistan	Chimtal	2017	11.8%	26.0%	2.9%
Afghanistan	Chishti Sharif	2000	0.2%	0.9%	0.0%
Afghanistan	Chishti Sharif	2017	21.2%	43.2%	5.0%
Afghanistan	Chora	2000	1.4%	3.9%	0.1%
Afghanistan	Chora	2017	27.9%	43.6%	15.4%
Afghanistan	Dahana-I-Ghori	2000	0.1%	1.5%	0.0%
Afghanistan	Dahana-I-Ghori	2017	21.0%	46.5%	1.8%
Afghanistan	Daman	2000	14.5%	20.0%	3.9%
Afghanistan	Daman	2017	46.9%	64.1%	39.4%
Afghanistan	Dand Wa Patan	2000	0.2%	1.1%	0.0%
Afghanistan	Dand Wa Patan	2017	30.4%	64.9%	6.6%
Afghanistan	Dangam	2000	0.1%	0.8%	0.0%
Afghanistan	Dangam	2017	13.2%	42.4%	0.9%
Afghanistan	Dara-I-Nur	2000	0.1%	1.1%	0.0%
Afghanistan	Dara-I-Nur	2017	25.3%	66.0%	6.1%
Afghanistan	Dara-i-Pech	2000	0.1%	0.5%	0.0%
Afghanistan	Dara-i-Pech	2017	20.1%	45.5%	4.0%
Afghanistan	Dara-i-Suf	2000	0.2%	1.3%	0.0%
Afghanistan	Dara-i-Suf	2017	15.8%	31.7%	5.3%
Afghanistan	Darqad	2000	0.3%	3.7%	0.0%
Afghanistan	Darqad	2017	30.6%	69.9%	5.2%
Afghanistan	Darwaz	2000	0.2%	1.6%	0.0%
Afghanistan	Darwaz	2017	18.8%	37.2%	7.4%
Afghanistan	Darzab	2000	3.8%	21.9%	0.0%
Afghanistan	Darzab	2017	40.0%	53.4%	26.4%
Afghanistan	Dashti Archi	2000	2.5%	10.7%	0.0%
Afghanistan	Dashti Archi	2017	35.4%	53.9%	14.7%
Afghanistan					

Piped Water Proportion by Second Administrative Unit *(continued)*

Country	Second Administrative Unit	Year	Mean	Upper	Lower
Afghanistan	Dawlat abad	2000	0.6%	2.7%	0.0%
Afghanistan	Dawlat abad	2000	0.1%	0.3%	0.0%
Afghanistan	Dawlat abad	2017	26.1%	39.0%	13.9%
Afghanistan	Dawlat abad	2017	8.5%	27.8%	1.2%
Afghanistan	Dawlat Shah	2000	0.4%	2.3%	0.0%
Afghanistan	Dawlat Shah	2017	20.4%	41.6%	3.0%
Afghanistan	Day Kundi	2000	0.1%	0.8%	0.0%
Afghanistan	Day Kundi	2017	15.2%	25.4%	7.5%
Afghanistan	Daychopan	2000	0.9%	3.0%	0.1%
Afghanistan	Daychopan	2017	31.2%	47.0%	17.2%
Afghanistan	Daymirdad	2000	0.1%	1.0%	0.0%
Afghanistan	Daymirdad	2017	14.6%	38.1%	1.0%
Afghanistan	Dih Bala	2000	0.2%	1.8%	0.0%
Afghanistan	Dih Bala	2017	25.9%	63.2%	3.3%
Afghanistan	Dih Sabz	2000	0.0%	0.0%	0.0%
Afghanistan	Dih Sabz	2017	10.3%	21.5%	6.7%
Afghanistan	Dihdadi	2000	0.2%	0.9%	0.0%
Afghanistan	Dihdadi	2017	27.4%	32.4%	21.9%
Afghanistan	Dihrawud	2000	0.6%	2.6%	0.0%
Afghanistan	Dihrawud	2017	18.0%	32.0%	7.3%
Afghanistan	Dihyak	2000	0.1%	1.3%	0.0%
Afghanistan	Dihyak	2017	15.6%	39.1%	1.9%
Afghanistan	Dila	2000	0.1%	0.8%	0.0%
Afghanistan	Dila	2017	16.0%	37.9%	2.1%
Afghanistan	Disho	2000	0.1%	0.7%	0.0%
Afghanistan	Disho	2017	14.1%	21.6%	6.6%
Afghanistan	Doshi	2000	0.2%	1.1%	0.0%
Afghanistan	Doshi	2017	16.4%	38.1%	3.3%
Afghanistan	Dur Baba	2000	0.4%	3.1%	0.0%
Afghanistan	Dur Baba	2017	21.7%	63.7%	0.5%
Afghanistan	Farah City	2000	0.1%	0.4%	0.0%

Country	Second Administrative Unit	Year	Mean	Upper	Lower
Afghanistan	Farah City	2017	14.4%	33.4%	4.8%
Afghanistan	Farkhar	2000	0.0%	0.3%	0.0%
Afghanistan	Farkhar	2017	16.3%	39.5%	3.4%
Afghanistan	Farsi	2000	0.2%	1.6%	0.0%
Afghanistan	Farsi	2017	22.5%	40.6%	8.7%
Afghanistan	Fayz abad	2000	9.1%	13.4%	6.4%
Afghanistan	Fayz abad	2017	30.3%	44.0%	26.2%
Afghanistan	Fayzabad	2000	0.1%	0.5%	0.0%
Afghanistan	Fayzabad	2017	23.4%	37.3%	15.6%
Afghanistan	Gardez	2000	0.1%	1.4%	0.0%
Afghanistan	Gardez	2017	21.2%	51.4%	4.0%
Afghanistan	Garmser	2000	0.1%	0.5%	0.0%
Afghanistan	Garmser	2017	16.7%	28.6%	7.0%
Afghanistan	Gayan	2000	0.0%	0.3%	0.0%
Afghanistan	Gayan	2017	17.5%	42.0%	6.2%
Afghanistan	Gelan	2000	0.1%	1.3%	0.0%
Afghanistan	Gelan	2017	13.9%	37.6%	1.0%
Afghanistan	Ghazni	2000	0.0%	0.0%	0.0%
Afghanistan	Ghazni	2017	4.5%	27.2%	0.7%
Afghanistan	Ghorak	2000	0.1%	1.9%	0.0%
Afghanistan	Ghorak	2017	17.7%	44.0%	2.6%
Afghanistan	Ghorband	2000	7.5%	25.0%	0.3%
Afghanistan	Ghorband	2017	48.3%	67.3%	32.5%
Afghanistan	Ghormach	2000	0.0%	0.3%	0.0%
Afghanistan	Ghormach	2017	7.6%	23.4%	1.1%
Afghanistan	Ghoryan	2000	0.1%	0.6%	0.0%
Afghanistan	Ghoryan	2017	13.5%	24.8%	6.5%
Afghanistan	Giro	2000	0.3%	2.8%	0.0%
Afghanistan	Giro	2017	19.5%	46.8%	2.6%
Afghanistan	Gizab	2000	0.1%	0.6%	0.0%
Afghanistan	Gizab	2017	12.1%	23.3%	3.6%
Afghanistan					

Country	Second Administrative Unit	Year	Mean	Upper	Lower
Afghanistan	Gomal	2000	0.1%	0.9%	0.0%
Afghanistan	Gomal	2017	18.6%	30.8%	6.6%
Afghanistan	Goshta	2000	0.2%	1.6%	0.0%
Afghanistan	Goshta	2017	20.6%	59.7%	0.6%
Afghanistan	Gul dara	2000	0.0%	0.1%	0.0%
Afghanistan	Gul dara	2017	18.2%	54.1%	1.0%
Afghanistan	Gulistan	2000	0.1%	0.7%	0.0%
Afghanistan	Gulistan	2017	15.9%	28.1%	7.1%
Afghanistan	Gulran	2000	0.1%	0.6%	0.0%
Afghanistan	Gulran	2017	18.5%	28.8%	10.0%
Afghanistan	Gurbuz	2000	0.2%	2.1%	0.0%
Afghanistan	Gurbuz	2017	25.3%	67.7%	2.4%
Afghanistan	Guzara	2000	0.1%	0.7%	0.0%
Afghanistan	Guzara	2017	22.0%	52.6%	6.4%
Afghanistan	Hazrati Imam	2000	0.3%	1.5%	0.0%
Afghanistan	Hazrati Imam	2017	24.0%	49.7%	6.6%
Afghanistan	Hazrati Sultan	2000	0.1%	0.9%	0.0%
Afghanistan	Hazrati Sultan	2017	10.9%	32.9%	0.4%
Afghanistan	Hirat City	2000	0.0%	0.0%	0.0%
Afghanistan	Hirat City	2017	9.2%	11.9%	7.0%
Afghanistan	Hisa-i-Awali Bihsud	2000	0.1%	1.8%	0.0%
Afghanistan	Hisa-i-Awali Bihsud	2017	14.5%	36.8%	1.9%
Afghanistan	Hisa-I-Awali Panjsher	2000	0.1%	0.4%	0.0%
Afghanistan	Hisa-I-Awali Panjsher	2017	15.2%	33.2%	3.3%
Afghanistan	Hisa-I-Duwum Panjsher	2000	0.2%	1.9%	0.0%
Afghanistan	Hisa-I-Duwum Panjsher	2017	26.0%	62.5%	3.8%
Afghanistan	Hisarak	2000	0.4%	3.1%	0.0%
Afghanistan	Hisarak	2017	27.2%	71.4%	3.3%
Afghanistan	Injil	2000	5.9%	13.6%	0.0%
Afghanistan	Injil	2017	38.6%	56.6%	24.4%

Country	Second Administrative Unit	Year	Mean	Upper	Lower
Afghanistan	Ishkamish	2000	0.0%	0.2%	0.0%
Afghanistan	Ishkamish	2017	13.5%	36.9%	2.1%
Afghanistan	Ishkashim	2000	0.2%	1.5%	0.0%
Afghanistan	Ishkashim	2017	19.3%	40.8%	3.4%
Afghanistan	Istalif	2000	0.0%	0.1%	0.0%
Afghanistan	Istalif	2017	18.6%	60.9%	0.4%
Afghanistan	Jabalussaraj	2000	0.0%	0.0%	0.0%
Afghanistan	Jabalussaraj	2017	4.5%	13.8%	0.4%
Afghanistan	Jadran	2000	0.1%	2.0%	0.0%
Afghanistan	Jadran	2017	16.0%	42.6%	1.3%
Afghanistan	Jaghatu	2000	0.0%	0.3%	0.0%
Afghanistan	Jaghatu	2017	9.2%	18.7%	2.9%
Afghanistan	Jaghuri	2000	0.1%	1.1%	0.0%
Afghanistan	Jaghuri	2017	14.0%	30.3%	3.2%
Afghanistan	Jaji	2000	0.1%	0.4%	0.0%
Afghanistan	Jaji	2017	12.4%	34.1%	0.2%
Afghanistan	Jaji Maydan	2000	0.1%	0.6%	0.0%
Afghanistan	Jaji Maydan	2017	12.3%	38.1%	0.2%
Afghanistan	Jalal abad	2000	0.0%	0.0%	0.0%
Afghanistan	Jalal abad	2017	12.3%	14.5%	10.8%
Afghanistan	Jalrez	2000	0.1%	0.7%	0.0%
Afghanistan	Jalrez	2017	8.9%	26.6%	0.7%
Afghanistan	Jani Khel	2000	4.6%	8.8%	1.6%
Afghanistan	Jani Khel	2017	33.0%	55.1%	17.4%
Afghanistan	Jawand	2000	0.2%	1.0%	0.0%
Afghanistan	Jawand	2017	17.2%	26.6%	9.3%
Afghanistan	Jurm	2000	5.5%	11.4%	2.0%
Afghanistan	Jurm	2017	33.6%	46.5%	20.7%
Afghanistan	Kabul City	2000	0.1%	0.3%	0.0%
Afghanistan	Kabul City	2017	29.3%	36.9%	19.6%
Afghanistan	Kahmard	2000	0.1%	0.7%	0.0%
Afghanistan					

Piped Water Proportion by Second Administrative Unit *(continued)*

Country	Second Administrative Unit	Year	Mean	Upper	Lower
Afghanistan	Kahmard	2017	13.0%	26.4%	3.5%
Afghanistan	Kajaki	2000	0.1%	0.5%	0.0%
Afghanistan	Kajaki	2017	13.4%	29.4%	2.5%
Afghanistan	Kalafgan	2000	0.0%	0.4%	0.0%
Afghanistan	Kalafgan	2017	11.6%	34.5%	1.6%
Afghanistan	Kalakan	2000	0.0%	0.0%	0.0%
Afghanistan	Kalakan	2017	13.6%	68.0%	0.7%
Afghanistan	Kaldar	2000	0.0%	0.0%	0.0%
Afghanistan	Kaldar	2017	6.2%	32.9%	0.5%
Afghanistan	Kama	2000	0.0%	0.2%	0.0%
Afghanistan	Kama	2017	12.2%	30.1%	5.8%
Afghanistan	Kamdesh	2000	0.1%	0.8%	0.0%
Afghanistan	Kamdesh	2017	14.5%	37.3%	1.2%
Afghanistan	Kandahar City	2000	3.3%	6.2%	1.1%
Afghanistan	Kandahar City	2017	29.7%	41.1%	24.8%
Afghanistan	Kang	2000	0.1%	1.2%	0.0%
Afghanistan	Kang	2017	18.5%	55.9%	5.0%
Afghanistan	Karukh	2000	0.1%	0.9%	0.0%
Afghanistan	Karukh	2017	15.4%	35.8%	3.5%
Afghanistan	Khaki Jabar	2000	0.1%	1.0%	0.0%
Afghanistan	Khaki Jabar	2017	19.1%	49.7%	1.4%
Afghanistan	Khaki Safed	2000	0.3%	1.8%	0.0%
Afghanistan	Khaki Safed	2017	21.7%	41.9%	9.4%
Afghanistan	Khakrez	2000	0.1%	1.1%	0.0%
Afghanistan	Khakrez	2017	17.4%	37.1%	3.3%
Afghanistan	Khamyab	2000	0.1%	0.6%	0.0%
Afghanistan	Khamyab	2017	11.5%	35.9%	0.7%
Afghanistan	Khan Abad	2000	0.2%	2.0%	0.0%
Afghanistan	Khan Abad	2017	23.6%	53.2%	2.9%
Afghanistan	Khan Char Bagh	2000	0.0%	0.4%	0.0%
Afghanistan	Khan Char Bagh	2017	12.2%	37.7%	0.8%

Country	Second Administrative Unit	Year	Mean	Upper	Lower
Afghanistan	Khas Kunar	2000	0.4%	2.5%	0.0%
Afghanistan	Khas Kunar	2017	44.2%	76.8%	19.0%
Afghanistan	Khas Uruzgan	2000	0.2%	1.8%	0.0%
Afghanistan	Khas Uruzgan	2017	17.6%	40.3%	4.6%
Afghanistan	Khash Rod	2000	0.2%	1.2%	0.0%
Afghanistan	Khash Rod	2017	20.4%	34.7%	8.7%
Afghanistan	Khinjan	2000	0.4%	2.5%	0.0%
Afghanistan	Khinjan	2017	19.5%	50.1%	3.4%
Afghanistan	Khogyani	2000	0.1%	0.7%	0.0%
Afghanistan	Khogyani	2017	17.7%	58.3%	0.7%
Afghanistan	Khost (Matun)	2000	0.1%	1.2%	0.0%
Afghanistan	Khost (Matun)	2017	18.0%	51.0%	1.8%
Afghanistan	Khost Wa Firing	2000	0.0%	0.4%	0.0%
Afghanistan	Khost Wa Firing	2017	8.0%	18.5%	1.8%
Afghanistan	Khulm	2000	0.1%	1.2%	0.0%
Afghanistan	Khulm	2017	19.5%	36.4%	9.7%
Afghanistan	Khuram Wa Sarbagh	2000	0.1%	1.3%	0.0%
Afghanistan	Khuram Wa Sarbagh	2017	14.3%	31.2%	3.8%
Afghanistan	Khushi	2000	0.1%	0.8%	0.0%
Afghanistan	Khushi	2017	10.5%	39.0%	0.2%
Afghanistan	Khwahan	2000	0.2%	1.5%	0.0%
Afghanistan	Khwahan	2017	22.5%	42.7%	8.7%
Afghanistan	Khwaja Du Koh	2000	0.1%	0.6%	0.0%
Afghanistan	Khwaja Du Koh	2017	11.3%	34.4%	2.1%
Afghanistan	Khwaja Ghar	2000	0.0%	0.2%	0.0%
Afghanistan	Khwaja Ghar	2017	9.8%	30.3%	1.6%
Afghanistan	Khwaja Sabz Posh	2000	0.0%	0.3%	0.0%
Afghanistan	Khwaja Sabz Posh	2017	12.1%	30.1%	3.6%
Afghanistan	Kijran	2000	0.1%	0.9%	0.0%
Afghanistan	Kijran	2017	15.2%	32.9%	3.0%
Afghanistan	Kishim	2000	0.1%	0.9%	0.0%

Country	Second Administrative Unit	Year	Mean	Upper	Lower
Afghanistan	Kishim	2017	13.6%	25.0%	5.8%
Afghanistan	Kishindih	2000	0.1%	0.7%	0.0%
Afghanistan	Kishindih	2017	14.5%	30.8%	4.8%
Afghanistan	Kohband	2000	0.0%	0.0%	0.0%
Afghanistan	Kohband	2017	1.4%	8.4%	0.0%
Afghanistan	Kohi Safi	2000	0.0%	0.1%	0.0%
Afghanistan	Kohi Safi	2017	4.2%	16.9%	0.1%
Afghanistan	Kohistan	2000	0.0%	0.0%	0.0%
Afghanistan	Kohistan	2000	0.2%	1.7%	0.0%
Afghanistan	Kohistan	2017	2.4%	13.3%	0.2%
Afghanistan	Kohistan	2017	22.8%	43.8%	7.3%
Afghanistan	Kohistanat	2000	0.2%	1.4%	0.0%
Afghanistan	Kohistanat	2017	18.9%	33.4%	9.3%
Afghanistan	Kuhsan	2000	0.2%	1.1%	0.0%
Afghanistan	Kuhsan	2017	23.1%	41.7%	10.0%
Afghanistan	Kunduz	2000	0.1%	0.5%	0.0%
Afghanistan	Kunduz	2017	16.2%	60.2%	0.9%
Afghanistan	Kuran Wa Munjan	2000	0.2%	1.5%	0.0%
Afghanistan	Kuran Wa Munjan	2017	19.6%	38.8%	9.0%
Afghanistan	Kushk	2000	0.6%	2.2%	0.0%
Afghanistan	Kushk	2017	26.5%	44.9%	12.8%
Afghanistan	Kushki Kuhna	2000	0.1%	0.9%	0.0%
Afghanistan	Kushki Kuhna	2017	14.6%	28.5%	4.3%
Afghanistan	Kuz Kunar	2000	0.1%	0.3%	0.0%
Afghanistan	Kuz Kunar	2017	14.7%	37.7%	3.7%
Afghanistan	Lal Pur	2000	0.3%	2.7%	0.0%
Afghanistan	Lal Pur	2017	20.8%	64.4%	0.4%
Afghanistan	Lal Wa Sarjangal	2000	0.1%	0.7%	0.0%
Afghanistan	Lal Wa Sarjangal	2017	11.9%	24.1%	4.0%
Afghanistan	Lash Wa Juwayn	2000	0.1%	0.6%	0.0%
Afghanistan	Lash Wa Juwayn	2017	19.4%	32.0%	11.1%

Country	Second Administrative Unit	Year	Mean	Upper	Lower
Afghanistan	Lashkargah	2000	0.0%	0.3%	0.0%
Afghanistan	Lashkargah	2017	6.8%	17.3%	1.1%
Afghanistan	Lija Mangal	2000	1.0%	4.0%	0.2%
Afghanistan	Lija Mangal	2017	28.4%	49.3%	14.4%
Afghanistan	Mahmud Raqi	2000	0.0%	0.0%	0.0%
Afghanistan	Mahmud Raqi	2017	1.6%	10.2%	0.0%
Afghanistan	Malistan	2000	0.1%	0.7%	0.0%
Afghanistan	Malistan	2017	9.8%	26.2%	1.3%
Afghanistan	Mando Zayi	2000	0.1%	1.3%	0.0%
Afghanistan	Mando Zayi	2017	19.5%	53.9%	1.6%
Afghanistan	Mandol	2000	0.7%	2.5%	0.0%
Afghanistan	Mandol	2017	25.1%	42.0%	12.9%
Afghanistan	Mardyan	2000	13.9%	15.6%	10.8%
Afghanistan	Mardyan	2017	40.7%	53.1%	32.3%
Afghanistan	Markazi Bihsud	2000	0.1%	0.8%	0.0%
Afghanistan	Markazi Bihsud	2017	17.3%	35.6%	4.8%
Afghanistan	Marmul	2000	0.0%	0.3%	0.0%
Afghanistan	Marmul	2017	8.6%	28.6%	0.1%
Afghanistan	Maruf	2000	0.2%	1.2%	0.0%
Afghanistan	Maruf	2017	21.0%	35.2%	8.2%
Afghanistan	Marwara	2000	0.0%	0.3%	0.0%
Afghanistan	Marwara	2017	15.9%	29.1%	7.8%
Afghanistan	Mata Khan	2000	0.0%	0.3%	0.0%
Afghanistan	Mata Khan	2017	16.4%	47.0%	1.3%
Afghanistan	Maydan Shahr	2000	0.1%	0.9%	0.0%
Afghanistan	Maydan Shahr	2017	15.9%	50.3%	0.3%
Afghanistan	Maymana	2000	0.6%	3.1%	0.0%
Afghanistan	Maymana	2017	21.9%	49.1%	5.8%
Afghanistan	Maywand	2000	0.1%	0.5%	0.0%
Afghanistan	Maywand	2017	13.5%	27.3%	3.9%
Afghanistan	Mazar-i-Sharif	2000	0.0%	0.0%	0.0%

Country	Second Administrative Unit	Year	Mean	Upper	Lower
Afghanistan	Mazar-i-Sharif	2017	25.2%	32.7%	19.5%
Afghanistan	Mihtarlam	2000	3.0%	12.5%	0.3%
Afghanistan	Mihtarlam	2017	70.1%	82.4%	47.2%
Afghanistan	Mingajik	2000	0.1%	1.2%	0.0%
Afghanistan	Mingajik	2017	13.5%	37.7%	1.2%
Afghanistan	Mirbacha Kot	2000	0.0%	0.0%	0.0%
Afghanistan	Mirbacha Kot	2017	6.2%	19.0%	2.4%
Afghanistan	Mizan	2000	0.3%	2.3%	0.0%
Afghanistan	Mizan	2017	20.0%	58.3%	1.1%
Afghanistan	Muhammad Agha	2000	0.1%	0.6%	0.0%
Afghanistan	Muhammad Agha	2017	16.9%	43.4%	1.3%
Afghanistan	Muhmand Dara	2000	0.1%	1.0%	0.0%
Afghanistan	Muhmand Dara	2017	18.8%	54.2%	1.0%
Afghanistan	Muqur	2000	0.3%	1.9%	0.0%
Afghanistan	Muqur	2000	0.1%	0.9%	0.0%
Afghanistan	Muqur	2017	33.2%	58.2%	10.4%
Afghanistan	Muqur	2017	11.9%	38.9%	0.9%
Afghanistan	Murghab	2000	0.2%	1.3%	0.0%
Afghanistan	Murghab	2017	21.0%	40.0%	7.3%
Afghanistan	Musa Khel	2000	0.2%	1.6%	0.0%
Afghanistan	Musa Khel	2017	18.1%	52.3%	2.3%
Afghanistan	Musa Qala	2000	0.1%	0.9%	0.0%
Afghanistan	Musa Qala	2017	13.4%	29.7%	2.9%
Afghanistan	Musayi	2000	0.1%	1.0%	0.0%
Afghanistan	Musayi	2017	20.9%	57.8%	2.4%
Afghanistan	Nad Ali	2000	0.1%	0.4%	0.0%
Afghanistan	Nad Ali	2017	9.4%	19.2%	3.3%
Afghanistan	Nadir Shah Kot	2000	0.1%	0.7%	0.0%
Afghanistan	Nadir Shah Kot	2017	18.1%	44.9%	2.0%
Afghanistan	Nahri Sarraj	2000	0.1%	1.1%	0.0%
Afghanistan	Nahri Sarraj	2017	14.5%	32.3%	3.7%

Piped Water Proportion by Second Administrative Unit *(continued)*

Country	Second Administrative Unit	Year	Mean	Upper	Lower
Afghanistan	Nahri Shahi	2000	0.1%	0.4%	0.0%
Afghanistan	Nahri Shahi	2017	11.0%	21.8%	6.9%
Afghanistan	Nahrin	2000	0.4%	1.4%	0.0%
Afghanistan	Nahrin	2017	16.2%	40.0%	4.0%
Afghanistan	Nali	2000	0.1%	0.7%	0.0%
Afghanistan	Nali	2017	17.2%	42.1%	1.4%
Afghanistan	Narang Wa Badil	2000	0.0%	0.0%	0.0%
Afghanistan	Narang Wa Badil	2017	28.6%	45.8%	20.4%
Afghanistan	Naw Zad	2000	0.1%	0.9%	0.0%
Afghanistan	Naw Zad	2017	16.9%	34.3%	4.7%
Afghanistan	Nawa	2000	0.1%	0.9%	0.0%
Afghanistan	Nawa	2017	14.4%	30.8%	3.6%
Afghanistan	Nawa-i-Barak Zayi	2000	0.0%	0.1%	0.0%
Afghanistan	Nawa-i-Barak Zayi	2017	8.6%	31.2%	0.7%
Afghanistan	Nawur	2000	0.2%	0.9%	0.0%
Afghanistan	Nawur	2017	21.2%	34.0%	10.6%
Afghanistan	Nazyan	2000	0.5%	3.1%	0.0%
Afghanistan	Nazyan	2017	22.2%	91.0%	0.1%
Afghanistan	Nesh	2000	1.0%	4.5%	0.0%
Afghanistan	Nesh	2017	26.7%	54.4%	10.1%
Afghanistan	Nijrab	2000	0.0%	0.3%	0.0%
Afghanistan	Nijrab	2017	4.4%	19.7%	0.0%
Afghanistan	Nika	2000	0.1%	0.6%	0.0%
Afghanistan	Nika	2017	25.5%	64.5%	3.8%
Afghanistan	Nirkh	2000	0.1%	1.0%	0.0%
Afghanistan	Nirkh	2017	12.1%	31.9%	1.1%
Afghanistan	Nurgal	2000	0.3%	1.8%	0.0%
Afghanistan	Nurgal	2017	36.3%	81.9%	10.8%
Afghanistan	Nuristan	2000	0.6%	3.3%	0.0%
Afghanistan	Nuristan	2017	22.5%	41.2%	8.0%
Afghanistan	Obe	2000	0.1%	0.9%	0.0%

Piped Water Proportion by Second Administrative Unit *(continued)*

Country	Second Administrative Unit	Year	Mean	Upper	Lower
Afghanistan	Obe	2017	18.9%	46.3%	4.5%
Afghanistan	Omna	2000	0.1%	0.4%	0.0%
Afghanistan	Omna	2017	13.7%	34.1%	1.7%
Afghanistan	Pachir Wa Agam	2000	0.1%	0.6%	0.0%
Afghanistan	Pachir Wa Agam	2017	25.6%	69.3%	5.9%
Afghanistan	Paghman	2000	0.0%	0.3%	0.0%
Afghanistan	Paghman	2017	10.4%	22.9%	3.6%
Afghanistan	Panjab	2000	0.1%	1.0%	0.0%
Afghanistan	Panjab	2017	16.3%	34.3%	3.7%
Afghanistan	Panjsher	2000	0.0%	0.3%	0.0%
Afghanistan	Panjsher	2017	18.8%	34.0%	7.7%
Afghanistan	Panjwayi	2000	0.1%	0.3%	0.0%
Afghanistan	Panjwayi	2017	10.3%	18.2%	5.1%
Afghanistan	Pasaband	2000	0.2%	1.2%	0.0%
Afghanistan	Pasaband	2017	16.9%	29.8%	6.9%
Afghanistan	Pashtun Kot	2000	0.3%	1.8%	0.0%
Afghanistan	Pashtun Kot	2017	26.7%	62.6%	6.3%
Afghanistan	Pashtun Zarghun	2000	0.0%	0.4%	0.0%
Afghanistan	Pashtun Zarghun	2017	11.3%	28.7%	2.5%
Afghanistan	Puli Alam	2000	0.1%	1.3%	0.0%
Afghanistan	Puli Alam	2017	13.2%	30.1%	2.2%
Afghanistan	Puli Khumri	2000	0.6%	2.5%	0.0%
Afghanistan	Puli Khumri	2017	12.8%	21.5%	6.7%
Afghanistan	Purchaman	2000	0.2%	0.9%	0.0%
Afghanistan	Purchaman	2017	18.5%	30.5%	9.4%
Afghanistan	Pusht Rod	2000	0.0%	0.1%	0.0%
Afghanistan	Pusht Rod	2017	6.2%	18.5%	1.7%
Afghanistan	Qadis	2000	0.1%	0.8%	0.0%
Afghanistan	Qadis	2017	16.4%	30.2%	5.1%
Afghanistan	Qala-I-Kah	2000	0.1%	0.8%	0.0%
Afghanistan	Qala-I-Kah	2017	16.5%	30.1%	6.7%

Piped Water Proportion by Second Administrative Unit *(continued)*

Country	Second Administrative Unit	Year	Mean	Upper	Lower
Afghanistan	Qala-I-Naw	2000	0.3%	3.5%	0.0%
Afghanistan	Qala-I-Naw	2017	23.2%	59.0%	1.7%
Afghanistan	Qala-I-Zal	2000	0.1%	1.2%	0.0%
Afghanistan	Qala-I-Zal	2017	16.5%	34.8%	4.0%
Afghanistan	Qalandar	2000	0.3%	3.3%	0.0%
Afghanistan	Qalandar	2017	19.1%	77.3%	0.5%
Afghanistan	Qalat	2000	0.1%	1.1%	0.0%
Afghanistan	Qalat	2017	19.0%	41.8%	5.7%
Afghanistan	Qarabagh	2000	0.1%	1.2%	0.0%
Afghanistan	Qarabagh	2000	0.0%	0.0%	0.0%
Afghanistan	Qarabagh	2017	7.4%	25.8%	1.2%
Afghanistan	Qarabagh	2017	21.3%	41.8%	8.4%
Afghanistan	Qaram Qol	2000	0.1%	0.7%	0.0%
Afghanistan	Qaram Qol	2017	11.8%	22.4%	4.4%
Afghanistan	Qarghayi	2000	0.2%	1.2%	0.0%
Afghanistan	Qarghayi	2017	35.7%	51.8%	23.8%
Afghanistan	Qarqin	2000	0.1%	1.3%	0.0%
Afghanistan	Qarqin	2017	14.3%	41.2%	1.7%
Afghanistan	Qaysar	2000	0.3%	1.4%	0.0%
Afghanistan	Qaysar	2017	18.2%	50.1%	5.6%
Afghanistan	Ragh	2000	0.3%	3.0%	0.0%
Afghanistan	Ragh	2017	39.5%	61.7%	23.5%
Afghanistan	Reg	2000	0.2%	1.9%	0.0%
Afghanistan	Reg	2000	0.1%	0.8%	0.0%
Afghanistan	Reg	2017	20.8%	37.1%	8.9%
Afghanistan	Reg	2017	16.1%	24.6%	9.0%
Afghanistan	Rodat	2000	0.1%	1.1%	0.0%
Afghanistan	Rodat	2017	22.9%	46.1%	7.9%
Afghanistan	Royi Du Ab	2000	0.2%	1.7%	0.0%
Afghanistan	Royi Du Ab	2017	16.9%	44.9%	3.2%
Afghanistan	Rustaq	2000	0.1%	0.6%	0.0%
Afghanistan					

Country	Second Administrative Unit	Year	Mean	Upper	Lower
Afghanistan	Rustaq	2017	23.6%	47.0%	10.2%
Afghanistan	Sabari	2000	0.2%	1.6%	0.0%
Afghanistan	Sabari	2017	17.5%	50.5%	0.7%
Afghanistan	Saghar	2000	0.1%	0.6%	0.0%
Afghanistan	Saghar	2017	14.4%	30.3%	4.8%
Afghanistan	Salang	2000	0.1%	0.5%	0.0%
Afghanistan	Salang	2017	14.7%	46.6%	0.8%
Afghanistan	Sangcharak	2000	0.0%	0.4%	0.0%
Afghanistan	Sangcharak	2017	9.0%	22.4%	2.0%
Afghanistan	Sangin	2000	0.1%	0.4%	0.0%
Afghanistan	Sangin	2017	8.3%	23.3%	0.6%
Afghanistan	Sar Hawza	2000	0.1%	1.1%	0.0%
Afghanistan	Sar Hawza	2017	22.2%	43.9%	6.0%
Afghanistan	Sar-i-Pul City	2000	0.2%	1.2%	0.0%
Afghanistan	Sar-i-Pul City	2017	11.4%	22.2%	4.4%
Afghanistan	Sarobi	2000	0.1%	0.5%	0.0%
Afghanistan	Sarobi	2017	22.2%	52.7%	5.7%
Afghanistan	Sayid Abad	2000	0.2%	1.7%	0.0%
Afghanistan	Sayid Abad	2017	18.2%	47.9%	1.6%
Afghanistan	Sayid Karam	2000	2.4%	6.4%	0.7%
Afghanistan	Sayid Karam	2017	32.9%	57.1%	16.5%
Afghanistan	Sayyad	2000	0.0%	0.4%	0.0%
Afghanistan	Sayyad	2017	10.2%	33.3%	0.8%
Afghanistan	Shah Wali Kot	2000	0.2%	1.0%	0.0%
Afghanistan	Shah Wali Kot	2017	19.2%	36.0%	6.4%
Afghanistan	Shahidi Hasas	2000	4.4%	7.3%	2.8%
Afghanistan	Shahidi Hasas	2017	25.2%	40.3%	13.6%
Afghanistan	Shahjoy	2000	0.1%	1.1%	0.0%
Afghanistan	Shahjoy	2017	13.8%	35.5%	1.5%
Afghanistan	Shahrak	2000	0.1%	0.8%	0.0%
Afghanistan	Shahrak	2017	17.5%	27.9%	8.8%

Country	Second Administrative Unit	Year	Mean	Upper	Lower
Afghanistan	Shahri Buzurg	2000	0.1%	0.7%	0.0%
Afghanistan	Shahri Buzurg	2017	22.8%	42.4%	8.6%
Afghanistan	Shahristan	2000	0.1%	0.7%	0.0%
Afghanistan	Shahristan	2017	13.0%	24.7%	4.0%
Afghanistan	Shakar Dara	2000	0.0%	0.0%	0.0%
Afghanistan	Shakar Dara	2017	3.5%	9.7%	1.1%
Afghanistan	Shamul	2000	0.3%	2.2%	0.0%
Afghanistan	Shamul	2017	24.9%	89.1%	0.0%
Afghanistan	Shamul zayi	2000	0.3%	1.5%	0.0%
Afghanistan	Shamul zayi	2017	24.4%	43.3%	8.0%
Afghanistan	Sharan	2000	0.2%	1.5%	0.0%
Afghanistan	Sharan	2017	22.6%	43.0%	7.8%
Afghanistan	Shekh Ali	2000	0.3%	3.1%	0.0%
Afghanistan	Shekh Ali	2017	21.6%	48.4%	1.9%
Afghanistan	Sherzad	2000	0.0%	0.4%	0.0%
Afghanistan	Sherzad	2017	19.5%	37.1%	7.5%
Afghanistan	Shib Koh	2000	0.1%	0.8%	0.0%
Afghanistan	Shib Koh	2017	15.5%	34.7%	4.4%
Afghanistan	Shibar	2000	0.2%	1.1%	0.0%
Afghanistan	Shibar	2017	17.6%	35.3%	4.9%
Afghanistan	Shibirghan	2000	0.1%	0.4%	0.0%
Afghanistan	Shibirghan	2017	11.0%	24.4%	3.6%
Afghanistan	Shighnan	2000	0.8%	2.2%	0.2%
Afghanistan	Shighnan	2017	25.5%	43.3%	13.5%
Afghanistan	Shindand	2000	3.0%	13.2%	0.1%
Afghanistan	Shindand	2017	38.5%	51.7%	21.6%
Afghanistan	Shinkay	2000	0.2%	2.3%	0.0%
Afghanistan	Shinkay	2017	20.4%	43.3%	3.8%
Afghanistan	Shinwar	2000	0.0%	0.1%	0.0%
Afghanistan	Shinwar	2017	16.5%	46.0%	3.2%
Afghanistan	Shinwari	2000	0.1%	1.2%	0.0%

Country	Second Administrative Unit	Year	Mean	Upper	Lower
Afghanistan	Shinwari	2017	22.2%	67.5%	1.9%
Afghanistan	Shirin Tagab	2000	0.1%	1.0%	0.0%
Afghanistan	Shirin Tagab	2017	13.4%	27.2%	3.5%
Afghanistan	Sholgara	2000	0.2%	1.4%	0.0%
Afghanistan	Sholgara	2017	18.6%	45.5%	3.4%
Afghanistan	Shorabak	2000	0.2%	1.2%	0.0%
Afghanistan	Shorabak	2017	19.1%	33.2%	8.1%
Afghanistan	Shortepa	2000	0.1%	2.0%	0.0%
Afghanistan	Shortepa	2017	18.5%	46.7%	2.4%
Afghanistan	Shwak	2000	0.0%	0.0%	0.0%
Afghanistan	Shwak	2017	6.5%	15.5%	3.4%
Afghanistan	Sirkanay	2000	0.0%	0.3%	0.0%
Afghanistan	Sirkanay	2017	31.2%	56.7%	16.3%
Afghanistan	Sozma Qala	2000	0.1%	0.7%	0.0%
Afghanistan	Sozma Qala	2017	14.2%	38.6%	0.9%
Afghanistan	Spera	2000	0.0%	0.5%	0.0%
Afghanistan	Spera	2017	14.4%	43.6%	0.5%
Afghanistan	Spin Boldak	2000	0.1%	0.8%	0.0%
Afghanistan	Spin Boldak	2017	18.6%	39.4%	7.4%
Afghanistan	Surkh Rod	2000	0.0%	0.2%	0.0%
Afghanistan	Surkh Rod	2017	14.4%	29.0%	5.5%
Afghanistan	Surkhi Parsa	2000	1.6%	6.5%	0.0%
Afghanistan	Surkhi Parsa	2017	29.2%	54.6%	12.0%
Afghanistan	Surobi	2000	0.1%	0.8%	0.0%
Afghanistan	Surobi	2017	16.2%	31.7%	4.9%
Afghanistan	Tagab	2000	0.1%	0.7%	0.0%
Afghanistan	Tagab	2017	14.1%	53.4%	0.2%
Afghanistan	Tala Wa Barfak	2000	0.1%	0.6%	0.0%
Afghanistan	Tala Wa Barfak	2017	12.6%	30.0%	3.0%
Afghanistan	Taluqan	2000	0.0%	0.5%	0.0%
Afghanistan	Taluqan	2017	8.7%	15.6%	5.0%

Piped Water Proportion by Second Administrative Unit *(continued)*

Country	Second Administrative Unit	Year	Mean	Upper	Lower
Afghanistan	Tani	2000	0.1%	0.7%	0.0%
Afghanistan	Tani	2017	17.3%	55.7%	0.5%
Afghanistan	Tarnak Wa Jaldak	2000	0.1%	0.8%	0.0%
Afghanistan	Tarnak Wa Jaldak	2017	16.0%	40.2%	2.5%
Afghanistan	Taywara	2000	0.1%	0.8%	0.0%
Afghanistan	Taywara	2017	11.8%	24.2%	3.1%
Afghanistan	Tere Zayi	2000	0.1%	0.6%	0.0%
Afghanistan	Tere Zayi	2017	12.0%	29.2%	3.4%
Afghanistan	Tirin Kot	2000	0.9%	2.9%	0.0%
Afghanistan	Tirin Kot	2017	19.3%	37.5%	7.7%
Afghanistan	Tulak	2000	0.1%	1.0%	0.0%
Afghanistan	Tulak	2017	16.3%	31.4%	4.9%
Afghanistan	Urgun	2000	0.1%	0.4%	0.0%
Afghanistan	Urgun	2017	13.3%	32.6%	5.2%
Afghanistan	Wakhan	2000	0.3%	1.8%	0.0%
Afghanistan	Wakhan	2017	20.7%	34.5%	10.0%
Afghanistan	Wama	2000	0.1%	0.8%	0.0%
Afghanistan	Wama	2017	21.6%	38.5%	8.8%
Afghanistan	Waras	2000	0.1%	1.2%	0.0%
Afghanistan	Waras	2017	13.5%	25.7%	5.3%
Afghanistan	Warsaj	2000	0.1%	0.6%	0.0%
Afghanistan	Warsaj	2017	16.8%	33.9%	5.9%
Afghanistan	Washer	2000	0.2%	1.3%	0.0%
Afghanistan	Washer	2017	18.1%	33.0%	5.8%
Afghanistan	Waygal	2000	0.1%	0.6%	0.0%
Afghanistan	Waygal	2017	14.8%	42.1%	1.1%
Afghanistan	Wazakhwa	2000	0.3%	1.9%	0.0%
Afghanistan	Wazakhwa	2017	21.6%	44.9%	7.7%
Afghanistan	Wolmamay	2000	0.1%	1.3%	0.0%
Afghanistan	Wolmamay	2017	16.0%	33.5%	4.5%
Afghanistan	Yakawlang	2000	0.2%	1.2%	0.0%

Country	Second Administrative Unit	Year	Mean	Upper	Lower
Afghanistan	Yakawlang	2017	15.8%	40.4%	5.0%
Afghanistan	Yangi Qala	2000	0.0%	0.7%	0.0%
Afghanistan	Yangi Qala	2017	14.5%	24.8%	6.6%
Afghanistan	Zana Khan	2000	0.0%	0.2%	0.0%
Afghanistan	Zana Khan	2017	8.8%	39.4%	0.0%
Afghanistan	Zaranj	2000	0.2%	1.6%	0.0%
Afghanistan	Zaranj	2017	20.0%	59.4%	3.0%
Afghanistan	Zarghun Shahr	2000	1.0%	5.4%	0.0%
Afghanistan	Zarghun Shahr	2017	30.3%	43.8%	18.6%
Afghanistan	Zebak	2000	0.3%	3.3%	0.0%
Afghanistan	Zebak	2017	24.2%	60.2%	6.0%
Afghanistan	Ziluk	2000	0.1%	0.8%	0.0%
Afghanistan	Ziluk	2017	16.2%	55.0%	2.0%
Afghanistan	Zinda Jan	2000	0.1%	1.1%	0.0%
Afghanistan	Zinda Jan	2017	16.8%	36.2%	5.2%
Afghanistan	Zurmat	2000	0.0%	0.3%	0.0%
Afghanistan	Zurmat	2017	13.6%	28.1%	3.4%
Algeria	Abadla	2000	48.7%	53.8%	42.9%
Algeria	Abadla	2017	48.9%	53.9%	43.1%
Algeria	Abalissa	2000	48.7%	54.6%	42.6%
Algeria	Abalissa	2017	48.7%	54.5%	43.1%
Algeria	Abi Youcef	2000	77.5%	80.4%	74.1%
Algeria	Abi Youcef	2017	77.7%	80.6%	74.2%
Algeria	Abou El Hassen	2000	75.4%	80.3%	70.8%
Algeria	Abou El Hassen	2017	75.5%	80.4%	70.9%
Algeria	Achaacha	2000	70.4%	74.8%	65.8%
Algeria	Achaacha	2017	70.5%	74.8%	65.9%
Algeria	Adekar	2000	78.0%	81.2%	74.2%
Algeria	Adekar	2017	78.0%	81.2%	74.3%
Algeria	Adrar	2000	49.3%	53.5%	45.0%
Algeria	Adrar	2017	49.5%	53.7%	45.4%
Algeria	Afir	2000	78.1%	82.1%	74.2%
Algeria	Afir	2017	78.0%	82.0%	74.1%
Algeria	Aflou	2000	80.6%	83.1%	77.8%
Algeria	Aflou	2017	80.8%	83.3%	77.9%
Algeria	Aghbal	2000	76.9%	81.8%	71.4%
Algeria	Aghbal	2017	76.9%	81.8%	71.4%
Algeria	Aghbalou	2000	74.7%	78.3%	71.2%
Algeria	Aghbalou	2017	74.6%	78.3%	71.1%
Algeria	Aghlal	2000	71.2%	74.6%	67.9%
Algeria	Aghlal	2017	71.4%	74.9%	68.2%
Algeria	Aghni-Goughrane	2000	76.2%	79.3%	72.9%

Country	Second Administrative Unit	Year	Mean	Upper	Lower
Algeria	Aghni-Goughrane	2017	76.2%	79.4%	73.0%
Algeria	Aghrib	2000	79.9%	82.7%	76.8%
Algeria	Aghrib	2017	80.1%	82.9%	77.0%
Algeria	Ahl El Ksar	2000	76.6%	80.3%	73.0%
Algeria	Ahl El Ksar	2017	76.8%	80.4%	73.2%
Algeria	Ahmed Rachedi	2000	80.4%	82.2%	78.7%
Algeria	Ahmed Rachedi	2017	80.6%	82.4%	78.9%
Algeria	Ahmer El Ain	2000	75.0%	77.9%	72.0%
Algeria	Ahmer El Ain	2017	75.3%	78.2%	72.3%
Algeria	Ahnif	2000	74.9%	79.1%	70.3%
Algeria	Ahnif	2017	75.0%	79.2%	70.4%
Algeria	Ain Abessa	2000	64.9%	67.7%	61.7%
Algeria	Ain Abessa	2017	65.1%	68.0%	61.9%
Algeria	Ain Abid	2000	76.5%	79.0%	74.0%
Algeria	Ain Abid	2017	76.6%	79.1%	74.1%
Algeria	Ain Adden	2000	70.5%	73.8%	67.5%
Algeria	Ain Adden	2017	70.7%	74.0%	67.7%
Algeria	Ain Arnat	2000	60.5%	62.3%	58.3%
Algeria	Ain Arnat	2017	60.7%	62.6%	58.5%
Algeria	Ain Azel	2000	62.8%	65.6%	59.5%
Algeria	Ain Azel	2017	63.0%	65.7%	59.6%
Algeria	Ain Bebouche	2000	65.0%	68.4%	61.4%
Algeria	Ain Bebouche	2017	65.0%	68.5%	61.5%
Algeria	Ain Beida	2000	62.7%	66.8%	58.5%
Algeria	Ain Beida	2000	48.6%	51.7%	45.7%
Algeria	Ain Beida	2017	62.9%	66.9%	58.7%
Algeria	Ain Beida	2017	48.9%	51.9%	46.1%
Algeria	Ain Beida Harriche	2000	75.2%	78.2%	72.5%
Algeria	Ain Beida Harriche	2017	75.5%	78.5%	72.7%
Algeria	Ain Ben Beida	2000	80.7%	82.3%	78.9%
Algeria	Ain Ben Beida	2017	80.9%	82.5%	79.2%
Algeria	Ain Ben Khelil	2000	77.6%	82.3%	72.4%
Algeria	Ain Ben Khelil	2017	77.7%	82.3%	72.8%
Algeria	Ain Benian	2000	74.8%	77.2%	72.6%
Algeria	Ain Benian	2000	76.4%	79.0%	73.8%
Algeria	Ain Benian	2017	75.0%	77.4%	72.9%
Algeria	Ain Benian	2017	76.4%	79.0%	73.8%
Algeria	Ain Biya	2000	69.5%	72.1%	66.8%
Algeria	Ain Biya	2017	69.7%	72.3%	67.1%
Algeria	Ain Bouchekif	2000	78.6%	80.1%	77.0%
Algeria	Ain Bouchekif	2017	78.7%	80.1%	77.2%
Algeria	Ain Boucif	2000	79.1%	82.4%	75.2%
Algeria	Ain Boucif	2017	79.5%	82.8%	75.7%
Algeria	Ain Boudinar	2000	71.2%	73.7%	68.6%
Algeria	Ain Boudinar	2017	71.4%	73.9%	68.8%
Algeria	Ain Bouihi	2000	76.8%	79.7%	73.5%
Algeria	Ain Bouihi	2017	76.8%	79.8%	73.6%
Algeria	Ain Bouziane	2000	81.2%	83.1%	79.2%
Algeria	Ain Bouziane	2017	81.3%	83.3%	79.4%
Algeria	Ain Charchar	2000	80.4%	82.5%	77.8%
Algeria	Ain Charchar	2017	80.6%	82.7%	78.0%
Algeria	Ain Chouhada	2000	80.6%	83.8%	77.5%
Algeria	Ain Chouhada	2017	80.8%	83.9%	77.8%
Algeria	Ain Defla	2000	75.7%	78.5%	73.0%
Algeria	Ain Defla	2017	76.0%	78.8%	73.3%

Country	Second Administrative Unit	Year	Mean	Upper	Lower
Algeria	Ain Deheb	2000	79.2%	81.5%	76.4%
Algeria	Ain Deheb	2017	79.4%	81.7%	76.7%
Algeria	Ain Djasser	2000	66.3%	69.9%	62.4%
Algeria	Ain Djasser	2017	66.9%	70.4%	63.0%
Algeria	Ain El Arbaa	2000	70.4%	73.3%	66.9%
Algeria	Ain El Arbaa	2017	70.6%	73.5%	67.1%
Algeria	Ain El Assel	2000	79.3%	82.3%	76.1%
Algeria	Ain El Assel	2017	79.4%	82.4%	76.2%
Algeria	Ain El Berd	2000	71.7%	75.2%	68.4%
Algeria	Ain El Berd	2017	71.8%	75.4%	68.6%
Algeria	Ain El Berda	2000	80.4%	82.3%	78.3%
Algeria	Ain El Berda	2017	80.6%	82.5%	78.4%
Algeria	Ain El Diss	2000	67.3%	70.7%	64.1%
Algeria	Ain El Diss	2017	67.4%	70.8%	64.3%
Algeria	Ain El Fakroun	2000	64.0%	67.1%	60.5%
Algeria	Ain El Fakroun	2017	64.2%	67.3%	60.7%
Algeria	Ain El Hadid	2000	78.4%	80.8%	75.9%
Algeria	Ain El Hadid	2017	78.6%	81.0%	76.0%
Algeria	Ain El Hadjar	2000	76.9%	79.2%	74.3%
Algeria	Ain El Hadjar	2000	77.7%	79.6%	75.9%
Algeria	Ain El Hadjar	2017	77.9%	79.9%	76.2%
Algeria	Ain El Hadjar	2017	77.0%	79.3%	74.4%
Algeria	Ain El Hadjel	2000	81.0%	84.6%	77.3%
Algeria	Ain El Hadjel	2017	81.1%	84.7%	77.5%
Algeria	Ain El Ibel	2000	80.8%	83.4%	78.5%
Algeria	Ain El Ibel	2017	81.1%	83.6%	78.8%
Algeria	Ain El Kebira	2000	65.0%	67.1%	62.0%
Algeria	Ain El Kebira	2017	65.0%	67.1%	62.0%
Algeria	Ain El Kercha	2000	62.1%	65.6%	58.4%
Algeria	Ain El Kercha	2017	62.3%	65.8%	58.6%
Algeria	Ain El Melh	2000	78.1%	81.7%	74.1%
Algeria	Ain El Melh	2017	78.4%	81.9%	74.4%
Algeria	Ain El Orak	2000	78.5%	81.8%	74.7%
Algeria	Ain El Orak	2017	78.7%	81.7%	75.1%
Algeria	Ain Errich	2000	77.2%	80.9%	72.6%
Algeria	Ain Errich	2017	77.3%	80.8%	72.7%
Algeria	Ain Fares	2000	73.1%	75.7%	70.3%
Algeria	Ain Fares	2000	73.9%	77.9%	68.9%
Algeria	Ain Fares	2017	73.2%	75.8%	70.5%
Algeria	Ain Fares	2017	74.0%	77.9%	69.1%
Algeria	Ain Fekan	2000	72.1%	75.1%	69.0%
Algeria	Ain Fekan	2017	72.2%	75.2%	69.1%
Algeria	Ain Fekka	2000	81.6%	85.4%	78.0%
Algeria	Ain Fekka	2017	81.7%	85.5%	78.2%
Algeria	Ain Ferah	2000	75.4%	78.2%	72.5%
Algeria	Ain Ferah	2017	75.5%	78.4%	72.7%
Algeria	Ain Fettah	2000	68.5%	71.7%	65.1%
Algeria	Ain Fettah	2017	69.0%	72.1%	65.6%
Algeria	Ain Fezza	2000	70.2%	72.8%	66.9%
Algeria	Ain Fezza	2017	70.5%	73.2%	67.3%
Algeria	Ain Frass	2000	71.2%	74.6%	67.7%
Algeria	Ain Frass	2017	71.4%	74.8%	67.9%
Algeria	Ain Ghoraba	2000	70.5%	74.4%	66.2%
Algeria	Ain Ghoraba	2017	70.8%	74.6%	66.6%
Algeria	Ain Kada	2000	70.8%	74.0%	67.4%
Algeria	Ain Kada	2017	71.0%	74.2%	67.7%
Algeria	Ain Kebira	2000	68.8%	72.1%	65.4%
Algeria	Ain Kebira	2017	69.0%	72.3%	65.7%
Algeria	Ain Kechra	2000	82.0%	84.1%	79.5%
Algeria	Ain Kechra	2017	82.0%	84.1%	79.6%

Country	Second Administrative Unit	Year	Mean	Upper	Lower
Algeria	Ain Kerma	2000	80.1%	83.1%	76.5%
Algeria	Ain Kerma	2000	70.1%	74.3%	65.3%
Algeria	Ain Kerma	2017	79.9%	83.0%	76.4%
Algeria	Ain Kerma	2017	70.4%	74.6%	65.7%
Algeria	Ain Kermes	2000	78.6%	81.3%	76.0%
Algeria	Ain Kermes	2017	78.7%	81.5%	76.2%
Algeria	Ain Khadra	2000	77.5%	80.3%	74.8%
Algeria	Ain Khadra	2017	77.6%	80.5%	75.0%
Algeria	Ain Kihel	2000	70.9%	74.1%	67.9%
Algeria	Ain Kihel	2017	71.1%	74.2%	68.1%
Algeria	Ain Lahdjar	2000	61.0%	64.5%	57.6%
Algeria	Ain Lahdjar	2017	61.2%	64.7%	57.9%
Algeria	Ain Laloui	2000	77.2%	79.6%	74.2%
Algeria	Ain Laloui	2017	77.3%	79.8%	74.4%
Algeria	Ain Larbi	2000	78.2%	81.5%	75.2%
Algeria	Ain Larbi	2017	78.3%	81.6%	75.3%
Algeria	Ain Lechiakh	2000	76.7%	79.9%	73.5%
Algeria	Ain Lechiakh	2017	76.8%	80.0%	73.6%
Algeria	Ain M'Lila	2000	65.9%	68.8%	63.1%
Algeria	Ain M'Lila	2017	65.8%	68.8%	62.9%
Algeria	Ain Maabed	2000	81.3%	83.8%	79.2%
Algeria	Ain Maabed	2017	81.6%	84.1%	79.5%
Algeria	Ain Madhi	2000	80.1%	84.0%	76.3%
Algeria	Ain Madhi	2017	79.9%	83.6%	76.3%
Algeria	Ain Makhlouf	2000	77.1%	80.3%	74.2%
Algeria	Ain Makhlouf	2017	77.4%	80.5%	74.5%
Algeria	Ain Mellouk	2000	79.1%	80.8%	76.7%
Algeria	Ain Mellouk	2017	79.3%	81.0%	76.9%
Algeria	Ain Merrane	2000	75.4%	78.1%	72.8%
Algeria	Ain Merrane	2017	75.9%	78.5%	73.2%
Algeria	Ain Naga	2000	48.5%	53.4%	43.9%
Algeria	Ain Naga	2017	48.7%	53.5%	44.1%
Algeria	Ain Nehala	2000	70.5%	74.5%	66.9%
Algeria	Ain Nehala	2017	70.8%	74.7%	67.2%
Algeria	Ain Nouissy	2000	70.5%	73.0%	68.2%
Algeria	Ain Nouissy	2017	70.8%	73.3%	68.6%
Algeria	Ain Ouksir	2000	78.5%	82.1%	74.2%
Algeria	Ain Ouksir	2017	78.7%	82.3%	74.5%
Algeria	Ain Oulmane	2000	63.5%	66.3%	60.0%
Algeria	Ain Oulmane	2017	63.8%	66.5%	60.3%
Algeria	Ain Oussera	2000	81.6%	83.3%	79.8%
Algeria	Ain Oussera	2017	81.8%	83.6%	80.0%
Algeria	Ain Rahma	2000	71.5%	74.3%	68.2%
Algeria	Ain Rahma	2017	71.6%	74.4%	68.4%
Algeria	Ain Rekada	2000	78.4%	81.0%	75.6%
Algeria	Ain Rekada	2017	78.6%	81.2%	75.8%
Algeria	Ain Romana	2000	76.3%	78.9%	73.3%
Algeria	Ain Romana	2017	76.3%	78.9%	73.4%
Algeria	Ain Roua	2000	64.5%	67.9%	61.6%
Algeria	Ain Roua	2017	64.6%	67.9%	61.8%
Algeria	Ain Safra	2000	79.3%	81.7%	76.4%
Algeria	Ain Safra	2017	79.5%	81.8%	76.7%
Algeria	Ain Sandel	2000	78.8%	82.0%	75.8%
Algeria	Ain Sandel	2017	79.0%	82.1%	76.0%
Algeria	Ain Sekhouna	2000	78.5%	82.3%	73.8%
Algeria	Ain Sekhouna	2017	78.6%	82.5%	73.7%
Algeria	Ain Semara	2000	78.3%	80.3%	76.3%
Algeria	Ain Semara	2017	78.0%	80.1%	75.9%
Algeria	Ain Sidi Ali	2000	79.8%	83.5%	75.6%
Algeria	Ain Sidi Ali	2017	79.9%	83.6%	75.8%
Algeria	Ain Sidi Cherif	2000	70.1%	72.4%	67.7%

Country	Second Administrative Unit	Year	Mean	Upper	Lower
Algeria	Ain Sidi Cherif	2017	70.3%	72.7%	68.0%
Algeria	Ain Soltane	2000	77.3%	80.3%	74.5%
Algeria	Ain Soltane	2000	76.8%	80.4%	73.4%
Algeria	Ain Soltane	2000	76.0%	78.9%	72.6%
Algeria	Ain Soltane	2017	76.3%	79.1%	72.8%
Algeria	Ain Soltane	2017	76.8%	80.5%	73.4%
Algeria	Ain Soltane	2017	77.6%	80.5%	74.9%
Algeria	Ain Taghrout	2000	61.0%	63.6%	58.1%
Algeria	Ain Taghrout	2017	61.3%	63.9%	58.3%
Algeria	Ain Tagourait	2000	75.5%	78.5%	72.4%
Algeria	Ain Tagourait	2017	75.7%	78.7%	72.6%
Algeria	Ain Tallout	2000	70.2%	73.7%	66.8%
Algeria	Ain Tallout	2017	70.3%	73.9%	67.0%
Algeria	Ain Tarek	2000	73.7%	76.7%	70.4%
Algeria	Ain Tarek	2017	73.9%	76.9%	70.6%
Algeria	Ain Tedles	2000	71.1%	73.5%	68.8%
Algeria	Ain Tedles	2017	71.5%	73.9%	69.2%
Algeria	Ain Temouchent	2000	70.7%	73.3%	67.8%
Algeria	Ain Temouchent	2017	70.9%	73.4%	67.9%
Algeria	Ain Tesra	2000	63.3%	66.0%	60.6%
Algeria	Ain Tesra	2017	63.6%	66.3%	60.8%
Algeria	Ain Thrid	2000	70.7%	73.2%	68.1%
Algeria	Ain Thrid	2017	70.9%	73.4%	68.3%
Algeria	Ain Tindamine	2000	70.9%	75.0%	66.8%
Algeria	Ain Tindamine	2017	71.0%	75.1%	66.9%
Algeria	Ain Tine	2000	81.7%	84.0%	79.6%
Algeria	Ain Tine	2017	81.8%	84.1%	79.7%
Algeria	Ain Tolba	2000	69.8%	72.9%	66.5%
Algeria	Ain Tolba	2017	69.8%	72.9%	66.5%
Algeria	Ain Tork	2000	77.4%	79.7%	75.1%
Algeria	Ain Tork	2017	77.7%	79.9%	75.3%
Algeria	Ain Touila	2000	62.1%	66.2%	57.5%
Algeria	Ain Touila	2017	62.3%	66.4%	57.8%
Algeria	Ain Touta	2000	59.2%	62.2%	56.3%
Algeria	Ain Touta	2017	59.4%	62.4%	56.5%
Algeria	Ain Turk	2000	69.2%	72.3%	65.6%
Algeria	Ain Turk	2000	76.6%	79.2%	73.5%
Algeria	Ain Turk	2017	69.4%	72.7%	65.7%
Algeria	Ain Turk	2017	76.7%	79.4%	73.8%
Algeria	Ain Yagout	2000	64.2%	66.8%	61.5%
Algeria	Ain Yagout	2017	64.4%	66.9%	61.8%
Algeria	Ain Youcef	2000	68.2%	71.2%	64.7%
Algeria	Ain Youcef	2017	68.4%	71.4%	64.9%
Algeria	Ain Zaatout	2000	56.8%	60.1%	53.5%
Algeria	Ain Zaatout	2017	57.4%	60.6%	53.9%
Algeria	Ain Zana	2000	80.9%	84.1%	77.1%
Algeria	Ain Zana	2017	81.1%	84.2%	77.2%
Algeria	Ain Zarit	2000	77.8%	80.2%	75.1%
Algeria	Ain Zarit	2017	77.9%	80.3%	75.3%
Algeria	Ain Zerga	2000	63.9%	68.9%	58.3%
Algeria	Ain Zerga	2017	64.0%	69.2%	58.4%
Algeria	Ain Zitoun	2000	61.1%	64.7%	57.7%
Algeria	Ain Zitoun	2017	61.3%	64.8%	57.9%
Algeria	Ain Zouit	2000	80.6%	83.2%	77.9%
Algeria	Ain Zouit	2017	80.9%	83.4%	78.2%
Algeria	Ain-Bessem	2000	77.7%	80.2%	74.6%
Algeria	Ain-Bessem	2017	77.9%	80.4%	74.8%

Country	Second Administrative Unit	Year	Mean	Upper	Lower
Algeria	Ain-El-Hammam	2000	77.4%	80.2%	74.0%
Algeria	Ain-El-Hammam	2017	77.6%	80.4%	74.2%
Algeria	Ain-Legradj	2000	67.7%	70.7%	64.8%
Algeria	Ain-Legradj	2017	67.8%	70.8%	65.0%
Algeria	Ain-Sebt	2000	74.6%	77.8%	71.4%
Algeria	Ain-Sebt	2017	74.9%	78.1%	71.7%
Algeria	Ain-Zaouia	2000	76.0%	78.7%	72.9%
Algeria	Ain-Zaouia	2017	76.1%	78.9%	73.1%
Algeria	Aissaouia	2000	77.3%	80.7%	73.8%
Algeria	Aissaouia	2017	77.5%	80.8%	74.0%
Algeria	Ait Aggouacha	2000	78.6%	81.0%	76.1%
Algeria	Ait Aggouacha	2017	78.7%	81.0%	76.2%
Algeria	Ait Aissa Mimoun	2000	77.0%	79.4%	74.8%
Algeria	Ait Aissa Mimoun	2017	77.3%	79.7%	75.1%
Algeria	Ait Bouadou	2000	78.3%	81.1%	75.3%
Algeria	Ait Bouadou	2017	78.5%	81.3%	75.4%
Algeria	Ait Boumehdi	2000	77.8%	80.8%	74.5%
Algeria	Ait Boumehdi	2017	77.9%	80.9%	74.6%
Algeria	Ait Khelili	2000	77.9%	80.2%	75.4%
Algeria	Ait Khelili	2017	78.2%	80.5%	75.6%
Algeria	Ait Laaziz	2000	77.5%	80.3%	74.4%
Algeria	Ait Laaziz	2017	77.6%	80.4%	74.6%
Algeria	Ait Naoual Mezada	2000	70.8%	74.1%	67.6%
Algeria	Ait Naoual Mezada	2017	70.8%	74.1%	67.6%
Algeria	Ait Oumalou	2000	78.7%	81.0%	76.3%
Algeria	Ait Oumalou	2017	78.5%	80.8%	76.2%
Algeria	Ait R'Zine	2000	71.9%	75.5%	68.1%
Algeria	Ait R'Zine	2017	72.1%	75.7%	68.4%
Algeria	Ait Toudert	2000	76.7%	79.9%	73.7%
Algeria	Ait Toudert	2017	76.9%	80.1%	73.9%
Algeria	Ait Yahia Moussa	2000	76.6%	79.4%	73.9%
Algeria	Ait Yahia Moussa	2017	76.6%	79.3%	74.0%
Algeria	Ait-Chaffaa	2000	78.1%	81.9%	74.1%
Algeria	Ait-Chaffaa	2017	78.3%	82.3%	74.4%
Algeria	Ait-Mahmoud	2000	76.9%	79.2%	74.1%
Algeria	Ait-Mahmoud	2017	77.1%	79.4%	74.4%
Algeria	Ait-Smail	2000	72.1%	75.5%	68.6%
Algeria	Ait-Smail	2017	72.4%	75.8%	68.8%
Algeria	Ait-Tizi	2000	70.9%	74.2%	67.8%
Algeria	Ait-Tizi	2017	71.0%	74.3%	67.9%
Algeria	Ait-Yahia	2000	77.3%	79.9%	74.1%
Algeria	Ait-Yahia	2017	77.5%	80.1%	74.3%
Algeria	Akabli	2000	48.8%	57.3%	40.8%
Algeria	Akabli	2017	48.7%	56.9%	41.1%
Algeria	Akbil	2000	77.8%	80.9%	74.4%
Algeria	Akbil	2017	78.0%	81.0%	74.6%
Algeria	Akbou	2000	73.0%	75.8%	70.1%
Algeria	Akbou	2017	73.0%	75.8%	70.1%
Algeria	Akerrou	2000	78.7%	82.3%	74.9%
Algeria	Akerrou	2017	79.0%	82.5%	75.2%

Country	Second Administrative Unit	Year	Mean	Upper	Lower
Algeria	Akfadou	2000	77.1%	79.8%	74.4%
Algeria	Akfadou	2017	77.2%	79.8%	74.4%
Algeria	Alaimia	2000	69.9%	73.0%	65.9%
Algeria	Alaimia	2017	70.1%	73.2%	66.2%
Algeria	Amalou	2000	72.2%	75.0%	69.2%
Algeria	Amalou	2017	72.4%	75.1%	69.5%
Algeria	Amernas	2000	71.0%	73.4%	68.8%
Algeria	Amernas	2017	71.2%	73.7%	69.0%
Algeria	Amieur	2000	69.5%	72.6%	66.3%
Algeria	Amieur	2017	69.8%	72.9%	66.7%
Algeria	Amirat Arres	2000	82.5%	84.5%	80.5%
Algeria	Amirat Arres	2017	82.7%	84.6%	80.7%
Algeria	Amizour	2000	73.5%	76.6%	70.3%
Algeria	Amizour	2017	73.8%	76.9%	70.5%
Algeria	Ammal	2000	76.8%	79.3%	74.4%
Algeria	Ammal	2017	76.8%	79.3%	74.5%
Algeria	Ammari	2000	79.5%	81.4%	77.2%
Algeria	Ammari	2017	79.5%	81.5%	77.4%
Algeria	Ammi Moussa	2000	72.8%	75.7%	69.4%
Algeria	Ammi Moussa	2017	73.0%	75.9%	69.5%
Algeria	Amoucha	2000	65.6%	68.2%	62.6%
Algeria	Amoucha	2017	65.9%	68.5%	63.0%
Algeria	Amourah	2000	76.6%	80.9%	70.7%
Algeria	Amourah	2017	76.0%	80.4%	70.0%
Algeria	Annaba	2000	80.4%	82.6%	78.0%
Algeria	Annaba	2017	80.6%	82.7%	78.1%
Algeria	Aokas	2000	73.4%	76.9%	69.6%
Algeria	Aokas	2017	73.7%	77.3%	69.9%
Algeria	Aomar	2000	76.5%	79.1%	73.5%
Algeria	Aomar	2017	76.6%	79.3%	73.7%
Algeria	Aoubellil	2000	70.4%	73.9%	66.7%
Algeria	Aoubellil	2017	70.7%	74.2%	66.9%
Algeria	Aouf	2000	75.7%	79.1%	72.5%
Algeria	Aouf	2017	75.9%	79.3%	72.7%
Algeria	Aougrout	2000	49.4%	55.4%	42.6%
Algeria	Aougrout	2017	49.6%	55.6%	42.9%
Algeria	Aoulef	2000	48.2%	53.3%	44.2%
Algeria	Aoulef	2017	48.3%	53.8%	44.3%
Algeria	Arbaouat	2000	79.1%	82.4%	75.4%
Algeria	Arbaouat	2017	79.2%	82.6%	75.6%
Algeria	Arib	2000	76.5%	79.7%	73.4%
Algeria	Arib	2017	76.6%	79.7%	73.5%
Algeria	Arris	2000	62.0%	66.0%	58.4%
Algeria	Arris	2017	62.1%	66.0%	58.6%
Algeria	Arzew	2000	69.0%	71.8%	66.2%
Algeria	Arzew	2017	69.3%	72.0%	66.4%
Algeria	Asfour	2000	80.3%	82.9%	77.4%
Algeria	Asfour	2017	80.4%	83.0%	77.5%
Algeria	Assela	2000	80.0%	83.3%	76.3%
Algeria	Assela	2017	80.3%	83.5%	76.7%
Algeria	Assi Youcef	2000	78.0%	81.0%	75.0%
Algeria	Assi Youcef	2017	78.1%	81.1%	75.1%
Algeria	Ath Mansour Taourirt	2000	73.5%	77.9%	69.1%
Algeria	Ath Mansour Taourirt	2017	73.7%	78.0%	69.3%
Algeria	Azails	2000	69.4%	73.6%	64.7%
Algeria	Azails	2017	69.2%	73.2%	64.5%
Algeria	Azazga	2000	78.0%	80.2%	75.5%
Algeria	Azazga	2017	78.2%	80.4%	75.6%
Algeria	Azil Abdelkader (Metkouak)	2000	73.5%	76.5%	70.3%

Country	Second Administrative Unit	Year	Mean	Upper	Lower
Algeria	Azil Abdelkader (Metkouak)	2017	73.9%	76.9%	70.9%
Algeria	Azzaba	2000	80.7%	82.9%	78.4%
Algeria	Azzaba	2017	80.9%	83.1%	78.7%
Algeria	Azzefoun	2000	78.0%	81.7%	74.0%
Algeria	Azzefoun	2017	78.0%	81.8%	73.8%
Algeria	Azziz	2000	77.5%	80.6%	73.5%
Algeria	Azziz	2017	77.7%	80.9%	73.7%
Algeria	Azzizia	2000	77.3%	80.5%	73.6%
Algeria	Azzizia	2017	77.4%	80.7%	73.8%
Algeria	Baata	2000	77.5%	80.8%	73.6%
Algeria	Baata	2017	78.0%	81.4%	74.1%
Algeria	Bab El Assa	2000	68.4%	72.9%	63.2%
Algeria	Bab El Assa	2017	68.5%	73.0%	63.2%
Algeria	Babar	2000	60.0%	63.5%	56.2%
Algeria	Babar	2017	59.5%	63.2%	55.5%
Algeria	Babor	2000	69.1%	72.2%	65.6%
Algeria	Babor	2017	68.9%	72.0%	65.4%
Algeria	Badredine El Mokrani	2000	70.7%	74.4%	67.1%
Algeria	Badredine El Mokrani	2017	70.9%	74.6%	67.3%
Algeria	Baghai	2000	61.9%	64.9%	58.8%
Algeria	Baghai	2017	62.2%	65.2%	59.1%
Algeria	Baghlia	2000	76.6%	79.7%	73.1%
Algeria	Baghlia	2017	76.7%	79.9%	73.2%
Algeria	Baladiet Amor	2000	48.7%	53.1%	44.4%
Algeria	Baladiet Amor	2017	49.0%	53.5%	44.7%
Algeria	Baraki	2000	75.3%	76.8%	73.8%
Algeria	Baraki	2017	75.5%	77.0%	74.0%
Algeria	Barbouche	2000	76.7%	80.3%	73.4%
Algeria	Barbouche	2017	76.8%	80.4%	73.6%
Algeria	Barika	2000	62.6%	66.1%	59.3%
Algeria	Barika	2017	62.8%	66.3%	59.4%
Algeria	Bathia	2000	78.0%	80.8%	75.0%
Algeria	Bathia	2017	78.2%	81.0%	75.1%
Algeria	Batna	2000	61.6%	64.4%	58.8%
Algeria	Batna	2017	61.9%	64.6%	59.0%
Algeria	Bayadha	2000	48.3%	51.8%	45.5%
Algeria	Bayadha	2017	48.6%	52.1%	45.8%
Algeria	Bazer-Sakra	2000	62.4%	64.8%	60.0%
Algeria	Bazer-Sakra	2017	62.7%	65.1%	60.3%
Algeria	Bechar	2000	48.3%	52.0%	44.7%
Algeria	Bechar	2017	48.5%	52.3%	44.9%
Algeria	Bechloul	2000	75.9%	79.6%	72.5%
Algeria	Bechloul	2017	75.8%	79.6%	72.4%
Algeria	Bedjene	2000	62.2%	65.9%	57.9%
Algeria	Bedjene	2017	62.5%	66.3%	58.2%
Algeria	Beidha Bordj	2000	61.9%	65.3%	58.2%
Algeria	Beidha Bordj	2017	62.1%	65.5%	58.4%
Algeria	Bejaia	2000	75.6%	78.8%	71.6%
Algeria	Bejaia	2017	75.7%	79.0%	71.8%
Algeria	Bekkaria	2000	64.1%	68.3%	59.8%
Algeria	Bekkaria	2017	64.5%	68.6%	60.3%
Algeria	Bekkouche Lakhdar	2000	80.4%	82.4%	77.7%
Algeria	Bekkouche Lakhdar	2017	80.5%	82.5%	77.9%
Algeria	Belaas	2000	77.7%	80.6%	74.7%

Country	Second Administrative Unit	Year	Mean	Upper	Lower
Algeria	Belaas	2017	77.9%	80.7%	74.8%
Algeria	Belaassel Bouzagza	2000	70.1%	73.0%	67.5%
Algeria	Belaassel Bouzagza	2017	70.4%	73.2%	67.8%
Algeria	Belaiba	2000	72.9%	75.5%	70.6%
Algeria	Belaiba	2017	73.2%	75.7%	70.8%
Algeria	Belala	2000	62.6%	67.0%	58.2%
Algeria	Belala	2017	62.7%	67.2%	58.4%
Algeria	Belarbi	2000	72.0%	75.9%	68.6%
Algeria	Belarbi	2017	72.3%	76.1%	68.8%
Algeria	Belimour	2000	67.6%	70.6%	64.4%
Algeria	Belimour	2017	67.7%	70.7%	64.5%
Algeria	Belkheir	2000	78.3%	80.8%	76.0%
Algeria	Belkheir	2017	78.4%	80.9%	76.2%
Algeria	Bellaa	2000	70.2%	73.1%	67.6%
Algeria	Bellaa	2017	70.1%	72.9%	67.5%
Algeria	Ben Allal	2000	76.7%	79.5%	73.3%
Algeria	Ben Allal	2017	77.0%	79.8%	73.5%
Algeria	Ben Azzouz	2000	80.4%	82.7%	78.0%
Algeria	Ben Azzouz	2017	80.5%	82.8%	78.1%
Algeria	Ben Badis	2000	78.3%	80.7%	75.8%
Algeria	Ben Badis	2000	70.3%	73.9%	66.7%
Algeria	Ben Badis	2017	78.4%	80.7%	75.8%
Algeria	Ben Badis	2017	70.6%	74.1%	67.0%
Algeria	Ben Chicao	2000	76.5%	79.9%	72.9%
Algeria	Ben Chicao	2017	76.7%	80.0%	73.1%
Algeria	Ben Choud	2000	76.9%	80.3%	73.0%
Algeria	Ben Choud	2017	77.0%	80.5%	73.2%
Algeria	Ben Daoud	2000	70.7%	73.2%	68.0%
Algeria	Ben Daoud	2017	71.1%	73.5%	68.4%
Algeria	Ben Djerrah	2000	79.5%	81.5%	77.4%
Algeria	Ben Djerrah	2017	79.7%	81.7%	77.6%
Algeria	Ben Freha	2000	70.1%	72.6%	67.6%
Algeria	Ben Freha	2017	70.3%	72.8%	67.9%
Algeria	Ben Guecha	2000	52.5%	58.9%	46.3%
Algeria	Ben Guecha	2017	52.4%	58.7%	46.3%
Algeria	Ben M'Hidi	2000	80.2%	82.4%	77.5%
Algeria	Ben M'Hidi	2017	80.4%	82.6%	77.7%
Algeria	Ben Srour	2000	79.3%	82.3%	76.2%
Algeria	Ben Srour	2017	79.4%	82.4%	76.2%
Algeria	Benabdelmalek Ramdane	2000	71.2%	74.6%	67.7%
Algeria	Benabdelmalek Ramdane	2017	71.5%	74.8%	67.9%
Algeria	Benaceur	2000	48.2%	53.1%	43.3%
Algeria	Benaceur	2017	48.9%	54.5%	43.2%
Algeria	Benaicha Chelia	2000	70.7%	74.2%	66.9%
Algeria	Benaicha Chelia	2017	70.9%	74.4%	67.2%
Algeria	Benairia	2000	77.3%	81.2%	73.8%
Algeria	Benairia	2017	77.5%	81.3%	74.0%
Algeria	Bendaoud	2000	77.7%	81.5%	74.1%
Algeria	Bendaoud	2017	77.8%	81.6%	74.3%
Algeria	Benhar	2000	80.9%	83.0%	78.7%
Algeria	Benhar	2017	81.1%	83.3%	78.7%
Algeria	Beni Abbes	2000	49.6%	55.8%	42.3%
Algeria	Beni Abbes	2017	49.8%	56.0%	42.7%
Algeria	Beni Aissi	2000	76.4%	78.6%	74.3%
Algeria	Beni Aissi	2017	76.6%	78.8%	74.5%
Algeria	Beni Amrane	2000	76.1%	78.6%	74.0%

Country	Second Administrative Unit	Year	Mean	Upper	Lower
Algeria	Beni Amrane	2017	76.2%	78.8%	74.1%
Algeria	Beni Bahdel	2000	69.1%	73.3%	64.5%
Algeria	Beni Bahdel	2017	69.1%	73.2%	64.4%
Algeria	Beni Bechir	2000	80.8%	82.8%	78.9%
Algeria	Beni Bechir	2017	81.0%	82.9%	79.1%
Algeria	Beni Bouattab	2000	76.5%	79.4%	73.6%
Algeria	Beni Bouattab	2017	76.7%	79.6%	73.8%
Algeria	Beni Boussaid	2000	69.5%	74.0%	65.1%
Algeria	Beni Boussaid	2017	69.7%	74.2%	65.2%
Algeria	Beni Chaib	2000	78.0%	80.7%	75.4%
Algeria	Beni Chaib	2017	78.1%	80.8%	75.5%
Algeria	Beni Chebana	2000	69.2%	72.5%	65.9%
Algeria	Beni Chebana	2017	69.3%	72.6%	66.1%
Algeria	Beni Dejllil	2000	74.2%	77.4%	70.6%
Algeria	Beni Dejllil	2017	74.4%	77.6%	70.9%
Algeria	Beni Dergoun	2000	71.1%	74.2%	68.3%
Algeria	Beni Dergoun	2017	71.2%	74.4%	68.4%
Algeria	Beni Fouda	2000	65.3%	67.6%	62.4%
Algeria	Beni Fouda	2017	65.7%	67.9%	62.8%
Algeria	Beni Foudala El Hakania	2000	60.5%	63.4%	57.7%
Algeria	Beni Foudala El Hakania	2017	60.1%	62.8%	57.3%
Algeria	Beni Hamidane	2000	81.4%	83.7%	79.2%
Algeria	Beni Hamidane	2017	81.5%	83.8%	79.4%
Algeria	Beni Haoua	2000	75.8%	80.9%	70.3%
Algeria	Beni Haoua	2017	75.8%	80.9%	70.3%
Algeria	Beni Ikhlef	2000	49.7%	56.6%	44.0%
Algeria	Beni Ikhlef	2017	49.7%	56.1%	44.0%
Algeria	Beni Ilmane	2000	78.0%	81.6%	74.9%
Algeria	Beni Ilmane	2017	78.2%	81.7%	75.1%
Algeria	Beni K'Sila	2000	77.1%	81.6%	72.2%
Algeria	Beni K'Sila	2017	77.4%	81.9%	72.3%
Algeria	Beni Khellad	2000	68.0%	72.0%	63.4%
Algeria	Beni Khellad	2017	68.4%	72.3%	63.9%
Algeria	Beni Lahcene	2000	78.3%	80.8%	75.7%
Algeria	Beni Lahcene	2017	78.4%	80.9%	75.7%
Algeria	Beni Merad	2000	75.4%	77.5%	73.0%
Algeria	Beni Merad	2017	75.5%	77.6%	73.2%
Algeria	Beni Mester	2000	69.5%	71.9%	66.9%
Algeria	Beni Mester	2017	69.8%	72.2%	67.3%
Algeria	Beni Mezline	2000	79.0%	81.3%	76.3%
Algeria	Beni Mezline	2017	79.2%	81.5%	76.6%
Algeria	Beni Mileuk	2000	77.1%	81.5%	72.9%
Algeria	Beni Mileuk	2017	77.2%	81.5%	73.0%
Algeria	Beni Ouarsous	2000	68.1%	71.9%	64.4%
Algeria	Beni Ouarsous	2017	68.4%	72.1%	64.6%
Algeria	Beni Oulbane	2000	81.5%	83.7%	79.2%
Algeria	Beni Oulbane	2017	81.5%	83.7%	79.2%
Algeria	Beni Ounif	2000	54.3%	59.9%	48.5%
Algeria	Beni Ounif	2017	55.2%	60.5%	49.7%
Algeria	Beni Oussine	2000	62.9%	66.2%	59.9%
Algeria	Beni Oussine	2017	63.3%	66.6%	60.3%
Algeria	Beni Rached	2000	77.8%	80.5%	75.1%
Algeria	Beni Rached	2017	78.1%	80.8%	75.4%
Algeria	Beni Saf	2000	69.2%	72.9%	64.6%

Country	Second Administrative Unit	Year	Mean	Upper	Lower
Algeria	Beni Saf	2017	69.4%	73.2%	64.9%
Algeria	Beni Slimane	2000	76.5%	79.9%	73.0%
Algeria	Beni Slimane	2017	76.8%	80.1%	73.2%
Algeria	Beni Smiel	2000	69.9%	73.7%	65.7%
Algeria	Beni Smiel	2017	69.8%	73.8%	65.5%
Algeria	Beni Snous	2000	70.0%	74.5%	65.6%
Algeria	Beni Snous	2017	69.9%	74.4%	65.4%
Algeria	Beni Yenni	2000	76.6%	79.3%	73.9%
Algeria	Beni Yenni	2017	76.8%	79.5%	74.1%
Algeria	Beni Zentis	2000	71.9%	75.2%	68.9%
Algeria	Beni Zentis	2017	72.0%	75.2%	69.1%
Algeria	Beni Zid	2000	81.3%	83.6%	78.6%
Algeria	Beni Zid	2017	81.3%	83.6%	78.5%
Algeria	Beni Zmenzer	2000	77.1%	79.3%	74.7%
Algeria	Beni Zmenzer	2017	77.2%	79.4%	74.8%
Algeria	Beni-Aziz	2000	72.0%	74.9%	68.8%
Algeria	Beni-Aziz	2017	72.1%	75.0%	69.0%
Algeria	Beni-Douala	2000	77.3%	79.6%	74.7%
Algeria	Beni-Douala	2017	77.5%	79.8%	75.0%
Algeria	Beni-Mellikeche	2000	75.3%	78.6%	71.9%
Algeria	Beni-Mellikeche	2017	75.1%	78.6%	71.7%
Algeria	Beni-Mouhli	2000	70.9%	74.1%	67.6%
Algeria	Beni-Mouhli	2017	71.2%	74.4%	67.9%
Algeria	Beni-Ouartilane	2000	68.3%	71.8%	64.7%
Algeria	Beni-Ouartilane	2017	68.5%	72.0%	64.9%
Algeria	Beni-Tamou	2000	75.4%	77.3%	73.3%
Algeria	Beni-Tamou	2017	75.6%	77.5%	73.4%
Algeria	Beni-Zikki	2000	78.2%	80.6%	75.8%
Algeria	Beni-Zikki	2017	78.3%	80.7%	76.0%
Algeria	Benian	2000	75.1%	78.4%	72.3%
Algeria	Benian	2017	75.3%	78.6%	72.6%
Algeria	Benimaouche	2000	71.6%	74.8%	68.2%
Algeria	Benimaouche	2017	71.9%	75.2%	68.5%
Algeria	Benkhelil	2000	74.8%	76.5%	72.8%
Algeria	Benkhelil	2017	75.1%	76.8%	73.1%
Algeria	Bensekrane	2000	69.9%	73.5%	66.6%
Algeria	Bensekrane	2017	70.0%	73.6%	66.7%
Algeria	Benyacoub	2000	80.9%	83.8%	77.7%
Algeria	Benyacoub	2017	80.9%	83.8%	77.7%
Algeria	Benyahia Abderrahmane	2000	75.2%	77.7%	72.6%
Algeria	Benyahia Abderrahmane	2017	75.4%	77.9%	72.9%
Algeria	Benzouh	2000	81.9%	84.8%	78.7%
Algeria	Benzouh	2017	82.0%	84.9%	78.9%
Algeria	Berbacha	2000	72.3%	75.4%	69.2%
Algeria	Berbacha	2017	72.3%	75.5%	69.3%
Algeria	Berhoum	2000	78.4%	81.0%	76.0%
Algeria	Berhoum	2017	78.7%	81.2%	76.2%
Algeria	Berrahal	2000	80.4%	82.8%	78.0%
Algeria	Berrahal	2017	80.6%	83.0%	78.2%
Algeria	Berriane	2000	61.4%	65.0%	58.0%
Algeria	Berriane	2017	59.5%	63.5%	55.9%
Algeria	Berriche	2000	64.2%	67.8%	60.3%
Algeria	Berriche	2017	64.3%	67.8%	60.5%
Algeria	Berrihane	2000	79.5%	82.6%	76.7%
Algeria	Berrihane	2017	79.6%	82.8%	76.7%
Algeria	Berrouaghia	2000	75.9%	79.6%	72.1%

Country	Second Administrative Unit	Year	Mean	Upper	Lower
Algeria	Berrouaghia	2017	76.0%	79.7%	72.2%
Algeria	Besbes	2000	80.4%	82.3%	78.3%
Algeria	Besbes	2000	55.6%	59.8%	51.9%
Algeria	Besbes	2017	80.6%	82.6%	78.4%
Algeria	Besbes	2017	55.3%	59.5%	51.7%
Algeria	Bethioua	2000	69.1%	71.8%	66.0%
Algeria	Bethioua	2017	69.4%	72.0%	66.2%
Algeria	Bhir El Chergui	2000	64.6%	69.1%	59.9%
Algeria	Bhir El Chergui	2017	64.7%	69.2%	59.9%
Algeria	Bin El Ouiden	2000	80.8%	83.2%	78.3%
Algeria	Bin El Ouiden	2017	80.9%	83.3%	78.4%
Algeria	Bir Ben Laabed	2000	76.5%	80.2%	72.6%
Algeria	Bir Ben Laabed	2017	76.6%	80.4%	72.8%
Algeria	Bir Bouhouche	2000	71.7%	76.0%	67.1%
Algeria	Bir Bouhouche	2017	72.0%	76.3%	67.4%
Algeria	Bir Chouhada	2000	67.5%	70.8%	64.0%
Algeria	Bir Chouhada	2017	67.7%	71.0%	64.2%
Algeria	Bir Dheb	2000	62.5%	66.1%	58.7%
Algeria	Bir Dheb	2017	62.7%	66.3%	58.9%
Algeria	Bir El Ater	2000	63.4%	67.4%	59.0%
Algeria	Bir El Ater	2017	63.6%	67.6%	59.4%
Algeria	Bir El Djir	2000	70.8%	73.1%	68.4%
Algeria	Bir El Djir	2017	71.3%	73.6%	68.8%
Algeria	Bir El Hammam	2000	74.4%	79.0%	69.3%
Algeria	Bir El Hammam	2017	74.9%	79.2%	70.1%
Algeria	Bir Foda	2000	80.9%	83.8%	77.7%
Algeria	Bir Foda	2017	80.9%	83.7%	77.6%
Algeria	Bir Ghbalou	2000	77.3%	80.4%	73.7%
Algeria	Bir Ghbalou	2017	77.4%	80.5%	73.9%
Algeria	Bir Haddada	2000	61.2%	64.5%	57.9%
Algeria	Bir Haddada	2017	61.5%	64.8%	58.1%
Algeria	Bir Kasdali	2000	61.4%	64.0%	58.6%
Algeria	Bir Kasdali	2017	61.6%	64.1%	58.7%
Algeria	Bir Mokkadem	2000	61.6%	65.1%	58.1%
Algeria	Bir Mokkadem	2017	61.9%	65.4%	58.4%
Algeria	Bir Ould Khelifa	2000	75.7%	78.5%	72.2%
Algeria	Bir Ould Khelifa	2017	75.9%	78.7%	72.4%
Algeria	Bir-El-Arch	2000	66.4%	69.2%	63.7%
Algeria	Bir-El-Arch	2017	66.6%	69.4%	63.9%
Algeria	Birine	2000	80.3%	83.5%	76.5%
Algeria	Birine	2017	80.5%	83.8%	76.8%
Algeria	Birtouta	2000	75.5%	77.4%	73.8%
Algeria	Birtouta	2017	75.6%	77.6%	74.0%
Algeria	Biskra	2000	47.8%	50.1%	45.2%
Algeria	Biskra	2017	48.2%	50.5%	45.6%
Algeria	Bitam	2000	60.2%	63.2%	56.1%
Algeria	Bitam	2017	60.5%	63.5%	56.4%
Algeria	Blida	2000	75.9%	77.9%	73.4%
Algeria	Blida	2017	76.1%	78.2%	73.6%
Algeria	Boghni	2000	77.0%	79.9%	74.2%

Country	Second Administrative Unit	Year	Mean	Upper	Lower
Algeria	Boghni	2017	77.0%	79.9%	74.2%
Algeria	Bordj Badji Mokhtar	2000	43.3%	48.6%	38.0%
Algeria	Bordj Badji Mokhtar	2017	42.8%	48.5%	37.1%
Algeria	Bordj Ben Azzouz	2000	49.6%	52.4%	46.3%
Algeria	Bordj Ben Azzouz	2017	49.8%	52.7%	46.5%
Algeria	Bordj Bou Arreridj	2000	63.5%	65.8%	61.0%
Algeria	Bordj Bou Arreridj	2017	63.9%	66.1%	61.4%
Algeria	Bordj Bounaama	2000	77.9%	80.8%	74.9%
Algeria	Bordj Bounaama	2017	78.1%	81.0%	75.1%
Algeria	Bordj El Emir Abdelkader	2000	78.2%	80.9%	75.2%
Algeria	Bordj El Emir Abdelkader	2017	78.5%	81.2%	75.4%
Algeria	Bordj El Haouasse	2000	51.8%	61.2%	41.1%
Algeria	Bordj El Haouasse	2017	52.1%	61.5%	41.5%
Algeria	Bordj El Kiffan	2000	75.4%	77.0%	73.6%
Algeria	Bordj El Kiffan	2017	75.6%	77.2%	73.8%
Algeria	Bordj Emir Khaled	2000	76.8%	80.0%	73.8%
Algeria	Bordj Emir Khaled	2017	77.0%	80.2%	74.0%
Algeria	Bordj Ghdir	2000	71.0%	74.0%	67.9%
Algeria	Bordj Ghdir	2017	71.4%	74.4%	68.3%
Algeria	Bordj Menaiel	2000	75.9%	78.7%	73.1%
Algeria	Bordj Menaiel	2017	76.0%	78.8%	73.2%
Algeria	Bordj Okhriss	2000	77.7%	81.2%	74.1%
Algeria	Bordj Okhriss	2017	78.0%	81.5%	74.6%
Algeria	Bordj Omar Driss	2000	49.9%	57.7%	40.6%
Algeria	Bordj Omar Driss	2017	49.8%	57.1%	40.7%
Algeria	Bordj Sebbat	2000	80.1%	82.8%	77.5%
Algeria	Bordj Sebbat	2017	80.3%	83.0%	77.7%
Algeria	Bordj Tahar	2000	83.8%	86.0%	81.5%
Algeria	Bordj Tahar	2017	84.0%	86.2%	81.7%
Algeria	Bordj Zemoura	2000	64.2%	67.4%	60.9%
Algeria	Bordj Zemoura	2017	64.6%	67.7%	61.3%
Algeria	Bou Caid	2000	78.2%	81.0%	75.2%
Algeria	Bou Caid	2017	78.3%	81.2%	75.4%
Algeria	Bou Hachana	2000	78.8%	82.1%	75.8%
Algeria	Bou Hachana	2017	79.0%	82.3%	76.0%
Algeria	Bou Hamdane	2000	80.5%	83.1%	77.8%
Algeria	Bou Hamdane	2017	80.7%	83.3%	78.1%
Algeria	Bou Henni	2000	68.4%	70.9%	65.8%
Algeria	Bou Henni	2017	68.6%	71.1%	66.0%
Algeria	Bou Ismail	2000	74.8%	77.8%	71.5%
Algeria	Bou Ismail	2017	75.0%	78.0%	71.8%
Algeria	Bou Saada	2000	81.4%	83.5%	79.5%

Country	Second Administrative Unit	Year	Mean	Upper	Lower
Algeria	Bou Saada	2017	81.6%	83.6%	79.6%
Algeria	Bou Zedjar	2000	69.3%	74.1%	64.1%
Algeria	Bou Zedjar	2017	69.5%	74.3%	64.3%
Algeria	Bouaarfa	2000	76.5%	78.7%	73.8%
Algeria	Bouaarfa	2017	76.7%	78.8%	73.9%
Algeria	Bouaiche	2000	78.0%	81.2%	74.9%
Algeria	Bouaiche	2017	78.2%	81.4%	75.1%
Algeria	Bouaichoune	2000	76.7%	79.6%	73.2%
Algeria	Bouaichoune	2017	76.8%	79.8%	73.4%
Algeria	Boualem	2000	79.7%	83.5%	75.9%
Algeria	Boualem	2017	79.9%	83.6%	76.2%
Algeria	Bouandas	2000	69.9%	73.1%	66.8%
Algeria	Bouandas	2017	69.7%	72.9%	66.5%
Algeria	Bouati Mahmoud	2000	80.1%	82.5%	77.7%
Algeria	Bouati Mahmoud	2017	80.4%	82.8%	77.9%
Algeria	Bouchakroune	2000	49.0%	52.5%	45.9%
Algeria	Bouchakroune	2017	49.2%	52.8%	46.1%
Algeria	Bouchekouf	2000	79.7%	81.6%	77.4%
Algeria	Bouchekouf	2017	79.4%	81.4%	77.1%
Algeria	Boucherahil	2000	76.7%	80.1%	73.0%
Algeria	Boucherahil	2017	76.9%	80.2%	73.1%
Algeria	Bouchetata	2000	81.1%	83.2%	78.7%
Algeria	Bouchetata	2017	81.2%	83.3%	78.9%
Algeria	Bouda	2000	48.9%	54.6%	43.0%
Algeria	Bouda	2017	49.1%	54.8%	43.3%
Algeria	Bouderbala	2000	77.5%	80.3%	74.6%
Algeria	Bouderbala	2017	77.7%	80.4%	74.8%
Algeria	Boudjebaa El Bordj	2000	70.0%	73.7%	66.8%
Algeria	Boudjebaa El Bordj	2017	70.1%	73.7%	67.0%
Algeria	Boudjellil	2000	72.4%	76.6%	68.7%
Algeria	Boudjellil	2017	72.6%	76.7%	69.0%
Algeria	Boudjeriou Messaoud	2000	81.3%	83.6%	78.8%
Algeria	Boudjeriou Messaoud	2017	81.3%	83.7%	78.9%
Algeria	Boudjima	2000	77.9%	81.1%	74.6%
Algeria	Boudjima	2017	78.0%	81.1%	74.7%
Algeria	Boudouaou	2000	76.1%	78.2%	74.2%
Algeria	Boudouaou	2017	76.2%	78.3%	74.3%
Algeria	Boudouaou El Bahri	2000	75.3%	77.6%	73.1%
Algeria	Boudouaou El Bahri	2017	75.5%	77.7%	73.3%
Algeria	Boudria Beniyadjis	2000	79.7%	82.4%	76.7%
Algeria	Boudria Beniyadjis	2017	79.9%	82.5%	76.9%
Algeria	Boufarik	2000	74.9%	77.1%	72.6%
Algeria	Boufarik	2017	75.1%	77.3%	72.9%
Algeria	Boufatis	2000	70.2%	72.8%	67.6%
Algeria	Boufatis	2017	70.5%	73.0%	67.9%
Algeria	Bougaa	2000	64.6%	67.8%	61.6%
Algeria	Bougaa	2017	64.8%	68.0%	61.8%
Algeria	Bougara	2000	76.1%	78.9%	73.1%
Algeria	Bougara	2000	78.7%	81.0%	76.0%
Algeria	Bougara	2017	76.1%	78.9%	73.1%
Algeria	Bougara	2017	78.9%	81.2%	76.3%
Algeria	Boughar	2000	76.5%	80.0%	73.2%

Country	Second Administrative Unit	Year	Mean	Upper	Lower
Algeria	Boughar	2017	76.7%	80.2%	73.4%
Algeria	Boughezoul	2000	78.6%	82.1%	75.4%
Algeria	Boughezoul	2017	78.9%	82.3%	75.7%
Algeria	Bougous	2000	80.8%	84.0%	77.3%
Algeria	Bougous	2017	80.6%	83.9%	77.2%
Algeria	Bougtoub	2000	78.3%	81.0%	75.4%
Algeria	Bougtoub	2017	78.4%	81.2%	75.6%
Algeria	Bouguirat	2000	70.4%	72.5%	67.8%
Algeria	Bouguirat	2017	70.5%	72.6%	67.9%
Algeria	Bouhadjar	2000	79.8%	82.9%	76.4%
Algeria	Bouhadjar	2017	79.9%	83.1%	76.6%
Algeria	Bouhamza	2000	71.5%	74.4%	68.1%
Algeria	Bouhamza	2017	71.7%	74.6%	68.3%
Algeria	Bouhanifia	2000	70.2%	73.1%	67.2%
Algeria	Bouhanifia	2017	70.4%	73.3%	67.4%
Algeria	Bouhatem	2000	78.5%	80.7%	76.2%
Algeria	Bouhatem	2017	78.6%	80.9%	76.3%
Algeria	Bouhlou	2000	68.1%	71.6%	64.5%
Algeria	Bouhlou	2017	68.7%	72.2%	65.0%
Algeria	Bouhmama	2000	61.6%	66.3%	56.4%
Algeria	Bouhmama	2017	61.5%	66.4%	56.2%
Algeria	Bouihi	2000	71.6%	76.3%	66.6%
Algeria	Bouihi	2017	71.8%	76.4%	66.8%
Algeria	Bouinan	2000	75.8%	78.4%	73.1%
Algeria	Bouinan	2017	75.9%	78.5%	73.2%
Algeria	Bouira	2000	76.4%	79.3%	73.5%
Algeria	Bouira	2017	76.6%	79.5%	73.7%
Algeria	Bouira Lahdab	2000	81.3%	83.4%	79.1%
Algeria	Bouira Lahdab	2017	81.5%	83.5%	79.3%
Algeria	Boukadir	2000	73.2%	75.8%	70.6%
Algeria	Boukadir	2017	73.4%	76.0%	70.7%
Algeria	Boukais	2000	52.7%	59.9%	46.2%
Algeria	Boukais	2017	53.0%	60.2%	46.5%
Algeria	Boukhadra	2000	64.1%	68.5%	60.7%
Algeria	Boukhadra	2017	64.1%	68.3%	60.6%
Algeria	Boukhenifis	2000	71.3%	74.6%	68.6%
Algeria	Boukhenifis	2017	71.6%	74.8%	68.8%
Algeria	Boukhlifa	2000	74.1%	76.8%	70.9%
Algeria	Boukhlifa	2017	74.6%	77.2%	71.5%
Algeria	Boukram	2000	77.6%	80.7%	74.5%
Algeria	Boukram	2017	77.9%	80.9%	74.8%
Algeria	Boulhaf Dyr	2000	62.4%	65.4%	59.3%
Algeria	Boulhaf Dyr	2017	62.3%	65.3%	59.2%
Algeria	Boulhilat	2000	61.2%	64.9%	57.4%
Algeria	Boulhilat	2017	61.6%	65.3%	57.7%
Algeria	Boumahra Ahmed	2000	78.1%	80.9%	75.4%
Algeria	Boumahra Ahmed	2017	78.3%	81.0%	75.6%
Algeria	Boumedfaa	2000	76.1%	78.8%	73.2%
Algeria	Boumedfaa	2017	76.2%	79.0%	73.3%
Algeria	Boumegueur	2000	62.2%	65.6%	59.1%
Algeria	Boumegueur	2017	62.4%	65.7%	59.3%
Algeria	Boumerdes	2000	75.5%	78.3%	73.0%
Algeria	Boumerdes	2017	75.7%	78.6%	73.3%
Algeria	Boumia	2000	62.2%	65.2%	59.4%
Algeria	Boumia	2017	62.4%	65.4%	59.7%
Algeria	Bounouh	2000	76.9%	79.9%	74.0%
Algeria	Bounouh	2017	76.9%	79.9%	74.0%
Algeria	Bounoura	2000	49.3%	52.7%	46.1%

Country	Second Administrative Unit	Year	Mean	Upper	Lower
Algeria	Bounoura	2017	49.4%	52.8%	46.3%
Algeria	Bourached	2000	76.7%	79.0%	74.2%
Algeria	Bourached	2017	76.9%	79.3%	74.5%
Algeria	Bouraoui Belhadef	2000	84.5%	86.9%	82.0%
Algeria	Bouraoui Belhadef	2017	84.6%	87.1%	82.1%
Algeria	Bourkika	2000	75.4%	78.6%	72.3%
Algeria	Bourkika	2017	75.6%	78.7%	72.4%
Algeria	Bousfer	2000	69.7%	73.5%	65.7%
Algeria	Bousfer	2017	69.7%	73.5%	65.8%
Algeria	Bouskene	2000	76.5%	79.9%	72.8%
Algeria	Bouskene	2017	76.6%	79.9%	72.8%
Algeria	Bousselam	2000	69.3%	72.4%	66.4%
Algeria	Bousselam	2017	69.4%	72.6%	66.5%
Algeria	Boussemghoun	2000	78.8%	82.1%	74.5%
Algeria	Boussemghoun	2017	79.2%	82.5%	75.0%
Algeria	Boussif Ouled Askeur	2000	84.1%	86.5%	81.8%
Algeria	Boussif Ouled Askeur	2017	84.2%	86.6%	81.9%
Algeria	Boutaleb	2000	71.5%	74.3%	68.5%
Algeria	Boutaleb	2017	71.4%	74.2%	68.4%
Algeria	Bouteldja	2000	79.7%	82.6%	77.2%
Algeria	Bouteldja	2017	79.9%	82.8%	77.3%
Algeria	Bouti Sayeh	2000	80.9%	84.4%	77.4%
Algeria	Bouti Sayeh	2017	81.0%	84.5%	77.6%
Algeria	Boutlelis	2000	70.8%	74.4%	67.3%
Algeria	Boutlelis	2017	70.9%	74.5%	67.5%
Algeria	Bouzareah	2000	75.5%	77.3%	73.6%
Algeria	Bouzareah	2017	75.7%	77.5%	73.8%
Algeria	Bouzeghaia	2000	76.0%	79.9%	72.2%
Algeria	Bouzeghaia	2017	76.2%	80.1%	72.4%
Algeria	Bouzeguene	2000	77.6%	80.0%	75.2%
Algeria	Bouzeguene	2017	77.9%	80.2%	75.4%
Algeria	Bouzegza Keddara	2000	77.1%	79.5%	74.8%
Algeria	Bouzegza Keddara	2017	77.4%	79.8%	75.1%
Algeria	Bouzina	2000	60.9%	64.6%	57.1%
Algeria	Bouzina	2017	61.7%	65.4%	58.1%
Algeria	Branis	2000	49.4%	52.0%	47.1%
Algeria	Branis	2017	49.6%	52.1%	47.2%
Algeria	Breira	2000	76.8%	81.2%	72.4%
Algeria	Breira	2017	76.9%	81.2%	72.5%
Algeria	Brezina	2000	76.4%	81.3%	71.9%
Algeria	Brezina	2017	75.2%	80.2%	70.4%
Algeria	Brida	2000	80.1%	83.4%	76.4%
Algeria	Brida	2017	80.2%	83.7%	76.5%
Algeria	Chaabet El Ham	2000	70.8%	73.2%	68.1%
Algeria	Chaabet El Ham	2017	71.0%	73.4%	68.2%
Algeria	Chabet El Ameur	2000	76.3%	78.9%	73.9%
Algeria	Chabet El Ameur	2017	76.4%	79.0%	74.0%
Algeria	Chahbounia	2000	78.9%	82.1%	75.0%
Algeria	Chahbounia	2017	79.1%	82.3%	75.1%
Algeria	Chahna	2000	82.5%	84.9%	80.7%
Algeria	Chahna	2017	82.6%	84.9%	80.8%
Algeria	Chaiba	2000	75.4%	77.8%	72.8%

Country	Second Administrative Unit	Year	Mean	Upper	Lower
Algeria	Chaiba	2017	75.6%	78.0%	73.0%
Algeria	Charef	2000	81.6%	84.3%	78.6%
Algeria	Charef	2017	81.9%	84.7%	78.9%
Algeria	Charouine	2000	49.2%	55.4%	42.5%
Algeria	Charouine	2017	49.4%	55.4%	43.1%
Algeria	Chebaita Mokhtar	2000	80.5%	82.1%	78.5%
Algeria	Chebaita Mokhtar	2017	80.5%	82.1%	78.5%
Algeria	Chebli	2000	75.7%	78.2%	73.2%
Algeria	Chebli	2017	76.0%	78.4%	73.4%
Algeria	Chechar	2000	63.4%	68.7%	58.4%
Algeria	Chechar	2017	63.8%	69.1%	58.7%
Algeria	Chefia	2000	80.2%	83.0%	77.1%
Algeria	Chefia	2017	80.3%	83.1%	77.2%
Algeria	Cheguig	2000	78.8%	82.5%	74.6%
Algeria	Cheguig	2017	78.9%	82.7%	74.9%
Algeria	Chehaima	2000	78.4%	80.7%	75.5%
Algeria	Chehaima	2017	78.7%	80.9%	75.8%
Algeria	Chekfa	2000	81.6%	83.5%	79.5%
Algeria	Chekfa	2017	81.9%	83.7%	79.8%
Algeria	Chelghoum Laid	2000	77.3%	79.2%	75.3%
Algeria	Chelghoum Laid	2017	77.6%	79.4%	75.7%
Algeria	Chelia	2000	60.1%	63.3%	56.4%
Algeria	Chelia	2017	60.0%	63.3%	56.3%
Algeria	Chellal	2000	80.5%	83.2%	77.9%
Algeria	Chellal	2017	80.6%	83.2%	77.9%
Algeria	Chellala	2000	80.0%	83.9%	75.7%
Algeria	Chellala	2017	80.2%	83.9%	75.9%
Algeria	Chellalet Ladhaoura	2000	77.5%	81.4%	73.1%
Algeria	Chellalet Ladhaoura	2017	77.7%	81.6%	73.2%
Algeria	Chellata	2000	75.3%	77.7%	72.9%
Algeria	Chellata	2017	75.4%	77.7%	73.0%
Algeria	Chemini	2000	76.3%	78.9%	73.9%
Algeria	Chemini	2017	76.4%	79.1%	74.1%
Algeria	Chemora	2000	61.0%	65.0%	57.3%
Algeria	Chemora	2017	61.3%	65.3%	57.7%
Algeria	Cheniguel	2000	77.6%	81.4%	73.3%
Algeria	Cheniguel	2017	77.7%	81.4%	73.3%
Algeria	Chentouf	2000	71.3%	74.0%	68.6%
Algeria	Chentouf	2017	71.5%	74.2%	68.8%
Algeria	Cheraga	2000	75.5%	77.4%	73.7%
Algeria	Cheraga	2000	82.5%	84.8%	80.2%
Algeria	Cheraga	2017	82.8%	85.1%	80.6%
Algeria	Cheraga	2017	75.8%	77.6%	74.0%
Algeria	Cheraia	2000	81.5%	84.5%	77.9%
Algeria	Cheraia	2017	81.6%	84.6%	78.0%
Algeria	Cherchel	2000	75.6%	80.2%	70.6%
Algeria	Cherchel	2017	75.7%	80.3%	70.6%
Algeria	Chetaibi	2000	80.7%	84.7%	76.3%
Algeria	Chetaibi	2017	80.8%	84.7%	76.5%
Algeria	Chetma	2000	47.3%	50.4%	44.7%
Algeria	Chetma	2017	47.4%	50.5%	44.8%
Algeria	Chetouane	2000	69.5%	72.0%	66.8%
Algeria	Chetouane	2017	69.8%	72.3%	67.1%
Algeria	Chetouane Belaila	2000	70.5%	73.9%	67.0%
Algeria	Chetouane Belaila	2017	70.7%	74.1%	67.2%

Country	Second Administrative Unit	Year	Mean	Upper	Lower
Algeria	Chettia	2000	74.8%	77.0%	72.6%
Algeria	Chettia	2017	75.0%	77.2%	72.8%
Algeria	Chiffa	2000	75.8%	78.1%	73.3%
Algeria	Chiffa	2017	76.0%	78.3%	73.4%
Algeria	Chihani	2000	80.4%	82.3%	78.2%
Algeria	Chihani	2017	80.6%	82.5%	78.3%
Algeria	Chir	2000	60.6%	64.6%	56.7%
Algeria	Chir	2017	61.4%	65.3%	57.5%
Algeria	Chlef	2000	75.0%	76.9%	72.9%
Algeria	Chlef	2017	75.2%	77.1%	73.1%
Algeria	Chorfa	2000	73.3%	77.3%	69.0%
Algeria	Chorfa	2000	80.3%	82.2%	78.1%
Algeria	Chorfa	2000	69.8%	72.3%	67.5%
Algeria	Chorfa	2017	73.5%	77.5%	69.2%
Algeria	Chorfa	2017	80.4%	82.2%	78.2%
Algeria	Chorfa	2017	69.9%	72.5%	67.5%
Algeria	Chouaiba|Ouled Rahma	2000	58.4%	62.1%	54.8%
Algeria	Chouaiba|Ouled Rahma	2017	59.0%	62.6%	55.3%
Algeria	Chrea	2000	61.4%	64.8%	58.1%
Algeria	Chrea	2000	77.5%	80.0%	74.8%
Algeria	Chrea	2017	77.6%	80.0%	74.9%
Algeria	Chrea	2017	61.6%	65.0%	58.2%
Algeria	Colla	2000	68.4%	71.9%	64.8%
Algeria	Colla	2017	68.8%	72.4%	65.1%
Algeria	Collo	2000	81.0%	84.1%	77.0%
Algeria	Collo	2017	81.1%	84.3%	77.1%
Algeria	Constantine	2000	79.9%	81.2%	78.6%
Algeria	Constantine	2017	80.0%	81.3%	78.7%
Algeria	Corso	2000	76.0%	78.7%	73.7%
Algeria	Corso	2017	76.1%	78.8%	73.9%
Algeria	Dahmouni	2000	79.5%	81.4%	77.9%
Algeria	Dahmouni	2017	79.5%	81.3%	77.9%
Algeria	Dahouara	2000	79.7%	82.2%	77.1%
Algeria	Dahouara	2017	79.9%	82.4%	77.3%
Algeria	Dahra	2000	72.0%	75.4%	68.4%
Algeria	Dahra	2017	72.3%	75.7%	68.6%
Algeria	Damous	2000	75.8%	81.1%	70.4%
Algeria	Damous	2017	76.0%	81.2%	70.8%
Algeria	Daoussen	2000	49.9%	53.0%	46.6%
Algeria	Daoussen	2017	50.1%	53.2%	46.7%
Algeria	Dar Ben Abdelah	2000	73.4%	76.3%	70.7%
Algeria	Dar Ben Abdelah	2017	73.5%	76.3%	70.8%
Algeria	Dar Chioukh	2000	82.0%	84.3%	79.9%
Algeria	Dar Chioukh	2017	82.3%	84.5%	80.1%
Algeria	Dar El Beida	2000	75.3%	76.6%	73.8%
Algeria	Dar El Beida	2017	75.5%	76.8%	74.0%
Algeria	Dar Yaghmouracene	2000	67.4%	71.4%	63.3%
Algeria	Dar Yaghmouracene	2017	67.6%	71.6%	63.5%
Algeria	Darguina	2000	71.5%	74.9%	67.5%
Algeria	Darguina	2017	71.8%	75.2%	67.8%
Algeria	Debdeb	2000	50.2%	56.6%	42.9%
Algeria	Debdeb	2017	50.4%	56.4%	43.3%
Algeria	Debila	2000	48.5%	52.5%	44.3%
Algeria	Debila	2017	48.7%	52.7%	44.5%
Algeria	Dechmia	2000	77.5%	80.9%	74.0%
Algeria	Dechmia	2017	77.7%	81.1%	74.3%

Country	Second Administrative Unit	Year	Mean	Upper	Lower
Algeria	Dehahna	2000	76.2%	79.1%	73.4%
Algeria	Dehahna	2017	76.6%	79.4%	73.8%
Algeria	Dehamcha	2000	68.9%	71.7%	66.0%
Algeria	Dehamcha	2017	69.0%	71.9%	66.1%
Algeria	Deldoul	2000	49.1%	55.0%	43.0%
Algeria	Deldoul	2000	80.9%	82.9%	79.2%
Algeria	Deldoul	2017	49.3%	55.2%	43.3%
Algeria	Deldoul	2017	81.0%	82.9%	79.4%
Algeria	Dellys	2000	77.4%	81.0%	73.2%
Algeria	Dellys	2017	77.6%	81.3%	73.5%
Algeria	Derradji Bousselah	2000	75.5%	78.3%	72.6%
Algeria	Derradji Bousselah	2017	75.7%	78.5%	72.8%
Algeria	Derrag	2000	78.5%	81.4%	74.7%
Algeria	Derrag	2017	78.6%	81.6%	74.9%
Algeria	Deux Bassins	2000	78.2%	81.5%	74.9%
Algeria	Deux Bassins	2017	78.4%	81.7%	75.1%
Algeria	Dhaya	2000	71.8%	76.1%	66.9%
Algeria	Dhaya	2017	71.9%	76.2%	67.0%
Algeria	Dhayet Bendhahoua	2000	63.1%	67.0%	57.9%
Algeria	Dhayet Bendhahoua	2017	62.4%	66.1%	57.9%
Algeria	Didouche Mourad	2000	81.1%	83.1%	79.2%
Algeria	Didouche Mourad	2017	81.3%	83.2%	79.3%
Algeria	Dirrah	2000	78.7%	81.6%	75.2%
Algeria	Dirrah	2017	79.0%	82.0%	75.5%
Algeria	Djaafra	2000	68.8%	72.1%	65.1%
Algeria	Djaafra	2017	69.0%	72.3%	65.3%
Algeria	Djamaa	2000	49.3%	52.1%	46.4%
Algeria	Djamaa	2017	49.6%	52.3%	46.6%
Algeria	Djamora	2000	51.8%	55.1%	48.3%
Algeria	Djamora	2017	52.0%	55.2%	48.6%
Algeria	Djanet	2000	50.6%	60.3%	42.0%
Algeria	Djanet	2017	50.5%	59.4%	41.9%
Algeria	Djasr Kasentina	2000	75.6%	77.0%	74.1%
Algeria	Djasr Kasentina	2017	75.7%	77.1%	74.3%
Algeria	Djebabra	2000	76.6%	79.2%	73.9%
Algeria	Djebabra	2017	76.8%	79.4%	74.1%
Algeria	Djebahia	2000	76.1%	78.6%	73.2%
Algeria	Djebahia	2017	76.1%	78.7%	73.2%
Algeria	Djebala	2000	69.1%	72.3%	66.2%
Algeria	Djebala	2017	69.2%	72.4%	66.2%
Algeria	Djebala El Khemissi	2000	78.9%	81.1%	76.5%
Algeria	Djebala El Khemissi	2017	78.9%	81.1%	76.4%
Algeria	Djebel Messaad	2000	81.3%	84.0%	78.0%
Algeria	Djebel Messaad	2017	81.4%	84.2%	78.1%
Algeria	Djebilet Rosfa	2000	76.9%	79.9%	73.3%
Algeria	Djebilet Rosfa	2017	77.2%	80.1%	73.7%
Algeria	Djelfa	2000	81.5%	82.7%	80.2%
Algeria	Djelfa	2017	81.5%	82.7%	80.3%
Algeria	Djelida	2000	76.1%	79.1%	72.9%
Algeria	Djelida	2017	76.2%	79.2%	72.9%

Country	Second Administrative Unit	Year	Mean	Upper	Lower
Algeria	Djellal	2000	62.3%	68.0%	57.1%
Algeria	Djellal	2017	62.3%	68.0%	57.2%
Algeria	Djemaa Beni Habibi	2000	83.0%	85.3%	80.4%
Algeria	Djemaa Beni Habibi	2017	83.1%	85.4%	80.4%
Algeria	Djemaa Ouled Cheikh	2000	76.8%	79.8%	73.7%
Algeria	Djemaa Ouled Cheikh	2017	77.0%	80.0%	73.9%
Algeria	Djemila	2000	70.3%	73.2%	66.9%
Algeria	Djemila	2000	81.3%	84.2%	78.6%
Algeria	Djemila	2017	70.0%	72.9%	66.7%
Algeria	Djemila	2017	81.2%	84.0%	78.5%
Algeria	Djendel	2000	76.1%	79.0%	72.9%
Algeria	Djendel	2017	76.2%	79.2%	72.9%
Algeria	Djendel Saadi Mohamed	2000	80.8%	82.9%	78.5%
Algeria	Djendel Saadi Mohamed	2017	80.9%	83.0%	78.6%
Algeria	Djeniane Bourzeg	2000	71.6%	75.9%	66.8%
Algeria	Djeniane Bourzeg	2017	71.2%	75.6%	66.3%
Algeria	Djerma	2000	63.6%	66.4%	60.6%
Algeria	Djerma	2017	63.8%	66.6%	60.8%
Algeria	Djezzar	2000	68.2%	71.1%	65.6%
Algeria	Djezzar	2017	69.0%	72.0%	66.3%
Algeria	Djidiouia	2000	69.8%	72.3%	66.7%
Algeria	Djidiouia	2017	70.0%	72.5%	66.9%
Algeria	Djillali Ben Ammar	2000	75.8%	78.7%	73.6%
Algeria	Djillali Ben Ammar	2017	76.2%	79.0%	74.1%
Algeria	Djinet	2000	76.6%	79.9%	72.6%
Algeria	Djinet	2017	76.7%	80.0%	72.8%
Algeria	Djouab	2000	77.2%	80.9%	73.7%
Algeria	Djouab	2017	77.3%	81.0%	73.7%
Algeria	Douaouda	2000	74.9%	77.3%	72.2%
Algeria	Douaouda	2017	75.1%	77.5%	72.4%
Algeria	Douar El Ma	2000	49.3%	55.4%	41.6%
Algeria	Douar El Ma	2017	49.1%	55.6%	41.9%
Algeria	Douera	2000	75.6%	77.3%	73.9%
Algeria	Douera	2017	75.9%	77.6%	74.2%
Algeria	Doui Thabet	2000	76.6%	78.8%	74.2%
Algeria	Doui Thabet	2017	76.7%	79.0%	74.1%
Algeria	Douis	2000	81.2%	84.3%	78.1%
Algeria	Douis	2017	81.3%	84.3%	77.9%
Algeria	Draa El Caid	2000	67.4%	70.7%	64.1%
Algeria	Draa El Caid	2017	67.8%	71.0%	64.5%
Algeria	Draa El Mizan	2000	76.8%	79.5%	73.9%
Algeria	Draa El Mizan	2017	77.0%	79.7%	74.2%
Algeria	Draa Smar	2000	76.0%	79.2%	72.5%
Algeria	Draa Smar	2017	76.1%	79.2%	72.6%
Algeria	Draa-Ben-Khedda	2000	76.2%	78.7%	73.6%
Algeria	Draa-Ben-Khedda	2017	76.3%	78.9%	73.8%
Algeria	Draa-Kebila	2000	67.2%	70.1%	64.3%
Algeria	Draa-Kebila	2017	67.4%	70.3%	64.6%
Algeria	Draria	2000	76.0%	77.6%	74.6%
Algeria	Draria	2017	76.2%	77.8%	74.8%

Country	Second Administrative Unit	Year	Mean	Upper	Lower
Algeria	Drea	2000	74.9%	77.6%	72.1%
Algeria	Drea	2017	75.3%	77.9%	72.4%
Algeria	Drean	2000	80.2%	81.9%	77.9%
Algeria	Drean	2017	80.3%	82.0%	78.1%
Algeria	Echatt	2000	80.6%	82.6%	78.5%
Algeria	Echatt	2017	80.8%	82.9%	78.6%
Algeria	El Abadia	2000	76.1%	79.0%	72.9%
Algeria	El Abadia	2017	76.2%	79.1%	72.9%
Algeria	El Ach	2000	74.2%	76.5%	71.7%
Algeria	El Ach	2017	74.0%	76.3%	71.6%
Algeria	El Achir	2000	68.1%	71.3%	65.2%
Algeria	El Achir	2017	68.1%	71.1%	65.3%
Algeria	El Achour	2000	76.1%	77.6%	74.6%
Algeria	El Achour	2017	76.3%	77.7%	74.8%
Algeria	El Adjiba	2000	75.6%	79.7%	71.7%
Algeria	El Adjiba	2017	75.7%	79.8%	71.7%
Algeria	El Aioun	2000	80.5%	84.9%	75.9%
Algeria	El Aioun	2017	80.7%	85.1%	76.1%
Algeria	El Allia	2000	50.3%	55.1%	45.6%
Algeria	El Allia	2017	50.9%	55.7%	45.9%
Algeria	El Amiria	2000	70.6%	73.0%	67.9%
Algeria	El Amiria	2017	70.7%	73.1%	67.9%
Algeria	El Amra	2000	76.3%	79.1%	73.2%
Algeria	El Amra	2017	76.3%	79.1%	73.2%
Algeria	El Amria	2000	70.3%	74.3%	66.0%
Algeria	El Amria	2017	70.8%	74.8%	66.6%
Algeria	El Ançar	2000	70.0%	74.1%	65.9%
Algeria	El Ançar	2017	70.2%	74.3%	66.1%
Algeria	El Ancer	2000	82.6%	85.0%	79.8%
Algeria	El Ancer	2017	82.6%	85.0%	79.8%
Algeria	El Anseur	2000	64.2%	66.7%	61.3%
Algeria	El Anseur	2017	64.4%	66.9%	61.4%
Algeria	El Aouana	2000	78.6%	81.7%	75.1%
Algeria	El Aouana	2017	78.7%	81.8%	75.1%
Algeria	El Aricha	2000	72.6%	78.1%	66.8%
Algeria	El Aricha	2017	72.8%	78.3%	67.0%
Algeria	El Asnam	2000	76.4%	79.6%	72.9%
Algeria	El Asnam	2017	76.1%	79.5%	72.7%
Algeria	El Assafia	2000	82.0%	84.0%	79.7%
Algeria	El Assafia	2017	82.2%	84.2%	79.9%
Algeria	El Attaf	2000	76.0%	78.6%	72.9%
Algeria	El Attaf	2017	76.1%	78.7%	73.0%
Algeria	El Atteuf	2000	47.8%	51.6%	44.2%
Algeria	El Atteuf	2017	48.1%	52.0%	44.4%
Algeria	El Bayadh	2000	78.7%	80.9%	76.5%
Algeria	El Bayadh	2017	78.9%	81.1%	76.7%
Algeria	El Beidha	2000	79.7%	83.4%	75.9%
Algeria	El Beidha	2017	79.9%	83.6%	76.1%
Algeria	El Biod	2000	77.4%	80.0%	74.7%
Algeria	El Biod	2017	77.6%	80.4%	74.9%
Algeria	El Biodh Sidi Cheikh	2000	77.9%	81.2%	74.8%
Algeria	El Biodh Sidi Cheikh	2017	77.5%	80.8%	74.3%
Algeria	El Bnoud	2000	72.7%	77.3%	67.7%
Algeria	El Bnoud	2017	70.5%	75.4%	64.8%
Algeria	El Bordj	2000	73.8%	76.6%	70.4%
Algeria	El Bordj	2017	74.0%	76.8%	70.6%
Algeria	El Borma	2000	49.8%	55.1%	43.6%
Algeria	El Borma	2017	50.0%	55.2%	43.7%
Algeria	El Bouni	2000	80.5%	82.0%	78.8%
Algeria	El Bouni	2017	80.6%	82.1%	79.0%

Country	Second Administrative Unit	Year	Mean	Upper	Lower
Algeria	El Braya	2000	69.8%	72.0%	67.9%
Algeria	El Braya	2017	70.0%	72.2%	68.0%
Algeria	El Dhaala	2000	60.9%	65.7%	56.3%
Algeria	El Dhaala	2017	61.1%	65.9%	56.6%
Algeria	El Djazia	2000	62.6%	67.4%	57.8%
Algeria	El Djazia	2017	62.6%	67.5%	57.7%
Algeria	El Eulma	2000	62.4%	64.7%	59.9%
Algeria	El Eulma	2000	80.1%	82.4%	77.4%
Algeria	El Eulma	2017	62.6%	64.9%	60.2%
Algeria	El Eulma	2017	80.2%	82.6%	77.5%
Algeria	El Fedjoudj	2000	78.5%	80.8%	76.1%
Algeria	El Fedjoudj	2017	78.8%	81.0%	76.3%
Algeria	El Fedjoudj Boughrara Saoudi	2000	59.8%	63.8%	55.3%
Algeria	El Fedjoudj Boughrara Saoudi	2017	59.9%	64.0%	55.4%
Algeria	El Fehoul	2000	70.1%	73.6%	67.3%
Algeria	El Fehoul	2017	70.0%	73.5%	67.2%
Algeria	El Feidh	2000	51.8%	57.6%	45.7%
Algeria	El Feidh	2017	51.8%	57.7%	45.8%
Algeria	El Gaada	2000	70.6%	73.3%	67.7%
Algeria	El Gaada	2017	70.9%	73.6%	67.9%
Algeria	El Ghedir	2000	80.7%	83.0%	78.4%
Algeria	El Ghedir	2017	80.8%	83.1%	78.5%
Algeria	El Ghicha	2000	80.7%	83.5%	77.5%
Algeria	El Ghicha	2017	80.8%	83.7%	77.9%
Algeria	El Ghomri	2000	69.5%	71.7%	66.4%
Algeria	El Ghomri	2017	69.7%	71.9%	66.7%
Algeria	El Ghrous	2000	50.3%	53.4%	46.8%
Algeria	El Ghrous	2017	50.7%	53.7%	47.1%
Algeria	El Gor	2000	70.7%	75.8%	65.3%
Algeria	El Gor	2017	70.8%	75.9%	65.3%
Algeria	El Guedid	2000	80.5%	83.4%	77.6%
Algeria	El Guedid	2017	80.6%	83.6%	77.6%
Algeria	El Guelb El Kebir	2000	77.1%	80.7%	73.2%
Algeria	El Guelb El Kebir	2017	77.4%	80.9%	73.5%
Algeria	El Guerrarra	2000	51.7%	56.9%	46.6%
Algeria	El Guerrarra	2017	52.1%	57.2%	46.9%
Algeria	El Guettana	2000	68.9%	71.9%	66.2%
Algeria	El Guettana	2017	69.1%	72.1%	66.3%
Algeria	El Guettar	2000	72.2%	74.5%	69.5%
Algeria	El Guettar	2017	72.6%	75.0%	69.9%
Algeria	El H'Madna	2000	69.7%	72.8%	66.4%
Algeria	El H'Madna	2017	69.9%	73.0%	66.6%
Algeria	El Hacaiba	2000	70.1%	74.1%	66.0%
Algeria	El Hacaiba	2017	70.4%	74.5%	66.2%
Algeria	El Hachem	2000	74.1%	77.5%	70.2%
Algeria	El Hachem	2017	74.3%	77.6%	70.5%
Algeria	El Hachimia	2000	77.6%	80.1%	74.8%
Algeria	El Hachimia	2017	77.9%	80.3%	75.0%
Algeria	El Hadaiek	2000	80.0%	82.4%	77.8%
Algeria	El Hadaiek	2017	80.3%	82.5%	78.0%
Algeria	El Hadjab	2000	48.1%	50.4%	45.5%
Algeria	El Hadjab	2017	48.5%	50.7%	45.9%
Algeria	El Hadjadj	2000	76.1%	79.0%	73.0%
Algeria	El Hadjadj	2017	76.2%	79.1%	73.1%
Algeria	El Hadjar	2000	80.4%	81.9%	78.8%
Algeria	El Hadjar	2017	80.5%	81.9%	78.9%

Country	Second Administrative Unit	Year	Mean	Upper	Lower
Algeria	El Hadjira	2000	48.7%	53.1%	44.3%
Algeria	El Hadjira	2017	48.9%	53.5%	44.3%
Algeria	El Hakimia	2000	77.1%	80.6%	73.4%
Algeria	El Hakimia	2017	77.3%	80.6%	73.5%
Algeria	El Hamadia	2000	69.4%	72.2%	66.7%
Algeria	El Hamadia	2017	69.6%	72.5%	66.9%
Algeria	El Hamdania	2000	77.0%	79.8%	73.8%
Algeria	El Hamdania	2017	77.3%	80.1%	74.2%
Algeria	El Hamma	2000	62.1%	65.0%	59.0%
Algeria	El Hamma	2017	62.3%	65.2%	59.2%
Algeria	El Haouaita	2000	80.6%	84.3%	76.0%
Algeria	El Haouaita	2017	80.7%	84.4%	76.0%
Algeria	El Haouch	2000	48.8%	53.6%	44.6%
Algeria	El Haouch	2017	49.0%	53.8%	44.8%
Algeria	El Harmilia	2000	62.8%	66.1%	59.6%
Algeria	El Harmilia	2017	63.0%	66.3%	59.8%
Algeria	El Harrouch	2000	80.4%	82.2%	78.3%
Algeria	El Harrouch	2017	80.5%	82.3%	78.4%
Algeria	El Hassania	2000	77.4%	80.4%	74.4%
Algeria	El Hassania	2017	77.6%	80.6%	74.6%
Algeria	El Hassasna	2000	78.7%	81.2%	76.3%
Algeria	El Hassasna	2017	79.0%	81.4%	76.6%
Algeria	El Hassi	2000	73.8%	76.5%	70.5%
Algeria	El Hassi	2000	63.9%	67.8%	60.3%
Algeria	El Hassi	2017	74.2%	76.9%	70.9%
Algeria	El Hassi	2017	64.2%	68.0%	60.5%
Algeria	El Houamed	2000	80.1%	82.5%	77.9%
Algeria	El Houamed	2017	80.2%	82.5%	78.1%
Algeria	El Houidjbet	2000	63.4%	68.3%	57.7%
Algeria	El Houidjbet	2017	63.6%	68.5%	58.0%
Algeria	El Idrissia	2000	80.8%	83.3%	78.1%
Algeria	El Idrissia	2017	81.1%	83.6%	78.3%
Algeria	El Kaf Lakhdar	2000	78.4%	82.0%	73.5%
Algeria	El Kaf Lakhdar	2017	78.6%	82.1%	73.7%
Algeria	El Kala	2000	79.6%	82.7%	76.4%
Algeria	El Kala	2017	79.8%	83.0%	76.6%
Algeria	El Karimia	2000	75.5%	78.5%	72.6%
Algeria	El Karimia	2017	75.6%	78.6%	72.6%
Algeria	El Kennar Nouchfi	2000	82.9%	84.9%	80.7%
Algeria	El Kennar Nouchfi	2017	83.0%	85.0%	80.9%
Algeria	El Kentara	2000	52.0%	55.7%	48.8%
Algeria	El Kentara	2017	52.4%	55.9%	49.3%
Algeria	El Kerma	2000	69.8%	72.0%	67.8%
Algeria	El Kerma	2017	70.0%	72.2%	68.1%
Algeria	El Keurt	2000	72.6%	75.0%	70.2%
Algeria	El Keurt	2017	72.8%	75.2%	70.5%
Algeria	El Khabouzia	2000	77.5%	80.4%	74.3%
Algeria	El Khabouzia	2017	77.7%	80.6%	74.4%
Algeria	El Kharrouba	2000	77.0%	79.5%	74.8%
Algeria	El Kharrouba	2017	77.3%	79.8%	75.1%
Algeria	El Kheither	2000	78.0%	80.9%	75.2%
Algeria	El Kheither	2017	78.2%	81.0%	75.4%
Algeria	El Khemis	2000	80.4%	82.8%	77.7%
Algeria	El Khemis	2017	80.6%	82.9%	78.0%
Algeria	El Khroub	2000	76.5%	78.7%	74.3%
Algeria	El Khroub	2017	76.7%	78.9%	74.4%
Algeria	El Kouif	2000	64.3%	68.9%	59.7%
Algeria	El Kouif	2017	64.6%	69.3%	59.9%

Country	Second Administrative Unit	Year	Mean	Upper	Lower
Algeria	El Kseur	2000	74.2%	77.7%	70.5%
Algeria	El Kseur	2017	74.2%	77.7%	70.5%
Algeria	El M'Ghair	2000	49.7%	52.7%	46.6%
Algeria	El M'Ghair	2017	50.0%	53.1%	46.9%
Algeria	El M'Hir	2000	73.4%	77.6%	69.3%
Algeria	El M'Hir	2017	73.2%	77.7%	69.2%
Algeria	El Madher	2000	63.8%	66.9%	61.0%
Algeria	El Madher	2017	64.1%	67.1%	61.2%
Algeria	El Mahmal	2000	63.2%	66.7%	59.7%
Algeria	El Mahmal	2017	63.4%	67.0%	59.9%
Algeria	El Main	2000	68.5%	71.8%	64.4%
Algeria	El Main	2017	68.6%	71.9%	64.6%
Algeria	El Maine	2000	77.1%	80.0%	74.1%
Algeria	El Maine	2017	77.0%	80.0%	74.0%
Algeria	El Malabiodh	2000	63.0%	66.8%	59.1%
Algeria	El Malabiodh	2017	63.2%	67.1%	59.1%
Algeria	El Malah	2000	70.3%	72.7%	67.2%
Algeria	El Malah	2017	70.4%	72.9%	67.4%
Algeria	El Mamounia	2000	72.3%	74.8%	69.9%
Algeria	El Mamounia	2017	72.5%	75.0%	70.1%
Algeria	El Marsa	2000	73.0%	77.3%	67.9%
Algeria	El Marsa	2000	80.7%	84.3%	76.9%
Algeria	El Marsa	2017	73.2%	77.6%	68.2%
Algeria	El Marsa	2017	80.9%	84.4%	77.1%
Algeria	El Matmar	2000	70.3%	73.2%	67.2%
Algeria	El Matmar	2017	70.6%	73.4%	67.5%
Algeria	El Matmor	2000	72.1%	74.9%	69.5%
Algeria	El Matmor	2017	72.3%	75.1%	69.7%
Algeria	El Mechira	2000	71.3%	74.1%	68.3%
Algeria	El Mechira	2017	71.3%	74.1%	68.2%
Algeria	El Mehara	2000	78.8%	81.8%	76.0%
Algeria	El Mehara	2017	79.0%	81.9%	76.1%
Algeria	El Menaouer	2000	73.3%	76.6%	69.5%
Algeria	El Menaouer	2017	73.5%	76.8%	69.7%
Algeria	El Meniaa	2000	50.1%	54.4%	46.1%
Algeria	El Meniaa	2017	50.4%	54.7%	46.5%
Algeria	El Meridj	2000	67.8%	73.1%	62.7%
Algeria	El Meridj	2017	67.8%	73.2%	62.8%
Algeria	El Messaid	2000	69.9%	73.7%	65.6%
Algeria	El Messaid	2017	70.1%	73.9%	65.8%
Algeria	El Mezeraa	2000	62.4%	66.4%	58.3%
Algeria	El Mezeraa	2017	62.7%	66.9%	58.8%
Algeria	El Milia	2000	82.6%	85.0%	80.1%
Algeria	El Milia	2017	82.7%	85.1%	80.1%
Algeria	El Mokrani|El Madjen	2000	77.0%	80.1%	73.8%
Algeria	El Mokrani|El Madjen	2017	77.1%	80.1%	73.9%
Algeria	El Ogla	2000	62.7%	67.3%	58.0%
Algeria	El Ogla	2000	48.4%	52.6%	44.3%
Algeria	El Ogla	2017	63.1%	67.7%	58.4%
Algeria	El Ogla	2017	48.6%	52.8%	44.5%
Algeria	El Ouata	2000	49.5%	56.1%	43.9%
Algeria	El Ouata	2017	49.3%	55.0%	44.0%
Algeria	El Oued	2000	48.4%	51.3%	46.1%
Algeria	El Oued	2017	48.7%	51.6%	46.4%
Algeria	El Oueldja	2000	59.5%	65.0%	53.7%
Algeria	El Oueldja	2000	72.5%	74.9%	70.0%
Algeria	El Oueldja	2000	69.3%	72.3%	66.2%
Algeria	El Oueldja	2017	72.4%	74.8%	69.9%
Algeria	El Oueldja	2017	69.9%	72.9%	66.9%
Algeria	El Oueldja	2017	59.4%	64.9%	53.6%

Country	Second Administrative Unit	Year	Mean	Upper	Lower
Algeria	El Ouinet	2000	66.7%	70.3%	62.6%
Algeria	El Ouinet	2000	78.6%	82.3%	74.8%
Algeria	El Ouinet	2017	78.9%	82.5%	75.0%
Algeria	El Ouinet	2017	66.9%	70.5%	62.8%
Algeria	El Ouitaya	2000	49.3%	52.0%	46.4%
Algeria	El Ouitaya	2017	49.5%	52.2%	46.5%
Algeria	El Oumaria	2000	76.7%	80.3%	72.8%
Algeria	El Oumaria	2017	76.5%	80.3%	72.5%
Algeria	El Ouricia	2000	62.9%	65.1%	60.4%
Algeria	El Ouricia	2017	63.0%	65.2%	60.4%
Algeria	El Rahia	2000	63.5%	68.0%	57.9%
Algeria	El Rahia	2017	63.6%	68.2%	58.0%
Algeria	El Tarf	2000	79.7%	82.5%	76.9%
Algeria	El Tarf	2017	79.7%	82.6%	77.0%
Algeria	El Youssoufia	2000	78.6%	81.3%	76.2%
Algeria	El Youssoufia	2017	78.7%	81.4%	76.2%
Algeria	El-Affroun	2000	75.3%	78.0%	72.4%
Algeria	El-Affroun	2017	75.7%	78.4%	72.8%
Algeria	Elayadi Barbes	2000	76.3%	79.3%	73.4%
Algeria	Elayadi Barbes	2017	76.5%	79.4%	73.6%
Algeria	Emir Abdelkader	2000	80.9%	82.6%	79.2%
Algeria	Emir Abdelkader	2000	68.5%	71.9%	64.7%
Algeria	Emir Abdelkader	2017	68.6%	72.0%	64.9%
Algeria	Emir Abdelkader	2017	81.0%	82.7%	79.2%
Algeria	Emjez Edchich	2000	80.3%	82.1%	78.4%
Algeria	Emjez Edchich	2017	80.6%	82.3%	78.7%
Algeria	Ensigha	2000	62.9%	65.3%	60.5%
Algeria	Ensigha	2017	62.9%	65.3%	60.4%
Algeria	Erg Ferradj	2000	49.3%	54.7%	43.7%
Algeria	Erg Ferradj	2017	49.7%	55.3%	43.6%
Algeria	Erraguene	2000	74.2%	77.4%	71.1%
Algeria	Erraguene	2017	74.3%	77.5%	71.2%
Algeria	Es Sebt	2000	80.6%	83.0%	78.2%
Algeria	Es Sebt	2017	80.7%	83.1%	78.3%
Algeria	Es Senia	2000	69.9%	72.0%	68.0%
Algeria	Es Senia	2017	70.0%	72.1%	68.1%
Algeria	Faidh El Botma	2000	80.2%	83.7%	76.2%
Algeria	Faidh El Botma	2017	80.4%	83.9%	76.4%
Algeria	Faidja	2000	79.3%	82.4%	76.3%
Algeria	Faidja	2017	79.4%	82.5%	76.4%
Algeria	Fellaoucene	2000	68.0%	71.3%	63.9%
Algeria	Fellaoucene	2017	68.3%	71.6%	64.3%
Algeria	Fenoughil	2000	48.7%	54.7%	42.1%
Algeria	Fenoughil	2017	48.6%	54.2%	42.8%
Algeria	Feraoun	2000	73.2%	76.2%	70.0%
Algeria	Feraoun	2017	73.5%	76.5%	70.3%
Algeria	Ferdjioua	2000	77.1%	79.8%	74.9%
Algeria	Ferdjioua	2017	77.2%	79.8%	74.9%
Algeria	Ferkane	2000	57.1%	63.7%	51.4%
Algeria	Ferkane	2017	57.2%	63.8%	51.4%
Algeria	Ferraguig	2000	71.0%	73.1%	68.3%
Algeria	Ferraguig	2017	71.1%	73.2%	68.4%

Country	Second Administrative Unit	Year	Mean	Upper	Lower
Algeria	Fesdis	2000	62.0%	64.8%	59.2%
Algeria	Fesdis	2017	62.3%	65.1%	59.6%
Algeria	Filfila	2000	80.3%	83.1%	77.4%
Algeria	Filfila	2017	80.3%	83.2%	77.4%
Algeria	Fkirina	2000	62.4%	66.5%	58.5%
Algeria	Fkirina	2017	62.4%	66.6%	58.5%
Algeria	Foggaret Azzouia	2000	47.1%	54.0%	40.6%
Algeria	Foggaret Azzouia	2017	46.9%	54.1%	40.0%
Algeria	Fornaka	2000	69.5%	72.1%	66.6%
Algeria	Fornaka	2017	69.7%	72.3%	66.9%
Algeria	Foughala	2000	50.0%	53.4%	46.4%
Algeria	Foughala	2017	50.1%	53.5%	46.5%
Algeria	Fouka	2000	75.4%	77.9%	72.9%
Algeria	Fouka	2017	75.6%	78.1%	73.1%
Algeria	Foum Toub	2000	62.3%	66.4%	58.5%
Algeria	Foum Toub	2017	62.3%	66.3%	58.5%
Algeria	Freha	2000	76.7%	79.2%	74.2%
Algeria	Freha	2017	76.9%	79.4%	74.4%
Algeria	Frenda	2000	78.5%	81.0%	76.1%
Algeria	Frenda	2017	78.8%	81.3%	76.4%
Algeria	Frikat	2000	76.9%	79.7%	74.0%
Algeria	Frikat	2017	76.8%	79.6%	74.0%
Algeria	Froha	2000	71.6%	74.0%	69.0%
Algeria	Froha	2017	71.8%	74.2%	69.3%
Algeria	Gdyel	2000	70.7%	73.4%	68.0%
Algeria	Gdyel	2017	70.8%	73.5%	68.2%
Algeria	Ghardaia	2000	49.8%	53.6%	46.7%
Algeria	Ghardaia	2017	50.1%	54.0%	46.9%
Algeria	Gharrous	2000	75.6%	78.9%	72.0%
Algeria	Gharrous	2017	75.8%	79.1%	72.2%
Algeria	Ghassoul	2000	78.7%	82.5%	74.7%
Algeria	Ghassoul	2017	78.8%	82.6%	74.7%
Algeria	Ghazaouet	2000	67.3%	72.2%	62.9%
Algeria	Ghazaouet	2017	67.5%	72.3%	63.0%
Algeria	Ghebala	2000	83.3%	85.7%	80.8%
Algeria	Ghebala	2017	83.3%	85.8%	80.8%
Algeria	Gherouaou	2000	75.6%	77.9%	73.0%
Algeria	Gherouaou	2017	75.7%	78.0%	73.1%
Algeria	Ghessira	2000	58.8%	63.1%	54.6%
Algeria	Ghessira	2017	58.8%	63.2%	54.8%
Algeria	Ghilassa	2000	73.5%	76.7%	70.2%
Algeria	Ghilassa	2017	73.6%	76.9%	70.3%
Algeria	Ghriss	2000	73.2%	76.1%	70.4%
Algeria	Ghriss	2017	73.6%	76.4%	70.9%
Algeria	Gosbat	2000	65.8%	68.6%	62.4%
Algeria	Gosbat	2017	66.1%	68.9%	62.7%
Algeria	Gouraya	2000	74.9%	80.2%	69.2%
Algeria	Gouraya	2017	75.0%	80.3%	69.2%
Algeria	Grarem Gouga	2000	81.8%	84.1%	79.3%
Algeria	Grarem Gouga	2017	81.8%	84.2%	79.3%
Algeria	Guellal	2000	61.1%	64.1%	57.9%
Algeria	Guellal	2017	61.3%	64.3%	58.1%
Algeria	Guelma	2000	78.0%	80.4%	75.8%
Algeria	Guelma	2017	78.3%	80.7%	76.1%
Algeria	Guelta Zerka	2000	64.1%	66.5%	61.5%
Algeria	Guelta Zerka	2017	64.4%	66.7%	61.7%
Algeria	Gueltat Sidi Saad	2000	80.1%	83.3%	76.8%

Country	Second Administrative Unit	Year	Mean	Upper	Lower
Algeria	Gueltat Sidi Saad	2017	80.3%	83.4%	77.0%
Algeria	Guemar	2000	48.1%	51.4%	45.1%
Algeria	Guemar	2017	48.4%	51.7%	45.2%
Algeria	Guenzet Tassameurt	2000	67.2%	70.5%	63.7%
Algeria	Guenzet Tassameurt	2017	67.5%	70.8%	64.1%
Algeria	Guerdjoum	2000	72.5%	75.3%	69.7%
Algeria	Guerdjoum	2017	72.7%	75.4%	69.9%
Algeria	Guernini	2000	81.0%	83.7%	78.1%
Algeria	Guernini	2017	81.2%	83.9%	78.3%
Algeria	Guerrouma	2000	77.5%	80.9%	74.3%
Algeria	Guerrouma	2017	77.7%	81.2%	74.5%
Algeria	Guertoufa	2000	78.5%	80.2%	76.6%
Algeria	Guertoufa	2017	78.5%	80.3%	76.7%
Algeria	Guettara	2000	62.6%	68.3%	57.7%
Algeria	Guettara	2017	64.5%	69.7%	59.9%
Algeria	Guidjel	2000	60.7%	63.3%	58.0%
Algeria	Guidjel	2017	60.9%	63.6%	58.2%
Algeria	Guiga	2000	62.9%	66.1%	59.5%
Algeria	Guiga	2017	63.2%	66.4%	59.8%
Algeria	Guorriguer	2000	61.9%	66.2%	57.9%
Algeria	Guorriguer	2017	62.1%	66.4%	58.1%
Algeria	Hacine	2000	68.8%	71.5%	66.1%
Algeria	Hacine	2017	69.0%	71.7%	66.2%
Algeria	Had Echkalla	2000	75.2%	78.1%	72.3%
Algeria	Had Echkalla	2017	75.1%	77.9%	72.0%
Algeria	Had Sahary	2000	81.7%	84.8%	78.7%
Algeria	Had Sahary	2017	81.9%	85.0%	78.9%
Algeria	Haddada	2000	78.0%	82.1%	73.0%
Algeria	Haddada	2017	78.3%	82.4%	73.4%
Algeria	Hadj Mechri	2000	79.5%	83.2%	75.8%
Algeria	Hadj Mechri	2017	79.8%	83.3%	76.1%
Algeria	Hadjadj	2000	71.5%	75.2%	67.6%
Algeria	Hadjadj	2017	71.8%	75.5%	68.0%
Algeria	Hadjera Zerga	2000	79.6%	82.6%	76.6%
Algeria	Hadjera Zerga	2017	79.8%	82.7%	76.8%
Algeria	Hadjout	2000	75.0%	78.7%	71.7%
Algeria	Hadjout	2017	75.2%	78.9%	71.9%
Algeria	Hadjret Ennous	2000	75.3%	80.8%	69.5%
Algeria	Hadjret Ennous	2017	75.4%	81.0%	69.7%
Algeria	Haizer	2000	76.8%	80.0%	73.6%
Algeria	Haizer	2017	76.9%	80.1%	73.6%
Algeria	Hamadi Krouma	2000	80.0%	82.3%	77.5%
Algeria	Hamadi Krouma	2017	80.1%	82.4%	77.7%
Algeria	Hamadia	2000	79.2%	81.6%	76.9%
Algeria	Hamadia	2017	79.4%	81.7%	77.0%
Algeria	Hamala	2000	83.1%	85.5%	80.6%
Algeria	Hamala	2017	83.3%	85.7%	80.8%
Algeria	Hamma	2000	68.8%	71.7%	65.8%
Algeria	Hamma	2017	69.1%	72.0%	66.0%
Algeria	Hamma Bouziane	2000	80.6%	82.6%	78.9%
Algeria	Hamma Bouziane	2017	80.8%	82.8%	79.1%
Algeria	Hammadi	2000	75.5%	77.0%	73.9%
Algeria	Hammadi	2017	75.7%	77.2%	74.1%

Country	Second Administrative Unit	Year	Mean	Upper	Lower
Algeria	Hammam Ben Salah	2000	80.6%	83.3%	77.7%
Algeria	Hammam Ben Salah	2017	80.9%	83.6%	78.0%
Algeria	Hammam Boughrara	2000	67.6%	70.7%	64.6%
Algeria	Hammam Boughrara	2017	67.9%	71.0%	64.9%
Algeria	Hammam Bouhadjar	2000	71.3%	73.8%	68.5%
Algeria	Hammam Bouhadjar	2017	71.5%	74.0%	68.8%
Algeria	Hammam Dalaa	2000	76.7%	80.1%	72.8%
Algeria	Hammam Dalaa	2017	76.8%	80.3%	73.0%
Algeria	Hammam Debagh	2000	79.7%	82.0%	77.3%
Algeria	Hammam Debagh	2017	79.8%	82.1%	77.3%
Algeria	Hammam Guergour	2000	64.6%	67.3%	61.9%
Algeria	Hammam Guergour	2017	64.8%	67.5%	62.0%
Algeria	Hammam Melouane	2000	76.9%	80.0%	74.0%
Algeria	Hammam Melouane	2017	76.9%	80.0%	74.1%
Algeria	Hammam N'Bail	2000	79.8%	82.5%	77.0%
Algeria	Hammam N'Bail	2017	80.0%	82.7%	77.3%
Algeria	Hammam Righa	2000	76.5%	78.8%	74.1%
Algeria	Hammam Righa	2017	76.7%	79.0%	74.3%
Algeria	Hammam Soukhna	2000	63.3%	67.1%	60.1%
Algeria	Hammam Soukhna	2017	63.5%	67.4%	60.2%
Algeria	Hammamet	2000	62.6%	66.0%	59.2%
Algeria	Hammamet	2017	63.0%	66.3%	59.5%
Algeria	Hamraia	2000	49.5%	54.9%	45.0%
Algeria	Hamraia	2017	49.8%	55.1%	45.3%
Algeria	Hamri	2000	70.1%	73.0%	67.1%
Algeria	Hamri	2000	81.4%	83.7%	78.7%
Algeria	Hamri	2017	70.2%	73.1%	67.2%
Algeria	Hamri	2017	81.5%	83.8%	78.9%
Algeria	Hanchir Toumghani	2000	62.9%	66.1%	59.5%
Algeria	Hanchir Toumghani	2017	63.1%	66.4%	59.7%
Algeria	Hanencha	2000	80.4%	82.4%	78.4%
Algeria	Hanencha	2017	80.6%	82.6%	78.6%
Algeria	Hannacha	2000	76.8%	80.2%	73.1%
Algeria	Hannacha	2017	77.1%	80.4%	73.3%
Algeria	Haraoua	2000	75.6%	77.5%	73.5%
Algeria	Haraoua	2017	75.8%	77.7%	73.7%
Algeria	Haraza	2000	75.8%	79.8%	71.4%
Algeria	Haraza	2017	75.8%	79.8%	71.4%
Algeria	Harbil	2000	66.2%	69.0%	63.5%
Algeria	Harbil	2017	66.4%	69.1%	63.7%

Country	Second Administrative Unit	Year	Mean	Upper	Lower
Algeria	Harchoune	2000	75.7%	78.1%	73.1%
Algeria	Harchoune	2017	75.8%	78.3%	73.2%
Algeria	Hasnaoua	2000	63.8%	66.4%	60.8%
Algeria	Hasnaoua	2017	64.0%	66.6%	61.1%
Algeria	Hassaine|Beni Yahi	2000	69.4%	71.7%	66.5%
Algeria	Hassaine|Beni Yahi	2017	69.6%	71.9%	66.7%
Algeria	Hassani Abdelkrim	2000	48.6%	51.7%	45.8%
Algeria	Hassani Abdelkrim	2017	48.9%	52.0%	46.1%
Algeria	Hassasna	2000	70.9%	74.3%	67.5%
Algeria	Hassasna	2017	71.1%	74.5%	67.7%
Algeria	Hassi Bahbah	2000	80.8%	82.7%	78.8%
Algeria	Hassi Bahbah	2017	80.9%	82.8%	78.9%
Algeria	Hassi Ben Abdellah	2000	48.6%	52.0%	45.4%
Algeria	Hassi Ben Abdellah	2017	48.9%	52.1%	45.8%
Algeria	Hassi Ben Okba	2000	70.4%	72.9%	67.7%
Algeria	Hassi Ben Okba	2017	70.8%	73.3%	68.2%
Algeria	Hassi Bounif	2000	70.3%	72.6%	68.2%
Algeria	Hassi Bounif	2017	70.6%	72.9%	68.4%
Algeria	Hassi Dahou	2000	71.5%	74.8%	68.3%
Algeria	Hassi Dahou	2017	71.6%	75.0%	68.5%
Algeria	Hassi Delaa	2000	78.9%	81.8%	76.0%
Algeria	Hassi Delaa	2017	78.5%	81.3%	75.6%
Algeria	Hassi El Euch	2000	81.5%	83.9%	79.5%
Algeria	Hassi El Euch	2017	81.6%	83.9%	79.6%
Algeria	Hassi El Ghella	2000	70.5%	73.1%	67.5%
Algeria	Hassi El Ghella	2017	70.6%	73.2%	67.5%
Algeria	Hassi Fedoul	2000	79.1%	81.9%	76.3%
Algeria	Hassi Fedoul	2017	79.3%	81.9%	76.6%
Algeria	Hassi Fehal	2000	48.9%	55.4%	42.9%
Algeria	Hassi Fehal	2017	49.3%	55.7%	43.7%
Algeria	Hassi Gara	2000	49.9%	53.7%	46.0%
Algeria	Hassi Gara	2017	50.0%	54.2%	45.6%
Algeria	Hassi Khalifa	2000	48.8%	53.7%	44.1%
Algeria	Hassi Khalifa	2017	49.0%	54.0%	44.3%
Algeria	Hassi Mameche	2000	71.1%	73.2%	69.3%
Algeria	Hassi Mameche	2017	71.3%	73.4%	69.5%
Algeria	Hassi Mefsoukh	2000	70.0%	72.6%	67.8%
Algeria	Hassi Mefsoukh	2017	70.3%	72.8%	68.0%
Algeria	Hassi Messaoud	2000	48.9%	51.9%	45.6%
Algeria	Hassi Messaoud	2017	49.1%	51.9%	46.2%
Algeria	Hassi R'Mel	2000	77.3%	80.1%	74.0%
Algeria	Hassi R'Mel	2017	77.5%	80.4%	74.2%
Algeria	Hassi Zehana	2000	70.7%	74.7%	67.0%
Algeria	Hassi Zehana	2017	70.9%	74.9%	67.2%
Algeria	Hattatba	2000	75.5%	78.1%	73.1%
Algeria	Hattatba	2017	75.7%	78.3%	73.3%

Country	Second Administrative Unit	Year	Mean	Upper	Lower
Algeria	Helliopolis	2000	78.6%	80.7%	76.1%
Algeria	Helliopolis	2017	79.0%	81.1%	76.6%
Algeria	Hennaya	2000	69.1%	71.8%	66.3%
Algeria	Hennaya	2017	69.4%	72.1%	66.6%
Algeria	Herenfa	2000	74.4%	77.4%	71.0%
Algeria	Herenfa	2017	74.3%	77.4%	71.0%
Algeria	Hidoussa	2000	62.7%	65.9%	59.3%
Algeria	Hidoussa	2017	63.1%	66.2%	59.6%
Algeria	Hoceinia	2000	76.2%	78.8%	73.3%
Algeria	Hoceinia	2017	76.2%	78.9%	73.4%
Algeria	Honaine	2000	67.2%	71.6%	62.5%
Algeria	Honaine	2017	67.1%	71.7%	62.3%
Algeria	Houari Boumediene	2000	79.3%	82.1%	76.7%
Algeria	Houari Boumediene	2017	79.5%	82.1%	76.8%
Algeria	Hounet	2000	73.2%	76.6%	69.7%
Algeria	Hounet	2017	73.4%	76.8%	69.8%
Algeria	Ibn Ziad	2000	80.4%	82.7%	78.5%
Algeria	Ibn Ziad	2017	80.4%	82.6%	78.5%
Algeria	Iboudraren	2000	77.0%	80.0%	73.5%
Algeria	Iboudraren	2017	77.1%	80.1%	73.7%
Algeria	Ichmoul	2000	62.5%	67.2%	58.3%
Algeria	Ichmoul	2017	62.6%	67.4%	58.4%
Algeria	Idjeur	2000	78.7%	81.3%	75.8%
Algeria	Idjeur	2017	78.8%	81.5%	75.9%
Algeria	Idles	2000	50.6%	57.0%	44.2%
Algeria	Idles	2017	50.8%	56.7%	44.6%
Algeria	Ifelain Ilmathen	2000	74.3%	77.6%	70.7%
Algeria	Ifelain Ilmathen	2017	74.2%	77.5%	70.6%
Algeria	Iferhounene	2000	77.6%	80.4%	74.5%
Algeria	Iferhounene	2017	77.7%	80.5%	74.5%
Algeria	Ifigha	2000	77.3%	79.5%	74.8%
Algeria	Ifigha	2017	77.5%	79.7%	75.0%
Algeria	Iflissen	2000	78.3%	81.7%	74.2%
Algeria	Iflissen	2017	78.6%	82.0%	74.5%
Algeria	Ighil-Ali	2000	72.0%	75.8%	68.2%
Algeria	Ighil-Ali	2017	72.3%	76.1%	68.5%
Algeria	Ighrem	2000	74.8%	77.7%	71.8%
Algeria	Ighrem	2017	74.7%	77.7%	71.7%
Algeria	Igli	2000	49.5%	57.0%	43.3%
Algeria	Igli	2017	49.7%	56.9%	43.4%
Algeria	Illilten	2000	76.9%	79.8%	73.8%
Algeria	Illilten	2017	76.9%	79.9%	73.8%
Algeria	Illizi	2000	48.8%	56.9%	41.4%
Algeria	Illizi	2017	48.7%	56.9%	41.3%
Algeria	Illoula Oumalou	2000	77.6%	80.0%	74.9%
Algeria	Illoula Oumalou	2017	77.9%	80.3%	75.2%
Algeria	Imsouhal	2000	77.6%	80.1%	74.5%
Algeria	Imsouhal	2017	77.9%	80.3%	74.7%
Algeria	In Amenas	2000	49.9%	57.3%	41.2%
Algeria	In Amenas	2017	50.0%	57.3%	41.1%
Algeria	In Ghar	2000	48.0%	53.3%	42.7%
Algeria	In Ghar	2017	47.9%	52.7%	42.7%
Algeria	In Guezzam	2000	48.5%	58.4%	39.2%
Algeria	In Guezzam	2017	48.5%	58.9%	39.0%

Country	Second Administrative Unit	Year	Mean	Upper	Lower
Algeria	In M'Guel	2000	48.1%	54.3%	41.4%
Algeria	In M'Guel	2017	48.0%	53.6%	41.7%
Algeria	In Salah	2000	49.8%	54.6%	45.2%
Algeria	In Salah	2017	50.0%	54.4%	45.4%
Algeria	In Zghmir	2000	49.0%	56.3%	43.2%
Algeria	In Zghmir	2017	48.6%	55.3%	42.9%
Algeria	Inoughissen	2000	63.1%	67.6%	58.4%
Algeria	Inoughissen	2017	63.4%	67.7%	58.6%
Algeria	Irdjen	2000	76.4%	78.6%	74.0%
Algeria	Irdjen	2017	76.5%	78.7%	74.1%
Algeria	Isser	2000	75.5%	78.0%	73.1%
Algeria	Isser	2017	75.7%	78.2%	73.3%
Algeria	Jijel	2000	79.6%	82.4%	76.4%
Algeria	Jijel	2017	79.8%	82.5%	76.6%
Algeria	Kadiria	2000	76.5%	79.1%	73.8%
Algeria	Kadiria	2017	76.4%	79.1%	73.7%
Algeria	Kais	2000	60.0%	63.3%	56.3%
Algeria	Kais	2017	60.2%	63.5%	56.4%
Algeria	Kalaa	2000	72.2%	74.9%	68.8%
Algeria	Kalaa	2017	72.1%	74.9%	68.9%
Algeria	Kalaat Bousbaa	2000	80.9%	83.0%	78.4%
Algeria	Kalaat Bousbaa	2017	81.1%	83.2%	78.6%
Algeria	Kanoua	2000	83.7%	87.6%	79.7%
Algeria	Kanoua	2017	84.1%	88.0%	80.2%
Algeria	Kasdir	2000	75.3%	79.1%	70.6%
Algeria	Kasdir	2017	76.1%	79.8%	71.1%
Algeria	Kef El Ahmar	2000	78.9%	82.6%	75.5%
Algeria	Kef El Ahmar	2017	79.0%	82.7%	75.6%
Algeria	Kenadsa	2000	48.7%	53.0%	44.5%
Algeria	Kenadsa	2017	48.8%	53.1%	44.6%
Algeria	Kendira	2000	71.3%	74.5%	68.6%
Algeria	Kendira	2017	71.4%	74.6%	68.7%
Algeria	Kerkera	2000	80.8%	83.4%	77.6%
Algeria	Kerkera	2017	81.0%	83.6%	77.7%
Algeria	Kerzaz	2000	49.8%	57.0%	42.9%
Algeria	Kerzaz	2017	49.7%	55.8%	43.7%
Algeria	Khadra	2000	70.5%	74.9%	65.7%
Algeria	Khadra	2017	70.7%	75.1%	65.9%
Algeria	Khalouia	2000	73.1%	75.8%	70.1%
Algeria	Khalouia	2017	73.3%	76.0%	70.3%
Algeria	Khams Djouamaa	2000	77.1%	80.7%	73.2%
Algeria	Khams Djouamaa	2017	77.4%	81.0%	73.5%
Algeria	Khatouti Sed Eldjir	2000	81.1%	83.9%	77.9%
Algeria	Khatouti Sed Eldjir	2017	81.2%	84.0%	78.0%
Algeria	Khedara	2000	79.7%	83.5%	75.0%
Algeria	Khedara	2017	79.8%	83.6%	75.1%
Algeria	Kheir Oued Adjoul	2000	81.9%	84.7%	79.0%
Algeria	Kheir Oued Adjoul	2017	82.2%	84.9%	79.2%
Algeria	Kheiredine	2000	71.5%	73.6%	69.0%
Algeria	Kheiredine	2017	71.7%	73.9%	69.2%
Algeria	Khelil	2000	62.2%	64.7%	59.3%
Algeria	Khelil	2017	62.2%	64.8%	59.3%
Algeria	Khemis El Khechna	2000	75.7%	77.6%	73.7%

Country	Second Administrative Unit	Year	Mean	Upper	Lower
Algeria	Khemis El Khechna	2017	75.9%	77.8%	73.9%
Algeria	Khemis Miliana	2000	76.4%	78.9%	73.2%
Algeria	Khemis Miliana	2017	76.6%	79.0%	73.4%
Algeria	Khemissa	2000	77.7%	81.0%	73.9%
Algeria	Khemissa	2017	77.8%	81.2%	74.2%
Algeria	Khemisti	2000	78.7%	80.9%	76.0%
Algeria	Khemisti	2017	78.9%	81.1%	76.2%
Algeria	Khenchela	2000	63.6%	66.2%	61.2%
Algeria	Khenchela	2017	63.8%	66.3%	61.4%
Algeria	Kheneg	2000	82.2%	84.5%	80.1%
Algeria	Kheneg	2017	82.3%	84.6%	80.2%
Algeria	Kheng Maoun	2000	83.0%	86.3%	79.4%
Algeria	Kheng Maoun	2017	83.2%	86.5%	79.6%
Algeria	Khenguet Sidi Nadji	2000	55.9%	62.0%	49.3%
Algeria	Khenguet Sidi Nadji	2017	56.0%	62.2%	49.4%
Algeria	Kherrata	2000	70.0%	73.4%	66.4%
Algeria	Kherrata	2017	70.3%	73.6%	66.7%
Algeria	Khezzara	2000	78.2%	81.3%	75.0%
Algeria	Khezzara	2017	78.3%	81.4%	75.0%
Algeria	Khirane	2000	63.2%	68.1%	58.3%
Algeria	Khirane	2017	63.6%	68.5%	58.8%
Algeria	Khoubana	2000	79.9%	82.9%	76.6%
Algeria	Khoubana	2017	80.0%	83.0%	76.7%
Algeria	Khraicia	2000	75.7%	77.6%	74.0%
Algeria	Khraicia	2017	75.9%	77.8%	74.2%
Algeria	Kimmel	2000	60.1%	64.5%	55.6%
Algeria	Kimmel	2017	60.2%	64.6%	55.7%
Algeria	Kolea	2000	75.4%	77.5%	73.2%
Algeria	Kolea	2017	75.6%	77.7%	73.4%
Algeria	Kouas	2000	80.2%	82.0%	77.9%
Algeria	Kouas	2017	80.4%	82.2%	78.2%
Algeria	Kouba	2000	75.6%	77.3%	73.9%
Algeria	Kouba	2017	75.8%	77.5%	74.1%
Algeria	Kouinine	2000	48.3%	50.8%	45.9%
Algeria	Kouinine	2017	48.5%	51.1%	46.2%
Algeria	Krakda	2000	78.8%	82.2%	74.8%
Algeria	Krakda	2017	78.9%	82.1%	75.2%
Algeria	Ksabi	2000	49.5%	58.8%	40.8%
Algeria	Ksabi	2017	49.5%	58.3%	40.8%
Algeria	Ksar Bellezma	2000	62.5%	65.7%	59.2%
Algeria	Ksar Bellezma	2017	62.7%	65.7%	59.3%
Algeria	Ksar Chellala	2000	79.2%	81.5%	76.8%
Algeria	Ksar Chellala	2017	79.4%	81.7%	77.1%
Algeria	Ksar El Abtal	2000	63.2%	66.2%	59.6%
Algeria	Ksar El Abtal	2017	63.3%	66.4%	59.8%
Algeria	Ksar El Boukhari	2000	76.6%	80.2%	72.9%
Algeria	Ksar El Boukhari	2017	76.8%	80.4%	73.2%
Algeria	Ksar El Sbihi	2000	71.3%	75.4%	67.2%
Algeria	Ksar El Sbihi	2017	71.3%	75.5%	67.2%
Algeria	Ksar Hirane	2000	81.7%	84.1%	78.6%
Algeria	Ksar Hirane	2017	82.1%	84.4%	79.0%
Algeria	Ksar Kaddour	2000	50.0%	55.0%	44.4%
Algeria	Ksar Kaddour	2017	50.8%	55.8%	45.1%
Algeria	Ksour	2000	71.0%	74.1%	67.8%
Algeria	Ksour	2017	71.1%	74.1%	67.9%

Country	Second Administrative Unit	Year	Mean	Upper	Lower
Algeria	Labiod Medjadja	2000	76.9%	79.4%	74.1%
Algeria	Labiod Medjadja	2017	77.0%	79.5%	74.3%
Algeria	Lac Des Oiseaux	2000	79.7%	83.0%	76.6%
Algeria	Lac Des Oiseaux	2017	79.8%	83.2%	76.7%
Algeria	Laghouat	2000	82.7%	84.3%	80.9%
Algeria	Laghouat	2017	82.9%	84.5%	81.1%
Algeria	Lahlef	2000	71.1%	73.5%	68.8%
Algeria	Lahlef	2017	71.4%	73.7%	69.0%
Algeria	Lahmar	2000	49.3%	55.1%	43.8%
Algeria	Lahmar	2017	49.8%	55.5%	44.4%
Algeria	Lakhdaria	2000	76.2%	78.9%	73.5%
Algeria	Lakhdaria	2017	76.4%	79.1%	73.7%
Algeria	Larbaa	2000	62.1%	65.9%	58.5%
Algeria	Larbaa	2000	75.5%	78.1%	72.7%
Algeria	Larbaa	2000	78.1%	81.0%	75.5%
Algeria	Larbaa	2017	78.0%	80.6%	75.4%
Algeria	Larbaa	2017	75.7%	77.9%	73.2%
Algeria	Larbaa	2017	62.5%	66.4%	58.9%
Algeria	Larbaa-Nath-Irathen	2000	78.8%	81.0%	76.5%
Algeria	Larbaa-Nath-Irathen	2017	79.1%	81.3%	76.8%
Algeria	Larbatache	2000	76.1%	78.3%	73.9%
Algeria	Larbatache	2017	76.4%	78.6%	74.3%
Algeria	Lardjem	2000	77.8%	80.0%	75.5%
Algeria	Lardjem	2017	77.9%	80.3%	75.6%
Algeria	Larhat	2000	75.7%	80.9%	69.8%
Algeria	Larhat	2017	75.7%	80.9%	69.6%
Algeria	Layoune	2000	78.5%	80.9%	76.2%
Algeria	Layoune	2017	78.8%	81.1%	76.3%
Algeria	Lazharia	2000	77.6%	80.6%	74.7%
Algeria	Lazharia	2017	77.8%	80.7%	74.8%
Algeria	Lazrou	2000	65.0%	68.1%	61.6%
Algeria	Lazrou	2017	65.2%	68.4%	61.9%
Algeria	Leghata	2000	75.6%	78.6%	72.5%
Algeria	Leghata	2017	75.8%	78.8%	72.7%
Algeria	Lemsane	2000	62.1%	65.6%	58.4%
Algeria	Lemsane	2017	62.4%	66.0%	58.7%
Algeria	Lemtar	2000	71.1%	74.4%	67.9%
Algeria	Lemtar	2017	71.3%	74.6%	68.1%
Algeria	Les Eucalyptus	2000	75.2%	76.5%	73.7%
Algeria	Les Eucalyptus	2017	75.5%	76.8%	74.1%
Algeria	Lichana	2000	49.2%	52.6%	46.3%
Algeria	Lichana	2017	49.5%	52.8%	46.5%
Algeria	Lioua	2000	49.1%	51.9%	46.0%
Algeria	Lioua	2017	49.3%	52.2%	46.1%
Algeria	M_Ziraa	2000	54.3%	58.8%	49.1%
Algeria	M_Ziraa	2017	54.1%	58.8%	48.9%
Algeria	M’Chedallah	2000	74.1%	78.2%	69.5%
Algeria	M’Chedallah	2017	74.2%	78.4%	69.6%
Algeria	M’Cid	2000	72.0%	75.7%	68.1%
Algeria	M’Cid	2017	72.1%	75.8%	68.3%
Algeria	M’Cif	2000	77.2%	80.7%	73.3%
Algeria	M’Cif	2017	77.1%	80.7%	73.3%
Algeria	M’Daourouche	2000	75.0%	78.0%	71.0%
Algeria	M’Daourouche	2017	74.9%	78.0%	70.9%

Country	Second Administrative Unit	Year	Mean	Upper	Lower
Algeria	M'Doukal	2000	61.3%	66.4%	55.9%
Algeria	M'Doukal	2017	61.3%	66.4%	55.9%
Algeria	M'Kira	2000	76.7%	79.4%	73.9%
Algeria	M'Kira	2017	76.9%	79.6%	74.1%
Algeria	M'Lili	2000	48.4%	51.9%	44.8%
Algeria	M'Lili	2017	48.6%	52.2%	45.0%
Algeria	M'Liliha	2000	81.9%	84.2%	79.4%
Algeria	M'Liliha	2017	82.1%	84.3%	79.6%
Algeria	M'Naguer	2000	48.5%	51.1%	45.5%
Algeria	M'Naguer	2017	48.7%	51.5%	45.7%
Algeria	M'Rara	2000	49.8%	55.4%	43.9%
Algeria	M'Rara	2017	50.3%	56.2%	44.3%
Algeria	M'Sara	2000	63.1%	67.4%	58.3%
Algeria	M'Sara	2017	63.1%	67.5%	58.5%
Algeria	M'Sila	2000	81.6%	83.3%	80.1%
Algeria	M'Sila	2017	81.8%	83.5%	80.3%
Algeria	M'Tarfa	2000	81.3%	82.9%	79.8%
Algeria	M'Tarfa	2017	81.5%	83.1%	80.0%
Algeria	M'Toussa	2000	62.4%	66.2%	58.7%
Algeria	M'Toussa	2017	62.5%	66.2%	58.8%
Algeria	Maacem	2000	79.1%	81.3%	76.9%
Algeria	Maacem	2017	79.2%	81.3%	76.8%
Algeria	Maadid	2000	77.7%	80.0%	75.1%
Algeria	Maadid	2017	78.2%	80.4%	75.6%
Algeria	Maafa	2000	57.9%	60.9%	55.3%
Algeria	Maafa	2017	58.1%	61.1%	55.5%
Algeria	Maala	2000	76.5%	79.7%	73.6%
Algeria	Maala	2017	76.7%	79.9%	73.8%
Algeria	Maamora	2000	77.8%	81.3%	74.1%
Algeria	Maamora	2000	78.4%	81.5%	75.0%
Algeria	Maamora	2017	77.9%	81.5%	74.2%
Algeria	Maamora	2017	78.6%	81.8%	75.2%
Algeria	Maaouia	2000	70.8%	73.7%	67.6%
Algeria	Maaouia	2017	70.8%	73.8%	67.7%
Algeria	Maarif	2000	80.3%	83.0%	77.3%
Algeria	Maarif	2017	80.5%	83.1%	77.4%
Algeria	Maatkas	2000	77.3%	79.7%	74.6%
Algeria	Maatkas	2017	77.3%	79.7%	74.7%
Algeria	Machroha	2000	81.0%	83.7%	78.7%
Algeria	Machroha	2017	81.3%	83.8%	78.9%
Algeria	Madna	2000	78.0%	81.2%	74.2%
Algeria	Madna	2017	78.2%	81.3%	74.4%
Algeria	Maghnia	2000	68.2%	72.0%	65.0%
Algeria	Maghnia	2017	68.5%	72.2%	65.3%
Algeria	Magra	2000	76.4%	78.5%	74.1%
Algeria	Magra	2017	76.6%	78.7%	74.4%
Algeria	Magrane	2000	48.5%	53.8%	44.4%
Algeria	Magrane	2017	48.8%	54.1%	44.7%
Algeria	Magtaa Douz	2000	69.0%	71.4%	66.3%
Algeria	Magtaa Douz	2017	69.1%	71.5%	66.5%
Algeria	Mahdia	2000	78.3%	80.5%	75.8%
Algeria	Mahdia	2017	78.6%	80.8%	76.2%
Algeria	Mahelma	2000	75.5%	77.4%	73.6%
Algeria	Mahelma	2017	75.7%	77.6%	74.0%
Algeria	Makhda	2000	74.6%	78.4%	71.6%
Algeria	Makhda	2017	74.9%	78.6%	71.8%
Algeria	Makman Ben Amer	2000	77.5%	81.4%	73.5%
Algeria	Makman Ben Amer	2017	77.7%	81.4%	73.6%
Algeria	Makouda	2000	78.2%	81.3%	75.1%
Algeria	Makouda	2017	78.3%	81.3%	75.4%

Country	Second Administrative Unit	Year	Mean	Upper	Lower
Algeria	Mansoura	2000	48.8%	54.5%	43.9%
Algeria	Mansoura	2000	71.8%	75.9%	68.2%
Algeria	Mansoura	2017	49.4%	54.7%	44.5%
Algeria	Mansoura	2017	72.1%	76.2%	68.5%
Algeria	Mansourah	2000	71.2%	73.5%	68.8%
Algeria	Mansourah	2000	70.7%	72.6%	68.4%
Algeria	Mansourah	2017	70.8%	72.8%	68.5%
Algeria	Mansourah	2017	71.6%	73.8%	69.2%
Algeria	Maouaklane	2000	65.3%	68.4%	62.1%
Algeria	Maouaklane	2017	65.5%	68.7%	62.4%
Algeria	Maoussa	2000	72.0%	74.3%	69.6%
Algeria	Maoussa	2017	72.2%	74.4%	69.8%
Algeria	Marsa Ben M'Hidi	2000	68.3%	75.0%	61.6%
Algeria	Marsa Ben M'Hidi	2017	68.5%	75.2%	61.9%
Algeria	Marsat El Hadjadj	2000	69.4%	72.3%	65.8%
Algeria	Marsat El Hadjadj	2017	69.6%	72.6%	66.1%
Algeria	Mascara	2000	72.8%	75.2%	70.5%
Algeria	Mascara	2017	73.1%	75.5%	70.7%
Algeria	Mazouna	2000	72.3%	74.8%	69.7%
Algeria	Mazouna	2017	72.7%	75.2%	70.1%
Algeria	Mecheria	2000	77.5%	80.2%	74.5%
Algeria	Mecheria	2017	77.8%	80.4%	74.8%
Algeria	Mechouneche	2000	51.1%	55.3%	47.2%
Algeria	Mechouneche	2017	50.9%	55.0%	47.2%
Algeria	Mechraa Houari Boumediene	2000	49.5%	55.9%	42.9%
Algeria	Mechraa Houari Boumediene	2017	49.5%	56.0%	43.0%
Algeria	Mechraa Safa	2000	78.1%	80.7%	75.8%
Algeria	Mechraa Safa	2017	78.4%	81.0%	76.2%
Algeria	Mechtrass	2000	76.8%	79.9%	73.9%
Algeria	Mechtrass	2017	77.0%	80.0%	74.1%
Algeria	Medea	2000	76.5%	79.5%	73.0%
Algeria	Medea	2017	76.7%	79.7%	73.1%
Algeria	Mediouna	2000	71.4%	74.2%	68.9%
Algeria	Mediouna	2017	71.6%	74.3%	69.1%
Algeria	Medjana	2000	65.6%	68.1%	62.9%
Algeria	Medjana	2017	65.9%	68.4%	63.1%
Algeria	Medjaz Ammar	2000	78.7%	81.1%	76.2%
Algeria	Medjaz Ammar	2017	78.7%	81.0%	76.2%
Algeria	Medjaz Sfa	2000	79.8%	81.9%	77.1%
Algeria	Medjaz Sfa	2017	79.9%	82.0%	77.2%
Algeria	Medjebar	2000	77.2%	80.4%	73.9%
Algeria	Medjebar	2017	77.2%	80.6%	74.0%
Algeria	Medjedel	2000	81.2%	83.4%	78.4%
Algeria	Medjedel	2017	81.2%	83.5%	78.6%
Algeria	Medrissa	2000	78.9%	81.5%	76.2%
Algeria	Medrissa	2017	79.1%	81.7%	76.4%
Algeria	Medroussa	2000	78.3%	80.8%	76.2%
Algeria	Medroussa	2017	78.6%	81.1%	76.4%
Algeria	Meftah	2000	75.8%	78.0%	73.5%
Algeria	Meftah	2017	75.9%	78.0%	73.7%

Country	Second Administrative Unit	Year	Mean	Upper	Lower
Algeria	Meftaha	2000	77.1%	80.2%	73.9%
Algeria	Meftaha	2017	77.4%	80.5%	74.1%
Algeria	Megarine	2000	49.2%	52.7%	45.6%
Algeria	Megarine	2017	49.4%	53.0%	45.9%
Algeria	Megheraoua	2000	76.8%	80.2%	73.2%
Algeria	Megheraoua	2017	77.0%	80.3%	73.4%
Algeria	Meghila	2000	78.1%	80.3%	75.5%
Algeria	Meghila	2017	78.3%	80.5%	75.7%
Algeria	Mekhadma	2000	48.6%	52.0%	45.1%
Algeria	Mekhadma	2017	48.8%	52.3%	45.4%
Algeria	Mekhareg	2000	82.4%	84.2%	80.3%
Algeria	Mekhareg	2017	82.6%	84.4%	80.5%
Algeria	Mekhatria	2000	76.2%	79.1%	73.1%
Algeria	Mekhatria	2017	76.2%	79.1%	73.2%
Algeria	Mekkedra	2000	71.1%	74.3%	68.0%
Algeria	Mekkedra	2017	71.2%	74.4%	68.2%
Algeria	Mekla	2000	76.9%	79.1%	74.6%
Algeria	Mekla	2017	77.0%	79.2%	74.8%
Algeria	Melaab	2000	78.1%	80.8%	74.9%
Algeria	Melaab	2017	78.3%	80.9%	75.1%
Algeria	Melbou	2000	73.5%	77.0%	69.4%
Algeria	Melbou	2017	73.6%	77.2%	69.5%
Algeria	Mellakou	2000	77.7%	79.8%	75.9%
Algeria	Mellakou	2017	77.9%	79.9%	76.1%
Algeria	Menaa	2000	59.3%	63.0%	55.2%
Algeria	Menaa	2000	80.7%	83.1%	77.9%
Algeria	Menaa	2017	80.8%	83.3%	77.9%
Algeria	Menaa	2017	59.5%	63.3%	55.5%
Algeria	Menaceur	2000	76.3%	80.1%	72.2%
Algeria	Menaceur	2017	76.5%	80.3%	72.4%
Algeria	Mendes	2000	76.0%	79.0%	73.2%
Algeria	Mendes	2017	75.7%	78.7%	72.9%
Algeria	Merad	2000	75.5%	78.6%	72.4%
Algeria	Merad	2017	75.7%	78.8%	72.6%
Algeria	Merahna	2000	79.1%	82.4%	75.0%
Algeria	Merahna	2017	79.3%	82.6%	75.2%
Algeria	Merdja Sidi Abed	2000	71.5%	74.1%	68.9%
Algeria	Merdja Sidi Abed	2017	71.7%	74.3%	69.1%
Algeria	Merhoum	2000	76.9%	80.7%	72.6%
Algeria	Merhoum	2017	77.1%	80.9%	72.9%
Algeria	Meridja	2000	51.9%	59.7%	43.0%
Algeria	Meridja	2017	53.1%	60.6%	43.9%
Algeria	Merine	2000	73.6%	78.1%	69.3%
Algeria	Merine	2017	73.8%	78.3%	69.5%
Algeria	Merouana	2000	62.1%	65.4%	58.7%
Algeria	Merouana	2017	62.3%	65.6%	58.9%
Algeria	Mers El Kebir	2000	70.5%	72.9%	67.7%
Algeria	Mers El Kebir	2017	70.3%	73.0%	67.3%
Algeria	Meskiana	2000	62.6%	67.3%	58.1%
Algeria	Meskiana	2017	62.8%	67.5%	58.2%
Algeria	Mesra	2000	71.2%	73.3%	68.9%
Algeria	Mesra	2017	71.6%	73.6%	69.4%
Algeria	Messaad	2000	81.2%	82.9%	79.3%
Algeria	Messaad	2017	81.4%	83.1%	79.4%
Algeria	Messelmoun	2000	76.6%	81.1%	71.9%
Algeria	Messelmoun	2017	76.1%	81.1%	71.1%
Algeria	Metarfa	2000	49.0%	54.5%	42.5%
Algeria	Metarfa	2017	49.2%	54.4%	42.6%
Algeria	Metlili	2000	49.9%	53.9%	46.4%
Algeria	Metlili	2017	49.6%	53.6%	46.2%

Country	Second Administrative Unit	Year	Mean	Upper	Lower
Algeria	Mezaourou	2000	70.8%	74.1%	66.9%
Algeria	Mezaourou	2017	71.0%	74.3%	67.1%
Algeria	Mezdour	2000	77.7%	81.6%	74.3%
Algeria	Mezdour	2017	78.0%	81.8%	74.6%
Algeria	Mezghrane	2000	70.2%	72.5%	68.1%
Algeria	Mezghrane	2017	70.4%	72.7%	68.3%
Algeria	Mezloug	2000	60.5%	62.8%	58.2%
Algeria	Mezloug	2017	60.9%	63.2%	58.6%
Algeria	Mezrenna	2000	77.2%	80.7%	73.1%
Algeria	Mezrenna	2017	77.3%	80.8%	73.2%
Algeria	Mih Ouansa	2000	47.9%	52.3%	43.6%
Algeria	Mih Ouansa	2017	48.3%	52.7%	43.8%
Algeria	Mihoub	2000	76.8%	80.3%	73.0%
Algeria	Mihoub	2017	77.0%	80.5%	73.2%
Algeria	Mila	2000	81.7%	83.9%	79.5%
Algeria	Mila	2017	81.9%	84.0%	79.7%
Algeria	Miliana	2000	76.3%	78.9%	73.1%
Algeria	Miliana	2017	76.5%	79.1%	73.2%
Algeria	Minar Zarza	2000	80.5%	83.1%	78.2%
Algeria	Minar Zarza	2017	80.6%	83.1%	78.3%
Algeria	Misserghin	2000	70.2%	72.4%	67.8%
Algeria	Misserghin	2017	70.5%	72.8%	68.0%
Algeria	Mizrana	2000	78.9%	82.5%	75.0%
Algeria	Mizrana	2017	79.0%	82.5%	75.0%
Algeria	Mogheul	2000	53.2%	59.7%	46.7%
Algeria	Mogheul	2017	53.6%	60.1%	47.1%
Algeria	Moghrar	2000	76.1%	80.1%	72.5%
Algeria	Moghrar	2017	75.9%	79.7%	72.5%
Algeria	Mohamed Boudiaf	2000	78.5%	82.0%	74.6%
Algeria	Mohamed Boudiaf	2017	78.5%	82.1%	74.6%
Algeria	Mohammadia	2000	69.5%	71.8%	66.7%
Algeria	Mohammadia	2017	69.7%	71.9%	66.9%
Algeria	Morsot	2000	63.7%	67.2%	59.8%
Algeria	Morsot	2017	63.9%	67.5%	60.0%
Algeria	Mostaganem	2000	70.8%	73.1%	68.2%
Algeria	Mostaganem	2017	71.0%	73.4%	68.4%
Algeria	Moudjebara	2000	80.9%	82.9%	79.0%
Algeria	Moudjebara	2017	81.1%	83.1%	79.2%
Algeria	Moulay Larbi	2000	77.2%	80.4%	73.6%
Algeria	Moulay Larbi	2017	77.4%	80.5%	73.8%
Algeria	Moulay Slissen	2000	69.8%	72.7%	66.8%
Algeria	Moulay Slissen	2017	70.1%	72.9%	67.1%
Algeria	Moussadek	2000	75.6%	79.6%	71.5%
Algeria	Moussadek	2017	76.1%	80.1%	71.9%
Algeria	Mouzaia	2000	75.5%	77.8%	73.1%
Algeria	Mouzaia	2017	75.8%	78.0%	73.3%
Algeria	Msirda Fouaga	2000	68.5%	74.0%	62.7%
Algeria	Msirda Fouaga	2017	68.6%	74.1%	62.9%
Algeria	Mustafa Ben Brahim	2000	72.3%	76.2%	68.6%
Algeria	Mustafa Ben Brahim	2017	72.5%	76.5%	68.8%
Algeria	N'Gaous	2000	61.4%	65.3%	57.5%
Algeria	N'Gaous	2017	61.3%	65.2%	57.5%
Algeria	N'Goussa	2000	48.3%	52.8%	43.4%
Algeria	N'Goussa	2017	48.6%	53.1%	43.6%

Country	Second Administrative Unit	Year	Mean	Upper	Lower
Algeria	Naama	2000	78.1%	81.6%	74.6%
Algeria	Naama	2017	78.4%	81.9%	74.9%
Algeria	Naciria	2000	77.0%	79.6%	74.6%
Algeria	Naciria	2017	77.2%	79.8%	74.8%
Algeria	Nador	2000	75.3%	79.3%	71.5%
Algeria	Nador	2017	75.6%	79.6%	71.8%
Algeria	Nadorah	2000	78.4%	81.5%	75.1%
Algeria	Nadorah	2017	78.6%	81.7%	75.4%
Algeria	Naima	2000	78.9%	80.8%	76.7%
Algeria	Naima	2017	78.9%	80.7%	76.9%
Algeria	Nakhla	2000	48.4%	52.1%	44.6%
Algeria	Nakhla	2017	48.7%	52.4%	44.8%
Algeria	Nechemaya	2000	80.6%	82.7%	78.1%
Algeria	Nechemaya	2017	80.8%	82.9%	78.3%
Algeria	Nedroma	2000	68.2%	71.8%	64.7%
Algeria	Nedroma	2017	68.4%	72.0%	64.9%
Algeria	Negrine	2000	57.9%	64.8%	51.4%
Algeria	Negrine	2017	57.9%	64.6%	51.5%
Algeria	Nekmaria	2000	70.5%	74.5%	66.4%
Algeria	Nekmaria	2017	70.7%	74.6%	66.4%
Algeria	Nesmoth	2000	76.0%	79.3%	72.7%
Algeria	Nesmoth	2017	76.1%	79.5%	73.0%
Algeria	Nezla	2000	48.9%	51.9%	45.8%
Algeria	Nezla	2017	49.2%	52.1%	46.0%
Algeria	Oggaz	2000	69.6%	72.2%	66.7%
Algeria	Oggaz	2017	69.8%	72.5%	67.0%
Algeria	Ogla Melha	2000	63.1%	66.7%	59.6%
Algeria	Ogla Melha	2017	63.4%	67.1%	59.8%
Algeria	Oran	2000	70.5%	72.4%	68.6%
Algeria	Oran	2017	70.7%	72.5%	68.8%
Algeria	Ouacif	2000	76.6%	79.7%	73.3%
Algeria	Ouacif	2017	76.8%	79.9%	73.6%
Algeria	Ouadhia	2000	76.3%	78.9%	73.5%
Algeria	Ouadhia	2017	76.6%	79.2%	73.8%
Algeria	Ouaguenoun	2000	77.5%	80.4%	75.0%
Algeria	Ouaguenoun	2017	77.3%	80.1%	74.7%
Algeria	Ouamri	2000	77.0%	80.4%	73.7%
Algeria	Ouamri	2017	77.5%	80.9%	74.2%
Algeria	Ouanougha	2000	77.9%	81.5%	74.4%
Algeria	Ouanougha	2017	78.1%	81.6%	74.6%
Algeria	Ouargla	2000	48.3%	51.4%	45.4%
Algeria	Ouargla	2017	48.6%	51.6%	45.8%
Algeria	Ouarizane	2000	69.9%	72.5%	66.6%
Algeria	Ouarizane	2017	70.1%	72.7%	66.8%
Algeria	Oudjana	2000	82.5%	84.6%	80.6%
Algeria	Oudjana	2017	82.7%	84.8%	80.9%
Algeria	Oued Athmenia	2000	79.4%	81.1%	77.0%
Algeria	Oued Athmenia	2017	79.6%	81.3%	77.2%
Algeria	Oued Berkeche	2000	70.7%	74.0%	66.7%
Algeria	Oued Berkeche	2017	70.9%	74.3%	67.0%
Algeria	Oued Chaaba	2000	62.1%	65.0%	59.1%
Algeria	Oued Chaaba	2017	62.5%	65.4%	59.5%
Algeria	Oued Cheham	2000	80.6%	83.0%	78.0%
Algeria	Oued Cheham	2017	80.9%	83.2%	78.3%
Algeria	Oued Chorfa	2000	76.3%	79.8%	72.8%
Algeria	Oued Chorfa	2017	76.6%	80.0%	73.1%
Algeria	Oued Chouly	2000	69.4%	73.0%	65.7%
Algeria	Oued Chouly	2017	69.7%	73.3%	66.1%

Country	Second Administrative Unit	Year	Mean	Upper	Lower
Algeria	Oued Djemaa	2000	77.6%	80.5%	74.2%
Algeria	Oued Djemaa	2017	77.7%	80.6%	74.3%
Algeria	Oued Djer	2000	75.8%	78.6%	72.6%
Algeria	Oued Djer	2017	75.8%	78.6%	72.5%
Algeria	Oued El Abtal	2000	73.6%	76.9%	70.1%
Algeria	Oued El Abtal	2017	73.7%	77.1%	70.1%
Algeria	Oued El Alenda	2000	48.2%	51.7%	45.3%
Algeria	Oued El Alenda	2017	48.5%	52.0%	45.5%
Algeria	Oued El Alleug	2000	75.3%	77.1%	73.4%
Algeria	Oued El Alleug	2017	75.5%	77.2%	73.6%
Algeria	Oued El Aneb	2000	81.1%	83.0%	78.9%
Algeria	Oued El Aneb	2017	81.3%	83.2%	79.1%
Algeria	Oued El Barad	2000	68.9%	72.2%	65.1%
Algeria	Oued El Barad	2017	69.3%	72.7%	65.5%
Algeria	Oued El Berdi	2000	76.7%	79.6%	73.6%
Algeria	Oued El Berdi	2017	77.0%	79.8%	73.8%
Algeria	Oued El Djemaa	2000	69.9%	72.5%	67.1%
Algeria	Oued El Djemaa	2017	70.1%	72.8%	67.3%
Algeria	Oued El Kheir	2000	71.1%	73.7%	68.4%
Algeria	Oued El Kheir	2017	71.3%	73.9%	68.5%
Algeria	Oued El Ma	2000	62.6%	65.7%	59.1%
Algeria	Oued El Ma	2017	62.5%	65.6%	59.1%
Algeria	Oued Endja	2000	80.7%	82.3%	79.1%
Algeria	Oued Endja	2017	80.8%	82.4%	79.3%
Algeria	Oued Essalem	2000	75.7%	78.5%	73.2%
Algeria	Oued Essalem	2017	76.0%	78.7%	73.4%
Algeria	Oued Fodda	2000	75.7%	78.0%	73.3%
Algeria	Oued Fodda	2017	76.0%	78.3%	73.5%
Algeria	Oued Fragha	2000	80.5%	82.2%	78.8%
Algeria	Oued Fragha	2017	80.6%	82.3%	78.9%
Algeria	Oued Ghir	2000	75.1%	78.2%	71.6%
Algeria	Oued Ghir	2017	75.3%	78.4%	71.8%
Algeria	Oued Goussine	2000	75.9%	81.0%	70.5%
Algeria	Oued Goussine	2017	75.8%	81.0%	70.3%
Algeria	Oued Harbil	2000	76.4%	79.6%	72.9%
Algeria	Oued Harbil	2017	76.6%	79.8%	73.0%
Algeria	Oued Kebrit	2000	69.8%	72.9%	65.7%
Algeria	Oued Kebrit	2017	70.4%	73.5%	66.3%
Algeria	Oued Lilli	2000	78.8%	80.6%	77.1%
Algeria	Oued Lilli	2017	79.1%	80.8%	77.4%
Algeria	Oued M’Zi	2000	80.8%	83.4%	78.1%
Algeria	Oued M’Zi	2017	80.9%	83.5%	78.2%
Algeria	Oued Mora	2000	80.6%	83.4%	77.5%
Algeria	Oued Mora	2017	80.8%	83.5%	77.7%
Algeria	Oued Nini	2000	62.1%	66.8%	58.0%
Algeria	Oued Nini	2017	62.3%	66.9%	58.2%
Algeria	Oued Rhiou	2000	70.2%	72.4%	67.8%
Algeria	Oued Rhiou	2017	70.5%	72.6%	68.0%
Algeria	Oued Sebaa	2000	72.4%	77.4%	66.7%
Algeria	Oued Sebaa	2017	72.6%	77.4%	66.9%
Algeria	Oued Sebbah	2000	70.5%	73.5%	66.7%
Algeria	Oued Sebbah	2017	70.8%	73.9%	67.1%

Country	Second Administrative Unit	Year	Mean	Upper	Lower
Algeria	Oued Sefioune	2000	71.9%	76.1%	67.6%
Algeria	Oued Sefioune	2017	72.1%	76.3%	67.8%
Algeria	Oued Seguen	2000	76.2%	78.7%	73.7%
Algeria	Oued Seguen	2017	76.3%	78.7%	73.7%
Algeria	Oued Sly	2000	74.3%	76.5%	71.9%
Algeria	Oued Sly	2017	74.5%	76.7%	72.1%
Algeria	Oued Taga	2000	62.1%	65.6%	58.4%
Algeria	Oued Taga	2017	62.5%	66.0%	58.8%
Algeria	Oued Taourira	2000	73.7%	77.7%	69.2%
Algeria	Oued Taourira	2017	73.8%	77.9%	69.2%
Algeria	Oued Taria	2000	73.8%	76.5%	70.5%
Algeria	Oued Taria	2017	74.0%	76.7%	70.9%
Algeria	Oued Tlelat	2000	69.7%	72.8%	66.7%
Algeria	Oued Tlelat	2017	70.1%	73.2%	67.1%
Algeria	Oued Zenati	2000	79.1%	81.9%	76.4%
Algeria	Oued Zenati	2017	79.3%	82.1%	76.6%
Algeria	Oued Zhour	2000	82.9%	85.5%	79.9%
Algeria	Oued Zhour	2017	83.0%	85.6%	79.9%
Algeria	Oued Zitoun	2000	80.6%	84.0%	77.5%
Algeria	Oued Zitoun	2017	80.8%	84.2%	77.7%
Algeria	Ouenza	2000	69.9%	74.5%	65.0%
Algeria	Ouenza	2017	70.1%	74.8%	65.1%
Algeria	Ouezra	2000	76.8%	80.1%	73.4%
Algeria	Ouezra	2017	77.1%	80.4%	73.7%
Algeria	Ouillen	2000	79.9%	83.1%	76.4%
Algeria	Ouillen	2017	80.0%	83.2%	76.5%
Algeria	Ouldja Boulbalout	2000	82.1%	84.3%	79.9%
Algeria	Ouldja Boulbalout	2017	82.3%	84.5%	80.1%
Algeria	Ouled Abbes	2000	76.0%	78.1%	73.4%
Algeria	Ouled Abbes	2017	76.1%	78.2%	73.5%
Algeria	Ouled Addi Guebala	2000	78.8%	81.4%	76.5%
Algeria	Ouled Addi Guebala	2017	79.0%	81.6%	76.7%
Algeria	Ouled Addouane	2000	65.9%	68.0%	63.0%
Algeria	Ouled Addouane	2017	66.1%	68.2%	63.2%
Algeria	Ouled Ahmed Temmi	2000	49.3%	53.6%	45.2%
Algeria	Ouled Ahmed Temmi	2017	49.6%	53.8%	45.4%
Algeria	Ouled Aissa	2000	49.4%	54.6%	44.9%
Algeria	Ouled Aissa	2000	77.1%	80.5%	73.6%
Algeria	Ouled Aissa	2017	77.3%	80.7%	73.7%
Algeria	Ouled Aissa	2017	49.7%	54.8%	45.1%
Algeria	Ouled Ammar	2000	70.7%	73.5%	67.8%
Algeria	Ouled Ammar	2017	71.0%	73.9%	68.2%
Algeria	Ouled Antar	2000	77.9%	80.8%	74.9%
Algeria	Ouled Antar	2017	78.0%	81.0%	75.1%
Algeria	Ouled Aouf	2000	62.1%	65.8%	58.5%
Algeria	Ouled Aouf	2017	62.4%	66.1%	58.9%
Algeria	Ouled Attia	2000	84.6%	87.9%	81.0%
Algeria	Ouled Attia	2017	85.0%	88.4%	81.5%
Algeria	Ouled Ben Abdelkader	2000	74.9%	77.6%	72.0%
Algeria	Ouled Ben Abdelkader	2017	74.8%	77.4%	71.9%

Country	Second Administrative Unit	Year	Mean	Upper	Lower
Algeria	Ouled Bessem	2000	78.6%	80.9%	76.3%
Algeria	Ouled Bessem	2017	78.7%	81.0%	76.4%
Algeria	Ouled Bouachra	2000	76.7%	79.5%	73.5%
Algeria	Ouled Bouachra	2017	76.9%	79.7%	73.8%
Algeria	Ouled Boudjemaa	2000	69.6%	73.4%	65.2%
Algeria	Ouled Boudjemaa	2017	69.9%	73.9%	65.4%
Algeria	Ouled Boughalem	2000	70.0%	75.1%	64.8%
Algeria	Ouled Boughalem	2017	70.3%	75.4%	65.0%
Algeria	Ouled Brahem	2000	68.4%	71.8%	64.9%
Algeria	Ouled Brahem	2017	68.6%	72.1%	65.1%
Algeria	Ouled Brahim	2000	77.5%	80.2%	74.8%
Algeria	Ouled Brahim	2000	76.9%	80.9%	73.3%
Algeria	Ouled Brahim	2017	77.0%	80.9%	73.3%
Algeria	Ouled Brahim	2017	77.6%	80.3%	74.9%
Algeria	Ouled Chebel	2000	75.5%	77.6%	73.7%
Algeria	Ouled Chebel	2017	75.9%	77.9%	74.1%
Algeria	Ouled Dahmane	2000	65.0%	68.0%	61.6%
Algeria	Ouled Dahmane	2017	65.5%	68.5%	62.1%
Algeria	Ouled Daid	2000	77.4%	80.7%	73.7%
Algeria	Ouled Daid	2017	77.5%	80.7%	73.7%
Algeria	Ouled Derradj	2000	80.4%	82.6%	78.3%
Algeria	Ouled Derradj	2017	80.6%	82.8%	78.6%
Algeria	Ouled Djellal	2000	49.3%	51.7%	46.9%
Algeria	Ouled Djellal	2017	49.7%	52.1%	47.3%
Algeria	Ouled Driss	2000	81.0%	83.9%	78.3%
Algeria	Ouled Driss	2017	81.0%	84.0%	78.3%
Algeria	Ouled Fadhel	2000	60.0%	64.0%	56.3%
Algeria	Ouled Fadhel	2017	60.2%	64.2%	56.5%
Algeria	Ouled Fares	2000	75.3%	77.8%	72.6%
Algeria	Ouled Fares	2017	75.3%	77.8%	72.6%
Algeria	Ouled Fayet	2000	75.5%	77.3%	73.8%
Algeria	Ouled Fayet	2017	75.7%	77.5%	74.0%
Algeria	Ouled Gacem	2000	66.4%	68.9%	64.1%
Algeria	Ouled Gacem	2017	66.6%	69.1%	64.3%
Algeria	Ouled Hamla	2000	71.8%	74.6%	68.8%
Algeria	Ouled Hamla	2017	72.0%	74.9%	69.0%
Algeria	Ouled Hebbaba	2000	80.7%	82.7%	78.4%
Algeria	Ouled Hebbaba	2017	80.9%	82.8%	78.6%
Algeria	Ouled Hedadj	2000	75.6%	77.4%	73.9%
Algeria	Ouled Hedadj	2017	75.8%	77.6%	74.2%
Algeria	Ouled Hellal	2000	78.0%	81.1%	74.7%
Algeria	Ouled Hellal	2017	78.2%	81.3%	75.0%
Algeria	Ouled Khaled	2000	78.3%	79.9%	76.5%
Algeria	Ouled Khaled	2017	78.4%	80.1%	76.6%
Algeria	Ouled Khelouf	2000	70.8%	73.6%	67.6%
Algeria	Ouled Khelouf	2017	71.4%	74.1%	68.4%
Algeria	Ouled Khoudir	2000	49.5%	58.4%	41.6%
Algeria	Ouled Khoudir	2017	49.6%	58.0%	42.2%
Algeria	Ouled Kihel	2000	69.2%	72.5%	64.8%
Algeria	Ouled Kihel	2017	69.3%	72.7%	65.0%

Country	Second Administrative Unit	Year	Mean	Upper	Lower
Algeria	Ouled Maalah	2000	70.0%	73.5%	66.8%
Algeria	Ouled Maalah	2017	70.4%	73.8%	67.2%
Algeria	Ouled Maaraf	2000	78.6%	81.7%	75.5%
Algeria	Ouled Maaraf	2017	78.8%	81.9%	75.8%
Algeria	Ouled Madhi	2000	81.0%	83.2%	78.6%
Algeria	Ouled Madhi	2017	81.1%	83.4%	78.8%
Algeria	Ouled Mansour	2000	80.8%	82.8%	78.7%
Algeria	Ouled Mansour	2017	81.0%	83.0%	78.9%
Algeria	Ouled Mimoun	2000	70.2%	73.9%	66.4%
Algeria	Ouled Mimoun	2017	70.6%	74.3%	66.8%
Algeria	Ouled Moumen	2000	79.8%	83.9%	74.8%
Algeria	Ouled Moumen	2017	80.0%	84.1%	75.1%
Algeria	Ouled Moussa	2000	75.8%	77.8%	74.1%
Algeria	Ouled Moussa	2017	76.0%	78.0%	74.3%
Algeria	Ouled Rabah	2000	83.5%	85.8%	81.6%
Algeria	Ouled Rabah	2017	83.6%	85.9%	81.8%
Algeria	Ouled Rached	2000	76.8%	80.5%	72.8%
Algeria	Ouled Rached	2017	77.0%	80.8%	73.1%
Algeria	Ouled Rahmoune	2000	73.2%	75.8%	70.7%
Algeria	Ouled Rahmoune	2017	73.6%	76.2%	71.2%
Algeria	Ouled Rechache	2000	62.3%	66.6%	58.0%
Algeria	Ouled Rechache	2017	62.4%	66.7%	58.1%
Algeria	Ouled Riyah	2000	67.3%	70.6%	63.6%
Algeria	Ouled Riyah	2017	67.5%	70.8%	63.9%
Algeria	Ouled Saber	2000	62.3%	64.2%	59.8%
Algeria	Ouled Saber	2017	62.6%	64.5%	60.2%
Algeria	Ouled Said	2000	49.4%	55.1%	43.3%
Algeria	Ouled Said	2017	49.7%	55.3%	43.5%
Algeria	Ouled Sellem	2000	62.7%	66.7%	59.0%
Algeria	Ouled Sellem	2017	63.0%	66.9%	59.4%
Algeria	Ouled Si Ahmed	2000	67.1%	70.1%	63.1%
Algeria	Ouled Si Ahmed	2017	67.3%	70.3%	63.4%
Algeria	Ouled Si Slimane	2000	62.6%	66.3%	59.1%
Algeria	Ouled Si Slimane	2017	62.5%	66.2%	59.1%
Algeria	Ouled Sidi Brahim	2000	81.0%	83.5%	78.5%
Algeria	Ouled Sidi Brahim	2000	72.8%	77.0%	68.4%
Algeria	Ouled Sidi Brahim	2017	81.3%	83.7%	78.9%
Algeria	Ouled Sidi Brahim	2017	73.0%	77.3%	68.7%
Algeria	Ouled Sidi Mihoub	2000	69.4%	72.5%	66.1%
Algeria	Ouled Sidi Mihoub	2017	69.6%	72.7%	66.2%
Algeria	Ouled Slama	2000	75.7%	78.5%	72.5%
Algeria	Ouled Slama	2017	75.9%	78.6%	72.8%

Country	Second Administrative Unit	Year	Mean	Upper	Lower
Algeria	Ouled Slimane	2000	72.2%	76.8%	68.0%
Algeria	Ouled Slimane	2017	72.2%	76.8%	67.9%
Algeria	Ouled Tebben	2000	72.4%	75.7%	69.1%
Algeria	Ouled Tebben	2017	72.6%	75.9%	69.2%
Algeria	Ouled Yahia Khadrouche	2000	83.8%	86.2%	81.2%
Algeria	Ouled Yahia Khadrouche	2017	83.9%	86.3%	81.3%
Algeria	Ouled Yaich	2000	71.3%	74.0%	68.4%
Algeria	Ouled Yaich	2000	76.0%	78.3%	73.5%
Algeria	Ouled Yaich	2017	71.6%	74.2%	68.5%
Algeria	Ouled Yaich	2017	76.1%	78.3%	73.6%
Algeria	Ouled Zaoui	2000	64.7%	67.7%	61.4%
Algeria	Ouled Zaoui	2017	64.8%	67.8%	61.7%
Algeria	Oulhaca El Gheraba	2000	68.3%	72.6%	64.0%
Algeria	Oulhaca El Gheraba	2017	68.6%	72.8%	64.3%
Algeria	Oultene	2000	80.8%	83.3%	78.2%
Algeria	Oultene	2017	81.0%	83.5%	78.4%
Algeria	Oum Ali	2000	62.9%	68.2%	57.6%
Algeria	Oum Ali	2017	63.1%	68.4%	57.7%
Algeria	Oum Drou	2000	75.5%	77.4%	73.3%
Algeria	Oum Drou	2017	75.7%	77.6%	73.5%
Algeria	Oum El Adhaim	2000	71.8%	76.4%	66.8%
Algeria	Oum El Adhaim	2017	71.9%	76.6%	67.0%
Algeria	Oum El Assel	2000	49.6%	55.7%	43.7%
Algeria	Oum El Assel	2017	49.7%	55.8%	43.7%
Algeria	Oum El Bouaghi	2000	64.0%	66.9%	60.8%
Algeria	Oum El Bouaghi	2017	64.1%	67.0%	60.8%
Algeria	Oum El Djellil	2000	77.1%	80.7%	73.4%
Algeria	Oum El Djellil	2017	77.2%	80.9%	73.6%
Algeria	Oum Laadham	2000	66.9%	72.2%	61.4%
Algeria	Oum Laadham	2017	67.5%	72.8%	62.2%
Algeria	Oum Toub	2000	81.4%	83.7%	79.0%
Algeria	Oum Toub	2017	81.4%	83.8%	79.0%
Algeria	Oum Touyour	2000	49.2%	53.6%	44.7%
Algeria	Oum Touyour	2017	49.4%	53.5%	45.1%
Algeria	Oumache	2000	48.5%	51.5%	45.6%
Algeria	Oumache	2017	48.8%	51.8%	45.9%
Algeria	Ourlal	2000	48.6%	52.1%	45.1%
Algeria	Ourlal	2017	48.9%	52.4%	45.3%
Algeria	Ourmes	2000	48.1%	51.1%	45.4%
Algeria	Ourmes	2017	48.5%	51.4%	45.8%
Algeria	Ouyoun El Assafir	2000	62.9%	66.1%	60.1%
Algeria	Ouyoun El Assafir	2017	63.1%	66.3%	60.3%
Algeria	Ouzzelaguen	2000	74.1%	76.7%	71.8%
Algeria	Ouzzelaguen	2017	74.0%	76.6%	71.8%
Algeria	Rabta	2000	71.9%	74.7%	68.8%
Algeria	Rabta	2017	71.7%	74.6%	68.5%
Algeria	Ragouba	2000	76.7%	79.9%	73.1%
Algeria	Ragouba	2017	76.6%	79.7%	73.0%

Country	Second Administrative Unit	Year	Mean	Upper	Lower
Algeria	Rahouia	2000	78.3%	80.8%	76.0%
Algeria	Rahouia	2017	78.7%	81.1%	76.3%
Algeria	Ramdane Djamel	2000	80.4%	82.3%	78.5%
Algeria	Ramdane Djamel	2017	80.5%	82.4%	78.6%
Algeria	Ramka	2000	76.6%	79.4%	73.6%
Algeria	Ramka	2017	76.7%	79.5%	73.8%
Algeria	Raml Souk	2000	79.2%	83.0%	75.0%
Algeria	Raml Souk	2017	79.4%	83.2%	75.2%
Algeria	Raouraoua	2000	77.5%	80.4%	73.9%
Algeria	Raouraoua	2017	77.8%	80.6%	74.2%
Algeria	Ras Ain Amirouche	2000	69.2%	72.0%	66.0%
Algeria	Ras Ain Amirouche	2017	69.4%	72.2%	66.2%
Algeria	Ras El Agba	2000	79.6%	82.4%	76.7%
Algeria	Ras El Agba	2017	79.7%	82.6%	76.9%
Algeria	Ras El Aioun	2000	62.9%	66.7%	59.5%
Algeria	Ras El Aioun	2017	63.1%	66.7%	59.5%
Algeria	Ras El Ma	2000	73.2%	79.0%	67.3%
Algeria	Ras El Ma	2017	73.5%	79.3%	67.7%
Algeria	Ras El Oued	2000	67.8%	71.3%	64.3%
Algeria	Ras El Oued	2017	68.5%	72.0%	65.0%
Algeria	Ras Miaad|Ouled Sassi	2000	62.1%	67.6%	56.6%
Algeria	Ras Miaad|Ouled Sassi	2017	61.8%	67.1%	56.4%
Algeria	Rechaiga	2000	79.2%	81.8%	76.7%
Algeria	Rechaiga	2017	79.4%	82.0%	76.9%
Algeria	Redjem Demouche	2000	72.4%	78.7%	66.3%
Algeria	Redjem Demouche	2017	72.6%	78.8%	66.5%
Algeria	Reggane	2000	46.8%	51.2%	42.4%
Algeria	Reggane	2017	46.5%	51.6%	40.8%
Algeria	Reghaia	2000	75.6%	77.5%	73.8%
Algeria	Reghaia	2017	75.7%	77.7%	74.0%
Algeria	Reguiba	2000	48.0%	52.0%	44.0%
Algeria	Reguiba	2017	48.3%	52.3%	44.5%
Algeria	Rehbat	2000	62.6%	65.9%	59.0%
Algeria	Rehbat	2017	62.9%	66.2%	59.4%
Algeria	Relizane	2000	70.6%	73.0%	68.1%
Algeria	Relizane	2017	70.9%	73.3%	68.4%
Algeria	Remchi	2000	68.1%	71.3%	64.6%
Algeria	Remchi	2017	68.3%	71.5%	64.7%
Algeria	Remila	2000	60.7%	64.7%	56.9%
Algeria	Remila	2017	61.0%	65.0%	57.1%
Algeria	Ridane	2000	78.0%	81.5%	74.4%
Algeria	Ridane	2017	78.1%	81.6%	74.4%
Algeria	Robbah	2000	48.4%	52.1%	45.2%
Algeria	Robbah	2017	48.7%	52.4%	45.3%
Algeria	Rogassa	2000	78.6%	82.4%	74.9%
Algeria	Rogassa	2017	78.8%	82.5%	75.2%
Algeria	Roknia	2000	80.3%	82.9%	77.8%
Algeria	Roknia	2017	80.4%	83.1%	78.0%
Algeria	Rosfa	2000	68.7%	71.7%	65.4%
Algeria	Rosfa	2017	68.5%	71.5%	65.2%
Algeria	Rouached	2000	80.2%	82.2%	78.2%
Algeria	Rouached	2017	80.4%	82.4%	78.5%

Country	Second Administrative Unit	Year	Mean	Upper	Lower
Algeria	Roubia	2000	76.9%	80.5%	72.8%
Algeria	Roubia	2017	77.0%	80.6%	72.9%
Algeria	Rouiba	2000	75.5%	77.0%	73.9%
Algeria	Rouiba	2017	75.7%	77.2%	74.1%
Algeria	Rouina	2000	76.0%	78.7%	73.2%
Algeria	Rouina	2017	76.2%	78.8%	73.3%
Algeria	Rouissat	2000	48.3%	52.3%	44.9%
Algeria	Rouissat	2017	48.5%	52.9%	45.0%
Algeria	Sabra	2000	68.9%	72.5%	65.6%
Algeria	Sabra	2017	69.4%	73.0%	65.9%
Algeria	Safel El Ouiden	2000	69.3%	73.5%	64.4%
Algeria	Safel El Ouiden	2017	69.5%	73.6%	64.6%
Algeria	Safsaf	2000	71.9%	75.1%	69.0%
Algeria	Safsaf	2017	72.1%	75.3%	69.1%
Algeria	Safsaf El Ouesra	2000	62.5%	66.9%	57.4%
Algeria	Safsaf El Ouesra	2017	62.8%	67.1%	58.0%
Algeria	Saharidj	2000	77.0%	80.6%	73.2%
Algeria	Saharidj	2017	76.9%	80.6%	73.1%
Algeria	Saida	2000	78.8%	80.3%	77.1%
Algeria	Saida	2017	79.1%	80.5%	77.4%
Algeria	Salah Bey	2000	64.8%	67.6%	61.6%
Algeria	Salah Bey	2017	65.0%	67.8%	61.7%
Algeria	Salah Bouchaour	2000	80.2%	82.3%	78.0%
Algeria	Salah Bouchaour	2017	80.3%	82.4%	78.2%
Algeria	Sali	2000	47.6%	52.9%	42.2%
Algeria	Sali	2017	46.9%	52.3%	41.5%
Algeria	Saneg	2000	77.1%	80.8%	73.7%
Algeria	Saneg	2017	77.3%	81.0%	73.8%
Algeria	Saoula	2000	75.7%	77.1%	74.3%
Algeria	Saoula	2017	75.9%	77.3%	74.5%
Algeria	Sayada	2000	71.3%	73.4%	68.9%
Algeria	Sayada	2017	71.5%	73.5%	69.2%
Algeria	Sebaa	2000	49.1%	53.7%	43.5%
Algeria	Sebaa	2017	49.3%	53.5%	44.0%
Algeria	Sebaine	2000	78.8%	80.6%	76.8%
Algeria	Sebaine	2017	78.9%	80.9%	76.9%
Algeria	Sebbaa Chioukh	2000	69.7%	72.5%	66.6%
Algeria	Sebbaa Chioukh	2017	70.0%	72.9%	67.0%
Algeria	Sebdou	2000	70.1%	74.5%	65.1%
Algeria	Sebdou	2017	70.2%	74.6%	65.2%
Algeria	Sebgag	2000	80.4%	83.2%	77.3%
Algeria	Sebgag	2017	80.6%	83.3%	77.7%
Algeria	Sebseb	2000	50.4%	55.1%	46.3%
Algeria	Sebseb	2017	51.7%	56.1%	47.6%
Algeria	Sebt	2000	77.6%	80.2%	74.9%
Algeria	Sebt	2017	78.1%	80.7%	75.3%
Algeria	Sed Rahal	2000	79.9%	82.6%	77.3%
Algeria	Sed Rahal	2017	80.1%	82.8%	77.6%
Algeria	Seddouk	2000	72.8%	75.9%	70.0%
Algeria	Seddouk	2017	73.0%	76.1%	70.2%
Algeria	Sedjerara	2000	70.8%	73.4%	67.7%
Algeria	Sedjerara	2017	70.9%	73.4%	67.7%
Algeria	Sedrata	2000	76.2%	80.3%	72.5%
Algeria	Sedrata	2017	76.3%	80.3%	72.6%

Country	Second Administrative Unit	Year	Mean	Upper	Lower
Algeria	Sedraya	2000	76.9%	80.3%	72.9%
Algeria	Sedraya	2017	77.0%	80.4%	73.0%
Algeria	Sefiane	2000	60.6%	64.3%	57.2%
Algeria	Sefiane	2017	60.9%	64.4%	57.5%
Algeria	Seggana	2000	60.2%	63.1%	57.0%
Algeria	Seggana	2017	60.2%	63.2%	57.1%
Algeria	Seghouane	2000	77.8%	81.2%	74.2%
Algeria	Seghouane	2017	77.9%	81.2%	74.4%
Algeria	Sehailia	2000	73.0%	76.2%	69.3%
Algeria	Sehailia	2017	73.3%	76.4%	69.6%
Algeria	Sehala Thaoura	2000	70.7%	73.8%	67.3%
Algeria	Sehala Thaoura	2017	70.9%	74.0%	67.5%
Algeria	Selaoua Announa	2000	79.2%	82.0%	76.4%
Algeria	Selaoua Announa	2017	79.5%	82.3%	76.7%
Algeria	Selma Benziada	2000	77.7%	80.7%	74.4%
Algeria	Selma Benziada	2017	78.0%	81.0%	74.7%
Algeria	Selmana	2000	79.1%	81.6%	76.2%
Algeria	Selmana	2017	79.2%	81.6%	76.3%
Algeria	Sendjas	2000	75.8%	78.0%	73.5%
Algeria	Sendjas	2017	75.8%	78.0%	73.5%
Algeria	Seraidi	2000	81.3%	83.2%	79.1%
Algeria	Seraidi	2017	81.5%	83.4%	79.3%
Algeria	Serdj-El-Ghoul	2000	69.6%	72.4%	66.5%
Algeria	Serdj-El-Ghoul	2017	69.6%	72.4%	66.4%
Algeria	Serghine	2000	79.9%	82.4%	77.2%
Algeria	Serghine	2017	80.1%	82.4%	77.5%
Algeria	Seriana	2000	64.4%	67.3%	61.3%
Algeria	Seriana	2017	64.6%	67.5%	61.5%
Algeria	Setif	2000	60.3%	62.1%	58.3%
Algeria	Setif	2017	60.6%	62.4%	58.6%
Algeria	Settara	2000	82.3%	84.7%	79.7%
Algeria	Settara	2017	82.4%	84.8%	79.8%
Algeria	Sfisef	2000	72.6%	76.3%	68.7%
Algeria	Sfisef	2017	72.8%	76.5%	69.0%
Algeria	Sfissifa	2000	77.1%	81.2%	73.0%
Algeria	Sfissifa	2017	77.0%	81.3%	72.6%
Algeria	Si Abdelghani	2000	78.0%	80.2%	75.4%
Algeria	Si Abdelghani	2017	78.2%	80.4%	75.6%
Algeria	Si El Mahdjoub	2000	76.5%	79.4%	72.9%
Algeria	Si El Mahdjoub	2017	76.8%	79.7%	73.3%
Algeria	Si Mustapha	2000	75.5%	78.2%	72.6%
Algeria	Si Mustapha	2017	75.7%	78.4%	72.8%
Algeria	Sidi Abdelaziz	2000	82.2%	84.6%	79.3%
Algeria	Sidi Abdelaziz	2017	82.2%	84.7%	79.3%
Algeria	Sidi Abdeldjebar	2000	72.5%	76.4%	68.7%
Algeria	Sidi Abdeldjebar	2017	72.7%	76.6%	68.9%
Algeria	Sidi Abdelli	2000	70.1%	73.8%	66.7%
Algeria	Sidi Abdelli	2017	70.4%	74.0%	66.9%
Algeria	Sidi Abdelmoumene	2000	69.8%	72.2%	67.2%

Country	Second Administrative Unit	Year	Mean	Upper	Lower
Algeria	Sidi Abdelmoumene	2017	70.0%	72.4%	67.5%
Algeria	Sidi Abderrahmane	2000	78.0%	81.0%	74.7%
Algeria	Sidi Abderrahmane	2000	74.8%	80.1%	69.4%
Algeria	Sidi Abderrahmane	2017	78.2%	81.2%	75.0%
Algeria	Sidi Abderrahmane	2017	74.8%	80.2%	69.3%
Algeria	Sidi Abed	2000	78.6%	81.0%	76.4%
Algeria	Sidi Abed	2017	78.5%	80.7%	76.3%
Algeria	Sidi Ahmed	2000	78.2%	81.2%	75.6%
Algeria	Sidi Ahmed	2017	78.3%	81.2%	75.7%
Algeria	Sidi Aissa	2000	79.8%	82.7%	76.9%
Algeria	Sidi Aissa	2017	80.1%	83.0%	77.3%
Algeria	Sidi Akkacha	2000	76.1%	80.9%	71.1%
Algeria	Sidi Akkacha	2017	76.2%	81.0%	71.2%
Algeria	Sidi Ali	2000	71.3%	75.2%	67.7%
Algeria	Sidi Ali	2017	71.6%	75.5%	68.0%
Algeria	Sidi Ali Benyoub	2000	69.7%	73.0%	66.3%
Algeria	Sidi Ali Benyoub	2017	70.0%	73.2%	66.6%
Algeria	Sidi Ali Boussidi	2000	71.3%	74.7%	68.0%
Algeria	Sidi Ali Boussidi	2017	71.4%	74.9%	68.2%
Algeria	Sidi Ali Mellal	2000	76.7%	79.1%	74.3%
Algeria	Sidi Ali Mellal	2017	76.7%	79.1%	74.3%
Algeria	Sidi Amar	2000	76.7%	78.9%	74.3%
Algeria	Sidi Amar	2000	75.2%	79.2%	71.1%
Algeria	Sidi Amar	2000	80.5%	81.9%	79.1%
Algeria	Sidi Amar	2017	80.6%	82.0%	79.2%
Algeria	Sidi Amar	2017	75.4%	79.4%	71.2%
Algeria	Sidi Amar	2017	76.7%	78.9%	74.3%
Algeria	Sidi Ameur	2000	79.1%	82.9%	75.2%
Algeria	Sidi Ameur	2000	81.8%	85.1%	78.7%
Algeria	Sidi Ameur	2017	79.3%	83.0%	75.5%
Algeria	Sidi Ameur	2017	81.9%	85.2%	78.7%
Algeria	Sidi Amrane	2000	49.2%	51.8%	46.2%
Algeria	Sidi Amrane	2017	49.4%	52.0%	46.5%
Algeria	Sidi Aoun	2000	48.3%	52.2%	44.6%
Algeria	Sidi Aoun	2017	48.6%	52.4%	44.8%
Algeria	Sidi Baizid	2000	81.4%	83.7%	79.1%
Algeria	Sidi Baizid	2017	81.6%	83.8%	79.3%
Algeria	Sidi Bakhti	2000	78.6%	81.1%	76.5%
Algeria	Sidi Bakhti	2017	78.8%	81.3%	76.7%
Algeria	Sidi Bel Abbes	2000	70.7%	73.2%	68.6%
Algeria	Sidi Bel Abbes	2017	71.0%	73.4%	68.8%
Algeria	Sidi Belattar	2000	70.5%	73.4%	67.6%
Algeria	Sidi Belattar	2017	70.7%	73.6%	67.8%
Algeria	Sidi Ben Adda	2000	70.6%	73.1%	67.4%
Algeria	Sidi Ben Adda	2017	70.6%	73.1%	67.4%
Algeria	Sidi Ben Yebka	2000	70.3%	72.9%	67.7%
Algeria	Sidi Ben Yebka	2017	70.6%	73.1%	68.0%
Algeria	Sidi Boubekeur	2000	75.3%	78.1%	72.5%

Country	Second Administrative Unit	Year	Mean	Upper	Lower
Algeria	Sidi Boubekeur	2017	75.6%	78.2%	72.8%
Algeria	Sidi Boumediene	2000	71.1%	74.0%	67.7%
Algeria	Sidi Boumediene	2017	71.3%	74.2%	67.9%
Algeria	Sidi Boussaid	2000	73.7%	76.7%	71.0%
Algeria	Sidi Boussaid	2017	73.9%	76.9%	71.3%
Algeria	Sidi Boutouchent	2000	79.4%	81.5%	76.9%
Algeria	Sidi Boutouchent	2017	79.6%	81.8%	77.2%
Algeria	Sidi Bouzid	2000	80.3%	82.8%	77.1%
Algeria	Sidi Bouzid	2017	80.4%	83.0%	77.3%
Algeria	Sidi Brahim	2000	70.2%	73.3%	67.1%
Algeria	Sidi Brahim	2017	70.4%	73.5%	67.3%
Algeria	Sidi Chahmi	2000	70.4%	72.4%	68.3%
Algeria	Sidi Chahmi	2017	70.6%	72.8%	68.6%
Algeria	Sidi Chouab	2000	73.7%	78.6%	68.4%
Algeria	Sidi Chouab	2017	73.7%	78.7%	68.5%
Algeria	Sidi Dahou Zair	2000	70.7%	74.2%	67.4%
Algeria	Sidi Dahou Zair	2017	70.8%	74.3%	67.5%
Algeria	Sidi Damed	2000	77.9%	81.7%	73.5%
Algeria	Sidi Damed	2017	78.0%	81.8%	73.7%
Algeria	Sidi Daoud	2000	76.1%	79.9%	71.9%
Algeria	Sidi Daoud	2017	76.3%	80.1%	72.2%
Algeria	Sidi Djilali	2000	71.8%	76.3%	66.2%
Algeria	Sidi Djilali	2017	71.7%	76.3%	66.2%
Algeria	Sidi Embarek	2000	62.3%	64.9%	59.1%
Algeria	Sidi Embarek	2017	62.4%	65.1%	59.2%
Algeria	Sidi Errabia	2000	76.6%	80.2%	72.6%
Algeria	Sidi Errabia	2017	76.8%	80.4%	72.8%
Algeria	Sidi Fredj	2000	73.9%	78.1%	68.5%
Algeria	Sidi Fredj	2017	74.2%	78.4%	69.0%
Algeria	Sidi Ghiles	2000	75.9%	81.1%	70.8%
Algeria	Sidi Ghiles	2017	75.9%	81.2%	70.7%
Algeria	Sidi Hadjeres	2000	81.2%	84.2%	78.1%
Algeria	Sidi Hadjeres	2017	81.3%	84.3%	78.2%
Algeria	Sidi Hamadouche	2000	71.1%	74.6%	67.8%
Algeria	Sidi Hamadouche	2017	71.3%	74.7%	68.0%
Algeria	Sidi Hosni	2000	79.3%	81.5%	77.5%
Algeria	Sidi Hosni	2017	79.4%	81.4%	77.4%
Algeria	Sidi Kada	2000	73.7%	76.7%	70.4%
Algeria	Sidi Kada	2017	74.0%	77.1%	70.7%
Algeria	Sidi Khaled	2000	49.4%	52.1%	46.7%
Algeria	Sidi Khaled	2000	71.5%	74.3%	69.1%
Algeria	Sidi Khaled	2017	71.8%	74.5%	69.4%
Algeria	Sidi Khaled	2017	49.7%	52.4%	47.0%
Algeria	Sidi Khelifa	2000	81.3%	83.2%	79.3%
Algeria	Sidi Khelifa	2017	81.4%	83.3%	79.5%
Algeria	Sidi Khelil	2000	48.8%	52.4%	45.8%
Algeria	Sidi Khelil	2017	49.1%	52.6%	46.1%
Algeria	Sidi Khettab	2000	69.7%	72.7%	66.2%
Algeria	Sidi Khettab	2017	69.9%	72.7%	66.4%
Algeria	Sidi Khouiled	2000	48.3%	51.6%	45.5%
Algeria	Sidi Khouiled	2017	48.6%	51.7%	45.8%
Algeria	Sidi Ladjel	2000	79.4%	82.4%	75.7%
Algeria	Sidi Ladjel	2017	79.6%	82.6%	76.1%

Country	Second Administrative Unit	Year	Mean	Upper	Lower
Algeria	Sidi Lahcene	2000	71.4%	73.8%	69.2%
Algeria	Sidi Lahcene	2017	71.7%	74.0%	69.5%
Algeria	Sidi Lakhdar	2000	76.0%	79.1%	72.4%
Algeria	Sidi Lakhdar	2000	71.0%	75.5%	66.7%
Algeria	Sidi Lakhdar	2017	71.4%	75.8%	67.1%
Algeria	Sidi Lakhdar	2017	76.2%	79.2%	72.6%
Algeria	Sidi Lantri	2000	78.5%	80.8%	76.0%
Algeria	Sidi Lantri	2017	77.9%	80.1%	75.4%
Algeria	Sidi Lazreg	2000	75.0%	78.3%	72.1%
Algeria	Sidi Lazreg	2017	75.2%	78.6%	72.2%
Algeria	Sidi M'Hamed	2000	77.2%	80.6%	72.7%
Algeria	Sidi M'Hamed	2017	77.3%	80.7%	72.8%
Algeria	Sidi M'Hamed Benali	2000	72.3%	75.1%	69.6%
Algeria	Sidi M'Hamed Benali	2017	72.4%	75.3%	69.8%
Algeria	Sidi M'Hamed Benaouda	2000	71.8%	75.2%	68.6%
Algeria	Sidi M'Hamed Benaouda	2017	71.9%	75.3%	68.6%
Algeria	Sidi Makhlouf	2000	81.1%	83.7%	78.7%
Algeria	Sidi Makhlouf	2017	81.2%	84.1%	78.4%
Algeria	Sidi Marouf	2000	82.2%	84.4%	79.7%
Algeria	Sidi Marouf	2017	82.3%	84.4%	79.8%
Algeria	Sidi Medjahed	2000	67.8%	71.9%	64.0%
Algeria	Sidi Medjahed	2017	67.9%	72.1%	64.1%
Algeria	Sidi Merouane	2000	81.4%	83.7%	79.2%
Algeria	Sidi Merouane	2017	81.6%	83.9%	79.4%
Algeria	Sidi Mezghiche	2000	80.6%	82.4%	78.4%
Algeria	Sidi Mezghiche	2017	80.7%	82.4%	78.5%
Algeria	Sidi Moussa	2000	75.7%	77.7%	73.9%
Algeria	Sidi Moussa	2017	75.9%	77.8%	74.0%
Algeria	Sidi Naamane	2000	77.2%	80.0%	74.5%
Algeria	Sidi Naamane	2000	77.3%	80.8%	74.0%
Algeria	Sidi Naamane	2017	77.4%	80.9%	73.9%
Algeria	Sidi Naamane	2017	77.3%	80.1%	74.6%
Algeria	Sidi Okba	2000	48.0%	51.9%	44.0%
Algeria	Sidi Okba	2017	48.3%	52.2%	44.3%
Algeria	Sidi Ouriache|Tadmaya	2000	68.6%	72.4%	64.5%
Algeria	Sidi Ouriache|Tadmaya	2017	68.9%	72.7%	64.8%
Algeria	Sidi Rached	2000	75.0%	78.3%	71.8%
Algeria	Sidi Rached	2017	75.2%	78.5%	72.0%
Algeria	Sidi Saada	2000	70.7%	73.3%	68.1%
Algeria	Sidi Saada	2017	70.8%	73.3%	68.2%
Algeria	Sidi Safi	2000	69.1%	72.9%	65.2%
Algeria	Sidi Safi	2017	69.4%	73.0%	65.6%
Algeria	Sidi Said	2000	74.1%	77.6%	70.9%
Algeria	Sidi Said	2017	74.4%	77.8%	71.2%
Algeria	Sidi Semiane	2000	76.5%	81.1%	72.1%
Algeria	Sidi Semiane	2017	76.8%	81.3%	72.5%
Algeria	Sidi Slimane	2000	49.0%	52.0%	45.7%
Algeria	Sidi Slimane	2000	78.1%	81.2%	75.2%
Algeria	Sidi Slimane	2000	80.2%	84.0%	76.2%
Algeria	Sidi Slimane	2017	80.4%	84.1%	76.4%
Algeria	Sidi Slimane	2017	78.7%	81.7%	75.9%
Algeria	Sidi Slimane	2017	49.2%	52.2%	46.0%
Algeria	Sidi Tifour	2000	79.6%	83.6%	75.4%
Algeria	Sidi Tifour	2017	79.7%	83.8%	75.4%

Country	Second Administrative Unit	Year	Mean	Upper	Lower
Algeria	Sidi Yacoub	2000	71.3%	74.3%	68.3%
Algeria	Sidi Yacoub	2017	71.6%	74.5%	68.5%
Algeria	Sidi Zahar	2000	77.8%	81.3%	73.8%
Algeria	Sidi Zahar	2017	78.2%	81.6%	74.1%
Algeria	Sidi Ziane	2000	76.9%	80.7%	72.5%
Algeria	Sidi Ziane	2017	77.0%	80.8%	72.4%
Algeria	Sig	2000	69.4%	72.0%	67.0%
Algeria	Sig	2017	69.6%	72.2%	67.2%
Algeria	Sigous	2000	69.9%	72.4%	67.1%
Algeria	Sigous	2017	69.7%	72.4%	67.0%
Algeria	Sirat	2000	69.8%	72.2%	66.9%
Algeria	Sirat	2017	70.0%	72.4%	67.2%
Algeria	Skikda	2000	79.7%	82.3%	77.0%
Algeria	Skikda	2017	79.8%	82.4%	77.2%
Algeria	Slim	2000	81.4%	84.2%	78.2%
Algeria	Slim	2017	81.7%	84.4%	78.5%
Algeria	Smaoun	2000	73.0%	76.0%	69.2%
Algeria	Smaoun	2017	73.2%	76.2%	69.4%
Algeria	Sobha	2000	73.2%	75.7%	70.3%
Algeria	Sobha	2017	73.5%	75.9%	70.7%
Algeria	Souaflia	2000	71.4%	73.7%	68.8%
Algeria	Souaflia	2017	71.7%	74.0%	69.1%
Algeria	Souagui	2000	76.7%	80.2%	72.7%
Algeria	Souagui	2017	76.9%	80.4%	72.9%
Algeria	Souahlia	2000	67.9%	72.3%	63.4%
Algeria	Souahlia	2017	68.1%	72.5%	63.6%
Algeria	Souamaa	2000	76.7%	78.9%	74.3%
Algeria	Souamaa	2017	76.9%	79.0%	74.5%
Algeria	Souani	2000	69.7%	73.7%	65.3%
Algeria	Souani	2017	69.9%	73.9%	65.6%
Algeria	Souarekh	2000	79.2%	83.3%	74.9%
Algeria	Souarekh	2017	79.3%	83.4%	75.0%
Algeria	Sougueur	2000	78.3%	80.1%	76.2%
Algeria	Sougueur	2017	78.7%	80.4%	76.6%
Algeria	Souhan	2000	77.0%	80.1%	73.6%
Algeria	Souhan	2017	77.2%	80.3%	73.9%
Algeria	Souidania	2000	75.4%	77.4%	73.5%
Algeria	Souidania	2017	75.7%	77.5%	73.8%
Algeria	Souk Ahras	2000	80.5%	83.2%	78.1%
Algeria	Souk Ahras	2017	80.7%	83.3%	78.3%
Algeria	Souk El Had	2000	76.0%	78.7%	73.9%
Algeria	Souk El Had	2000	76.0%	78.9%	73.0%
Algeria	Souk El Had	2017	75.9%	78.8%	72.9%
Algeria	Souk El Had	2017	76.0%	78.7%	73.8%
Algeria	Souk El Khemis	2000	77.3%	80.0%	74.3%
Algeria	Souk El Khemis	2017	77.4%	80.2%	74.5%
Algeria	Souk El Tenine	2000	73.0%	76.6%	68.8%
Algeria	Souk El Tenine	2000	77.4%	79.9%	74.8%
Algeria	Souk El Tenine	2017	73.3%	76.9%	69.0%
Algeria	Souk El Tenine	2017	77.5%	80.0%	74.9%
Algeria	Souk Naamane	2000	66.4%	69.7%	63.0%
Algeria	Souk Naamane	2017	66.6%	69.9%	63.2%
Algeria	Souk Oufella	2000	75.1%	78.1%	72.4%
Algeria	Souk Oufella	2017	75.1%	78.2%	72.4%

Country	Second Administrative Unit	Year	Mean	Upper	Lower
Algeria	Souk Tleta	2000	67.9%	72.7%	63.1%
Algeria	Souk Tleta	2017	68.0%	72.9%	63.1%
Algeria	Soumaa	2000	79.8%	82.0%	77.6%
Algeria	Soumaa	2000	75.5%	77.9%	73.0%
Algeria	Soumaa	2017	75.7%	78.0%	73.2%
Algeria	Soumaa	2017	79.9%	82.1%	77.7%
Algeria	Sour	2000	71.2%	73.8%	68.6%
Algeria	Sour	2017	71.3%	74.0%	68.9%
Algeria	Sour El Ghouzlane	2000	77.1%	80.5%	73.7%
Algeria	Sour El Ghouzlane	2017	77.2%	80.6%	73.8%
Algeria	Stah Guentis	2000	62.9%	68.1%	57.9%
Algeria	Stah Guentis	2017	63.1%	68.3%	58.3%
Algeria	Staoueli	2000	74.9%	77.1%	72.4%
Algeria	Staoueli	2017	75.0%	77.3%	72.5%
Algeria	Stidia	2000	69.8%	72.4%	67.3%
Algeria	Stidia	2017	70.0%	72.6%	67.5%
Algeria	Still	2000	48.9%	52.4%	45.1%
Algeria	Still	2017	49.1%	52.5%	45.2%
Algeria	Stitten	2000	79.0%	82.1%	75.9%
Algeria	Stitten	2017	79.1%	82.2%	76.0%
Algeria	T Kout	2000	61.2%	65.8%	57.4%
Algeria	T Kout	2017	61.4%	65.9%	57.6%
Algeria	Tabelbala	2000	49.5%	57.8%	41.5%
Algeria	Tabelbala	2017	49.4%	57.1%	42.0%
Algeria	Tabia	2000	70.9%	74.0%	67.7%
Algeria	Tabia	2017	71.1%	74.3%	68.0%
Algeria	Tablat	2000	77.7%	81.2%	74.0%
Algeria	Tablat	2017	77.8%	81.3%	74.1%
Algeria	Tacheta Zegagha	2000	77.4%	81.2%	73.9%
Algeria	Tacheta Zegagha	2017	77.7%	81.6%	74.3%
Algeria	Tachouda	2000	68.6%	71.6%	65.4%
Algeria	Tachouda	2017	68.7%	71.7%	65.5%
Algeria	Tadjemout	2000	80.7%	83.1%	77.9%
Algeria	Tadjemout	2017	80.9%	83.3%	78.0%
Algeria	Tadjena	2000	76.2%	80.0%	72.4%
Algeria	Tadjena	2017	76.7%	80.5%	73.1%
Algeria	Tadjenanet	2000	71.4%	74.0%	68.6%
Algeria	Tadjenanet	2017	71.7%	74.4%	68.9%
Algeria	Tadjrouna	2000	79.4%	84.4%	74.3%
Algeria	Tadjrouna	2017	79.6%	84.6%	74.4%
Algeria	Tadmait	2000	76.5%	79.1%	73.7%
Algeria	Tadmait	2017	76.7%	79.3%	73.9%
Algeria	Tadmit	2000	81.3%	84.0%	78.6%
Algeria	Tadmit	2017	81.5%	84.4%	78.6%
Algeria	Tafissour	2000	75.2%	79.2%	70.5%
Algeria	Tafissour	2017	75.4%	79.3%	70.6%
Algeria	Tafraoui	2000	70.5%	73.4%	67.4%
Algeria	Tafraoui	2017	70.7%	73.6%	67.7%
Algeria	Tafraout	2000	77.4%	81.0%	72.6%
Algeria	Tafraout	2017	77.6%	81.1%	72.8%
Algeria	Tafreg	2000	67.9%	71.4%	64.0%
Algeria	Tafreg	2017	68.1%	71.5%	64.2%
Algeria	Tagdemt	2000	78.6%	80.5%	76.6%
Algeria	Tagdemt	2017	78.4%	80.3%	76.4%
Algeria	Taghit	2000	49.7%	56.3%	42.9%
Algeria	Taghit	2017	49.8%	56.0%	43.3%
Algeria	Taghlimet	2000	71.2%	75.1%	66.6%
Algeria	Taghlimet	2017	71.4%	75.4%	66.9%

Country	Second Administrative Unit	Year	Mean	Upper	Lower
Algeria	Taghzout	2000	76.4%	79.5%	73.3%
Algeria	Taghzout	2000	48.2%	51.4%	45.6%
Algeria	Taghzout	2017	48.5%	51.8%	45.9%
Algeria	Taghzout	2017	76.6%	79.5%	73.4%
Algeria	Taglait	2000	74.5%	77.8%	71.9%
Algeria	Taglait	2017	74.7%	78.0%	72.1%
Algeria	Taguedit	2000	78.8%	81.8%	76.2%
Algeria	Taguedit	2017	79.1%	82.0%	76.5%
Algeria	Taher	2000	80.9%	82.6%	79.3%
Algeria	Taher	2017	81.1%	82.7%	79.6%
Algeria	Taibet	2000	48.2%	52.4%	43.8%
Algeria	Taibet	2017	48.6%	52.4%	44.2%
Algeria	Takhemaret	2000	77.7%	80.1%	75.2%
Algeria	Takhemaret	2017	78.0%	80.5%	75.6%
Algeria	Tala Hamza	2000	74.7%	77.6%	71.4%
Algeria	Tala Hamza	2017	74.9%	77.7%	71.6%
Algeria	Tala-Ifacene	2000	67.8%	71.2%	64.6%
Algeria	Tala-Ifacene	2017	68.0%	71.3%	64.8%
Algeria	Taleb Larbi	2000	49.9%	56.2%	43.5%
Algeria	Taleb Larbi	2017	50.1%	56.5%	43.8%
Algeria	Talkhamt	2000	62.3%	65.9%	58.1%
Algeria	Talkhamt	2017	62.4%	66.0%	58.1%
Algeria	Talmine	2000	49.4%	57.1%	42.9%
Algeria	Talmine	2017	49.6%	57.3%	42.8%
Algeria	Tamalous	2000	80.4%	82.7%	77.7%
Algeria	Tamalous	2017	80.4%	82.8%	77.6%
Algeria	Tamantit	2000	48.8%	52.6%	44.0%
Algeria	Tamantit	2017	48.8%	54.3%	43.8%
Algeria	Tamekten	2000	48.4%	53.9%	44.0%
Algeria	Tamekten	2017	48.6%	54.2%	43.8%
Algeria	Tamelaht	2000	78.1%	80.7%	75.5%
Algeria	Tamelaht	2017	78.2%	80.7%	75.7%
Algeria	Tamenghasset	2000	48.7%	53.2%	43.6%
Algeria	Tamenghasset	2017	49.1%	53.5%	44.1%
Algeria	Tamest	2000	48.8%	54.8%	42.7%
Algeria	Tamest	2017	48.3%	53.9%	42.6%
Algeria	Tamezguida	2000	76.8%	80.0%	73.2%
Algeria	Tamezguida	2017	76.9%	80.1%	73.3%
Algeria	Tamlouka	2000	73.2%	75.9%	69.9%
Algeria	Tamlouka	2017	73.7%	76.5%	70.4%
Algeria	Tamokra	2000	70.9%	74.1%	67.3%
Algeria	Tamokra	2017	71.1%	74.3%	67.4%
Algeria	Tamridjet	2000	72.1%	75.5%	68.4%
Algeria	Tamridjet	2017	72.2%	75.6%	68.5%
Algeria	Tamsa	2000	81.2%	83.5%	78.9%
Algeria	Tamsa	2017	81.4%	83.7%	79.1%
Algeria	Tamtert	2000	49.8%	55.7%	43.9%
Algeria	Tamtert	2017	49.8%	56.0%	44.5%
Algeria	Tamza	2000	61.5%	65.0%	57.8%
Algeria	Tamza	2017	61.6%	65.1%	57.9%
Algeria	Tamzoura	2000	70.3%	73.4%	66.5%
Algeria	Tamzoura	2017	70.4%	73.5%	66.7%
Algeria	Taouala	2000	80.4%	84.3%	76.2%
Algeria	Taouala	2017	80.5%	84.3%	76.3%
Algeria	Taoudmout	2000	76.2%	80.2%	72.4%
Algeria	Taoudmout	2017	76.4%	80.3%	72.6%
Algeria	Taougrit	2000	74.4%	77.4%	71.1%
Algeria	Taougrit	2017	74.5%	77.6%	71.3%
Algeria	Taoura	2000	76.4%	79.4%	72.9%
Algeria	Taoura	2017	77.3%	80.2%	73.9%
Algeria	Taourga	2000	77.5%	80.7%	74.3%
Algeria	Taourga	2017	77.6%	80.8%	74.4%

Piped Water Proportion by Second Administrative Unit *(continued)*

Country	Second Administrative Unit	Year	Mean	Upper	Lower
Algeria	Taourit Ighil	2000	76.3%	79.7%	72.4%
Algeria	Taourit Ighil	2017	76.4%	79.6%	72.5%
Algeria	Taouzianat	2000	60.9%	64.5%	57.4%
Algeria	Taouzianat	2017	61.0%	64.7%	57.6%
Algeria	Tarik Ibn-Ziad	2000	78.1%	80.8%	75.3%
Algeria	Tarik Ibn-Ziad	2017	78.1%	80.9%	75.3%
Algeria	Tarmount	2000	79.5%	82.3%	76.4%
Algeria	Tarmount	2017	79.7%	82.5%	76.6%
Algeria	Taskriout	2000	72.3%	75.6%	68.7%
Algeria	Taskriout	2017	72.5%	75.7%	68.9%
Algeria	Tassadane Haddada	2000	78.2%	80.9%	75.7%
Algeria	Tassadane Haddada	2017	78.5%	81.1%	76.0%
Algeria	Tassala	2000	71.8%	74.5%	68.5%
Algeria	Tassala	2017	72.2%	75.0%	69.0%
Algeria	Taxlent	2000	62.8%	66.5%	59.2%
Algeria	Taxlent	2017	62.9%	66.7%	59.4%
Algeria	Taya	2000	65.2%	69.1%	61.2%
Algeria	Taya	2017	65.5%	69.5%	61.6%
Algeria	Tazgait	2000	71.6%	75.6%	68.1%
Algeria	Tazgait	2017	71.8%	75.8%	68.2%
Algeria	Tazmalt	2000	72.5%	76.6%	68.7%
Algeria	Tazmalt	2017	72.7%	76.7%	68.9%
Algeria	Tazoult	2000	61.5%	64.8%	58.8%
Algeria	Tazoult	2017	62.0%	65.0%	59.2%
Algeria	Tazrouk	2000	48.1%	54.7%	41.0%
Algeria	Tazrouk	2017	46.7%	53.0%	40.5%
Algeria	Tebesbest	2000	48.9%	51.8%	45.8%
Algeria	Tebesbest	2017	49.1%	52.1%	46.0%
Algeria	Tebessa	2000	62.4%	65.4%	59.5%
Algeria	Tebessa	2017	62.6%	65.6%	59.7%
Algeria	Telaa	2000	61.9%	65.2%	58.0%
Algeria	Telaa	2017	62.1%	65.5%	58.2%
Algeria	Telagh	2000	71.5%	75.4%	67.0%
Algeria	Telagh	2017	71.7%	75.7%	67.1%
Algeria	Telassa	2000	74.6%	79.5%	69.6%
Algeria	Telassa	2017	74.8%	79.7%	69.9%
Algeria	Teleghma	2000	74.7%	77.5%	72.0%
Algeria	Teleghma	2017	74.7%	77.5%	71.9%
Algeria	Temacine	2000	48.8%	52.2%	45.0%
Algeria	Temacine	2017	49.0%	52.7%	45.0%
Algeria	Tenedla	2000	49.0%	52.8%	45.6%
Algeria	Tenedla	2017	49.3%	53.0%	45.8%
Algeria	Tenes	2000	75.8%	81.2%	70.5%
Algeria	Tenes	2017	75.9%	81.3%	70.6%
Algeria	Teniet El Abed	2000	62.8%	66.6%	59.2%
Algeria	Teniet El Abed	2017	63.0%	66.8%	59.4%
Algeria	Teniet En Nasr	2000	70.2%	73.9%	66.1%
Algeria	Teniet En Nasr	2017	70.6%	74.3%	66.5%
Algeria	Tenira	2000	71.0%	74.6%	67.0%
Algeria	Tenira	2017	71.1%	74.7%	67.1%
Algeria	Terga	2000	69.5%	72.7%	65.7%
Algeria	Terga	2017	69.8%	73.1%	66.0%
Algeria	Terny Beni Hediel	2000	72.2%	74.4%	69.7%

Country	Second Administrative Unit	Year	Mean	Upper	Lower
Algeria	Terny Beni Hediel	2017	72.4%	74.5%	69.9%
Algeria	Terraguelt	2000	67.1%	71.7%	62.6%
Algeria	Terraguelt	2017	67.5%	72.0%	63.0%
Algeria	Terrai Bainnane	2000	83.0%	85.0%	81.2%
Algeria	Terrai Bainnane	2017	83.1%	85.1%	81.3%
Algeria	Tesmart	2000	65.7%	68.9%	62.2%
Algeria	Tesmart	2017	65.9%	69.1%	62.4%
Algeria	Tessala Lamtai	2000	82.2%	84.6%	80.0%
Algeria	Tessala Lamtai	2017	82.4%	84.7%	80.2%
Algeria	Tessala-El-Merdja	2000	75.1%	77.0%	73.3%
Algeria	Tessala-El-Merdja	2017	75.4%	77.3%	73.6%
Algeria	Texenna	2000	80.9%	83.3%	78.6%
Algeria	Texenna	2017	81.2%	83.6%	78.8%
Algeria	Thelidjene	2000	62.6%	67.1%	58.2%
Algeria	Thelidjene	2017	62.8%	67.2%	58.4%
Algeria	Thenia	2000	76.6%	79.4%	74.0%
Algeria	Thenia	2017	76.7%	79.6%	74.1%
Algeria	Theniet El Had	2000	79.1%	81.4%	76.7%
Algeria	Theniet El Had	2017	79.5%	81.7%	77.1%
Algeria	Thleth Douair	2000	77.8%	81.2%	74.0%
Algeria	Thleth Douair	2017	78.1%	81.5%	74.3%
Algeria	Tianet	2000	67.8%	71.5%	63.9%
Algeria	Tianet	2017	67.9%	71.6%	64.0%
Algeria	Tiaret	2000	79.1%	80.7%	77.5%
Algeria	Tiaret	2017	79.2%	80.8%	77.8%
Algeria	Tiberguent	2000	79.7%	81.5%	78.0%
Algeria	Tiberguent	2017	79.9%	81.7%	78.2%
Algeria	Tiberkanine	2000	76.1%	78.8%	73.6%
Algeria	Tiberkanine	2017	76.5%	79.1%	73.9%
Algeria	Tichy	2000	74.0%	77.1%	70.3%
Algeria	Tichy	2017	74.3%	77.4%	70.6%
Algeria	Tidda	2000	77.4%	79.6%	75.0%
Algeria	Tidda	2017	77.6%	79.7%	75.1%
Algeria	Tidjelabine	2000	76.1%	79.0%	73.9%
Algeria	Tidjelabine	2017	76.3%	79.2%	74.1%
Algeria	Tiffech	2000	78.8%	81.0%	76.5%
Algeria	Tiffech	2017	79.0%	81.2%	76.6%
Algeria	Tifra	2000	76.1%	79.1%	73.0%
Algeria	Tifra	2017	76.3%	79.2%	73.1%
Algeria	Tighanimine	2000	61.5%	65.3%	57.8%
Algeria	Tighanimine	2017	61.5%	65.2%	57.9%
Algeria	Tigharghar	2000	56.5%	60.3%	52.5%
Algeria	Tigharghar	2017	56.6%	60.5%	52.6%
Algeria	Tighenif	2000	72.9%	75.6%	69.6%
Algeria	Tighenif	2017	73.1%	75.8%	69.8%
Algeria	Tigzirt	2000	77.1%	80.7%	72.4%
Algeria	Tigzirt	2017	76.9%	80.6%	72.3%
Algeria	Tilatou	2000	58.9%	61.9%	56.3%
Algeria	Tilatou	2017	59.3%	62.5%	56.6%
Algeria	Tilmouni	2000	71.6%	74.6%	68.9%
Algeria	Tilmouni	2017	71.9%	74.7%	69.2%
Algeria	Timezrit	2000	77.3%	79.9%	74.2%
Algeria	Timezrit	2000	74.4%	77.8%	71.0%

Country	Second Administrative Unit	Year	Mean	Upper	Lower
Algeria	Timezrit	2017	74.7%	78.0%	71.2%
Algeria	Timezrit	2017	77.5%	80.1%	74.4%
Algeria	Timgad	2000	60.4%	63.7%	57.2%
Algeria	Timgad	2017	60.3%	63.9%	57.0%
Algeria	Timiaouine	2000	50.4%	59.9%	40.9%
Algeria	Timiaouine	2017	50.2%	58.8%	40.5%
Algeria	Timizart	2000	78.4%	81.5%	75.4%
Algeria	Timizart	2017	78.6%	81.7%	75.7%
Algeria	Timmimoun	2000	49.7%	53.8%	45.0%
Algeria	Timmimoun	2017	49.9%	53.7%	45.5%
Algeria	Timoudi	2000	49.5%	57.4%	42.0%
Algeria	Timoudi	2017	49.3%	56.6%	42.8%
Algeria	Tin Zaouatine	2000	47.2%	55.7%	37.6%
Algeria	Tin Zaouatine	2017	46.9%	55.0%	37.9%
Algeria	Tindouf	2000	50.0%	55.4%	44.9%
Algeria	Tindouf	2017	50.3%	55.6%	45.1%
Algeria	Tinedbar	2000	74.3%	77.6%	71.2%
Algeria	Tinedbar	2017	74.4%	77.7%	71.3%
Algeria	Tinerkouk	2000	50.1%	54.5%	45.6%
Algeria	Tinerkouk	2017	50.4%	55.4%	45.5%
Algeria	Tiout	2000	79.1%	81.8%	76.3%
Algeria	Tiout	2017	79.2%	82.0%	76.3%
Algeria	Tipaza	2000	75.4%	79.4%	72.0%
Algeria	Tipaza	2017	75.6%	79.6%	72.2%
Algeria	Tircine	2000	78.2%	81.2%	75.6%
Algeria	Tircine	2017	78.2%	81.1%	75.7%
Algeria	Tirmitine	2000	76.6%	78.9%	74.2%
Algeria	Tirmitine	2017	76.8%	79.1%	74.5%
Algeria	Tissemsilt	2000	79.1%	80.7%	77.3%
Algeria	Tissemsilt	2017	79.2%	80.9%	77.5%
Algeria	Tit	2000	48.8%	55.9%	41.4%
Algeria	Tit	2017	48.9%	55.9%	41.8%
Algeria	Tixter	2000	62.9%	66.0%	59.8%
Algeria	Tixter	2017	63.0%	66.1%	59.9%
Algeria	Tizi	2000	71.1%	73.6%	68.3%
Algeria	Tizi	2017	71.4%	73.9%	68.7%
Algeria	Tizi Mahdi	2000	76.9%	79.8%	73.3%
Algeria	Tizi Mahdi	2017	77.1%	80.0%	73.4%
Algeria	Tizi N'Bechar	2000	66.6%	69.8%	62.9%
Algeria	Tizi N'Bechar	2017	67.0%	70.2%	63.3%
Algeria	Tizi N'Tleta	2000	78.0%	80.7%	75.1%
Algeria	Tizi N'Tleta	2017	78.2%	80.9%	75.3%
Algeria	Tizi Ouzou	2000	76.7%	79.0%	74.9%
Algeria	Tizi Ouzou	2017	76.9%	79.2%	75.1%
Algeria	Tizi-Ghenif	2000	77.1%	79.8%	74.3%
Algeria	Tizi-Ghenif	2017	77.3%	80.0%	74.5%
Algeria	Tizi-N'Berber	2000	72.7%	75.8%	69.1%
Algeria	Tizi-N'Berber	2017	73.0%	76.1%	69.3%
Algeria	Tizi-Rached	2000	76.6%	79.0%	74.2%
Algeria	Tizi-Rached	2017	76.8%	79.2%	74.4%
Algeria	Tlemcen	2000	70.5%	72.4%	68.1%
Algeria	Tlemcen	2017	70.7%	72.6%	68.4%
Algeria	Tolga	2000	50.5%	53.5%	47.4%
Algeria	Tolga	2017	50.8%	53.9%	47.7%
Algeria	Touahria	2000	70.3%	72.5%	67.6%
Algeria	Touahria	2017	70.5%	72.7%	67.8%
Algeria	Toudja	2000	76.9%	80.5%	73.1%
Algeria	Toudja	2017	77.1%	80.7%	73.3%
Algeria	Touggourt	2000	48.9%	51.9%	45.8%
Algeria	Touggourt	2017	49.2%	52.1%	46.1%
Algeria	Tousmouline	2000	77.8%	81.4%	73.6%
Algeria	Tousmouline	2017	78.1%	81.7%	74.2%

Country	Second Administrative Unit	Year	Mean	Upper	Lower
Algeria	Tousnina	2000	77.9%	80.3%	75.6%
Algeria	Tousnina	2017	78.2%	80.5%	75.8%
Algeria	Treat	2000	80.6%	83.7%	77.8%
Algeria	Treat	2017	80.7%	83.7%	77.9%
Algeria	Trifaoui	2000	48.6%	52.0%	45.2%
Algeria	Trifaoui	2017	48.9%	52.3%	45.3%
Algeria	Tsabit	2000	49.1%	54.0%	43.5%
Algeria	Tsabit	2017	49.3%	54.9%	43.5%
Algeria	Yabous	2000	60.9%	64.9%	57.2%
Algeria	Yabous	2017	61.1%	65.2%	57.4%
Algeria	Yahia Beniguecha	2000	78.4%	80.8%	76.2%
Algeria	Yahia Beniguecha	2017	78.8%	81.2%	76.7%
Algeria	Yakourene	2000	79.4%	82.1%	76.3%
Algeria	Yakourene	2017	79.5%	82.2%	76.5%
Algeria	Yatafene	2000	77.1%	80.2%	73.6%
Algeria	Yatafene	2017	77.3%	80.4%	73.8%
Algeria	Yellel	2000	70.6%	73.2%	67.7%
Algeria	Yellel	2017	70.7%	73.2%	67.8%
Algeria	Youb	2000	73.5%	77.9%	69.6%
Algeria	Youb	2017	73.6%	78.0%	69.6%
Algeria	Zaafrane	2000	80.9%	83.4%	78.3%
Algeria	Zaafrane	2017	81.2%	83.6%	78.7%
Algeria	Zaarouria	2000	80.1%	82.6%	77.6%
Algeria	Zaarouria	2017	80.4%	82.9%	77.8%
Algeria	Zaccar	2000	81.6%	83.9%	79.2%
Algeria	Zaccar	2017	81.6%	83.9%	79.3%
Algeria	Zahana	2000	70.1%	73.7%	67.5%
Algeria	Zahana	2017	70.3%	73.8%	67.8%
Algeria	Zanet El Beida	2000	65.6%	68.9%	62.0%
Algeria	Zanet El Beida	2017	66.0%	69.2%	62.2%
Algeria	Zaouia El Abidia	2000	48.7%	51.7%	45.6%
Algeria	Zaouia El Abidia	2017	49.0%	52.0%	46.0%
Algeria	Zaouiet Kounta	2000	49.0%	55.7%	42.6%
Algeria	Zaouiet Kounta	2017	48.8%	55.1%	43.2%
Algeria	Zarzour	2000	74.3%	78.6%	69.9%
Algeria	Zarzour	2017	74.5%	78.7%	70.0%
Algeria	Zbarbar|El Isseri	2000	77.1%	80.3%	74.1%
Algeria	Zbarbar|El Isseri	2017	77.3%	80.5%	74.3%
Algeria	Zeboudja	2000	77.3%	81.4%	73.4%
Algeria	Zeboudja	2017	77.4%	81.4%	73.7%
Algeria	Zeddine	2000	76.1%	78.8%	73.3%
Algeria	Zeddine	2017	76.4%	79.1%	73.6%
Algeria	Zeghaia	2000	81.3%	83.1%	79.5%
Algeria	Zeghaia	2017	81.5%	83.3%	79.8%
Algeria	Zekri	2000	78.7%	82.1%	74.8%
Algeria	Zekri	2017	78.8%	82.2%	74.9%
Algeria	Zelfana	2000	47.6%	52.2%	43.1%
Algeria	Zelfana	2017	47.8%	52.5%	43.3%
Algeria	Zelmata	2000	75.6%	79.2%	72.6%
Algeria	Zelmata	2017	75.8%	79.4%	72.8%
Algeria	Zemmoura	2000	71.7%	74.6%	68.9%
Algeria	Zemmoura	2017	71.9%	74.9%	69.0%

Country	Second Administrative Unit	Year	Mean	Upper	Lower
Algeria	Zemmouri	2000	76.1%	79.1%	72.7%
Algeria	Zemmouri	2017	76.4%	79.3%	73.0%
Algeria	Zenata	2000	67.7%	70.8%	64.1%
Algeria	Zenata	2017	67.9%	71.0%	64.4%
Algeria	Zeralda	2000	75.0%	77.4%	72.5%
Algeria	Zeralda	2017	75.2%	77.6%	72.8%
Algeria	Zerdeza	2000	81.2%	83.2%	79.0%
Algeria	Zerdeza	2017	81.4%	83.4%	79.2%
Algeria	Zeribet El Oued	2000	51.5%	57.7%	45.6%
Algeria	Zeribet El Oued	2017	51.6%	57.9%	45.5%
Algeria	Zerizer	2000	80.0%	82.5%	77.1%
Algeria	Zerizer	2017	80.2%	82.6%	77.3%
Algeria	Zerouala	2000	71.2%	74.7%	68.0%
Algeria	Zerouala	2017	71.4%	75.0%	68.2%
Algeria	Ziama Mansouria	2000	75.3%	78.7%	71.8%
Algeria	Ziama Mansouria	2017	75.6%	79.1%	72.2%
Algeria	Zighoud Youcef	2000	82.1%	84.1%	80.1%
Algeria	Zighoud Youcef	2017	82.2%	84.2%	80.2%
Algeria	Zitouna	2000	80.5%	83.3%	77.5%
Algeria	Zitouna	2000	83.4%	86.5%	80.1%
Algeria	Zitouna	2017	83.4%	86.4%	80.2%
Algeria	Zitouna	2017	80.4%	83.1%	77.3%
Algeria	Zmalet El Emir Abdelkader	2000	79.2%	81.8%	76.0%
Algeria	Zmalet El Emir Abdelkader	2017	79.4%	82.1%	76.2%
Algeria	Zorg	2000	65.4%	69.3%	60.6%
Algeria	Zorg	2017	65.5%	69.4%	60.7%
Algeria	Zouabi	2000	74.1%	78.3%	70.1%
Algeria	Zouabi	2017	74.3%	78.6%	70.5%
Algeria	Zoubiria	2000	77.0%	80.3%	73.5%
Algeria	Zoubiria	2017	77.4%	80.8%	73.9%
Egypt	’Abdin	2000	98.1%	98.7%	97.0%
Egypt	’Abdin	2017	100.0%	100.0%	100.0%
Egypt	’Ain Schams	2000	99.1%	99.5%	98.5%
Egypt	’Ain Schams	2017	100.0%	100.0%	100.0%
Egypt	’Ataqah	2000	96.2%	99.3%	89.9%
Egypt	’Ataqah	2017	99.9%	100.0%	99.1%
Egypt	10 Ramadan 1	2000	89.3%	100.0%	9.3%
Egypt	10 Ramadan 1	2017	99.7%	100.0%	98.5%
Egypt	10 Ramadan 2	2000	87.5%	100.0%	16.9%
Egypt	10 Ramadan 2	2017	99.4%	100.0%	94.2%
Egypt	15 Mayu	2000	99.6%	100.0%	97.9%
Egypt	15 Mayu	2017	100.0%	100.0%	100.0%
Egypt	Abnub	2000	93.9%	96.4%	88.3%
Egypt	Abnub	2017	100.0%	100.0%	100.0%
Egypt	Abu al-Matamir	2000	90.0%	97.7%	75.7%
Egypt	Abu al-Matamir	2017	99.9%	100.0%	98.7%
Egypt	Abu Hammad	2000	63.8%	64.7%	62.8%
Egypt	Abu Hammad	2017	72.6%	74.5%	71.2%
Egypt	Abu Hummus	2000	99.1%	99.9%	93.7%
Egypt	Abu Hummus	2017	100.0%	100.0%	100.0%

Country	Second Administrative Unit	Year	Mean	Upper	Lower
Egypt	Abu Kabir	2000	60.2%	62.2%	57.7%
Egypt	Abu Kabir	2017	76.0%	78.7%	73.5%
Egypt	Abu Qurqas	2000	69.6%	73.4%	64.2%
Egypt	Abu Qurqas	2017	99.9%	100.0%	99.8%
Egypt	Abu Radis	2000	82.1%	94.6%	54.1%
Egypt	Abu Radis	2017	98.1%	99.9%	92.9%
Egypt	Abu Tij	2000	96.2%	98.7%	90.0%
Egypt	Abu Tij	2017	100.0%	100.0%	100.0%
Egypt	Abu Tisht	2000	60.9%	64.8%	57.0%
Egypt	Abu Tisht	2017	98.9%	99.8%	97.7%
Egypt	Abu Zenima	2000	92.4%	100.0%	16.0%
Egypt	Abu Zenima	2017	100.0%	100.0%	99.5%
Egypt	Ad-Dab'ah	2000	90.1%	97.2%	79.4%
Egypt	Ad-Dab'ah	2017	99.9%	100.0%	99.0%
Egypt	Ad-Darb al-Ahmar	2000	99.4%	99.7%	98.9%
Egypt	Ad-Darb al-Ahmar	2017	100.0%	100.0%	100.0%
Egypt	Ad-Dawahy	2000	98.9%	99.8%	96.7%
Egypt	Ad-Dawahy	2017	100.0%	100.0%	100.0%
Egypt	Ad-Dilinat	2000	91.7%	96.1%	83.6%
Egypt	Ad-Dilinat	2017	99.5%	100.0%	97.7%
Egypt	Ad-Dukhaylah	2000	99.5%	99.9%	98.4%
Egypt	Ad-Dukhaylah	2017	100.0%	100.0%	100.0%
Egypt	Ad-Duqi	2000	97.6%	98.3%	96.6%
Egypt	Ad-Duqi	2017	100.0%	100.0%	100.0%
Egypt	Aja	2000	82.9%	85.4%	80.1%
Egypt	Aja	2017	100.0%	100.0%	100.0%
Egypt	Akhmim	2000	88.5%	92.5%	82.4%
Egypt	Akhmim	2017	99.9%	100.0%	99.8%
Egypt	Al-'Ajuzah	2000	95.6%	97.1%	93.5%
Egypt	Al-'Ajuzah	2017	100.0%	100.0%	100.0%
Egypt	Al-'Amriyah	2000	97.3%	99.8%	89.9%
Egypt	Al-'Amriyah	2017	99.9%	100.0%	99.5%
Egypt	Al-'Arab	2000	99.6%	99.9%	98.8%
Egypt	Al-'Arab	2017	100.0%	100.0%	100.0%
Egypt	Al-'Arish 1	2000	98.7%	99.9%	93.3%
Egypt	Al-'Arish 1	2017	100.0%	100.0%	100.0%
Egypt	Al-'Arish 2	2000	97.9%	100.0%	84.7%
Egypt	Al-'Arish 2	2017	99.9%	100.0%	99.9%
Egypt	Al-'Arish 3	2000	92.4%	100.0%	51.6%
Egypt	Al-'Arish 3	2017	99.8%	100.0%	98.2%
Egypt	Al-'Arish 4	2000	91.1%	100.0%	52.3%
Egypt	Al-'Arish 4	2017	99.5%	100.0%	98.1%
Egypt	Al-'Atarin	2000	99.4%	99.8%	98.2%
Egypt	Al-'Atarin	2017	100.0%	100.0%	100.0%
Egypt	Al-'Ayyat	2000	47.7%	52.6%	42.7%
Egypt	Al-'Ayyat	2017	96.7%	98.4%	94.6%
Egypt	Al-'Idwah	2000	93.8%	95.1%	91.9%
Egypt	Al-'Idwah	2017	100.0%	100.0%	100.0%
Egypt	Al-'Ubur	2000	83.4%	97.1%	63.6%
Egypt	Al-'Ubur	2017	100.0%	100.0%	99.8%
Egypt	Al-'Umraniyah	2000	98.4%	99.1%	97.3%
Egypt	Al-'Umraniyah	2017	100.0%	100.0%	100.0%
Egypt	Al-'Usayrat	2000	16.2%	20.5%	12.6%
Egypt	Al-'Usayrat	2017	99.2%	99.5%	98.8%
Egypt	Al-Ahram	2000	93.1%	97.0%	85.7%
Egypt	Al-Ahram	2017	99.8%	99.8%	99.8%

Country	Second Administrative Unit	Year	Mean	Upper	Lower
Egypt	Al-Arb'in	2000	99.7%	99.9%	99.0%
Egypt	Al-Arb'in	2017	100.0%	100.0%	100.0%
Egypt	Al-Azbakiyah	2000	98.2%	98.9%	97.3%
Egypt	Al-Azbakiyah	2017	100.0%	100.0%	100.0%
Egypt	Al-Badari	2000	75.1%	86.2%	62.8%
Egypt	Al-Badari	2017	99.9%	100.0%	99.9%
Egypt	Al-Badrashayn	2000	69.6%	74.0%	64.7%
Egypt	Al-Badrashayn	2017	99.4%	99.5%	99.1%
Egypt	Al-Bajur	2000	72.8%	76.8%	68.3%
Egypt	Al-Bajur	2017	100.0%	100.0%	100.0%
Egypt	Al-Baliyana	2000	69.8%	75.2%	65.4%
Egypt	Al-Baliyana	2017	99.8%	100.0%	99.3%
Egypt	Al-Basatin	2000	99.7%	99.8%	99.3%
Egypt	Al-Basatin	2017	100.0%	100.0%	100.0%
Egypt	Al-Burulus	2000	30.1%	48.0%	15.7%
Egypt	Al-Burulus	2017	96.7%	99.2%	89.9%
Egypt	Al-Fashn	2000	90.1%	92.6%	86.9%
Egypt	Al-Fashn	2017	100.0%	100.0%	100.0%
Egypt	Al-Fath	2000	98.0%	98.9%	96.6%
Egypt	Al-Fath	2017	100.0%	100.0%	100.0%
Egypt	Al-Fayyum	2000	99.2%	99.7%	97.9%
Egypt	Al-Fayyum	2017	100.0%	100.0%	100.0%
Egypt	Al-Fayyum City	2000	99.6%	100.0%	97.9%
Egypt	Al-Fayyum City	2017	100.0%	100.0%	100.0%
Egypt	Al-Ganoub	2000	92.7%	98.0%	82.9%
Egypt	Al-Ganoub	2017	99.8%	100.0%	99.0%
Egypt	Al-Ganoub 2	2000	81.8%	87.9%	72.8%
Egypt	Al-Ganoub 2	2017	99.9%	100.0%	99.5%
Egypt	Al-Ghanayim	2000	88.6%	91.4%	85.5%
Egypt	Al-Ghanayim	2017	100.0%	100.0%	100.0%
Egypt	Al-Ghurdaqah	2000	79.1%	88.4%	68.1%
Egypt	Al-Ghurdaqah	2017	98.8%	100.0%	92.0%
Egypt	Al-Ghurdaqah 2	2000	72.4%	94.3%	42.2%
Egypt	Al-Ghurdaqah 2	2017	99.3%	100.0%	95.2%
Egypt	Al-Hammam	2000	90.9%	98.0%	72.5%
Egypt	Al-Hammam	2017	99.9%	100.0%	98.9%
Egypt	Al-Hamul	2000	66.6%	76.5%	57.1%
Egypt	Al-Hamul	2017	99.0%	99.9%	95.7%
Egypt	Al-Hasanah	2000	86.6%	96.7%	63.9%
Egypt	Al-Hasanah	2017	99.2%	100.0%	96.1%
Egypt	Al-Hawamidiyah	2000	86.1%	87.7%	84.0%
Egypt	Al-Hawamidiyah	2017	99.7%	99.8%	99.4%
Egypt	Al-Husayniyah	2000	66.5%	74.6%	61.2%
Egypt	Al-Husayniyah	2017	95.1%	99.2%	87.8%
Egypt	Al-Ibrahimiyah	2000	63.8%	65.8%	61.7%
Egypt	Al-Ibrahimiyah	2017	99.8%	99.9%	99.8%

Country	Second Administrative Unit	Year	Mean	Upper	Lower
Egypt	Al-Jamaliyah	2000	96.0%	98.9%	90.6%
Egypt	Al-Jamaliyah	2000	99.4%	99.7%	98.8%
Egypt	Al-Jamaliyah	2017	100.0%	100.0%	100.0%
Egypt	Al-Jamaliyah	2017	100.0%	100.0%	100.0%
Egypt	Al-Janayin	2000	96.3%	99.9%	82.5%
Egypt	Al-Janayin	2017	99.9%	100.0%	99.2%
Egypt	Al-Jumruk	2000	99.5%	99.9%	98.5%
Egypt	Al-Jumruk	2017	100.0%	100.0%	100.0%
Egypt	Al-Kawtar	2000	93.3%	96.1%	89.8%
Egypt	Al-Kawtar	2017	100.0%	100.0%	100.0%
Egypt	Al-Khalifa	2000	99.5%	99.7%	99.1%
Egypt	Al-Khalifa	2017	100.0%	100.0%	100.0%
Egypt	Al-Khankah	2000	80.5%	84.0%	77.1%
Egypt	Al-Khankah	2017	99.9%	99.9%	99.9%
Egypt	Al-Khusus	2000	99.4%	99.7%	99.0%
Egypt	Al-Khusus	2017	100.0%	100.0%	100.0%
Egypt	Al-Laban	2000	99.4%	99.8%	98.2%
Egypt	Al-Laban	2017	100.0%	100.0%	100.0%
Egypt	Al-Ma'adi	2000	99.3%	99.7%	98.3%
Egypt	Al-Ma'adi	2017	100.0%	100.0%	100.0%
Egypt	Al-Mahallah al-Kubra	2000	90.0%	91.6%	88.1%
Egypt	Al-Mahallah al-Kubra	2017	100.0%	100.0%	100.0%
Egypt	Al-Mahallah al-Kubra 1	2000	94.5%	96.3%	92.6%
Egypt	Al-Mahallah al-Kubra 1	2017	100.0%	100.0%	100.0%
Egypt	Al-Mahallah al-Kubra 2	2000	92.5%	94.6%	90.2%
Egypt	Al-Mahallah al-Kubra 2	2017	100.0%	100.0%	100.0%
Egypt	Al-Mahmudiyah	2000	97.2%	99.1%	91.2%
Egypt	Al-Mahmudiyah	2017	100.0%	100.0%	100.0%
Egypt	Al-Manakh	2000	99.6%	99.9%	98.9%
Egypt	Al-Manakh	2017	100.0%	100.0%	100.0%
Egypt	Al-Manasrah	2000	99.3%	99.8%	98.2%
Egypt	Al-Manasrah	2017	100.0%	100.0%	100.0%
Egypt	Al-Manshah	2000	65.3%	71.9%	56.4%
Egypt	Al-Manshah	2017	99.9%	99.9%	99.6%
Egypt	Al-Manshiyah	2000	99.4%	99.8%	98.2%
Egypt	Al-Manshiyah	2017	100.0%	100.0%	100.0%
Egypt	Al-Mansurah	2000	91.9%	93.5%	89.4%
Egypt	Al-Mansurah	2017	100.0%	100.0%	100.0%
Egypt	Al-Mansurah 1	2000	93.7%	95.7%	91.3%
Egypt	Al-Mansurah 1	2017	100.0%	100.0%	100.0%
Egypt	Al-Mansurah 2	2000	94.2%	96.2%	91.8%
Egypt	Al-Mansurah 2	2017	100.0%	100.0%	100.0%
Egypt	Al-Manzilah	2000	83.6%	94.9%	65.0%
Egypt	Al-Manzilah	2017	99.5%	100.0%	97.7%
Egypt	Al-Maraghah	2000	83.1%	87.2%	78.3%
Egypt	Al-Maraghah	2017	100.0%	100.0%	100.0%
Egypt	Al-Marj	2000	98.9%	99.5%	98.1%
Egypt	Al-Marj	2017	100.0%	100.0%	100.0%
Egypt	Al-Matariyah	2000	97.5%	99.6%	90.7%
Egypt	Al-Matariyah	2000	99.5%	99.7%	99.2%

Country	Second Administrative Unit	Year	Mean	Upper	Lower
Egypt	Al-Matariyah	2017	100.0%	100.0%	100.0%
Egypt	Al-Matariyah	2017	100.0%	100.0%	100.0%
Egypt	Al-Minya	2000	74.7%	78.8%	69.6%
Egypt	Al-Minya	2017	99.9%	99.9%	99.8%
Egypt	Al-Minya City	2000	95.6%	96.7%	94.5%
Egypt	Al-Minya City	2017	100.0%	100.0%	100.0%
Egypt	Al-Muntazah	2000	98.8%	99.4%	97.8%
Egypt	Al-Muntazah	2017	100.0%	100.0%	100.0%
Egypt	Al-Muski	2000	99.3%	99.6%	98.7%
Egypt	Al-Muski	2017	100.0%	100.0%	100.0%
Egypt	Al-Qanatir al-Khayriyah	2000	87.7%	89.6%	85.3%
Egypt	Al-Qanatir al-Khayriyah	2017	100.0%	100.0%	100.0%
Egypt	Al-Qanayat	2000	16.4%	20.0%	13.6%
Egypt	Al-Qanayat	2017	99.3%	99.6%	98.8%
Egypt	Al-Qantarah	2000	83.1%	87.4%	77.1%
Egypt	Al-Qantarah	2017	99.9%	100.0%	99.8%
Egypt	Al-Qantarah ash-Sharqiyah	2000	92.5%	97.3%	81.6%
Egypt	Al-Qantarah ash-Sharqiyah	2017	99.8%	100.0%	99.2%
Egypt	Al-Qurayn	2000	3.0%	9.8%	0.2%
Egypt	Al-Qurayn	2017	10.2%	10.4%	9.6%
Egypt	Al-Qusayr	2000	90.0%	96.8%	79.9%
Egypt	Al-Qusayr	2017	99.6%	100.0%	98.0%
Egypt	Al-Qusiyah	2000	78.7%	82.8%	73.6%
Egypt	Al-Qusiyah	2017	100.0%	100.0%	99.9%
Egypt	Al-Wahat al-Bahariyah	2000	82.0%	99.0%	21.1%
Egypt	Al-Wahat al-Bahariyah	2017	98.5%	100.0%	89.8%
Egypt	Al-Wahat al-Kharijah	2000	89.1%	94.6%	80.6%
Egypt	Al-Wahat al-Kharijah	2017	99.8%	100.0%	98.8%
Egypt	Al-Waili	2000	99.4%	99.7%	98.8%
Egypt	Al-Waili	2017	100.0%	100.0%	100.0%
Egypt	Al-Waqf	2000	91.6%	95.2%	86.6%
Egypt	Al-Waqf	2017	100.0%	100.0%	99.9%
Egypt	Al-Warraq	2000	87.0%	88.8%	85.4%
Egypt	Al-Warraq	2017	100.0%	100.0%	100.0%
Egypt	Al-Wasta	2000	75.8%	79.8%	71.8%
Egypt	Al-Wasta	2017	99.9%	99.9%	99.9%
Egypt	An-Nuzhah	2000	97.9%	99.0%	95.8%
Egypt	An-Nuzhah	2017	100.0%	100.0%	100.0%
Egypt	Ancient Cairo	2000	99.3%	99.5%	99.0%
Egypt	Ancient Cairo	2017	100.0%	100.0%	100.0%
Egypt	Ar-Rahmaniyah	2000	92.5%	97.3%	83.3%
Egypt	Ar-Rahmaniyah	2017	100.0%	100.0%	100.0%
Egypt	Ar-Raml 1	2000	99.2%	99.7%	98.3%
Egypt	Ar-Raml 1	2017	100.0%	100.0%	100.0%
Egypt	Ar-Raml 2	2000	99.3%	99.7%	98.6%
Egypt	Ar-Raml 2	2017	100.0%	100.0%	100.0%
Egypt	Ar-Riyad	2000	77.8%	83.0%	68.6%
Egypt	Ar-Riyad	2017	91.4%	94.3%	89.5%
Egypt	Armant	2000	89.5%	93.9%	80.5%
Egypt	Armant	2017	100.0%	100.0%	100.0%
Egypt	As-Saff	2000	51.7%	57.3%	44.1%
Egypt	As-Saff	2017	98.1%	99.8%	92.3%

Country	Second Administrative Unit	Year	Mean	Upper	Lower
Egypt	As-Sajil	2000	99.6%	99.8%	99.4%
Egypt	As-Sajil	2017	100.0%	100.0%	100.0%
Egypt	As-Salam	2000	99.2%	99.7%	98.3%
Egypt	As-Salam	2017	100.0%	100.0%	100.0%
Egypt	As-Salum	2000	38.4%	55.0%	19.3%
Egypt	As-Salum	2017	97.0%	98.9%	91.9%
Egypt	As-Santah	2000	85.6%	91.9%	75.5%
Egypt	As-Santah	2017	100.0%	100.0%	99.9%
Egypt	As-Sayidah Zaynab	2000	99.2%	99.4%	98.8%
Egypt	As-Sayidah Zaynab	2017	100.0%	100.0%	100.0%
Egypt	As-Sinbillawayn	2000	29.6%	33.4%	26.2%
Egypt	As-Sinbillawayn	2017	94.8%	99.5%	87.4%
Egypt	Ash-Shalatin	2000	81.5%	90.3%	66.8%
Egypt	Ash-Shalatin	2017	98.7%	99.8%	95.6%
Egypt	Ash-Sharabiyah	2000	99.5%	99.8%	99.2%
Egypt	Ash-Sharabiyah	2017	100.0%	100.0%	100.0%
Egypt	Ash-Sharq	2000	99.5%	99.9%	98.4%
Egypt	Ash-Sharq	2017	100.0%	100.0%	100.0%
Egypt	Ash-Shruq	2000	94.1%	99.8%	71.9%
Egypt	Ash-Shruq	2017	99.7%	100.0%	96.9%
Egypt	Ash-Shuhada	2000	86.6%	89.5%	83.3%
Egypt	Ash-Shuhada	2017	100.0%	100.0%	100.0%
Egypt	Ashmun	2000	89.0%	91.1%	86.5%
Egypt	Ashmun	2017	100.0%	100.0%	100.0%
Egypt	Aswan	2000	98.3%	99.8%	92.9%
Egypt	Aswan	2017	100.0%	100.0%	100.0%
Egypt	Aswan City	2000	91.9%	100.0%	48.1%
Egypt	Aswan City	2017	99.7%	100.0%	99.3%
Egypt	Asyut	2000	98.8%	99.4%	97.8%
Egypt	Asyut	2017	100.0%	100.0%	100.0%
Egypt	Asyut 1	2000	98.4%	99.2%	97.4%
Egypt	Asyut 1	2017	100.0%	100.0%	100.0%
Egypt	Asyut 2	2000	97.7%	98.7%	96.3%
Egypt	Asyut 2	2017	100.0%	100.0%	100.0%
Egypt	At-Tall al-Kabir	2000	87.2%	95.5%	74.4%
Egypt	At-Tall al-Kabir	2017	99.8%	100.0%	98.2%
Egypt	At-Tebin	2000	95.8%	99.2%	88.1%
Egypt	At-Tebin	2017	100.0%	100.0%	100.0%
Egypt	At-Tur	2000	90.7%	97.4%	78.9%
Egypt	At-Tur	2017	99.0%	99.9%	97.0%
Egypt	Atfih	2000	44.3%	50.6%	39.5%
Egypt	Atfih	2017	97.9%	99.6%	91.7%
Egypt	Awlad Saqr	2000	57.0%	80.3%	41.3%
Egypt	Awlad Saqr	2017	95.0%	99.8%	79.8%
Egypt	Az-Zahir	2000	99.4%	99.7%	98.8%
Egypt	Az-Zahir	2017	100.0%	100.0%	100.0%
Egypt	Az-Zaytun	2000	99.6%	99.8%	99.4%
Egypt	Az-Zaytun	2017	100.0%	100.0%	100.0%
Egypt	Az-Zohur	2000	99.6%	99.9%	99.0%
Egypt	Az-Zohur	2017	100.0%	100.0%	100.0%
Egypt	Bab ash-Sha'riyah	2000	99.4%	99.7%	98.8%
Egypt	Bab ash-Sha'riyah	2017	100.0%	100.0%	100.0%

Country	Second Administrative Unit	Year	Mean	Upper	Lower
Egypt	Bab Sharqi	2000	99.4%	99.8%	98.7%
Egypt	Bab Sharqi	2017	100.0%	100.0%	100.0%
Egypt	Badr	2000	55.2%	70.2%	42.5%
Egypt	Badr	2000	84.1%	100.0%	8.3%
Egypt	Badr	2017	97.3%	99.9%	88.1%
Egypt	Badr	2017	99.2%	100.0%	91.7%
Egypt	Banha	2000	87.7%	89.8%	85.2%
Egypt	Banha	2017	100.0%	100.0%	100.0%
Egypt	Bani Mazar	2000	81.2%	83.9%	78.1%
Egypt	Bani Mazar	2017	100.0%	100.0%	99.9%
Egypt	Bani Suwayf	2000	91.4%	93.7%	88.1%
Egypt	Bani Suwayf	2017	100.0%	100.0%	100.0%
Egypt	Bani Suwayf City	2000	96.1%	99.3%	80.3%
Egypt	Bani Suwayf City	2017	100.0%	100.0%	99.9%
Egypt	Bani Ubayd	2000	89.2%	98.4%	64.9%
Egypt	Bani Ubayd	2017	100.0%	100.0%	99.9%
Egypt	Baris Shurtah	2000	88.2%	100.0%	23.6%
Egypt	Baris Shurtah	2017	99.6%	100.0%	94.4%
Egypt	Basyun	2000	83.9%	87.6%	79.6%
Egypt	Basyun	2017	100.0%	100.0%	100.0%
Egypt	Biba	2000	85.5%	89.8%	80.8%
Egypt	Biba	2017	100.0%	100.0%	100.0%
Egypt	Bilbays	2000	67.8%	72.1%	64.0%
Egypt	Bilbays	2017	99.3%	99.8%	98.3%
Egypt	Bilqas	2000	78.8%	90.9%	60.4%
Egypt	Bilqas	2017	97.3%	99.9%	90.9%
Egypt	Bir al-'Abd	2000	89.8%	98.4%	69.7%
Egypt	Bir al-'Abd	2017	99.6%	100.0%	96.8%
Egypt	Birkat as-Sab'	2000	70.2%	74.2%	64.8%
Egypt	Birkat as-Sab'	2017	100.0%	100.0%	99.9%
Egypt	Biyala	2000	82.7%	88.5%	63.8%
Egypt	Biyala	2017	96.9%	99.0%	93.2%
Egypt	Bulaq	2000	97.7%	98.5%	96.4%
Egypt	Bulaq	2017	100.0%	100.0%	100.0%
Egypt	Bulaq al-Dakrur	2000	98.8%	99.2%	98.3%
Egypt	Bulaq al-Dakrur	2017	100.0%	100.0%	100.0%
Egypt	Burj al-'Arab	2000	88.7%	99.2%	57.4%
Egypt	Burj al-'Arab	2017	99.7%	100.0%	97.6%
Egypt	Dahab	2000	73.9%	98.2%	36.6%
Egypt	Dahab	2017	97.6%	100.0%	85.2%
Egypt	Damanhur	2000	94.0%	97.3%	88.0%
Egypt	Damanhur	2017	100.0%	100.0%	99.9%
Egypt	Damietta	2000	99.3%	99.9%	97.4%
Egypt	Damietta	2017	100.0%	100.0%	100.0%
Egypt	Damietta 1	2000	99.3%	100.0%	97.0%
Egypt	Damietta 1	2017	100.0%	100.0%	100.0%
Egypt	Damietta 2	2000	99.5%	100.0%	98.1%
Egypt	Damietta 2	2017	100.0%	100.0%	100.0%
Egypt	Dar as-Salam	2000	74.6%	79.7%	67.0%
Egypt	Dar as-Salam	2017	99.1%	99.8%	98.1%
Egypt	Daraw	2000	99.2%	99.9%	96.7%
Egypt	Daraw	2017	100.0%	100.0%	100.0%
Egypt	Dayr Mawas	2000	77.9%	81.0%	73.8%
Egypt	Dayr Mawas	2017	100.0%	100.0%	100.0%
Egypt	Dayrut	2000	85.4%	88.5%	81.6%
Egypt	Dayrut	2017	100.0%	100.0%	100.0%
Egypt	Dikirnis	2000	94.4%	98.0%	85.6%
Egypt	Dikirnis	2017	99.9%	100.0%	99.0%

Country	Second Administrative Unit	Year	Mean	Upper	Lower
Egypt	Dishna	2000	89.8%	95.3%	75.9%
Egypt	Dishna	2017	100.0%	100.0%	99.8%
Egypt	Disuq	2000	98.5%	99.3%	95.0%
Egypt	Disuq	2017	100.0%	100.0%	100.0%
Egypt	Diyarb Najm	2000	31.5%	35.3%	28.0%
Egypt	Diyarb Najm	2017	99.0%	99.7%	97.3%
Egypt	Fa'id	2000	88.7%	97.5%	61.0%
Egypt	Fa'id	2017	99.8%	100.0%	98.4%
Egypt	Faisal	2000	95.4%	99.8%	83.8%
Egypt	Faisal	2017	99.9%	100.0%	98.9%
Egypt	Faqus	2000	84.3%	86.5%	81.5%
Egypt	Faqus	2017	94.5%	94.7%	94.2%
Egypt	Faraskur	2000	99.3%	99.9%	97.6%
Egypt	Faraskur	2017	100.0%	100.0%	100.0%
Egypt	Farshut	2000	75.1%	80.2%	69.2%
Egypt	Farshut	2017	100.0%	100.0%	100.0%
Egypt	Fuwah	2000	99.0%	100.0%	92.9%
Egypt	Fuwah	2017	100.0%	100.0%	100.0%
Egypt	Gamsa	2000	98.4%	100.0%	87.6%
Egypt	Gamsa	2017	100.0%	100.0%	100.0%
Egypt	Giza	2000	83.5%	84.0%	83.0%
Egypt	Giza	2017	98.8%	99.5%	97.7%
Egypt	Hada'iq al-Qubbah	2000	99.7%	99.9%	99.5%
Egypt	Hada'iq al-Qubbah	2017	100.0%	100.0%	100.0%
Egypt	Hawsh 'Isa	2000	91.3%	96.0%	79.8%
Egypt	Hawsh 'Isa	2017	100.0%	100.0%	99.9%
Egypt	Heliopolis	2000	99.4%	99.6%	99.1%
Egypt	Heliopolis	2017	100.0%	100.0%	100.0%
Egypt	Helwan	2000	98.9%	99.6%	97.0%
Egypt	Helwan	2017	100.0%	100.0%	100.0%
Egypt	Hihya	2000	58.1%	59.8%	56.4%
Egypt	Hihya	2017	88.8%	91.4%	84.9%
Egypt	Ibshaway	2000	99.5%	99.9%	98.6%
Egypt	Ibshaway	2017	100.0%	100.0%	100.0%
Egypt	Idfu	2000	98.4%	99.6%	94.8%
Egypt	Idfu	2017	100.0%	100.0%	100.0%
Egypt	Idku	2000	95.4%	99.3%	80.5%
Egypt	Idku	2017	100.0%	100.0%	100.0%
Egypt	Ihnasiya	2000	93.4%	96.7%	88.8%
Egypt	Ihnasiya	2017	100.0%	100.0%	100.0%
Egypt	Imbabah	2000	85.4%	87.4%	83.1%
Egypt	Imbabah	2017	100.0%	100.0%	100.0%
Egypt	Ismailia	2000	85.9%	90.5%	79.7%
Egypt	Ismailia	2017	99.9%	100.0%	99.7%
Egypt	Ismailia 1	2000	98.2%	99.2%	95.9%
Egypt	Ismailia 1	2017	100.0%	100.0%	100.0%
Egypt	Ismailia 2	2000	97.6%	98.6%	95.8%
Egypt	Ismailia 2	2017	100.0%	100.0%	100.0%
Egypt	Ismailia 3	2000	99.1%	99.7%	98.1%
Egypt	Ismailia 3	2017	100.0%	100.0%	100.0%
Egypt	Isna	2000	96.0%	99.0%	84.1%
Egypt	Isna	2017	100.0%	100.0%	99.7%
Egypt	Itsa	2000	99.1%	99.9%	95.6%
Egypt	Itsa	2017	100.0%	100.0%	100.0%
Egypt	Ityay al-Barud	2000	61.6%	67.8%	56.6%
Egypt	Ityay al-Barud	2017	99.9%	100.0%	99.8%
Egypt	Jirja	2000	72.4%	74.9%	69.3%
Egypt	Jirja	2017	99.9%	100.0%	99.9%
Egypt	Juhaynah al-Gharbiyah	2000	94.8%	97.6%	90.6%

Country	Second Administrative Unit	Year	Mean	Upper	Lower
Egypt	Juhaynah al-Gharbiyah	2017	100.0%	100.0%	100.0%
Egypt	Kafr ad-Dawwar	2000	97.8%	99.1%	95.0%
Egypt	Kafr ad-Dawwar	2017	100.0%	100.0%	99.9%
Egypt	Kafr ash-Shaykh	2000	95.6%	97.2%	92.8%
Egypt	Kafr ash-Shaykh	2017	100.0%	100.0%	100.0%
Egypt	Kafr az-Zayyat	2000	75.1%	77.9%	71.6%
Egypt	Kafr az-Zayyat	2017	96.6%	99.9%	91.8%
Egypt	Kafr Sa'd	2000	98.5%	99.4%	96.4%
Egypt	Kafr Sa'd	2017	100.0%	100.0%	100.0%
Egypt	Kafr Saqr	2000	59.1%	66.5%	49.0%
Egypt	Kafr Saqr	2017	98.8%	99.9%	97.1%
Egypt	Kafr Shukr	2000	90.5%	92.9%	86.9%
Egypt	Kafr Shukr	2017	100.0%	100.0%	100.0%
Egypt	Karmuz	2000	99.4%	99.8%	98.2%
Egypt	Karmuz	2017	100.0%	100.0%	100.0%
Egypt	Kawm Hamadah	2000	65.5%	71.7%	60.2%
Egypt	Kawm Hamadah	2017	99.6%	99.9%	98.1%
Egypt	Kawm Umbu	2000	96.6%	98.8%	91.4%
Egypt	Kawm Umbu	2017	100.0%	100.0%	100.0%
Egypt	Kirdasah	2000	87.0%	88.8%	85.1%
Egypt	Kirdasah	2017	100.0%	100.0%	100.0%
Egypt	Luxor	2000	96.3%	97.4%	94.6%
Egypt	Luxor	2017	100.0%	100.0%	100.0%
Egypt	Maghaghah	2000	85.6%	88.5%	82.1%
Egypt	Maghaghah	2017	100.0%	100.0%	100.0%
Egypt	Mahalat Dimnah	2000	97.9%	99.7%	92.0%
Egypt	Mahalat Dimnah	2017	100.0%	100.0%	100.0%
Egypt	Mallawi	2000	84.5%	86.7%	82.0%
Egypt	Mallawi	2017	100.0%	100.0%	100.0%
Egypt	Mallawi City	2000	87.2%	90.1%	82.5%
Egypt	Mallawi City	2017	100.0%	100.0%	100.0%
Egypt	Manfalut	2000	94.5%	96.8%	88.1%
Egypt	Manfalut	2017	100.0%	100.0%	100.0%
Egypt	Marina al-'Alamayn as-Siyahiyah	2000	89.6%	100.0%	52.4%
Egypt	Marina al-'Alamayn as-Siyahiyah	2017	99.7%	100.0%	96.7%
Egypt	Marsa 'Alam	2000	80.5%	91.4%	65.4%
Egypt	Marsa 'Alam	2017	98.6%	99.8%	95.8%
Egypt	Marsa Matruh	2000	58.4%	65.1%	49.2%
Egypt	Marsa Matruh	2017	98.6%	99.6%	96.5%
Egypt	Mashtul as-Suq	2000	62.5%	67.3%	56.7%
Egypt	Mashtul as-Suq	2017	99.9%	99.9%	99.9%
Egypt	Matay	2000	73.7%	76.6%	70.6%
Egypt	Matay	2017	100.0%	100.0%	99.9%
Egypt	Minuf	2000	77.6%	82.1%	72.1%
Egypt	Minuf	2017	100.0%	100.0%	99.9%

Country	Second Administrative Unit	Year	Mean	Upper	Lower
Egypt	Minuf City	2000	74.2%	84.3%	60.9%
Egypt	Minuf City	2017	100.0%	100.0%	100.0%
Egypt	Minya al-Qamh	2000	69.6%	74.2%	64.4%
Egypt	Minya al-Qamh	2017	99.9%	100.0%	99.9%
Egypt	Minyat an-Nasr	2000	98.5%	99.8%	94.4%
Egypt	Minyat an-Nasr	2017	100.0%	100.0%	99.9%
Egypt	Mit Ghamr	2000	68.2%	75.3%	62.3%
Egypt	Mit Ghamr	2017	99.9%	100.0%	99.6%
Egypt	Mit Salsil	2000	97.5%	99.6%	91.6%
Egypt	Mit Salsil	2017	100.0%	100.0%	99.9%
Egypt	Monshat Nasr	2000	99.4%	99.7%	98.8%
Egypt	Monshat Nasr	2017	100.0%	100.0%	100.0%
Egypt	Mubarak - Sharq at-Tafri'tah	2000	86.7%	94.7%	72.7%
Egypt	Mubarak - Sharq at-Tafri'tah	2017	99.8%	100.0%	99.3%
Egypt	Muharam Bik	2000	99.5%	99.8%	98.7%
Egypt	Muharam Bik	2017	100.0%	100.0%	100.0%
Egypt	Mutubis	2000	82.9%	90.7%	64.0%
Egypt	Mutubis	2017	99.9%	100.0%	98.7%
Egypt	Nabaruh	2000	98.8%	99.9%	94.1%
Egypt	Nabaruh	2017	100.0%	100.0%	100.0%
Egypt	Naj' Hammadi	2000	81.3%	86.7%	75.6%
Egypt	Naj' Hammadi	2017	100.0%	100.0%	99.9%
Egypt	Nakhl	2000	77.8%	92.7%	58.6%
Egypt	Nakhl	2017	98.1%	99.9%	92.8%
Egypt	Naqadah	2000	91.9%	95.1%	86.4%
Egypt	Naqadah	2017	100.0%	100.0%	100.0%
Egypt	Nasir Bush	2000	87.6%	90.3%	84.6%
Egypt	Nasir Bush	2017	100.0%	100.0%	100.0%
Egypt	Nasr	2000	96.4%	99.2%	88.1%
Egypt	Nasr	2017	100.0%	100.0%	99.9%
Egypt	Nasr City 1	2000	94.1%	97.8%	87.0%
Egypt	Nasr City 1	2017	100.0%	100.0%	100.0%
Egypt	Nasr City 2	2000	99.4%	99.8%	98.7%
Egypt	Nasr City 2	2017	100.0%	100.0%	100.0%
Egypt	New Akhmim City	2000	56.1%	100.0%	0.1%
Egypt	New Akhmim City	2017	95.8%	100.0%	54.3%
Egypt	New Asyut City	2000	94.6%	98.9%	78.3%
Egypt	New Asyut City	2017	100.0%	100.0%	100.0%
Egypt	New Burj al-'Arab City	2000	86.3%	100.0%	22.8%
Egypt	New Burj al-'Arab City	2017	99.5%	100.0%	96.2%
Egypt	New Cairo 1	2000	86.9%	98.5%	63.2%
Egypt	New Cairo 1	2017	99.8%	100.0%	98.9%
Egypt	New Cairo 2	2000	74.0%	96.5%	44.6%
Egypt	New Cairo 2	2017	99.5%	100.0%	93.0%
Egypt	New Cairo 3	2000	96.6%	100.0%	81.6%
Egypt	New Cairo 3	2017	99.9%	100.0%	98.9%

Country	Second Administrative Unit	Year	Mean	Upper	Lower
Egypt	New Damietta City	2000	98.7%	99.9%	90.4%
Egypt	New Damietta City	2017	100.0%	100.0%	100.0%
Egypt	New Minya City	2000	97.8%	99.1%	95.0%
Egypt	New Minya City	2017	100.0%	100.0%	100.0%
Egypt	New Salhiyah	2000	76.2%	82.9%	71.1%
Egypt	New Salhiyah	2017	90.4%	91.0%	89.6%
Egypt	New Sawhaj City	2000	99.5%	100.0%	97.0%
Egypt	New Sawhaj City	2017	100.0%	100.0%	100.0%
Egypt	New Tushka City	2000	89.2%	100.0%	11.1%
Egypt	New Tushka City	2017	99.8%	100.0%	98.6%
Egypt	Nuweiba’	2000	75.8%	92.9%	48.4%
Egypt	Nuweiba’	2017	97.2%	99.9%	89.2%
Egypt	Port al-Basal	2000	99.4%	99.8%	98.5%
Egypt	Port al-Basal	2017	100.0%	100.0%	100.0%
Egypt	Port Alexandria Police Department	2000	99.5%	99.9%	98.5%
Egypt	Port Alexandria Police Department	2017	100.0%	100.0%	100.0%
Egypt	Port Fuad	2000	99.2%	99.8%	98.1%
Egypt	Port Fuad	2017	100.0%	100.0%	100.0%
Egypt	Port Fuad 2	2000	98.1%	99.7%	94.2%
Egypt	Port Fuad 2	2017	100.0%	100.0%	100.0%
Egypt	Port of Damietta Police Department	2000	99.4%	100.0%	96.7%
Egypt	Port of Damietta Police Department	2017	100.0%	100.0%	100.0%
Egypt	Port Sa’id Police Department	2000	99.5%	99.9%	98.5%
Egypt	Port Sa’id Police Department	2017	100.0%	100.0%	100.0%
Egypt	Port Suez Police Department	2000	98.0%	100.0%	81.2%
Egypt	Port Suez Police Department	2017	100.0%	100.0%	99.9%
Egypt	Qaha	2000	58.8%	65.3%	52.2%
Egypt	Qaha	2017	99.9%	99.9%	99.9%
Egypt	Qallin	2000	82.5%	86.0%	78.0%
Egypt	Qallin	2017	100.0%	100.0%	100.0%
Egypt	Qalyub	2000	96.2%	97.0%	95.1%
Egypt	Qalyub	2017	100.0%	100.0%	100.0%
Egypt	Qasr an-Nil	2000	98.1%	98.7%	97.1%
Egypt	Qasr an-Nil	2017	100.0%	100.0%	100.0%
Egypt	Qift	2000	97.0%	99.2%	90.8%
Egypt	Qift	2017	100.0%	100.0%	100.0%
Egypt	Qina	2000	90.4%	93.2%	83.4%
Egypt	Qina	2017	100.0%	100.0%	99.9%

Country	Second Administrative Unit	Year	Mean	Upper	Lower
Egypt	Qina City	2000	95.3%	99.7%	80.5%
Egypt	Qina City	2017	100.0%	100.0%	100.0%
Egypt	Qus	2000	81.4%	85.5%	76.7%
Egypt	Qus	2017	100.0%	100.0%	100.0%
Egypt	Qutur	2000	92.4%	94.0%	89.5%
Egypt	Qutur	2017	100.0%	100.0%	100.0%
Egypt	Quwaysina	2000	67.9%	72.4%	61.1%
Egypt	Quwaysina	2017	100.0%	100.0%	99.9%
Egypt	Rafah	2000	37.2%	47.2%	23.0%
Egypt	Rafah	2017	96.5%	99.4%	88.7%
Egypt	Ras Gharib	2000	69.4%	85.7%	51.4%
Egypt	Ras Gharib	2017	99.0%	99.9%	96.9%
Egypt	Ras Sidr	2000	89.9%	97.3%	75.2%
Egypt	Ras Sidr	2017	99.4%	100.0%	97.2%
Egypt	Rosetta	2000	81.8%	90.0%	65.0%
Egypt	Rosetta	2017	100.0%	100.0%	99.9%
Egypt	Rud al-Faraj	2000	97.8%	98.6%	96.6%
Egypt	Rud al-Faraj	2017	100.0%	100.0%	100.0%
Egypt	Sadat City	2000	95.8%	99.1%	84.5%
Egypt	Sadat City	2017	99.9%	100.0%	99.4%
Egypt	Safaja	2000	68.0%	89.7%	47.0%
Egypt	Safaja	2017	97.1%	100.0%	82.9%
Egypt	Sahil Salim	2000	93.9%	96.6%	90.2%
Egypt	Sahil Salim	2017	100.0%	100.0%	100.0%
Egypt	Samalut	2000	77.0%	79.3%	74.5%
Egypt	Samalut	2017	99.9%	100.0%	99.9%
Egypt	Samannud	2000	91.0%	93.1%	87.9%
Egypt	Samannud	2017	100.0%	100.0%	100.0%
Egypt	Sant Katrin	2000	61.4%	80.0%	21.3%
Egypt	Sant Katrin	2017	91.6%	98.7%	80.8%
Egypt	Saqultah	2000	78.0%	82.9%	72.2%
Egypt	Saqultah	2017	100.0%	100.0%	99.9%
Egypt	Sawhaj	2000	91.5%	93.7%	88.3%
Egypt	Sawhaj	2017	100.0%	100.0%	100.0%
Egypt	Sawhaj 2	2000	95.5%	96.9%	93.8%
Egypt	Sawhaj 2	2017	100.0%	100.0%	100.0%
Egypt	Sharm el-Sheikh	2000	47.9%	76.5%	14.7%
Egypt	Sharm el-Sheikh	2017	87.3%	98.9%	69.3%
Egypt	Sheikh Zawid	2000	81.5%	96.6%	50.1%
Egypt	Sheikh Zawid	2017	99.2%	100.0%	93.6%
Egypt	Sheikh Zayed	2000	97.7%	99.9%	86.6%
Egypt	Sheikh Zayed	2017	100.0%	100.0%	100.0%
Egypt	Shibin al-Kawm	2000	93.4%	95.5%	90.6%
Egypt	Shibin al-Kawm	2017	100.0%	100.0%	100.0%
Egypt	Shibin al-Qanatir	2000	83.1%	85.2%	80.2%
Egypt	Shibin al-Qanatir	2017	100.0%	100.0%	100.0%
Egypt	Shirbin	2000	81.2%	86.0%	75.8%
Egypt	Shirbin	2017	98.6%	99.9%	95.2%
Egypt	Shubra	2000	98.7%	99.2%	98.0%
Egypt	Shubra	2017	100.0%	100.0%	100.0%
Egypt	Shubra al-Khaymah 1	2000	99.7%	99.9%	99.4%
Egypt	Shubra al-Khaymah 1	2017	100.0%	100.0%	100.0%

Country	Second Administrative Unit	Year	Mean	Upper	Lower
Egypt	Shubra al-Khaymah 2	2000	99.7%	99.8%	99.4%
Egypt	Shubra al-Khaymah 2	2017	100.0%	100.0%	100.0%
Egypt	Shubra Khit	2000	92.9%	97.4%	75.3%
Egypt	Shubra Khit	2017	100.0%	100.0%	99.9%
Egypt	Shurtah al-Dakhlah	2000	95.3%	99.5%	87.4%
Egypt	Shurtah al-Dakhlah	2017	99.9%	100.0%	98.8%
Egypt	Shurtah al-Farafirah	2000	89.7%	100.0%	55.6%
Egypt	Shurtah al-Farafirah	2017	99.6%	100.0%	97.0%
Egypt	Shurtah al-Qasimah	2000	81.1%	97.9%	55.1%
Egypt	Shurtah al-Qasimah	2017	97.9%	100.0%	87.2%
Egypt	Shurtah Rumanah	2000	92.1%	100.0%	49.5%
Egypt	Shurtah Rumanah	2017	99.6%	100.0%	97.9%
Egypt	Sidfa	2000	91.5%	94.4%	86.6%
Egypt	Sidfa	2017	100.0%	100.0%	100.0%
Egypt	Sidi Barrani	2000	91.7%	99.7%	48.2%
Egypt	Sidi Barrani	2017	99.6%	100.0%	97.1%
Egypt	Sidi Jabir	2000	99.2%	99.7%	98.4%
Egypt	Sidi Jabir	2017	100.0%	100.0%	100.0%
Egypt	Sidi Salim	2000	73.3%	75.8%	69.2%
Egypt	Sidi Salim	2017	89.5%	92.0%	87.1%
Egypt	Sinnuris	2000	96.5%	98.3%	92.1%
Egypt	Sinnuris	2017	100.0%	100.0%	100.0%
Egypt	Sirs al-Layyanah	2000	82.6%	89.1%	73.9%
Egypt	Sirs al-Layyanah	2017	100.0%	100.0%	100.0%
Egypt	Siwa	2000	93.4%	99.8%	71.6%
Egypt	Siwa	2017	99.8%	100.0%	98.6%
Egypt	Sixth of October 1 City	2000	97.2%	100.0%	76.8%
Egypt	Sixth of October 1 City	2017	100.0%	100.0%	100.0%
Egypt	Sixth of October 2 City	2000	91.4%	99.9%	59.5%
Egypt	Sixth of October 2 City	2017	100.0%	100.0%	99.9%
Egypt	Suez	2000	99.7%	100.0%	98.8%
Egypt	Suez	2017	100.0%	100.0%	100.0%
Egypt	Sumusta al-Waqf	2000	81.1%	88.4%	73.4%
Egypt	Sumusta al-Waqf	2017	100.0%	100.0%	99.9%
Egypt	Taba	2000	83.4%	100.0%	13.0%
Egypt	Taba	2017	99.1%	100.0%	93.6%
Egypt	Tahta	2000	97.3%	98.3%	95.3%
Egypt	Tahta	2017	100.0%	100.0%	100.0%
Egypt	Tahta City	2000	99.1%	99.8%	97.2%
Egypt	Tahta City	2017	100.0%	100.0%	100.0%
Egypt	Tala	2000	68.3%	78.2%	60.1%
Egypt	Tala	2017	97.1%	99.8%	95.0%

Country	Second Administrative Unit	Year	Mean	Upper	Lower
Egypt	Talkha	2000	97.5%	98.9%	94.5%
Egypt	Talkha	2017	100.0%	100.0%	100.0%
Egypt	Tamiyah	2000	97.9%	99.3%	94.1%
Egypt	Tamiyah	2017	100.0%	100.0%	100.0%
Egypt	Tanta	2000	82.1%	84.7%	79.4%
Egypt	Tanta	2017	99.0%	99.9%	98.2%
Egypt	Tanta 1	2000	82.5%	86.0%	78.5%
Egypt	Tanta 1	2017	100.0%	100.0%	100.0%
Egypt	Tanta 2	2000	83.2%	87.2%	78.1%
Egypt	Tanta 2	2017	100.0%	100.0%	100.0%
Egypt	Tibah Police Dept.	2000	98.0%	99.4%	94.5%
Egypt	Tibah Police Dept.	2017	100.0%	100.0%	100.0%
Egypt	Tima	2000	88.3%	91.9%	83.9%
Egypt	Tima	2017	100.0%	100.0%	100.0%
Egypt	Timay al-Imdid	2000	91.7%	92.9%	88.2%
Egypt	Timay al-Imdid	2017	97.8%	99.9%	94.6%
Egypt	Tukh	2000	78.8%	84.4%	70.8%
Egypt	Tukh	2017	100.0%	100.0%	100.0%
Egypt	Turah	2000	98.3%	99.1%	97.0%
Egypt	Turah	2017	100.0%	100.0%	100.0%
Egypt	Unorganized in Al Buhayrah	2000	98.3%	99.9%	91.3%
Egypt	Unorganized in Al Buhayrah	2017	100.0%	100.0%	99.9%
Egypt	Unorganized in Al Fayoum	2000	99.0%	99.9%	96.4%
Egypt	Unorganized in Al Fayoum	2017	100.0%	100.0%	100.0%
Egypt	Unorganized in Al Iskandariyah	2000	87.9%	100.0%	46.8%
Egypt	Unorganized in Al Iskandariyah	2017	99.4%	100.0%	94.5%
Egypt	Unorganized in Al Jizah	2000	63.8%	67.6%	59.5%
Egypt	Unorganized in Al Jizah	2017	94.5%	95.6%	93.1%
Egypt	Unorganized in Al Minya	2000	81.0%	85.8%	75.3%
Egypt	Unorganized in Al Minya	2017	99.5%	99.7%	99.2%
Egypt	Unorganized in Al Qahirah	2000	96.9%	99.8%	88.6%
Egypt	Unorganized in Al Qahirah	2017	99.8%	100.0%	98.5%
Egypt	Unorganized in Al Qalyubiyah	2000	71.0%	80.8%	56.9%
Egypt	Unorganized in Al Qalyubiyah	2017	98.8%	99.5%	97.8%
Egypt	Unorganized in Al Uqsur	2000	99.4%	99.9%	97.4%
Egypt	Unorganized in Al Uqsur	2017	100.0%	100.0%	100.0%

Country	Second Administrative Unit	Year	Mean	Upper	Lower
Egypt	Unorganized in Ash Sharqiyah	2000	55.5%	60.9%	30.2%
Egypt	Unorganized in Ash Sharqiyah	2017	95.6%	99.3%	89.2%
Egypt	Unorganized in Aswan	2000	95.3%	98.1%	90.2%
Egypt	Unorganized in Aswan	2017	99.8%	99.9%	99.4%
Egypt	Unorganized in Asyut	2000	79.5%	82.6%	76.1%
Egypt	Unorganized in Asyut	2017	99.9%	99.9%	99.7%
Egypt	Unorganized in Bani Suwayf	2000	87.4%	91.0%	83.5%
Egypt	Unorganized in Bani Suwayf	2017	100.0%	100.0%	99.9%
Egypt	Unorganized in Qina	2000	82.8%	86.5%	78.8%
Egypt	Unorganized in Qina	2017	99.9%	100.0%	99.8%
Egypt	Unorganized in Suhaj	2000	64.4%	69.2%	58.7%
Egypt	Unorganized in Suhaj	2017	98.9%	99.6%	97.7%
Egypt	Wadi Al-Natron	2000	98.3%	100.0%	87.8%
Egypt	Wadi Al-Natron	2017	100.0%	100.0%	100.0%
Egypt	West Nubariyah	2000	90.3%	99.9%	57.4%
Egypt	West Nubariyah	2017	99.7%	100.0%	97.0%
Egypt	Yusuf as-Sidiq	2000	98.7%	99.9%	93.0%
Egypt	Yusuf as-Sidiq	2017	100.0%	100.0%	100.0%
Egypt	Zamalik	2000	97.7%	98.5%	96.4%
Egypt	Zamalik	2017	100.0%	100.0%	100.0%
Egypt	Zaqaziq	2000	45.4%	49.4%	41.6%
Egypt	Zaqaziq	2017	99.5%	99.8%	98.8%
Egypt	Zaqaziq 1	2000	61.9%	69.5%	53.3%
Egypt	Zaqaziq 1	2017	99.9%	100.0%	99.9%
Egypt	Zaqaziq 2	2000	56.1%	63.6%	47.5%
Egypt	Zaqaziq 2	2017	99.9%	99.9%	99.9%
Egypt	Zarqa	2000	99.3%	99.9%	96.7%
Egypt	Zarqa	2017	100.0%	100.0%	100.0%
Egypt	Zawiyya Al-Hamra	2000	99.8%	99.9%	99.6%
Egypt	Zawiyya Al-Hamra	2017	100.0%	100.0%	100.0%
Egypt	Zifta	2000	81.7%	85.8%	77.3%
Egypt	Zifta	2017	100.0%	100.0%	100.0%
Iraq	Abu al Khasib	2000	56.1%	73.4%	38.7%
Iraq	Abu al Khasib	2017	38.5%	57.7%	21.6%
Iraq	Abu Ghraib	2000	91.6%	98.2%	79.8%
Iraq	Abu Ghraib	2017	79.8%	93.3%	57.7%
Iraq	Ad Diwaniyah	2000	78.9%	91.0%	62.1%
Iraq	Ad Diwaniyah	2017	58.0%	75.8%	40.4%
Iraq	Adhamiya	2000	90.9%	97.5%	81.4%
Iraq	Adhamiya	2017	79.5%	91.8%	65.6%

Country	Second Administrative Unit	Year	Mean	Upper	Lower
Iraq	Afak	2000	76.4%	92.4%	52.8%
Iraq	Afak	2017	58.0%	78.5%	36.3%
Iraq	Ain Al Tamur	2000	69.5%	91.0%	41.0%
Iraq	Ain Al Tamur	2017	49.7%	75.8%	25.0%
Iraq	Akre	2000	82.0%	94.7%	60.8%
Iraq	Akre	2017	65.4%	83.3%	43.6%
Iraq	Al Amarah	2000	68.7%	77.0%	60.2%
Iraq	Al Amarah	2017	53.2%	63.0%	43.1%
Iraq	Al Ba'aj	2000	78.0%	86.0%	68.8%
Iraq	Al Ba'aj	2017	59.2%	71.1%	47.1%
Iraq	Al Door	2000	77.3%	92.7%	56.9%
Iraq	Al Door	2017	59.7%	81.1%	35.9%
Iraq	Al Fallujah	2000	86.5%	90.1%	81.0%
Iraq	Al Fallujah	2017	70.7%	77.2%	63.0%
Iraq	Al Faw	2000	83.4%	96.5%	58.0%
Iraq	Al Faw	2017	70.4%	89.5%	38.4%
Iraq	Al Haditha	2000	82.7%	90.8%	72.9%
Iraq	Al Haditha	2017	62.0%	75.1%	50.6%
Iraq	Al Hamdaniyah	2000	71.9%	85.4%	54.1%
Iraq	Al Hamdaniyah	2017	53.6%	71.4%	34.2%
Iraq	Al Hamza	2000	76.8%	90.3%	57.2%
Iraq	Al Hamza	2017	58.9%	78.0%	36.7%
Iraq	Al Hashimiyah	2000	73.6%	90.2%	54.6%
Iraq	Al Hashimiyah	2017	52.5%	75.3%	32.0%
Iraq	Al Hayy	2000	76.2%	90.7%	54.6%
Iraq	Al Hayy	2017	58.5%	76.8%	36.3%
Iraq	Al Hillah	2000	73.9%	89.1%	55.6%
Iraq	Al Hillah	2017	50.1%	70.3%	34.4%
Iraq	Al Jadwal al Gharbi	2000	74.7%	90.0%	54.0%
Iraq	Al Jadwal al Gharbi	2017	53.7%	76.4%	30.5%
Iraq	Al Kahla	2000	71.8%	90.2%	47.5%
Iraq	Al Kahla	2017	53.0%	76.7%	32.2%
Iraq	Al Khalis	2000	70.3%	88.0%	47.3%
Iraq	Al Khalis	2017	49.8%	72.2%	28.2%
Iraq	Al Khithir	2000	60.6%	70.3%	50.0%
Iraq	Al Khithir	2017	35.6%	44.8%	26.5%
Iraq	Al Kufa	2000	77.9%	88.6%	58.1%
Iraq	Al Kufa	2017	58.8%	77.1%	36.9%
Iraq	Al Kut	2000	79.2%	88.3%	67.0%
Iraq	Al Kut	2017	61.8%	73.4%	48.2%
Iraq	Al Madiana	2000	66.6%	81.6%	46.8%
Iraq	Al Madiana	2017	46.7%	63.6%	27.9%
Iraq	Al Mahawil	2000	76.4%	91.9%	53.9%
Iraq	Al Mahawil	2017	57.2%	79.6%	34.6%
Iraq	Al Manathera	2000	81.2%	92.0%	65.3%
Iraq	Al Manathera	2017	63.3%	78.5%	46.1%
Iraq	Al Miamona	2000	68.7%	88.5%	41.7%
Iraq	Al Miamona	2017	52.4%	75.7%	30.1%
Iraq	Al Mijar al Kabir	2000	64.4%	81.9%	35.6%
Iraq	Al Mijar al Kabir	2017	44.9%	65.5%	20.9%
Iraq	Al Miqdadiyah	2000	72.6%	90.0%	51.8%
Iraq	Al Miqdadiyah	2017	49.6%	72.8%	28.5%

Country	Second Administrative Unit	Year	Mean	Upper	Lower
Iraq	Al Misiab	2000	70.0%	92.6%	35.4%
Iraq	Al Misiab	2017	49.9%	83.0%	17.3%
Iraq	Al Noamania	2000	72.5%	87.3%	53.3%
Iraq	Al Noamania	2017	49.4%	68.8%	30.1%
Iraq	Al Qa'im	2000	76.2%	84.2%	66.8%
Iraq	Al Qa'im	2017	56.2%	67.1%	44.9%
Iraq	Al Qurnah	2000	64.6%	76.5%	49.8%
Iraq	Al Qurnah	2017	47.4%	62.1%	30.9%
Iraq	Al Shikhan	2000	86.7%	96.6%	69.7%
Iraq	Al Shikhan	2017	72.0%	88.9%	46.9%
Iraq	Al Shirkat	2000	62.0%	73.2%	47.8%
Iraq	Al Shirkat	2017	39.5%	52.8%	25.9%
Iraq	Al Zubair	2000	76.4%	85.6%	65.9%
Iraq	Al Zubair	2017	58.9%	72.3%	45.5%
Iraq	Al-Faris	2000	70.1%	89.7%	48.2%
Iraq	Al-Faris	2017	48.9%	71.6%	28.7%
Iraq	Al-Mada'in	2000	85.3%	96.6%	61.7%
Iraq	Al-Mada'in	2017	69.5%	89.9%	41.5%
Iraq	Ali al Gharbi	2000	75.7%	87.0%	61.0%
Iraq	Ali al Gharbi	2017	59.3%	74.9%	43.2%
Iraq	Amedi	2000	82.9%	92.4%	69.3%
Iraq	Amedi	2017	68.9%	82.8%	51.3%
Iraq	An Nasiriyah	2000	82.9%	91.1%	71.0%
Iraq	An Nasiriyah	2017	62.8%	75.3%	49.0%
Iraq	Anah	2000	71.0%	83.9%	57.3%
Iraq	Anah	2017	51.6%	67.7%	36.3%
Iraq	Ar Ramadi	2000	88.1%	92.1%	83.6%
Iraq	Ar Ramadi	2017	71.0%	77.9%	64.2%
Iraq	Ar Rutbah	2000	70.0%	75.3%	64.6%
Iraq	Ar Rutbah	2017	53.2%	60.1%	46.5%
Iraq	Arbil	2000	88.5%	95.0%	79.9%
Iraq	Arbil	2017	75.5%	86.8%	61.2%
Iraq	As Salman	2000	81.8%	89.4%	72.0%
Iraq	As Salman	2017	64.5%	76.7%	50.9%
Iraq	As Samawah	2000	69.2%	76.4%	61.6%
Iraq	As Samawah	2017	39.4%	47.3%	32.5%
Iraq	As Suwayrah	2000	74.0%	82.7%	63.0%
Iraq	As Suwayrah	2017	51.7%	63.3%	38.9%
Iraq	Ba'qubah	2000	74.1%	88.4%	54.5%
Iraq	Ba'qubah	2017	52.1%	72.3%	30.3%
Iraq	Badrah	2000	86.0%	94.2%	73.9%
Iraq	Badrah	2017	71.8%	85.6%	58.7%
Iraq	Balad	2000	67.8%	89.5%	38.0%
Iraq	Balad	2017	46.9%	72.0%	25.0%
Iraq	Balad Ruz	2000	80.4%	90.2%	69.6%
Iraq	Balad Ruz	2017	63.6%	76.9%	49.7%
Iraq	Basrah	2000	63.6%	75.8%	48.2%
Iraq	Basrah	2017	45.0%	60.9%	27.5%
Iraq	Bayji	2000	80.8%	90.7%	65.1%
Iraq	Bayji	2017	61.8%	77.1%	41.8%
Iraq	Chamchamal	2000	68.2%	82.1%	50.7%
Iraq	Chamchamal	2017	48.9%	66.2%	31.9%
Iraq	Chibayish	2000	77.4%	91.6%	54.3%
Iraq	Chibayish	2017	60.2%	78.9%	36.0%
Iraq	Choman	2000	88.4%	97.4%	74.1%
Iraq	Choman	2017	73.4%	91.1%	53.9%
Iraq	Dahuk	2000	74.8%	87.6%	58.5%
Iraq	Dahuk	2017	58.7%	76.9%	39.2%
Iraq	Daquq	2000	81.4%	92.9%	65.6%
Iraq	Daquq	2017	66.6%	83.5%	49.9%
Iraq	Darbandokeh	2000	69.3%	84.0%	45.6%
Iraq	Darbandokeh	2017	53.3%	73.7%	30.0%

Country	Second Administrative Unit	Year	Mean	Upper	Lower
Iraq	Dibis	2000	76.7%	96.1%	46.2%
Iraq	Dibis	2017	57.4%	86.5%	24.7%
Iraq	Dukan	2000	62.6%	84.5%	37.3%
Iraq	Dukan	2017	42.6%	66.8%	20.9%
Iraq	Halabja	2000	81.5%	92.1%	68.7%
Iraq	Halabja	2017	64.7%	80.1%	49.9%
Iraq	Hatra	2000	74.1%	86.9%	56.5%
Iraq	Hatra	2017	55.1%	71.9%	36.1%
Iraq	Haweeja	2000	86.4%	95.2%	73.5%
Iraq	Haweeja	2017	74.3%	87.0%	59.8%
Iraq	Hit	2000	84.5%	89.3%	79.0%
Iraq	Hit	2017	65.2%	72.6%	57.7%
Iraq	Kadhimiya	2000	95.6%	98.8%	87.6%
Iraq	Kadhimiya	2017	87.0%	95.3%	74.2%
Iraq	Kalar	2000	75.6%	91.7%	50.2%
Iraq	Kalar	2017	56.8%	80.2%	33.1%
Iraq	Karbala	2000	72.5%	82.1%	60.0%
Iraq	Karbala	2017	53.6%	65.4%	39.6%
Iraq	Khanaqin	2000	76.8%	89.2%	60.2%
Iraq	Khanaqin	2017	56.9%	73.4%	39.6%
Iraq	Kifri	2000	76.0%	89.3%	58.5%
Iraq	Kifri	2017	58.0%	74.7%	38.3%
Iraq	Kirkuk	2000	84.6%	93.1%	73.3%
Iraq	Kirkuk	2017	70.3%	83.3%	56.9%
Iraq	Koisnjaq	2000	75.2%	90.4%	55.8%
Iraq	Koisnjaq	2017	59.7%	78.0%	41.2%
Iraq	Mahmudiya	2000	79.9%	95.5%	57.7%
Iraq	Mahmudiya	2017	60.7%	87.0%	34.7%
Iraq	Makhmur	2000	80.3%	92.9%	64.4%
Iraq	Makhmur	2017	64.6%	81.7%	48.1%
Iraq	Mergasur	2000	74.3%	91.1%	50.3%
Iraq	Mergasur	2017	56.7%	80.1%	32.1%
Iraq	Mosul	2000	76.3%	84.9%	66.0%
Iraq	Mosul	2017	56.6%	69.0%	42.8%
Iraq	Najaf	2000	71.3%	80.0%	63.2%
Iraq	Najaf	2017	56.0%	63.9%	46.9%
Iraq	Penjwin	2000	73.3%	89.2%	53.7%
Iraq	Penjwin	2017	53.8%	75.7%	32.1%
Iraq	Pshdar	2000	83.7%	97.1%	61.4%
Iraq	Pshdar	2017	69.7%	91.1%	42.5%
Iraq	Qal'at Salih	2000	77.4%	93.0%	51.5%
Iraq	Qal'at Salih	2017	59.5%	81.2%	36.7%
Iraq	Rania	2000	82.7%	95.1%	63.2%
Iraq	Rania	2017	66.4%	86.5%	42.9%
Iraq	Refai	2000	76.1%	86.7%	63.9%
Iraq	Refai	2017	55.9%	71.1%	43.5%
Iraq	Rumaitha	2000	61.6%	71.5%	51.6%
Iraq	Rumaitha	2017	37.4%	47.4%	27.2%
Iraq	Samarra	2000	79.3%	89.0%	67.0%
Iraq	Samarra	2017	57.9%	71.3%	43.0%
Iraq	Shamiya	2000	76.1%	89.1%	54.7%
Iraq	Shamiya	2017	53.8%	71.2%	32.6%
Iraq	Shaqlawa	2000	83.0%	95.5%	65.9%
Iraq	Shaqlawa	2017	68.0%	87.4%	47.0%
Iraq	Sharbazher	2000	82.5%	95.9%	57.0%
Iraq	Sharbazher	2017	65.5%	86.9%	38.8%
Iraq	Shatrah	2000	79.8%	94.0%	52.6%
Iraq	Shatrah	2017	61.5%	82.3%	38.4%
Iraq	Shatt Al Arab	2000	52.8%	76.8%	27.3%
Iraq	Shatt Al Arab	2017	33.5%	60.4%	12.6%
Iraq	Shekhan	2000	78.5%	94.9%	53.6%
Iraq	Shekhan	2017	59.8%	86.0%	30.9%

Country	Second Administrative Unit	Year	Mean	Upper	Lower
Iraq	Simele	2000	71.3%	90.3%	48.5%
Iraq	Simele	2017	53.9%	76.8%	31.4%
Iraq	Sinjar	2000	74.4%	87.1%	60.1%
Iraq	Sinjar	2017	55.6%	74.1%	39.3%
Iraq	Soran	2000	80.3%	90.8%	65.3%
Iraq	Soran	2017	61.7%	76.5%	44.7%
Iraq	Sulaymaniya	2000	76.7%	85.5%	66.1%
Iraq	Sulaymaniya	2017	57.9%	71.2%	44.6%
Iraq	Suq ash Shuyukh	2000	77.0%	92.1%	53.7%
Iraq	Suq ash Shuyukh	2017	56.8%	79.3%	33.5%
Iraq	Talafar	2000	74.8%	85.3%	61.1%
Iraq	Talafar	2017	55.3%	69.0%	40.5%
Iraq	Tikrit	2000	72.5%	86.0%	54.8%
Iraq	Tikrit	2017	50.8%	68.5%	33.1%
Iraq	Tilkef	2000	68.8%	87.6%	46.5%
Iraq	Tilkef	2017	51.4%	75.5%	27.2%
Iraq	Touz Hourmato	2000	81.0%	91.3%	69.0%
Iraq	Touz Hourmato	2017	60.1%	76.8%	45.2%
Iraq	Zakho	2000	78.3%	88.1%	66.8%
Iraq	Zakho	2017	68.5%	82.3%	50.8%
Jordan	Aghwar Shamaliyyeh	2000	94.1%	94.8%	93.2%
Jordan	Aghwar Shamaliyyeh	2017	61.0%	64.4%	56.5%
Jordan	Ajloun	2000	85.2%	85.7%	84.7%
Jordan	Ajloun	2017	38.7%	39.4%	38.0%
Jordan	Al-Balqa	2000	94.0%	94.6%	93.4%
Jordan	Al-Balqa	2017	61.8%	64.1%	59.7%
Jordan	Amman	2000	91.8%	92.2%	91.4%
Jordan	Amman	2017	54.8%	55.8%	54.0%
Jordan	Aqaba	2000	97.4%	98.6%	94.6%
Jordan	Aqaba	2017	83.9%	88.6%	74.7%
Jordan	Ar-Ruwayshid	2000	92.0%	94.5%	89.2%
Jordan	Ar-Ruwayshid	2017	70.2%	75.4%	64.9%
Jordan	Ardhah	2000	95.2%	95.6%	94.6%
Jordan	Ardhah	2017	65.4%	67.3%	63.2%
Jordan	Ayy	2000	95.3%	96.1%	93.8%
Jordan	Ayy	2017	75.6%	79.3%	69.8%
Jordan	Azraq	2000	93.5%	96.3%	86.6%
Jordan	Azraq	2017	77.6%	84.1%	67.3%
Jordan	Bal'ama	2000	95.1%	95.5%	94.7%
Jordan	Bal'ama	2017	63.9%	65.6%	62.3%
Jordan	Bani Kenanah	2000	71.4%	73.4%	69.2%
Jordan	Bani Kenanah	2017	24.2%	25.6%	22.6%
Jordan	Bierain	2000	94.4%	94.7%	93.9%
Jordan	Bierain	2017	61.7%	63.3%	59.8%
Jordan	Bsaira	2000	98.6%	98.8%	98.3%
Jordan	Bsaira	2017	86.9%	88.2%	85.6%
Jordan	Dair Alla	2000	97.5%	97.8%	97.2%
Jordan	Dair Alla	2017	81.5%	83.2%	79.5%
Jordan	Dhiban	2000	91.7%	93.5%	89.4%
Jordan	Dhiban	2017	58.4%	62.1%	55.0%
Jordan	Faqqoo'	2000	89.5%	90.5%	88.4%
Jordan	Faqqoo'	2017	53.4%	55.4%	51.1%
Jordan	Ghour El-Mazra'ah	2000	93.7%	94.8%	91.8%

Country	Second Administrative Unit	Year	Mean	Upper	Lower
Jordan	Ghour El-Mazra'ah	2017	63.7%	68.2%	58.9%
Jordan	Ghour Essafi	2000	89.7%	92.9%	85.3%
Jordan	Ghour Essafi	2017	69.4%	77.6%	58.1%
Jordan	Hariema	2000	85.2%	86.0%	84.4%
Jordan	Hariema	2017	35.4%	36.9%	33.9%
Jordan	Hesa	2000	97.7%	98.6%	95.6%
Jordan	Hesa	2017	84.5%	88.8%	77.6%
Jordan	Husseiniyyeh	2000	95.8%	97.4%	92.4%
Jordan	Husseiniyyeh	2017	78.0%	83.2%	71.2%
Jordan	Iel	2000	95.9%	96.9%	94.4%
Jordan	Iel	2017	75.3%	78.9%	70.8%
Jordan	Irbid	2000	82.5%	83.7%	81.4%
Jordan	Irbid	2017	32.4%	34.4%	30.5%
Jordan	Jarash	2000	90.1%	90.5%	89.7%
Jordan	Jarash	2017	49.4%	50.3%	48.3%
Jordan	Jizeh	2000	91.3%	93.5%	88.5%
Jordan	Jizeh	2017	60.0%	65.8%	54.4%
Jordan	Karak	2000	91.3%	91.8%	90.6%
Jordan	Karak	2017	48.6%	50.4%	46.7%
Jordan	Kofranjah	2000	89.9%	90.6%	89.1%
Jordan	Kofranjah	2017	46.6%	48.6%	44.9%
Jordan	Kora	2000	78.8%	80.0%	77.5%
Jordan	Kora	2017	26.3%	27.8%	24.5%
Jordan	Ma'an	2000	89.2%	91.1%	86.6%
Jordan	Ma'an	2017	57.5%	60.8%	54.0%
Jordan	Madaba	2000	95.4%	95.7%	94.8%
Jordan	Madaba	2017	67.4%	68.5%	66.1%
Jordan	Mafraq	2000	94.3%	94.7%	93.9%
Jordan	Mafraq	2017	62.7%	64.1%	61.3%
Jordan	Mazar Janoobi	2000	92.2%	92.6%	91.7%
Jordan	Mazar Janoobi	2017	53.7%	55.1%	52.1%
Jordan	Mazar Shamali	2000	76.5%	77.8%	75.0%
Jordan	Mazar Shamali	2017	27.3%	28.5%	25.8%
Jordan	Mowaqqar	2000	87.4%	89.9%	83.7%
Jordan	Mowaqqar	2017	48.4%	55.0%	42.6%
Jordan	Na'oor	2000	92.6%	93.1%	92.0%
Jordan	Na'oor	2017	55.4%	58.1%	52.8%
Jordan	Qasr	2000	88.0%	89.1%	86.7%
Jordan	Qasr	2017	44.6%	46.7%	42.2%
Jordan	Quaira	2000	96.9%	98.3%	95.0%
Jordan	Quaira	2017	85.4%	89.9%	80.4%
Jordan	Ramtha	2000	90.5%	91.8%	89.1%
Jordan	Ramtha	2017	51.0%	54.3%	47.9%
Jordan	Sabha	2000	94.2%	95.7%	92.4%
Jordan	Sabha	2017	74.0%	77.3%	69.2%
Jordan	Sahab	2000	93.8%	94.6%	92.0%
Jordan	Sahab	2017	60.8%	63.9%	55.8%
Jordan	Salt	2000	92.7%	93.1%	92.4%
Jordan	Salt	2017	58.1%	59.3%	57.1%
Jordan	Sama Serhan	2000	95.5%	95.9%	94.9%
Jordan	Sama Serhan	2017	70.1%	72.1%	67.7%
Jordan	Shoabak	2000	95.4%	96.4%	94.1%
Jordan	Shoabak	2017	73.8%	77.9%	68.2%
Jordan	Shooneh Janoobiyyeh	2000	87.7%	89.4%	85.6%
Jordan	Shooneh Janoobiyyeh	2017	60.2%	64.1%	56.0%

Country	Second Administrative Unit	Year	Mean	Upper	Lower
Jordan	Tafileh	2000	96.2%	97.1%	94.3%
Jordan	Tafileh	2017	81.1%	83.5%	77.9%
Jordan	Tayybeh	2000	78.8%	79.8%	77.7%
Jordan	Tayybeh	2017	37.0%	37.8%	36.1%
Jordan	Um El-Basatien	2000	91.2%	92.1%	90.2%
Jordan	Um El-Basatien	2017	51.4%	54.6%	48.7%
Jordan	Wadi Arabah	2000	94.0%	98.2%	85.8%
Jordan	Wadi Arabah	2017	75.7%	88.1%	57.6%
Jordan	Wadi Essier	2000	94.4%	94.6%	94.2%
Jordan	Wadi Essier	2017	61.6%	62.5%	60.7%
Jordan	Wadi Musa	2000	94.0%	94.5%	93.5%
Jordan	Wadi Musa	2017	58.5%	60.3%	56.9%
Jordan	Wastiyyeh	2000	85.7%	86.4%	85.1%
Jordan	Wastiyyeh	2017	36.4%	37.4%	35.3%
Jordan	Zarqa	2000	95.3%	95.8%	94.5%
Jordan	Zarqa	2017	67.0%	69.0%	64.9%
Libya	Al Butnan	2000	82.7%	86.4%	77.4%
Libya	Al Butnan	2017	83.2%	86.9%	77.9%
Libya	Al Jabal al Akhdar	2000	69.3%	71.9%	66.2%
Libya	Al Jabal al Akhdar	2017	70.2%	73.1%	66.9%
Libya	Al Jabal al Gharbi	2000	35.2%	40.3%	29.7%
Libya	Al Jabal al Gharbi	2017	33.5%	38.5%	28.7%
Libya	Al Jifarah	2000	41.6%	48.7%	35.0%
Libya	Al Jifarah	2017	42.3%	49.5%	35.7%
Libya	Al Jufrah	2000	92.5%	93.5%	91.5%
Libya	Al Jufrah	2017	92.8%	93.6%	91.9%
Libya	Al Kufrah	2000	97.3%	99.0%	94.5%
Libya	Al Kufrah	2017	97.1%	98.9%	94.1%
Libya	Al Marj	2000	50.7%	55.6%	45.8%
Libya	Al Marj	2017	51.7%	56.5%	46.5%
Libya	Al Marqab	2000	23.0%	27.1%	19.8%
Libya	Al Marqab	2017	23.9%	28.0%	20.8%
Libya	Al Wahat	2000	94.1%	95.2%	92.9%
Libya	Al Wahat	2017	94.4%	95.2%	93.6%
Libya	An Nuqat al Khams	2000	21.2%	24.8%	18.1%
Libya	An Nuqat al Khams	2017	21.3%	25.1%	18.1%
Libya	Az Zawiyah	2000	57.3%	61.9%	53.4%
Libya	Az Zawiyah	2017	57.7%	62.7%	53.6%
Libya	Benghazi	2000	96.0%	96.5%	95.5%
Libya	Benghazi	2017	96.2%	96.6%	95.6%
Libya	Darnah	2000	94.1%	94.9%	92.9%
Libya	Darnah	2017	94.1%	95.0%	92.8%
Libya	Ghat	2000	95.3%	96.0%	94.7%
Libya	Ghat	2017	95.5%	96.1%	94.8%
Libya	Misratah	2000	73.4%	75.4%	71.5%
Libya	Misratah	2017	74.2%	76.1%	72.2%
Libya	Murzuq	2000	93.9%	97.4%	87.4%
Libya	Murzuq	2017	93.8%	97.6%	87.0%
Libya	Nalut	2000	67.1%	69.7%	64.3%
Libya	Nalut	2017	68.0%	70.8%	65.1%
Libya	Sabha	2000	99.7%	99.7%	99.5%
Libya	Sabha	2017	99.7%	99.8%	99.5%
Libya	Surt	2000	90.0%	91.1%	88.8%
Libya	Surt	2017	90.3%	91.5%	89.0%

Country	Second Administrative Unit	Year	Mean	Upper	Lower
Libya	Tripoli	2000	31.7%	36.1%	27.0%
Libya	Tripoli	2017	31.3%	35.4%	27.2%
Libya	Wadi al Hayat	2000	99.0%	99.4%	98.5%
Libya	Wadi al Hayat	2017	99.0%	99.3%	98.6%
Libya	Wadi ash Shati'	2000	99.0%	99.2%	98.8%
Libya	Wadi ash Shati'	2017	99.1%	99.3%	98.9%
Morocco	Agadir-Ida ou Tanane	2000	89.9%	95.7%	81.2%
Morocco	Agadir-Ida ou Tanane	2017	89.6%	95.2%	81.0%
Morocco	Al Haouz	2000	48.0%	61.6%	33.9%
Morocco	Al Haouz	2017	47.7%	61.0%	33.8%
Morocco	Al Hoceïma	2000	30.5%	44.2%	17.7%
Morocco	Al Hoceïma	2017	28.3%	41.9%	15.8%
Morocco	Assa-Zag	2000	55.2%	99.2%	1.0%
Morocco	Assa-Zag	2017	54.4%	99.0%	0.9%
Morocco	Azilal	2000	29.1%	43.5%	17.2%
Morocco	Azilal	2017	28.1%	42.6%	16.5%
Morocco	Ben Slimane	2000	77.3%	91.0%	60.9%
Morocco	Ben Slimane	2017	76.6%	90.2%	60.0%
Morocco	Béni Mellal	2000	65.3%	81.8%	44.0%
Morocco	Béni Mellal	2017	64.1%	80.9%	43.0%
Morocco	Berkane Taourirt	2000	54.8%	90.5%	14.1%
Morocco	Berkane Taourirt	2017	53.1%	89.0%	13.6%
Morocco	Boulemane	2000	40.0%	76.2%	9.8%
Morocco	Boulemane	2017	38.9%	74.9%	9.1%
Morocco	Casablanca	2000	92.3%	96.2%	87.8%
Morocco	Casablanca	2017	90.5%	95.3%	85.2%
Morocco	Chefchaouen	2000	30.4%	50.4%	15.9%
Morocco	Chefchaouen	2017	29.4%	50.1%	14.8%
Morocco	Chichaoua	2000	14.7%	28.5%	4.9%
Morocco	Chichaoua	2017	13.7%	27.7%	4.4%
Morocco	Chtouka-Aït Baha	2000	70.1%	88.3%	42.9%
Morocco	Chtouka-Aït Baha	2017	69.9%	87.8%	42.1%
Morocco	El Hajeb	2000	39.3%	57.2%	14.6%
Morocco	El Hajeb	2017	38.2%	55.3%	13.8%
Morocco	El Jadida	2000	53.3%	65.4%	38.6%
Morocco	El Jadida	2017	53.2%	66.0%	37.6%
Morocco	El Kelaâ des Sraghna	2000	41.7%	63.3%	22.1%
Morocco	El Kelaâ des Sraghna	2017	40.7%	62.3%	21.6%
Morocco	Errachidia	2000	68.1%	86.6%	51.8%
Morocco	Errachidia	2017	67.0%	85.6%	51.3%
Morocco	Essaouira	2000	39.0%	64.8%	13.2%
Morocco	Essaouira	2017	38.5%	63.9%	12.7%
Morocco	Fahs Anjra	2000	44.1%	59.6%	30.0%
Morocco	Fahs Anjra	2017	40.8%	59.0%	26.0%
Morocco	Fès	2000	93.4%	98.4%	78.2%
Morocco	Fès	2017	92.2%	98.1%	76.9%
Morocco	Figuig	2000	43.5%	80.4%	12.4%
Morocco	Figuig	2017	43.3%	80.1%	11.9%
Morocco	Guelmim	2000	88.7%	97.8%	70.2%
Morocco	Guelmim	2017	88.8%	98.0%	70.7%
Morocco	Ifrane	2000	82.9%	96.5%	60.0%
Morocco	Ifrane	2017	81.2%	95.9%	59.1%

Country	Second Administrative Unit	Year	Mean	Upper	Lower
Morocco	Inezgane-Aït Melloul	2000	88.3%	93.2%	82.2%
Morocco	Inezgane-Aït Melloul	2017	85.6%	91.0%	78.8%
Morocco	Jerada	2000	32.3%	67.4%	6.3%
Morocco	Jerada	2017	31.2%	64.9%	6.1%
Morocco	Kénitra	2000	68.5%	82.2%	52.8%
Morocco	Kénitra	2017	66.9%	80.7%	50.6%
Morocco	Khémisset	2000	35.9%	88.6%	15.2%
Morocco	Khémisset	2017	35.3%	88.0%	15.0%
Morocco	Khénifra	2000	65.6%	84.2%	45.2%
Morocco	Khénifra	2017	64.2%	83.5%	43.3%
Morocco	Khouribga	2000	38.8%	50.8%	31.0%
Morocco	Khouribga	2017	38.2%	49.6%	31.0%
Morocco	Laâyoune	2000	39.3%	98.7%	0.0%
Morocco	Laâyoune	2017	38.7%	98.7%	0.0%
Morocco	Larache	2000	65.0%	78.8%	50.9%
Morocco	Larache	2017	63.2%	77.7%	48.9%
Morocco	Marrakech	2000	88.9%	94.6%	80.8%
Morocco	Marrakech	2017	88.8%	94.6%	81.1%
Morocco	Meknès	2000	85.6%	91.3%	73.4%
Morocco	Meknès	2017	84.9%	91.6%	71.0%
Morocco	Mohammedia	2000	97.8%	99.6%	94.4%
Morocco	Mohammedia	2017	97.6%	99.5%	94.0%
Morocco	Nador	2000	47.4%	66.1%	31.4%
Morocco	Nador	2017	47.7%	66.9%	30.8%
Morocco	Ouarzazate	2000	59.8%	77.5%	37.5%
Morocco	Ouarzazate	2017	58.7%	76.2%	36.7%
Morocco	Oujda Angad	2000	93.4%	98.4%	84.9%
Morocco	Oujda Angad	2017	91.9%	97.7%	82.2%
Morocco	Rabat	2000	98.5%	99.4%	96.5%
Morocco	Rabat	2017	98.1%	99.1%	95.3%
Morocco	Safi	2000	45.6%	59.3%	35.3%
Morocco	Safi	2017	45.3%	58.5%	35.6%
Morocco	Salé	2000	97.1%	99.2%	92.6%
Morocco	Salé	2017	96.7%	99.0%	90.9%
Morocco	Sefrou	2000	64.6%	78.6%	47.0%
Morocco	Sefrou	2017	62.9%	78.0%	44.4%
Morocco	Settat	2000	23.7%	40.7%	11.1%
Morocco	Settat	2017	23.2%	37.9%	11.4%
Morocco	Sidi Kacem	2000	48.7%	62.8%	38.2%
Morocco	Sidi Kacem	2017	47.6%	60.4%	37.7%
Morocco	Skhirate-Témara	2000	91.9%	98.8%	78.5%
Morocco	Skhirate-Témara	2017	91.5%	98.8%	78.8%
Morocco	Tan-Tan	2000	38.7%	66.6%	12.3%
Morocco	Tan-Tan	2017	37.4%	68.9%	8.9%
Morocco	Tanger-Assilah	2000	47.4%	54.6%	40.0%
Morocco	Tanger-Assilah	2017	43.4%	51.0%	35.9%
Morocco	Taounate	2000	12.3%	21.6%	5.0%
Morocco	Taounate	2017	11.9%	21.2%	4.7%
Morocco	Taroudannt	2000	58.6%	73.9%	43.2%
Morocco	Taroudannt	2017	57.7%	73.1%	42.0%
Morocco	Tata	2000	59.2%	87.6%	21.7%
Morocco	Tata	2017	58.8%	87.7%	21.8%
Morocco	Taza	2000	54.7%	66.5%	41.8%
Morocco	Taza	2017	54.3%	66.4%	41.2%
Morocco	Tétouan	2000	64.1%	73.7%	50.9%
Morocco	Tétouan	2017	62.5%	72.9%	46.5%

Country	Second Administrative Unit	Year	Mean	Upper	Lower
Morocco	Tiznit	2000	72.1%	90.3%	44.3%
Morocco	Tiznit	2017	71.3%	89.7%	43.9%
Morocco	Zagora	2000	74.5%	96.2%	49.5%
Morocco	Zagora	2017	74.5%	96.5%	48.5%
Morocco	Zouagha-Moulay Yacoub	2000	75.8%	85.0%	66.3%
Morocco	Zouagha-Moulay Yacoub	2017	74.4%	83.1%	64.6%
Sudan	Abu Hamad	2000	61.1%	64.0%	58.2%
Sudan	Abu Hamad	2017	44.4%	47.7%	41.2%
Sudan	Abu Jubaiyah	2000	4.5%	6.4%	3.2%
Sudan	Abu Jubaiyah	2017	2.2%	3.1%	1.6%
Sudan	Abyei	2000	6.5%	8.3%	5.3%
Sudan	Abyei	2017	3.4%	4.4%	2.7%
Sudan	Ad Damazin	2000	25.3%	33.6%	19.2%
Sudan	Ad Damazin	2017	15.1%	22.1%	10.5%
Sudan	Ad Damer	2000	57.2%	66.8%	47.4%
Sudan	Ad Damer	2017	40.3%	51.7%	30.3%
Sudan	Ad Dinder	2000	48.1%	52.0%	44.2%
Sudan	Ad Dinder	2017	32.0%	36.4%	28.3%
Sudan	Ad Douiem	2000	40.5%	45.6%	35.5%
Sudan	Ad Douiem	2017	25.7%	29.7%	21.6%
Sudan	Addabah	2000	71.3%	74.0%	68.2%
Sudan	Addabah	2017	55.6%	58.8%	52.4%
Sudan	Al Deain	2000	18.4%	20.8%	16.4%
Sudan	Al Deain	2017	10.3%	11.8%	8.9%
Sudan	Al Fasher	2000	13.8%	15.5%	12.0%
Sudan	Al Fasher	2017	7.4%	8.5%	6.4%
Sudan	Al Faw	2000	32.0%	35.1%	28.7%
Sudan	Al Faw	2017	19.6%	21.8%	17.3%
Sudan	Al Fushqa	2000	36.1%	39.7%	33.1%
Sudan	Al Fushqa	2017	22.7%	25.5%	20.6%
Sudan	Al Gadaref	2000	36.3%	42.1%	30.4%
Sudan	Al Gadaref	2017	22.1%	26.7%	17.7%
Sudan	Al Galabat	2000	28.8%	32.3%	25.3%
Sudan	Al Galabat	2017	16.7%	19.3%	14.5%
Sudan	Al Gash	2000	41.8%	47.1%	36.4%
Sudan	Al Gash	2017	26.7%	31.2%	22.3%
Sudan	Al Geneina	2000	15.0%	17.0%	13.5%
Sudan	Al Geneina	2017	8.2%	9.5%	7.1%
Sudan	Al Gutaina	2000	36.9%	41.2%	32.5%
Sudan	Al Gutaina	2017	23.1%	26.6%	20.2%
Sudan	Al Jabalian	2000	41.4%	48.6%	35.6%
Sudan	Al Jabalian	2017	25.3%	31.0%	20.7%
Sudan	Al Kamlin	2000	70.1%	73.6%	65.7%
Sudan	Al Kamlin	2017	52.7%	57.7%	47.4%
Sudan	Al Kurumik	2000	20.0%	24.7%	15.4%
Sudan	Al Kurumik	2017	11.2%	14.3%	8.2%
Sudan	Al Mahagil	2000	71.0%	75.0%	66.4%
Sudan	Al Mahagil	2017	56.3%	61.6%	50.7%
Sudan	Al Matammah	2000	60.3%	64.5%	56.1%
Sudan	Al Matammah	2017	43.6%	48.5%	39.6%
Sudan	Al Rahd	2000	31.9%	36.6%	27.3%
Sudan	Al Rahd	2017	19.3%	22.9%	16.0%
Sudan	Al Roseires	2000	24.4%	29.1%	19.6%
Sudan	Al Roseires	2017	14.3%	18.6%	10.7%
Sudan	As Salam	2000	5.6%	7.9%	3.9%
Sudan	As Salam	2017	3.0%	4.4%	2.0%

Country	Second Administrative Unit	Year	Mean	Upper	Lower
Sudan	Atbara	2000	56.5%	62.6%	50.1%
Sudan	Atbara	2017	39.0%	46.0%	33.0%
Sudan	Bara	2000	24.0%	28.7%	20.1%
Sudan	Bara	2017	13.9%	17.4%	11.0%
Sudan	Baw	2000	20.3%	25.2%	16.3%
Sudan	Baw	2017	11.5%	15.0%	8.8%
Sudan	Berber	2000	54.0%	60.1%	48.7%
Sudan	Berber	2017	37.3%	43.4%	32.3%
Sudan	Buram	2000	19.2%	21.9%	16.6%
Sudan	Buram	2017	10.8%	12.4%	9.0%
Sudan	Dilling	2000	3.6%	4.9%	2.8%
Sudan	Dilling	2017	1.9%	2.6%	1.5%
Sudan	Dongola	2000	71.9%	74.3%	69.2%
Sudan	Dongola	2017	56.3%	59.5%	52.7%
Sudan	East al Gazera	2000	77.3%	80.5%	74.1%
Sudan	East al Gazera	2017	63.6%	68.0%	59.5%
Sudan	En Nuhud	2000	7.7%	8.9%	6.8%
Sudan	En Nuhud	2017	4.0%	4.7%	3.5%
Sudan	Geissan	2000	18.4%	22.4%	15.2%
Sudan	Geissan	2017	10.2%	12.8%	8.2%
Sudan	Ghebeish	2000	7.7%	9.3%	6.1%
Sudan	Ghebeish	2017	4.0%	4.9%	3.2%
Sudan	Halayeb	2000	33.6%	40.5%	26.7%
Sudan	Halayeb	2017	24.5%	32.9%	16.3%
Sudan	Hamashkorieb	2000	44.5%	50.6%	38.5%
Sudan	Hamashkorieb	2017	29.1%	34.8%	24.2%
Sudan	Id El Ghanem	2000	17.7%	20.6%	14.9%
Sudan	Id El Ghanem	2017	9.8%	11.7%	8.2%
Sudan	Jebrat al Sheikh	2000	29.3%	31.9%	26.4%
Sudan	Jebrat al Sheikh	2017	17.7%	20.0%	15.5%
Sudan	Kabkabiya	2000	14.6%	16.7%	12.7%
Sudan	Kabkabiya	2017	7.9%	9.0%	6.8%
Sudan	Kadugli	2000	3.4%	4.2%	2.8%
Sudan	Kadugli	2017	1.7%	2.2%	1.4%
Sudan	Karary	2000	67.9%	71.4%	63.9%
Sudan	Karary	2017	50.8%	55.1%	45.9%
Sudan	Kas	2000	17.4%	24.0%	12.3%
Sudan	Kas	2017	9.5%	13.5%	6.4%
Sudan	Kassala	2000	39.7%	46.2%	33.6%
Sudan	Kassala	2017	25.1%	30.6%	20.7%
Sudan	Khartoum	2000	67.2%	70.9%	63.2%
Sudan	Khartoum	2017	50.4%	54.9%	45.8%
Sudan	Khartoum Bahri	2000	64.4%	70.8%	58.8%
Sudan	Khartoum Bahri	2017	47.7%	55.4%	41.8%
Sudan	Kosti	2000	37.7%	44.0%	32.0%
Sudan	Kosti	2017	23.5%	28.9%	18.9%
Sudan	Kutum	2000	14.9%	17.1%	12.9%
Sudan	Kutum	2017	8.1%	9.5%	6.9%
Sudan	Lagawa	2000	5.0%	6.2%	4.0%
Sudan	Lagawa	2017	2.6%	3.2%	2.1%
Sudan	Mellit	2000	15.4%	17.7%	13.3%
Sudan	Mellit	2017	8.5%	10.0%	7.2%
Sudan	Merawi	2000	71.4%	74.1%	68.8%
Sudan	Merawi	2017	55.5%	59.1%	51.9%
Sudan	Mukjar	2000	15.3%	17.8%	13.2%
Sudan	Mukjar	2017	8.4%	10.0%	7.1%
Sudan	Nahr Atbara	2000	38.1%	42.1%	34.2%
Sudan	Nahr Atbara	2017	23.8%	27.1%	20.7%

Country	Second Administrative Unit	Year	Mean	Upper	Lower
Sudan	North al Gazera	2000	74.4%	79.3%	68.6%
Sudan	North al Gazera	2017	59.4%	66.0%	51.6%
Sudan	Nyala	2000	17.0%	20.7%	14.0%
Sudan	Nyala	2000	16.5%	19.8%	13.4%
Sudan	Nyala	2017	9.3%	11.4%	7.5%
Sudan	Nyala	2017	9.1%	11.3%	7.1%
Sudan	Omdurman	2000	60.9%	69.2%	52.4%
Sudan	Omdurman	2017	44.3%	53.6%	35.5%
Sudan	Port Sudan	2000	25.1%	30.4%	20.2%
Sudan	Port Sudan	2017	15.2%	18.8%	12.3%
Sudan	Rashad	2000	4.4%	5.8%	3.3%
Sudan	Rashad	2017	2.2%	3.0%	1.7%
Sudan	Sennar	2000	49.2%	52.9%	45.1%
Sudan	Sennar	2017	33.0%	36.2%	29.8%
Sudan	Seteet	2000	38.9%	43.1%	34.9%
Sudan	Seteet	2017	24.8%	28.2%	21.6%
Sudan	Sharg En Nile	2000	62.0%	68.3%	54.2%
Sudan	Sharg En Nile	2017	44.7%	52.4%	36.4%
Sudan	Sharq al Gazera	2000	71.3%	76.2%	63.7%
Sudan	Sharq al Gazera	2017	55.3%	61.9%	47.4%
Sudan	Sheikan	2000	28.5%	32.1%	25.9%
Sudan	Sheikan	2017	16.7%	18.8%	14.9%
Sudan	Shendi	2000	58.6%	62.6%	54.9%
Sudan	Shendi	2017	41.9%	46.3%	37.8%
Sudan	Singa	2000	47.6%	51.7%	43.8%
Sudan	Singa	2017	31.7%	36.1%	28.2%
Sudan	Sinkat	2000	30.9%	33.1%	28.4%
Sudan	Sinkat	2017	18.3%	20.1%	16.7%
Sudan	South al Gazera	2000	71.2%	75.1%	67.2%
Sudan	South al Gazera	2017	56.5%	62.0%	51.0%
Sudan	South Khartoum	2000	66.1%	70.7%	61.4%
Sudan	South Khartoum	2017	49.3%	54.8%	43.7%
Sudan	Sowdari	2000	27.6%	30.6%	24.4%
Sudan	Sowdari	2017	16.4%	18.8%	14.2%
Sudan	Talodi	2000	4.1%	5.7%	3.0%
Sudan	Talodi	2017	2.0%	2.8%	1.4%
Sudan	Tokar	2000	30.3%	32.4%	28.5%
Sudan	Tokar	2017	18.3%	19.8%	16.8%
Sudan	Tulus	2000	18.3%	25.0%	12.6%
Sudan	Tulus	2017	10.1%	14.4%	6.7%
Sudan	Um Al Gura	2000	77.7%	83.8%	70.0%
Sudan	Um Al Gura	2017	63.9%	72.6%	54.1%
Sudan	Um Badda	2000	57.2%	65.8%	49.6%
Sudan	Um Badda	2017	39.9%	48.9%	32.7%
Sudan	Um Kadada	2000	13.6%	15.8%	11.8%
Sudan	Um Kadada	2017	7.3%	8.6%	6.3%
Sudan	Um Rawaba	2000	25.1%	27.4%	22.8%
Sudan	Um Rawaba	2017	14.8%	16.5%	13.2%
Sudan	Wadi Halfa	2000	70.4%	74.2%	66.3%
Sudan	Wadi Halfa	2017	55.5%	61.4%	49.9%
Sudan	Zallingi	2000	14.2%	16.2%	12.0%
Sudan	Zallingi	2017	7.8%	8.9%	6.6%
Syria	'Ayn al-'Arab	2000	59.8%	77.2%	39.9%
Syria	'Ayn al-'Arab	2017	59.4%	76.3%	41.4%

Country	Second Administrative Unit	Year	Mean	Upper	Lower
Syria	A'zaz	2000	75.2%	88.5%	58.2%
Syria	A'zaz	2017	75.4%	88.6%	57.9%
Syria	Abu Kamal	2000	80.7%	87.6%	72.1%
Syria	Abu Kamal	2017	81.4%	88.0%	73.4%
Syria	Afrin	2000	51.1%	68.7%	32.6%
Syria	Afrin	2017	51.7%	69.4%	33.9%
Syria	Al Bab	2000	56.8%	71.3%	41.1%
Syria	Al Bab	2017	57.7%	71.1%	42.3%
Syria	Al Qamishli	2000	80.3%	93.4%	57.3%
Syria	Al Qamishli	2017	80.3%	92.9%	57.8%
Syria	Al-Haffah	2000	84.1%	93.9%	68.1%
Syria	Al-Haffah	2017	84.8%	94.2%	69.6%
Syria	Al-Hasakah	2000	83.4%	93.5%	63.2%
Syria	Al-Hasakah	2017	83.3%	93.2%	65.0%
Syria	Al-Malikiyah	2000	70.2%	88.4%	46.3%
Syria	Al-Malikiyah	2017	71.0%	88.2%	46.3%
Syria	Al-Mukharram	2000	75.3%	86.8%	60.8%
Syria	Al-Mukharram	2017	75.5%	86.9%	61.4%
Syria	Al-Qusayr	2000	71.3%	85.2%	54.0%
Syria	Al-Qusayr	2017	71.7%	85.3%	53.5%
Syria	Al-Qutayfah	2000	81.4%	95.3%	54.3%
Syria	Al-Qutayfah	2017	81.7%	94.8%	56.8%
Syria	An-Nabk	2000	97.4%	99.8%	88.0%
Syria	An-Nabk	2017	97.2%	99.8%	88.3%
Syria	Ar-Raqqah	2000	94.7%	96.7%	92.0%
Syria	Ar-Raqqah	2017	94.1%	96.3%	91.2%
Syria	Ar-Rastan	2000	82.6%	92.8%	68.0%
Syria	Ar-Rastan	2017	82.6%	92.6%	67.7%
Syria	Arihah	2000	81.7%	93.3%	64.3%
Syria	Arihah	2017	81.6%	93.0%	65.7%
Syria	As-Safirah	2000	55.8%	69.9%	40.4%
Syria	As-Safirah	2017	59.2%	73.5%	44.8%
Syria	As-Sanamayn	2000	92.0%	97.2%	82.7%
Syria	As-Sanamayn	2017	91.4%	97.0%	82.0%
Syria	As-Suqaylabiyah	2000	89.6%	96.3%	80.2%
Syria	As-Suqaylabiyah	2017	89.7%	96.5%	80.4%
Syria	As-Suwayda	2000	98.7%	99.5%	96.5%
Syria	As-Suwayda	2017	98.5%	99.5%	96.3%
Syria	Ash-Shaykh Badr	2000	85.8%	92.7%	75.4%
Syria	Ash-Shaykh Badr	2017	85.9%	92.8%	75.7%
Syria	At-Tall	2000	97.4%	99.6%	91.5%
Syria	At-Tall	2017	97.7%	99.6%	92.4%
Syria	Ath-Thawrah	2000	82.9%	89.6%	73.7%
Syria	Ath-Thawrah	2017	82.1%	88.7%	73.1%
Syria	Baniyas	2000	95.2%	98.6%	89.2%
Syria	Baniyas	2017	95.2%	98.7%	89.0%
Syria	Damascus	2000	95.8%	97.3%	93.7%
Syria	Damascus	2017	96.0%	97.4%	94.0%
Syria	Daraa	2000	91.8%	97.4%	82.0%
Syria	Daraa	2017	91.8%	97.4%	82.3%
Syria	Darayya	2000	44.9%	56.8%	31.7%
Syria	Darayya	2017	44.5%	56.0%	31.8%
Syria	Deir ez-Zor	2000	86.0%	91.5%	77.9%
Syria	Deir ez-Zor	2017	86.2%	91.6%	78.3%
Syria	Duma	2000	63.4%	71.8%	53.1%
Syria	Duma	2017	63.3%	72.1%	53.4%

Piped Water Proportion by Second Administrative Unit *(continued)*

Country	Second Administrative Unit	Year	Mean	Upper	Lower
Syria	Duraykish	2000	90.4%	96.3%	78.0%
Syria	Duraykish	2017	90.7%	96.4%	79.0%
Syria	Hama	2000	91.1%	95.8%	82.2%
Syria	Hama	2017	90.7%	95.5%	82.2%
Syria	Harem	2000	75.4%	94.1%	50.3%
Syria	Harem	2017	75.1%	93.9%	49.1%
Syria	Hims	2000	87.0%	93.1%	77.8%
Syria	Hims	2017	86.8%	92.8%	77.3%
Syria	Idlib	2000	81.1%	95.7%	54.2%
Syria	Idlib	2017	80.9%	95.6%	54.5%
Syria	Izra'	2000	89.7%	97.7%	71.0%
Syria	Izra'	2017	89.5%	97.6%	70.9%
Syria	Jabal Sam'an	2000	89.5%	94.8%	80.4%
Syria	Jabal Sam'an	2017	88.9%	94.8%	79.6%
Syria	Jableh	2000	87.6%	95.1%	74.8%
Syria	Jableh	2017	87.2%	95.3%	73.3%
Syria	Jarabulus	2000	55.7%	77.6%	34.2%
Syria	Jarabulus	2017	54.8%	77.4%	33.1%
Syria	Jisr ash-Shugur	2000	81.6%	93.0%	64.1%
Syria	Jisr ash-Shugur	2017	81.7%	93.0%	65.0%
Syria	Latakia	2000	86.5%	95.2%	70.8%
Syria	Latakia	2017	86.7%	95.5%	71.3%
Syria	Ma'arrat al-Numan	2000	79.7%	89.4%	63.4%
Syria	Ma'arrat al-Numan	2017	79.6%	89.4%	63.9%
Syria	Manbij	2000	52.8%	68.1%	39.3%
Syria	Manbij	2017	53.8%	67.8%	40.3%
Syria	Markaz Rif Dimashq	2000	61.5%	71.7%	50.0%
Syria	Markaz Rif Dimashq	2017	61.7%	71.7%	49.6%
Syria	Masyaf	2000	90.5%	96.1%	80.0%
Syria	Masyaf	2017	90.9%	96.5%	81.0%
Syria	Mayadin	2000	97.2%	99.0%	94.1%
Syria	Mayadin	2017	96.9%	98.8%	94.2%
Syria	Muhardeh	2000	98.1%	99.5%	94.9%
Syria	Muhardeh	2017	98.0%	99.5%	94.8%
Syria	Palmyra	2000	87.8%	91.7%	83.3%
Syria	Palmyra	2017	88.1%	91.9%	84.1%
Syria	Qardaha	2000	70.4%	85.6%	48.3%
Syria	Qardaha	2017	70.6%	85.4%	48.9%
Syria	Qatana	2000	75.7%	89.5%	58.6%
Syria	Qatana	2017	76.2%	89.9%	59.3%
Syria	Quneitra	2000	95.0%	98.7%	87.7%
Syria	Quneitra	2017	93.9%	98.5%	84.7%
Syria	Ra's al-'Ayn	2000	71.2%	82.7%	54.8%
Syria	Ra's al-'Ayn	2017	71.4%	82.8%	55.6%
Syria	Safita	2000	96.6%	98.7%	92.8%
Syria	Safita	2017	96.6%	98.7%	92.7%
Syria	Salamiyah	2000	68.4%	81.1%	49.2%
Syria	Salamiyah	2017	69.4%	81.2%	52.4%
Syria	Salkhad	2000	98.1%	99.5%	94.9%
Syria	Salkhad	2017	98.1%	99.5%	94.9%
Syria	Shahba	2000	95.7%	98.6%	90.2%
Syria	Shahba	2017	95.7%	98.6%	89.0%
Syria	Tal Abyad	2000	81.1%	90.2%	67.8%
Syria	Tal Abyad	2017	81.2%	90.5%	68.7%
Syria	Talkalakh	2000	94.0%	98.2%	86.5%
Syria	Talkalakh	2017	94.2%	98.2%	87.1%

Country	Second Administrative Unit	Year	Mean	Upper	Lower
Syria	Tartus	2000	92.7%	97.4%	85.8%
Syria	Tartus	2017	92.7%	97.4%	85.6%
Syria	Yabrud	2000	99.6%	100.0%	98.3%
Syria	Yabrud	2017	99.6%	100.0%	98.1%
Syria	Zabadani	2000	96.9%	99.6%	89.1%
Syria	Zabadani	2017	97.1%	99.6%	90.3%
Tunisia	Agareb	2000	49.9%	67.0%	36.0%
Tunisia	Agareb	2017	50.0%	67.5%	36.1%
Tunisia	Aïn Draham	2000	71.3%	76.1%	66.1%
Tunisia	Aïn Draham	2017	71.2%	76.0%	65.9%
Tunisia	Akouda	2000	57.2%	70.4%	41.5%
Tunisia	Akouda	2017	57.3%	70.5%	41.7%
Tunisia	Alaa	2000	54.9%	59.7%	50.3%
Tunisia	Alaa	2017	54.9%	59.6%	50.2%
Tunisia	Amdoun	2000	75.0%	79.3%	70.1%
Tunisia	Amdoun	2017	75.2%	79.5%	70.2%
Tunisia	Ariana Médina	2000	78.1%	81.2%	74.9%
Tunisia	Ariana Médina	2017	78.6%	81.6%	75.4%
Tunisia	Ayoun	2000	80.7%	84.1%	77.4%
Tunisia	Ayoun	2017	80.7%	84.1%	77.5%
Tunisia	Bab Bhar	2000	78.2%	80.9%	75.8%
Tunisia	Bab Bhar	2017	78.2%	80.9%	75.8%
Tunisia	Bab Souika	2000	78.8%	81.0%	76.6%
Tunisia	Bab Souika	2017	78.8%	81.0%	76.6%
Tunisia	Balta Bou Aouane	2000	75.3%	78.9%	70.7%
Tunisia	Balta Bou Aouane	2017	75.4%	79.1%	70.8%
Tunisia	Bardo	2000	78.9%	80.5%	77.4%
Tunisia	Bardo	2017	78.9%	80.6%	77.4%
Tunisia	Bargou	2000	76.2%	81.9%	70.1%
Tunisia	Bargou	2017	76.0%	81.7%	69.9%
Tunisia	Béja Nord	2000	76.0%	79.9%	71.3%
Tunisia	Béja Nord	2017	76.0%	80.0%	71.2%
Tunisia	Béja Sud	2000	76.4%	79.8%	72.6%
Tunisia	Béja Sud	2017	76.4%	79.8%	72.6%
Tunisia	Bekalta	2000	58.0%	71.9%	42.2%
Tunisia	Bekalta	2017	60.3%	73.6%	44.8%
Tunisia	Belkhir	2000	62.9%	68.8%	57.2%
Tunisia	Belkhir	2017	63.1%	68.8%	57.5%
Tunisia	Bembla	2000	59.4%	74.3%	42.8%
Tunisia	Bembla	2017	59.9%	74.7%	43.3%
Tunisia	Ben Arous	2000	79.0%	80.9%	77.1%
Tunisia	Ben Arous	2017	79.0%	81.0%	77.0%
Tunisia	Ben Guerdane	2000	47.2%	52.7%	41.6%
Tunisia	Ben Guerdane	2017	47.3%	52.6%	41.6%
Tunisia	Beni Hassen	2000	58.8%	72.9%	43.2%
Tunisia	Beni Hassen	2017	59.1%	73.1%	43.5%
Tunisia	Beni Khalled	2000	76.8%	79.9%	73.4%
Tunisia	Beni Khalled	2017	75.8%	79.1%	72.3%
Tunisia	Beni Khedache	2000	47.3%	54.4%	41.7%
Tunisia	Beni Khedache	2017	47.1%	54.0%	41.7%
Tunisia	Beni Khiar	2000	72.7%	77.7%	66.6%
Tunisia	Beni Khiar	2017	72.7%	77.8%	66.5%
Tunisia	Bir Ali Ben Khélifa	2000	48.9%	59.7%	38.0%
Tunisia	Bir Ali Ben Khélifa	2017	48.9%	59.9%	37.9%

Country	Second Administrative Unit	Year	Mean	Upper	Lower
Tunisia	Bir El Hfay	2000	49.9%	55.0%	44.5%
Tunisia	Bir El Hfay	2017	49.8%	55.0%	44.4%
Tunisia	Bir Lahmar	2000	45.7%	52.5%	38.4%
Tunisia	Bir Lahmar	2017	45.6%	52.1%	38.5%
Tunisia	Bir Mchergua	2000	75.5%	80.5%	70.1%
Tunisia	Bir Mchergua	2017	75.5%	80.4%	70.3%
Tunisia	Bizerte Nord	2000	74.9%	80.2%	67.8%
Tunisia	Bizerte Nord	2017	74.9%	80.2%	67.8%
Tunisia	Bizerte Sud	2000	72.9%	78.0%	66.0%
Tunisia	Bizerte Sud	2017	72.9%	78.1%	65.9%
Tunisia	Borj El Amri	2000	75.2%	81.8%	68.8%
Tunisia	Borj El Amri	2017	75.3%	81.8%	68.8%
Tunisia	Bou Argoub	2000	74.0%	77.7%	69.6%
Tunisia	Bou Argoub	2017	74.7%	78.5%	70.2%
Tunisia	Bouarada	2000	75.0%	80.1%	69.3%
Tunisia	Bouarada	2017	74.9%	80.0%	69.2%
Tunisia	Bouficha	2000	69.2%	78.8%	59.4%
Tunisia	Bouficha	2017	69.1%	78.9%	59.2%
Tunisia	Bouhaira	2000	77.5%	81.9%	72.8%
Tunisia	Bouhaira	2017	77.5%	81.9%	72.8%
Tunisia	Bouhajla	2000	53.0%	58.4%	47.7%
Tunisia	Bouhajla	2017	54.0%	59.4%	48.8%
Tunisia	Boumerdès	2000	57.6%	72.1%	41.3%
Tunisia	Boumerdès	2017	57.6%	72.0%	41.1%
Tunisia	Boumhel	2000	78.3%	81.7%	74.9%
Tunisia	Boumhel	2017	78.2%	81.5%	74.8%
Tunisia	Bourouis	2000	75.1%	82.1%	68.4%
Tunisia	Bourouis	2017	75.1%	82.1%	68.5%
Tunisia	Bousalem	2000	73.2%	76.8%	69.0%
Tunisia	Bousalem	2017	74.9%	78.4%	70.5%
Tunisia	Carthage	2000	76.1%	81.2%	70.1%
Tunisia	Carthage	2017	76.2%	81.3%	70.3%
Tunisia	Chebba	2000	57.0%	71.6%	42.4%
Tunisia	Chebba	2017	57.1%	71.7%	42.4%
Tunisia	Chebika	2000	50.7%	55.6%	45.5%
Tunisia	Chebika	2017	50.4%	55.3%	45.3%
Tunisia	Chorbane	2000	55.9%	69.4%	42.6%
Tunisia	Chorbane	2017	55.8%	69.2%	42.5%
Tunisia	Chrarda	2000	53.0%	61.3%	45.7%
Tunisia	Chrarda	2017	53.2%	61.5%	45.8%
Tunisia	Cité El Khadra	2000	78.1%	81.4%	74.6%
Tunisia	Cité El Khadra	2017	78.1%	81.3%	74.4%
Tunisia	Dahmani	2000	76.4%	81.4%	70.7%
Tunisia	Dahmani	2017	77.4%	82.1%	71.9%
Tunisia	Dar Chaabane El Fehri	2000	73.1%	78.1%	67.1%
Tunisia	Dar Chaabane El Fehri	2017	73.2%	78.3%	67.0%
Tunisia	Degueche	2000	74.4%	78.6%	70.3%
Tunisia	Degueche	2017	74.4%	78.8%	70.5%
Tunisia	Dhiba	2000	54.0%	65.8%	42.4%
Tunisia	Dhiba	2017	53.9%	65.7%	42.3%
Tunisia	Djerba Ajim	2000	44.0%	50.2%	38.0%
Tunisia	Djerba Ajim	2017	44.0%	50.1%	38.0%
Tunisia	Djerba Midoun	2000	45.9%	50.7%	41.0%
Tunisia	Djerba Midoun	2017	46.3%	51.2%	41.4%
Tunisia	Douar Hicher	2000	76.1%	78.8%	73.6%
Tunisia	Douar Hicher	2017	76.1%	78.8%	73.6%

Country	Second Administrative Unit	Year	Mean	Upper	Lower
Tunisia	Douz	2000	73.4%	77.4%	69.3%
Tunisia	Douz	2017	73.3%	77.2%	69.2%
Tunisia	El Alia	2000	73.7%	78.0%	70.0%
Tunisia	El Alia	2017	73.7%	78.0%	70.0%
Tunisia	El Amra	2000	55.4%	72.1%	38.6%
Tunisia	El Amra	2017	55.3%	72.0%	38.8%
Tunisia	El Battan	2000	75.8%	81.5%	69.8%
Tunisia	El Battan	2017	75.8%	81.6%	69.9%
Tunisia	El Ghraiba	2000	47.6%	60.1%	32.2%
Tunisia	El Ghraiba	2017	47.6%	60.1%	32.2%
Tunisia	El Jem	2000	56.9%	72.7%	41.4%
Tunisia	El Jem	2017	57.9%	73.7%	42.4%
Tunisia	El Krib	2000	77.2%	82.6%	71.7%
Tunisia	El Krib	2017	76.9%	82.3%	71.6%
Tunisia	El Menzah	2000	78.6%	81.4%	75.8%
Tunisia	El Menzah	2017	78.6%	81.4%	75.7%
Tunisia	El Mida	2000	73.5%	78.8%	68.2%
Tunisia	El Mida	2017	74.0%	79.2%	68.7%
Tunisia	El Mourouj	2000	79.2%	82.2%	76.3%
Tunisia	El Mourouj	2017	79.2%	82.1%	76.4%
Tunisia	El Ouardia	2000	79.0%	80.7%	77.0%
Tunisia	El Ouardia	2017	79.0%	80.7%	77.0%
Tunisia	El Tahrir	2000	79.0%	80.9%	77.3%
Tunisia	El Tahrir	2017	79.0%	81.0%	77.3%
Tunisia	Enfidha	2000	64.1%	74.0%	51.6%
Tunisia	Enfidha	2017	63.9%	73.8%	51.3%
Tunisia	Es Sers	2000	76.1%	82.2%	70.0%
Tunisia	Es Sers	2017	76.3%	82.4%	70.3%
Tunisia	Ettadhamen	2000	78.4%	80.6%	76.4%
Tunisia	Ettadhamen	2017	78.6%	80.8%	76.6%
Tunisia	Ezzahra	2000	78.1%	81.1%	75.0%
Tunisia	Ezzahra	2017	78.1%	81.1%	75.1%
Tunisia	Ezzouhour	2000	81.4%	83.3%	79.1%
Tunisia	Ezzouhour	2000	78.9%	80.7%	77.2%
Tunisia	Ezzouhour	2017	81.4%	83.3%	79.1%
Tunisia	Ezzouhour	2017	78.9%	80.6%	77.2%
Tunisia	Fahs	2000	74.5%	78.6%	70.4%
Tunisia	Fahs	2017	74.5%	78.7%	70.3%
Tunisia	Faouar	2000	73.3%	78.6%	67.0%
Tunisia	Faouar	2017	73.3%	78.6%	67.1%
Tunisia	Feriana	2000	80.4%	83.7%	77.1%
Tunisia	Feriana	2017	79.9%	83.2%	76.3%
Tunisia	Fernana	2000	72.6%	77.5%	67.2%
Tunisia	Fernana	2017	72.6%	77.6%	67.3%
Tunisia	Fouchana	2000	79.2%	82.5%	76.0%
Tunisia	Fouchana	2017	79.2%	82.5%	76.0%
Tunisia	Foussana	2000	80.2%	84.2%	75.2%
Tunisia	Foussana	2017	80.2%	84.2%	75.2%
Tunisia	Gaafour	2000	76.4%	81.8%	71.3%
Tunisia	Gaafour	2017	76.2%	81.7%	71.2%
Tunisia	Gabès Médina	2000	46.9%	50.7%	42.8%
Tunisia	Gabès Médina	2017	46.9%	50.5%	42.9%
Tunisia	Gabès Ouest	2000	46.4%	50.3%	43.1%
Tunisia	Gabès Ouest	2017	46.1%	50.0%	42.9%
Tunisia	Gabès Sud	2000	45.6%	49.3%	42.1%
Tunisia	Gabès Sud	2017	45.6%	49.3%	42.1%
Tunisia	Gafsa Nord	2000	70.8%	75.5%	67.1%
Tunisia	Gafsa Nord	2017	70.9%	75.5%	67.1%
Tunisia	Gafsa Sud	2000	76.3%	79.1%	73.6%
Tunisia	Gafsa Sud	2017	76.2%	79.0%	73.6%
Tunisia	Ghannouch	2000	46.8%	50.9%	41.9%
Tunisia	Ghannouch	2017	46.8%	50.9%	41.9%

Country	Second Administrative Unit	Year	Mean	Upper	Lower
Tunisia	Ghar El Melh	2000	73.5%	80.2%	66.3%
Tunisia	Ghar El Melh	2017	73.5%	80.2%	66.4%
Tunisia	Ghardimaou	2000	76.1%	80.3%	72.1%
Tunisia	Ghardimaou	2017	76.1%	80.2%	72.2%
Tunisia	Ghazala	2000	73.1%	76.9%	68.6%
Tunisia	Ghazala	2017	73.1%	77.0%	68.5%
Tunisia	Ghomrassen	2000	47.9%	53.7%	41.8%
Tunisia	Ghomrassen	2017	47.8%	53.8%	41.7%
Tunisia	Goubellat	2000	74.7%	80.3%	69.8%
Tunisia	Goubellat	2017	74.7%	80.3%	69.9%
Tunisia	Grombalia	2000	77.3%	80.2%	74.0%
Tunisia	Grombalia	2017	77.6%	80.6%	74.3%
Tunisia	Guetar	2000	75.6%	80.2%	70.3%
Tunisia	Guetar	2017	75.3%	79.7%	69.8%
Tunisia	Haffouz	2000	49.0%	55.0%	43.1%
Tunisia	Haffouz	2017	49.2%	55.1%	43.3%
Tunisia	Hajeb El Ayoun	2000	47.9%	54.2%	41.0%
Tunisia	Hajeb El Ayoun	2017	48.0%	54.4%	41.2%
Tunisia	Hamma	2000	50.2%	54.8%	45.1%
Tunisia	Hamma	2017	50.4%	54.9%	45.4%
Tunisia	Hammam Chott	2000	76.5%	80.2%	72.1%
Tunisia	Hammam Chott	2017	76.5%	80.2%	72.0%
Tunisia	Hammam Ghezaz	2000	73.2%	79.7%	65.9%
Tunisia	Hammam Ghezaz	2017	73.1%	79.7%	65.8%
Tunisia	Hammam Lif	2000	77.1%	80.4%	73.3%
Tunisia	Hammam Lif	2017	77.1%	80.4%	73.3%
Tunisia	Hammam Sousse	2000	58.6%	72.8%	42.4%
Tunisia	Hammam Sousse	2017	58.7%	72.9%	42.6%
Tunisia	Hammamet	2000	73.5%	79.6%	66.3%
Tunisia	Hammamet	2017	73.7%	79.9%	66.8%
Tunisia	Haouaria	2000	72.8%	78.8%	65.5%
Tunisia	Haouaria	2017	72.7%	78.6%	65.6%
Tunisia	Hassi El Ferid	2000	70.9%	75.8%	65.6%
Tunisia	Hassi El Ferid	2017	70.7%	75.3%	65.3%
Tunisia	Hazoua	2000	71.5%	79.2%	62.1%
Tunisia	Hazoua	2017	71.6%	79.2%	62.1%
Tunisia	Hbira	2000	54.3%	65.4%	44.3%
Tunisia	Hbira	2017	54.2%	65.3%	44.1%
Tunisia	Hencha	2000	55.3%	70.9%	41.8%
Tunisia	Hencha	2017	55.3%	70.6%	41.6%
Tunisia	Hergla	2000	58.7%	69.5%	45.2%
Tunisia	Hergla	2017	59.6%	70.1%	46.6%
Tunisia	Hidra	2000	80.2%	85.5%	74.0%
Tunisia	Hidra	2017	80.1%	85.5%	73.9%
Tunisia	Houmt Souk	2000	45.5%	50.9%	39.9%
Tunisia	Houmt Souk	2017	46.0%	51.5%	40.3%
Tunisia	Hrairia	2000	78.8%	80.7%	77.2%
Tunisia	Hrairia	2017	78.9%	80.7%	77.2%
Tunisia	Jammel	2000	57.7%	71.4%	41.4%
Tunisia	Jammel	2017	57.8%	71.4%	41.5%
Tunisia	Jebel Jelloud	2000	79.0%	80.8%	77.1%
Tunisia	Jebel Jelloud	2017	79.0%	80.8%	77.1%
Tunisia	Jebeniana	2000	56.5%	72.6%	40.3%
Tunisia	Jebeniana	2017	56.6%	72.6%	40.5%

Country	Second Administrative Unit	Year	Mean	Upper	Lower
Tunisia	Jedaida	2000	76.3%	82.0%	70.3%
Tunisia	Jedaida	2017	76.6%	82.2%	70.7%
Tunisia	Jedeliane	2000	78.6%	82.1%	74.2%
Tunisia	Jedeliane	2017	78.4%	82.0%	74.1%
Tunisia	Jelma	2000	49.6%	55.2%	44.6%
Tunisia	Jelma	2017	49.0%	54.6%	43.9%
Tunisia	Jendouba Nord	2000	73.1%	77.4%	67.7%
Tunisia	Jendouba Nord	2017	73.3%	77.6%	68.0%
Tunisia	Jendouba Sud	2000	74.1%	78.1%	69.7%
Tunisia	Jendouba Sud	2017	74.2%	78.2%	69.7%
Tunisia	Jerissa	2000	75.5%	81.2%	68.6%
Tunisia	Jerissa	2017	75.5%	81.1%	68.6%
Tunisia	Joumine	2000	75.0%	79.9%	69.8%
Tunisia	Joumine	2017	74.9%	79.8%	69.7%
Tunisia	Kabaria	2000	79.0%	80.8%	77.1%
Tunisia	Kabaria	2017	79.0%	80.8%	77.1%
Tunisia	Kairouan Nord	2000	51.6%	56.5%	47.1%
Tunisia	Kairouan Nord	2017	52.0%	56.9%	47.4%
Tunisia	Kairouan Sud	2000	53.1%	56.2%	49.9%
Tunisia	Kairouan Sud	2017	53.2%	56.3%	50.1%
Tunisia	Kalaa Kebira	2000	57.0%	69.3%	42.5%
Tunisia	Kalaa Kebira	2017	57.1%	69.3%	42.8%
Tunisia	Kalaa Khesba	2000	78.0%	84.0%	70.7%
Tunisia	Kalaa Khesba	2017	77.9%	83.9%	70.6%
Tunisia	Kalaa Sghira	2000	59.5%	71.7%	44.9%
Tunisia	Kalaa Sghira	2017	59.5%	71.6%	44.9%
Tunisia	Kalaat El Andalous	2000	74.5%	79.4%	69.9%
Tunisia	Kalaat El Andalous	2017	76.1%	80.5%	71.4%
Tunisia	Kalaat Senan	2000	76.4%	84.1%	68.2%
Tunisia	Kalaat Senan	2017	76.4%	84.0%	68.2%
Tunisia	Kasserine Nord	2000	79.4%	81.9%	76.6%
Tunisia	Kasserine Nord	2017	79.4%	82.0%	76.6%
Tunisia	Kasserine Sud	2000	80.5%	82.4%	78.3%
Tunisia	Kasserine Sud	2017	80.5%	82.4%	78.3%
Tunisia	Kebili Nord	2000	75.0%	79.1%	70.7%
Tunisia	Kebili Nord	2017	74.8%	78.8%	70.6%
Tunisia	Kebili Sud	2000	74.9%	78.7%	70.7%
Tunisia	Kebili Sud	2017	75.2%	79.0%	71.0%
Tunisia	Kef Est	2000	78.8%	83.5%	73.0%
Tunisia	Kef Est	2017	78.7%	83.4%	73.0%
Tunisia	Kef Ouest	2000	77.5%	82.4%	71.6%
Tunisia	Kef Ouest	2017	77.7%	82.5%	71.7%
Tunisia	Kelibia	2000	73.6%	79.4%	67.1%
Tunisia	Kelibia	2017	73.5%	79.4%	67.1%
Tunisia	Kerkennah	2000	51.6%	67.4%	35.6%
Tunisia	Kerkennah	2017	51.7%	67.6%	35.7%
Tunisia	Kesra	2000	71.3%	77.8%	64.9%
Tunisia	Kesra	2017	71.7%	78.2%	65.5%
Tunisia	Kondar	2000	55.8%	63.9%	45.7%
Tunisia	Kondar	2017	55.9%	64.0%	45.8%
Tunisia	Korba	2000	74.0%	78.4%	68.3%
Tunisia	Korba	2017	74.0%	78.4%	68.3%
Tunisia	Ksar	2000	76.9%	80.3%	73.3%
Tunisia	Ksar	2017	75.2%	78.6%	71.6%

Country	Second Administrative Unit	Year	Mean	Upper	Lower
Tunisia	Ksar Hellal	2000	59.6%	74.2%	44.5%
Tunisia	Ksar Hellal	2017	59.5%	74.1%	44.4%
Tunisia	Ksibet El Mediouni	2000	57.0%	72.0%	41.0%
Tunisia	Ksibet El Mediouni	2017	58.8%	73.5%	43.0%
Tunisia	Ksour	2000	77.1%	81.4%	72.0%
Tunisia	Ksour	2017	77.1%	81.4%	72.0%
Tunisia	Ksour Essef	2000	57.9%	72.7%	42.5%
Tunisia	Ksour Essef	2017	58.2%	73.0%	42.6%
Tunisia	La Goulette	2000	77.4%	81.5%	72.4%
Tunisia	La Goulette	2017	77.5%	81.5%	72.5%
Tunisia	La Marsa	2000	76.3%	81.3%	69.6%
Tunisia	La Marsa	2017	76.7%	81.6%	70.1%
Tunisia	Lake Ichkeul	2000	71.4%	77.8%	65.1%
Tunisia	Lake Ichkeul	2017	71.6%	78.0%	65.1%
Tunisia	Laroussa	2000	75.4%	81.6%	68.7%
Tunisia	Laroussa	2017	75.3%	81.5%	68.7%
Tunisia	M'Hamdia	2000	77.8%	82.7%	73.3%
Tunisia	M'Hamdia	2017	77.8%	82.7%	73.4%
Tunisia	M'Saken	2000	56.6%	68.8%	41.8%
Tunisia	M'Saken	2017	57.1%	69.4%	42.3%
Tunisia	Mahdia	2000	59.3%	73.3%	42.7%
Tunisia	Mahdia	2017	59.2%	73.2%	42.7%
Tunisia	Mahres	2000	50.5%	66.3%	36.4%
Tunisia	Mahres	2017	50.5%	66.5%	36.5%
Tunisia	Majel Belabbes	2000	77.2%	82.2%	71.5%
Tunisia	Majel Belabbes	2017	77.5%	82.3%	72.0%
Tunisia	Makthar	2000	76.2%	79.9%	71.7%
Tunisia	Makthar	2017	76.2%	80.1%	71.6%
Tunisia	Manouba	2000	78.2%	80.5%	76.4%
Tunisia	Manouba	2017	78.4%	80.6%	76.5%
Tunisia	Mareth	2000	46.1%	51.8%	40.0%
Tunisia	Mareth	2017	46.1%	51.9%	40.0%
Tunisia	Mateur	2000	72.9%	78.4%	66.2%
Tunisia	Mateur	2017	73.1%	78.6%	66.4%
Tunisia	Matmata	2000	50.9%	58.1%	43.0%
Tunisia	Matmata	2017	51.1%	58.0%	43.3%
Tunisia	Matmata Nouvelle	2000	47.2%	53.2%	40.8%
Tunisia	Matmata Nouvelle	2017	47.2%	53.0%	41.0%
Tunisia	Mazzouna	2000	49.9%	57.8%	41.5%
Tunisia	Mazzouna	2017	49.8%	57.6%	41.3%
Tunisia	Mdhilla	2000	74.8%	80.5%	69.4%
Tunisia	Mdhilla	2017	75.3%	80.9%	69.9%
Tunisia	Médenine Nord	2000	45.0%	50.2%	40.6%
Tunisia	Médenine Nord	2017	44.9%	50.2%	40.5%
Tunisia	Médenine Sud	2000	46.2%	51.0%	41.8%
Tunisia	Médenine Sud	2017	46.9%	51.6%	42.4%
Tunisia	Médina	2000	78.7%	81.0%	76.6%
Tunisia	Médina	2017	78.7%	81.0%	76.6%
Tunisia	Mégrine	2000	79.1%	80.7%	77.5%
Tunisia	Mégrine	2017	79.1%	80.7%	77.5%
Tunisia	Mejez El Bab	2000	76.1%	81.1%	71.1%
Tunisia	Mejez El Bab	2017	76.0%	81.0%	71.0%
Tunisia	Meknassi	2000	49.1%	53.6%	45.2%
Tunisia	Meknassi	2017	49.3%	53.8%	45.2%

Country	Second Administrative Unit	Year	Mean	Upper	Lower
Tunisia	Melloulech	2000	56.1%	71.4%	40.5%
Tunisia	Melloulech	2017	56.0%	71.3%	40.4%
Tunisia	Menzel Bourguiba	2000	74.2%	78.8%	68.8%
Tunisia	Menzel Bourguiba	2017	74.2%	78.8%	68.9%
Tunisia	Menzel Bouzaiene	2000	54.4%	61.4%	48.3%
Tunisia	Menzel Bouzaiene	2017	55.3%	62.1%	49.1%
Tunisia	Menzel Bouzelfa	2000	75.7%	79.6%	71.4%
Tunisia	Menzel Bouzelfa	2017	75.7%	79.6%	71.4%
Tunisia	Menzel Chaker	2000	52.0%	65.0%	40.5%
Tunisia	Menzel Chaker	2017	52.1%	65.1%	40.8%
Tunisia	Menzel Habib	2000	53.4%	60.1%	46.9%
Tunisia	Menzel Habib	2017	52.7%	59.4%	46.3%
Tunisia	Menzel Jemil	2000	73.7%	76.9%	70.2%
Tunisia	Menzel Jemil	2017	74.0%	77.3%	70.5%
Tunisia	Menzel Temime	2000	74.6%	79.2%	69.8%
Tunisia	Menzel Temime	2017	74.7%	79.4%	69.8%
Tunisia	Metlaoui	2000	73.7%	78.3%	68.7%
Tunisia	Metlaoui	2017	73.8%	78.3%	69.0%
Tunisia	Metouia	2000	44.3%	50.3%	38.0%
Tunisia	Metouia	2017	44.3%	50.3%	37.9%
Tunisia	Mnihla	2000	78.0%	80.4%	75.8%
Tunisia	Mnihla	2017	78.5%	80.9%	76.1%
Tunisia	Moknine	2000	60.4%	74.1%	45.5%
Tunisia	Moknine	2017	60.5%	74.3%	45.7%
Tunisia	Monastir	2000	57.1%	70.6%	40.6%
Tunisia	Monastir	2017	57.3%	70.8%	40.9%
Tunisia	Mornag	2000	77.5%	81.4%	73.5%
Tunisia	Mornag	2017	77.2%	81.2%	73.3%
Tunisia	Mornaguia	2000	76.9%	81.8%	72.1%
Tunisia	Mornaguia	2017	76.9%	81.8%	72.1%
Tunisia	Nabeul	2000	74.3%	79.8%	67.4%
Tunisia	Nabeul	2017	74.1%	79.6%	67.3%
Tunisia	Nadhour	2000	66.9%	72.7%	60.7%
Tunisia	Nadhour	2017	66.9%	72.8%	60.7%
Tunisia	Nasrallah	2000	49.9%	55.7%	44.0%
Tunisia	Nasrallah	2017	49.8%	55.7%	44.0%
Tunisia	Nebeur	2000	76.2%	79.8%	72.2%
Tunisia	Nebeur	2017	76.2%	79.7%	72.1%
Tunisia	Nefta	2000	74.2%	78.5%	69.2%
Tunisia	Nefta	2017	74.1%	78.5%	69.3%
Tunisia	Nefza	2000	73.9%	78.4%	68.9%
Tunisia	Nefza	2017	73.9%	78.5%	68.8%
Tunisia	Nouvelle Médina	2000	78.8%	81.6%	76.0%
Tunisia	Nouvelle Médina	2017	78.8%	81.7%	76.0%
Tunisia	Omrane	2000	78.8%	81.1%	76.6%
Tunisia	Omrane	2017	78.8%	81.1%	76.6%
Tunisia	Omrane Supérieur	2000	79.0%	81.2%	76.8%
Tunisia	Omrane Supérieur	2017	79.0%	81.2%	76.8%

Country	Second Administrative Unit	Year	Mean	Upper	Lower
Tunisia	Oued Ellil	2000	77.3%	81.2%	73.5%
Tunisia	Oued Ellil	2017	77.3%	81.3%	73.6%
Tunisia	Oued Mliz	2000	73.5%	77.6%	68.9%
Tunisia	Oued Mliz	2017	73.3%	77.5%	68.6%
Tunisia	Ouerdanine	2000	57.9%	71.8%	42.1%
Tunisia	Ouerdanine	2017	58.2%	71.9%	42.4%
Tunisia	Oueslatia	2000	61.7%	69.1%	54.0%
Tunisia	Oueslatia	2017	61.7%	69.0%	53.8%
Tunisia	Ouled Chamekh	2000	56.4%	66.3%	43.7%
Tunisia	Ouled Chamekh	2017	56.0%	66.1%	43.4%
Tunisia	Ouled Haffouz	2000	48.5%	54.8%	42.3%
Tunisia	Ouled Haffouz	2017	48.8%	55.0%	42.8%
Tunisia	Oum Larais	2000	78.4%	83.7%	72.7%
Tunisia	Oum Larais	2017	78.4%	83.5%	72.8%
Tunisia	Radès	2000	78.7%	80.5%	76.3%
Tunisia	Radès	2017	78.7%	80.6%	76.3%
Tunisia	Raoued	2000	76.6%	80.6%	72.1%
Tunisia	Raoued	2017	77.2%	81.2%	72.7%
Tunisia	Ras Jebel	2000	74.2%	81.3%	66.8%
Tunisia	Ras Jebel	2017	74.5%	81.4%	67.4%
Tunisia	Redeyef	2000	78.0%	82.2%	73.3%
Tunisia	Redeyef	2017	77.8%	81.9%	73.1%
Tunisia	Regueb	2000	47.9%	52.2%	43.1%
Tunisia	Regueb	2017	47.8%	52.2%	43.1%
Tunisia	Remada	2000	56.3%	62.5%	49.7%
Tunisia	Remada	2017	55.7%	62.1%	49.6%
Tunisia	Rouhia	2000	72.5%	77.0%	66.8%
Tunisia	Rouhia	2017	72.3%	76.8%	66.6%
Tunisia	Sabalat Ouled Asker	2000	55.3%	60.7%	50.1%
Tunisia	Sabalat Ouled Asker	2017	55.4%	60.9%	50.3%
Tunisia	Sabkhet Sijoumi	2000	78.5%	80.6%	76.3%
Tunisia	Sabkhet Sijoumi	2017	78.5%	80.7%	76.3%
Tunisia	Sahline	2000	57.5%	71.2%	41.6%
Tunisia	Sahline	2017	57.6%	71.3%	41.7%
Tunisia	Sakiet Ed-daier	2000	52.1%	67.6%	35.9%
Tunisia	Sakiet Ed-daier	2017	51.9%	67.5%	35.7%
Tunisia	Sakiet Ezzit	2000	49.7%	66.8%	33.7%
Tunisia	Sakiet Ezzit	2017	49.6%	66.7%	33.7%
Tunisia	Sakiet Sidi Youssef	2000	78.5%	84.3%	72.6%
Tunisia	Sakiet Sidi Youssef	2017	78.5%	84.4%	72.6%
Tunisia	Samar	2000	47.3%	54.4%	40.8%
Tunisia	Samar	2017	48.0%	54.9%	41.5%
Tunisia	Saouaf	2000	70.2%	77.8%	62.2%
Tunisia	Saouaf	2017	70.2%	77.8%	62.3%
Tunisia	Sayada-Lamta-Bou Hjar	2000	59.1%	74.0%	45.0%
Tunisia	Sayada-Lamta-Bou Hjar	2017	59.1%	73.9%	44.9%
Tunisia	Sbeitla	2000	74.7%	78.0%	71.7%
Tunisia	Sbeitla	2017	74.4%	77.7%	71.3%

Country	Second Administrative Unit	Year	Mean	Upper	Lower
Tunisia	Sbiba	2000	73.8%	77.9%	69.4%
Tunisia	Sbiba	2017	74.2%	78.2%	70.1%
Tunisia	Sbikha	2000	56.7%	61.2%	51.5%
Tunisia	Sbikha	2017	56.6%	61.3%	51.2%
Tunisia	Sebkhat Sidi El Hani	2000	55.6%	66.9%	41.3%
Tunisia	Sebkhat Sidi El Hani	2017	55.8%	67.1%	41.5%
Tunisia	Sebkhet Ariana	2000	74.6%	79.5%	68.5%
Tunisia	Sebkhet Ariana	2017	75.6%	80.4%	69.7%
Tunisia	Sebkhet El Moknine	2000	60.5%	74.7%	45.8%
Tunisia	Sebkhet El Moknine	2017	61.6%	75.5%	47.0%
Tunisia	Sebkhit El Kabla	2000	54.5%	63.1%	43.6%
Tunisia	Sebkhit El Kabla	2017	54.6%	63.2%	43.5%
Tunisia	Sejnane	2000	74.2%	80.6%	67.7%
Tunisia	Sejnane	2017	74.2%	80.6%	67.6%
Tunisia	Sened	2000	65.9%	70.5%	61.2%
Tunisia	Sened	2017	65.6%	70.1%	60.8%
Tunisia	Sfax Médina	2000	53.1%	68.5%	35.7%
Tunisia	Sfax Médina	2017	53.1%	68.5%	35.7%
Tunisia	Sfax Ouest	2000	52.9%	68.8%	35.8%
Tunisia	Sfax Ouest	2017	52.9%	68.8%	35.8%
Tunisia	Sfax Sud	2000	51.0%	67.4%	35.5%
Tunisia	Sfax Sud	2017	50.8%	67.2%	35.2%
Tunisia	Sidi Aich	2000	72.4%	79.5%	64.5%
Tunisia	Sidi Aich	2017	72.4%	79.6%	64.6%
Tunisia	Sidi Ali Ben Aoun	2000	54.5%	59.2%	48.4%
Tunisia	Sidi Ali Ben Aoun	2017	54.4%	59.2%	48.2%
Tunisia	Sidi Alouane	2000	57.4%	73.4%	41.3%
Tunisia	Sidi Alouane	2017	57.5%	73.4%	41.4%
Tunisia	Sidi Bou Ali	2000	57.7%	69.1%	45.2%
Tunisia	Sidi Bou Ali	2017	58.4%	69.7%	46.3%
Tunisia	Sidi Bouzid Est	2000	46.5%	49.9%	43.1%
Tunisia	Sidi Bouzid Est	2017	46.9%	50.4%	43.4%
Tunisia	Sidi Bouzid Ouest	2000	47.9%	52.5%	43.5%
Tunisia	Sidi Bouzid Ouest	2017	48.0%	52.6%	43.6%
Tunisia	Sidi El Béchir	2000	78.5%	80.7%	76.3%
Tunisia	Sidi El Béchir	2017	78.5%	80.6%	76.3%
Tunisia	Sidi El Heni	2000	52.3%	61.0%	41.4%
Tunisia	Sidi El Heni	2017	52.2%	60.9%	41.4%
Tunisia	Sidi Hassine	2000	78.6%	81.2%	76.0%
Tunisia	Sidi Hassine	2017	78.8%	81.5%	76.1%
Tunisia	Sidi Makhlouf	2000	44.6%	49.7%	39.5%
Tunisia	Sidi Makhlouf	2017	44.5%	49.8%	39.2%
Tunisia	Sidi Thabet	2000	76.4%	80.4%	72.0%
Tunisia	Sidi Thabet	2017	76.9%	80.9%	72.7%
Tunisia	Sijoumi	2000	78.8%	81.0%	76.6%
Tunisia	Sijoumi	2017	78.8%	81.0%	76.6%
Tunisia	Siliana Nord	2000	76.7%	80.2%	73.1%
Tunisia	Siliana Nord	2017	76.7%	80.2%	73.1%

Country	Second Administrative Unit	Year	Mean	Upper	Lower
Tunisia	Siliana Sud	2000	76.3%	80.7%	71.2%
Tunisia	Siliana Sud	2017	76.5%	80.9%	71.6%
Tunisia	Skhira	2000	47.2%	57.9%	34.7%
Tunisia	Skhira	2017	47.3%	58.1%	34.9%
Tunisia	Soliman	2000	76.8%	80.8%	72.7%
Tunisia	Soliman	2017	77.7%	81.6%	73.7%
Tunisia	Souassi	2000	57.0%	70.2%	43.6%
Tunisia	Souassi	2017	57.2%	70.6%	43.2%
Tunisia	Souk El Ahed	2000	75.9%	79.9%	71.1%
Tunisia	Souk El Ahed	2017	75.7%	79.6%	71.1%
Tunisia	Souk Jedid	2000	46.7%	52.5%	40.9%
Tunisia	Souk Jedid	2017	46.6%	52.4%	40.8%
Tunisia	Soukra	2000	77.7%	81.4%	73.2%
Tunisia	Soukra	2017	77.7%	81.5%	73.2%
Tunisia	Sousse Jaouhara	2000	60.4%	73.5%	44.3%
Tunisia	Sousse Jaouhara	2017	60.3%	73.5%	44.2%
Tunisia	Sousse Médina	2000	60.1%	73.6%	43.9%
Tunisia	Sousse Médina	2017	60.1%	73.7%	43.9%
Tunisia	Sousse Riadh	2000	60.2%	73.2%	44.5%
Tunisia	Sousse Riadh	2017	60.2%	73.1%	44.5%
Tunisia	Sousse Sidi Abdelhamid	2000	60.4%	73.9%	44.1%
Tunisia	Sousse Sidi Abdelhamid	2017	60.4%	73.9%	44.1%
Tunisia	Tabarka	2000	72.3%	78.2%	65.4%
Tunisia	Tabarka	2017	71.8%	77.9%	64.7%
Tunisia	Tajerouine	2000	74.1%	79.7%	69.1%
Tunisia	Tajerouine	2017	74.1%	79.7%	69.2%
Tunisia	Takelsa	2000	73.7%	79.9%	67.6%
Tunisia	Takelsa	2017	73.7%	79.8%	67.7%
Tunisia	Tamaghza	2000	75.4%	81.7%	66.9%
Tunisia	Tamaghza	2017	75.2%	81.7%	66.7%
Tunisia	Tataouine Nord	2000	48.0%	52.5%	43.3%
Tunisia	Tataouine Nord	2017	48.1%	52.5%	43.3%
Tunisia	Tataouine Sud	2000	49.2%	54.3%	44.2%
Tunisia	Tataouine Sud	2017	49.4%	54.8%	44.2%
Tunisia	Teboulba	2000	60.3%	73.9%	45.6%
Tunisia	Teboulba	2017	60.1%	73.8%	45.5%
Tunisia	Tebourba	2000	74.4%	80.2%	67.4%
Tunisia	Tebourba	2017	74.5%	80.3%	67.5%
Tunisia	Téboursouk	2000	78.2%	84.0%	73.0%
Tunisia	Téboursouk	2017	78.1%	83.8%	72.9%
Tunisia	Testour	2000	75.2%	79.5%	70.1%
Tunisia	Testour	2017	75.2%	79.4%	69.9%
Tunisia	Thala	2000	79.9%	83.5%	74.7%
Tunisia	Thala	2017	79.6%	83.2%	74.3%
Tunisia	Thibar	2000	76.7%	81.1%	71.8%
Tunisia	Thibar	2017	76.4%	80.8%	71.4%
Tunisia	Tinja	2000	73.0%	78.2%	67.3%
Tunisia	Tinja	2017	73.2%	78.4%	67.3%
Tunisia	Tozeur	2000	74.3%	78.6%	69.6%
Tunisia	Tozeur	2017	74.1%	78.5%	69.6%
Tunisia	Unknown	2000	79.0%	80.6%	77.6%
Tunisia	Unknown	2017	79.0%	80.6%	77.6%
Tunisia	Unknown1	2000	58.6%	73.3%	42.1%
Tunisia	Unknown1	2017	58.6%	73.3%	42.1%

Country	Second Administrative Unit	Year	Mean	Upper	Lower
Tunisia	Utique	2000	73.5%	78.9%	68.8%
Tunisia	Utique	2017	73.4%	78.9%	68.8%
Tunisia	Zaghouan	2000	75.7%	80.2%	70.6%
Tunisia	Zaghouan	2017	75.7%	80.2%	70.7%
Tunisia	Zarzis	2000	49.3%	54.8%	44.3%
Tunisia	Zarzis	2017	49.4%	54.9%	44.5%
Tunisia	Zeramdine	2000	57.6%	69.9%	42.1%
Tunisia	Zeramdine	2017	57.9%	70.2%	42.1%
Tunisia	Zriba	2000	73.7%	80.3%	66.8%
Tunisia	Zriba	2017	73.7%	80.3%	66.7%
Yemen	Abs	2000	54.2%	74.1%	31.0%
Yemen	Abs	2017	44.0%	66.2%	18.6%
Yemen	Ad Dahi	2000	37.3%	71.0%	3.3%
Yemen	Ad Dahi	2017	31.0%	63.3%	1.0%
Yemen	Ad Dhale'e	2000	63.7%	75.5%	50.0%
Yemen	Ad Dhale'e	2017	55.6%	71.7%	40.0%
Yemen	Ad Dis	2000	37.4%	73.3%	8.5%
Yemen	Ad Dis	2017	31.6%	65.0%	5.7%
Yemen	Ad Durayhimi	2000	49.8%	86.4%	14.5%
Yemen	Ad Durayhimi	2017	42.8%	81.8%	10.4%
[truncated: 546,996 more chars]
